# Gall Wasp Transcriptomes Unravel Potential Effectors Involved in Molecular Dialogues With Oak and Rose

**DOI:** 10.3389/fphys.2019.00926

**Published:** 2019-07-24

**Authors:** Sébastien Cambier, Olivia Ginis, Sébastien J. M. Moreau, Philippe Gayral, Jack Hearn, Graham N. Stone, David Giron, Elisabeth Huguet, Jean-Michel Drezen

**Affiliations:** ^1^UMR 7261 CNRS, Institut de Recherche sur la Biologie de l’Insecte, Faculté des Sciences et Techniques, Université de Tours, Tours, France; ^2^Institute of Evolutionary Biology, The University of Edinburgh, Edinburgh, United Kingdom

**Keywords:** oak gall wasp, rose gall wasp, gall induction, venom, ovary secretions

## Abstract

To gain insight into wasp factors that might be involved in the initial induction of galls on woody plants, we performed high throughput (454) transcriptome analysis of ovaries and venom glands of two cynipid gall wasps, *Biorhiza pallida* and *Diplolepis rosae*, inducing galls on oak and rose, respectively. *De novo* assembled and annotated contigs were compared to sequences from phylogenetically related parasitoid wasps. The relative expression levels of contigs were estimated to identify the most expressed gene sequences in each tissue. We identify for the first time a set of maternally expressed gall wasp proteins potentially involved in the interaction with the plant. Some genes highly expressed in venom glands and ovaries may act to suppress early plant defense signaling. We also identify gall wasp cellulases that could be involved in observed local lysis of plant tissue following oviposition, and which may have been acquired from bacteria by horizontal gene transfer. We find no evidence of virus-related gene expression, in contrast to many non-cynipid parasitoid wasps. By exploring gall wasp effectors, this study is a first step toward understanding the molecular mechanisms underlying cynipid gall induction in woody plants, and the recent sequencing of oak and rose genomes will enable study of plant responses to these factors.

## Introduction

Cynipoid wasps (Hymenoptera: Apocrita) constitute a diverse lineage of plant and insect parasites. Although most cynipoid lineages are parasitoids of insect larvae, the well-described family Cynipidae consists entirely of plant-galling wasps (Ronquist, 1999; Ronquist et al., 2015). Around 1400 species of gall wasps parasitizing different plants have been described (Ronquist et al., 2015). While some species gall non-woody herbs (Ronquist and Liljeblad, 2001; Abe et al., 2007), the most familiar species belong to two gall wasp tribes, the Cynipini and Diplolepidini, which induce galls on oak trees and rose bushes, respectively (Ronquist and Liljeblad, 2001; Ronquist et al., 2015).

These gall wasps have the capacity to modify plant host physiology, resulting in the development of complex gall structures that resemble novel plant organs but which are never produced by healthy plants (Harper et al., 2004). Gall tissues provide food and physical protection for the larvae developing within them (Cornell, 1983; Price et al., 1987; Bailey et al., 2009). Structurally, cynipid galls can be divided into two parts: the larval chamber and the outer gall. The larval chamber, which is structurally similar in almost all cynipid galls (Stone et al., 2002), is lined with nutritive plant tissues on which the larva feeds, and is surrounded by a thin wall of sclerenchyma. The cynipid larva completes its entire development within this chamber. The diversity observed in cynipid gall morphology is the result of variation in the targeted plant organ (Shorthouse et al., 2005) and in gall tissues that develop outside the larval chamber, such as surrounding layers of woody or spongy tissue, complex air spaces within the gall, surface coats of sticky resins, hairs or spines (Stone and Schönrogge, 2003), and extrafloral nectaries (Nicholls et al., 2016; Pierce, 2019). Mature galls formed by members of the same genus may differ enormously in size, color and shape (Stone and Schönrogge, 2003; Bailey et al., 2009), and are diagnostic of the inducing gall wasp species. In consequence, galls are considered to be the extended phenotypes of galler genes (Dawkins, 1982; Stone and Schönrogge, 2003). Overall, this suggests gall wasps have evolved a genuine molecular dialogues with the plant that allows establishment of a precise developmental program resulting in the formation of specific galls as well as preventing or diverting plant defenses. However, while many studies have analyzed the developmental and morphological aspects of gall induction, the molecular mechanisms used by the gall wasps to modify plant host physiology during cecidogenesis (i.e., gall formation) are unknown (Giron et al., 2016; Nabity, 2016).

Cynipid gall development by wasps can be divided into three major steps: (i) initiation, (ii) growth, and (iii) maturation (Rohfritsch, 1992; Stone et al., 2002). Several lines of evidence suggest that gall initiation and development are governed by specific factors produced by gall wasp larvae (Leblanc and Lacroix, 2001). For example, pioneering work (Molliard, 1917) showed that injection of total and crude extracts of larvae of a herb gall wasp, *Aylax papaveris*, into the pistils of *Papaver rhoeas* flowers resulted in developmental responses mimicking the hypertrophy of the parietal placenta observed during cecidogenesis. Continued production of some stimuli by gall wasp larvae is suggested by the fact that in galls of *Diplolepis rosae* on *Rosa canina*, chambers hosting living gall wasp larvae are significantly larger than chambers hosting dead gall wasps or hymenopteran parasitoids. This shows that gall wasp larvae induce specific plant modifications that cannot be triggered or maintained by other Hymenoptera opportunistically developing in galls (Brooks and Shorthouse, 1997). In contrast, the role of maternal secretions (ovarian fluids or venom deposited on host plant cells or injected into them by adult female wasps during oviposition) in cecidogenesis is still a matter of debate. The initiation phase of gall induction is often considered to result from the action of secretions derived from the egg and larva and not from maternal secretions (Stone et al., 2002). However, some observations are compatible with initiation stimuli being present in maternal secretions. Magnus (1914) observed early modifications of plant cells (hypertrophy and hyperplasia) before hatching of *D. rosae* eggs that could not be explained simply by the mechanical wounding of plant tissue. Stimuli driving this response could derive from injected venom or ovary secretions lining the eggs. Bronner (1973) detected proteolytic, cellulolytic and pectinolytic activities at the surface and along the egg stalk of *Biorhiza pallida* naturally laid on oak buds. Bronner (1985) later observed a substance “deposited at oviposition” at the other end of *D. rosae* eggs and in contact with plant epidermal cells that died shortly after oviposition. More recently, cytoplasmic condensation, enlargement of the nucleus and nucleoli and fragmented vacuolation were observed in plant cells adjacent to *D. rosaefolii* maternal fluids deposited during oviposition on the abaxial surface of leaflets of *R. virginiana* (Leblanc and Lacroix, 2001). Limited autolysis of plant tissue led to the creation of a chamber into which the newly hatched larvae subsequently migrated. In this system, as in several other plant-cynipid associations (Shorthouse et al., 2005), tissue changes occur rapidly in zones adjacent to or below the egg’s attachment to the host plant epidermis, where the female wasp deposits maternal fluids during oviposition, suggesting that the very first initiation steps of gall induction could depend on adult female gall wasp secretions. Furthermore, a morphological comparison of the venom apparatus in 25 species of Cynipoidea revealed that most gall inducing wasps have better-developed and more prominent structures than closely related parasitoid (i.e., non gall-inducing) wasps (Vårdal, 2006). This is compatible with the hypothesis that the venom of gall wasps could indeed be of functional importance in the interaction with the host plant, an issue recently addressed for other hymenopteran species interacting with plants such as fig wasps (Martinson et al., 2015; Elias et al., 2018) and a seed-parasitic wasp (Paulson et al., 2016).

In this study, we present anatomical data on the venom glands of two gall wasp species, *B. pallida* and *D. rosae*, the large sizes of which further suggest an important investment of the wasps in venomous secretions, and the first venom gland and ovary transcriptomes for any gall inducing cynipid. Our overall aim is to identify candidate genes involved in interactions between the gall wasp and either its host plant or its natural enemies, such as bacteria and fungi that may attack young galls (Taper et al., 1986; Taper and Case, 1987; Wilson, 1995). To achieve this, we first identify transcripts coding for potentially secreted proteins that are substantially differentially expressed between these two tissues. We then use annotation information to identify candidates with possible roles in gall wasp-plant or gall wasp-natural enemy interactions. We use these data to test the hypothesis advanced by Cornell (1983) that gall induction involves symbiotic viral partners by asking whether venom gland or ovary transcriptomes show any evidence of export of gall wasp genes or proteins within viral particles, a mechanism known to be involved in delivery of effectors used by hymenopteran parasitoids to manipulate the physiology of their insect hosts (Drezen et al., 2017). Finally, we assess the novelty of transcripts in gall-inducing cynipids through comparison with published venom gland transcriptomes for a panel of parasitoid Hymenoptera, including figitid cynipoids that represent the sister group and putative ancestral lifestyle of gall inducing cynipids.

## Materials and Methods

### Gall Collection and Tissue Dissection

Oak (*Quercus robur*) bud galls of *B. pallida* were collected in May 2010 in Tours (France, 47° 21′ 22″N, 0° 42′ 10″E). Wild rose (*R. canina*) bud galls of *D. rosae* were harvested in November 2009 in Thilouze (France, 47°14′35″N, 0°34′43″E). Galls were incubated at room temperature until wasp emergence. At this time mature eggs are already present in the ovaries suggesting the wasps are ready for oviposition and that virulence factors are already produced as in most parasitoid wasps. A total of 135 female *B. pallida* and 146 female *D. rosae* were dissected immediately after emergence to isolate ovaries and venom glands (along with their contiguous reservoir for *B. pallida*). Dissections were performed on ice in a sterile phosphate-buffered saline (PBS) droplet. Five venom glands from each species were immediately used for microscopy observations (see below). The remaining venom glands and ovaries were preserved at −80°C in RA1 RNA extraction buffer containing β-mercaptoethanol according to the NucleoSpin RNA II Kit instructions (Macherey-Nagel, France) until enough material could be collected to perform total RNA extractions.

### Fluorescence Microscopy and Confocal Microscopy Imaging of Venom Glands

For observation under fluorescence or confocal microscopy, each venom gland was transferred to a microscope slide with reaction wells containing 35 μl of 4% (w/v) paraformaldehyde for 15 min of incubation at room temperature in a dark moisture chamber. Venom glands were washed three times in PBS and cells were permeabilized in 0.1% (v/v) Triton X-100 for 5 min and washed three times in PBS. Actin was stained with fluorescein isothiocyanate (FITC) conjugated phalloidin (0.5 mg/ml final concentration in PBS) for 60 min at room temperature in a dark moisture chamber and washed three times in PBS. Nucleic acids were stained using 40 μl of Hoechst 33258 (10 μg/ml final concentration in PBS) for 10 min and washed three times in PBS. Microscope slides were then covered with a square glass coverslip and observed immediately under a fluorescence microscope (Olympus BX51 with CCD camera DP50) for *B. pallida*, or analyzed under an Olympus Fluoview 500 confocal laser-scanning microscope for *D. rosae*. Filter 330–380 nm and filter 465–495 nm were used for observations of nuclei and actin staining, respectively.

### RNA Extraction and Quality Control

RNA extractions were performed using the NucleoSpin RNA II Kit guidelines (Macherey-Nagel, France). Total RNA yields were 120 μg and 19 μg for 130 *B. pallida* ovaries and venom glands, and 16 and 34 μg for 141 *D. rosae* ovaries and venom glands, respectively. Absence of RNase in samples was confirmed by comparing agarose gel electrophoresis profiles of RNA subsamples incubated for 2 h at 37°C to untreated RNA subsamples. RNA quality was evaluated by analyzing samples on a Bioanalyzer (Agilent, France).

### cDNA Library Construction and Sequencing

cDNA libraries were constructed from RNA extracted from ovaries and venom glands of *B. pallida* and *D. rosae* using the SMARTer^TM^ PCR cDNA Synthesis Kit (Clontech, France) according to the supplier’s instructions. In brief, 1 μg of total RNA was used to perform the first strand cDNA synthesis using a primer hybridizing to the polyA tail. From the single strand cDNA libraries, a cDNA amplification was performed by LD PCR to obtain the optimal amount of double stranded cDNA, which was used for 454 pyrosequencing using a GS FLX Titanium platform (GEH Biogenouest^®^, Rennes, France). It is noteworthy that the selection of molecules having a polyA tail for cDNA first strand synthesis is supposed to prevent sequencing of transcripts of bacterial origin. Indeed, only a very low level of bacterial RNA contamination could be detected in the sequenced libraries as illustrated by the very low level 23S ribosomal RNA contigs (Bp_contig 01854, Dr_contig 01575) of *Wolbachia* bacteria, a symbiont known to infect both *B. pallida* (Rokas et al., 2002) and *D. rosae* (Plantard et al., 1999).

### Bioinformatic Treatment, Functional Annotation, and Sequence Analysis

Raw data generated by 454 pyrosequencing (Genbank/EMBL/DBJ accession number: PRJNA517634) were preprocessed using SnoWhite software (Dlugosch et al., 2013) to remove nonsense sequences including (i) adapters used for reverse transcription and 454 sequencing, (ii) primers, (iii) very short (<15 bp) sequences, and (iv) low quality sequences. In a first step, the preprocessed sequences were assembled with optimal parameters (-minlen 15 -it 0 -ig 0 - icc 0 -icl 5000 -mi 95 -ml 10 -ss 1 -sl 16 -a 15 -rip) using runAssembly (Newbler software, Roche version 2.6) supplied by Geneouest (France). In a second step, the same preprocessed sequences were mapped to the transcriptome resulting from the first assembly using runMapping (Newbler software, Roche version 2.6) with optimal parameters (-minlen 15 - notrim -ss 1 -sl 16 -sc 1 -ml 80% -mi 95% -a 15 -d 15) to obtain the final transcriptome used for the analysis. To annotate the obtained contigs we compared them with the available non-redundant (nr) NCBI protein database using blastx software with an *E*-value cut-off of 10^−4^.

To assess the potential function of proteins encoded by contigs, we used the Gene Ontology (GO) controlled vocabulary and more particularly GOSlim, a subset of GO terms, which provides a higher level of annotation (Vincent et al., 2010). To this end, an automated GO-annotation of contigs was performed using the Blast2go software and a stringency cut-off of 10^−6^.

To identify peptide signals and transmembrane domains the assembled sequences were translated into the correct open reading frames (ORFs) using Prodigal (Hyatt et al., 2010) and amino acid sequences were analyzed using signalP 4.0^1^ (Nielsen, 2017). SignalP identifies potential signal peptides in amino acid sequences and locates their cleavage sites. In a previous study, we found that most proteins corresponding to highly expressed genes and predicted to contain a peptide signal by SignalP were indeed confirmed by proteomic analysis to be present in the venom of the parasitoid wasp *Chelonus inanitus* (Vincent et al., 2010). In addition, we also used the DeepLoc-1.0 program that predicts the localization (subcellular or extracellular) of eukaryotic proteins^2^.

Gall wasp contigs were specifically compared using blastx and tblastx programs to available parasitoid wasp transcriptomes from NCBI public databases and from a homemade database collecting sequences of venom components reported from a selection of parasitoid species. This set comprised two species (*Leptopilina boulardi* and *L. heterotoma*) (Colinet et al., 2013a, b; Poirié et al., 2014) in the family Figitidae, the sister group of Cynipidae within the superfamily Cynipoidea, two species in the family Pteromalidae of the superfamily Chalcidoidea [*Pteromalus puparum* (Zhu et al., 2010) and *Nasonia vitripennis]* (Danneels et al., 2010; de Graaf et al., 2010), and four more distantly related species in the Hymenoptera (*C. inanitus* (Vincent et al., 2010) and *Microplitis demolitor* (Burke and Strand, 2014), in the family Braconidae and *Pimpla hypochondriaca* (Dani et al., 2005) and *Hyposoter didymator* (Dorémus et al., 2013) in the family Ichneumonidae).

For gene expression analysis, the number of expressed reads was counted and normalized using RPKM (reads per kb million reads: number of reads × 10^9^/contig length × total reads of the library) (Mortazavi et al., 2008). Contigs were considered as differentially expressed when RPKM values were at least 20 times higher in one tissue than in the other. Comparison of expression levels by qRT-PCR and 454 RPKM in our previous work using the same experimental approach revealed that the number of reads obtained using 454 sequencing reflected the level of expression and that this value was a good cut-off to define over- or under-expressed genes (Chevignon et al., 2014, 2015).

## Results

### Fluorescence Microscopy and Confocal Microscopy Imaging of Venom Apparatuses

**FIGURE 1:**
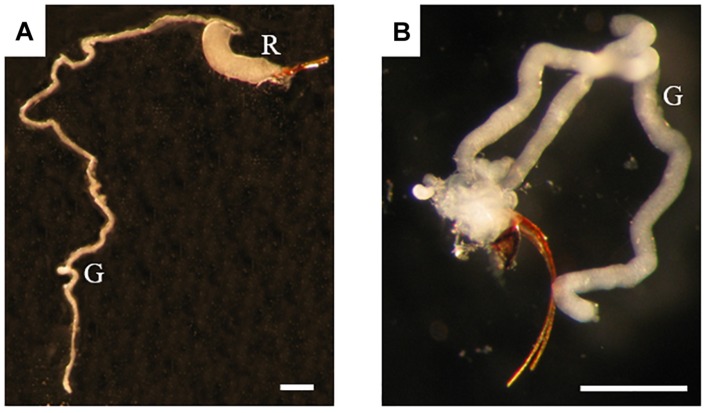
Venom apparatus of *B. pallida* **(A)** and *D. rosae* **(B)** females. G, venom gland; R, venom reservoir. Scale bars = 0,6 mm.

**FIGURE 2:**
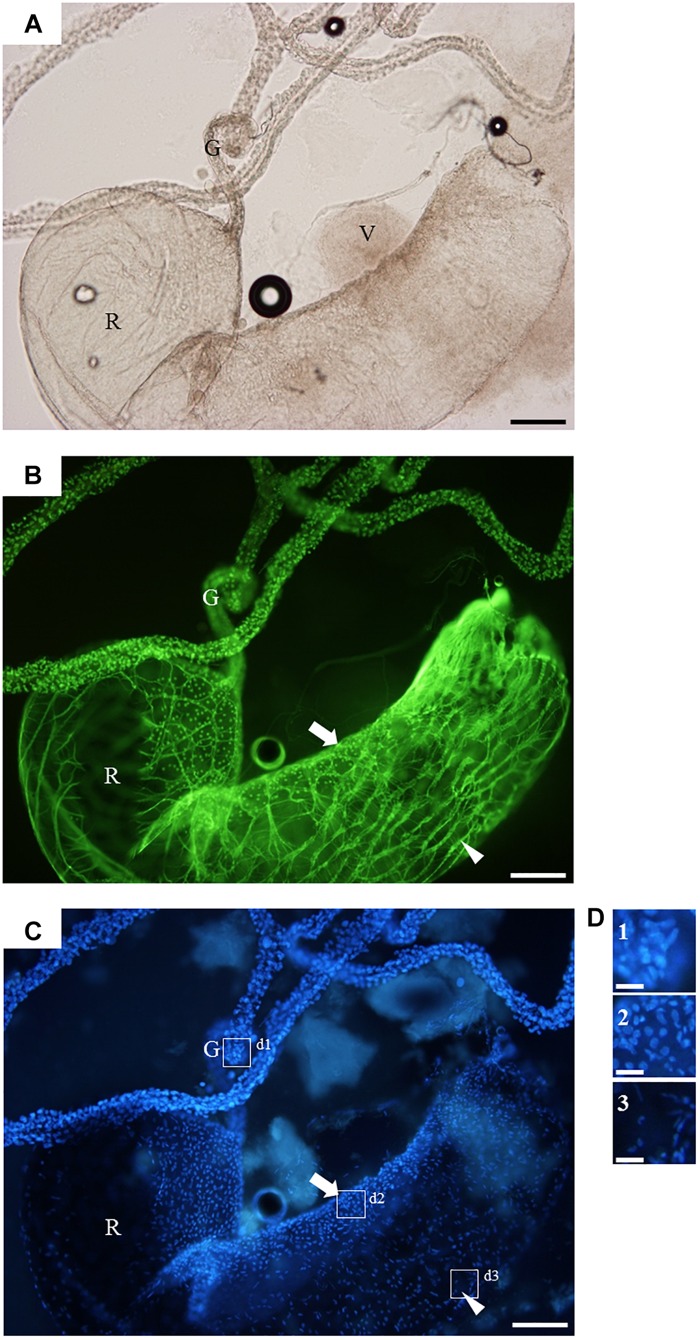
The venom apparatus of *B. pallida* observed in fluorescence microscopy. **(A)** light microscopy, **(B)** FITC-conjugated phalloidin staining for actin detection, **(C)** DAPI staining for detection of nucleic acids, and **(D)** detailed view of **(C)**. G, venom gland; R, venom reservoir; V, venom; arrow, secondary secretory zone; arrowhead, muscular fiber; boxes d1, d2, and d3 refer to detailed views shown in panel **(D)**. Note the high density of the venom leaking from the ruptured reservoir in panel **(A)**, and the different shapes of nuclei that allow to discriminate between the different tissues composing the organ. Scale bars = 0,3 mm for **(A)**, **(B)**, and **(C)** or 50 μm for **(D)**.

**FIGURE 3:**
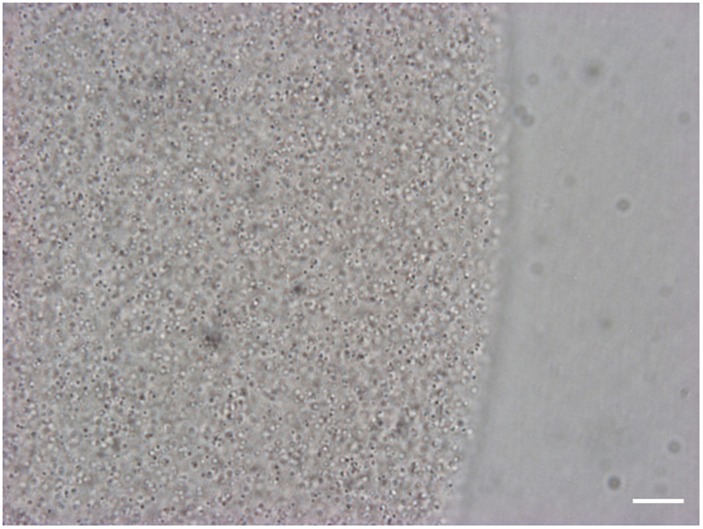
View of the venom of *B. pallida* in light microscopy. At high magnification, numerous spherical particles of approximately 1 to 2 μm in diameter are visible in the venom of *B. pallida*. Scale bar = 50 μm.

For informed selection of tissues to use for transcriptomic analyses we performed a detailed examination of the venom apparatus of the two gall-wasps species studied, which revealed two different organizations (Figures 1A,B). The venom apparatus of *B. pallida* includes a very large reservoir (approximately 2.5 to 3 mm long) relative to other similarly sized Hymenoptera, and a single tubular gland composed of a simple glandular epithelium (Figure 2A). Each glandular cell is connected to a central canal via a small duct (ductule) rich in actin that was stained positively by FITC-conjugated phalloidin (Figure 2B). Nuclei of these cells are large and round (Figure 2C). The dorsal side of the reservoir incorporates a loose secondary secretory zone characterized by cells with small isolated nuclei, round to triangular in shape and organized in a simple epithelium (Figures 2B–D). The remaining surface of the reservoir wall consists of a very loose simple epithelium, the cells of which have an elongated nucleus (Figures 2C,D). On its external side, the reservoir wall is lined with a loose network of long and striated skeletal muscles with fusiform nuclei (Figures 2B–D). Rupture of the reservoir in saline buffer resulted in leakage of a very dense and viscous venom (Figure 2A). Observation at high magnification revealed that the venom of *B. pallida* is full of spherical particles approximately 1 to 2 μm in diameter (Figure 3) that remained unstained by DAPI (for detection of nucleic acids) or FITC-conjugated phalloidin (data not shown).

**FIGURE 4:**
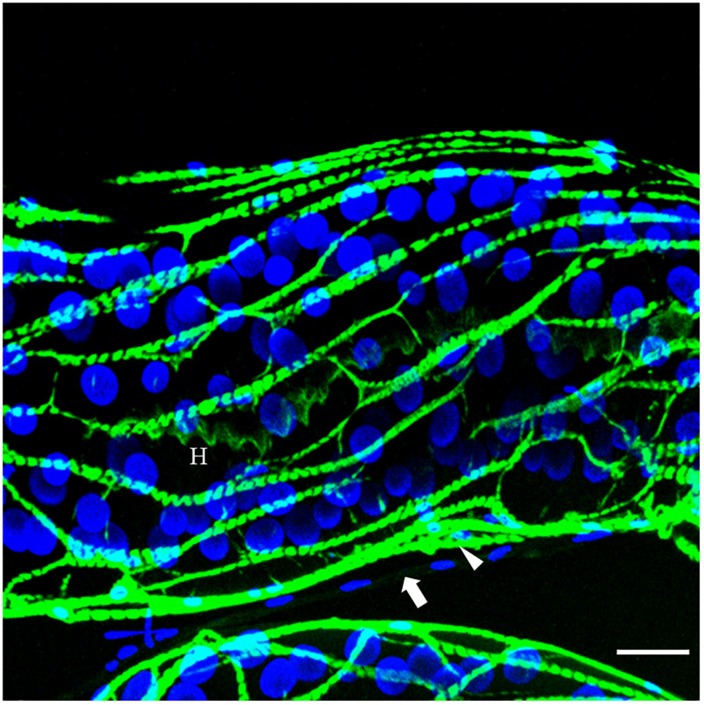
The venom gland of *D. rosae* observed in confocal microscopy. Merge image of FITC-conjugated phalloidin staining for actin detection (green) and DAPI staining for detection of nucleic acids (blue). H, internal helix containing actin; arrow, surrounding mesenchymal tissue; arrowhead, muscular fiber. Note the round shape of the nuclei of glandular cells. Scale bars = 30 μm.

The venom apparatus of *D. rosae* consists of a long and branched gland (approximately 400 μm long) whose central canal is supported by an internal helix containing actin (Figure 4). No ductules or reservoir are visible. Each glandular cell contains a large and round nucleus. The gland epithelium is surrounded by a loose network of skeletal muscular cells. Surrounding the muscular fibers is a mesenchymal tissue, the cells of which have fusiform nuclei. The venom could not be distinguished from saline buffer after rupture of the venom gland, indicating a lower density than venom of *B. pallida*, and neither did it contain spherical particles (data not shown).

### Gall Wasp Ovary and Venom Gland 454 Sequencing Statistics and *de novo* Assembly

The 454 run provided a total of 292784 and 304506 reads respectively for *D. rosae* and *B. pallida* samples. Quality control using SnoWhite software resulted in a total of 287438 cleaned reads for *D. rosae* and 298180 reads for *B. pallida*, with average sequence lengths of 336 bp and 369 bp, respectively (Table 1).

**TABLE 1:** General features of the *D. rosae* and *B. pallida* transcriptomes.

**Species**	***D. rosae***		***B. pallida***	
**Tissue**	**Ovary**	**Venom gland**	**Ovary**	**Venom gland**
Number of reads	163275	129509	182605	121901
	292784		304506	
Number of cleaned reads^a^	160430	127008	178899	119281
	287438		298180	
Average cleaned read length (bp)	343	326	368	370
	336		369	
Number of unique sequences assembled	64627		61682	
Number of contigs	2061		2304	
Number of reads assembled into contigs	224872		238802	
Range of coverage of contigs (reads)	2–17971		2–11614	
Contigs size range (bp; mean)	15–1350 (330)		15–1390 (339)	
N50 of contigs (bp)^b^	447		462	
Number of contigs with a hit (with no hit)^c^	1117 (944)		1253 (1051)	

**^*a*^Adapters, primers, very short reads (<15 bp), and low-quality reads were removed using SnoWhite software. ^*b*^N50 size is the longest contig such that at least half of the total size of the contigs is contained in contigs larger than this value. ^*c*^Contigs which present a hit in the NCBI nr database with the blastx approach with an E-value < 10^–4^.**

To obtain an optimal assembly, sequences from ovary and venom gland cDNA libraries from the same species were merged together, using parameter settings that allowed us to obtain large contigs and to avoid chimeras (see Materials and Methods). The cleaned reads were assembled into 2061 contigs for *D. rosae* and 2304 contigs for *B. pallida* (Table 1). Similar high quality of sequencing and assembly was obtained for both species with contig sizes of an average length of 330 and 339 bp (N50 447 and 462 bp) for *D. rosae* and *B. pallida*, respectively (Table 1 and Supplementary Notes S1, S2).

### Initial Annotation and Origins of Expressed Genes

Contig annotation was performed by blastx comparison with sequences in non-redundant public databases using an *E*-value cut-off of < 10^−4^ and a match was identified for 54 % of contigs from each wasp species (1117 contigs from *D. rosae* and 1253 contigs from *B. pallida*) (Table 1). The *E*-value of most contigs from both species ranged from 10^−15^ to 10^−50^ and 35% of contigs from *D. rosae* and 32% of contigs from *B. pallida* shared very high similarities with sequences in databases (*E*-value < 10^−50^) (Supplementary Figures S1a,c).

For both *D. rosae* and *B. pallida*, 97% of matched contigs were identified as insect sequences, primarily hymenopteran (Supplementary Figures S1b,d) as expected. Interestingly, among the non-matching contigs, two translated sequences coding for cellulases were similar to gene products from bacteria (58% similarity).

### Relative Expression Levels of Contigs in Different Tissues Within Species

**FIGURE 5:**
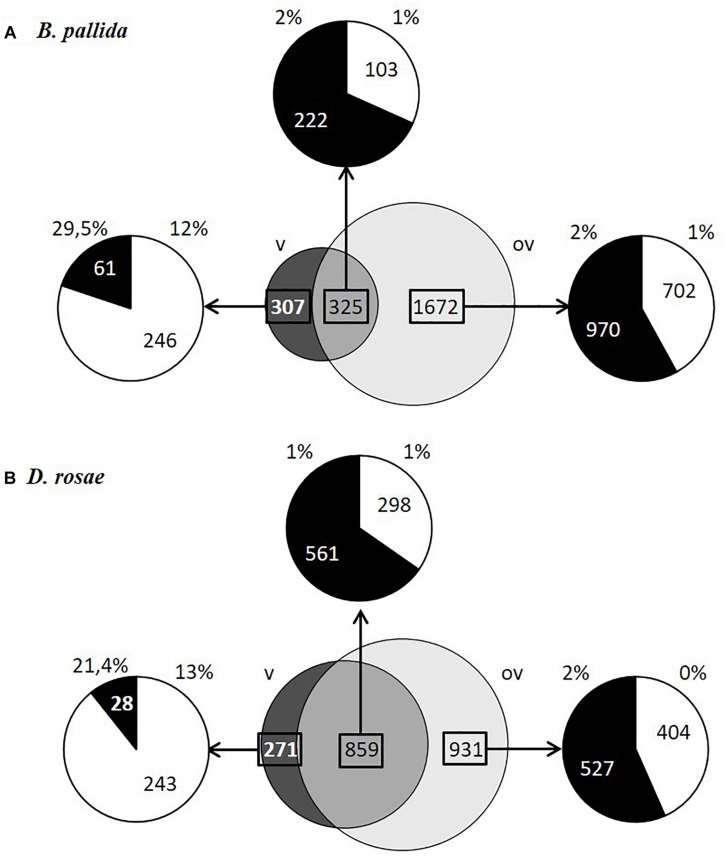
Contig expression levels in ovaries and venom glands and percentage of contigs encoding proteins with signal peptides in *B. pallida* **(A)** and *D. rosae* **(B)**. In dark gray, contigs expressed at least 20 times higher in venom glands than in ovaries (v); in light gray, contigs expressed at least 20 times higher in ovaries than in venom glands (ov); in gray, contigs expressed in both ovaries and venom glands with relative expression levels differing by less than 20-fold. The distribution of contigs that had a blastx hit (black) or that are not assigned (NA; white) and the percentage of these contigs coding proteins with a signal peptide (SignalP4.0 prediction) are specified for each category of contigs.

For each species, contigs could be divided into three categories based on their expression levels normalized using RPKM: (i) contigs expressed at least 20 times higher in venom glands than in ovaries (dark gray in Figure 5) – reported below as “differentially expressed in venom glands”; (ii) contigs expressed at least 20 times higher in ovaries than in venom glands (light gray, Figure 5) – reported below as “differentially expressed in ovaries”; and (iii) contigs expressed in both ovaries and venom glands with relative expression levels differing by less than 20-fold (intermediate gray in Figure 5). This latter category, which contains mostly housekeeping genes, is reported below as “equivalently expressed” in venom glands and ovaries. In contrast, the two differentially expressed categories represent transcripts of candidate genes coding for potential virulence factors, which in parasitoids are generally abundantly produced (either in venom gland or in the ovaries depending on the factor) and injected in large amounts into the host. The same proportion of genes (13 % of the total number of analyzed contigs) was found to be differentially expressed in venom glands of both species. The vast majority of contigs differentially expressed in venom glands of *B. pallida* and *D. rosae* were novel sequences with no significant sequence similarity with known gene products (respectively 80 and 89% of contigs) (Figure 5).

### Gene Ontology Classification of Contigs

**FIGURE 6:**
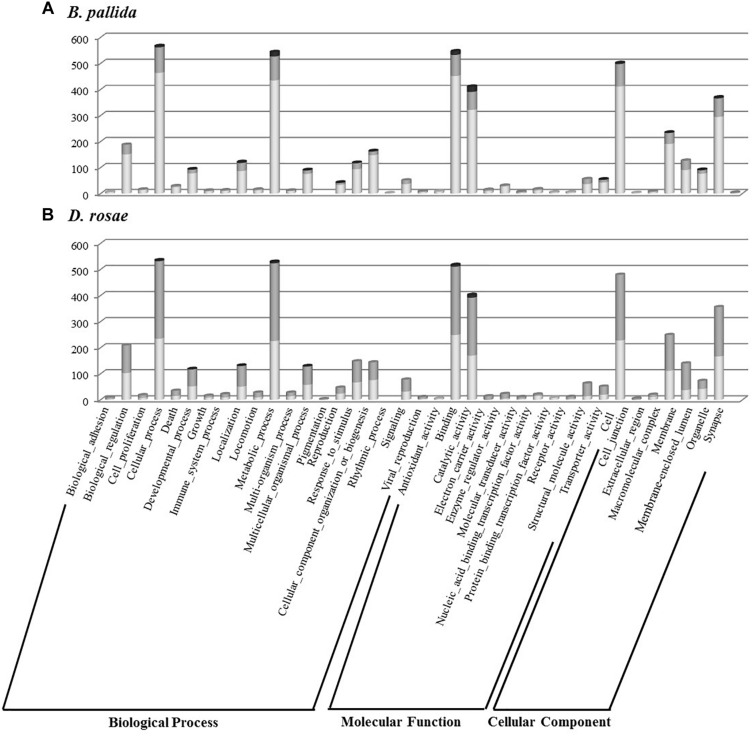
Histogram presentation of Gene Ontology (GO) classification of *B. pallida* **(A)** and *D. rosae* **(B)** contigs. Results are given in the three GO categories at the level two of GO analysis for contigs highly expressed in venom gland (dark gray), those highly expressed in ovaries (light gray), and those equivalently expressed in the two tissues (gray).

Gene ontology assignments were used to classify the functions of the gall wasp transcripts into biological processes, molecular functions, and cellular components for both species and for each relative expression level category (Figure 6). Contigs showing a blast hit (807 and 844 contigs, respectively for *D. rosae* and *B. pallida*) could be categorized into 19 different GO functional groups for each species. Globally, both species showed a similar profile of GO annotation, with high numbers of contigs mainly involved in “cellular” and “metabolic” processes and “global cell structure,” as well as “binding” and “catalytic” activities.

### Identification of Potential Virulence Factors

To identify potential virulence effectors more likely involved in gall induction we focused on genes coding for potentially secreted proteins, since virulence factors are likely to be found among secretion products of venom glands or ovarian epithelia, based on what we know about parasitoids. We therefore considered here proteins that are expected to be secreted by the classical ER-Golgi secretory pathway and do not contain a transmembrane domain that would suggest they are anchored to the cell surface (see Materials and Methods). Since numerous transcripts lacked the 5′ end and hence the region potentially encoding a signal peptide, we also retained proteins showing homology to known secreted proteins present in the databases. A total of 167 *B. pallida* contigs and 134 *D. rosae* contigs encode proteins containing a signal peptide or which are homologous to proteins known to contain a signal peptide. In both species, a higher proportion of genes potentially encoding proteins with predicted signal peptides was expressed in venom glands compared to ovaries (Figure 5), reflecting the important secretory function of the glandular epithelium of venom glands. In *B. pallida*, among contigs differentially expressed in the venom glands at least 29.5% of the contigs and 12% of contigs with no match in databases encode proteins with predicted signal peptides, which therefore likely correspond to venom proteins. By comparison, only 1 or 2 % of contigs belonging to the other categories coded for proteins with a signal peptide (Figure 5A), suggesting that ovaries are less actively secretory than venom glands (which could be expected since egg production is their major function). Similarly, in *D. rosae*, 21.4 % of the identified contigs and 13% of non-assigned contigs differentially expressed in the venom glands encode proteins with predicted signal peptides, compared to only 1 or 2 % for the other categories (Figure 5B). Interestingly, the DeepLoc1.0 analysis of the deduced sequences from contigs differentially expressed in venom glands from *B. pallida* and *D. rosae* confirmed a probable extracellular addressing for 38 of the 46 contigs predicted to encode a peptide signal for *B. pallida* and for 12 out of 17 contigs for *D. rosae* (Tables 2, 3). It is noteworthy that *B. pallida* unassigned contigs differentially expressed in venom gland share no similarity with *D. rosae* contigs, except for one sequence (coding for a protein with a putative DNA binding domain -Pfam 05485-) belonging to the “equivalently expressed” category in *D. rosae*, indicating that most of the corresponding proteins are species-specific. In the following sections, we consider possible candidate proteins putatively secreted by venom glands and ovaries in turn.

**TABLE 2:** Biorhiza pallida contigs differentially expressed in venom glands^a^.

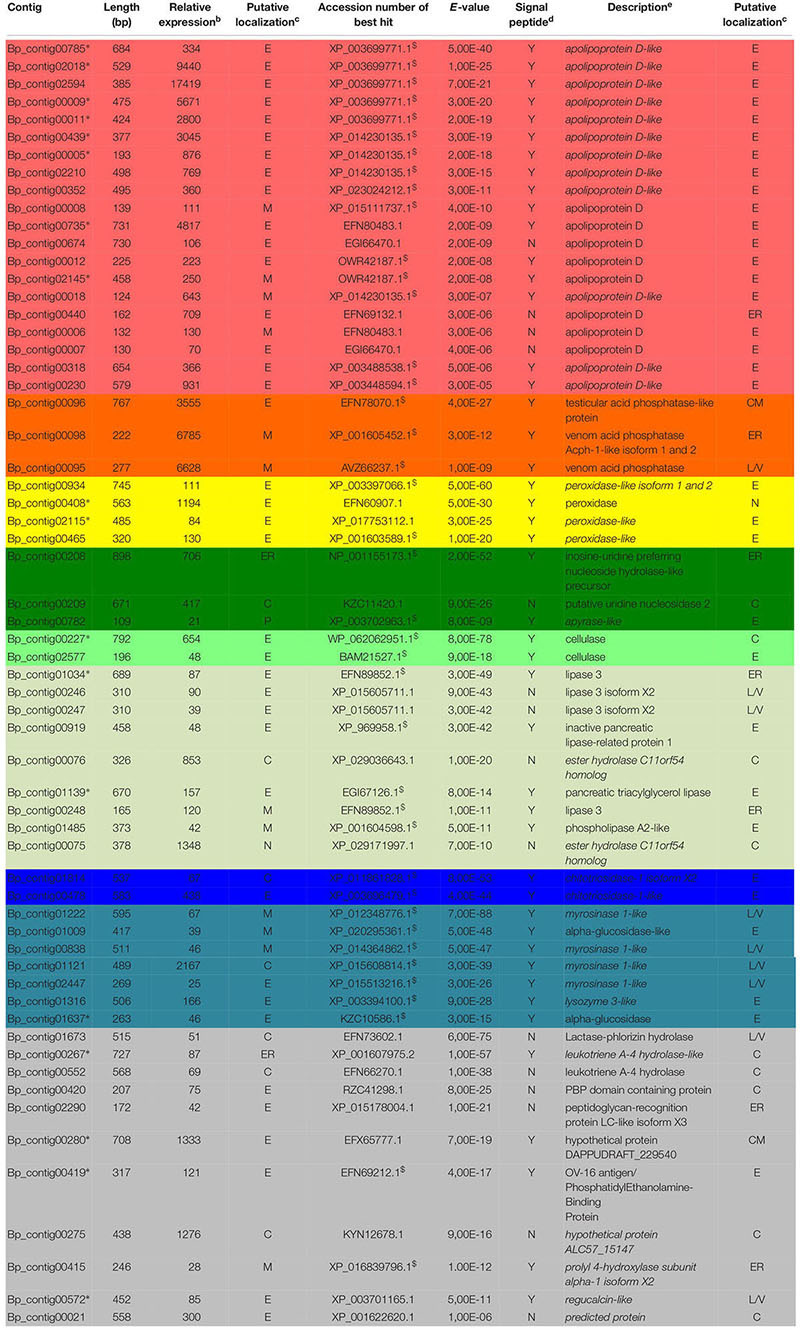

**FIGURE 7:**
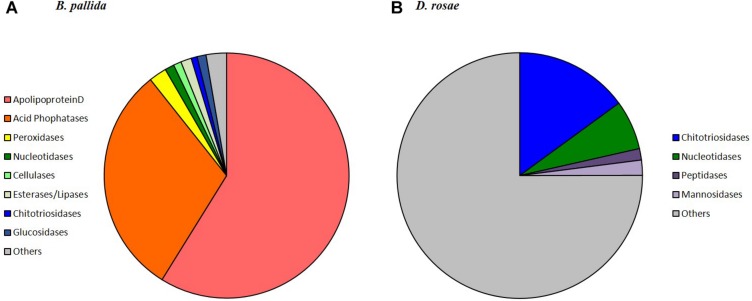
Relative expression and annotation of *B. pallida* **(A)** and *D. rosae* **(B)** contigs differentially expressed in venom glands and potentially coding secreted proteins. Contigs considered here were only those returning a significant blastx hit (*E*-value < 10^−4^) and having a predicted signal peptide or homologous to a protein possessing a signal peptide (contigs indicated by Y in Tables 2, 3 from SignalP4.0 prediction). For each category, the sum of the reads (RPKM) in venom gland was calculated. For detailed description of contigs see Tables 2, 3.

^*^Contigs coding for a signal peptide according to SignalP 4.0. ^$^ Best hits coding for a signal peptide according to SignalP 4.0. ^*a*^ The annotation of *B. pallida* contigs was performed using a blastx approach (*E*-value < 10^–4^). ^*b*^ Ratio between normalized (RPKM) contig expression in venom gland and ovaries. ^*c*^ Putative localization predicted using DeepLoc-1.0 : C, cytoplasm; CM, cell membrane; E, extracellular; ER, endoplasmic reticulum; L/V, lysosome or vacuole; M, mitochondrion; N, nucleus; P, plastid. ^*d*^ Contig or best hit coding for a signal peptide. ^*e*^ Gene names in italics are predicted description. Colors refer to functional groups defined in Figure 7.

**TABLE 3:** Diplolepis rosae contigs differentially expressed in venom glands^a^.

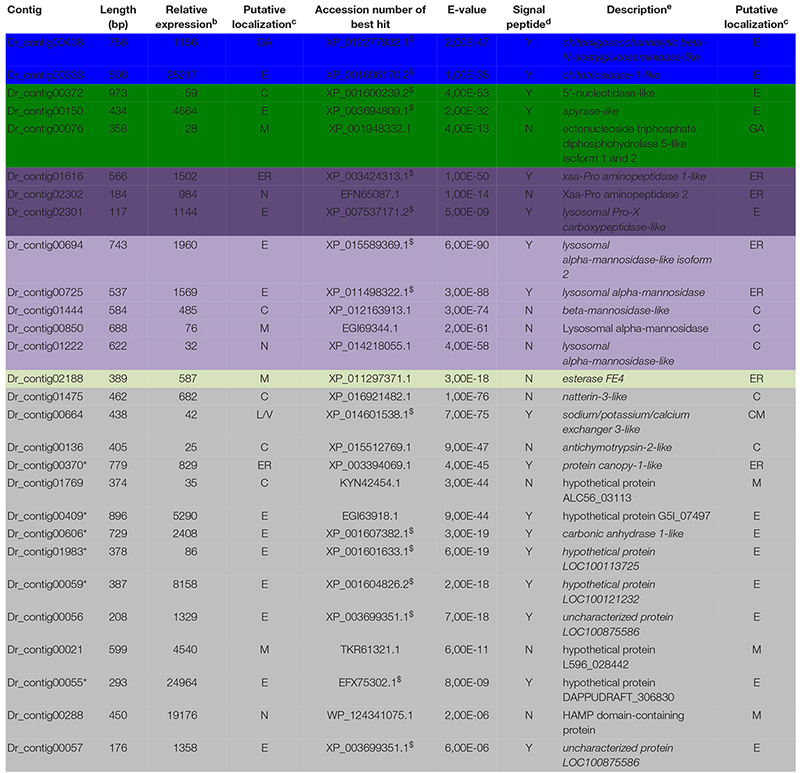

^*^Contigs coding for a signal peptide according to SignalP 4.0. ^$^ Best hits coding for a signal peptide according to SignalP 4.0. ^*a*^ The annotation of *D. rosae* contigs was performed using a blastx approach (*E*-value < 10^–4^). ^*b*^ Ratio between normalized (RPKM) contig expression in venom gland and ovaries. ^*c*^ Putative localization predicted using DeepLoc-1.0 : C, cytoplasm; CM, cell membrane; E, extracellular; ER, endoplasmic reticulum; GA, Golgi apparatus; M, mitochondrion; N, nucleus. ^*d*^ Contig or best hit coding for a signal peptide. ^*e*^ Gene names in italics are predicted description. Colors refer to functional groups defined in Figure 7.

### Identification of Potential Virulence Factors Differentially Expressed in the Venom Transcriptome of *B. pallida*

For *B. pallida*, 307 contigs (61 annotated) were differentially expressed in venom glands relative to ovaries. Of these, 46 potentially encoded amino acid sequences with a predicted signal peptide or with significant sequence similarity to known secreted proteins (Figure 7A and Table 2). These contigs are grouped according to functional annotation in Table 2. They encoded proteins mainly involved in transport, hydrolytic processes and protection against oxidative stress (Table 2). Genes encoding Apolipoprotein D (ApoD), a fatty acid transport protein, had the highest levels of expression with 20 different contigs displaying up to 75889 RPKM in venom glands. Genes encoding acid phosphatases were also highly expressed (up to 65960 RPKM for Bp_contig00098, whose expression was 6785 times higher in venom glands than in ovaries). The third most expressed category of genes encodes secreted peroxidases (up to 6168 RPKM). Other highly expressed contigs in venom gland matched genes whose products are potentially involved in nucleotide hydrolysis (two nucleoside hydrolases and one apyrase), plant tissue and glucoside degradation (two cellulases, two β-glucosidases, four myrosinase-like proteins), fatty acid degradation (at least seven esterases or lipases and a phospholipase A2-like enzyme) and peptide hydrolysis (two leukotriene A-4 hydrolases). Genes involved in potential protection mechanisms against bacteria and fungi were also identified, including a lysozyme 3-like enzyme and a chitotriosidase 1-like enzyme of the GH18 chitinase superfamily. Several genes were identified that code for proteins with potential involvement in regulation of plant signaling, including an OV-16 antigen/phosphatidylethanolamine binding protein (PEBP) and a gene encoding a regucalcin-like protein potentially regulating Ca^++^ signaling. In contrast, ‘housekeeping’ genes involved in general cell metabolism were mainly equivalently expressed between venom glands and ovaries.

### Identification of Potential Virulence Factors Differentially Expressed in the Venom Gland Transcriptome of *D. rosae*

In *D. rosae*, 271 contigs were differentially expressed in venom glands, most of which potentially code for proteins with no similarity to known proteins in databases and only 28 of which could be annotated. Of these, 17 contigs code for proteins with a predicted signal peptide or with high similarity to proteins possessing a signal peptide (Table 3). The global pattern of gene expression in the venom gland of *D. rosae* differs from patterns observed *in B. pallida* (Figure 7B and Table 4) – in particular, the cellulase genes whose transcripts were detected in the *B. pallida* venom gland were not detected in *D. rosae*. However, some venom gland transcripts were differentially expressed in both *B. pallida* and *D. rosae*, including one encoding a chitotriosidase-1-like enzyme (25217 RPKM), one an esterase (587 RPKM) and one encoding an apyrase (4664 RPKM), all of which were important components of the *D. rosae* venom gland transcriptome.

**TABLE 4:** Comparison between *B. pallida* and *D. rosae* sequence*s* with known venom components from parasitoids^a^.

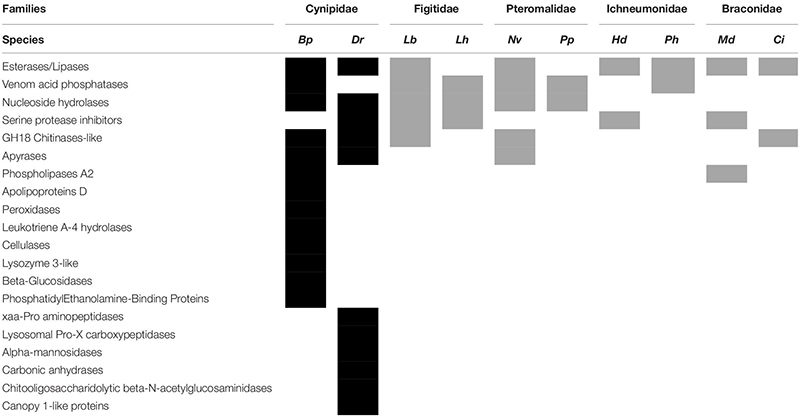

*^*a*^ The most expressed sequences in venom glands from *B. pallida* and *D. rosae* (black cells) were compared to sequences expressed by venom glands of parasitoid species using a tblastx and a blastx approach. Only significant results concerning proteins with identified functions or domains are shown here, using a coding system to indicate presence (gray cells) or absence (white cells) of similar sequences in available transcriptomes and/or proteomes. *Bp: B. pallida; Ci: Chelonus inanitus; Dr: D. rosae*; *Hd*: *Hyposoter didymator*; *Lb*: *Leptopilina boulardi*; *Lh*: *Leptopilina heterotoma; Md: Microplitis demolitor; Nv: Nasonia vitripennis; Ph: Pimpla hypochondriaca; Pp: Pteromalus puparum.**

We also observed a different type of nucleotide hydrolase (ectonucleoside triphosphate diphosphohydrolase) to that observed in *B. pallida*, expressed at a relatively low level in *D. rosae* venom glands (28 RPKM). In *D. rosae*, 28.5% of the highly expressed venom gland genes identified were similar to genes encoding lysosomal enzymes (α-mannosidase, α-mannosidase-like isoform 2, α-mannosidase-like, β-mannosidase-like, chitooligosaccharidolytic beta-N-acetylglucosaminidase-like and Pro-X carboxypeptidase-like), some of these genes reaching up to 1960 RPKM. A contig differentially expressed in the venom glands of *D. rosae* (829 RPKM) codes for a protein with a predicted peptide motif that shares similarity with Seele, a saposin-like protein of the Canopy1 family. A second contig (25 RPKM), for which no putative peptide signal could be detected, corresponds to an antichymotrypsin-2-like protein, a member of the serine protease inhibitor family. In addition, 28.6% of the highly expressed genes of the venom gland transcriptome correspond to eight contigs coding for potentially secreted proteins without any known function (the most expressed reaching 24964 RPKM).

### Identification of Potential Virulence Factors Differentially Expressed in Ovaries of *B. pallida* or *D. rosae* or Classified in the Category “Equivalently Expressed”

Genes equivalently expressed in ovary and venom gland or those mainly expressed in ovary generally encode proteins involved in global cellular metabolism. However, in *B. pallida* transcriptomes, we found four contigs corresponding to genes coding for proteins previously identified as virulence factors in some parasitoid wasps (Supplementary Table S1). Three of them (carboxypeptidase B-like, venom acid phosphatase Acph-1-like isoform 1, putative alpha-N-acetylgalactosaminidase) were more expressed in venom gland than ovary, but by less than our chosen threshold for differential expression. The fourth contig codes for a protein similar to venom protein 5 from the ant *Brachymyrmex patagonicus* and was most expressed in the ovary. This suggests that the 20-fold difference in expression level is a very stringent threshold and that some virulence factors might remain in the “equivalently expressed” category.

Similarly in *D. rosae*, 73 contigs (4% of the contigs which are not differentially expressed in the venom gland) satisfy our criteria of potentially secreted virulence factors (Supplementary Table S2). Of these, two corresponded to gene products encoding members of the GH18 chitinase-like superfamily, classified in the category “equivalently expressed” in venom gland and ovary but more expressed in the venom gland. Five other contigs, more expressed in the ovaries (over or below the threshold) coded for proteins with sequence similarities to one venom serine carboxypeptidase-like, one venom carboxylesterase-6-like, one phospholipase A2-like, and two exonuclease 3′-5′ domain-like 2, that could correspond to virulence factors secreted by the ovaries.

### Comparison Between Gall Wasp and Parasitoid Wasp Venom Gland Transcriptomes

The 307 and 271 contigs differentially expressed in venom glands of *B. pallida* and *D. rosae*, respectively, were compared to venom sequences for parasitoid wasps from four different families to identify similarities and differences between gallers and parasitoids. The comparison identified seven groups of putative gall wasp venom proteins that are functionally related to gene products from parasitoid venom glands (Table 4). Esterases/lipases, nucleoside hydrolases, serine protease inhibitors, GH18 chitinase-like enzymes and acid phosphatases are shared venom components in both cynipid gall wasps and the selected parasitoids. The studied gall wasps also expressed apyrases or phospholipase A2 in their venom glands that are similar to sequences from the pteromalide *N. vitripennis* or the braconid wasp *M. demolitor*, respectively (Table 4).

This comparison also confirmed the novelty of most of the contigs differentially expressed in venom glands of both gall wasps (Table 4: eight groups of proteins for *B. pallida* and six groups for *D. rosae*), which lacked functional equivalents in previously described parasitoid venoms, including *L. boulardi* and *L. heterotoma*, in the family Figitidae, sister group to the Cynipidae within the Cynipoidea (Ronquist et al., 2015).

## Discussion

This study presents the first global transcriptome data for the venom gland and ovary of two gall-inducing cynipid wasps, *B. pallida* and *D. rosae* and constitutes a first step toward identifying virulence factors associated with cynipid gall induction. We have compared these new data with data for closely related Figitid parasitoid wasps, given their phylogenetic proximity to gall wasps, in order to identify on the one hand factors that are potentially conserved between insect and plant parasites and on the other hand factors that are specific to plant-parasitic gall-wasps.

Parasitism is a complex trait and involves multiple mechanisms that act in combination to overcome host defenses and to manipulate host physiology. In parasitoid Hymenoptera, manipulation of host physiology generally starts at oviposition and is mainly controlled by secretions of the female venom glands and/or ovaries, injected with the eggs during oviposition, which can contain symbiotic viruses or virus-like particles produced by the female wasp (Strand and Pech, 1995; Moreau et al., 2009; Drezen et al., 2017). Our goal was to evaluate whether any of the proteins corresponding to genes differentially expressed by the venom glands or ovaries of the two gall inducing wasps studied here could potentially play a defensive role against natural enemies and/or be involved in interactions with molecules from their host plants. We suggest at least three main roles for the venom proteins of *B. pallida* and *D. rosae*, based on identification of putative components: i) controlled lysis of host plant tissues by venom hydrolases, providing space and food for the larvae after hatching from the egg, ii) moderation of the host plant response in reaction to tissue lysis and iii) defense of gall wasp eggs against antagonistic microbes that could benefit from a lowering of plant defenses to invade the newly colonized host tissues, and particularly the fungi that can cause high mortality in cynipid galls (Taper et al., 1986; Taper and Case, 1987; Wilson, 1995; Wilson and Carroll, 1997).

### Venom Components That Potentially Contribute to Controlled Plant Cell Lysis

Our transcriptomic study revealed that the contigs highly expressed in the venom glands of *B. pallida* and *D. rosae* were almost all classified in the GO categories “metabolic” and “cellular” processes, “binding” and “catalytic” activities. Over-representation of the two latter categories has already been reported for genes expressed in the venom gland of a braconid parasitoid wasp *C. inanitus* (Vincent et al., 2010). It confirms that the venom arsenals of the two gall-inducing cynipids comprise enzymes, including hydrolases that are able to interact with a wide diversity of ligands. In *B. pallida*, the main hydrolases expressed in the venom glands were acid phosphatases, which have also been reported from a range of parasitoid wasps venoms (Table 4; Dani et al., 2005; Moreau and Guillot, 2005; Zhu et al., 2008; Manzoor et al., 2016). Since venom acid phosphatases are thought to play a role in cell histolysis and degeneration of targeted tissue (Zhu et al., 2008), we hypothesize that venom acid phosphatases in *B. pallida* may be involved in lysis of plant tissue. This could contribute to the observed collapse of plant tissues around cynipid eggs that precedes hatching, and which provides a space into which the emerging larva moves. The same function could apply to observed high expression of a cellulase (β-1,4-endoglucanase) gene in the venom glands of *B. pallida*, an enzyme involved in the degradation of cellulose plant cell walls. Genes coding ester hydrolases and lipases were also highly expressed in the venom gland of *B. pallida* and, to a lesser extent, also of *D. rosae*. These hydrolases are involved in fatty acid hydrolysis, and have been found in the venom of several parasitoid wasps (Table 4). Lipases and/or esterases in the venoms of the cynipid gall wasps could play a role in altering host plant tissues. In addition, we have observed high expression of a cellulase (β-1,4-endoglucanase) gene in the venom glands of *B. pallida*, an enzyme involved in the degradation of cellulose plant cell walls.

Cellulase has already been characterized in salivary secretions of root-knot cyst nematodes (Davis et al., 2004), but this is the first report of its presence in venom. In nematodes, the acquisition of this gene has been clearly shown to involve horizontal gene transfer (Danchin et al., 2010). In terms of mRNA abundance, myrosinase-like enzymes are among the most expressed venom gland products in *B. pallida*. Myrosinase enzymes are β-glucosidases with thioglucosidase activity, that are involved in protection of cruciferous plants against herbivores, glycosinolate hydrolysis producing compounds toxic for caterpillars (Rask et al., 2000; Burow and Halkier, 2017). Since oaks do not use the glycosynolate defense system one may reasonably consider that these enzymes more likely correspond to β-glucosidases and may provide readily available sugar to the larvae. Other hydrolases were found expressed by the venom gland of *D. rosae*, including several chitooligosaccharidolytic, proteolytic and peptidolytic enzymes. We also found expression of a natterin-3-like protein by the venom glands of *D. rosae*, suggesting a possible evolutionary convergence with the venom of a fish *Thalassophryne nattereri* (Magalhães et al., 2005). However, SignalP 4.0 did not identify a signal peptide in the amino acid sequence encoded either by the corresponding contig or the best hit sequence identified by Blastx comparison, so there is no indication yet that this protein is secreted.

### Venom Components That Potentially Moderate Plant Responses to Damage

*Biorhiza pallida* and *D. rosae* venom glands express a second category of molecules, whose products appear to be involved in modulating the defense reactions of the host plant. Such a process is crucial for the initiation of long-term interactions, as observed for instance in nodulation induced by bacteria in the legume plant symbiosis (Nakagawa et al., 2011). A peroxidase was highly expressed by the venom gland of *B. pallida*, representing to our knowledge the first reported peroxidase in hymenopteran venom. This peroxidase may limit the oxidative stress induced in the oak root following oviposition by the sampled sexual generation of *B. pallida*.

Other putative regulators of plant “early danger signaling” (Dubreuil et al., 2010; Guiguet et al., 2016) identified among the transcripts from the venom glands or the ovaries of both species include an apyrase from *B. pallida*, a peroxiredoxin from *D. rosae* and several 5′ nucleotidases including ectoapyrase, differentially expressed in the venom of both gall wasps. The ectoapyrase could play a role in breaking down extracellular ATP (released by the action of venom hydrolases on plant cells), which is known to induce some plant defenses (Tanaka et al., 2010; Heil et al., 2012).

We also noted expression by the *B. pallida* venom gland of a regucalcin-like protein, similar to a regucalcin in the saliva of pea aphids (Carolan et al., 2009). In pea aphids, the regucalcin binds calcium ions to prevent calcium-mediated shut down of damaged phloem sieve tube elements, allowing prolonged aphid feeding (Carolan et al., 2009), *B. pallida* regucalcin could regulate oak Ca^++^ level, although its precise role is difficult to predict (Stafford-Banks et al., 2014). The venom gland of *D. rosae* expresses the genes of two proteins able to interact with serine proteases, including a serpin, which belongs to a superfamily of irreversible inhibitors of serine proteases. Serpins have been described from several parasitoid venoms (Table 4), in which they prevent triggering of host immune responses (Moreau and Asgari, 2015). It is possible that the gall wasp serpin functions in an analogous way in the plant host. In plants, serpins are thought to have a role in the complex pathways involved in up-regulating the plant immune response (Habib and Fazili, 2007).

### Venom Components That Potentially Contribute to Defense of Gall Wasp Eggs Against Antagonistic Microbes

The third important role that venoms of gall wasps seem to fulfill is protection against opportunistic pathogens. Antimicrobial properties of venoms are among the most conserved and widespread properties of hymenopteran venoms (Moreau, 2013), presumably because bacteria and fungi may compromise the quality of larval habitats (nest or host organisms), thus reducing the reproductive success of the species. Venom glands of both species differentially expressed genes coding for chitotriosidase 1-like enzymes of the GH18 chitinase superfamily, a group of chitinases already described in the venoms of several parasitoid wasps (4). In plants, chitotriosidase 1 degrades fungal chitin into monomers of *N*-acetyl-D-glucosamine, which seems to be the least effective compound for elicitation of an immune response (Kasprzewska, 2003; Cabrera et al., 2006; Hamel and Beaudoin, 2010). Degradation of fungal chitin by gall wasp venom chitinases could thus fulfill the dual functions of protecting the egg from direct fungal attack, and preventing a strong host plant defensive response.

Other venom-gland expressed genes may target gall wasp vulnerability to, and plant defenses against, bacteria. Transcripts of a *lysozyme 3-like* gene were identified in the *B. pallida* venom gland transcriptome. Lysozyme is an important component of the insect immune response against bacteria, characterized by its ability to break down bacterial cell walls. We also identified expression of an α-mannosidase gene in the venom gland of *D. rosae.* Mannose is a major sugar of bacterial cell walls and can elicit plant defense responses via the ROS pathway (Hwang and Hwang, 2011). Gall wasp α-mannosidase could limit the triggering of such defenses at the oviposition site, whilst degrading the outermost layers of challenging bacteria.

### Contrasts in Venom Gland Structure and Gene Expression Patterns Between *B. pallida* and *D. rosae*

While there are some similarities in the venom gland molecular repertoire of the two gall wasp species studied here, they differ substantially from each other and also from parasitoid wasps (Table 4). The two gall wasps also differ substantially in the structure of their venom glands. We hypothesize that these differences at one level reflect deep phylogenetic divergence between *Diplolepis* and *Biorhiza* within the Cynipidae (Ronquist et al., 2015), and further reflect differences in host plants and plant organs galled. One of the most striking findings of this study is the apparently high production of apolipoprotein D (ApoD) in venom glands of *B. pallida* but not *D. rosae.* ApoD could be involved in the production of the abundant spherical particles we observed in *B. pallida* venom (Figure 3) that are responsible for its high viscosity, resembling plasmatic apolipoprotein particles observed in vertebrate models (Bergeron et al., 1998). In animals, ApoD is involved in the transport of fatty acids, steroids and other hormones (Rassart et al., 2000). ApoD has also been detected in some plants, where it plays an important function in defense against oxidative bursts (Charron et al., 2008). We speculate that the gall wasp venom ApoD could facilitate transport of fatty acids for the newly hatched larva, be part of a transport system for an as-yet unidentified vesicle-delivered gall wasp product, or play a role in regulation of oxidative stress in the newly colonized plant tissues.

Our microscopic observations have confirmed Vårdal’s (2006) observation that, relative to the other Hymenoptera of similar size, *B. pallida* has an exceptionally large venom apparatus. Fluorescence microscopy revealed the existence of a secondary secretory zone located on the dorsal side of a large reservoir, both of which structures are absent from *D. rosae*. Future investigations will help to determine whether the secondary secretory zone is where the abundant spherical particles in *B. pallida* venom are produced. In *D. rosae*, instead of “a small sac-like structure” described by Vårdal (2006), we found in all the examined specimens a long and branched gland connected at the base of the ovipositor, the secretory epithelium of which was supported by a central helix of chitin and lined with muscles. *D. rosae* females (but not *B. pallida*) also possess two small accessory glands that were not included in the sample for transcriptomic analyses. In some gall-inducing sawflies (Symphyta), accessory glands produce gall-growth promoting substances (McCalla et al., 1962; Yamaguchi et al., 2012). Therefore, it would be of interest in future to sequence the transcriptome of the accessory glands of *D. rosae*. Another point of interest is the presence of muscular cells surrounding the reservoir of *B. pallida* and the venom gland of *D. rosae* indicating that venom ejection is probably under the control of a voluntary muscular contraction, in contrast to previous observations suggesting that cynipoids lack reservoir muscles (Vårdal, 2006). After venom ejection, the venom apparatuses could passively return to their initial state either thanks to an internal helix of chitin (in *D. rosae*), or to the high viscosity of the venom (in *B. pallida*).

### Possible Acquisition of Gall Wasp Cellulases by Horizontal Gene Transfer From Bacteria

Another significant finding was the strong sequence similarities between cellulase genes expressed in venom glands and/or ovaries of *B. pallida* and genes of bacterial origin. Previous studies have identified horizontal gene transfer from bacteria as an important route for metabolic innovation in insect herbivores (Wybouw et al., 2016), and the similarity between cellulases in *B. pallida* and bacteria could be explained in this way. Alternative explanations are the production of the cellulases by bacterial endosymbionts or entirely convergent similarity between bacterial and gall wasp cellulases sequences, the latter representing an ancient insect trait lost from some lineages. We consider it unlikely that high expression of cellulase genes in the *B. pallida* venom gland could be due to bacterial endosymbionts because the bacterial sequences in our libraries (all associated with *Wolbachia*) are present only at a low level and mostly in the ovaries. Wider phylogenetic analysis would probably rule out ancestral possession of these genes in insects and other Metazoan lineages. However, the case for a HGT origin would be strengthened by demonstrating that the observed transcripts originate from genes in the cynipid genome (e.g., by demonstrating the presence of flanking genes of definite insect origin and/or presence of introns) and by sequencing flanking regions of the cellulase genes or obtaining a high quality *B. pallida* genome assembly.

The acquisition and venom gland overexpression of a cellulase gene may have been important in the adaptation of gall wasps to parasitism of plants. Why ApoD and (putatively bacterial) cellulases are highly expressed in *B. pallida* but not in equivalent tissues of *D. rosae* remains an open question. However, study of parasitoid wasps has shown that major virulence factors may vary greatly even among congeneric species (Colinet et al., 2013a). Such diversity is probably the result of arms race coevolution between host and parasite, the latter having to shift regularly from one molecular strategy to another to circumvent host resistance (Colinet et al., 2013a).

### No Evidence for a Role of Viral Genes or Viral Particles in the Gall Wasp Venom Gland

We observed that several genes, similar to those encoding lysosomal enzymes, were differentially expressed by venom glands of *B. pallida* and *D. rosae.* This supports the hypothesis that some genes encoding venom enzymes originated by duplication of genes coding for lysosomal or other cellular enzymes (Moreau and Guillot, 2005). Interestingly, in *D. rosae*, several typical venom components were found expressed by ovaries, suggesting that this organ has a secretory function complementary to the venom gland. However, we found no evidence for expression of genes of viral origin or for the production of symbiotic viruses or virus-like particles by the ovaries of the gall wasps studied. This contrasts with several parasitoid lineages in which complex viral machineries are used to deliver virulence factors and to manipulate the physiology of the insect hosts (Herniou et al., 2013; Pichon et al., 2015; Burke et al., 2018; Leobold et al., 2018). However, since the analysis of venom gland transcriptomes from *B. pallida* or *D. rosae* revealed a high proportion (80–89%) of novel sequences compared to venom glands from parasitoid wasps (17% in *H. didymator* and 50% in *C. inanitus*) (Vincent et al., 2010; Dorémus et al., 2013), such unannotated transcripts may comprise as yet unknown virus genes. Indeed, this was initially the case for genes later shown to be involved in symbiotic virus particle production of ichneumonid parasitoids (Volkoff et al., 2010). Ongoing sequencing and annotation of gall wasp and host plant genomes will contribute to future advances in this field. Moreover, successful knock down of a venom protein expression in a parasitoid cynipid wasp using RNA interference (Colinet et al., 2014) suggests that functional analysis could be used to study gall inducer factors after mass spectrometry confirmation of their secretion and of their injection at the oviposition site.

## Data Availability

The datasets generated for this study can be found in the genbank/EMBL and PRJNA517634.

## Author Contributions

SC and OG were involved in the experimental approach, detailed analysis of the data set, and draft manuscript writing. SM was involved in the dissection of venom glands, cDNA library constructions, and sequence analysis. PG provided expertise on bioinformatics analyses. JH and GS provided expertise on oak and rose gallers and DG on plant–insect interactions. EH and J-MD were involved in the project conception and direction, and with GS and SM in final manuscript writing.

## Conflict of Interest Statement

The authors declare that the research was conducted in the absence of any commercial or financial relationships that could be construed as a potential conflict of interest.

## Footnotes

1 http://www.cbs.dtu.dk/services/SignalP/

2 http://www.cbs.dtu.dk/services/DeepLoc-1.0/index.php
