# Supplementary figures and images for: Gall Wasp Transcriptomes Unravel Potential Effectors Involved in Molecular Dialogues With Oak and Rose
Source: Front Physiol. 2019 Jul 24;10:926. doi: 10.3389/fphys.2019.00926 (PMC6667641; doi:10.3389/fphys.2019.00926)

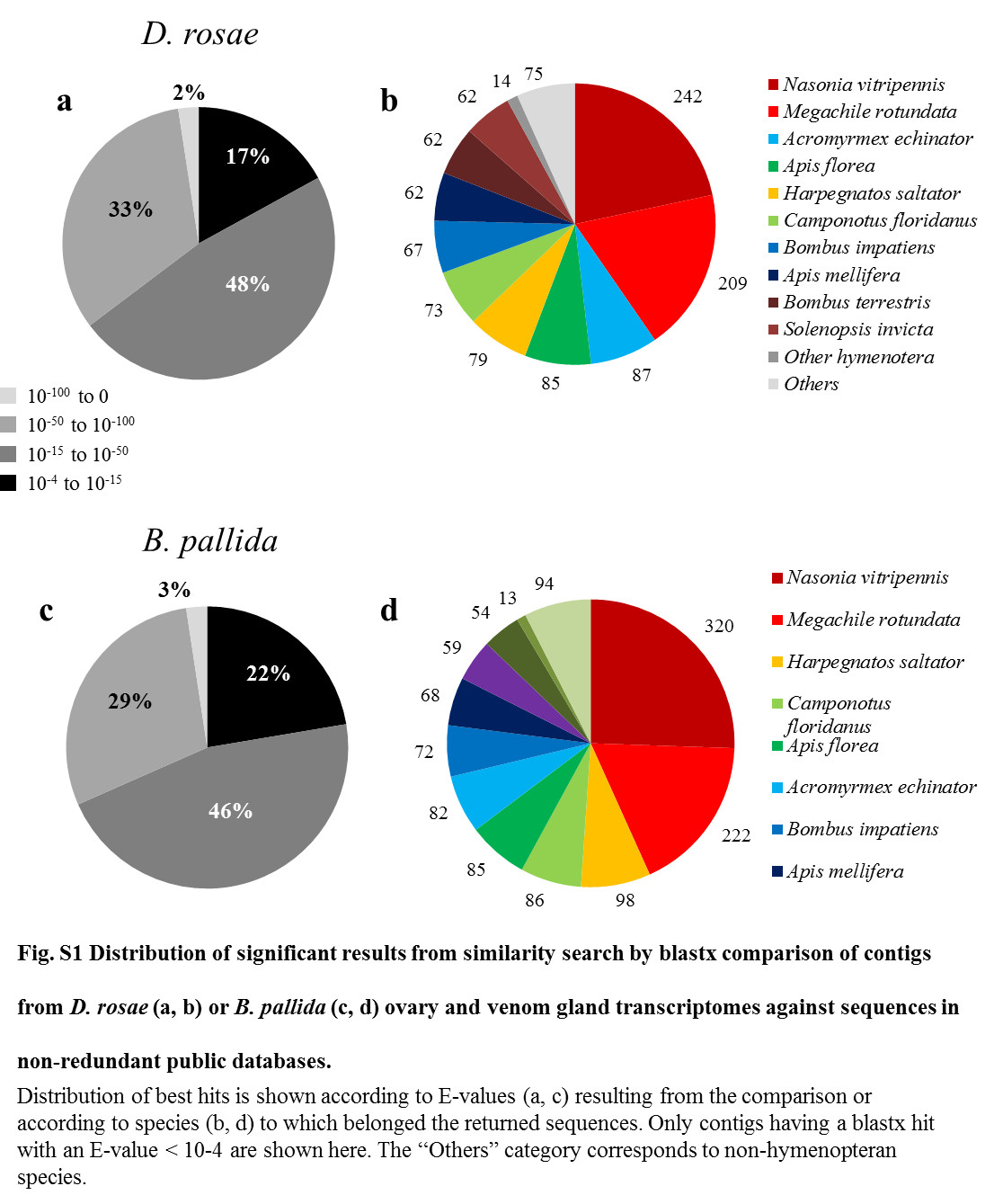

Supplement: Supplementary file 1 [file Image_1.JPEG]
