# Supplementary material for: Gall Wasp Transcriptomes Unravel Potential Effectors Involved in Molecular Dialogues With Oak and Rose
Source: Front Physiol. 2019 Jul 24;10:926. doi: 10.3389/fphys.2019.00926 (PMC6667641; doi:10.3389/fphys.2019.00926)
Supplement: Supplementary file 2 [file Data_Sheet_1.docx]

**Supporting Information**

Article title: Gall wasp transcriptomes unravel potential effectors involved in molecular dialogues with oak and rose bushes

Authors: Sébastien Cambier, Olivia Ginis, Sébastien J.M. Moreau, Jack Hearn, Graham Stone, David Giron, Elisabeth Huguet, Jean-Michel Drezen

**Note S1 All contigs identified from the sequencing of transcriptomes from *B. pallida* venom gland and ovary.**

Sequences are presented in FASTA format.

>contig00001

TtCtAATtAGTAGTTTCTTGTCTTCTAAGTTGGTTTCTTTGTTCTAATTAGTCGTTCTATTAAGTCTTGGTCTTTGTAGTTTGTTTCGTTcttGTTTGGTTGTCCGGTTaTTTCTTCGTCctAAAaTTCCtAAaGTATTTtGTtAGT

>contig00002

ATTTATTtCTTGTAGTtAGTTTTtCgTTCTAATTAGTAGTTTCTTGTCTTCTAAGTTGGTTTCTTTGTTCTAATTAGTCGTTCTATTAAGTCTT

>contig00003

ACCTAAcTAGTtATTCTAGTTTGGTTATTAGTTTTTCTTTTCTATTTACTAATTTTCTTTAAAAAGTATTTTTCTTTATTTGTaCTAAATTTATTTTAACTAAtTAAATTTTTTACGTAATTTCTTTCTATTAAATTCCTATTAACTTTAGTCGTTCGTTTAAAGTCTTGTAAAGT

>contig00004

TGTTGCACAGTATGTAgggCTTTGGTATGAAACacatAAATATGTTGCACTTTATGAacTGTTTGGACAATGCATAACTGCAAAATACACACT

>contig00005

GGCTCAAGTTGTTGATTTTGGAAATTGTCCAGAAGTGACCGTTAAACAGGATTTTGATgTTGCACAGTATGTAGGGCTTTGGTATGAAACAAACAAATATGTTGCACTTTATGAACTGTTTGGACAATGCATAACTGCAAAATACACACTAAATTCAGATGGTTCAGTAGATGTTGTGAACACGCAAATCAAC

>contig00006

GTTGATTTGGGAGTTCACAACATCTACTGAACCATCTGAATTTAGTGTGTATTTTGCAGTTATGCATTGTCCAAACATTTCATAAAGTGCAACATATTTATGTGTTTCATACCAAAGACCTACATACTGTGC

>contig00007

ATGTAGGTGTTTGGTATGAAATAGATAAATATTTTGCATTTtATGAAATGTTTGGACAATGCATAACTACAAAaTACACACTAAATTCAGATGGTTCAGTAGATGTTGTGAACACGCAAATCAACAATAT

>contig00008

ATATTGTTGATTTGCGTGTTCACAACATCTACTGAACCATCTGAATTTAGTGTGTATTTTGCAGTTATGCATTGTCCAAACAGTTCATAAAGTGCAACATATTTGTTtGTTTCATACCAAAGCCCTACATACTGTGCAA

>contig00009

ACCAAGAGTATCAGTTGGTATAGTTTTCTTTCATTACAATTTCTTAAAAAGATTTtCTTATTGACAAATTATTTAACAATAAATTTTTACGAATTCTTCATAAATCCATAACTTAGCGTCGTTAAAGCTGAAAGAATGATTCCCGTTGTATTCCTTCTTCTGACCTGCACTGTAGCTTTGGCTCAAGTTGTTGATTTTGGAAATTGTCCAGAAGTGACCGTTAAACAGGATTTTGATGTTGCACAGTATGTAGGgCTTTGGTATGAAACAAacAAATATGTTGCACTTTATGAAcTGTTTGGACAATGCATAACTGCAAAATACACACTAAATTCAGATGGTTCAGTAGATGTTGTGAACaCgCAAATCAACAATATAACTGGAATAGCATCATCGATTGAAGGCACTGCAAcATTTATTGGTGATAAGAATGTAGGACAACTGAATGTCGTTTATCCAGTCGGTCCCTTCAAtA

>contig00010

ATTTCaTTaCAaTTTCTTAAAAAGATTTtCTTATTGGCAAaTTATTTAACAATACATTTTTACGAATTCTTCATAAATCCATAACTTAGCGTCGTTAAAGCCTGACTTTGAGAACGAAACCTACCCTGATGTAGAATTGTACTCAC

>contig00011

AGTATCAGTTGGTATAGTTTTCTTTCATTACAATTTCTTAAAAAGATTTTCTTATTGACAAATTATTTAACAATAAATTTTTACGAATTCTTCATAAATCCATAACTTAGCGTCGTTAAAGCTGAAAGAATGATTCCCGTTGTATTCCTTCTTCTGACCTGCACTGTAGCTTTGGCTCAAGTTGTTGATTTTGGAAATTGTCCAGAAGTGACCGTTAAACAGGATTTTGATGTTGCACAGTATGTAGGTCTTTGGTATGAAACACATAAATATGTTGCACTTTATGAAATGTTTGGACAATGCATAACTGCAAAATACACACTAAATTCAGATGGTTCAGTAGATGTTGTGAACTCCCAAATCAACAATATAACTGGAATAGCATCATCGATTGAAGGCACTGCAAgATTTATTGGTGAAAGAA

>contig00012

CCCTTCAAAATTCCTGGACCATATTGGATTTTATATACCGATTATGTTGTATCTGTCGTTTACTCCTGTAGTTATGCATTCTTTGTCCATaCTACAAATCTTTGGATTTTGGCGAGAATACCGGCACCAACAGACGAACAACTCAAGCCAGCTTATGACGCTATTGACAAGAACCACTTGAGCAGAACTCTATTGATGAAAACTACTCaGgATAAtaaaTTAAGT

>contig00013

CTtGtCACTTCGTTTATTACAAACTTTGCTCAACCCTATGTGATAGTCTAAAGGCAACGGATCGAGGCACCGATTTGGCTTTTtACCTGACACCCTTTCTTCCATATTGTAGAAGGACAATAgGGAAAGGCTTCTTGTTGTTATTATCCATACAATAAATtCAAACGACAGTAACAATTTACGAATTAAACTCAAACACTTCAAATGTACGACATTCCAGAAAAACAATTTTAATTATTAAATTTTCATGCCGAAAAAGTTAACTTTTTtACAAAAAGAATTACATTTCAAGACGTATTTTCAACTAATAAGACTAATTTTCCTCTGGAAGTGACATTCTTGACCAAACAAAAATAAAAATTTCTACCAAAaGAAATAAATAGTTCTAAAGTCAATTAATTTTCCGAGCAGTATATAATGTTAATTTTTATGTTTTAGCACGTTGAATCTCGTTAATTTGATGAGATATATAAAAAATTTAGAACAAGAACATTATGAAGCTATAGTTAATACTTATGTTTCTATTTCAAATATTATTTTCTGGTTTTtGAATACCAATTGTTAAAGCATATTATTTTtCATTATTTTTTtGGCAGAAATTTTTtAAATGTGAAAATTTTATTTTCCTGGTGTCGTATTTCGCGTACAAT

>contig00014

CCAAGaGtATCAGTTGGTATaGTTTtATTTCATTACAATTTCTTAAAAAGATTTtCTTATTGGCAAa

>contig00015

AACACCGGCACCAACAGACGAACAACTCAAGCCAGCTTATGACGCTATTGACAAGAACCACTTGAGCAGAActCTaTTGATGAAAACTACTCAGAATAATTGT

>contig00016

ACCAAgAGTATCAGTTGGTATAGTTTTCTTTCATTACAATTTCTTAAAAAGaTTTTCTTaTTGaCAAATTATTTAACAATAAATTTTtACGAATTCTTCaTAAATCCATAACTTAGCGTCGTTAAAG

>contig00017

GATGTTGCACAGTATGTAGGTCTTTGGTATGAAACACATAAATATGTTGCACTTTATGAAATGTTTGGACAATGCATAACTGCAAAATACACACTAAATTCAGATGG

>contig00018

ATGTTGCACAGTATGTAGGTCTTTGGTATGAAACACATAAATATGTTGCACTTTATGAAATGTTTGGACAATGCATAACTGCAAAATACACACTAAATTCAGATGGTTCAGTAGATGTTGTGAA

>contig00019

CCCAAATCAACAATATAACTGGAATAGCATCATCGATTGAAGGCACTGCAAGATTTATTGGTGAAAaGAATGTAGGACAACTGAATGTCGTTTAT

>contig00020

TTATTCCAAAAAAaTTATTCCTCTATCTGGAAACAATGAGGGTTCTTTGCATTCTGTTCATCGCTTTAGCGGTTTTCTTGGTTTCTTTTGTTTTCGCCGAAGACCCGTGGGATAAAGTCGAAACAAAAGCAGCCACAGAGCAAATTATAAATGCTGTTAAATTACGATCTGATTCAGATTGCGCAAATACTCTCGAACCTTTTACATATCTTGTGTACACGGGGACTCAAGTCTTTGCATATCTTGATGCACATAGTATACTTCACAAATTATATCAACTTAAGGAAAGTCGCTGCCGCCAGATTGGAAGAAGTACAAAACCGCATTTGATAGGCGCTGAACTTCCACAGTACGTTATTAACCGCAATATCAACCCAAACAAACCTACAATGAACTGGGGAAGAAATCGATTTTGGGCATGGTAATGCGTGACTTTGCTTAAGTGACATCGTTGACATCGTTTATTATATTTATCAACTTAATATTGTAAGTCAATTATAATTTAGTCAAATATCTATTCAATGTCGTATTTCAATTTGACTTTAGATGAAAAaCGATGTGTTAATTGCAATGAGCAAAATATATTCTTCAATCAAATGATTTGCATATATG

>contig00021

GAAGATTAATTCTGGCCCCTATCCTGGCGCTATCAGACAaTTGGAAcTCTGATGAAAAAAAATTTTGAAATGTTAAAATCCTCAGTGGGCTATTAGTGGTGGTATATCTTCACCCTTaGgAAGGAGATGATGGTACAGAAGAGGTTGATGTAACATCTTCTAcACTAGAGGATGGTGGTACATCTCCTTCtTAAGATGAGGATGATACATCTTGAGGCCCAGAAGAATCTGCTaTATCTTCATCTTGAGGATCAATTGGAGACACATCTTAGTGTTTAAATTATTTTTTtAGGCGtAATAaTAGATGCAGGTTTAGGATTGGATGCTGGTATAATTTCGGGCTTAGATTCGGCTGGTGGTACAACTTCGGGCTTAGAAGCGGCTGGTGGTACAACTTCGGGCTTAGAAGCGGCTGGTGGTACAaCTTCGGGCTTAGAAGCGGCTGGTGGTACAACTTCGGCcTTAGAAGCGGCTGGTGGTACATCTGCAtCAGtGGCTGGTGGTACTTCATCTCCTGGTTTAGTTtCGGTTGGTACAACTTGTGTGTCAGAAGGTTTT

>contig00022

TTtAAaTTTTAAAATtGTTTCTTATGTTTACCTTTAGCAAATCATGTAATATGGTTGAGAATAAaGTTAATAGATTTAATATTGATAACCTTTTCAGCCGTTTAACGAACAATACAATATATTGAATATTAAAAATTATGATTTaTATCATTTCTTGTATATTTTTATTATAATTTAATAATTtAACATGAAGCTTATTTTTTTAACGTTAAAGACTTAATCTTTATATTTTACGATTTTACCATTTCACTCTACTTGAAAATTAAAAaCCTTAAAAaCCGATATTTCTATTGTATGTAAGGTCTATTTTATAAACCTAAATATTTTTTTAATTATAGAAACAGTTAATCATTGCTTAATTCAAG

>contig00023

ACTATGGAGTCCCAGAACGTGTATCAGCCATCCTAAAGCTTCACAAGCTGTTTCGCACCTATAGAAAAaTCAATTTCTTTtGTTTGACGAGAAAATGAAGTTCTTTGTTGCTGCTGTGCTTTTCAGCTCTGCGTTGGTTTtCGAATTAGGCTCAACTTTCCCGTGGGAAACACCAGCTGCAACACCAGTTACAGCAGTTTCACCTAAGAAAGAAGAAAGCTCCCTACCCACTTCTGAAGGGTTAGGTGCACC

>contig00024

CccACTAAGAGACACTGAAGTTAAATTTGACGACTGCAGcctAGCTATAATTTACATACAACGAAAAACATTTTCTTATCAAAGGAATCAATTATCTCAGAGCAGTAGTCATATTGCGGCTGACaatAAAGGCATTTAGTCACTTgGAAAAAAAaTAGgAACTGTCAATTTTTAAATATATTTGGCTCAAAGAAGGAAGATCATCTAAAATGAATTCGAAAATGACTACAATTTTCTTAATTTTCTGCATCATCATAGGTGTTTACGCGGCAGACGGGCcAgCTCCAGTGAATCCCAGGAATCAGAAATCAAGAATCCATCGATCAGATATGGACAACC

>contig00025

TTAGAAATTTCTTATGTCGCAGTAGGTGATAATtATTTGGTTGCCTATCATGATGGAGGATCTTCTCCATGACCCTCACCTGGAGCTGgCCCcGTCTGCcGCGtA

>contig00026

AaTcTAAAaTAGTAGCTGGgACGTCAAATCATCAAATACACAATAAGAGACACAGAAGTTAAATGTGACGACTGCAGCCCAGCTATAATTTATATTCAACGTGTCTACGCGGCAGACGAGGCATCTCCAGATGAATCCCAGGAATCAGAAATCAAGAATTCATCGACCAGATATGGAAAACT

>contig00027

CACAAAGTTTTTAGATTTTCCACAAACGTCCAAAATATTACAAAAACTGCGAAAGATGCTAAATCTCCCATGATTCTAATCAGCTTT

>contig00028

GCTGCTGTGCTTTtC

>contig00029

GGTTTtCGAATTAGGCTCAGCTTTCCCGTTGGAAACACCAACTGCAACACCAGTTACAACAGTTCCACCTAAGCAAGAAGAAAGCTCCCTACCCACTTCTGAAGGGTTAAGTGCACCAAAACCTTCTGAGATAGAAGTTG

>contig00030

GATTGAAAaGAACCAACATAGATTTTAAGAGATTTAAAaGAATTTAGGGTATGTATTTTTAAATACTTTAAGGAATTTCAAATATTACAAGAGATATGAAAAGATTCTAAAGATTTTGGGGTATTTtACAAGATTCTAAGAAATTTGTAAGAGCTTTATAAAATTGTAAGCGATTTCAAAAGATTTCATAGGATTTTTGAAACTTTAaGGAGCTTCCGAAAaTTTtAATGATTTGAAGGCATTAAAAAGGATCGATGGGGTTTCAAAGAATATTGGAAGACTGGGAAGTATTTtATAAGaGCTACATGGTTCTGGGATATTTAAAAAGATTCAGAAGGATTTAAATAGATTTTAAAGTATTTTtAAAATTTTATcgTTATTTAAAAGATTCCAAAGAATTTCAATAGATTTTCAGAGATTTtCAAAGATCCTAAAGATTTTGGGgTATTTTAAAATATTGCATAAGATTGTAAAAATTGCATTGAATTTCCTAAGATTTTAGTgATTTAAGTGGATTTAGAGgAAGCCTTtGCGGTTTTAAAGAATATTGGGAGATTTGAAAGGATTTTACAGGATCTATAAGGTTTTCGGATATCTTAAGACATTCCAAAAACATTCAATAAATTTAAaCAAATTTAAGCAACTTTTtAAAcATTTTAAAGACAATATTAATAGGAATGATAAATAATTCTTTAAGTAATTATTTTTACTAACCTGCATGTGTTTCC

>contig00031

TTtAAAACTATCCTAAAaCATTCTAGTTCTAAaTTTTTATTATTTtATTGAAATGCCGTGGATTTTTTTagCACACGAAAACCATATATAGGATTATTAATTAATTGTAGGAAATATCTGTTGAAATCCAAGATTTGGGTCCAGCTGAGCTCCCATGACACGGCCAGTTTtGGTTCTGAacGTCGTAAaGtgT

>contig00032

CTTTACGtACgTTtcAGAaCCAAAaCtaGGCCGtGTCATGGGAGCTCAGCTGGACCCAAATCTTGGATTTCAaCAGATATTTCCTACAATTAATTAATAATCCTATATATGGTTTtCGTGTGTTTAAAAAAATCCACGGCATTTCAATAAAaTAATAAAAaTTTAgAActAGAATGTTTTAGg

>contig00033

CCAGCCGCTTCTAAgCCCGAAGTTGTACCACCAGCCGAATCTAAGCCCGAAATTATACCAGCATCCAATCCTAAACCTGCATCTATTATTaCGCCTAAAAAATAATTTAAACACTAAGATGTGTCTCCAATTGATCCTCAAGATGAAGATATAGCAGATTCTTCTGGGCCTCAAGATGTATCATCCTCATCTTAAGAAGGAGATGTACCACCATCCTCTAGTGTAGAAGATGTTACATCAACCTCTTCTGTACCATCATCTCCTtCTAAGGGTGAAGATATACCACCACTAATAGCCCACtGAGGATTTTAACATTTCAAAATTTTTTTttCgTcAGAgTTCCAATtGTCTGATAGCGCCAGGATAGGGGCCAGAaTTAATCTTCAAAAT

>contig00034

AACTCTCAATATTTAAATATATTCAGCTCAAAGAAGGAAAaTCATCCAAAATGAATTTGACAAAAGTTACAACCTGCTTTATTATCATAATCATTGCAACTTTAGTGACATTAGTGCCGAACACAAGTGCTGCTCTTAATTTTACCTGCATTACTAATTGCCTGAAAAACTACGCAGGGAATACCCTTGACAAACTGGGTTGCGCGGCTCTTTCTTGCACAGGTTAAGCGGACTAATAAGGACAACTCAGGTACCCTCCGAAAATCATTTATTAAACAATTAAACACCAATATGGAAATGTATTATTATGGCGGAGGTGACATGGCCTGCTTACAATTTGTCTTATTATTTCAATAAAaTTTAAAatGTTaC

>contig00035

CCTTCAAATGTAGGGCGTCACGCTAGTGGTGCCAGTGTTTtATTTTCTGCTATCCTTCATAAAaCTAACTCAGTGCATTTGTATGGCGAGTCAGCCCCCcAATGTTTCCAAGAATCTGTCAAAAGCTGGATCATTTTTGATTTCCTGGTCAAGTCCAAACTTCTTGGCTTCCTCTTCTTTCATAACTGTCAGCTGGGGCTTTGTTTTTtCCAACTGACTTATGTGTAGTTTAAGAAGCTTCCTAGGGTTCGCAGTTTTTtCACCATGATAAAATGGACGAAGAAAAGGACGACCTTCGAGAGCATCAACTCTTCCAACGACGTAATAATTAACTATTTTTACAGGCATAGTAATCTCTGCTCTTATGTAGCTCTCTGT

>contig00036

GCAGGTTTAAATGTTACACCGGgTGGCATtcGGACATTATTATAGTAGTATACGGATGAATGACTCAAGTCAATATAAAGAACGACGACCGCTAAACTGAAGCCGAGAGTAGAACTGACCCTCATTTCCTCTATGATAGAGAAAACTGATGTCCCAAGGAATTCGTGATCGTAGAAAGATTGATGACAATGTTCCGCTTTTTtGTTGTATGTAAATTATAGGTGGGCTGCAGTCGTCAA

>contig00037

GACAGAAGTTAGATTTGACGACTGCAGCCCACCTATAATTTACATACAACAAAAAAGCGGAACATTGTCATCAATCTTTCTACGATCACGAATTCCTTGGGACATCAGTTTTCTCTATCATAGAGGAAATGAGGGTCAGTTCTACTCTCGGCTTCAGTTTAGCGGTCGTCGTTCTTTATATTGACTTGAGTCATTCAACAGTATACTACTATAATAATGTCCAAATGCCACCCGGTGTAACATTTAAACCTGCAGGTTCAGAGAGCTACATAAAAGCAGCGAGTTCTATGCCTGTAAAAATAGTTGATTATCACGTCGTTGGAAGAGTTGATACTCTCGAAGGTCATCCTTTTCTTCTTCCATTTTATCATGGTGAAGAAACTCCGAACCCTAAGAAGCTTCTTAAACTACACATAAGTCAGTTGGGAAAAACAAATCCCCAGCTGCTAATTATGGAAGAAAAGGAAGCCAAGAAGTTTGGACTTTACCAGGAAATCAAAAATGATCCTGCTTTTGGCAAATTCATGGCAACATTAGGAGGGTGACTCGCCATACAAATGCGCTGAGTCAGTTTTGTGAAAGACGGCAGAAAaTAAAACACTGGCACCACTAGCGTGACGCCCTACATTTGAATG

>contig00038

tAaGGTACTACGGAGTCCCAGAACGTGTATTAGCCATCCTAAAGCTTCACAAGCTGTTTCGCACCTATAGAAAAaTCAATTTCTTTtGTTTGACGAGAAAATGAAGTTCTTTCTTGCTGCTGTGCTTTTCACCTCTGCGTTGGTTTTCGAATTAGGCTCAGCTTTCCCGTTCGAAACACCAGCTGCAACACCAGTTACAGCAGTTTCACCTAAGAAAGAAGAAAGCTCCCTACCCACTTCTGAAGGGTTAGGTGCACCACCAGCCGCTTCTAAGACCG

>contig00039

AAATTTATTACTCACATGTACATTTTTtAACATCAGAATGTGGAAAATAAGTCCCAAATAAGCATTTTTtACCCTCTGCTAAATTACAATGATAGTTGTGGATGACAATATAATAATAAAAAaTCATGGTCTATAAAAGTACAGTGAAAAATAAG

>contig00040

ACAcACGATCCAGAACAGAAGCTAGGACGCCTACTAATCCAATACACCAGAAAGAAAAGACACGGAAGTTAAACTTGTCGGCTGTGGTCCACCTATAGTTTGCATACAACGTA

>contig00041

TAGGACGCCTACTAATCCAATACACCAGAAAGAAAAGACACGGAAGTTAAACTTGTCGGCTGTGGTCCACCTATAGTTTGCATACAACGTATATCCCGGAAAACGGCTGGACACGTGCCAACATGACAAGGGCATTGTCATCTGCTTTCATATTCG

>contig00042

AAACCACAAATGTGTGAACGGCTAGAATTAGCTGTGCTAAATCCATATTTGACCAGTCGCAAGCATTCTTCTGGCATTCGAAAATAATGTAACAAAACCTATTTTTGTCAACTTCAAATGAAAACAATATTTTtACTTCCCGGAAAaTCAATTAATTTACTTGCAGTAAATTTCAATATCGTCAATAAAAATTATACATGTATAAAAATGTAATACAATTAATAAAACACTGAATTTTG

>contig00043

CTGCGGCAGATTGTTCACTCTGCATAGACAAAGAACCTACCAACACCTGCGTG

>contig00044

TAATTGTATACATAAACCACAAATGTGTGAACGGCTAGAATTAGCTGTGCTAAATCCATATTTGACCAGTCGCAAGCATTCTTCTGGCATTCGAAAATAaTGTAACAAAACCTATTTTTGTCAACTTCAAATGAAAACAaTATTTTTACTtCCcGGAAAATCAATTAATTTACTTGCAGTAAATTTCAATATCGTCAATAAAAaTTATACATGTATAAAAATGTAATACAATT

>contig00045

CTGCGGCAGATTGTTCACTCTGCATAGACAAAGAACCTACCAACACCTGCGTGAGGTATGATGGTCGCTATCAGAACCATAATATAGATTATG

>contig00046

AAAGCATCAAGTAAAAGAAAAGGATTCCCcAATATAAAaGGTGCGTTATTTTAatATTGTAAATTATATTGAATTTTACTATATTGGTGAGACTTCTGTACAaTAAATAATAAAACACACTGTATATAAAAGCGAAAATCAGGAAAATAATCATAATTATCGGATGGTATATAACATTGATTTTTTtCTCA

>contig00047

ATATTTTCCGTCAGTTCCAAGTAATTTCACAACGGGAGTCAATTCTGTTGCTTGTTTGTCTTTCACGCCA

>contig00048

TTTCACGCCTATACTGTTTATTGTGATATTTGCTGAATTAGCATCCGTCTTCGTCCAGTTTAGAGTAAGGCATGTCACTTGGTTATTAATTGCAAAATTATATTTCGCTACCATATACCAAAGTCCGCCAAAT

>contig00049

AAaGCATCAAGTAAAAGAAAAGGATTCCCCAATATAAAAGACAATTGAATTTGTGTATGCAAATTTTGAAATCTTCATTCCCCAGTATTCTCGACCGACCTGCGCTTTGATGTTTTTCTTCAATTCTGTTGAAATGATTTCAGTTTATACTCCCGGCTAACGAGTATTACACACGCTGAGCCAAATTGTTCGAAATAAAaGGT

>contig00050

TTTCACGCCTATACTGTTTATTGTGAtAtcTGCTGAATTAGCATCCgTCTTCgTCCAGTTTAGAGTAAGGCATGtCACtTGGTTATTAATTgcAAaTCATATTTCGCTACCATATACCAAaGTCCGCCAAATGCTGCGCTATCAAAATTCGGCACTGGTTTCACTTTTGGACAAGCTTGAGATTGCGCAGTAGTAACTGCAAAAGCTCCAGTGAAGAGTAGAACGAGGAATCCCACTTGAATCATTTTTCCACCTTTTATTTCGAACAATTTGGCTCAGCGTGTGTAATACTCGTTAaCCGGGAatGT

>contig00051

GGATAAAGCAAAATATTTACTGGTAAGTGAAGATATTACATTTAATTGCTCAAATGAGTCAATAGGAAAAGGACAAACTGTATTTtATTTCATTAGTAGAAAGCTGCAAGTACCGATTGTAAAATTACTTATGTTTGGAGGTCGTGCTCGCAGTTTGACAATTTTTCCAATCTTCTGTAACCATTGACACATCTATTAGGCCGTTATCTCTAATAAATTCCCAAGCCTGCCCCAGAACATCTGGTCCTGGCAGGCGATTTCTTGCAAAAATAACAACATTATCTTTTCCGTCAGTTAATTTATCACCGCAAGTTAATCGAATGgCCAACTTTTGATATTGTACAAATACAATAAA

>contig00052

GCTGCGCTATCAAAATTCGGCACTGGTTTCACTTTTGGACAAGCTTGAGATTGCGCAGTAGTAACTGCAAAAGCTCCAGTGAAGAGTAGAACGAGGAATCCCACTTGAATCATTTTtCCACCAACAGAATTGAAGAAAAaCATCAAAGCGCAGGTCGGTCGAGAATACTGGGGAATGAAGATTTCAAAATTTGCATACACAAATTCAATTGTCTTTTATATTGGGGAATCCTTTTCTTTTACTTGATGCTTTaGaC

>contig00053

TTAtaagaaGTAGTAAGAGTTAAAaTAATAAaCGATCATGGTcTTGAAAAGTACAGTGAGAAATAATAGTTGTAAGTTTATTTTTtGGATGTCTTGTTGTAGCTCgACTACTTGAATTGATGCAAATGGTGAAGGGTATTAATAAAGAAGGTGTCAATGGTTCTCGATTTATCGTCCAGGATTAAATTCCGGACTATTTCCATTGGGGACTTTATGATCCACCGCCTCTTTTCGTATCAGAACCAGATGTGATAGAATGACGACTGGAACAAGAATCGGCCATCTGAAAAAGTGCAATGATGCACACTAGGAGGAAGACAGTCACAGT

>contig00054

AGACGTTTTTCGAGCGCAGCAGAGAACAGGTAACGAAAATGGCATCTACAAAATTTTTGATGATCATACTTGTGGTATTGGCACTTATGGATGTTGCCATGGCGCTCTGTCCAGCTCCACCTGGTAGTGCAGATAACTGTTGCAGCCATTGTTGCAGCCCAGAACCGATTTGTGGAACATCATCACCGTCTGATGTGTCAAAAAATTGCATTGCATAAGTTCAGTGATTATAAAaTCATTGACGACAAAAAaTCATGACGTAGAACTTTGAGATGTTACGCACTGTG

>contig00055

ATGGGGACGACCTCAGATCAGGTGAGACTACCCGCTGAATTTAAGCATATTATTAAGCGGAGGAAAAGAAACTAACCAGGATTTCCTTAGTAGCGGCGAGCGAACAGGAAAAAGCCCAGCACTGAATCCCGCGGTTTTTGCCGCTGGGAAATGTAGTGTTCGGGAGGATTCACACATCCCGTGACGTTGCACCGAGTCCAAGTCCATCTTGAATGGGGCCACTTACCCGTAGAGGGTGCCAGGCCCGTAGAGACCGGTACGCGTTTCGGGAGAATCTCTCCTTAGAGTCGGGTTGCTTGAGAGTGCAGCTCTAAGTGGGTGGTAAACTCCATCTAAGGCTAAATACAACCACGAGACCGATAGCGAACAAGTACCGTGAGGGAAAGTTGAAAaGAACTTTGAAGAGAGAGTTCAAGAGTACGTGAAACCGTTCAGGGGTAAACCTGAGAAaCCC

>contig00056

GATTCTGAGATGTCTTTCATATGGCTTTATATAGTCCAGTGTCACCAGAAAGTGGGATTC

>contig00057

GTACTTTTATAGACCATGATTTTTtATTATTATATTGTCATCCACAACTATCATTGTAATTTAGCAGAGGGTAAAAAATGCTTATTTGGGACTTATTTTCCACATTCTGATGTTAAAAAATG

>contig00058

TTTCGGTAAATTCCACAGTTAGCCTCCAAATCCTTTtCTTGTATATTGTAATAAGCCTTTAATAAAAaTgTtATGAAATCTTctcTTGGGCATATTTCAACATCTCCAGATTCGCAGAAACCGATAGAAATTGCCATTAAAGCAAGGACTAaGAACTtCATgATGGT

>contig00059

TCGAGATTTCTATTGAAGGCAAATGGACATTAAATATTGACAATAATCTCACAATTATTTAAATTTTCCATAGTTTTGCAAGTTTTTAAGTTTACTCTTTCTGAACTCAAACGATATAATAAGGCGGGCATCATCAGCGTGACATACACTACGTAAAGAAGCAGGACTATTTCTTTtTGTATTAAATATCTTACTTTCTGCATTTCTGATATATTATTATACATATAGG

>contig00060

AAACTCGAGATTTCTATTGAAGGCAAATGGACATTAAATATTGACAATAATCTCA

>contig00061

ACTTCTTTTtGTAACTACTATTtATTACTATTTTttGGTTTTttGGtCGtATtATTACTTTAACTAGTTTtGGTCTAAGTATtACtAAACtACtAGtCGTTTtAC

>contig00062

CAATTATTTAAATTTTCCATAGTTTTGCAAGTTTTTAAGTTTACTCTTTCTGAACTCAAACGATATAATAAGGCGGGCATCATCAGCGTGAcATACACTACGTAAAGAAGCAGGACTATTTCtttatGTATTAAATATCTTACTTTCTGCATTTCTGATATATTATTATACATATAGGACCATcATGAAGTTCTTAGTCCTTGCTTTAATGGCAATTTCTATCGGTTTCTGCGAATCTGGAGATGTTGAAATATGCCCAAgagAAGATTTCATAAcATTTTTATTAAAGGcTTATTACAATATACAAGAAAAGGATTTGGAGGCTAACTGTGGAATTTACCGAAATG

>contig00063

CTGTATATTCTTGAACACTTAGAAGAGCTTTTCCTAGTTTAGACCACCTAT

>contig00064

TTTTGGTTTTTGGCGATATACTTAACAGTtTGGCAAGATACAAACACAGCGTTTACGCAGAGTTTtGAGAAATTATCTTCCTCCATGCATGGTAAcGCTGTTTTAAAAACATTTAGCTTCACCTTATTGAAATTAGcGTTATTCAAACATTGAACTTTAACGA

>contig00065

TTTtGGTTTTTGGCGATATACTTAACAGTTTGGCAAGATACAAACACAGCGTTTACGCAGAGTTTTGAGAAATTATCTTCCTCCATGCATGGTAAtGCTGTTTTAAAAACATTTAGCTTCACCTTATTGAAATTAGTGTTATTCAAACATTGAACTTTAACGAGATTGTCGAGGTCCTCATCAGTAACTTTTTtCTTCTTCTTTGCTTCATTCGCTATATTATTTATGACAACAG

>contig00066

AGCgTTACCATGCATGGAGGAAGATAATTTCTCAAAaCTCTGCGTAAACGCTGTGTTTGTATCTTGCCAAaCTGTTAAGTATATCGCCAAAAACCAAAaTGTATAATGTGTTCAAAAGAGTACGTGAAGAGAACCAAAGGATAaAaGACGTGACGACTTTTGATATTTCTaCAATCCTATTATGTATACCTTATTATTATtatAAGCAAGAcA

>contig00067

AATTTCGGTCAATTCCACAGTTAACCTCCAAATCCTTTTCTTGTATATTGTAATAAGCCTTCAATAAAAATTTTATGAAATCTTCTCTTGGGCATATTTCAACATCTCCAGATTCGCAAAAACCGATAGAAATTGCCATTAAAGCAAGGACTAAGAACTTCATCATGATCTTATATGTATAATAATATATCAGAAATGCAGACAGTATGATTTTTAATACAAAAAGAAATAGTCCTGCTTCTTTACGTAGTGTATGTCACGCTGATGATGCCCGCCTTATTATATAGTTTGAGTTCAGAAAGaGTA

>contig00068

AAAAGCAGCAAGTGAAAGTTGAGTATTCCTTAATCTAAAAGGTGGAAAAATGTTTAAAGTGGGATTCCTCGTTCTACTCTGCACTGGAGCTTTCGCATATACTCCTAAGCCATCTGATACTTGTAAAACTGTGAAACAAGTGAAGAAA

>contig00069

CAACAACCAAGAAACAACATATAGTGCCTTTGCGACATTCCTTGGAACTGATGGAAAATTCAATATAACATTTGATAAAGGACAAACTCAAATGGAGGAGTCGTGGATATATGTAGACTCTAAGTATAAGGAATGGGCCGTTAAATTCATTTGCGCTGATAAAACATCTGCCGGACAAAATAATGTTATTATTTTGACAAGAGACAGAATACCAAAACCAGGTATTTTCAGGAAGGCTGACAAATTTCTTCATGATCACCAACTAAACGGCAAGATAATTACAGAAGATTGGAAGAGTTGTAAATAAAAGTTTTTTTTttACAATCGGCACTTGCAGCTTTCTACTAATGAAATAGAACGAAGTTTTTCAATTTGCTATTGACTCATTTGAGTAATTACATGTAATATCTTCACTTGCCAGTAAATGTTTtG

>contig00070

ATTTGGTGGAACTTGGTTCGTATTCAAGAAATATGATTTGCCTGCTAATAACGCTCGAACATGCATTACAGTAAACTGGCAGCCGCAGAATACTAGTGCAGCAGATGTTAAACTTAGCATGAAAGTCAACAACCAAGAAACAACATATAGTGCCTTTGCGACATTCCTTGGAACTGATGGAAAATTCAATATAACATTTGATAAAGGACAAACTCAAATGGAGGAGTCGTGGATATATGTAGACTCTAAGTATAAGGAATG

>contig00071

ACATCGCAGTCTTTGTTCACAAAAATCCAGGGTCCTTCAGCAGCACCAATTGCAAATAAATTCGGGTCTAGTTTCAACTGTTTCACGATATCTTTAATGTCGAATAATATTTGCTTTGTTATGTCAGTATTATACAGAGATGTTCCCCCAATGTCTAGAA

>contig00072

CGCCAAATCTCGATTAAAaTGAGAGTTAAAATTATtGTGCTCTTCGTTTCTTTGTATTTGGCTAGTTCAATTGGAAGAAAGGTAGCAATTCCTCAAGACTGTATTTATCGTATGAAGCCGAATGGTTGTCATtGTGATTCCAGCTCCCAGTGTATTTACGAGACTTGCGATCGTACTGA

>contig00073

AAATGCATTGTTCCTTTTAagCCGCCACATTAAGAGAAGAAATTTAAGTCTTTCCTACAAGCTTTGGTTGTATAATGTGCTGAGTGTTtCTTGTGTAACCAAAATGTAGCTTCTTAaTTGGAATGTTGTACAAAAATAAAAaCTTAAAaCAATTTTGAGCCcGTTTTTGGTTCTAACCTTCGGCTCTTATCCAGGCtCTTCGAGCGTAAACACCAGGGCCCTGACGCAATTTACACAATGTGACGCAGTTATCC

>contig00074

AGAAACCTGTACCTAAAGGTAGCAACTTGACAAGGATTCCGAGTTTTTATCTAGATGAAAGTTCTGATAAGGTGACTGGAGTCGGTACTTTT

>contig00075

GTTTCTGCCAAAACAAAACTTGATCTTACTCAAAATCACTTTCATGTGGCATCTAAAACAAGAATCGATTGTGGACATTATGTCCGTGACACCACCCCTGCAATTGTAGAATATGATGGATATTTTACACCTGCAGAAGTTCTTTATAGAGTAAATCAAGCACCTATAAATAAGCTGCTTGTGGGAAGCAGTGATCATTTCTGATTTAATTCAGTGACACGTTTCTCACACTAATTTAAGTTTAGAAAGTTTGTTTCTAGATTTCAAATCATAAATGTAAATTTACTTTTCACATTTAAGGTGAAATATGTTACAACATTAAAGCAATGTTTGTGAAATGTCAGGGTAATTCAATAAAATCTTTGACAAAAtATcAaT

>contig00076

CTAGAATGTTCGCAAAAAGTACATTTGTCTTTCCTGTTTGCAACGCAAAGACTCCTCCAAGACCAACAAGAATTGGTTCTTTGTAGAATTGTGTAATACTCCTTCTCATACTAGCAATGAAACTATCTTCGCCTGTTCGCTTCTTAGCATAAACTCTTAGGACCTTTCCTCTCTTCCCTTGGCTTAGAAATAAATTCCCATGTACAGCAATTCGAGTTTCATTATAAGGCAGAGCCTGACATACAATTTTTCCACGAATTGTGTCCATTGTGATAATATAAGAGCTTGTAGTTGATTTCTTTTTACCCAAAACTAAGTTTACTTGC

>contig00077

GAAATACAGTCGGAGCCGTAACCACACTGACAACCATTCGGATTCTTAAGATGAATACAGGTATTAGGGA

>contig00078

CGAAGAACACGATAATGTTAAGCCTCAGTTTAAGAGAGATTTGGC

>contig00079

GTACATGGGgACAAATTTTCATTGAAGTTGGTTCAATCGTCTTTTGATTATTTATTCAACACCATGGAAAAAGGGAATGAAAAGAAAGTTGTATTTATCTGGTTCATATTTTCTCTTCTTTTCTGCAAATTATTAAATACAAACGCTATAGTATTGAAAATTGAAGAAAGAAAATTGTACGTGCCTCCCTTGGCTGAATTACAAAAAGTTTTCCTTGCCGAGCTTAAAAAAAaCTTTGCTGAAGTTTGGGTCGAAATCGTGGATTCTCCTGACTTGACTAAAGAACCTTTCAATCTAGCAGCTCCTGGAATAGGAGGAAATGAAGTAC

>contig00080

GTACATGGGgATTCAACGTTCGACATGGCATCCGTAAGAACTGTTACCGTCTTCCTCCTGGTGTGCATTATTGCATTTTCTCAGATGGCTGTTTCTCACGCCGATCCAGAGCCACCGCATCATCAGCAAGAACATCATGATTAGAATGAATGGCACCTTCTGCTTGAGATCTTTGACAACTTGAATCAATTTAAAATGTTGATCTGCAACAGGATAACCAAAAATAAATTTAAATTTCTTATTTTTCACTG

>contig00081

GAGAAGAGTGTCCCTGATTGACCGCTATCGTCGAGCTAGTCTGAAAACTGCTACTTATGCATCTGTGTTTATACAACAGCAGGACGAACAACGAATGAGCATCTGCGGCGTGGTGCGAAATGCAATTATGATTTGGACAAGAAAAaGTGGAAATGCATTGTTCCTTTtAAGCCGCCACATTAAGAGAAGAAATTTAAGTCTTTCCTACAAGCTTTGGTTGTATAATGTGCTGAGTGTTTCTTGTGTAACCAAAATGTAGCTTCTTGATTGGAATGTTATAC

>contig00082

GcATCATCAGCAAGAACATCATGATTAGAATGAATGGCACCTTCTGCTTGAGATCTTTGACAACTTGAATCAATTTAAAaTGTTGATCTGCAACAGGATAACCAAAAAaTAAATTTAAATTTCTTATTTTTCACTGTACATGGGgCTtGTAAGAATGAAGCTCGTCAGCAGAACACTGCTCCCTGTGATCATAGTTTTAATTCATATACTTGCATTAAATGCTACGTCCCAGACGCCATGGTATGACCATCTTCGTAATGTAGATCCTCAACATCAAGCAGAAACATCAAGGACTGACGAAACACAAAGACCCTCAAAAAAaCCACGACTATCAGAACCAAATCAAAAGCaTTCACTACTAAATTCgCTACGACATCCAAACTTCGAACTACGATCACAATAACAAGCGATACCAAATACACAACATTAACTACAGCTACAA

>contig00083

ACGGCCAGTTTtGGTTCTGAacGTCGTAAaGTGTACGCCACAACAATCATATGGTTGAGGTTTAACATCGTTTCTCTTTTCGTGTTTATTTTCAGGCAAAAGATTTAGATGTATCACCATATTTGTAGAAGGATTTACCTTCACCTGATAGGTGTTGCTATAaCAAGTGATATATGACACATCTGAAGTAGGATTCAGTCTGGCTGGATCATCTGCTTTAAAATCTCCGCTACGTTTTGGTGGAATATTCACTCCTAtAAGTTCTCCCTTTtCATTACGATATGCGATACATCTTGCGTGACCGTTAAGACATTCTTGGATAGCAAAGAGCTTGTGTTTTTtCAGATTTtCAAGAGGCGGAATAGAAGCACTGAAAGTAGAATCAGCAATAGCACAAGCGACCATTCTAGCACTGCCGGTAACAATTTCTGTAGTGGCCCTAATATCCTTATCAAAAAaTTTtAAAAaaTGCTTGTCCCTTTTATAGTTCGAGCGTATGGt

>contig00084

ACCATACGCTCGAACTATAAAAGGGACAAGCATTTTTAAAaTTTTTtGATAAGGATATTAGGGCCACTACAGAAATTGTTACCGGcAGTGCTAGAATGGTCGCTTGTGTTATTGCTGATTCTACTtCcAGTGCTTCTATTCCGCCTCTTGAAAATCTGAAAAAaCACAAGcTCTTTGCTATCCAAGAATGTCTTAACGGTCACGCAAGATGTATCGCATATCGTAATGAAAaGGGAGAACTTaTAGGAGTGAATATTCCACCAAAaCGTAGCGGAGATTTtAAAGCAGATGATCCAGCCAGACTGAATCCTACTTCAGATGTGTCATATATCACTTGTtATAGCAACAacTATCAGGTGAAGGTAAATCcTtCTACAAATATGGTGATACATCTAAATCTTTtGCCtgAAAATAAACACGAAAAgAGAAACGAtGTTAAACCTCAACCATATGATTGTTGTGGCGT

>contig00085

ATAATTTTAAAGATTGAAAAGAATCAACATAGATTTTAAGAGATTTAAAAGAATTTAGGGTATGTATTTTTAAATACTTTAAGGAATTTCAAATATTACAAGAGATATGAAAAGATTCTAAAGTTTTTGGGGTATTTTACAAGATTCTAAGAAATTTGTAAGAGCTTTATAAATTTATAAGCGATTTCAAAAGATTTCATAGGATTTTTGAAACTTTAGGGAGCTTCCGAAAATTTTAATGATTTGAAGGCATTAAAAAGGAaCGATGGGGTTTCAAAGAATATTGGAAGACTGGGAAGTATTTTATAAGAGCTACATGGTTCTGGGATATTTAAAAAGATTCAGAAGGATTTAAATAGATTTTAAGGTATTTTAAAAATTGTATGGTTATTTAAATGATTCCAAAGAATTTCAATAGATTTTCAGAGATTTCCAAAGATCCTAAAGATTTTAGGGTATTTTAAAATATTGCATAAGATTGTAAAAATTGCATTGAATTTCCTAAGATTTTAGTAATTTAAGTGGATTTAGAGAAAGCCTTTGAGGTTTTAATGAATATTGGAAGATTTGAAAGGATTTTACAGGATCTATAAGGTTTTCGGAAATCTTAAGACATTCCAAAAACATTAAATATATTTAAGCAAATTTAAGCAACTTTCTAAAAAaTTTTAAAAGCAGTGTTAATAGGAATGATAAATAATTCTTTAAGTAATTATTTTtACTAACCTGCATGTGTTTCCACCTTATGTTAATTGATAACTGGAGCAGTAATGTAAATGTTTAGAAAAACCTGCCATTTt

>contig00086

TAGTTTTAAAGATTTCAAAAGATTTCATAGGATTTTtGAAACTTTAGGGAGCTTCCGAAAATTTTAATGATTTGAAGGCATTAAAAAGGATCGATGGGGTTTCAAAGaTActGG

>contig00087

CGGACACAACACGCAACGTAAATTATAAACAAATCTGCCTTCCTGAAATCAAGGTCAtCCAAGCCAGCTATCAACACGAATGCGTAGcAATTTGCTGAAAGCCGGCGATCGa

>contig00088

AGCCACTGAGGAAATCGTtcGgTCGTTGTCAGTCCTGCTCTCCAA

>contig00089

CTTGGATGACCTTGATTTCTGGAAGGCAGATTTGTTTATAATTTACGTTGCGTGTTGTGTCCGCAATCGAAGAAATTCTGTATTCAGAAAATAAATTCCTCGATGGATCGACTCTACAAGTTTTAAACTCTACAGGCATCAAATTTGCTTCTGCACGCTTAGTATTCTGGCGGCTTCAAATTTAAACGATTTTACCAAAaTTCATTGGGAATTGAACTGAGATACTTTTAAAAaGAATGGATGATGAAAATGGAAAAATGGAAAAAGAAAaTGAAGCAGAGAAGACTTGGGATTTTGAAAAAaTACCCGATTTGTTtCTTATCGAAATTTTTCGGCATCTGACAACGATAGCTGACAAAATTCGTATAGAAAGAGTTTGCAAAAAGTGGAAAAATTTGAGCCAAATTTCGTGGGATACTTTTAAATCTCTCGATTTCTCAGCTAAAACTTGGGGAATGGAAGAATATCGTCGGGATCGACGATCAATAAAAAGAGTCGATTTGGATCAAATTATAATACGCTCGGGGCAaTACCTTACAAGTATTAGTATAGATATTCAAAaCTTTTCCCAAATTTGCCACTTGCTTCCcAACATTCGAAAACTGCAATTGATCGATTCTTGTCTTGATGAAAATCGAGAATGTGGACGGAACCTCGAAAACAaCGACTGGGACATGACTCGCGA

>contig00090

TTGTCTTTGAAAAAaTGTGTATTTACCCCTCGCTTCGTAAAAAaTAGACCAGTAGCGTCTTAAAAACGTTACAGCAGTAACGACTTTCTAATAAATTCCGTAATTTCGACTGCATTAGGTTAAATTATATCGATCGCCGGCTTTCAACAAATTGCTACGCATTCGTGTTGATAGCTGG

>contig00091

TAATACTTGTAAGGTATCGTCCCGAGAGTCTTATAATTTtaTCCAAATCGACCCTtCTtATtGGtCGTTtAtAAACACGACCtgCTTtcATTCCCcAAGTTTtAtCTGAGAAATCGAGAGATtcAAAaGTATTCCACGAACTTtGGCTcAAATTTTTCCAtCTTTTGCAAACTCTTTCTATACGAATTTTGTCAGCTATCGTTgCcAGATGCCCAAAAATTTCGATAAGACAAacATCGGGTAATTTTTCAagATCCGAAGTCTTCTCTGCTTCATTTCCTGTTTCCATTTTTACATTTTCATCATCCATCATTTTcAAAaGTCTCTCAGTTCAATTCCCAAtGAATTTTgTAAAaTCGTTCAAATTTGAAGCCGCCAGAATACTAACCGCGCAGAAGCAAATTTGATGCCTGTAGAGTTTAAAACTTTTAGAGTCGATCCACCGAGGAATTTATTTTCTGAATACAGAATTTCTCcGATAGCGGACACAACACGCAACGTAAATTATAAACAAATCTGCCTTCCTGAAATCAAGGTCAAG

>contig00092

AAATGTGTATTTACCCCTCATTTCGTAAAAAATAGACCAGTATCGTgTTAAAAACGTTACAgCAGTAACGACTTTCTAATAAATTCGGTAATTTCGAGTGCATTAGGCTAAATTTTCGATCGCCGGCTTTCAGAAAaTTGCTACGCATTCGTGTTGATAGCTGGCTTGGTTGACCTTGACTTCAGGAGGGCAGATTTGTTTATAATTTACGTTtgCTTGTTGTGTCCGCTATCGGAG

>contig00093

TCCCAAGTCTTCGCTGCTTCATGTCCTTTTCCGTTTTACATTTTCATCATCCATCATTTTTAAAaGTATCTCAGTTCAATTCCCAATGAATTTTGGTAAAATCGTTTAAATTTGAAGCCGCCAGAATACTAACCGTGCAGAAGCAAATTtGATGCCTGTAGAGTTTAAAACTTTTAGAGTCGATCCATCGAGGAATTTATTTTCTGAATACAGAATTTCTCCGTTTGCAGGaCACAACACGTAG

>contig00094

TTATGAAAAATTACCCGATTTGTGTCTTATCGAAATTTTTCAGCATCTGCCAACGATAGCTGACAAAATTCGTATAGAAAGAGTTTGCAAAAAGTGGAAAAATTTGAGCCCAAGGTCGTGGACTAATTTTGAATATCTCGATTTCTCAGCCAAAaCTTGGGGAATGGAAGAATATCGTCGGGATCAACGATTAATTAGCAGCGTCGATTTGCATAAAaTTATAGGACTCTCAGGACGATACCTAACAAGTATTAGTTCAAATAtGacAATATTTGCAAATATTTGCAAATTTCTGCCcAAAATTCAAAAaCTGCAGCtAaTCGATCATTGTATTGATAAAACTAAAAcATGTAGATGGGATgAATATATCGG

>contig00095

ACATTTTTGTGTATTTTTAAAaGTGTTCTAATATAATTAATCTTTTAGGATTTTAGTTATAGTTCAATCGGAGAATAAGTGTTTGAATACGTCCATGATTTTTGAAAAAATATTAGAACCCAATGCACCTCGAACTTTGTCGCATATCAAGTGAATGCCAGTTGGAATAACATTTTTCATCAGACCGCAAAATTGGTCTAATGAACATAATTTCTCACATCTAGGAATCTGTAGAGTTTTCATCTCCGCTGGAACGCCCAAGTAATAATTAACCTTC

>contig00096

GCATACTAAAAaTATTTCTTAaTCTCTTCTGGCGTTATTGACAAGTTTCTAACATTAGCTTTATATTTTAACATTCTAAAATATATACAGAGAAACGTGTATATACTGTGAAAAATTTCCTTTGATCATTGTGCCAAAGAAACGCGAGTGCTATCAGAAGGAAATTAAAAAATAATAAAGATTTTGGATCGAATCTATTAGCGCGAAGAAGATGGTCTCACATTTGTGGGTCCAAGGCCTCATTTTTATAAGTGTCGGATACAATCCTGTTTTCACAGATAAATCAACTCTTAAATTGCTCATTGTGATTCCGCGTCATGGAGACCGAGTTCCAGCACCTCCATTTGAATTTTACCCAACTGATCCATACGCAAAGCAAAAGTTTGATCCTCCAGGATATGGAAAACTAACAAAAAATGGCGAGTTAAGGGAATTAAATCTGGGTAAATTTCTTCGAAATACATACAATGATTTTTTCGGATCTAAATACGTCGAGGGAGCTGTTGAAGCTAGAAGCACTGACGTCAATAGAACAAAAGCATCTGTAAAATTGATTTTGGAGGGTTTGTATCCAGGAGCAGTAATACCTTCAACATTTGACAATAAACTAAAGGATGTCCTGCTTTTCCCACAATTATGTCCTGAATACGTCAAAGATTACTTGGAAGCAAGATTGGCAACAAAGGGAGAGTTGAAAAAATTAAAAGGATTTATGACCAAATTGAGCAAATGGACAGGAAAAACTATTGAGTCGTCGCTTGATATGT

>contig00097

TACTtACGTTTGGTAAGTCTTGTAAAAGTTTTATTGTAATtC

>contig00098

TTGGGGCACATGCGGAAAGTAAACACCCAATGCCTTGAGAACAGCAACAACGTTAAGATCATGAGCACCGTATAACATGAGTTTTCGGTTTTTATCCATTTTTCCACTTTTTACAGACTCCATATCTTCTTTGATCTTTTTTATCAACCTGCCCCCGTTGTTTCTACACATCTTATCGTCAAAATTCATTACTTTAAATTCTAGAACAGTTCCTGTAAAAAG

>contig00099

AATAGTAGGAATAaGAATAACCAATaCAAGTACCAAAGAATAGGACAAGGGtAAA

>contig00100

TCGCCATGTGGATAGATACCATCAGTCCAAGATGGCAGGGTAA

>contig00101

TTGTATTTTATTTAATTCAACGTGATGTCTTGACACAATTATATAAGGAGACGCGCACTGCCGCCGATAGACGAAGCTTGGAATCTGCCAATCAGCGCAGAAAtGTCTaGTAGACAGCAGCAACAGCCACcGCCTTCCGGGGgTCCGGGTTATAAATATTCTGGAGCACCTGCAGGACCTCCACCTCCTTATCCTCAGGGACAAGGACAGGTTgCCACTGCAAAGAGATTTAaGGTAGAAGAGCCTGCATCGCCTTGCAGCCAACAAAGGCCGCCCGCATTTTATTtGTCCCcGCAaCAATTGCAAATGTTACAATGTTtGCAACAAAATCAGGCCAATCTATCAGCTCACCAACAAAACGTCTTAGTGCAGTTGCAACATCAGT

>contig00102

ACTCTTTTGAACaTCTTATACcATtcgTGTTTTGgacgATATGCTTAACAGTCTGGCAAGTTACAAGCACAACGTTTGCGAAGAGTTTCGACCAATTATCATCCTTCATGCATGGTACCGATGTTTTAAAGACATTTCGAAACGCTTTTTTGAAATCATCGTGATTTAAACAATTAGCTTTAACAAGATTTTCGAGGTCCTCGTCAGTAATTTTTTGGTTTTCTTTGCATTATTCGCTATATTAGTTGTTATATCAACTGTAAGTCCTTGAACACTCATAAGAGCTTTTTCTACTTGAGACCACTTAGATGCTTGAACAATTTCACATTcTGCGTCCAAATCCTTTGCTTGTATATTGTAAAAAGTCGTTAATTCTTGTTTTATTAAATCTTCTCCATTGCATACGTCAACCTCTCCAGATTCGGAAAAACCGATAGAAACTGCCATTAAAGCAAGGATTAAGAACTTCATCTGG

>contig00103

GCTGCTGGTTATAACTTTTtATTTAATAATGCATATCTTTCTTATAATAGTAATAGGTTATTAcAAGATTGTAGAAATATCAAAAGTCGTTAAGTCTTTTATCCTTCGGTTCTCTTAACATACTCTTTTGAACGTCTTATACATTTTGCATTTTGACCATATGCTTAACAGTCTGGCAAGTTACAAGCACAACGTTTGCGAAGAGTTTCGACCAATTATCATCCTTCATGCATGGTACCGATGTTTTAAAGACATTTCGAAACGCTTTTTTGAAATCATCGTGATTTAAACAATTAGCTTTAACAAGATTTTCGAGGTCCTCGTCAGTAATTTTTTtGGTTTTCTTTGCATTATTCGCTATATTAGTTGTTATATCAACTGTAAGTCCTTGAACACTCATAAGAGCTTTTTCTACTTGAGACCACTTAGATGCTTGAACAATTTCaCATTCTGCGTCCAAATCCTTTGCTTGTATATTGTAAAAAGTCGTTAATTCTTGTTTTATTAAATCTTCTCCATTGCATACGTCAACC

>contig00104

CGATAGAAACTGCCATTAAAGCAAGGATTAAGAACTTCATCATGGTCGTATATGTATAATAAtATATCAGAAATGCAGAAAGTATGATTTTTAATACAAAAAGAAATAGTCCTGCTTCTTTACGTAGCGTATGTCACGATGATGATGCCCTACTTATTATAT

>contig00105

CGTTTTAGTTTAGAAAGAGTAAACTTCAAGACATGCAAAACTATGGAAAATTTAGAAAaTTGTGAGCTTATTTTCAATATtGAATGTCCATTTGCCTGCAATAGAAATCACGAGTAT

>contig00106

AAGCGCCACATCGAGTTGAGAATTTGTGATGGTATATAAGAAGCACTAAGAAAGTTTGCGATAATTACAACTGGGACAATCATGGGGTACACTTTGCTAACGCAG

>contig00107

AAGATATAAAGAATCTTTACACAAAAAAAAGGAAGCCTTGGTAGAGCGTTGGCCTATTATAGTAAACGAAGGATTCATCCGGGGTTGGAGGGACGATAcGAATATCGCTCATAATGTATATGATTCTAATGGCAATAGATGTCACAAACTTAGATCAACAGGTGAACTTCGGTGCgAAGATATTCCAGATGTGGAAAAAaTAGTTTTGCCAGCCTCAATGAAACCGGCAATAGGGGGgAAACTCGTGCCAAAGCTACCCAAAAAAGAGGATGTTGTTTTTAAGGCTGAAGATAATGCAGTGCGCGAAAGCATACAGAGAAATTCAAATTACATTTTAAATTATATTTCTTCTGTTTCTTAATGCAATAGTAATGACTAATGCTTAATTATCTGCATCTTATATTTTAATCCGATTTTACATGAAAAATTGTATTTTGTGATGAGGAAAaTATATTATTCAaTTAaCTAAa

>contig00108

TTCTtCCATtCCCCAAGTTTTAGCTGAGAAATCGAGAGATTTAAAAGTATTCCACGAACaTTGGCTgAAATTTTTCCACcTTTtGCAAa

>contig00109

CTCTTTCtATAcGAaTTTtGTCAGCTATCGTTGGCAGATGCCGAAAAATTTCGATAAGACACAAATCGGGTA

>contig00110

gTTTCCATTTTTACATTTtCATCATCCATCATTTTT

>contig00111

CTAATACTTGTTAGGTATCGTCCAGAGAGTCTTATAATATAATCAAAATCGATGCTTCTTATTGATCGTTGATAAACAC

>contig00112

GAAGGAATAATGAAATCAGATAGATGTAGATGCTGCTTAAGAGGCTGCTCAGGCAGATATTTGTCATCCTACTGTCGGTAAGGGCTCAGCAACTTTCTACTGATGATTCAACTGGCGTGACTATGGAAATCTCTGAACATTATATTAATAACCGATGAAAACAATATTTCGTTTGAC

>contig00113

TATGATATCAAGCTCAAACAACTCTGCCCCACGGGCACAATGCCCGAAGCTT

>contig00114

ATACACGTCGGACTACCAGTCAGCGAGGCTAGGATATAAAAGTCGAGGCAAATCGGCTGAAGTTtCTAATGTGAGTTTTAATCCTATACTCAATTTTTGAAGGAGAATATAAATTCAAAATGAATTCAACTATTTCCGTGAAATTTCTCCCATTGGGGATGTGTATCTTCATGGCATTtGTAATCTTTCAACAAGTAGAATTTTTCAACTGCGGAAAAGTTAGCGAAATTGATAGACTAGTGCCGTTATGCGGCAAGTGG

>contig00115

CGGACTACGAGTGAGCGAGGCTAGGATTCAAAAGTCGAGGATAATCGGCTGAAGTTTCTAGTGTGAATTTTAATCCTATACTCCATTTTTGAAAAAGAACATAAATTCAGAATGAAGTCAACTATTTCTGTAAGATTCCGCCCATTTGGAATGTGTATCCTCATGGCATTTGTAATCTTTCGACAAATAGAATTTTTCTACTGCGGAGAAGTCGACGATTTTAATAAAATTGTGAATTTATGCGGCTTAGAGTATGATAACAAGCTCAAAGAAGTGTGCCCCAAGGGCACAATGCCCGAAGCTGTATCTATACTTAATAATGAAGACAGAAATAAATGCTGCG

>contig00116

CATaTaGtCCATCATGGCTTTtAGTTCTTTAAACTTAACATCTTTGAGTATGAAAaCGGGgTGCTTATCATAATGTTCGCTGAGAAGGCCCTCAAAGTAAGGACTGCACGCGGAGAGGACCACTTTGTGAGCCTTTAGGt

>contig00117

AcAGGGGAGAAGTGAATATCTCCCAAGACCAATTGGCAGCCCTCCTTAAGGCCGCAGAGTCTTTACAAATAAAAGGCCTCTCGGAAAGTAGAACTGGTGGTAATGCCTGTCCGAAACCAGACACCAGGCAAAGTAAAGTCAGCTCACAACCGACGGCCCCGTCGTTAGACATTCCACATGCGTCTTCCGGACTTACAATAGAGAAGAACAACAAAGTTCCCAGGCAGAGTTTGGCTCAGAGCTCTGTTGGTGACCTACCTGAAGATTCTGCCAGCCCCTCCATCCCCAAAGGTCTCTCCTCCAGGGAAGGTTCCCAAAGTCCTGTCTCCAGAAAGAGAAAAAGACTTAGAAGAAGAAGCGTCGGGGAAGACAACTCTGTAGAAAACCATGAATCCAACTCTAGTGATATGACCCAAGCCAATGTCCCAGCCATGGGAATCGCCCCTGTTGCCGATGAGAAGTCTCATGCCGACCCTGCCGACTCCCTCGGCAGATCGGCGCTGATGCAACAGTTGACGAAACCTGCCGATGAGATGCTTCAGCTTCCTGTCGAAAAACCTGAACCTACCGAAGACATGATACAACCAAAGTCTGAGTATTTGGACGACCCGGAGGAGAGTGTGGAAGACTTAACGTTAGACGACGATATGAATGACCTCAATGAAATGGAAGATAGTAATAGGGCTGGTCCAaGT

>contig00118

AGGAAGAGGACGAACAGGAAGAAGAGGAGGAAGAAGAAGAGAACTGAGCAAGACATCAAGAATTTTTCTTTAAGACTTATGAACTTTTTAAGTCTTTTAAGACTTCTAGGTTTTTATATATCGTAGATAACTCTTTTTGTCAATAAACTCCCAGCCCATTTTTTAAAAGACTTCATATGGTTGACATAATCACGTGGGCGAAACATTGTTCACGGGAAGCAAATCCATTATGAGACATTTTTCCTCGTAATAACAAGACAAATTGTATTTAAAAAAACTTAAAaCATGCTACTTGAATCATTTGGAAAGTGTATTAAACTCACAATAATATTCGATTAGGTGAAAACTATTCCCCTAAACACCCGACCACGCAACACCACTTCGGTTCTTTTCGAGTTCCGCAATCAGCGGACTGATGTTTGTGGGCGGGCATTCACTTAGGGGGATCATTTACAAAATAAGTGATACGTATTAATGAGTGATAATTTATCACGCATTTTGCGAATGACCCCTTAGATTTTTTTAACATTTTGATGCGAAAATTTACCATTTTAATTAAACTAGAAAAAGTAGCAATTTGGGTTTTCAGTGAAGTTTTAAAACAACCAAACTCAATTTGaTGT

>contig00119

aCTCGAGCACTTGAAACGTTTACATTTTCCAAAGCTTCTGACTTCGCAAAT

>contig00120

ACAACTGTAATATCATTGTTACGgcAAATATCGTCAAcAAAAaaCCCTTTGTCTATCATTACCTTGTCATTTGCATCTAAACATTGAACTAGGCCACTTTGTTCAAAAaTAGCTTTATCAGAAGCGCGACCACCATAAGCTTTAcTTGTAAAAGATATTGTTCCAGCAGGAGTTACCCCAGTCATGTATTTTACAGTATAATCTGATTTTTAaTTGGAATAAACGACTAaCTGACAGCACAAATTAtATGgTTTTtGAATCGAGATTTCAGTGCAGTCTAACACGACTCGAACGTCAGGAAAATCGCGAAAGTGAACAGGAACATTTTGCGAAATTTCTtCTTTACTCGGCCAAGTAATTAAaCATTTCAAAGAGGAACTCAaGACGTCTATCATGCGATTAAATACTTTTtGACAGTATCGGTCTGAACAACCCTTAAAAAAAaCTGCCATTGCCGCATAACTTATATTCTGTTtCAA

>contig00121

CAGTATTCGTTTGTCGATTGAGAGCATTGTTGTGAAGTTTGCAGAAGCTGCATAACCCGCTTCAGCCAACAATCAACTTTGCCTTTTAACCGAAAAACAACTTCTAAATCTTCAAAATGCGTGAGTGTATCTCAATCCACGTTGGTCAGGCTGGAGTCCAGATTGGTAATGCCTGCTGGGAACTTTACTGTCTCGAACATGGCATCCAACCGGACGGTCAAATGCCATCTGACAAGACTATCGGAGGTGGTGATGACAGTTTCAACACCTTCTTCAGTGAGACTGGAGCCGGCAAACACGTTCCCAGGGCGGTGTTCATCGATCTCGAACCGACAGTAGTCGACGAGGTCCGAACGGGAACATACCGCCAGTTGTTCCACCCCGAACAACTCATCACAGGCAAAGAAGACGCAGCGAACAACTATGCTCGCGGTCACTACACGATTGGAAAGGAAATTGTCGACTTGGTTCTGGACCGAATTCGCAAACTCGCCGACCAATgT

>contig00122

AtaTTCCACAAAAGTAAATACaCCGTAAATGTGACCACATTGACTTTAAATGGAGGTTGCACGAAGAAAAGCTACTGCGaagAGTGGAAGGTGATCTGTGCATCATgAACATTTGTTTGAGTGAATGAGGAGAAAATGAATGTGTTGCAATGCATTCGACGATATATCACCTATTTTGAGAGAAAAaGAaGAAAAACAAAaGAAAGGAGCAAGACCAGGTctGTTaTTCGGCGGATTTGTAATTCTAGTAGTAATGGGCGGTGTTCGTTATAAATATATAAATATaTATAAATACATTATATAATGAATCATAGGTGATATGATTTGTGTAATAATGATAAGTATAATGATAATTATAACAATAaTGATACAATATACGGTAATAaCATTAAAGATTTtttGGCACACGACTCTTGTAAGATAGTAGAGG

>contig00123

TGTAGAAAATGGCATCTAAAAAaTATATGGTAATCATGCTTT

>contig00124

GTATTGACAATCATTTTTGGCAAATTGGCGCAATCTGAAtCAAACAAAAATTCCGGCTCTGGACGAAAGTAGACAACATCATTGCTCTGACTGTCTCTGTTCCCGGAACACTTTTAAGTGTAAAAAGGTTAGGGGCTAAAATCAATGGAAAGAATGGCCTAAAATGCATTAGAAAGATTTTATAAAACGATTATTTAATCACTGACGAAGATAATACTTTGTCATGTTGTAAACATTCAATAAATAAACTGCATAaC

>contig00125

ATATGCGTTGAACAACTcgTGACAAGAGTCACGTTCCAGC

>contig00126

TACAATTCATGGCTGGTGATTCTCGCTTGGTTAATATCTCACAGTGAAAaTATTACAAATTtAAAAaTAAAaCTaGATCATAAGCACTACAAAGAAGCCGATTTTGCCAGCaTTTTGGGGCAAATGAGACGACTGGAGTCCTTTTTTTtATACCATTGTGATTATCAGGAATGGAGTGGTAATTTTCTACTTAAATTGCCATTGGAGACAATTCAggagATTATTTTGACGTGTGATGGTAGAATGCCTTATGATACTACTaTATTGTCAACGATGATacaagaaTGCAAAAACCTTCAAACGTTTGGCATAGCTTTAGATGAAAATAAAGCACCTAGAATATTGGCATCTTTACATTTACATGCTGAATCTCTGAaAAATTTGAAACTAAAACTACGTTTCAAATGGAGCGGgCAATTGCAATCTCTATTATTGTCAACACTATCAAAGTTGACGCACCTTGAAAAATTAGATTTAAGCCAaTCCTGCTCAATTATTAGGGATGATGTTTTTGAAGGATTCGCAAAAATTTGCgAAAAAaCTGACGTATTTAGACTTGTCTTATTGtAATAATGTAACGGATCGTGGAATTTCTGCAATATGTAATTTgAG

>contig00127

GAGTACTATGGATCTTATGATCAACTTCTTAGAAACATGTTTGACCACACTAGTGACAAGAGTGATAATTCTTCGTACGTTCCAAATGCTCTTCAATATTTTTTCACCAGCATGAGGGATCCATTGTTTtATCGAATCACATATATGGTTATGAACATCTTTTTGAGATTCAAGGAACGTCAACCACTCTATACACATGAAGAACTTTCTTATCCAGATGTAAAGATTGAATCAGTTGACACTGGAAGTATTTTAACCTACTTCGAGCCCTTCGATGTGACTATCAATAATGCTCTAAATGTAAAGAATCTTAAAGATGGACTTTCCTTTAACATCAAAGCCAGGAATTCTCGTTTGAACCACCAACCTTGGAATTTAACCTTGACTGTAAACAGTCAGAAGCATACAAGAGCATCAATTCGGGTCTTCCTTTGTCCAATTGATGAAAATCAAAAATTGTCCGATTTCGTTCGTAAAAACTACATGAAATACGTGGTACAAATAGACAAGTGGACGGAGAGTTTGAAACCTGGtACCAACATCATCATACGGAGTAGTCGAAATGCGATGTTTACGCAAAACTTTGAACAAAATGCAGATTCTTACCTGGAATTTTACAAGAAGCTCCAGAAAGCTGAAGAAAAATACGAAACATTTGAATTTTCTCCACCTCGCTATGGTTTCCCTAACAATCTGCTGATCCCGAAGGGTAGGCCCGAGGGTATGCGATATAAATTAATAGTTTTTCTTCATAATTTCGAAGTGCATAAGAGACACGCAATTCCGCTCTTCGGTGAACATGCATATGATGGAAAaCCTCTTGGGTTTCCGCTTGATAGACCAGTGGAACCATGGATATTTGAACTTCCTAATGCTTTTGTCACTGATACAATGATTTATCACAAAGATGATGCTCTTAGTGACTGTTCTACAAAAAATGAATTTTAATTTtGAAGATCATATTTAAAAACGTAGCCTTTACCAATAATGCCTTGTGTTATAAATTTTAGTTTAGAAATGT

>contig00128

aCTTTCGCATTGCTAATCCTTTCTGGCTGTCAGCAACAAGCTACAACCTGCCGAAAaTTTTAGATTtAAAAaTTCGTGGTGAATATTTCTTTTTtATAAACCAAAAATTGATGGCCCACTACCACCTTGAGCGTTTGTCTAACCGGATGAGTGAAGTAGAACAATTAAACTGGAATACAGATTTtCCTGGATTTTCCTCAAACCTGAAATTTCCGAACGGTGTCCAATTGCCTCAACGCAATGAATCCTTCACTCTGCCTATATCCAAAaTTTCAGACCTACAGAAAaTAAGCGAAATTGAATCGCGATTGATTGAAGCAATTGACTCTGGATATTTTATTGACAAAGTTGGCAACAAGATCAGCATCTTCGATTCTGATAGT

>contig00129

GACTtGAAGAATGCTATCGAAAAaTTAGATGACACCGAACTTAATGGTCGCAGAATCCGTTTGATTGAAGACAAGAGACGCGGACGTCGCTCAAGGTCCTCCAGTTCCAGATCAAGGTCACGATCCAGGTCCCGATCACGTCGTCGATCTCGCTCCCGTTCAAGGAGTCGTCGCAGCTCTAGAAGCCGCAGTCGCCGCAGCAGCCGCTCCAAATCAAGGGCTCACTCAAAATCTAAATCAAAGTCCAAATCCAAATCCCCCGAGCGCAGTCGCTCCCGATCCAAGTCCAAA

>contig00130

TAGAACGAGATCTGGATTTGGACTTAGATTTCGACTTTGAGCGGTCTCTTGA

>contig00131

ACGATgAATATTTgCTTGATGGAAATTCATTACCCTAATTCATCAACCAAAATTAGAGACGAATTTGGCTTCTTGGATGtAATCAATATaTAAAAAAAGAAATAAAGAAAaTGATCTTAATCCGTATTTAGAACCACTTTCACGTCTTTTtATGCTCAATCGTCCTTCTGCTTCGAATCTGGTGATTTATCACCATTCATAGGCGAATGCGAACGACTACGGCTCTTACTGTGCTTGCTCAGCGATCGAGATCTCGATCTATCTCCTCTGGAACGTTCCCTGCTGGATCTCTCCTTTGAGGGCGACTTCGCTTTGGACTTCGACTGCGACCTCGACTTTGACCTCTCAGCCTTGGACC

>contig00132

TACGCAATATTATTAAATTAAATAAAGATAATTATAATTAGGTGTTGACAACTCAGTATTCCTGAGATGACTTGTCTCCTTTGGATGCGCCTCCTCCAGAGCCAGTCTTCTTCGGCAATAAGACTGCCTGGATGTTTGGTAGGACTCCTCCCTGCGCAATGGTAACTCCAGAGAGAAGTTTGTTTAATTCTTCGTCGTTCCGGATTGCCAGTTGAAGATGTCTCGGGATGATTCTCGTTTTTTtGTTGTCCCTTGCGGCGTTGCCCGCCAATTCGAGAACTTCGGCGGCTAAATATTCCATCACtGCAGCCAAATAAACTGGAGCACCAGCCCCAACTCTTTCAGCATAGTTTCCTTTTCTCAGAAGTCGATGAATTCGACCGACAGGGAACTGAAGGCCGGCCCGGGAGCTCCTAGTTTTCGACTTTGTCTTGGCCTTTCCACCTTTGCCGCGTCCGGACATTTTGAGATATTAAGTAGTGCGAAAAATAAATGAcAAACgACTAATTTCTTCGGATGACGAGAGCCGAATCCCcATGTACACTAATTATCTAAATGGTGAAATCGGATATCACGCTATCTACGGTTTACTTCAAtACAGCCTACATtACGAACAAATTaCAAaTTAaTTaCTCTaTTTGTAcATAAGAATCATCTCTAATCGCTAAGTCTAAGTTTCAAAAACGATCCTTCCGGTTTAATAGAGGACGTATCACAATGAAATAGCAATTCTTAATAAAATATACGGCAGAAGTTTGAATCGAAACACGGTCCAATCATCCGTCGATTAAAAATCACTGTTTTCGCATCGCGATTCAACGAAAATCAACATCTATTTGGAATTAGATCGGGACTTTAAATTCATATACGATAAAAAAAAAAaaCTTtAAGTAGAACGCTTTGCGACTAGATCGCGGATTTTTCGAATCTTTAGGAAGTTTTAGTCCAGGATTAATTTCGAAGCGTTTGGCAGTGAGAATAATAATAAAAGATATCTGAGAAAACAAATCGTCTGAAATTATATCAACCACGTAGTGCATTAAAAACATCAATGCTTTAAAGTATAAGAAAAAAGCGAAAATAAAGTATAGTCTGTAATTCGAGGCTTAAGCACGTTCACCACGAATTCGTCGAGCCAACTGGA

>contig00133

AGTTTtGCATCAGACGCCGTGAGCAATtGCAGAAATTTGTGCTCGTGAATAATAGTCTCTGAACTAATCTCTAATCAGCgTAAATAATGGCTCGTACcAAGCAGACAGCGCGTAAATCTACGGGAGGAAAAGCCCCCAGGAAGCAGCTCGCTACCAAGGCAGCACgTAAAaGTGCGCCATCCACCGGAGGTGTGAAGAAACCCCATCGTTACAGGCCCGGTACGGTCGCTCTCCGAGAAATCCGAAGATACCAGAAGTCCACCGAGTTGTTGATCAGGAAATTGCCGTTCCAGCGTCTGGTCCGTGAAATCGCGCAAGATTTCAAGACGGATCTGCGTTTCCAGAGCGCGGCCATCGGCGCTCTCCAGGAGGCATCCGAAGCATACTTGGTCGGTCTTTTCGAAGACACCAATCTGTGCGCCATCCACGCCAAGCGGGTAACCATTATGCCCAAAGACA

>contig00134

ACGTTTGGCAGTGAGACTTCTCCTCTTTTTTTTtGTAAACAGAAAaCGAATTTAATGTATGCAAAAaTGGAGTTGATTTTGGAGTGAGTTTTTGGAGAGGAACTGTTTTCTTTGTTTACTGACTTCGCTGTTATTTCGCCGTCATTTTCATGATTtCTATTATCATCGTTCTCATTATAATTGTATTTGTAaCTACACAAtAGTGTGTCCTTAGATAAGGTCTTTCATAGTGTGATCCAAGAGTTAagCGAGTAATTAAGACGAACACCTGGTTGT

>contig00135

ACTTTtGTCGGATGATTTTTAATTTGATTCCAATTACAAACTCTTATTTGATGATCCATTGAATTGAAATGGAGCGAGATGTAGAAAGGACTTTTGAATAATCTAAATAAAaTTGTTATTCATCAAGGACGAGATTATGTTAAAATTTTGGACACAAGCTTACCTGTGATCTACGCATCTAATTGTATGGATTGTGTCATAGTATCGATTTACGTTAAGAGTGAAATGTAACGCAATAATATTAAATCGGAATGAAAAGACTTCATCCATCGTTACGAATATTCATAATTATTACTGATAATAATAGCAAAAGGTAATGTGCGAGTTAAGCTCCTTAAGCACGTTCACCACGAATTCGTCGAGCCAATTGAA

>contig00136

GTAGTCGAtCGGAAGTATAAGTCTTCGTTCTATTATCGTGTCCAATCGTGCtG

>contig00137

GTGATAaGAGTCTCAATGTCaGTAGCTGTCGAGGCCATGACAAaTTCCAAATCTGCTAATCCTGACTTTGAGAGCTTTAGTTGGGACGATACCACAAAGAATTTGATTTTAAGCTCCTTCTTTtGGGGTTACGTCTGCACCCAATTTCCTGCAGGGTTGATCGCACAACGCTGGGgTGCCCAGAaGATGTATGCTTTtGCGATAGGTTTGTGCGGACTAGTAACCTTATTGGTCCCAATGGCaGCTAACAaTGGAAGCTGGCAGGCAGTCTGCGCTTGTAGGGTTATAGCCGGATTGTGCCAGGGGGTTGCACCCCCcATTCTACATACCCTCTTAGCCAAGTGGGTGCCATTCCAGGAACgCGGGATGCTGTCGACATTTGTCTATGCCGGTGGATGGGTCGGAAACGTGATTGCCCTGCAAAGTTCAGGTGTCCTCGCCGCATCATTCATGGGATGGCCCAGCATTTTCTATTTTtGGGGAGGCATCGCCTTGCTTtGGTCGATCGTCT

>contig00138

CCCAAAGTTCGCAAGATCCTCAAGCTTTTCCGCCTTCTACAAATTAACAATGGTGTTTtCCTAAAGTTGAACAAAGCAACAGTGAACATGCTGCGTATTGTTGA

>contig00139

GtaCtCGtCAGCGAGATTCGGCCTTGTGCCCTTGTGTCAAGTCAATTCGTGCTGTGCTGGTGGTGCTCGAATTGGCTCCAAATCGCTGAGGGTCACGATAAGTTCGGTCGTCTCGTCCTCTGCAGcATTTCCGTCCCAAACATGGATATGGACATGGGTG

>contig00140

CTATATACTGTTGCCAGCAGTTTGTGCAGTATGCGCCGTTTGTCTGCTCTACGTTTTCGGCTTCAAAAaTGCCGAGGTGCCACCTTTCGCCCAACTCTCCGTCAGTTACGACGTGGCCAGTAAAAAAAaGCGAAGCAAAACCAGGGAAAAGAAGGCTACCAATGGGCAGGTAGCTACCGAAAaGGCGAGTCCTTCGAAAAAGGTGGCTGCTGTCAAAGCTGAAACGGCAAAAAaGTCATC

>contig00141

CAAGCTACAGCTAGGAGTTCAGCTTATTCAGAACTTGGCGAGAAAAAAaCGAAATTAATATTTTCGACTGTGGTGCTGTCTAAAAT

>contig00142

GCAAAGCTGCAGCTAGAACTTCAGCTTATCCAGAACTCGACAAAAAATATTAATATTTtCTACTGTGATGCTGTCTACAATAACTTAAGATGACT

>contig00143

TTATGCTAACCATACTGGTGGGATTGGTACTAATTAATAGTGCAGTAGGAGAGCCTTTCAGATaTCAATTCGTGACAGCCTGCAAACCTGAGACCTGTTCCTGTAAACTaGGATCAACTTCGTATTTCCAAAGTTGTTGCGAAGGCCGAAATCTAGTTTGTCATCCTACCTTAAAAGTTTGTCAAGTAACACCAAGTGCTGCAGGGccGgCATAAATTATTAATTAAACTGCAGATCCAAATTATGATTTATTTTCTGATTCCTATAACAATTTTTtATTATTCTTTTCATTTCTATCTTATTTTTtGTAtAcTtAAAaTATTTA

>contig00144

AATACTACCcAAGCTACAGCTaGGACTTCAGCTTaTCCAGAACTCGGCAaGAAAAAaCGAAATTAACATTTTCGACTGTAGGGCTGTCTACAAT

>contig00145

GTATGCTAACCATACTGGTGGGATTGGTACTAATTAATAGTGCAGTAGGACAGTATTTCAAATGTAAAATCGTGACAGCCTGCCCACCTGAACCTGTTACTGTTCACCAGGATCAACTTCCTACTTCCAAAGTTGTTGCAAAGGCCAAAATCTAGTTCGTCATCCTAACTTAAAAGTTTTTCAAGTAAAaCCAAATGCTTGAGTGCCAGGAAAAATTACTAATTAAACTGCAGATCCGAATTATGATTTATTTTTTGATCCCTATAACAATTGTTTATTATTCTTTTCATTTCTATCTTATTTTTGTATAATTAAAaTATTCATCTGTTTAGTATTATTC

>contig00146

TGCCAAAGCTTGTTATGCTAAACATATTAGTGCATTACAATATGAGCACAATTCAATTCATAACTCTGTTAACGGTTGCATTTTGCTAAATTGTACTCCACAGTTTACCGTCAAAGATTGCAATAACATGTTTCCCcGTCAAAATCTATAAGGTCGAAATTTCAACGCGACAGCTGAAAATTGAACGTGGTCCGAAATTTGAGAAGAAAAACTCTACTTGATTTATGCACAGCCTTATTGATTGTAGGATAAAGAAATTTtAAGTTtAAATGTTCCATCTCTTTATTCGATACAAATATATATa

>contig00147

TAAATTAGATTTTGCGGACAAGTTGGGATTTGGGTTGAAAGCTAAATGTAGGGACACTTTTATTGCCGCAACGAAAGCAACTGTTGATGCTAGAATTCTACAGATTAAAGCATGTCACGATGATAATGTTAAGGACAGGAGATATGGAGT

>contig00148

AGGCACCTACGGGCCGTTAGTTCTACCGAATGATCTGAACACTTTGAAAAGAGAAAATACCGAGATAGTTAGAGCGATGAATAATAAGTACTTGACAACTGTTGCGGG

>contig00149

GCGATAAAATATAGCACCAAAAATATAGCAAAGAAGGTCTTCTCTCTGAGATCCATGGCTGGTTTTTTtCAGATTTCCGAATTCAGGAAAATGATTTAATACTTTCTGACTGCTTTTAAAGGTGATAAATGTTTATTTTtCCACgAATTGTATATCCAACTTGaTATTCAAACGAAGAATGA

>contig00150

CCTGCGATAAAATATAGCACCAAAAATATAGCAAAGAAGGTCTTCTCTCTGAGATCCATGGCTGGTTTTTTTCAGATTTCCGAATTCAGGAAAATGATTTAATACTTTCTGACTGCTTTTAAAGGTGATAAATGTTTATTTTTCCACAAATTGTATATCCAACTTGGTATTCAAACGAAGAAT

>contig00151

ACTTCtAAGTTAATTAAAATTCGGTGATGTCCTAAATTAAAGCACGCTGtCTTCCAGTGAATTGTTcATGTCGCCTCGATCATCGCCAATCACCTCCTGTAAAACTTCGACGAGGGCTCTtGTCtCGtCCAaCatCTCACGACAAAGACGATACtCCTCTtCCATTTTtGCTAAACGCTCTGACAAaTCTTtGTTTtCCTCTAGAGCATCGTCGAGCGCtATTCTTCTtCTTTCTGCcAAGACTtCCcAGTAATTTTCGCTAGGACCAGCTTCGGAAGTTAAATCTTCGACTTCAATCTTGATTTtCTCTCCTTGAGCAGTCTGAATCGCTTTGTCCTTCGATGTGTTCTTTTTACGTTTCGTTGATTCATTTTTAGGTGTATCGTTTCTTACTTTGGTGTTGTCGCTTCTCACCTTGGTAGACATCTCATCCTTAATTGCCTCTTTGACTGCTTCTTTTAGGGAACCGCTTGATCTTAGAGTCCTTCCAGCTCCGACTAGATTTTCTTTATCGGTAGCTGCcGG

>contig00152

AAGGTCACGTCATGAGGACTGGAAACGAAACACGAGTTtCTACCGCCAAAACTGGAGCTCCAG

>contig00153

ACCCCTACTTCCGGAATCGATCGTGCGACAACCGCCAAAAGGGGAAGCGCGATTTTCTCGTTTTTCTACAAACAAT

>contig00154

AAGGTATTAATTCACTTTATTAACAGAAAAATTCAgTCGTcTATATCAAATTGAATCACTCGCGAATTTTGAGTGCAAAAATAaGTATTTCGTCCGAAATTTGTGTTGTTTATTGACC

>contig00155

TttcTgTAGCTACACAGTAATCACGAATTATTCCAAGAAAATATTTCCTCCAGCTGGAAACAATGAGGCTTCTTTGGATTCTGTTCATCACTGTCGCGGTTTTATTGGGTTCTTTAGTTTGCGATAATTACAACTGGGACAATCATGGGGTACACTTTG

>contig00156

CCTATGAAATCCATCATGTGTTGTTAATAAACGCTTTTGAAAACGATCAAATCCTCGGAAATCTTATAAAATATTTTCTGATATTtCAAAATATCTTGAACCCTTATGCGTCCTGAAAAATCGTTTtATATCTTTCAAGATCCTTTGAAACTGCAAGAAAATTTTAATAAATACCTTGAAGTTTATTAGAATATTTTtAAATGCCTACAAAAaTTTtAAAtCTTtGAAAtCCctCCAAATtAGTGAAAtAATTTAAAACGTCTTTGGATTCTTATTTAATtCTCTAAAaTATTTATAATCCTTCGATATTTTTtAAAACATTTTtAAGATCGATCAATCTATTGACAATTCCTTGAAATATTTAAAATTCTTTAAAATGACATTtCTtACAAatCCTtACGtGAGCGGCAaCCaCAAaTaCTGTGGTACAGAAACTCTTTGTCAAGGTATTTTAAAATGGTTTTGCTCCACTTTCTTACTCCACATCATCATAAAaCCCTTGCTTGTTAAGTTACTAATATTTACAGTAATTTTGACTTATTACTTATTGATATAAATTTTATCGTAATGAAAaTTGTATGAGAAGCTTCTTTTAGATTTTAGACATATTTCGGGCAGCCTTCTCGATTCGACACTTTTAACGAGGTTGGATTCAGCCcGTTCTTGGTTATAGCTGGAAGGATAGTATTCGTGAGTTCTTGGTCGTCTACCTTtCTTTTTTtGCAAAAaCCCAAATATTAGCTTGTGCCAAAaT

>contig00157

GCATTTGTAAACGCCCATAAATTTGTCATAATCGATGTAAAAAATGTCGTAACTTATAGCAGGAAGTATTCCTAGTTTGACACTTACTCTTCCATCTGTAGTCGTTTTGCCAACGTGATTGATTGCGACTTTAATATTTCCTTGTAATATTTTTAAAGGGCCAGTCCAACTGACTTCCACACTCGGTGGAGT

>contig00158

ttCTTTtGTtCtAAGGtATTTttAAAatGGTTTttGtctCCACTTtCTtACtCcaCaTCaTCaTAAAaCCCTTGCTTGTTAaGTTACTAATATTTACAGTAATTTTGACTTATTACTTATTGATACAAATTTtATCGTAATGAAAATTGTATGATAAGCTTCTTTtAGATTTtAGACATATTTCGGGCAGCCTTCTCGATTCGACACTTTTAACGAGGTTGGATTCAGCCCGTTCTTGGTTATAGCT

>contig00159

TATACTTGAAATTCAAAaGAAATAGAAAAATTTACGGCGTATTGCACTAATAAAAGCAGAAATTACCGGGACAATCGAAGCCTCATTTCAATAAAaTAGGGTCTGCGTTATTTGTTCTGTGTTTCATCGTAGTGGGATTCATTAATCCTAGTGGTAAAAAAaTGATTCATGTAAGATTCCTCCTTCTACTCTGCATTGCAGCTGTGACTGCTGGAGCTCCTGCTCCTCCAGCTCCTGAGCTtGACTCTTGTGGAAATaGTGTGGCGGT

>contig00160

GAAGCCTCATTTCAATAAAaTAGGGTCTGCGTTATTTGTTCTGTGTTTCATCGTAGTGGGATTCATTAATCCTAGTGGTAAAAAAaTGATTCATGTAAGATTCCTCCTTC

>contig00161

ACTCTATTCGGAAGTTCAGTTTAGAAATTCAATAAAATCGAATAGTTTACATTAAAAATCATTTAGAGGAAGTTGAA

>contig00162

AGTTATAGTCCTCGCCTTGATAATGACGGTTGAGCATtaGGCCAAACCCAAATTTGGACTGGTTGACAAGAAAAaTAATTATTTtCTTGTCTACCTAAGaTTTtATTTTATTTAATTCTACGTGATGTCTTGACACTGTTATATAAGGTAAAGTGTAACCCCCcTTTT

>contig00163

ACAtGGGgAGACGATCCAAAaCAGTAGCTAGGACGTCTACTAATCCAATACACCAGAAAGAAAAGaCCTGAAGTTAAACTTGTCGgCTGTGATCCACCTGTAGTTTGCGTACAAC

>contig00164

AATGAATACTAAAATCATCACCATCTTCACTTTGCTCTTTACGGCTGTTTTTGCTGGATACCCACCAACCTGGTATCTACCTCCAATTTGTGCCGGACGAAAAGTTGGAGTCGAATGTAAAGAAGATGAAAAATGTTGCAACAGTTCCTGCGAAAAGGGTGTGTGCGCAGAAAGCCTTTACACGCTCGATTGCTGTAAAGATCCTAAAGAGTTAGGCCCAGAATGTGTACTGGCACTGATTGGAGAAGATTGTGACTGTGATGAAAAaTGCTGCACCAATATATGCGGTAAAAAGGgAAAATGCCTTGGTGGAAaGAAACCGCTCGACTGCTGTAAGAAGGTTAAATATTAAACGATATGTGCTATTCACTGGACTCTTCTTCTGGTGATATTTCAAATTATTCTATCTAAATCGTGTGCACAATTGTTTCAAATACATTATATAGCCAAGCATtC

>contig00165

ATGCTGCTTAAGAGGCTGCTCAGGCAGATATTTGTCATCCTACTGTCGGTAAGGgCTCAGCAACTTTCTACTGATGATTCAACTGGCGTGACTATGGAAATCTCTGAACATTATATTAATAACCGATGAAAACAATATTTCGTTTGACATTTGAAAAACATTTAGAAATCCTTTTTAAATTGTACAAAaGTATATAAAaTGTGTATATTGATGTGAGATCAa

>contig00166

ACATgACGGACTACCAGTGAGCGAGGCTAGGATATAAATGTCGAGGCAAATCGGCTGAAGTTtCTATTGTGAGTTTTAGTCCTATACTAAATTTTcgaaGGAGAACATAATTTCAAAATGAAGACAACTATTTCTGTGAGATTCCGCCCATTTGGGATGTGTATCTTCATGGCATTTGTAATCATTCAACAAGTAGAATTTTGCTACTGCGgAGAAGACGAGAACGAACCCAGCGATTTTGATAACCTTGAGCCTTTATGCGGTGTAAACTTTGATAACAAGCTAATAGGCCTGTGCCCCTTCGGCACAATGCCCGAAGCTG

>contig00167

GggCTCAGCATCTTTCTCCTGATGATTCAACTGGCGTGGCTATGGAAATCTCTGAACATTATATTAATAACCTATG

>contig00168

GATACGATCTAAAATAGTAGCTAGGACGTCAAATCATCAAATACGCACTAAGAGACACTGAAGTTAAATTaGACGACTGCAGCCCAGCTATAATTTACATACAAcGAAAAACATTTTCTTATCAAAGGAATCAATTATCTCAGAGCAGTAGTCATATTgCGGCTGACAACAAAGGCATTTAGTCACTTGGAAAAAAAaTAGGAACTGTCAATTTTTAAATATATTTGGCTCAAAGAAGGAAGATCATCTAAAATGAATTCG

>contig00169

TGTTTACGCGGCAGACGGGCCAGCTCCAGGTGAGGGTCATGAaGgAGATCCCCcATCATGATAGGCAACCAAATAATTATCACCTACTGCGACATAAGAAATTTCTAATTTACAATTAGAAAATGTAAAAAAAaGAAaTAGATTTTTTtGTCGTATGAAC

>contig00170

ACTtGAAgCCcAGACCGaTCCTATTCTCTCGTATAGATGATTGTTTGCGtGgAaTAGTAAGGTTTTTCGCTATGTTCTTAATGTAATACTTGgTAATTTGTTTAaTTTTTTtCTTCTCCTTTATTGATACTATTCTGGAATGATGCTGAAATTTGATGATTTAAATTtAGGTTCAAAGTGTCGCTGCTTTGGACAATtGATAGACATTGTCTGTGTGGAaGGGCAGAATTTGATGGAAaCAAaCAAACAAAACAAAAAAaGTAAAAGAATATATAACGAGTCATCGAATGAAGGCCTATGCCCcAACAAaTCGGTTAATCTTAATATCGATCATGTCTCTCTTGTGGTCTTGACAaTTTGTtGTTTTCCTCGCGGCACATTCCAGGCTAGTTGGTGTAGCGATATTATCTATTAGAAG

>contig00171

ACAAATTGATTACAAGCAGCACATGGAAAaTTTCTGAACTTTTTCTGATAACAAATATAATGAATTATTTTCAATGTTTTCATTCAAATTAAATCCAAGAGATGCTTTATTTGAATCAATTAAATTAATTAAGATACTTATATCAGTAACAAAACTATAATTGGACAGTGAAAAAGATTTGTTATTGGGAAAATTCAGATTGAAATTTTtAAaTTTtAAATtCTTTAAGATTGTCACTCAGCATGAAACGCAtCTTTTTTTGATGtATTTTTTTtCGGCAATCtGTAAGGAATCAGTAACCCTTTtATTTtCTAGaCATATTTtGTTCTATTTtCTTTtGAAGGAATAaGATAGAAGAGaGGGTGCAAAGTAGAAAAGAAGTGTGTCACTGATTCTGAAAATCGAGGTCATATTTTCCTTTTCCTCGGTAATGACGATCGGATGTGCTCGAACGATAGTCCGAATTCTAAAGAGTATTTTTGTTGGTGGACTTTTTGTAATACG

>contig00172

ACTGTTCTTCGCAGCGGGTTCAACTTTCTTGGTGGCTGGTTTGCTCTTGGATTTTTTTTtCGAGTCATCGCCATTCTCGCTTCCTCCATTCTCAACTACCTCTTGACCATTTCCATTTTCTTCTGGCTCCGCCGACTCTTCTTCATCCTCCTTCTCGCTGCCTTCAGCAACCTCATCTTCGGCTTCATCCGCAACGTCCTTGGCCTTCTTACCCCTGCCCTTTTTCGCACC

>contig00173

ACCGAAGTCGCGGCCAACGAAGAGGGAGGCGATTCCGAATAGTAAtGTTTTTAaTtCTTGATTCTTGATaTtcAaCATCCGtCACTGCAGGgCTTGGAACTTGAACAGGACACtATtGAGATATGTCTCGTAGTAAAaTTAATATtAAATTTTtATAAACACTTtGAAAAGgTAATAAATAaTCAATCACACGAAAGTCCCGAGCCCTAGCGAATATTTACTATATTTACATCTATAAGAATGTAaTGTGACTTGTGTATAGTTtCATTATTCTGTGTGaTTTtGAAATACAATACTACGTGtATTATTTTAtAaTCCCTAGCAATAGAATAGATGtATTTTTCGATGTTTAAAAAtCGAAaGCATCGAATCCaTCGAAGT

>contig00174

CAaCCTCATGGTTCAGTCGCAATtGTTTCGACGAATTTTCACGGGACTTCTAAAaTGTTTGTGACGCGAGCACTTCATACAAATTCAAGCGAATGTAGCGGGTTTTTCCCAAGTGCTGCTGCTAATCCACCATATCAATGGTATTCGTATTTTAGTTAGTCCTAAGGTTTTATTGGCGAAATGTTGATCGTAACCGATATTGTACTTTATATTGGATTACTTTTtCAAATCGATAAAATACCCATTCTTAAAaTATTGTTAATATGGTTGTATACTTATTTACGTGGTACGCCCCATCAATTTATAACAATAAGTAATTCCAGCGACGACGAAGTTCAAGTTCTACGACACATTACATTTTCAAATTGCTTTTGAAAAAGAaGTAGAGATTTTTTtGtAGTCTGCTTATGCAATTTTCAGTACAAAGTTCGTAATTTTCTTTtCGTTTCGAAGTGTTGTGTGCTGAAATTGAATGCCGGAGACTAAAaTCAGTTTCGATGCAAaTTTtAAATTAGATTTCGTAATGATTCAAGCACTTTTATTGAAGTCACTTCTTCAGTGTGATGAAATCTCCAGGTGTCGAAGATTTTAAtCT

>contig00175

TTCATCGGCGCCCGCCTTTTTCGCCGCTGGTTTGCCGCGAGCCTTTTTCTTTGGAACTTCAGGGACTTCGTCGGATTCGGCGTTGTCCTCGTCGTTCATAGGTTCGTCCTCATCATCGGATTTTGCAGCTTTCTTTGCCTTCTTTAAAGTAGCGTCGTTCTTCTTGGTTGATGCCTTGGACTTCTTCACCGGTTCTGGTTCTGGCGTGACCTCGGGTTTTTtATTTTTCGCCGCACGGGGAAATCGGGGCTCTCTGGCGTCATCGCCCTTCTCGGTGACGGCTTTTCGGCCTTTCGCCCTTTTTGGTGGAGATGCGTCATTTTCACCGGCGCCTTCAACAGCAACCTCCTCTTCGCTGCGCTTGTTCGTCTTTCGTGGAGGCATTTCTAAAACTTGGACTTAAGTTTAGCTTGAACACGCCGAATTTCTATCGATGGTTTTTCAGGGACTTCGGGTACACTACCGAAGCCGGGATTTTGAGATCGAGATGAATTTGAGGTCCGAAGGAAAATTCGGAACTGCAGCGCGTCGTTTTGTGGGTTTCACCGGTATTTTATAAGAACTTGATGAACTGCCGGATCACAAGCTTGTTATCTTCCCACGCGACTGCACGTTGGCGCTCGAAGTCTCGAACCACGAAt

>contig00176

AGCGGCTTTCGTTTtCTTTTTtGACCTAGAATCACTGATTTCCTCCTCTTTTTtCAGCTTATCGTCAGTTTGTTTTTTtCTCGAGTTCTGGCTATTCGTTTTAGTCGTTTC

>contig00177

AGTTTGTTTGTCCAGATAATTTGGTGGGCATTTGGTTTGCTTCGAGCGATTCTTATTCGGTGCGGAAATCGCAACCGCGTACCTGATGTAAGTATTAGTCTACTAGGAAAAAAaTCTGGCGCTGCGTTTTTCTTCGTTATCCTCTTCTAATCTTTATATCTAAAGTCTCGATCTCGAATCTCCAGGCTTCAGATTGTTCTTTAACTTAGTGAATATTTCTCTAATTTCCAATCTTACCATTTTAAGTGAGTGGCATCACGTAAATTGAATTTAGCAATTGTAAATTAAATTCAAGAGAGTATCAACAACGCATTGACTCTGGGGTCGTTGGATTCTGCGAAATCATTTtAAAGATGTTTTCTTGATTAACTTTTTTTtctACTGTAaTGTAAATTGTTTtGACGAATAAAGGTAACTTATCTAt

>contig00178

GTACAAGGAATATCTTAGTTTCCCTTCCTTATTCTACGCGACTTTACATCTTCTTTTTACTATTTATTAGTTAGTCGATTAAAATACAAAATTATTCACTCATTCTAAATGTCTCGCAACTCACTCATACACACTTAAAATTGCCTTTtAAAACAACAACGGGTAGTATTTATTTATTATTCATTACTTAATTACTGCTGCAGCCGACGATCGGAAAACTATTATTTGTTACTGTTAGAGTTACTTCTTCTACTAGTAATGTAATGTAACTGCAAGTGCGTTTAAACTAAGATGAATGGtACTATGTACAGTTGACAAGGTGTGCTTCGGGATCTAAAACATGGGATGACAAAAGACATATTATTGACGCGCGCGTTATCCACTATTATCCAGTCTAGTCACGGTCATCATCATCTGGGCCATCAGCTGCGTTCTTCCTGTATGCAAACATCTCCCTATCGTATCTGTCTTTATCTTGTTCGGCCATCTCTGCGTATTTGGCTCTATCTTCGGCGCTGATGACGGCCCACTTTTTGCCCAAAGCTTTTGCAATGTCGCCCACCGTGAACGATGGATTTTTCGATTTCACCTGAGCACGCTCGTCTTTGCAGAACCAGAAAAATGCAGACAAAGGTCGTTTTGGCGCGTTTGGGTCCTTGACGCGTTTCTTGCCGCCGCCCCTACTCCTCTTTTCCGTTGCTGCTTTCGGCATTTTGATAAAAATTCCTGGTATTCTCCAGAAAAAaGGGCA

>contig00179

TCCAAaTAATAGTGATCGTAGTGAGAACCCAAAAGAGAATTAAATCCTCGCATTTCGTATCTCACTGGAATCGGTTCCATATCCACAGAaGACTTCGTGGATTTCTTGTAACGAaTCCAAAGCGTGTATTTGTTAACCTTCTCTCCGAATTTTGTAACCAAACGCCATTTTTCGCAGGGAAGTCCGTTAATTACTTCTTCGCCAATGCACTCCATtCCAGTGGTGTCTGGAATAATGGTTTGAGGCtGAATTTTCATATCACTGGTTCCATTCACTTGGAGACAAACTTCATCGTTTTCTTTGGTTTCGGTTGTAACCGGCGCCACTTTAATACTCGATCCATAAGATCCcTCGTTACTTAACTGGAAAGTCTTAaCCATTCCTCCATAATAGTCGATTCGGCTAGATCCACTTTTGGCGTCATACCAGGCATAGAATGGTTCTCGAATTTCCGCATAGGGAATGTAGAGAGTTCCTTTTGCAGTATAAGCGCTGCTAAATGTTGGCGATTTtACTACATTTGTAGCGT

>contig00180

CGTTAAAACCGGTGGCGAAGCTTTGCGTTTGAGTTCACGAGACAACAACCCAACCTTAACTGTGGTGT

>contig00181

ACTTTTTGGAAAaCTtATAGAGAAGACATTTTTTATCATCAAAAAGAGG

>contig00182

ATTTtCTTGATTTAAAACTTTTAtaGCAGATGTCACAGtgAAATGACGAATGAACTTCTTGATGTATTCCTTTCcTATGTTtGtATAGCGAATGTCTAGATTtGAAACTTTtATGGCAGATGTCACaGTT

>contig00183

GAATTTTTATCTGTTCTTTCTGAGTTCAG

>contig00184

CAGACAATTTGCTACACTTGGGGTagACGATATAAGTTGCTGAGAAAAACtCAtATTTtgTGCTTTTTCTTTAGCTAAATCTTCCTTTAGAATTAtATTTtCTATTTtAGGTGtCCcAaTTTCATtCAAATATTTtCCATTTAAACTCTTATTCCTCGCCTTATTCTCATCCAATTGAGGGTTAACTAtCGTTAG

>contig00185

ACGACCCTTGGCAAGTGATTGTCTCTTGGTTAATATCACACAGTAAAAGACTTACAACTTTtAAAATGGAAATTAAACATAGTCACTATAAAGAACAGGATTTCTCCGAATTTTTAAGGGAAATGAAACAACTAAAATGTTTCCATTTATATAACTGCGaGTATCTAGACTGGAGTGGCAAGTGTCTACTTCAATTACCGTTTGAGATATTGCAGGAAATTTTtCTGATGTGTAATTGTTTAAAGTTG

>contig00186

TTTATATTCGCTCCAGTtCCAGTGACGCATAAATCAAGtCCGGTATTAGTATTTCGCGTTCTAACAATCTCAGCAGCAAAActCAAAAGCAAATTAGTAACTAAAGGACATTCGCTAACATCAAGTGCCtGGAGTTGATGTGCTTTTTC

>contig00187

CACTAGATACTCTACGTtAGTAAACTAAAGAATAAGAGTTGGTTTTtATAAGCATATGTAGATATtATGAAAACTCTTCGAAATGAACATTCTTATTtGTAAACAtCAAAaGTTAATCCAGAATTTAAAGTATTTAAAAAAaTTAATTACGCATTTAATtATTTTCGGTCAGCAATACGTATTATATTTAAAACCTAAATGATGTTACCTTTAAATACTATCAGTGCTGGCAGACAATGGTTCTCTGTAAACACTTTCAATGCCTGTAAGAaTATTAGTATTTAAGTGTCATGAATTTTAATAGTTCtATTTCATTTCAAATCTTCtAGTTAATTTTTtAGATCGATTTACTAGAAATATTTCAAGTGCATGTAGACTAATGAAATtCTTTCTTATAATCAATTTTTGATCCGTGATATTTGACTTGAGaTATTTGAACCTCTGGAATATCTTGAAaTTTAAGGACATGCTACTGTATAAAAATTTGAAG

>contig00188

TACAGAAGGAGCATCATCTTCGTTGTCGGATTTTGAACGTGCAGTGCTGTTTTATTTTCTGCTGCAGGTCGTCCTCTCTTTTTtGCTTCCTTTGCTACACCCTTGTCTGCTTTTGCTGGTCGGCCCCTCTTCTTCTTCTGATCATCTACTACTGCAGAGTTGTCGTCCGACATTTTGTTGTATCTTCTTCACTGCTGTTAAAACTCAAGTTTGCTGTGTGGAGACAAAGGCAGCTACCGGTCGAAACGGACGCTGAATACTCTGCTTGTGCTTGTCTGGCGTTTGGCTTTAGTGT

>contig00189

AtttGGGgACACTCACTCTCGTCACTGCTCCGCATCACACGTTGACGCGTCCACTAGTAACAAGAATCCGTTTGAATCACACCGATCGTCATGGCAGAAGAATATTTATACGGAATCACCCTCGAAGGCCCCAACGCCTCCGAAGTATGGGACCCAGAGCACAGAGCAGAGGATCCCGACGGCACCAGT

>contig00190

ACGTTCACGTTGGTTAAGGGTAGTGGACCCGTTCACATAGTCGGACACAATCTTCTGGGGTCGCACATTGAAGAATTCGAAGATTTTGAAGATATGGAAGATGAAATGGAAGAAGAAAACATTGACGATGAAGATGACGAGAAGG

>contig00191

CAGCACATTGGTGGGGACCAGAAACTCATTATCAAAATGGCACTCTTAGGTCCGGAGGCTAAAGTAGGCGAACTAAATGTTCTCGAAGTAGAGGCGACGGGGCTCAAAGGACCTATTAAAATCCCAATTGCTTTACTTGAAATGGGAAAGACGTCACAGATAATTCTCGATTTAAGTTTTCCGGACCCACCAGTT

>contig00192

ACAATATAATTACACTCGAGAGCAGGCTTAAAAAAAAaCAACTTTCTGTCGGAGGTTTtCAAAAAAACAGGTCAAAAAGGTCGATAATAATAATCTTGCGCCAAATATTTCCAATTATTGAAAAaCACGGTGTAATAAAAAATTGATTGCATGGAAACATTTGCCAAAGTTGATTTCAAAAATCTCGGGCCACGTCTGCCTGATATTACGACGATATTCGTTGAAACGTTGATGATAGTGTTTACGAACTTATTGTAAAAAGCCTAACCTATGAGTGTTTTTCGTTTtGTGTAGTCTTAAAAGTTCAAGTTTATTTTTtCTTGTTCTTGCTAGCCTGACTTTtGTTTTTAGCCGCAGATGACATTTTGGCATTCTTCTTCTTAGGTTCGTCGTCTTCGTCATCTTCCTCTGGGT

>contig00193

CACCACAAAaGAAAaGAAAGCCAACCGCAGAGGCTAAAAAAAaTGGCACCAAACGCCCAAAGACGGACGAAGCAGTAGATAAGTGATATGTTAGgAAaTCCaTTCCGTATTATTAATTTtaCTTGCGtGCATGGATACGTTTTAcATTGACTCGTCTATTAATTATTATTGATAAACGTCGCATTAAAaGTAACGAAgAAaCAAATCTGATCGCCATAGAAGatCCTAGATGT

>contig00194

ACCAGCAGAaTAAAAGACTTTGCTGAGTCACAACGCTAGGCAATATGACTACATGTGACAGTGGTCAAGGCTCCGCCGATGAGTTGCTGTGCACTCCTCGTCGAGCGAAGCGACTTTTTGGAACTGGTGGCAGTTCCACTAAACGCAGCCTCTTCCAGCAGG

>contig00195

AGATTTTGGAGATCGAGTGCAAGATTTTGGCGAGTCGGGGAAGATTTtACGTCGTGGAGAGGAACGAGCGGTTTTGGTGACGTGTCTTAGAGTGCTAAATGGAGAGAGACGTTTGTCGGCGCTTCCAGATGGCTTTAGACGATCCCAGGTGAGATTCATGAG

>contig00196

CGGAATTTGAgaGGAGACGCTGCTGGGACTGCGATCTGTGAGAGCGAGTGGcGACATTGGACCCAGGTCGGACTCATCGCCTGAATTTGTGTCCATTTGGTTTtGAAGACCGGCCTGCTGGAAGAGGCTGCGTTTAGTGGAACTGCCACCAGTTCCAAAAAGTCGCTTCGCTCGACGAGGAGTGCACAGCAACTCATCGGCGGAGCCTTGACCACTGTCACATGTAGTCATATTGACTAGCGTTGTGACTCAGCAAAGTCTTTtA

>contig00197

CtGGTCTTCAAAACCAAATGGACACAAaTTCAGGCGATGAGTCCGACCTGGGTCCAATGTCCCcACTCGCTCTCACAGATCGCAGTCCCAGCAGCGTCTACTCTTCACCAGGTCGCCAATTCCTCTCTCCTCTAGCCTCACCTGAAGGCTCGCCTCAAATTCCACTCATGAATCTtACCTGGGATCGTCTcaaGCCATCTGGAAGCGCCGACAAACGTCTCTCTCCATTTAGCACTCTAAGaCACGTCACCAAAaCCGCTCGTTCCTCTCCACGACGTAAAATCTTCCCCGACTCGCCAAAATCTTGCACTCGATCTCCAAAATCT

>contig00198

TTGGGGTGCTTCTGGACAATGGCAACTGGAAGATGAAACTGTTGGTGCACCAGAGCCTTCAGCGCCTTCAGAACGTTCGGTTCCAGGATCTACTGGAATTTGGCGTGAATCAGTTGCTGGA

>contig00199

ATGAAAaGAAATCAAACGAATAACACGAATAGAGTTTACGAAGCGAACgAATACGAAACAACAACAAACATGCCAGCAATTGGAATTGATTTGGGAACCACCTACTCCTGCGTGGGAGTATGGCAACAGGGAAAAGTGGAGATCATCGCCAACGACCAGGGCAACAGAACAACACCCAGCTACGTTGCCTTCACGGACACGGAACGtCTGATCGGCGATGCCGCCAAGAACCAGGTGGCGATGAACCCGGCCAACACCGTCTTCGACGCCAAAaGGTTAATTGGACGCAAGTTTGACGACCCCAAGATCCAGAGCGACCTCAAACACTGGCCGTTCAAAGTCGTCAACGAAGCAGGCAAACCTAAGATCCAGGTTGAGTTCCGCGGTGAGGTGAAGAGGTTTAATCCGGAGGAGATCAGTTCAATGGTtcTGACGAaGATGAAGGAAaCAGCAGAAGCGT

>contig00200

GCTGTATATTTGTGCAAACGAACAATGCAGAACAAAACTCGATTAGAGCTCGCAGACTACGAGGCTGAGAATCTAGCAAGACTGCAAAAAATGTTCTCCAGGAAGTGGGAGTTTATCGCGATGCAAGCTGAAGCCCAGAGCAAAGTGGATAAGAAGAGAGACAAGTTGGAGAGAAAAGTGCTCGATAGTCAAGAGAGAGCTTTTTGGGATGTTCATCGACCAATGCCTGGTTGTGTCAATACAACAGAACTCGACATAAAGAAGGCATGTCGGAGCCACAAACCAATTGTTAAAGCCAGTAAGCATTTACAGCTGGGATTAGCAGGTGGAAGGATTTCTCCGCCTCGT

>contig00201

GGTTCCAAACTCCATATTTCTGGCCCTCGAATTGCTGGGAACCAGAAAATACAGATTAT

>contig00202

GgtGTTGTCATTCTTTACAGTCAAACAGTGGTCGtCTATGGGgAAAAAGTaTCCATGGGATGCCATAaGATGTGCGACGTGGAGAGCCTC

>contig00203

ACATGGGGGcAGTTAtAcTCCTCGCCTCGAAAaTGACGGTTGAGCATTaGgCCAAACCCAAATTTGGACTGGTTGACAAGAAAAaTAaTTATTTtCTTGTCTAcTtAAAATTTTATTTtATtcAATtCTACGTGATGTCTTGACACTGTTATATAA

>contig00204

TGTGCAATTAGATAGACAAATATCGTTTtAGTATCCTATACGGATTTGTAGGAATTCTACTATTTATAATGTAATAAATATGt

>contig00205

TGCTtAAAGTTCTATGAACAATTgAaCATATTTAgATACCCTaagTTAAAAAaCTtAaCATAtCcTTAAAAaCCACTTGATGGGTTtCTGAATATAAAagAAGTGGGTCGGAGAAAaGGTGGCCCGTAAAATTCACcAGTGATGTGACAAGGCagTtaTTCCTCTCTGAATCATtGGTATGCCgGAAGCTGTAGCAAATCGTgCATTTCCTTGGACATTTGTGGCCATCATACCTAATTcaTTTGCGATTTCGTTTGTAATTTCCATCAAGGCGCCCCATCGGCTGAGCGTCACCATTTGCTCATATTCTCTTTGCCTtTGCTCTCTGAGAGCTCTCCACTCATCGGcGTAAGGTGCAGAATCCATTTCGTATTTGTTGAATGTCGTATTTTtA

>contig00206

AGCCTTGTCACATCACTCGTGAATTTTACGGGCCACCTTTTCTCCGACCCACTTTTTTtATATTCAGAAATTCATCAAGTGGTTTTtAAGAATATGTTAAGTTTTTTAATTAGGGTATCTAAATATGTTCAATTGTTCATAGAACTTTGAAGCATA

>contig00207

AGTCCACAAAAGGGAACCGGCGAGAGAAGAACTCCTGACGAGCCACACGGATATTCTACGTTTGAAGTGAAACAAAATTCCGCTTGAAATAGTCTTTCGTACTAAAAATACGACATTCAACAAATACGAAATGGATTCTGCACCTTACGCCGATGAGTGGAGAGCTCTCAGAGAGCAAAGGCAAAGAGAATATGAGCAAATGGTGACGCTCAGCCGATGGGGCGCCTTGATGGAAATTACAAACGAAATCGCAAATGAATTAGGTATGATGGCCACAAATGTCCAAGGAAATGCACGATTTGCTACAGCTTCCGGCATACCAATGATTCAGAGAGGAAT

>contig00208

ACTTTCTGGATCAGCAGCAAAATTAAATTCGACATTAGGACGTTCATTTCCAAATCCAGAAACACTAGATCCCATGATAAATAATTCATCCACGTGATTTATAAATGTTGAATCGATTGTCATCGCTAAGGCTAAATTTGTGTGAGGTCCAAGTGTAATTATGCTTATCTCTCCTGAATGTAATTTTACTAAATCTATCAAAGCCAAAGATGCATGTTTAGATCGATTAATTTTGCCCGCAATTTTTTCAGTAAATTCAAAGTCTCCTAATCCATCATTTCCAAAAATCGTTGAACCTTTAAAaTTTTGTAACAAAGGTCTCTTTGCTCCTCCGTATACTGGGATATCATTCCTCTTTGCAATtGTTAACGCTTTAAGAaCaTTTTTCTCCACATTTTCTTCCTTTGTGTTACCaTAagtAcAAGTGATTGCAATTATTTCAATACTTCCTTTATTAGCTGCTTCATATTTtAATGCTAAAAGAATTGCTAAAACATCATCACATCCACCATCCGTATCAATTATAAATTTtCTTGGCTTTCTTAATTCTGGCATAAGCACACTAGCACTTATAATAAGTATTACTACCAAAaTGCAGTATGTTAAAAAaCATATTTTCTTCATTTTAATTTCTTCATGAAATAAAATTTTCACAAATCTTTCCCGTTTTTtCGTAATGTTTAATACTTCGCACTAGTTTTACGACCAGTTACTGCGATATCACGAAATTGAGATAAGGCTGAATTTTCTGTGATTTTTTCTCTTATATTCGATTCCACGTTGTATGTAAATTATAGGTGGACTACAGTGTCTTTTATTGTAAGTTTGATTAGTTGTCGTCCTAGCTACTTTTTtGGATCACATGAAACTTGGTGAAACAGACGTTCCCTATTTaCAC

>contig00209

AATTTCATGGTGATGAATTCTATTAAAAAGCCATGTATTCTAATGCCATGGGAAACTGTTTACAACTCCAACCCTGGAaGGGAATGGCGCGAAAATGTTTTTGGGAAATTAAAGTCTTCTTCAGTGGACTTTTTAAATAAAGCAGAGGCTGTATTCTTCCGGTTATCAAAAAaCTCAATTTGGTCAGCTGCTGATGCATGGGCTATAGCtATAGCAATTTGGCCAGAAATGaTAAAAAAGTACGTAATTACAAATGTTACGCCCGTTTATGATGGAGCAGCAAGAGGGTCAGTTCTCGTGGATTATTGGAATTTAACTCAAAATCCACACAATGCCAAAaTAATTCAAGAATATGACTTGGAACTTTTTAAGGAAAAaTTaTTGCAATATTTTTCTGATCCCTGAAATTATACATATTTCGTTAAAAaTAAATTCAGGAATTTTCTGTGAAAGCTTAGTTTTAAAACtGCAATTCGAACCGAGCAGATTCCCTACACATAAAAAAaCaGAAGATAAACTTtACATCGATTTTAGATGAAAGATTCACAGCAACAAAAaTTAaCCTGGCCAGATAATCTTTACACAAATGTCGATTAAaGTTTCTTTTGTTTTtCCCtGAAAACACATGTATTTGAAAaGACaCGATGTTGtCTTAATAAAACTTTATCCCT

>contig00210

CCTTGATGCTGGCATCCTCGAAGGATTTGCGAACATTATCTTTGGTTGATGTTTGAAGAGCCATTAGTATTTTATTAATTAAAATAAATGCACTGCACGAAATTTACGTCGTTCTGCTTCGATTGGTATATTAAAGATGT

>contig00211

CTAAAAAtAAaTTAATTATTTGAAATAAAGTATAACCTTTTGACTCGGTAAGTGGCGACAATTCATTAGACTGTCTCTAACTCCTAGGACGAATTTTAAATATCCCGCAGTCATGAAAAATATAAGAATTTAAAGATCGATATCGTCAATTAATAGCCGTTTCTCTATCTCGAAATTTTGAAAATGTGGGCCAAATATGAAACGGACTCTTATTTCAAGAGTATAGTAGAAAAATGTGTTTAAAATATGTTTAGGCTGGTAAAACTCGAAGTTTTAAATATTATCCTAAAAGTCAGCGTTGAGTGTGAAATTACTCCTCTCCTGCATGACACCCCACTTTTGGTATTCGCCAACTTTCTTTtCGAAAAAATTTGTCTTTCCCTCCAGTGAAATGTGTTCCATGAAGTCGAATGGATTTTCGGAATTGTAGACATTTT

>contig00212

TGGCACATCAGTTTGCAGTTCATTCCGATCATTTCTACGGGCAACGCGTCTGTCAAAAATTcTtGCTCAATTACTACTGCATCTTTtATTATTGAAATTACACCTTCTTGTTGAGGTTTCTGAACGATGTGCTTGAACATGAGGCAAGCAAAATCACAATGTAGTCCCTCGTCCCTAGAAATCAGTTCATTGCTGAAAGTGAGACCAGGCATAAGACCTCTCTTCTTCAGCCaGAAAATAGCTGCAAAACTGCCGCTGAAGAAAATGCCCTCGACTgCTGCGAATGCGATTACACGTTCCGCAAAaGTAGCAGTATCATTACTtATCCAATTTAAAGCCCATTTTGCTTTCTTCGTCACGCAGGGTAGAGTTTCAACTGCATTGAAAAGGAAGTCCCTTTGTGTCGGATCACTGATGTAAGTATCGATGAGAAGAGAGT

>contig00213

ATGCCAATCTGGCAAGTCCTTGGATAGATCTACTTCTTCTACGGTCCAAAaTGAAGCTTCGGCC

>contig00214

GTACATTTGCCAAATATCTGGCCACTGAATGGGAAATATGACGAATCTACGAGGATTTTCTTTGAGTAGAGGTTCCAACTCTGGGTCGAAAaCATCTTTATCTTTAAGGTTTAGATTCCTCACAGCTTTTGTTGCATCAATTTCTGGTCTAATTCCATTTTCTTGAGAGATTTTTGGCGAAACGCAGTGCTTTTTCGGAGACAAATTTT

>contig00215

AcATAGAATTCGTGGCTGACCGGTTGCTTGTAGAATTAGGATGTC

>contig00216

CTATATaCCcACAAAAaTCACAAGTAAATTGTCGTTTGacTGcTGCATGT

>contig00217

ACAATGTCTAGTCAACGGGtAAGAATATTgtATAAAGCCCATTCCTTcTtCTACTTATTTTtACCAAATTTACACTaCCAaTTtCAATAAaTTCCGCaTTAAAAAaTAGTAATTTaTATCGGTaTCGAAGGAGAATAAATTGACTCTTTCGGAACCATCAgcGAATTGAAAGTAATGCAAAAAAAaGTTTTTTATATGGTATTAGATATTTTCTCAAGTTTAATATTTCATGTAGgTTTTTGTTtCTTTAGATGGCGTGAAGTAATTTGCCTTAACAATtGTGATTTCTCATTCG

>contig00218

GTGATAAATCATAAAAATTAACATTATACAATGCTCGGAAAATTAATTGACTTTAGAACTATTTATTTCTTTTGGTAGAAATTTGTATTTTTGTTTGGTCAAGAATGTCACTTTCAGAAGAAAATTAGTCTTATTAGTTGAAAATTCGTTTTTTTTtGTACACATTTTTTtCTTTCTTATTGAAAaGTATGCTTGTATTTTTTtGCTTAAAAATTCGACTGATTTAAAAAATACATATTATTTTAAATAAATTATTAGCTGAAAATTTGTTTTTAGATGAAAGTTCTATTATTTGGTTGAAAATTGTTTCATTTTTATAAAAATCTATATTTTCTAGTAGAAAaTAATTCCACTTGGTTTAAAaTGTATTTTTCTGGTTGAAaTGTAATTCGTTTtGTTAAAAAGTTAACTTTTTCGGCATGAAAaTTTAATAATTAAAATTTTTTtCTGGAATAGCGT

>contig00219

ACATTTAAAGTGTTTAAGTTTAATTCGTAAATTGTTACTGTCGTTTGAATTTATTGTATGGATGATAACAACAAGAAGCCACTCCCTATTGTCCTTCTACAATAT

>contig00220

GTTGAATCTCGTTAATTTGATTAGATATATGAAAAATTTGGAACAAGAACATTATGAAGCTATAGTTAATACTTATGTTTCTATTTCAAATATTATTTTCTGGTATTTGAATACAAATTGTTAAAGCATATTATTTTtCATTATTTTTTtGGCAGAAATTTTTTAAATTTGAAAaTTTCATTTTTTAtattCCTGGTGTCaTaTTTtGCGT

>contig00221

ACGACATTCCaGAAAAACAATTTTAATTATTAAATTTTCaTGCCGAAAAaGTTAACTTTTTAaCAAAAaGAaTTACATTTCAAACAGAAAAATAAATTTTAAACCAATTGGAaTTATTTtCTACTAGAAAATATAGATTTTTATAAAAATGAAACAATCTTCAACCAAAtAATAGAACTTTCATCTAAAAaCAAaTTTtCaGCTAATAATTtATTTAAAaTAATATGTATTTTTtAAATCAGTCGAATTTTTAAGCAAAAAAaTACAAGCATGCTTTtCAATAAGAAAGAAAAAAaTGTATACAGAAAAAAAAAAaTTTtCAACTAATAAGACTAATTTtCTTCTGGAAGTGACATTCTTGACCAAAAAAAATAAAAATTTCTACCAAAAGAAATAATTAGTTCTAAAGTCAATTAATTTtCCGAGTAGTGTATAATGTTAGTTTTTATGATTTAGCAT

>contig00222

ACCATTGAGTTGAAAGGTGCCGTGCAGTTACTTAAAAGCTGGTGGAAGCTCGTATTTGAGAGATATTCGCAGATAGTCGAGGAAGAACGCCGTTGTAAATTTCCTTCTAGTATCTTCTGAAGAATCGCAAAGATGCCGAACATCAAGTTGCAGAGCTCTGACGGGGAAGTTTTCGAGGTCGATGTGGAGATAGCAAAATGCTCGGTGACCATCAAAACTATGTTGGAAGACTTGGGAATGGATGAGGACGAGGAGGAGGTGGTGCCACTGCCCAACGTGAATTCTGCAATTTTGAGGAAAGTTATCCAGTGGGCGACTTATCACAAGGACGACCCTCCTCCACCCGAGGACGACGAAAACAAAGAAAAGCGCACCGACGACATCAGCTCCTGGGATGCTGATTTTTTGAAAGTTGATCAGGGAACTCTTTTTGAATTGATTTTGGCCGCAAATTACTTGGACATCAAGGGTTTGCTCGACGTGACATGCAAGACTGTTGCAAACATGATCAAAGGAAAGACGCCCGATGAAATTCGCAAGACTTTCAACATCAAAAATGATTTCTCCAACGCCGAGGAGGAACAAGTGCGCAAAGAGAACGAGTGGTGCGAGGAAAAGTAGATTGTCCCCGTGACTTCACCCATATTTCATATTTAGGTTCTTTGTTTTT

>contig00223

GAAAAATCAAGAAAAATCAAGGGAAAAAAaTAGATGTTTtAATAAAAGTGAG

>contig00224

aCGAAtGAGATATTCAACTCTGATTCTTTTCGTCCTTTATAAGAAAAAaGTTGTCTCAAgAATAAGAAAAAACAATATATAGAAATTAGACAATTCAAGAATTCTTTTTCTTTTATCTCGACGAtgTCATAATGTCATATTACAAGTAATTTTtAGAAAAGGGCGTATTGAAATTAAGCATGATTCTTACGcATACGACTTGCGTCGTGAAAGAAGTTTTCAATCaGAAAaTTAAAGAAAAATCAAGAAAAaTCAA

>contig00225

CTTGTATAAAATCTAaTTCTaCATAATAAACATTTCAGCTGAATGGTATAGTTTAAACTTtAAGTTCAATGATAATCTATATTTGGAGTGTTTCTAGGCTTCCTAATTCAGCATAGCAATCAGAAGTCTTTTGCTGCCTCCGTCACAATCACT

>contig00226

ACGAACGCCtCTCTGGAAATAGCATTGAAGATGCTGTAAaGAGAGAATTCTCCGGCTCTCTGGAAGATGgATACCTCGCAGTTGTAAGATGCGCTCGTGACAAGACTGTATATTTCGCGAAAAGATTGCATGAAGCAATGTCTGGCATGGgAACCACAGACAAAaCTCTCATTCGTATAaTTATCGCCCGATCAGAAATTGATTTGGGTGATATTAaGGAAGCGTATCAAGAAAaGTATGGAAAGTCTCTCGCTGGCGATATCGAT

>contig00227

ActAGTATATGCAATATTTTTGTAGCCAGTAaTTGGGTCAGAAGCAGCCTGGACAATCTCCTGATCCCAGCTGGGTGTTCCGACTAAAaTTAGGTTTGTCTTATCAATTTTCCGGATTTTCCTAATTACGGCTTCAGCATAAGGCTTCACGTCGTATTTCCATGAGGCATTTTTCAATGGCTCATTGAAGAGTTCATATATTAAGTTGTTTTTACCAACATAAATCCGTGCCATTTCTTCGAAAAATGCAATAGCAGCATCGGTATGTAGGTAAGCATTATGATCATGCCAATCAACGATGGCATACATATTAAATGCAATCGCAGCATCGACAACAGTCTTCAATCTTGCTTTGTTACTTTGGGGATCTTGTAAaTAGCCACCATCACCTTCAATTCCTAAAGCAGCGCGTAACACCTTAGCTTTCCAGTCCCTCCTCAACCAATATACTGCTTTCTTATTGTAGAATTTTTCTCCACCCCAGTTGTTGTTGGTCCAAAAAAAaCTCATACCAGCGAAACTTCCTTTGCGACCACCAATCAATATCTGGTTTTCTGTCACAGATAACGGCAACACATCTGCATTACTGATGGATAAAAAAGCACAAAGGAGAGTAGCAACAATGAGAGCCATATTGTATGGCTGAAGTAACCGTAGTCAGTAAAAATTTTTTAAAGTGACTGATGAATTTTTATCAGAATGCCTTGTAGTTTTATATGCTTCACGACTTGAATAATTTACAAATTTAGCAAATCTTAAATGATAATTTCATCAATAATCCGTTGGCGAACT

>contig00228

ACAtGGGgACACGCGCGCGTAACGTCCGATTGGAAACGTTAGTTAAATTAAAAAATCACATTCATTCGATTCTGCGAAAAAATCATTATTATTCAAAAGAAGTGGTATACGCGCATGTTTCTCCT

>contig00229

AGATAGAAGTTGGTTAATCATGTTTACAGTTCGCCAACGGATTATTGATGAAATTATCATTTAAGATTTGCTAAATTTGTAAATTATTCAAGTCGTGAAGCATATAAAACTACAAGGCATTCTGATAAAAATTCA

>contig00230

TTTCAGGGAATTTGGTACATAATGGGGGAATATTATTCTGAAGATAATTTGGTACAAAGATGCACAAGGACAAGCTGGACGGTGAACACGACTGGTTCAATAAATGTTGAAACCATCCTGATAGACACGGCACACGACAAACAAGTAACATTTTATGGCGTTGCTAAATTAAATAAAAAGGAAGCAAAATATAGTATAAAATATGAAGGTTTCGAATCTAAAGAACATTATATTTTATATACAGACTATTTGAATTTTGCCATTGTATACACTTGCGCTGATTATACTTCAGAGGACGGAGAAAATAATGTTATTCTTTTGACAAGAGTAAGAACGTTCCGAGAAGTGGTTCTTTCGGATTCTTTGCAATTTATTATACGTAACAAGCTAATCGAATATCCACTTTATACCGAAGATTGGGAAAAATGTCCATCTTATAGAACGGGCTGCACAGTATAGTAATTGGTACAATCGGTACTTGCAGCTTTCTATTAATGAAATATAACGAAGTTTTTGAATTTGCTATTGAATCATTCGAGTAAATAAATGTAATATCTTTACTTGTCAATAAATATTTtG

>contig00231

GTTCAAAAaCAGCAAGTGAAAGAAGAATATTCCCAAATCTAAAAGGCTCAAAATGATTCGAGTGGTATTCCTCGTTCTACTCTGCGCTGGAGCTTTCGCAGATTATACTAAGTCGTCTCAAGATTGTCCAAAAGTGAAACCAATGCCAAATTTCAATGTGACAACA

>contig00232

ATTATtCGTGGAAGTAAGTGAAAATGAGGTTCAAAAAGCGTTTAAGAGGGAGATGTCTGGTAAGAAaCTGTAAAAACGCTTTAGAAAaGCATA

>contig00233

ATGgTAGAAACGTGACTTATCATCGT

>contig00234

ACTTTtGCGAAATACCTtgTAAACAAAaCGATCACGATTATGACCGTAAATCAGAGGCAAAATACATTTCTTACGGAGTGTTAAAGGAATGTTTGCAAAAAAaGaTGATTCCTGTGATAAAGGAAAGCACGCCGAAAaGATCTCCATCACCcACTTTACAtGAATGCAAAGAA

>contig00235

AGAACCATCGCGTTCATCTAATAAGATGTATTATTCAACAATACGTAAAAGCGAGACTTTTTTACgTAG

>contig00236

ATACTGTCCTAAACAGTAGCTAGCTCGTCAACTAAACAAATACACGATAAAAGACTCTGGGTTTGAATTCGTCGACTGTCGTCCACCTACGATTTACATAAGTCCCACATATTGCGCAGGACACAATAAGCACACAGCCAAAGAGTTTTCGCAACTCGGTCAAACACATCTTAAGGGCGGTAGTAAGAACGTGTTCGCTGACAGGTGTTAAGAGAATTTTACGGAATAGAACCAAAGCTAGTTGAAACTATAACTTGAAAGAACCACTATAATGTCTTCTATTTCCACGTTTGTGCTAATTGCAGCATTAGTATCAGTGGATAGTTCCTACATTGTAAATCCTACAACCTGCTCAAATGGAAAAGTCGGCGATGTGGGAGCAGCATGTCTAGTAACATGTGGAGACATAACTAGACCTCACACGAAATGCAATTCAGCATATGCAGGGACACCTATTCAAACCTGTGTATGCAAGGATGGATATGTGTTGAAAGAACtgtATGGCAACGAATGCGTTCCGAAGAGTTATTGTGCCACATTTAACACAAGACCTCATTAATACACTTTTTTGTAAGTTTGTCTTTCTAATTGTATCCTATAAAGGACAATCTATATCCAAGCATTTCAAAAAaTTAAaTAAAAAaTTCAAGTTC

>contig00237

GTCTGCCTTTTAAAAATTCTGTGGACAGTCACATTGCGGCATGGTATAAATAGGGGACCATGGTATCACCAATTT

>contig00238

AAAaTTTCAGGGACTTTGGTACGCAACAGGGATGTATGAACTCCCTTCTCCTAACGTAGAAACATGCGGTACTGTAATCTGGTGGCCGTGGGCTAATGGTTCAGCAGCTGTTTATATCAGCTCGATAGTAGACGAAAAAACAAAACCATTGGATGGCGTTGCGACATTAATTGGAACTGATGGAAAATATAATATAACAGTTGATGGTGGAAAAACTCAAAGAGAAGAATGGATTGTATATGTAGAATATAAGAGTTTGGCCATCAAATTCACTTGCGGTGCTAAATCATCTGACGGAATAAATAATGTTATTGTTTTGACAAGAGTCAACACACCAGGACCAGATGTTATCAAGACGGTTAAACAATTTCTTGACGATCACCATCTAACCGGCAAGATGGTTTACGCAGATTGGGATGGTTGTCCCGGCTTGGGTTGCTAATAAAaGTAATTTTTACAATCGATGCTTGCAGCTTTCTTGTATTAAAATATAACGAAgTTTTTAAATTtGCTATTGACTCATTTGAGTAATTACATGTAATATCTTCACTTAAAaGTAAaTATTTTGTTTtATA

>contig00239

AAGTTGAAAATATATAAAGTAAAaTAAACATTTAAATATTGaGAAAACTCGACACTAGAAAGTAGTAaTACCATCaTCGTATTTGATCAATCTTAAAGtAGTTtGTTTGATCAACATCGTCAAAGGCaTGGCAAAAAaTACTCAATGTCACTTTGATTGTCAAATCCCACCctATCAAAACGTTTGGTTTTCGTAGCACATATTAAtGAGCTtCCAAATaTTCATTTTCATTATCATtcTCATTCTTATAAAGAAATTtGTTTtCTTTTTtAACTTTtGAAGAGATAG

>contig00240

ACGTCAAATAAAATATTTCGATGCTtCAGTTAAGAAAaTATTTTGCACTTGCTTATTAAATATAAAGTAAACTTGATAAACAAATTCGAAATCGAAGAAAT

>contig00241

ATTTtGGAAGCATAGGTATTGACACCAACTTGCCATCCTTGGTCGTGATTTTCGGACTTGCTGGCGACTGACCGATCAATTTCCCTTTTGG

>contig00242

CGGGACTCTTCATTGCATCGACAGCCGCTGCCATTTCCAAATCGAGTCTTCGCACTTGAT

>contig00243

ACAGTTTtCGTtACTGTGCGCATAGATCCTCTAGACTTGTGCTTTTGGTTCTtCTTGATCGTTTTGACGGTTACGTTTTtACCTTTACGCCAGTCAATGACGCAGCCCTTGCTTTTGtAAATTTCCGGACCCTCAAAACTGAaTGGATCACTCTTCTCTGGCTCACATTTCATGACGTATTCCTTGGTTAAAaCAGAATTGGTgAaTATTCATTTGATTCAAAGTGGAaCTCTAAAaCAAAGCCCATTGGATCTTTTtCAAGGAATATCACTTTCACGTCATACAAGTGTTTAAGAATGGGTTCATCATGTTCCTGAACCATGTCCGCTAACATGCCCACATTTTtAAATATTGTGaGCCAAAAaTCTGGAATGCCCTTAGCGTTCTCGTTCCCAGAATCCTCCTTGTcTTTTttGCtCTcAGCCTCTTTTTtATCTCACTCTCATCCTTGA

>contig00244

ACTTctAACATtACTTCTCAGATTCTGCGGAGACCTGATGTTCTAGCTGCTTTACAAGAACGATTTGACGCTCACAagATGGAG

>contig00245

ATCTATCCACGTCAATTGGATGGGCCAACAAATCaTTGTCACCATAAAAAaGTGCGAtAGGAATTT

>contig00246

GGATCTTCGATAAATCGTAATCAGGAGGCACAATGCTATTATAAATTCTCATATTTCTTAAGATTCCATAATCATAAGGTCGAAATTTATCAGAAATTATTtCTTGACCAAAaTGAACTAGAGATTTAGTCGACGTTCCAGCAGGTGTGTGAGCTAGAACCACTGGCAAAaGAGTCATATTAAATTGACTCGCATCCAATCCAAGCAAAaCAAAAATTGCATTAGCACATATTTCTTCTTCTAGAGTGTCAAGTTCGCATCCGAATTTCGCGAGAAATCTCAAAATTCCACTTTGAGGAAGAAATTCGTT

>contig00247

GGATCTTCGATAAATCGTAATCAGGAGGCACAATGCTATTATAAATTCTCATATTTCTTAAGATTCCATAATCATAAGGTCGAAATTTATCAGAAATTATTTCTTGGCCAAAATGAACTAGAGATTTAGTCGACGTTCCAGCaGGCGTGTGAGCTAAAaTCaCTGGCAAAAGAGTCATATTAAATTGACTCGCATCCAATCCAAGCAAAaCAAAAaTTGCATTAGCACATATTTCTTCTTCTAGAGTGTCAAGTTCGCATCCgAATTTCGCGAGAAATCTCAAAaTtCCACTTTGAGGAAGAAATTCGGT

>contig00248

CGCTCCGAGAAATTTCGCTATTAGCTCAATATTATTTGAAAAaGGTGCTAGGATCCTAATTGGACTTTTTAAGTGTCTCATAAAaGCAACTGGAGCGAGGCTAAACATGACTCGTATGTTTGAATTCATGTCAGGTCTTTGTGCAGCCATCACGTAAAATGCAGT

>contig00249

AAAaTGAACtAAATACCTATTTTTGCAACCTGGATATTTGCAGGTATATTTGACCCTATCtAAAGGAGcAaTCTCAATtACCGTGTGCAAGTTACGCTCTCAATTAAGATTATAAAATCTCTTTTAAATGAATTCTACGAAACTAGATATCACAGGCCTCATCAGACTTGCACTCTCTtGCACCTAATCTAGGAcTtAACTACAGTATTTAtAAAGACTTTGAATCTACATTCTCTTCCTTTTTTtACTTAAAATTAACTTACGCTAGGTTAACTTTaCTGAATAGACTACATATCACAGTCTTCTATAGTTTGGCTTCATCATCACATCTTGAAATCTTCTgaCATGTtCAATTTGATCTTtGGAGAATTCAGCAGCTCAGCAGTAGCTTGTTGAGTTCCTTCTTTAGCAGTAGTTTGTCGTTTCCTAAGTTCTGGATCAGGT

>contig00250

AGATAAAAAGAACTGACGTGTCACTTTGCAAAAAATGACCGCGATTAGAGGATTCGGATTACTCGCGAGTCGATGTTATCAAATCAAGGAGCAACTTTTTTCATCAAGAAACTTATTTTACACAAATGTCGGAATCTCTATTTCACTGTCTGCGGCTGGTGATGTTCTTGAACAACACTACGAAA

>contig00251

ACTTTtAATCTCGACGATGTTTTCTTTTCCCTGAAAACCGTATCGATGATTTCTCAAAAAATGAATCGATCGAAGGTCTAATTCGGTGATCATTGACATGCCAGAG

>contig00252

ACTGTGACTTGTATCATCATGATGTATTCTTAACAATTTCTAAAGCCTATACTATGTCCGACATGCTTTAAATCATACCACTCTGTAATAGCATCAAGTGTAAGAATTTTTCGATAAATTAATGTTGTGATGAACATAAAATGGAATATTTCACGTATGTTACGAGTGTGAGAAAGATCATTTCTTTGTAATAAAATAAGAGCAAGTGAGTAATTGATGTCCTGTTTCTAACCAAGTATACACTGTGAGTGAATGTCAAATTTGTATTAAAGTAAATAAATTCGCAGAATaCTCCt

>contig00253

TGATGGTCTTTCCTGTCAAGGTTTTCaCgAAAatcTGCATtCCTCCTCGGAGtCTGAgAaCCAAATGTAAAGTAGATTCTTTCTGGATGTTGTAATCAGAAAGaGT

>contig00254

CcTtCTGGATGTTATAGTCTGAAAGTGTGCGACCATCTTCCAAcTGcTTtCCTGCAAAGATCAATCTTTGCTGATCTGGTGGGATTCCCTCCTTATCCTGAATTTTCGCCTTAACATTTtCAATCGTATCaGAAGCTTCAACTTCCA

>contig00255

ACTCAGACTCCGAGGAGGTATGCAGATCTTCGTCAAAaCATTAACTGGAAAaCTaTCACCCTGGAAGTCGAGGCGTCTGACACGATTGAGAATGTGAAAGCTAAAaTTCAGGATAAGGAGGGAATCCCACCAGAtCAGCaaaGaTTGAtc

>contig00256

ATATACAGTATTTACAAGATTTACAATGTTAACAATAAATTCATAATGCATTtCGTGCTCATAATGTCTCAGATATGTATCTAAACATCAAAaTATtAAAATgAGGGgTTGTTCAAaGTAATACAATTTTAAATTATACCTCTTGAAGGATACATAATTTTACCGCGAAAGTCTTTCAAAATTTTAAATTTGCAAAATGTAGTAATCGAATTTTATCATTCTAAATTATAAAGTTTAAAAATGCTATCGTTAA

>contig00257

TCTGAACCTTCGCCCACTAGTATTCTAGACTATATAAAGGCGACGACCGCGCCCGGGAGTCAGTCCGTTATACGCATTTTTAGTATTGTAACCAAATTTATTAATAAAGAACGGTTGAATTGAGG

>contig00258

TTTATATATATATAaTTTAAAAAAAAaTCGAATGACTATGTGACGAAGAAGGTAAGTAGTGTATGATAGCAAAAAAAAaGGGCCTTGCGATAAGGAAAGGggtGCGGGGGCGGTGCTGTGATCCCAAGAAACACAAGAGTCACGATAATCATGTTTGTAAGATTtttATTTtATTATCCCATAAGTCTAAAGTAGTAGTCTTTGCGTCGTGT

>contig00259

aCGTGAGTGGTCTTGCACAATACAATGGGAAtAAATTATAATACTTTGGCTACGCTTGCGGGgTCGAAaCAACCGTGtCTTTTTtCTtGTTCGTTTGAGCGAGCGTTTGtGGGACTTTCGAGtAGTCGGTGgTCTCCCCcTACTATCGTtcTGAAGAAAAGAAAAGCTTTCTCTCCGTAAATATCGGtAaCCGtATATAAAATAAGAAATCATTAATAaGTCAGGGTGCCCATCAGAATGTCGTCTCCAACCGAGGCACAGcAGATTTGcGTTTtAAAAGaGCATCCCGGCTGACCGAACACTAACCTACCTAACCACAGGCTGGCAATTCTTcTTGTTACTCTAAATATTATTTCATACAAAGATCTTGGTACGATTGAAAAaGTGGAGTCTTAGGAGTCGCGACTCGTTTTATTTTAAAGATACaTTTGTGTGTCTCGTTAAACGATATGAAAAATGAAAAaGAGCACTATCGATCATCGTTAGGATGCCTCTGCAACAACCGTGTTCCGTGCGTGTTTTTCCGaTTCGAAAGAAGCTAGCTACCTTCATCATCCCGGAGAGACCAGAGGGACACCGCGGAAGTTGATCTTGCGTCCGCCTTTCAAGTAGCGATGCAGAGGCTGCTCTCGGGgTCAGGGAATGTGAAAAATGCGAGGAAAAaTTAGCGACTTGACGACGACGCTTAGAAGCAcTtCCTGTGGACGATTGAGGGACCGGACTCGTCGTACTCCTGCTTCGAGATCCACATCTGTTGGAAGGTCGAGAGGGAGGCGAGGATGGATCCTCCGATCCAGACGGAGTATTTCCTCTCTGGTGGTGCGATGATCTTGATCTTCATGGTGGATGGGGCAAGGGCAGTGATCTCCTTCTGCATCCTGTCGGCGATTCCTGGGtACa

>contig00260

ACGTTTACTTTGAACAAATTAGAGTGCTTAAAGCAGGCTATCTTCGCCTGAATaCTGTGTGCaTGGAATAATGGAATAGGACCTCGGTTCtATTTTGTTGGTTTTCGGAACCCCGAGGTAATGATTAATAGGGACAGATGGGGGCATTCGTATTGCGACGTTAGAGGTGAAATTCTTGGATCGTCGCAAGACGGACAGAAGCGAAAGCATTTGCCAAAAATGTTTtCATTAATCAAGAaCGAAaGTTAGAGgTTCGAAGGCGATCAGATACCGCCcTAGTtCTAACCATAAaCGATGCCAGCTAGCGATCCGCCG

>contig00261

ACCTGCGTCGTTAGCATCTTCCAATTTCGGAATTCCTTGAAGCTTCCTTTGCTTAGATTTTGGACCCAGTTTCTGCAGTTGACTGTCTGCTTTAAATTTGGCCCAAGACAACACGGATTCTACAATTCCGATTTtAGTTACAGTGGTGATAAaCTTTTCACTAAGCACACACTTTGAACCAAAGCTCTTAGCCTGAAGTGTCATGTTCTCTTTAGTTTGAGAATCAAAGGTAGGATTGTTAACGAGACAATTCACAAAGATCCACATGTGATTTTtAATCTGGAaCGGTTtAATAGCGATACCCCCTTTGTTTTtCTTCTTCAGAGTCTCGAGGAGTTGTTTGACAATCATGTCTGTTACGTGGTCCACATGTCGACCGCCTTTtGTTGTTGCAATGCTGTTTACAAATGACATTTGTTGGAATCCTTTGTCAGACAACGTCATTGCTACTTCCCAACGAGGCCCGCTGTGCTCGTAGGCAACTTTAAGTGGGTTTCCGACGTCATCTTCCTTGCCTTTGATATACAGATCAACGTAATCTTTAAAGTTCTTGACTGGAaCTCTTGT

>contig00262

GAGCGTTCAGAACTGCACTGGATGTGAAGGAAACCGAGATTGAAGATTTTTGGAAAAaTATTGATGAGTTGAAATTAAAGGAAGTGTTTCGTCGTATCCCcGAGGGCTTACATAGCAAGATTATGAGAAAGAAAAaTACCCTGAAAGACAAAAATTCAAATTATGATGAAAAAGGCCCAAAaCTTACGAACGCAAAAACTAAACGTCGCACATCATCGATAGCTGGAGAAAaTAAAGAAAaTATGGTTGGTCCTGATGACATGAAGGAaGATTTACCTCTCACCAGCAAAAAAGACCCCGGATTGAAAGAGAAAaCATCAGGTGCGATTAAGAAAAAAaTtAATGAAAAGAAAaCGGAAAAaGGTGATCATTGTCACACAAGTGTAACAATTGCAACAAACACGATATATTCACGTTCAGATGACGTCGGAAAAACAGTGAGAAAATCAAAGAACATACGTGTGTTAGCTGCGAAAGAAAaCTTACCGACATCCCAGGAAACTG

>contig00263

AACTAcGAAGAGAGTGAAAaGGAATATAAAAATGAGCGAAAACgTATTAAATAACGTCACTgATCATGCTGGTGAAGAATTTAATGACACAAGTGAGG

>contig00264

TTCTAtCAGTTTCTACCtCGG

>contig00265

GATCGCGAGTTGTTTCGTAAAAaTTGAGGTtAGCCGGTTAACAGCGCGAAAATGGAAAAAGTGTCTGAATTAAATAAATCAGGATGCAAGGAACTGTTGGTGTGGCGATTGTTTATCGCTATCACATTGAGTATACTCTTtCAGTTTTTCCtCATGAGCGTTGTTATACTCATGTCAAATTTGAATCCCATTAGTCCACTCTCGTGGGTTCAAAaCACGtGGAACGTCGTCTTCAGCTTTCGAaTGTGGTGTTATTTTCTGCTCCTTG

>contig00266

CTTGTCGAATCGATTTTttATATTCTGCAGTATGCTGGGACCGAGAAACGTTTTACTTTGTTGTG

>contig00267

AGTCTtCAtACGTCTTtGGAGCGTTGTGGTCACGAAACAGTTGTTAAAATGCAGTCATTTATATTGTTACTGCTACTATGTATGCTTACATTTAGTGCTAAGCCTTCGAAAAGTCGGGAAATGGGTCTAAGCCCcAATGATCCGAGTTCTTTCGCTAGACCTGAACTCGTAGCTGTCACCCACATTCATTTAAATCTTTATGTAAATTTTGATAAGAATATTATAAAAGGATATGTAATTCTTGATGCTGAGAGAAAAATAAAAACTGCTGACACCCTAATTTTGGATATCCGAAATCTCACGTTGATAAAAATAaCGAACTATCAAGATGATTCTCAACTCGAATACAGTGTGGGAGAAACTGTTTCGTCTGGTTCCAAGCTGAGTATAAAaTTACCCAACATTCCTGAATCAAAAGATGGTAAAaCGAGGTATAAAaTTAAAATTCAGTATGAAACTTCGCCAAAaGCAACTGCTTTGTCATGGCTCGATCCTAAACAAaCTTTtGGTGGCCGGCATCCTTATCTTTtCTCACAGTGCGAGAAAATTAATGCCCGGTCGATGCTACCTTGTCAGGATACGCCCGGCGTGAAAGCTACATACAGTGCTGAGATTATTGCACCAATTTGGGCAACAGTGTTAATGTCAGCTTtGCACAGtGGgACCAAaCAtGtCGAtCAATTtGCAAAaCTtAGCAGATTCTATCAaCCaGTTCCAATTCCCTCGT

>contig00268

ATGGTGGTTGAGGATCGGGTTGAAAAGCTGGAGGTTCTTCACTCACTGAAGAAAAaTCTTCTATTCCTGTTTcAAaGCTTGATTGCACGACTTCCTTTGTATTCCCGAGTTCCTGTTCTCTGATTTTCAATATTTCTAGCGCTTCTTCGATGAAACATGTTTCGGGACTGTGGGGATCATCAGAAAATtCGAGTTGCAGCTGAACTCGTTCACACTCGATTATGTCCAAAaTATCGTTAGGAGTAGCCAAAAGTAGTTTAGAGTAAaCTTGGCAGTGGGAGTCATCCTTAACATAGAAG

>contig00269

TtCAATAGCAGCAAGGGAAAGAAGAATATTGCCAAATCTAAACGGCGAAAGAATGATTCAAGTGGGATTACTCGTTCTACTCTGCGCTGGAGCTTTCGCAGCTACTACTAACTCTTCTCCAGCTTGTCCAAAAGTGACCGCAATGCAGAAGTTTGATGTGAAAAAATTTCAGGGATTCTGGTACGCTGTTGGAATATATGCTTATCCTCTTAATTTCTTACAAAAATGCGTAAGCCAAAGCTGGACGCAGTTCAAGAATGGTTCAATAGGATTACGGATAAACGTAACAGACGTGGAACAAGACAAACGATTAAGCTATTTTGGCTTTGCGACATTTTTtGGAAATGAAGGAAGATATGATGCAGTATATGAAGGTGTACCAATT

>contig00270

GACCAACAATTGATTTTATATACAGACTATCATACCTTTTCCATCCATTTCACTTGCGCTGATACATCAGTTCACGGACAGAATAATGTTTGGATTTGGACAAGAGAGAAATATCTGCTACAATCGGCTTATAATCAGGCTTCTCAAGTTCTTAAAGATAACCAGCTGAGTGTAGATTTACTAAATATAGAAGATTGGGGAAAGTGTCCACCTGTGCACGTGCACGGCTAATAACTTTTTCATTAAAATGTCCCTGTTATTAAATTTtCATTAACAAAAAaTTCATACAAATTATCTGTTTGAACTTTTTAAATCGAAAATGAAATTAAAATTCAATATAGTAATTTCAAAAATGGTTAGTAGAACGTTTCTGCAAATAAAaTATAAGGAATTTTTtCAAATTGCTGCTAACTCATAGGAGTAATTACATGTATTTTCCTCACTGAAAAATAAaTATTTttGTTTTaCtCG

>contig00271

AGTCCCAAGAAGGGAAAaCCAACCCCcAAGAAGGGAAaGGCGgCCAAGGGTAAGGGCAAGAaGAAGGGCAGCGATAGCGACAGTGACGAAGATTGGGgAAAGAACAAGAAATCCGGTGGTGGTGCGAAAAAAGGGgCcGCGAAGGGCAAGGGTGGTGGCGGATACACACGTCCCATCACGCTATCCCcAGAATTGGCTGCCATCGTCGGTGCCGAAGAAATGGCCCGACATGAGGTCGTCAAGAAGGTCTGGAGCATCATCAAGGAGAGAAATCTCTATGACCCGAAGAACAAGCAATTCGCCATCTGCGACGATGAaTTGATGAAGGTTATCGGCGTCAAGCGCTTCAGGACTTTCGGTATGATGAAATACCTCAAAAACCACTTCGTAGACTAAGTCGCTGAACTTTTTttCAGTCACCCacAaGTTgtaCGTTgCtGCAACAACGCTTTATATTTtCCGCTGtGCAAGACAATCTGCACTTTTCCATCTCGCGGTTAAAATCC

>contig00272

GTACTCCTCGTCGCTGGCGTCTTCGTCACTCGCACTTTCTTCGTCGTCGGAAGAGGCCTTCTTGCTGCGTTTGCTTGCTGGTGTTGCTGCTTTCTTGGGAGGTCCTCGTTTCGCGGGTTTCTTCTCCTCCTCTTCCTCTTCTTCGGAAGCTTCTTCCTCCTCCTCCTCTTCACCCTCCTCAGACTCTTCACTGGCGGCCTTCTTCTTTTCTTTCCTCCGTCTTGTTTCTCTTGCAGACACTCCATCACGAGATCGTCAACTTCCTTTTtCCTTTCTGAGAGGTCAACGTCCAGTTTCTCTTCAATTTGTTGCCTCACTTTTTtGGCTGACATAGTTGTGAGGTCCGCATCCTTAAGAATAGCGGTGATTTCTTTACGAAGATCGTCCTTGGAAAGATCGGCCATGGTGATGTTTCTTGTTGT

>contig00273

CTTTAAAAAAATGTAAAAATAATATTAATTACAGTTTTGAATTGTCGCTGAATTTTGATATTTATGTAAAATTACATTATTTATTAATAAATTCAAGTATTAAAGTAAATTATAATTGTAAATAATGATTTTATTGCAGTCAGAATGGTATAAGAAACCTAAATTAAAACCAGGGGTTTTGCCATCTCAATTTCCCAACTGCCCCGTGCATTGCTCGAAGGCTGCTAAAAACATCGTCAGCTAATGCGCAATTTTTTGTCAAAGTATTCTGAAACGTCTAATAAAAAAaTATCAGGCAAACGAAAACATTCAGATATTTTAGAAATATTTGAAAATGATGCGAATGAGAAAAGTGCTACTAACGGTATTATCTAATAATGCATAATAATTGCTATTATTTCATTTTATCGCAGAATGTTTGTAAATAATTGAAATAATTATATTTATTTCAATAAATTAAACTCGCATTTATTACTCATATATGCCCTCCTACTTTCTTTACTCGATATTTAATTTATATCTCCATCTTTTAaTACAaTTTTTtAATAAATAAaTGaCTAAGTGT

>contig00274

TTACCCTCTGCTAAATTACAATGATAGTTCTAGATGaGAATATAATAATAAAAAAaCATGGTC

>contig00275

ATAGTAAGGGTCTTAAAATATTTAAAAAaTTTTTTTAAGTATTAAAACTATAATTTTACGCACTATCACAATAATCTAAAAGTTTGGCAACAAGAATTGTATATAAAAATTTTTAAATGAATGTAGTCGTAAAACTGTCAGAGCCATGCGATTATTAATTTAGGTGAAAGGGTGTAAACAAATCGTAATACTTACCGCCATAACCGTTCCATCAGGAGCTAAAACTTCCTTTTTCCAAATTATTTCATCCCTTAAAAAGTGATCTTCACACACTACGTGTCCACGTTTGACAATAAAATTATTTTTTtCACGTGTAATTGCTTTTTGCCAACATTCAACCACAACCTCATTTTTtGGAACGGAGAAAAAaCCTTTATTTACTCCGTTTGAGCGGGTTGACACGTACCCTGATCGACAGGTTGGAGCTACACGTCGATG

>contig00276

ACAGACGAGGcTCTTATGCGCAAAACAATGAAAGGCAAAAGGAAAACTTTtGAAAAGGAAAgCGAAAaGGAAAGCGACGGTGAACAGCCCAGCGACGAAAaTGGCGTCGCGTCCATTTCCAATTCAGATAATGGTTCTGATGACTCTGCTAGTGAGAATAAAAaCaCAAAATCACGCAAgAaGAAGCGCTCATCCCGATCCCGTGGAAAGCAAGAAGATCAGCTTGATGAAACGCCCGAAAACGAGGAGCAGGAGAAGCCGGCCGGAAGTGAAAaTGGGCAGAAAaGTTCGAAACGCCGTAAGAAGGACGAAAAAAaaCCTGCTGCCGCTTCTgAAGAAAATGAGGAGGATGAATACGAGGTCGAGAAAGTAGTGGGAACACGCACCATAAAAGGTCGCCGTCAGTTTTTAGTgaGATGGAAAGGATATGCAGAAGATGCT

>contig00277

GACACTTGGGaGCCAGagAAGAACTTGAATTGTCCCGATTTAATTtCAAAaTTCATGGAAAaGgTGGACTCGGCCAAGAAAaCTGACCGAAGAGAACTCAGGGCAAATCCATCTCTgaCAAAaCCTTACACTCTTGCCGCGCCTGGGAAAaGACATTCTCGACGTAATGTTGATAAGCAGAGgT

>contig00278

CCCAGCGACAAGCGACCAAAGACGCGGGAGCGATCGCCGgAaTAAaCGTCCTCAGAATCATCAACGAACCCaCAGCCGCGGCCCtCGCCTACGGCCTTGACAAGAATCTCAAAGGCGAAAAGAACGTCCTCATATTCGATCTCGGCGGCGGAACCTTCGACGTTTCCATCCTCTCCATCGACGAAGGCTCGCTCTTCGAAGTGAAGTCAACGGCAGGCGACACGCACCTCGGTGGCGAAGATTTCGACTCGCGCCTTGTTGACCATCTCTGCAAGGAGTTCGAACGAAAGTTCAGAAAaGACCTGAaGAAGAACCcGAGGAGTCTTAGGAGATTGAGAACAGCGGCTGAAAGAGCGAAGCGGACCCTTTCCTCGAGCACCGAAGCTACCATCGAAATCGACGCTCTTTATGAGGGAATAGACTTCTACACGAAaGTCTCGAGGGCGCGCTTCGAGGAGTTGTGCGCAGATCTGTTCAGGGCTACCTTGGAGCCTGTGGAGAAGGCCTTGACGGACGCCAAGCTGGACAAGAGAGCCATCGACGAAGTCGTCCTTgTCGGGGgTTCAACGAGGATCCCGAAGGTCcAGAATATGCTGCAGAACTTCTTCTGTGGCAAGCAGTTGAACCTCTCCATCAACCCTGACGAGGCCGTCGCTTATGGCgCGGCAGT

>contig00279

CGCAGCGACAAGCGACCAAAGACGCGGGTGCGATCGCCGGAATAAACGTCCTCAGGATCATCAACGAACCCACAGCCGCGGCCcTCGCCTACGGCCTTGACAAGAATCTCAAAGGCGAAAAGAACGTGCtGATTTTCGATCTCGGCGGCGGAaCCTTCGACGTTtCCATCCTCTCGATCGACGAAGGCTCGCTCTTCGAAGTCAAGTCAACAGCAGGCGACACGCACCTCGGTGGCGAAGATTtCGACTCGCGACTGGTTGACCATCTCTGCAAGGAATTTGAGCGAAAGTTCCGAAAAGACCTGAAGAAGAACCCGAGAAGTCTTAGGAGATTgAGAACAGCGgCGGAAAGAGCGAAGCGAACCCTTTCCTCGAGCACCGAAGCCACCATCGAAATTGACGCTCTTTATGAGGGAATAGACTTCTACACGAAAGTCTCGAGGGCTCGCTTCGAGGAGTTGTGCGCAGATCTGTTCCGAGCCACCTTGGAGCCTGTGGAGAAGGCCTTGACGGACGCCAAGCTGGACAAGAGAGCCaTCGACGacGTCGTCCTCGTCGGGGGTTCAACGAGGATCCCGAAGGTCCAGAaCATGCTGCAGAaCTTCTTCTGCGGCAAGCAGTTGAACCTGTCGATCAACCCTGACGAGGCCGTTGCTTATGGCGCGGCAGT

>contig00280

GtCTtCAGTTCAcTTTTTTtCGTCTtAAAGCTAGATTAAATTACTGGATGTAAAGTATTTTTAATTGGCTTCAAAAATGGCTTTCGCAAAAATATATTTGATGCCGCTGATGGTTTTTATGGTCATTGCTGAAATTTTTGGGGAGATTGGCGAACCTGAATTTGTGTGTAAAGAACACTTAGATTGCCCAGACAGTAAACCTGCTTGCGTTGGCACAACTTGTATGAATCTTTGTAAATTGAAGGATATATGTGGAATTGATGCAATTTGCAAATTGCAAGCAAGAATTACTCCTGTGTGCTATTGTGAAGGTGGAATGATTGGAGATCCTTTTGTGAAATGcATGAAACCTAATATCGTTATCAGAGATTGTCAGTCTCAGTCTGATTGTCCAGGAGATGATTATCAATGCGTAGACAAAAAaTGCATAAATTCATGTGTGTCTTGTGGAGAAAACGCTGTGTGTAAAATTAACCCTAGAGATGGCGTAGCCAGGTGCACTTGCCCAAGTAATATGCCTGGAAATCCACTCATTAGTTGCAAATCACCAGATGTTCCTGCTCAGCAAgCcTAACAATAGAAATCAACAAAAGAAACCTAAACTGATAAGGAGACCTCCTGATTCTGGAAATGTATTAAACTTAAAGTGATCTGTATAATTATGCAGTTTTATCTCTAAAATGAATTATAGAAATGTATTGGCATTCG

>contig00281

CCGGAATCCGAAGAGCGCACAGCTGAGgTTCTCGACCTTGATtCTCAAAAGGTCCAGACTCACAGGTATGAGGAAGAtCTGATCAGAGCCCAGCGTCAGGAGCAGGAAAaTTTGATCAgAGCCCAGCGTCAGCAacACGAAAaTCAGA

>contig00282

GAGCCCAGCGTCTGCAACAGGAAAaTCAAATCAGAGCTCAGCGTCAGGAACATCAGAtGCAGATGCAGCATTATGCTATGCAGCAGCAGCAGAgaGCTGcTCCACATTCGGTGAGcTCGATGTTAAGtAGTTGGCCTTCACCACAAATtcACcACGAGTATCATCCAGGAATTTCTCcgATGGcACCCATGGGCTCTCCTTCACCAATACCCGATCAgAgCCAGTTTATACATGCGCaCcAAAtGCCTCTTgAACCATCCAGACACGGTGGTTCGCCTATTATCACGAG

>contig00283

AAAaGGGCCGATTGTGTATTCAGCTCATGAAAACATTACAATTATAACATTCTCCTTTAATTTACTTCTTGGCCGCAGCCTTCTTTTTGGGAGAAGCTACCTTCGCCTTTTTTGGAACAGCCTTCACAGCCTTAGTTTTAGTTGTGGTTTTTGCCTTAGGTGGTTTCGCAGCAACCTTGGTTGCTGTCTTCTTCTTTTCAGCAACAGCCTTCTTTGTTTTCGGGGCAGCCTTCTTCGCGGAAGCTGGCTTCTTTGCCTTTGATGCAGATTTGGCTTTtGCAGCAACCTTTGcAGATTTTGGCTTGGCCTCagTTTtACCcACAGGTAACTTGAAGGATCCGGCAGCACCTGATCCCTTAGTCTGGACCAAaGCTCCg

>contig00284

ACTTCTTTGGACTGTGACCCTCTGCGTCTGGACAGTGTGGTCTCTTGGCAGCTGGACCAGAAGCTCCATGCTTCCAGCCAACATGTCTGCCGGAGTGTCCTCTGTCACAGTCAGGACCATGGCAGAGGGTCTTTCCtCCCTACTCCT

>contig00285

ACGTTTACTACGTGTCGCTGATTAATCTCCTTCGAGTTATAATTTTCAATAACAAATATTTT

>contig00286

AATAAGTGAAaGAACAATACGATATTTACTTTTTGATTGTTTATGTATACTCCGTCGGTAAAGTTTTTGAAATCAGGCGACTTATtCGATCCTGTTCAGTCATATGTTTCGAACGTGAGTGAAAGATACACAGAGGATTGTTTtCAAACTGTCACTTCCTGATATCGCATTTACTTACAGTTTAAACTTCCCAGTGGAAGGGgATTTTtATTCTTACGAATGTTCTTTATGTCATTTGTGGCAGGTTTTTTGAATTTGGATGTGCGGCAGTGTAGTGACTTTTGAGAAACTATTTTTTGAGCAGTGAAAAATTATATCTGGAGGAGAGGAACATCATGATAAAGAGATTGACCCCGTGGA

>contig00287

ACGTCATTCCCCcAAAaGGAAAGGAACTAGTGAAAACGGATTTGCAAATTGAGGTTCCTGAAGGAaCTTATGGTAGAGTGGCTCCACGTTCCGGACTTGCTTGGAAAAaCCACATAGACATCGGTGCTGGTGTTATTGATCAAGACTACAGGGGTAATGTCGGTGTCGTTATCTTCAATCATGGTGAAAATGAATTTAAAGTTCAACCCGGTGACCGAGtAGCTCAGTTGATTTGCGAAAAGATCGAATACCCCGAACTCCAAGAAATGAATTCGTTGGATTCTACAGAGAGAGGAGAAGATGGTTTCGGATCTACCGGTATGAACGAGATTGCAAAGAAGTAACACTCACCATCAAAGATTTACACTACGCTCTTGTTCGTCAGCAGTATTTTATTTCTTACTAAGATATATTTAACATGATCACAGCGATAAAaTAAGATATCACACTTATTACAAAACGACAAGAACTACAAATGTATCCTGATAT

>contig00288

GAAGTTTACTCTTTCTgAACTcAAACGATATAATAAG

>contig00289

ATACACTACGTAAAGAAGCAGGACCCTTTCTTTTTGTATTAAAAATCATACTTTCTGCATTTTTtATGTATACTACCATGATGAAGTTTCTAGTCCTTGCTTTAATGCCAATTTCTATCGGTTTTTCCGAATCTGGAGAGGTTGACaTATGCAATGGAGAAGATTTAATAAAACgtGAAaTAACGACTTTTTACAATATACAACCAAAGGATTTGGAGGCAAAATGTGAAATTGACCAAGCAACTAAGTGGTCTAAAGAAGAAAAAGCTCTTATGAGTGTCCAAGGACTTACACTTGATATAACAACTAATATAGCGAATAATG

>contig00290

ATTGCATATGTCAACCTCTCCAGATTCGgAAAAAC

>contig00291

tACACAGCTAGATTACCAAaTATTAAGTAAaTGGAATGAACAAAGTAGATTTTtCTTCTTCAAATGCATtGATATAGTTTCTAATTCAATCGTACAAAGTGCGAGTCCATCCTCGTTCTTAAACAAAAAGTGGAGTCAAAAAATTCCTGTCTTGCCCTTCTTTCTGGGAAGTTCAAGACGGATGTTTTTTTtACAGAACTTACACACAAATGAACGATTGAAGTAATTAAGTTAAGTGGTTACAGCGTGCCGTCTGTCTTAGGATTACCTAACACAAGGTTGCATAGACGCTTCATAAAaTTGAAAGCGCTTCTTAATTCTCAGATGTTGAGACGAGTGGTTTGGTCGAATTTGGTACAAGAAAAAaTGCGCTCAAGGCTCTACCTTCAGCAGCTCCACATCGAAGACCAGAACAGCATTGGGCGGAATAACGCCAGGGTGTCCTCGGGATCCGTAAGCGAAATC

>contig00292

CGAACTTTTTATCTCGAAGATG

>contig00293

CCATGCACCTTTCGGCCTCATCTTtGTTGCTGTCC

>contig00294

ATTGTGAACTGGTTAGAAGCCGCGTCGTTTGCTCAGCGTCTATTTCTCAACTTTGCGTTCGATTTACAGAAGAATTTGACTTCGTGCTCCGCTTTCCTGAAAGAGACGGGTCTCCAGTAAACTTTTAACATTTTTtAATTCACACGCGCACCCTAAATTCGGTAAAATAACCATGGCTGATAGATCaAGCAACGGCGAGTTCAACAAATATCAGCGGGATGATGCTGACGACAAAACGAGGGTTGTCGTGGACAAGGACGAAAaGGATTCCCAAGTAGATAAAGCAGGGGAGTAC

>contig00295

ACATTAGATCATTTATAGCACAGCCAGCATTTAGCCGTAATCGATTCCCGAGAATATAAATTCTAACAATAAAATAAGGTTTTtAAGTGCTTTtAACAACTTAACGTATTTTAGGAACTTTGAAACCTTGAGATAATTtAATAATTTGAGAtACATGATACGATACTTAaTATTACATCTTAACTTTAATTCAAATAAATTATTATTTTAATCATTTCCGTTTAGATATGGATATTTTGTATAAGCCAaGTCCTAAAAGACTTGTTTATGACTCGATTTTTGTGATGGCACTTTAACGAATTATTTCCAATCAGGTTAGAGAAATGAAATTTtGACCTGATGAAAAAATTTTCTAATACATAAAAGaGTGACAGCTTTAGCAAATGCTTCATATGACTAAGATCAGAGAATTTCCAATTATTGCATTATTGTTTAAAGAATATGACGCGGATACGATTCACGCATTACGAATTGTAAATATTATTTAAGAAAGGGGT

>contig00296

GTCCGAGAATTGTTGCAGGAGAAATTGGAAATTGACACGCAGAAGTGGCCGAATGCTCTTCGTCTACTTGACCAAGAGATTCAGAAAACTCAGGCCATAGGAAAGCCTCTGAAGGACCATAAATATGTGGACATCTATCGTGAGAAGCCCATAAGAGTTGCTGTCAAGGTTTtGGTGCCGGTTCGT

>contig00297

ACTtAACTGGGCCACCCAgTTGACGACAAAAAATCTTTTGCGAGGATTTTTCTCCTACTACGCCGAATTCGACTACAGATATAATGTAGCCTGCCCTCTTCTGGGAGTTGTAAAAaCGAAGAAGGAATTTTTATATCTTCAACTCTTGCCTGACGAAATGAAAAGCTACGTGGAACACGTAACGAATGAGCCCGATCCAGAATTATTCCGTATTGATTCTGCAATGTGTATACAAGATCCTTACGATCTCTCACACAATCTTACCAAAGGTGTCAAGAAAGTGGTTGTAAATCGCTTTAGAAAATTGTGCGGAAAAAGCGCACAACTTCTGGCCGGCGATCACTAGGATTTTAAATCAC

>contig00298

ACATGGGgACATCTCGTAAACTCTGTGTTGCCGTTGGTCTAGAACTGTGTGTGCTTCGAATCTATTCGGAAGTTCAGTTTAGAAATTCATTAAATCGAATAGTTTATATTAAAAATCATTTAGAGGATGTTGAATATACATGAAATTCAAAAGAAATAAAAAAaTTTACCGCGTATTGCACTAATAAAAGCAGAAATTACGGGGACTATCGAAGCCTCATATCAATAAAATGAGGTCTGTGTTATTTGTTCTCTGTTTCATCGTAGTGGGATTCATTAACCCTAGTGG

>contig00299

ATCAACTCGTGGCCGAGGTTCGTTAGGCCTTTCAGAGGACGAAAATAAAGCATATCATACACCTATCGTACCACAGAAGGGTGATTTGATCGAAGATATGAATGGAGTTCTTTACGCAGTATGCTACACCAAAGATTTGTATATCAATATCACACCACAAGGAAAATTCGTAATGGTACACGATTATCTGACGAGAACTGATATTAAGCCTGGTTCTATGTTGATCCAGAAAAaCGAAGAGACTGCATCATATGGTTCAAACAGTGGGTGGTCTTACACAATTAAGGATAATCGGGgAAAAAaGCGTAAATACAATTTCAAATTCGATTTCGCCGGAAAAAaTCTTCCTGTTTAGAAGTAGTCTAAATACTAAAaTGTATGACACTTAAATTCAGAATAAAAAAaTTCGCTTCAAACCcT

>contig00300

ATCTCAAGATGGAGGTAGCATTAGACTCGGCAtCGTAATTAAaCATCATTTtAAtCGCCAGGGgAAAGGTGGAAACTTtGCTTTtGTCGTCGAtgACGAGTTGATGATCGTTGTCCTGATTCGGGGTCAGAGTTTtCAGATGAGCCGCGTGAACGACAGCTGAGCAtCCTTtCGCGATTACTGGCCCGATCACGAAATCTTTCaGATtGGTTGTGGTTGATTCGTCCTCGCCCAATGCCGTTTCGTAATTTTtGTCGTTATGGGgTTtGTTCCaCTGTAATTTCGACACTGCTTCCCTGATTTCCCAGCAGACGCCTTCCAATTCATCTtCTTTGGTCAGGATCCCGGTTCCAGAAGCTAGAGAAACACCGACGAGAGCAAAAAACGGTCTCGAATCTCCGAAAaGTAAACGACTtGctGCTCGACGTCGTAAATCTGCTGCTAGAGTGTTGGTGACTCTTTTCAAGATATtGTCGACGAATAATCGTCGAGCCTGGGCTCCCAGAAAGCCAAGGTGCCTACCAGCAGCTGAaGAAaTACCGGT

>contig00301

CCAAAAAGAAGACCTCAGTGAAAGGAATTCgaTCCATTGTCTCTTtCACCGGCAGGAAGGACACGGCAACTTCTTAATCGAAAaTATTCTCGCCTTCTCAGAGGCCAAACATTGAAaGgAACGCTCGCAATTCCCTTCCTCTTTCGCAACGTTCGATGTTCGAAAAGGAATAACAGAAACGTTGGCAGTGAATAGTTCGATCCACTTGTCTCTTGAGGAGAATTCATcgTTCACACTTCGCTTTTtCAtGCTGCCAAAaCTTaCACGATCGGATGACAGGGCCGATTTTTCTAAGACAGAATAATTAGCGCGATAGTTTTGTTTtCTACGCGTAGTTCGTCTACTTGGAACTCGCAGCCAAAGCTGCAGGAA

>contig00302

GTGCAGGAACCATGGCTCGTAAATTTTTCGTTGGCGGAAATTGGAAAATGAATGGGAATAAAAATGAAATTAATGATATCGTTAATTTTTTGAAGGCGGGTCCCCTTGATGCAAATGTTGAGGTGGTCGTTGGAGTTCCGTCTATTTATTTAACTTACACAAAGAGTATACTTCCAAACAACATAAGTATTAGTGCTCAAAATTCATTCAAAGTGGAAAAaGGAGCATTTACTGGTGAAATCAGTCCTGCTATGCTTGTGGATAATGGAATCCCTTGGGTTATTCTTGGACACTCGGAAAGAAGGAACGTTTTTGGTGAAACGGACACACTGATTGCCGAAAAAGTGGCGCATGCATTGAATGCTGGTGTAAAGGTCATTGCTTGCATTGGAGAGAAACTGGAaGAAAGAGAAGCTGGAAAGACGGAAGAAGTTGTATTTCTACAAATCAAGGCTATTGCTGATAAAATAAAATCTTGGGACAACCTTGTCGTAGCAtATGAGCCAGTTTGGGCAATTGGAACAGGGAAAACAGCAACACCACAGCAAGCCCAGGAAGTCCaCAAAAAaTTGaGGCAAtGGTTTTtCGAAAAtGTTAGTCCTACAGTGTCTGAATCTTTAAGAATTATATATGGAGGATCTGTAACTGCAGACAACGCTAAAGATTTAGCCAAAGAAAGTGATATAGATGGCTTTTTAGTAGGTGGAGCGTCATTGAAGCCAGGGTTTGTCACAATTATTAATGCTAAGCAaTAaTAAaTTAaCTTAtATA

>contig00303

tGgATACGCTACTTTGGTTGAAGGTTGAAAGGTCATACGGAATAAGTTGTTGGTCAGAGCTAATTTTGGTATGATTATTTAAGTTCTCTAAAAAAGTATTTTTTGTTTTAAAACACGTTTATCACCAATTAA

>contig00304

TCAGCACCtGCCTGAAgAGCTTCCATGAAAGCCTTCGTGCCTGATTTTGCGATCGTTCCGAGGTTGTTGACAAGGTCGGATTTCGTCATACCGATGCCAGAGTCAATGATGGTGAGCGTCCTGTCaTTTTTGTCGGGGATGATCTTAaTGGAAAGTTCCTTGCAGGAGTCAAGCTTCGATGGGTCTGTCAGCGATTCATATCGGATTTtGTCCAGAGCATCTGAAGAGTTGGAGATCAATTCTCGGATGAaGATTTCTTtGTTGGAGTAGAATGTGTTGATGATCAAACTCATCAACTGTGCAATTTCAGCTTGAAaGGCGTAAGTTTCAACATCACcGCCCTCAGCCAtGGTTTGGTCTTCCGGCATTtaGATCTTTAAAAAAaGgT

>contig00305

CGGATATTAGTATCTTAAAAaCTCAGATCTTGTTAATATTTCGATTCTTTAAAGTTGAAAGTcTTGTGTGTGTGtGGGTGTGGT

>contig00306

AAGAtGCtATtAtCtAGACtGgAAGCCGAAGgATGCTAAATCAGCAGATGTCCAATGCCGCTTTATAGACGTAGCAACGAATAAATCAGAAACAaGCGATACTAGTGTGAAAaTAGTTGGAACTGAAGCAAAaCTAaTAGAAACATCTAAAGGTGAACAACCTTtAGAACATTGGTTtGTATATACCGACTACATAAATGTGGCC

>contig00307

CCACCTGACAGCAAGTTTtCCCATTAAAGAAaTTTTtCACAATCGATACTTGCAGCCTTCTGCTAATGAAATATAACGAAGTTTTTCAATTTGCTA

>contig00308

ACGTATTTGAGAAATATTACTTGCCCTATAATATGCAAGCAAGATGCTATTATCTAGACTGGAAGCCGAAGGATGCTAAATCAGCAGATGTTCAATGCCGCTTTATAGAGAACCAATAAATCAGAAACAAGCGATACTAGTGCGAAAATAGTTGGAACTGAAGCAAAACTAATAGAAACATCTAAaGGTGAACATCCTTTAGAACATTGGTTTGTATATACCGACTACATAAATGTGGCCATCAGATTCACTTGCAAGGATAAAGAAACtGgCGGAGTGAATAATGTTCtcGTTTtGGTAAGATCGAGAAGTCCAGGACCAAACGTTCTAGGGgTGATTTCGAAaTTTCTTATAGATAAACACCTAGTAGGAATGCCAATTTTtACAGAAGATTGGACAAGTTGT

>contig00309

ACATAGCGGGCGTGTTGAAGGTCTCGAACATGATCTGGGTCATCTTTTCTCGGTTGGCCTTTGGGTTCAAGGGTGCTTCGGTGAGAAG

>contig00310

GTGGCCTTCCCACAATTGAGGGGAAGACGGCACGGGGtGC

>contig00311

AAATGCCAAAACCTTCAAACATTTGGCATATCTTTAGATGAAAACCAAGCACctGTAATATTGGAATTGTTacaTTTACATGCTGCATCTTTGAAcAaTTTGAAaCTACACCTAGGTTTAGgaCGGAGCAGGCAATTgCAACCTaTATTATTgtCAACATTGGCCACGTTGACGCACCTTGAAAAATTAGACTTTAGTGGATCCTTCTCAGTTTGTGATGAATTTCTTGAAGGAGTCGCCAAAATGTGTAAAAACcTGACCTCTTTAAACTTGACAAATTGTAATGATGTAACGGATCGTGGCATTTCAGCGATATGTAATTTGAGTAAACTGACTTATTTAAATATAAGAGGATTGCCAAAaTTAAGGAACCCACCACTGGAAAAaTTGACAAGTTTGGTAGTATTAGAGTCGTGGGGGGCTTATGATTTACAAGAGGAATGTCTATCCACTTTTTTTAaGAAAGCACCTCAACTCCGGGCATTTGATGTAAGAGATTGTCTTTCAGTTAGTAAGAAACTATTGAATCTTATTATTGAGGTTGCTCAAATTCGAAATCCGAACAGCGCGCTTGATTTATACGTCAATCATGAAAATGAAATAGATATGAGCGTtcTTGGTGAAAAACcACCCTTCTTGAAtATAATTATTCATCATtATTTtAGTGTT

>contig00312

ACTTTCAATtACTTTtCATtCtAGTTAAAGTTTTACCACATTTAAaTTGGATATTCAatGCAATtAAAAAAAaTAaTTCAAGTTTCTTTtcGGtCtACTTTAATtAaCATAGAAGGATTTCAAATGGATCCACAATaCCATATTTGgCAAGTGG

>contig00313

ATTCTTCGTTTGATTTTGTCGAAAGTAGAACCTGCTGCTGAAAAATGACTGGTCGCGGAAAGGGAGGTAAAGGTTTGGGAAAaGGAGGAGCCAAGCGTCATCGTAAAGTTCTTCGTGATAACATCCAGGGAATCACGAAGCCTGCGATCCGTCGTCTTGCTCGTCGTGGAGGAGTAAAGCGTATTTCTGGCTTGATCTATGAAGAAACCCGTGGTGTCCTTAAGGTGTTCCTCGAAAaTGTCATCCGTGACGCTGTCACCTACACCGAGCACGCCAAAAGGAAGACTGTCACAGCCATGGACGTCGTGT

>contig00314

TTTCCAAATGCTGAAAAACTTCGATTAGACAGTCGTCAGTTAATTTTTCGATTTCTGTAGTCTCAATTCCTCGTCTTTCTCCAATTTCTGTTTCCTTTTtAATTTTTTtAACATTTCTTATTGTTTCAACTTCCGCCATTTAATTTTCTGTATAGCTTTTTGTAAAATTAGCAAGATGACCGCGAGAAGTAAACCAAAAGGAACCGTAAAAGTAAAATTTAACGCACAATAAAtCTTGCAGACCTTAGGAAGATTGTGTAACGAACTTTtCTTACTCAGGAACAAGTGAGGACT

>contig00315

ACAAATACATGTTAGGTATTTtCCAGAGAGTCTTATAATCTTATCCAAATCAATGTGTCGTATCAGTAATAATTCGCCGGAAGGGTTTCCTTTCATTCCCCAAGTTTTGGCTGAGAAATCCAGAAATTTAAAATTGTTCCACGATCTTTGACTCACATTTTTCCACCTTTTACAAACTCTTTCTATACGGATTTTTTCAGCTATTG

>contig00316

GTCCTCATTTtcTCCTGAGTAAGAAACGTTCGATACACAATCTTCCTAAGGTCTGCAAGTTTTCTTGTGCGTTAAATTTtACTTTtACCTTTCCTTTCTGTTTACTTCTCGCGGTAaTCTTGcTcATTTtACAAAAAGCcATACAGAAAATTAAaTGGCGGAAGTTGAAACAATtaGAAATGTTAAAAAATTAGAAAGGAAACAGAAATTGGAGAAAGACcaGGAATTGAGACTACAGtaaTCGAAAAATTAACTGACGACTGTCTAATCGAAATTTTTCAGcATTTGGAa

>contig00317

GAGTTTCTGTTCAAAaGCAGCAAGGgAAAGAAGAATATTCCCAAATCTAAAAGGTGAAAGAATGATTCATGTGGGATTGCTCGTTCTACTCTGCGCAGGAGCTTTCGGAGCTACTACTAACTCTTCTCAAGCTTGTCCAAATGTAACAGCAATGAAGGATTTTGACGTGAAACAATTTCAGGGACTCTGGT

>contig00318

ACCTAGTACGAATATATGATTATCCTACTAATTCCTTACAACAATGCGTAAGCCAAAGCTGGACGCAGTTCAAGGATGGTTCAATAGGATTACGAGTCAACCTAACAGACGTGGCACAAAACAAAaGATTAAGCTTTTTTGGCTTTGCGACATTtGTTGGAAACGAAGGAAGATATGATGCAGTATATGAAGGTGTACCAATTGACCAACAATTGATTTTATCTACAGACTATCATACCCATTCTATCCATTTCACCTGCGCTGATACATCAGCTCAAGGAAAAAaTAATGTTTGGATTTGGGCAAGAAAGAAACATCTACTAGAATCGGCTTATAAGAAGGCTTCTCAAGTTCTTAAAGATAACCAGCTGAGTGTAGATTTACTAAAAAAaGAAGATTGGAAAAATTGTCCACCTGCGAACGTAATCGACTAATAACTTTTTCATGAAAATGTCTCTGTTATGAAATTTTCATTAACAAAAAaTGCATAAAAaTTATCCGTTTGAGCTTTTTAACTCGAAAATGAAaTTAAAATTCAATATAGTAaTTTTGAAAATGACTACTAGAAtGTTTTTACAAATAAAaCGTAaGGAaGTTTTTCAATTTTCTCCcAaCCCATAagAGTAATTACATGTATTTTTCTCACTGAAAAaT

>contig00319

GATTCTAGATGTTCTTTATGCAACCGAGGATGAAGGAAATGGCCAGGAGTGCGAAGAACAGACCTTTTAGATTCTACTCAATTTTCCTTTTTCTTTTTGTGTAAAAGATCGTTTAAAAATGAAGG

>contig00320

TCCAATTCGAATtAACTGACATCTTAAAGTTGCCATTCAtGTGTCTGATATTtCGAAACCTTGTTCTTtAAAtGTTTTtGGATTGTAGGCACTAGAAAAACTATTTGTtCTTCACGAGAACAAGATGCGAAACAATCTAAACTTGTGAGGGAAAAAAaCC

>contig00321

ctctAtAtCCTTtAAGACCTCCATTGCGATAGTGAATCTCCACGGGAGTGCACACGCTCTTtGTAGCGCGCATATGTATCACTTCAAACACCGTTCGAAGTAAAATTGTCCTACGtATCCACCTCTCATGGCcTTtGCGACATTTtGTTCAAACaCTCTACGATTATTCTGCAaCACTTCGGCTGCATCTTTGTTTAGTGGATCTTCTGGATTTGGCTCCAGAAATAGATACTGTAATCCATATACGATCGTGTTGATCGTTAGGACCGGCTTCCAGTCTTCTCTTAAGATATTCAGGCACACATTCCCCTCGAGATCAATGTTCGGATGATAAACTTGGGTTTCGCATTTGACTTTAGGAGGTTCGTGTGGATAATTCGGTCCAACTTTAAAaCTAAATACGAATCTGCCGCCTCGATAAAAACCCTCgTCGGGGCAAATAGTTATTTtAAAaCTTAGTAGATCATCTGGATCTGGATGATCCAACTGACACGTTTTCGGCAAATTAAG

>contig00322

CATACACGTTCTTTTATTTCGgaCGTGCTAACGAACGACACAAAAATATACGAAAGTCGAGACTTTTGACAGCAAATTAACTAAACCCGTCTCTC

>contig00323

TAGTCGAACACTGTGAGAGTCATGTAaTTTTAGAAAATtATATGCATTTTTATCGATTCCACAAAAAaTTGTAGAAAaTACTTTAATATTTGAAGTTTTTATTTGAAATATTAACAaCAacAAAACCaGTGGCGCCATCGCTTaGAAAAAAaTAAAAaGTTCGTGGGAACTCGAGAAACCAGTAGAGTTCTTAAATTCTCGAGAATTTATGTTtGGGTTATATAACCGAGAATATAACAGAATTT

>contig00324

CctcAACCCCtCgaCATTGCATTTTTCAtCCtGAAaTTTGCCGTTCCATCCAAGTGCCAAGTTCCCCGTTtCCCTGTGTTAGTCTCGCAAGGGacaGAAATCGTGAGCTCGAGAAATGGCCTGCGCCACCCTAAAAAGATCTCTGGAATTCGAtCCGGTtCACAGCCATGGACGTCCAAGCAAGCGAAGGaGATGTGCCCCCATGTGCGTTTCGCCCGGAAGTTCTTCGCCGAGCGTCGCAAAGAGCCAAAaTCCCTCGCCATTTGGTGAGGTTTCTCGGAAATTGACGCCTGAAAAAATGGCAGCGAACATTAGAGAGGAAATCCGTCGAATGCACCGGCGCAAACAACTGCACTTCAATCCGCAAGGAAACAATGGCGACTCCTCAGATATGGAAGGTTCATCAAATCCTTCCAGTCCATCGGCTTGTGGTTCAAATACCTACAGTAGCACTACATCTAACAAAGAGAAACCTCTTTtCACATTCAGACAGGTGGGTCTGATCTGCGAGCGGATGCTGAAGGAGCAAGAAATACAAATTCGAGAAGAATACGACCAGATCTTGACCATGAAGCTTTCCGAGCAATACGAATCTTTCGTCAAATTCACCTACGATCAAATTCAAAAAAGATTCGAATCCGCAGCTGCTCCAAGTTATCTATCATAAGTAGGATTCTTACTCGTAAGAATACGTTTCTGTGAGAAAATATTAGAT

>contig00325

aCCTTTTCATTTGGTAAATTaTTtCATACTGAATTTCAGAAGTTATTTATGGATTAGAGAAGTTTCtAGTTTTTTAATTTtGTTTCTTATTTGACCACTGAAATGTTTTTTtGGAGATATCTTTtATTTtCGACTTTATAGTTTTtGGATAGTATTCTAGGATATCCTGAGCCAAGCTAAATGCTATTATTaGGTAAAAatATTTCTTTtCTAtAGTTAATTTTTTtGTGTCAATATCGAAAAACTTGTTATTAAAGCAAATGTAGCTTTtACTTTTTTtAGTTTACAGTTGATCCGTTTTAAGTTCAAACATCAAGTAATATGTGATtATTTtCATTCGCGTGTTTTTGACATAAAAAaTATTTTCTGTAGCAATGTCTTAAATTGGCGCTTTAAATCTTGACGAAATAAAATT

>contig00326

TTCTCTAAATACGCCGTCTCCTCACGgAAaCTTGGCTACAAAGAACGTGAATATTGACCGAACAAAGCAGCATAGTTTtACGAAAGAGACGCGAAACCACCACTCAAGTCAAGAGACGAATTtCTGCAATTTGATAGAAGTCGATAGTTCGCCGGAAAGTTCGAGAAGTAAAGAAAGCACACCGAAGTCAATGAATAAACcAAATTTtGAtGTAAAATCGAGCACTTCgAaGAGACACTCAAACACTCCGAAAAATCAACGAAAGAAAAGT

>contig00327

ACAGATTTTGtCCGGGTGGGAAACAAAaTGATGCGATCTCGTCAATATCCATGGGGCACAgTTCAAGTGGAAAATGAGTCGCACTGCGATTTTGTGAAACTCCGCGAAATGCTTATCAGGACCAACATGGAAGATATGCGGGAGAAAACACaTTGTCGTCACTACGAACTGTATCGTAAAAAaGaCTCGaGCAGATGGGATTCAGTGATGTAGACAGCGATAATAAaCCTGTCAGTTTCCAACAAACTTGTGAAGCAAAaCGCTCGACCCACTTGCAAGAACTTCAACAGAAGGAAGATG

>contig00328

GCAGACACTCCAGTTCGCACCaTCGGCAGATACACCCGGGAATTCATCCTGGCAACTCCTCTCCGCGATTTCATGACAGCACTTCAGAACGAAAaCACCATCGCAGAGACTCCCCAGAAGGAGGAGAGTCCCGAGGTGAAACAGATCaCGCCTTTAGGATCTGTGAACAATAAGAATGTGAGGACTCTCGACACGAAATCTTTTtACGGgAGTGGAAAGCAGCAGAGACTCAGTGCCTTCGGCGTCGACTGGAAGCAGTTTGAGCAGACTAAAAAGAGACGCAGTCTTCCTGCTCATCATGCAAAGTCTACTGTCCGACAGTTTCAAAaGAGACACAAAAAGGGGGAAaTCAaCTGCGGCGTCTCtCACGGGCTGAAG

>contig00329

GgTATGCACAATATGATGAGTGGAGGACAGGACAACTTTTCGAATGTGTTGACAGTGAATACTCAGAACCCTCcGTATTGCATGTCTCCAATGATGCACTCACCAAGTCCCcTGAGTCTCTCTCCGCATTGGGGTCTTCCATCTATGTTCTCTCCACTTCCGGTTCATC

>contig00330

CACCTGCTGACAACGC

>contig00331

TCTTCTCCAGTAAATCCCGCGGACTCGACTTCCTCTAATCTAGATCTGTCAAGCTTGCTATCAGATCTGACTGTTAGCGATCGCCGAGCACCGGGATGCGAGAAGAAATCTTTGGAAATGGTGCAACAGAATCTCACGCCATTTGACTATGAGCAGAAGAAAATGCTTGCGGTGAAAGCAATGCAAAGTAAACCAAGTCCTACAGACTaCCGaGT

>contig00332

ACtACTacTTTCTTtCGAAACCTCTGGAGTCGTCTGTTTCTCCTGGGGTTGTTGCTGTTGGTTGGTTGAGGCCTGTTTCTCCTTTTTCGACTCATGATTGTCCTTCTCCTTAATATCTTTTGTGATTTCTTTCGgAGCAGATTCTTTGTTACTATCTCTCTGAGGTGCTGCTGCTACTGCTTCCTTAACTTCCGGCTTAGAATCAACCTtCGTGGGTGGTATCGCATTCACGAAGGCATCCaTaTCCGTGCCTtCCTTCTCAGCtCcTCTGCGATTGATAtCGCGGCCTTtGAGTTTGCTCTTGCCTTTCTGTTTCTGCATCGCAGATTTAC

>contig00333

TTCTTCTTATGTCCCATCTTGAAGGACTTTGTCAAaTAAgCACAGTAAACCACAAATATGAAAAGTT

>contig00334

GGAAGTGAAAGTCCTGTATTTGGTCATGAAGCGGGAGCTGTAGGTGGAAGATTAGCAGCCCTTGAACTTCCGAATGAAATTtCTGCTCCCTATGAAGTTCCCCAGTTTCCCATCGAGCAAATCGAGAAGAAACTTCTCATTCAACGACAaCTTACAGTCAAAGCTGCAAAGGATCTGGaGgAGAGAAGAAGTCATTATGAACCATCTCTTGGTCCAGGAGTTCCGGATGATATCGAGCATCCCTTCAAACTTGAAGAAAATGACTTTGTGCCACATTTCCAACGAGTATCCATCTCCGGGGAGGATACTTCCGGGGT

>contig00335

TTTtAAAAATCAAACGATATAATAGGCCGGACATCAACATTcTAACATACAATACAGAGGAAGCAGGAATATTTCTTTTTGTaTTAATAATCATACTGTCTGCATTTTTTGGTATATTATTATTCGTATACGACCATGATGAAGTTCCAAGTCCTTGCTTTAATCGCAATTTCTATGGGTTTTCCGAATCTGGAGATGTTGAAATATG

>contig00336

CCCAAGAGAAGATTTCATAAAATTTGTATTAAAGGCTTATTACAATATACAAGCAAAGGATTTGGAGGCTAAATGTGGAATTGACCAAAAaGATAGGTGGTCTAAACTAGGAAAAGCTCTTCTAAGTGTTCAAGAATATACAGCTGTTGTCATAAaTAATATAGCGAATGAAGCAAAGAAGAAGAAAAAAGTTACTGATGAGGACCTCGACAATCTCGTTAAAGTTCAATGTTTGAATAACgCTAATTTCAAtAAAG

>contig00337

CCCAAGAGAAGATTTCATAAAATTTGTATTAAAGGCTTATTACAATATACAAGCAAAGGATTTGGACGCTAAATGTGGAATTGACCgAAaTTATAGGTGGTCTAAACTAGGAAAAGCTCTTCTAAGTGTTCAAGAATATACAGCTGTTGTCATAAcTAATATAGCGAATGAAGCAAAGAAGAAGAAAAAGTTACTGATGAGGACCTCGACAATCTCGTTAAAGTTCAATGTTTGAATAACaCTAATTTCAATAAGgTGAAGCTAAATGTTTTTAAAACAGCGTTACCATGCATGGAGGGTgATagTTTgtCGAAaTtCcaCGTAAACGCTGTGTTTttATCTTGCCAGACTGTTAaaTATATCGCCAAAAATCGAAATGTATAATATGTTCAAAAGAGT

>contig00338

AGTTTATTGTCAACGATGATACAAGAATGCAAAAACCTTCAAACGTTTGGCATAGCTTTAGATGAAAaTAAAGCACCTAGAATATTTGAatCTTTACATTTATATGCTGAATCTCTGAAAAATTTGAAACTAAAaCTACGTTTCAAATGGAGCAtgCAATTGCAATCTCTATTATTGTgAACACTATaAAAGTTGACGCACCTTGAAAAaTTAGATTtAAGCCAATCCTgCTCAATTATTAGGGATGAtGTTTTtGAAGGATTCGCagAAATTTGCAAAAAACTGACGTATTTAGACTTGtCTTATTGTTTTGATATAACGGATCGTGGAATTTCTGcAATaTGTAATTTgAGT

>contig00339

TACAATtCATGGCcagTGATTCTcgCTtGGTtAATATCTCACAGTgAAAaTATTACAAATTtAgAAaTAAtAacTGGATCataGgCACTACAAAGAAGacGATTTTGCCAGCTGTTTGGGgCAAATGAGACAACTGGAGtCCTTTTTTTtaTACCATTGTGATTATCAGGAATGGAAaGGTAATTTtCTACTTAAaTTGCCATTGGAGACAATTCAGGAGATTATTTTGACGTGTGATGGTAGAATGCCTTATGATACT

>contig00340

ttttctcTCCGATTTTCTTCACAAGTTGTAAATAGAAGTTGTAGTAAGTATTGCACAGAGACTTGTtATTTTAATTTTtCTTCTCTCTGAGGCTGTGCGTTAAAGATACTTTATTTTT

>contig00341

AATAGATTTATTATTATCgTCACTACGTTCTAACTTGTCACAAATTTACCTATCTTACTATCTATTGATAGAATCTATCTATTGATAGAATCAATCTATTGATTGGTTTTTTTTtCtCGAAATTATTAATTTATGAGTGAAGATCAAGTCAAAGTGAGTGAGTGAGTGAGTGAGTTAGAACATGAAGAATCGCATTTtCCTATTGCTAAATGAGATTTATATTATATATAGATATGTATATTGATTGAAGCAAGTTTAGCATCATTGTTAAAAAAAaaTCTCAATTTGCTGATGATATGATGGTGTGCCTTCGGCTGGAGAGTG

>contig00342

aTAAACACAACCTCCTTTGATTCCCCAAGTTTTAGCTGAGAAATCC

>contig00343

TACGAATTTTGTCAGCTATCGTTGGTAGATGCCCAAAAATTTCGATAAGACAaTCaTCGGgTAAcTTTtCAGCCACcGAAGTCTTCATTtCTTTATCTTCTgCTtCCTTTTtCAGATTTtGATCATCCATCATtCTtGAAAGTCTCTC

>contig00344

CcaTCAAGAAATACGTTGCTGCAACTTATAAACTTGATGCTGAC

>contig00345

ACTTgAAGAGTGCCGTCGCCAGCGGTAAATTGGTGCAGCCCAAGGG

>contig00346

CCAGGTGCTGAGAGACTTTTGCGTCACTTGGTTGAAAaCAACGTTCCAATTGCACTGGCTACAAGTTCAAGCAAGGACAGTTTtGAACTAAAAaCAAAGAAATTGATGGATGTTTTCAATTTGTTTCATCACAGAGTGCTTGGCGGTTCTGATCCTGATGTAAAAAaGGGAAAaCCTAGTCCCGATAtATTTTtGGTAGCAGCTAGTCGGTTTCCTGATAATCCAGATCCTTTGAAGTGTCTTGTCTTCGAAGATGCTCCGAATGGAGTGGATGCGGCTCTTGCTGCAGGAATGCAAGTGGTAATGGTGCCAGATCCAAATCTCCCTAAAAGTTTCACGCAAAAAGCAACCACAGT

>contig00347

TACGATAAGACGAAATCCTTCTTCGAtAATATTAGTTGCGAGGCTGTTGAGAGGAGTAAAGGACGTTCGCAGCGCACGGATTGGCGAACGGAGCGAAAAATGAATGCGGAGACATTCGGCGTTTCGTCGATGCGACGAGGTGGTTTCCGCGGACGCGGTTATTACAACAACCACCGCGGCATGGGAGGAGGAATGTATCGCGGCGCTGGTGGTCCAGGAAGTGGAGGACCAGGAATGGGCTACCGTGGCGGATACCGAGGCAACAATCGAGGCTCGAATCG

>contig00348

CCGAATAATCAGAACCGAGCCAGCAACGAACAACCGAGCGCAGCAGCTGCTCAGCCACAACAGAACAATCGACTCGTCACCGGCACAGCGTAAAAgATaGTCAGTGTCTCGGTAAACAA

>contig00349

GGATATCGAAGCAAAAGTTAGCAGACCATTCGCAACTAGAGAAAATCCAGCATAAAGAAGAATGCACATCATCAAAACCTCGGCATGCTGATGACTTTGCGTTTtCTTTACTATAAAAGTCCAATTTAATCCAAATAAGGTCCAAGATTCGACAGTTGCTGAAAATTGGTCAAGTTCTTCTGTCGATAAAAATGCAGTCGCTAATATATTTATCATTAAAATTGACACAGTAAGTCCAAACGTGCCAATAAGTCTAGTTGCTAATGAAAAACTTATGCACAATTTAGGCATtATAAAATCAGTCACTAAAagTTAAGAAGCGTAATCGAATGTGTATATTCCTgTCCGAAGAGAATTTCCACTTAAACCAAATTACTCTCTATCTTAAT

>contig00350

ACTGATTTCAGCATTCGTGGCAAGAGATCTCCCTGGTATATGCCGATTtCTAGTATCTATTTaGTTCTGACTGCCTATTTCCTGATAATCGtCTGTGCTGCCAAAAAaGAaTGGGCCAATtCTCCAAGAGAAAACGAAAaTATTTATATTTCCACAATCACAGGAACTTACGAATTATCACAAGGATTAAACATGCCAAAaTCTCCATCATATTTATATGTTCAGAAGTCATTTATGGAATCGCCAAAACCCaTGGTTGAAtCTAAATACaCACAAGTTTGACAAATcGCGAAAAGTTAATAATTAATGACTCAATGTAGGACTG

>contig00351

ACtATtGGGgATataaaaGCAGCAAGTGAAAGAAGAATATAACCAAATCTAAAAGaaaattgaatttgtgtatacatattctgattctcttcaattctattGgTGAAAAAATGATTCAAGTGGGATTGCTCGTTCTACTCTGCGCTAGAGCTTTCGCAGATACTAAGTCGTCTGAACATTGCCCAAATGTGAAACCAATGCCA

>contig00352

AATTTTGATGTGAAAAAATTTCAGGGAGATTGGTTCGGAATAGCGAAATATAATTTGTCTAGTAATTTGTTACAAAAATGCATCAAAGAAACCTGGATGCTAAACAAGACTGGTTCAGTAAATGTTGAACTCAGCGAGATAGACGTGGTATTAAAAACACCACTAACATTGAATGGCGTTGCGACATTTGTAGGAAATGTAGGAAAATATACAGCAGAATATAGAGGTGTAAAATATCCAGAACAGTGGATTGTATATACAGACTATCATACGTTTGCAATCAGATTCACTTGCAATTATTTTATATCAGGGGACGGAGTAAATATTGTTATTGTTTTGGCAAGAATGAGAACGCCAGGACCAGATGTTCTCGGGCTGGCTTTGAAATTTCTTATAGATCACAAGCTAATCGAAGAGCCACTGGATATAGAAGATTGGGCAATTTGTCCACCTGaGAAAAaGACCTCCTCaTAAAAGTAaTTTTTtATAATCGGT

>contig00353

TGTATGCATGTGTAATAACATCGTGATCTAGGCTGGTGCTGGCGAAGCAGTAGCCATCTGTCACGCATGTGTGGTTGCTCTC

>contig00354

TTAAgTAATGTAGCAATGGGGAATCCGCCCgCTGGCTGCACTAAGCTCCAACACAATGCAGACTGTGATCAAGAGAAGGATCAAAACGGAAATGGTCGTCAAGATCGTTCAGGCAATTGTTGCCCTCAATTTATGTGTTATAAAACCGAACGCCACTTTTCGAAAGGAATGTGTAAATATACATTTGAACTTCCCGATTAACTGCTAAATATGCAATACGAAGACATACTAATTATTATAAAATGATTGACGAAAGTACCAATAAAaTTATACTATATTATACTACtATTCaTCAATGATAAAAAaCGTACTAAATGAAaCCTAAAAATgTT

>contig00355

CCCTGCCGGTAGAGTGGCGGGGCAGAAAAATCATACGTTCTTAAGACGCTGATTTACATTACCAATTCATTCGACGCAGATCTGTACGTATAGTTGGGACATCAAATTATCAGAATACAAAAAACGAACCATAAATTAATTTGTCGACTTTGATATCACCTGCAATCTACATTTCACATAACGGAAATGCTGTCCTCAAAATGTATGaTgACCATACTTGTGGTATTGGTG

>contig00356

aTtACAATTATTTTGGATTCTTTAACAAAaTATATTTtACGtGCAAATACCATTACGTTGTTTTTCGGATTCGTCCTTTAAAATCTTcTTTtCCCCTtCcTTTtGTGCCATTGGATTTTACTGCGCATTCTATATTTGAAGATCTTTTTCGtCCTCGGTCTTGCTTGTTTCGACTAAATTGTCTTCGTCTTGGACCTTTtCTTCGAAAGATAGTTCGAGAGACATTTCTTCGTCCTCGGAATCCGTGTAAACTATTTGTGAGAGAATAGTATCCAAAGTTTGATTCCGTTTGTCTAGTGAATTACCATCCAATATTCTTGTTTTTTCGCGTGCTAACTCTTCCCTTAAATGAATGTTTtCCTTTTTGACTAATCCAAGTTCTTTCATAGCTTTGCCGATAGCAGTATAATTTTCTTCCAGTAGATTTTCCATTGTCGTTTGAGGTGATTTTTGTCCAGCTAATTGTTCTTTTAACTCAATATTTTCGATTTTAAGCAAGCCAACCTCGTGCATGCATTTTAAAATTATACTACTTTGCTCCGCAAGTCTATTTTTGACGGTTTTTACTtTTTCGATCAtTTCTGATTTTTCTTGGATGTGAAGTTCAATAGATtGTTTATGCTTTTCATTGTCACGCTTTAATCGTTCAATTTCCAGAATGAAcTTAT

>contig00357

ATACaCTGTATCCAAGGACAATACCGAACAGTGTTGAAATAGAAAGCGCTTTGCATATTAATGGAGAAATcAGACACCATAAAAATCATGG

>contig00358

ACCTGCTTGTGTGCCCaCTCCGAGTGCAGCAAAaGTTGgTGACGAGAAATACGTAACTGGCCTCGAAAACGTTTCCCGGCTATGGAAAGCATGGTCGAAGAAAACGGATCAGCTGtAGATCAATTGTCACAGGGATCGCAAAGTGGCGATGCAGCTCCTACAATTGCCACATCGATACAGTCTGTCATACAACCCAACCAACAGTCTGTAATTCAGACAGCGACCAACATTCAACCTGTTGCCCTTTCCAAAGGAAACGTCATCCTTGTAAAACCCAACTCAGTCATTCAAACAaCAGGAGGTTTACAAACTCTCCAGGTGGTGGACGCCGGGAGCGATGACGAAaGCTATTCCGACGAAGAATCTCCAAAAAAGAGGCGTGATATTCTCACAAGACGGCCATCATACCGAAAAATTCTCAACGATTTAGGAGGTGGAGAAATAGAGGGTCGATTACACACCTTAGAATCCTCATCTGAGTGTGACTCTAATGTGGACAGTGAAGTGTCTTCAC

>contig00359

CTTATGCGCCTACTACAGCTGGTTGTGCAATATTCTGCAAAGGTCGTACAGCACCAAAAGCTTATTGTGATAAGGATAATTGCCCTGCTACTTGCGAATCGGGTTGTCAGGACCTGAAGCATATGAAAAAACCATATGGACCCTGCAAAAAGTTTTCACGAACAAATTGTGCTCGCCGCTGATGGATCATTGATACAAAAAaCTAC

>contig00360

TTTtAAAaCGGAAGGCAATTTGAAGAAACGTTATTATTGTCTACATATTGTGATCCAGAAAAATTGTTCTAAAGATTTTCGAAATTTTCCAAGATGAGGAGCAATACAATATTTGCGGTTTTGCAAACGATTGCAATCTTGATGGCCTTCTTCTTTTCTGGTG

>contig00361

ACTTTTAGAGTTGAAGAAAAGTTACTAGATCATAAGTATACAAACAGAATGAGGAATTTTATTCGTAGACGTCCTCTTTATCAGCTATGTAACGAATTATCTCCGCTGGTCTCATTAATCTCTCTGCATCCATGTCGGGAATTTCGAAACCAAATTCATCCTCCATCGCCATTATGATCTCCACATGGTCCAGGGAGTCCAGGCCCAGATCGTTGATGAAGTGCGACTCCAATTTCAACTTA

>contig00362

TCAGCAGTGATTTTATCATAAGCAGCAACGACTTTCATCACGCGTTCTTCTATTTCCTTGGCTGAAGGCTTTTCTGCCGTCGAATATTTTCGAATTTGTTTCTCGACCACCTTGTGAATTGACAGTGGATTTCTCGTCGCGCTATGAATTAACCTATGTTCGATTCTCGAAGTCGTTTCGGCTCGGAGAACATCCAAGCACCGCAAGGATTTCCTCAAAATACCGGTGTTTCGCACGAAAAAGCGGACACTCGTCAACGACGCCATTTTCACACGTGCAGGAGAAAACGGAGTCTC

>contig00363

GCAGGGTCAATTTTGTCATAAAGTTTGAGAACTAAGAGCACCCGATCGGTGATAAGACTCAGACTTTTGGGTTCCGGTTCGCTGTAGTGGCGCACCTGCTTGACTCGAGGATCCGTTAAGCCCTTGTGAATTGACAGTGGATTTCTCGTCGCGCTATGAATTAACCTATGTTCGATTCTCGAAGTCGTTTCGGCTCGGAGAACATCCAAGCACCGCAAGGATTTCCTCAAAATACCGGTGTTTCGCaCGAAAAAGCGGACACTCGTCAACGACGCCATTTTCACACGTGCAGGaGAAAACGGAGTCtC

>contig00364

GACCCAGTCAAACTGGGGGCAGTCCGTTCGACGCGTCCAAAGCCACTTCTTCCGCTGTCCCCCcTCCGGACGGTGTTTTTCTTTTtCGGTATTGGAAGATGACGTAGACGATAACAGCATTGCCAACAAAGAGCAAAGCGATAAGGAGGGTTGGTAGAACAACGTCTACGGTGTTGTCGGCGTTGTTCAGCATGTTCGGCTGCGTCGAACCCGAATCCATAATTTGGGTTGTAAATGCGACTGCGTTGTCGGTTGCGACGCCTCTTCTTACCTCAGAGCCTAGGTCGTCTCCAGACAGACTGG

>contig00365

TGGAATATTAATTAATTTATCAGTTAGACACACTCCTAGAGTGTCACTGTGGTTCGCAACTCAACAGTTAGTGGCAGCCAACTCAACTATTTCTAACCCTAATCCGGAGCCCGATCCTACCCcATCTTCGACTGCTACGACGAAAaTCGTTCCTAAACGGAAAaCTCAAGGgCGCCCGTCTCATGTCAAGATCGACGTGCAAGGCCT

>contig00366

ATTCGGTCGTCGTAGGCTGGCCAATCGAAGtG

>contig00367

ATCCATCGATaTCTCGGTGCAGGTTCGCCTtC

>contig00368

GCCTCACATTCTATCAAG

>contig00369

ACCTCGACCCCCATCAAAGATAAATACGCCAGTGATGAAAAAGAATTGGGCGACATGATGGCTGATTTTATTCAGCACAAGTATCAAGTCGCCGACACAATGACTAAAATCAGTCACGATGAGCCTGACGACGACGAGTTCAAAAAACGACAGAAAATAATCGAAGACGCCAGCAGATTAGTCTCTG

>contig00370

TCATCGGAAACAAAaGCGAATTCGAGTTCTCCATCTCCAAAATCGGCCCTCTCTAAGAAAATTGGCCTTTCAAAGAAaGATAAATCGCTAGTATCCGGAATCGCGAAGGAAGTAGAAAaGAGAAAGCAGAAAACTCCGAAGAAGACGGAGACCAAACAAAaGGAGAAGAAATCGAAAAaGGATACAGGTTCTGAAAaGGTTAAAG

>contig00371

ACACTGAGCGCGTAGTAATTCGATTGCCAGTGTCTTCTACACCTGtGATAAAtAG

>contig00372

TTAGAATCGAAGGTGTCTACGAGGAACAAACAATTCAAaTAAAACCTGGAACCTCATCTCaCACCcAAAtCaGACTtCctGGAAAGGGCTtGAAAAAAaTGAGCTCCCTGGGAtaTGGCGACcaTTATGTtCACATCAAAATCACAGTGCCTGTCAAa

>contig00373

AAAaTACAATTGTATTTTtCCTAATTCAACATACGCTATGCTATGTCTTtCTACGAACGCACGTGAAGCCTATCACGCCCTGCCTTTCGTCTCATtGTAAAAAAACCCCCATAGCGTGTGTGATACTTTTtCATTTGAAGGCTTGATAGACTCTCCACGCGGTAACGCCGAGAGCCgTTAAGgTAATCGTGTTGTGGAGAATCCTCCTCCTTGTGTcATcTTTCCTAACTAGAAATGTGGGTGAcGaGGGTCCTAAGACAGCGAGAAGCTCGACATGTGGCAGTTTACTGGTATCAGTTTcgACCTGATACTGACAACAGGGATCGAACTGCCAAGAAACTGtaTAAAGTCCACAGGTTATTACAGACGTGAATCCTAGCGGACCACAAATGTAGAGCTTGTCtCCCCAcATGAGACCTG

>contig00374

ACTTCCAGACACGGAATTTCAATGTCAGTGGCGCGGTTGTGGAAGGACGAAAAAGTCTGTGCCACCATTCCCGAGTGTGCAAAGGCTAGCGAGGCACGTTAAGGAAGTTCACATTCTtAAaTCAAATGGACGAATTATCCCACCtGGCGAaCGAAGCAAAAATTTTGTTGCCTCGTCGAAAGGACTTGCCGCGTTACCTCCGATGGAAACCGG

>contig00375

TTACACTGAATtGATAAAACTATCAAATTTTCACCCAAAGGGCCTATTTTCTTCTctCTGTGCAATAGAAAAAGaTCTTtCTTCTTtAAAAaCAAAAAaaGTCAAAATCGGCCCCcTTtCCCCcGAGAGCACAGATTGTGAAATACTATAAAATATAAATaCACATCaGTCTCTTATTCGCGGCTGAGCGCGAACaTTCCCGTGTAGCGGCGGCGGCGGCGGCGgcAGTCACAAAAAACTGCGCAAAaCTCCCACCAGTTGTCAACTACAAAGTTTtATTGATAGCCAAGACGTCGCGCATCATCATGTTCCtCAGAGTCcACAaGGCGTTCACCACGTTTCCATGaTtGCCAACTCCATTCCCcAaCCATtCTCCGGgCAATTtCGTtATTtCGGgAaTCTGCGTATTTtCGGGAGTCGCACTCATCTGCTTGTCCCAGTTTGATATGTAACGATTCTCTACATTCAAGCCCTCGATATACCTTAGATAAGCATCCGAGTGCAAAACTCTGCTTGGTCTTGGAGGAACTGCGATAAAAAGTGGTTCCGGTTGCTTTGTCGTTGGCAGATTATTAGTCTGTGTTGCAGCAACAGTAGTTT

>contig00376

TGAGTTACGCGGCGTAAaCcATAAATTACGCTTACCCAAaG

>contig00377

AAATGGCTGACCCAGCGCCGAAGTCAAAAACGAAAGCGATCAAGCCTAAGCCCACGGAACCAGTTCCGTTGAAAAAGAGGCCCTTCAAAAGACACGGACGTCTTTATGCCAAGGCGATTTTCACCGGTTACAAGCGTGGACTTCGTAACCAACATGAGCACACTGCTCTTTTGAAAGTTGAGGGTGCGAGGTCAATCAAAGACTCCGACTTTTATGTGGGAAAGAGATGCGTTTACGTCTACAAGGCAAAGAACAAGACTCCAGTTCCCGGCAACAAGACGAAAAAGACAAAGGTCCGAGCCGTTTGGGgAAAaGTAACACGTCCCCATGGTAATAGTGGATCCGTAAGAGCCAAGTTCAAGCGAAACCTTCCAGCCAAAGCCATGGGACATCGTATTAGAATTATGCTGTTCCCcAGCAAAaTTTAAG

>contig00378

AGTTCATATTCAGCCATCTTGGAGAGTGTGCAAAGATAAGAAGAGGACCCGGGAATATAAAAAAaCCACCCGCATAGGATTGATCAAAAATTGACGTTCCACAGTATAAGAAGAGTTTAGCTGATGTTGCAATTTCCAAGTATATTTAATTAATATATTCTGAAAGTATTAGATATAGATATAGACCAAAATTATGGAATTATGTGTGTAAACTAAATTTGACCAAATATTTTTGTGATCCTAAGATGGTATATGGTATTTAAAATAAGC

>contig00379

ATTCTGCAAATGTTCAGTTTCTTGTTAAATCTCTTTACTTAACAAATCTTTTGTCAGACAAAAATTTCGATTGCAAAAATATATTCCTATAATTCACTGTTTGAAATGTACAGAAAATTGGCATTCACTTTCTTAATAATTTCAACAGGATTTTTCACGCCCTTTTCAATAAACATTGGCCAGAATGATTGTCGTGATGATTGTCCTCGTCCTATCGAAAAGAACAGCGTGCTGATGTGCATAATACAGAATAACCAAACTGTGACGATGCCTCAAGATAGGGTCGACTGCTTGACAATCTGTAAAAAAC

>contig00380

CTTTGCATGTAACTTTGCATGTAACTTtGCAATGCAACTTTGCAAGCACCAAAACTACATGTCTAATGCAAGACTGATTCACATTGTCTTATTGAAGAGTCCGGTGAGTCAaCCCAGAATGAAACATAAAAAaTCTTATTTTTtATTtATTTTTTtCATTtAAACAAATGAaTTGCAGAACAAAAaTTAATATTATCGATCCCAATAACTAaCTAACTTCACTTATTTCA

>contig00381

ACGCAAAATTTCAAGAAACATTGCGGACAACTTTGCAGGCAaCTTTGCATGCAACGTTGAAAGCAACTTTTGAAAGCAATTTGGCAAGCAATTTGGCAAGCAA

>contig00382

GGCTCAATAACATTATAAACGAGGTT

>contig00383

GTGGAAAAGGTAaCAAAGTTCTTCGGAGAGACAAaTTTGCGCCAATGCGGTTTTCCCACTCCCAGTTTGGAAACTGAAAGGCCATTCCAAATCAAAACCTATTGGCACGACTTTTtCCACGGATCCATCTATTCGAAGtAACATTGCGTCACATTTtGCAGCGATATCGTAGAAGTCATTtAGAAAGTtAATCCGTCCATtGAAGAGTATCGGTGGTAAGGAATCAATTTCGGGaTCTCTTTCCACTAATTCAGTTTCTTCTAAATTCTTCTTTAATTCCGGCGGGAGATGCCTTGGTGATCTACGCACAATCTTTGGTTGGTTCTCTTTCACgCCCTCTTTTtCAAGTGCAATGTGAGATCTTTTtACATCTAGCAACGACATGC

>contig00384

CCcTATAACCTATCTCCAGAaCATCAATAGGGTCATTGTCACCTTTGAATCCAGTAGCTTCATCTAACACTTCAGGATTTTCCCAtGTCTGTGGTAATGCCCCATAATTCCATATGTAGCCGTGGTGTGGAAAGCAATTTGCCACAAATCTAAGTTTACCTTTCTTTATATCTTGCTTtATTGGGTTTAAaGTTTCTTTGAGATTAATTTCCATTTtAGCATtGGTCCATCGTGGTATTTCAaCAACCATATTGACGGTTTTACTCAATTGTTCAGCATATAGTGGAATATCGTGCATTGGAGAAATAAGACTGTCTTCATTTTTAAAATATATCCTATAATCCATAGTATTCAAAGT

>contig00385

ACAAGCAATGCCTCCAGCTGGTGTTTCCCTTTTAACTAAATTAGCCCAATGCTGATGCACCTCTTCAATGACGTGTAAAGCGAAATCTCTTGATTTGGTTTCACCGTTAAAAGCAAACTGATTTTCGGGTTTGCCATCAGGAATTTTATAAATTTTGAACCACTCAATAGTTGCTTTTATTAGACCTGGAAaGTGTTtGTCAATATCGTTAACATCATTCAAATGATCAGCCATTGGATCGTtGACATCAATAACAATAATTTtCCAGTCCGTTTCCCCTTCATCTaTAAGTGCTACAGTTCCAATAACTTTtACTTTTAATACCTCGCCTCTTTTAGCTA

>contig00386

ACAAtACACATTGTtCGGTTGCCGCTGTGAAGATAATTAGACATCACAATCACTTACCATCACGACACGGGATTTtAtcAATCGAGATCTAATGTCACTCCT

>contig00387

CACACtCCCCtGTGAcGGAGACCCCTCGCCAGTATCCAATGACGAGGCTAtATTTTTCCCcGAGGAACAAGAGAAATCAAAGCGAGAGAAACGCGAGACTACCAAaCCCGTAAAAAaGGAAGTGCACTCTCTTCCGAAaGAATCcGAATTAACAGCAACCtaCAACGCCAAGTCAGACTGTTTGAACACGAAATGGGAAAAaCAAAaTTGCAAGGACAGCAAGAaGAAAGTGAGCAAAaCGCCTTCAAAATCTAATCAGCATCGTAAGCCGAACCGGCGCAAGAAGCGTGCTTCCCAAACTTCCGCCGAACGCGAGAACTTGAAATCAGCGACCAGGAAAAAaGAGAAGGCGAATTCGTCAAGTTCAGAAGCTTCTAACGAT

>contig00388

GgTTGGACATACAGTTTGCATTAACATTAAGTGAATATTTTTTATCTTTGAAAGCAGCTGATAAATTGTGAAATCAATTTTTtAAACACAGAATTTGAAGCTTTTAATCATTTCCcATTCCAAGAAACGGCCATGAATCTGAAATACCAGTGTTTCCTTTCTGTATTGTTGGTGTTATGTGTAACTGCGGCAGTCTTTGCAAAGGCTGTGCCTAAGCCAAAAACACCTCAGGATACGATGAAAGAGATCAAGAAACAAACACATGACACCGTTAAGAAACAAATTGGAAGCAAAGGTGACTGTTGGACAAGTTTTACGTACTCATACAAGACATATGCCGATATTAGATTGACCGAAATAACCGGTTGTAAGCCAACAGAAAGCAATTGCATTACTGACGCTCTTAATGGGATGAGAGGCGACGCAAAaTTAGCTATTGGTtCTGCTAAGAATG

>contig00389

gAATACATAGGGAAACTTCGTAAaCGAGcGGACAAAACATTGTTGACTGTAAACCTGTCTCAAAAAACGTACGTATGACTCATATAGTGGATTATTTTCCGTTCCAGAATAAAATGTCATAATCATTTTTGTATGTAATACATGGTTTTGGACAAT

>contig00390

AGGAAAGCAGATTTGTTTATAATTTGCGTTTCGTGTTGTGTCCGTTATCGGAGAAAGTCTGTATTCAGAAAaTAAATTCCTGGCTGGATCGACTCTAAAAGTTTTAAACTCTACAGGCATCAAATTTGCTTCTGCATCGTTCGTATTCTGGCGGCTTCAAAATTAAACGATTTTACCAAAATCCCTTGGGAATTGAACTGAGAGACTTTTAAAAATGATGGATGATGAAAaTGTAAAAATGGAAAAAGCAGCCGAAGCAATGGAGACTTCGGAGATTGACAAATTAAACGATGATTGTCTTGTCGAAATTTTCAAGCGTTTGCCAACTATACCTGACAAAATTCGTATAGAAAAAGTTTGCAAAAAGTGGAAAAATGCGAGCCAGAAGTCGTGGAATCATTTCAAATCTCTCGATTTATCAGCTAAAACTTGGGGAATGGAAGAATATcGTGATGATCAAAGATTAATAAGAAGCGTCTATTTTTATAAAATTATAAGACTCTCGGgACGATACCTAACAAGTGTTAGT

>contig00391

AGTCAGTGTTTGTGTCAGgATTTGTCCTGTTTGTGTGAATAAATAAAAACGATAAAAACTATAAAACTTGCATCAACCACAGTAAATGACGTTTTTGAAAAATAATGAAATTAATCTAAAACGAAAAAGAGTGAGATTTGTTTATTTTCTTGACTACATTCAATTTATCTTGGATGGATAATATGGTTATATTTGGATGAAAATATACTAAAG

>contig00392

ATAAATATGGGCAGATGTGTGATAAAAGGTTGTCCGAATACGCATCGACGGCACGCACT

>contig00393

GATGAAGGGTGCTATAATAAAGCGCTTGCAGATACTAAAACTGACGGAAATAACATTTATAATGCCGCTAGGACTTGCCTCAATCCGAAAAAaTCTCCCTGCTATAAATAAGATAAGGCGTACGGAAGATATTAGTTTCGTATGCACTCAAAAAACACTACTATTACTACTTAAATGTTATAAAATTGAGTTGAAAAATATCAAAATCTATTCAAAATTTAGTTCCTTTTTCTAGTTTTCTCGAAAATTTAACAAGATGTAGCTAAACCTACTACAGCCAATCTTACCAGTTTCTTTTCAATGTATGCACTTTTTTTATAATCTTAAGAATCACCTTTATCTGTTATGGTATTGTTTAAATTACACTGTGTGAAATACAACGTACTCTTCTTGTACTGATATc

>contig00394

aCATGGGgATACTTGAAAAATAGAAGCAAAACAAATAATTCAGCCATGCAGTGTGTCATATTCCGCTGCTTACTGGCATTTTTGGCCTTCGCAACAGTCTTCGCAGTTACCTATGATGAGACTGCTCATAGTATGGATACGTTCACTGCTACGATACAGAATGCTATGATAGATTTCCGAAATTGCGAAAAAAAaGACGATATATGGAAGAACAAATGTCAGAACATTTTCAAAGATCCAATCGTTATTAATTATGAGCACAAATTGATACAATTGCAAAAATGTAAAAAAAAG

>contig00395

TGCGAAGGCCAAAAATGCCAGTAAGCAGCGGAATATGACACACTGCATGGCTGAA

>contig00396

TCTTCCCGCCGAGTCTAAGGTGCGAGAGCGGAGAAGCAGGCgaGAAGTATCATCCATGGTCCTGTTATCGTGTTTGATTAATCCCATATCCGTCGTTATATCTTCACCATTATTTtCCACACTGTCTTGATTCAAACTTTGTTGACTATTTGAAATTGAACTGCCGTGTGTGCTACTGCAATCGGAGTGAATAGAACGCCTGGACATATTAGGCATAGCCAGCACACCCGGTGCTATTTCGTAACGACATTGCGacGTTCGAGAATAAGTGTAGTCAGTTTtAGGAAAGACA

>contig00397

ATCTTCACCTCCTTGATTTAGAACTGACATTGCTTTACTGTGTTTtCTTCAAC

>contig00398

GACCTTTATAATTTCTGACACGTTATGGAGCATATCATACGATTAACTGTTCGATCTAATGCAGTTTTCGGATATTCCAGAGGAATTTCAAAAATAAATTGGCAGCAACAAATGAAGTTTAATAGAAGTATAACTTCTCAAGAAGTTTTCGATAGAGAACATAAATATGGTGCTCACAATTATCACCCATTACCTGTTGCAATTTG

>contig00399

CAGAATGGCAGAGGAACGTTTGGATATCGTTATTTTTGGGGCGACAGGCTTCACCGGCCAGCATGTCGTTCAGGAGGCTGCTCGTTTATCAAAAGAAAAAGGATTCACGTGGGGTGTAGCTGGTCGTCGTAAGGAAGCTCTGGAGGCAATTGTCGCATTTTCTGAAAAGATTCCGGTAATAGTTGCCGACTTGAAGGATGAAGAATCTTTGAAGAAGATGGCTGAGCGTGCCAAGGTAATCGCGAACTGTTGTGGACCATACCGATTTtATGGAGAGGCGGTTGTAAAGGCTTGTATCGCCGCTAAAACTCATCACGTGGACGTTAGTGGAGAACCTCAGTATATGGAAAGAATACAGCTCGAATATAAGAAAGCGGCACAAGAAGCCGGCGTATATGTGATCAGCGCCTGTGGTTTCGACAGTATTCCATGCGATCTTGGAATAGTTTtCACTGAAAGTAAATTCGATGGTGAAGTCAACAGCGTTGAGACCTATTTGAAAGCTTGGAACACTTCAAATGCCGGCGGAGCTTCTATTCATTATGGT

>contig00400

ACTTTCAAGtCCGGCTGTAGAAGAGACGGGgATGCGACTCACGAGGACAAAGACGaGGGCTCTGGCTGCAGCCAATGAAAATGGAGAACATGTGAGTAATCCTCCGACGAACACGCAAAAaCTGGCGAGGAAATCGCTGGCTAAGGCGAAACAGATTTTGAGAGCGAAAGATGCAAAGGACGTTGAAGAATGTAAAG

>contig00401

AGAAACTTGTGCCGAATGaTAGAGTAGTGCAAAAGCAGCAACAGAGAATCACTCCAAATAGTAAGATTCGACCTCCGATGCCTTCTTCTACGAGTAGGATTCAGACGACTCCATCTAATTCACAAACTCTTTCCAGTTATTCGAAGGCAATGACGGGATCGCGCACAAaTATCGTGACCAACGTAGATTCCTTCATTCAGGCTAAAGGTTCAGCGCAAAAACCAaGCTCTGCTGAAAAAaTGTT

>contig00402

GGAAGAAAaGAGAAAAAGACAaCAGG

>contig00403

GAGTACAAAACCACTTTTGTCGGAATGTGTCGTTATTTATCTTGGGTACAGC

>contig00404

GATGAATTTGAATCTGAATCTCTGGACGTGGGTGACTTTGATGTGGTAGACGAATGCGAAAGTGAAGAAGAATCGGAAACTGAAGAGAACTCGGAAAATAAATCAAAAAACGATTTGCGAGAGAAAGATAGTGATTCGATAGATAAAGTCAATCTcAAGAACCAAACAAAAACGATTCCTGAGCCGAATACAACAGAAGCGATGGATGCTTCTGATAGTAATGAAACAATTACTACAGAAGCCGTAAAATCTGATGTTTCAAAAGAAACTGAGGTGGAAGTTACAGAAGGTCAAGCAAAAACAGAAAAATCAACCGATAACAAAACTGACCTCGCTGATTCTGTAAACGAAAaGATCACAGAAGCTAAGACCTTAGAGAACAAAaTTACTGTAGAGAAAGAAAGAGAAGATAAAaCTTCTGAAAaGATGGAAGTAGATGAAGAAAAaCCTa

>contig00405

GACAGAACTGACTCCCTAAACGAGTGGACGGACGAATTTTTCCTGTTTAACAAACTGGGTTGCCGATTTCTTCCTTTGCTTCTTCGTATTGGCTTGTGATTACTTGAATGAT

>contig00406

ACTTGTtCtAAATGTTGGTGTGTTTTgTtCATTAAAAAaTAAAaTTTCAGTCTGAAGAGGTCAACGAACCAAGTTGACGAAACGTTACAAAaCCAAaTTGTGAGTTTtATTGAAaTTtACCCCCTACGATCGACAAAAAAACCTAcTATTCAACTTAAaTATGGAGGGTGATACTTATTTTTTtG

>contig00407

AAAATAAGTTTTAATATTTGAGGCTTTACAAAGTTTACGTGCTGGCCTCTTTCAAAAGACTTGGAATtGAAATTGCCCGATTAAGCAAAGAGAAACCTTtCAAGCATATTTTAATAGAAAATTGCATATAGGAATGGAAaCACATTTATCACCTTTCGAATGACAATTTTTtAtATTTGAaGGTTATAATTGCGTATTTCAAATGATAGTTTCTTtcAAAGGAATAAAGCTTGAACTTGGTAATTCCCGTATTTTtAATATAACTTAAAATCACTTTTTGATGTTCGtCATTATCTGTTTAGCAATATTAAAATTAaGaGATTCACAAGATAACTAAATTTCTGCGTTTTTCTACATTATCGATGAAATTAGCGACCTTTATCAAGCACTGTTACCATCTCCATAAAGTCTTTtGGATATTATATTCAGT

>contig00408

AATTGTtGAATACACTCTACtCCACTGTTTTTGTAATCTGGATCATCCTTGGGTATAACAAACGGATAACACAAAGGTTTTTTAAGTAATCTTTATCTAGCTTCCTTTGATTTGAGCAACACTGGTTTTCCGATGTGGCTTTTGCACGATCTACCAATGACATATCTTTtGCAATAAACTGGCCTTGTGCCATtGTCGCTAAAGTAAACTTTTTATCGAGAATAACGTCGTTGGGTGTTAAAGTGAAGCTGAGAGTTCTAGGAAGTGGCAATGGTTTCCcACTTTGGGCCAATCGAGGAGCACGAATTCCATCAGCATAATCTGCAGGAAGTAGGCGTAAAAGTCTTGTGTATGGCATTCCCCAGGTGGGATGCAACAAaTTATTGCAGGAACCATTGAAAGTTCGATACCTGGATTCTTTACAGGAATATGGGGAAACATCACCACATTTAATGTCCATATACTCTGGCGTTATTTCTTGTGGAACGCAATTTTGTTGAGCAGTCACCCGTGCCAGAAGTGACAGCAGGATGAAAAGATATATTTGAATCATCTGTATACGT

>contig00409

ATATAGCCACAAAAATTCTAAGGAAATAAACTTTGTTCATTGTGAATCACGCTCATTCCAAT

>contig00410

aCTTTGTTCATTGTGAATCACGCTCATTCCAATGTAAGTTGTTACATATTTTCTATTGCAATATTTTGAAAATTTGTCTAAAAGTTCTCTGGTACTTTTTTAAAG

>contig00411

ACATAGCTATCAACAAGCAATTGTTTCAGCACATGGCGGATTATATagCGGCTGGATACTTTTGACGAATTTCCTGAAGCCCTTCcTTTCAGTCATGGATAGTGGCTGGTTATCCACAGCTATTATGTATAAAAGGGCATTAGTTGCATCTGGAAGGCATTTCTGGAGGCATCTGAAGCGTATTTATGGAAGGATGCACGACTCTG

>contig00412

aTTCGATCCAAAGTTTTGCAGCTGGGATCTACTGAGATATACCTACAATCTACATACAATATAAAGAAAaTGTTCTTTtCAAAaCTTTtGGCAACCATGCTTGTGCTACTGGCGCTAGTTTATGGCGCGGCTGCTGAAGATTGCACAAACCCTACAGTATGTAAGGGATTGATGGGTTATAAACAAGATTGTTGTGAAGGACATGTTTGTGGGGTGAAATACCCAAATACTGTAACACAGACTTGTCACTATACTGGTAAATTACCGCCTAAATGAAGCGTTCAGATGTTTTACATAATAAAATATTAAATTGTGGAAaCT

>contig00413

ATTTATAATTTAGCATTTTCGATCTTCGATCAGGAATTGCAAGTTTTTATCCAaCGATTTTGAAGAACAAGAAGAGCAGACAGAAACAgAGGAATGAAATCGAATTTAGGTATCTTCTATTTGTAAGCATCCTGGCAAGTGTAACcTCCAGCAACAGAGAGTTTGCAAACTTCATTTTAGCAGCCAGTACATTGACATTGACAAAACCAACAGTAACCCTTGCAAATTTCTGCAGCTCCGAGGCCAAACACACACATCAGAGCAAAACAGGCAATGATTAACGAATGCATGTTCATTTTTtCTGAAAAATAGACTGaCCtC

>contig00414

AAAAAGGTtACAGTGATATTTTTATTACTTTAACAGAAAAATATTAGGCAGATATGGTTTtAAACATTTTTTtAAGTACATACGAAAGAAATAAACGT

>contig00415

CcTAGAGAATGCAAAaGTGCGAAATAAGGCCACGGGTGAAGTGGCAACTTCTCTCTCTAGAATTGCCAAAAGTGTCTATCTTgAAGATAACGgAAaTAAaCAAaTAAAGCTTGTGAGGCGTCGAGTGGAGCATATGACAGGTTTGGTATTTGAAAAaTCCAAGAAATTTCAAATTGTTtATTACGACATCGGAGGCTTTTATACACCACATTATGATGCCGTGGTTAAaGAGGACAAACACTCTTT

>contig00416

CTTtAAGAGCTAAGGATtCTTTGCTGTCATCATTAATACCAACTGGTATTTCGACGTGTTCAATGCCATCTTCGATGTCACCTCTCACTCGTTTTGGATGCATGTagTGCATCAAGTATTGTCGACGTTTTTtCCTCCATCGTTTGCTCGGATATGTGTAGATTTGTCCATGTAAAAGGCCAGGAATTCGTTCCCTCGCGTTCATGAAGAGCGCAGAGTGATTCTGCGCCACTCCAGTCTGCGAGTCTAG

>contig00417

GTAAACCAGTCCAGCATCTATTCCAGCAAC

>contig00418

AAAAaCCAAGCCTTTCGTTCCATAAACCATCATTTTAAACCTCCTCAACGATTTAGTTATACTGAACTGATCATTTTTCGATCCAAGATAATCAACTGCATGTTTTTtAACAGGTAAAAGGTGATCCAACGCATAATAGAGTAGCCCAGTATACAGGAGAAATGCTAATCCTAAATGTGAtGCGAGGCGATACTGTGACaCTCTTGGt

>contig00419

CCAACCAATGTAAGACTTGACCAACACTCGCATCAGGATCTGACATTATcAAAGTATACAAGGCGTTGGCATCACACGCATAATCAATTTCCGGTGCATTTTTCACCTGTGTTGGTGTGAATTCTTCACCAAaCGTCACCTTCTTcTCTCCATATTTTACCATAACTTCTTTAGTGGGGgCTTTGTTTATAACATCAGGAACGATTTtAGCTTCTACGAAGTCAGAAGAAATATCTGCGGCCGAAAAGCCGACCATGAATAGCAAAATTCCTGACTTCAAGAGATTCATTCTTACGGCTGTCGAAATGgTTACTCCC

>contig00420

ACTTTtCTGCAAATTtCTTGGCGGAGAATCCTCGACGACCGGCTCCTCcATTAGAAGTTAACTGAGATTCATCGAAaTtAATTTTtGCAGTCTGCTTGTGAACCAAAAaGACGTATCTGTGAaGTCCAgTTCCTTTCGGGGGgCCTGGgCCAACATAAGAGGCCAAAAGATTACTTTGGGAAATATTATCTCCAGGAATATTTCCAG

>contig00421

GTaTtAAATTCGTGGAGGAATAAaGATCTaCAAGATGAATGTCTATGCAGTTTTTTGAAGAAAGCGCATCACCTCCGGGCACTTGATGTAAGAAATTGTCCTTCAGTTAGTAAAcAACTTTTGAATGTTGCTATtAGgTtGCTCAAATTCGAAATTCGAACAGTGCGCTCGATTTTTATGtCAATcAtGAAACTGAAATAGATATGA

>contig00422

ACATTtCGCGTTTTTGCTGTAAGTCGACTGTTTTTGGACGAATAAGGATAACACGCGCATCGTAAATAATTGAAAATATCTTTTACCTTCTAAGAATAGTTATATTGATTAGATTATCGATATTCCTACAAAAAAAAaCAGGTCTTGTAAGGAAGAAAATTGTTTGAAGTTTACCTTAAAATCCAGTTAAATCATCACACAGTTTACAGGTTATAAAGTTAAGAAAAGTGAGTTTGTAGAAAAAAGTAAGAACTACATTAGCAAAACGGCACCACCAAAAAAATCTAAGAAAaTGGCGGAAACTGAAGAATTAGAG

>contig00423

ACCCTGTtGGTTGATACACAGGATTtGATCtGGCAGCCACTTGCGACTGTGGTAAAGATGCTTGATGTGCAGGAGGCAATTCCTTACCTGTTGGCACCGAAGCTTGTTGTTGTTGACCCATAGCTTGAGCATGACCTTGTCTAGTGTAATAATTGCGGGAATGCATATTTtGATCTCCTGTATTTTCAGTTGTGCCACCACTTTGTATATACGTTTGATGGGCAGTAAGTTGATCAGTT

>contig00424

GAGGCGAATATAACGCAAGAAGTGACAGCTATGCGAGTGTGCTGCTTGTAAAGAATATGATTTGTCATGTGCAATTGTAAGCAAGTCATAAA

>contig00425

TtttttAcaGGTAATGACTACCTTCGTGTTAGGAATTGGTGTTGGGCTCTTTGTGATAACATGCCTTTGGTTCTTTGCTGCTCTTATCTTTCTGATTTCTTTGAGAATTAATAAAAAAGTTGGCTTTGCTGTGATTGCAATTTCTGGGATCATCACTATTATACTCGTCAGTGTTCCTCGCGCCTATGAAAGTTCAAAGTTATTTGAAGATAAGCCATACGACCATTTATTCATCTGGCGTTTGATACTGCTAATGCTGATAGCCGCTTCATCTTTGGTAGGACTCATAGGATACGTGAAATTTGAATTGACGGAAACCGTTCAAACAGTTCGCATAACAAGCTGGGTGTCTTA

>contig00426

TGAAGTCATTTTGTAGATTCATTCTCTGATTCTGTCGCTGCAGCAAAAGTTGTTTGGCTCTCTTCACTCGATTTCGATAAGCGATCTGCGCTGGACTT

>contig00427

CTTCACTGAAGCTGCGGTCATCGACGTTTAGTGTTGGATCCACGGCTGAAGTTTCGCGGCTGCGAGATCGCGAACGTGGTCTCGAGCGCCTCTGTCGGTTAAATtGTTGACTGCGGCCAAGCATGTTGAGATTTTGCATATTCAGCCTGAAtGGTGATCTTCCCCTAGGTAGCTGCTGCaGATTTCCCAtAATTTtAtCGTTTTGTCTCTGTCCAAGATTGAGCGAACGCTGTCGAATCTTaGGAGAAAACTCATGTCCTAAAGTGCACGTGAAGTTGACGTTtGAAATTGCGTTCATGTGAGCtGAGgTTtttttgtcatacTTAAGTCTCATAAGCTTATTTTCATATTGTGCTCGTCTCTGTGCCCGAACTATTTGATCCGACCTCAGTCCACCGGTGCTAAAACGTCGCTGGGATGTTAAAATTTGTTGTCGTCGCTCTAATGCTCTCTGATGTAGTGATGTTAAAGaCTTTAAAGcGCCCCCcATCC

>contig00428

GGTAGCGATCCGCGGTTCCCGCTACCCAGCCGATCTCGAAAaGTTCAGAGGGTGGAATGTGCGGCACGCCCGGAaTAAAaTTGTTCTCGTCGTCATtGACCTTGTTGTTCTCATtGTTTTtCTTCACGTATGTCTTAAAaTGGAACtCTTTATCCGGACTCGTGATTAGCAAACATCCCGAATCGTTCCcACTCTTtCTGcGATCCATGAAGGCTCTGATTGCCTTTATTCGACACGTCTCATTGTTCAGGCGACAAAGATCTGGTTGCATGCCCcACGGATGTCGTTCAGCGTTTCCATCT

>contig00429

CAAAGGACTATATATAGAGCCGATTCTACAACGACAGTCATCATTCTCGGACTATTGAGTTTGTTAACAAACCTTGACATTTCAACCATTCTTCTCTTAAAGAAAGTTCAATTTGAAAAAGAGTATCTTCAAGTTTATTCAGTGTTCAACATGACAAGGGCATTGTcATCTGCTTTCATATTcGTCATCACAATTTTgTTCTGGGCCTCCAATTAAGTCGCTGCGGTGGAATGTTTtAGCTGCGTTCA

>contig00430

CTGCGTGAGGTATGATGGTCGCTATCAGAACCATAATATAGATTATGCTAACTGCATGGGTGATTGCGGATCAGGTATGGTAATTGTATACA

>contig00431

AtACGATCCAGAACAGtAGCTAGGACGtCTACTAATCCAATACACCAGAAAGGAAAGAAACTGAAGTTAAACCTATCGGCTGTGGTCCCCCTATAGTTTCCATACAACaGAA

>contig00432

TTtGTGTGTCCAAaGTGCATTGATAGTATATACATATATATCTCTACTATAATCATAACACGAGATCAAAACAGCGTTTCCTTCTCAAAGAATAGGTATATAATATATATATACATATATAGCCTTAAAATCTTCCTTTGCGTAGATGAAAGAGAATTGGAGAGAAGtCCTtGGGAGAGCTCGTAAACGCATTTAATTTGAAGCGCTTGTTGAGACAATtAGGATAATCGACAGTTGTCtACGGACTTAGGATCGTTTTTtGAaGAAaGAATCGCCATTACGTTtCCTt

>contig00433

AATCTGTCCTTTGATCATAATATGACGACAGTTCACGATTTGTAAATAATTTTTGTCTTATATTATAATTTTAGTCTAATATTGACTCCAAAGTTGGAAAAAACTCGGTGTGAAAGTGCGAAAACTATTTTTGATCCCGTGACTTCATTTAGAATAtCTTTTCACAATGTCTGTAGcAGaGTTTTTGAAAGGGTTGCCCTCACAT

>contig00434

GCAAATTTTCACACTGACAGTAACAATCGGACATGTATCAAAaGACCTTCCGTTTATCTGCCAACGAAGGAGTATCCTTCGGAAC

>contig00435

AAGAACTCGGGgAGAAAAaGGGAACTGCTGTCAGCCAATGCAGACCTACAAGAAGACGGAACGAACAACC

>contig00436

GGAATAAGACAAGTTAGTGGAGAGGTCAGCAAGGTTACATGTACCTAGCAGGTCTTCACAGTCCCTAAGCAGCTTCCAAGACTTCGCTTTTGTtGTTGAAAaTTGAAATATTTtGTTCAACATTCATCTTTTTGGTTTCAGAACATAACTCTTCGTGAAGAAAaTTAATCTTTTTGGTTTAAATATTAACCTGCTTCGGTCGAAGATGATCTTTTTTGTTCAAACTTAAAAAaTTtAATTGACATTTtCTtAATTCACGAATAAAAAaTGTTAATTAAATATTCAACTGCATGGTTGGATATTTTGTACTTAGAAA

>contig00437

ACATGGGgATTCCACGAGGAAAAACCTGTATTGCTAACAGATTGTCTGAAAATTCGTCGTACACTATGGCTATAAAATTATCATGCACTTTCTTATTCATGAACGCAACTTTATTCTCGCGTATATTTGCTGTTCCTGTACCTGCCAAAAA

>contig00438

TATTCAGGCAGAATATCTGAAATGTATAGAGAACTGTCCTACTAACGAAATTGAAACTTATTGTGCAAGGCTTGGTAAAACCCCTTTGAATGTAGGCTTACAGGAATTAGTTTGCCTAAACGGTTGCGGAACA

>contig00439

ACCAAGAGTATCAGTTGGTATAGTTTTATTTCATTACAATTTCTTAAAAAGATTTTCTTATTGGCAAATTATTTAACAATACATTTTTACGAATTCTTCATAAATCCATAACTTAGCGTCGTTAAAGCTGAAAGAATGATTCCCGTTGTATTCCTTCTTCTGACCTGCACTGTAGCTTTGGCTCAAGTTCCTGGTATTGGTTATTGTCCAAAAGTGACCGTAAAACAGGATTTTGATGTGGCAAATTATGTAGGTGTTTGGTATGAAATAGATAAATATTTTGCATTTTATGAAATGTTTGGACAATGCATAACTACAAAATACACACTAAATTCAGATGGTTCAGTAGATGTTGTGAACACGCAAATCAACAATAT

>contig00440

AACTGGAATAGCATCATCGATTGAAGGCACTGCAAGATTTATTGGTGAAAAGAATGTAGGACAACTGAATGTCGTTTATCCAGTCGGTCCCTTCAAAATTCCTGGCCCATATTGGATTTTATATACCGATTATGTTGTATCTGTCGTTTACTCCTGTAGTTA

>contig00441

CACACAAAGGAAATGCAGTCTTCAAAaTTTCTGATGGCCATTCTTATGGTATTGTTTTTAATTAATGTAGCAATGGGGGCTGGCTCTACATGCACTAAGCTCCATCATAACGAAGAATGTGATCAAGAGAAGGATGCAAACGGGAATGGTCGTCAAGATCGTCCAGGCAATTGTTGCCCTGAATTTGTATGTTATAAAAGGGCAGCCCACTACTTGAGAGGAGCGTGTGCATATACAACTGATGTTCACCATTAACTGCTAATTATGCAATACGAAGACATAATAATGATTATAATATGATTGaCGAAAATGCGAATAAAaCTATACTAGATTATACTATTCCG

>contig00442

AcAtGGGGATtCGgcGCAGAGCTGTaCGTATAGTCGGGAGATCAACTTATCAAAATACAAAAGAAGCTCCATAAATTAATTTGTCGACTTTGTTACCACCTTGAATCTGCATT

>contig00443

ACTGCAGTCAGCAAGCAGTGAAAaTGATGAAAGAATCTGGTCAAGAAGGtCACTTGATAAATATAAACAGCGTTGTAGGACACAAACTGATTCAATTTCCGGGAGCATATACAAACGTATaTGGCCCAACAAAATATGCAGTTACTTCAATAACAGAATCCTTAGgAaTGGAATTAATTGGAACTAAGATAAGAACAACG

>contig00444

AGCATCAGTCCAGGATACGTGCaCACGGATATTATTAAAaCAGTAaTAACTGATCCTCaGATGGAAAAaGaGATTTCAAAAaTGCcTGCATTAGAATCAGAGGATATTGCAGATT

>contig00445

TtgATtCCAGAGCAGtgCGACATTGATAAGGTTTCATCGAATCTCTCCTCTGATGGCGTTTTGAGCATCACCGCCCCcaGgAAGGATCAACCAAAGGAACAGAATGAGAGGACGATCAAGATCGAGCACACCGGGAAACCCgcGATtCGCGAAAAGGCTGAGCCAAAGAAGGACGAAAaGGAAGAGAAGGCGCCTGAATGTCCTaaGAAGAAATAgAGCCTCtGTCTTCGGTTTTCATTAATCATTCCCTTT

>contig00446

AATTTtATTCACATATATTTCTTTCAAGCTATTCTCTTTATTTATCTTTCTCATTCACATTCAAGCTCATTATTTCGTCTTTCAGAGTATTTCCTACTTCATTAGCATTGCTATTTAAGTTTATATTATTTACATACGtAGCTTAGGTTTtAtAAAATAAACtAGAATGTTaTGag

>contig00447

AGTTTATTGTCAAACCTCATTGCTTCACGAGTTATTTCTTTTGCTAAAAAaTCTGTTTTGCCTTGATTTTTGAAATGATTGgAGGAGTTGTATTCgTTCTATTCCTAA

>contig00448

TCATCGCAATGATTTTCCCGGCCATGTTTGCATTTGATGACTGTGCAACTGATTGCGAAAAAAAaTTACCAAAGTCTTATGCTTGCGTATCTGATTATGATCACAATTATTACAATATGACTCTCGGGGAATTAAATTGCCTTAACGCTTGCGGAGCAGCTAGGATACAGTTAATGAAAGAAGCAAAaGATTGTCATAAATAGTATTAAAATCGTGAATAGAAGGTGCTGAATAAAAATATTGTAACAGGCGGACGCATATATGCGAGGTCATCCAGAATAATAAGGAAACCTATTTTTGTCCACTGCAA

>contig00449

TATCTACAGGAGCTCTGTTCCTaTTATAtACCGATTAGAGAAACATGTTGCGCTCATATAGGCCAAGGTCTCCTGTCTTTTGCtGTTCCTGTTAAAGGCGATACCGAC

>contig00450

ATGTTTACGAGCGAGGAGAGTCACTGGCAGCATTGCTTGCGCATTCCGTCTGAACTCATTGTCGGTTTCACGTCGATTGGTTCTACATTCTGTGGCCGAATCGTGTCACCTTCTGGAGGATCTCTTATCTGCTTTTGCgAGACGATTCTATAAATTTCAGTTAATAtATTTTGAAAAGCCGTCTCGACATTGGTCGAATCTAAGGCAGAGGTCTCAATGAACGAGAGGCCATTTTTTtCGGCAAAGGCTTTGGCCTCGTCTGTTGGGACAGCCCGCAAaTGCCTCAAGTCAGACTTGTTtCcGACCAACATTATCACGATATTCTGATCAGCGTGGTCTCGTAATTCCCGCAACCATCTTTCTaCATTTT

>contig00451

CAATTCGTTATCGTTTtGCTCAATTGCACAGCAAAaGTAAaGACTCGCGTATCTTTtGTAAACAACTTTGACATCCTTCCACTCGAGGAAACTTGACATTTTTGGTTTCCGAGCCAAAaTCGTCGTTATTAACTCTCGAGTGATCTTCTTCTTGAGTTTGTCAGGATGTGCGACATACCATTTCTGTAGTCGCAATTTCCCTTGGCGACTGAACAGTAACATGAATtGCA

>contig00452

TTCTAAAAACTGAGGTTAATTACGTTCTAAAGTGCAACAGTAAAAGGTAGATCCAAAATCACACACTCGCCACCCTTTTTTCGGGTGATATCACTTTTTGTTTGTGATATCACCACGTAGCAACAacAAATATTTCGAACAACAAAAAGTCCACGTCCACCgCACTCACAACATGATGAGGTT

>contig00453

ATAAGTGCGACTGCAATTATTTCCGGTTCCCACTGAAGACATAAAGTTGTGCACAAaCTGTCATTGaCAAAAGTCcAGGCCATTTGCACCATTTTTtGCAACTTATTCTTGTCACCTTTTAAACACTTGGCGTATTTTAATAGATAAAGATACGGGTGCTCGACTTGCAGGTCGAATTTAATTGTTTGAAGCAAAaTCCTTTCAAGCGTCATTACTTCTT

>contig00454

CcTTTAGGATCTTCACCGAAAGACAGGAACTTTTGATCAGTCAGCAATGTTTTtGCAGTTTTGATAATGTCTTTGCACTTTTTtGGAGTCTCTTCCACTTTGCCGGCCAAAaTAAaCAACTGCAGGCAGTCACGTATCGTGGAAAaGTTTtAAATGAGTGAAACATGTAAAaTCGGTGGAAATAAACGACTCCAGTCGCCATGGtGTTGTATCCGAGATCCATTTTtGtGCCAGTGTCGATAATAAaTCGCGCGCCTTCTTTTCTGT

>contig00455

TATGGGCAGCGGTGGTCACAATGCGCCTCGTGGTGCCGGGATCGTCAAGCGAAACACCCACACCCcTCGTGAACTGCCACCCAAACCTGCCTCTCGAACTG

>contig00456

TAAACAAAGTTCAGCCAAGTCGCCCcGTGCCAAAAACTGGAAACGTTGGAAGTCGTGGGgATACGGGgAAAGTTGACGAACTTAGTTCGCAGATCATGGATTTGAAAATGACAGTCGATGGACTCGAGAAGGAAaGAGATTTTtACTtGGAAAACTGaGATCGATCGAAGTTATGTGCCAAGACTGCG

>contig00457

ACATTGTTGTGTTGTTGCAAACGTTCCATTGTCGTTtACTTTTtCTATGCTGTCAGTCAAATGGAAAAATATTAAtCGTGCACAATCACGTAAGACTGATGAAATACATTTTtAAAAGTAACGTAAAAAaTaCAGAAAaTTAAATTTCTGAAGGAAGAAGAAAATTaGCATCTTTtGTTGAAGAACCAAC

>contig00458

ATtACTAAaGCAAacATACAGACATCCATATCGAATCATAATTGTgaCAAaTGTTCTCGAACTTATCGTTCGTT

>contig00459

AAGTAGTTTATATAGACATAAAAGAATAGAACACGCAGCAGTCAAACCACAATTTGTATGTGATTTTtGCGGATATAAAACAAAGGTTAAA

>contig00460

CCGGTTTAcGTTGTCGTTCGGGGTGATCGGTTTGTTCTCCGTAACTTCGTTTTCTTGGCAACGATGACGTCCAAGGTATCGCGTGATACTCTTTACGAGTGTGTGAATTCAGTTATTCAGAACTCGCAGGAGAAGAAGAGGAAATTTGTGGAGACGGTGGAACTCCAAATTGGATTGAAGAACTACGATCCACAGAAGGACAAGCGTTTCTCAGGCACCGTC

>contig00461

AAGCTAAAGAATATTCCTCGACCAAAAATGCAGGTTTGCATTTTGGGAGATCAGCAGCATTGTGACGAAGCCAAGGCCAACAATGTTCCGTTCATGGACGCCGAGGCTTTAAAAAaGCTGAACAAGAACAAGAAGCTGGTGAAGAAGCTTGCAAAGAAATACGATGCCTTCCTTGCCAGTGAGTCCCTGATTAAGCAGATTCCTCGTCTCCTGGGTCCAGGTTTGAACAAGGCCGGTAAATTCCCCGGTCTTCTCTCCCACCAGGAGTCTATGATTGCCAAGATTGACGAAGTTAAAGCGACGATCAAGTTCCAAATGAAGAAGGTATTGTGTTTGTCGGTGGCTGTTGGACACGTtACCATGTCGCCAGAtGAaTTGGT

>contig00462

AGGGTTGTAAAGAGAAGGATCGTAGTAAATTATTCGAGCATTTGACGGAAaTCGCCTTTAACAGGGATAATGCcTATCACCTGAAAAGGGCTGTGTGGAATGATGTTCGGGACAACTGGATTCACTACACCGaGGAGGAAAaGTCCCTTcTCAAAaGAaGGAAACcTCAGAACCTCACGCCTCCCGGTTCCAGTgaTGGATCCAGTGG

>contig00463

AGTGGACAGTCTCCAAATTCCGTTCATCCAGGTTCACCGCCGGCGATAACAGCGCCACCACTGGCACTTCtAAAGTCCAAGAGACCAGGAtACTATGAGGGTAACgACGGCTTACCcACGAAGAAaCCTCGAATaTCTCATTATCGGAAACCGGAACCtCTTTTtGTGAACCACGGAGAAAaCcGAaGgACGGCTGGTGGTAGTGTTAATAGTGGTGATAGTCGTGTCAATAGTGGAGATAGTCGCTTCAATAGTGCTGATAGTCGTATCAGTAGTGCCGAGAGTCGAGTtAGTAGTGTTGAGAGAGTCTCGGGTAGTGTTAATAGTGTCGAGGTAATCAGCGGTAGTAATAGTGGT

>contig00464

tACAACGAGATTCTTGAAGATTCTTTAAAGGGgATAGATATTTCTATGCAAACTTTTTCCCTATCGTGCATTTTTtGTTtgTTGACGAAATGAGAATTTCATAATTTCAAAAATAAAGTTTGCATCATTTTTTtGAAGCAAACATTCTGGAAAAGTTTTAAAACCCCACCTCAATAGTTTTATAATGCAAAAAAaTAATATTTTAACTTAATCTTATAATTTtGTAGCAATTAAAGAATAATTCAAACATACAGTTTGAAAGTACAATCCACAATTTTTATTAAACGTAACTATTGCAGAGAAAAATACCATATCGTATTAGTTTATGCTATTTCTTGGGTGCACTTTCTTTCCACAATGATAAATCGATCGAAGGAATATCTTTACAATCAACCAGTGGATTCTTTTTAGAAATGAGCTCAAATCCTTTCAGCTGCATTTTTTtAATATTATTACCGTTGTCACATATTAATCTGGAGATACTTGCCTTCCGAATTTCATTCAGTTGTTTGAGGGTAAAaGGTGTTCCACTTTTtCcATTTTCATACCAGAAACGATCACCTGATCTTGCTTTAAGAAATTGTTGAGTCAAAAGGCAGTGATTTGTTGGCCCAGCCATAG

>contig00465

TTTAGCAATGCGATTGTGTTCTCTAATCAGAATAaTATTCACAATTGCTAAGAGCGGATTTTGATTtATTCTCGCGTCACCAGTTCTGTAACAaGTGTTTACTGCACCATTGCAaGTAAAATATTGCTCTTTTATGATCTCTGGCCAGTCGTGATTGTTTCGTCTATCGACAaTCAATCGACCACCTTTTCCTTCTCGTATACTTTTATCGGCTTTATCATTAAATCCATAAATTGGGGATAAATCTAAGGcGGGTGTGAcAACTGTtAGCTGTGTCACAGCTCCATTTCGTATAGAACAGCCTCTATCCAAATCAGTAG

>contig00466

aCGAGAGAGTGAGAGAGACGACCCTGTCCAAGGATCTGTGAGCCTTGCTGTTTTAtGAAACAATACTTCTtCCTTTTtGAATACAAATGTAATGACATTATATATtCTCCtGTAATTGTTAATCTTAAGTTAAGGGTTCTAACTTATAAGGATAATGGGAcGTGcACAGACGCGTTTGGACGCGTGTGTGCACGTTCTAACACCTCCAATCTGCGTTGGCAaGGCGTAATCATATTATGAATTAGGTATTTACTATTtAGGTATTAGAGAAAAAAGTAAAAAaTaTTtCAGAAAAaTGTATAATTAAAATaTGAACGTaTCGCGGCATCGAGAGAGAACGCGCAAGTGGCAGAGGGCCGTTTGGAAGTTGAGATAGATGTTCTTCTTGATGGGGATATtCTTTTtGAATGATGGATAGACTCTGTTACTCGCGCATTCCCGCCTTTGCCCAGTGCTGTGTGAAAATGATACGAATATGGCGATAATATTAAcaCCCCcGCATTGT

>contig00467

ACACTtACGATTtCAATGGgAGAAGTAACACTGAACAAaCTATAAAAGCCAGTTAAaGTAAAAAAAaaGTTAaTGAAGTGAAaCTCGAGAATAATAATTTTTTtGGCATTAGTATATAGATCTGCGTGTGAAATTTaCAGACTAGATgCATCTAAATTGTATCGGTTTAAAAgtCGAAaCTTGACAACaTTTTTtGGACCTTCTTCTTCTTCTTCTTCTTCTTCTTCTTAAATTACAAAAaTGCAAAAaGGAAAAAAaGCgAAaCACAAAAGTATTAGTGGTTTGGACTT

>contig00468

CTAAAGAGAAAGTGCTACAGTATGATAAAACCGTAAGATAGGGAACTCGTAGATGGAGTATGATATACGAAATATACTCTGTGCCAAATGACCTAAAAAAAAaaaaGtCATTTTtCATCGtATTTTtATGTCGaGACTTtGTGTCAaGAAACTGAAAACGGTTGGTATTTGCTTTCaCTtAAAACTTTTtCCAATtATTTAATTTCTTTCAAATTTTtCTTAGACTTTCGATTTTTtCTTTCAAGATGATAGGAACTTTCATTGACACTaTTTAAAAATATTATACAATATACGGAAAATTTATATGAATAATATTTTTtCTTTGGGGTTTtCGTGTTTGCTGTTTGTCCAATTTGAAaTTGAACTTATCCCAaGTAAATGTGAATGTGTCAGAATTCTTTTGAAACTGATATGATGAAaCCCATTGAGTTGGCTTTAGATTGTAGTGCGTCCTTTGTGCGTAGT

>contig00469

AATCCTAAAACTATTGTGTTGTCAAGTATTATAAAAaTCGCAATTGAAATGAAACACAATAAATCCCAGGGATATTTTTCATCTGAAGTTGGATAAAAACCACAATTATCCGACGTTGGATAAAAATTATCTGACGTCGCATAATTGTAATTTCCAATTGTTTGCAACGATTATGTCCAATTTCAGATAGAAAATATCTCTAGGATATACTGTGAAAC

>contig00470

ACTCGAGATTGTCATTTtGTAGGTATATGGTTTTTTTTtCtAAGCATTTGTTGCACAAaTTTAAA

>contig00471

ACTTTtCATGCGTCAATAAAAATTAAaCTAAATCcTTACTCGTGAaTGAAGATGATGATTTAAAAGTGAAG

>contig00472

ACCATTTAGTAATCAATCGTCCGATtcGCATAATGTTGGTCTTAATGGTCTTAACTGAaTAGGATCAATTGTTTCACATAAAaGATGCCGAAGAAGTGAAGGGTGCTTCTTGGAACAAGAGATCTCCTAACGACCTCTGATGCAGAAATCCCcTTGAATTTTCGAAGTAGCTGCAGTTTACACATCGAAGAGGATGAAGCGTAAaGAAAaTCTTATTCAGGATCTAATCACGATTTCCTTCAATTTTCGAGAAAAaGAATCCGAAACAATCATGAGAATTGAGATGAATCGACGGATGCTTTTAGAGCGAAATGGTGTCTCATTTtGTaTCAGAATTAATTATGTCCTGAAGACGGATAACCAATCGTCCTACAAATTCACGGAGAATCGGTTGCCTACTTCTTCCAACTTCCATGACTTCTTCGTGATTTGCTTCGCCATGGTCTTCATATTCTATTACACACTGGTTTGTTATGAGACTGAAGGCAAATACTGTCAGGTCACAATGTCTGGCGGTTatCaCTTCAT

>contig00473

ACTTGATTCTGATTTCGCTTCAGTCTCAGGTTtCAACTCCGGCGTTTTCTtATCTTCAGTCTTACTCTCGGGAATAGTAGCTGCTTTTGATTCGGATTCTCCCACTTTCTCGGTTTCAGTCTCTTTTTTtCCTTCTGGGAGATTTTCCTTtGATTTCTCGACGACaGGAGTTTCGACCTTCTGCACAaCAACCTCCTGAGGTTTTGtCGACTTTGTCGGGCTCGTCTACTACCATCTCTTCGTCTTTTttAtCATCAGCTTTTGCGGGCTCTACTTTAGGTTCTGCGCTTGTCGCATCTGTcGACTTTtCTTTGGAATCTTCAACAGAGGATTTTTtCGACTCTTCCGCTGGAACCTCGGTCTTCTTGTCTTCTtCCTTCTGCTCAGCTTCAACTACAACTTTTGATTCTTTTTCTTCAGGCTGTGCAGCAGCCTCtGGTTTTTTGGCAGTTTCAGCCAAAATGTCCTCCGAAGTATCCATTGGCTCACAGGGgCTTTCATCAGTCTTACTATCAGTTTCGCTCTTATCGCTCACTTCAGTCTtACCGTTCAACTCTTTCTTGGcAGTTTCTTCTACTGCTGCATCCTtCTTTtCCTCACATGTGTCCACCTTTTGTCGCTTgCTCGAGGACTGAAAaGATACgTCCTCGAAGGATGTGATCGTGGAGAAGGCGGACTTTTCCTCTTCGCTGAGAGGTGCGTTGCCTTtACGCACGACAATCATTGCTGCTATTCCAGCCTCTTtCGCTGCCTGaGCCTCTTtAaCGaTATCGGTGAGGAAAAGT

>contig00474

TGATTAGCTACTATATATGGGTCAAAAATTAATTTTAATTTAT

>contig00475

AGCTTTAGGATTTATTTTTTtATTTtCTATTGGAGGAATAACTGGTGTAACACTAGCTAATTCATCAATTGATATTATTATACATGATACTTATTATGTTGTTGCTCATTTTCATTATGTTTTATCAATAGGGGCTGTATTTGGGATTATTGGAGGAATAATTCATTGATATCCTTTAATTATAGGTGTATCATTAAATATAAAATGATTAAAAATTCAATTTTATAGAATATTTATTGGAGTAAATTTAACATTTTTCCCACAACATTTTTTAGGATTAAGAGGGATACCTCGACGATATTCTGATTATCCAGATTCTTATTTATTATGAAATTCAATTTCATCTATTGGATCACTAATTTCTATAGTTAGAGTA

>contig00476

AaATAAATATTTGATTAAATTTAAATTTACAAGATTCCTCTTCTCCTTTAATAGAGTGATTAATTAAATTTCATGATTATACTTTAATAATTAATTTAATAACAACATTTATTATTATTTATATAATAATAAATATTTTAGTTAATAAATTTATTAATTTAAATATCAAAAGTCAAATAATTGAAATTATTTGAACATTATTTCCAATTTATATATTAATTTTAATATCTATTCCTTCTTTAAAAATTTTATATTTATCAGATGAAATATATTCTCCTATTATATCAATTAAAAGAATTGGTCACCAATGATTTTGAAGATATGAATTTCCTGATTTTAAAAATGTAAATTTTGAATCTTTTATATTAAGTGAATTAAATAATTTAAATTTATTTCGATTATTAGATGTTGATAATCGATTAATTTTACCAATAAATTTACAAATTCGTATATTAATTAGATCTAATGATGTAATTCATTCATTTACAATTCCTT

>contig00477

ACTCTTCGTCGGAACTTCGTCAAAaGaTaTTGTTTtGAGAATTTCGTCGAGGGATCGGTTCCCATCGTCTTAGGTATTGTTACGAATTATATAATTGTCGAACTACAGCGGATATTGTTACCCTATTTTGGATTAATTCAACCGAAAGTTGACGTTTGGCGTCTATAaGTTtATGAGACGTCATTTtGTACAAaTATAGGTGCACATCTGCTCAAACACTTCAATTCATTTGTGAATTTCGTTGCACACCCAATTTCTTACAATCGTTATACAGAAAACCAAGATATGAGCAAACAAATTCTGATTTTGGTGGTTTCTTCAGTTGTTGCTGCAGTTTATTCTACTGAAAAAAaCGTCATATGTTACTATGCAACTTGGGCCACCTATCGTTCATCTCTTGGAAAaTATGCCATTTCCGACATCGATGCAACTCTTTG

>contig00478

ACTCATTTAATTTATGCTTTtATTGGTGTTTTTGATAACGGCACTGTTCATATTTTAGATCCATGGCTAGATGTAGATAGCAATGGATTCAAAAACTTCGTGGCTCTTCGAGATTTGAATCCCAATATCACAATAATGGCTTCCATGGGAGGATGGAATGAGGGCTCAATGAATTATTCCACGATCGTGACTAATGCAACGTtACGAGCAACATTTGTAGACAaCGTCTACGCGTTTCTCAAGAAATATGACTtCAAtGGTTTTGATCTCGATTGGGAATATCCTGGCAAACGCGGAGGACTTTCTTCTAACAAGCGAGATTTGATTtACCTTTTGAAAGAaCTGAAAAAAAAaTtGAAGAAaGATAATCTCCTCCTATCTATTGTCGTTGCTTCTACTGAATCATCTGCTGATATATCTTACAATATTTGTGAAATTGCAAAAAATGTCGATTTtATAAATTTGATGACCTACGATTATCACAGTCCGGCTGATGGTGTTGTTGGTCATAATACaCcaCTTTaTGCTGCTAAAaCCGAAaCTGGaGaGAaGGGAAAaTTGAaTaTaGATACTTCCGTGAaGT

>contig00479

ACTcTTGACGGTTGACCACTTTGATCAAtaTTTtGTGTTTAATTATTGAGGAAGTTCAATCATTTGTATGCAGAATGGTAGTATTCACGTGCAACAACTGCGGCGACTCTTTGCagAAGCCAAAGGTTGCTAAACATTATCAATTTAGTTGCCGACATAATCCTATCGATCTCACGTGCGTTGATTGTCACAAGGATTTCCGGGGAGAGGAATATGTTAAaCACACAAAATGTATAACCGAGGATGAACGATATGGTGGAAAaGGTTTTaCCCCcAAAGCGAGTGCCAATAAAGGAGAACGAAAGCAGCAAGAATGGATAAaCGTCGTTCaGAACTTACTCACTACTTCGACGAACTTGACATCTGCCGAACGAAATATATT

>contig00480

TTgGAAGTTTCTCTGGcAAAAaG

>contig00481

TCAGAAGTGGCCGGAAGTCGGACGGAGCGGAGAGAGTTTTTtGAAGAGTCTGATCCGCaCTCCGATCTTAAGCAACGAATTTCCTCCACCGCCGCCTGCGCCAGAAACTGTTGAACTTATCGAAGACGAAGAAAaGTTtGTCGATTGGAGCTCTTCGGTATGGGCCACTCCGCCAAAGTATGCGTATTCAGAGAGCGAGCAACTCACAAACGTCGAGGT

>contig00482

ACCTGCTATGACTCTGCCAGCGTGGAATCAAAACACAACAATTGATGATGCTTCGGATCTTTTGTCGCCGACGAAGAATCTATTGCCTGCTTTAGCAAATCCACTGAATCCGAGAaTTTCTCCTtACTTTGCTGgAAaCCGTAACAtGGTtGTTGCTCCACATCCctCTGCACTACCGCTACCTtCTGTTtCTTTAACGGCACGAAAAGACCGAtCTGAACGGaTAAGTCCTACCAGTtCcGCGAGTGGAAGTTtgAaTgAACACGAAACTGCAGACAGGGTCGACACAACTCCTACTAAACGCTCATTCTCTGGAAAGCGTCGGTTGGCTGCCCAATTCGATCAGCCCATCGACTCCTGAACGAAGCACTTCCTGGAAAAACTTTCAAGCACAAAAAAGCGGACTAGATTCCAAAATCTAGTCCCACCTTGAATTTTATTGCGAAGAAaTAAAAGTCAAATACATAATATGTAAGATCTCGGTATGCCGCTGCGCGCTAGGCCAAATACCTAAACGAGCGTTGATCGCTCGAATAGGTATTTTCCTAATTTCATTtAATCTTGAACATTTCTATCGATTAAAATTtCACGAGCGAGATACATGCTAGAATGCAAAACTCTTTGATTGTGAAATGTATGAGCGGCATGAAATACCGAGTGTATCGATGATTCCATATTGGGATTGT

>contig00483

ACTGTCCAAACTGTGATAACAAGTATGGAGCATCATAATCAATTTCATCTCTCCTGAA

>contig00484

ATCAAAATCGTGCGATTCGAACCGATC

>contig00485

TAAGTATtATCAGTGTCGACTaGGAAAAAAAaGTCTCTCTCGATGCTTCCGTTTCCTTTGAATtATTTtCACAAaTACCAACAAATTATCATGAAGAAAATGATTGCGAACCTCTTGGgTAGTTGAGAGAGACGAACGTTGTTGTTTTTATtCTGGGAAGCATTTTCAGTGTTTAACGTGTGCGCTAAGTGATTGTGTGATTATACAGAaCTTGCAGAAGACCGTTTTTACTTGAAATTGAGATGACCAGGGgTTTtAGAGGAAAGACAAGGAAATTGCGAATCACGTTTCTATAGGTTTGTCGTTTTTAAGTCCcAAGTCCAAATTCGCGAAGACGTTATTAATATCACGCGCTGGAAATAAGCTTTTTTtCATGCTCAGTGCGAGGTGCATGCCGGCGATCTTAATGATCTCAGA

>contig00486

CCTCGTGTGTATCAGCTCATCATCTTATCTTCCCACGACAGATGTGTTTAATCGAAGTGCGTTTAGATTGGGAATTTAAAAATGGCAGTATTTtGTAAACTATTTAGAATTACAAACAAATGTGAATACCTGAAGAAAaCCGTGATTtCGAACGCGAACGCGGGTTTACGAAATTTTTGCACAGTTGTGGAACAACCAAGATCAGAAGGTTC

>contig00487

aCGTTTTTAGCTTACAAGACTTCAATGGTCGGGAACTGGAAGGAAAAGAATTAGAAGAGATTATTTCTACCTTCCCTCCTCTGAAGCCACCCAACCAAATTCTTCCTTTAAGAACACCGCATCGTCGTTCATTAAGAAATATTAAAaCaTGAGAATCCAAGACTCAATTTAGCTTTtCTGGCTAATTAGCCAGAGAAAATTTCGTAAAGATTGAaTGGAAACGAAaGGCAGAAGGgTTATGATGTTCTAGTATTCtAGTCAACTGGAAACGAGTTCATCTTCCATTTTTAGAAATAGTATTtCGTTtGCTCATTCCATTACCAAGTAAGGATTTAGGAAATGATTGCATTATATCGATTTTtATATCAATTTACTTGTCAAgAAGAAAGTTTTTGATACACTTTGCTTTGCGATGCCTTTtGGTGATTGTTATGTATTCTTAAGGaGATCCATGACGTCTGTGTTCTTCAAATATTGATCAAAGTATCGTTAGTCGAAAATaTTAGATAAGTTTATACAATTTAGAGATACTTAATTATGCTTTTATTCAACTAAATGAATGAATACAGATCATTTATATTTTACGTTTAGTTATTAATTTTGCTTCTTatAAAGT

>contig00488

ACTTTATTATTATTTTACATTATACaTTAAATGTTtACTCTAGACAATTTATACTATATTACACAATCTTGCATGCAATTATATAACTTATACACATATACATTTTTtAATAAATATtATTTTTtAAGAAGGGCCTGAAAGTTtAGCTTCGCTTAAAACGTTATtATAATAATAAAATCCTATTATAATAGTAAAACGATGtAATAATATCTTTACAGATGAGACGTAATGCTTTAGCGTCGGCTTGTTTGGCACGCTTCACTACATTTCGTACTAATTGTTcaTTACTTTTCTGCAGCCGCCATTTTCTTTTTtAAtGTTTTAGCATCGGCGTGTTTGATCAGCTTCACTACAtCTTTtCCTAATTTATGATTACCAACTGCCAATACTTTAGGATCCATCACGTTGAATTCTCCACCATTGGTATCAATTACTGTTCCACCAGCCTCTCTGATGATAagTAcTCCCGCTGCTACATCCCAAGGATATAAATTATCGGAATGATAAGCCTCGGTTGCGCCCATTGCCACATAACATAAAGTTAAaGCTGCcGAaCCTAGTGTCCTGATTCCATGAGCAATtGATACGAAAGCTTCAAGCCTACCAAGAATTGTATCCCTCAtATTTTCTATGGTtGCATACGAAGCCTCAaTaCAAAGGAGAGATTGCGTGAGATCCTCGACTTTtGAAGTTTCAAtACGTTTtCCaTTTAAAAAAgCTCCGCGGCCTCGTCTTGCCGTgAACAATTGTTCTAATAGTGGATTGTAGACGATGCCAATTTGGAGTTCCTTGTTGACAGCAAGTGCCACGGAAATGCAGGTGTGTGGAAATGAGTGAACAAAGTTTGTGGTTCCATCGATTGGATCAATTATCCACGTGGGCGTATCAGTCAGTTCAGGCATGAAATTTGTCTCGCAAACCGTTTCTTCGCCAATAAATTTGTGATCGGGAAATTCGTTTGATAATCCTGTTATAAGGATGTGTTCTACTTGTTTGTCATACACTGTCACTAAGTCCCAATTTCCTGCCTTTGTGTCAGTAAGTTTACCACCTTGGAATGCATTTCGAATCACCTTTGCGGATTCGATGATTAATTTCAAAGCAAAGTCATAACAGTGATTCAGTTCCTGCACAGAGGTCATGGTCTTTAGAGTGTTGAAGATTTTTGAAAATAAATAAAAGGAAATCTAGCGCTTGAACTTGATAGCAAGGATCCTTGATCGCAGCCGAGGAAAATTACTGTTTTGGAATAACGACTCGTTTAATAGATAAAATGAAAAACTCCTTCAATCGCAGAAAACCTGGAACTTTATAAACTATAAACTGGATGAAAATTCTTTCGCTGGTGTAGATCAAACCCGTCCTCGACTTTTCGGCACACAAGAAGaCT

>contig00489

TTTTAAATGCGAGGTCATTCGCAGTCTCAAAACATTGCAGCTTCAAGACAGCGGaCTAGTGAAGCGAATCAGAGGTGTAGCCTGCAGTTTAAGAGTCTCTCCCGCTATTTCAAATCGAATAGTCGAAGCTGCCAAaGGAGTATGCCTTAATTTCATCCCAGATGTCTACATTCACACAGATGCAAGTAGAGGGGCTTCAAGTGGAAAATCTCCAGGATTTGGAATATGTCTAACGGCCGAAACAACGACCGGAGTTT

>contig00490

ATTTTTAATTTTTATAATTTAAAATTAAATAAAATAATAATAAAATTTTTAAATAATTTTAGTAATAAATATAAAaTAATTATTTtAATAATAATTTTTAGTATTGTAAAaGAAATCTATTTtATTAAAAAAAAGATAAATTAATTTTTtATACCTTTtGTATCAGGGTTAATTAAAAATTTTTAATTAAATTAAAATTTTCGAAATTAATTGAGCTAAAATTTAATTTTAATTAATATTTTAAACTTATTAAAAaTTAAATTTTAGAAATTATATATTATTCATAATTAATAATATCTAATTTTTTAATAAATGAATTAAATTCATTAAATATTTTTTTtATAAATATTTTTATATATAATAATAAATAATTTTTtAAAAAaTTTtAAGGGATAAACTTTAAAa

>contig00491

ATTTATTAAAATATTTAATTTAAAATATAATTTAAAATTTtAATTTTTtAAAAATATTTAATAATTTATAATTATAATTAAAaTAATTTAAAATTTtAATTTTTAATATTATTATTAAAATATAATTAAAAATTTAATTTAATTAAAAATAATGATTAAATTAGTAAATTTTTCAAAATTATTATTATTTATTATAAATAAATATAAATATTAAATTAATGAACTAAGCAAATAATAATATTTACCTGTTTATTAAAAACATGTCTTTTAAAATTATTAATTAAAAGTCTAATCTGCTCAATGAATAAAAAATTATTATTATTAAATAGCTGCAGTATTTTAACTGTACTAAGGTAGCATAATAATTTGTCTTTTAATTTAAGACTAGAATGAAAGATTTAATAAAATATTATCTTTATTAATTTAATTAAAAaTTAAAATTTATTTTTAAGTAAAAAaTCTTAAATATAATTATTAGACGATAAGACCCTATAGAACTTTATAATTATTAAATTTAAATTTAAAAaTTTTATATAAATTATAAATTTATTAATTATTAAATTGGGGATATTAAAAAaTTTtATAAAaTTTTTtATAAAATTTTACATTTATAAATGAAAAAAAATaGATTAGAAAAGAGTGTTAATTTAAAAATTAATAGAATAAGTTACCTTAGGGATAACAGCATAATTTTTTtAAAAAGATCTTATTGATAAAAAAGATTATGACCTCGATGTTGAATTAAGATAAATTATAAAAGAAGAATTTTATAAATTAAGTCTGTTCGACTTTTAAAATCTTACATGATTTGAGTTAAAATCGGTGTGAGCCAGATTGGTTTTtATCTATAATTAAACTTTAAATATTTGTACGAAAGGACTTTATTTAATAAATAATAAAAATTAATTAAAaT

>contig00492

TTTGGTTTTCGATTCTACCATGGTTCACTGCTGAGGTCGGCCACACTGAACCTTAGACTCGATCTAACTTACATCAGACATAGACTAATACGTAATTAAGCTTTTAATAATAAAATTATTCTTGAATAAGGCACTTCTATCCTTTGGTTGGTCAAAACTGGAAAGGATCTGCCATCCGACGATATAAAGAAAAAAAaTTATACATTCCATAATAAATTCGATGAACTTCAATAAGCGGATA

>contig00493

GCAGGGGGTCAGATTCTTTAAGTTTATAATTTGAATGaTAAGTTGCGCATCATCTTCATAAGTcACCTCCGCCTCTATAGTCTATCTGGAATTAAAACGAAATaCCCTTAaCaCAACTTTCAAACAGAaTGCATTGGTtATTTCAGTTGCATGGGT

>contig00494

ACCTCAACTAAatATCTAAATATTATGTATATATATTaGGTGCGACTCTCCGCATGAGCCATACAAAaaTAACAGTCGTGAATCATTtGGCGACCTTACGCCCTCTTCCTTTCTTTCCTTCTTCTTCTTCTTCTTCTTCTTCTTCTTCTTCTTCTACTCATTACTCGAGTTtGTGTGtGTTTTGTGtGTGTATGTGTCtGAGAGAGAAAGAGAGAGAAATATAATCATGCAGTATTCACTTTtCGTTTtCGTTTGCTTTCATTAGATTCATTTtCCCTGTAAGTCGAGTGCCACAAGCAGAaCTAGGGCTTTGATTCGCGCGGACGACTTTCTGAAATGTAAAAaGAGGCAAGTATTCACGTCACAAAAaCCACCTACTACTACTCCAAGTCACACTTtAATGCGTCCGGCGTTAAATGCAATTATTCATTTtCCTTCAATCACAaTAAAaCACAATTTCCTCCTCCTCCTTCTCCTTTTTCGGCATTACTTTACCTCTTTTTTGAGATTCTCAGCTCTCCTCGACTCGTGGCACTCGACCATCATCCAGGAAAaTCGGCAGTGTGAAGAACGGCTCGGCGCGTCCCGTTCATAAATCAAAAACCTTCTAGTAAATGTATAAAaGTAAAACAATTCATTTCCTCTTAACTCATCATAACGCGGTCATCGTCTTAAATAAATAAAGTTTAGAATTCAAGAGATAATGACGTATTGTCCTAGTCTACTGGCGACAGGATGCCCATTCCCATTGTTTTTtG

>contig00495

ACGtCGCTCCAAATCGAGTGCTTCTTCTTCTTCTTCTTCTTCGTTTCGTTTGTCTTCAAAGAGTTTTTAGAGGCAATAATCAGGCACAATCTATTCAACTCCAGAGAGAAAAAaTTCCATAATGCTTCTTCACAAAATGACAAAaGCATCGCACaAGACTAAACGTTCCTCTCTCGATTAACTCGATAATATTCGAGGCACGCTTTTTCAATCGTTTATTATAATGTTAATCTACAAGCACTCGACCAAAAAAAATTGGCTACTTACAGTAACAACTTTTATGCTAATTATACGCGCAGGACTTGGTGCACCAGTAGAATTACCACTTGGCCCGAGCGTGGTAAAAAAAAAAaTAATTAACCCcGAGCCGTTTCATCCCCcACTATTATAGACAACAACAATTTtACGGACC

>contig00496

GCCtGTGAAAATTTTCGATAAAATTGATAACGACGATGAAGGATTTGGGCAGTGAAgCTggAAGtGATCAGGAAGTAACTGAAGGTCATTCAAAACTCATCGAGGTAGTCAGGGAACTTGATAAAGGCCAAAGAGTCAAGAAGCCAGAACGAAGTGAaCCTAGCCTGACGATTTCAGAATTCCATTTAGTAAAAAGTGGAGTGACTGATAACGATGTAATACAAGTCTCCGACTTAGTAAAGACTTTAGGTCAGAAAGGACGCCACACGGAAATTGAAAAGCAGCTTAA

>contig00497

CTACTGAAAGGATCAAACGAGTAGTT

>contig00498

ACTTACACCACTAGGAGAACAAATAaCTAACCACCAGTCCCcGATTATTTACAATAATTAGCTAAaCAAGAAAAAAaTTGCAAAAaTaTCCGCGTTTACGATAATTATTTTAAAaGAAATTGTGTGTGTGAGAGCGAGATGCGAGACGAGATTTGTATGaGTGACCTTTGTATTTTAACATATTGATAGTAAACAATGTAAAGTGGCATAGGACAGAGGAAACTGAGAAGCGATTCGATGGTGTCCGATTCTTCtCTCGGAAAACGCAGGGgCTGATTTTTTtGCTCTCCGCTCTAGCTTTCAATTTTTCTCGTCTCTCGaCCCTCGATTtAATATTTTTtCTTTtAATtCTTTTtATTTTTAATtACaCGtAAAAATCGAGTGTCGAGCGACTCGTgTAAGTTACGTTGAAAaTtGGAAGGGAATTTCCGTtaGTCGGCCCATTCGGTGGCAGTGAACATGGAATCATGTGTATCGATAAaTAATCCTTGT

>contig00499

ACCtGTAAGTATTAGTTTACTAGGAGTTAAAaTGGCGCTGCGAaTCTTTTTCGCAATCATCTCTGCTTTCTGATTTCAAACGCGGATTaCTCACcTCCTATCCCGAGGCTTCAGAGATCCTGTCTGCCTTTGTTTtAATTTtGATTATtGTAGAGCAGATACTCTATTtATtATTCAATCCAAGTTTTtACAGTTtACTAAGTAAACTTTtAACTGATTTtGGCCCGAAAGCCTCGAGGTTTGGATCCCAATCATGTTTCGCgATCAATAAATGTTTTTTTTtAAACATtCAAGTTCTTTTtCCGGAATTTTGAACAATTTAGCGACGAAACGAATAATTGTAATTAACGATGCGCGGTCGCAATTAAATGGACGCAAAACATGTAATTTTTGTTTtGTATTGCAATATAAAAaTAAGTGACTAATGCGAAGTCGAGGATTGTCAAACATCTACCAAAATGTGAACCACGTTTTTCTGTATTTGGTATTTCTCCAGTTAATGtAAAAATTGTGTATTC

>contig00500

GgAGGAAAAAAAaCCACCTACAATCGCAGCTCCGTCCTGCGAATTAGGACCAAACCCTAGctGTCCTCCTAGTTGTCATGATACTTGTCCAGCCTTtAACACACCAGAaGGAGGTGGTGATCAGGGCAACAaTCCGTTtAAaTATTGGAAGgAaGTTCTGCTTGCTTTATTGCTCACGGGTGTAGCCATTGTCACGGTATCGCAaTTaTCACCCGATAAATTGGAATCtCCGAAGAAAGTGaaaaagaaGGATACAAAGGGTGAAAAGAAGAGGCGAGAAGTAGTGAAAAGCCCTCTAGATTCTAGCCTTATTCCATCAGAAGTCCCTTATTTGTTAATAGGAGGAGGTCCCGCCTCATTTTCAGCATTTAGGTCAATTAAGTCTAGAGATCCCAAaGCCAAGGTTTTAATCGTAACGGAGGAGGGTTACAATCCCTACATGAAACCTCCCTTGTCCAAaGAaGTTTGG

>contig00501

TCGAAACGAAGAGAAGCTGTAATGGAGGACACAATTTGGCCAATCAGTCTGTTCAGATTGGTGTAGGTGGGTCTTTCAATGTCCAaGTTGCGTCTGCAGATATCGTAGATAGCTTCGTTGTCGACCATGAAAGCACAGTCaGAATgTTCCAAAGTCGTGTGAGTTGTCAGAATAGAATTGTAGGGTTCGACAACTGCTGTGGAGACCTGTGGaGCTGGGTAGATGGCAAACTCCAACTTTGATTTCTTTCCATAATCAACTGAAAGGCGTtCCATCAAAAGGGATGTGAAACCAGAACCAGTGCCGCCGCCGAAGGAATGGAAGATCAAGAATCCTTGtaGTCCAG

>contig00502

ACCAATGCAAGAAAGCTTTCCTCCTGAACATcGCCGTAAaCTGTTCAGAGATACgCTTGAACAATTCCTGGATGGCGGTCGAGTTTCCAATAAAGGTGGCAGACATCTTGAGTCCACGAGGTGGAATGTCACAGACGGCCGTCTtGACATTGTTTGGAATCCATTCCACAAAGTAGGAGCTGTTCTTATTCTGAATATTGAGCATCTGCTCGTCAACCTCCTTCATCGACATCCTTCCTCGGAAGATGGCAGCCACCGTCAGGtACcTtCcGTGTCGTGGGTCACATGCCGCCATCATGTTCTTGGCGTCAAACATCTGTTGTGTCAATTCCGGAACCGAGAGTGCTCGGTATTGCTGGCTGCCACGAGACGTCAGGGgTGCAAATCCGGGCATGAAGAAATGGAGACGGGGGAAGGGTACCATGTTGACGGCGAGTTTTCGCAGATCAGCATTGAGCTGACCAGGGAACCTCAAACAGGTCGTCACTCCGGACATGGTCAGGGATACGAGATGGTTAAGGTCGCCGTATGTGGGTGTGGAAAGTTTAAGTGTGCGGAAGCAAATgTCATAGAGAGCTTCGTTGTCGATACAaTAGGTCTCGTCTGTGTTTTCAACGAGCTGGTGAACTGAGAGAGTGGCGTTATAGGGTTCAACGACAGTGTCTGATACTTTTGGCGACGGtACCACTGAGTATGTATTCATGATTCTGTCGGGATACTCCTCACGAATCTTGGAAATGAGCAGAGTGCCCATACCGGATCCGGTACCACCTCCAAGTGAGTGGGTCAGCTGGAATCCCTGCAAGCAGTCGCAGCTTTCAGCTTCTTTCCTGACAACGTCCAAGACAGAGTCGACTAGCTCGGCTCCCTCGGTGTAGTGTCCCTTGGCCCAGTTATTACCAGCTCCACTCTGGCCAAACACAAAGTTGTCGGGTCGGAAGATTTGACCGAAGGGGCCCGATCGTACAGAATCCATGGTTCCGGGCTCCAAGTCGACGAGGATAGCACGGGGTACGTATTTTCCTCCAGATGCCTCATTGTAGTAGACGTTGATTCTTTCCAGCTGGAGGTCCGAGTCTCCATGGTAGGCACCAGTCGGGTCAATGCCATGCTCGTCACTGATGATTTCCCAAAACTTTGCTCCAATTTGGTTTCCGCACTGACCGGCTTGGAGGTGAACGATTTCCCTCATCTTGCTTGCTGCGTTGAACTCTTTTTTtATTAGTTAATCGACAAAAACCGAAGTGTATAAAACTTGGTACGTTACACTTCACGTAAGTCAAGCAACCACAAGCAACTgaCCAcTG

>contig00503

AGTTTAGTGCAGTTTAGTGCTTGTGTTATCCTGCGTCTCTAGTTACTTTtCTCAATCTTCTTTGCAATTTGCAGTTGTATATCTCCGAGGGATATATTTtGTTTGTCGTATGTCCATCCAGAATCTCAACACATTCGACCCCTTTGCAGATGCAATCAAGGGTTCAGATGATGATGTCCAAGACGGTCTCGTGCATATAAGGATTCAACAACGGAATGGTCGCAAGACCTTAACAACGGTGCAAGGGCTCTCTTCTGAATATGACTTGAAGAAAATTGTACGAGCATGTAAAAAGGAGTTTGCCTGCAATGGGACTGTAATCGAGCACCCTGAGTACGGGgAGGTGTTGCAGCTGCAGGGGGACCAGCGTGAAAaTATATGCCAGTGGCTAACCAAATCAGGTCTGGCAAAaCCTGACCAGCTCAAGGTTCACGGTTTTTAATGTGCAAACCAACCATCCATGCCACTATCTAATATAACGACTCCACCTTCTTCTCCATCACCTTTTCCAT

>contig00504

ACGCATGTCGTTGTCGTTCTAATCCGAAATTCGGTCGGTAAGATATAGAAGAACGATCGAAGGAAAGAAAGCAATATTTTACAACAGCCTTGTGATTTAAATATGTTCTGCTCGCTTTTATCACAATTAAAGTAATAGCGTTAAATAATAGAATTTTGCGAAGATATAGACATAGATGAGATAGATACAGGAAGAAGCAACGGATGGATCTTGATTTTGACATTAGATAGTATGTATCTGGGAGCCATAGACGTGTTCAGAGAGAGGAGATTTGAGAGAGATATATTCATTCTCCTAGGGTTCTGATTGGCTTCTCATTTATAACTACAGCTTGTTTCAATTTtCCTATATACAGTAAGGACCATACTTTGCGCTTTCAAGACGTCATCACTTTTTTTtGTTtCAACTTTTGTGCCTTTTTATTCCACTGATTGTATTAGTTGTAAATGACGTTGGAAATATGTAACAAGGTGGGTGGTGCGCCTCTTTCGGCCGGCCTACTTGTTTCAATTCTTTCTAAAGCAGTATTGGTA

>contig00505

AAAGTGAGAATTCGTTTATATCATAATGAAACTTGTTAGATAAAAAAaTGCTTTATGAAAAAAAAAaTTTtCATTCATGGATAATAATAACAGGTGCATGGAAAAAATTATAATAAGTCTTCTAAAGCAAGTAAAAGAATAATAATAAAAaTAAATTTGCGTATTAGATTTTTTtAAATAAGTAaTTAATAACAGGCGCACAAAAATAATTTCTCGAAAGCAGAAGTTAAATACCTTCTGGTACACCTGTTGTGCTGGGCCGAATATTATGTATTAGTTTGGCATGCTTGCTGCGCTGTTCAGCTAAGT

>contig00506

ACCCcAGGCTCTTGCTGGCAGAACTTgTTTGGGGCGtCCGTTGCGACAAAATCGGTATAACTGCGCCAAGCCGATGCGCAACTGGTGCCcAGCAGGTGGCGCTGTTGCTGtGCCAAAaTTCTTtCCATAAaCCAGTAATCCGTGTAATAATTAGTAGAcAATTTCATTTAACCAaTAagCCAACATTAATCAAGgaCGATTTTAAATGATCAAAATtACTGTTGATCAATATTAATATTTtCTtCGTCTTATTTCACACTTGGGAAGAGGTtCGTCAAAAaCGAGTTGATATTTGAGAGCGTCATCAGTATCCcACAAAAAaTAATCGAGCTGGGTCACCGTAACCTGGAGgTTTTCTATGAAGAATGCACGCACTATTAGTTCGTTCCCATCTGGTTCTAATGCGCAAACCATTATACTTCCGTCTAGTAGTCCATGAGTTGATGCATTTtGCGGATTATTTAGCAGTAAGATTCCATTCTTAGTATAATAATAGATATTTTTtGTAGTTGTCAGATCCAAATAGCCTtCTTTCAGAAGAGTTGTATTGT

>contig00507

ACTTCGGTGCATtCGTAAAaCTTATCTAGATCAGATTAATAaCGTTAAAAAaCCATTTTCATTGACGAGATAaTATTCATGGACAATACtAATTTTTtCTTtCTTATTTTtGTTTtGTTTTAGATCAATTTtAACCcTTGACTGGTCATGTGCTGCTACTCGACAATATGTCGATTAGCATaTGGTACTATTCCTGTTGAGGGATAATATTGGCGTTAGTCATAGTTATGGTACTTATCCAAGCAATCCAAGTTCCAGTCTTGATTTTATTAAAGTGAAAAaGTTTTtATTTTTATGAACGAGGCGATTGCATTTATGTGAAACTTAAATTAAAAAAAAaGAaGTTAGATTTTTGAGTCTGGCAGGCGAACATCGCAAAGATCATCATAACCTATATAGGAAAAGATTCATCTGTGTGAAATAAAGATGTTAGTTCAACGTATCAAAGTTGTTTAGGtACAAACATTTTGACTGGTTCAATCTACGTAGATGAGTTTATAGAGTTAATCGAATCTCACAACAACTGCCGT

>contig00508

ACATGGgATGAAAAtATtAAATAAAAAaTTAAATTtAaTAAAAAAaTAAAAAAAaTaTTTTTTtATTGCCTACTCCGATTAATATTAATATTTtATGAAATTTtGGATCAATTTTAGGAATTTGTTTAATAATTCAAATTATTTCAGGATTATTTTTATCTATACATTATACTTCACATATCAATAATGCATTTAATAGAATTATTCATATTATACATAATATTAATTATGGTTGACTTATACGATTAATTCATATAAATGGGGCATCTTTTTTTTTTTttCTATATTTATTCATATAGGACGAGGgTTTTATTTTAATTCTTATTTTTTATATTTAACTTGATATAGAGGGAGATTAATTTTTATCTTTACTATAGCTACTGCTTTTTTAGGATATGTATTACCTTGAGGTCAAATATCATTTTGAGGGGCTACTGTAATTACAAATTTAATATCAGCTATTCCTTTAATTGGAAATGATATTGTAATTTGATTATGAGGAGGATTTAGTATTAATAATGCAACATTAAATCGGTTTtATTCATTACATTTTATTTTACCTTTtATTATTTtAATAATAATTTtAATTCATTTAATAACTTTACATTTAACAGGGTCAAATAATCCTTTAGGT

>contig00509

ACAAATAGAAATTTATATAAAATTTATTTTCATTCTTATTTTACAATTAAAGATATTCAAGGATTTTTATTAATAATTATAATAATATTATTATTATGTTGTTTTTTtCCGTATATTTTAAGAGACCCAGAAAaTTTTAATATAGCTAATCCAATAATAACTCCTATTCATATTCAACCTGAATGATATTTTTTATTTGCATATGCAATTTTACGATCTATTCCTAATAAATTAGGAGGAGTTATTGCTTTATTAATAGCTGTAATAATTATTTtAATTATGCCTTTATATAATATAAATAAATTTCAAAGATCTATGTATTATCCATTTAATCAAATTAGATTTtGAATATTTATTAATATTATAATTTtATTAACATGAATTGGAATAAAaCCTATTGAGCACCCATTTATAATAATTGGACAAaTTTTAACAaTTTtAtATTTTTtATATTATTtAATTAATCCAATAaTTTTtAATTTATGAAAT

>contig00510

CGAATCATGtCCTTTTACGATtGAC

>contig00511

ATGATGAGACTAtGAGGCTTGGAGTTCTGGGTAGTTCAACTGATAAATTAGAGATCTTTGACATAAAGATGAATCTGActCTGAATCTCAAAACTGTATTGACCATCAAGAACGAAGGGACTGGTGTTCTGGCCTCAAGGCCCGACTCAAAAGTATTTACAACCGGAGGTTGGGATGGTCGACTACGCATCTTCTCCTGGAGATCACTTAGACCCCTCGCAGTTCTGGATCAGCACCGTGGAAGTATTTATGATATTGCATATTCGGGGTGCAAAGTCGAGGCTtACaGCAGCAAGGGTCTAATGGCTGCAACTGGTAAAGATGGaGTTGTGTCTCTTTGGGA

>contig00512

ACGTGtAATGTGCGTGAAaTCGAATCGCCAGTtATCTGATGGTCCTtAAAAAAAaGCAGAGCCTAACGTCAAaCTTtGGAgCGTtCAGAaTTGTTGACCAGTTGCTTCGATCATCGACTTCCATAATATATCGGTTCGTAACAATCTGTTCAAAACAAAATAAACCAAGAAaCAAAAGTGTGCGTTTtCTaCTTGAAAAAAaaTAaTAaTAATAAAAGaCTTTCTGGTGATTAAGATAGGTTACTCGTTgATTTtGAACTTAGAGTTCCGTGTCTTTtGAGTAAATTATATGGCATgCATATAACAaCTAAATCTAAaCATTTGACGGGTGTTTTTTtCCGGGAAACTTTAATCGACATGCTCCTTTCCGAAAAAATCTTGCGTTGTGTTACAAAaTTtCAAGGCCGATCCCTTGGGTTCATATATCATAACCCTGTTCTCGATTTTAGGAATTTCTTCCTGGACATTATTCGCTTTtAGTGAATCGTCAACGACCACTGACTTTTTGGTAGTTTCCATTGATGAAGAAACTGATGAGGATTTTAAATCGGAATACAAGATTTTTGAGTCGGgATCCGATTTTTtAAAaTTAAAaTCTGAGCTTTGTGATTCAGAATCCGAAGCTTTCGAATGCATATCCGACGACTTCAAATCAGAATCTGAGGCCTGGAAAGCAGAAActGAATCTTCCTTGCTAAAACCTACATTTACTTGGGTCTTTGAATGAGAATCTAAAACCTTTAGATCAGAaTCTAGTACCGATTTCGTATTATTGGGAAGCTTAGAAACAGGATTTCGATCAGAATCCAATATTTtGTGGGTGGCATCTAAAATATCTTTGCCAGAATCTaCCAcTACTGTCTTtGCTACTTCAGAAAAAaGTTTCGGAAGAGAATTCAAGACTTTTGACTCTGATTTCACTACTGTTGGAGCAGAGTCCGAAATcTtGAGATTAGAACCTAAATCTTTtGTATCAgAAACTAAATCTTTtGGAACAGACACTAAGTCTTTTAGATCAGAAACCAAATATTTTGGTTCAGAAACTAAATCTTTTGGATCAGAAACTAAATcTTTTGAATCAGAAaCTAAATCTTTtGAATCAGAAACTAAATCTTTTGGATCAGAAaCTAAaTCTTTtGTGTCTGCCTTGGATTCCGAGCTTTtCAaGTTAGGATAGTCAGGACCCTGAATATTATTATTATCGTCGCTGTTTCCAGTCGAGGAAGT

>contig00513

aCCGtAGATTtAAaGGTAAAGCTGTATTATTCTGCTGGATGTTtGGAAACATATTTAGGAATAGACTACACCGATAAATtGAAGGAGACAACTGaTCAAAATGtCAAAGCTGATGATGTTTTACAAATGCTGTCTAATTTtCTTTCATCACCAGTTCATTATAGTCTGGACTCATTTTTCTCTTCTCTTGTCAAGGATGATaCTTTCATACCGGCGGGTGAATTGATACAAGGACATTATGTGAAAGGTAATAACAGCACAAGAAAaTTTGAAGTTTATAAA

>contig00514

AAAAACATTGTCTGCTTCAAGGTTTCAaTAAAACGATGGCTACAGAAGCAaGAGACAATTTTAAGAtCAATAAGAAACAGGCACGCAGAGTATATGAAATTTtAAGGTtGAAGATAACGAACATTTCAAATGAGGCGGAATATCGTGATTATAGGTTAAATGTGAAGAGAAGACTTAaTATTCCTCTCAAACGAGAACAGCGTGGATTGAAAAAAaTCGAATTATCATCGAAAGTTTTGAATCAAGGAACTGAATTTGGACCAGAATATTACATGGAAATTCTCGAAAAaGAGT

>contig00515

ACCATTTtGTTAGGAAGTTTTTCGAATTCACAACTCCAGAGCATCGGGGTGAGCATTTtCTCCGATCAAGAATCCGGAATCCGCTGAGCGGCTTCGAGTAATAGTGCAGGAAAAGTGCTTCGAGTATGGCAGCAGTCGACTTTTATCAGAACTCCCAACAAGGGGACCAACAGGCTACTGCTGGCATTCCCGGTAAAGATGACGAGAGAAAaTTATTTGTGGGAGGTTTGTCCCGGAACACGACCGAAAATGAACTCCGGGAGCATTTTGGAAAGTTCGGCGAAATCGAGAGCGTCTCCGTGAAAaTCGACCCCTACACCGGTGTGTCCCGAGGATTTGCATTCATGGTCTTCACGAATCCGAAGACCATTGACAAATTATTGGCGTCGGGTGAACATTACATCAACAAACGCAAGGTTGATCCTAAGCGAGTAAGTAAGAAAGCACAGCATGGCAAGATTTtCGTTGGTGGACTCACCCCTGAAATCACGGACGATGACATCAAGTCCTATTTCGGCCAGT

>contig00516

ACTTCCTGCCTGGTTtCAGGCACATGACTCGCATTGCACCAGGGACAACGAGGCGTTTCCTACGATCATAGGGAGGTGGACAACCTTCGTAGACTTTTAGTCTCCTCAAAGCATCCTTTCCTCGTTGCGTTTTGTGAGGGATCATTCCACGGACTGTTTtCCATAGAATTTTGCTAGGGGCACGGAAGTGGAATGGTCCACGAGCAGGATTGACGTTACATCTTTTACGGAGGAAAGACATAAACTTGAGCTTGTTTCTGAAGAAGTTTCCAGAAATGTTCAACTGTTCACTTCGGACAACAATAATTTTGTTGCCTTCGAGAGTTTTTTtGGCGATGATGGCTGCCAGCCTGCCAAGGAGATGGCCACGGCCATCTATAAGAATTGGCTTTTCACTGAAGCCCGTCATTTTGCCCGACCGGAAAaG

>contig00517

ACTTTTTGGAATTGCTGCCACGCCTTTTTGAAGAGTATGTTTCaGCAATATTTGAGCCGAAGATTTCTTGTGTTTCTTTCCTATTTCGAGAACTACTGGATTTGATAGCAAATCAGGAAGGACATCAGTTTTtCCCAAAAGTTTGACGAAGCCTCTGGACCCCAAGGGCGAGTAAGCAGTCACTGCGATTCCCTTCTTTTtACAAAATTCAACAAGTTCCTTTTGCTGAAAAtAtAGGTG

>contig00518

CACTTTTGTTATTTGGGCAATATTGAAATTGGACaGGCCAATaGCtCGAGTTTTTCCAGCGACGACTTGTTTTTCCATTTCTG

>contig00519

GTAAACAGCTGCACAATCAATGTGTCT

>contig00520

GCACAATCCAAAGCTGTTACGATTTCCTCCTCGGATGCCTGCCAGGTTCCAAATCCCAAAGCAGGCATATTTtGCCCATTAGCGAGTGGAATGTTTAATTTCGAAGCCATTCTTCTCTCACTAG

>contig00521

AGTAGGATAAACCGAGAATTTTTACGGAGCTCTCAAAAGTGCTGACGTCC

>contig00522

aCAATCGCCTATGAAaTTtCGGCGAGAAAAATCCATTTTACaCGGGCAAAAATGAAAGAGTGGCTCTGAAAAATCACAACTACGCCGAGG

>contig00523

CAGTCTCGGAAATGTGAGAGATGGTTTCAAGTGGATTCAGAGAAACGCGTAATTTTTtCCGATACGAGGAAAGAAAACGGTAGTATTAAAACTGGAAAGTTCTGCGTAAAAAAAaTACAAATCTAGGGCATCAAGAATCAAGAAaCCTACTAAAAAAAaTCGCCAAGGGCTGTTTATTCTTTTTTAGGCTTGTTAGCAGCTTTATTTTTGATACTTGTGAAAGCACAAAaGaGAAAaCTTGAAGAGTATTAATATATTGAATGAAAaTGCTTTTGAAAaTTAaTTGTTGATGTAGATTTTCtGAGATTTTCGTCTTTTGtGGTTCAGTCGCCTTGAATCGGAGCGAAGATATgAGAAAGTATTTATTTTGGAAGTGTATCTTAACATTTTTTtGAGAATTCTAGCTCGTTACTTAATTTATATAGAGATTGTC

>contig00524

TAAATAGCTTATCGACGAATAGGtGCGAGGATATCCAGAGCTTTGCTGATATCACCGTCTTTCGTCACCAGAAGTTGTGTCAGCCATCCTCCTTCGTTGGAGAATCCCATTTCCATCATTGTTTCAaTAGCGCGCTGGATTTttGGATTCGAGTGGTATTGAGGCTTGTTAGTTtCTCCTGGCTGTGCAGCTTTATAAATTCTTCCTTCCTCTGGCAATggggACACAAAGAGCAAGCAGCGAAGAAGGAGCAGTTGAGTTGTCGTATAACGTTGAACGTTTTCGAAGCCAATTACCTCACGTGAATAAAaTACGAAATACGTTACTGAACTCTTCAGTATTGTATTTGTGATTGAAGTCGAaTACGAAGAATCAGCAAAAAaTGTCTCTTGTTCCACTGTTGTTCTCTGACTGGTGGGAGGACCTGGAGCGACCACATCGTCTCGTCGATCAGAACTTTGGTCTCGGCCTCCACCGGGACCAGCTTTTGGACCCAGATTTTCTGGACCGCTACGTTACTCCCAGAATCGAAAGAAGATCCAGGAATCCTTCGATGATTTACCTGAGGCCATGGGGAGAATTGCTTCGCAAAcACGAGGGTGGTTCTTCGACTGTCAAGGCAGACAAGGATCAGTTCCAGGTTTCCCTGGATGTCCAGCAGTTCAAGCCCGAGGAGATtAACGTAAAAGTGGTCGACAAATGCGTTGTCGTGGAGGGCAAGCACGAGGAGAAGCAAGACGAGCATGGCTGGATTTCCAGACAATTCGTCAGAAAGT

>contig00525

ACttATCGGGCTTTCGgCGGATAATaGgCATTCaTTCTCTTGAATCACAATGTTGAAATTACTCTACATCGTgTTtAAAAAAAaaTGCTCaCGAAGCTTTCCCACGTATTTCGGAGGTGTTTCGGCGaTCCCGCTCTCGCCTCTCCAACATCCAGGCATTACGCGCTGGATTGTCGAATCATCGAGAGAGTTTATTATCAACAAATCTCGATGTGTTAATTGtGGCAGATTGGGTATCACCACGTTATATATATATATTTtACTCTCATGTTTATTATTTTCTCtAGCTATTAATTTAGACGATAAGTGCTGTATGCTgAGgTcGgACgTTTTTTTTTttAAAGCTATCAATATTCTTTAAACAAGACCAACAGATTGAGAGTGAGTAATATTCCAGT

>contig00526

ACATCCACCATATCCACGAGTTTATATTCATATTATAGGAGATATAATATATCTGGATAGCGTGTGAGAGAGGCTTCTTTCTTTATGAAATATTGTCATTGTTGCTGATAGATATCATTATTTGTTCAATTAAAATGATTATACATTAGTTTGTAGTTTATACAGCGAGTTGGCTATGTCCGATTCTTTGGTAATTCCCAATCGATTAACATGAGAGGCTTCTTGTTAATAAATGCGCTCTTTAGCGTTAACTTAATAACAGACACCATTAAAATGAGAAAATGAAACAAAAAAAaTAGAAAATTATTCGA

>contig00527

AATATGCCAATaGCTCaTTTCCAAGAGGGTGtCTTtCACAGgCAACAATACGATTTTTtGATTCTTCGGTGAGGCCAATTTGCTGGGCACACAGagTGACAGCGGATGCCGGATTATTTGCAGTCATAACGCATCCTACAAGATCAACCAATCGTTGTTGTTGTTCCTCAGgTTTAACTGAAGTTtCGATTTCATGAAtGCCGCAAGCCTGAGCCCTATTTCCTCTGCATTCGGCTGGTCCATGTTGACAAACAAATTCCCAGGGACCAGTTGTATCGGCTTTCGAGTGCGAGGCCTTTCCaTAAGGAATGAAGGaGATTGAGAGATGATTTTTAAGATCTGGATaGGCCGGGACGAGTTGTGTCCGT

>contig00528

CAtACagTTCgTGGCAAATTATTCTCTCTTGGTTAATATCTCATTgTGAAAaTATCACAAATCTGAAAATGAAAGTGTCTCACAGACACTACAAAGAAGAGGATTTCGCCAGTCTTTTGGGGGAAATGAAACAACTGAAATCGTTTTCTTTATGCAACTGTGATAATCTAGACTGGAGTGGTAATTTtCTACTTGAATTGCCATTTGAGACAATTCAGGAGATTaTTttG

>contig00529

AATCGTCAAATAATGCGCTTTGATATCAATTTATTGGTCAaGATGTTACGAAAATGCACAAACCTTCAAACATTTGGCATAGCTGTAGATG

>contig00530

GTATTTAGACTTATCTTATTGTTTTGATTTAACGGATCGTGGAATTTCAGCGATCTGCAATTTGAGTAAACTGACTTTTTTAAGTATAAGAGGAGTGGCAGAACTAAGGAACCCACCACTGGAAAaTTTGACAAGTTTGGTAGTATTAGAGTTGAGTTTTACTTTATATTTACAAGAGGAATGTCTATGCACTTTTTTGAAGAAAGCGCATCAACTCCGGGCACTTGATGTAAGACAAAGTCATTCAGTTA

>contig00531

TAACATTCGAAAaGCGAACAGCGAGCTTGATTTGTATGTCGATAATc

>contig00532

TGAAGTCACAGCTTCTACTGATGAGGATTCCAGCCAATCTGAGGAAGAGACTCTTATGTCTAAAGTCAATCGTTTAGCTTCTGAGGACAGCAACGATGATCCCGATTTTCTTCCCGATGGTTACTGTTCATCTGATGGCACAACGGAGACAAGCTTGGACGATGAGGTTTCACTAACTGAAGCTAAGGAACTTGCTGAtGAAGCTGGTGACGTTCTACCAAACTTgACTAAGTCTTCTGAGGAATTTAAGAaGAGGACAACTCCAGAACCTATTCAGCTCAACTCAAAGGAAGCAATCCGAAATCTAGCGACTTTGGCAACGGCGAAGTGAAGGTGAAGGCAGGGCCCGTTTCGTCTGAGAACGATATCTCAAGGGTTtCCAGGAAAGATGTCACTAATGGGCACGTCGAAGGAAaGAAaTCAGGAAATAGTGGCAAaGTCAATCACAAACaCAGACGTACtAACCATGGTAATCGCAAAAAAAaGTAaTCATAGAAAGTATATTGTTCTTGTCCTCCGGGTGGAAGTGTTTGGAATGAATAATTAATTATTTGATTCAACGGAATGTTCTATTACTTCGATCAAATGAGCCCTCCTTAAAAAGGATGTTATGTGTTAAATATTGTATCTGTGCCATAAAAAAAaTTAATGACTTTTTCGACTGGTTATCGATTATAATATTAAAAATTAAAATAACTGCGCCAAAGACGCGAGGATAAATGATGTTTCTACAATATGTTAAATGTAAAGTCTGTTAGCCTTAGAAATACTTTTAGAACTTAGAGTGAAACTCATCAAATTAATCATTCGACATATTTAACTTGGAGTATAACTTTTAAATTGGAATATAACAATATGATCATTTTCATTTCCATTAGTTCGCAATTCTTCATACTACAAATGTTGACCAAAGTTCGACTATGGAATCGGGCCATTTCGTGTTTAAGTGATGAGTTTTTATGAAAATTTTTGTTGGATTTCCAATTTAATTATAACTGATAGCAAGTCCTTTTTATTGTTAGTGTGTATTTGAATATGTGTTACTAATAAGTTATTTAATCTCGTAAACAGAATAGTGTTGTGGATTCAGTTAGTGTTAATATGTTAaTTAGT

>contig00533

GCTGGTAGTGACCTTTGAGATTTTAACACCGCACTAGCACGTCGCAGAGCCGCCTTCGTAAGATCCACTCGATATTTGTTAGCTGTAAGCAGAGTTTtCAGTTTGTGGAGAGCACGACGTGCACCGTTTTTCATGGTACGCCTTACAATTGCCTTAGCGGGCTTCGTAGCTGACTTTGCCTTTTtGTAAACAACAGTGAATCCTTTCTTGTCAGGAGTGTCTATGATACCGACATTTTTGCGATGAATTAAACCTGAATAGCGGTAACTGCTGAGATTCGTTAAATTGCTCGGTTCAGTAGAAAACGGCTTTTGAATTGTACGCTTCTTCAGCAGAAACGCATTATTGTTTCGAATTATCATCCACGTCAATTGCGACATTTTGGTTAACTTTTTCTAGAAATCAGCAACGTAATTCACCACGTGTCTAGAGTAGAAG

>contig00534

ACAATCGCGCTGGCCAaGTGACTAATATAAGCTTCCTAAATCGCAGGGCAACGCTTTTTtcTCCAGCcGAGTAAGTGCTTTCGAAGCGAGGCACTCTGTAGATATGTGATATTTACTCTTCGCTAATTTCGAGCCCACTCGGCTCACATTTTGAATaCATTCGTCACACAACTTCCAGtCTCTGGATTTCTTTtAAAAAGAAGTTTTTTTTCGCCAGTCAGGAATTCAGGTATCAAAATATAAATTTACAAaTAGAAGAGAGGAAGTTGTGGgACGTTCCTATCGACCAAGCACCATCTATCCGAATTTtCGACCGAAGCGTAAAACATAGATTGTATGATTACAAATACATCTCTTTCTGTCTGCTGATACTTTCGCCGACAATGGTCGCTCTTTACtAGTTACAATCACGCAAAAAAAAAAAAaCAGgCTCTCATGCACGCTAGTCATACTCTCAGCACACGCGCGAAATctCTCGTCATTAATTATCTTACAATACAGGAAAGCATAGATTATCGAGTGGAAGGTTCACATATAAATAGGTGATGGCCCATTTGAGGCTGTCAATAGACGTAGATGGTCTTCAACTGATCCTTGAGTTCCGGTGGAAAGGCACATGTATCTGTTTCCGGTTTCCTATTCACAAGGCGACGCCagCGTCCCCCAAGATtCTTtCGGAaGCCCTTCGCAAAGCCTTGCCTGTCCGATGaTAGTCCG

>contig00535

ACAGTGTATCATCTTTCACGTCTCAAGAAGGCTGATAGTTAATAAAAAaTAAATTAAAAATGTGGCCCATTGTGTTGGGCGCTTTTCTTCTGGCCAGTGGCCTCTCAGCACTTCCGGTTTCTCCTGATGCAGAAATCTTACCCCCATCGGAAGACGTTCTACCGACTTTGGCAACGGAACTGATGCCTCCtCTTCCACCAAAaGAAGATGgaTCaGAAAAAAAaCCAGTGCTGTTTCTTATTAATTTGTTCGGCGTGAAAAATCAAACAAGTGATGAAG

>contig00536

ACTtACAAAGAAACAAGAAGGACCCTACTGGCCACGTCTTACCAAAGAAAaCAAgAAaTTTCATTGGTTAAAAagTGATTTCAaTAAATGGAAGGATGAAGATGATACGGaTGAAGAAGGTGGATTTGAAGGAGGGCGTGATTTGGAAGAGATGATGCgCCAGATGGGTGGTCTAGGAGGAGCTGGCGATAGTAAACCAAATTTTGATGATCTGTATATAGGAGATGATGCTGATGGAGCAGACAGTGACGATGAAGAGATGCCTGACTTGGAATGAGGGTGTCTtATTtcTAAATTAAATCAACACTTCAGAACAAATTGTGAACAtAACTTGGCGGATACCATAACTGCTGTAaCACATTAGATTTTTtAAGtGCAAAaTTAaTTTTTGCAATTCATTATGCGCGATTAAG

>contig00537

ACCtAGTAAtATAAATTTGCTTTTTtAGTCCTGGAAAAaTTGGATAAGGCCATAACCAATCTGGCGGCAAGGTCGcGCCAGGTCCTTGATCTTCTACCGCAATTGTTTttaTTtCTTTTTtCTtCTTTTTGTtACAAAaCCAACCCTCTTAAACTTGCATTCTTCTCTCATACACGCATACGGTCATAAGTATCTGTAATCGGTTTAGTCAGTTGAGTCAAACGGTCCcAGTCATACAATTAGCTATGTTTTACATTTtACTCATCTGCCTCTtCTGTTTtATTTTtCATTTGCGCCTGGGTGGgCCTCTTTCCGAATACTTACTCGGTTGCCCTAGTATAAATTCTTCTCACTCTTCAATTACcATT

>contig00538

TGATGTGTAAACATTtGTTTCTTTAAAATCATAAGAATCATAATTtAATAATAATTGTAATGATGATCATCATCGTCAATATCCATTACCTTCGCATtATTCAAtACGATATATGGTAGCGCTtCAGTGATTTtCAAGACAGAAAATAAAGaTTTCTGTGGCATAATATTATtATTTTTATATAGATATATTTATTTTTTTATTTTTGGAATTATTATAAAAAATTTAAAACATTTCTTTCTTAACATTTACAGTTCATAAATCCAAAatCAAGCTAGTGTGCGAATA

>contig00539

CCTTTtAAAAAAAAaTTACGTTTGCACTTTTTTttCGACTTCCTTTCCTTCTAGCGGCTATACCAaTGCTTTCTGACTTtCGTAAAGATATCGACATCAGCATAATACATAGGt

>contig00540

ACCTCAATTATTATATAAATAAATAAATATATATATATATATTCTCGGATATAATATAACACTGTGTTGTGAAaCGATAATTTTCAGATAAATaTTAAGAATGTTACGT

>contig00541

ACTTGAAGACCCGACACAAGATGTTACTTTTGGTTCTGGATGAAATCGATCAGCTCGAGACGAAGAATCAGTCCGTTCTCTATTCGATTTTCGAATGGCCCTCGAAaCCAAAATCAAaGCTGGTTTtAATCGGTATAGCCAATTCTTTAGATTTGACGGACAGAATGCTGCCGAGATTGAACGCGAAaTGCGAACTAAAGCCGAAGTTGTTGCACTTTGCTTCCTACTCGAAGCAACAAATCGAGAATATTATTATGGAGAGGCTCAAAGAAGCAAATGTGATGGATGTTTTTGCTCCGAATGCTATCAAaTTATTGGCTGGCAAAGTTGCTGCGGTTtCGGGAGATATTAGGAGAGCTTTGGATATTAGTAGAAGAGTTATTGAATTGACGGAGTCTCAGAAAATTCTACAGCCGACGAATGACAATGAAGCAAATACTGCTGGAGAGCTAAAATCAAATCCGATCGaCAAAGCAGCTGATATGAAAGATGTGCTTGCGGTGTTGAATCTAGTGTATGGTGGCACTCAGACGAtGACTgAaGAGt

>contig00542

ACTTTGTTCTTTAATTCTGATTTTGAACAAGGGCAAAAatAAGGACGTAACTGTTTCAAAGTTGCACCAGGTCTACAGTAAAGTTTGCGAGAAacGGCGAATAAATGCTGTCGACCATTCGGAATTTGTGGGTTTATGTTCTTTGATAGAAACTCGCGGAATTTtGAAAGTTGTCACGAAGAAGCAGATTCGCTCGTCgAAAGTAGCTTTAGAATGGGACCAAAAAGaCGTAGAAGTAGAGTTGAAGGACAAAGCGTTGATGTCTGATATTATAAACGACgTTtCGtGTCTATGCCAGTGAATCGAACTAGTTTGAAAGAGGCTGTTTACTATTAGGAATAACGAATTTTTATTtATTTtAAATGAATTGCTTTACGAAAGTTTGACTGTTGAGGCCGCGTTCATTTGCTTTTGTTtCAAACGTTGAATCGAATGATTCATTTtATTATATTTGTAAGAAaCCC

>contig00543

AAtCTTTGTCGAAAaTATGACCCAACCATAGAAGACAGTTACCGTAAGCAAGTAGAGGTCGACGGACAGCAATGTATGCTGGAAATTCTAGACACAGCCGGCACGGAACAATTCACAGCCATGAGGGACCTTTATATGAAAAaTGGCCAaGGTTTTGTGTTGGTTTATTcGaTAACGGCGCAGTCCACGTTTAATGACCTGCAAGACCTCAGGGAGCAAATCCTTCGGGTAAAAGACaCAGATGATGTGCCGATGGTATTGGTAGGAAaCAAATGTGATTTGGAAGACgaGAGGGTAGTGGGCAAAGACCAGGgCGTCAACCTTGCCcGGCAaTTCAATTgCGCCTTCATGGaGACCTCTGCCAAAGCCAAAATTAATGTTAACGATATCTTTTACGACCTGGTGCGGCAGATCAACAAAAAATCaCCGGAGAAGAAAaTAAAGCA

>contig00544

AATCTGCCTTATGGTGTTTTTtCGACTAAAGATTGCCC

>contig00545

CTAATTACTTTACTGGGCCACATTTGGAAAAGAATCAAAATGTTTTCCGCCAGGACAGTCTCAACGATTTTATGGCTCTAGGAAGACCATCTTGGATTGAAGCTAGAAACACTCTTCAGCGCTTATTATCAGCCGAAAACCCATTTTTGCAGGACCCACGAACTTGTGCAAATTCCTTTGTTCCTCAAGAAAAaGCAACAATGCACATGCCTGCAAAAATAGGAGACTATACAGACTTCTACTCTT

>contig00546

GCTGAAGATCACTGTATTtAATTGAATCGGCCAGACTTGTAAAAAaGATACTCAAGTGCAAAAAATGCTATGTCAAAAAAATGTGCCGCCATAAAGGAATGTCAAATATTAATCTTCTTCATTTTTGGTCGAGATTGTGGCAGCAGTCTAGTATTCCTCGGCTCCCTCTCCTTCGCCTTCGGTGGAGTCCATGCCGACTTCTTCGTAATCCTTCTCCAAAGCTGCAAGATCTTCCCTAGCTTCCGAGAATTCACCTTCCTCCATACCCTCGCCGACGTACCAGTGAACAAACGCTCGCTTGGCATACATCAAGTCGAATTTATGGTCAAGTCTTGCCCACGCTTCAGCAATAGCCGTTGTATTAGACAACATGCAGACAGCACGCTGAACCTTGGCCAAATCACCGCCGGGAACAACGGTAGGGGGTTGGTAATTGATTCCGACCTTGAAACCGGTGGGACACCAGTCGACGAATTGAATGGTACGCTTGGTCTTAATTGTCGCAATCGCAGCATTCACGTCCTTGGGAACAACGTCTCCTCGGTACAACATGCAGCAGGCCATGTACTTTCCGtGACGAGGATCACACTTGACCATCTGATTGGCTGGCTCGAAACAAGCATTGGTAATTTCACCAACAGAAAGCTGTTCATGGTAGGCTTTCTCGGCCGAGATAACTGGCGCATAAGTCACCAAGGGGAAATGGATCCTCGGGTATGGCACCAAGTTCGTCTGGAATTCGGTGAGATCCACATTCAAGGCACCATCGAACCGGAGCGATGCTGTAATTGAGGAGACAATCTGTCCAATCAGTCTGTTGAGGTTCGTGTAGGTGGGTCTTTCGATATCCAAATTACGCCGGCAAATGTCGTAGATAGCCTCGTTATCAACCATAAAAGCGCAGTCAGAGTGCTCGAGAGTAGTGTGGGTGGTGAGGATAGAGTTGTAGGGCTCAACAACAGCTGTGGAGACCTGGGGTGCTGGGTAGATGGCGAATTCGAGCTTGGATTTCTTTCCATAGTCAACAGAGaGACGCTCCATCAAGAGGGACGTGAATCCGGATCCGGTGCCGCCGCCGAAAGAGTGGAaGATGAGGAAACCCTGTaaTCCGGT

>contig00547

ACATCtcTTCGtATTTGCTGGCTATAAACATtGCAGtGACTCCTACCAaCTGCAATCTCTTTCTATCTATCGACCGGAACGACTGTAAAAaaCGAtCGATGATTGAGATGGTTAGATAGAGTGTTTCTTGCATCAAGCGAAATTGTTGATGCACTTCAACTAGCCAGTCAACGAGCACACACCTCATTTtAGGCGTCACTTCTTGTCCCGACAAAAATCCCTTTTTAATAGAAAaTTCcGCCTCTAGTTTTCTCAGATAAGAATAAATGTCGTTGCTGTAGATAGATACTAGGATGGGATTTTCTTtGTCATCTTCGTCGATGTCTTCTACCTCTAGGAGATCGGATGAAAAGGATTCCACTTCTTTCTTCAGCTCAACTTCCTTCTTGCTGA

>contig00548

TTTTTTACCACCGGCTTCACGATCTGAACTGGTGGCTTGTCCAATGTTTTTTCGGATTCTTTGTTGCTTAGTTGCTTGAGCGTCGACTTTTTtAAAACGTTGCTGGGGGGCAGCAGACTAGATTTGTGAATTGGATCCACGCCTCTGTGAATCGTTACCTTGTTGCCAATTTCACCCAACGCGGCCCTCCGCGTTGGTTTCGGGACTCCAATCGCCGTGGGCTTTGCATTTTTCGTGTTCTCTTGATTAATATTCGTAACTGGAATCCGATTTCTCAAAGCCATCGTCAGCTTCTGTCCTCGAAAGAATGAAAGTAACTCAAAAATATCTTCCAAAGGAGCCGAAGCCAGTCTCCAAATTCGAAACAACAAACTTTTCTCGACAACCGACTTTGTTTGAATTTGTTTGCTGAGCTGAAGTTGCTGACAGTTGCTGACTACTACATGGGT

>contig00549

CTCATGCTACTAATTACATGCATCGTAATTCATTTTTGAATCTAATATATATATGTATATAAAGATTAaTTATTGCATAAATAAGAGTAAGAAAAGGAATGTTGGAAGGACAATGGGAAAGGAAGAGAGAGGAAGGATAAAGTGGAAAATTACACCTAATAATTCTTAATAAATAATCTTCATTCGCCTTAGGATCCGACAATGATGTTGTTGATTGGGATGTGAATAAGTTTAACAAAGAATCCTATGAATCCCATTATGCAAAAACCTATCGCCGTAGCGATGGCGATTTTCTGGAATTCTTTTCGGTCAGGCTTCGTACATCTTTTCACAAGGCGAATGCTGTCCTTGGCGAATTGCCTTCCAGGTTCAGTGAACTTTTTGATTTGATCCATTTTGTTTTAATAGATAAATGAAGCAGTTCTTTGCTTTTGGTGGGTTGATAGATTACTTAGCACTCAGCGTGTGTTACGTTCCTTTGACGACTAATTTCGTTCACTTTCGTGTTCTTGACAaCTACGT

>contig00550

aCAGCCTCACAGtGATGTATGAGCGTATTGACCATTTCTCCAGTGTTGGCGTCCcAAACtCTGACGGTGCTGTCCGAAGATCCAGATATGATGGCCTTGTCATCGTATTGAAGGCACAGAACTGACCCTGTATGTCCGGTGAGCACCTTAATGCACTGTAAGGTATTTCTGTCCCAAATTTTAATAGTaTTGTCCcTAAGACCAGATACTATCTTCTGGTCGTCGTATTGCAAACAATATACTCCCTTGGAATTCTCACTACGGCAATTTATTCTCTGAAGATTGTGTCGACCCATTCTCCAATtGTTGTCTATACTATCTATGTCCTTTACGATTTTtGGAAAAAGAGTCCTGTAAAAGGCGTGGTTTGGATGGCTCTCGCCCGGTCGTGGCTTGAaCAAATACTGTATCCATCCTCTCCTTTCtGCCAGTCCTCTCCATACGGAGTCTGTCCTAACTTTGCGTtCgATTAaCTTTTtCCAGAGCATACCTTCGCTTATCACTCGGT

>contig00551

GTAGAGAGTGCTTTTGTCATATAATTGACGAGGATAATCGACTTATCGCAATTATTTTCAAAATAAAaCTTTTCCAAATCAAGAGTTCGAGTATCTGCGAAAGTGATTCCTCAGAGCAAAAGGAATCaGAATCACAAAAATCTTGGAAACGGATACCTCGA

>contig00552

ACCAACCTCGCTAAACGATGGATTTCGTGGGACGAAAATACTGCCTCGACTTTTGTGAAATCGGACATTACGAATCTATCAGTCAAGCAAaGAACACAATTTCTAACAGTATTGTTTAAAGATGATACTGTGCTCCCAATTACGAAGCTGCAAAAAATGCAAAAGATCTATAAGTTCAAATATCTGAAAAATACTGTAATAAAATTCATATGGATTCGTCTGGCTCTGAAATCTCGATGGGAATTGAAAGTAGCAGAAGCTCTGGATTTCGTATCAAAAATTGGTTTACTGGAATACGTCAGACCAATTTATCGGGATCTTTATGCTTGGACAGAAATGAGGCAGAGAGCAATAGACAATTACCTGAAAACTAAAGGTGAGATGATGCTCGTTACAGCCGAAATGCTAAAGAAAGACCTGCATCTTGACGGTAATTAACGTGGAGCCCAACTGAAAATCGAAACATTTGTGCATTTAATATTCTAGAAGAATTATTCTGAACTGGTTGTATTTCAAAaTTTGAATTTTTtGTGAATTATACACTGGAACCTGGATTTATGCAAGGTTT

>contig00553

aCTTCGAGGTGTAACGAAaTTCTGCCACAGTATTACTAGTAAAAGTAATAGcGACGATGAAGAAGATAATAGTAAAGATAAGAAAGATGTAAAaaTTtCATTGGACAAAGTTATTCTGGCAGATTCGCCGGAGAAGTGTGATTACGCCGTTCAACGTATTCGCAGGGACTTGTCTGATGGAATATTGGGTTTTGATTGCGAGTGGGTCAAAGAAGGATCCGTTTCATTACTTCAACTTGCCACAGATAATGGTGTCTGTGCTTTATTTCGTCTTGGAAAAATTGGATATGTTCCTCCCAAACTAAAaGAGCTGCTGTCTAATAGACGTCTACTGAAGGTGGGCGTAGCCCcGTtCGAAGATGGGAAGAAaCTAACGAaCGATTATGGATG

>contig00554

ACGACGCATTAACTTCTGTTTTtATTtACCATCAGATTCTGGAACGGGCGAGAAAAAAACGATCCTATTGGCAGAGCTTCATTCtCTTTTGAAGGGCATGTGGGAAGAGGACGAAGaCGAAAGATTTCATGGATTACCTAATGGCGTCATCGACGCGAGGTTCAAGGTTCAACGGGGTAGCATGATGAGTaGTAGCAGTAACAATTCTAATAAAAATTGTAAATTGAATGAAATAATTAGTAGAGGTGTCGATGGAACTAAGAATAGCATTCCTGCAAGGAATAAACCTTTGTATCATAATTGTTATCTCCAAGCTCCTGATGGAGACATTTTG

>contig00555

CCCTTTCCAGTCAAGAAGGAAGGAAGGAaCAAAtCTCAAACGAGTGGACTTCATTCAaTAAAAAAaGAGAATTTCCTACTaGTAAAAAaCGCTTAAGCGAGAGTGCTTTTCtATTTtGGTTTtGGTTTTGTTTTTtCTTTtCTTTtCcTTTtCGCCTACGTATTTATAGGTCCTGTGTTCACTATCACAGTTTATGTTTAAAGCAGCAGCAGATAaCAAAGAAGAAATTCTTTAATTAAAAGGGCTCAAACACGAATGCTTACGAGAAAGTTTGATAAAGACTCACTGGACCCAAAAAGCTCCCTCACCACTCCCAGAGTCCAGTAACTCCAGTTTTACCCTGGAAAGGATTTTTTtttCTCTCACTTCCTaTGT

>contig00556

TCTGGCATCAGTTCTATATTCTGGATCACTGTGGAAAGCAAATGGGCCTTCTCGACAAATGTCCCTTGaCCTTCGAAaCAa

>contig00557

ACCTTTTTCCAaGAAGTCGATGAGTCTTCCGGGAGTGGCGATACAGATCTCGACGCCTCGTTCCAAGTCACGTGCTTGCGGGCCCTTGGGCGAGCCGCCGAAAaTGCAGGTGTTGCGAATGCACGACGAGGAACCAAAaTCTCGTGCGACTGTCTGAATTTGTTGTGCGAGTTCTCTCGTAGGCGCAAGGACCAACACGATtGGACCTTCTCCACGACTTAGGCGGGgCTGATTGTTGATGTGGACTGTTGCTGGTAAGATATAGGCTAAAGTTTTTCCAGATCCGGTCTGTGCAATTCCAACCATGTCCCTGCCACTGAGAGCAATAGGCCAGCCTTGAGCCTGAATAGCGGTCGGTTCCAAAAaGTTCTGCTTCCTGATTTCCTCCATTACATAATCAGGGAAGTTGCTTTCCTCAAACGCCTGTATCGGAAAGGGGGTATTGTTTCCTTTGACGGtGATTTCTTTGTCTATgTGGT

>contig00558

ACTTCCCAAGGCTAAGTGTTTGGAGGCTTTAGCTGCACTCAGGCATGCCAAGTGGTTTCAGGCTAGAGCAACCTCACTGCAGAGCTGCGTAATGGTTATACGCATAATGCGTGATCTGTGCAATCGTGTTCCAACTTGGGGACCATTGAACTCATGGGCGTTGGAGCTGTTGACTGAGAAGGTGATCAGCACCGCTGGCGGGCCACtcAGTCCAGGAGAAGCTTTAAGACGACTGCTAGAATGTGTGGCAGgaGGAATTCTGCTCCCTGGAGGACCAGGATTGTCCGATCCTTGtGAAAaGGATCCAACCGATGCAATAGGAACCATGACGGCTCAACAAAGAGAAGACATCACTGCTTCGGCCCAGCATGCACTCAGGTTAGTGGCTTTCCGCCAAATTCACAAAGTCCTCGGGATGGAACAACTACCGCCACCAAAaTACAAGGGACGTTTtGCTCGCAAACGAAGACGCGACAATAGC

>contig00559

ACTCCAAAGTTTtGTCaGCcAATTTGAAGGCACCAATGACGGCAATCGActCGGGTTGGTAGATGACGGTTTGgAACCACCTGTTCACATTCTGGTAGGGTTTGCGTAGGCTTGGTTCCAGGACATACTGATAAAGATTCAACAAGGTCGTTGCTACACTAATATCAGCCAGGGTCAAGCGTTCTCCGACTAAGAAGGTTTtCGTGAGTAAGTGAGTGTTCAACGCATTCAGTGCTTTCTTCACGTCCTCCTTCGCATTTtCCACTGCCTGCTTGTTGAATGGCATGATTCCTAGCAAAGGAAATACCCACGCGCAACTTGCAGGCAGAATTTCCGAGTCGGCGAACCCAAACCATTGGATGACCTCTGCGCGTTCCAGATCCGTCTTTCCTCTCAATTGTTCGTTGGCCACATAGTATGCGATGGCATTACTTTCAGTCAGACATTGGCCATCCTTGGATTCGAAAGCTGGAACCTTGCCAAGGGGGAATTTTTtCAAGAAGGCCTTGGATTTGTTGGTATCACCGAAGACAAAaTCGTCTGCGATCTTGACCTGGGCCCCAGAGTATTGGGCCGCGATCAGAGCCTTGTAGGCGCGGAAATTTTCCGGGTATGTGTAGAGAGT

>contig00560

ACGCATAAACTCGTTTTCGGTAGATTTAGTGTATAATTTgaTCTTGGATCCCTTTCAAAAtGCAGAAAAtATTTCCtACATTCCTAAAGTCTGTTCAGTTTTtAATAATATACAATatATAAAAAaTAAAaTAAaCAAAaTTGCGTGCAGCGGCCAGCGAgaGCGAACTATCGAGAGTTTTTGTTCATGAAAGGAATCCTTtCAACaGGGgCAAaGTTtAAAAaTTAAAAAATGATTGgCCGAGAAAATCATTTGAACCTATCGATAGATCACAGTCTCCAGCCTTTTCCCACCGATTTtAGTTTTAACTTTCTAGAAAaTATCTATATTTACAGCGACTGCAGATCGCGTGTAAAATTTtCTTTAAAaGTATTTTGCTCACATGAGTAAGTCATTTCTTTCGTGTTGATCACAAGGTATTGAAAAAATT

>contig00561

AGAAACGAAACAATCTTGTGTGCAGTTCAAATAGCATGCAtAGAAATAAAGACTTAGGTATTGAACATTACCTCGGTTATCTCTCTCGAATTCGCAAGAGTTtCTCGATTCAGGGTCGATTTTCTCTTGTGAtAGTTTAAAGTTTAAGTCTCTCGTTATACTTATAATACATTTCAAGCTTGCTTCTTCTTTGCCAAGGCAAGCATCGTGGGCGATTTgaGTTCTGTTCGCAAACTGACCctcATGTTTTTCGTTAGTG

>contig00562

GTACCACGAAACGGAAAAaCATTAGTGCGTAAATTAAAGATAAACTTCGAACATGTTAAGGTTAGCGAGAAGGATCCTATTGTATGGAGGGGTGCGGACACAACCAAATATAATCTCGAATTTGACAAAGAACTTTAGTAAATGAATTATTGAAATTTCGGACTGCACGCCTTCTTTtATGTCGACCCCCTCCcTTGCTTGAGGAGAAaCCAATATTTCCTGCCGTAGTGCATCGGATACTTTATCTCAAATAGGCAGGATAACAGCTCTTTTTCTGAAAAAAACGGCGCTTCATTGCACAGCTTACAATAAAGTGCTATTTTGCTGCCGCTCTGCGGGTAGATAAAGCACGCTTATTCTGCCTATTTCAGATAACAGGATATATTGGTTTATTAGTTACACGGAATTATCTACTAGTTATCAAACGGATAACTGGTTAACGGAAAGAATTTTGGCGCAGCGGACGCCTCAAACTGGTTCTCCCAGCAAGAGCCTGGGGTATTTtGATGCGCATGCGTGAATAATGGTTTAATATTGGCTCTGATTGACGCATGCGCAGTAGT

>contig00563

CGAACCTCTGGGTCTTCCATTTTAAATGGATTTTCAATACCATATTTGGcAAGTTGGAtACTACTCTCATCATATTCAAAaTTaGTtATATTCAGGAAGGGCGGTTTTttCGCCCAGAACGCTCATATCTATTTCAGCTTTATtA

>contig00564

GgACAACcTCTTACATCAAGTGCCCGGAGTTGATGCGCTTTCTTCAAAAAAGTGCATAGAcATTCCTCTTGTAAACATTTAgTCcaCCACGAAtTAATACTACCAagCTTGTCAATTTTTtcAGTGGTGGGTTtCTTATTTtATTCAATCCACTTATATTTAAATAAGTCAGTTTActC

>contig00565

CCCACGATCAATTACATTtcGACAATGAGACAAGTCTAAATACGTCAGT

>contig00566

gTTGGCGAGTCCTTCAAGAATTCCATTA

>contig00567

CACtAATTTTTCAAGGTGCGTAAACGTTGCCAATGTTGACAATAATcCAGATTGCAATTCCCTGCTCCATGGTGGACATAGTTTtAGTTTCAAATTTTTCAAAGATGCAGCATGTAAAGGTAACGATTTCAATATtCcAgTGCaTtatCTTtATCTAAagCTATGCCAAatGTTTGTAGGTTaTTGCaTTTTCCTATCATCGTTGACAATAAATtcATATCAAAaGgCAGTGcAtCGTGACtGCCAAAaTAATCTCCTGAATTGTCTCAAATGGCAATTCAAGTAGAAAaTTACCACTCCAATTTGGATAATaatcGTTGTATAAAGAAAATGATTTCAGTTGTTTCATTTCTCCTAAAAAACTGGTGAATTCCTCTTCTTGTAGTGACCATGAGACACTTTCATTTTAAAATGGGtAATATTCTCACCCTGAGAAATTAACCAAGAGAGAATCACCTGCCATGAATTGTA

>contig00568

ACGAAGtGCTCGTCTCTTAAAAAAtCAAGAAAATTTTCGCAAAATCTCTTCTCATTttGgAGGCCTTGAATCTCGACGATTCCCACTACcTAGAAAaCGTAAAaCGAAATACAGAATCAAGCAGCTTGAAGCCATAATgCACGAAAGTAGAAATAATATAATCGGGCTTtAAAAAAAAaTTGTCAACTCTACAAATTGCGAAATTCATTTtGGTCTCAGAAATGGAATAATAATAGAATGAGAATCAGTAATTGAAGAAGTAAATTGAGCGTTATAGAATAATTTGCAGAAAGATGAACATTCTTTAAAAGATGCGTCACGAACTGTCAAATGCCTTCCTGATTTTTAAGACCTTGAATCTCATTAAAGTTTTTTtACGCTGAAAGCGCaCAGTAGAATAGGAAATGGAATTTTTTTtAGTTtGTGATAATATTTATGTAACTTCTAAAGCTTCTGCAGAAGCTGAACTGTTACATTGAAAAATATGGGAATGGAGAGTAAGTCGAGCGATATTATATAATATATACTATACATAATATATTTTT

>contig00569

CATGCCAGGGTAGCGTTTGTAGAGCGACCCACGG

>contig00570

GTCGTAGGTAATTTCCCACCTCGGAACCGATACAATAATATTCTCCATTTTCCTCGACTTGGAAGCTTATGGGCTTGTCTCCGTAGGTGCGAAGAGCCATTTTGTAGGATATTCAAGCTGACGAGACTTTTTGAAATTGTAGAAATTATTAATCAGTGGTGTCGTCCGCTTTTTCCTAATAAATATTCCTGCACTTCACTTTCGTAGAAAAAACGACTTTTTTGTTAAAATATGAACCAACAACAAATCCTAACCTAGCTTCTGTTATCCGCCATGTTTACACAC

>contig00571

ACTCAAATCCTGCTTAGAAGAAtCCTTTCCATTGACCATTTTCTGTTTCTCCTGCTTCTTACCAGCCTGATTTTGAGCCTGCTGCTGCTGCTGATTTTGCTTGTGCTTCTGTTTATTCGACTTGGCGACTTCTTGCTCGGGTTCAGGCTCCTCCTCGCTATCCTCATCCTCATCCTCgTCGTCGCTGTCATCATCACTCTCCTCTTCATCGTCATCAGCCTCCACCAATTTGGTCTGATCCTCGTCGTCAGAGTCGGACTCTGCATCCACATCCAAGTCGTCATCCTCGGACGAGTCCTCGTCATCGTTAGCTTGCTTCTTCATTTTTTActGGCATTTGTGGCGGCAGGtGATTCGACAGCTTTtCTTTTtGGTTTCACCAACTGTGGCGCTTCCTCCTCCTCGTCCTCCTCCTCTTCCTCAAGGTCGCCGAAATCGAGATCTTCGTCGAGGGTCAGATAACCGGTCAAGTGCACGTGGCCGAAACCATTACATGTGAATGCAATCTTTGTGCCTTCCTGGAAGTTCAGAtCTAAGGGt

>contig00572

AgTggagAGTAATATTTTTtgCAcTTCTTTAGGACAGATGGAATCgAAAAACTTTAAACAATGAAAGTTATCTTCACAATTTGCGCTTTTAGCGTATTTCTTCCTTTAGTAAATTGTTTAAAGGTTGAGAGAGTTGTCACCAGATCACAATTAACAAATGGTCTTTACTGGGATCTAGCAACCATGAGACtCTTTTTCGAAGACACGTATAATAGTTCACTCTGCTCGTATAATCCGTCGACTACAAACCAATATTGCGCTAATCTAGGCAACAAATATCTCGTTTCATTTGCTGTTCCTCTTGAAAAACCTCCTCGTCAATTTGTAGTGGGTGCTAGTAATGATATCGTATTAATCACATGGGATGGTAACACAAACGTAACGACTTCATCCACAGTGTTAGCATCTGTGGACAAAAATATCAAAAACAACAGAATCAATTATGGAACAGT

>contig00573

ACTTAGCAAGAATTTATTTACTTATGAGGACGTATGTATTTTATCAATGAAAAAaTTATATATTCTTATCTCTTTACGATGACATGGCCAACAACCATAAGAAATAAAaTCT

>contig00574

ACTTAAGTTTGAGAGTCTGTGTGAGTCtCAGCAAGACCAAGAATTAAGtATTGAAATGGACGCGAAGACGATTGTCgAGGTTTGCAAAGGCCAGGGATTGAAGGGTGTTCCGAACTGTTCGATACTGAGTGATCGATCGCCACCTCCTACACCTCCAGATCCTCCcAATCAGAGGTTAACGAAGGAGCAACTCTTACCACAAACGCCAAGCGTTTTCCTAGAAAaCAAAAaGGATGCTTTCAGTCCCCAACtCCAGGAGTTCTGTCTGAAACATCCAAtAGCTGTGATTCGTGgCTtGGCTGCGGCTTTGAAGCTTGATCTCGGTCTCTTTTCCACAAAGACTTTGGTAGAGGCGAACCCCGACCACGGAaTCGAAGTTCGGACGCAGGTTCAGCAGACTAGCGACGAGAACTGGGACCCAGCGGGACGGAAAGTTTGGGCTTGCATAAGCCATCGGAGTCACATGACGATAGCCAAATACGCGAATTACCAGGCGTCCAGTTTTCAGGACAGCTTGAAGGAAGAGAGGGACAAGTCACAGGGGATTCACTCCTCGAATTTGTCGGACTCGGATTCGAAGGATAGT

>contig00575

ACttCCGGCTTTGGAACAATGCTtGCCGCCAAGAGAAGAAGTGATGATTCCCGACAACGCCGAGGaGTGCATGCTCACAGaCTTGGACCCTGAGCAGGAGGCAAGGCGGAGAGAGACTCGTCCAGCCTACGAGGAAGACGAGGGAGGACCTTCTCGTGTGCAATGTGCCACTCAGTAATTCCGTTTCGATGTTTCCATCGCCAGCGAGACGGCCATTGCTCGTCTTAAACTA

>contig00576

ACCGATCCCAGGTGGAGAAGGAACTCAGAGACATTTGTGCTGACATCCTCGGAGTTTTGGAAAAGCACCTAATtCCcTGCGCaTCAACGGGCGAATCTAAAGTTTtCTATTACAAAATGAaGGGAGACtACCaCCGTTATCTGGCCGAGTTTGCGATtGGCAACGATAGGAAAGACGCTGCGGAGAATTCCCTTGTAGCTTACAAAGCAGCAAGTGACATTGCAATGACGGACCTGCCTCCGACTCATCCAATTCGTCTAGGACTAGCGCTCAaTTTCTCAGTGTTCTACTACGAAATCCTGAACAGTCCCGACAGGGCGTGTCGCCTAGCAAAGGCTGCCTTTGACGATGCGATCGCCGAGCTAGACACCCTTTCCGAAGAGAGTTACAAGGATtCCACCcTCATCATGCAATTGCTCAGGGATAATCTTACCTTATGGACCTCGGACATGCAGGGAGATGGTGAAGgTGAACAGAAAGAACAGTTGCAGGACGTGGAAGATC

>contig00577

ACTTAAATAGAAaTAACAAaTCATaTAAAAATAATACACATGACAGCTCTTTTCATTTtACAAAATTTCGAAGATTATTATTTttAAATTtACTATAATTCTAATTCAATTTtttATTTtAAAAaTAAATAtATACAATTtCTTAAAAACTGAGCAAATTTACATATTTTTTAACATACATTCATATTTttAaTTAAAAaTTATCCAGTGTTTATTTtATTATCCCCTGTTGGGaGGTTCCCCtCAATATTCCTAAGACAATGAAAAaTATCTTGAATAACAAAAaTaTTTGATCGTCCcTATTCAGCAAAAAATGATTATACCAtGT

>contig00578

GCTACATTTTTCGTAGTCCTGAtCcaTTCAACCTCGAaCCAAaTAAATTTGGACAATCTGACGGAATTCCCGTGGATAATATTTTTTCACATGTGCGAtGATTTTCTTAGATTGAATGGATTGCgCCcTaGTGCAGCGACGACAGTtGTTTTTtATAGCTTCCGGAAtCAaTGCTTTAATtCATTGCCCATCTTAtCACAGGGCATGCGATTCATGACGCAGTCGATTTGATCTTGCACAAAaTCTTCGTCCTTCAGCAGCTCATCGATTTCGTGCATTtGGGAGTtCGTCAACGTTGTCAAAGCGAAGAAAAaGAAAATAGTAATTATTTTtgAATTCATTTCGACGATTTCAACGGAAGTAGATTTCACTCAAAAAGTCAAATTACACAAATTTTtGTCACGCGTtAATTTTTTtCTCAAAATGAAATTCTGGTTGTTAGGA

>contig00579

CCccAATAGGTTTCGGCTCTCGAATTGTGTCAGTTTGTAAATTATTCTCTTATCCGCGTTTAGATAGAATTACTTGAAATTTTATTAAATTACTACGATGTCTGGATTATTAAATGGACCTTTTTGCCAATATGCTGAAGCGACTTTAGAAAATGTTTGTGATCGTTTGCCGTTGGGAAAAACCGGATTTAAAGCCTTCAAAGCTGTGAAAACTGGAGTCATTAATTATAAAAAATTTGCAGAGGAAGAGAAAAATCGAATTAAATGTAAAAACATCGAAGCTCATATTGAAGGAGCTTCTAAAGGTTTAAAACCTGTTATAGAACTCAAAAATATTCTGATCCCTGAAAGCAAATCTGCTGACCGAGCAAAACCTGGATTAAATCCATCAGCCTCTTTGAATGATTACACTCCACAAAAACCA

>contig00580

ATTCTCGGCCGATTTCTGATATTTTGTATGCATATGTGTTCTCATCCAGTTTATAAGAAATGCCAGCAGCGTTTGAAGTAAAGTCCAGCTGGGCTGATGAGGTTGAAGAAGAGGGAGGAACCGTTTTGCCACCAAATTCTGAAGTGCACGAAAATGGCTTCAAAATTCTCACAGAATACAAACTCAACGACGACAACAAGAAGGTGAAGGTTGTCCGCACCTACAAGATCGAGAAACGTATAGTTTCAAAAACTATTGCCGCTCGTAAAAACTGGCACAAATTTGGCGACTCGAGTGATGACCGACCTGGACCAAATCCTGCGACTACCGTCATCGCTGAAGATGTCTTTATGCAGTTCATTTCAAGCAAGGAAGAGGATAACAAACAGGAAGAAGATTCACTTGATAAATTGAAAAGTATGGGCGAGAAAGGTGTCGTCAAGTGCCGTAATTGCAATGGAGACCATTGGACGACAAAGTGTCCTTACAAGGaTACTGTTCTGAGTGGAGATGATAAAAAGCCACTGATGAATGCTCctGGTGCTGGTGGAGCTGCTGCCATGGCCGAAATCGGAAAACCACAAGGAAGTAAGT

>contig00581

AGgTtCCtCCCAcAcTTTCCCCTtCCAACAAGATGAGtATCCATCAATTGAAGTTTTtCAATTTTGGgCAGCAAGTGGCAAATTTTgaCAAAGTTTtgAATATCTATACAAATACTTCTtAGGTATCGTCCCGAGCGTCTtATAATTTTA

>contig00582

AAgTTTGTCaGCTATCGTTGgCAGACGCTTG

>contig00583

cTTTtCAATCTCGGAAGTCTCCATTTCTTtATATTCTGCTTCCTCTTtCAGATTTtGATCTTCCATCATTTTtAAAaGTCTCTCAGTTCAaTTCCCAATGAATTTtCGtAAAaTCGTTTAAATTTGAAGCCgCcAGAATACTAACCATGCAGAAGCAAATTTgATGTCTTGAAATctAAAaCAATAATtCTTCGGTCCAAAAAATTTtCcTTTTTtCAGCAGCcGTTTTGAGTTTTGTTGTTGTtgTAAAAGAAgACAAGAGTCACAATAAAGTAACTTtCAGAGTCgaTTCATCTAA

>contig00584

ACAATGGGATACCCTTGATGGCATCGAATATGGACACTGTAGGAACATTCGAAATGGCAAGATCGTTGGGAAaGCATGGCCTATTTACAACTCTCCATAAATATTACACAGCAGATGAGTGGAAAGATTTTGCTGCTAAGAATCCAGATTGTGTAAAATATACAGCTGCTAGTTCAGGAATCGGAAATGAAGATTTTCAACGATTGACTAGTGTTTTATCCGCAGTTCCTGAACTCTCTTTTATTtGTATAGACGTTGCAAATGGATATTCGCAACATTTCGTtGAGTaTGTTAAAAAACAaGaGCAGAGTTtCCAAATCATACAATAATTGCAGGAAATGTTGTAACAGGCGAGATGGTGGAAgAATTAATTCTgTCAGGGGCAGATGTGATCAAAGTTGGCATTGGTCCTGGATCTGTCTGCACAACaCGAATGAAAACTGGAGTTGGATACCCCCAATTGAGTGCTGTGATTGAATGTGCTGATGCAGCTCATGGATTAAAGGGTCACATCATTTCAGATGGAGGCTGTATATGCCCTGGTGACTTAGCTAAAGCCTTTtGGAGCAGGAGCTGATTTTGTAATGGCAGGAGGCATGTTCGCCGGACACGATGAATGCGGTGGTGAAGTGATAGAAAAAGATGGGAAAAAAGTTAAACTTTTTTACGGCATGGCATCAAATACGGCTATGAAAAAACATGCTGGGGGTGTTGCTGAGT

>contig00585

TCTGTCGCTCAAACAGTTTTGAGTAGTTCGGACGTCTTCTTTCCATCGTTTCCACCTGCGAGATTTTACAACAAAAGACTTGAAGCTGCCCGTTTTAATATGAGAAGGATTTGTTTACTTCTCGCCTTTGTGGCCGCCGTTTCCTGCGATTTAGGCATAGTCGACGACAAAGCTTCCAAAGGACCTAAAGTCACCGATAAGGTATGGTTCGACATCGAAATCGGAGGAGAGCCAGCAGGAAGGGTAGAGATCGGCGTTTTCGGCAAAaCTGTTCCTAAAACTGCAGAAAACTTTGTCGAGCTCGCGAAAAAaCCCGAAGGTGAAGGATACAAGGGAAGCAAATTCCACAGAGTTATTAAAGACTTCATGATTCAAGGTGGAGACTTCACCAAAGGAGACGGAACTGGAGGTCGCAGCATCTTCGGAGAGAAATTCGCTGATGAGAACTTTAAACTGAAGCACTACGGAGCTGGTTGGTTGTCCATGGCAAATGCCGGACCAGACACGAATGGATCTCAATTCTtCATCACTGTTAAAACAaCTCcTtGGCTCGATGGCAGACACGTCGTTTTTGGAAAAGTTATCAAAGGAATGGATGTTGTCAGGAAAGTTGAGAATACAAGCACAGACAGTCGAGACAAGCCATCAAAGGATGTCGTGATTGCTGATTGTGGTGCAGAGAAaGTGGACGTTCCATTCGGTGTGTtGAAGGaGGATGCAACTGAATAAAAATaGTGTCGAaGT

>contig00586

TatGGGGgtAATTACCCTTGAaGTGGATTGTATCAATACAAATCGATGATACTTCTGCTACATGTCCCAATTtGAACAAGGCCCACTCATTTCCATAAAaCtGAAGCATCCCcTTTtCATCAATTtCGATGACAGGAGGCCTGTCAAGTTTCCTGGCAGTTTCCCAACCATCGCCCAtGTTAACACCTCTCCCAGGTTTAATTAAATtCCTTGGGTGGCCAAAATGGGCGTTACTGAATCCCACACAGAGACCTCCATTTTCGACTGATAAAaGATCAACCAACTTATCAGATTTCAAAATGTTCCAGTCGGGTTGAGCGAAACCATAAACTCGTAATCTCGCAATTCCACCATCAGGAAAAATGTTGAGTCTCAGATGAGTCCATTTTTCATCGGATGAGACTGCAACGTAATTTTTGCTCGTCTCTGGATAACCGGGTTGTAATTCCGTCATCGATACGAGAGTCGTCC

>contig00587

TTCATCGCTTGGCGCAG

>contig00588

GGCaCTTTCATCTGTAGGCGATAAGCGTGCGGCTtGAATGGAAAaTCTTGGGgCGTAATTGCCAGTAAAGAAAGCAGTATCTACGCAGACGCCTTTGATAaCTGAAGgtCGACCCAAAGCAATTATCGCCCAGTCATGACCAGCAATTCTTTTCCTTCTGGTTTCCCAGCCATCCATCCACTTTCCGtATGtCGTATAGAGATTATCTTTCCATACTGAaTCctCATCCTTCAGTAGATTCTCGGCAACAGCGAACCAATCGTCTGTGGCAAAAaGTaTTCTTGCTCCATTCCGTTCTGAaGCAAGTTCGTTAAGTTCGGCAAAGTCAGGAGTCTTCTTTTGAACTGGCATGATGAAAGAGTTTGTGGTTTCTTAGATCCTGGATtCcTGTTCGCTCCTAaCAAaTGCCGGGCT

>contig00589

ACTAtGGGgtATTCATTCTTCAGTTCCTAGTTTCGAGAAGGTCGCTCTCACATCATTCTGGtcATTCTGGTAAGAACTTTCGAACTGTGAGGATCATCTCTGCTTGGGAAATTTCTTATTTTTGACCATAGACGTCTGGACAACTGGAGACTAAAGGAGACTACAATAAGTTCAGCAGAATGATGATGTTGACTATTTTGTTTGCGAGTCTGGTCGGTCTTGCTCTCGCCGGACCATCTCAAGACGTAACGCAGTTTGACCAGGAAAAAAATTGGAAATCCGCAGTCTTTTACGACTTCTCGGCTAACGACATCGACGGAAATGTTGTTCCTCTATCAAAATATAAAGGAAAAGTTCTCATTGTCGTTAATGTCGCCAGTAATTGTGGATTGACCGATGCCAACTATAAGCAGCTTCAGGTCCTTTATCAGAAATACAAAGATCAAGATTTGAGGATTCTTGCATTCCCCTGCAACCAGTTCAAAGCACAGGAGCCAGGATCGTCAGAAGAAATCAAGGAATTTGTTAAAAAATACGATGTGACCTTTGATATGTTCGAGAAAATCGAAGTTAATGGCGAAAATGCTCATCCTCTCTGGAAATGGATGAAGGCTCAACCCAAACCTGCTGGAACTTTCAGCCAGGACATCACATGGAACTTTACCAAATTTCTCATCAGCAAACAAGGAGAAGTTGTTGCCAGATTTTCACCCCACACCGaGCCGAAAGAGATGACAGAAACACTCGAAATCTACCTCCAGAAGGAAGCTGAATTATAGACTTAGTTTTGATATTTTTAATTATTTtCTTTTTGTTTTGTGTTGTGAGTTTTTATAGAATTGATACGTTTTTGACCTCTCAGAGGAGAAATTAACTTCGATAGGATAAAATAAGATAAGGTTTGTGAGATTGAATTTGAAGTTTGACATCATGTAAATAATTATTG

>contig00590

ACAGACTTAGCTTTTAGAAAAGCAGATAATCCGAAaCTAAGGCTGATTTGCTTCTTTcACATTTTA

>contig00591

CAATCGCAAGCTGATTATTATTATACAGTTATGCATTGCTTTGGAATTtCACGATTATATTGTGCAAGTGCAAATGTGTCGTCGTCTTGTGTGCTATTAGAATTTTTGATGGCTCGATTGATGGGGAAACATATGATTTtGCTTTtAAACAAAGAATCTGTAACTATTTCATAATCTATGagaCTGTAAGGCCTGGTCGAAAACATTTTTAACACTCATGATCCACGTCGCgCgATTTTTTtGCATGATGTCAAATCGATTTTTTTtAACTCGTCTATATAAaTTTATCAAAAAAATTCGACGCAAAaTTATTGTCTtAAaTTaTaT

>contig00592

GTGGaGTtATAAAAATTGTAACACAAAAtATGTTTtCTTAAAGGAATATGATCAAGagACATTGCAAATTGACAATTCATCTGTCCTAATAATATAAGATTTTtGCCAATGGATTACAAAaTTCGAAGATATCTATTATGCTCTTtCCCTTTCCCTAAATTTTTATCGTTTGCAAGTTACAGCTTTGCTCGATAATTCCTTGGACAGAATACTGAAGTATTGAGTTCTGGAACATAATGCTACGATTTAGTCCTCATCTTCCCCAGATTCTGATTCCTCTTCCGCGTTTtGGAGCCACTCGACAAATTTTTtCATCTGGTCTAAAAACAGCATTTtGCCCTTGACAGAATGTCCCTCCT

>contig00593

AAAATCACTTCTTCTGAAATTACGTCGGTTTTGTAGAAAAGTAAGACTATCTTCTGGAAGACTTTCATGAAGTTCATGTTTtCGTAACAGAATTCCTGAACTTTAAGGATGAGCGCGAGTTCAGATCTGGCAGTGGTTGTGAAAGCACCGAATAGTGCAGTGTAAATTTTTAAATGTTTCAAAGCTTGTTCAGCAACTAATTCTTCTTTCTTGTTCCATTCCGCCTGACCCATAACTACAGTCCAAATCAAACCAATGACTTCATGTTCTGGAaTAACACATCTCAGAGCCATGTCCTTGAGATCAATGACAATATCTTTtAtAGCTCGGTTGTCGGCCAAGTTGTCCAGAAGAAGTTGTTGCAGATCACGTTTTGCTTCCTGGCTAGCCTGGGCCTTGtGCAATTTGACTATGTCTGCCAAATCGACAGCCTCGAATACAGATCGAAAGTATTCCTCTGATCTCTTGTTCAGTGG

>contig00594

ACCAaCCTCCCATTCGGGATCGTTCTTCATCAGCTCTCTTTCCTCATCTCGGTTCTTTTTGAGTTGTTTCAAGAATGCTCGATCTCTTTCGGCAAGAAGCATAGGAAATATTGCAAATCGTGCACTCTTCATTTCCTCTTTTTCATCCTTAATTTtCCTGTAATTCAGTCCGTAAAAATAAAaTCCGATGAATTGACTCGCTACGAATAAGCCGATGGTAAGTGGGGCTGTCATAAAATTTCGTATTCTTATTCGCTCCGTTTGGATCGGGCCATAACCTCCAGGAGGAGGCATATCCTGGGGCCTCGATTTAGCTGCTGTTGCCATTCTGCCCTAATTTTCCCTTCTTATTATCATAAAGAGTAGAAAATATACGACCTGGACATACCCGCCCAGGAAGAGAAATAAACTCTGCCGATTCGGCGAAAACCGAAAAGGATTTAAGAATTGTTACCACGGGAGAATTTTATTACAAAAAaTATGTTTTTATCTCGATGATCGGAGCTCGTAGCAGAAGAAAGGACAAGAAATC

>contig00595

ACCCGATTCTACAAGAAGGCTCTAGTAATGGAACGCGAGTCGAATCCTATGCAAGCACTATGCCGCAGCGGCTGTGGATTTTATGGTTCGCCGGCAACAGACGGCCTCTGTTCGCTATGTTTCAAAGAGAACCTAAAGAAGAAGCAGCAACCCCCCGtATCAGCCACCCcTGTCTCAGCTTCGCAGACCGTTTCCAGTAACGCCGGCACGTTGCAAAGCGGTTTCGGCAACCCAGCCTCCACAGGAACAACCGCACAGCCTACCATTCCTACCATCCCCCAAACcaCGACAGATTTACCCGGTCCTAAAGAATTAAGCAGAGACGATCAGGAAGATGAAGTCGGAGTCAGCAGTGGAGCTGCTGAAGGGTCGATCAGTAGCGGaGATGTGGACGACTGTTTCGATGGAAaaGAGACTGACAAAGACTCCAAAAAGaGAAAAACCGCTGTGCCCTTTGTCGCAAG

>contig00596

GACtGATAGAATAAGGTTTttCTTGTTGCGAGAATTGTTCATCGGGTTCCATTTtGACCATGCCAGTAGATTCAGTGAGTGTAGCCTCaCATGCGCTGAGTGCACTTTGCAAGTGGTGCAGTGCCGCCTCTGTGTGTTCGGTATTTAAGTAATTGGGAGTATTAACTACTCCCAATTGAGGTGGCGGAGGTCCGGTAGAAATaGAGAGCTGAGGaGAGCTAGTGATGCTaGTAGAACCAACATTTACAGAGGTTTCCATGAAGCTAGTGCTGGTGGCGTCATCCGATGTATCGTCACCCTtaTACGAGCCaGCATCATCCTGCTTTGTAGGCGACATTTCGGCGGGAGTAGTGGAGGAGTtACCGAGTtGAGAATTCACAGTTTCATTCAAGGAACTATGTTCCTTGGTTGCCTGGTTAGACAAACCTCGAACCTGGAGAGCTTCAGCTGCCGACATAAGCTTGTTAATTTGTTGCTTCTCAACGTAGACTTCCCCTCGaTACATGAATTCAACGAGAGCCTGAAGCTCCCACATACGCATATCCCTCAAAAAGATGATGGGATGTGT

>contig00597

aCAGAATTTATAGTTCCCcATTTAGAAGCTTTTtATCAAATAAAAaTTCACCAAGCGGTCCATGTGTAGACATCATTTTTCCCAGAGTTGAAATTTTtCCAGCTAAATCACGTTGTCCTTTGTATTGTTCGTCTAGGAAGTCTCCAGTTAGGTAATCAACcAAGTGATAATCGTTGAAACTACTGGTTTTCGGAGTTTCACAGGTGCGAATAATCTCTTGAATGCTCTTCGTAACTTGAGTTTCTAAaTTAAGAGCTTCAGAAAGCGCTTCAACGCCATTTCCCCAGTCCTCTCGTATTGGTCTCAGAGGAAATTTGAGAAGCTTGCTAACGTCACTTGTGAGCTGGCCACGCATAAGTAAATATTCAATTACTTTTATTGCATGTTCTCTTTCTTCACTGGCAGCTTCTATGAAGAGCTTAACGAATCCTGGTCGGTTTACAGAGTCACGGGCAAAaTGTGCACCCATAGCAAGATAAGTCATGGCAGCTTCAATCTCAATTTtCACTTGATTCTCCATAACTTTCACGCATGGATCTACCATATCAATCCATGTTgAaGGGATATCGgCCGGCTTCAATgT

>contig00598

TCTGAGTCTGGgAaCaGATCTGTGGGCCGTTGGAGTGCTGACATACATTCTCTTGAGTGGTGCCtCtCCTTTTTTGGGGGAAGACAAGCAGGAAACGTATGCGAATGTaGCCGCGTGCCATTATCAATTCGACGAGGAATATTtCTGC

>contig00599

CAAATGTATTGGCATTCTCAATTTTTTtCGCATTTCCCAA

>contig00600

ACTTTTTTGAACTCCTCGAATCCATTTTCATCTCCGATTTCTCTGTTCGGTTTACATATATCAGTGTGCTTCTTTAATCCAATTACGGGAATTTCAGAGTCACAAAGACCACAAACTAGGATGATATCATCTTTGAGCAACCCGAACATTTTGTTTAGACGAAAAGATCAATTTCACCAGATTTTACGCTCCACACTT

>contig00601

ACTATTTTTATGCGCGTCTGCTTCTTTTATTTTCAGCCATTTCATTTCTTAGCAGTTATTAAACATCTTATTTTTTTTATTTTTCtATTCTTCAGTGGATGTGTGCAAGAAACTCGATAGTTTTAtCAGGGTGTCTAGCGAACTTGAAACCCTTGAAAACCTGGAACTCTCCACTACATTTCAAATATTTTAAAACaTTTCACAAaTATTTCAAAAGATTTGACGAAGATTTCAAAGATTTCATAAATATTTTGAAACTTTTCTTGGTAAATAGGTATAGTGAACCAGATTTCTAAGATTTCAAAaGATTTTACACC

>contig00602

TTTtAAAAAaTGACGTTTGATTTGATGGCGGGAAACGTTTATCAaCGCATTCTGTCTG

>contig00603

aGTGTAGAAGGAAGCGCAGAGAAGATAGGTTATAGCAAACCTCTGCCTCTTGTTACCGACATCGAGCGAGAAAATGGGTTCTTATTCCGAGGATCAGCTGGCTGAGTTCCAGGAAGCTTTCCAGCTTTTCGACAGCCGTGGTGATGGAAAGATCCACGTTGCACAAATCGGCGACGCTCTGCGCGCCCTGGGACAAAATCCGACCGAATCTGACGTGAAGAAATTCACGCATCAGCACAAACCTGACGAAAGAATCAGTTTCGAAGTTTTCCTGCCAATCTACCAGGCAATTAGCAAAGCACGCACATCTGATACTGCTGACGACTTTATTGAGGGTTTACGTCACTTCGACAAGGACGGAAATGGCTTCATTTCTTCTGCTGAACTCAGACATC

>contig00604

ACTtccAACACGGTCAGATTGTCGTTACATTtGACCgCATAGTAtGGATCAACACGGGgCAATTTTTCCTtCCAGATTTGATATTTCTGCACAATGtCtCCGATGTCGAGGACATAGAAGGCTTCCTCCTGGAGACCGCTCATCGCGATGTCCCTGATCACGGTCATTGGGCTAGACGCATTGTCCAGCACGTGGATGCGCTCATCTAAGTTTGTTACCTTCATTATGAGACTGACTGATGGGCTAGTTGTTAGTTTAATTTCAAATGAAAaCtCTGTAGAAGCTAGGCAgCAAAGAGGCAGCTGTCAGTGAGAATTGCGAGAGTCGTGAGGAGGCGGGTTAAAaCAaCGAATCGgTTGGGGgTGCTAGCAAAGCCACGGAGGCTCTGCGTTCAGGGCCAACTTCCAGTTGCCGCTGTTGCTTAGAGATCCTACAACAGACTGGCCTACCAGTCTATCGCAGATAGAACCCTTGTCAAATGATCGCCACCCTGCGGGCCGGCACTCGGTTATTGGCGGAATGATGGAAGTTTtCGGTGACTGGATCCCAAAAATCAGTCAGGAGATGTGCTCAAGTGACTCAATTTtCTTTTTGAACGAGCAGGATGCACGAAATtCACTCGCGAATTGTTCACAAAATTGAACGGGTAGAAACATTCGAATGAGCGCGCACGAAGTGAATTACGCTTGCCTAGTGGACTCGCCACTATTTGAGACGTAAATCGACCTTCGTTGGGCGACTTTTCGAATGGCAGTTTCGCGAAAATTCGCAATCACAAATTCCACCGAATTTTCACCACCACTGAGCGGCCATATGCGCCTCGCAAAGGGccCcAa

>contig00605

ACTCACCTTCGGTGGGTCAGGAATTCCATCTGGAaTAAaCATTCCAAACTACGATGAAATTAGGCAGTCGGAAGGCTTtAAGAATGTTtCCTTAGGAAACGTCATTCCTGCCAATATGAAGGtAGATGTGTTGCCATTCTTGTCTGATGAAGATCAGCTACTGATGAATAAGTATCGGGTC

>contig00606

CCcACtcACGGACAAACcGATCGAGAAATACTtCCTGAAaGGGgAAaCTTATGATTCGATATTCGGTTCCATGGGCTCAAGCTATGAAGAATGCAGAGCCGAAGCGGTTGGACTTtATTTGAGTCTGGACAAAGATGTTCTGAAGATCTTCGGTCATTCAGAGAaGGAGGCTGATGAC

>contig00607

AGCAAACATAATAACTGGTGCATTAGTATTTCCTtTtGTCACAACTGAAaTGACAGACGCATCGATAATTCTTAAttctttAATTCCATAAACTCGTAACCTTGGATCAACAaCAGCTCCcGAATCTTCC

>contig00608

GgTATTAAATACACTAAt

>contig00609

TTttCTtCTCTGACGGCATTTGATAAaGCATATtCATCAAAGACGTCCaTTATAGTGATtGGCAAGTT

>contig00610

ACTCCATCTCAGATTCAATAACGGAATGATGGTCACATGTTCGAAGGATCGTTCAATAGCCGTTtGGGATATGACATCACAGACGGAAATtGCACTGCGACGCGTTCTGGTGGGCCATCGTGCAGCGGTGAATGTTGTTGATTTTGATGAAAAGTATATAGTGTCGGCTAGTGGTGACAGAACAATTAAAGTGTGGAATACGTCCACTTGTGAATTCGTGCGAACGCTAATCGGTCATAAGCGTGGTaTAGCGTGTTTACAaTACAGAGATCGATtAGTGGTTAGTGGTAGCAGCGaTAATACGATCAGACTATGGGATATTGAaTGTGGTGCGTGCTTGCGCGTCCTAGAAGGtCACGAAGAACTCGTCAGATGTATCAGGTTCGACAGCAAACACATTGTCAGTGGAGCTTACGAT

>contig00611

CCAGATCGTGTCTTCGTCGCATGACGACACGATTCTCATCTGGgATTtCTTGAATTATAATCAGGACAGTTCGTCGAACAGCGGCGCGGCGGCACACATTACATCTGACGCTGTTAACCTATCGGACACCAGATcTCCCTCTCCATTTGAGTGACGTTTCAACACAAAGATTTCTTCATTGTGATATAACTTCAAGTGGTTAGTGAGTGAATCGAACATGTTCACGGCTATGTGCGCAGAGAACGAAGAACCAGTGTCCAATGTGAGCATCGTTTTAATCAGGCGCATTTTCATATGAAAAAAAAaGAAGAAATCATGGCCAATGACTTTTGTTTCTTGCTTGCTGTGAAaGGCAGAAGAAATGATCTGGACTGTAAaTCATTTCACGAAA

>contig00612

TTgTATTATATACGTTGGGAAAATCGTGAAGAGAGTCTCGTTCTAGCATTGAATAAATAGGGAATATTCGTAAACAGACACACTAAAATTTGTATACTGTGAGGCTATCTTAAACAAAATCCAAAGAAGTTGCGTTCAACGTTGTCACTTCTTCAAGATGCGGGGACCAGAAATGTTCTGTTTTCTACTAATGATTGCAAGTTTATTGACCTACGCCATTTCCGCTGCTAATGCTACACTTCAAGTATCCGATGAGTGCGTAAATTCTTGCTCAGGACGAGGAAACCCTATGTTATGTGATCAGGTAGACGGTTGCCAATATTACTGCGGCGACGGTTGTGCGAGAGGATTAAATGTTGACACTGACAAAGTATCAAGTATTAAATCATGTAAACCACAATACAATAAATGTCCTGGACACTAATGCATCATTGCCGCGAAATACTGGATTGGTCAACATCGTACGAAGATTTTGAAAAaGAGATATCAATTTAAACATACTGCCCGTAACTGTTGTCTTCATGTTGGACATTAATTGATTTTTAATTAAGTTGCAGAAAaCCGAAATTTTAATTATGCTTCTGTATACCTTCAAAAAaTACTAAAaTAAAaCGAGATACAATTCTGTTGGGgATTTGAAAAaTACGCGAAGTGACAAGCATAAAGCATATTTGCAGAGACATCTAGTGTATAAATTTTGTATTCATGGTTATGTAACCCTGTTTGCACAGTCAGTCGTTTGCTAGCTTCCATAGGATCAAGTAACTTTCTTTCAGTTTtGTAACAaTATACGGAAATCT

>contig00613

ACTGCCTACGCTCGAAAaGCTGTCAACCAGGTCTTCCAAATGTGGAAAGACGGAAAAGTGAAGCCCGTGGTGGACTCAACCTGGGCG

>contig00614

GAAGGCGAGAAGAAAAaGGAaCCGGAATTGACTAACGGAACGTCCGAGG

>contig00615

ACGAAGtttatGGtAAAGTTGGTCACCCGTAAAGCaGGCGAAaTCCAGCTCGACGGTTTCAATCCTTCAGCGGAAGAAGCTGATGAGGGAACTGACGTAGCTGTTGAATCTGGAGTCGACGTCGTTTTGAATCATCGACTTATGGAGACTTACGCATTTCACGACAAAAAGTCTTACACTCTCTACTTGAAGGACTTCATGAAAaaaTTGGTAGCTAAATTGGAAGAgAagAatCCaaGTgaagTCGaaGTCTTCAAGACGAATATGAACAAaGTGATGAAGGACATCTTGGGCAGATTTAAAGAGCTTCAATTCTTCACTGGAGAATCCATGGATATCGACGGATTGGTTGGTCTCaTGGAATATCGCGATATCGATGGAGAATCCGTGCCGGTTCTCATGTTCTTCAAACACGGCCTGGAGGAAGAGAAATTCTAAGCAATTTTTAG

>contig00616

GAATTAGTTTCGCCCAGTTTCGCCGTGTAAAGCTGTGTAAAGCTATAACTAAAAGTGTTCGTTCGTCGACGAAATACCGCACGAGACATAACAAAATGCCGGAACTAACAGAGTTGGACAAAGCGGCGTCGTCCGAACCTACCAAGGTCGAAGCCGCAGCAGCTGGATCAGGAAGTGATTCGGATTCTGATGACACCATTCCGGAACTCGAAGATGCTGGAGCAGGAGCGCCAGTATCCTTCCCAGGATCCACGGTTGCTGGCCTTCCCATTGACATGGTTTCAAAAGCTAAACAGAGCCGTGGCGAGAAGAAAGCTAGGAAACTCATGAGCAAATTGGGATTGAAACCAGTTCAAGGAGTCAACAGAGTAACAATTCGCAAGTCCAAAAATATCCTCTTTGTCATCAACAAACCAGACGTCCTCAAGAATCCAGCTTCAGACACTTACATTGTCTTCGGTGAAGCCAAAATTGAGGATCTTAGTCAGCAAGCGCAGGTGGCAGCAGCCGAAAAATTCAAGGAaCCACCAGTAaTTGcAGCAACCGAAGCAGGTGGCAGCACGACGGTGGTGGCACCAATTCAAGAGGAGTCTGAGGAGGAAGTAGACGAAACGGGCGTCGAGGAAAAGGATGTTGACcTCGTTATGTGCCAAGCCAACGTTTCTCGAGGGAAGGCAATCAAAGCTCTTAAGAATAACCAAAATGATATCGTCAACGCCATAATGGAGCTGACGATGTGATGAAACTACGTTGCAGCATAATAGGCTACGGAGCTGACGATGTGATGAAACTACTTTGGAGTGTAaTaGGCTACGGT

>contig00617

ACttAtGGGgaTTCATTAGCTACACAGTATCACGAATTATTCCAAAAAAaTATTTCCTCTAGCTGGAAACAATGAGGGTTCTTTGCACTCTGTTCATCGCTATAGTGGTTTTATTGGATTCTTTTGGTGCCGAAACTGACTGGACTCCTGTCACACTAAAGGTGGCTGAAGCAAAAGATATAAAGGACCTCATAAAAGATGCTACTTTCGGGACGACAGCTGAACTTCCTATGAATCTTCTACTACTAGATGGTAAACTATTTGGATGGATGGACGCTCAACGCAATTCTCATGCATTCTATAAGAAGGATAAGAAAACTACGGGAATTGTTCAAATTGCAGCAAAAATAGCAAAAAaTTACATAAATAAACAAGTGCCAAGCACTGTACAATATAAAACTAATAGCGTCAAGTGTTGCGGATGGTTTTAATAAGAAGGAAAGGCTGACACCATGGACAAAATAGATGACGAAACGGAAGTGGCAGCAAAGCGAAAGCATTGAATGAAAATATTAAGAAAAATTTAAATTACATAAAAAATTTCATTTATTGTGTTTCTGAACTGTCTTAAGATGAATCACTGGATGGAATCTCACTGATTTTTTGTGAAATCCCACTGGTTGTAACAGCTAGAAGTGTGTCACTGGCAGTCAGTGATATTTCACTGTTTCAATTAGTGGAATGACGAAAACAGTCAAATAAAATATTATAAAATTATTAAATAAATGATTACCTTTAACTAGACATGTCATAGACCTAATAGTGAAGTATTATAAAGCTAACTACACTTGAAATTTGGAGCAAACTATATGTTCTATAAATTGATAGCATCATGCTTTAAATTCTGAAGTGTGATGCTATGCTTGCAATCGTAAAAAaCTTTTTAAaTTCAAAGAaGGA

>contig00618

ACCAATTACCGAGTTAAACTCAAAGGAGGATCGGAAATCACACTCAAGGAAGCTGATATCAAGCCAACGAGTAGATCATCCAGAAGTGCAACGAGGACGAAAGGACGACCAACGAAAAAGAGCCCTACTCGAAGTTCGCCAAGTCGACGTTCTCCGAGTAGAAGGTCTCCTGGTCGACCCACGAACCAGAGTAGAAAATTGCCAGTTCGGAGTTCAAGACTTGCACAAGTTTCACCTTCCGATGATGATAAAACAGAAGGTTCGAGCGAAACTAGCGCAAGTATTCGTGATGAAGACCCAGCAGAGTTGGCACCTCTTCAGTCCCGACTTCGCGATGTTGGGCCAACAACAAGACGATCGATAAGACTCCTGTCGAATACTGCAAAAGCTGAACAGGAGCAAAAAttAATTATGCTGACGaGAACCGTCGATCGTGCTGCTTCTC

>contig00619

AATTCCGCATTTTCATCCTGCAGTCTTGCATTCTCGACGCCACAATAAACGTCTATTTTATATTTTCTTCTATCGAATCTAAAATTAAATTGAAATCTTCAAACCCCTAAGGCGTCAATAaCGATGTTTATTGCATTGCAGAATAGCATCCATTG

>contig00620

AaaaaTGATTACCCTGAACGGTGGATCACTTGGCTCGTGGGTCGATGAAGAACGCAGCTAATTGCGCGTCATATTGTGAACTGCAGGACACACGAACATCGACATTTCGAACGCACATTGCGGTCCACGGATACAATTCCCGGACCACGCCTGGCTGAGGGTCGTTTATAAACTAAAAaCTGCTTAtcGCCAAAGTATATGTATTATTATACTTTGCGCGATACGAGCGAAATAATGAACGTTCGTCCGTCTCTGAATAAAATTTACTTTTtATTGAATGAGTTCAGGGCGTCGTTTGAAAGAAGTTGAAAaGCCGCCAATGACCGTCCGtCTGTGTGTTGTTTTTGTTATATGAGATTTATCTTGTATGCATATATACGCACAGATGTTGAACGCAACCGCTTGCACAtACTCTGTATATGCGGTCGTGGATCGGTCGTCGAGTCCTGGAGCCTTAAAAAaGCACGGACAGACGATATTAAT

>contig00621

ACTTATCACGCGAAGGAAAGGTAGTCATTGCTGGTATTGCCGAAACGAAAGACGAATCACAGGCTGATAATAAGGAGAGTGGTTTCAGCGTCACGGGAATCAACCTTCGAGAGTATAGGAAGAATAACCATGAATTATATCGCCAAGCTGGCGAGGACGCCCTATCATATTTCGAAGAGGGACTTATCAAGCCATCATGCTCGTTAATAGCTGGTCTTTACAAAATTAaTGAGGCGATGAAGTCAGTTACTAATAAGAAATCTTGTGGAAAGGTT

>contig00622

ACAGGAAGAAtCGAGTCCtGtAGGACCctGGCtCTtGGCACTTTTTATTTtCGtGGtGtGtGGTtCtGCAAtCTTtCAGATAATACAAAGTATACGGATGGCGTAATTACTCAAACATGGAAGACCTGTCTCTAGTAaTTAATAAAGAAACTCTACCATCTCgTTtAAACATCATCATCTATCACTATTTTAAAAGTTTtAACgATGGATTGAcTTcAGGATTCTCGAACTCAAGATGACGCTTAAAAGGGCTGAGAAATGTGCCGTTTCCCCTTTCATCAGCTCAGAGATTTATTTATAAAaCGTAGCATTTATAAGCGTTTATTATGGATGAAGGAaCTTTTTAACGTCTTGCAATTTGGCACGTCGATGAAAGAATCTCCAGA

>contig00623

ACGAACTCAAGATTCGATGAATGGCGGTATTCGTAAGAATTTTGATTTTTAAGAACTTATCTTCTAGGATACACTATGACTACAATTTTTCATCGAGAGAATGATAGGTGGACctGGTCTCGGGTATACGTTTGTAAGCACACAGTATTTTATATTGTATGATTTTCGAATTTGTAATATTGCTGAATGTCCTCTGGATTAGGAAAAAGCAATTTtCACTTACCCTTTTTTTtCAAGtCGAAATAAATAACTTAAATTAATGAATACTGaTGTTTACTCTGATGTATAGCTGTAGAGCTGAAAAAGGCTTAGATTCTAGATACTCTAGTATAAGCGATTCCAAATtCTTtATAGTCCGTTAagAATAATCTTTTCAAAgTCACTGATATATTGTGCGCGAATATTTTTCCTACGTTTATACGACATAAGTTaGAAGCATTTCTTTTAAATTATG

>contig00624

GAGGAAACAACAGAAAGAACAGGAGAAGAAACAAAAAGAACAGCAGAAAAAACAGAAAGAAGATGAGAAAAAACGAAGAGAAGAACAGATGAAAAAaCAGAAAGAACAGGAGCAAAAACAAAAAGAACAGCAGAAAAAaCAGAAAGAACAGGAGC

>contig00625

ACTTATTATTACTTTGATGCACAAAATTGGAGGAAGGTAGCTAAGGAGTTCCATTTAGATTATACAAAACTGGAGAATaGACCACATCTTCCTCCCTCGTTCCATCCGGCCCaGCCTTGACTACAAATCTGCCTGCAGACCGTTGTGCTGCATCTAAGCTGGAAGCAATTCAGCAATTGTATAAATgAaCAAATTTAATAATCTTTAATAAAAACAAGAAATTCAATAAGTAATCAATAAAAGTCCTTGCTAAGAACGCCAATCTACTTTTCCATATCGTAGGCGACATCCGAGTCTTCGAAGTTCCTTTtGCCTCCTTTTTTTtATTTTtGATTTCTTTTTCCGCAAAaGAaCTTTTTTAAAtGCACTTTTTTtCAATTAGACTAGAATCAGCTTCTCTGC

>contig00626

TGGGggCATTTCTTCTTCTGGACTCTCCCGAGTCGGAAGCCAGCTTTTCTCAAGGAGGTGCGACGCCGGTATATACAAATAACAGACCAGAAAAGAAATTTTCCTAACATTCTAAGAATATCAGGGCGTGAAGCACCTGGCTCGTCAAAATGAGGCCAGACGATCTTCTTCAAACCGAGAGGAGGATGACGGAAACAATGTCGGAAACATCGGAGGGCACGGGGACCACAAGCATTTCCGGTAAATCAAATTTCGAGACCGAAATGCAAAATTTCGCGACCCTCCTGGGTATCGCGAATCCCGAGGAT

>contig00627

GACGCAAGTTGGAGCATATGCTTCTCGTTGACAATGAAACTACAGAAGCTGCCGAAGCATTTTTCCAGAAGGTTATGGAAGAAACGAATACCTTCGTCACATGGCCTTCCCGACTCAAGATCGGAGCAAAGTCGAAAAAGGATCCTCACATAAAAGTCGCTGGACGACTCGATGACGTGAGGGCTGCCAaGGAAAAaTCATGCAAATTTtGGACACGCGGCAAAGCAACAGAGTTACCATGAAGCTCGACGTTAGCTACACGgATCATTCACATATTATtGGCAAGGGAGGCTTAACGATAAAGCGCGTAATGGAGGAAaCAGGTTGCCATaTTCATTTtCCGGACAGCAACCGCAGCAATCaTCAGGAGAAGAGCAATCAAGTTTCCATTGCTGGCGAAATGGAAGGCGTTGAGCGGGCTCGCgCACGAGTCAGGAaTTTAACTCCGTTGATCTTTtCATTCGAGTTGCCGATAATGGGTGCGTCACAAGCGATGCCAGACTCCACGTCGCCTTACGTGGTAAAGATTCAGGaaCAGT

>contig00628

ACTGCCAGGAAGTGGCAATGTaTTTTCCAGCTCCGTCTTCGATCTGCCGGCCGAAGAAGAATTTGAAGAAGACAGTCTTGAAGGATTCCCTGGATCTAGTtACTTCCCCTTCCGTTCCAATGTCTTCGATGGATTCTTGTCCAGTATGCAAAATgCCATGAACAGACTAAGGGAGCAGATGGCTGGTGTCTTAGCAGGTCAAATTGGACAAGGTATTGGACCTTGGGGAAaTGTTCCCGAAGGAGCGAATACTACTTCCACCACCAAGATCATTGACGGCCACGTCGTGACCATCAACGAAACGACCTACACAGACGGTGACGAAAatAGCGGCGCGATATTCCGTGTCAGAGTCATCGATGTTAAACCCcAAAaCGAAACTCTCGAAATTGAAGGCAACCCcGAGGCAAAACCAGTGATTCCATCAGATCCCAAAAaGAACGTCGAAAACACAACTCC

>contig00629

AAGATTATAAAAGATAAGCTAATTGATTTATTTGTAtATTGTAATACTGCATTTTGATGAGAGACAAATCATCGGATTTTTAGTAGTATAGGTGATAAATTATTATAGTATTTTtGTAGTTCTATTACTATATTGTtAATTTATTATTTTAGAGCTGTTTaGT

>contig00630

GATCCACACAAGACGACAAGAGACGAACACTGATCGACGGGCGCGGCTAGCGTTTCGGCTGGCGTTTTTCACGCAAAAATGTCGTCCGGACTTGTGCAAATGTTGGATTTGTGCCGGTTGTGTCTCGTGAAAGATCGTGTGGGTGTGCCGATATTCGAGGATGAAGGAGACGTGCGACAAGTTTTCTTCAAGATCGCCGCCTGTCTGCCCGTCAAGGTGTCTAGAGAAGACAAATTACCTAAGAAAATATGTGACGACTGTATGTATAAACTAGAATTATTTTATCAATTTTGGAATACGACGGCAAACGCCGAGAAGCAGCTTTTACAATGGCTCGGCGAAGTCGAAATGGAtGACAAACAGGGTTACGTAACGGGAGTTCTCAACCCGAGCGTAATGAAGCAGGAGCAACAGCAGCATCAGCAGCAGCAACAGGCTAGTGAAAATAGGTTAGATGGTGGGAGTGTTGTTGTGATGCAGCAACAGCAGGCGCATCAACAACAACAAcAAAaTGTGGGTATGGTAATGATGGACAGTATAaGTGGTGGGCTCGGAATGCCCATCATTATCTCGACAGCCAATCAACAACAACAACAAATGACCTCAGTAGTAGTtCCTATGGACACGAGTGGCGGCAGTTCTGTCCAAACTGTTCAGCAGCaGCAGGCTCTGCCGGGaCCCaGT

>contig00631

TACACCTGGCATCATCCTTCGGTGGCAAtCGTCGAGAAAAAAaTCTTCGCCATGGCAACCTCTTAAGtATTTCTtAAGCAaCTTcTATAGGGACCGAGAATGCGTTGGGAGGGGAtAGAAGAATCGGTCGTCGCGCTCGCAGTGGCAAAAaGTGgCGTAAACGGAGTAAGATAAATAGGCAGGCGAATGACAAATCTACGAAATCTTTtATtATTGAAAaTAAAACCGAAGGAGGgCAaTAATTtCCATTGAAACTTATTGACGATATTGATGGTC

>contig00632

ACATTTtCAAATAGCATGCACTATTGCAGCAATAAATGAATGCCCAATACCAATCAATATTGCTATGCAAAAATGCAACACTTTCTTTAAATATACTGAGTAAGCGGGgTTGTGCACAAAAaTTTAATTAATAATTAGATAATTCTTCATATATTTATTTATATTTACGAAAGGATAATACAATGGCTGAGCAAGATTGCTACAATTTtGACCGGAAGATTGCATTCATATATTGCTACAaCATTGCcAaTAGTGTGATGCAACATTGAGtGTTATTGGGgCGAATGTGAAATCCAAAaTTTCCGATATTTTGTGTGTCTATTTtCAGCGCTCAATTTCGCAATGAAACTGCGCAACATtCAGCGCCAAAAAGAAaGACATACATTCATTCTCAGTTTtCGATCCcAC

>contig00633

ACTCCGGTTCAGCAATtGGctCATCACACTGTGACTGGCTGCAATATAAAGCCAGGTGATTTAATGGCTTCCGGAACGATAAGTGGAGATACCCCTGATTCTTACGGAAGTATGCTTGAATTGAGCTGGAAAGGAaCTCAGCCTATAAAGATGCAGGACGGGaCAATAAGAAAGTTTCTTCAGGaCGGCGATGAAGTTATAATGCATGGTTACTGCGTTGGCGATGGATtCcGCATTGGCTTCGGTTtGTGTCAAGGAAAAGTATTACCAGCaCAAAAAAATGAAGACTAGGAAGGTCCAAAAATCCGAA

>contig00634

AGTAATGGAACATGTTTACACTATTTACAGCTTACGCATCACCaTAAGCGATTAGATTCAAGGCTGTAATATCATTCGCAAAGACAGTGCATATCTCGtAGTTTtAACAGATATAACGCaGAAAACGAACTCACACCGTGTGAGAATGCACTCATATTTCCGATACGGTCTCTTGCCTAAACTTACACCAATTTCACATTATATACGATAGAACGCGTAACATAaTGTTCATTGTCGGTGTGAAATAAAaTTaGGCCGTTAGTAAATGCAGAGGAAATGCGAG

>contig00635

GTATCAGAATTAAAAAtCAAAACAAAATTTATAGCAACCCTTATCGGCACTGACTGTATGCACTGTTGTTAAAtGATATTtAAGACTTTAAGTCATTTTGTTAACCTAAAATTTGTTTTtGAGTTTGGgTCCAATCCTAACGCGCTGaGAAAAaaGTAaCATCTTAAAaCTGTATCTAAAaCTTAGGGaCGTATCCTTCGTGATTTCGTCCATCGTTTTCTAATGTCGGCTTTAaTTTTATTGTGT

>contig00636

ACTGTTCTATGATTGCTTTACTACTTTTCGTGGATTTTGGTATCGGCAGATTTAGTTCTTGAATTATTTTTGAATAGATTGCACTCGCTGATTtCATAGCTGTGCAGTTGATATACACGGTTTTAAaCGAGGATTTGTATTTTGACTTTGCCATAATTGTTGACAAAGAAGCGGTTTTTCCGGTGCCCGGTGGTCCGgAAaCATAAAGTGAACCTGAAGATTCTTCATTTAAGTGCTCTTGAATGAAATTTTCAAGCTGTAATAATTCTGTTTCTCTGCCGGGTAAACTTTCAGGGACTGAACTGTGCAATGCTTTTCGAGCAT

>contig00637

TTTtCTgTATCTGAaTCTGACTCGGATTCACTGCTGCTCAAAACCATTAGTTTTTTCGTCGTTGGTGTATATGTTGCATTCGGaGTGCTtCTgaGAGAAGCATTTGTAAACAATTCCTGCTTTGGAGtATTTTCTTTCGAAGAaTAAAACGAACATTTCTTTCtAATATTAAAGGGAATCGTCGTTTGGATACTCGTCATGTTGTCTAGAGAATATACGCTTCCTTATAAAaTTAAAAGACCGTTGCACTTTTtAACTGTTCAAATTGTGCAGcTtCGTAACACAAGTTATTCGTCAAAACCCGGATAAACAATGT

>contig00638

AGTCGTGaGCCGAGAGCCAGTCGgTGACGGgTTGTGTCTGTGTCTTCTTAGTGCGGTTAGTAGCGAACAGTTGAGTTTTTACCCCCCTGTGTTCAAAAATGAGCTGGCAGGATTACATTGACAAGCAGCTGCTCGCCTCGAGATGTGTAACCAAAGCGGCTATCGCCGGGCACGACGGAAATCTCTGGGCAAAaTCGGAGGGCTTCGAAGTGAGTAAAGAGGAACTTGCGAAGCTGGTCAAAGGTTTCGAGGAGCAGGACATTTTGACGTCGTCGGGAGTTACCTTAGCTGGCAATAGGTATATTTACCTGTCCGGAACAGATCGAGTGATTAGGGCAAAATTGGGAAAGGTTGGAGTTCACTGCATGAaGACGAGCCAAGCGGTGGTTGTATCCCTTTACGAGGACCCCATTCAACCCCAACAGGCTGCCTCCGTCGTCGAAAAGTTGGGCGACTACCTCCTGTCCTGCGGCTATTAG

>contig00639

ACcAAGAAGTCATAGCTTGCGTTCTCATTCCAGCAAAAaTTGATTGATGTCGTTAATGAAGAaGAAATGTTACTCACCAAAaTTTGCGCTTAaTTCGATAATGTGAATGAAAGTGTAGTGATATTTCGGTTATAAGtGTTTTATTACGAACATTTTAAAGATGATTAGGAAATCGATATTTCGAAGGTTAGGATATTTTCCCTGAATAGCTTTATAATTTACTACACCGTTTATTAATATGATATGTAATATCTTGAGTATTTTTTGCG

>contig00640

ACCAGAGCAAGACGCCGAGGCACGAAGAGAGGCATCGTTTTACAGAGACTGAGAAAAAGACAGCACAATGAGCAAAACTAAGGATTCTCCCGCTACCGAAACTGAAACAGAGGAGGAATTCAGCGTCGAAAAAGTCTTAGACAGGCGAGTGGTGAAAGGAAAGGTGGAATATTTCCTAAAATGGAAAGGATACTCGAACGACGAGAACACCTGGGAGCCAGAAGAGAACTTGGATTGTCCAGATTTAATCGCCCAATTTGAAGATGCGAGAAAGAAAAAGGAGGCAGCTGCCGCTGGAAAGAGGCACGAAGAGAAAGACAAGAAGAGAAAAAGCTCCTCAACTCCCACGCCGACACAGGCCAAAAAGAAaGTAACTGAAGAGAAGAAAGCTGAAGGATTTGATAGAAACTTGGACCCAGAGCGCATTATTGGTGCAACCGACTCTAGCGGCGAACTCATGTTCCTGATGAAGTGGAAGGGAACGGACGATGCGGATCTTGTGCCTGCAAGAGAAGCTAATGTGAGATGTCCACAGATCGTAATCAAATTTTACGAAGAGAGGCTGACCTGGCACAGTCCTGCTCATGACGAAGAGGGTTCCACAAAACCCGACCCAGAGTAGCCCAGATATtCCTTTTTTtGTCGTCGCATCTACACAGCATGTTTtAGAATTTGTCGCTTTtAAGGCGATACGTCTAAATCGtGGTTTTTTTTtCTATCTGAAGCAAGCTTAATGTATAtCTTTTGAACGAGAGGGCCCAAAAACAGAGCACATCGGTCGTATTACACATTTtACCATTTTtCACGTAACTTTATGTATAATTCGTTATTGTCCCTCATATATATATAGTCAATTCTTCAATTTTTTTTTTtttCCAAGTTTCAAGATTGT

>contig00641

CTAGAAATATATTAATTTATTCATTTATATTGACAATTAAGACAGATTAAAATTTGcAAaGTCTCGCTAAATTCGCCCATGATTGGATTTTCATCGAGTCTGATTCcTTtCTTTTTCTGAATGAACAATTGCTCTTTTTTCAATAAACAACTGTCAGTTGACCCGTCACCGATTAAAAAaTTAAAtCCCGTCGCGACCCGTATTATTTGAGGTTTAAAACGTAAAATACAGGAGCGAGGAAACGAGTAAAAAAAaTTAAAAGTGCTGGGAAATTTtcTTAAAACAACAGCTTCGGATTGATTTTTGATTTCTCCCGTGGAGAGAAATTTGCTAAATCTGAGATAATTGGTAAATTGAACATTTCTGGAAAAAAAATTTTGTTCGATTCAACAGGCATTTCATCTGGATtATTTTTTTTtGTAACAATTTGTGCGATTGAATCAACAAATTAGTATGCGTGGTTGGCGACAAACAATGCTGCGTCTCCTGCTGCTCCAGTTACACCTCCAGCGTATTTtCCAAGTGCCGACTCGCTGTTGGCGtCAAaTAAAAAaCGaCAGAAAGAAaGGTGTGAGTTTTAGTAGGCGTGCGATTTGACGAAGAGGGAAGCATCTCCAGCTTTGCTAGAAATGCTGCCAGAGACATATTTGCCctGACAAGCTGCAGCATTCGCCTTGGCTCTCTTGATAaGTTCGTCCTGAGCAGCACCGACTTGGTCCTTCTTTCCCGCCCAAGCACGGAGAACGGAAGCCTGGAGAGCACggCCATAACTGAAAGTCAGTGCCCAAGGTTTCTTCAACGGGTATTTGTTGATAGCATCCAAATTGACGGAAGCCTCCTCCTCGGACTGTCCACCGCTGAGGAAAGTGATTCCAGGAACCGCTGGTGGGACAGT

>contig00642

ACAAATAGAAAGGAGAGTGCAGACTATATAGTTAAAAAGTAAAGCGATAACACCCGGCTGTATaCGATCCTCGAAGCATCAATTTGCGCATTAGTAAAATAAAAGGCATGCATTTTTGTATAAAGAAAAGTAGGACAGTTGAAAGAAGTTTCAAAGGATGCAAACCTCAAATGCAAAGTATGTCTCCTGCGgTATTTTtCGAAATtGAATTGCGAACACTCAATCGATGTATGCATCGTAGATTCcTtCAATTACACACATAaTATTTAAGATGCTTTCGACGAaTACGAATTtaCtcTAtCtATtACTATCTTTC

>contig00643

TTTGTAGTATAATCTCAACAATCTTTGTCGGTCCCGATGCCAGTG

>contig00644

CGGGATGCTCCAATGCGGATTCTGGGTTCTGGTTTGCCtATtGTTTtAAATAGAcTtAAAAGATCTCAAGCGAAAGTCTAGCTCAGGCTGTGAGGGACACTCTCTCGGTGACGCTTTGGTATCAGGcGGGTCACGTCATCGCTTACTTACTTACTTACTTTGTAGAATAGTTTCTCTAGAAATTTTACTGAGAAAAATTTGCCTAGATCGTAGGTTCTTAAAAGTTATTATTGTCAGATCGTGGAATAGAATAAATGATGTGTGGT

>contig00645

ACTAACTTTTCTTTCAAACTCTTTGTTTTTCTTAATAGCCGTCTTCTTCTTTGATGTGGCCTTCATCGATGTTTTTTTttCCACGTCCAGCaGCTGGAACTCGATTATTGGTTCCTCTCGTTTGGAATGAGGGACGAACCATGGGATTAAATCCCcGAGgAAATGGGTATTtCATAccgCCTATAGAAGTCACCTGAGGCGGAAGTCTGCTACGCGGATgCCCCTGgaGAGGTTGTGGCAAACGTCCAGgTtGCCCGTAaTTtGCACCCCATTGaCCCACAACGACTCCCGCAATTTCCAACTGTCCACCCcTCGACGGCACCGGGGGACGTCGCATTAATCCGCGCTGAGCGGCGACATTCGAGCCACGaCCACGTGTCAGCTGCTCgTtAATTGGACATCCAGCAGGCATAATGTTCGGAGGCACTGCTACTCGAAGACCTGGATT

>contig00646

CGCATTCTCTCGACCTTAACGACTCGATCAATGTGTCCACCGCAGAGTTCCAGGAGATTTTTAGCAaTTGTGGCTCTTGCCAGAGTATGATGGAACAATTTTCCAtCGAAATAAAGCCAAGGGCAGCACATTAACCAAGGAAGCGGGGCCCCaCAGGCGTCATTTGCCAAGAGGGCAGTTTCCACGCCAGCCATGAAAAGAGTCGCGAGCTGGACTCCTCGGGAAGTTACAAGCGGCAGCTGCAGATCTTGGAGATACtGAGAGTTCATTAATTCTGGAGTGAAGGCATGTGCAATGAACGCGTCCAACTCTTGTCGTcTtAGAATTTtCGTTTTGTCAGAGAAAaTCATTATGTATCtCaaCACGCAGGCCATGATCAGAAATTGCTG

>contig00647

ACAACTAACCTGGATATCCTCTTtGCGTCATTTGGACAAAAAaTCACACCTCCCTTCCcTAGTTTACATGTTTCCCGACGCAGACTTGCTTACTTAATTCCTTTGATACGTGTTTtCGtCATCAAAAATCGTCCtCTTtCTtCTTTCTTTtCTTTCTTTTGCTTACTGTGTTTAATATGTGTATTTGTgTTCGTTATGTTACATGCCTCGCGGAAATTAAACGTAAAAaCATATGGTGATTTGATTGAGCTCCCCTTTGAAATTAATTTATTTATTTAAACTATTTACTGTCTTCTTATTGATCCCAGGAAATTTTTCACGAGCCGCGTCCATTTAATATTcTtACTTTGCTTTGTTTTTTtCAGCCATTACAAAAaTTACAAATACTATTCTCTTCCTCGTCGAAGGCCAGATCTGAAATCTATTTACAACTAAGTAAAAaTCTGATCATTTGTATTCTAACTTACATTTCATTTCTAAGTAATCTAAACAGTATTCTAAATAACAACAATTGTGCTTTATCAACATTCCTTTtCTAAAAACGT

>contig00648

CAGCTATTCATGAAAATCTACGGCCCTCTTCTATCCTTCAAACTTCAAGGGATAATATTaaaaTCGAAATTTTGAATCAAGATTTACCTTCAACTCCTAGTGAAGTTGTGTTACAGAACACGGCCATGGTTCAAACATTAATGCACAACGTAGCACCTCAAGCAATGGCTTTACAGCAATCAGTTTCTCCACCAGTAAGGAATACAGGAACTGGAaCAATCAGAGAACAAATATCTGCTCCAAC

>contig00649

GTATATCGCCaGCAAaCAACTATCAGATTATGTATATACAGCTCTtATTGTCGAGGTTGTTTGAGAAAAAATGTATTTGCTTAAAAATTTTTAATCTCCGGTCACGTTAGAACGTAAGCTTTCAAGCGACTGATTACTTtGCAGGAAGCGCCCAGTATAGTTAATGACATCAACACTAAAGATTTCTCATGTGAATAAACACTCTTTTCACATTTTTATTTTATTTTCCATGGACAAAAAGAAACCGAAATAACGGATTTGTTAATCACTCGTATCTTCTAATCCATTTTATACTACAGATTGAAGTATTGTGACCACACTTAAAGAGTGACACAGT

>contig00650

ATTTCCATTCGGATTGATATATGACGCTTTTCTTTGTCAAACAATAAAAGTAAATGCAATCGAGTGTTTACAAACTAAGTGTTAAACCAAATATGCGGAAAAGGAACTCGATTTCGAAGTCGCAACGTTTTTTCTTGAAATAAAAACCAGTTTTATTGCGAGAGGATCTTTGAAGCTAATGAAAACATCGtATTGTTGATTAAAACTCGTGACAAATTTCCAAAAGTATTCGAAATGAACAAGAAATGTAAAGTCAACGAGGACAAAACCAAACCTCATGGTTTCAAAaGGAAACTCCAGCCTGAGAAAaTCATCGGCGCTACGGACTCTAGTGGAGAATTGATGTTTCTGATG

>contig00651

TGGAAAGGCACGGAGGAAACGG

>contig00652

CTGGTAAATTTATTTATACAGAATGGCTAaCGTTtCAA

>contig00653

ACGTGTAAGTTACTTATTTTACTTTAATTGAAAATACAGATAAGTTCGTTTTAATACTGTTTTCTGTATCGTCTaTATTTTTGaTATTGAAaCA

>contig00654

ACctatacAGTTTTaCAGAGTATTCCTCGCTTCTTACGATGTGCAAATTACaGAAAaGAtCaTTAATCCACGGACCATTGAATAAATATtACTTTACGATTAAAGTGTTCGAaGTAGTTACGGGGTTTGTTTCCAGGGTGAAGGTAAATTTCGTATCTTTTATATTCCCTTAGCGAATTGTAAAACACCCCATAAATAAGCCGTATTTTTTTCTCAATTTTTCATCTTCAGAACTCTTAACTCGAACAATAAGCGGTTCAACTCATCATACGCTTATTTAAAATATTAAATACTCCCAGCTCGAGTCTACAAATGTATTGTGTCTTAAAGTATTT

>contig00655

ATCGCAGATTGTATAGAATGAAGAGATGTCCGTCGTTgaCAGAGGATTCAGGTCTGATTGCATTTAGTGTGTAACTTTCTAGCAAGAAAGTGTGTAA

>contig00656

ACGATTCGAGGCCGTTTTTGGCAGAGATGGTTTCCTTCTGCTTCTCATCTTCGCTTCTGAATTTCTCGGCTTCGTTCACCATCCTCTCAATGTCCTCCTTGCTCAAGCGACCCTTGTCGTTGGTGATGGTGATCTTGTTTTCCTTACCGGTTGACTTGTCGACGGCAGAGACGTTCAGGATGCCATTGGCGTCGATGTCGAAGGTGACTTCGATTTGCGGAACGCCACGGGGTGCGGGTGGGATTCCACTCAGTTCGAACTTTCCAAGAAGGTTGTTGTCCTTGGTCATCGCACGCTCACCCTCATAGACCTGAATCAACACGCCAGGTTGGTTATCGGCGTAAGTGGTGAAGGTCTGGGTCTGCTTGGTAGGAATGGTGGTG

>contig00657

TaTATtCAGAGCAGaGCGAGGTTTACCGTCGAAGtAGCGGACTTCGATTTtGGGAAAAGCAATTCGGAAATGGAGTATAAAaCTTTtAGGGaGAGATTACGAGaTTCGTTCAATAACGCTGGTtACAAACCGCTGCACGATTCACAAATTTTGCAATGAGGGCGCTTTCAAAACAAAATACGTATTCCTGAAACaTTCGCCTGTTTGTGTTaGGAGAAGAAAGTGACTCCGTTTGTaGTGAC

>contig00658

acTtcaTtGCGTCCATGGAGCTCACGACCTGAGAAGGTGCTCCGaGTCCTGCGGATATGGCTGCAGCGGATTGCTGAGCTGCTGCTGCAGCAGCCGCTGCCATACTTGCTGcTGAAAGAGAACCATAtgctGgCccatAatAagAacTCATTCCTGCTTGACCcATGCcACCAGCCATTCCGGCTCTGAGTGCATCCATTGGCACGCTGGgATAGGGAATGCTGTAAGGgTATCCCTCTTTCCTAAGAGTTTTTGGCTTTCTTCTGGGTCGATACTTGTAATCCGGATGCTCCTTCATATGCATAGCTCGTAACCTCTTtGCCTCA

>contig00659

ACTTTATCTGTTGGTTGGGTCACTAGTTACAAAACTAACAGCACTGGCAACTACACAAGCGCACATATTGAAGAGATGTTAAAAACCCTGAAGGATAACAACGTGACTCAGACAGTGAC

>contig00660

CATTGAAAACCCTTTTGGAGAAGAGTTCTAATAAAAGTTCAACACTGACTAT

>contig00661

ATGGGgACGTTTGTGTATGCATAAAAGAGGAGCGAAAAGTAGGGAAACTTATTCATTCGACGTAACTGGAGATTGAAGGAGCCTAAAAAATTCAGCAGAATGATGAAGTTGACTATTTTTTTTGCGATTCTGGTCGGTCTTGCTGTTGCCGGACCATCTCAAGACGTAATGCAGTTTGACCAGAAAATAGATTGGAAATCCGCAGTCTTTTACGACTTCTGGGCCAACGACATCGACGGAAATGTTGTTTCTCTATCAGAATATAAAGGAAAAGTTCTCATTGTCGTTAATGTCGCCAGTAACTGCTCATTGACTGAACCCAACTATAAGCAGCTTCAGGCCCTTTATCAGAAATACAAAGATCAAGATTTGAGGATTCTTGCATTCCCTTGCAACCAGTTCAAAGCACAGGAACCAGGATCGTCAAAACAAATCAAGGATTTTGTCAAAAAaTACGATGTGACCTTTGACATGTTCGAAAAAATCGAAGTTAATGGCGAAAATGCTCATCCTCTC

>contig00662

CACATTTTTGAGTGTATACGAGCGATTTtATAAAGGC

>contig00663

ATGTTAATGTCTTGCCCGTTGAGTTGACGCGGCAGACGGTGTTGCGAATGTTTAGTAAATGAAAATTTCCTTAAAATTAGAAAACTAGTTTAAAAATGAAGTGGTTTAGTCTCGGCCTCTTTTTAATTGCGTGGTGCAATTACGCGAATGCATATTTCATTACCGTGGACGCACACGCCGAAGAATGTTTCTTCGACAAGGTGGAATATGGAGTCAAAATGGGTTTGACTTTCGAGATCGCGGAAGGCGGATTTCTAGACATTGACGTGAAAATCGTCGGTCCAGATGGGAAAGTAGTTCACGAGGGTGAACAAGAGAGCAGTGGCAAATACACTTTTCAGGCTCATACACCCGGCG

>contig00664

CTACTATGAAGTGAaCACAGAAGTtGTTCGTGAGACGATGAGGGTTCAAACTCCTGCCATCACTGATCTTTCAACCGTCGGAACTTATATTGCTCGAGAGTGCCAAAACTTGCCAACATACATGCTCGAGAAAGTCGATACCAATGGAATTACAAAGCGCAGCATATCACCGGAGGGTCGCGTCTTCCGTCAATTCGCAGGTGAAACGATCCTCGAGTTGAATATCGTTTACTGAGGACAAAGCAAGACCTCGAGAATACAAAGTCATCACTTTAAGCATTAGTAGAATAGTAAGAAACTAGGGTTTGCTGTTTCACGATGTTCGGAAACAACCATGTCACGATCCTGCGATAATCATGATCATGATATGGGTGTAATATCTAGAATAATCTCAATCTCAATCTCGtCgCTGCTATCAATTTTtCAAAG

>contig00665

ACAAAGAtGCATGGCCCTTGCGACACTTTtgaCCGaTCAAAATTTCCCAGCCATTGGAATTCACAGAGGGATGACACAAGAGGAAAGATTATCgCGGTATCAGCAATTCAAAGACTTCCAAAaGAGAATTTtGGTGGCGACGAATTTGTTTGGACGTGGAATGGACATTGAAAGAGTAAACATAGTATTCAACTACGATATGCCAGAAGATTCTGACACATATTTGCACAGAGTAGCGAGAGCTGGACGTTTCGGCACGAAGGGTTTGGCGAtcACGCTCGTAAGTGACGAAAGCGATGCCAAAATTTTGAACGATGTTCAAGAAAGGTTTGATGTGAATATCACCGAATTACCAGATGAAATTGATCTTGCCTCCTACATTGAAGGAAGATAAATATTTAGCAGTCGGACAATTTTTCGAAATATTCCACTGGATGAGCTGAATTTGCATGCCCCCTAAAATGAACTTGAAAGCATTTTATAATCTACAATCGACTCTAATTACAACA

>contig00666

GAAGCAAAGTCAAGGACAATTCAAACTATATgAaCAAATTTCCCAAAGATGTGGAAACAGCAGAGACAAGAACAGGAGGAGATCCTAATATTTTATCAAGTGAAAAGTTCTGTCCAGAAGAAAaaCCCGATATATCTTCTCTCTTCGGAGAGAaGAAGCTTCCGAaGAAAAAaCGCGAaGATTCTCGAagAAGCGATGGCGCGGcAATCTCTAAAAAACTCACTCATATCAGATTTATGAGGCAGAGTGATCATCAAGTTCGAATTCGcGAGCAAGCGGCTATCTCGCCAAACTCCGACAGTTCctCTATATTCCCCTGTATCAAATGTAAcTTTACTTGCACGCGAGTCAACGTTATAATCGGTCACATAAAGAGTCATCAATATGATGATCCAACTGAAAaGAaGTCTTCGGTTAAGGTTCACTCAAAACCGAaGAACAAGAGTTtGGATCTTGACACTCCAAaGaGAAAaTATGTCCGAAAaTCCAGTTCCAATAAAaGTAAGGATGAACTCACTTACG

>contig00667

aaCCCTCGAGTCACACCCTTCTTGTCCACGGCCGGCACTGTCGCCGTGTTCAATATCAAGGCCTTAACACCAAGATCGGTAACCACTCTCGGACAATCACAACAGTTGCAAAGAATCTTCAGTAATAGTCCAGCGGCACTTTTtCCCATCACTTGTGCATAAaTTACATCACCCACGACCAAACTCTCTAAAAaaTTtCGAATGCGCTGAGATTTttCCATTGTGACGAATGTATCAAGCCCTGGCACAGTCGCATAAAaCTCTTCAtCACCAAGTTGTTGCTTGATTGGCTCAACAGTGGTTTCCAAATTCGACTTGTCATAAAGAGCGTCGGCCTTTTTAGCAATAAACTGCTGTAGTTTAAGCCTCTTTCCTCTGTCGCTAAAGCTGAGCGTTTTTtGTCTCTGCTGATAAATTTCAAAaCTGGGTTCTTTGAGGTTTAGCATAGCAAGCTCATTTtCGTTGCGCTCGCTCTCCCAAACTTTCTGCAACTGTTGACCATGGTAATTCAAGGCTTGCGCTACGAGACGCGCTTCCATCGACTCCATATCGGCAGATCAACCTAACCTGTAATACTTGCTAACTAAAaTTACGTTCCGTTGTGCTAAGAACGTAGATATAAAATAGTCTCAACTAATCACCCTCGGCGATTAAAACAATTTGCTTTTCTCACTACATCCCGTGAACTCTTTCTTTTTTCCGGGTCACGATTTTCGAGAGCTGCTGCTTCTAGTTCCCTAGCTAGAAC

>contig00668

AAGTTCATgCAGaCCAAAGaTCAGT

>contig00669

GTCGCTTTAGGATTTTGGAGTTTGTAACTGTAACATAACCTCTTCGAATTTGTGTTTTTAGCGTTTTCTAAAGAAATATTAAGAAATGGTAGCTCCAAAAAAGCCTCAAAAAAaGAAAACAGACTGTTGAAACGTCTAAGAAAaTCATCAAAGAAGTTCAAAATGATGAGGAGAGCGATTCGGAAGAAACTTTGAATTCGGGAAGTGAAGATTTATTAGTATCAGAAGTAAACGACGAAGATGACAGTAGTGACGAGGAAGCAGAGGATCTCAAAGAAATTCCCGACTCAGACACAGAAAAAGaTCCCTTAATTTTTGACTCTGATGGTGAAATCAGCGAGTTGGAATTAGTAACAGATTCCGAGGAAGATGACGACGACGCTGACGAAAGTGAGGAAGAAAaCG

>contig00670

CAACGATTTGCCTTTGATTGAAGAGATCTTTACTACCTGCAAAGATTTATCGATACAGAAACAGTTGGCCTTCATGCTCGGTCGCCAGCAGATCTTCCTTGACCTGCCAGAATCCACAGTCGAGT

>contig00671

ATAAagaatCTGAtttATCAAGcTtCATtATGGAtAAAaTACTTAAGGATGATCGTTTTACTCACATTTTCTATGATCCAAAATTCAGAAGAATTCCAAAGAATGAACGAAAaGTGAAAaTTGACAAACGATTTCAATCGATGTTTAAAGATAAAAAaTTTACTGTAAATTTtACCGTAGATAAAaGGGGAAAaCCTAGTGTCCAATCTTCCGGAGAAAATCTTCGGAAGTATTACAATATATCTGATAGTGAAGATGACGAATCGCTTAATACTGATTCTGGAGTTGTTAGAAAAaTAGTAAaGAAaGGAATAAAaGAAAAaGGATgTGAGATAAATAaTGCATTTAATAAAGGCAAAACTTTaCTtGTTAAAATCaGTGATGAAGAGA

>contig00672

ACTTGTCAGTATTGGTGGCTGAGTGGTGACTAAGTGTTTCTTTATTTGTGTTGGTTTTTTCACTGTCCTCACAGCTCCTTCTCTGATCAATCTTTCCACGTCTGATTTCATAAGAAATTGAAAGTTTGTCGGAGGTTCTCCAGATTCATCGTAAACAGCAaCTGCAAAGTAATCATCTGAATCTTCTACTGCATAAaGGGCTAGTTGTTGACCAGATGCTGTGACAAACATAGCGATaGTTTCTTCTTCCTCTTCCTCCTCGCATTCGCTtCCcTTTtCAATGTAAAaTTCTGTtCTTTCATCGTCGCTATCACACGATTCCATGGATTTtGATGGGACTTCGcTTTCCGCAGACCATTCTTGCTTAACTCCAACAAACACTGATTCCGAGTTTCTGCTGAACTCTTCGTCTTCATTGTCGCTTTtGTCCTCGTAAGAAGAGTCTGGAAGATTGAGATGTTCAGAAATCTCTTTTTTTATCAGGCTAATATTATCATCAGCTTCAGCTTCAGCTTCCTCATCCATGACCGTTTCGTCAAGTTCATTTTCGTCACTTTCTtCCATCACAATATTTTtATAAATAACATTTTCACTCTTGAATGACATCTTTATTCTCCTCGTTCAGTTTAATTCACTATTTTTtAACCCCGATTGATTAAtAa

>contig00673

ACAAAtGGCTCGATATGGCAGGCCAACTTCGCCACACAGAAGTCGCGGTAGTCGTCGACGTGGTGGAAGgtcacgtagTCGCAGCAGAGATCGTCGACGCTCCCGATCTCGATCCCGTTCCaGGTCACGTAGTCGCGACAGGGATCGCAGCAGGCGTTCTTAcAGTCGTAGTCACAGCCGCTCTCGAtCGGACAGCAAAAGCTCACGAGGAAAGTCTCATTCCCGGAGCAAGTCAGCCGACAGAACGAAGGACAGTCGTTCCAAATCTAGGGACTGAACGGCTACAAGCGTAAaTTGgAtGTAATCTGTAAAAACAGGCATAATCGAGATGCTCCTGTGTGAAAGCAGAATATTCTGAAAAaaGGTATTCTATTATTCTATaCCTTCACAATACTACTTGGCACATTCAATTCCcTTTtATTATtCGCGTATTAACcATGgTGACTCATTTTTGAATAGCAAAAaaCTAGGaTCAATCATTTTATtGTAATTCTCTCAAT

>contig00674

ACCGATTTtAAAATTACTTATGTTTGGAGGTCGTGCTCGCAGTTTGACAATTTGTCCAATCTTCCGTAAACATTGGCAGTTCGACTAGGCGGTTATCTCTAATAAATTCCCAAGCCTTCCCAAGAACGTCTGGTCCTGGCAGGCGCTCTCTTGCGAAAATAAGAACATTATTTTTtCCGTTAGATGAATGAGCACCGCAAATGAATTTAATGGCCAACTTCTTATATtgTATAAATACAATCCAATCTTCCCTTTGAGTTTTTCCaCCATCAAATGTTATAATATATTTTCCATCAGTTCCAAGTAATGTCACAACGCCATTCACTGTCTGTGCTTTTGTGTCTGTCACGCCTATACTGTTAAATGTAACATCTGCTGAaCTAGCATCCTTCTTCGTCCAGTTTAGAGTAAGGCATGTTATTGCGTCATTAACAGGCAAATCATATTTCGCTACCATATACCAAAGTCCGCCAAATTCTGTGCTATTAAAATTTGGCACTGGTTTCACATTTGGACAAGCTCGAGACAACGTAGTATAATCTGCGAAAGCTCCAGCGCAGAGTAGAACCAGGAATCCCACTTCGATCATTTTTtCACCAACAGAATTGAAGAAACACGCCAAAGCGCAGGTCGGTCGAGAATACTGCTGAAGAAGGATTTCAAAATTTGTATACACAAATTCTATTTTCTTTTAGATTTGAAAAAATATTCTTCTTTCACTTGATACTTT

>contig00675

ACtCTCGGATGTTGTCGATCATTTGGAAAATTCCAAAACAACCTTCGATCAGTATCTCAATGAGCAAGATATGCAGAAAATATCTaGCTTGGAGGATCTtGAAAatGTCCAGGAAGACAGTTTTtCAGACCTTCCAGAaGCTGACAGAAAATTAGCtGAaTTTGGTGATCCCTGGTCGGACTtAACATCAGAGAaTTTAGTTC

>contig00676

AAAGCCaGTcGATCAATGATAGTCTCtCAACcAAACAGGAAATGAaCAGTTCATCCCAGAGTCTTGATGCTAaGgATAGTGACATCGGTTCAGAGaTTTtGGATTCTAGTAGACATGAGTTGGATtCTAGCAGAAATGATTTTGATTCAAGTAGAAATTTGGATTCCAGTAGGAATAATTTAGaTtCCAGTAGAGATCTTGATTTGAAGGAaCCTATATCATGTAAAGAGGCATTAGGAGTGGATGAGAAAGACTGTTCTCCAAACAGAGACACTTGTGAAGGATTTTtAGgAaGAAAAACTGAGGTAATTCGAGTTGAAAAAATtATGGATAATGATGAGTGCCTTtCTGATTCTACGAAGACTGATCTCGAGAGTTCGATTAATACTTCTGGGGAtGTAGAGAATGAGAAACCGAGGTTTTGTCATtCCAAAAAGGtGGTGAATGGTATGTCCCAATTtGGGCCGATTAGCGTTAAT

>contig00677

ACTACATAGTCCAAGTAGTGTCAAATTCaGTGGTAACGAAGAAGTCGACCAAAGTCTACtAAAAGAAATATTTTCAGCAGCTCTTGGATTCACTGTAAAACATAGGGGAAATTGGAGTGGCATCTCCATTAGCGATCCTTTCACGTTACCAGAGGCAATAGTTGGTATAGCAGTCGAAGGTGTAGAtACATtAAaTTCACCAAGAGGTAAAaTATTTCCTCTTAACGTAGATGAAATTGAAGAAACAACCTGGCAAGCTCTgAGTGGtcGTCTTGAAGGGAGAgATGATGATAATACGCTCATAAGAATTTATCTCGGTGATGGACTGGATGCTCTGGGACAATCTGCTCTTGGTGAACTGAAaCCGACGTCTATAGACCAGTCTTCTCTGCGCGCTTTAAaTCTAAAAAaTGAGGAAGaTAAAAAGTTTCTTGAAGAAGTTCAACTTTtACGCGCTATTGCCAAAAAAATACCaTCTACTaTTACAGCAGATTCtAAGCCCGATGTCTATTGGCTTGTAGTATCTGGTTTACAACCTGTAATTGAATTCCATGGTAAAGAATCAGCCTCTGTGATGGAAGCTCTTTCCCTTTTGAACGATGCTTTTAATGATCTTAGCAAATCATTTGTTAATGTTTACAACGATAAGGTTTTAATCACGTCATTTACCAACGATGTTAGTCAAGT

>contig00678

ACTTAATTATAGGCGaCtATTGATTGGAAAtAAAGTaTTTCATCACAGGTCTCGAGtAtCTtCACTtAGCCtGtGCTTTtCAaTtCTTTTtCCATtCACGACTtCAAtAAATtCaTATCCTATaGACAAAGAGGAATGAGACGCTAAaTGCTTtGTTTTTCTGTGATCATTGTTTGCTTGGTTTtCTTAGATGATTAAATCAACGTCGTTtCCTTCACCTTGGCCTCGCcGGGgTGAATTTAAAGCTGGCCACAGTCGGCGACGACAACTTTCCTGGTTGTCTTTCCGTTGGGAGTTCCAAAGCTCTCCAGTTTTTtCACCACGTCCATGCCGTCCACGACGGCGCCGAAAaCGACGTGCTTGTTGTCGAGCCACGAGGTCTTGACAGTGGTGATGAAGAACTGCGAGCCGTTTGTGTTTGGTCCAGCGTTaGCCATGGACATGATTCCAGGTCCGGTGTGCTTCAGTTCGAAGTTCTCGTCTTCGAACTTCTCGCCGTATATGGATCTTCCTCCAGTGCCATTGTGCTTGGTGAAGTCACCACCCTGACACATGAAGTTGGGAATGACACGGTGGAAAGAACTGCCCTTGTAGCCGAAACCTTTCTCTCCTGTGCAAAGAGCACGGAAGTTTTCGGCCGTCTTGGGGACCACGTCGTTTCTCAGCTCGATGACGATGCGACCAACTGACTCACCGTCGGCGGTCATATCAAAGAAACATCTTGGCAAACCCATTGTGAATAAGTTTCAGGTGCACTTATCAAATAAGTTAAATTCAAAGCGGTCCAGCCGATAGTTGCAAAACGTGTGGAACGGAATGGACTTCCCTCTGCAGTGTGT

>contig00679

CATCCTGGTGAACTTCTCCATCTTTGTCGACCCAGTGGACGAGGAGACGGCCATCAGCCTGTTTCTCAATCGCTTTTGGTTTTGCTTCATAAACAAAGTGCACACCTCGTTCTGCCATTTCTTCAGCGACAATGTTTGCCATTTGTTGATCAAATCCACGCAAAATAATGGACCGCACCATTATCGTTGTGTCGAAACCCATTCCATTTAGGAAACCGGCGCACTCTAaTCCGATGTAACCAGCACCGACGACCAGAGTTTtGCCAGGCGATTGCTCCAAACTGAAGAtATCATCACTCGTGATACCGTATTCG

>contig00680

ACGtCTttGTtAGTTCCACACTATACTTACTGTATCCTGGTTCTTGGCTTTAAGCTTCCTTTCAATGACATTTTCCAGATTTTCACTCCTCGCGCTGAGACCcTCAATCTTTTCAATTAGCTGTTGATATGGCGAAGCTGGTTGTCCGCCCATTACAACGTGtCCCAATTTACTGTCAATTTTCGCATCCAGTCTCGCATTACGGATCAAATTCACAATCCAGCATTCTGCGATCGCAGCCTTCATGTTCAATTTCtcAGCAAGCATTTCGATACTGATGCACTGATGAATTCTGCAGAAAGTTtCGAAAaTCATCAATCTCGCGTTTTCGACAAACTCGTTTGGCAAAGAAATTAGGAAAAAGTCGTTACACACGACAGTTTGACATTCTTGCAATTTCTGGCGAGCACCATCAAAGTCAAAGTTTACATACAAGTGCTCCAAGAATTCAGTAATTGGATCCCGGTAAGTGTAGGATTCCTGTTGGATCACCTTAACCAGATCCTTCAACGTAGACCTTCTTGAGCGATTGACAATGACAGCcGCAGCCAAATAACGCAAAATGTGAGGGCACATTGTCTGAATGGCGTTCAAATAATGAGGCCGGTAGAGGAACATCTCGATAATAAGGTCTCTGCCTTTAATGTCGTTAAAGAATACAAAAAGACTCCAGTGAATGAGCCATGTGCGCTGTTGAAGGACTTCCAGGGAATTTCCAATCACATTACTATCAATGTATTCTCGCAGTTTATTAACATCTTCCAGGGCAGTTTCCCAG

>contig00681

AAAAAGCCCCAATATGAGTTCTCGAGGTCGTGGATTTAGTTCCCGCGGAGGAAGTTCATATCGGGGTCGTGGAGGAGGAAGTGATTGGAAGAATAGTTCCAATTCCGGCGGCCGAGGTAGTTACTCTTCTTCTCGAGGAGGTAGATTCAACAATTATACCTCTAATCAGAACTATGATTCACGCAACAAATACAATTCGGGAGGTGGATCCGACAGATACTCAAGTCGAGGAGGGGGAAGAGGTGAACACTCCAATAGTTATAAACGGCCACGCGATTCCTACTCTGGCCGAGATGATCACAGATCGTCCAGCGAAACTAACCGCAAAaGAATGAGAAACGATTCTTACCAGGGTGGAGGTAGCGGCAGTTCCCAGAGGTATTCGTCTTATGGGGgCTCTTCTGGGTCGGCACAGTATGACGATAAGAGATCGTCAGGATCAGCATACGAGGACAAAAGGCAGAATGATAGAACCTCTtCcTATCATCGCTCTGAGgACCGGCATTCTGCTTCAAGGAGCTATGC

>contig00682

ACCCccTCGGATAAGCGAAATGGCGCCTCCGGCTCCAAGATATACTTCCAGTCGAGGAAGAATATCCCACCAGAACTCCAATTATCGGGGTCGCTCTTCGACTCGTGGCAGAGGGGgTGGATTTCACCGTGGCTCTCGATCGGATGCCATCTTGGCTCGCAAGCGT

>contig00683

GGTTTtCCCACGAACCATCTTGAAGtAACTGTATCTCATTAACATCTGACAAAAGCTTGTTAGAATTAAGAACTTCCTGGAAGTAACCATCGATAACAAGATTATCGTATAAGGCTGCCTTATCGCAAACAGGACAACTCCAAGTTGGTTTTCGTTCATTCATTAATAGGAATAATGAGGCATCAAAGCACTGTAAATGTGAACACGTTGAAGCTCGACAAGGCGTGCTCATTCTCATTTTTCCTAGAGGGCAAGCCAATGATACTCTCAGAGACGTGGTCGCTATTTCACTGTCTGCATCTTCGTTCAACTTTTCTTTAATtAGTCCACGGGTGTAATCAGAGTGTTTAGCACCTCGATTTTTTAGCCTCGTCAACAATTCTGAGCTGgACAaTTTCTtCACAAGGTAAACGGCAATTGCATATCTCCTTCCATAGTCTGCAGACCATGTGACGTGAATTtGATTGCCTACAGTAGGCGACAaTTTTACTAAAGGACTAATATTAaCTGGCCTaGGGGGCCTTTTtGGTTCAACTCCTGGTTTATTGGTAGGTATCGGATTCGGTAATGGGCAAATTTtCCGTTtACTTTtACGgCTATATTAGgCGGGAAATAAtCTtCTtgTTCACACGATGTCtCTTGCAAGCAAAAaCGCATTTGAACCTGCACTGTGTAATCCATTTTACTACCctGGCGACAATCACGTGAACTAGCTATATCAGTTGATTGTTGCGGAGTTAAATGAAACATAAATGAATTTtCCTGCATCCTCATCGCCCCTTGTGGCGTCAAACTAGAAGGCTTCAATAATTCTCCGAGAAGATCAAAGAACGGCAATTtCttAAACTTTACATCTGGA

>contig00684

ACCTAATGGATTtGATGGAACCAGATTCGATGGAATTTCGCAGCAAGTCCTTCCTCCACCACCACCAGGTGTAGCTGCAAATGCAGCACAATTaGCAGCAATGGCAGGACACAGTGTCGcTCTCTCGCAAAaGAAAGGTTCGTTTCTTGGAGGAGGAACAGAGGgTGGCTACACCTTTtGGTAAACCAAGCGCTCAACGCGAACCGTTAATGTTATCACAGGCATTGTtGTTGAGATCCGTGTAGGCAAAATTCAACTGTCTTAGAGATTATTCTATTCCGTCAGACGCAATCTCCcTatAGCGTGCGTCTGAGTGTATGGACcGTCcGAAAGaTATCTGGAACGTAACTACTGCACGCCTATTTtCATTTGTTACCAAATATCCAGAAGAGAACGAAGaCGCgCGGGGCAACACAATTCTGCCGGTAGAGATTGAATAAATAACAAGAAAGAAAaCCCG

>contig00685

ACTaTcAAAGCTATGAGCCAACTCAGGATAGACAGTGGGGAAATGaTCCGCCATGGCAACCAGCAAACTTGGCTATACCGTTAACaGGCGGCGATGACAAAGATGGCGGTCCCAAATCGATTCCAACTATGGTCCCACCAGTTTCGATGCCTCCTATGGTCTCCTTACGAGATCAAaGAGAGGgAAGGGATaGAGATAGAGACCGTGATCGGGACTtGgAATCCGCAGGACGAGACcGAGATCGAGAgaGaGATCGTGACAGGGACAGAGAAAAAGAATCTATGGTCAAGGATCGAGACCGAGAAAaaGAATCTGTTGCAAGGGATGAAGATCGGGATAGAGATAGAGCaCGTTCaCAATATCGACATAAAGATAGGCATCGACATCGATCGCGATCGCGATCACGTAGACACAAGTCCAGATCACGAAGTCCTAGTCGCCGGAAGAAGAAATCCAGACGCTCAGAGCGGGAACGATCAAAaGAaGAGAGCGAATGATCTTAAATATTCTATTTAAACATGATATTTTAAATATCGATGAATTCAACAACTTGCAGTCTCAAAAACCACATAAGAAACTTCATCAAGCTAAAGGATTCAGTGAAAAATATGTTCTAAGTTCTCCGATCTACAAACATATTTTAGGT

>contig00686

tCAAAaTCGCtGAATCCTtCGAGTTGCTGGCTtCCTCGCTTGTAGGACTGaCATCAGCCAAAATATACA

>contig00687

TCTTGTGAGTAAAGAATTCTCTGGATAATaTAAATCTTGATTGGTGTGGGTGTATAATCGGGAGCCTTCGATATTCCCGTCAATAATTCTTTGTCGGTATtCTTTTTGAAGATCGGTGATGCAATTTTTCTCTTCAGTGTAATATTTTGCCTCGAATGGACTAAAGACTTTTTCTTGCCATGAGAAAGTGCGCGTCTCTTCGTCTAAAAAATCTGGGTCCAGGTATTCCTTTGGGTCCTCGAAG

>contig00688

CATGGGTTGTTCTACTCGATAAGTCCCTGCAA

>contig00689

CCAAAATTGGTTTGATTTTATAAAAAAAATGTTGAGGTTATAATGTAGCCAAACGCCGCTTGACTCACAT

>contig00690

ATTGTGTTGTATTTATTTTTCTAAATTTGTTACATTCTTTTTCAGCTAATGACAGTGATTCTGACAATGACGTGAATTTTTCCAAATTATTACAACTTGCGAATGACAGTGGCtAaGCAGACATTTTttAAAATATTTTCAGAGATGCAATGTCAAGAAAATACCACTTATCTTGTGAGTTCTAATTTTTtGGATCACTCTAGTGTCAAGAAAAGATTTAAAATTGCAGAAGATTTTGAAATATTTGATATTTATATTTTGTAAAGAGCACAAATTAAGAATTTTATCGACAATCTCCTTAAAATTGCAACAAAAAGAGT

>contig00691

CAGACATGgTGCCAATGAAGTATTTGCTTCCAAGGACAGTGCCATAaCTGATGAAGACATCGACACGATTCTCCaGAAGGGAGAGAACAaGACCGAAGAGCTGAAGCAGAAGCTTGAAAGCTTGGGAGAATCATCTCTCCGTAATTTTACGG

>contig00692

TTCCGTTTATCAATTCGAGGGTCAGGACTACCGAGAGAAGCAG

>contig00693

CCTACTTCAGGGAAGCGCTCCGAG

>contig00694

TTTACTATTTtAGACAGACGGTTGGATACAAGGTtCCAAAGAATCCAGAATTAGGTtCTGACGCAGCGAGAATTCAGAAAGAGGAGCAGCGTAAGATAGACGACGCTCAACAACTTTCTGATGAGGAAATCGCAGAGAAGGAAAGCCTGCTTACGCAGGGTTtCACCAACTGGACCAAGAGAGATTtCAATCAGTTCATAAAAGCAAATGAAAagTATGGTCGCGATGATAtCGAgAaCATTGCAAAGGAAGTCGAAGGCAAAaCGCCTGAaGaGGTGaTGGAg

>contig00695

AaCAAGTGACAACGCGTAGCAAACAATTTAGTTCCGAATATTTTCTTTTTCCAATATAAGAAGTAAAAGACGGTATAAAATTCATTTATTCCCACATTTTCCATCATATTCTCTTTGTATGAAGAGAGTTTtCTATTCCATATGTGCTGATCTACACAATCTATCAAATTATGGACGGAATGCCTGGAAGTGAGGTTGGCCCACCTCCACCTATGATGTTGGACTCACAACAGCTGCGACAAATGGAACAGCAGGAAATTCAAAaGCAACTAGcAAGCGTGCCATCCCCTATGAATATGCCACCTCCTCAAAT

>contig00696

ATGGgAATTCCACCACCCCc

>contig00697

ACTtAACATGCCTAATGTGAAACTACGTCAAGTTGAATTACTCGGCATTATAAGAAGAATTACCTCTAGAAGCAGACTTGATCTGATGATCGACGACGGCACTGGAAaGATTCAGGTATTATTACAATTTTCGCAAATGGgTGAAaTGAAGGAGGAaCGGAAACTCATCGACgCGAAATTTCAGAGATTCGAAAAAaTCGCTGTAATACAAAAACGCGGCCCAGAACACATGCAAGATTTGGAGAGAAGATGGAAGCAGGAAACTAAAAATGGAACCCTTGGCACGAGTTTTAAAGTTTtCGAACATGtCcACGTTATTGGCACAATTCACGTGGACTTTGGAAATCACAATTTAAACAAAaGGAGTCTTTGTCTCGATACTGCTAAATTtGTcAAaCCTGTAATCCACGCTAAAAGGATTTATCATCTAAGCGAAGAGGAATACAACGAAAAACTACAAAGTTGGaTTTCAGGAGTCGT

>contig00698

GAAAatCtCtGACTGGACCAAACAAAGACGCGAAAAATaGAAAATGGTATTTGtATCTCTCTTTAAGGCTGTTGTTAGAACATGGAGGATAGAGATTAAAACaTTAAAGTGTGTCTTATTTGTATTTCCAGGCATTTTtGGgCAGTCCGGAAgTGGGGggAgATGGTTTGGAAATAAAGACCGAATTTCCTAaTTGAAAATAAAAAAAAaGAAAaTGAGAATATTTAAATTAAATGACCCTTCTGACACTCCTGGCCCGGTATTATTATATtATAC

>contig00699

GTATATACACTTATTAACTACTTACTATAAATAAACATGCATTTGATACAAATTAAATATCTATCACAAGTCATACATATCCCGGAATAAaGCCTACAAGCCTTACAATTGCACCCTGAATATTTGGTTGGCAGTTACAAGATTCTGAACTGCATCTTATAATTAAAaTGTTTGCAAATTTTCCCTtGGCCaTCTTAATCTACTCACGTCGGCTTCCAAGCGACGATTTGCATTTTGTTATTCAGTTCTtgCAAAaTAAGGGAGCAGGTGAGCTGTTTTtCTAGCGATAATCCACTCACACCCTCcATTTCATCATCA

>contig00700

ACGTCtAAGACAGTATCGCAAAaTGCAATGGCTCAGTCCGTATTCACGCACATTACTTAGAAACTTTAGTTTATGCCTGGGTAGtATGTAAGTAGGTGTTACTTTATTAAAaaGTTGTTTCTCTCCGCTGCTGTTGTTGATATTGTTGGCAATATTCTGTGGTGACTCGTTCTCTTGTTTCTTTTTTTACCAATGGAAAGGTATCGATTGGCTAAGCGGGTAGTTGGTTGTGAACGGTTGAAACTGAACACAATTATTTGTCGAGGGCAGTGTTGGTTTTGATGGCGATCACAACTtCTTCGCCGCATGCTTTCAGCACGGTGCACAaTAAtCTTTtCCTGCATCATAATCGGCACGTAACTGAGTTCCCAATTCTCCGTCGGGTATCTTCAGATCCTCACGGAGGTCTCCGTTATCGGCCATCAGGCACAAGTAGCCGTCGTCGGATATATCTGTTAGCTGATAATCTTCCCGTTTAACAAATGGCACGTCCATGTTGTGTGTGGAGGGGCAAATATCTTCGTATTTCTTGGAGCTAAAAATATCGATGCCCACTAGGTGGACCTTGGCGTGACCGTGCTTTCCGGTCTTAGAAGTGGACATCTCCACAATTTTGCAAGGTCGTGATTTCAACATGACGAAACCATTTTTACGTAATGCAGAACATTGCATAGGATACGTTACGGAAGCTCCAGAGTCTCCAGTCTCGAAGTGGGTGTCCTCGATATCTGCCATCTTTGATCTTAGAAGTTATGTGATCAAAatGGATACTCTaaCCAC

>contig00701

GGTATTCTTTTCAATCTATTACTCACAATTACACTTGTTTACCATTAACATATGGAAAaCACATCCTTAGTAAGTATTTGTTACCTTCTGCTAAATTACAATGATAGTTGTAGTTGACAAAAAaTAATAAACACTCATGGTCTTGAAAaGTTAAGTGAAAAATAAAaGATATAAATTTATTTTTGGTTTtCCCGTTGCAGATCAACATTTTAAATTGGTTAAAGTTGTCAAAGGTTTCAGGCAGAAGGTGTTATTCATTCTATCACTTGTTGCTTTCCGGATTCGGTCCACTGGGTaCATCATGATTATCTTCTTCAGGCTCCGAGTCCTGTTCCTGGGTTTTCTCATCTTCTGAAGGCTTATTAACTTCTTCAGGATCCGGATTCTGTTTCTGGGGTTCCTCAAGCTCTGAAGGCTTATTAACTTCTTCAGGATACGGATTCTGTTTCTGGGGTTCCTCAAGCTCTGACGGCAATTCAGCGGCATGTGAAAAAGCCACCTGAGAAAATGCAATAATGCACACTAGGAGGAAGACGGTAACACTTCTTACGGATGCCATGTTGAACGTTGAAt

>contig00702

ATTTATGACATCTTCT

>contig00703

CGCTGACTGGCGGTTTtATTGATAGATCTTCTGCAGTATCTAATTCTTGAATGTTGCCAGGTCTATTTCGAAGAAGTAAACGCCTCCTGCAAAGGTTGATTGGGGAATCTTCAGTTAAATCGATGAAGTCAACTGGTATTGGCAAAGTTGGATTCCCTTTATTTGCCATTATGAAGTGGACAATTTTTTTCAAACTTTAACAGACGATCGAAATTGCTTAAAGAATTGATTTTAAGTGCTGTTTATAAAAAATTAGACTTTCTGCAATTATTCCCGATTATGTAGAATTTTCCGTAAATGATTTATTAAACGCTGTGTGCAGGAAGACCGAATTAATATCTTTTAGTCACTTCGACAGGATCTAGTTTTTGACTTTCTTCCACAGTTTTTGACTCGACCATATCGAAAC

>contig00704

GTGCCTGTTTAAGaTTTAACGGTTTCACGGTTTCAGTTGAGTTTGTGTTTGAGGAAGCTGATTTATTGTGAGTTATCGTGAAAGAGAATAATTTTCTCGACGAAATTATGACAGCCACTCAACAAAAAaCGAAGTGTATCACACTGAAGGGGTCAGCGGAATTAGTGAAGCAATATTTAACATACGGAATCAACAGTATCCTTTACCAACGCGGAATTTATCCTCCTGAAACATTCGAACCATTAGAACGCTTTGGAATTTTCTTTTTGATGTCAGTAGATGATAAAATCAAGACTTTTCTGGAGACGGTTCTCGGACAAGTCCAAGAATGGCTCGAACAGAGAAAGGTTCACAAGGTTACGTTGGTTATTACAAATATTAATACCAAAGAAGTTCTTGAGAAATGGGATTTTAAAGTGGACTACGAAGGCGAAACTCCGAATGGAACAGGCAATAAGGAAGACCATGCTATTATGCCGGATGTTGGAAACAAAGATATCACAACAATACAAAAAGAGATTAGGGAAGTCATTCGGCAAaTCaCaGGT

>contig00705

GCAACGTGTTCCAAGCGAATGACGTCTCCACATTTtATTGGCTTtCCTCGTGCACACTCTTTtCCAGTTtCAGCTTTAATTAACCAATGCGAATTTCCATCTtCTTTGGCATCcGTTCCTGTCACTGACTGTTGTCCACTTCCAGTTCCATATTTtATATCATGAGAGTGCAACCTCACTTTAT

>contig00706

CGAGGATGACCGTTTTGCGCGAGAAGACTGGCCAAAaCT

>contig00707

AAGTAGATGGAAAGAAGATGCACCAGTCTCCCGCTATTTCtCGTTATTTtGCTAAAAaGTGCGGCCTTGTCGGAAAGAATGACTTGGAAGCCTATGAAATCGATGCGGCCGTTGACACCATTCATGACCTTCGTGCAAGAATCGGTGCCTACAGCTATGAAACTCAGGCAGAAGCTAAAGAGCAAAAACTGAAGGAGAT

>contig00708

ACTATAAAGGACAGATTGCCAGTAATTTTAACCAAAATAGTCGACACACTGAGTCGGGATAAACAAGAGATTATTAAAAAGTATGGAGGGGAAACCGCAGAGGAGATAAAGCACATAATAGGTCTTATCAGTCAGCTGAAGAACGAGCTGGTGACAAATAAGGTCTTGAAACCGCTGAAACTCATCCCCGGAAAaGCTGATGACGATGCCGTTGTTTGGAATACATTTTTGGATAGAAAAaCTGAGATGGAAGGAGAAACGCCAGTGTGGTTTAATACAATTtGGCTATATTGCGAATGCTACATGTATCGC

>contig00709

ACCTTACGCATCTCGACTATGTTGACaCGGAAAGAGTCATGACGGAAAAaTTGCAGAATCAAGTCAATGGAACTGAAtGGTCTTGGAAAAATCtCAACACGTTGTGTTGGGCGATAGGAAGTATTtCGGgAGCGATGCACGAAGAAGACGAGAAACGTTTTCTAGTTACTGTAATAAAGGACTTACTCGGTTTGTGCGAGCAGAAAAAGGGgAAaGACAACAAAGCTATCATCGCCAGTAATATTATGTATGTCGTAGGACAATATCCGAGATTTCTCCGAGCCCATTGGAAGTTTTTGAAAaCCGTtGTCAATAAATTATTCGAGTTTATGCaTGAGACACATGaGGGTGTGcAGGaCATGGCATGCGATACTTTtATtAAGATTGCACTGAAATGCAGACGACATTTCGTCACGTTACAGGTTGGCGAGGCCATGCCGTTTATAGAAGAAATTTTATCTACTATTAGCAGTATCATTTGTGATCTCCAGACGCAACAGGTGCATACATTTTATGAAGCGGTTGGTTACATGATTAGTGCCCAAGTCGATACAATAGTGCAGGaGCACTTAATAGagAAGT

>contig00710

TCATTATCGGTGACaCAGGAGTTGGGAAaTCTTGTCTTCTTCTACAATTTACAGACAAGCGATTTCagCCAGTCCATGATTTAACAATAGGTGT

>contig00711

CATATTACCGTGGAGCAGCTGGAGCACTCTTGGTTtACgATATTACCCGCAGAGAGACATTTAATCACCTCACAACGTGGCTGGAGGACGCGAGGCAACATTCCAATTCTAATATGGTCATAATGCTAATTGgTAATAAGAGCGATTTGGATTCGCGAAGGGAAGTTCGAAGGGAaGAAGGTGAAGCTTTCGCTCGAGAACACGGACTTGTTTTtATGGAAaCGAGTGCCAAGACTGCGGCAAATGTCGAAGAAGCTTTCATTAACACGGCCAAAGAAATTTATGAGAAGATACAAGAGGGCGTCTTCGATATCAACAACGAGGCAAATGGAATCAAAATTGGGCCTCAACATTCTCCAACCAGTCCTGGTGGATTATCAGGAACTGGaGGACAAGGTAATGCACAAGGCGGTGGTTGTTGCTAGGGGCTGGGTATTGCTTGTCCACCGATTCATCCTTCAATAATTTGAACTGATCGATCATCCCATTCCTGCTCTTCGTTTTAgCCCCGACCGCGTAAaTTAAATTTGT

>contig00712

GTCATTCGTCGGTGTTCGTCGGTGCAGGAAGTGCTAATTATCCTAGGTGCCTCCTCCCATCTTTCAAAAAAGGGGTTAACAAGTGACTATTCAATTAGGAAATATTTCTTTAGAAACTTTTTTGGTGAAAaTGACGTCCGAAGATTCTACTCCAACTTCCACGACAACAAAATTGTTTTCACGAGCGGAGGTTGCTAAGCACAACGACAGCAAGGAAACGTGGCTGATTATTCACAATAACGTCTATAACGTGACTGCATTTCTTAACGAGCATCCTGGCGGGgAGGAAGTTCTTTtGGAACAAGCAGGACGTGACTCAACCGAAGCTTTCGAAGATGTGGGCCATTCGAGCGATGCTAGACAAATGATGGAACCCTATAAAATAGGAGAAATAGTAGAAGAAGAGAGAACAAAGACGGACAGTAAAGTGACCAAAGATTGGGCGAGTGGAACTGTAGAAAATGATGACTCGAGTGGCTCTTGGAGATCCTGGTTGATTCCGATTGCACTTGGAGTTCTAGCCACTCTCGTCTACCGTTATTtCATCaGTGT

>contig00713

AttGGGATTCCTCAAATAAAaCCTATTAATTGATTATTTTTATTTATTTATTTTATATTTAGATTTATTTTTTTCAATAATAATTTATATTTTTTTAATTATAAAAATTTTTTAATAAATAAAATTAATAAAAATAATTTAAATTATATAAAATTTAAATTTAAATGATAAATAACTTATTTTCTATTTTTGATCCTTCAAGAAGGGAATCTTTTTCATTAAATTGATTAAATATATTGTATTTTTTAATTTTTTTACCAAGGTTATTTTGATTtAGTCCTTCAAAATATTTAATTTTATTTAATTATTTATCAAAATTTATTATTAAAGAAATAAAAATTTCATTAAATAAAAGTTTAAATTTTTATAATTTATTAATATTTAAATCTTTATTTATTTTTATTATATGAAATAATTTTATTACATTATTTCCTTATATTTTTAATTCTTCTAGACATATAGTATTTTCTTTATCTTTATCAATTAATTTATTTATTGCATATATATTATTTGGATGAATTAAAAATTCTTTTTATATATTTATTCATTTAACTCCTCAAGGGACCCCCTATATTTTAATACCTTTTATAGTAATAATTGAATCAATTAGAAATATAATTCGTCCTTTAACATTAGCAATTCGATTATCAGCAAATATTATTGCTGGCCATTTATTAATAACATTAATTAGACAATCAAGAAATAACTTAAGAATAATTTTAATTATTTtAATAATTTTAATTCAATCATTACTTGTAATTTTAGAAGTTGCAGTTTCTTTTATTCAAGCTTATGTATTTTCAATTTTAAGATCTTTATATTCATCT

>contig00714

AAGTTTTTTTtAACATTACTTTTTAATTAACTTGTATGACGCGAGTAACAACAGTTGAAAaGAATACATATGTTAAGTATGTAAATGTAAAaTATCACATAAAAAGAACATATTCTTAATCACATTCAACCAAAaCCGTAACTGTGTTTtAATTTATAATATAAGCATCTAACAACTGAGCTATTAGTGCAGATAACTCTCCTGACTTCGAACGGGATAACATTCAATTGTATGCCAGGTCTGTAATTCGGGATAACGTTTATtGCcAATCTATGT

>contig00715

ACATTCGCGATGATCAGCATCAGTATCGCGTCTGTCTTGTTTCGGTGACGGTGTTTTGACGCGCGATTACTGCGAAAATTGCTGTTTACCCGTCAAGATCCGCTTGTTTCCCTCAATCGGGATTTGTAAATTCGTAATTGTTTAGTTTTTCGTGTTAAGTAAATAATGGCGAATTTGAGACAAACCCcACTCACTTGCAGCGGCCACACGAGGCCTGTTGTGCATTTAGCATTTTCAGATGTCACGGAATCGGGATACTATGTCATTTCGGCTTGCAAAGATGGTAAGCCTATGCTGAGACAAGGTGATACAGGAGACTGGATTGGCACGTTCGAGGGTCACAAGGGGGCAGTTTGGGgAGTTGCTTTGAACCCTCAGGCCACCCGGGCAGCAACTGGTGCTGCAGACTTCAATGCGAAGGTTTGGGATGCAATAAAGGGCGAAGAAATTCACTCTTTtCAACACAATCACATTGTCAAGTCAGTCAA

>contig00716

ACAAACGCGCCGTGTGGTGTGGTGGATCCGGTGTATCCAATTTtCACGCTGGTTCcGCCAgAAAGGTCGCCGAATCCATCGCgAGcTCTCGCGAGTGACGTGCGTTACATAAAAAaTACCACATTTATCTGAAAAAaCTTCTACATATCCACCATGAGTGCTGAAGAAGAGGTTCTGCGAATCCAGAAAaaGCTGACTAAAaTGTCCAACGGCGACGGATCGGGCCAAGAaCAAGCaTTGGaCTTGCTCAAAaCTTTaCAAAAGTTGCCGGTAAATTTGGATCTTTTGACGAAAACTAGAATCGGCATGACGGTCAACACTTtCCGAAAATCGAGTCaGGACGaGGAaGTTATTtCCTTATCGAAGGTCCTCATCAAGAACTGGAAAAAGTTCTTGTCCACAGATAAAGGCAAAGACTCGAGTTCGTCgaGTAGTTCGAAAAAGAAGGAGGACAAATCTGAGAAAAATTCAGACGACAACGATTCAAAAaGAGAGAAaGACGTTCCAGATGGGGATGTGAAGATGAAGGAAGAGAAaTCGAGTAAAGATGAGAAGAAAAAaCAAACCTCATTCCCACCTCCATCCACAACTGACGCTGTCAGGCTCAAGTGCAGGGAACTACTAGCAAACGCTTTGCGCGTTGATGGGCAAACTTTTGAAGGTTGTGCGACGCCTGAAGAATTGGCGGAGGAATTGGAAGATGCAATTTTCCAGGAATTCAGAAACACGGATAACAAGT

>contig00717

ACTTTTtACATGAGAAAAGAAAATGTtAaTGCAAAGAAGAATAACAGTTGCCGAAAAAaGTGTTTATAAGAAGAATTACGTCAAGTATCGATTCCCGATAAAGAGATTATTGCTTTCGTATTGGTAAATAATAGCATAGGAACTTCGAGACTTACCGACCCTCAACGGATCTTAATAAAGGCGTATTTCGTATTTtGTGATTGTTTTCATGGATATTTAGCAAAAGGGAATGCAATAATTTTTCTCTTGTTGCGGCTGCTGAGTGATTTtAAACGAGTGTATAATAGTGTCTTCCACTTTACAAACTCAAAaCTTTAAAaGGTAGATGAATTAATTGCAAATTGAGATATATTTtCCATTCCTTTTTGCTAGATTTTTACTGAATAAATGTAGCATCTTTTtCGTATGTCGTAAACTTGTAGGGAAATGAAGCAGATGTATAAGTCATTCCGCATGACTTTAAaGAaGACTGTTTTTC

>contig00718

TAAAGATATGGCGATTTTCCATAGCGAGCTAAACTATCACTGTAAaGTTTTATCTTACGGACTGTATTTATTGCACTTTGGCCAATATACTCATCGTCCCTGTAAAGGGCAAGGGCATGCCCAGTAAAGTCCTGGGTGTTCTTGTCGAGACTGAACTTGTTATAGAGGGCGCTCATGCTGTTGCTGAATGGATCGAAGCCATCCCAACTCTTGGGGtCATCTTCTTGCATATTCTGAACCCAAGTGAGAAAATTTTTGAAGCGCCTCTTCTCGAACATGCCCATCAAGTCGCTGGACAATGCCTCCGATTGATCTATTGGCACTTTCGAAATCTTCCCGGACTTGTAGACATAAGAACCTTCGATCGACTTAAATTCCAGATATCGCGTGACTCCTGTGTGGATCAGAAGTTTCACGAGGATTCCATTTGCCATCAAAAACTTCGGAATTAGGTCTACATTCCAGTCACGGCCTCGACCATAACTCTCGTCTGGTGGTGGGGCCTTGAA

>contig00719

ACTGAAGAGGTCCTCCAGGGGCGTGATGGAGGCGGATTCACCTCCATAATACTTGTTGCGGTCGACGTGGAGCACCTTCTTTCCGCTGACAGAGAGCATTCCCGAGAGGATGCACTCCTTGAGGCCGGTGCCCAGCACTATAGCGTCGTATTCCTCGTCCATGGTGCACTGAACGCGGTTTCGCGATTTCCAGAACTCGACGAGGTTCTTTTCTGGAGATTCGTTGCTCTTTGCTCGAGGAAGAAGAGAAACCGGACGCGACACGC

>contig00720

AGATAGATTGAGCAaTCCAAGATTTATTTAAAATaTCCGTATTGTCGTGGGGGgCGCGATACCTCCCTTTTTaGTtGGAACATGGTAGTGCAAGAAGAGAGCTTTATTCTTTCTtGTAGCCTTGGCCTGTCTCTGGaTAATCGACAATTTAGTTtAGAATAaCTAGATTAAGAACAATGCAAaTTCAAGAGCTGATTCGTGACATGAAATGTAGAGGGTgCCACCGCCGAATCAGAAGAAACTAATCACGCATTCAACTAAATTATCAATGTCGCTTAGAAATACTTTGATCATTTGTTTTCCCCACGAGTTTTGGCTAAGTAAGGTGAAAAaTCTTGATTAATTGATTGATTGATGATTATAAAACGTTGAGCGAGCAGAGATTTTGTCGTTGATGATGCATCACTGTGGCGCCACGCAGAGTGTCTTAATAGCGTCGTCGTTATCCGGAGTCTCGTGGATGTTTCAGT

>contig00721

ACGATGAACTCTCCTGGACAGaTGTtAGAGAATTATTCAGAGTTTGGAGAGAAAATAATGAACGGAAAAGTAaGGATGTAGTCGACTTGTGGGAATCGACTCTAAAGTCGAAAGTGGATAATCTTGGGAATGAAAAATATTTGGTTTTGGAACAAGTATGCATCGCAGCTTTAGaCTGcTATCGTCTTTCTCTAGCCGAGCAATGTTTAAAaGTtCTTATGATCGAGTTTCCTGGAAGTCTAaGAGTCCACAAAtATCACGCAaTGCATTTGGAaGCCTTGGAAATGTATGACGAGGCAGTAGAAGTATTaGATTCGATAaTCAAAAgAGACGAAACTAACGCAGCTCCTAGAAAAAGACGAATTGCTATTTTAAAGGCAAAGGGACAAATCACAGAAGCTATCAAAGAACTTACGGAATATTTAAAAAaGTTCATGAGTGATCAAGAAGCTTGGCACGAATTGTGTGATTTGTATCTTCAAGAACAAGATTATTCCAAAGCAGCATTTTGTATGGAGGAACTCATACTCCACAATCCACATAGTCATTTAATTTACCAAAGATACGCGGAAATTAAATATTCACAGGGTGGATATGAAAATATGGAGTTAGCTAAGTCATATTTCTGCCAGGCAGTAAAACTTAATCCAAACAACATTAGAGCGCTTTATGGATTATTATTGGCAACGAATCACCTCGCATCTTCGTTGAAAaGT

>contig00722

ACTTTGGATTATCTTCCCCCAGAGATGGTATCATGCCAACGATACGACATCTTCGTAGACCATTGGTGCTtAGGAATTCTATGTTACGAGTTCCTCGTGGGAAGAACTCCCTTCCTTaGTGATTCTCAACAAGAAACTTACGAGAAAATTAGAGCTGTGAAAATTCCCTGGCCATCGCTAGTTACCCCTGGAGCTAAAGATCTGATTTCAAGGCTGATCAAGAaGGAAaGCAGTGAAAGAATTCCATTAGTGGATGTGAAAaGACACCCTTGGATTGTCGCAAATAAAGATAAAAaGTagTTAGTAATTATATTTTTACATCATACTTTTAGAATAATTCTAATAATTTAAGTAAGTAGTATTATGGAATTGCGAGTTGCAGAAGTGCCTGAGGAATTTGGTTAATTTTCTGCTATGATTTTAATTTAAGGAGCAACATAATTTTAACTCATTCTCGTTTAACCGACAGTGAAATTCGTGTATCATCAATTTATAACACTTTTTCATATTGAAATGAAACATTAGTTTTTAATAATGCATTTATTTATACATGTATAAACAAAATTCACGCTGATTGTATGACAAAGTATATTTTATTCTTTAAGAACTTGT

>contig00723

ACCTCtgTTTTttCATCCcAATAAAAaTCATAAAaTaCACAATTAAGAGGCTCTCCTCTCCTCCCCCATATAAAGAAAACTCCcTATTCTGCACAAGAATCAAAACCTTCTAAAAACCTaGCAGATATTATTGGGAATGGCGGTCGATCATTTCACCATATAGTGCCGAAAATCAGAACTAGTAAGACTAGGTGGAATTATAAAGATTAGTTtAATTGATGCATAGAAGTGTAAATTACTATTGTTTTATGGAGTAAGTTTCAATATATTATAAGCTtGAATGCACTTATTATTCTATATGTAGCAACAAATTTgATTCaGgTTATGCAAAGAATATGAATAATAAACTATTGGTATTGAAATACAAAtGTTAA

>contig00724

AATAAGTTtACTTTCTTCTTGCTTtCCcAAAAAaTAAGTTAaTTtAAGAttAtAACTTTTTTtATGCAATAACTGCTTTAACCTATACAAAaCAATCATAAAaCCGCTTTTtATGCACAAaTTAATTAAAATTCACCTTTAAAACTCGACCTACTCTTTCTAGTTCTGATTTTTttGCCCCAGATGACATATCGCAGTTTAGCCGTTCCCAATTCTACAAAATAATACAGTCTATTATGTCCAGAATCTACCaGATCTATCCACAAAATAAAGAAGTTTTCCAATTCATTTGAaTTCAATAACGTCTACAATTTtCAAAATTCATTCGGTATCACATTCACTTGGAAAATGTGTTACATCAATGATTCTCACTCTGAtGTGTATGACTGACTCTAACTTAATactAAACaCGT

>contig00725

ACCGGAGAGCGTAAACGTAAATCCGTGCGTGGTTGCATCGTGGATGCCAATCTTTCGGTTTTGGCTCTTGTTATCGTCAAAAAaGGAGaGCAGGAAATTCcTGGATTGACCGACAATAACATTCCACGTCGCCTTGGCCCTAAAAGGGCCAGCAAGATCCGAAAGCtGTTtAATCTCaCCAAACAGGATGATGTTCGTCGCTTTGTCGTGAAGCGTCCGATCCAGAAGGAGGGTAAGAAGGAGCGCTTCAAGGCACCCAAGATTCAACGATTGATCACACCACAGACATTACAGAGGAAGAGACACAGGATTGCACTGAagAAGCGTCGTTGTTTGGCTCGTAAGGAaCAAGCCGCTGAGTATGCAAAACTCTTAGCTCAGCGACAGAaGGAAGCGAAAGCAAGACGTCAaGAGGAATTGAAGCGAAGGCGTAGCGCATCGATTCGCGATTCTAAGTCTTCGAGCCAATCGGCaCCAGTTGCAAAG

>contig00726

ACCGTCTCtGCAtCAAGAGTTGGAAtAtGtGACAGACGTAATCCATCTTTCGGTAagAGCGAAATAATGTCACCATTGTGGAGGGGAACATTAACATTAGCGGAACGCTCTTGCACAACAGCTGACACTAATAGCGGACTGCCTTGCATACTGAGAACCATTTCTCCTtCtCGCGGGCGACGCAGCACACTCATAGgaGTATTCGGTTTCACTTTTGGTGTTACAGGGCCAAATTCATTACTTGTTTGCTTTAGTAAGGCTGGTGTCTTGAATTTACTTGCAGACTGGTTTATGTAATCTTTCGACGTTTTTGATATGATTTTGTGTGACGCTTGGCTTAATTTAGTCTTTCTGCTCCTCGATG

>contig00727

GTCTAGTTCCTGCCTCtGTtACGTATCCATCATCAGATGTAGCTGTCA

>contig00728

ATAGAAAAGATTGTTCAAGGTTGTGAGAAGACACATCATTTTCCTTGTCATCTTTAGATTCTAGAGCAAGAATTTCTCCTAAAGTGAGTTTTCTGATATCCGAAGGTAGACGTGACAATGCCATATTtACTTCGGTTTCCAAACCCCTGACTCTCATTTCTATTTCTGCTTCCATTTTGCGAATTTTCAAATTGCCTTGTCTCTCAAAATCTTTGATTAATGAATCTGTCTCGTCTAAGGAATCACGAGATTGTTTTATTTTGCGTGCTTGCTTTGTGCGTGGCATGATGTGTGGCTCTTTTAAATTTTGTAAATGTGATAATTCCAAGT

>contig00729

ACtAAAGGCAAATCATGTTTACGACAAATTACACGAGAGATGCAAAAATAAACTATATCGAATCTAGAGTGTCTTTTTTTCTCGCTTATTATCGTATTAA

>contig00730

ACCTTGAACGAATTTCTGTATCTGATCATACTGCTCTTTTGCTTGACCCTCCTTCACTAAATCAATTTtATTTTGCAAGATAACAATATGTTTtAGTTTCATAATCTCAATTGCAGCTAAATGCTCCGATGTTTGCGGCTGAGGGCAAGATTCATTTCCAGCTATTAATAGTAATGCAGCATCCATAACTGCTGCTCCATTAAGCATTGTTGCCATAAGAaTGTCATGACCTGGACAATCTACAAAAGATACATGACGAATTAAATTAAATCGACCAGAACATATTGGACGCAGGCACGGAAAAGAATCATCTTTGCTAGACCcTCCAGAGATGTAACACCCAGGTCTTGGAcaCTTGTCATTGTCACACTTGTATATCTTCGCATTAGCATATCCTAATTTTATAGTGATATTTCGCTCCAACTCATTTTTAAAaCGAACAGTCTGCACTCCAGAAaTAGCTTTAACAATGGTTG

>contig00731

GCGTAGTATtGTGTCACTCCCTTtAAAGTCAGCTCTTCCATCAGATTGATCTCGTATGGATCTTTCAAGTGCTTCTCCATAAACTgCTTCACTGTCAGAGGAAAAGTGGCTGAATACAGCAAAATCTGGCGTCTCTGAGGTAaTCTTGAAATCaCATGATCTAACATTCCTTtGAAGTCTTGAGACAGAAGTTTATCAGCTTCGTCTAACACGAGGATCCTGCAGTGTTCCATATTGGCAACGTTCTGGTCGATGAGATCAAGAATTCTACCTGGGGTGGCTATGATAACTTGCACTTTCTGATAAATCCTCAtGATATCGTCGCGCAAGTTAGTTCCACCAGTGGTAACCATCACTTTAACATCCATGTGCTTTGCAAGCTCGATCAATATTTGTGAAGTCTGTAATGCCAACTCTCTGGTGGGCACAATCACTAACGCTTGTATCACGTCTTtCCTTGGGTCAACCTGTTCCAAAACAGGAATAGAATACGCTCCAGTTTTGCCTGTTCCGTTTTTGGCACGCGCCAAAATGTCTTTGCCGGAGAGTGCAATGGGAATACTGGCTTCTTGAATTGGAGAAGGCTTCTCCCAGCCTTTTTCGAAGATCCCCATTAGCAATTCCCGTTTCAGACAAAACTCCTCGAACTCATTGCCACGTGTGTCGGTAACATCACTAGTTTtCATTCGTATATCCTTCGGTGGGATTTtcAGCTTGGCTTTCCAACCCACATCATCCATTTTtGCTAGATCTGTTTTTGGACCCAAACCAGAATTCaGCATGTGATTGGAATTTATATGTGTTTCGGTCAtCATCTTGGAAGCTGTGcT

>contig00732

ACAAGCtGCAAATAATTTAACCCAGGCTTTAGTCGATCATCTAAATGTTGGGGTTGCCCAAGCAT

>contig00733

CTCGACGCTGAAGCCAAACAGTTACAAAATAGTGCAACAAATTTCGCTAAACAAACTCAGTCATGGCtAAATCTGGTTGAGTCTTTTTCGAGTGCTTTGAaGGAAATTGGAGATGCTGAAAATTGGG

>contig00734

CATAAATGGAGTTTtA

>contig00735

ACCGATtGtAAAATtACTtATTTTCGGAGGTCTTTCTCGCAGTTTAACAATTTTtCCAAtCTtCtGtAGCCATTGACACATCTATTAGGCCGTTATCTCTAATAAATTCCCAAGCCTGCCCGAGAaCATCTCGTCCTGGCAGGCGCTCTCTTGCGAAAATAATAACaTTATCTTTTCCGtCAGTTGCTTTATCGCCGCAAATTAATTGAAtGGCCAACCTTTTATATTCTATAAATACAGTAAAATATACCAGTTGAGTTTTTCCACCATGGAATGATATTTGATATTTtCCATCAGTTCCAAGTAATTTCGCAACGGTACTCGTTTTTTTtGCTTGTTTGTCTTTCACGCCTATACTGTTTATTGTGATATTTGCTGAATTAGCATCCGTCTTCGTCCAGTTTAGAGTAAGGCATGTCACTTGGTTATTAATTGCAAAATTATATTTCGCTACCATATACCAAAGTCCGCCAAATGCTGCGCTATCAAAATTCGGCACTGGTTTCACTTTTGGACAAGCTTGAGATTGCGCAGTAGTAACTGCAAAAGCTCCAGTGAAGAGTAGAACGAGGAATCCCACTTGAATCATTTTTCCACCAACAGAATTGAAGAAAAACATCAAAGCGCAGGTCGGTCGAGAATACTGGGGAATGAAGATTTCAAAATTTGCATACACAAATTCAATTGTCTTTTATATTGGGGAATCCTTTTCTTTTACTTGATGCTTTAGA

>contig00736

ACAAGACATTCTCTGTTTTGTATCTTGATTTTGAATAATAAATTaCGCTTTTTtGCGGGGGTGGGGGgAAaCACTAATCCATCCAAAGATTAAAaGAATTTCAGGATCGAGAGGAGCTTAGCTTTCGCTAATTGGTATTTCACGCAACTGGGTCCATtcGCTGATACTCTGTCCTTTtCATTTCTATTTGTGAGTTGCTTCTCGATGAGTTTGAAAaCTAAAaCAAaTTACAAGATTTATGGTGGAAGATGACGCAGCAATTACGAAAaTAATATGAGTTTTTGTGAAGTTTACATTTtACTCAGTTTTATAGTTGAAAGTTTtATTAAGTATACTAGTGCAGTTCAGTTATGAGAAAGTTACAATCATTTTTAAAAAGAGCCACAAAGTTTTAAAGTAATTGAGTGATAAAaTAAGTTATGTGAAGTTAATGAAATCGAAGTTTACAACTGAAAGAAGATTAGTCCAATGCAGTTTTAAGGGCCTTTCAAAGGATTATTCATTTTATGGAAAAACGTCCAGTAACATGGCGGATTTAAAGAAaGAGGAACAAATTtCAGTAGGAGAGAAAATAATTCGAGGCTGTAAGTTTTTTtCTtCATTTTTGGAATTTTAATCCACTTTTTtAGACTGTCTGAGAATCAGAAGCAAGATTATTcTTGATCATGAGGCTACCAATTCAAGAATTAAATATTGACTAGATCACTAGAAGATCCAAAAAAAaTCTCTAAGGAAGT

>contig00737

ACACGGTtGGATTtATGGCATCACTTCGAGATGATCGAGATATCATCAGTGCTGAAGAGGCAACGAGAGCCACCGCGTATGTCGAGAAAGTTCGAGGGATTCGAGATGTTCTCAGACGAGATAACATGAAAGTTGCTTTCTTCGGAAGAACAAGCAATGgAAAaaGCACAGTAATAAACGCCATGTTGCGTGATAAAATATTACCCAGTGGCATtGGACACACgACAAACTGCTTTCTTCAaGTCGAaGGTTCAGAAAaCAATGAATCCTATTTAGTGACTGAAGGCTCTGATGAAAaaC

>contig00738

CCAGGAGTCGACGTAaCTCCGAATCTGGATGAAT

>contig00739

ACAAATCAGACCAACAATGATCGATCAAaGTGTTGTAGGAATCTGCATTTTAGTTTTTCTAGCCATAATATTTAATTTCTCGTtACATCGCATCGATGAAGgaCACGTCGGTGTTTACTTTCGAGGCGGTGCCTTATTGCCTCAAGTAAGTAATCCaGGATTCCACATGATGGTtCcTCTCTTAaCTTCGTTTCGACCCGTGCAAGTAACTTTGCAAACCGACGAAGTGAAAaaCGTTCCATGCGgAACTAGCGGAGGCGtGATGATTTATTTTGATCGTATAGAAGTCGTTAATATCTTGGATGCAAATGCCGTCTATAACATGGTCCGAAATTTCACGGCGGACTACGACCGGACCTTgaTCTTCAATAAaGTCCATCACGAATTGAATCAGTTTTGCTCCGTCCACACTTTGCACGAAGTTTATATCGATCTTTTCGATCAAATAGACGAAAATCTAAAGACTGCACTCCAAAGGGATTTGAACGAATTGGCACCTGGCTTGAATATTCAAGCAGTTCGAGTTaCGAAACCAAAAaTACCGGAAGTTATTAGAAAAAaCTACGAACTCATGGAAGGTGAAAAAACGAAGCTTCTAATTTCTGTTCAGCATCAGAAaGTAGTGGAAAAGGACGCTGAAACGGATCGGAAAAAAGCGCTtATAATGGCTGAGAAAGAATCACAAGTTGCAAAAaTTCA

>contig00740

ACTCAACGGATTTGGCGGCCTCAAGAGCGTTAAGGCCCTCAGGAAGCCCGAGCCGACCCTGGGCgAGGGagaGGTCCTCATACGAGTCAaGGCATgTGgTCTCAATTTCCAAGACCtGATGGCAaGACAGGGTGCCATtGACTCTCCGCCAAAAaCACCCTTCATCTTGGGGTCTGAGTGTGCTGGAGATATCGAGCAAGTtGGTGAAGGTGTCGAGAATTtCAAaGTTGGTGATAGGGTTGTCGCTTTACCAGACCACAAAGCCTGGGCTGAACTCGTAGCCGTTCCTGCAGcTtCCGTTTTtGCTTTGCCACCTGACAtGAGCTATCTGGATGCAGCGGCAATAaCTATGAACTACACtGTGGCTTACATTTtACTTTTtGAAATGGCAAACATCaCGCCCGGAAAAaGTTTaCTTCTTCATAGTGCTGGAGGTGGTGTGGGTCAAGCCGTtGTGCaGCTCGCCAAAACTGTtAaGGATGTAACCATCTTTGGAGTTTGCAGCAAATCAAAGCACGAACAACTTAAaGACTCAGGAATCGATCATCtCTGGAACGTGgCGTTGACTACGTGAATGAAGTCAGAAAGATTTCCGCTGAAGGCGTTGACATCGTCTTGGATTGCCTATGCGGACAGGAATGCAATAACGGTTACGCCCTTTTGAAACCGATGGGACGATATATTCTATACGGATCCAGCAATGTCGTCACTGGCGAGACCAAGAGCATTTTCTCGGCAGCGCGATCCTGGTGGCAAGTGGATAAGGTTTCCCCCATCAAGCTCTTCGATGAGAACAagACGATTtCcGGTTTCAATTTGCGTCACCTGA

>contig00741

TAGTGAATCGATGTTGAAAaTGCTTGGTTT

>contig00742

AAAGACAAAGAACCTAAACGTCCT

>contig00743

ACTAGTTTAAATACGGAAAGTAGGTTCGGAGAAGCATCTGGAAAGGTGTATTGTGGAGTAATGAAGATTAATGATCAACAGTGGGTCATTGGATATATCGGACCTGACCCcGGAAATCAAAaCGCAGATATGAGTCCATTCGCTCATCCCGATCAGGAAAaTCATCAGCATCAAAACaTTAAAATACGCGTAGATCCACTAGATTCCTACAAGTATTCTATTTtAACCGAATATACATCTTtCTACGAGCGGCCCCATACAACAATTCGTGAATGCTTGATACCCAATAGGAGTCATCGTCATAAGCTTGAATCACTCGACCcGCCGAcTACCCCAGGTGCTGGTTCGTCCGgAGGGGGgaCGTtCTTCGATCAAACGACTCAACCGCAGGACAGAACTCGTGCTcAAGGCATATATCAAAaTGCTCAAATAGATCAGTGGACCGGCAGACATGGC

>contig00744

AGTTTtGTTTTTTTtGTAATTTGATCGTTGCCTCCTTCTCGTCTGATCAATAATCCTGGGCGATTATATTTTGACTGATTTGCCCAAAATCGTTGAAACCAGAAATCATCGAGTATTGCCCAGTAGATGATGTTTTACGTAAAAATTCCTTGTTTCGTCCTGCGCTGACGCAACCCAAAGATTGGAGGA

>contig00745

CTTTCAAGAACTCTCGAACGTAATTTAGCCATGGTGAAGAAGGAAGAGTCCGAAAAGGCAAAGCCATCGAGTATCGTTCGACTTTACGAAGCAGCACTTCACAATTTAGTGGAGATATCGCAACTTCAGGATGATGAAGAATTCTTACAAGAACAGGAGGCGAAGACTAAATCCTATCGAGCATTTAGGTGTTTCTACATGGCTCAATCACTTGCCAATGTTCATAGATGGCGAGATGCTATGGTTTtGTATCAAAGATCCCTGCAAC

>contig00746

CGCGACGTTGCTTCATGCCCAGAAGTTGGATCCCCAGAATAAagCAATTCAGCAAGAGTTGtCGGTTATAAAAatCAaGACGGTGAAAGACGCGCACAAGGAGAAAAaTATGTATCGCAaGATGCTGGGAACGAAAAaGGAaTtGCCGATGCTAAAaGCGAGGAGAAGAAGGAAAGCAAATTAAAGACAACCACAATTGCTTGGAGTATTATTGGTGGAACGATTGCCGCAGCAGTGGGAGTGATAGCGTATAAATTAATTTCGTGAATTTGTTTCAGACATAACGTGAAACGAGATAAAAaGTCTCGTTTATCGGTTAACAATAAATCTACCAAGTCCATTTACGATTCGTATGTTTTCTAATTTTATCACTCGTTAATCTCGCATTGTAGCGTGGAAAaTGTATATGTGT

>contig00747

AGTGCGATTCGAATGTCTAGTGACGAAGGTTGACGCTTAACGTCAAAATCCATTTTTACGCTCCGTGAGTCTTCAACAAATGTGCCGACTCTTTtGTGAAAGTGTGTGTGTAACGTTGTGATTTGTCTTATATGAACTTAAATATATGCATATAAaGACCGGTTTTAGTGGTGCGTCAGTTTGAAATTTAGCCGACAATCTGCAAAACGCGGTCGAATGCAATTGAGCAATTACATTAATGAGGAAAAAaTATACCAACCACTGAAACAAGCGAATGGTGATTTTATTATCTATCAGCAAGTTTTTAAAaTTATAGATTTGAAGCTCTAATTGATTACACCATGCGGCTTCACTGCTTTaTCCTGTCATGAAAAGAATGCGGACTTCCTGGTGAAAAATTTATTCTAGCAAAaCGGATtGCTGCCAGTCTCGTGAACaCTGCaTgAacAaCCAtCGAGcaGCCTTTgAaGTATTCCATGTTTGCGCAAACAGAGCATACGACCAGTTTtACTCAAACGGAACAGAACAATTCAAGCTACACTGGTCAGCGACaGACGCAGCGAGCCGCGATGTTCGACCCCcAACAAATTCAGCAGCAGCAAACTGCTCAAGGTCAGCAGTCGCAGCAACAGCAGCAGTCCCAaCAA

>contig00748

ACCAGATTCTTCGTATCGAAGAGGAAATCGGAAGCGCTGCCAAATTCGCTGGAGCCAAGTTCCGTAATCCACAAGCTTAAAGCATTATCCAGACCTTGATTATTATTGTTCTAATTACACTCTAGCTGGTCCTGTTACACTGTAATATAAAAAGGCATTATTTTACGGTCAACGTCGCGAGTTGGCGGTGATTCATAATCCAAAGGCGAGGTTCTACAACCTCGAAATTCAATCTTGAGTCAATCGAAAGGGATTTAAACACGAAATTAGTCTAAATTTAAGTCAGCAATATTTCTACTTTTGAATAAAGCAAGAATTTTTTtAAaTCCAtGCGCACTTTGCTCTACTACTCGAAGTaGAGagCGAAAGTTGCACTCTTCTGAGAAAATTGGTTGATTGGGATTCCTAATTTATAGTGAGACGAGACATTTttG

>contig00749

TCGAGGGAGTGTCAGCACAGAACTTCACTCACCcACTCTGAGCAGGaGTCCGGAGCGAAGTATAGACTCACTAT

>contig00750

GGATtCGTCGTTAATCCTGCACAAGTTCTAGGCCAAATTCGATTCAGAGGTAAagAAGAGCTCGACTCGGACCGTCTCTGCAGCGAGCGTT

>contig00751

AAttGtCTCAGTCTCAAGACGATGAGATTGGCgaTGGAACCACCGGAGTTGTtGTaTTAGCTGGTGCTCTTATGGAACAAGCCCAAATATTACTGGACAAGGGAATTCATCCCATCAGAATTGCAGATGGATTCGAATTGGCAGCACGATGTGCCACGAAACATCTCGTTACAATTTCCGAAAGTTTCCCAGTCAATCCCAACGATCTGGAACC

>contig00752

AAAGTTTtCaGTCATCCTCAAaTGCCAACGAGCTTGCAGAATGTAAAATTGGCAATCTTAACTTGTCCATTTGAACCACCGAAACCAAAACAAAGCATAAGCTCGATGTGACTTCCGTCGAAGATTACAGGGCGCTGCGTAGCTAtGAAAAGGAGAAGTTTGAAGaGATCGTAAATAAaGTGAAGGATGCTGGAGCGACTCTTGCGATTTGCCAGTGGGGCTTTGATGATGAGGCGAATCATCTTTTGCTCCAGAAGCAATTGCCTGCAGTCAgaTGGGTCGGAGGTCCAGAAATCGAGTTGATtGCTATCGCAACTGGTGGCAGAATCGTTCCACGATTTGAGGAATTGACACCGGAGAAACTTGGATTTGCTGGAGTTGTCAGAACCTTGTCGTTTGGT

>contig00753

TACTTTATATTACATGATAGAAGTTGTTTAATCGAGCAAACATTTTCCTATAATCTTATCAAGAGGATAAGGGTGACATTCCAATAGATCTAGGATCTTGTTGACCTGGTTGCGTTGGTCCTTGTTGAGCTGAAGGACCTGTTTGTGGACCGGCTTCCAAATTCTTCAAAAGCTCATTTAACCAATGTTCAGTGGACATATCCTCTTGTAAACAAGTTGCAAATGTGTTGTCTCTTAACTGGTCAGGTAAACACTGCCTCAAGGCTAAAAGAGCTCTGGGATTATCAGACAGACGAAGATAGCATCGAACGACATGTTTCAGCAAGCGAGCCGACGGATCCTTGGCTAGTGAtAAAaCCaTTTTGCCcAAGATCATTGCAACATGACTGAATCTATCATATGTTTGGCAAatATATGATAGTCCACTGTCGTCCAGC

>contig00754

GGTTGCGACTGTTTTGCACAACTCTGaT

>contig00755

CGGTGGTTAaTAAAAACGTAATAACCTCTTGTTCATCAGTTTTCACAAGTGCCCCAATCACTCCCAAACTAGTGAGTCGCAAATACTCAAACGGACGTGTCTTTATAACAGTGTGTAAAAAAGGATACAGAAAAAGGGGAACATGAGCCTGCAAGAATGCaGATCTTGTTTCCGGGTGACTAGCAAGGCACTGGAGTAAGGCTAATGCATTGCACACACGGTTGCTTTGATGTGCGGTAAGAGTTGCGGGGTTAATAGCCGGATATATATTTATAATTTCTTGAAGTAATGAAGCTGCAGT

>contig00756

ACTAACAGGTCATTATTATAGTGAATCGATAAGATGAGTAATCGTCATCATAATCATCATTTTTtGATAATAATTAATTtCAGCGTTTTtAtGCATTTTtAGATTCAGATTCGATGTGTGTCGATAAGTTATATACACTGTGCTGATAGTGATTTTTTAAtGTCGCTTCTTAACTATCTGCGTCGTTACATATTTAATTTGACGAGGTTATCGGTTCGAGCCGTTGTGAACTCGATATTATTATTATTTTTATTATTATTTTtATtATtATTGCATAGAAAAACTCAAACGCAATTTAAAAAAGaTCTCATACGTGAATTCTCTTTCtCTACGATATAATTAGAGATTAGGCATTTTTtATTtGCCGTAATTAtCATTTGATtcGTTTTTTCCCAACGATGAGATTAATTTCACGATtGTCAAAaCATATTGTAATACAAtATtAACGGTCTTACCTAaTGaCCGAAGAAAaCTCGAAATTATAATTAATAAATGTGTTTGt

>contig00757

TAGATTCCTTTATAGTCTTCCATTTAGGCAAGAATGAGGACTATTTAACTAAATTATAAGATACATCAATGCATTCTTTTAAATAAATTTAACAATTAATTTTGACACAACATAGAGAAACTAATGACAACGTATGTATGCTGCACAATATGGTTGAAAAGTGTGTAAATAGCTAAATAGTGATTTTACTAAATGGGATGAGACAAATTTGTTAAAGAGAGTTGATCAATCATTTCTAACGAAATTCATGAAAACTTGTTCATAGTAAAAGTAAGTCTTGTAAAAACTGTTTAAACTTAGCTCTGTGTCACGTTAAA

>contig00758

GTTAACTTTCGCTTGTCTTGGTAGTCTT

>contig00759

GACATTAATTATAACATTTt

>contig00760

ACCGGCAAAACGACGGAAGAAAACCCGAAGGaCCATCAAGTCTGGTgaCGCCGCGATCGTGAACTTGGTCCCCAGCAAGCCAATGTGCGTCGAGGCCTTCCaGGAGTTCCCTCCCCTGGGACGTTTCGCTGTCCGAGACATGCGTCAAACGGTCGCGGTCGGTGTCATCAAGGCTGTCAACTTCAAGGACGCCTCCGGAAAAGTAACCAAGGCTGCCGAGAAAGCCCAGAAGAAAAAaTAACTAGTTTCCGCGCCAACGACGACGACGACATGCCGGCGGATGATCAGAGTGAAGACAaCAGCCAACAACAACAGGACGAAgaCCGGCGCCGCCGCCATTACCGCCGCCGCCGCAGCATAGTGGTTGAGGTAGTTtGCCTTTCTTCTTCTTGTtCTtGTTCTTCTtCTTGTTCTTCTTCTTCTtCTTCTTCTTCTTCTTtGAAAACCTTGACCGCGATGAATAATCGCGTTTCGTTTCTCGACCACTCGGTAGAATCGGTAGAATCaTTAGGACTACTTTCCTCGAGAAAGAAGAGCCGGGGgCGGACGAGTGATGAAGAGCGAGAGATGCGGGgtCTCATTCTGAGGAAGGGCGTGGATGTTGAGCTTCCCAGTTTcTTCAtCACTCGAGTGTTTCATCATCTTTCAAAACGTTTacTAAGaCACTTTTTCCaTCGAAAAGAATTCCGCAGGAAAGATGGGTGATAGATCGGGCCGCCCCCGCGCGCGCGCGCGaGAGAGAGAGAGACGGGAAAAAaGGCAAaCTACGCAGGCTGCTGGTGCTGCTCTGGCTGCTGGCTGCTCTATATGATATTTCGT

>contig00761

ACGCAAATTTCCTAGTTTATTGACAAATTTATGTTAAAATAATGCAATCCATAATGCAATAACATCAATAGTTGATGAAACTACGGATTCAATTTATGATTTCTTAAAaCGAAAAATTATCAGT

>contig00762

TTTTTTTttATTTATTTTTtCTTTCTCGAGGCCATTTTATACGATTCCTGCTAAATTCGGCAGATGTCCGGTCGATGAAAAAATTTGACCAGTACTTTATTCTGCTAAGTTCAGCTTCGTTTCCTTTTTATGACTTCCTTTTCTCGACGTGTCGCTTCTTGTGCTCGGCATTCATCAGCTTGGTAACGATCTTCCTGCAATGGCCTGACGGATCGAAGTGGTTTGTGTGCTGCTCAGCTTCTAGCTTACTTCGTTGTTTTCCGCTGTTGCCGGAGAAAATGTGCTTCTCATAATCGTAGTTGAAATGCTCGTACTCGTCAAAATGCTCCTCGGACATAATTCACTGGTAGATTTTTCTCCGGAGATTCAAAAAAaGGTTTTTtcGAAAAAaTCGCCCTGAAGTTTTGATTGCGTACTgCTTCGTTTGGTGTCCACACCAATACT

>contig00763

AATGAGTGCTTTaCTATTGAaCTAAGAACaTGTTCCGAaTTGTTTCTGCCGCGATATTTTTCACCTTAGCTTTAGGTGGTCGACCTCCACCATTGGAAGAGTGTCCATCTGAAGTCGGGTATGTTATAAATGCTACAGTGACAGGATGTGAATTTAAGAATGAAAAATATATTTTGgaGGATGGTGCTCAAGCTGGTATTAAAATATATTTTtGGACATatgaaaaagTGCAGAACTTCAAAGCAGTTGCCAAAGGAAATTTTGGTAGAAtGTTAG

>contig00764

CGATGATATTtAAaTCtATAATCTTTTTAAa

>contig00765

CTCGGATTCATCGGCAGTTTCAGCATCGAATCTTTGAAGTAAAaCGCGTATATCTTCATTtAGTTGATTCCAATATCGATTAACGACGTTTAACACAGCATCATCCTGAGTTTGCCGTTTTTCtaGTTGTTCTATTCTCTGACGGAGTTCGGCTTCCATGCGATGCCGCTGTTCCAATCTCTGGGCTAGTTTTTtATTTTGAAATTGAaGGACCTTCATGTCCATTTCCTCGAGGGtGGAAATAGGTCCTATGAGAATTGGTTCAAATTGAACTTTTTTtATCGGAGGTTGAGAGCTTGTGTCTCCGCCTTCTGCTGAaCGTTTTGCCATTTTGTCCCGATTTTTGTCCTCAGACTTCGCAGATGAAAAAAaCGAaTCGACGGTGAATTCCGCCCAAGTAAAAGTTTGCTTTATAAGT

>contig00766

ACGTAAtAtACAAGAAGCCTGCCTGCCtGCGAGAAaCTGGTTCCTATCGACCTCAGTATtCGAGTTTCAACTACTCACCAAGAGGGAAATtCGTTGCTCTCAACATAGCATAATTATAAAAACGCAAGATTAATTTTGTAAGGGAGGAGTCTTCGGATCGAAACGAAAAAGTTATCTATATAAACGTGAGCAATCGTTAAGTAATTGTAAAAAAaTTGCTTtCTTTTGTAAATTGAAGGACGGTGAGTAAAGAGACATCAGTCTTGAGTTACTCACCTTCGTGAAACTTAAATTATTAACAGTAGAGCAATTAATAGtCGG

>contig00767

GGAATGCGTTTTGAATCAAGACCTACTAAAAGTAATGCTTTCTGCTGTGTTTTTGGCTGTAATAGTAAGGCCCGCCGAGATCCTACTGTAAGATTTCACAAATTTCCTTTAGGAGACAATTTTGTGGATGTTTCTTCGAAAaTTGGAATTGTAGAGAAGGTGGATAGAAGAAAaGCTTGGGAAATATCCCTAAGAATGGGAAAGAAAGTGACTCCTGGTTTGCAAGTTTGTTCCTTGCATTTTCAAACGGACGACTATATATTACCAGATGCAGCTGCTTCGAAAaGATGTTTGAAGAAAGCTGCAGTTCTATCGTGCAATTTACAGAAGAGTTCTGTGGAGGTTCCCAAAAATCAAAAaGTAAAAAGAGCTAGAGAAGAAAGACACCTTCAGCGAAGCAACAAaCAGGAATaCAaGgAAGAAGAATtACCTGGTATGAATCTaCAAATAGATGCAGTTCAGATAGAAaGCGAAGGACCAAGAATTGATATGGTAGAAAaTTCTGATATCTTAAACATTAACGAAGTGAATGTGGATAGTCATGAAAATTCTGATGAGGATCTTTCGCcAAAGTTCGTAGAAATAGGTGTTCAAAtCAAAAGCGGGGACTTGATGACGTTCGATTTcTtGTCGTTTATTGAAACTGATCAaGAaTTAAGT

>contig00768

ACCTTGAACtcTTCCACTTGTTCCTGTGGCAATTCTATATCTCCATCTGGACGGATTATTTCATCTGAAGCAGTAATATTAGGATTAAGTTCATTAAGAATTTCTTGAAAaGAATATCTACTAaTCTTCTGATCACAGTtCAAGCACATCACTCTGAAAGCAGTTCCATGAAGCTCTATGATATTTTGACTTCCAGcTTTTATGTGTAAACTATCAACATTTTGTATtACAACACAACTAACTTTACCACAGTCTtCtAATTTTTtCAGTAGCTTGTGAGTAACGTTTGGCTCAAAGGCTGAAAACCTTGGCCAACCGATATAATTTCTAGCCCAGtATTTTTTtCTAACTTtaTCGCTATCACAAAAATCTTTGTAaGAAACTGGTCTTCTATCGTTGGTAGCAAAAAGaCCAACACCTTCAGACCTGTAATCAGGTATtCCACTTtCCGTAGAAaTtCCGGCCCcGGTTAAAACGCACATTCGAGCAGAGAATTTTAGATaTTCTTTCAGCTGTTCTAATTCTTCAGCATG

>contig00769

ACTtCCTCATATAAATATTTTCAATGGGTTCAAATGGTTCGAAAAAAGTTTTCAACTGGTCCATGGTCAGACCCTGAAGTGGGAAACCCTTCGCATAAACTGTCCTGGCTTCCTGGGCTTTTCTGTATTCTTCATCGTAGACTGGAAGTGGATGATCAAGGGAGCGGCGAATCTTTGTCTTGTTTTCCGAAGTCTCAATGAGTTCGCTTGTCTCTAGCGCTTTTACAATTGTGGCCGTGTCTTGGGTCATTGAAGCCAGCATTTtGAACTTCAACATCACCTCCAGTGGAATCCAGCCCtcGCTCAATTTTGTTTGCTCGATAaGGAaCTTGTCCCTTTGCATGTTCACATCACCAAaGTAAAaCTcAACTTGTTtCTTGATCTTTtCTAAAaGTTGTGGAGTAACTTCGGGAACTTCGAGGTTTtCCTTATCCTCTGTGACTTCTTTATCCTCACCATTTTTCGGCTTCTCAGTTTCTGGCTTTTCAGTTTCTGACTTCTCAGGTTCTGGCGTATCAGCTTCTGGTTTCACCGGTGTTTCTGGTGTTTCTGCTAACTTAACCTCTTCAGACTTGGCGTCGTTCGACTCTTCAGTCATAGTTGCATCGTTACCGTTCTCCATTCTGCTAGTTCACGTTAATTCAAGTAAAGAAAAATATGGACTCGACTTTCGCAGATCAGAAAAGACTTATAAAGTCTTTTTTCCTGTCTTTTCTTACAAACAAAAAACGGTCCGATATAACACGTGCAAATGACGAGGAGGAAAGTGGAAATGTGTGCGTGCAC

>contig00770

ACAAATTGTGCAAAGTGAGGCGAGTTCAGACTGGACCAAAGGG

>contig00771

CCTTTGATCAAGGTCAACGACTCGATTCAGTT

>contig00772

GAAAAAATCTCGAGTTTCACGCGTTCGCGTGGCTCTAGGTGCCCTTTAGGCTAGTTTCGTGTGTGTGTGACAAAGTTTGGTTAATCGGCAGGATTAAACTGTTCAAGATTCGGGCCCGGAATGCTTTAGGTAATCGAGACATTGAGGTGGCCAGTGGTGGAAACAACGCAAGCAAAATCAACAATCTTCTCGAGAGGATGGCGCTTCCAAGTAGAGCCAGGGTTTACACAGATGTTAATTCACACAAATCCAGAGATTACTGGGATTACGAGTCGTATGTCGTCGACTGGGGACAACAGGACGATTACCAGCTAGTGAGAAAACTTGGACGTGGAAAATATAGTGAAGTCTTCGAGGCTATTAATATCACAAATAACGAAAAATGTGTTGTCAAAATATTAAAGCCCGTTAAAAAGAAGAAGATCAAGAGGGAGATTAAAATATTAGAAAATCTTAGAGGTGGGACCAACATAATTACGCTCCAAGCAGTTGTTAAAGACCCAGTATCAAGGACACCAGCATTGATATTCGAACATGTCAACAACACAGATTTTAAGCAGCTCTATCAGACGCTGACAGACTACGACATAAGATACTACCTCTACGAATTGTTAAaGGCATTGGACTATTGCCACAGTATGGGCATAATGCATAGGGACGTGAAGCCGCACAACGTGATGATAGATCACGAAAATCGAAAGCTGCGGTTAATCGATTGGGGTCTCGCGGAATTTTaCCATCCAGGACaGGAGT

>contig00773

ACCATTAACATTTTtcTTgTCAAAtGAGCGCTCAATCTCTAACAACGTCTTTCCtCTGGTTTCCGGCAAATAACAAAATAGtAAGAAAGTTCCAGTtGCAGCAACGCAACCATAAGCCAAAAAaGTCCCCCATGCCAGGAAaTATTTAAaCaTAAAAGGTGAAATCATCACCACACAAAAAaGGCAAAAAGaTCCAAATCCGGaTGTTAAACCTGTGCCCAATCCTTTATATGACATAGGGAACAATTCCCCAcATAaCATCCACGAAAGTGGAACCAAACCTACAAAaGCTGTAAAGGTATACACAAATAAGAGTGAGACAGGTGCCCATGGTTTtCCAATTTCGAAAAACTGCACAATTGCAAGTAAAATGAGAACCGCAGCTGATCCCATACCAGAACCCAAAGCCAATCCTCTGTTAGAATGTCTTTTGATCAACCAACAGGTCAAGCCACAAACCAAAACTCTCACCACGTCTAAAATTAAGGTCCACATAAAAGCTCCTTCACGACCAGCCACTTCTTCAAAAaTAGTAACACTATAAAAAGTAATTGCATTCACACCACAGCACTGGGCAGTGAAAAACAGGAGACACACAATGCCCAGAGGCTTTAAAAATGATTTCGAGACCATGATATCCTTCCAAGTCAATTCTTTCTCATCATCCATTTtGCTCTTGATTCCAGAAGAAGCCTTTtGTGAAAaTCCTGGAGCGtGGgCTACTTTtAAGTTTTCAAACTCTTTAGAGTTTGTAGAtCCCCTTAGAAACGTCCAAGTATCCAAAGCATTCTCGATTCTTCCCTTCTTCAGGT

>contig00774

GGCGGTTCGTAACCCTCGGGTCTGTCAACCTTGGATCCGGCTTCACCACCTTCTCCTTTGCTGCTGCTGCCGCCTCCGTCGCCGTGTAATTCAAGAAGTTTGCTCAACTCGAAACGGGGCTTCTTCAATACTTTAACCTTTCGGATGTAGACATCGTGAAGGGGGTAAATTCCCTGGCAGGCCTTTTCGATATCCTTGGCAATTGCGTCGGGCAAAAGTTTGCTGACCACGCCTTTAAGATCGCTCTTGGCAATATCGTCGTTGATGATTTCCACCATCTtCTtGCGAATGTtGCGCACCTGGGCGTGTTGGGCGTAGCAAGTTTTCCTCTGGCTTTGTTGATCTTTGTTCGTGAAACCAATGCAGAATATTCTCAGGAGAtATCCATCTGTGGTCTTCACATCAACGTGAGCCTCGATAAGGGTTTGCCATTTCTTGACCATGGACCTCAGTTTGTCGGTGGTTAAGTCCATGCCATGGAAGTTGGTCAGGACATTGCGACCCTGAACATCTTCTGCGATCAGCCTGAATTTACGGAAGGACCTCTCAGCGTCATTGTCGCTCTGAAGATCAGCGAGCGAAACTTCGAATACTCGGAACTTCAGACCCTCGGAGGCGATCTTTGTTCCTTGTGTTCTGTTCACCAAAGTCTTGCCCACTTGGCGGCTAGTGAACATCGATGGAGCTTTGACATCGT

>contig00775

ACCGAAGATTTtCTAATGATACAAACCAATCCAAATTATTAAATCATAATTTACATTTCACAACTGATTACCGAATGAAAAATACTGTCTAGATCTAGTTTTGAGTAGTTTTGTTCTTACATAAAGTTTAATGTTATATATTGTTACTAAAAAATTTGTTAAAGAATTTTCCAGATTAACTTGCAACTAGAGGCATTATAACATGAATCAAAAACATatGTATCAGTGCATTTCACACAAGATGCAATCGTGAaTGTGATAAAAAAaaTaTAATAGATTGTCTTaCTCGTAaTCGCGTAT

>contig00776

ACGATtaTTtGAAtGCAAGgaTGACGGCTAtGGCTCCAAATCTAACTGTTCtCCTTGGAGAATTAGTTGGAGCAAGACTGATTTCCCACGCAGGGTCACTGATCAATCTTGCGAAGCATCCAGCTTCGACTGTTCAGATTCTCGGAGCaGAAAaGGCACTTTtCAGAGCCTTGAAGACAAAGAGAGACACTCCAAAGTATGGTTTGATTTATCACGCAGCACTGGTGGGCCAGTGTTCGACGAAAAATAAGGGAAAGATGTCCAGGATGTTGGCGGCAAAGTCCTCTCTCGCTATTAGGGTTGATGCTTTAGGTGAaGATGTTAGCTTCGAATttGGaGcGGAGcACAAGGCAAaGCTCGAAGCTAAaTTGAGGATTTtGGAGGAAGGAAACTTGAGGAGGATTAGTGGAACTGGAAAGGCTAAAGCGAAGTTTGAGAAGTATCACACTAaGAGTGAGATTCTTCAGTATCCGGCGGCTAATGATTCTACGATGCCGGTG

>contig00777

AAAGAtAGGAAGAAGACTTGGGTTGTGGAGTCCGACTGGAATTTGTTTTTGACGCAGTGAaGgAACGTCGTTTGTTtCCACGGCTATTGCTCATTtGGGTCGAAGATCTCAGTGGACTGGAGTTATACTCGGAGAGGATCTGGATGTCATCATTCTCAtCGTTTtCATtGTTTGCGAGgTCTACAAATATTTCTTTtCCAAAAAAGGTTTCATtGGAACGAAGCCGTTTGCTATCGCTTACAAAgTCGGGGTCATaGGTGaGAGCCTCGTTtGCTGTGCGCTTTCTAGTATTTCGATCTGCCCACGAG

>contig00778

CGGAAAAaGTCAAaCACGTTGTTCACCATGTTCATCATGACCACTTaGCGTGATCAGACATCAACAGCAGCAAATACTCTTTCGTCCTAAACCTCGATAATTCCATTCAGTGATGACCATCAGTTTACGAGGAAAGCATGATTTGTTTGGTCATCACAAAATGCGATGAAGTGACCACGGGTGATGCTTCATGAGTAACGTCAAACGCCAAGAGTCCACGTTCAATTCACATTATCACATTGCAAATTTTTATGTATTCGACGTTTCTGACTTGTAGAAAACCCGCCTTGGTGACGTGAAAACTTAAATTAGGGTGAAAGGAACTTGAACTATAAATGGGGAAAAGGAACTGAAACTTTAATTAAGATGAAAGGAACAGGAGTATATTTtATTTATTATTTTTTATGAACTTTTTAGCTCGCAAAATGCGAGAAAGCACGGAAAGCACTCTGGCAAAAGGGGTTATGTGC

>contig00779

CAGTTATCATCGAAAAAaaGaTCACTGTCTCCGAATGAAAAAAACCaGTATCTTCCCACTTCAGAAACGAATGGTCATTTTGAATCCCTCAAATCACCTTACTCGAGACCACAAACGATCTTAAATTCCGAAAATCTCCGATCATCGATCGTAAATCGAAATTCGGATTTAATTTCTAAAAATTGTGATTTTAAATCTGATGACCGTTACCAAAAAAATTCGAGAACGAGAATCGATAATTGCGAATCGATTATTGATGATGAGGAaGAAATTGATGTTGTCCGAATCGACGATCCTAGCGATCCTATGTGGAGGCCTTGGTAACATCGTTTTTATTtCTACG

>contig00780

TTTGAATATACCTAAGAATCATATAGTGTTTAATAGGTGGTTCTAAATGATAAACAATCCGTTTTTTGTAGAGATTTTATATCAGCAAGAACTTCTGGT

>contig00781

TTCAGGATACATGGCGGCCATTCGAAAGAAACTCGTCATTGTGGGCGATGGTGCTTGCGGTAAAACTTGTCTACTCATAGTTTTTAGCAAGGACCAATTTCCGGAAGTGTATGTGCCTACCGTATTTGAAAACTATGTAGCTGATATCGAAGTAGACGGAAAACAGGTCGAGCTGGCCCTCTGGGATACAGCCGGACAGGAAGATTACGACAGGTTACGTCCCTTGTCATATCCTGACACAGATGTAATCCTGATGTGCTTCTCGATTGACAGTCCCGACTCTCTGGAGAATATACCGGAGAAATGGACGCCCGAGGTGAAGCACTTTTGCCCGAATGTGCCGATTATCCTCGTAGGTAATAAAAaGGATCTGCGCAATGATCCGAGCACGATCAAGGAACTTGGCAAGATGAAGCAGGAGCCAGTCAAGCCCGAGGAA

>contig00782

CGATGAAGTGAAATCAGTAAATGCTGAAGCTGAAAAaCTGAAATCTCAAGGAGtGGAAATTATCATAGTTCTTAGTCATTGTGGATTAGATGTCGATAGAATAATGGCT

>contig00783

CATCAGTTACAACAGCTAACGCAG

>contig00784

AAAGAAGAACGCGAATTCAGGATGAAGTCTATTATTCTAACGACGTTAGGCCGTTTTTGGACTCATGTTCTCATGGCCCTTGTAATCTTCAAACTTGCCCAATACGCTGACAGCCAAACTGGCACACCTCCAGTACTCCCTTATCGTTGCTGTCAAGATCTTGAAAAAGCTGTTTCTCCACTGTGCGTAAACGCTGACTATTATATAGACCCGTATTCTGAACGCGCCAGAAATTGCTGCACAATAGGATGCTCAGATTGGGCCCTTTCACAGTTCTGCACCAAGGCTAAAAAAAAGCACCCATTCCTCATTATTGTGGCAATCTTGAGCGCGCTGTTTTTCAGGTGTGTGGACAAAaTAATTACAAAATAAACCATCGTTCTCCTGCCGCCAAAGACTGCTGCTCAAAaGGTTGCGTCGACACGGAACTTACAAAGTTCTGCACCACTCGTCATTTtGACCCTGTATCAGAATACGACGTTTTTtGCtGTGATGATCTTACATCAACTGTTGAAGGCATGTGCGGAGCAAaTAACTATTATTCGACCGACCcGTCTGGTAAACtCGCTCAACAATGCTGCAACAAaGGTTGCAATTTGGATGAACTTTTTCAGTTCTGCAGAACAAATGTCACTAATGGATAGACAAGACAAGATCTCGGCAATGAAATTGATACCAAGTGAACACAGATAGTATAAATATCGTTGAAGAAAATAATGCATATTCCTAAATAGTGTAAAGAAAATCAAACATGTATAATTTACGTTATGaa

>contig00785

ACAATTATTCtGAGTAGTTTTtATCAATAGAGATCTGCTCAAGTAGTTCTTGTCCATAGCATCATAAGCTGGCTTGAGTTTTTCGAATGTTGGTGACGGTGTTCTCGCCAAAATCCAAAGATTTGTAGCATGGACATACAGTGCATCACTACAGGAGTAAACGACAGATACAATATAATCGgTATATAAAATCCAATATGGGCCAGGAATTTTGAAGAGAGTATCTGGAAAAACGACATTCAGTTCTCCTACAGTGTTACTACCAGTAAATGTTGCAGTCCCTTCAAGTGCCGATGTTCTTCCGGTAATGTTGTTGACTTGCGTGTTCACAACATGTACTGAACCATCTGGATTTAGTGTGTAGTTTGCAGTTAGGCATTGTCCAAATATTGAAAAAaGTGCAAAATATTTCTCTATTTCATACCAAAGACCTAGATACTGTGTCACATCAAAATTCTGTTGTACGGTTACTTGTGGACAATATCCAATACCAGGTACTTGAGCCAAAGCTACAGTGCAAGTCAGAAGAAGGAATACAACGGGAATCATTCTTTAAGCTTTAACAACGCTAAGTTATGAATTTATAGAGAATTCCTAAAAAATTATTGTTAATTAATTTGTCAATAAGAAGAGATTTTTAGGAAATTGTAATGAAAGAAAACTATACCAATTGATACTCTTGGT

>contig00786

ACAgTGcGaCAAGtCTaTTCAGACGGAGCCGATTAGATCGCCGATCAAACGTAGAATTCTGTCGGAGCAGGATCGCTTTtATTACTCCTCGACCGAAGCTGATGAGGCTGCAAATCAAGaGATTGAAGAGAC

>contig00787

GAAGTTAACTCTTTCTGAACTCAAACGACATAAAAAGCCAGACATCACCATTCTGACATACACTACGTAAAGAAGCAGAACTATTTCTTTTTGTATTAAAAATCATAATTTCTGCAATTTTGATATATTATTATACATATACGATCATGATGAAGTTCGTAGTCCTTGCTTTAATGGCAATTTCGATCAGTTTTTCCGAATCTGGGCATGTTGAAATATGCGCAAGAGAAGATTTAATAGTATCTTTATTAAAGGCTTATTACAATATACAAGCAAAGGATTTAAAGGCTAGATGTGAAAATGAGCAAAAAGCTAGGTGGTCTAAAGTAGAAAAAGCTCTTATAACTGTTCAAGAATATACGGATGGTGTAATAACTAATATAGCGTATGAAGCGAAGAGAATGAAAAAaGTTACTGATGAGGACATTGACTTTCTCGTTAGATATTATTGTTTGAATAACTATGATTTCGAACAGGTGAAGCTAAATGTCTTTAAAACATCGGTACCATGCATGGACGATTATAATCTGTCGAAACTCCTAAGAAACGCTGTGCTTGTAGCTTGCCAGACTGTTAAGTATATTGTCAAAAaTCGAAATGTATAATATGTTCAAAaTAGTACGT

>contig00788

CGTGACGACTATTGATATTTCTACAATC

>contig00789

ACTCCACCGCCAACATTTTACCAGAACTTTTCTCGGTCGCGATCAAGGCCTGTTGGTGTAAAGTTCAAGCGCAAGATATCCTTCCGTTTGTTGtCAAGAATCTTCAACTCTCCAGCGACCATGACCACTGCGCAGGAATTAAATTTAaGTCACTGTTATGACTTTGCcTtGAAGTTAGTCATCGAGTCCTCAAaGGTGATTCGAAATGCAATCCAAGGAGGAAAAACTATTGATACCAAG

>contig00790

GGAACTTGAAaTTGGAATCGTCTACAATCCAGTATTaGAACAAATGTTTACGG

>contig00791

CAAAACGAGGACGTGGAGCTTTCTTAAATGGAAAAaGAATACAAAGCTCAAAATGCCAAGATCTTGCACAATCTCTTATCTGTATCGAGGCTTCGTATGCAACAATGGAAGACATCAGAGATACAATTCTTGGTAGgCTTGAAGCTTTtGTAaCAATTGCACATGGAATCAGAACATtGGGTtCTGCAGCTTTAACTTTAtGTTATGTGGCAATGGGTGCAACAGATGTTTATCATTCCGATAATTTGTATCCTTGGGATGTaGCAGCAGG

>contig00792

ACGtATTGCGACCATTAATtACGAATTTCGCTTCGTGATGCGGATTAAATTGTGCTTGGTGTTGGCACTTTATgTACtATGTTCAATACAAGGAAATCCACATAACCGTATTCTTGAAAaTGGTTCGATTCAAAAACAATTAAAGATTTATGAACTTTTTTtATACATCAATCAAGATTCTTTGATTGATGCGACATATTATGAAACTGGTAAAAATTACGATATCACAAAGAATTTGGAAAATTACGACAATGGGACAGTGGTTGAGGATTTCCTTAATCGGTATGAACAAGGTCTACTTCGACGAGATATGTTATATTCCGCATACAACGAGACTCAACTTCATAATATGCTAGCTCTCTACAAAATATTATACTATGCTAAAGACTTTTCAACATTCTATAAAACAGCCAGCTGGGCTCGTTTGCATGTCAACGATCTCATGTTTCTTGATGCTCTTCTAACAGCCATAATTTACCGAAAAGATACAAAAGATATAAATTTTCCGATGCCATACGAAATTTATCCTAACTTATTTTTTGACTCTAAGATCATCCAGGATGCCCAACGGATGCAAATGGCAACAGACGTAAATCACGTGATCAACAACAATTTCGCCCTGATACACTCAAATGACACTAATGAGTGTTCCTTAAAGAACAAACCTAACTCTGAATACAAAGTGTCATACTATTTCGAAGATATTAGCTTAAACGCCTAT

>contig00793

ACGAGAAGGTCGAGAGGTCACCAAGAAAaaGGAGTTtCCCCTACTTCAGACAATCCAATATGCATGAATTCTGGAGACCCCGCAAGCTCCACGAGATGCATAAAAAATC

>contig00794

AAGGTGGCGGAGATCCATGAACACTCGACGGAATCGCAAGAGATCGACTCTTtCTCTCCCTCTCTGCGTAAGGACGGgACAGGCGTATTCATTGGGCaCTGCTTGTCCGTTAGCTTTAATGCTGAACTTCTTTtCTTtCGAACAGTGCTTTACGGCTTCCCAGAATTAAACAAGTCTTTCCGTTtGAATATTACTTATTCCCCCTACAAACATTCCTcTTGGCTTTTTCGGACACACCTTGCCACCTTCCGTTTGAACTCCGTATAACTCTCTCTCCACTCTTTGGCGGCATCTACATTGGCGGGACTTTCATCATTTGGATCAGCCAGCATGCTAATCACGCTTATAAGAATTGTTTCAACTGTGTGAACTGGAAGCCACCGCTCAGAAGCCTTTTCGTAGCCCCACTTATCATCTCCCGGCTCGTGCAaGATCGATATGCACACGTCACCATTTTTTtCGATGTTCGGATGCCATATTTCTGTAATAAATTTCATTCTTGGAGGCCTAAGGGgATATTCTTTTGGGAATTGCAAATGCGCTTTGAAAAATCCACCCTCGT

>contig00795

ACAGCGGATCAGCAGATGAAAGAGTGATGTtCTATTCATTATTTAaTAATATCAAaCATTAATATCATTgaTATCAAACaGT

>contig00796

ACCAATGGATCTAGAAATTGTCGAACGACCGAAAACTCCCCAGGCTCCTGATCCACCTCCAATACGAGAACAGCCAAATATTCCTCCCGTTTTG

>contig00797

CCTGgtCCTCTTCCAGGATTGCCAAATTTGCCGATACCTCCTCGATTCCTCAACCATCCCATGAAGCcAGAGGATTATTCCATGTCGAAGCCGAAAGCTGTTGTGGATCGCGAGAAACCAATTTTACCACC

>contig00798

CGCtAAACTtCCAGCCATTTTCCACGTCAACTGGTTCAGAAAGGCAAAGAATGGAAAATTCTTGTGGCCCGGATTtGGTGAAAACTCTCGCGTTCTtGACTGGATTTTGCGTAGGATAGAAGGAGAAGACATCGCAAAGGACTCAGCCATCGGTTTCGTTCCAAAaCCCGACTCTATCAATTTAGAAGGCATTAACGAGAATATCGACATGGAAGAGCTCTTCAAGTTACCCAAAGATTTTTGGCAAAAaGAAGCTGCTGATTTGAAAGAGTATCTTGAGTCTCAGGTCGGAGAAGATCTACCAAAACCAaTAATGTtACAGCTGGAAAATCTC

>contig00799

ACCCGGACACGATAGTTGTAGGAATGTCGATCGTTTTCAAGTAGG

>contig00800

ACtAtGGGATGAAAaTATATTTTTATAATCATCCCTATCATCTTGTTACAAATAGTCCTTGACCATTTATAATTTCTTTTAGTTTATTTATTTTAATGATTGGAAGAATTAAAATATTTAATTATATAAATTTTATATTAATAATTATAGGATTTTATACTATAATTTTAATTTCATTTCAATGATGACGAGATGTAATTCGAGAAAGAACATTTCAAGGAATGCATAATTTAAATATTCATAAATTATTAAGAATAGGAATATTTTTATTTATTCTTTCAGAATTATTATTTTTTATTTCTTTTTTTtGATCTTATTTTCATTCTGCTTTATCTCCAAATGTAGAATTAGGGATATTATGACCCCCTAAAAATATTAATCAATTTAATCCCTATGAAATTCCTTTACTAAATACAATTATTTTATTATCTTCTGGAGTAACAATTACTTGATCCCATCATTATTTATTATATAATAAAAAAaTTAAAAGATTAATTAGTTTATTATTAACAATTTtATTAGGAATAT

>contig00801

ACGGTGACCTGGCTGAATTTGAGAATTGCTTCCTCTTTGCCTGtCCCAAaTTCCTTtCCCTGGTTCCACCAGGTCCTGGCAAAGCAAACGAGGaCTACGCAAAAGAGGCCAtCAAACACCAGACTCAAGTATTCaTGGACGAAGTAGTCCAGCAAAAaaTGCTACCTACTATCCGTTCCTATcTGAAACTCTACACGACTCTGCCGCtCACGAAACTTGCGACATTCATGTGCAGCTCGAGAACTGATGAATGGGATCTTGATAAGGAAGTGGCAAATCTGACTATTCATTTGCTTTGCTTCAAACATAAGATGAAGAATATTGTCTGGACGAAAGGAGCCTCTGGGTTGGAAGGAAGGTTCCAGTCCGGTTCTGAGCTGGACTTTTATATTGATCACGATATGATTC

>contig00802

ACCACTTTCCGTTAATCGCTACATTTTCTTCATCTGTTGCATTTGTTTTGTTTTCAGTCTTTATGTTAGAACAAAGGTtcAATTtCTTTtGCCATCTTCTTGGAAATGTCTCGGCTCCtCTGtGGCAGTAGTTtCCTTTTtGATTtCTTTTTTtGtCACTGCTGCTAAGGTATTCGTGCAAACTTCATTGAAAAGTGCATCGGTTTCATCGTCCATCGGTTCTGGGGTCGAAGACTTGCCCTCAACTTtctcACTTTTAaTCTCCTCAGTTTTAATCTCCTCGCCTTTAATTGCGAGGGACATGATGAAAAACTAGGAACTATAAATATCAGTGACTTGATGTGACTAGAGACCAATTCTCGAATCCGTCTTCACCTCCAGGAATCCTTGTCGAAAATGGAACCGATGAGATAAACAAGAAGGTGCAGAACACAACAAATAATTGGGTTATACAATAAGAACTAAATATG

>contig00803

GAATCACTTTGGAAACACACAACAGAaCACAAC

>contig00804

ACAGGAGGTCGGCAGCTTCCAGATAAAAATCAGGTCCAGATTACCAACTGCGATGTTCCACCTTGCAAGCTGAAGAAGAAAACTCGCGTTGCTATCACACAGACATTCACACCAAAAAGAGATATTCAACGATTGAGAACTTCAGTTCAAGCCAATATTATGGGCctCCACCTTCCATTCATtGGtGTGGATGGCACTGATGCATGCAACAaCGTTTTTAACACTG

>contig00805

TTTCCCTGGTTGTTAGGAGTGCTCTCAATTCCAGGACGCTCATTCCTGTGAAGGAAC

>contig00806

CAGTGAGGCTTCATGCATCCGCTGGGTTGAGTTTCCCAGTAGCAGGCAaTGGATTTACGAT

>contig00807

CGGTTGCACTGGATTCGACGTGGCTGCCGAAGCTGTCGTCTGGGTTGTGGAGCTAGCAaTTTGCATCAGATTCCcGTTGATGTTtATTACAGTCGGCTGCTGTGCCTGTTGAACTTGTCCTTCAAGCTGAACAGGCTGATAGATGTAAGTCTGCCCATCTGGAGTTTGAATAaTTTGCGGAGTTTGCGGTTGCTGTATAACAATTTGTTGCCCTGCACCcGCCAAACCCGAGACCGGGAGAACTTGAATTtGACCATTTGGCGCCGCTACTTGAATAGCTTGTGGAAGCATCATCGCTtGGCCTCCTTGACCCATTtGCACGACTTGAaCTGTCCCGCCTGCACCTCCAACCATGACGgCTTGGCCCTCTCCAAGCTGATCCATCATTAACTGATTGGATATTTTCGTAGGCACTTTCCCAATGATTGGTCGGCAAATTGAGGACCAATGGTCGACGATAGTC

>contig00808

ACCAAGGCCGCCAATCACGCTGAGACTTATCGTGCCAGCGTCCCAGTGCGGTTCGCTCATCGGCAAAGGTGGATCCAAAATCAAGGAAATCAGGGAAGTGACGGGTGCGTCGATTCAGGTCGCTTCGGAGATGCTGCCCAATTCAACaGAACGAGCGGTCACCATATCTGGCACCAGCGAGGCGATCACGCAGTGCATATACCACATATGCTGCGTCATGCTAGAGTCCCcTCCcAAaGGTGCCACGATCCCTtATCGCCCcAAACCCcAaGTTGGtGGGCCAGTGATCCTGGCTGGTGGGCAAGCCTTCACCATCCAGGGTAATTACGCGGTGCCCGCCCACTCGGACATGGGGAAGCTCGGAAACAGCCCTTtAGCCGGaTTGGCAGCGTTGGGTCTCGgAGGATTAGGATCACCAGCTAACTCCGGTGGACtCAATCCAGCTGactgTtCcctagCACTGGCGGCTCTGGCCGGAAGTCAGTTGCGCACCAGCAACACAAACCGGCAaCAGCCAGCAGCCAACAACCAAACGCATGAGATGACAGTGCCAAACGAGCTGATTGGCTGCATCATtGGAAAaGCTGGCACCAAGATCGCTGAGATTCGTCAAATCTCCGGAGCCATGATTAGAATCAGCAACTGCGAGGAACGGGAAGGTGGAGCCACCGATCGCACAATCACGATCACAGGCAATCCAGACTCCGTAGCGTTGGCGCAATATCTCATCAACATGAGTGTCGAACTGCAGAAAGCTAACCTAGAGGCCCAAAATACCCAAACCCCTGGCAGCGGT

>contig00809

ACTgtCATTGGTCGGTCAATAAGTTTGATTCCCAGCTGTTCACTCGGCGGCGCAATCATTGTTATTATTTATACGAATTTtACACACTCATTCGACAGTAGCGAAAaCTTTATCAGGGTTCTAATTCCCCGGTTCCCcGCcATCCTCGTGTTTtCTTTTTTtACCtCGGGCtGGAGATGATGATTCTTCTTCCTCCTCTGGCTCATCTtCaTCGATATCATCATCGTCATCGTCTTCATCCTCCTCTCCCAAATAATCTTCCTCATCACTTTCCTCGTCATGATCTTTGTATACGGCTTCCAACCCTACGCCACCTTCATCCAATTCATCGTCATCTTCTTCATCAGAGACTTCTTCGCTATCATCCTCGATACCGTCTCCATCTCCATCATTACCATTAACTTCATCATCCTCGCCTTCGCTATCATCAACTTCACAATCGTCGATGTCAAATCCATCCAAATAACGTAGACTGGGAATAAGACTGAAGACTTTCTCTCTGTAATTATCCATATTTGTCGCTTCATTGTTGAACAGGTCCAAGCTTTTCAAGTTTTTGAATTCTTTTAGAGGCTCTAGTGTGTCCAGGTCCTTTATCTTGTTCCCACTGAGGTTAAGATGAGTCAaTTTTGGGCTTGTGTTCAGAAGATTCAACCCACCAGAGATCCGGTTGTCGCTCAATTCCAGCTTCTTGAGATTTGGTAGCTTGGGGAAGCCTTTGAGACTCGTAAGGCCcaCATTGATCAGGCTCAACTTTTCCAAAGCTACATATTCATCTGTTAAGCCAaCGaTATT

>contig00810

AAtGggtAAaCCGGgAGGTGGaCTTGGAAaCATGTAACGCGTTTACATGAAATACGTAAAGAAAGCAGCGCGAATCAACCcTTATTTAGAATATACGGGATTCGCATCATTTTTTTTATTTTAAaCGAAACTTTGTTTTATTTCTCAATGACAAACTATGATAACCATAATTCTCCTTAACCTCCTCCTAAACCGTGAAATCGCAGCATGGATATAATTAAACACGACGGCTCAATATGATGAGAGGATGATAGAGGCGTCTAGCATAAAACTTATctAATAAATTAAGATGAACTGTTtATGTTAATACCGATTAGGGATATAAGACTATAACCGAAGACTCGGCGTTATGTAAGAAAAACTCGTGAAAAATGT

>contig00811

ACTTCTATTCAAAGGAAATACCAATATTGAGTAGAGAACATCAAGCGATGAATTTtAATCAAACTTCTGTGAGCAGCCAACCAGGATTGTTAATGATATCTCCAGGAACAAATTGGAAATCAAAATGATACAATCAACCTCGCTAAGAAAAGAGTGGTAGGCCGACTGATTTTTTCtgCAGTTTTTACCGTTCCTATTGGACTCAAGCGAATTTACGCAACATTTCCTGATGCTGTAATTATCAGAtAATTTTCTAGTCTACTTCTACTCAAGAGgaCTTCAGTTGATTTTAACTCTGGGATGTCTATTGACGAATAATATTGTTGTTTTTTtAAtGGAATCGAAtAAGTTGAATGTTtCTTTTTTTtGCATAGGATTTtCATCTTCTTGATTAATTAATACAGAgCATTTTTtAAatAAAAAAAAAaaTGTCTTAGGaGGGAGCTCCcTGCAGTAGATAGAGCAACCCACAATAGATATCCATCAACTAAAAATACTAATAAATATAAAATAATCAATTTAATTTAATTAaTTGTTAAGATGCTATATGCTTTTATAAaTCAAAaTGCATTATTTtATGTATTATATTCGT

>contig00812

AAGCATTTCTGCGAaTTGATAAaGATCACTATTACTTTGTGCTTTtCTGACGACCTCAATTTTCTCCAaGGTCTCTGCTAATTGTGCCAAAGCTCTTCCAaGAGCAGCTCCTTGTTCGCCaTgA

>contig00813

ACCTAGGAAATACGATTCGGCATTTTCATTTTCAAATATAATAATATGACTAATTGTAGAAACGCGCGCGAATTTTCACGCGTAACCGACTTGGGTGGGGTTGCTTCTTTTCTTCTTCTTCCGAATATAGTATAAAAATTATAAAACAAAAAGGGGAGGACCCCGTCAACATGCTCGCTTCGGGCTCAGAATGTCAGGGTTTAGAAATTCTGACCAAGATTTTTGTTGTTTTGATTTGTTGTTGTTGTTGTTGCGAGAATTCAGCATGCCAACAGGTGGAagCGAACATGTtGAAAaTTCGATtCCTATGGAGGTGTTtATACACATGGTAAGTGTTtAaTAAAGAAGGCCATCAAGGCCTCGTATTATAGGCCTTTCATATATAATATATATGGGATTGAAATTAAaCTCAGAGTTTCCGCTtCCTATCCGAAAAATTCTCAGCTCACTGTTGTGTTtCGTtCCcGACAACAGGCAGCGTTtCGAGA

>contig00814

CCCTAGTGTGTTGTtGTCAGCAGCAGTAGCAACAGCAGCAgcagcagcAgCAGCAGCAgcagcaTCaCTGGTTGCTCTCCcTAAGGCATTATTAATAaTAAAATGAGCGATAaGGAGTAAATGTGTGCTTTCCTAATTCCGTCCTTTTTGCACTTGAGAaGAaTGACAAGACGAAGACGTtACTCTTACTCaCAAGATACTCTGATGCATGAaTCGGTTCAAACTTGGAGAACAAAGAGAtCAAtGGTGGCTTTGAGCAGAtATGCCAAAaCGTAGaTTaTTcA

>contig00815

CATTTCACTGGGAGGAGGTCCTGATGGATACAAAGCAGAATTTCGGGAAGTTACAGCAAATGGTGTATCTTCTCTTCGAGTTAAGGGTTTAAGAACTCAAATTACTGATGATGAAATTCAACTTCAGCTTTCCCTTAGCATCCCAAAAATTAGAGCCACTGCTAAATACAGATCAAGTGGCACTTTGATTTTGGTTCAAGCTAGTGGTGCTGGAGATTATTGGGGTGAATATgATGGAGTCGAAGCCAAAGTTTtcATTAGGGCGAAGCCTTTTTTGGTGCAAGATCGACGATACCTTAGACTTCAGCAATTGAAGATGGACTTCaGTGTTAAAGACAtAAAAATGGGCGTCGACAACGTCCACGATGGCAATACTGTtCTGCAAGCTGCTCTGAATCTGTTCATCAaCAGTTACAGTcAAGAGTtATTAAaGGAAATGAAACCTGATTTAAGAaGAAAGCTCATTCAAGTGATGACCAGTTTCGTTGAAAAACTTTtCGCTCAAGTTCCATATGATGCCTGGATAGAAGaTTAAAAAAaTAGaTTATTAGGCACTATGCGCGCGCACACACACACACAcacAtAtATATATAcTCAGAATTCATTTTTAAGAATAACACAATAGTTGTCTTATATTATACCATATCAAAGTCATATTTATAGGGGGGGAATTAATTAATTTATTACtATTTAAGCATATCGATTTAAAAGTATTTAATTGCAAAATTGACACTGCAACGAATTCCTATCGTTCATAATCGCCAAGGTGAAAATTTtCACTTGGAAaTAAAcA

>contig00816

ACCGATTACAAGAATAGATCCAGTAAAAGAAAAATCCATAGTCCTAACTGGGACAAAGTGCAAGTGAtGGACAGTCAAGTGTGTGGAGACGGCAgACTGTTAGACCTGATTGACGACGCCTGGAGGAAaGAAAAACTTCCCCTGGACGAGATCCTCGTTCCATtATCCGAATTACCCGATCCTGAGAGCGACAACGGCGACTCTCACAtGACTCTGAAAGAaCTTGAACAAAaGTGGAACAACCTAGCTCTCGGCACTCTCAGTGAAAATCATCTTCACTCCCcGACCCCATCGCACAACTAACTTAATTTCAAGTTTAT

>contig00817

ATCCTGACGGACTGCAAGCAaCTAG

>contig00818

TTTAGTAAAAAAATTTCACGGGATATTTTCATTGAAACATTATAGAAATAATAATTTTAATCTCGCTGAGTCGATTGCATCCTGAGTCGGcGGTTTATTTTCTATTCTTAATATGCATGTTTGATAATTTACAGGGTAAAATGTGTCTTATGATTATAATAATCTAAGTTCAATTTTTCTGCTTTTCTTTCGCTTTCTTCTCCATGGCAAGCAGGGCTTTCTTTCCTTCTACTACCAATTCGCGTTCCTGTTCGGTAAGATTTTTGAAGATCTTGGTGATAACACcGGTTCCtAATGTTATACTGCCATCCCTTAACGTGAAAcGTTGACCCTTTTCGGCAACCATTGGCCTGTaGAaTTTAAGAATCAACGCCGAATCTTCTCCTGGCATAGCCATTGCTTTtCCTTCCGGGAGGACCACTTGAGCCGGACAATCCCAGGTTttGCTGAACATTTGAAGTTGCATATAACTCGAGATTGGTTTTTtCcTGCCgCCTTCTCGGGCACTAAGCAAATAAATCTGCGCTTGGACATGATCGACGGCTTTCATTGTTCCCGGTTTGCACATAACCATACCCCTTCTAATTTCGTCTCTCTTTATTCCTCTGACGAGAGCACCCAGCTGATCCCCAGCTTGGGCTTCCTCCAGGATCTTGTGGAACATTTCTATCCCCGTTATGGCACTTTTGATAGTTTTGCTGTAACCGACAAACTCACAATCCATGCCCTTCTTCACGATTCCCCGCTCGAGTTTGCCTGTGACGACCGTTCCTCTGCCGGGAATTGaGT

>contig00819

AaTGTGGATTTGAAATCTATACTGAAGATTTAAAGAAGAACGCGAATTCAGGATGAAATTTAGTATtCCAACGAGCTCAAGCCATATTAGGACTTATGTCCTTATGGCCCTTGTAACATTCGAACTCGCAGAATTGGCTTACAGCGACCATTTTTCAACTCAAAAACTCACTAAGCATTGCGGTAGCAATCTTATAACAGCTGTTGCTTTAACGTGCGGAGAGAATAACTATTTTATAGACCCATATTCTACTGACGCTCAAAATTGTTGCAAAGTAGGTTGCTCACCTTTGGGACTTTCAGTGTTCTGCAAAGGGCCACCTAATCCAAAGCGACGACGTTATTTTTTTtGCGGTGTGGAATATTATAGCGCTCTTAATTCGCTGTGCTATGATGGTTATGATCTAACAAAGGATCCTACCCTCGAATCAAAGTGCTGCACGAAAaGTTGCACATTATTAGAACTTGAAAACGTCTGCAAAAAGCGaCCTTTTCCAAGTCCACCACGTTTtGTGCTTTGCGGAGCTGATTTTTATACCGCTCTTGATTCACTGTGCCCAAATAAATATTATGTTTCGAAGCATTGGGACTTACAATCACAATGCTGCTCGCCAGGTTGCACATTATCGGAACTTGAAAACGTCTGCATCATACCAAAAGCAACTCCCTACTAAGAATTACACTCGCTCGGCAAGGTGATGCTCTGAAAATGAGATGTATATCCAGTGAACACAGATGGTGGACTTTTTACTGAAAAAAGGAAGTGTGAATCTAACTCCAATTTTAGAGT

>contig00820

TTCTTtCCAAATGTAGCTTTGCTATTTtCGCTAGATTCGTTGACCCACTTAGTTCCATTGTCGTTCTTTttGGAAGTAGGTTTATtGACGTTCCTCTCAGAAGTCAGGGGTAGAGGAATACCGTTTACTTGATCCTTGcTTTttCGAATTGTAGGAGACcATGATTTTTTAAACTTAGGGCTTTGTTCTAGGGTCGAGTTCTTCTTTGGTGCTGTAATGGGTCCGCTTGGCTTAACTTTCTTTGCCGGTATGGGTCGCAATGAAGAGTAAGGAACGGTCCTcTTTtCTCCAAGTTCAAG

>contig00821

TAAAAACGACAACGGGTCCTTCATTTTtCGtCATTtCTtGAATGTGTCCACTTAAGAGAATTGAGtCTCTcTTTtCCGTTCCACCTGAAGCCCTTTCGTCACTGAATTCGTTTtA

>contig00822

GGTGGCTTAGTTCGTTGTTATTGGCAAGGTCATCCGGCTTTATATTTAAAtCCACTAAACATTTCCcGCCAaCTTGGAACTCCATTCCATTGAATTTAGTCCATCCGGAATTTTTtACTTCCCGTCTGATTTCGTGCCATATATCAAAATCAGTATTTCGGTAAATATCAGGATCTAGGGCTTTTGCAACACGATAAGGAAACGGAGTATGTCCCTTATTAATCATCTTTATTTCGTCGGTTTCAGTGAATCCTTCCTCCATTTTAGACAGTAACTCCGCTGCCAGAGATTCTCTATTCTCCAAGTTGCCCTTGGGAAAAAACTTTTCATGTCTAAACGAGTGGGTCCTATCATGAAGCATCTTA

>contig00823

CCTGGGgCTTTtCGCTAAGACATCGATCATGGCGGCCCGTTCGATTTCAAAGAAGAGGAAGTTTGTAGGAGACGGAGTTTTCAAAGCAGAGTTGAACGAGTTTTTGACTCGTGAACTCGCCGAAGATGGCTACTCTGGAGTTGAAGTGCGCGTCACGCCCCAGCGCACTGAAATCATCCTCATGGCCACTCACACCCAAAGCGTTCTTGGAGAGAAAGGTCGCAGAATCAGGGAATTGACCTCTGTCGTCCAAAAGAGGTTCAACTTCAAAGACAACACCGTAGAACTCTACGCCGAGAAAGTTGCAACGAGAGGTCTTTGCGCCATCGCCCAGGCTGAGTCGTTGCGATACAAGTTGATCGGAGGTCTTGCCGTGAGGAGGGCTTGCTATGGAGTTTTACGATTCATCATGGAATCTGGAGCCAAGGGATGTGAAGTCGTTGTCAGTGGTAAATTGCGTGGACAGCGAGCAAAGTCGATGAAATTCG

>contig00824

CCAAGGAAATCGAAATTGAAATCGAAGAAGCCAAAAAaGATATTGAGCAGACGAAAaTCGAATTGGAGGAGGCAAAaCGCGTTCGTAAAAATAGGATAGAATACGACGTTTtGGCCAAGGTTATTAACGAACAACCTGATCGTGTAG

>contig00825

TGCGAAACTTGATACACTACGAGATGAATTGGGAAG

>contig00826

GGATGAAGTCGACGAaGAAATAATGGACGTGAGTCTC

>contig00827

ACGAGGATACAGACGTTGTAAAGTCTCC

>contig00828

TAAATATTCAGGTGAAAATAATTCGTGCGAACTTCAAGTGGAAATAAAAGCGAAAAGTTCACGTAAATCCTGCATGAAAAAAGGTTAAAAAAACATTTGACTTGACTGCTGATTATATAACTTATAACCTAACTTAAAATTATATCAAACTGAGAAATTGAAACCCCGAAAAAaCCAAAAACAAGCGGAAATAACAAGAATGGAATAATGGAAAATAATAAAAGTGTAAATCTGTGGATTTCCTGAAGGATTTAGATTATTAGCCAAGAATTGTTTTGTGCTtCgaTGCGATtCCAGGTTTACCAATTtAATGGAAAaTATTGGAACACGTCGGCTATGGGTAGAAGCAATCTGCCTGACATGCGAAATAAAGGAtATTtAGTGTCTtCAACTCATTCTGCTTATCTTCACAGACATGAAA

>contig00829

ACTGCTGGGTTCCcAAGATCACATGCTTGCTAGAGTCGCGAACCaTTGCTCTCGAGaCGTAACCGAATTTAATTTGATCCGATCCTGCGAGCAAAGCCTGAACAGTCCACTTGGCCAATTTGCAAGCGTTATTTCTCAGTTCGTTGGCCAGAACAGCACCGCGTTGAGAATCAAGCTTTTGGCGCCACTCAACACCATTCGCGAGTTTAGAATCCCACTCGTTCAAAGCTTTCACAGTCAAGAACTGGGTATCGTTATTGGGCCCTTGTATGACTGCATCGTGCTCGCATCGAGCGATCAGAACAATGCCGTtGTtCAAGtCCCACTTGCGGTAGCGATAAGCTACACTAGCGACATCTTCTTCCTCTTCCTCGGAAATGAAGGGATTT

>contig00830

AGTTCCAGGACAGCCGACTTCTCAAGCTGCCCCGCAGCCAGGAGCACGCTAAATCTCTTAACAAtGTGTGATAGCACGtAaCTTGAATGCATTCGCTTAAGTGTCTCACACACTATTCATAACCGCCACCATCGCCATCCGAAGGGTCAAaTCAAGCGACTCTCTAAGTCTGGAGCAAACGATTTCAATCGTATTGAATAGACAAAATAAGTAGATATAACAACCACCGAGTTCCAAATGCTTAGAGTCTCGGTTGATGCAACTTTTCGAGTCgAAAGCCAAGCAAAGTCAGAGGCTgACTTtCATTATTAATCGTCATTATCTCCATCATCGATtATCACTGAAGTAAAGTCATCCGGAGTCCAGTTGGCGAGTGATTGAACCTTGAATTATACACACAC

>contig00831

ACTTGTGAATAATATTTTAGTAGAAATAAAGGCGTATTTACTATACTTTTGGATCGCGCGGCGACATTGTAAATACtCACGACAACTCGATGATTGTGTTTCAAAAtGGCTACATtGCTTAATTGTATATAAACGAAAAGTCTCAACTAATATCAGAGCTAGATGATGGTGTTAGTGAAAATTCGTCTTTGCCGTTAGAAAGAAACAATGGTTTGCCGGTCGATGATCTCGAATCGGCATCAtCGAGCTGCGAAGATATCTTCCTTTGTTaTCCATCGATAACCAAGTTCTGTTAATTtATTTTTAATACACCGCCTTTTTGTTATTAAGTATTACTACGTATTTATTGTGCTCTCTTTCATTTtGATAGGTTTACTGTTtAACCGAaTATACGTAAaCGTCTCTAGGTCGCGAATAGATTGAAACCC

>contig00832

CGTAACTTATAGATCGTTTTACTCACATAACCAATCTGGATAATAGAAATTTATCGTGTAAAG

>contig00833

TCAAGCAGCAATGGTTCCATCTCTAGTGGCAGTATTTCGAACGGAAACTCGCACAGCAATGATAATACAGATGGTAACCCGGTAACCGGAACTGGATACGCAGGAGACCAGCAGAGTAACGCGATGTCAGTGCAAAATGGAAGTGGAAATGCAAATTCGAGCGGCTCTgAAAaGTCGACAGATTTAGCTCCCGGCACCGGCAGCATGTCCAATAAGGTTCAGCGTAATCGCGTTCCTCCTGGTGGATACTCCTCcGG

>contig00834

CCCACTTGAATTTCATTGACCTcTAAAAAAAaCTGTaTTCG

>contig00835

AAaTGAAAaGGGCTGACATGGAAGCAATGTGCTCTAATTtATTCAAAAGAGTCGAATCAACTCTTAAGCAATGCTTGGCTGATTCTAAATTGAAGTTGGATGATATTCATTCTGTGGAAATTGCTGGCGGCTCGAGCAGAGTTCCAGCAATAAAaCGTTtGATTGAAGAGGTGTTTTCTAAAGgAaCATCAaCCACTTTAAATCAAGATGAAGCAGTAGCTAGAGGTTGTGCATTACaGTGCGCGATGTTGAGTCCAGCtGTTAGAGTTCGAGATTtCTCCGTGATCGACATTCAACCGTATCCTATAAAACTAaCTTGGGATGCTTCTCAGGgTGAAGAAGGACAAaTGGAAGTGTTtGGACACAACCA

>contig00836

TGCTTGAGCCGGATCTTTCACATTAGGTtCGTTCAGTAGATCTtGGATTCCCAAAAGAATTTGCTTGATTGTGATCGCAGGCCTCCAGTCTTTCTCTTCATCGAGAAGCGACAAaCAAACGGTTCCGGATGGATAAaCATTTGGATGGAAGAGAGGAGGTTCGAATTTGCATTTtGGAGGACTCGAAGGATAATCGTCCTTGAAAaTCATGCGCAATTTGTAGAGTCCTCCCTCCCAAGGTGTGGATTTTTtGCCGGGTATCGCACACTCCCAGCTCATCAGGTTAAGCGTGCCatCCGGGTTTTTtGTCGGGCGCGCTACAAaTCCGAAAGGATGGTCTTTCCTCCATGCTTtCCTTtCTTCCGCAAGCCTAGCTATTGCAATGCCTGACATTAGTCAGTTTGATGTGGATGCCGCTATATTCAAACACAAAGCACTTTCTACGCCTAAA

>contig00837

ATCAAACACTTTtAGCGGCACAAGCGAAGCAACCGATGCT

>contig00838

CTtgaCCCTGTCCCcAAATGCGGAAAATGCTTTGCTGGCGAAGTCaTTAAACCAGCTAATGATTAAGGGATTAGCCCAACCACCCATCTCTTGAATTTTGTATGGTAAATCCATGTCATAAAGTGTTACAATCGGTGTTATTCCATtCTTTAGCATCTCATCTATTAAATTATTGTAATATCTGATACCGTCTTTGCTTATGTAaTCTGAGAATCCACTGGGAAATATTCGAGTCCAGGATAAGGAAaTTCTGTATGTATTCGCACCAAGTTTTTAACAAGATTCACATCAGTTTtGAATTTGTTGTATGAATCAGCAGCCACATCTGCATTGCTCATATCTTTTACCAGCCTTGGATTTAAATGCACAACGTTATCCCAAAGTGACTCTCCAATGTCTGTAAGATTATAACcGCCTTCTATCTGATAAGCAGAAGTAGTAAATCCAAAAAGAAACTGAtCTGGAAAACGATTTTCAGCAAAGGAaTTGGCAGCAACTATAGACACGTGTG

>contig00839

TgTACAGTTTGAGGGAGTAAGGTTATCATCAACAGTCAAGCTGTCTTCGTGTCAACCCTATAAAATAAGTTGTTATTCCTTCGTAGTTATCATTTATTAATACTGTTAGTTATTGATACAATCAGTGTATATCGTCATGTTACAAAGCGCCTTTGATGATCTGAAACGTATGAACAAACGTCAGTTCCTTTACCAGGTACTGAGTTTCGGCATGATCGTTTCTTCAGCCTTAATGATATGGAAAGGTTTAATGGTGATTACCGGAAGTGAAAGCCCAATTGTTGTTGTGTTAAGCGGTAGTATGGAACCAGCTTTTCATCGTGGAGATCTTTtATTTCTAACGAATTATGAAGAGGAACCTGTTCGAGTCGGCGAAATTGTAGTTTtCAAAATCGAAGGGCGTGACATTCCAATAGTACATAGAGTGCTTAAGTTGCATGAAAGAAACGAAGAAAaCAATACTGTGAAGTTTCTAACTAAAGGCGATAATAATTCAGTGGACGACAGAGGCCTATATGCACCTGGTCAACACTGGTTAAACCaTAAAGATGTGGTTGGAAGAGCACGAGGTTTCCTACCATACGTaGGCATGGTCACAATATACATGAaTGAGTATCCAAAATTCAAATATGCTATATTAGCATtCCTtGGTCTTTAtGTATTAATTCATAGAGAGTAATATGTTTATATTGTTACGTTTTtCAAAAAATAAGTTGCTAAAATATCCTCGGAACCATTTTTtATGCAAT

>contig00840

AAtCTTCGAaTaTTTtaGT

>contig00841

ACacTTTtCCTTtAGAGACATATACGTGACTGGTTACTTCGCTAAATTAAAaTTtAAGAATTTAATAATTATACGATACTTCGTAaGtAgaCAGAAAGAaTtAACACCTGGAAAaTTAaTAATAGGAGACCCTCCCCTTAGACACTACGCATGTTTGCACTTATATACACTACATTGTATTTCTTAGCACGTATATTTTGTGCTTGGTTCCACGTATAAATTCAATCAAGCCATTAAATACTTTTTCCCAAATACGCAAGTGCGTAGCGGTCTTTCAAAATGAAATTCATAATTTTTGATATACATTATGCACTTTTTtCAGGCAAGTTATCAACTTTCAGTTCACTTCCTTTtCGTCAAAaTCGATGCCAATAGAGCGCGAGCTTGACCCATGAAAGCAACAAATCAAGGACTTACTGTATCAGTCGTTTCAGCTTAGCGATTCAGGGTCTCCTCGAGCGAATTTATTTtCATTCAGAGTCACTCTCGGCCTGTGATTTCTTACGAGACTCGCGATTCCGGGTAGCGCCAGGAGTGTTCTGAGTTCTTCTTGCACGGgTTACACGACCTtcACCGGATGCGGCTTTGCTTGGAGTGGATTCGGGAGCATCGCcTATCCTCTCATTTTGCTCCTTTGCCTTCTTGCACTTTTCCGCGGTGGGTCTGCCGGACACACCATTTTtCTCCAAGAGGTCTTTCAGACACTTGATGCGGTCGCTATTAGTCTTGCAACCCTCCCAAACATTCGGGTATTTTGCCTTTATACCAGCCATAGCTATGT

>contig00842

TGTCgCTgCcACGtGGAAAatGAtCGAAACTtCGTTtATtAGAAGTtCTCGGTCAGaTGAaGAAAGTCCTAGGCCTTCTAGGGCACAATCGCCTGCAATGGCAATGACTTTGTGACCGAATTTCGGCATCTTAtCTTTCAGAACTTGAAAGATCGGATCTTCGAATAATTCTTGCATTCTGTTTTCGATATTTTTCTCTTTCTTTGTTCTCACCAAAAGATAGATCGTCGATAAATTGGGACAACTTCTTAAAAGCTTTTCAATGaGAATTTTTCCCAAAAAaCCAGTTCCCCCTGTGATGAAAATTTtCTGCCCAGcATAAAATTCTTGGATAGGAGTTTCTTGTCTGATTCTGAATCCGTTGCCACCAATAGTATTAGCAATCTCATTGTTGTTTGTTTCATCTTCAATGATTTTTGAaTACGCATTCTGCGATtCTCGTGGAATGTCAAGTTCCTCTAGCAtCTtATTTtGTATGAGGAGCGTTTCCGGTATCGATCCAAGGACTCCGATGGCGAaTTtGAtGATTTTGCGACAATCAAGTCGCTTTTTTATTGACACGTTCTCTTTTCTTCTTTAGT

>contig00843

TAGAAGGATTGAGAGCTTGTGATCCAGATGGTTATGAATTCGATGCCATGGATTTGCCAAAATCGC

>contig00844

AGTGGAGAAACAAGTGTTATAATATCCAAGAAAAGTTTCGTATCAAAaTTCCTACTTGTAAGTAATTTTtAACTAAaGaGACTAAAGCTGTAAAGACTTCTCAAATACACTAAAAATTCTACACAAATTAATCGTCGCGAAGGTTTTAACTTCTCGTAAAaTAATAAGCTACAAAAAAAaGGGTGATGACTTGAAGAAATTCGCTTCTACATCGATAGGTCAACAGAACATTTAAAACTGATTATTCCTCGGATTATTCTTAAAaTATTTTGAATGAGAAAAATAGATTATCGTAGGCACTGCAACTTTGTTACAAAaTAATTTTCCAGGAAAGGTAGTCCCTAATTTTCCAAACGAAATGTCCCAACATTTCTGAACTATTTGGACCTTTCGACTTGTGGAAAATTCCCTATTTGCAGTTTTTAGAGAGAGAAATGTAGCTATACAAGATATTATAGTCAATGGCCTAATGCCTAGAGGAAACCGTAATACTTTtCACATTTTTTTtAAGATTAAGtGCAGCCGAAAaTATTTCTGCACATGGGAGAGACTTTTCTTTGATGACTCAACGACAGTATTTtGGAATTTCGACTTTTTGTCGCTTATTCGGTTCCATTTCGGAAAAGAACTCTTGAATATCTTCGGCTCTGACGACCTTCTTATTGTCGGCGAATTTCTGAAGGT

>contig00845

ACGAAGACGCGATCGGCAAAGCTGTTCCCATTATGAACTCCACCATGAAGCCAACATCCGCTGCCAATCAAACTCGCACCATAACTCAAACGCCCGCAGTCAATGTAACCCGTAATCTCAATTCCGCTGCGAATGAAACCCGAACTCTGAATTCTGCTGCCAACGAAACCCGCAATCTCAATCCCACTGCAAATGAAACTCGGATTATTAAATCCAACTCGAAGGAACCACTCGA

>contig00846

CTGGATCAGTTCCGAATTCCACTACGAAAAAAGTGGCAGAACTCGCCAACGAACGAAACCaTTCTCCGTTTtCTGTTCCTctGTTGATTCCAAAGATCGCGGTTAGCAAACTGAACCCAGTCGTGGAATTAGATACCTTAATGACTGACGATGACTCCTCTCCAGAGAGGAAACTTCCGAGAG

>contig00847

CATCGATAACAGTCCACAAGCTTTTGGCTACCAGTTGGAAAATGGTATACCAATTGAGAGCTGGTTTGCTGATCGTTCAGATAACGAACTGATGAAGCTGTTACCTTTTCTAGAAAaTCTT

>contig00848

CAGCTTCTGtAAATTCTT

>contig00849

GCTCACTCTAGCTGAAGCTTTAAAAACTTTGCGAACAGGTTTTGCTTTAACAACAACATAATCGTCCTCTTTCTCCATGTTATAAATCAGAATCTTATGATTGTTTCTTTCCACAATTTGTTCATAATTTTGCAGAATTATTATAGTTTATTTtATATTATATTAAATTCGTGATCAAGAAAAGCTGTTAGCTATATACCCTTTACATTGAACCACCACAGAAT

>contig00850

ACCACTAGAACTTAAATcTTAAACAAAAATCTTAAATCTGAAAAaaGAagaTTTCCCAAAAGcATTTTAGGatATTaTTCTTTTTTtGTTtATCACTAGTCAAAAGGGCgATTTGAATGTAACTGTGCCCTTcTCGAAGATTtATTAAtCTGTTACGTTCTTGCTGTTGAaCACGAATCCTGTCGCCCCTTTTTAgagggaTTATtAcACTCGTATGGcATGAGACTTCCGTATCAGTTATAGCAGAAACTGTCGCACACTTtGTGAGTTTTtGCGTTCCAGATGCACCTCcTcgAGAaCTTAAAaGAaTCCAGTAGGAATAATGAGTGGATTCTCCAAAGTAAAATATCTGTGTTGAGATCATGTAGAGGCCATTGGTGgTAACTTCTAtAGACTTCTCACCcTCGTCTAGATGAAATTTATTGAAATCGTAGCGCGATTCGTTtCTGGAACTTGTCACCCAGGGTCCGATGTAAATATCtGAGTCAGTCACGAGTTgTtCGGGTCGAGCTCCAACAAAaGTtGCTATCAAaGGTCCTCTGCCATCGTCATCTCTTCGCGCTCCaGCCGCTCTATaCTCGCTGCTCTTCCAGTCAAGatcatcatGATCATCAtgatcatcaGCCTTATTGTTGTCGCTCTCGAGCCACGAATCAGTCaaaTCGGTtAAATTCCTTTTCTCCAACTTCTGACTTTTGTTATTCAAACTTtGGtAAATGCGTCCTCGTTGGaGAAAGTTATCAGTCTTTTCtaGCTTATCGT

>contig00851

CTGCAACACTTGCAGTTGTCCAACAGCTCCACAaTAATTTCATGCCAAAaCTTGTATTTtGAACTCCAGTCTTGAGGATTGACAGATCTATTtCGAAAGTCTGCAAACAGTGAATTCATCCGGTCGTCTtGTTTCCAGCATTCTGGCATTTtATTTtCAGGCAGGGAAGGGATATTGTTAGGAGAAAGACTATCTTTCGATGCCATGTTTAAAAAaTGACAGAATATACAAAAACTTCTTGAGACAAACGT

>contig00852

ACTCTGCTGTCGGAGAATCGGACATGCAAAAACTGAGTGGTAACGACTCTGCTTGCCCAAGTCTATTTCAATAGGCAGCTCGTCCTTTCCGTTCCAGATACCTGTGACTTGTCGCTGTTGCATGACTTGCTTGATATTCAAGAGAGC

>contig00853

GtAAAGTTCCcATCAGGGACTGCACCTCTTTTtCATACCGGCTCACAAaCTGCGGTAGATGTTTCCGGGCATACATTATCGCCTCGGTTTGTTtGCTTGGCCcTTGCTGCACTAGCCTTATAAATTTCAGTCTATGTAATTTGAATTCAAGCGATGAGCTTTGTGCAAGAAGAGCGTCCTTGTGTTTTGTCGCCCATTCGAGTGCTGGTTCCAGATTTTTttGCTTCAAaCAATCGAGGATATAATTTAATTCCGTGAAGGGCTCCTTTCGGCCTTCTTCAGTCTTAATTCCAGCTTCCGCTGCAAGTTCATCCGCAATATCCAGCATGCCTTGACGATAAAAGTGTTGGCAGATGACTTGATTGAGAAGATGTGTCTTTTCCGCTCCCGAGAACACATCTTCCCTGCTTGTGCTGGCAAAATCCGCTATGAAATTCCGGTCAATCGCTTTGCCAACTTTCGAGA

>contig00854

ACTCAACAATTTAGTTTTCACTGTGCTCCAGCGCTCGACGTTGATTGGCAGACGAATACGTCCTTTGCGAGTTGCTCGACTGATCAGTGCATTCATGTCTGCAAGCTCAATGTTGATAAGCCCATCAAGAGCTTTCAGGGACACACGAACGAGGTGAATGCGATCAAGTGGGATCCACAGGGAAACTTGTTGGCCAGTTGTTCCGATGATATGAGTTTGAAAATCTGGTCGATGAAGCAGGACACaTGGGTCCATGATCTCcaGGCgCAtAGCAAAGAAATTTATACAATCAAGTGGTCGCCGACGGGAGCGGGCAC

>contig00855

AaCTCTAGCGAGTGCCTCG

>contig00856

CGACCTCTTCCtCTGCCGCGCATATTGTTTCCTCCTCGATTCATCCATCCACCGCCGCCGCCTCCGCGATTTCCTCGAGGTCCTCCTTGGTGCGGTCCTTGATTTTGCCCCTGATTtCCTTGCATGTGACCTGGTGGTCCATGCATCATGCCATCTTGCtCcTGCCCATCACCACCTcGTTTTCCCTCGCTACTaTACTGCGACATTCCACTTCCGGCTCTCGCACCTCCATGACTGTCACGTTCACGATGTGATTGTTGCACACCCccGGGCGTTCGATTTTTCCCCAATCCTTCCAAcATTTCCCGACTGCTGCTGATGCTGCTGCTGTtGTTGGTGTTGTTGTTGATGACCAACCTGAATTCCTTGATCTGTCGTTGGACTCTTCCTCGTCATCAaGTTAGCAGTTGGAGTAGTTGATCGAGATCCTCCACTTATCAAATCTAAGACGCTTGGCTGTTTGTTCATTGGTCCGCGTGGGTCCCTGGTTGGTAT

>contig00857

ATCATTGGATGACCATAGCCAGGTTGAGGTTGATACGACTGCTGGCCCATCGACGCCTGCACTGTCATCTGAACGATCGCTGGATCATTGGGTATCGAGCTAACATTGCTAACTACTCGAATATCCTTTATGTCCGTTCCACGGAAGAGGATGTATTCGTAGATCTGATTTTGGGGTGCAACCGGAATTTGTGTTTCGCGGTCCT

>contig00858

TTTACCTTCCATACATTGCGTTCATTATATTAAAATGTTACAAATAAGATACACTTCTTTTTACACACTTTATCTGACAATGAAAAACCGCTGAAATATTTGCGTATATTCAAATAAATACAACTTCCCTTATATTAAAATTATATTGAAACTAGATCTAAACATTTAAGATATATCTAAAGACTCCTtCCAATTACTAATTGATTACGAATAAAAGTAAAaTTGAAaCAAAaTAGTTGATTTTaTTAATTAGA

>contig00859

CATTTCTCATTAGAATTTTTTCACCGAATGTaTTGTAATTCAAGAGAAGAAACAAGAAAaTTTACTATCGCCCTGTTCCGCCACCACCAGTTGGACCACCTTCAGGATTTTGTGCCGACAGCATGACGAATAAGATGAAaCCCGGCACAATGAACATCCAGTATTTAGCTAAGAAGGATCTATTGTCCTTGACATCTCCTCTTTCCCTAGCTTCACGTTCCTTCTCTAACTTAAGTATATAAGTGGCTGTGTCTGGCATGGGACCACCGTCAGGACATTTTACTTGAACGTTGGTAGTCCACATTTTTGTCGATGGCACTTCCACATGGCAAGGACCACGAGCCACTAGATTTATTGCTACTGGTTCTGCTGCACTCCCGAGCCATATGTATAGCAAATCCTCCAAATCCGTCCCCAGCAAATGACACGCGAGT

>contig00860

ACCtAGtGGACTTTTTAGCAACTTGAAAAATCTGAAATCTGTCCAACTTGATGGCTCGAGCTTGGAAAATCTGCCAGAGGATATCTTTCAGGGTTCACCAGAGTTGAGGGATATTTTTTTGGCTGGAAATAATCTGGCGACTTTGCCTAAACAAATTTTTGAGGGTTTGGGCAAACTCCAGAAACTATTTCTTCATGGCAATAGGATTGAATATCTACCTGATGATATTTTCAGCACAAACCAGAATTTGGAGGATCTGAACTTGAACCATAATAGCATCAAGAATATTtcTGGCTCGCTGCTCTCTGGTCTGAAaTCGTTGAAAAaGTTaCAAATGCAGAATAaC

>contig00861

ACCttGATATCCaTCCCgAaGGAAAaGTTCCAGCTTttATTGGTCCAGATGGCAAAGTAGTtGTTGAAtCAGATGTCATCGtCAATaCTTTAgaCGAAATGTATCCGGACCCTCCACTGTATAACGAAGAAACAAAaGTCGAGACAtGGAATTGGTCAAAAaTTTTGGAAAGATTATTAGCATATTTTCGGACTGTATCCATGgAAGAGACAGtAGACCTCTTGATGAaGTCGTCGCTgAAATATCGAAGCACTTAGAACAATTCGAGGAGGAACTAGAGACTCGAGGAACAGATTTCTTCGGAGGTGATAATCCCGGCATTGTTGACATTATGATCTGGCCCTGGGTTGAACGATCAAAGGCACTTCCGCTAATCTACAAACAACCACTAAATTTCGAAAAAGAAAaGTTCCCTCTCACAATAAAGTGGGTTATGAATATGAAAGCTCAAGACTTCGTAATTGAAAaTGCAGGACGAATTGAAAAATTCGCCAAATTGATTCTGTCCATGAAAGAGGGAAACGTCGACTTTGATTCCATCTAATTAATCTtCTTTTtCGCTCGAAaCATATTCCAAAAaGAAATGTGATAAACAAAACTAAAAGTAaCAaTAAGATTAGCGTTGCACATAGTGCAGAACCATTTGCTtACAGATCTTCCTAAAGAAAAGCACAGAATACTTCTCGCAAATATTCTAAACATatCGAGTTTATTACGAGAATTACAATAATGAATAAAGTCGCTGAATAAATCAACCGCGTAAGATACAAAAATG

>contig00862

ACCGTGTTGTGTGTATCGTGCTGGTGTTAAATCACGGCTCCTAAGT

>contig00863

GCACTTTTCCAAGTCGTTTTTTTtCAATCGTTTGGGTCGTGAACCTAATATCGGAATTGCACTTTTCTTAcTGAGTCTGGTTTAGTTGCATTACTGAGGATGTATATAACACTTTATCAAGAGTATGAGAACGATGTGCCTGAATCATACACCAGTGATTTGTTGATAATATTCAGAAATTCGTCAATTCCGAAACAGAGGGCTTCGAGAGAAAAAGGTAACACGCTAGAAAAaGCACTCAATAAAATTGGTCGTGAGGACATAGTGAAAAaGTGTATATTCAACGTtGAACTTGTGACAGACGACGTTGAAAAaGCGGTTGCGAGAGT

>contig00864

GCACAGATAAGTCGGTTCAGTCTTGTGCACAG

>contig00865

CCGAAaGTTCTTCGGTGTCTCTTAACAGGAAGTCGAATTCTTTGCCTTTtGTGCTGCTATACGTTGACTGCGTCGCAAAGTTCGAGGCTGAGAAGTTTCCGT

>contig00866

ACCAAGAGATCAATAACAACGTCGGCATACTGGTTGACGAATTGATTGCATTCTTGCGAAATGGTTTTGGGCAAATGGTTGCAAACCCCGTGGATTATATGTTCAACCTCATCTTTCGTCTTCTTGTTGCTCATAGCTTTTTCAATGTATTGCATAACGAATTCGCAAACGACGCACTCTGTGTCTTCTTGA

>contig00867

CACAATTTAATGTAGACACAGACTTCTTGGGGTGTAAGATCAGCGATAAGCATTTCAATAAGTTCTTTGCTGTAACCTTTAACCAGGTCGTAACATTGCCCGTTCAAGCTCTTCGGCAAATGATCGCACAATTtGTCCAATTGGTTGACGATGTTAGCCTCAGTTTTGTCGTTTTTGATGGCGTTATAGATTTGCGTAACCGCAAGTAGACAAAGTGGACAGCTTGGTTTGTCTTCGACTTCGGTTTGTAGCGTCATTTCCGACGGGATTTTTGCCCAGAGATCCATAACCTCTTGTGTCGGACAAACGTGAATCATTGGACATACCGTTGCAGGATCAATTTCTTGTGCGAGAATTGCTACCACAGCATCACCATAAGTATCAATAAATTCGTCACAGGTATTCTTGATCGAAGAAGGAAGCTTCGAGCAAACTTTATCGATGACTTGCTTCACTTCAGCCTCA

>contig00868

ACtCCCtCTTCCTTGTTCGAAACTGCAaGgACTTtATCATCGTTTTtGCAGGTTTTtATTtGTTTAAATAATTCTATCTGCGATACCAAATGCTTCAGACGGGATCCTtGTGGATCAGAAAGCGACTCCATTAATTCGGTCCCAAGCGGTTCATTTAGTTTTAAACCATCCAATAGTTTTCCAGCATGCTCCAATTGAGATATTATACATACAGCAGAATTTAATTCTTCTTCATTCTTAGCTCTCTCCTTAAGTTGACTTAATTCCTTTATTAAATCTTTGATTTCAAAaTTTAaGCGTTCGAATCTCTGTGGTGGAGTTtCTTTTtCACTCCCTCCAAGTAATTCACATTCGCCACATCCAACGCTATAGCCAGTTCTTGACTTGTGTGATATCCGATCCGAAAaGTCAACTCCTTTACTTCCAACATGTTTCCCTTTGAACTTATTGAATTCCTCACTAGCATTGTAGTTGAATAGTTCAACAGAATCGTTTTCATCCTTGGAAAAAACAGGCAATTGCTCGGATTCTGGAAGATCTGTAGTTTCGTAGACGTCTGCTTGATCATATGCAATGCCAGGAAGATCAGCATATTTAGGGTCTGCCATGACTTATAGTGTAATGAAATTGTGTCCTTGGCTGTCACTAGTTCAACAGATGCACTTCTTCCCTTTTATTCCTTCCTACTTCAAACTTTTTTCCTTCTTTCTGAAAaTAATGGTTACTTCTAT

>contig00869

GCTAAATGGCTCAGACTTATCAACAATGATTGGACCAGGAACTGGCGCAGCTTCTTTtGACGCATTTGGTGTTTCAAAGTATCAAAATCGACGCACTATGAGTAgTTCAGATCGAGCCTTAATAAACGCCTTCCGAGAAATCAACGGAATGGCGGATCGCATAAATCTCCCAAAAACGATCGTAGACAGAGCAAATAATCTTTTCAAGCAAGTCCACGACGGGAAAAATTTAAAGGGTCGCGCAAACGACGCCATCGCCTCGGCATGTCTTTATATCGCCTGTCGCCAGGAAGGTGTGCCCCGAACTTTTAAAGAGATTTGCGCTGTGAGCAAGATCAGCAAGAAAGAAATCGGTCGATGTTTCAAGCTGATTTTAAAGGCTCTCGAAACGAGTGTCGAGCTTATCACGACTGGCGACTTTATGTCGAGATTCTGCGCAAATTTAGGTTTACCTAATGTGGT

>contig00870

ACTTAGCtATCGACAGACATTTTTtGATCATCCTCGCTTTCGTTGTCACTACTGGCCtCGCTTTtCtCACTCGATTCACTCTTTCCATTTACTTtATTTtCGTTATCGTTTtCGTTTGCATTCGCTTCCGAAGAGTCATTTTCTGAATTTTCATCGTCACTCTCAGAGGATGAAGTGCTGTCTGACTGACTGTCACTGTCTGAATCTGAAGAACTTCCGGCATTTTTAGCTGCCATCATTTGCTTGAATTGCCTAACTGTGATTTTTCTTGGCATTATTTCTCGGAGAAATTCTAGAGAATCACTGGTCTGCACAACTTCCGCTAAATGCTTGTAGTCTATGTTGTGTCCTTTCTTGGCTTGCGAATGCGCCTCTTCTGTCAGGAAATGAATGAATAATTCCGTAGCTTTCGTTACAAGAAAGAGTCCATCCTGGCCTATCGCTTCAACATAAGGAGAACTTTtCATAATCGTTTTTACTCGCGAAATAGGCAATCTCAATTCCTTCAGTTTTGCGGGCGATGCTTGCGGGGTAGCCATGTTTGTTTGCTT

>contig00871

ACGATTGAAGAATGACGCGGTAAGTATGAATTAAAaaGGAGGtCATACTGTTGTCCTATTTGAATCTCCTGCAAGCATGTCCATAATTCGACTGATGCCTGGCATGTTCAAAAaTGTTCCTAAGATTGGCACTCTCCGTAAAAAGTTTATCGCCaCTGGTAAGAATCCACTAAACAAAATAaCGAAGCCATAGGTTTCGACGATCATGCCGACCAGAGGCCAGCCCAATATAaCAATAATTACGCCTCCTAAAAATGAAGCACTTCCTTTCAATTTGtGTCTCTGGAaGAAAAAGCTTAAAGTCCTCCTAGGTCCAATAACACATCCCAGACCCGAGATAAACAGGAGGTTGCCAATTGCAAGAAGACCTTTGTCAAATAACAGTAATACTCCGAGAAAGAGGAAAGATATCCCAAAGCCAGCAAGTCCGACTCCGATTTTTTGCGTATCTGTTATTTCGAacATATTTACAAGATCGTTTATCGCACGAAATAAGAGTTTTAATTATTATGCGGCGTGGAATACTTCCACGACAG

>contig00872

TtttAAGGACAAGAAGCGAAGAATGAAGAACTTGCATTAGTGTCTGCGCGTATCAAGATAAAATAACTTATTGGCCGTCGAGTTGTAATAATTAGATTCAGgAGGAATTAGAAAGGGCTAAGTAATAAGGCATTAATTGACGAATAAAGCTCAATAATGGCGAAAACCATTTTTCCCGAGCAAGGCTTTCGCATCAACGATTTATTATCTCCAAATGGTG

>contig00873

GTCTAATGATGAAATGCTCAAATTTTTGAATATGGACGATTTCGATAGTGACGATGATGAAGGACCAGCTGTCGA

>contig00874

ATtCcTTTTGGGTGAAGAGAAACCGCCGCCATGGGTCGTATGCACGCACCTGGAAAGGGTATATCCCAGTCAGCATTGCCTTACAGGCGAAGCATAGCAACATGGCTAAAACTGTCGCCAGAAGATTGCAAAGACCTTATATTCAAATTGGCGAAAAAGGGACACACTCCTTCTCAAATTGGTGTAATTCTTCGTGATTCTTATGGAGTTGCGCAGGCGCGTTTCCGCACAGGAAACAAGATCCTCCGAATCCTGAAGAGCCTGGGACTCGCACCCGAAATTCCAGAGGACTTGTATCATTTGATCAAGAAAGCGGTGGCGATTCGTAAGCACTTGGAGAGAAACCGTAAGGACAAGGACAGCAAATTCCGTTTGATTCTGGTAGAATCCAGAATCCACAGACTTGCTCGTTACTACAAACAGAAGAGCGTCCTTCCTCCTAGTTGGAAATACGAGAGCTCGACAGCGAGTGCCCTTGTAGCTTGATTTCTTACGTTATATTTTC

>contig00875

ACACTtGACATATATGAAAGACAAGTAATTTGAGAAAAATGATAGGGAAAGCGGAAGCATGTAAAACACACACACACACACAAACGCACACATGAAGAAGAAGAAGAAGACAAAAAAAAaGAAGCATCCCGTAATCTCTTTTGTATAATAGTTATACAGGGGCACACTGAAATGACGACTAAGCGCGGGGTTTAATTGTATTAACATGTAATTAATAATTATTGTCGTAAAGTAGGATTTTTtCGGTGTAAAAATTTACCTAATTAAGGCTTAGTGCGCGTGTTTTTTtGGATATAAATTTCACTTTAGCTGGCCTAAATTACGATAAGTGACACTTGCATGAATCCAAAAGGAAAGAAAaGCAATGACGAAATTGTAGGTGTGGCTCCAGGTCCTTTAACCCTTTGTCGAGCTAGATTCTAATTATATATCTTGTATTTTATATTTGAATGAAGCTTATTCGAATATTTCGAAGGATTGTGTCAGAAAGGTGAGAAATCTTGAGATGAAGGGTTATAGGTTGAGAGAGATTGATGCAGTTGTTTGCTATGATTTTAATTGAATATGATTTTATATGTGAATTTGTTTGGACTAAGAAGAATCAATAGGACGGGAGAAATAGCCACTTTTTAGTAATTAACATGTCTATGTTTtCTATATGACAGAATAATGCATTATATAAATACAAACCTGGATCATTGTTCCATGAGTGT

>contig00876

TCGAATCTCGACATAATCGGTGCAGTCAACATGACATTTTGCTGCAGAGATTTCTGTCTGTCGTAAcGACCCCcGATCGGATT

>contig00877

TTATGGGTTCTCTCGAATGGGCGaTCGGTTCAATCGGTGGCTTCTGCGTCGGAACATCATTTATAATCGAGCACCAAAGATTG

>contig00878

ATGATGAAGTCTCCAAGGTCTGCTTCGAAGAAGACAAACGAAATCAGCCAAGAAATCGGCAAGGTTTTAGAGGCAATGCAGATAGACTCT

>contig00879

CTCAAATTTTTCTCAAAGTGAAATAGAGCGAATCGTCTCCGATCCGAGCATCGCCGACGATGACGAGGACAACATCCGTATCAAGGAACAAGGAATCCTCCAGCTCGGAGAGCTTTACAAAAAGGAGGGCAAGGCTAAGGAATTAGCTGACCTCATCAAAGCTACTAGACCTTTCCTTAGTCTTATCAGCAAGGCAAAAGCTGCTAAACTGGTCAGATCCTtGGTTGACTTTTTCCT

>contig00880

ACTATCCTTACGATATTCCAGAACAGAAAAGGAATCATAATGAAATTCTTCACGAGGAATTCGATGTGTTAGGCCATGACAGACTTGATTTTTtAGGAACGCGGGAGAGGGTTCCGACTTGGGAAGTTTTTCtAACTTTCTTTGGAATGATCGGTGTAACGTTTGGtATATACATTCTAGTAGATGACTACAAACaTTTCCGACCAGTGGTGGCAAAGCAGTTTCCAAAATCTGGGGAAAAACATTACACCTTTGAATCGGCCAAATAAAAGACCATAAATCCAAGAAGAAACTTTGAAAaTTAATGTGCAGTAATAAATGCTATATAAATAGAGTGCATTTTACTTCACTTCGGTGCATTTTTGCCTCTGCACAGTTGCGCACGATCGTAATCCCGTAGGTGAAATTGTATATACGCAT

>contig00881

GTCGTGTGCCGTGCTAGCTTTCGTTAACACCTTCGGATTtCCACGCGTGTtAAGGATTTAAGAACGaCCAAATTGTCAAAAaGAGGAACAAAGAAACGAACAGTAGGAAAAATGGCACTCTCATTCGTAATGGCAGTGACCAATTTTGACAGCAGAACAATGAATGTAATAAAAAGGCGTCTTCAACTGATTGATTTC

>contig00882

TTTTTAAAtCTGGAAtCACCTATATCTCCAACcTTAAGTGAAGTTCCTAGTGGATATGGCAGCCTCAATTCGCCTGCTATTTCAACGTCTACAAATTCaCCCAAAGAGTCACCAAATGGGTCACAAAAAGGGTCACCAAAAGGATCACAAGAGGGGTCAGcAGAAGAGTCAAAagAGTCTTCAGAAGAAAATCCAGAGGACTCTCCGGAAGACCCTCTTATTTCTGGCACTACTAGTTTAATTtCAaCAATGAACAAaGATATTTTACaGGAAAGTCCACCACTACGTGTAATGCaCGAGCGTAGAGATATTTTAAATTTAGGCGCAAATCTCAGAGAACCAACCTGGCACACTTGGAATCCAAGAAACCATACATATGATGAAGAAAAGGAAGGTTTAAAGAGAAaTTCTGTATATTTCCGTCTCAATTCCAAAGGCGAAGAGGCtCTCATAGAAGATTAtCCAGTCAATCTTTGCACACACACGGGTTGtGAaTTTACGCAGCCCTCACaCTtCCTATTTCCGGTtA

>contig00883

CAGCAAACTCGGCAAaTGTCTCGAGTTTGATGATCCCGAGCTCGAAGAaCCGGCAATTCCGAcG

>contig00884

CACTTGGACATCGCTTATACCTGCGAGGGCGGCCTCTAACAATTTTTGCTCTCTTCCGGATCT

>contig00885

AAGATCGAGTAACCTTCTTGAATCAGTTGAGCCAAATACATGGAGAGGTATGTGTCTGATATTAGTTGAGGACCACTCAGCATGGAATTTAAATTGAACCACTGACTTCCCAATTTCCTAATCGTGAACCAGTGACCCTTGTAGTTACAA

>contig00886

CCAATATTGCACTTGATACCTAATCCATTCCcGAAAACcTGGCATACACAATACGACAATAGAGAAAGTATAGACTTGACAACTACCGAAAATaTCAACAAAaTTCTTCGACTATTTATCGCTTCGTATTTACATATCAAaGTCTAAATTAATACGCGAGTCGAGTATTCAGCCAATACAGAATTCTATCAATGAAAGTGATCATATATATTGTTTAACTGTCGTCATTATAAAGAAAAAaTCTCCATTCTAAAaCTAAACGCTGAAATGAAGAGGACCTTCGGAGATATaTTTtAAAAATtGTTTGCAAGTTTTCTGAGAAAATCTTTAGAGTTTTAAAAATAATAACCATTTTTGGCTGAACAGTGATGAACACTCTAAAAATAATaTCGCTCAAAGATGTGGCATTGAATGTGATTAGCTTTTTTtAATTTGtATACCAGCGAGTGCaTTTAATATGCTTCTTAAAATATAGTGTTCTACTAAAGAAAGAAAAAaGaTTCCATGAGAGAGTAGATTCTGAAGTGTAAGAATATCGACAATACTGAGAGTGCATTATATTTGATAAATTCATGGCAATTTTTGCCAATCTATTTTTATGCGTTATGTGTGAAGTGTGAGACTTAATAGT

>contig00887

ACTCTTTAGATCCCACGACGtCGGGCTTCATGGTTTTCTTGCCTGGTTTCCAGCCAGCTGGACAGACTTCTCCGTGTTTATCGGTATATTGGAACGCCTGGACTAGACGCAGAGTTTCGTCAACGGATCTTCCGACTGGAAGATCGTTGATGGTAACCTGTCTCAGGTTTTGCTTATCATCAATGATGAAGAGACCTCTGAAAGGGATGCCGCTCTCTTCATCCAGAACTCCATAGTCCCGGGCGATCTTCGAACTCTTATCGGCGAGGAGGGGAATGCTCATTTCTCCAAGTCCTCCTTGTTTGCGGGgCGTGTTGACCCATGCCAAGTGACTGAAGTGCGAATCCGTAGAAGCTGCAACGACTTCGCATCCGATATCACGGAATTCCTTCACACGATCAGAGAAGGCAATGATCTCAGTTGGGCAAACGAATGTGAAGTCCAGAGGGTAAAAAAaTAGAACGACGTATTTGCCcTTGTAATCAGtAAGCGATATATCCTTGAATTGGCCGTTAACAACAGCAGTGCCTTTGAAGACTGGTGCTGGTTTTTGAATTGCAGGAACTGGCATTGTGAATTATAGTAACTgt

>contig00888

ACGAAGCTGATCAGTTTCTTCCATCTTCGTGTCATGAGGaGgaGACACAGATCTCCTCCAAAACaGTGTAAACCACTAAAGTGCTGTTAAaGTGATTTtGTGAATAAaTAAAAaaTTGTTTTCAAaGGTGgATTaTTTTGAAAAAGGAATATCTTGTGAGAAACGATTGAGTGAATAGCGTGATCACTGAACaCAGTTCAGGGAACGAAAaGCTTTCGTCGCACCTTCAGCGT

>contig00889

AGGCTCAATCGGCAGgTGG

>contig00890

aCCaTTtAGTCTTGTTTCGGCCAAAATCTCGTTACGCTTAATTAAATCACAATATTGAGATCGGGCCAATTTtGAGTCAGGTAATCTtGTAAAaCGTAGATCTTAGAAGTAaGTGGAaGAGAAAAAaTGGCAATATtCTTtCCATATATGAAATTAAAAAGACGATAAAATTCTTTAAAAAAAAGACCGCCGATCTTGGCAACTTTTCGAATTCGTTTTCACATAACTGTGAATTCAAGCTCCGTAAATGAAGAGGATAATCCATCGAATTTGATTCCTTGCGCGCCGGGTCTTTTTGAGTTGAATATTTTGCCATAATTATATTGCCTATCGAATTTCTCACTGACTGGTTGGTGGAGATCCTCCGTTGTCGTATTGGCTCTTATTTTTGACGAGGTAACCGGCTAAATAGTTGATAGGGTCCGGTGGTCGCTCTTTGGCTAAACTGGAGAGTGCTTGGAGTAATATTGGAACGACAGTTTGGTCCAAATATTGCCGGGTAGGAAGAGACTGGACTTCTACGCGTGATTTTTTCGCTGGTGCTGCTGCTGACATATCAGGATCTTTATCCATCGCTATTGCTTTCTTCAACGTTAATAAAGCAAGACACTATAGGCAGACGTATTAGGTATATAATAAATCAAGATGATACAAATCGTTCATATTGAGGTTATGATCGTAATTAAAATCTTATGAAAAT

>contig00891

ACCGTTgCCGGAAGTGGAGTtGGCAAAGCTTCCCTAGAAGAAGACGACGAAGTGCCCGATCTCGTAGAGAACTTCGACGAGGCCAGTAAAGAGGAGGTGGCATTAAAGAAAGAAGTCGAAGAAATCCTCATTGAGAAACCTGCAGAAGAGAAATCTGAATCATCGGTTGTAAtCACAGAAGAAAAGAAAGACGCACCTCTGGCCAAGGTTGAAGTCTAATGAAGTTATGAGTGTGTATACGGTATGACTTACGATTTTtATtGAAGTTAAACAATATCAATAACAAGCTATTCAAGAGTGACTGCATATAAAGATCAGTGGACCTTATATTTtACCCCAAGCCGCCGAACCCACGctCTTTCCAATTCCCGACTCTCTTTttAATTTTTGTCCcTTTGTCGCGCATTTGTGGACAGTGCCGTTAaTAGATAATGAAAGGTGTTACAAACGCATACTATATAGATTGTAACACTGTTATAAAAGCAGGTTGAATaTGAAAATAATGGCATCCCATA

>contig00892

TAAATTTTTGTTGCGC

>contig00893

aCCTTCCGTGTCTtGGGtCACATGCCgCCATCATGTTCTtGGCGTCAAACATTTGTtGTGTCAATTCCGGAACTGAGAGTGCTCTGTATTGCTGACTTCCACGAGCCGTCAAGGGTGCGAATCCGGACATGAAGAAATGGAgACGGGGgAAGGGAACCATGTTGACGGCGAGTTTTCGCAGATCAGCATTGAGCTGaCCAGGGAaCCTCAAACAGGTCGTGaCTCCGGACATGGTGAGGGATACGAGATGATTAAGGTCGCCGTAGGTGGGTGTGGAAAGTTTAAGTGTGCGAAAGCAAATATCATAGAGAGCTTCGTTGTCGATACAAAAGGTCTCGTCTGTGTTTTCAACGAGCTGGTGAACTGAGAGAGTGGCGTTATAGGGTTCAACGACAGTGTCTGAAaCTTTTGGTGAAGGGACGACTGAGTATGTATTCATGATTCTGTCGGGATACTCTTCACGAACCTTGGAAATGAGCAGGGTGCCCATACCGGATCCGGT

>contig00894

AAGATTTAGTATCAAGAACAAATGT

>contig00895

ACTGATCTCCTCATGAAGGCTGTCAAGGCCGCTTAAAAGCCCcTTtCCTCGCCtCATCCCAATAATTTATCCCCCcGTtCGAAATCATTGTAAATCATCGAAGATCACGTTATCCTAACTCGTAGATCGGCCTTGCGGATGTTAGGCTCATCGAGCATTCTCGGGCTCTTCCGAGACGTTGGAAGATGTCGAGAGCGACTTTATTCGATACTATGGTTTTAGAAAAGAAAAAaGAAACGGTTCACTAGCCAAACTAGAGAAATGGCAAGACcTGgCcTGACCTGGCTGAGGAGCGAGACAAGTAACGGTATCATCAGCACGTGTTTGAAATTATATAGTGAAACTAGAAAAACCACAGCTGCTCAGAGAACGAGTATTCGCAaCAAAAAAAaCAGTAATTAAAGAGATTTtCTTTTCAGCGCTGCTAAACATAGTTTTCAATAGAAAGAGAGAAATAGAGAAAGaGAAAAAAaGGAAGCATTGAACaGACATaGAAAaGTAAAAGAAAAAaCTGTCgAACAGTtGTGTAATT

>contig00896

ACCAACTTTTATAATGATCGATCTTTtGGCTTTAATGAGACTGCGCGAAAAGAAGTTGAGTAAATTTTCGGAAAATTATAAAATTTTAGTTATTAGACTTTTAAAAGGTAGTTTTGTTTCGGAGAAACTTTAAAAGTTGGTTATTATATTTA

>contig00897

AACAAGACCAAAGATATTCCAAGACCATCTGGGATTTAAAtCACCGCGGCGATCACGTGGCGGACCATATTATCGACCGTGGTGACTCCGGCTCTCTCATTGGCCGAAGACCCACGTGACTGCACCTGACGTCATATCGTCTGCTGCTTTCCGTCTG

>contig00898

AGtAGTTGCTATTTTTACGCTTTCTCCTCCACTCTGAGGGTTCTTCGG

>contig00899

CTTCTATTCACTCGCAGGACTCCTaCACCCAGCGTGT

>contig00900

aCGTCATCCAAAAAAATATCACCGAACTAGCAGACTGCAAaTCCcATGGACCCCcACCAAGTAAAAAGAATTGGGTGAGctGGGGATATGAtAAGACCGACAAGAGAATTGATCGTCATTACATACATGAAACTATGTTCATATCCGTTACACTCTGTCtAGTTTtCGGTTTTtATtACATGAtGTATTTACCAAACACGaGAATGCAAGATTGGGCGCAACGAGAAGCTTACCTCGAACTTCGACGCAGaGAAGAACTtGGTCTtCcTCCAATCGACAAaGaCTATTtCGATCCTGCGAAcTTTtACCTTCCCACCGACGAAGAATTGGGAGACACTCCGATCATTATTTAAACATAAACTCACTGCGCATTGACTGAAAGTGGTAGTGTTTTAAaTAGGGGCCCGAACTACACTATATTGCTGTTCTAAACAAGTTCTTCATAGTTTCGATCAGGTTCCTGGTTTATTACATTtAGTTATATAAATATTACAATATCGAGGCGAATAAATTATGTGATTCAAGAAATACAGTATAGCTTTCCGGCTAAGATTTtGTCAGATTTCTTTttA

>contig00901

ACCTCTtcTAAACtGTCAATTGTAGTTGGTGCAAAAtCAGCTGGCTTCTGAGTAGCTGTTTCTGGAGtAGTTGGTTCCAGAGTAGTTGGTTTCTGAGTAATtGGTTCCAGAGTAATTGGTTTCTGAGTAGTTGgttccagagtagttgATTtCTGAGTAGTTGGTTCCGGAGTGCTTGGTTCAGAAGTAGATGATTCGACAGTAGTTTCTTCcATTGTTCTGGAAAACAGAAAACTACTGAAATAATTAACCATCTCAGGAATGTTCCTCTTCTGTCTGATGTTAAATtGGTTAAaTCTTTGAGCAAGAAGCGGTCGCTCAAATTTCCCTTTTCCTGaaaaTAAAGGATGTTTCGATTGGCGTTGTTGAAGCCTACTtCTAAtCCTTTCTCTAATAGCACGATATAATGCACGTTGGAATTTTATAAaTAAAGCTGGCAGTTTAGCCACATTTTCTGGTTCCAGAAATACAGCTACTAATTCCTGATCGTCCATAAAGCTGTCTATTAATTGAATAATGGCACTTCCTGATGATTCTTCTTCCAGATTATTGTTTCCAGAATTTTtATTTGTTTTATCATCCGGAAAATTGcTGTTATGGTTAAAATTAAATGTAGACACAACACTTCTTAGCACTATGAAGCTGATGAATAAAGTTAAGAACTTCTTCATTTTtGAGTATTTGGCTTGTCTGAAATTTGAAATCCTTTGTCGATATCGCTATTCGAACAGTCACTTTGGCTTCTCAATGGACACT

>contig00902

AGATTCGACCAATAGGTCG

>contig00903

AAGGCGAGCCTCTTCTTTtCTCCTACGTCGTTGTCATCAACATCCAAATCGT

>contig00904

AACTTTGCATTGAATTCGAGTCGGAAGAACCAATTCCCATTGGaCTGTAAGCATCTGCATTTGACGAGTAACTGCTTCCGGATCCGTGGATTAGAGAAAGCCAGCTGTGGCCTCCGTCGGGTGAAGTTTTTcTCTTTGAAGTTACTTTGCTCTCTCTCGTCGGCATGTAAGAAGGGATCGGTATGTGCCGTTTTTTCAGTTCTGCAATTGGCGTTGGATTGTAGACGCATTTTGCTATAGAGGACGGAACACCGATGTGTTTTCCGATGGTGGTGGGAAGAGGAGGATCTGGAaCTCTGGATGAAGAAACTAGAGATCCGGAGGCGAGAGTTGGcTCTAGTtcTTtCACGACCTTcGACACTATGTTATCGGAATATGCAGAGGCCTCCTGGTCAGCGAGGGCCTTCTTCACAGCTTCTGTAACTAGTTTCTCCAGGaCTTCAGGGTTTGGAGCAgCAACGgCAGCGACGGCAGTGGCGGTGTCTT

>contig00905

TGATCAACGAACCGAATACCGATCCGATTCCAGCACCTGATCCTGCAACACCAACGGTGGCAGCACCTGCCCCGATGAATTTGGCGGCAGAATCAATGTCGCGGCTGATGGTGGAGGTTTGGAAATTGCGCACGATTGGCGACAAGGCGACGGTGGATGGAACCAGCCTTTGGTTTtCCTGGAGAGAAGACTGGCTTTGGCCGAGTGCTGCACTGCTCAGTGGCCGGAGGTAGCTCTTGGTTCCAGCGACCAAGGTGGACCTGGCGACTGGTGCGATAAATCGTGTGCAGT

>contig00906

CATAATGACGGTTGTAGTAGGTCGTTCACTTCAAGAACCAAAGAGCAGTGTCGACGGGgAG

>contig00907

ACGCCAATCAGAGAGAGGCTTATCCATCTCCTTGCgTTAAGGCCgTAtAAGAAACCTGAAATCTATGAACGTATA

>contig00908

TCGGCCcTCCAAGTCTTGGCCTTAAGCGAAAGTTTTGCAGCTCCAAGACCCAAAGGAACATGCTTAGACGAACGTAAATTTCACAGACGCACGATC

>contig00909

AAATCCAACGTCGCTGATCTGCTCGTTcGGTAATCTATCGTCGTTTTtCTCTAAGGTaTCCCCGATCGCTTGATGATCTCGTGGTGCCGGAAGGGGTGGTGGTGGATTAtCGTCTTTGCTGGGGTGACTTACCTGTTGCTTTtGTTGAGATGGACGAGCAACCTGCTCATTtGCACTATGCGCTCTCGACTTGTCCTGgTTTGTTTCTGGTTCCCGCTGCTGAGAAGCTGCACGATTTAGCCCGTAGTCGGGAGAGGTGATGGCTTTCTTGgCCAGTTCGAGTTCCcTCTTGAGTTGAAaGACTTCCTTTTCTAAaTGGAGCATAGAGctCTTTGAACTGACTGCGTCCTTGACTAAa

>contig00910

ACGCCGCAAAgCGATTCAGAAAAGCTCaGTGTCCGATCGTCGAGCGCTTGACGAATTCCCTGATGATGCACGGACGAAACAACGGCAAGAAACTCATGGCCGTCaGGATCGTCAAACACGCATTTGAAATTATCCACCTCCTCACcGGAGAAAACCcTCTCCAGGTTTtGGTGACGGCGATAATCAATTCGGGACCTCGGGAAGACTCGACGAGAATCGGTCGTGCCGGAACCGTTCGCCGACAAGCGGTGGACGTCTCGCCACTGCGTCGTGTCAACCAaGCTATTTGGCTTTTGtGCACTGGAGCCCGAGAGGCCGCTTTCAGAAaCaTCAAGACAATCGCCGAGTGTCTCGCCGATGAGCTCATCAaCGCTGCCAAaGGATCGTCCAACTCTTATGCGATcAAGAAGAaGGACGAGCTTGAGCGTGTTGCCAAaT

>contig00911

ACCTTAAGTTCGTCCAGCACATTCAAAACACCGTGCAAAGGCTCAACAAGCAAACATCGAACGGTAAGACTTGGAGCCCACACAGAGTGGAGTTGGCCCTTTGGACACACTACGTAGCTTGTGAACTAAAACCTGAGTTACTCGATGAAATGCCGATGACGGCAGAGAACGGAAATTCTCACCCCGCCAGCAATGGCGAAGCGACGGAGCCGTCGGACGACAGTAATCAAGAACCCGCCGTTGTCGTCTCGAATGgCAAGGAGCCAGAGCCAATTCAAATTCACCCAGCCGTCATCGACGaGAACAGCACCACCAACTCGTtCACAGAAGACTCGATGGACAAGGCACCCACTCCCGTCGCCGTCAACGTGCCCAGCGAGGACGACgACAGCAATATTACTCCTCGTCCCACTGACAGCGAAGACACCGAAGACTCGCAGGACGCTCAGGAGCCACCGACCAAAAAGaGCAAGAAGTGACCCTGCAGCCGATCCCCAATAAACCCTGTAAGTCACCTCGTA

>contig00912

CGGCCGCCAATCTCGCGATGATCGCTGCCAGACGCTTCTAGCTCGAAATCGCGAAATATAACTTAATAAAATCGTCTGTCGAGCCTTCCTTGCAATTTCCTTTCTCTCCTTTTCTCATCTCATGAAGACACTGGCCGGGTCCCTCCCCTCCAGGACCACGAGAGACACCTACTGTCTTTCGTTTGCATCGAATTTTAATACAAAATAATTGGAGGCAGTCTTTCCAGGATTCG

>contig00913

TGCTTCAGACAGAGAATATTGCCTGTAACGTAATGTAGATAGACTTtCCCTTAATAAGGGCACATGAATTTCACGCATTGATTTCAGCTCAATTGACTTGATACAGTTCGTAGATTGCAGAAAGATATCGAAATGTAGTTAAGTGTTTCTCGTTGCGAATGAATGTCGACTATTGAATAATACTTACAATATTAAATCCTTTCCCcTTGTCGACTAATGTGAAATAAACACCAAATTTCAACCAAACAATACAAAAaTAAAAAatATAAAAAATAAAAAATTaGATCTGTGTAAAaGAAATATAAaGATCGAAATGACAACAAAAAaTTCCAACGGATTTTACAGATGCTCATGTAAATCGTGTTTTCCTAGAATTGTAATTAATATTAATGAAAAAT

>contig00914

CTcAAACTTtCTAAGGAATACGACCAACAGAAACaGGAATAATATACATCACCATCCCTTCGGAGTAAGAACATTTCCCAACGGAACTCGAATTTTGtCTCCTTTAtAATTTATATTTTGAGAACGTTCTCAGCCATGTTTTCAAATGTAAATAATTTTCAACTTTCCGTTGGTGCATAATTCTTCCAATTTTTAATCTTACGGAAATTCCGCAAAaTATAAAATTCCGGCACTATTTACGAGCTTTCTTGACAGTTTGATACAAAGTATTTTTTAAACACTTTCGACCGTTTATGACAAATGTGAAAGCTTCGACGCATTGGTAGGACTTGAATCATTTCAGCACCGTCGCATTTAAATATTATAAaTAGTTtGTCCATTTTTTtACTGCCGTTCTCGAGTGTGAATTTATTtGTAGAAAGAAaCAAATTTATATTAGAAGAAaTATAATGACTAAGTAAGGTATTTTAATAaTTAGATCAAATGTTTTTGATTAGAAACAAAAGTTATTTTCAAACTTGTAATAAGTTTAGATTTTTAATTTCTATTTCATTTGATATGTTTATTTAAAACTTAAAAATACATTGCACGAGTAGAAGCCATGGAAGGCGTTTGTGTTTGATATACAAATTAAGTTTTGATCCCGCTCCGGAAATTTTGTAGAAAAACACTGTTTTTCCATTGTGAAAATCCAAATTTATTGCAGTATCCGAAGCGGGATTTTATGAAGGGAATATTTAATAAGACTTCAGT

>contig00915

ACtAGCATTCGACCCACAAACAAAAATGCTCATCCTGAAATGCCCGTCGTCGAGTGGAAGACCGACGCTAAACGATGTGCACATAGTAAATTTATCGAAGGTGTCGGATGtgCAGGTGAAACGAGAAGTGAGTCCGACGATGAGTGAACCACCACAGAGTTTGAATTTAaCTAGGCTAAATACGCGAGTGCGTAATCAGATTGAGGAGAAGAAGAGGATGGTAAaGGCTCTGCAGGCCGGAGTTTCGCCCGATGGTCAGAAGCTCTACATAGCAATTGCAAAAaCCATTAACGATATTACGTGGAACGGACAGAACATTgTtGTTTTCAACACCGTCACCaTCATACCGCCATACAAaGTCGACAATGTGCATAGCAAAGTTGACGATCAAGCCTACATCCACGTCAAAAAaGTaGTCGAAAAACaTATGAAGGATCaGGCGGCGGCGGAGACACAACAACCGGAaCAAcAGGAACAACAaCAGgCACAAAAaGG

>contig00916

ACAAACACTTTTATGCAAAAaTTAAAATgCGGTTTTAAAACTGTGCATACTCTTTCTAGCTCCAGtgTTCGGCActAGGTGGCGAAaTGGTAGACCACCATTCCCAGTGGCTTGGAGaTAAAGATTTGTGTAACAGCTTTTAATAAATAATCGAGATTCCGTTCGTAAaGtATTTTtAGTAGaGGGGAAAAAAaTTAACGAACTGCGATAGTaTTTATTTATATTATTATATTTtGAAaGAAGAGAGAAAAACAAATTGAGAGTGTTTtCTTTCGGTCATGTTTAAGTTTCTTTTTTAGTGTTTCTCGATGTTATTTTAAGtCTtATCACAAtGCAATCCGCTGTTGAGAATAATTAGGGATTAAGAGAGATATGCTGTAAATtCACAGAAATTTTAGTTATtAtAGAAGGATAACACAGCAGAGGAGCGAGGCAATG

>contig00917

ACTTAGCCATACTTGTTGTGTAGAACCAATAAAAGCAACATCGAACCAAAACGCAAGGCCGTGGCAAGTGCCACTTTCTAGTATGTGAAAATCGACGTCTATTTCTATTTTATGTAAATCAGTCTCATCGGCTGTCTGGAAATCTACAATGTGTCGAACAGATTTAGCCATGCAAATGCGAATGTCGAAGGTATCTACAATAGGCTGTCTAAAATATTCTTTTATCGCGTTGTTCCTCATCGCGGACAAGTCAACTCCGTGAAAaCACGTTTGACACCAAAAGTTGGCTTTGTTAAACTGCTCCATGTAGAGACTGTCATCCGAAAATGGAGCAATGTGAAGATCTCCTCTCGaGGgAAACATTCTTCCTCCTGGCGCAAGCCACTTCTTCGCGTGCAGGTATGTTTCGAGCATACGTTCGTTATACAACATGTAACCCAtGGGTTCACTAACTATGCAGTCCACTCGTTCAGGCAGTTCAATTTCTtCAATTTTtCCAGCTATCACTATCACCTTATCCGACAAATTATTTGCTGCTACTAATAATTCTGCGTGATTTGCCATGTTACTCGCCTCGACGGCGTAAAcTTtCCTTGCACCAGCCTGCACCGCAAAGAAGGATAAAATTCCTGAGCCCGCACCTACATCTAATACTACTTTGTCTTTAAAATCTGATATATTTCCCAGTATCGCTCGTTGGTAGGTGCTGGTCCTTATGTAATCTTGCATCATaTTTTGCTGTTgggAAAGGT

>contig00918

GCcTgAGGgCTCGAACCCTCTTGTATGGTGGACTGCACGCCATAGACATTTtGGCAGGATATCTGTCGTCCCTGTGCGAAACTCGAACCCCGCTTGGAGCTGTCACGGATATGGCCGGAGTCTTGTAGTTGTTATTCATGGGATGAGGCTGGTTAGTTTCACTATCACTGTAGTCCATTGAATTGTTGAAGGAGAGCTTTCTTGGTTGTGGCAAAGGGgAAACTCCAAAATCTTCCGCAGAAGAGTCAAAAACATCATCTGTATCATAGGCCGATCCGGAGCAGTTCGTTACACAGCTAATATTCAGTTCCTCGTCCTCGATATCGCAAGCGCTGAAATGCAACTTTTGCGTATCTTCCATGTTTTCAAAGTGTCACCATAAGTTGGCGATGTATACTGGCCATTCATGCAGAACAAAATTTTCACGAAATTAATCACTGACCGTTAAACATTTACGACTTTCGTAGAAAACACTTCGTTCGAATcAaCCATGCACGCTAC

>contig00919

ACCGCGTATAGGAAAATACATCGgCGAATTTATCAATCTTCTATACAAAAaTGGAATGAAACCTGAACAGACAACTCTTATCGGTCATTCTTTtGGAGCTCATATTatGGGAATtGCTGGTCAATCTGTAAATAAAAAaGTGAACTATACTATCGGTTTGGATCCAGCTTGGCCTTTGTTTTTtAtGTTACTATAAaTGATCGTCTTTCACCTGACGATGCTCTTTATGTGGAAGTCATACACaCAAACGCTGGAGTAGCGGGTTATCCATTCAACTTGGGCCGTTACGATTTTTATCCTAATGGAGGCTCGTTACAAGCAGGTTGTGGGgCAAATATTATTTCTGCTTGCTCCCATGGACGAtCTTATAGATACTTAGCAGAACAAATATCtAaTCCTGGTAGAGACTTCTGCTCATTGAAATGCGCATCATATTCAGATTTtAATAATAAAAGGTG

>contig00920

aCCAGCATCAATTCCTCGAAAAACGACAGAAAGACCGCCAACAGGACCAGGAAGTGAATTAtCATACCCACTGCGaCGAAAAaGCATCAACACAGCCACTGTTCTACCTTCGAAGGCGATTCCTCCAAATTCACCTGTGCCAAAAAGTAGATCGTCTCTCTCGGCCAATAACACAACTCTGGGTGCTAAAGTTGCCCTTTCTGCAAGAAGAGGAATCTCTAATAATGTAACAAATAGTCCATCGCTTTCTAAAAGAAATACCTTTGGCCCCAACACTTCTCTCAGGGgACCTACGCACCCTAGCACCGCGAAACCAAGTTTGCAGACTTCGACTGGAACACCAGGGTCGACCAAGTTCGGATTTTCTAGAAAaTAAATAAGCATACAATTGGCTCGAACAGTTTACAAAATAAAAGTATTAGAAAATAAATTAATGTTAGaCTGAGGGCAAAAAAAAaCTCAGGGAGGAATGAATAGGAATATATTTTAATTaTTTGGAGATTTAGGCGAGTATTTTTtCAAATAATCGTGATAGTCTAATCGTGCATGAACGTTTTTGTGATGAAATTTCAGTGATTGTTTGTAAAaGTCTAGAGTCATTGC

>contig00921

ACGATTTATCGTAGAACTCTAGAGAGCCGCAACAGAGGACGTCCTCGCCATCTTTCACGGCAGGCAGAGACAATTTCGCCAATCGAGGAAAGTCCATTTCTTCAATGGTGACCCAATCCGGACGCACGGTGACTGAAGCGTCGCGGTTTTTGATTTGAGGTTGGTTTCTATTGTTGCGAAAACCACCCTGTTGTCGACCCCAACGTTTCGACTGGCCCTTGCGTtCTCGTTCACGGAGTTTCAGCTTGCCCAAGGCTTGCATCTGGTtCATTCCTCCGCGTTGACCTCCACGACCTCGCATGTTACGCTGGTTCCTGCCGAATCGCCCACGCTGGTATGGTGGCTTCTGGGCTCTGGTGTTGTCCACCAGATGGAAAGTCGTTTCATCTTCATCGTGGTAGTAGGCGTATTGACTTCCCGAACCAAATTGCGATGCATATTTGTTCGTGAATTTCTTATCCTGGTAAGCCGATCCGGTCCAATCTGCGATCTTGCCTAGCCTGTCTCCCTTGGAAAATGGCTGGTATGGCATGTCTTTAAATTGATCCGGCAATTCACAGGGACCCCAGCCATCCGGATTGTCTTGAATTCCTGGTGCCACAAAGTGCAATTTATCATCCTTAACGTCCGCATCTTCGTGTATCGGGGTTTCCTCAGTTGCAATCATTTAAAAAGATTTGAAAGGACACTTCGTGATCCTCGATGGCGTCCAGGTCCAGTCCCACAGAAAT

>contig00922

ACTATATTTCATTGAATAATTTCCAAATAACTTCTTCTAAAAGAAGACTTTAATCTGCTGGTATATAATTGTTTAGAAACAAAAACAATACTATGACATGACTCATTACAAATACCAGTCTCGGGATACAAGATAATAAAAAGCAGAGGAAAATaCTTCTAAAAATTTCAACAATATAATTAAATCATAAAGGTAGAAAAaCACCAAATTAAaTtGACTAGTTAAAAATCACAGATTTTAAtCTAAAGCACTTTAGCGGCTACAATTTTCATA

>contig00923

TTTTATAGTTTATTCGTATGGTAAATGAGATTCAACAGTTTAAG

>contig00924

CGTCTTTCCGGGTTCTAATAAGAAATCAATTATTCATTTAAAAaaTAATGGAATTTTTCATGTTGAAAACTTTCTTTTATTTGTTTAAAAACAGTTTTTATAGAATGAGAATTTATTCTATTTTAATATGTAAAAAGTAAATGGTAAGAGATTACTCGATATTTATAATCTGACAAATATGAGAATTGCTAAATAAAAACGATTTTTGATTGTGTAAATTATAATAAGT

>contig00925

ACTGAGCAATGTTGCTGTGATTATTTGTCTACCGCTGAACTGTTTCGTTGGAAACGATTCTACATCTATGCTGAATCTGTCTACAACATTGACGTTGGACGCAACAGCAGATAAATGTCGAATTACAaTATCGACACAACATTTTGATTTTATAGTtCCCTCCGAATCCACGACaGTGTATTTGTCTCGGCGTGCCGATTTTTCAAGGCATACTTGAAATTTTAGACGAAAATCGTAGGGATTGTAGATAGTCAAAATTtGTTTATGAGAGGATAcATCGTCGACGTAGAATTTGATACTGGATGGTAAGGTGAAAACTGGCAGtCCTTTtAACTTAGGCATCATGCAGTAAAATGCATTATTCCTATAGGATCAGAGCCCGATGAACTGTCGATTTAACAATAGCACTTTTATCACAACGAACACGAAGACAAACTTCATTAGCACAGAATAAATAGTCCCGATGTTTATtAAAAaGCTG

>contig00926

GAAGAAAAAGAAAAGTATtcGATTAAAGATTTTCTGTGAAAAACATACGCTAAAAAGTGTGAAAAATTGTTGTTTTTTTCGGGATTAAAGTAAAAAATTCAAAATGGGGAAGGGTTTTAATAATTACATGTGTAAAAAGTTTTTCCATCCAGCCTCGAGGGACAATTTGAAGCGAGTATGGATGGCAGAACAGCAGGCTGATGCTTATAAGAAGAAACAGGAGGAACTTCGAGTTCAATACGAAAAGGAACAGGATCTTCATAATAACAAGGCACTGCTGAGTAAAGAAAGCAAAGACAAACTCA

>contig00927

ACTgTCtGCtAAAGGCAAGAAAGTCACGTATCAGACAAAGGTTTTGGAAGTTAATGTAGATAAAGGAATGAAAGATTCGCAAAGAaTATTATTTAGAGGTGAAAGTGAACAAATGCCTGACACACAACCAGGAGATTTGGTCATTGTTTTGCAGCAGAAGGCTCATGAtGTTTTtGACCGATCCGGAGACGATCTTTACTTGACAAAAACTATTGGTTTAGCCGAAGCTCTTTGCGGAAtAAATATAATTGTTAaGCACCTGGACGgAAGGGAaTTAAACATCACGAACCCTCCTGGTCGTATCATAAAaCCCGGAGACTGCAAGGGAATTGTTGGTGAAGGTATGCCTTTCTACAAAAaCCCCTTCGAAAAGGGAAACCTCTACGTTAAATTCGATATTACCTTCCCTGAAGACAACTTTGCTAACGAGAAAATTCTAGAGACCCTTGtCTCTATCTTTCCACcGAAAGCACCGgTgATAGTTCCCCGCGACGCTGAGGAGGTCGAACTAGAAGAATATGATCCTAATTaCAAaGACACGTCGAATGCATCTCGTTCTAGTGAGGCTTATGCCTCAGATGACGAAAGCAGCATGCACGGTTCCGGAGTTCAGTGCGCACATCAGTAACATTATTCATTGCTTTCTAGCTCTAGAACAATAAAGTAAACAACTTGCTCCTTCGTAAGTAAACCAATCATCCGAAAAaTCTGAAGAAGAAAAAaTCAGTATGCTAtGCATACTGGAC

>contig00928

ACTGTTTACTGTCTTTAGGAGTGGGTTTGATTGTTTTTGAATCTGGTTGCCAGTTTGCCGGGCAAACTTCTCCGTTCTTTTCAAAGAACTGAAAAGCTTTAATCAGTCTCAATGTTTCATCAACGCTCCTTCCCACAGGAAGATCATTAACACTCAGCTGTCGTAAAATTCCCTCCTTATCGATGATGAAAAGCCCTCGGAGAGCAATTCCAGCATCTTCGATAAGAACTTTGtAATCTGTCGATATTTTTtAttGAAGTCACTCAAGAGtGGATATCCTAAATCTCCTCcAAGTCCGCCCTGCTTTTTCGGAGtGTTGATCCATGCCAAGTGACTGAAGTGCGAATCGGTTGAAACTCCAATGAGCTGAGTGTTCAGGGATTTGAAATCGTTtAACTTTtCCGCGAAGGCTATAAGTTCCGTTGGGCAGACAAAaGTAAAaTCTAGAGGGTAGAAGAAGAGGACTACATATTtCCCTTGTAATCACTGAGCTTTATAGTTTTGAAGTCACCGTTAAC

>contig00929

ACTTCAGtCTCGTATTGCAACTCGAGTCTTTGCAGAAaTCTGCGCATACGATTCATGCTCATTGTTCTTTTTGTGACATCTATTTGAAGTCCaGCCTCCAACAATTTGCTGAGCAGTTGAGGAAAATTGTAGAGGTCTATCATAACCCCTCGTTCCCAGTGGACAtACTGCCTCTCCACTtCCGGTTTtGATAGTCCACTCCATGGCAAaTGCATTGTAGTCATTTCCCAAATCAGTAAAGAAAGTCCGTAAACATCGGTGGCTTCAGTTGGAGAGCGACCACAAAAAaTTTCGGGAGCTTGCCATCTAAATATTTCTGCTCTGAGACGAGCCTCAAATTCTTGTTTAGATTTGGGCTGATCTTTTtGGgTGGCTAGTTCCCATCCTCCCAACTTGATGACTCCTGATGCCAGAAAAACaGAGTGAGAACTAATTGCTCCGTGGATGAATCCGCGCATGTGAGcGAATCCTAGGGCCTCGGCGAGTTTtCtCGCGCATCTCGAGACTTCCTGGACGGAAATACGTTCGCCCTTCTCGTGGATATAACTGTaGAGAGTGCAATTTAGAAACTCGAAAaTTGATACGATACCGTGATCTTtCATCTGGGAAGTTGCGATCAGGAGAACGATGTTtGGATGACGGATTTCCGTGAGAACTGCGAGATCAGCTTTGATCgCATTTtCGCATTCGGGATGcGtGTGTTTTTtGACGGAAaCTTTAGtGTTGTCCCATTCGGCATAGaT

>contig00930

ATCAAaGTCATCATCCTCCTtATCatCATCACTCGGTGCGCTAAAGTCACTG

>contig00931

ACGTTACATCCAGCCTTTTTGATCTGCTTCACTATGTTCAGAATGTAGGCCCTTTCTTCCTTCAGGACACGGTCCATTGCAGAGTAGTCGGAGACAACCACGTTGTGATCCATATCAGTTTTTGGAGGTGATAAGCAGAACTGGATGAGACCAATTTTAGCTTTCTCGATACGTCTGGGTCCATTGACGTTGCAAGATTTCTGCGTGAAGACAAGGCCGTCTATCAATTCCGTGTCTTCCACAGTGCCACCAAGTTTCTTTATTACTTTGATATCCCTCAAGTCGACAGCGTTTTCCTTGCCAGGTTCAACAACTCTTAGAACTGCCTCCACTGCCAAAGGAGCGAGGAtGCTTGATTGTTGAGAAACtACTTTTGAATTCAAAGAAGTAGAAGCTGTTTCGATCAGAGCTGCTTTATCGGACATATtGACGGCTACTCGCAGGTCATTTAAAA

>contig00932

GCAATCTGGTAAGATTCGCTAATCGAAGT

>contig00933

AGAGCCGTCGTTCGTGTGACGCGGTCACGGAGTCAGTGAACGTGGCCTGTCCGACTGAGTCGGTTCAGAgTAGACCGGAGCATAAATAAGGGTCCCTACCCCAATTtAATTACGGTAAACGCGAACATAAAACTAGTCAAAATTGTGATACcATTTTTTCTAAATCGATCACTTAAAGTGCTACCAACAGTCAGCGCCAAAGTGATCACTaTCAAAAACTAGTGACGTTTGAGGAAAAACTGTGCTATTGTGCGAGGTGATCACAAAAATTTCAAAGGTGAAGAATCATGATTAAACGCTGAAAATGTAAACCCTACGATCAGGAGTGTTTGTGATTTtCGTTTTCTCGTCTTTTCCAGAAACTTTTATTTtAATCTAACTAAATCCACTGGACAAATTTGAAGTGATCGGACCTTTtGTTTACATTACTTTCTTGTTTTGATTCCACGTGCATGTAATTCAGCAAAAATAATGATGAAGTTCGGTTCCTTAAATTTtCTGtATTTTCCTTAcGTGAATCAACCGTGATAAATGTGAAATCTGCGACTTTGTGAAAAAAaTTACTGAATAAATCAGTAAGATGtcAAGCAaGACAaTATGAGTGAGATAGCGAGCGAAGAGCAGCATCATCAACAAGAATTTGTTGGTGATGGCTCGAATTCGGAGCATAATTGCAAGAATCACAGTGTTTCCTTTGAAAGAGAGAAGAGCCGCGAGGCGCACCTTCAGT

>contig00934

ACTGTGGAGGGTGCGAAAGGCTCCACTTGCAAAGTCATTAGAAATGCTAAGATCTACTGAAGGATCaTAGTCGTTTACATATCCTTTAGTTTTATAAATAAGTTTGTTCTTGTAACAACTCTCGCGACCTAAAaTTAtAGACAGCCACTCATTATATAACATATTTTGATAAAAGGCAATAAGTAATCTTCTGGCTTCTTGGAAAATGATTTCATCACACCAATGTGAATTTAGTTTAGCCAAGAAGTTAGCAATTCGATTGTGTTCTCTAAGCAGAATAGTCTGCAGAACTAcTAaTtCcGgaTCTtGATTtATTCTtCCGTCACCAGCtCTATAACAAGTGTTTGCCGATCCATTGCAAAGAAAATATTGCTCTTTtACGATTGGTGGCCAGTCTTGATTGTTTCGTCTTTCGACAGCTAATCGACCACCTTTATGTTCTCGTAAATTTTCACTGATTTCATAAGTGGATCCATAAAGTGCGGACATATCTGCCGCACTTGTCACAGTTGACAGCGGTGTCACAGCTTTTCCTCGTGTAGAACAGCCTCTGTCAACATCAGCAGCCGCACGTAAGTTTATGAAAAAACACTCAATAtCACTGTTCTTGTAACCTGGATCATCTTTGGGTATAAGAAACGGATAGCACAAAGGTTTTTTTAAATAATCTTTCTCTAGCTTCCCTTGCAATGAACAGCACTGAGTATCCGAtGTGTCATtAAGGATATCTATGGAtGaTGTATcA

>contig00935

ACCGATCACAGATTCCACGGACGAAGATGAAATTGAGAAGCAGCtGTCAAAGATGAATCTGGAGGAATTAGTAATGAAGGCTGGAAGTTCAGACCCTGTGGAACAGTTACAAGCCGTGCAAGCTGCACGAAAGTTACTCTCCTCGGACCGTAATCCACCAATCGATCCATTAATAGAAAGTGGTATTTTACCAATATTAGTTCACTGTTTGGAACAGCATGACAGCCCAtCTTTGcAGTTCGAAGCAGCTTGGGCTCTGACGAACATTGCAAGTGGAACATCGGCTCAAACACAAGCAGTTGTCGCGGCTGGTGCGGTCCCTCTCTTTttACAGCTTTtGCTTTCAAGCCAACAGAATGTATGTGAGcAGGCTGTTTGGGCTTTAGGAAATATTATTGGCGATGGCCCaGGACCAAGAGATTACGTtATCAGTCTTGGTGTTGTTCAACCATTATTAACGTTTATCAAACCAGACATACCTCTTAGTTTCTTTCGCAATGTGACATGGgTCATTGTTAATTTATGTAGGAATAAGGATCCACCACCTCCGGTTCAAACTATCAAGGATATTTTACCGGCACTCAATATGCTGATCCACCACACAGACATTAACATTCTGGTTGACACGGTATGGGCACTGAGTTACTTGACTGACGGTGGTAACGATCAAATTCAAATGGTAATTGACAGTGGCGTTGTTCCTCGACTAATTCCGCTTCTCTCGCACAAGGAAGTCAAGGT

>contig00936

CGTGACTGAGGAAACATGCACGGATGCAAAGGTTTCTGAGGCAGCATGAATGTCGCGATATC

>contig00937

CAGCAACATCGAGAGCTTTTCCACCCAGAAGTCCCATGTTTGCGTGCGTGTCCGAACTTCCTTTAATACTGTTCCCAGCAATAATAACCCTGACGATGGAAGCATCTTCCTTTtGAGTAGTTTCATCGCCAAtCATTCCACAAAGCCATTCTACCAATAAATTAAGTTCCAAATTTTCTGGT

>contig00938

ACCTTTTtCTGAGTGGTGTCGTAAATGATGAAGGACTTGCTGTTTATTTGGGTGTAAATTCTGCTGACGAACGCATCGATCTTTATAGCAAAAACCTAGATTCAATTATCGGCTTTATTGGTGCATACCACGATGAAGCTTTAAACTTAACAAAATTTACATCAGCTGTGAAGAGTTTTTATTTtAAGAATACCCTGGTTTCCACTGACAAAACTTTGTTTACGGATGGTGTCATAAAATTTACTGGTGATGCGTTTTtCG

>contig00939

GGAGAACGATGtCCATTGagCGAGGAATACATCCTCGAGGAAGTTCTGCctGCGTTTCCTGGATTCgcACTTTTTGAAAATCGACAAATATCGAAGCGTCCGTATCAAGCATGAAACGGCGACGATTGTTACAAACCGGATTTTGACAAATTGTAGGATTTGTAAATTTAAACTGCTGCTCAACATTTTTGATAACTGCGTTGCAGTCTAAGCAAACGAAAGTGCCACTGATCAATTCTGGATGAACTGGAtGAGTTCGCACAACCTGG

>contig00940

ACTtGATCGCTACCAACAACAAAGTGGATGTTTACTCTGTAGAAACAGCTGGAGTCGATGAGGAAATTCAACTTGACTCAAAGATTATATGCGTGGAATTCCTCAATGATGGTCTAATCGCTATCGGTTTCGAGGATGGGAAAATCGGCTTCTACGATCTCGAGGAAAAATCCCATACCCTCAATATTTCAGCACATGATGCCCGATTGAAGTGCATGCAGCACTTTGATGATCTCTTGGTGACTGCTTCCAGTTCTGGGGAAATAAAACTCTGGCGCTTTACGAATGACGCGATTACTTTGCTGAATCGAGTCACTTGCAA

>contig00941

ACGAATACGAGGCAGGACACATTGAGGGGGCCTTGAATCTCTACTCGAAGGAACTCATCGAGCAGTGTCTTCTCGCTCCTCTGCAGCATAAACCGGAAATTGAGCCAGACACCCACAAAAGAAGTATCATTGTCTTCCACTGTGAATTCTCCTGGGAGCGAGGTCCCAACCTTTCTCGCTTTCTCCGAAACATCGACAGACAGAGGAATAAAGAGCACTATCCAGCTCTCCATTACCCGGAACTTTATCTTCTCCATGGCGGCTATCAAAAGTTCTACCATGAGCAAAAAGAGCTTTGcTTACCCcAGGGTTATAGGCCGATGAGAGATCCGAATCATGAAGCTGATCTGAGACAGTTTCGAAGCAAGAGCAAAaGCTGGCAAGGTGAGAAGTCGAGAATCAACGGATCTACGGCGAGAGCGAATCT

>contig00942

GgTTTCaCTTTCGTCAGCTTTCGTGCGTAAATGGTGGTTTTCGCTAATGTTTGTGGAAAGTTAATCGTGCACTTATTCCGTAAATATTAGAGAAACCGAAAGTTTTTACTTCGCCTGTAACTGATTTCATTGACAAAATGGTATCAAAACCCCAGAGGAAATTCGTTCAGGGCGACAAAGTTTTTGCCAAGGTTCGAGGTTACCCGCCATGGCCAGCCAAAATTCAGGGAATCACTGACCCAAACACAAAAGGCGCAAAATTCAGTGTTTTCTTCTACGGAACTCAAGAAACAGCGCTCTGTAAATATGAGGAaCTTTACGATTACGAGGAGAACAAGAAAaCATTCGGCGCTAAGGCCATCAAGCGAAAACTTTTCGTGGAAGGTTTACAGGAGCTCGAGGAGGAACTTGAAAAAGACAAAGCCTCTGCTGTTTCTACTCCTAACAAGGAGAAAAAAGCT

>contig00943

AAaGCTGCAGCAACT

>contig00944

ACATCAATAAAGGCACGTATCTTAACAATTATGGCACTGGTTTATACGAGTATCAAAGGTCAAAAAaCAGATGATTATAAAaTATAAAAAaTCCTtATAGTCGGAAAAaTCAACAGACTTTTTAGACCTCGATTTTTTGTTATGGCTTTAGACACCTATGAAaTTaCAAAGAGTATAAAATCGATTAAAACCTTTCATGCTTGTCTTtATCTTAGTTTCTGAATAAAGTAGGTATTTAACTCCTGCTGCGTTAAAAAaTATAAGAGTTCTCAAAACCAGAATCGAAGCGTTCGTGATTAAGAAATAGTTCAGACGAAACTAAAACaGGAAATTAAATGGAAAaTTTCTGGGCCTTACGAGAGGTCCTTTTTTtAGCACCACGACGGTTtCTGGAAAATCTGAAGGTTACATTTCCTTGAGAaTCTTTGGAAACGTGCGTCTTGGGGTCATCCCGTCCTCGAGAAGAAATATTTCCATATGTATTACTCGGGGGTctGTAATCAGAATTGCCTCGGGAACCGCCTCCAAAaGTCCTTTTCCACCAGGAATCATTTCGACCACCAATGGAATTGCTGGGAtAACTGGGGT

>contig00945

AGTTATGGTGCCTATGAAGTTAAGTCAAAAATATTGTTTAG

>contig00946

AAAaGGAACAAAGTTATTGGACCTTCACAAAGAGCTGGAAAAGCTCTTCAACTCGGATTCTAATGTGCGGCTTAAATTTtCACAAAaCTTTTTCTATGCTAATCTTTGTCAAGTGTGCCATAGGAGTGCAAAGGATTCTACTTTGAAGAGATGCTCCGGATGTAAAATGATTTATTATTGTAA

>contig00947

AGCGACGAGATCGCGAAAGTGGTGGTGCGCGATTAAAaTCGTCGTTTACGGCGACAGGGGGATAATTCCTCCCTAAAAAAGTGTTCTTCATTTTTTCTCGACGC

>contig00948

CAAAAAGTCGTTTCTCCATACGGAGGAGACCTTATCTCTGTCGTCGCGCACGTTTTCGGCGGCTCGTGCCGTCTTAACAATGGACAAAAAGTCCAAATTTCCGCAGTCCTCGT

>contig00949

ACCAATCACGATTTGCTTCCAAGAAGAAGGCCCGAGCAAAGAAGAAAATTCTTTAGATTTAAAAaaGAATTTTAATCAAAGAAATTGATTTTTTAaTTtATCTTAAAGAATCGACAAAAaCTCACGTGGAAATTTATCCTGAATATTTTAGAAAGTAGCATGAAATTTCGTgAaTTCTATCGACAATTTCTAGGGTTTtATATTtCTCATTCCGTGAAAaTTGATGAaGGaCAAaTGGTAAGAATCTACGAGACTCTCTCTGGGAGGTTTGGAAAaTATTGAGAAATTTtCTGGGACATTTTtGAAAGTATGATACAATTCTGTCAAAAGTGTGATTTGGTAATTTCTAGGATTCTCTATTGAagTTTCTTTAAGAAAAAaGAGTGTTTCACGTGGGAAAAATTGTTTGTTTCCCccTCGTTTCCTTTCTTTTTtGTAGAAAAATCGTTGTTGTGTTTATGTGCCTCGCTTGGATTAATTTAAAGTTTCTCCACATTATTTAATTCCGTtCTAGACAATAGGAAATAGACTTAAAaTTGAATACAAGTAGTGGATCTTGCACACGGATGAATTTTGTCTATCTATAAATCTCGGCGGAGAAGGAAAGGAAGAATGGAGAGCACAAGGGAAGAAAGAAAGAAAGAAAGAAAGAAAGAAAGAAAGAAAGAGAGAAAAAAACCTGTCCCCAGTCTGAGAAACGAGAAAAAATCTAGAAGTTAAAGAAAAAGGTTCTCAGT

>contig00950

GACCCGTCCTCCCAGTTCGAATTaTtCTCTGAAGTTGATTCGTCTTCACCCAAATGATCTCATCGACTCTTTCAtATCCCCACAGTTGAAGACATTCTCTGCCGAGCTCCATCGCT

>contig00951

ACTTTGAGGGACAGTCTTCTTGGCACCCTCCAGGAATTCTTTAGCATTGACTGGTTTAGTATTTCCACTGACAGGTGCACTAACCGGTCCCGATAAAATGTCAGCATTCAGCCAAAGAGGGACTGTCAGATTCAATTTATGTTTTTCGAGGACTTCCAATCCTGCCTTGTAGGCCTCAATCGATTTAAAATCCAGCTTTACACCTTTTGTGTGATTCTTGAGAACCAGGGTGAGGAATTCATCCAGGGATAAATCACTTGTTTCGTTTGGAGGATGTGCCATAATAGGTATCTTTGTTGTGTTTGAAGTAGTCAGATTTCCCAGGACGATGTCAGCTTCCAACATCATGGCGGCTGATGCCAAAGCTTCTGTAAGTAAGGTTTTGCTGTTTACTGCATGCTTCCAAGTAATTTTTGTTAGATTGCCGTTGATGTTCTTCTTGAAAAATTCATTTGCACTTGGTATATCAGCCATGGCAGTTTGAATCACTGCCAGGATAACCAAGGAAATAATGCTGATTCTTCGCGCCATGCTTATACAATTTTCAGGAGCGGAACACTTTACGACCCGAATGACTCGAGAGGCTGGAAACGACT

>contig00952

GGCTGCGACACCAGCAGATGTTCGAAGGAAAAaGaTTTTGGATTCGTTCAACGCAGTTCGGCACAATCAAAGCCCATGCATGAGGGAATTTGGACTCTCAaTTGGAGGAGAGTTCGAGAAaGTGAA

>contig00953

ATGATTCTtGACTGCTGCGCGGAAAATCGGACCTATGAAAAGTTCTTTGGACTTCTCGCTGGgCGTTTCTGCGCGATTAATCGAATCTACGTGACACCTTACGAGCAAATCTTCCAAGATTCCTACGACACAATTtATCGTTTGGATACGAATAAACTTCGCAATGTAGCCAATTTTTTCGCCcATCTAtttGTTACCGATTCGATTtCTTGGGaGGCACTTTCCTGCATCAAGTTGAACGAAAACGACACGAACAGTTCTGGTAGAGTCTTTATCAAGATTTTGTTTCAAAAAATGTCCGAGGAGATGACACTTCCAAAACTCAACGAAAGATTTAAGGATCCGACTTTCCAAGATGCTTTTTCAGGACTATTTCCCAGGGACGATCCGAGAAATACTCGGTTTTCGATCAACTTTTTCACGACGATTGGATTAGGAGGATTAACAGATGATCTTCGTGAGCATTTGGAGTCTCGCCCAAAA

>contig00954

TCAACTTTAGTAATTTGCCTtAAtGtAAAtCGATTtGATtACAtAAtACtCCtAGtAACtGtGAATtAAaTTtACAATTCCAAATAGAATACTTAaGGTAATAtCAATAACTTATGATTGTAATACTAATGATAACAATCGATTATGATTGACTGATCATTGTATTACAGGTAACAATTTCATTTGCATTAACTATGTAGCGATTCATGAGCAACGTGAGAGATTCGGATTCACAGAGATGATAGCTAAGTCTTGACTTAtACCTTTTTAATTTGgACGATGAGTGTTATTAAATATTTTTAAAGTTGAAaCCGCTGAATTTtACTTGTGCaCGCTTGATAAACATCGCAATAGTATTTTTGTTTAATAACTTATAATCATTCGAGTTGTTTCCGTAATGACTTCGTCAGGTGTTTCAGTAATTGCATGCAATTATGTCGCCGTTTCTTTCTTCCCcGCCCGTGCATCATCCTGCTTTTTAAGCTGCTTTTCTCTCATTCTATCCGCGTCTCGTtGTTTTCGTTGTTCTAGGGTCAAGCCTTTGTTGTTTTCTTGTTCGGCAGCAGCCTTTTTTTTGTCTTTCTTCATATTTTTtGCACGTGCCAGTTCACGTTGATTTCCACGTGTCATATTTtCTGTGTATTGATCTCCGATTAACAAGT

>contig00955

TTTTTtCTTGCGTATTGTCCACTAAACCACTGACAGCTGCCATAGATGTGATGGAGGACTTTGAGAACAATCTTTCAGCTTCTTTAAATTTTCTGTTGCGTTTGTCAGCTTTACTGTTGGTATTCTTATTACTAGCTAAATATCTGTCCCAACCTCGAATTATGTTGCCATATAATTGCGTATCCTCTAGGTAGCTGCCTTCAAAAGCATATATTTGTCGTTCTAAATTTGCCAATGTGTCGGCAATTTCTGCTTTTCGTTTAACAAGTTCTGCTAATTCTGCTCTAAGATCAACAGAAGGTCTTAAGGACATATTTACGTAATTTTGTTATTCATTAAGACGCGAGGAACACTGTGCAACACTATTACCGTTCTACTATGAAAACGATGAAAACAAAAAATTGATTAAGAAATCACCAAAACTCCCAAGATCTTTTAAGATAT

>contig00956

ACTTGAGTATCGATAGCAATAGGATTTATACCAAGATCCTCGAGCATAGGTAGACCTTTtATCACATTATCACTAATGTGTTCTCTTtCTAAGGCTTGCCAATGCAAaTATCCTACTGGATAGGCAGGACTTAATTGTTCTGTTAGCGTAaCTTTtAATTTAAAGAGAGGACAATAATCAATGTCCAACCTCCTGTAtCCTGCTTCATCAGCTTCACGTCGCAAAATTCTCATGAACcAATCCACCAGATCTGCAAGTTTATAACGTcTCGGCCCcACAAACTGATAagTTCGTCCAATCGATTCGGAATCTTTCATGGCAGCTATTACTCCGGCTGCCACATCACCAACCCACACAGGTTGCTTCTCAGTTTCTTCtCCTTTTTtATACAGTGGAACACCATTTAACAAATGATGTCTCCATTTACTTTGATACAGCATCAAAAAaCGATCTTCTTGGCCATAAACGTCAGAAGGTCGAATAATGGTAGCTTCTGGAAATTCTTGCCTAaCAGCCAATTCCCCGTCCCATTTTGACGTAAAAAATCTAGATGGCTCTTTCAAAAAAATTTTCTTGGGGTTAGAGTTTGCATTAAGACAGGATAAATGAATTAATCTTTCAACTCCACTTTCTTTACAAATCCTGGAAAGTTTTCTTGCCCCTTCTATGTGAACATCTTCGAAACTAAAATTTTTTGTTTCATACTCTCTTCCAATTAAATTGATGACGACATTGGAGT

>contig00957

ACTTttCGGCgaaaaTTTTGCCGCTAGGTGTTGGCGtAGAAAaGgCAATGTCGTCCATTGTCATTaTATAGCCAAAGTAGTAGGATCTCCTAATTGTATCGACGAGTTGCCGAGCAATCCCCTGTCTGCGGTGACTCAtCGCCGTCCAGACAACATTGACGCCACACTTgaCAGGAGTGCTTTCCGAGCTACAACAATCCAATTCCACCAGTTCAGGAATCATCCGATGAGCTGCCTTTACCGGCTCAGCGACCAGAACTCCCAAAaCaTTTTTAtCTCTCACATACAGGTAGACATTCTTGTCACCATAATAAGCCAACTGCATATTAGCAAGTCCcAAATCCGTGTCCACGACATTCAAAAtCTCCGACACCTTCTtCCAGTGCTGCTTCGGATCaCTCGGCTCAATCAGAATAATCCTGcTCGATgTATGCTCCTCATCCCACACCAATTTCTCACTCTTCCATCCCTGAAACTTGAGTGTTCTCACATTATTGTGATAATTCAAGTGGGCATTCTCATCCTCGGCGTCACCAACTTGATAAACAATTCCGCACTCGGTGCACTGGGTCGCGCCAAAGTTCTTCTGTCCACAGTCGATTTGATACTGGTCATCAGCTCCACCCGCTTTTGATGAAAGTTGCCAGTTCGCTGATCTCTTCACTCCCCGATTATTATTCTTGGGACTGGCGACAACTTTAGGGCTCGTGTAACCTTTCTCGAAGAGAGGGTAGAAAGT

>contig00958

CTCTCCAGTGCtctGtGCGACATGAAGTCCTTGGCCGaGAACGCTATTtATAAAAaCGGTCGTATCTGCAAAGGCGAAGCTACTtCGTCTCCAAATTTTGTAGAGAGGGTGGACCtCGAAAAGGAaGAAAGCcGTTTGATACCGAGCGCTTTTGGATCCTACGATGGCCgaTCTTCTCCAGCATCAAGCACGACTGATtCAGAGATCATTCTCTATGTCAACGATATTGAAAGGGATTtGGGGGATGACTACGAAGTTATATACACCAaCATTCCAAGGATAAAaGAAGAGGAAGATATTGACGACGGAGAAATTGTGGTCTTGAAGGAagTTTTtCGAAACGATTTCGACTCTGTCCGAaGGAGGCTTGAGCTAGCTAGTGGTGGgAAAGTGGATGCTGATTTaGGAATCATAATGCAAGCCAACGGTCATGTCAAGCTGGAACCTAAAGTGGAGCCGATGGAGGCCGAACCTGTTTCAAAAGCGACACTTTCCCAAGT

>contig00959

CGGGAATGAATCCATTTTCTGGAGGTGGACTAGCTGTTGTTGGcAAAAGCCACGTGACtGCTGCACtCTTTTCACAaTATTGATCAGTCAAAAGTCCTCGAATTTGAGCATAATAAATACCACCGtCTTCATCCTGCATGGAAACTATGTCTCCAACTTGAAAATACGAGCCCTTaTAAAATACAAAATCACTAGTAACCACCGTTGCCGTTGCAGTTGGTGCTTTCATCGGCGTTTTCTTAAAAATATTCCGTCGTCctCTaCCTTTAGGTGCAGAACTTTTCGAATGAGGTGCTGCaGGaTTTGGTTTGCAGTATCGTGTAACCCTTGT

>contig00960

GTTAATAATCCAATTTTAACTCCTAGCGCTTTAAGCCTTAAATATGTTACATGGATTTCTTGGCAATACATTTTGTTGCATTTGTAGATTAGTGTTTATAATTTttCTCGAATAGATGTGAATTTATGAATCTAAGCTAAGAAATTATTATT

>contig00961

GCTCCTTCAAGTCGCTCGGGATCGCCGCAATGTtCtGGACAGAGCCATTGTTTGCAATGATCTCATTCTTCATGTCTTCATCCCAAAGATCTCGGTCCGTGAGATCTTTGAGAAGATGAGGATTGACAACCTGAAATTCTCCGGAGAGGACTCGCCTCGAGTAGATGTTGCTCgTGTAGGGCTCGATCGACTCATTGTTTCCTAAAATTTGTGCCGTCGAGGCAGTTGGCATTGGAGCAATCAACAGAGAATTGCGCACtCCGTGCTtCTtAATTTTCtCCTtcAGAGCACTCCAGTCCCACAAaTCAGTCGGTGTCaCATTCCACATGTCGTATTGCAAAATCCCTTTGCTGACTGGGCTTCCCTCGTAAGTCGAGTAAGGACCTTTCTCTTCAGCGAATTCACAACTGGACTCCAAGGCCCCGTAATaCAGAGTTTCGAAAATCTGAATaTTCAGCTTCTGAGCTTCCTCGCTctCAAaTGGAAGACGCATCAACaGAAATGCATCTGCCAATCCcTGGATACCAATTCCAATAGGCCTGTGTCTgCTGTTGGATCgCtcGGCCTCGGGAATCGGATAaTAGTTATTGTCAATGATCTtGTCCAAGTTTTtCGTCACGACCTTGGCCACTTCCTtGAGCTTGaCAAagTCGAAAGTCTTGCtGCTGGTATTGACAAACATGTTGACAGCGATCGAGGCCAAATTGCAGACAGCCaCTTCCtCCGGaCTCGAGT

>contig00962

GGTCCGATGAAATGATTTTtCACGAAGTAAGAAGACTTCTGATTGCTCTGTTTC

>contig00963

CGTGACGATTCCGTTCCTCTCGCGCACGTcgCCATTGCCGTTGAAGGTGTCGGATGGGCAAACCCCGACAACATTCCTCTCAtGGTGGCCAAtACTCTCCTCGGCACCTGGGACCGTAGCCAAGGtGGTGGTGGAAACAACGCAaGTAaTTTGGCGAGAGCATCTGCCGAGCAAGGATTATGCCACAGCTATCaCaGCTTTAACACCTGTTACAAGGACACCGGACTGtGGGGAATTTACTTtGTGTGTGATGCCCAGCAAGTTGAAGATATGACGTTTACCCTATTACAAGAGTGGATGCGAATTTGCACTTCAGTTACAGAGAaGGAAGTTGCGCGTGC

>contig00964

ATTTAACTATAATTTTAATCTTAATATGCTAGATCTCATCTCTGCAATCCACACATTCTGATGTGCAGCTCTGGGTTGTTAACAAATAATCCTGCTTTGCAGTTCCAGATTAGAGATTGAAACATAGAGAAATTGGAGTTTGTGCTCGGACCTAGTCCTCGGGAATGATGTTCCCTGCAACATGGAATAGAGGCTGGGCCAGGATATTGTCAATGTTTTAGAAAAAAGGATGAAAAAGTCGAAGATTAGGTAAAGGATCGAGAGGgAGGAGGATCTGGGATATCAGACTtGTCCTCCAACATCTTCCTGGTATCGGAGAGTGAGATATTCAGCGGCTTCGGTCATTCGAGCGATGCTACGATGGAGGCCTCGCAGATGACGTCTgAAGTTCTCTGCTGCTTGATTAGCCAATCGATCACTGGGTTCTTCGATTTCGAGGCCAGAGTCGTTCTCGATACCGGCGTCGGATTCGGAGTCTGAGGAGCTAGAGACAATCGATGATGCTGATTGGACGCTCTGCATCGATGTCGTGCTGGGACAACGAGCGTGACCGAGACGCACACTGCTTGTGTCGGATTGAAATTGGCTCTTGATTCGCTGGCTCTCCTCGCGATCGAGGTCCACTTGGGTTGCCTGGGGGGACTCCTGGGACCAGAGAAGCTCGACCTTCATCTTGTTGACAATGTTGTAGAGGCGATACAGGTCAGTGTTCACCATCGTGTCCTTGATGGTAGT

>contig00965

ACGTTTGGTCTTCAGTTCTTTGTCTGTCTGGACATCGGTTGGAATGCTATTAAGGCCCGTTTCCAGAAAAGACCACTTCTTGCCAATTATATCATGAGAACGGTTCTTGTCACTGGCGCAGTGTTGCTGGCCGTTGCTGTGCCATCGATTGGACCATTCATAGCACTATTAGGAGCATTTTGCTTCTCGATTCTGGGACTTCTGGTTCCCGTTTTCATTGAAACCGTTACCTTCTGGGACATTGGCTTTGGAAAaGGAAATTGGGTAGCCATGAAAAATGTAGTAATTACGATTATAGGTATTCtGGCTTTAgTGTTTGGATCTCgAAGTTCCTTGATAGATATAGTGAAAGAACTTtAtGGTAAAGATGGTAATAGCCCCCTTcTGAATACAACAATGGCAAGTGTTCTTAATGCAACGACAGAAAGTAGTTTACTTACAACTaTAGGCAATTAAATCGTAGTGTTAACACTTCAAGTGGCTACATTTGACTCCTAGCTGTCTCGAGCCTCTGACTGACCTCAAGACTACATTCGTGAGCTGTAGAATTTCTAAAGACAAGAAAAAAACTTTGCACAATTGCGAGCCTCACTCTATATTTCGAATATATATTATTGCACTCTACGAAAAAAAGGTTAACGAGGCATCCAGAGACAGATTTTCATGAAAGCAGTTTCTGATTCGAGTAAGATTTTGTAAATTAGTTGCATCATCGACGCAGGAAAATCTCGT

>contig00966

ACTTACCGCATCCGCATGATTCaGGAAAGACTCTATTGACTCAAGAGGCCGTAGTTACGGTTCAAGGTGTTCCTCTTACTCACTACATGGAGGACCTCTtGACTTCGAGGATTTCTTTCAATGCTAGCAAGGGAAGGCAAGCTATGGAATGGGTAATTGACAAACTTGAGTGTGAAGTTAAAGACTT

>contig00967

TGTAAAAacAAAaCGGAGTATGGACGATCTTCAAaGTGCTGCAAAGAAaTCTTTCGACGGAATtcACAATTTaGCAAcACCACCGTCATCTCAGTCCATGCCC

>contig00968

TAGATAGGAGAAAGTTtGTCTCTTATGTAGAGATGTTTtACTATCAGATGTTGGATTTTTtACATCCTCCTTACGATAATTAGTGAAATCTGGCACCTGGATGTCGGTATGTGCCAAACGAATTTGTTGAGCTTGAGAGCTCACTGCGATTCCTGAGCTCACTCGAATTGGG

>contig00969

aCcAAGCAAACTGCTCGTAAATCCACTGGTGGAAAGGCACCAaGAAAGCAaCTGGCCACCAAAGCcGCTCGCAAGAGTGCCCCgCAaCAGGAGGAGTCAAGAAACCTCATCGTTACAGGCCAGGAACTGTCGCTCTCCGTGAGATCCGTCGTTACCAGAAGAGCACTGAGCTTTTGATCCGCAAGCTTCCTTTCCAACGTCTtgTTCGTGAAATTGCTCAGGATTTCAAGACTGATCTTCGTTTCCAAAGCTCCGCaGTCATGGCTCTTCAGGAAGctAGTGAAGCTTACctaGTTGGaCTTTTcGAagAtACCAACTTGTGCGCTATTCACGCGAAGAGAGTTACCATCATGCCCAAAGATATTCAATTGGCTCGCCGGATCCGTGGAGAACGTGCTTAATTtcTACCTGtCCTAAG

>contig00970

ATCTTtGGAATAACGGGCTGAACTGGTATAGCTTTGTTGAAATTTTTCGTGTCTATGCCCAGTTCTTtGTCTTtGGgAATCACCcTTtgcAGGTTTCCTTCTCCCTTGTCTATAAGTATATGGAGTTTCGAATCCACCAAATGCTTTAAAaTGTGCATTGCTTGATAGTGATGCATTCTTGATAAAaGAACAAAGAGTTCTGCAATTGTGTGGTTAAATTGACTCCACATTGTCAAAAGTTCATCCGTGGGATTCTTCTCATTACGCAGTTTCTGAATGGTGTCGAAATCAAATTGCATCCGCTGGGATCCTGCTAATTCTTCCCATTTACCATTTtGGTTTAAAaTTTtGCATAAATCCATTCGAACCAAGTAGTCCATGTCGTAAATATACTTGGCTTTATTGACATTCGAatCAGAAGACATTTTTATTATGTATGCACAAGATCAACAATTGCCatCAATCACCAACAATGAT

>contig00971

aCCCAGAAGGTGAAGGTCTTATCGAAGTAGGCGTCGTGGAAACATACTGCTGCTGCGAAGATTGAACGTAGGGGCCTTGTTGATGTTGAGGTTGGGAGTAGTATTGTTGATTCTGTGACACTGGCGGAGGGACATTGTAATTACCATAGGCCACTTGAGCGGGAGGAGGGTTATAATAATGGTGGCTTTGCTGAGAGTGAATctCATGAGTGTTTACAGGTGTGTCCTGAATaGGGACGGtGATCAAGTTAGTCCTCATAGGGGGATTATTGGGTGGAGGCGGTGCGGAATTTTGAGAGTAACTTTGCATGGACATCATTGACTGAGATCTGTGTTGTTGCCGGTGGTCCACAGGTtGCTGAGTCACGAGAGAATTCACTCTGGGATCGTTTCCCATTATAGGTTGAACcATATGACCTGACTTCATGCGACTGTTTtGTAAAGCCACTGGCGAGACTTTTGATAATTGAGACTCCATTTCTGATTtCGGGTGTGCGTATTGGGATTGgTCGACTTGCATtCCCTGGACAGATTGTTGTGGAGCTGATATATGACGATGATTGATGTGAGCTTGTAAATCCCTTTGGCTAAGGT

>contig00972

AAGGAAGtCTTtCCAGAACAAGGATTATgTTTACAACGTAGTTTGGGAATCAGTTTATGGaTCGCTCTCGCGGCCACTCTGGGCCGTAAGTGTAGCTTGG

>contig00973

ACGATAAGAtGCTTTCTGCTACATGGTCCAGTCCTTTCTTCGGCAAGAGAGATATAAAATCCCTTTGTAGCATTGGTTTtAGGTAGGCATtGATGTGtCCATGCTGGTAGTGGCACATTctCGCTAAAAGTTGTTCCACAAaGTGCACTTGTTCTGGTTCGCTCCATCTTtCAAACAACTTCATACAAGCTTCTCTCTCTTGACCAAACTGAAAGGAAGCGCCTATCGGCATTTCTTTCTTCCGTGTCGTGTCGTTTAATATTGTCATCGTATTCTGCAAATTGATTGAGTCCTCGTGTTTGGTGTCCGTCTCCATGTTGATCATTgTTTAACcGGGCGGACCGCACAAGTGTCTATGCAGGACGATGCACCCGTTGATCCTAATTTtGCGGAAACGGGATCAAATAAaCGGGAAAAAAaCACTAAACCGGACACACCACTGTTTGACTCGTTTGACTAAAGAACTCGACGACGGACGACGCTCACGGTCCTTCCCCATGCACAGGAAGTCGATGCAGCATTTCACCAACACAACAGATCATAAATAACGGTCATTTACGTCATTTACGTGATGGGCGACGGCGCACTGGCCAGCTTTTGTGGAAAATACGTCACTTTtCCCGTTTCCAGAACGACCACGACGCGAAGAGTATCTCGTTTCTAGGAGAACTCTTGGAAAAaTCCGCTGCTCATCAaCATCATTCGAGATCGAGTC

>contig00974

TTGTGTTtGCAGAATAAGATAATaTACAGAAAATAATAAAGATATTATTCCCGACTTGCCTGAAAGTTGACTTtCGAGACCTTTGAACGTGACCACGTTATCATTTTTTtGCACAAGGGAAGAAAACAATTTTGAGTGTGTATGCGTtCAGCAGAATCAAAAGTTCCTGGTAAATTAACATAAaTTTCCGTAAATTACCATAAA

>contig00975

ttACACCCAAGTCAAACTTAACCAGTGTGTAGTCTGTGGATCCAGCGAAAAGTTCGTCAGGAAGAACGTCGTCCCGAGAGAATACCGCAAATATTTTCCACTTGTAATGAAAGCGCATCAATCTCACGATGTGTTGTTGCTCTGCCCATCTtGCCACGAAGTTAGCAACTACCACGATCTAGAGCTCCGAAGGCGTTTGTCAGAAATTTGCGACGCGCCTCTACCTGGGCCGTCGTCTCACACGAGGAATGAGATTCCCGCTGGCTGGCGAAAaTTGCAATCGGCAGTCAAAGCGCTGAAAGGCAAGGCTACGATTCCTGCACAAAGACGCAAGGATCTCGAGTCTTACATTCTTTCCTGCACTGGACAGCGTGAATTCTCGCCGATGCTGTTGGACGTCCTGAACGAGCGACTGAAGAGCAGACCTGAGGTCAAACATTCAAACAAATGTGACCCACATGGTCTTAAGGTGGTGCGACACTTTGAGAAGCTGGAAGGCGGTCTCGTGGAACTCGAAAGGGTATGGAGAGAGCATTTCCTAACAGTAAtGAAGCCTCGATTCCTTCCGAAATTGTGGTCTGTTTGTCACAATCAGGAGCGCCTTGCAATTCGCCAAACGCAAAATAGGATAGAGCCACAAGATGCGCGAGTGGCTGGCCTAGCACATtAAATTTTtCGAAAATATTTTtaTAAaCG

>contig00976

CATTtGATTTtACTATCCTCAGGCCAAACTACAAAGGGCATTTGTATCTTACAGGCTCGTCATCAGTGAAACCACTGAGGTTATTTTTAAAACTTTtGTTCGTAGTGTGGATTGAAAGACAAACGCTTAGCAATACAAAAAaCTTATACACATTTCGATTAGTTGGATCAATTCGACGTAGGATATACACGCCGATTTGAATAAATTTAtATAAAaTGAAGAGAATATACATtGCAAGTGCGCGCCTAGGCAATTAaGGGAGAGATTGAGAGCGAAGGAATTaTTAAAAAAATGTGTAGGAAAAGGGAAATGAATTGTGGGAGGGAGAACTGTTTtGGATGACAACtAAtCAATGTAGAtCcACATACCCATTTGTAACACAATTAGTGGGTTATAACATAtAGTTTACGATTATTTGTAAAGGATtAC

>contig00977

ACTTACCTTGATTGCAACACAACGAAGTGTCAGTGTTTTCAAAGCGTAATTGAAGGAGATTTAGCGATTTTTAAAAAGAAAGGGATCAGTCGGGAATTAATTAAAAGTGCTAATGATAGAGGGATATTATACCAGTTAATAGACGGACGCTTGTATCGTGAAAAAGACTGCATGTTtCCCTCCAGATGTGCTGGAGTGGAACATTTCATTTtAAAAGTTATTGGAAaTTtATCAGACACAGAaCTCATTATCAACACTCGTGATTATCCTCAATCTAGTAAACATCTGGGTGAAATCTTACCTGTATTTTCATTCAGCAAGacaCcacaTtaTtatgataTtacgtatCctgcTtGggcTTtttGggAaGgTGGACCAGCTATTTCACTCTACCC

>contig00978

ACTCAAGATTTCGTTCAGAGTTACGAAACCTATCACCTCGTTTGTTTTGTCGGTGACCGGAAGTTGGTGGAATTTTCCTTGTTGCAAGATATGAATGGCCTCGTCGCAAGTCGCTCCTTTGGGCAGAATCACAGGTTTGTTTATGGGAAGACTGGAAACCGGGAGGTTCCACCACCATTTATTGCACTCGATTGGCTCTGGAGGATTCATGAAACCTCTGCTCTCCATCCAATAATCTGAAACGAATTTCGTCAAATAaTTTCGAATTCCATCTGGTAATAAAATCACAACTCGTTTGTCTGCAGGCAGATTTTTCGCCACTTTGAGGGCAGAAGACAAAATTCCCCCACTGCTTCCTCCAACAAGTAAACCTTCTTCTCGAATCAGACTTCGAGCTAGTTGGAAAGATTCGCAGTCGTTTGaTTTAACCCACATATCGATAACATTGTTATCCAGAACTGTGGGCACAAAATCGTATCCAATCCCTTCAACTTCATAAAACGTAACTGGaGTTTGGTTTAATTCTGGAGGATCGGCGAGAATACTGCCTTCGGGGTCAACAGCCACTATTTTAGTCTGAGGCGATATCTCTCGGAATTtACGACCAATGCCACTAATAGTTCCTCCAGTTCCTGCACCAGCAACTAAATAGTCGATTTTCCCTTCAGCCTGCTCCCAAATTTCCATGGCTGTTTGGTCGTAATGAGCCAATGGGTTTCCACTGTTTG

>contig00979

CTATTTTTATTTTTTTATTAAAATaCATATTGTCATACTTAAAAGCAATTTCGGCCGGgTGAAAGTaGGATTTCACTAAATCGGTCCAGGGTATCAAATCGACTATATGTAGATTAAaCAGCCTACAGCACCAACGCATAAaCTCAAATAGTAAATATATTAATTAGTTTACAATCGAAAATCGATTTCCTGACAACTCCCAAAATTGCATCTTAaTCACTTGAAAATGTAAAAAATTAAAAGGCAGCATTGCTAACTTATAATATAATTACAtATGTCAATAAATCTTTATAGTTCAGGAGATtGGTTTTTTTTtAGTCATCCATTACCTCAATTCTGCACGCATATCAGTAACAAATATATCATATGCGCTCTTTaTTATAATGTATAGATACATGTAATTGCATAACATCTAAAGCCAAGCTCCTGTAATATACACAACAC

>contig00980

AAGATTCACCACGTCTAACATCTCGAACATCCGGAACCTGATTGGTCACTTCTCTGGGGTTTTCTGTCAACAATTTTACAAATTAACAGTAAAAAGACCGTAGCGGACTTAGGTTAAATAGCTTAATTAGATTTACACGATTTTTCGCAGTTCCATTCGTCATTCAAAGATACGTGAGTGCACTGTCCATAAAAATGTGTATTGATTTCTTAtACTATTCATCATTAAACTATCCATCATTATTaCACTATAaCTTACTCTAATAATGTGCTCATAGTCTGACTATCCAGTTAATAAaTTAAAaGTAACTGCTGCTGATGTtcTTtCTAAAGCAGAGAAGAAAATTCTATGGATGGAACATTTACGATAGGAAACCAgAATTTTTTTTATCTATAAGTATACTTTTCCGCCATACTCTAATGGGGTTttGCACTCTTAGTATTtACAACACAAAAATATACTATGATTAGCTACAACAAAAACAATAAAaTACTTAAAATTACACCTCTGTCAATTCACATTTGATTTCAACTAATTCGATTAAaCACTGATTCCCCTTTTGAAAGCAATCTATTAACAGTATTTGATTAGCTCTATTACCGAGATGGTGTTGGTTGTTTTGGAAAGCTGATGAATAAAGATTATGTGCGTCCTTTATTCTGCTGAGAATCCTAGCATGATAACGTGTTTAAACTAATAGCATTATTACGATGCTAGCGCTAATCTA

>contig00981

AtAATtATTtGCAGGGAAACACACCAAAATTGCAGGCTCGTCTCCGGAAAaGGGGACAGAAAGGTCACTGGTCCTACATTCACACTATTCGACGGCCGAAGATGTGCGGACAAGTCGTTGAAGTCAAAACGCAGTTGACACaTAGGGATTATCTGAATATGCTGGCTCAGAGGGATGATTCACACTTTaCTATTTTCAAAAGACGACGTTGCTTCTTGATCAACAATCAGTATTTCCAACTGGATATaTACAGGGAGCCGGGACACCCGAGATGCAGGGGATTAATGTTACTCGAGACCTATACAGCCTTGaCTGGTGACgATTTAAAAaaTaTTTtACCTCAATTCTTGACAaTTGAGAAGGAAGTAACCGGAAACCcGGATTACAGTATGTTCAATCTCAGCCTGCGTGAGGAATGGAACGACACGAATAAATATTGT

>contig00982

ACCGAATTtGAtAGCGaGACaGAGAATCCACTTGTTATcGATATGCCCGAACACAAtCAaGgACAAATGGGCGGAACTATGCgaTTAGGAAAACGGACAACTATTTtCACGGAAGGAGAATCTGTGATAAAGCAATTGTATGGTAACAAAGACAGCGTGGAAGAGAGACATCgTCACAGATACGAAGTCAATCCGAAATTTGTAACAGCTCTAGAaGCCGcAGGCCTGAAGTTTGTGGGTCAtGAcGAAAAaTTAGAGAGGATGGAAaTTATcgAACTGGAAGGTCACCCTTACTACGTTGCGACACAGTATCATCCTGAATATTtGTCCAGGCCGCTTAAACCTTCTCCGCCCTTTTTAGGATTAATTCTGgCCAGTGTAGGAAAATTGGAATCCTATTTGTCTAAGGACAATCCTAAAGCTTCTCCAGAGATTCTGAGCGaCAATGATTCAGGTTcAAAAAG

>contig00983

ACTGAAGTCCGGAACAAAAACTGTCCTTTTtCtGAATCACGAAAATTTGCTTATCATCCATAGTTTGTTAAACTAAAGGAATTTTCGGTATATACACAATACAAAGGAAATTCAGCATTCTAAATTAACATTTTCAAACTAACTTaTTCTGGAAaGTATTCGAAGTCTTAAGTTTTCGTGTCAGACGAAATCGCAATTAATaCATTtAGTAaTTTTCAAGGGTTCTAGGCAGGCCAATAGATTTTTCTTTCACCTCTGCATTtCTTATGAAAGATAAGATCGTGATTGCACTATCAAAATGGAACCATTTtACCTTAAGTTTTAATGCCAATGGAATATTTaGTAAATTAATGTTAGGACAaTTCCAGATgAaGAAATTTtCACAGTGAGAAGCGCTGGAAGAATATAACTTTGATAATCTCCACCTCAACAGAACTGAtAAACATTTTCCGATATTAGGGAAGTATATTCATAaTAAaCAACATTAATtA

>contig00984

CATTACATCGGAAAATTGAGGCTGATG

>contig00985

CGTCCTTTttAAaCGTTTtACCGATCAGGCCTctGTTCGGAGCTGCTTCGAACACATCGACTAACTTTGGTTCCAAC

>contig00986

CTTTtCTGCCACCAACTTGACTCCAGAAGCCTTTGAGTGTtGGGTGAGATCGTAGGCAGACCTGTCAGCACAACCTACACATTCTATCCAACCATAGGATGTAaGGCACTCTGCGTCCCAaCAATCGCAAGCA

>contig00987

ACCTGGAATCTGGTTACAAGTCtAGAAAaTTCGAGTTTGTtGAAAaCGGGACGCATGCAACATCTTATGaGTAAAAAaTAAGAGGTaTATTATCGCAAAAAATAATTGGGATTTGCGATAAACGGAAACTCGAaGaGATACCGAAATCTTTTtGACGACAGCTGCTTGGTTTTGAAAAGTTGCAAaCGTTTACGATCTTAACTTCTTTCTGATAATGAACGAAAGTATTtATTTTtCGCAGTAAAGCGTTTTTtAAAaGATTtAGAACTTAAGTTGTGCGtGAAAGACTCAAGACCATGCAGCTtaTTTATAAATACTtCAAGTTTAAATAAACGAATTATTTTTTtATAATGTTTTTTGCTTCTAAAGTAATTATCCATGCCGAAaCTATTtGCCACTTGTGTATCTATTTCATTTTGCACACAAATTtGATTTTtGTTGATTATATGGTAAATTAaTTCTAAAGCCTATTCTGAAAATAaTCATTGTAaTTTGCACTGACAATGTATCTAGATTCCACGAATAAAATATT

>contig00988

TACCTAGAAAATAAAGAAAAaCTCTTAGAATTAaCGCCAACGCAAAAGAaGAAACTTCAGCACTTGACGATTGTCACATTAGCCaCAAAGTCAAAGTGCATACCTTACTCAGTtcTTTTGGAAGAGTTGGATATCAAGAATGTGAGAGATCTAGAAGACTTGATTATAGAAGCTATTTATGCGGATATTATAAATGGAAAaTTGGATCAAAGAAATTCACAGCTCGAGGTTGATTTTGCTGGACTTGGACGAGATGTTCGTCCTGGAGATGCCGGAATTGTTGCAGGAACTTTGTCTGCTTGGGGTCAAGCCTGCGATACAATTTTGGCGTGTATCGAACAGCAGGTCGCAAAaGCTAAtCTTGAGAAGC

>contig00989

ACCATtAACCTTATTtAATAAAaGTAGATACACTTCTTGAAAaaTGCTATACTTtAAAAaTaTTTGAATCGGAGAAATGTGTATTGAAACAAAGAACTTGGATAACTACTTTGTTGTATGTATGTAAATTTCaTGGATaTCCCTTGCCGGACTTGAATGATATCAAGCATCCTGTGCTAAAaGTGGTCGTATTGCATGGCTCATGAaCATTCAGTCTTAGGAATTTtATtGAGAAACGCAGATCGCAATAGCTAGATATGACCATTTtAATTaTAACAGTTTtAATTGTTGCTACAGTTACAAAAaTTAGTGATGGCACATTAAGACCCAGCGGCCcAAGATCAGTTTGCCTaTATGTATTCACATAGGTTGATACTGTGGATACTTAATTACA

>contig00990

CCTGCGCCTAGACCAGCTAAAaGtCGTTTTtGCGGAGTAaGCTTCCCGTCTGCGTCAACCATTCGCTGTTtGAAAGATTCGAAAGCACCGAAACGAACCGCTGATTTGGGAATAGAGCCATAGAGAAGGACTGAGAGTCCTCGATATAGTCCAAAAAATCCACGATTTTTCACGGTTTTTGTCACACAGTCCCATATTCCAGCATACTGTTTtCCTGCACCTGCTTTGCCATCCAACTGTAGTTGTGTCTTAACATATTCAGTGGGATATGTTATGCAAATTTCTATTCCCCCcGTAATTCCACCGGCAACGATTCCTTTGAGTCCGATGTTGCCACTGGGTGTAGCCGCCGCTGCTTTATCCTGGA

>contig00991

TTCCTGTAGCTGCCCATTACGATAAAACGGGCGAATATCCCTGGGAACTAGTAAAAAAaGCTTGGTCCCTTGGACTTACGAATGGACATATTCCTCAACATTGCGGTGGTTTGGAAGCCGGTGTTTTTGACGGGTGCATCGTTGCGGAAGAGTTGGCATATGCCTGCACAGGTATCGAAACAGCTTTGGAAGGCTCCGGTCTTGGTCAAACACCAGTCATTCATTTTGGCTCTAaGGAaCAaCAAAAAAaGTATTTAGGAAGACTGATCGAGGAACCCCTGGTTGCTGCTTACtGTGTTaCGGAACCAGGAGCAGGATCTGATGTGGCTAACGTTAAAACGAA

>contig00992

GGATTTTCCTTAACTTCTTGTTCGTATTGATGCTTtCTTTtGCTGACGATCACATCTTCTATTCCTGAGCGATCTCCATATTtcTTttCATGTATAGTATAAGCCTTaTAAATTGCTTGCGTCCTATCTTTCGAGAGATTTTCCAAAGCATACTTGTAAATCACCCTGACTCTATCGTGCtCTCGTTGCCcTTCTTCAAATCTGGCAAACGCAATTACAaGGTATTCGTCTAAACTTTCATCTCCAAAAAaTGTCACTGCGCGTTCATAAACCTGACGAGCTCCACTTATAAAGCCATGATTTTCTTCAAAACGCGCATATTTAATCCAGTGTTTGACATCCGGATGAACCATAACAAATCGTTCGTAGATGTCCCTGGCCCTTTGAATTTCTTTGTAaCGAAGCTCGAAATtGATGTAAGTCTGccaagcctgctcatctGgTTCCCATTCCATCCACCTTTCGAACACTTGGCGGGCACCAGCAATATTTtCCAACATTTCCTCCATGTAGGTAtATT

>contig00993

TATTAATGtcGCGTGTCATTATCTGATtTATGAGCAATATTCGCATTTCACGATTACTCTTCTCGTTATTTtAATTCCGAGTTTCATCAACAATGCTGTCAGCTTCAGGATGCGTCAACAAGATCGAGAGATGCTTCCTGATACAGCTCCAGCTGaGAAAAaCGAAGCTGGACTTTTATTCGCATATCGCGAAATTTTCTATCAATAATATTGCAAaTCGATCCATTGAGGCGTTATTGGGTCAGCTTGAGCTACGCAATAAAATCTCGAAAGTGCAAAAAAATGGAGGATACTGCTGGACAGAGACAATATTATTTAAAAATGGTGAaGGAAAaCCaGGaCaTTGCaCTCATCACAGTATTTGAATGCTTtaTGGAAaCaGCCCCTCAACAAATAGT

>contig00994

CATCACTTCGGCAGACAATGTCGAGTAGCTGATTATGGTTTTACCAAACTGATAGATTTGCTGGAAGCACTCACTCATACTGTCCAGGTGATGGGCGAAGGAAATAAACGTGTTGTTACCCTCTCTCACAGAGCGCAAGTTCGTCGTTTCACTTCAGATTTGCTTCGTGTTCTCAAAGCTCAAGCCAGCAAACAGGTCGCTCTTTCAGAATTTCCGAGTGTCTACGCCAGAGTCGTTGGGAAAGCATGGGATGTAGTCGACTATGGGGTTTGTGAAATTGGAGATATTCTTGGAGAAGTCTCAGAAAATACAGtCGTCGTTTCCAGTTTTAATGGAGATGATCAGCTGATTgCTATCCCGAAACGAGAGCAAACACCAGAGGAAaTAGAAaGAACTAAGCAATTCGCTGTCGaGGTTGTAGAGCTTTTGCGACATGCGCCACAGTGTAGAATGCTCTTCAATAAaTTCGTGCCTTCTTATCATCACCACTTCGGTCACCAGTGTCGGGTTTCCGATTACGGTTTTACGAAATTGATCGAGCTTTTCGAAGCGATTCCCCAAGTCGTGAAAATTGAGGACGTGAGCGGAGGTGAAAGAAGAATTGCTTTGACTGAAAAAGAAGGTCTGCTGATTCTCGACGAGCAAATTTCGAAGCTAGTTGCTCGTTCCAGAGGAAGTTTGAACGTTTCTGCCGTTCCCCACGAGTTTTTGCAACTCTACGGTT

>contig00995

TTCCTGGGGTCTCTCTGCTCTCCAGGATgTTGGTTtCGCGCTTCCATCaGTAAaCGATGTTCAATATAAaTGTCCAGCGCATCTTTtGCAACAACAGGCCTTTCTTtGAAGTCGGGAAGAATTTCTTGAaTGAGCTCGAGAAGTAAATTAACATATCTCCTCGCATTGCCAGCAACAAAaGCAGCGAGATCTTCATCAAACTCCTGAAGGTCATCGAGATCAATTACAAAATTGACTTGCTCCCTGTGCGCTACGTTAGTGAGCTGTTcTCG

>contig00996

AATCGATgTCTGGAAGAACTGAAGGATCTCATGGTCGATGCCTTAGAGACTGAAGGAGAGAACATCAGTAAACTGGAGAAAGCCGACATTTTAG

>contig00997

ACtacAtCAACGAtGGCGTTTATGGATCCTTCAATtGCTTGCTCTACGACCACCAAATTGTTACTCCCATGCCACTGAAGAGcGGTTGTGGGAAAATGATTCCTTCAAGCATTTGGGGACCGACCTGTGACGGTTTGGACCAGGTCGTCGAAAATGTTCTCCTgCACGAAATGGAACTTGGGGACTGGATCCtCTTTGAAAATATGGGCGCCTACACACTTCCGGTTGCTTCCAaTTTCAATGGATTCCCCGTGCCCAAAGTTCATATCGTCGCTGATGAACACATCTGGCTTtaTCTGAAGGACGCAGTTCCACTGACCgAGGACCACTTTGTGATTGGCAACACTCCCGCAAaTTTGAGACTTGGTCTTGACATTGGCGGAACGGACTTGGATGCCTGGCGAAATCCCGATGTCGACATTTCTGCCAAGGATATGATGATGGACTCTGCCAATAATTCGCCAACTTTCATCTaCGATTACGTCGAGGTTGATCCTC

>contig00998

ACTTAactaTACACacGGCCACTCTCAaCACAAAAACGTAATCGTAAACAATATCAGAGTTTTGCGATCGACACTAaGGTCTTTATGGCGATGCACTGATGATTATGTTGCTGCATTCtGACATCATGTTTAGACTCCGTTACTTTCGGCTTCAACAACTGGTGCTTCATCAAGCTTTGCTTTCTTCTCAGGACTCGCACCGTCCTTTGAGGCATCATCGGTCGCATCGGCTCCGACAGACTTCCTCTTTATACAACAGGTGTCAACTGGAGCGACTTTGTCGGTGTCACCACCATTCTTGGCGGGTTCCTCCTTCTCTTCAGCTTCCTTCTCGTCATTTTCCTTCTCTTCGCTCGTGCCATTTTCTTTGGTCTCTGTGACTGTTCCGTTTCCATTTTCTGACGCTTTGGAGTTGGAATCTTCGTCTACCTCCTCCACAACCTTCTCCACGACCTTTGCAGGCTTTTCCTTCTCCACGACCTTTGCGGGCTTTTCCTCCTCTACCACCTTCTTCTCAGGTGTGCTGACAACTTTGGTTTCTTTTGCTTCAACATCAGCCATTTTGATAGATTTGATGTGGATATTTACGAAAGCACTGGTAAATACACTGAAAAATCTTCAACACTGAGGAGCACTTTGAAGATACGAAGAACACTACAGTCCGCGCACCGAACGACAAATGGCAGAGGACTCGACTCGCTC

>contig00999

ACCTAAATCTCACTTAATGACAGAAACTGAATGGCGAAATTTAGGCGTTCAACAAAGCCcTAACTGGGTGCACTACATGATGCATGGTCCAGAGCCACATGTTTTGCTGTTTCGAAGATTACGAACTGACATCCCCATAACTACAGGAAATGGTCCCATACAATATG

>contig01000

ACTTGtGAAAaGAAACAGTCATGACTCtGtCGGTAGTATAAAaaGCTTCTTCAACTCCATCACCATGGTGAACATCGATGTCGATGTAGAGAACCCTCTGGTGGTATTTGAGCAATTCCAAAATCCCGAGAACTATGTCGTTTACATAGCAAAATCCGGACGCCTCGCTCTTTTtCGCGTGATGCAAACCACCACCCCAATTTATACAAATTtCGGAAGCTTGTTtGTTGAGCTTCACGGCCGCTGCCACTGAGCCACCGGCCGATAGCTGACAAAaTTCGTATAAACCGTCGAAAACGGGACAATCTTCTCCAACGTTAAATCTTTGCATTTGTTTATTGTATTCTGTCATGTTATCGGGCCTAATTGACCTCAGGAATCTGATATACTCGTCACTGTGAAACTTTGTCATTTCATCAGCAGTGGCTTTGTGCGGACGGTATATTTCCATTTTACGATAAAGTCCATAATTTAAGAGTAAATTGTGTGTCATCCTTATGCGATGCGGTTTCATGGGATGGCCCTGCCCATAGTAATAATTGCCTATGTCACTGTCGTAGTAGTAGCAAACGCGTTTTCTGCTGTGCTGTAAAGTAGACATGTTTGTGTTTCTTTTTTATTTATTTAAATATATTTATTGAAAATTAAACGCGCGGTGTTCAACACTAGtCACACTAGCCCacGACCCGACCGAACAC

>contig01001

CAAGTTTtCAGCGTGGCTGGTTACATTGCAAATTGTGCACACTATGAAGCTGCTCTCCACGAGAACTTCGGTATCCACAGAAGTGTTCTGGTCGTTGCATTTGTCAGCGACACATGTGATACAAATTGTATGAAGGCACTCTAGCAGTTTGAGGTCAtCGGTGGAGGCGAAGGTCTTGTTGCAAAAGACACATTTGGAAAGAAaTGCGCGAATGTCGCTTtCATGGCTTTCCGTTGTGCACTCGATTTGTTGTTCATGGTCTTCCGCGTTGTtATAATTACTTTGTTGTTGGGATTCCTGCACATTTTGTTCGGTCACTGATTGTAATtGATCCCCGATTTCTTCCTTCACTGAAACTCCCGGTCCAGGAGTtAAGCTTTCACCTCCACATGCGTCCATTTTCCGCCGTGAAAATGAAAATTTAATTTATTTTtCTCATCGACTCTCAAAAACACAACAGGCATCAAGCGCCCTGAATCGCgCaCaCtCCcATgT

>contig01002

ACGTTTTTCTGGTGCATGCTGTTGGC

>contig01003

GCGAATTTGATCCCGACGATATAGTTTCGCCAAAGACTTCGCCAAAAAAGAATGTTTCGAAAACCGCACATCGTTTTTTGAAACCTTCCCTCAAATCTATTCGGAAAGTTGAAGTGAAAAAAGCACCAAGTTTCATGGCTTCGCTCTCGACTTCTGTTCCGATGTCGTCTAGTCATCCTGAGGCTAGAGTGTATAAACAGAATTTTAAAAAGTCCAAAGAGGCATTGTGCAAACAACTTTtCTCTATATTCAATGAGAAAGTATtCGAAAaGAAACTtCCGGACGATATGCTGATAGAATGGAGCACACGGATGACAGGCACCGCTGGCTTTtGTTATAaCAAAAaGtCTATCAGAGCTCTGGGCAGCGTtaCGAGATCGAGtAGAATTGTGTtAGCAACTAAGATTtGtGATAATCCTGAAAGAGTAAGGgATACTCTAGTGCACGAAATGTGTCATGCTGCCACATGGATAATAAACGAAATTTCAGAGGGCcACGGACCTCTGTGGAAGGGaTGGGCAGCAAAGGCAATGAATGCTTTTCCAGAAATACCGCATATAAGTCGATGCCACGACTACCAGATTAAAaCGAAaTATACTTATAAGTGCACAACTTGCGGAtATTGCATTGGAAGGCACTCAAAGTCGCTGGACATACAGAAGAAACGCTGTGGATATTGTTACGGAAAATTTGAGGTATTCGTCAACCGAACAACAAAATC

>contig01004

ACagAATCCACGTGTGCAATTTCTGCGGATTAATCGCAATTGCCAACTTGAGAAACAACACTTTCGAGTGCAGAGGCTGCAAGAACAAGACGCAAATCTCTCAAGTTAGACTTCCTTATGCTGCAAAACTCCTTTTCCAGGAGCTAATGGCAATGAACATCGCTCCCCGTCTCATGGTAATTTAAGCTATTTATTTATAGTTTTGATCACATCTATTTTTAAGGTAAAGATCAAACTCCTATGGTCTCTTTCCATACTTAATTTtGTTGTGATAAGAGTCTTTTTACG

>contig01005

ACGTAAATTACTTAAAATTCGACGAGAACGAAAATAaTtgCCAATtCGTGTCTCTCTCTTGATTTTtCCtCTCAAACAAGAAGGAGAagAAAACTTGACGCAGAATTtAATGGCGAaTCcACaGGGCAGTAAAAaCGgATCCATCTCCCCcTCTCCAAATCAAaCTCAAaTTAAATTCAaTTAAAaTCAACTTCATTCAAACACTCGATaTtAAAAACAaCCAAAAAAaaTaGCTTtaCaCCTGAAAAaGtgCGGATGTCTAGTTGAaTTAAAaTAAaTCCTCGAAATTCTCTTACTCTCAAGTCCATACACTAACAACTACTCTGTGGaGGAAAAAAatATCAAATCATATGCTCGaGAACGCTTtGCGAAACCTACACTACGATTTATTTTATTTtATTTTtCAATTTtAtACACAAAAAaCaCTTACGAAGAAGATGaTGAtGAAGAAGAAGAAGAaGAAGAAGAAGAAGAAGAAGAAgAaaaCATTTCTCTACTGGGGCGACAATTTCTTGCATGGCGAAGAAGAAGGCTCCTCAGCCTGATTTTCTTCAGGCTtGCGTTTTTTCCGGATCAAATGGGAAATGTCGCTGACGGGTTTCGAGCTCGAAGAACCGGAAGAAGTTCCGAAAGCCGAAGAGGAAGAAGAaGCTGAAGAAGAAGATGAtAAGCCGAAAGCTCCCGCCAAAGAAGACGCGCCCGAAGAAGATGGCCCATTGCT

>contig01006

ACCTTTGCGAACTTCAGGAACTTTTAAATACAGTGGGCGTCAGTTTAAGGGATGTCGAGCAGGCGATCAACAGTTTAAATCCACCTCCGGGTCCGTTTAACTTGGCAAGCACTTCTTTTCTTGGTCAGGAAACAACCCAAGAAAGGCAGGCTCTTTATAGCTCTTTAATTAATAGTCATAAATGGACAGACAAAGTTCATGAATACAGTAACATTGCGCAGTCTTTGCTGAGTCAAAATAaTCTAAAaCGCTCCTATACAAaTGCGAGCAGAGGAAAAAGaGGCaGAaTTCAGtCCAGCTCACACAATTTATCACCACCGCAAGTTGACAATGTTATTGGaCAATTTGATCGACTTTTTCCTGACATGACGTTCACCATCTCACGACCATTTGCATCCAATGCGGTTCTTCAAaTAACCCTGGGTCACGTGTTGAAAGCAGTGGTTGCTTTCAAAGGTATAATGGTCGAATGGATAGTAGTGAAaGGTTATAATGAGTCTATGGATCTCTGGACTGAATCTCGATAC

>contig01007

CTCATGCTGCCATGCTACATTTTTACTCGCCTGCTCTTCCCGAATTGGCTTTAAGATCCTTGATGACTTGGTTCCACAGTTTGATCAGCTTGTTCAACGACCCATGCAAGCGGTGTGGCTTATATTTGCACAGTGCTTTGCCaCCAACTTGGAGGGATTTTCGT

>contig01008

TTCGAAACGCCCGAAGCCGCAGGCAGAATAGTCAAATTAAAGCACTTTCAGAAGTTTGCTGACACTACCGAAGCCTTAGCAGCAACAACCGCCGCAGTCGAGGGAAAACTCAGTAAATCCCTGAAGAAGCTTTTAAAAAAGCACTGCTCCGAAGTCCATGAGCAACTTGCCGTTGCAGATGCGAAGCTAGGAAACGCTATAAAAGACAAATTGAGTCTATCATGCATCAGCAACACATCAaTCCAAGAaCTGATGCGATGCATCAGAAGCCAAATGGACAGTCTCTTGGCTGGTTTCCCCAAGAAGGAAATGACTgCAATGGCTCTTGGTTTGGCCCAcAGTCTCTCCAGGTATAAACTCAAGTTCTCCCCTGACAAAATCGACACCATGATCATCCAGGCAGTATGTCTCCTCGACGACCTGGATAAAGAATTGAACAACTATGTAATGCGCTGCCGCGAATGGTATGGATGGCACTTCCCcGAGTTGGGAAAGCTTATCTCCGACAATATCTCCTTCGTTAAAACTGTCAAGATCATCGGCACAAGGGAGAACACATCTTCCTGCGATTTATCTGACATTTTGCCCGAAGAAGTCGAGGAGAAAGTGAAGGAAGCTGCAGAGATTTCCATGGGAACTGAAATCTCAAACGATGATATCATCAACATCCAACACTTGTGCGATCAAGTGATAGAGATCTCACAATACAGGACGCAAT

>contig01009

TAATTgggtagCCGGCAATCATGACAACCCAAGATTAGTGTCTCGAGTTGGCGTCCATCGAGCGATGGCAATCACAGCAATGGTGCTTCTGCTTCCTGGAGTTTCTTCGACATATTATGGTGAAGAAATCGGAATGATCGAtGCACCTGTTGTCTCCGATGGTCTAGCGGACGTTAGAGATCCAGAAAGGTCTCCAaTGCAatGGGaTAaCTCCACTTCAGCCGGATTTtCAACTAATTCAAAGACATGGCTTGCGGTCAACCAAAaCTACAAAACTCTTAATTTAGAGGCTGAAAAAGTTTTTGACGAATCATATTACAATTACTACAAAGCTCTGGCTACTCTCAGAAATTTGCCAGCTGTGCGATCTGGTAACTTAAAGTTGGAACTGCTCGACAACGACGTATTAGCATTTTC

>contig01010

TTTGAGGAGCGAAAGGAGGAACTTGGTTTAGAGGATTTGGATTATATGAATCTTTGCAATAGTTCTTCAGATTTGGAGAAGTGAAAGGAGGAACATGTGTCAAAAGAATTGGATTGAACAAAAAGCTGCCATAGTTATCCAGATTTTGAATAGCTGTATTCTCGGATAATTTAGTCCTTTTGTTTTTGATGGTGGAATCGTTTTGCTCATCTTCATATCCTCTTTTTCGAATTTTTGGCGTCTGAGTAGAATTAATATCCAACTTGACGAaGGTTCTTGCGAAGTTGTTTTCATCTAAAGACTGGTCTCGTTTGCATTCTGCAAAATTCATGTCCGCTtCCTTTTCTGCTTTGCTTCCAAGTTCGTTTTGCTTATGCTTGTTTCTCGATAATACTCCCTGCCTTGCCTGACTCAAAATATAGTCGACATATTCATGAtAACCGTGACTATTAAATAAAGCAATCTCGCAGTTTATTGGGTTACACTGTTGTATAGAATATGCTAGAACTGCaCTGTAGATTTCTTCTGGGAGTTTCATATATTTCCTCGTATTATGACGAATCTTCcTtCCACCAATACTCATATATCCCATGTTTTCAGTTCCCAAAAGATCTTCAAACAATAATCGTATCATTGTTTTATGTTTTAAAAAACAAGGTAGGCTTATGCATTCCCTGTATATTGTTTCTAATTCGCTTCTTGATATCCAGACATTAGAT

>contig01011

ACCGAAAATATATATTTTTAGAAACCTCAAGTTTCTTAAATTGAAAGGAATGTTGCTTGCTCTCAGAAAGAATTATTATATAGGTATATAGCACATATAGCTTAAAATTATCTAATACGCTCCACATTTTTTTATGCGGATTTATCTTCCCCGATCACAACCTCCAGGACAGCCAGTATTTCATTTTttGAtGATTTTttGtGGTTTTTTTttCAAAaGTATATCGTCCAGCCTATGCAAAAaTTCAACCAAAaTAGGCGCCGCAGTGAGaGCAACGTCtaTTTTTtAATAAAaGGCAACAGATGACTCCCAACGGAAAGAaGAAAaTTGCACATAAAATTCCCAGGCACGTGTAATCATCTTCCAGAATTCCCACTCGGCATGCAGGACAGGCCCCAACGAGGATAATTTCAGGAACGATAATAGTCGTAGAAGGTTCTTGTTGATACGAAATAGCAgaGCTCTGGTAATATCCGGGAGGCGGTTGCCACGAaGACCCTTGTGGTGTAGAAGGTGTATAAGGCGGTGGCTTTTCAAGTTCCGGGAAAAGCTGGCGCTTTTCCGAaGCcATTtCCTTTAAATTATTGTTTTTCAGACTGACAGAAACACAAAGGATGAAGCAATTTGACCAAATTTAATTAACCGCAGCTGATCGTTAGCAGACGGACGAGTTCTCCGTGAGTCAGACTCCACGGTcACAACaCaCaCacaGAAAAGT

>contig01012

TTACTCAAATCTATTAAAGGAATCGCAACTTTGATATCATCAAGtGAACTCGATTTTTTCACGTCGAAAATGTGCTCTTCCAGGGGCAGAGAAAAGACCATTGCTGGATCTTCTGTCGAGGCAAAAAGAAGAGTCAAcTTCGGTCCGAAACAAGCGGCTGCAACACGACCATTTGGAACTGTCCATTTTTCTGATACCCAGGGTGATCCgcTaCCAATGTTCCAAACCCTGAAGACTTT

>contig01013

TTTTCAAAGGGACTCCAGTTTCTTtCGAGACGTCCCAG

>contig01014

aGTGTCTGCTCTACTGGCTGCTACATTGCCAGCCTGCTATTCTACTCGTTCTACTCCCACGAAATTATACGAATACGAAATTGAGAGTGTTTCTCTTTCCTTTTCGACGTGAGTGACGTTTTCAAAAGAACACTTGCCTCTTTCCCAACAGTGAAAAATCTCCTCTTCAAGTTCATTCACAGCGATAACCTACAAAACAGTGACACTGTGTCAAATTGTGAATAGTGAAAGCGACTCGTGTGTGTAAATTCAGTTTtCTCACTCAAATATCCCGATCTCTCTTTTTtCCAATTTTCAAAAATCCTTTGTGTTTGGCAGATATTTCTCAAATTTCAGGTTCTGAGATCGAGAATTTATTCAAAAAAGGCAGTTCGTGTGTGCTTTTTTtATAGATATATGTGAATTTTGTGAAGTTAAAAGTGTCAAAaGTGGAAATAaGAATCAACATAACCTAAGAATTAGTGATCAGTGAT

>contig01015

AaTCTGGgACCGGgTGGCGGtAGAGGACCAGGAGACATCGGATGTGGAGGTAaTCTAGGTCCGAaTCCcGGTtGAGGTGGtGGACCAAAaCCAAATCGCGGTGGGGggAATGGCACGCCGTTtGCGAAACCACGTCGAGGTGGCCCATATCCCGGTGGACCcATCATTGGACCAAACGGAGGATGTGGTGGAGGTGGCCTTCCtGCCGGAAGCCTTCCTGCCGGAGGCCTTCCTGTCGGAGGCTTTATTGCCGGA

>contig01016

ACAGATAATTTCTGAAaTAATCCTATAAAaTATAGCTCTAGAGACGCATTTCTTCGAAATCGTAGACCCTCTGACAATAAATGCAAATAAGTGCTTATCAGGTTCTCAaCTATTCTCTGCTCGCATTTCTaCAAATAATAAATCTaTAAAAAAAAaCaGTTTATATTAAGGATATATCTtGACTTTTTTtCAGCTCGTCATGaGTCTAAAAAAAAaTATATATCGAcAATTTCACTCTCAGGACATAATCAATCATACTtCAaTAACAAAGTTGTTCAAGTGGAAAAaTGGAATTAATATTTAGATTTCCCTCGTAAAaCTGGCTTCAAACTGGGAATGTCATCATAAACAAGTCATGCCGCAAGGCCCTTCTTCAAGTTCTCTGCCAATTTGTCCATGAATTCAAAGGTTTCCAGGtAATCGCTTCTCTTGACATTGTTCATTCCCTTGATGCAAATTGCGAGGTCCTTCGTCATGCTTCCAGACTCGATTGTGCTGATACaGACCTTCTCCAGGGTTTCTGAAAAGTTCCGTAGTTTGTCGTTGTTGTCGAGCTTGGCTCGGTGGAGTAGACCTCTCGTCCAGGCAAAGATCGAGGCgaTtGGGTTGGTCGAAGTTTCTTTTCCTTGTTGATACTGACGATAGTGACGAGTcaC

>contig01017

ACTTGATCCTGGCTGGCGCGAATCCCGCGgCAAGAAATTTgAGCGGAAATACGGCCCTTCACCTCGCATGTGCTTGTGGAGATCTTGCCTGTGCAAAGGCTCTCACAGATCCACTTTCTCCAGGAGAAAGAAATCTCTTTCCACCAGGAAAACTCGTCCCAGCTCTCCCGCAAAACTTGGAGCAAAGAAATTATAACGGTGAAATGTGTATTCATATAGCAGCCGCTGGTGATCATGTGGAACTGGTtAGGCTTTtGCTTCgCCTTGGtGCCGATTTGGACGGAAGaGAAGGTCTTGCCGGCAGAACAGCGTTGCACTTGTCaGTCGAGTATCGCTCTAAATCcGTGTTGAACTTCTTGCTGAACGAGTGTAGGCCACATTTGGATGTAACGAATTACGCTGGCATTACCCcATATCAAATTGCCCTCTGCGTGGACAATCAAATGGCCATGGACCTCGCGAATTTAGGCGCCAATACGGAGCCACTTTCAGAGACTGACGAATCAGAAAGCGAAGAAGaTTCCGAGAGCGAGGAGGaGGaGgCTTtCGAACACAAATTATCATCACGATTCAGTAAGACCAGCATATCCGCTGTTACaGCTTAAAGATTTtCCTTTTtAGAATTGTATTCAATGCGAAAGCtGGGAACaGTTCTCATTATCACAGGGATTTTtAGAAAAaTTGAAAaTTTAGACTAGTAGAGACAaGT

>contig01018

CTATTTCAGTTACTTCCTTCGATTTAAGAACTCCAGAGTTTGGTCTTACGCAATACCTTTTTGGAGCAGTTGTCTTTATTTtAAAATATACTTTGTGATTTGTGGGATTAGTGAGCTTCATGTAAGACGTAAC

>contig01019

CCATCCGATGCGCTGAGTTGTGCTTGGCGTGCGATGTCATGCATTTTTGTaTtGTTgaTCTTAGTGAAAGtCTGAATAGTTTTtCCtGCCATAATCCAAGCATTCCTAATGTGGATTTCCATTTtGcACCACTGTTGATACAACTTCCAAGAATTAGATGCATCcTtGTTTGCACCTTGGTtAaTCTGCGAGTCCTTCCAGAGCAAGAAGGCCcACAACTGCGCTTTTTGGTcATTATTAGTGTTTTcTCGAAATTGAGAAACTTTAGAACAGGCTCCTTCCCTGGTCCGATGATCCTGAAATTCTTCGATGAAGTGAAAAGCAGAGTGGCATTGTCCACAGACTAAAACATCTAGTCTTCCCATGTCTTCTTGTGCAGCTTTGATTGTTTTCGGATTGATATGAATGGCTGAAGGGCCACcgCCTTCTGCCATTGTGTT

>contig01020

TAACTCCCGCACATTCGATTCTCATtATTGCCACTTGATCTtGTTTGACGAACCTTGGTCTCGTCTTACTTtGTCGCCAGTTTtCTTGTCTACcAAaCAAATGAGTGCCCTCACAGTTACTTCCTCAGCGGCACAATGTATGTGCATCACAGCACTGTATCCTGCACAGATAATGCTCTtATGTTCTAAGaTtACTACTtGTGCATCGAAAACTTTTCCcGTCTTGATTGGATTGTTACTATCGCAGAGGACGAAGCCGGGACTCACATCTTCTTCTTCTATGCCCTTTAATTTTATTTTTACATTCTCTCCTGGTCCCACCGAAGTGACTTCTTCATCGTCAGACCACAATTGATCAACCGTTACAGCCGTCCTATTCGGCATTACAAGAAGAGACTGTCCTTTCTTTGCTTCTCCAGCCTCAACTTTGCCCATGACTACTGTTCCCATATCCT

>contig01021

TTACTTCAGACCAAGTCGCGAACAtCTGAAGCAATTTaCACGAAATTaTTAGAGCCTAAAAATCAATCTCCAATATGaGATCAGCCAGTAGTTATATTTATAGACACATGAAAAAAaatATATAAATAGTTTGC

>contig01022

ACCTTTTCAGTGCTTCCACTTACTATTACTGAACCAGAAGGGTTCATAGCCAAACTGTATATGGAATCCTTGTTTCCGGAAAGGGAAGAAGTCGTTACGGTGTTATTACTCGCTGTTAAAGCTGTGAGCGTATTTACATCCCATAGAAAAATGGCTCTATCCAAACCTGCACTCGCCACTTGTTCTTTATCTTTTGCATAGGCTAATGACTTAACATAATCTTTGtGCGTTCTTAACGTGGAcATGCAAAATCCTTtATGCGCACTCCAAACTTTTACTGTAGTATCTGAACTTGCAGATATGAGGTTTTTGCCTCCACAACaTAAAaCAATATCATTGACCCAGTCTGTAtGATGTTCCAGTGATTGAATGTAGGGATCATTAAtATTTGCGCAGTTCCACATCCTAATAATACTGTCCCTGCCTGCTGAATATAACCTGTGCAGGATAGGGTCATATTGAAGGGAaTtcACACCAGCTCTGTGCCTCCTTTCTATTTCGTCTCTTACAACCATCGAAACCTGCACTTTCTTTCGGGCACTTTGCCCACCTGTCTTGTGAGCTGCCATTATGTTGTAAATCTTTTCTGTTTTGCAAAaTCCAGGAAAACATTCGAAAaCTAGCTGATCTTtGCAAAGCTTGCAAACGCTtCAAGTATGTTCATTTTtGAAGAACtCACATTATATGTAAATATATTTGGAATTTTTCGA

>contig01023

AGGATTTGCCTAAAAGGgatGATTTCTGGACGTGCTGAATATTAGTAAAGCATACAATAAAATTTATTtATtACCTTATTTTTGtAAaTTTATTAATTATTAATCAGGAAGTTCATTTGAAGGGgCCTAAaCCTAAaCGAAaTGGCAACCcTAGAAGaTAAaTCCATTTGGGAAGaTGGTGAGGAAAGTCTTGGAGAGGaTGTTTTGCGAATGTCAACAGATGAAATTGTGTCGCGAACGCGATtGTTAGATAACGAAATAAAAATCATGAAGAGTGAAGTAATGAGAaTATCTCaCGAATTACAAGCACAAAaTGATAAGATAAAAGAGAATaCAGAAAAGATCAAAGTTAATAAGACCCTTCCGTATTTGGTTTCCAAtGTTATAGAActATTAGaCGTTGACCCACAGgACAtGGGAGAAGAGGATGGGgCAGTTGTCGACTTAGATgCTCAGCGAAAaGGCAAGTGTGCTGTGATTAAAaCTTCAACACGACAAaCATACTTTTTGCCTGTAATAGGACTCGTTGATGCTGAATCCTTAAAACCAGGAGACCTAGTAGGCGTGAATAAAGACAGTTATCTTGTTCTGGAAACaCTACCCGCCGAGTATGATGCCAGAGTCAAGGCGATGGAAGTAGACGAACGACCTACTGAACAGTATTCTGATATCGGAGGGTTGGACAAGCAAATTCAAGAACTtATtgAaGcaG

>contig01024

GCATACGAGTTTATCATTTTTGTCGTAAACTTCGACACTCATTGTTGATGGATTTCCGCCAGTCAACAACTCGAGTTT

>contig01025

GCtACGCATTCGTGTTGATAGCTGGCTTGGaTGACCTTgATTTCaGGAAGGCAGATTTGTTTATAATTTACGTtGCGTGTTGTGTCCGCaATCGAaGAAATTCTGTATTCAGAAAATAAATTCCTCGATGGATCGACTCTAAAAGTTTTAAACTCTACAGGCATCAAATTTGCTTCTGCAtGGTTAGTATTCTGGCGGCTTCAAATTTAAACGATTTTAcGAAAATTCATTGGGAATTGAACTGAGAGACTTTTAAAAaTGATGGAAGATCAAAaTCTGAAAGAGGAAGCAGAATATAAAGAAAGGGAGATTTCGGAGATTGAAAATTTAACTGATGATTGTCTTCtcGAAATTTTCAAGCGTCTGCCAACGATAGCTGACAAAaTTCGTATAGAAAGAGTTTGCAAAAAGTGGAAAAATTTGAGCCAAAGGTCGTGGACTGATTTtAAaTCTCTCGATTTCTTAGCCAAAaCTTGGGAAATTGAAGAATATCGTCGGTTTCCACAATCAATAAGAAGCGTCGATTTGGAT

>contig01026

AGTCAAGCTCGAgaCACCGTCGTGAAAGGTTCGTTCGAATCTCT

>contig01027

ATTTTATAAATTCCAGATTCTCAAGAATGGCTGAAGTGGGGACAAAAAAaTCCATGAATGTCGTAAGTAAAAaGAATAaCGAAGTGTGCAGTGTTCGTtCTAACAACAGTTTGATTGATTTGGATAATATAAGCACGAGTAaCAACCAACTAATTTCACTCTGCGACGACGAAGAGGACGATGACGAGCTtAtC

>contig01028

ACGCTAAAGTTTGCAGTTGCGTTGAAAGCAAAaGaTCTTCCATGTGAGCTGGCTGCGAGAAGGAGAAaCCGGGCCGTTCTTCCATCGTAAAGGTCGACAAGCCATTGGTTTCTTTGCCTGGCAATTCAGGCTGAGACAAACATGTTCTCATTAATACTTCCTCCTCGtCAATATGAAATCTTTTTGCTCGAAAaCCGTCAACAACATCCCGCTCGACGGGTTTtCCATCTTCGGAGAAATTGTTGCGGAACcACGCGTGCTTCTTAATCTCGgCGATCTTGAACCTGcGAGAAGGTTCATGAACGAGAATTTGTTTAATCAaGGAAAGTGAAaTTtATCCAGTTTCCTCCAGGGTGTCACCGACATACACTTTCCATTTTTCCACGCTTCATATTCTGGACACTCTGCCGAGGCTTGATCCCAAGGCAGTTCTCCGGCCAGCAGAGCAACAAGGATAATTCCGCAAGACCAAATATCTGCAGGTTCGGCTTGATATGCCTGTTCGAGAaCTTCCGGTGCCACATAAGGTAAAGTtCCACACCTCGTATCGAGAGAACGCTCTTTCCCTTGTAACCTATACACAGTCGCAAGACCAAAATCAGAGACTTTCAAATTATCGTTTTCATCCAAAAGTAAATTTTCTGGCTTCAAGTCTCGATGGGCAACACCTCTACTGTGCAGATATTCCACTGCAGAGATTAATTGCCTAAAGT

>contig01029

ACTGCGCCTTTTTCAGTTCCGTTAAAAATTTATGTCGAGTTTCCAGTCTACTTTTACG

>contig01030

GTAGGTAATAAGCTTCGGCAATTTAACTGCAAGTAATCGAACTtCAGGACCCTTTAaTtAAAaTTGCTTtGAAAGTATCCAAGTCTGGTGTCAATTGaCTTTGTGCTATGCGAGATCCACTGTGGATTTTTCGTCAGGATCGTCGATATCATAAGCGGATTAAACTGATGTGCCCTCAaGTAT

>contig01031

TATGCGCAACAATAAACTAAAAGATTTGAGAGCTGAGTGGATGGACAGTAGATTTTtCTTTGGAAAAAaCAAAaTAATGGCTCTAGCTTTTGGCAAATCAGAAGAATTAGAAGTTTCTGAAGGTGCTCACAAGTTGGCAAACGCACTCAAAGGACAGTGTGGTTTGCTTTtCACGAATAGACCTAAAAAGAAGGTATTAGATTGGATGAAAGAGTATGGAgAAGAAGATTTCGCTCGTTCTGGTTTCGTTACCGAaGAAaCCATCGTTTTACCAGAAGGTCCAATGCCAGATTTTCCACACAGTAtaGAACCACACTTGAGGCAATTAGGAATGCCAACATCCTTGCAGAAAGGAGTCGTTACTCTTGTAAAAGAaTACGAAGTCT

>contig01032

CTGTCCTGATGAGGCATGACAATATTCTCGGATTCATCGCTGCtGATATCAAAGGAACCGGATCTTGGACTCAGATGTTGCTGATCACGGATTACCATGAACGA

>contig01033

AACCCGCGATTGCCCATCGCGATATTAAGACGAGGAATATTCTTGTGAAGAGGAACGGGGAGTGCGCGATCGCCGATTTTGGCCTCGCTGTGCGGT

>contig01034

ACCCATTGAATGAgCAATATATATCAAACtCTtATTTTTCTTTTTtGTTACGTAAGAtATCATCGCTGGCAAATCgTGAAGGCCcACcTCGTGAAAaCTAAaGTCCCAAAATCTTGAATCTGTTATGGGAATAGTTTTGTGGGATCTGGAaTAaGTGTTTCCACGCATATTtCCcAACCAGACATCaTATCCtaaatccgaaaggatgaaagcGAGGGCTTtGCCCcTTCCAGTCATAACCCAATCAGCAGAACTGCCcAGCaagCCGTGTTGCAGAAATGCAGGGGTTGATCCTtCGGgTCCAGGAATACGATGCATTGTTAaTAAAAAaCCATCTTCCGTCTCTACAAGATGTGCCTCGGCCGGATATCCAGCTTGTCTTATCAATCCAGGCGTATCTAAAaTAACAGCGGGATCAACACACAATAATTGTCCAGCGCAGAATCCGGCGACCAATAAAaTAGTTATTAAATAACTATTCATTTTtCCGCTTCTTtCCTTCCAATAATTATTCTCGTTACAACTCTGTAAATCGAGGCTTGGTTCACTTTCAAAAATTGACAGAATTTCTAAAAAAAAaTTATTTtAATAACTTTTCATTAAAAACTGTTTTAAAaTTTAAGAAATAGGAAAATTTTTTATCATTTGGTTCGAGAATGAACGTTCTGGTCTGAGACCGCGAAGAAACT

>contig01035

GGAAACCGCGGATCTGTTCGGCATAGGTGTTTTTGTTGTGTTTTTGTCGTGGTTTT

>contig01036

AATCGCAATGAGTGACGAAGAAGAAGAAGAATCAGGATCCGAGACTGGATCAGAAAaCGTGAATTCAATGGATGGAGAAGCAAATATGGAAAGTGCAGACGAAGGAATGGACAATAGTTCTGC

>contig01037

GtAAGAGtCtGGAGAATCTTTCTGCAGCCAAAGAGaTTGTAGCTCAAAAAAAAaGAGGAGTCGAAAAATCGTCCATCGACTCGAGCTGGCCTTCGTCGAGGTCTTAAACGCAAGTGCAGATTTTCTATGCCCTGCAGCTGATTGGAAaGAaTTGAATGATTTTTGATGAGTGTGCAATCTTAATTTCGATGGAGATATCGATGCCTTATGACTAGATTCTGTATAAATaTTCCTGTAAaTTATAAAACGAGGATGTTGTGCCGAACTTTTGCTTTTTTTATAGGtCAGTGGTCGATATATACATTTTTCTTtGtATGTGTGTGTG

>contig01038

ATCCGTAATTATGTATTtGTTGATAGCGTAAGTGGAGGAACTAACTATAACTAAAGTTGCCGCATTTGCAaTGAGACGTGGCATGAGCCCTGC

>contig01039

GTtAAAtaCTTtAAACGGTTTTTtGCTTTCTCGACTtcGCCGTTAGCTTTTCCGCCATCAGCTTCTtCTTCTTGTCGAATTTGCTGCCTTTCTGCTTCTTCTTCTTGTCTGCTTTCTGCCCTTGGAAAGACTCATTTTGCGGACTTTtACCCTCAACCTTCACCCTCTGCACTTCGCCCTGAGAACGTTTCTTGTTCTTCTTTTGCTTTCGGAATTCGGCGTTCTTACTGTTGTGGGTAGCGACTGCAACGGCATCGTCACCATCCTTCAGTTTTTTCGGTGATTGAGATTTCTCGGAACCTGTCGACAACGACCTTTTCCTTCCCTTGTTGATTCTTTGCTTCTTTTTGCCGTCGTTTTTGATGGGAGAAACCcTAATTTCACGATTTCGGATTTTAGAACCATTGAGTTCCAGGGCAAGAGCTGCTGCGTCTGCAGATTTGAAATTGACGTAGCCGATTCCTTTGTTCATTCCAGATTTGCGATCTTTCACGAGTCGAACGGACTCGATCGGTCCACATTCGCCGAAATGATTCCACAGGGTGTTCTCGTCACACTCGAAAGGCAAATTTCCCAGGAAAACTCCCTTCTTCGGATCAATTTCCCCCTGTTGATAAGCAGAATCAACCCTCAAAACATTATTCTCAAACATCTTTCCATTCATCGACAACGCTTTCTTCGCAGACTCCtCTTCTTTATAAGTGACGT

>contig01040

TCTATGTgaTtCaCTGGAAaCAATAACAACTCT

>contig01041

ACTTTTCAATCCTTTTTCCACAAGCAATAATATCGTCAATCTGATGTTCAAGAGTCAGCATGGCTTGAGGTTCAAGTTCATGAGTATCCAGGTAGCCAGCTTGGATCAACCTGTAAGCaTTATAATCCCTCATTTCTTTTGAACATAATTGACCTGACGATTTCTTCATTTCTTCTTCCGGTTTGACTGTCTTTtCtGGAAGTGAAGAACCCAGTGGGTGTTTCATGCTGATCGTCACGATTTCATTATTAATATGCATAATATGTAATCGCTCATAATTGTTGATGAGAGACGCAACGGATTCTTCGGCTGAAGAATCTCGTTCATCCACTTCTTTCTTCTGATCTCGAGGAATCTCTAAGGTTGTTAGCTCTGTCTTTGAAGACTTCTGGATTAATTGTTTATTATCACCCATTGTAGGGAGAAGTTGAGCAACAACTCGCTTCACCGAATGGACTCCTCCCATTTTGTAATACTTATATTACCTTTTCGTCACTGGCACGTATCTGCTGACTTTTGCGGGAAACGTGTCTATAATCTTAAATAAGTTCGCTCTTCTTGAATAATTTTAATATTTAAGAAATCAATACTTTTTATTAAAAAACTGTTTGAAAATTATAATTATTCTGTAATGGAGTAATAACACTAGCTCTACGACGAAAATCCTTTACCTTCTTGTGTTCCTGACTCACTGTCaCT

>contig01042

AcTTTaTTtAGTAAAATTGCGAAATGTGTGTTAAGTCAGAAAAATGTTAAATTAGAGATTGTATTTTGAATTAGACCCTTAGGAATTAAACGAAAAAACTACAAAACGAAAGGCATTTAATTGTATTCAACCATCATTTCATTCACATATGTTGAaGgAACGTGCCTGCCGCGAATTTtACTGCAAATATTTGCCTTGACTCTTCAaTTTTCTATAAATATCTTGAAATtcGTGAGATTTAAATTTGGAGACTCAAGATAGAAGtGTGCCATATTGAGCTTAAAAAGCAGTATTCAAGCTCaTTCTGAaGCTATTTTAGCAGtGAAGTGGAGGTGAGCAGTCAATACTCGTAAaCAACATGCTTTTTtGAAATATTAAAaTTCaCTGCGAAaCGGACTATTCATCCACAGTAATAAAaTGGGGTCTATCTTATCCAGGTTTCGTAAAAaGAAAaCAACGATAGAAGTTCTGGAGgaCCTTGATAAGAAAaTCAaGGATATTGAAAAGTATGGGCTTTTTACGGAACAAAGGCATAAGAAGATCGTTGGCACTTTGATAGCATACAGTGTCATAATGTATATAATAGCAGCATTAACATGCTATTTCTACTTTTTCCCCACCTCGCCATACGACCAATTAATTTACGTCACACCATTGCTCATCTTTCCAATATTAATCCTATTTGTGAAGAGATTGGTGTCATGGT

>contig01043

ACTTTCGAAAGGTCCAAGAAAaTCCAGAAACTGcTCCCAAAATTGATTGGGCCCACTATAAGAAAGTTATTTCTGTTCCGGGAATGGTAGATTCATTCCAAAAACAATATGAATCAATCCAGATTCCCTATCCCGCTGATAaTTATACTGCAAAAGTAGAAGCCCAGGAAAAaGAAGCGCTCGTTGaCATCGAGAAGTTCATTCAAGAGTCTAATAAAGAGATTGAGAAAGCCAAAGgTGAAATAACTAGAATTCAGGGCCTGCTCCCAATCAATGAGATGACTATGGAAGACTTCAAAGATTCTTATCCTGAATATGCACTAGACCCTCTTAACAACCCAACACTTTAtCCTCATGATCCCGATAGTCAACCTGGTTCCGAAAAaGAAGAAGAAAAGTCAAGTCACTAAGCATATTCTATTCAGCGAGCACGGTAGTCTG

>contig01044

CAATGTGTAGAAGTAGTTCCACTTTGGATTAATGGAGCGATTTCGTCCGAATTACTTTCAGACAGTCTTCTACTTGAACTTCTCTG

>contig01045

CTCGCACACGGAAAAGGCGCTCAAGTTTGTGTCGGACGCTCTGGCTGAAGGTGGCAAGAAACCAGTTGTTGGTGCTGCTGCTGCTGCTAATAATACGGCGGCTGTGCAACCACCAGCTGCTGCTGCTGCTGCGCCTGTTGTTGCTCAGGCGAATGCTgATGGCGGCGTCAAgAaGGAGGGTgACGACAAGAaGAaGAATGCTGCTGCCGcGGCGGCGGCGGCTGCTGCTGCTGCTGCGATTCTGCCACAGAAGGAGGATGGAAGCGATGGGAATTTGTTCTCGTTCCAGAAGGAGAAGGAGGACGCGAGGATTCTTCGTGGAGTTATGCGAGTTGGAAATCTTGCGAAAGGTCTTCTTCTCTCGGGCGACAATCatGTCTGTCTTGTGGTTCTCTGTGCTGAGAAACCGACTAGGACTCTCTTGAACAAAGTTGCAGAGATTCTGCCAGCTCAACTGAAAATTGTTGCTCCCGAAGATACGT

>contig01046

ACCGCTCCGGAAATCGCACGCCTGCGTGCTCTTTCTCCTGCCTTGCGCACCTTTTCTGCTGTTGTTTCATCTCCATCGTAGTGAACGGCAGCTAACTTTGGTGGCACGTAGACTCCAGATTTTCTCGGCTTGGAAGCATCTTCGTCGGCATCGTTGTCTGAATCATCTGAGCTTCCATCGACTTTGCCAATGAGTGCATCGGGATTTGCTTtAAAaTTGCTGGgATCGCTGCTGTTTGTAGTCCCAGTAACTGCAGTTTtCACCAGCTTATCTATCTGATACTTGAGTTTGTGGTCGATCGGCCTGATTCTCTCCAAAaCAGTTCTGATTTCGATCAAGCGATCTATCGATGGATCCCCCTCAATTCGTTCTCCGGAACACTTTCTCAATACGATGTAAGTTAAATTGATAAGGTAGGAAAGTAGCATGTGGTATTTCATCTCTAAGAAACTAAGTCCCTTATCTGTCGAAATTtCCCCTTTTTTCACGCGATCTAGCATGTTATCGACAAGCTGGTTCACCTGCAGGACATTTGCATTCATTTCCCCCAGGAGGCGAAATGCCTGGGGCAAATCCCTCTGCTCCATTTCATCGATTGCCTGCACCATTGTTCTGAAACAAAAATTAAGACAACAAACACATATTTAAACAACGAAACTTGCAATT

>contig01047

TGAACAGGCAGTGAACGTTCAAGTTACCGAATCGTTTTGGTGCAGAAGATGTTTTTTTTCAAGAGATTTTAGACAAATACTTTTAAAGGGAATGCTTTGATTCACTTGAAAGTATATTCCTAAATATTATTAATTTATATGTAAAGCATTTATTATTTTATGGACACACTCAGCCCTTAATTGTTAAAAATCAATATATTTAATATTTTGTTTTATATTCTGATATGCAGACATTTTAATTTtCTTTCAAAATTTAACGGAAAGCAAAAAAAaTTTGCAATAAAGTTCTCACAATCAACTAAATTTAAAATAATTATGATTTAGAGGAATTCAAAAaTTAATTCAAGTGAAGTTGTCCTAGTAAATACATATTTTTAGATTT

>contig01048

ACTCTTTGGCGTGGCACAACTACGAGCCGCGATGAGAAGATGAAAAGCGCCAGGCCGAGCACGGAATCTTCGTtCTCTTCACGAAAGGACATAACAAAGAAACTAGGATAATTATTATGATCTATTTACAACGACGCCACAACGCGAACCACGGAAAAGGATTCtcTTCTCCATTCGTTGGAATAAATAAAAGAGAAACTTTGTTCTGCTCTGATAATACTTTTTATCACTTGTAATGCTTATAaCAAATTCCTAGTTTcAATACCTTGTAACTCATCCCTCGCATAAAATTTTCAAACAACAAGAGAaCAATAGAAAAAGTTAGCCTTTGATTGACTTCAATCAGGAAATAATATCATATTCTTTTTTAAAACGAGAATCATTCTCTGTGGTCTGCGTAGCAGAAACTCGTGTCTTTAGCGGGTGCACATTTTTGCAGATCCTCGTAAGGAACACGAGATCGACGACTACATTATTTACATAAGGACTA

>contig01049

AGTTtCTATTCAAAAGCAGCAAGTGAAAGAAGAAAaTTTCCAAATCTAAAAAGGAAGCTTTCATTTTAATAAACGAAATTCTGCAATCTCCATTTATCAGGATCCTCGATTTAACGAAGATGAAGATCTATTTTGGTGCTTCTCTCCTCACTCTAGCGATTTACTTCAATTTCATTGAaTTCAGTTCGCAGTACAATACAAAACTTCTTAAAGAAGGCGATTTGAAAGTGCGAACTGATGTTGGTCTCCATTACAATTTAAGATATGATGTAATGGTAATGATAAACGATCCGAAATTtCCATCAATTCATGGACTACTCGACGACCAAACAATTGTTTGCGTATTAGTAAAATAGGACAACAAAACAATGGTGCTTCCATTAATGATAGAGGACAAACAaGTTAAGTTGACGAAGACCaCTCATTTTTtGtaGACTGATGATGATAATCACAAAt

>contig01050

GTTGGAAAAGAGCGTTACACTGcAATTCATCGTGAGGTTAAAGGTGTTCTAACTTGTGTA

>contig01051

TGATTCAGTTGAGGCAGAAACTCACCACGATtCCTAAAACGAAACCAGAATG

>contig01052

AAAGATGATCAAAACGAATTCTCATTTTTCTTCACAATTCAGCTGAGAT

>contig01053

TCGTAAGGCCTAAATCGCCACTAATTATTTTTTTCATTCATATAATTCTACATTTTCTAAAGATATACGAAAGCTTTATTGTAGTCTTATTATCCATAATGAAAATTACTCTCTTGATATTTTACTATTCCTATATAA

>contig01054

TTTCTGATGCATGACTAAGCATTACAAATGATATCTtAGCACcTTATTAAAa

>contig01055

CGAAACCTCcaGCAGCAATCACCAATATTCAATGTTCACctACCCCGATACCCCGGCTAAAAGCAACTTCCTCCAGTATCGGAAAGAAGACCAAGGCTCCTCTAATGGCAAAagCGCTTCAGTTAaTAAAAGGGAGATATAGGCGATAGTAGTCACTCCAAATCTCCCAACTAGAGTGAATTTAAaTTTCGCGATAGTTGTtGATTTATCCCACTTTAATCGTAGGT

>contig01056

ACCACaTgTTGCAAAAGTTGCAGACAAAGAAGGGCTGAAAAATCGTATCCCTGCTTTAAGTTCTAATTTCATAAAGtaTGACTCAAAGAaTAAAAATCGtGCACCAACAGTGAAATCTCCCGTTCTTCGAGATAGAACGAACGAGGTTTTAGGCTTTTCGAAAACTGATCTCTCGCCAaTTGTTTTACTCTCCAAGATCAAACAAAAaGtGACAGATAAGAAACCTCAAGTAATCGTTGAACGTTTAGATCAGCTtACTTTTGAAACAAATTCGGACGTAGAAAAAGAGGAGAAATTACATAAATTACAGATTTCAAAg

>contig01057

CAGAGCATCGTGGAGTTCAGACCGATTTTTTGCACCAGTCAGGACAATTTtGCCGTTAaCAAAAaTAAGTAGCACAACTCTCGGCAGGACCATTCGATAAATCAAACCAGGGTAGAGTTCTGGCTCGTAGCTTGAAAATTGGCAATGCTTTTCGTTGAAATTGTCCAGcTTAATCGGAAATTTAAGATCGGCAGTGGCTACTATATTCTGGaTTTtGAAGTTTAGGAATTTGGCAGGAAATCCGAGCTTCTGAATAATCCTCGCGAATTTtCTTGCTGCAAGGAAaGAATCTTCCTCACATCTGGCTCCAGTGCAGACTAATTTTCCGGATCTAAaGATGAGTGCAGTTGCCCTAGGATTTCGAATTCGCATAATTAATCCCGTAAagC

>contig01058

ATTATGTAACTTTGATCGGGAGTGACGGAAGCAGAACGCGAGGCTTTCGAATTACTTCCTGATGACGAGAGACAGTGCGAGGCATGTAAAaCTACCTGTTTCTTAAGCGCTGTAACCTGTTCGTGTCAAAGTTCGCAATTAGTTTGCTTAAGGCATTTCACGGAACTTTGCGAGTGTCCGCCGGAAAAGCACACTTTGAGATACAGGTATACTTTAGATGAGTTACCAATCATGCTGCaGAAGTTGAAACTCAAGGCCGAGTCCTTTGACTCATGGGTGTCGAAAGTGAAGGAGGCGATGGATCCC

>contig01059

CTAATAGAGaTAAAAAaGCAACGCTACTGCaTTAAACACTGGGCATAACATTGGTGACATCAAATTACTTAATTAATGTAAAaTTATTCATCTGTCTTTCAGACAGCTCATTTCTGTTAGCTCAGCTAACGATCCTGATAAGCGGAATCTATCAATTAATTAAAGAGCAACGTCATAACAAGGTAAATATAAATTATCTaTAAAATGGCTTTAGCCGATAAGACGTTTACAAAATTAAATTAAAtATTTTtAGtCCAGACGATAGCAAATTTAATACAACTAAACAAAAAaTGTCCAGTCGTTACGATTTCACTTTtAACACtATTTTGACTCTGCATCAAATAATAAATTGTTCTGGAaTTTCAAAAGAAAtAATTTCTAATAAAAAAaTAAAATTAtATTAAGTCTTTTTCAACATTTTTACATGATTTAAAaTAACTTTGCTTTATAAATCGATCAAACGAATCGCGTCGaCAAATTGTGT

>contig01060

ACCCAAGAGACGGAAATGTAAACTACATTCCCCTAGGAACTTTCGAGCTAGCTCTAGCGTTTTTACCGCGAAAATCGTTCCAAGAATACGCGGTTCTTCAATGATCGCAAGACATGCAATACGTTCACCGCTGACAGTGAAAAATCATCTATCGCGCTtATTAACGAACAATTCTTCCGGTCACATGTaTTTAAAAaCAaGATTGGATaCTGAGGGACTGCTGCAAAGTTTAAATGAAATTCTAATCATGAGGCACTATTTGATGTCTTaCAAAaTTAAAATGAACAaTtCGCAAAACTTTCGAAAAAGTCCTTTGACAAATGAAAaGCAAAAATGGAATGAGAAAaGATACAATTACATTTTtGTGGATTTAAAAAAAAaCCTTCCCAAATTCAAGTGAACTTCACAACATCGCTATAAGATCGCAGACATGATATCATTTCTTATGGCTAAAATATTCAAAAGAGGATATCAATGCCATGTGTGGTTAATTGTAATTGAAAAAACTTCTTGTGGTCTGAATTTGAGGTGTTAATCTATTTATTCTTGATTAAAAaTAAATCGGTTCTCGTCTACTGTCGAGACAAAATTAAAATTCAAAACACCACACACGAAACCATATGAgCctCA

>contig01061

ACTCTCAGGATGGTATATTTTCGAGgATgTTAATTACTTTGACTTGATTTATTTCAATTTATTTCGGCGCATCTGCTGCCTTCTTAAGTTGGTCACTAAGGCTTTGTGTTGTCTGGACAGCAAGGTCGACAGCTTGCTTGGTGAATTTGGCAATGTCCTCTTGAACTTTGCCTGAGTTTTCGTTGAAAGCTTTGCTGGCCTTTTGAGATTCCTCAACTACAACTTTGATTCCATCTTGTAATTTCGCTTGAAGAGTGGCAGCTtGTtCTTtAGCGTTAGGAATCTGGCTGTTGATGTCATCGGCAACTTTGGTCAACCTGGTCTTAACTTCTTCCCACAGCTTTTCGATTTCTGGGCTCTTGGCTTTTATCTCTTCTGTAGCCTTGGTGACGACTCCCTTCAAACCTTCAGCGAAATTTGTGCTCTGAGTCTTGATGGTGTTTACGAC

>contig01062

GGAAACTaCCTATTCCACGCCGCaGCTTTATTGAAACTCTTGC

>contig01063

GCGATATGAaGAAGAaCATGTCCCCCGAGGAaCAGCAG

>contig01064

ATTATACTCGACCATAATTGTatGtATaTCTCCTTCATATTGTTTTtATTATTAGTAGAAAaTATTGACTGtGCTATTGCaGTTAAAGAAAAAAGAAAGaCAAATAAAACTGACTTAATACTATGAATAATAGACGATTTATAAATAACATATAGCATTACATGTAAGATATTCAATTGATTAGATAGGAAATGAaGAAGGTGTAATAAATCAATATAAATATCATAAATTGATTCaGAATAAATACGTAGTTATGTAtAAGTAGATAAAAaTGCTGATGTATGAGTGGAATTTTGAATTTTAAACCAATTATTGAGAAGGAAATTTTAGAATATTTTACTGTTTGAATTAAGATACTCTAGGGAAATAGATTGTCTGATTACGGAATGTTTTTCTGGAAATTTTTGCGAGTTCTTATTAAATTGAGAAACTTAGTATACACAATTCATTGGAAGAGGGTGAAAATTGTTAATGGAAATAAGGAAAGT

>contig01065

ACTTTTCATGGTAGGAGAAATCGTAGGTGGAATCTGGTCGAATAGTTTAGCGATTGCTACGGATGCTGCTCACCTGCTAACGGATTTtGCATCGTTtATGAtAtCCCTTTTTTCGATATGGGTGGCAAGCAGACCAGCTACGAGAAAAATGCCGTTTGGCTGGtATCGAGCAGAGGTGATAGGAGCATTGACATCCGTTTTGATGATATGGGTAGTGACGGGAATATTAGTCTTTCTTGCAGTAGAGCGAATTATTAATAAAGACTTCGAATTAGATTCCACTGCCATGTTAATCACTTCTGCTATAGGCGTTGCGGTAAATCTAGTAATGGGTTTGACCCTCCATCAACATGGTCATGGTCACAGTCACGGGGgAGgACACACTCATGACCATCACAAaCAAGAAAAAAGTAAACAAaGTGACAAATGTAAaGAAGAAGGCGTTGAAGATAAAAGCGAAGAGAAAAGAAATATCAACGTTAGGGCTGCCTACGTTCACGTGCTCGGTGATTTtATCCAGAGCGTGGGAGTATTTATAGCAGCAATCGTGATTTATTTCAAGCCTGAGTGGAACATAGTGGATCCAATTTGT

>contig01066

ACGCCTAAAGTGTTACAgCACCGACGtCtATCGTGGTGTAACTCCGAATCACGGTGCGCCTGATTTTAATCCAGAACCGCCAATTTTTGCcTGCCGTTTTTGTCCTATAAAaGGCCACGAGCAATACATAGCTTTAGCTAACGAAGATGGCAATATTGCTTTGCAAGACACGACTAAAAaaGGACATCCAAATGAGCAATTGCAAGGCCACGAGGCGCATaGAAATGCAATTTTCGATTtATCGTGGATGCCAAAGgAAaTGAAACTCGTCACTGCaTCCGGAGACCACTCAGCTCGATTATGGGATGTTtCACCTACTGGCTTCAATTtAATTCAATCCTTCGTCGCTCATACACGAAGTGTAAAAAATGTCGTTTTtCGTCAGCAAGATAACGCGGTCTTTGCGACGGGAGCTAGAGATGGAGTAATTATGGTTTGGGATACTCGAGCTAATCACGGCGTGAATCCAAAACCaGACAATTGCATGATGAaTGCACACGCTATTGCCAAAAGTCTCTCTAATAGTGCAAAGAAAAAATGTCTGACACCTGCATCTCGATCCCAAAGTATTACAGGCCTCGCCTTTCAGGATGATTTTACTTTAATATCTTGTTCAGCTGGAGACGGTTTAATCAAAGTCTGGGATCTGAGAAAAAACTACTCAGTTCACAAAAAAGATCCAATGGCGAAGCACATTTTAGAGT

>contig01067

TAGAATTTCAGCCAACGTCACAGGATTTGATCTCCGGTATAAAGATGCCAACAAATGAAGTTAAATATTTtACATTTAAAGATCGTAACATTCACAGATTAACGTTTTTtGTtAaCTACGAGAACTATTTtATCCAGCTGCGTATAGACTGCGAAATGATTATGTAAATGGTGAAGATAACACAAGATAAGACCAATCGGATATGTATAGAGCATCGATGATTGTTGACTATGTgTAAACAAAAAGAATTATAAAGAATCGTTGCAAAAAAAAaCGTAGATCACAAAAGATCTTAAAATTATTTTTGATCTACGCGACATAGAAACCCTAAGGGAATACTTGCGTGGTCTTCTGAAACTTCAAATGTAATTATATGTATAGCATGTGTATATCTTGGTTATTGCACCTGACATTTAATAGAGTA

>contig01068

ACGATCTGAAGAAATTACCTGGGGAAAaGAaGGCTTCGATTTTGGGAAGTGGAGCTGGTGCTCCAGCAGCTGGTGGAAAACCGGCCGCCgCAGAAGAGGaGGACGATGTCGACCTCTTCGGTTCTGACGAAGAGGAAGACGCAGAGGCAGCGAAAATCAGAGAGGAGAGGCTGAAGGCATACGCCGAGAAGAAAtCGAAGAAACCGGCCCTCATTGCCAagTCGAGTATCGTTATCGACGTCAAGCCCTGGGgAgATGAGACAAATATGGAGGAAATGGAGAAGGCGGTCAGATCCATCGAGATGGACGGcTTAGTTtGGGGTGCCTCAAAaTTCGTTCCTGTCGGCTACGGTaTCAGAAAGTTGCAGATCATGTGCGTCGTTGAAgaCGa

>contig01069

ACTGCTGGACAGGATTAAGGCGTGCATGTCCAAATCCGTCGCAACTGCTTCGTCTTCACTGCTCAAATCTTCAAGTTCTGGCCTATCCAAGTTTCGGGCAGCCTCGTTGAGATTCAAGGCTGGCATGTGCATCTTTCTTGCATACGATTTGCTCCAATCGATTGGCAAAATATTACCAAACGTGCCTGTAATTGTCCACCACATTTGGCACTCGTTCATGATTTCTTCTTGTGTGCGGACTTGAACCGGAGCTAGGGATTCTACATCGGTGCCATAGTCACGGAAACAGGAAGTGATTTTCTCGTCGAaGGTGTTCACTaGGTCCTCCAGGCTTCCGCTGAAGGGGCTAAAAGTTCCCTCTTGAAGAATGTCTGTCCCATTTTCATGTGTTGTAACAGGCTCGTTCGCATTGCAATTTTCTCCAAAACTCGCCTCGAGGTTCGCATCCAACAATTGTTCTGTCAGCAGGTTCATGTTCTTGATACTGGCCGTATCCTCCTCCTC

>contig01070

ACTGTTGGCAACCTAGCATTGTTACCTATAAAAACTCATTTTAGAGGACCCGCCCcTACATTAAATGGTGACGAATTGGATATTATAGATGAAGCCTTATACTTTTTCAGAGCAAATGTTTTTTTtAGAACATATGAAATTAAGAGCGATGCCGATCGAGTTCtCATCTACATCACtCTTTAtATTACTGAATGCTtAAAGAAaTTGCAAAAATGCGGAAACCAAAATCAAGCCCTCAACGAGTTATATTCTTtGGCTTTGGCAAAATTCGACATACCTGGAgATCCAGaTTTTCCATTAAATTTGGTTTATGCAAAACCAAGCAATACTACTGAGGCTGATTTAATGAGGCAATATTTACAGCAGGTCAGACAAGAAGCTTCTCAACGCCTTATCAAAAAAGTATACGAAGTAGGTGGAAAGCCTAGTAAATGGTGGCTATGTTTtGCCAAGAAGAAGTTCAT

>contig01071

GGCAGAAGGAATCGACGATTCAGTAGTAAAGCCCTCAGGCAAACCTCAATCAATCGGCCTGGAGGATGGTTTTAAACATATCTCCCTGGTCGACGAAGTCGAATCGCGACTTGGtGCTCTCCTCCGAAGACTGACGGAACTCGCAACTGAAGACGGTCGAATCCCCGTCGCCATGAAaCTCACTGTTCGCAAGCAGGACTTCAACAAaCCAAaTTCCGGAAAGAGAGAAACGAGACAATGTGCTTTACCCCAGCACCTTCTGCCTCATaCAAAAAaCGGGGCCTACGATCACGCCAAAaTGCTCGCCCTCGCCATGAAGCTCTTCCACAGAGCAGTCGATGTCTC

>contig01072

GAAGCTCGTCtCCACTTCCATTATTGCtCTCCGGGTTTtCGTTGACACCGATCAGTTCCAAAACGTCTTCTTCGTCCGAACGTTCGCCACGGGAAGACAtCTTTTTTtAAaGGTTtCCTTACTCTGATTaTGGAaTTAAAaCTTTCTTGAGATTCAAACTTTTAGAAGTCACTCTCAACCTGGTCCTTGTCCTTGCTATCTGTTTGAGTCTCTTCGGAGTCACTTTGCTGGCTTTGCTCAACGGATTTGTTTATTCTGTCCAAAAACGAAaCCAACAATCGCTTATTCAAAACATCTGTTAGTGTCAGTTCGCGAACAGGTTCATTCTCCGGTTGCTCTTGCGCGTCTCTTGGCTCCTCGTTTTGC

>contig01073

ACATTTGTGACATTTAGtAAGACAAAtATtCAATTATAAATAAAAAAaCCAAAAAACGAaTCGTGTtAAAATTaTAGAATATTATATGGACGTGTCAATGCTCTACGTGAACACGTCGATTTGTCGCTTAATATTAAAATTTTTGTTtAGTTCGTAACTTACAATtAACTAATTTCTACTTTCTCAaTTAAATTgaTCACAGTATTAATGTCtCTTTCAAATACTGAtATTAaTCATGCATAAATATGACTATATTTGGGaGTTTTCCTtGcaGATTTtCGCTCGTGTCGGAAAACCCTACATTTTtGTGAAATTCGTGACAAATAATACTCATGTGCATTCTTTAAaatCCAAACAACCCTTAAATACCTATATGCTAAAATACAtAATGTGCAAACAAtAAAAATCAGAATGCTCGGAAACATTTTTTtCATTCAGTCATTCAGTAaGTCAAAATGTTACCAAAGTCACCCCCcATCCAAGGATGGCGGAACAGGTCCAAGCTATGTATTCAAATTTTAAGGTGGGATGAGGGCGGAAGGGGTAGTGCATTTAAGAAATCTTCGCCTGTTGATATGCGAATCACTTGCGAGAGTTCAcGAGTTAAATGGTCAACAAACAGGTGTTCCGCCGTCCGTGCCCCCTTTaTTTACAAAAATGCGTCGatATTAATCTAaTGTAATATAATTGACAtAGATCAAGGT

>contig01074

CCAAGGAGaGTTTtCAGCTCATCGGCAACAGGCTTTAGTGTATATTTCAAATTTTTA

>contig01075

ATATTCAACTtGTTTGGcGATTCTACAGTCGCAAAAGCTCCACCGTTCGGATCAGTTTCTTCTAATGCAAATTCAGGACTTAAATTAAGAAACGTGGGAGCTCCAGGAGCGACTCCTGGTAAAATTTTTACCCAAAAATCCAAGGGTTTGAGTATCCGTTCAACGTCTGCCTTAAATATTCAATCTTCTTCGAATCAATCCTTCGGAGTAAAAAAaGATGGTGCTGGAACCTCTTTTTTAAAGCCAATTGATGTTTTTGGAAAATCCACATCGCCAAGCAAAAATAAATCTCCATCCAAACAGAAATCTCCAAACAAACAGAAATCGCCAAACAAACTGAAATCTCCCAAGAAATTATTTGAGACTTCAAACCCAGCTCCAATCATATTTtCGCCAAACGCAAAGTCGCAgACGAGCGAGAAaTCTGAAGAAATTGTTTTTAAAAAaCCAAAGACTCCaCGAAAAaTTAAAAAAaTCGatgTGGATTACCCAGAGCCAGAAGGATTGGCCCCGATTGGCGACATCGATACTGAGCTCTTCGATCTGACCAATCAGAACTTCCTTAAATTATTtAACAACAAATATTTCCTCGTTGACGTAGATGAAGGATTCTTGTCAGACACAGAACCGGTGCCAATCAACACAGATGAAATTCGT

>contig01076

TtCAAGCCTCTCAAATCTCTTGTAAAGTGCACAAAGTGTGATCAAAAAGTTATAAAGCATGCGTATCATACAATGTGTGGACCTTGTGCAAGGgAAAGAGATGTTTGTCCTAAGTGTGGAGAAAAAGCCGAATTAGTAGAAGGAAAACCTTCACCAGGAGAGCAGTTGAAATTGGATATAGAATTgCAAGCGATGCTCAAGGAACT

>contig01077

ACCTtcATTACTACTCTTTtCCTCTCCCTTTtCAACTTCTGCTTTTtATCCGCCATTtCTTCTACTTTTttAtCCGCCATTTCTTCGACTTTTttAtCCCCTATTTCTtCTACTTCTTTACTGGTAGGCTCACTTTCTGATTTCTGATTCACAGTTTGATCGACCACTTTTTCATCTATCACTTTTTTATCTACCAC

>contig01078

CTGTAGTTTCCTTCGTTTCAGGTGCTTCCGAATCAGCAGTTTTTtCATCAGATGCCGCCAACTCTTTTTCAACTTCCACCTGGAGA

>contig01079

GAGAAGCCTCTTCCGAGACACCATTTTTaTCTTGCTTTTCAGAGCCTGCTGATTCACTAGTTCCAGATGATCCCGGTTGATTTGGATCGTCCAATTTGTTCATTCCAGGTGGCGCGTTAATGTtAGCGGTGtGTCGGAAAAAGTaGTAaGGCTTTaCTTGAACCAAaTTtCCACATCCTtGCCACACGTCCTCTCTATCAtCTATTATGCATACCATATCGTCTCCACAAGGAAACAAAGCTTTAAGATTAGGGtATTTTGAGCCAGGATCAAAACACTCGTCTCTGGAAaGTATCCTATGaGAAAAGAgTTCTCCATCTT

>contig01080

TGGAATACCCTTTGGGTTGAAACTTATACACTAAGCGATGAACGTGAGTTGAGATAAAaCTGATAACCGGAAAATCCAAAGGTGATAATGTAGAGGATTGCTGCGATCCAGCAATTGATTGCATTTtGCTGGAAACCTTTATCGGCTGCTAAGAAAAACTCTTTCGAGCTCGAGAACGTCTTTCCTTCCAGGGGAAGATCTTCAACTAATGCAACACTTTTGCAGTAGAAGAATATTCCCATTAGCCCGAGTTGAAC

>contig01081

TTCTGAAGCGAGAATGGCCAAAGAACTGGGAGTCGTTCATCGGGGACATCGTCGGGGCGAGTAAAACAAATGAGAGTCTTTGTCAGAATAACATGGCCATTTTAAAGCTTTTGTCAGAGGAAGTCTTCGACTTCTCAAGTGGGCAGATGACACAGACAAAAGCGAAACATCTGAAAGACACGATGtGCAGCGAGTTCTCGCAAATTTTtCAACTCTGCCAATtCGTGAtGGACAaCTCGCAAAAtGTTCCACTCGTCGCCGTtACTCTCGAAACCCTGCTCAGATTtCTCAATtGGATTCCTCTCGGATACATATTTGAGACGAAGCTGATCACTACTTTGATATTTAAGTTCTTAAACGTTCCAATCTTCAGAAACGTCACGTtGAAATGTTtGACGGAAATCGCTGCCGTGACAGTGACGAATTACGATGACATGTTCATAGTTCTTTTCGCAAGCACGATGCAACAGCTGGAACAAA

>contig01082

TTCCTCTGCACTTACTTGAAAGAGCATGGTCAACTTGTGGAGAAAAGGCAGCTCAACGAGACCCTTTTGAAAGCCTTGCACTACCTCGTTCTGATTTCGGACGTCGAGGAGGTGGAAATTTtCAAAATCtGCCTGGAGT

>contig01083

AGCAGAGGAATGAtGAAATTGAAGGCGACCGTCCACGTGTCTACTGCCTATGCAAATTGCATTCGTTCCGCAATTGAAGAAAAATTTtaCGAACCTCCAATGGAT

>contig01084

TCGACAGACTGTGTCTTCATTTCCAACGAtGTCGGTAAACACGTGCGGACGATATTTTtCGATCCATGGTGTGTTGGATGATTTTtGAGAAAAATTCGTTTTTGGTTCTTCAGTTTTGGTCTCTTTACTTGTCGATGGGACGATTTCAACGTCCATCGGCTCTTCAACATtCTTTTCTACGTTTTCTCCTCCCATTTTAaCCAAATCTAACCACAAAAaTTGTTAAATTATTTTACTCGTCGTTACCGTCTTCGCGGGAAAC

>contig01085

CACAGCCTATTCGTAAaTCAAGTTCCTGACGAGCATCCACACTTCTTGCCTCATTTtCGTtAGGTTTTgCAaGATTGGCAAGTGCTGCTTTTATGTCTTTGTCTGTGATAGCAGAAAATTTAGCTCGCCAAaTGTCGTTGGgATTTAATCGGCGAGTAGTTCCTTGTTGAACTGCGTGAATTACTTCG

>contig01086

GTCgCAaTCCAACCAGAGGACTAAATAATCGCAGTGCGAACC

>contig01087

GAAGGAGATGCTGGTGCGCTCCTGGAAGATAATCCAAAAGGTGCTCGAAAGCAaGGAACGCCTCGctGTCTTCTTCGTCTAAATGCCTGTTCGATAAGTTTTGCCTCTGACTGGGGATCTATCCTCCAGAAGGAACCTTTTCCCGGTtCCTCCTGACTCCTCGGCACTTTGATAAAGTAGCGATTCAGGGACAGGTTGTGTCGAATCGAATTTTGCCAGCCCTTgtCGGCTGTTCGGTAATaCGGATAATTCTTGGTGATGTAAGAGTAAATGCCTGACAACGTGAGCTGCTTGTCCTGAGCCGAGGCTATCGCTTGAACAATGAGTTGGGCATAGGAATAAGGTGGCTTCGAGTCATCTTTtGGAGGGCTATAACGAGCGGAAGTTCCATTTGCAGCCGAGCTTCCGCCTCCTCCACCTCGATAACGTGGTTCCGCTCCAGGTTCCGGGCTCATCTGCCTGCTT

>contig01088

GAGGCATGCATCCATCAGTTGCAGCAATTACTAAAaTGGAaCCATCCATCTGAGCCGTTCCTGTAATCATGTTTTTGATAAAaTCGgCATGACCGGGACAATCAGTGTGTCCATAaTGGCGATTTTCAGTTtGATACTCGACATGAGCAACATTAaTTGTAattcctcgcgccttctcttcaggtgcattaTCTATTtCATTGTAGCCTTTTGCTGTTGCCAACGATTTCTCCGAGAGAACTTTCGTTATGGCAGCTGTTAAGGTAGTTTTTCCATGATCTACGTGACCGATAGTTCCTACATTGCAATGGGGTTTTtCTCTACTGTAAACCACCTTCTCGGCATAAAATCTCTGAGCAAAGATTGTTGGtAGGAATAACTGACTG

>contig01089

ACCTCTGATTGCCAAAAACAGCAAAACGGAAGTAATAGCATTGGCAAAGTTTGAAGTCTGGTGGCACTTGATCGTCAAAATCTACGACGATATTCCCAAGTTTGTCACGTCCATCTTGGCGCCCTTTTTGAACTTTTGCTTTGGCCCACTTGGCGACACACCTTTGCTATCTTCGACACTGGAAATTGTCGCTtCTCCCGGCAAAAGGTTTCCAAAGaCAAAGATGGCCTCGCTGGATGCGTTACTGCAGTTGCTAGCAGCCAATGAGGCATATTGCGGTGTTTTTGCCCCGGTTTtAAAGGAGAGAATTCCATGTGCTGTGACGACAGCGAATTTTGAaGAGAGCTACAAGACTTTTaCGCATAGTGTTTTTGAAGCATTAATTATTCTTGGACAGTTTGAGG

>contig01090

ATGGACGAGTTTGCTTGCTAAGATTGAGGATGCGGCTGATACA

>contig01091

CAGGCAGATTTGTTTATAATTTAcGTTGCGTGTTGTGTCCGCTATCGGAGAAATTCTGTATTCaGAAATTAAATTtCTGGATGGATCGACTCTAAAAGTTTtAAACTCAACAGGCATTAAATTT

>contig01092

TGGgCTGAAaCGGaCAAaGAAatGAcTCCCTACAGAAGAGTTCTGTTGTTCGAGGCTTCGAAACTTCCAaCAATCTTGGATAAAAT

>contig01093

GAACACGGAAGGAAAaTATTCTAAATtATTCTAAAaGGTATTCTAAAGTTGTTGATATTTTTTtACTTACACGTTACAACTCATTTGTGTCGATATTGTATGTTAG

>contig01094

AACTCATTTATCTTTAAGAGTTCTTCAATTGTATCCCTTTTTC

>contig01095

GAGTGACAGTTGAGTGACTACTGCAATCAAGTCAATATAGTGTGCGAGATTTGCCGTCATCCTCGGTTTTTCTGATTTTTTGATGGCAGAACATGTGTGCATTGAAGAGTGTCATCATCACTTTGCCTAAGGGTCTTAAAAAAGaCTCTGGCATAATTTCAACCAACGACCTCAGATCCGCTACCAAaCTTAaCTTCACAACCTCCATTCTC

>contig01096

GCTGAGGATTGATAACA

>contig01097

GAATTCGTGTTGGAGCGCAAGCTCCCTTGCAGTGTGATTTGGATTAAGTGCCGGAAAAGACGA

>contig01098

TCAATATGGCAAGTAATATCCGACAtGCACTAGCaTTAGTGCtCTCTGTtCTTCTGCTgACCGTGACAGCAAATACCCCTGTTCATATAATGTCCAAGTATCAGCTGATCACGTCGACCACCTTAAA

>contig01099

CCAGCTTtCGCTTCAGTGAAttCGtAATTTTTTtCTCATCCGCTTCTGTTATTCTGGAAGCGAACTTAAaTTTGTCAAaCTCTTCTTTGCAAGCATCTTCGAAATCTTGAGCAGTTTGTTTAACAGTTACTTCGGTGTCATCATTGACTGGTTGACCTTTtaGCTTATTGTCATGTTCTACTCTCAATTCATGATCAGACTTACAACtATTTTCGAaTTCGATTtGGTTtAgAGAATCTTGAAaCCtCTGTTCAAAaTCAGTCATTGGCTTTGTAAGAATTGGATGACGTTCTAAaCAAaCTGCGCTTATTAAATCCCACTTCCATTTTTTCACTGGTGCAGTTGTAGTtGAACAAGAGGCTTCTTGCCGAAGGTAAACACTAATTGCATTTAGGCTGCTTGGAGTGATTAATTTTAGAGGCTGTTTGAACATTTTGATTGATATTTAGGTCTaTTTATTTCTTCAAtAGCCCATTACCTAACCTAgAAGtcTTGCAACAAAATCACAGAAGATGCAGAAGA

>contig01100

TAAAGTATGTGACTTCATTGTTTATTTACTGAGGAAAaTTCATAAAaCTGAAAAATTTCCTTATTTAATTCGGGCGTCTGCTCTGCT

>contig01101

ACTTCTACTTGGCTCCATTAAATGCGCCAACAaGCTAATTTTCAACATCTGGTTTTAAAGCTGCATACCATTTGACATCTTGCATTGGCGAAGAGCTTCATTGAATCCCTGGCAAAGGGTGATATCAGACTGCTGTTCAGCGCACTGCAGGAACTGCTTGATTtCCCAGGCACAGGCACCGCCAGCGCCCGATTGTGCAGATTgCTGGGGAGCGTATTGCTgcTGAGGTGCagCTGCCGCTGGTGCGGCTGCTGCCTCGCTGCTTCCTCCGCTGAACATTCCGGTCATTGCGTGGCCAATGGTGTGCCCAACGGCAGAACCGATGGCAACTCCTCCAGCAGTGGCAGCCATTTGTCCCATCAGACCTGGCCCCTGTTGTTGAGGGGCCATCATCGGGGCTGATGGGGCAGCTTGAGCTGGCACTGGCGCAGTCTGAACTGGCCTAGCAGCTGGTCTAGGTGCTGATCTCACGGTCCTGGGAGTAGGAGCCGATGCACGCATACCACCTGAACGTCCACGAGCCATTTTCTTTtAATATTTAGCAAATCACGTAAAAACACGTCTTTAACACTGAAGATTCACTCTCTAACTGTC

>contig01102

ATATAGAGAATAAGAATGCTGTGTGCTTAAaGATAGAAATTCAAcACTAAAGCTTTTttAtGCcGGCCCTATTGTATTATCTAAAaTCCTATGA

>contig01103

AATGAGATGTAaTTtATTATtGAGTGAATTTATTCCTAAAaTCTTTACAAAaTATTGGTATTACtAGCGTAAATATCTTtCCTTAAATTTAATCTtAATGCTAT

>contig01104

GAAATTTCAAAGGCATCCTGAAAaTGATTCATCACTGAAACGTTTTtGtCTTGG

>contig01105

GAAaTGGAtAAATTCTTGTGGCCTTCGTGTAGAGATGGTTTAAAGTTATTTTATTATTTCCGCAAATATATTTAAAAAAATaTTAACAATAATAATATTTTATtCTAAAAaTaCAATCTGGATATTTTCAAGTTTATACATTCGTTAAAAGTTAAAACTACAAAGGCAAGTGACGGCACTAGCCCCGAAaTTTTCATGGCGAACGACAATAAATATACAAATTTaCAaTTCCGTCAaGTGAAaCaCAAAaCGGCCTTCAACTGAAAAtAAAAGT

>contig01106

AGGATTCATTGGCGAAATGGACACAAAAAAAGAAACGCAATCTAACAATCTGTTTGtGTTTCAAACaGCAGTATCTCAGGCATTTAATGGTATCTCAGAATCCGTCAGTGAAGAAGAATTCAAAAGCATATTTGAAATCCTTCAATCAAAGTCATCGACTGCAAGAAAGTTGTCGAAAGCATTTAACGAAGACCTGAATAAAAaCCTGAATATATCGCTGGAAGAAATTTTAGTGGAAGGTAGTTTGGCTGAAGGCTTGGAAAAaGTTTCTAGTATGTTAGAAGAAGCTGCACCTATTAAATGCAACGTCT

>contig01107

CAtaTTTtCGACTGAAGATTCTAGAGATGACGAACtGCTTACAGAGATTTTTCGtAAATGTCTAACAAGTGTGCAAGAaGCGTCGTCCATATTCAATATAtGAGATAAATTAGGTAGGTAGTTATGTT

>contig01108

TTCCTGTATATTATCTTGCTCTAAAAACTGCTTCTCACATTTATCTAGTAGACCGGAAAATTTCAaGAGGAGTGACTTTGCATTTTCAAAGAGTATCG

>contig01109

CGTGGGCCTcGTCGTCGGAAATATCAAAaGGTATGACTGCGAGTTCTTCAACGTTCTGAGTGTAGAGTTTTCGTATGTGTTTCTTTtGAGGCTTGATATTGTCTTTTTtCCGGCGTTTTGATTTTTCGAAGTAAGAACTTGAATTGCTGCTTGGGCTGGGAGACTTtGTGACTTTTCTGGAAGCTTtCTTTTTTGGAAATTTCCCCCTTTTGTAAGGGTTCAAAGTCTTCAGAGGAAGACGTGGAAGATGTGGAAGAAAGTCGACTTTTTtGGAAGTTTTATCGACGTCACTCAAGCCTGTTTGGTGAAGAAGCCGaGCGGT

>contig01110

AAAAAAaGACAATTTTTTGTCTCAAAATTGAGGTTACCAGGAAAGAGAGGTTATGTCACTAATTTTGAACGTCGCTAAGGC

>contig01111

AtGTAACGGATAAACGGAGGATTTttGtGCGGATTTTCGTGCGGATCTACCATTTTTGGTAGAGTGCTACGGGTAGTGTGTTTTTAAAGTTTTGGTGTGCAACAACTCTGTGCAaCAACTCCGTCCTAGTCCACGAAGAAGGTCACGAGCCGAAGGGCCTCAGCTTTtCAGTTTAAATCACCTAAGTTAAAAAGTTAAAAaCGGCCCATTTTTTtATtAGTCAACACGTCaGCTGGTCGGTTCGGCTGTATGAGATAGGCGAGAGATTGAAATGGCGTCGATAGTGGCAGGTGTCCAGTATGGTTTGTCTTCGCaCGCCAATTTtAACGAAAaCAAGTCCCTCATCTTCGTCAAACTCACCGATTCAGCTCAACGTGCCATCGAAGACTTTGTGCGCAATCGGAATAAACTCAAGCAGAACCCTACAATACAGTTCGACGGAAGCGAaGGGCGACTCtCCTTTCCATCCTCACAATCCAGCCAtGGAACCGCGAATTTtACCTTCAGCCTTTCCGGTAATCAAGACaTTGAGGgTCCCcAGGGTGGCTTCGAATGTATACATCAAaCTGGACCAGGtagCCTGGAGAGTCTGGGAGCTATACCGAGTAAAATGCGAATACATGCCAATGACGATGTCTACGAAACTACCAGGCATCGTATGGCTGTCGCCGAAGAAAATAATAAGAATAAaT

>contig01112

ACCAATCATCAGGAGACCGGGGAATGCGTAGTTCTCGAGGATGATTTGATGAATGATACCTGAGCCGGGGTTCCAGAATCCGACTCCGTATTTGGCTCCTGCGGTTTTCAGGAAATTGTAGACTTCCTTGTTTATTTCCTTGGCGCGCTGCAAATCTTTCTCGCCACCCATCTGGGCTTCGATCAaGTGATCGCAGTGGATTGTGGAGGGAACTGCGACCTTTGGTAGGCCAGAACTGATGAATTGAAGCATGGCCATTTGGGCTGTGGCATCTTGCATAGCCACGcGATCTGGTCGCAATCGCAGATAACTCGTTCCTCGTTCAATCTCTTGtGTTTtGGGTTCATCAATATGGGAATaTAAAaCCTTCTCCGATAGAGTTAGTGGCCGGTTGAGTCGTTTCTTCACTGTGTGGATAGTAGCTTCCAACTTTtCGTATGGAAGATAGGCTGTCGAATCGAATTTGCTCATTGCCACCTTTGCGGCAGCAGAGGTAAGAGGACTCGCATGGAAACATCTTTGCTGAATATCAGCTGCGAATCCAGCTAATTTCTGTGCGTGTGAAATTCGAGTGCAGT

>contig01113

ACTTTTTGCCTGATGCAAAGAACTTAATGGTAGGATAGCTGTTTATTCCATACTCACTAGCCTTCACTGTGTTTGAAGTGGCATCGAGAGCACCTAGCTTGACTTTTCCCTTGAGTTCTGTGGCTGCAGTTGCCCAATGAGGTGCCAAATTTTTtACAGTGACCACACCAAGGAGCGAAGAATTCGACCAACCACATGTCTTCTGATCCAAGAaCCAGTTTGTC

>contig01114

TTCTCATCAGTCAGTTCAATAACGTCTTtAGAGTCTTTTGACTTATTGCTGCcaCCCTTTTttAACTCCCAAAAAACCGCGAACTTTTTTtAGAtGCTGCACTTAATGCAGCATCTACAATGTCAGCGGCTGTtCGTGGTCCATTATAATCTTCTGCGTTTGTTTGGCCTTTGCTGAAAaTTTTTatAGTGGgAAAGCCTCGAATGTTGTATTTTCCTCCcAATGATTTGTGGTCATCGCCATTCACGGCACCCACTTTCACCACACCcTtCAacGATGTGGCGGCCTTTtCATACTCTGGTTTTAACTGCTGACAGTGTCCACACCATGGTGCATAAAATTCCACAATCCATACACTATCGCTATTCAACACTtgaCTATCAAAGTTGCTGGGTTTCAACTCAACAACATCCGTATTTGAATACATACAATGAACCTCGGCCAAGATCCCGAACATCAAAATAGGCCATT

>contig01115

AACTAATTTTTCTAGAACTGGGAATTGACGCTTGAAAGACTTGATCAATTCGGCTGATTTAACCCCATTATTCATGTTAAAGCTGATATCGACTTTTATTTCAGTTTCCTTGTCAGTAAGTTTGACGATTGGAACACTCGCTTTATCCAATACTTTGATAGATGAGGGTTCCGCTATATTTTGATCTAGtAGAGCACGCTCAAGTGTCCGAAGTGGCAAATTTGACCAGATTCCGATTACAACCAAATCAATATCACTTGTTGGTAAATACAAGCCAGTCCGAAAACTTCCGAATACTTCAACTTTAGAATCTGGCCATAGTTCATTAaTAATTTGTTCTATtCTTTTAACCACGCGCAATCGTAAACTATGTTCTTCAGTGGATGGGCACATGTAAGCAAAGAAaTCTTCAATCTCTTCATGCAACCCAATAACACCTTTGGAGTAATGTTTGTTtGGTATGCGCCATGGACAACCACCATATGCACCAACTAAAGCATTATAATTATAATTCATACCATAAGTGCTAGCACGATTGTCATTTTTTCTGCGAGAAGGATTGTAATAATTATTACAGCTACTGTGTTGGAGGCGATTAACCGGATTAACTCGATCTTCGCTATTGCGACTGTAGCTGTCGGTCAATGTATTGTCTTGtAGTATTTTATTACCCATTAAaCTTGACACGCTTGAAATG

>contig01116

GCGTAGCGTGTCTATATAATTGTGCAGAGGTGTTCTCTCCATACATAATCTACTCGTTAA

>contig01117

TCCTCTtCtGCAAGTTCAGTTCGGtCCAGGCCCACACAGTGCATGTGGAACCACCCGTTACACCCGCcATCACACTGAACCCAGTCAACTTCGTGACCGCTGGGTCGCAGGCAACTGAACGCAGCACAGTCTTCTTCGTCTTCAGAAGTATCTTGTTTAGtCTTCTTGCGACCCACCCGCTTCTTCGGCACTTCCTCCCCTTCCTTTcGCTTCATCTTCCGCGGTCCGCGCTTCACTTGTCCCGTAACCGACAATTTCTTCTCCTTATTGGCCGCGAAACCTTTGCGCTGCTTGAACAGCATTTTCTCATCATGTCGTTGATTCGCCTTCATCAGGTCAAAATCGGAATCAGACTTTCGTTTCCGACCGCGCTTTTTAACCTCTTTCACCTCTCCATCCTGATTATTGGTCTTGATATCATTATTCAAGGATGCGTATTT

>contig01118

ACGTAAtCCAGAAATGATGAaGCACTTTGGGCATCACTGCAAAAgTGTCTTGTGTCATTTCCGcAAACGCTCGTGTGAGAATGAGACTGCGCATtCACGACCGGTTAATCATCATGCTCATCATCATCATGGACATAAACATGGATGTCGCCGTGCGGGAAGAaTAGgTATGCCCcGCACcATCACTGTTGCtAATGAAGGTCTtGAGcATCCTCGcAATCGCCGCTGTTCAAGCcTtCCCCCcACTGTTtCTGTCAaTAaCACTGATtGTCAGCAACTCTAGGCcTAGGGgACACTACTATACGgAACAAGTGGTCATCGAGAAaTTACGAAGAATTTAGTGTTGAGTTTCTTCCAGAAATCAACTTCGGACCACATTGGCTAACCAATTAGCGTGAAGAGTATTATATAATTtATCCAATATCACATTATATAAAAAAAaTGAAAAACTATAAGTTGCAC

>contig01119

tcAACaCaCAAATTTTTGCTTTGTAA

>contig01120

ACTTCAACGTCATTGCGGATCCTGGGAGGTTCACTCTTGATGAGTGGATGTCGGTAGATGTAACCTGTGCGGAATCCAGCATTATCAAGCATTCTCATGAGCGCCTCAAATTCCGTGCCACCGATTCTCCATCGCTTCTCCAGCTTTTCCGCGAGTGGAGAAGGTTCAGCAACTGAACTGGGTTGGGgAATGAGTGGTTTTCTGGAAGCCTCGAAAATAGCTTtCTTCGATTCCTCGGAGATGAGGTTCATGTTGTCTAATCCAGCTGGCATCAATTTGAGTTGTGTTTCGCAGGCCAGCACGGTGTTGTAAACGTTGAAAAATGCCTCCTCGACTGTCGCTCCACAGCAAATAGCACCTCGATTGGTCAGGAGCATGACTTTGTTGAtcGGGCCCAGATTTCTCGATATTTTCTCTCTCTCTTCGGGCTCGAAAATACCGCCCATGTATTGATGGTTGCTGACATCACCTATTACTATGCTTTCTTGACCGAGTGGAAGAAGACCGCATTTCATGGATGAGACAGCTGTGACGGACGGAGTGGCGATGTGAACGATGCACTTGATGTCTGGTCTAGCAGCGTGTATTGTGGAGTGCAGTTGGAATCCTGTGACATGAACCCCAAAaTTTGTCGTTCCTTGCTCCACAACCGCGCCCTGCATATCGACTTTGACTAAGCTAGAGGCAGTGACTTCG

>contig01121

CAGGCTGTGACAGCCTCaCTTAAAtAATATATgCTTGTCAATCCGAGGTTTCTGAATAaTGAaTAGAATtCAGATTCAAAAaCcTtCTTTtCCAGGGgAAGGGCCATTCATATAAACCAAAGTCAAAACCACATTAAATCCCATtACGgaTTtATTAAATTAGTAAGAGCTTAAGTGCATTCAAAaTCCACTGGCTGCAATCGGCGACTATTGAGAAtGCATTTCCACCAGTTAGCTGATTCTTTTTtGGTtCTCTTtCTCATGGGATCGTTGAAATCAACCTCAACGATACCAAACTTTAATCCGTATCCATGTGCCCACTCGAAATTGTCGAGGAGGGACCAAATGTGGTAACCCTTgACGTTCACACCATCTCGGTTAATAGCTATTAGTAGTTCCTTTACGTAATCATGGAAGAATTGAATTCTAGTtGGAtCCTTtAACCCTTCATCTAGCGCCACTCCATTTtCAGTGacATATATTACTGGA

>contig01122

ACTTTGATTGTtACGGCtAAGAAGAGGGCAAAGTCTTAAACCTATTGGAACATAAAATACGAAAGTTGCTATATATATATTATtATTACACCaTTCTGCAAAAACaCAACTCACCCTCGTGACATCAAAATTaTTTAGTTATGTATTTTGAAGTAGAGTCACTCCAAgaCCGCTCCATCTCAAGCATGAaCAGGTCGGTGgAAAaTTtGTTGATGTCAAGTCACGAaGTGATCAaCAGCATAAATGCTTCATCGTTGGCAGGACAGAGTCCTGTAGGAAGTCATGGgAATGAGGTATCCCTTGATAATGTGCTT

>contig01123

CCGGAGCCATTTGTCTCCACAGTAGACATGGGCGCGCTTTCGGgTTCCACAATTGACTCTACTCTTTCCATGGCGAGCATACCAATCCTACCAATTCCCGTTGAGGAGAAGAAGAGCGAGCACCACAAGAGCGAGAAGAAAAAGAAGGaGAAGAAACATAAACACAAGGACAAGGACAAGAACAAGGAGAAGCACAAACACAAGCACAAGGATAAAGATAAGGAACGAC

>contig01124

ACGCGAAATGCAACTGACCTTCCATTTTTTTTGTAAAATGTCCAGATTTTTTATACTTTTAAAAAaTTCtGTTTTACTCGTCTTAAAAaTATTCAGAAGCTaGCTGATTGAAGAAAAACTCTTGTaGGCGCTTTTTTTTtCATCCAAGGAATCATTATAAATAACCTTACTAGAAAGATTAGCAGACGATGCACTCCTTGGATAAGAATGCTGCCTATGTGAGTtAacTTTTTTTtATAATACATTTAAGCCAATATGCAGTCTTGTAGTGAGTGTAACTTAACTGCAGAAATTTACACAGTGCAAGTAATCTACATTATTATAACGACATTTGTTCTAAGTATTAAGTATTTCGACAAATAAGTGGTATGATTTGAAGAATTTACTGTAATAGGAATTGCTGATTTCCTTAGATAATAAATAAGAGATAAGCAATTTtGAAATTGGTATTAAATTGTAAAAaGGAACAATAGTGGAACATTATTAACTTGTCAACAACCCCGACATTGCaTTAGCATTTATAAAAaGAAAaTGGAGAAAAATTTACATTTAAGTAAACAGGGaCAATTGAGACTTGTTCAaGTTTCGCTTGGAAAGAAAAGAtACGAGAGTTATTTTGAGTtGCACGAtGAAAATGGGAAATTTTGATTGAAgATTCATCTGAAATAGGCAAATTATTATTAGCTCT

>contig01125

CCGAGACTACATCCACGTAGTCGATTTGGCAACAGGTCACGTTGCTGCTCTATCAGCTCTTTACAGAAAACACCTAaGGCT

>contig01126

CAATGTCCACACGATATATGAAAAAaGTTTATGGGACTGATTtAATCCCGAAATTAAGTGAATTAGAAGATTCAAGTGATACTGAAGCACCAATAATCAGAGATGTCAAGCCGAAAAATTtCAACGCaTTCAGCTTGCTGAACTGCAATGGTTCCGAAGAAGATGAAGACCCAGTCGACAaCCTCAACGACAACGCTGATGAAGATGGTAaTGaCAGCAAGGATC

>contig01127

ACCCCTGTTTAGATATGTTTACATCCCCATCGGGAACCACCCAATCGCTAAATTTGTGACGATGCCTGTTGACGAACGAGACATTTCGTGCCTTAGAATATTCAATGTATCGTGCTTCATCCAAATTTTTCGAAACCATTTCTGCGCGGACACGTCTACTGTGTTTAACATGATCCACTTCAAGTTTATCCTCGCACAATTCACCAGTTGTGTCTAAACCTGTCAAGTAGATTTCCCACGGTCGTTTAGTCCTATTGAGCTCATCAATATGCTTCATGTCGGgCAACACTACTGACAGGTCGGAATTAGCGACTTTGTAAATTGATGACTTAAGATCTTTtAAATCAAGGTATTTtATAAGGCGATGCATTTtAACTTTATCTCTACGTAAAAGAAATATGAAATCTTCTGTGTCGACCACGGAACTCCCCcTTCTATCCGCAATCTTGCATGCTTCTTGGACAATTGACCTCATTTGGCGCAAAaCCAT

>contig01128

CTTTTTTAATGTAGATAAAGGTGCCTTAAATTAATCTGAATTCCTTTAGACCTGTCTTTTCTGGTGACATTAACATTTTTCACGCGTAATAATTATTTAAATTACAAAATTATCTTACCTTCAAAATAATCCGTGAATTTATTATATTCTAATTACACTTCATTtAGCTAATTAAGTAGAGTTGTGTAGTTACTGTTTAATTGTAAGAaTCATTCTTTCAAAAATCAAGGAAGTTAAACGATAAGTAAGAGCCATGTGCTTTTtGGCGTTAGTTCAAGCATAATGTATGGTTTTGTGAaGATCAAGACAAGAAAACTTATTACATTTCCTGTATATTCATAATAAAAAGTTCTAaTTTTtACCATTAGGGAgAATAAAGAGAAAACATTTCGATACATTTTtAACGTTCGAGAAATCGAATTAGGCAGATTtATTATTAAGATTGACTATATATTaTAGAATACTtATTTtAACTCACtGTTcT

>contig01129

ACCGGATCCATATCCGAATGAAGAGGCTGCGCGTGCAGCAAATAATGGTGCATACCCACCAGATCTTTCGTTTATCCAGAACGCAAGACACAATGGGgAGAACTACATCTTCGCCCTATTGACCGGATACTGTGATCCACCAGCTGGAATTACCCTGAGGgAGGGTCAGGCATTCAATCCCTACTTctCGGgAGGtGCCATCGGTATGGGTCAAGTCGTTtATGACGAAGTATTGgAaTTtGAAGACGGAACGCCAGCAAGTGCGTCGCAGGTGGCAAAGGACGTTGTCAATTTCCTCATGTGGACTTCCAACATGGAACACGACGAAAGGAAGAaGCTGGGTCTCAAGGTCCTGGGAGtGGGATCATTCCTGTTAGTTTtCTGTTACTACCTCAAGAGACATAAGTGGTCTGTAGTAAAAACCACAAAACTGGCGTtCATtCCTCCCAAAAAGAAGTAAAGTAATTGTGGAGACTAAATCAATTGAACTGTTAgCCGAGAAAGGTtGCTTGGAATATTAGATATGTCCGGGTGCGAACAGTAGTTTTttAATTTAAGGAATTCTCAGCAGCAGCAAAAGAAGGAAAAAAGAGAGATTGTTGGGGCCGGGAATGAAtCCAAGGTCCTCTCGATAATCTTTATCCTTTCTCAACAAGCCCCCCAGCATTATCAGCACGAAGTCATCTAATGAAA

>contig01130

TGTGACGATCATTGGACCCGCGACTGTtGGAGGTGTCAAGCCCGGTTGTTtCAAGATTGGAAACACTGGAGGAATGATGGACAatATCCTCCACAGCAAATtATACAGACCTGGCAGCATCGCCtACGTTTCTCGATCCGGAGGAATGTCAAACGAGCTTAACAATAtAATTTCGAAAGCCTCGAATGGAGTTC

>contig01131

AGTTACCGTGAAGATTATTATTGTCAAAGTTGGGCATACCTTCGCCCTTTTTCCTAATTCGAGAATTAGGTTTTGTTACCTTATCTCTCGTTATAGTGACTCTGTGGCCGTCTAAATGAGTAATATGGACGGTGAATCCAATCAAAGCATCTTGCAGCGATATCGTCACATTAGTGTAAAGGTCATCATTTATTCGCTCAAATACAGAATGTGGCTGGGTTCGAATTCTCAGAATTAAATCTCCtGGAtCACCGtCCAAATGAGGTTCACCTtCCGCAGTGAATTTCGTTTCTTGACCATCAACCATTCCCGGTTCGACTTCCACCTCCAGAATTCGTTCTTCATTGACTAATTCCACGTTTGGACATTCAGAGCAAACATTCTGTTGCATCATTTGGAACCTTCCATTACCTAAaTTACGAGTGACTAATTCTTGTCTGCAATTGCACTTTCTGGTCCCCTTCGCGGCTTTCATCACAGGCTTATTtCTCGTTATCTCAATAAAATTTCCACTATACAATTCctCGAGCGTAACCGGAAGATCCATGACAATATTGGCTCCCTTGGGAGTTTGGTGCTGTTGCTGTTCGCCACCAAAGTGGAAACCGAAATCGCCAAAGAAACTGGCAAAaGGATCGGCATTGTTCATCATTCCGTCCTTTTGCAGACACTCTTCTCCACATCTATCGT

>contig01132

GTGGAAaCTATTCGTTGATATCGACGCCC

>contig01133

TCTTTCGATGATAATGCCCAGTTTCGGCAAAAGGATATTTTtGCTCTGGaGGACGACAGTGAGAAAGATCCCaGAGAAGCGGATGCCaCAAAATTTAATTtAAATTACGTA

>contig01134

ACTTACACTCAGAGGTGGTGGATTGTCAAAGAATTCGTTGTTTAAAGACAAAGTGGATGAAGTCATCGGAGTCTTCCTAGTCATGATCCGGATGGCTCTATCCAAACTGTGATAAGCCTTCAAAACAAAATCACCGATACCCATTTTTTCACCTTCACTTTCTGTAGAAATATGACCCAAAGAATCGCACATTTCATCCGCGGGGGTTGGAGATTGAAACTGGTTtGGTGAATGGGTTCCCGAATCTATATCCAAATCCATATGCCAGGTTTGTTGTCTTTGCAAaGTTATGCCTGGCACTTTCGGTTCATCAGTGTCCTTCGAAATCTTATCGTTATTTTCGTTGAAGGTCGTTGGAAaCAGATTCTTAGAGGTAAGAAGTTTGTTtCTAGGAGATATAACTCGAGCCGATTTGAAGTTTGGTTGAGTAAGGCTGCGATGAATTCCATCTTTGGATTCTGCTTTCCTTAAAGGACTTTCAGTAaCCTCcACATTTCCCAGGGCAGATAGATTAAGATCGTTACAAGAATTTGCAGTGGCGAGTGGCGAGGGAGCGGTCATAATTTTAAAAAGTCGATAGACCACATCCCtcACAACATtGTTCaCTCCCTTAGGAaTCGACGGCAGAAGATTACTtCCACTCTCTTCAtATTTGGaGCTGCTTACACtGTCATTATTGGTGTGACCTGT

>contig01135

ACCCTGGAATTTtCTTTtCAACATCTTTACTTACCACTGGTTTCATTCAAAaTAGACCAGTATTGAGTTTTTATAGTGTATAGTCATCATGATTGTAGAATAAATTGCGATGCATTCGCATTTGTGCCTGCCTCTGCTATTAGGTATTGACCTTTTTCGAGGGTGACAGTTTtGTTTACAATTTtCATCGCGTGTTCTGGTTAGGATATCGGACAAATTCTTTATTAAAAATATAAaTTCCTAGATGAATCGACTCTGAAAaTTAATTTATTGTGACTCTCGTgTTCTTTTACGACAACAACAAAACTCAAAACTGCTGCTGAAAAAAGTAAATTTTTTtGGATCGAAGAATTATTGTTTTAGACTTCAAGATATCAAAGTTTCTGCATGGTTAGTCTTCTGGcGGCTTCGAATTTAAACGATTTTACCAAAaTTCATTGGGAATTGAACTGGGATACTTTTAAAAATGATGGACGATGAAAaTAAAAAAaTGGAAATAGAAACCAAAGCATTTGAGACTTCGGAGATTGAAAAATTAATCGATGATTGTCTTGTCGAAATTTTCAAGCGTCTGCCAACTATAGCTGACAAAATTTGTATTTCAAGAGTTTGCAAAAAATGGAAATATTTGTGTCCAAGGTCTATGGATTCACGAAat

>contig01136

TCGgTAGCGgTAGGTGCaC

>contig01137

aCTTAtgtcATGgaTCtGAACTTGTAAGGTTTGTATTTCTCTGGTGAGATAGGAGATCACGACAGCAAATACGATCATTACAAGACAAACCAGAAGGTTTCTTATCCTCCTAATCAATTTTCTGTTTTTtGAACCTGAGAGCACTACGTTCATGTCAACGTTCCACTCGATGTGTTTCGTCGTGTTTTCGGAAGGGTTCACTTGAACTTTTGTGTCATTTTTGGGAGGTTCCATTTTCAGATTCCAGGACATCTTCCTGCTTTCATTGTAATTTTGTTTAGGGAACCCACCCTCAGATATCCGAGACGTAAGGTTGTTCTCTAGCTGAGAACTGGTCTTACTACCCTGCTGGACAAACACATCCTTGGTGTCATTTCTGTTGATGAACTCGTAGCTACTCTTTACGTTGgACCATTTTTTtaCGGTCCCGGTGACTTTTGTAGCGATATCAGTTTTTTTCTGCACAGCCTCATGTTGTTGTTCGGGCTGCTCTTCCGGGTCAGCCATGGTGATAATGATGTTAAGGTGACGATCCTAGATGTTCCCGTCTACCCGTCCTGTCCTTCTGTTTACGCTGTTGGTTTcGTTTCCTTCGaTTTTCTTTGGTTTCATCCCATGATCTCaggCAATCTgAGgCTGaGTTGACtCAGAGTTGACT

>contig01138

TGGGCCAAATAGGAAGTTaCGTCTAAaGCAGTGGAATAGTTTAGTTTTCCAGCTATAGCTAAgTTTAGAGCATCaTCGATCAACTGAGCTCTGTTTATTGTCG

>contig01139

AGTGACTAACAGTAAGAAGTAGCATATTAATCGAATACATTTTTAGAATACTACCTGAACCAATTTTTTGTGGATTGTGAAATTACGCCCAAATAAATTGCAGCTCTGCAAAAAGATGAAATTTGCATtCATAATTTTATTAGCTACGTTGTTGAGTTTTGGTCTGGCGATACCCAATATTACTACCGACAAACAAACATACTCGATGCCAGACACTTCTAAATTTAATGGAAAAGAATCAGAAATAGATGAAATTGAGGAAAGTAAGGAGTTGCTTACAAATTTTTTCCAAACACAAATTAATATACTTGGCAAAACATCACTTTCGATTTTTGACGATTTTGAGAAAGTTGTCAACTTTGACATAAAATTGCCGGATTTTAATTTACTTAATGTTTTTGCCGACCTTGAGAAAGCAGTAACTTTTAGACTTTATACTAAAGCATCCGGCGATAAATTCCAACTTTtGGGTGTCAATAATGAAGTTGCATTAAAAAAAaGTAaTTTTGATACGAAACGACACACCTACATCATGACGCATGGATATATGGCTTCTTCCGACGGCTTATCATGCACTTTAGTTCGAAATGCTCTGCaGAaGaCAAAaGaTTCGAaTaTAaTCATAGTAAACTGGAGCCCTTTATCTAACGGTTCCTATTCAGTGgCTAGA

>contig01140

GGCATCAAtGACGGACCATGCcGTGCTCcATcTTCTCCCCcACTGACTCCACTtCCAActAAATAAAaTTCCTTtCTTTtCTAATTCCTCGGTTCGTCTCTGTGTATCCTGATATTCGGAATTTCCACCATCGATGATTATGtCCCCTTCGGATAAAAGTGGGACCAATTTTCCGATAAAATCGtCCACAGCTGAACCGGCTTTAACCAGTAACATTACTATTCGTGGTTTTTTtAaTTtGCTGACCATTTCCTCCAAACTGAAGGCACCAACGACCTTTGTTCCCTTAGCTTCATTCGCGAGAAATGATTTGACCTTATCCGTTGTTCGATTAAAGGCACAGACTACAAAGCCATGAtCATTCATGTTtAGAATTAAaTTTTGACCCATAAcGGCCAAGCCGATCAGAGCAATA

>contig01141

ACTTCCAAaGCAACTaTTTATTTGTGTTCATTATCCTGGTTATTTATTGCCTGATAAC

>contig01142

GTTTTtAATCACAGATTAaCATTAGCCcAAGTATATtCTCTGaTTGGcGCCTGCTCTCTGCCTCTTTTTtATTTAGTTGGAGCTGGTGCTGCGCTCTTCTGGGTCCTTGGTGTCTCCTGTTTTTTGATAACCGTTCACGCAACTTTCTACAACATCGATTCCATTCTCTGTCCtGGAGAAGACGAACTTAACTCGTTAGTGATGCAAGAGGTGTGAaTTTAATCTTTtGAGTTGaGTAAGTGAAAaGTAaC

>contig01143

ACCGATTCTGAATACATCTAGCATTTaGCATCTAGCATCTAGCCAATGTCGGGCTTCCGCTGTTGTGGTCTTGAATTTGTTATTTGCTCTTGAAAGAACAACATTCTTTCAAGCTGATGTAAGTTAAGCCAAAAGGTAAAAATAGCGCTTTAAATTGTCTtACTGATTATGATTATACACTTGGTTGGATATAGGCGTaTTATATaGATTCAAGCTGGGAACGAGTTTTAATTGTTGGaTTAAAATTATAATCTATTGTGGTAAATCCTCAATTAAATCTTTTTACGAAAGTATTTTGAACTAGTTGCAAAATTAAGCAAGGgTTTGCGAATTGCAGAATCGTTGGTTAAAAAATTCCAAACACAAGGTTTTAATAaCAGTGCTCTAATTtGTGCACGAGTAAACAaTTTAAAAAGCGCATAATCTTTATTGCGAACCTACATTGGCGATGAAAATATTGTTAaCTtCATGAGACTACTACGACTACTCCACAGTAATTTGT

>contig01144

AaaaTTCCTTCCAGGTCGATTGGAGACGATGGTCGACAATCTGATGGAGAAAATACCAGATCAAGAAGTGGCAATGCCAGAGAAAAATACTGCCGAGGTAGAAGGAGAAACTAAACGAAAGTTTCGTCGGAAGATCTGGGTTAATATGCTCAGGAAGGAAGCTGTGACCCTCTCTTCCAATAATGCACACAATAGGATACCATATTTTGTTGGCGCAGCAGAGGCAGCTAAACGATTGAGCGAATTAGAAGAATTTAGGCAGGCTCAGGTTATAAAGATTAGTCCCGATAAACCCCAGGAATCAGTGAAGAATATAGCTCTCGAAAATGGTAAAGAAATTCTCGTTCCAAAACCAAGACTGAAGTCTGGTCTATTCAATCTCGTTAGTCGTATCACTAATCCTACTGAGGAAGAATTAAAAAaTGTTGTGACGAGACATGGCATTCAACAACTCGAGAAACCAGTTGGTTTAGACGTAAATTTAAAGGTGGATCTCGTGGTTCTGGGTTCGGTATGTGTAAGTCCTACCGGCTACAGAATCGGTGAAGGTGAGGGATACGCAGATCTGGAATTCGCGATAATGACCAAGATGGGAGCCGTGAACGGAGACACGATcaTAGCGACAACGGTCCACGATCATCAAATtaTCGaCGaT

>contig01145

ACCATTTTTTtGGGGATAATTCCACAAATATTTTCTATTTTGAACTTCTTtCATATCTCATATATATTTTCAGAAATAGTCGCCATTTTGTTAATTTTCAACTTGCTAAATTAaCAATTTGACACAAATTTAGCTGCTCGTGAAAATTGAATTGAATCTTATACTTCAAATTAAAACTTTAAAGCTTTTACATATTTCAAGTTTTTAAACTTTTTAAACTTTAGAAATAAATCAATTGCAaTTTATAATTTtACACTTCATTTTATATCCCCTTGTaTTTCAAAGTTTTAAAAAaTAAAaCAAAAATTAAGAAGCATTTAGATGCAAGAAAATACTTTGCTGTGTGAAAGTCCCCTTCTTCATCGGAGTGCATTTCTAACCTAAAAATCGAGCAaTCTTTGATTTTtCCAGCAAGATTGAATAATCGAatAAAaCGTTTAAAAAAaTTAGTTACCACAATTTTTGTAACTATtAGAGCCCTTGaGGTTACCAGAAAACCTGAAAATTTGATTTCCGCTATTCACACAATATAAAAaTGTAATTCcATGTC

>contig01146

AAAATTATTTACCCTTTCAACTTTCCAGATTAATATtAATATGAGAAGATGAAAaTACTGAAGCtGAGGATTAACTCTAAGAtCGTATTGAAAAATAATATCTTTTCTCATCTATGTTTT

>contig01147

ACCTCACTCGAAAGGATTAGAGATCTGGAGCAGATTTtACAAAGACAAATTTCACTGCGAAACAAATAAAGAAAATAGGAAAATAAATTGCATGGTGCTGCTTATTTTGCATAATAGACGCATATTTAAAATATTTAGAAGCACTAGAATTACTTTCAACTAAAAATTAaTTTGACAATACTGAAGTTCATtGCTGTGGATCTATtGCTATTGtAAGGgCCGGgTTTtCATCTGGGATAGATGCTATTTATCAATTTtATTTCTAAGACATGGGctCAAACCATCCTTCATTTGTAACTAGAATTTAATTATaTCTTAAGTTTtGTAGAAAATATTTTTGCACTCGGAGAACAGAGTTTtATATATTTTTTGAGGACAACGTTTTTtAAACTAAGAGCAaTcTTTAGAAATTTTATTCATTTTTTtAAAGTTACGAATTAAATTAAGATATGATTGAATTTATGTCTTtCGCACATTGTTATATGTTTATTGGAAAGTTGCTTCACATTtGAAATTGAAAGATGTAGAAGTGCAAAGTTTtGGCGTGGAATTCAGAATAAGGGACAGAATTTtAGCATTtGACGGAAAACGTGCCTCCGGCAAGTCTGCAACCTACAAAAGTaTGCCTAAAATTGT

>contig01148

AGTAATCCCATGGGCTTTGAACGAAAGCAACTACATTAAGTCATCCTCGCAAAAATTGGATCCACAGAAGACTGTATTTGTCGGAGCTCTTCATGGAATGATGACTGCTCCTGCATTGGCTTTGATCATGAACGATCTCTTTGATGGAGTTATTTACGCTGGAATTGACACGGATAAGCACAAATaTCCGATTGGATCCGCCCGTGTAACATTTGACAACACTTGCTCATACATTAACGCCGTATCTGCCGCATTCATTGACATCAAAACCGCCAAATTCTCGAAGAAGATTCAAGTAGATCCTTACATCGAAGATGctCTCTGCTCCGCATGCTCCGTGCAACAGGGACCCTACTTCTGTCGCgAATTGATGTGCTTCCGTTATTtCTGTCGCAACTGCTGGCAGTGGCAACACTCAACAGATTCAATGCGAATGCACGAGCCGTTGACTCGCAATTCGAAAAGTaGCCAGATGATTGGAATGTCATCGAATTTGGGAGCCAGCCTTTCCCGATTGTCCAGCAGCTCTGCGATTTAAAAAGCTCGTAGTCGATGCAGAGCCACGTTCAGTTTGTTTTGACTAGTTATTTGTATACGGACACTGCACCGATAATAATTTATTCTCAATTACGGTGCAAGCTCAGTTTTATCGCAATGAGCTGCGGGCAGCTGGTCTTGACGAAATAGGT

>contig01149

ACCCTTtCGAGTGTAATCCTTCGACCAAGAaCcACTTtATAAGAACTTCACAAAaCGAAGGCAACGGTTCAATTCATCTGATGACACATTCTAGAAAGCTTAATTGAAATAAAGTAATGTTGTGTCAGTGATtGTTCGGgTTCGAGTTCGAAGGATTGGAaGAaTCAGCGGCAGAGTGACTGTCTTGGTATGAGAACTgAGGGAAaTGCTCTTGCTCTTCGGCTATcGAaTTtAACCCAcTCCCgCTACTTAAAaCACCGCCTTGATCGGGAGGAGTTAACTCTACGCTTTCACCAGTGAACATTCTGTCGAAGTAACGAGTGTCTATATCAGATTCTACTTGGGgCTTGAATGGAGGAGCAATTTTTTtCtGCACTAGCTCGGACCAATCTACTGACGAGAAGAAAGCGTGGTCCATTATGTCTTTtGCGTCGTTTGGCCCTCCACCGAGTCTCTTGTTTGGAtCTTTTTTCAGCAAGCCAGTTAGCATGTCTCTCG

>contig01150

ATAACAGTAAATATGTGCCTTATGCATTTAATGATACCGGGAGTAATCGACTCAGTGATGATATTACAATTTCGCATTTTACTCATGATGTTTATGAATGTTCGATCGATTATCTCTTTATGCTTAAAaTTATATTATTAAAGTTCTACGTGTAGAAa

>contig01151

TTTATGTAAGTGCGCGATATTTACAAGATCGTCAAAGTAAATGCCATCATATTCTACTATtAGGACTGCATTGACGTTTCAGAGCACTTGTGTGCGTCGCACGTGGGACTCCAAAATGCGATTAAGAATGTCAAGACACCACGCTCAATTCAACTCGCGGTTCCTGGTTGAaGGAAAAaTGGAGCATACACGTGTCCAGCTACT

>contig01152

ACTTTCCTCCTCCTCGCTTCTTTCaCTCTCaGCTTCACTGTCGCTGACATCAGAATTCTTCTCTTTCGACGACTCCTTAGCCACCTTATGTTCACTCTTCTTTCTCGCATTAGGCGTATTATCTCCTTGACTCGTCTGaTTTTCTCCTTGAGCCTTTTCCTGTGACGCTGCTGCTGCTGCTGCTGCTGtgTTTTACCTACTTTCTTATTACTACCTTTCTTATTGTGGGACTTTTTCTGTCCCTTTTGCTGCTTGAGCCAAGCAGGTCGCTTGACAGTTTGAtTCTGTTCTTCGGACGGTTCTTTTtCTTTGACTACGTCCACTTTGGCATCGTCtATTATCGTCTGTTCTTtCACTGACTCGTCTCCAAAGaGATGCTTCATATCTTTGCGAATCAGCATTACAGTTACTGTCACGAC

>contig01153

TTTGGGGTAGTAATGGCCTTTtCAATTGCAGTGATGAGTCTTACCCTATAAtAAAAaTGCAAGGACAGCCATTGGCGATTGATtACAATCATGATATGATAATTGATCTTTTTGGTTtAGACTCTgAAAaTAaTAGAATGTTTTGGATATTCAATTGCAATAGAACAACTCCTACCGCGATCAAAaTGAAGGTTCCAGCTGGTCACGAACCTTCTATAAGAAAACCCCATTCTAATGCATTCCTTGATTTCAATGGTGATTTTATTCCAGATCTCCTTGTAACTACCGAGCATcACTTtGAAGTTtGGTTTGGAAACGATAGTGAAGGTTTTTtCCATTCTGGTGAtATACACTTACCTTATAATTTTACGATAGGTGAAGGTTCAACATTCCCTTTCCACATAGGACAATCTTTATACGTAGATGTGGAatCAaCT

>contig01154

AAAATAGACCATGAAGCGAAGACCAGTGTTAAAaGATGCTCCT

>contig01155

GGCTCTTTGTTATATCAAC

>contig01156

GCGACGGCTGCAGCTTCCGCTTCTTCCGCGAAGGTGACTAAACCCGCAGCGAAACCCGCTGCTGCAAAAAAACCCGCAGCAGAAAAaGCCGCTGCTGCGGCGAAAAAGGCCCCGGCGGCGAAACCCGCTGCAGCAAAAAAaCCCGCAGCAGCAAAACCAGCAAAGCCCGTTGCTAAGGTTGCTGCTAAGCCGAAAAAGCCCGCACAGTCGGCCAAGAAAaCACCCAAGGGCGGCAAAGCTCCCACTACTCCCGTTGCGAAAGCTTTGAAGACACAAAaGAAGATCCTTAAaGGTGTCCATGGGTCCAGAGTCAGGAAAATCAGGACTTCGGTCCACTTCCATCGGCCAAAAACGTTCCGTCCACCAAGGAACCCGAAGTATCCAAGGAAAaGTGTTCCGAGCAGGAATCGCATGGATGCATTCAACATCATCAAGTATCCACTGACGACGGAAGCAGCGATGAAAAAGATTGAGGACAACAACACCCTGGTCTTCATTGTGCACACGAGGGCCAACAAATATCATATTAAGGCCTCCGTGAAGAAGT

>contig01157

GTTATAATTTGAGAAT

>contig01158

TGTCCTCTGCAAGTAATCTCAAtATTTTTgAAaG

>contig01159

ACTtCtAAAaCAACCTCTTCAACAAAAaCCATGTCACGcTATCGGGAGTGGGACTtaTCGTGCAAAGTTTaCGTTGGAAACTTGGGCAGTaGTGCCAGTAAACACGAAATCGagAGtGCGTTCAGTAAATATGGTCCGCTAAGAAACGTTTGGGTGGCCAGAAACCCACCTGGATTTGCATTTGTCGAATTCGAAGACCCCAGAGATGCCGAGGATGCaGTCAGAGGATTGGATGGAACGCGTtGTTGTGGCACAAGAGTGCGAGTGGAGATGTCATCTGGCAGAaGTCGCCGTGGTGGTGGAGGACGCAGACCAGGTCCCAGATATTCCTCTAGGTCCAGAtCCCGgaGTCcGCCCAGGAAGTCGATTGGTCGCTACCCGAGATCCTACTCCAAGAGTCCACGAAGATCGCCGATTAATCGATACTCCAGGTCTCGGTCCCGCTCACCACGTAGGAGATCTCTGACGCGAAGCcGAAGCCGAGATCGCCGTTCACGTTCGGACTCCCGTGACAGACgT

>contig01160

TGGGgaGTTAAAGCTCAACATGGCATCCCTGAGAACCATTACTGCCTTCCTCATAGTGTGCAACATTGCACTTTCTCAGATGGCCGGTTCGGAAGCCCGTCCTCATTACGAGCCAGATGAGTCAGATGAGCCAGATGAGCCAGTTAAGCCAGTTAAGCCAGTTAAGCCAGTTAAGCCAGATAAGACAGTTGAGATAGATGTGCAATTTGATCTATTTAAACCACTTGAGATAGTTAAAAAaTTTGTACCATTCAAGCCAGTTAATCCATTTAAGGAAATTGAGAAATTTAAACGATTTAAGCCAATTGAACCATTTAAGATATTTGAGAAATTTAAACCATTTAACCCAATTAACCCATTTAAGCCAATGAATCCAGTTAAGCCAGTTGAGGAATTTGAATcctcGCCGCCAAATTACCATGACGATTCTCGCAGATAATTAATGAAACCTTCGTAGGAaCTTTCACAATTGgAaTCAATTCAAGTTGTTGAACTGCAACGGGAAAACCAACAAATATACTTACAAGTATTATGGCTCGCTGCACTTTTCAGGACCAATGTTTTTTTATTATTTtATTGGCAATTATTAATCCGATTGTAATTTATAAAaGAATAAGAAATACTTGTAAATGGTGTGTTTACTATATGCTATTTCTAAAAGATGGTGTACAtGTGAGCAATAAaTT

>contig01161

AACCTTGGAGGACCGTAATGGTTCCTGACGTAGGTAaCGAGGTCATTTCTGGAGATTGACTTGATGTTCTtGGTTGGTCCCAAGATTGTCCTTCCCAAGGGAGTGCCTTGGAAAGCAACTGAgTGCAGGTGGTCAAATACGACCTCCTGGAGaTTCGTCTCGACGTCCTGCATTTCTCTAAGAATTACTCCTCGTTCCCTCTCTATTTCGCTCTCGCCCAATTTCGAATTCTGAATTATGTCGCTAAGAATTtCGACCATTTtCGGCACGTCTTGCGACAAACACCTGGCATAGTAGACAGTCTGTTCCCTGCCTGTGTATGCATTCAAGTGAGCTCCCATGTTTTCGATTTCTAATTCAAGGTcGATTTGCGAGCGTTTCGCGGTTCCTTTGAATGACATGTGCTCGAGGAAGTGAGCGACTCCATTATTTTCATCGGTCTCATAGCGACTTCCGGCATCAATCCAAATTCCTACAGTGGCAGTAGCAGCTCCACTGTCTTCACTGGCTACTCTCATTCCATTGTCCAAAGTGGTAACCCTGGTTGCTGGCTGATTTATAAGAATTTCTTGCAGCGAAGCCTTCGATGATCTCCATCGC

>contig01162

ACAaCGtGGATATtCCTCGGTCCGAATCAACCAAAACCCcAAAaCTTATGATGGTGAAGAAGCGCGGAATAGTTTGGAACTATTtCGAGAAGAAaGTAGACGGCACACAAGTCaTaGCTTTCTGCAAATTCTGTGATCAGTCCTACACACAGAATGCAACCCGAATGGAAAAACACTTGGAGCGCTGTCCGAAATGCCCAGACGACATAAGGTTGCAGTTCATGCAAGTGGGTGCAACCAAAAGGTCCAAAGCCAGACTTCTGGGAGACACCATGGACGCCTGGGTGAAACAAGACGGTTCTATCGAAGGTAATATTACCGAAACCGAAATTGAAGAATTAGAAAATTGGGAATACAGAACTCAGACTTATGCAGATGAAGGTTCGACTAGTGCTCCACAAACCGTTGAACTGATCATGCAGGGCGATCAGTCCTGGGTCGGCcGTGAAACGGAaTTAGAAAaTGCAGAGCaTGAAGAAaCTGTAACGAAAaCCGAGTGGATCGCTAGTCAGAGCAA

>contig01163

ACCGCTGGATTGAGAAACACAGACGGGGTTGGGGGTGGAGGATTGAGTGCTCAagAATAGCTCTcGAcACCCcAACCTATACCTCAGCTCTCATAAATTCAGCGTCCCGGAATCACAGATCGCGTCCCAAGCTACTTCAGAATAAAaCACACCGCCAACTGGTGCATGGTATCGTCAAGGTCGTAGCCATTCTCGCCGACAGAATTCTGGTTTGTAATATCGGTTTCATCGATCATTCAATTAAGACTGAGTTATCTTGCTTCGCCTATCGTCTtACGGAAATCTTATGCCACTCGAGGATCGTAACATACTTAGGATATCCGACGAACGGAGGGACTTTAGATGTGAAaTTTTTCCTATTCGAAATGTCTGTCGCGTCGACGGTATTCGAGATGCGTCTTTCgAAGGGGTGCAGATCGCTTTtGTTCCAAGGCATGCGAAATGCGATACCGATTTTCCTGGGATTTCTGTTAACACCAGGTCCCGTCTCATCTTGCAGATGATCGTGGTTTAATA

>contig01164

GGTGCTCAATCCATCACATAAATGATAAGAATGAAAATTAGC

>contig01165

ATTtGCATTtAGAATTtACGACATGGATAATGATGGTTtCATCAGTAATGGGG

>contig01166

GTATTAAAGATGATGGTAGGGAATAATTTGAAAGACACACAGCTGCAACAAATAGTCGACAAAACAATACTATTTGCTGACAAGGATGAGGATGGAAAAATTAGTTTCGAAGAGTTTtGTTCAGTTGTTGGTAATACGGACATTCACAAGAAGATGGTAGTTGACGTTTAAGTAATCAAACTCTATTTGACATGTAAAAACTTTAAACGATGCTAAGGCGGTAATGTAATTTCAACTTCGTGAATTCATTACTAATAATAGCTTTATCGGATAGTTATAAGTAAATTAAAGATCTG

>contig01167

CTATAATAATTAGACAACGAtCACTAAATAAACGTATTTAGTCTTTTGACGCGCGTTTGAAGAGCCGTTGAAATTGTTTtACATTGGCGCGCGACCGCATGAAATTATTGTATTTGCGCTATAATGTTTGCCGTCAATCATTGAATTTGAAATTCTTGAGACCCCATGTTTTTGTCGtCTTTTTtAAATTCTTACGCTtGATTGTTCCCGAGACGAAAATTAAAAAAaTTCGATCCCTCGTGGAATCGAATGAGCGTAAAAATTG

>contig01168

ACTCGCTACTTGTTTTATTAACGCTAAGGCCCGTCCAGCCGAAAATTACGATGCTCATGGAAGCGCTGGAAGCCTTTCAGGACAACACCAATCGGAATATAATGGCCAGATCGGCCAAGAGAGCGTAAAAaaGTAGCTCAGAAAGCTTATCaGTTGATGGacAATCAGAAAGCGGACCAAGTTTTGCGTTTAAAaCCTTATCAGGAAGTGcTtCATTTGgtATTTCAGCTAACaTAGAAAAAAGCGAATCGGGTAAaGAAaTAGGTGaTgTGTCAGAGTCAATGAGCGGATCAGAGAAGAAaTCAGTTGATGGACAAGGTAACGAAAaCGGTATGAAAACTGTGACAATTGAGATGTCAGGTAGTAAAGTAAAaGGTGGTAGCTCACATAGCAAAGATGTTGATGTAAAAGTGGACATTTTTCTGTTTGGTGGCCATCATAGCAAAT

>contig01169

ACAAGCAAAGTATCAAGTATTATTAAATTATTGTCTTAATATATTGTTTTATTTAACACTCAAAGCGAGGCGATCCTTTGTTAATcaGCaTCCAGTAATAAAGAGATTAGCACAATACCGGCAGCTGCTAAACCAGCTTGATTCATCCCGATCTCACATGtCGGACAATCTGAAAAAAaCATTAGATTGCtCTGATATTCGAAAAAAAaTGCTTATGCCACAGAATTTTGTCACGTTCAAGACCGAATCTAATACTGTTTTTTtAAAACAAaTAAAAaTGAAAAaCACATTGGGGgAAAAAaTCCTATTGAAGAAATTGAAGGAAAATTAGTAGATAACaTGAGTGGCAATGAAGTAATTGTCCAAAaGGGCAGAAGAATGATAACATaTCAAATAT

>contig01170

AGAGCtCTtCCAAGTTTTTAAAaaCTAAGGAATGAATGGCGTCGCAaGAGGCGCCATTAATTGTGTTAAAAaTGACGACCtgCCTGAATCAGCACATTTAGAAGATTTTGATtGTtACTGGACAGATTTTCtAAGGTCGTTGACAAACTGTATCTGTTGAAAAACAGCCTTCATTGCAaCGCGtAAAACtagTAGgACaCTCGGCCATAgAaCCAtGAaCGAATtCCgCTtAtCATACTTATCACAGAGTAAGTATAACTCGATCGAACGCTTtCtGCATTCACTTTtGATCCCTAAaTTGATCCCACCGGgACCcGtAAGTGGGAAaCTtGCATTCGATCACGCGCCTCACGCTtAGTTtAaTTAAaTTAAaTTAaTTAAaTACCTAaGCTTAAAGTGACTGTCAAaGAaGAaTAGCTGTGCCTGAaCGctCGACTGATAGCGACCATGAGAATCATGACACTTGATTATTCTGCGCCTTTCTCAAAaGAGAGTGATATATACCGCGTCTTGAAGGATATTCGGCTTCGTTAATACATTGATACGCTTTAGTCGTTGATATTTCGAGGTTTTTCGTGTATCGGTCTTATGTTTTGTTACCGGGATTTTGAAAAAAaCAATGTAACGATTAAAAGATGAAATATAACACTGATCTCTTAGGCATTTTCAAAGATTCTGAGAGT

>contig01171

CTCCAACATTCGTGTAAAACAtATAAAATAGGAGATCGTCCTTATACCGATTtAGTTTAACAGGAGCCAGTTTCTCCCTGATaGAAGCGTTTATGAGATATtCTGGTGGAACGTGAAAGTCAATGTCTTGTGGTCGAcAAGTTGTTTCTGCCCAAGGtCCtCCAAAaTTCTGATAGAGGTTTtCCGTAGAATTAAGaTTAAGTCCTAACGCAGTAAGATCCTGCCCTAACGCTAGGGATACGAGATTTtGATCCGTTTCTGCCGCCCTAATAAaGGTCAAAaGACCcACCATTCCGAATTGATCTTTGACCATACTCGACGGTATATTCGTTACTTTACCATCaGgAGAGGTCTGGATCCCTCTTTTCGACGCCAGCGACTTTtCCAAaCCTGGTGTTCTGTTGGCTTGCAaTACGTCTTGACTAATTTCGGGACCAAGACCAACATTCATACCCTTTtCTCCCGACACGCCACCTCCAGGCGAAGGTCCTTCCCTACTTTGAGTTCCGGGCAGAGCGGGGAAGTCTTCCgaGCtCATCGTAAATTCACTCGGTTCACACGTTGGCT

>contig01172

CATTCATTTTGCTAAACTTCTCATCATTTTCTCCATTCTCAAATTTCAACAGCTCGCGTTTGGTTATCTCAGATAAGATAATAAGCCAATTCtCAATAGACTTCATTTGCGTTCTCGTATGGTGTTCATCACTGATTTTCATtATCTGGGAAATCGATTCCgAAAGGGCAGGAATCAATTGATCGACATTAAACAAATCTGTAAGGGCGTTCGACTCCATCGAAGAAATTGTAGAaCAAAaa

>contig01173

ATTGCGGTGTCGTTTCCTCGTGGTCGAAAATTGTGATTGCATTGTTCAATTCGCGTTTTAATAATTGTAACAAGTCTAATATCGGCCACAAATTTTATTCCCTTCTACGTTGAAGCCCCTTTTCTTTGCAATTTCAAACTCCCTTGCTTTAGAAACAAAATACAATCATGTTTCCGGGCTCCGCTCCAATCGTCGTTCTGAGCGAAAATACAAAACGTGATTCTGGAAAAAAaGTTCAGAGGGAGAATATCCAGGCTGGAAAGGCAATCGCGGACGTCATCAGAACATGTCTGGGACCACAAGCAATGTTGAAGATGTTGATGGACCCGATGGGAGGAATCGTGATGACCAATGACGGAAATGCGATCCTCCGTGAGATCACAGTTCAGCATCCAGCTGGAAAaTCTATGATCGAAATTGCCAGAACTCAGGATGAGGAAGTCGGTGATGGAACcACATCAGTCATCGTCCTCGCTGGGgAAaTTTTGGCAGCTGCAGAGCCATTCCTTGCACAGGGAATGCACCCCACAGTCATTATCCGAGCTTACCGTCAAGCTCTCGAGGACATGGTTGCTGTTCTCAATGATCAAATAAGCATCGAACTCGaTCGCACGGACaGAAAAAAaCTCGCGgAAGTCGTCAGGTCATGTGTTGGT

>contig01174

ATTCTGGAATTCCCTTTttCGTCTTGATCAGTTGCACC

>contig01175

ACTTTTCtGAAGGCCACCTACTCGCTCCTACTAGGGACAGTGTTTTCTTTGGATCATCCTTATTTCGTAAAATTCATTCGAAGAaCAATGATCCTCCGGAAACTCTTCTTTGACACGTTAAAGTGATCATTGCTTTGTGGATGGCTCCGCAGATAGTTATGATGTATCAGTAATAATAATtACTCTTTCTTATAGACTTCTTGTAGTCTTAAAGGATGTTGAGTTGGGGAAGAGGTCTTAAGATCCTAAGGGTCGCGTTCGAAGCAATTCTAGAaGCTTTCACTTCTTTTtGTTTTtGTtGTTTGGACCCCTGGGCGGTGTCACCGGCCGTCCcgCATTCAGACCTCCATACGTGAACTTCCTCTTGTCGGCTGGCTTCAGAATTTGAAAGCTACACATCAGCGTCTCGTCCACTGACATCATGGCACCTGCGTTATCGAATTCACCACAGTAATTTGGCGCCGAGAaTAAGGTTACCAaCTGCCGCTTGGCAAAGAACTCGTAACCaTCTTCAACCACCTGATGAGCGCGACAAA

>contig01176

TTTGTACTTTTTAAaGTCAAAAGAATGATTCAAGTAGGAATCATTCTTTTACTCTGCACTGGAGCTTTGGCACAAGTTAGTTGGTTTGGTTTGTGCCCGGCTAAGGTGGAAGGTGTGCGGAATTTTAGTGAGAAGGA

>contig01177

ACGAAGTGCAGACATATACAAAAAATCCTGAGAAATGCAGATATGTAAAATACACAAAGAATGGAGATGATACAGTAGGAGTTGTCTTTGGGAAAGTCGACCCTCAAACTGCACAATCAATAACATCAAATAAAGGCCAACTGAATTTtATTGGCC

>contig01178

ACCGCAAGAAGCAGAGCGATGTGCTTCGTTTCTTGTTGCGTATCAGATGTTGGCAGTATCGCCAGATGACAAAAATGCACCGTGCACCCCGGCCTTCCCGCCCcGACAAAGCACGTCGTCTGGGATACAAAGCCAAACAAGGTTTTGTCATTTTCAGAATCCGCGTACGTCGCGGTGGTCGTAAACGTCCAGTTCCCAAGGGTGC

>contig01179

TTCTCCTCATTTTTATTCTGATCCTCTTTTTTA

>contig01180

ggggACGCACGAGACGAGCCACGAGAGAACTGGTTCAGCGCTGATCAATTTTTCtACGATAAATTGGTCATCTCGACCAGGAAAaTAATTAAACAATGGCAAGTATGAAAGCTGAAAACCCGGAAAaGCCTGGACCCGATGTTGCCCCGGTTCATCGAATCAGGATAACCTTGACTTCCAGAAATGTCCGATCTTTGGAAAAAGTGTGTTCGGAGCTGATTAACGGGGCAAAGAAGCAGAAGCTCAAGGTGAAGGGACCAGTGCGAATGCCGACCAAGATCCTGAGGATCACGACTCGCAAAaCTCCTTGCGGAGAAGGTTCCAAAACCTGGGACCGATTCCAAATGCGAA

>contig01181
[truncated: 534,924 more chars]
