# Supplementary material for: Gall Wasp Transcriptomes Unravel Potential Effectors Involved in Molecular Dialogues With Oak and Rose
Source: Front Physiol. 2019 Jul 24;10:926. doi: 10.3389/fphys.2019.00926 (PMC6667641; doi:10.3389/fphys.2019.00926)
Supplement: Supplementary file 3 [file Data_Sheet_2.docx]

**Supporting Information**

Article title: Gall wasp transcriptomes unravel potential effectors involved in molecular dialogues with oak and rose bushes

Authors: Sébastien Cambier, Olivia Ginis, Sébastien J.M. Moreau, Jack Hearn, Graham Stone, David Giron, Elisabeth Huguet, Jean-Michel Drezen

**Note S2 All contigs identified from the sequencing of transcriptomes from *D. rosae* venom gland and ovary.**

Sequences are presented in FASTA format.

>contig00001

ACAACAGTTATGATTACTAGCAATAGAGGTCTGGTGACGTATGATTATTTTTGGGATAAAAATGTGAAGAAAGATAATTACTTTGCTCTAGTCGGTTCAGAAGTATGTCCAAATAGAGAAAAAATAAGATTATCGAATTAGTTCGACAGAGTCCTCTTGGGGTAATAGTTCTGGAAAACAAaTTGGAAAaGACGAATATTTTAGATCAGGTCTGCCGATAGATTTTTGCATTGGTGAATT

>contig00002

ACGCCTATTtAGCAGGCTAGAGTCTCGTTCGTTATCGGAATTAACCAGACAAATCGCTCCACCAACTAAGAACGGCCATGCACCACCACCCACCGAATCAAGAAAGAGCTCTCAATCTGTCAATCCTTCCGgTGTCCGGGCCTGGTGAGGTTTCCCGTGTTGAGTCAAATTAAGCCGCAGGCTCCACTCCTGGTGGTGCCCTTCCGTCAATTCCTTTAAGTTTCAGCTTTGCAACCATACTTCCCCGGAACCcAAaGCTTTGGTTtCCCGGAAGCTGCCCGCCGAGTCATCGGAGGAACTTCGGCGGATCGCTAGCTGGCATCGTTTATGGTTAGAACTAGGGCGGTATCTGATCGCCTTCGAACCTCTAACTTTCGTtCTTGATTAATGAAAaCATTTTtGGCAAATGCTTTCGCTTCTGTCCGTCTTGCGACGATCCAAGAATTTCACCTCTAACGTCGCAATACGAATGCCCCcATCTGTCCCTATTAATCATTACCTCGGGGTTCCGAAAACCAACAAAATAGAACCGAGGTCCTATTCCATTATTCCATGCACACAGTATTCAGGCGAAGATAGCCTGCTTTAAGCACTCTAATTTGTTCAAAGTAAACGt

>contig00003

CTGGGCCGCACGCGCGCTACACTGAAGGAATCAGCGTGTCTTCCCTGGCCGAAAGGCCCGGGT

>contig00004

CCCAGTAAGCGCGAGTCATAAGCTCGCGTTGATTACGT

>contig00005

CCCTTTGTACACACCGCCCGTCGCTACTACCGATTGAATGATTTAGTGAGGTCTTCGGACTGGTGCGCGGCAGTGCTTCGGCATTgCCGATGTTGCCGGGAAGATGACCAAACTTGATCATTTAGAGGAAGTAAAAGTCGTAACAAGGTTTCCGTAGGTGAACCTGCGGAAGGATCATTAACGTTACAAGTTGCTTTGAAGCAGCGTTTGTATCAGATATATATATATTTGTAATATAATACATATATAAAATTTGAACTTTAAAaGAAAATTGGCAAAAAGAGAGAGATAAGTTGAACGACAAAATGAATGAGAGAAGTGTTGAAGAAGAGAAATAGGATAAATCTCTCTCTAACTTTTCATATGTTATTGTGAATAAAATCAGTTATTACACAAAAaTAATCGTGTAAACTGTAAAGACAGAGAGAGATAAATTGAGAAGAGTAAAGAGCTATCAGCAGCAGCAGCAGCAGCAAAAAaGAAaGAAAGCAGTCTGTAGTTAAAGTTAATTTTGAACTATTGGGTATAACAATATGTGTATAAATTGAAAAaTAAGAACTGAAAAAGAGATGATGTAATAAAACGAAGGAGGATTAAAAGTGGCTTAGGAAAGTGCCT

>contig00006

CCAAGACTTGTCTGCAAATCCAATTGTAGAAAGTCAAAAAATCCGCTCATTGATGTAAAATGTTAAAAGGACTACAAGAATTGGACAAATCAACCTATTCCACTCGACTTTAATCCTATAGTTAGATACAATTGAATTTAATGTATGTAAAGTGAATTCTCTATAAAATCTGTCAAAATCTGAGAGGAATACACCACTTCCAATTTAAAAATGTTTTCCGATTTTATTATTTTCTTATTATATTGTTGTTCTTCCTTTAATTTGACATTTAACCACAATTCAGTGTCAACTTAAAATATATTGAACAGCTTTGCCCTAATTGTTCTAAGATTTCCCAAAAaCAAAAAaTATGCATAAAGTTATGTTAAGATTAAAATTTGTTATGTAAAAGTATGAAATTCAATTTTATGTGACCGAGCTCAACAAAATATAACGCATGACAGGAATGCTTATAATTATGTGAATGTTTATTTTAAAatGG

>contig00007

GGGgATTCTTGGATTTACTGTTCTATGAAGAAGATTAGTTAATATTTTCTGCAGCTTACTAATTGAGAATATACATCAAAATAAATTACCAAGAAGCAATTGAATATAGAAACGCCAACGAAtACCTGTGTTTCTTATTTTAGTATGAATACACATAACATAATGCTACGACT

>contig00008

ACAATATAGTTCTCGGCAGAACATTATGTTGTTTTTCATTCTTTTTGCAACGTCTGTACGAAGTTCCTCGAAATTACGTTGCTTATTCCTTGCTATATCATTCGCATTTGCTTCGTTTGTATTGGTATTCATACACAGCCTGCAACCTCTTATGTCTTTACATGGTGGCGCGCATTCTGTACCTTTAGATTCGTCTTCAATGGATCCAATGACTGATACAACAATTAGTAAATTAATAACGAACAGAAAAAATATAGTAGACTGGACCATTTCTGACCAACGTGTTGTCCAGAATATTACCCGACTTTATAGTATAAGATTCAAGAGCTCGATCAAGTTCTTAAATATTAATCTTAAATATTCACTAGTTACAAACTCTTTAAGCACTGTGCACCTCGTAATGAGCAATGAGTTAACACAGCACGCTTGTCTCGAACGTTATCAATGGCAGTCCCcATGT

>contig00009

CCATGAAATAACTCTTTGGGCTTTGACAAAAGATCTAAGCACATGGAAAGAAGCTTGGCCAAAACTGAGCTATCATTTGAAACGCCTCGAAATAGACGCAAgATCTTGAAACGGTTGACCACACTCGTTGTTATCCTCAAGAATAACATGGTTAGGATACGGCTTGATCACAGGAATATATATGTCGAtGAATAGAGTTTTACATTTTACTTTCTATGATTTAAAATCTTTACATACTTAGGGCCAGTCGATCCAACCTTCTGATAAGTTCCAGATGTATTTATCGCATGTTAAATCTTATGGAATAAATTTATTTGGAACTCATCAGAAGCTGTATCAAGCGgCCCTTACTTTTtCAAATTATAAGTATTATTTGTCTAAAATCTGAAAATTTTAAATTTATTATTTTGTTTTTGTCTTgATATGAGTTAGCgATTAAGATAGTGCATAAGCTGTAACAAACGATTTTgTTAGCTATTGTGTGCAATTaTTTTTTGCAATA

>contig00010

ACCACTATGCCTTTTTATACACATGCACTGTAGCAGTAACTAG

>contig00011

GGggATTAGCAAGAAGATTTTTGCACCCACCAAAATGTCTCCTATCTTGTTCCTATTACTCCTTATCACAGCAACCGCTTTTTCAACTGAAGAAACAAAGGGCTTGGTCTCCACTCCTGAGCAACCCTCGATGACCAGTAGCCAAATTATACCTCAAGTGGCTAGTGGGGCTACGGATGCTCAATGTGAAGGCTACTGTGTAAACATTTGCAAG

>contig00012

aTtAGTTGGATGATTTTTATACCCAGCAAAATGTCTCCTATTGTGATACTTTTATTCCTTATTGCAACCGCAACTGCTCCAAAAAATGAAGTGCCGAGTAAAGGACAACCAAATCCAAAGGGGAATGATTGTAATTCGATTTGTTTTGGAATTTGCGAGACATGTAAGAACCCAGAATACTGTCAAGAAACTGTACCCACGGATAAATGCAAAAGCACTTGTCATCACGGAGGATATATAATTTGTTAAAAATATTATGAGAATTGGACACTTCAACTTACTTCACACAACTTTAATCTCAAAATTATGTTCAACTGAATTAGATAAATGTAATGTAAATTCATCTCTATTAAACTCGTCAAAGCCTGAGATGATTACACCACTTTCAAACGAATTAAAAATATGGTGCAAATGTTTATAATTTTGTTAACATTTATTTTTAAAaTAGGAAAGATT

>contig00013

ACTGACCCGAAATCAATTTACTGTCAAGATAAGAAACACACGAGAAAGTGCGGTCTCGATTGTAGAAGTTCCAAAAATCCGAAAATTGATATAAAATGTTAAAAGGACTATAAGAATTGGACACATCGACCTATTCCATTCCACTTTAATCCTAAAATTAGATACAATTGAATTTGATGAATGTAAaGTAAGTtCTCTATGAAATCTTtCAAAACCTgaGAAGATTACACCACTTTCAATTTAAAAaTATTTGCCGATTTTATTCTTTTCTTATTATATTGTTGTTCTTCCTTCAATTTGACATTTAACCATAATTCAGTGACAACTTAGAAaTATATTGAAAAG

>contig00014

AAAAaGGTAGCAAGAAATAGGGGgACgATTGTAAGATCTATGCTTGAAGACA

>contig00015

ATGTCTTCTGTTTTTGCTTTGGACTGGATCTGAAGCTCAAATTAGAAAATATGTGGCTCGCTGTCCGAAAGTAGTACCTATGAAGAACTTTGATTTTGATAAGTTTATGGGTGTTTGGTACCAAGCTATGCTAACGCCACTAGATTTTATACATGGAACATGCAAAACCGATACGTTCACAAAGGTAGCAGAAAATAGGGGGACGATTGTAAGATATCGGTTTGAAAAAAaCGATCATGTTGTGTTTAATGGTGATGTTACATACGATTATTTAACACGTACAT

>contig00016

GAAATAACTCTTTGGGCTTTGACAAAAGATCTAAGCACATGGAAATATTCTTGGGAAAAATTGAGCAATGTTTTGAACCGCCTCGACCTAAACGCAAGTGATCTTGAAACGGTTGACCACACTCGTTGTTATCCTCAAG

>contig00017

AATcGCATGGTTAGGATACGGCTTGATCACAGGAATATATATGTCGATGAATAGAGTTTTACATTTTACTTTCTATGATTTAAAATCTTTACATACTTAGGGCCAGTCGATCCAACCTTCTGATAAGTTCCAGATGTATTTATCGCATGTTAAATCTTATGGAA

>contig00018

GTGTCTTCTGTTTTTGCTTTGGACTGGGTCTGAAGCTCAAATTAGAAAATATGTGGCTCGCTGTCCGGaTATAAAaCCTATGAAGAACTTTAaTTTtAATAAGTTTATGGGTGTTTGGT

>contig00019

ATCAGACAAAATTCAATCTTTATTCATAATGAACAATAAATAATAATAATTTtAAAATTAATTTGAAATTACAATTTTCTTGATAAATTTTACTGAATTACTTCATAAGTAATGAATAATATTTTAAAGTGACATGTTTTAGTACGGTGA

>contig00020

TTATTAATAATAAAaaTTaTAaGAACATCTCATAaCACTTTtGAaGATATTaTTGTAT

>contig00021

TTGATATTACCTTCTTCCATACTGATTGCGTTAATATTTTGACCCACTGCAAGAGTAACCTTGTTATTCTTACCAGGTGTTTTTTCAGAAACCCGGAACTAGCGAACTCGCTGTTTCCATCGGAATCCCAGGTGTGGTGAAGAATGTGTAATGTTGTATCACTTAACGACTTAAACACCCATCTTCCTTTGTACGACTTTTtATTTTTAGTAGGCAATGAAAACATTTTGAATTTCTTGGTTTCCCAGATACCTTCCAAAAAGGATAGCGATGTATCATTATCTTTCGATACCAAATCTTCCGGTGTCGAATTGTCCGGTACCGAATTGTCCGGTGCCGAATTGTCCGGTGCCGAATTGTCCGATGCTGAATTGTCCGATGCCGAATTGTCCGATGCCGAATTGTCCGATGCCGAATTGTCCGATGCCGAATCGTGCGGTGAAAAATTGTACGGTGCCAAAATGTTCAGTGACAAATCATCTGGAGTGGCTTTGGCTAATAAATGCACAGTGTTTTGCAAGAAAGTGATCAAGAAGATCAATACACCAAATCTAATAGATGCCATTTCAATAAACTTAAAAAAATTTCAAGTAATGTaC

>contig00022

ACATCCTGAAATATATCTATGTCTCCTTCTTGACGTATTTTGTCCTTATATTGTTTTTTTAGTCTTTGATGGTTCCTCTGGAGTTGACGCGAAAAAGaCTTTCTTTAAGATTG

>contig00023

ATCATATTAGGAAATGACCAACCAAACAAGCAGGATATTCAAAAGACAAAGCGAAAGTTAAAAGTGGGAAAGGATGGCAATCTTAAAGAAAGCTTTTTCGCGTCAACTCCAGAGGAACCATCAAAGCTAAAAAAaCAATATAAGGCAAAATACGTCAAGAAGGAGACATAGATATATTTCAGGATGTGCCCAAACAATGTTTGATGATTTATCTCGATTTCTTATTTTTtGTGTTTTTGTGTGTATACAATAATATCTTTCAAAAGTGTTATGAGATGTTCTTATAATTTTTATTATTAATAATCA

>contig00024

GAGAAGTTTCAGAAACTtACGTAAaGaCGGAGGGAAAATCTAAGAACTTAATTTGTTATAAaTTaCTTGGGgCAATTAAGatGGTTCGTATCATCCTCGCTGTGCTGATCATTTGGGCCGCTTCAGAAGAAGCTGCTGAGACAAATAAAGAGGATTACATGGTAAAGTTCATGTCTGGGGCGGAAACAAAATGCGTTGGATTCATTATAGATGGTAAaGCCATTGGATGTAC

>contig00025

GCAAGGTTGTGGTAAAAaCTTCGATAaTTCTTTtGAACTGAAGACTGAACCAAaTTACGTtGCtCTTTTtGgTACAAAGCAGATTGCAATTAAAAACAACAAAGCGACACTGGTGAAAGAAGGGACGGCGATCATGGCTATTAATGCGCCTGCACCGCCCATGAAACTACCAAGTGTTGACTTTGTGATGACTGATTTATCAGTGGATCATCCACTACAATACATTGGAATTGACAATTGTGGAAAAaTAGTCAACTTGCCTGTCAAAaTTGCAGATAAAGGAGCTTGTAATTCAAAaTTTTTAGAAAAGGGATATCATTATTGTGCAACTATAACGGTCGATTCCATAATTCCTTGTCTGAAAGCCGAATGCGGAATTTtATTTGACGGCGAGTCATTCGCTGGAATCTTTGTTGAACAAaGCGCAGGCTGTATACATTCATTTtATTTtCTTAGAGCTGATGTAATTTCCAAAGTCTATAATGAAGCTAAATCCAAATTATAAAAATACTTCAAACATTTCAACTAGTGCAGGACAACACAACACTAAAGTCTGGGTTTGAACCACATTTTCACTACACAGTGAAAAAAaGCATGtACTATTATTCACCTCTCGAACATTACGAAATaTACTATTTTGTTCGCTATATGCCGAAa

>contig00026

AACGACACCGACAAGGCCAGCTTCCGGT

>contig00027

ACATAGAATTATTTTTTGAAATTTGGGAAATAGTAAATAAGATAGAAATTGACGTAGATAGATTACTTTTAaTTTTGAACATGAACAAAAATTTCCGATGGATTTtGCTAAAaTTAtACTTTTTTtCATTTTTtAAATCAAATTTTTTtATTTACGTCTGTTTGTATCATCTCTACTATTTCCCGCATTTATAAAACAGAATCTTTATGTAATTTGAATACAAATATGTAGAAACCTGAAATTATGTAAAATTTGCTAATCTCTTAACTATTACCCATGCAGAAATATTTGTAGGACTTTGGCCAGTTTTCGTTACTTCTAGTCTGTCAAAGTCTTGGAAAAGAGTCAGACCAGACGATTTTGGCTTAAATTCAGCCACCCGAAATGTACATGCAAAAATAACAACATATTTCGTTTTtATATTGTATAAAAGATTGTATACAAAAaTGATTGTTAGTGTTAGTGTTTCATGTATGGTCGAATTTTtATTATATAATCCGATAGTTTCCACAATATATATTTtATTCATATTGATAATATTTtAATTAGGGTCAGATAGGGTATTTTCTGCACGGATATATACCCTTACTGTTaTTTCTCGGT

>contig00028

ACCTGTTCAGAAATTATTTATATAGAGATTTAATTTAATATTCTTAGAAAAAAAAaTCTTTCAAATGTTTAAATAATAAACTCAATAATAACCCATCGTTTGGTCTGAATTTGGTCAATGATAATTGGTTTCATTAAAATTTTTGAAGGTCAAGTTTTTGAATTTACATACTTAGACTCAAATTCACTTCGTAAATTTTTtGAAAAaaTaTTTTCACTTCTATCAATTTAAAATGGGGTaCcGAACATTAACTATCAGAACACGTGCATGAAaTATGCCGCTTTTTGAAGATGCAGAGCGTGCCGAAAaTTTCCAAATTCaGATTGgTGCGGGAAACTAATCCTCACCAGTGTCACGCATGCGCAATTCAAATAAAACATCAAATTtCctATGACCTTCCCcACCCCcGATGATAAaCTCCAAATTTGGGTCATTTtAGTTAAGTGAAACAATTGGTATTTGATTtGATTTATCGAatAAACTTTGACAACAATAAACCAAAAaTAATTTGTTGACGAACCACTTtCTGCTTTCTCTACAAGCACGATATGTGGCATATTTCTTACATATAAGGAAATCATGCAAATATCACACTTGTCTGTAATTGTaTGCTTACCTCACTTATTACACAAtATACCGTTCTGGATAGGT

>contig00029

GCtGGACACCATCTCTGGATTAATAGTATTTtAAaCAGCAATGGCAACGGAAaGTAAGGCGAAAATGGGGgAAAaGCAGATGGAAAaTTGGAGGTCATACaTGAGGAAACTGAATATtAAAATAGTGGATCTGAAA

>contig00030

CCGAAAatCGGATTTtCAAaGgAGGCGCTATGAAAAAAataTTTAATCATACTTTTAGAAATTGTAaTATTATTTTtAATAaTTTCGTCCCAATAAAGGAAAGtCATtATTTGAAAACAATATTTACAACAGAAATCATTTGTTTAAAAAATTTCATTTCTGACCAAATTTTTTtATAAACATAAAGATAATTGTTTTTAAAaTgATGGAGTATTATAAaCGAGAATTTTTTTtCTCAaGAAGAACATTTTTTACAACTTGACTATTTCCAATTtCGGAAAaTTAATTTCTCATTGCCTTGAATCATAAAAAAaTAaTTTTTtGCAAATACAAAaTT

>contig00031

CGCAAACGATGTCAAGACCACAACCAAGACAACTACAACTACAGCTGGTGGAAAAAACCTGTATCTCCAACTGGTGTAAAAAAACCTGTATCTCCAACTGGTGTAAAAAAACTACAACTGCAACTGGTGTAAAAGAACATACGACCATAGCTAAATCAGGCGACACCATTGCTGCAGCTAAGACAAAAGAATCTCTGACTGTAACCCAAAC

>contig00032

tAATGGGgAAGTCGACAACGTAAATTTCAAAATGAAATCAATAATTTTCATCTGCCTCATCTGCCTGATTATAGAAACATGTGTTGACGGCGCAAACGATGTCAAGACCACAACCAAGACAACTACAACTACAGCTGGTGGAAAAAAaCCTGTATCTCCAACTGGTGTAAAAAAACCTGTATCTCCAACTGGTGTAAAAAAACTACAACTGCAACTGGTGTAAAAGAACATACGA

>contig00033

CACCATTGCTGCAGCTAAGACAAAAGAATCTCTGACTGTAACCCAAACAGAAGAAGATGGAAAATGTGAAGCACCAGAAAATAATCCTTCTAATACTAAACCAGGGCCAAAGCCTGCAAAGGGTGGTGCACTTTCAGGCACCAGACCTAGTTCACCAGGAAaGAAaGATCCAAAATAACCTTAAaGAATATCTCAACGTCAACCTTATTGGATATCAGGATTtCGGCaTGGTTGGgATTCCTGTTTTATTTCCGGT

>contig00034

TtaTtGGGGAtATAAGTCGACAACGTAAATTTCAAAATGAAATCAATAATTTTCATCTGCCTCATCTGCCTGATTATAGAAACATGTGTTGACGGCGCAAACGATGTCAAGACCACAACCAAGACAACTA

>contig00035

AATTTTATTGTGAAGAAAGATTAATTAATTTTTACTACCTTTTGTATCAGGGTTAATTAAAAaTTTTtAATTAATATTAAATATTTCGAAATTAATTGAGTTAAAGTTTAATTTTAGTTAATGTATTATAATTATTAATAATTAAATTTTAGAGATTAAATGTCATTCGTAATTAATAATATCTAATTTTTTAAGAAATGAATTTAATTCATAAGGTTATTAATTTATAAAAATATTATTTTTTTtATAGATAGATAATTTTAAATATTTTATGGGATTAGCTTTAAAATAATATATTATAAATAAATTTAATAAGTTTTTTtAAATATAAATTAAAATTTTAATTTTTtGAAAGTTTATAATAATTTAAATTAATAAAATTTAATTTTAATTATTTTATTAATGATATTTTAATTATTTTATTAAAATTTAAATTCTAATAATATAAATTTAAAAATAATGATTAAATTAGTAAAGTATTTTATGTTAATTTATTAATAATAAATGAAATATTAAGTTAATGAATTAGGCAAAATTTTATATTTACCTGTTTATTAAAAACATGTCTTTAAGGTATAAATTTAAAGTCTAATCTGCTCAATGAAATAATTAAATAGCTGCAGTATCTTAA

>contig00036

TTATTAGAATAAGTTACCTTAGGGATAACAGCGTAATTATTTTAGAAAGATCTTATTGATAAAATAGATTGCGACCTCGATGTTGAATTAAGATAAATATTAAAAGTAGAAATTTATTAATTAAGTCTGTTCGACTTTTAAAATCTTACATGATTTG

>contig00037

TAATCATTTGTTTTTtAATTGAAAAATTGTATGAAAGATTGGATAAAATATATACTATATTAATTTAATTAAATTTtAAATTTATTTTAAGTAAAAATCTTAAATTTGTTTATTAGACGATAAGACCCTATAGAACTTTATATTTA

>contig00038

GTTAAAATCGGTGTGAGCCAGATTGGTTTTTATCTATAAAAATAATTTTAAA

>contig00039

TTTTAATTATATTCATTTATATATGTTAAAATTAATAAAAAATTTAAAAaTTTTTTtATACCCCCAATATAATATTATTATAAATTATATACTAATAAATTAATCTAAATAAATTAA

>contig00040

GGTAGCATAATCATTTGTTTTTtAATTGAAAATTGTATGAAAGATTGGATAAAATATATACTATATTAATTTAATTAAATTTTAAATTTTATTTTTAAGTAAAAATCTTAAATTTGTTTATTAGACGATAAGACCCTATAGAACTTTATATTTA

>contig00041

CGACAAAAGGAAAAAGATTTTGCACTGTAACTCCTGTTGCTCCATTTATTGTTTTTACTTCACGCCATTGTTTGAATATAGTTGATTATAACGGAGCTTCTTTAATAATCGAGGAAGAAAAAAaTGATGTAGTCGGTAAATTGGAATCTGACGTCGGTAATGCTATCGATTTAGCTGCCATAAAAGTAAGGGATAAAATCAATAACGTTAAGTTATCATTACCAAAGAACTTTCTAACCAACGAATATAATATTCATGTGGGTGATGAATTAACAATATTCAAATTATGTGGTTCTAACTATTGCACAGAGGGTGTTAAAATTACTGATCCCAACAAGACCGACATTACGAAAGCACTTTTAAATTTATACAGAATAGTAAGCAGCGATGGTAGTCCATTCGATTCGGAAAACTATGGAAGTCCTTTGGTAATAAATAATAGTAAAGTCGTGTTAGCATTTGCTATGCAAAGAAGGGATGATTATACTGGTGTCGGTAAACCTCCTACATCAAATTACTATATCACAAGAGTCGTTGATCACAAGGAATTTATTCATAAAGTATTTCAAACATTTGGAGGCAGTCGATAATAATAAAGCTTCTGTTTCTCGTCCATGATCTAAGAAGCGAAGGCAACAACTTAAACGTTCTTCTCAAAGGCTCTAAGTATATTGATACTTATATTGCACATTTTACCCAAAATAATAAATATTTTTtCTTTAAAATTTGGTGATGGTAGTAGTTTTTAACTTAATTTAAGTTTTTtCTAAGCTTATCGTTTTTGCAAAACGCTAGACGATCTGACGATAAGGTTGGTATATATATAAATGTACGAATTCTCTACACTTGTGTGAATTTAATGTAAAAaGAATTGTCCACAACAAAaCAATTtCTTGAATTAAAAaTAAaTCTtCTTTGC

>contig00042

TTcTTAGAGGGTAAATATTCACAAAATGAACAAACTTTTACAAAAGCTGAGAGTCCTTATGGTGAGCTGGGTTATTTAGTTT

>contig00043

CGACAGAAGGAAAAAGATTTTGCACTGTAACTCCTGTTGCTCCATTGATTGTTTTTACTGCACGCCGTTGTTTGAATGTAAAATTTTATAACGGAGCTTCTTTAATAATCCAGGAAGTAGAAAATGATGTAGACCGTGAATTGGAATCTGGCGTCGATGGCGTCGATAATGATTTCGATTTAGCTGCTATAAAaGTTTTGAAGAGGATCAAAAACGTTATGTTATCATTACCAATGACcTTTAAAACtCACGACTATAATATTCATGAGGGTGATGAATTAACAATATTCAAATTATGTGGTTCTAACTATTGCACAGAGCCTGTTAAAATTACTGAAGCCAAAAAGACCGGCATTACGGAAGGACCTTTAAGTGTATACAAAATAGAAAGCAGCGATGGTAGTCCATTCGATTCGGAAAATTATGGAAGTCCTTTGGTAATAAATAGTAATGTCGTAATGGCATTTGCTATGAATATAAGGGATGATTATTTTGACGTCGGTAAACGTCCTACATCAAATTATTATATCACAAGAGTCGTTGATCACCACGAATTTATTTACCAAGTATTTCAAACATTTGGAGGTTCTCCAGTCTATGATAATACATTTCCTTTTCCTCGTCCAGAATTTAAGAAGCCAAGGCGACAACGTAAACGTTCTTCTCAATGGCTCTAAGTATATTGATACTTATATTGCACATTTTACCCGAAAGAATGAGTATCTTTTCTTTCAAATTTGGTGATGGTAGTAGTTGTTAAGTTAATCTAAGTTTTTTTAAGCTTATCGTTTTTGCAGAACGCTAGACGATAAGGTTGATATATATATGCACGGATTCTCTACACCTGTGTGAATCTAAT

>contig00044

GACTTTGTTCGGAAATCTTTTAAACATTTAAAATGTTTTGCATTTTCGTGCCTTTCTTGTTTTTCTGGATCGCTTCAGCAGATCCAGGGAAAGAAC

>contig00045

TTTTtAATTCAAGAAaTTGTTTTGTCTTGGACAAATTTGTTtACATTACATTCACACAGGTGTAGAGAATCCGTACATATATATAGCAACCTTATCGTCAGATCGTCTAGCGTTCTGCAAAAACGATAAGCTTAAAAAAAaGTTAGATTAACTTAACCACTACTACCATCACCAAATTTGAAAGAAAAAATACTCATTATTTCGGGTAAAATGTGCAATATAAGTATCAATATACTTAAAACCTTCGAGAATAACGTTTACGTTGTCGTCCTGGACGAGAAACAGGGGCTTTATTATTATTAACTGGAGAATCTTCAAATTCTGTAATTACTTTGTGAATAAATTCGTGGTGATCAACGACTCTTGTGATATAATACTTTGATGTAGGTCGTTTATGGCCAGTAGTATAAGTATTTCTTATATTCATAGCAAATGCTATTACGACATTACTATTTGTTACCAAAGGACTTCCATAATTTTCCAAATTGAATGGACTACCATCGCTGCTTACTATTCCATATACACTTAAAGGTCCTTTCGTAACGTCGGTCTTTTTGGAATCAGTAATTTTAACACCCTCT

>contig00046

TTTTtCtGGATCGCTTCAGCAGATCCAGGGAAAGAACTTAATTCGGCATTACTTTT

>contig00047

GAGGGTAAATATTCACAAAATGAACAAACTTTTACAAAAGCTGAGAGTCCTTATGGTG

>contig00048

GTGCAATAGTTAGAACCACATAATTTGAATATTGTTAATTCATCACCCTCATGAATATTATAGTCGTGGGTTTTAAAGTTCATTGGTAATGATAACATAACGTTTTTGATTTCATCCGTTACTTTTATAGCAACTAAATCGAAATCATTATCGACTTCATCGACGCCAGATTCCAATTTACGGTCTACATAATTTTTtACTTCCTTGATTATTAAAGAAGCTTCGTAATAATCAACTATATTCAAACAATGGCGTGAAGTAAAAACAATAAATGGAGCAACAGGAGTTACAGTGCAAAATCTTTCTCCTTTTTTCGAAACTAAATAACCCAGCT

>contig00049

GTGAATCCCGTTGTGCTTGGGCGGCTTCGTGTGCTGCGAGAAAGACTTCATCTTGCGCTCCTGGTCCACCTCCAACACCACCTACGACTCCTCCGGCGTTACTtCTGTCACCCGTTACGTGCCACGCTGCTAAGCCATGAGGTtGGgTaGGATCGTGGGT

>contig00050

ACTTCTTTTGCTCAGGTAGTAATTTCTTTCGACTATTCCAAAGTCTGTGCTCGATGAATCCTTTTCCTGATGATATATCGTAGAAGTGCTCACATCCAGCAGCAGTTTTGTCAtCTCTTAAGCATGGAAACTCACGAACAATTGCTTTAGCCAAAGCCCACTTTACTTCTTTCTTTGGATAATGATTGTAGTTTTGAGCTTTTATCAAGTCATCAACGaGTATCCGGTTGAAGCATTTCCTGTCATCTTCCCCAAAaTGTCCACTTTCGAGAGCCTTtAAGAGAGCTTTtCCTTGTAAATGGTTTTGTAAAGTTTCTGTTAGACTTGCATGAGTTGAGTATGCTTCATTGTTCATAGAATTTGAATCGTAATCGTAAAAAAAaGGTCCAAATCCAGCAGTCTGCATCATCAAGTTTTGTTGACTTAATGCTGTAGTTCGTTGAaGAATATCTTGAAGTCGGTGCATTTCTCCCTTATTCAAATCTAGGAAGTAGTCCGAATCCTGTTTATCTTCTTGTCCTGTTCGTCTCCTGTTGTCTT

>contig00051

AACTTTTAATTGTCTGTGTTGTTGAATGTGCGATTTGCGCTTGAAACTTTTCTGACATAAACTACAGACAAATTTCGGAGTTTGTCCACATTCATATTGAAGATGTCGaCGCAAAGTGTAAGACTTTCCATAAGACCTTCCGCATTTGTGGCAATGGAATCGTTTCTCAAAAGCTTCTATTCCTGAGACGAAAGAATTTCCAtAGAATTCTT

>contig00052

ATtGGGgATGTCAGTGAAGAACGAAAACTTCGAAATGAGACAGTCAAATATTTTAATCATCTGCTTTATCGCCGCAGCTCTCCAATTAGAAGCAGTTACAGGTG

>contig00053

GCTTATTCGATTTTTtGAATCGGAAAAaGGAAAAAAaTCTGATAATGAAAAAAAGTTGACACCGAAGGTGAAATCAAAAGTGAAGATGGTATAAAGGGAAATGTTCCGCCCGAAACAATCGACC

>contig00054

AAAGTGACACAGAAACGGATAATTATGGGGGAGGgAGACGTTTCAACTCGGACCTTGATGAATATTTtCTACATCGTATGAAAATTATATTTTATTGCCGATCCATTAACTAACTACAAATCATTTAAATCCGTTTTCAACCGAGCCGATtATGCTTtCAATTATTGAAATATATTCTGTTTTCATATATCGAaTGAAT

>contig00055

ACTGAAGGAACAACCGTGTCATATATGATAATATCTCGCATACAGTAGTGCTTAGGCTTATATTCTCTTGTTATTTGTATACATGTTTTGGAATGAGTTATATCCTTATTTACTTTTATAGGATCCGAAAGACAAGTATGGTCGTGAATCGAAAAATCCCAATCTACTGTTAAATTGCTCTTCCCATTGTCGCAAAACTTGTTATGTTTGAAAGCTTTTGCTCCCAATACTATCATATTAATGATAAAAATGACTTTAATCATTTGATTCGACATTTTGTTCAATGAATTAAA

>contig00056

TGTTTTATAATCCAGTTCCCTTAAGGGAGTTATTATATGGCGTCTGAGGCAATTTCTGACTAAATCTTTCAATTTCTTCACTTCTACGGGATCTGCACAAGGGTGATAAAGCGCAACGATGGCCCCTTTCTGAAGAGCACGAACCCATCTCTGTTTCGGACAATATGTATATTCACCATATTCAGGCCCGATTAGTCTACTTCCTCCT

>contig00057

CTATCATTAATATCAGAAACTTCCTTTGCGGGAACTAAAAGTCTTTCATTAAATGCGCCATTAGCTGGGTCATTTTCCACTCCCTGAAGAGCGTTGAAGGCGATGAACTCTTGAACCCTGATGTCTTTGACAAATGACATCGTAAATATACACTTCCAACTCACTAATACCATAGG

>contig00058

ACttATGGGGATAGTTCTTTCTGCTGCTTGTCTACAAGTTTAGTGCCGTGAAACGTCAAAGTTGCAAATCTAAAAAAA

>contig00059

TTTAATTCATTGAACAAAATGTCGAATCAAATGATTAAAGTCATTTTTATCATTAATATGATAGTATTGGGAGCAAAAGCTTTCAAACATAACAAGTTTTGCGACAATGGGAAGAGCAATTTAACAGTAGATTGGGATTTTTCGATTCACGACCATACTTGTCTTTCGGATCCTATAAAAGTAAATAAGGATATAACTCATTCCAAAACATGTATACAAATAACAAGAGAATATAAGCCTAAGCACTACTGTATGCGAGATATTATCATATATGACACGGTTGTTCCTTCAGTAGGAGGAAGTAGACTAATCGGGCCTGAATATGGTGAATATACATATTGTCCGAAACAGAGATGGGTTCGTGCTCTTCAGAAAGGGGCCATCGtt

>contig00060

ATGGACATTTTTTtATtCATTCATTTGACACAACAAACGTAAAATATATAAATAAAATCTAGGATTACTGAACAAAACTTAAAaTTTACAAAAAGTATCCGCTTATGTTATTTTATAAATAACATTAACATATTtATTGCTTCAGGAAGATGCAACTTTACACGCTTTTTCCGAAATCAAACATTTTGAGTGTATATGAAATTATTAAGTGAATCTAAAGAATACTTAAAGTTATTAAATATGATCGATATTAATCACACGTTATTTAATTGGAAATAAAAACATAAAATCAAATATTGTCAAGTACGTTAAAAGTTACTAAATGCTGGGCACACTG

>contig00061

ATGGGgAATTCTGGGTTTACTGACGCATCGAAGTTTTTCGAGAAGTTATCCAGGCTTTATCTTCGAGTTTCCAAAAAGTTTACAAGATCTTGTAAAATGGCTGACAAAGTCGAATCTGCTGCTCCACCTGTGGTTGCAACACCTAAAAAAGCTGCTGCTGCTGCTAAAGTAAAGAAGCCGAAGGCAAAATCGTCTCATCCACCAACAGCATCAATGGTGAATGCTGCTATCAAGGAATTGAAAGAAAGGAATGGCTCATCTCTTCAAGCTATCAAGAAGTATGTTGCTGGAACATACAAACTTGATGCAGAAAAATTAGCACCTTTTATTAAGAAGTATCTGAAGAGTGCAGTTGCCAATGGTACT

>contig00062

CCcTAAAAAGGGCCGTTTACTATTTGATGACCTCAAAAATGTTGTTACATTTTTTACTCCTTCCAATTTACTTTTTGGCGACTTTCTTAGCTGCTGGCTtCTTTGCCTTTGGAGCCGAAACTTtCTTTGATGGTGATGCAGCTTTAGCGATCTTCTTTGGCGCAGCCTTGGCCGCTTTAGGCTTTGAGCTTGCAGTCTTTGCTTTTGGTGGTTTAGCGGCAACTTTGGTAGCTTTCTTCTTTTCAGTGGCGACTTtCTTtACCTTGGGAGCAGCAGGCTTCTTTGATGAAGCTGGCTTCTTAGTCACCTTAGATGCAGCAGGTTTTGCGACCTTAGCCTTCTTGTCCTTCACTGCTAGCTTGAAAGATCCAGTTGCACCCTTACCTTTGGGTTGAACCAA

>contig00063

CTCTAAAATAACATAAATTAACTATATATGTGATTAAATTATATACGAATAAACTCTACACAAATCAATCATACACAACAAAATCTACACTTAATAATACATTCGCTTTATTGTAAGTAGAATTTTCTTAAAAATAATATAGCCATGCAGAACTTATTAATTTTTATCGAAGAAAAATAATAAAATTGACACGTTTTTAGTTTCATAGTACATAATAAACTGTGTAGAGTTTATTTAAACTATTTTCGAAAAaTAAGGGAATTGATTTAAAAATCTCAATAATCGTGAAACTGTTTGTCCTGAAAAATTTGTCTAATTTGTATTCCACATTTGTAAAAATAAACTAACATAGTACAATTCCtAATTTGTGTATATTTGCTCTGGTATAAACTAATGAGTAATAACCTTTTAATATCTTGAATAATAAGTTTCAATACCAAAaTAGCTATTGTATCTTAAAACATGTATTACGAGTCAATATtAATTTTTtCTGTGGTATTTGTGGGTGTAAAATAaTtGCTTATTGGTATAAGTTGTAAAAAAaTTCCTGGCGCAAAAGGATGTGCCTAGAATAGGCTGATAGGTTTAGCACTTAATCAGAaTGCCATTGTTTAaTAaTAATGATTTAGCGATAAACgAaGAaTtCATGGTGAGTTTATGACTCATCGATGATTCTTCGCTCTTGCATATACCTAAATAGTACACCAGTGATATATCTAGGATTAAATTTATAAAA

>contig00064

AcatGGgATTCAGAAGAAAAGTTCTgTACAGTTAACAAGTCAACCACAAATTCAACAATTCCAAGTCGTCATGTCTTTGAGACACGTCACCCTCACTTTCTTCTTCATTTTGGTCGCTGCAGGCTTATTTATGGGCTGTTTAGGTGATGAAAGCGAAGTAGAATCAGCTTCAGCGAGTGGATCTGCTGCGAGAGCATTTATACAAAATAATCCAATACAGAATATGGATATAAGTCAAATTTCTTCAATGATGGTCGGATTAACCCAAATGGCAGACGAAATGATGAAAAAAaTATCTGGAATCCGCAATATGTTAGGAGTTGAACGTAACGCAGAACAAGAGTAGAATGAATTATAAAAATATGTCCTACGTA

>contig00065

ACCAGCAACGAACAATCCAGTGCAACCCAATCACAACAGAATAATCGACTTGTCACCGGCACAGCGTAAAAGTCGACTTCTCGGGAATAAACGCGATACTATCAAATAAGTATTCTCTAGCCAGTAAGATTTTTACAATATGCGCTCTGTCGAGAACTGATGATGGTATCGAAATTGGCTGTCGACGGCAAATGTAGTATAaTGGAATAATCTTGATTATTGTATTAATtGAGCGCGCTCGCTATACTGTTAGATTATTtCAAatGGCGCGATTGAATcGTGCATGGACTCTACTGGCGCACAGGACTAAAAATAGTGCGCTGAGCGCACTTAAGGCGCGATCATTGACCTCGCATAAAGAAAGCTTTTACTGAATCATTATACGAATTtCAGTCAACGATCCTTTTtGCGAAGAGTTATtAATCTTCACTAAATAATCTGTGATTCTTTATGCCAAGTTTATTATGGGT

>contig00066

AGTTGTCCGATGAGCACGTAAAGCCTGGACTTAATACTGGTCATGGCCCGGAAACGGATGACACTCACCATCATTTCCCCTAAATACGGTTTGTTGGTGCTAGTCTTATTCAAACTAGAATATGGACTAATGAAGGATGACCAATGTAATGTTGGAAGTTTAAATAAAaGAAAATTAAAAaTATTAATGTTGTAATTTTCCAAATTATTTTAGTCGATTATGTCATTACTTtATCAAATTATTAAAGCATGTTGGATTAAaCT

>contig00067

CCACAGGTTGACCAGGTTTGTTGTCTCTAGCGTCGATCACA

>contig00068

ACAGTCGAGTGAAATATCGCAAGCGCTGCATATCGGTCTGGAAATTCAATCGAATAAAAaCATa

>contig00069

TGTGATCGACGCTAGAAAAACGAACCTGGTGAACCTGTAAAGAAGTTACCCGATGGGCACGTAATATCTGGACTTGGTACTGGTCATGGGCCGGATGATGGATCACATCCATAATTTCTTCCAAATATGGTTCGTTGGTACTGGTCTTATGCAAACTAGAATATGGACTAATGAAGGATGACGATTGTAATGTTGGAAGTTTAAATAAGGGAAAATTAAAAaTATTAAT

>contig00070

TGTGATCAACGCTGGAGGCGACAAACCTGGTGAACCTGTAAAGTTGCCCGGTGGGCCCGTAAGGCCTGGACTTAATACTGGTCATGGGCCGGATGACGGATCACATCCATAATTTCTTCTAAATACGCTTCGTTGGTGCTGGCCTTaTGCAAACTAGAATATGGACTAATGAAGGATGACcAATGTAaTGTTGGAAGTTTAAATAAGAGAAAAaTTAAAATaTTAATGTTGTAATTTTCCAAATTATTTTAGTCGATTATGGTATTGCTTTATTAAATTATTAAAGTATGTTGC

>contig00071

TAAGTCAGAAGATGGTAAAATCAAGAGTGGATTAAAGAGTGAAGATGGTAAAATCAAGCGTGGATTAAAGAGTGGAAATGATAACAAAAAaGAGTTTCCGACCCAGCCAGACTATG

>contig00072

CAGGTGGCCTATTCGATTTTTTGTGTCGGAAAAAGGAAAAAACC

>contig00073

ACttttAtGGGgACGTCAGTCAAGAaCGAAAACTTCAAAatGAGACtGtCAAATATTTTAATcaTCTGCTTTATCGCCGCTGCACTCCAaTTAGAACCAGTT

>contig00074

ACAGGTGGCTTCTGGAGTTCACTTAGTACTATCAATAAAAAAATCTTATGATGCGAAAAATGGTGACATCGAGAGTGGATTCAAGAGGGGAGGTAGTAAAAAAAGATGTTCCGTCCTATCCAAACTATCAAAGTGGCCCAGGAACATATAATTATGGCGGATTGGGGCATCACGTAACTAAAGAAAGTCATGAGAAACTAAGGGGTGATAACAGATTTTCCGACGCGTACCTGAGTATTTTCCATCCTCCGCAATTTCAAAGTGCATATGGAAACAATACAAAATAaGATGGAGTATAATCAGACTTCAACTCGGACCTTGATGAATATGT

>contig00075

GGGAACCGCATATTAATGACGGATCGAGTCATATGTCTAGAAAAAGTGCAGAACAAATGGGGGgTAGTGATATGTTGAACGATTTGTACAGGAGTACGTTCCATCCTCCGCAATTTCAGTATCAACAACAATACCCTTATCAAGAACCATAATCAGACTTCAGCTCGGACTCTGATGAATATTTTCTACATCGTATGAAAATTCTATCTTATTGCCGGTTCATCAACTAATTATAAAATATTTAAATCTGTTTTCAACCGAGCCGATTATACTTGCAATTTTTGCAaTaTATTCTGTCCTAATATACCcAATCAATAAAGTGTAATACTGATATTT

>contig00076

aCCACGACtACAAAACTATGAGATTtATGCCTCTTCAGGTTTTGCCTACACAGCTGATAAAGCCGGGCTGCTAAATCCAAACGGCGAAGGAAAAGTAACGGTGAAATCATATTATGAAGCTGGAAAaCATATGTGTCAGGATGAAAAAGTAGACCAAAAGAATCCATTTAATTgCTTGGAATTAACATATGTTTATGTCTTACTAAGCAAAAGTTTTAATTTGCACCAGGACACAACAATAAATATCATTAATAATATTGACTGCCACGAAGTAAATTGGTCTCTCGGATTTGCATTTTCTAAAATAACAAACTTGGATATTAAATAAAAaCAAATTTCTTAGTTGGCATTTTCGCAA

>contig00077

TTCCTTtATTCAGaGAACATCTAaTTTTAtACAGAGTTTTtGAAaTAAATACATTTTTAAaTGCGAATTAAAaTTTAATTTAAATACATATTCCTtAAAATTtAGAAaTCAAGTGCATGCCTTATTGAAAAAATCCTCGAGGAAATCCTTATCCGAAAATAACACGAGGATATCCAAGAGGGAATGTGCGAGGATTGTCAAATTCCACGAGGAAATTTGCGAGAATTTTTGAATTCCTCGTGGATTtCcTCGTGGAAACCC

>contig00078

CGTGCCAcTTtCGGATAGGGATTCTTTCAATAAGGCGCACACTATTGCGAAAATGCCAACTAAGAAATTTGTTTTATTTAATATCCAAGTTTGTTATTTTAGAAAATGCAAATCCGAGAGACCAATTTACTTCGTGGCAGTCAATATTATTA

>contig00079

GGATGATTTTTACACCcAGCAAAaTGTCTCCTATCTTGATTCTTTTAATCCTTATTGCAACCGCAACTGCTCCAAAGAATACACGGGACGACACTAATCCACCAAAACAAAATGGG

>contig00080

CAAAATTGTTACTCGATTTGTCATGACTTGTGCCAAACATGTAAGGACGTACAATACTGTGAAAAAAaTGGAACACCCACGGATATATGCCTTAACGATTGTGGGGCGAAAGCAAAGGTAGATTGTAAAGAATACTAAGAGAATTGGACACTTCAACTTATTTCACATTACTTTAATCTCAAAATTAGGTACAACTGAATTGGATAAATGTAATGTAAATTATCTATTATACCCGTCAAAGCCTGAGATGATCATACCACTTTCCAACAAaTTAAAATATGGTGCAAATGTGCTTATAATTTTGTTAAAA

>contig00081

CAAAATTGTAACTCGTTTTGTCAAGAAAAGTGCAAAACATGTAAGAACGTACAATACTGTCAATATGGAATACCCAAGGATATGTGCTTAGTCGATTGTCAGGCAGGGAAAACGATAAATTGTTAAAAATATTATGAGACTTGGACACTTCAACTTATTTCACATCAGTTTAATCTCAAAATTAGGTACAACTGAATTCGATAAATGTAATGTAAATTATCTATTATACCCGTCAAAGCCTGAGATGATTACACTACATTAAAAAAATCAAAGATATGGT

>contig00082

AGATTGTTACCAGGTTTGTACTGGAATTTGCGACTCATGTAAGGACTTAAAATACTGTCAAAAAGATGCAACACCCACGGATATATGCTTAAGCGCTTGTGGAGCAGGAAAACATATAGATTGTTAAAAATACTATGAGAATTGGACACTTCAAGTTATTTCAAATCACTTTAATCTCAAAATTAAGTACAACTAAATTAGATAAATGTAATGTAAGTTCTCTATTAAACTCGTCAAAGCTTGAGATGATTACACCAGTTTCAAACGAaTTAAAAaT

>contig00083

TACGGTGGTGGCGGAGGAAACTTATAGAGGCTAAAAaTTCGCCGAAGTTAAACATGTTGAACATAATATCAATACATTTAAATGTGAATTACCCCTTATAAGTATAAAATATAATTCACGCGTTTTCTGTCTTATGATCGGTATATAAACCACTTTAAAAaCTTATAAATTTAATTTtATCAAACATCTTATATGTTCTAAATTGTTACCTTACATTCTATTATAATTTACGGCCTTATTTCTGTGCAATTGATTGAATAAAATGATATACATGATCCTGA

>contig00084

GGTGGATCTGGTGGAGGTGGTGGTCAtggTGAaGGCGGTGGA

>contig00085

AGTCAAAAAAGAAGCTTTGTCATGAAACTGCTATATATTTTCATCTTCTGCCTAAGTGGCACAACTTTCCTAACCGAAACAGCTGCCCATATTGGTGGACTTTCAAAAAATATTCACGCAGATCTTCAGGTTCATAAAGATCTAAGTCTTGGTAaTGGTGGagtcAgTGGAGGTc

>contig00086

aTtGGGgATGAGTCGACAAAGAAGTTTCGTCATGAAATCACTAAATATTTTGATCTGCTTCATCGGCATAACTTTCTTTACCGGAACAGCTGTCAGCGAGCTAAATGGTAGTGGTGGACTTTACGTTGATAAAAAaGCGGGAGTCGAAGTTGGTGCATTCAGTGGAACTGTTGGAACCAGTAAAGGCATTGGATATGGAGGAGGCACTGGATTAGCTGAAAGTAGGGGATACGTTCAAAGCAGTAAAACCAGTGCATTAACTGTCAGCGGCAGCGGAGGCAAACGTGGCGGTAAAGGCATAGGAGGCGGT

>contig00087

GGATTCAGTGGATTCGGAGAAAGCAGTGAAAGCAGTGAAAGcAGTGAAAGCAGTGACAGCAATGATGACAGCAGTAACAGcaGTGAGAGCAGTAACAGCAGTAACAGCAGTAGCAGCAGTAACAGCAGTAGCAGCAGTAACaGCAGTGaTGACAGCAGTGATGACAGCAGTGATGACAGCAGTGATAGCAGTGAAGTCACTTGGT

>contig00088

CTGTCTGCTCTGCTCGGACAaGCTTCTCGAAATACGTGTTCAATAAATGGTTGCTTTGCAGCGAGTCCTCCTCCAGGTGGTAGATTGGGCAGCTCTTCGTCCATAAGAAACTGAAGTAGCTGAACAAAGTCAAACCCTTTTTtGCCTTCCTGCTGCGTAACAATTTCTTTGATGCTTCTTTCGATGACCATTTCAAGTTCAGCCTCTCTTTTCTGTGCCTCAGGACTTGGTTTtGGTGCTCCAGAGAAAGTTACCAATACTATGCTCATATTGTCTCGGCTGCCC

>contig00089

CATCGTCACTTCTTTTGACTAGGTATTGTTTTTCGTGTTCGATTAGTTTTGGCTGCTCCTGCCACTAATTAACTTGTTTCATGTTTTTCGTCAATGCAAGAATATATTTCCGTCAGCATGCCTCTCAATTCAACACACAC

>contig00090

ACGCAGGTGCTCTTGTATCTGCGCATAGTTCAGAACATTTACTAGAATGTATAATGCAAACAGAAGAGTTCAAAGCTGAAGATGTAATAAAaGGGATTCACTCCGGTTTCCTTCAACTCgATGATGAAATGCGGCAgCTCCCACAGATGAGTTCCGGTTCTGACAAATCGGGCTCTACCGCAGTCTGCGCTTTCATTTCGCCCCGAAATATCTACGTAGCAAATTGTGGCGATTCCAGAGCTGTTCTGTGTAGAtCCGGAAACCCAGTGTTCTCCACGCGTGATCATAAACCGGGTTTGCCAGTTGAAAAAGAGAGAATTCAGAAGGCTGGAGGAAGTGTTATGATTCAGAGAGTTAATGGATCCTTGGCTGTGTCGCGAGCTCTGGGTGaTTACGAGT

>contig00091

AATACGACGTGACTCCAGCTCTTAAGTAGCACGCGCCTTTTTAAACAAATTCGCTGAAAAAAA

>contig00092

TTCTTCTGTTTCTGCTCGAAGGAACTCCCTCATCAGCAAAAGATCCTGCAACCCCCAGAAGAACGAAGAGGGCACACGCCAAGGGCACATTCATTTTCAGCATCCT

>contig00093

GAAGAAACGATTAAACAAACAAAAATATATTTTTTTtACAGTTACCACTTTTTTtCAAAGAGAATATAATGCGATCACGATGGTAAGACAGTTCGTAGCTGAATGACT

>contig00094

GTAATATGTATTCTGCGTCATTACTACCGAACGAAAACTCCGTGGAGAGCGATTTCTCCTCAAGAATATTGAAGCGAGATGTAACAGAAGCAAGCCAAGCAAACGAAGTCGAGTCTACGGTTGCTCCCTTAAAAAGTTCGGATTCAGACATCAAGACTACGGAGGAATTTGGAACTACTCCAGCTTTTATTCCTAATACTAAGCAACCCTCCAGCTCCACTGAATCAAATCCCACAGACTCTCCTACAACAAAACTTCCAGGTGATTCCAATAATATTCCTTCTCTAGG

>contig00095

GTCCAAATCCATTCCCTCAGGGACCAAGTGGCTCTTTTGGACCAGCGTTCGATTTCCAACAAGTATTTGATCAGTATTTCAGAAACATACAGGCTCAAAATGAGGCCCTGCAACATCAAATCCAACAACAACAACAAaCTATTTTTGAATTGAATAACCGTCTTGGAAGCGGATCATCATACGGTGGATCACCATACGGAGGATCATCTTTTGGAGGATCACCTTtCGGAGGATCCTTTGATGGAAGTTCA

>contig00096

ATACGCAAGGCACTCGAAAACCgAaCCAACATGGAGGATGACCGGGtCTCCTTGTTGGAACAGCAACTTGCACAAGCTAAACTCATTGCCGAGGAGGCGGATAAGAAaTACGAGGAGGTTGCCCGGAAGCTAGTCATGATGGAGCAGGATCTCGAGCGCGCTGAAGAGAAGTCCGAAATTTCTG

>contig00097

CGTTCACTCTCATCTGCAGCCTGAGATGCCTCTGCCAATTTGGCAGTGGCGGTGGCAAGACGTTCCTCTGATCTTTCGAGGTCTTCCTCTAAAAGCTGGATACGACGGTTAAGGGCAGCAACCTCGGATTCAGCTTCtGAaGAGCTTTGTCT

>contig00098

CTTCAAGTTTTGCATTGACTTGCATCAGAGCTTCTTGAGTCTGGTCAAGCTCGTTTTCAATGGTCTGGATCTTCTTCTGGAGAGCCCGAGCTTCTTCCTCTGCCTTTTCAGCACGCGCGTTGGCATCGCGAGCTTGCTGTTCACAGAGCAACGCGCGGTCCATCGCGTTGTCCTTCTCCAGCTTCATCCCTTGCATCTTCTTCTTGATTGCGTCCATGGTGGCTATTTAGCTTTGTTCTTTCTGtAAAGAAaTGTAAaGT

>contig00099

GACTCTCCAGCCTCCGCACGTTCTTCGGCGCGCTCTAGATCGGCCTCGACCATGGCCAaTTTA

>contig00100

CGGGCAACCTCATCGTATTTCTTGTCTGCTTCCTCCGCGAGGAACCTGgCTTCTTtCAGCTGATTTTCCAGTGCGTCCATACGCtCTTCATCTGCCAAGCTGCGATTCTCGAGGATCTTGCGGGCG

>contig00101

AAGCAATCAGTGGAATTTGACTGATTTACAGTAACATATTTTTAGCTACTTGAATCAGTCAAATTTCACTGATAATCAGTGgATTTTGACTCATTCATATTTAGAGAGTAGGAAACCATGTGGGAAaCCGTATCATAATTTGAACTGGGGTCTTATATACTTTCCAATACAGATTCCTATGGCCAATTTTGTTTATTTCGTGATTGTTTTAAGACGATAAAATATTGAAATTTTGAATGTTAAATATCATTTTTGAGTGATGGAGAAGACCTTATTACAAATTATCCATAATTTAAGATGCAGTTTTAaCATTCAAAaTTTTGATATTTTAAAaTTAtAAAaTAATTAATTAATTAATAAAAAaTTGGGTATATGAAACTGTATAGGAAACTATATGGGAAACTATATCATAATTTGAACTCGGGATATAAAATATTGAAATTTCGAATGTTAAAGATGATTTTtGACTCATGGAAAaGATCATATTATAAaTTATCCGTAATTTAAGATTCAGTTTACATTCACAATTTTGATATTTTAAAATTATAAAACAaTTAATCAaTTAaTAAAAAaTTGGGT

>contig00102

TGCAGGAAATTACAAAAAAACTAGATACCCTCTAAATCTGAATCAGTCAAATTTTTgCTGATTGAGTCAATGATTTACCTTTTCACTGATTCACCAGTTAAAAAATATATTTCACTATTAAATCAGTTGATTATCACTAATTGAATCAGTGGAAAAAATATTTTCACTGTTGAATCAGTTAATTTTCACTG

>contig00103

ACCTGCCTATTTCAACGACTCGCAGCGTCAGgCAACCAAGGATGCTGGAACCATCTCCGCCCTGAACGTTCTGCGTATTATCAACGAACCcACCGCCGCTGCCATTGCCTACGGTCTTGaCAAAAaGgCAGTTGGTGAACGCAACGTtCTCATTTTCGATCTTGgCGGCGGcACCTTCGACGTGTCCATCCTGACCATCGAGGATGGTATCTTTGAAGTCAAGTCTACAGCTGGAGATACTCATCtCGGAGGAGAGGACTTCGACAACCGCATGGTGAACCACTTTGTCCAGGAGTTCAAGCGCAAGt

>contig00104

ACtAAAAAaGtAACTCACCAGCAACAAACGAGCCCTCCgTAGATTAAGAACTGCTTGCGAACGTGCGAAGAGAACTCTCTCTTCCTCGACCCAGGCTAGCATTGAGATCGACTCTCTCTATGAAGGAATTGACTTCTACACGTCCATCACCAGGGCTCGTTTTGAAGAGCTTTGCGCTGACCTCTTCAGAGGCACCCTCGAACCCGTCGAAAAATCTTTGCGGGATGCCAaGATGGACAAGTCTCAGATCCATGATATCGTCCTCGTCGGTGGTTCTACCCGTATCCCAAAAATTCAAAAGCTACTCCAGGACTTCTTCAATGGAAaGAaCTCAACAAGTCCATCAACCCCGACGAGGCTGTGGCTTACGGAGCTGCTGTTCAGGCAGCCa

>contig00105

ACAGtGATCACGGCATTAGTGACGGTCTTGCCGAGGTAAGCCTCAGCAGTTTCCTTCATCTTTACCAAGACCATCGAACTCACTTCTTCTGGGAAGAATGTCTTCGTCTCTCCTTTGTATTGAACCTGAATCTTTGGTTTGCCACCGTCACTTATTACAGTGAAAGGCCAGTGTTTCATGTCAGCCTGGACAGTGGCATCTTCGAAACGGCGACCGATTAGTCGCTTGGCATCGAAGATGGTGTTATTGGGGTTCATCGCGACCTGGTTTTTAGCAGCATCCCCTATAAGACGCTCTGTGTCCGTAAATGCAACATAACTGGGTGTGGTGCGGTTTCCTTGGTCGTTTGCAATGATTTCGACTTTCCCATGCTGGAAAACGCCAACGCATGAGTAGGTTGTTCCAAGATCGATACCGACTGCGGGAGCTTTGGCCATTTTTtCGACCTTTTCTGTTTTCTAAGAGGTAATGAGTAGATACAAAGAAGTTAACCCTACAACGATTCGCAACGTGAGTCCCcAT

>contig00106

ACGTGTTTGCCAGCTCCAGTTTCGCTGAAAAAGGTATTAAAACTGTCATCTCCTCCGCCCAAAATTTTGTCGGACGGCATTTGACCGTCAGGTTGGATTCCATGTTCCAAACAGTAGAGTTCCCAGCAGGCATTTCCAATTTGAACTCCCGCTTGACC

>contig00107

TACTTtCTGGTAAATTGTCGTGAGATCCATCCAtGTTCATCTTGCTTTTCCTCATGTTtCCCcTCGACCACAACACATTTGTCCACAACCTTCACACTAATTTCTTCTGGTTTGAATTGCTGAACATCTAGT

>contig00108

ACTGGTCTTCAAGGTTTCCTCATTTTCCACTCCTTTGGCGGCGGCACTGGCTCTGGTTTCACGtCCCTTTTAATGGAACGCCTCTCAGTTGACTACGGAAAGAAATCGAAATTGGAATTCGCAATCTACCCAGCTCCTCAGGTATCGACAGCTGTTGTGGAGCCCTACAACTCCATCCTGACCACACACACGACccTGGAACATTCTGACTGTGCcTTCATGGTAGACAATGAAGCCATTTACGACATTTGCAGGCGAAACTTGGACATTGAGCGACCCACCTACACAAATCTTAACAGGCTGATTGGACAAATTGTTTCCTCTATCACAGCTTCCCTTCGTTTCGACGGTGCCCTTAATGTAGACCTTACCGAGTTCCAGACTAACTTGG

>contig00109

CCTTATCCCAGAACAATGCGACGTCGAACAGGTCAACTCCAGTCTCTCATCAGACGGAGTGCTGTGCATTACCGCGCCCAGGAAGGATCAACCAAAGGATAAGAACGAGAGATCTATCAAGATCGAGCAGACTGGAAAACCTGC

>contig00110

AGAAAGTGAAGTTTCCGGAGTCCCTCCACTTCATGCTGATGGTCCAGGAGCAGAAGGACCTCACACTCACATCCCTGGAGTTCTCTCTTATGCTGAAATTTGCTCGAGAATAATTGAAAGTGCTGTAGGCCGACTAAGACGAGTTGGAGATCCCTCAAAGAAATATGGAAGTTACGCTTATCAACCTTACAATGATCAAACTGGAGCAGAAGGAATTTGGGTTGGTTATGAAGATCCCGATACTGCAGGAAACAAAGCTCTCTATGCCAAaGCCAAGGGACTTGGTGGAGTTGCCATCTACGAGCTTTCTTTGGACGATTTCCGAGGCATTTGCAATGGTGACAAGTTCCCGATTCTTAGGGCTGCCAaGTTtAaGTtGTAAaTTTtGTTTGTtAAaCcTCaTCGATCATTCACATTTATCAGATTCAaTTtATAAAAaCT

>contig00111

CGGTTCGTTCAGCTTAACTATA

>contig00112

ATACTTTGATAGTCATAAAaCGGAATTACGTGAAATTTGGTCTTTtCTCCATCCAAATAaGAAAGTCCTGTAATTTTATTCTTCCTCCATACTATAAGACTGCCCGGGGgAAAaGTCAATTTTTtGTtGTTTTGGGATTTTCTCATACTCATTGAATCACGTTTTTTTtgAAATAGGCTATGTTCTCTGTCATCAAACCTTTCGTATTCCATCCAAACAGTTCTTCATCAACTATTTCTCTtaCAaCaGGTTCTTGGTAAAaCAaTTTTaCTAAA

>contig00113

TTCGTTAAAaTAGACAAAAAaTGTGCTGTTGTAGCTAAGACAGCTGACTTAGTTTAATTTTTACAGCAGCTAATTCTTCTTTCTCCTAGTCTTCAAGGCGGCGTTTTTTAAGGTTTATTTCGCAAGCTTTAGGGATCGGTTCGTTCAGCTTAACTATATTAGTTGGCTTTaCTTTTCGAATAAATCCTTCGATACTTTGATAGTCATAAAACGGAATTAAGTGAAATTTGGTATTTCTTCCATCCAAATAAGAAAGTCCTGTAATTTTATTCTTTCTCCATACTATAAGACTGCCCGGGGGAAAAGTCAATCTTTCATTTTTTtGTGTGGAATAGGCTATGTGTTCTGTTATTATACCCCGCTTGTTCCACCCAAACAGTTTTCTTGCATGTATTATTCTCACACGAGGTTCTTGGTAAAACAATTTtACTAAAGATGTCAATTTGGT

>contig00114

TAGGTTCCTCACCTGAAAAAAaGGTAaCTGGTAGGAGAGCGGACGTGATATTGGCCGCGcATTACGCATCTCAAGctGCaCTAaGACTTCTTAAGCCAGGCACTGAGACTTACATGATAACAGGAaCGGTAGAGAAAaTTTGTGAAGCCTACAAGTGTAAACCTATCGAGGGAATGTTGAGTCATCAATTGAAACAATtCAAAaTCGACGGAGAGAAGACCATTATtCAAAATCCAAACGACGCGCAAAAAAAGAACATGAAAaGTTTACCCTTGACACACATGAAGTGTATGCTATGGaTGTTCTAGTAAGTTCAGGAGACGGCGTAGGTCGGGAGcAGGACACTCGTGTAACTATATAtAAGAAAACTGAGGAAACGTATCAACTGAAATTGAAAACCTCTCGgATGTTCTATTCTGAAGTGACACACAAACATGGTCTTATGCCATTCAATCTTCGT

>contig00115

CATTTTGATATTCTACCTGGAACTCCTGTATTTAACCCCACATATGGATTGTTATATGGACTTACTGTAGGAACCAAGGATGAGGAATTCATGGTTCCATTTAATTTAATTTACAAAATTTTAAGGATATCAAGAAATATATAGA

>contig00116

ACCAAATGGAGAGTAGACTCTTtCTGGATATTGTAgtCGGACAAAGTGCGACCATCTTCAAGCTGTTTtCCGGCAAAGATCAGtCTTtGTTGGTCtGGAGGaaTTCCCTCTTTA

>contig00117

TcAGTTTCAAAGCATTCTTCAACGAAGTCGAAAGTATTCACTGTGAACTAATTTTtCTCTGAGAAGATATTTATTTGTGTTCCCGAAATACACGGCATATAAGTAATAGGCAAACATGCAAATTTTCGTGAAAACATTGACGGGGAAGACCATAACTCTTGAGGTTGAGGCTTCTGACACCATTGAAAACGTGAAAGCGAAAATTCAGGATAAGGAGGGAATCCCTCCTGACCAG

>contig00118

TCACCTAACAGTAAGCTGGAATATTTCGGAAGAGCTGCCACACCACT

>contig00119

ACTAAAATGTATAATGAAaCTAATCCGATATGAAAaTAATCGCTTTCCCAATGGCTGCTTTATTGTCCAGTTATTAAGCCTTAGTTTtGCGTCTTCCGAACTTTCCACAGCGAGATAAATTCTTCACTTTTGAGGGTTTCAGACTGCAAGTATCACCGTAACAGCCGGAAGAGTCAGATGAAATTGAATCATTTCGACGAGCAAGTTTTTCTTTTGTTTAGCAGTCCTACCCGTCTTTTTGGGCTTTGGCGGCGCTTTTTTTtAGCCCCTGATTTTCGCTTCGGTTCAGCTTCGGATTCCCAGCAGCAATCTGAGTAAGAACAGGAACATTCCGATAAAGAACATGAGCAATCAGAGGAGCTGTCTGAACTATCAGTTGAAGATGAGCAGAAGCTACTGCTCGATGCATCCGAATCAGATTCTGAAGATAGAGCACAACAATTTCGAGTTGATCGCGACCTTGATTTTGATTTTTTCTTCAATGATTTGATTGGAGCCATGTTCTAAGAC

>contig00120

CaCAAaTTACGTAAGTAAtATGTATAACCCcTAATTTtCAAAAATAAAAAaTATTATCAGGCACACATTTAATTAAATAATTTtAATTTtAAAaCTTTTTAAATTCTATCCTAATTAAAaCGTATATTTtGCAAATACATTTTCTGTGCGAATACTTAAATGTCAAAGACCTTGATTtAAAATTGATTTtAATCTACAAATTTCGTTGCAGTAATAACTTCAAAGATtATATAAaTATTTtGGGCTCAACATTTTCAATATTGATTCGAAATTtACAATCATCGAACGTTATTTTGAAACGTTTTTCTTATCTTTTATATCCCTGCCTTGAAACTGCACtGG

>contig00121

ATGGGgAGTCTCTTatGAGTCACTAAACGAAGAAGATGAAGTTTGATATTGTTGctGTATTTTtCACTaTTTTAGCAGTCCCACATTGtgAaTCTGCATTAGACGGAAAAGGgcTtGAATCTAAGAGCCAGCCTATTGATATCCGGCCTGCAATCTCATTAGCCTTCATAACAGACAAAATACAACTAAGAATGGGCGCGGCCGTAGTGaCTCCATCCTGCGCTATCACTCTTAGAGCACCTCTAGACAAATATGCTGACAAAACAATTGAATCTGGAAAACTTTATATTGGAATTGGAGCAGAGATTGCTAATATACAGGATgaGAAATTCTTACATAGTGTGAAAAGCGTAACTAGCAATCAAGGAACGTCAACGGCTGCACATTCGTCTCATCTtGTTGCAA

>contig00122

CGATTCTGACTCAGATTCCGTGTAGTAGCATCTAAGAACTCTTATCATATCTTTCTGTAGTAGTTGCATTCCGGGCTGGTCGGCAAATGACTCATTTGATGTCTGAGCACTTTTTTTGATTATCAAATTTAGCTTGCTTCCGTCCTTGATTCCTGGGTAGAAACTCAGTGGGTTCTCG

>contig00123

TTgTtATTtCAAAGAGTATTtACTTCAATAaTTCATCAACTGTAAAAGTAAACcGTGTGTTGCAGGATTTAACGATTAATAAGTGTCATCAACAATATGAAAATCACAGTAAAAATTCTGCAGGGAAAACAATGTGTAGTTGACATTTTACCTTCAGAAACGGTTTTGGAACTCAAGCACAGAGTCAGTGCTCTTCTAGGAATCGACGTATTTCATCAAAAATTATTACTTACTGGCAAAACACTAGATGA

>contig00124

ACTGttCTTtGCAGTGGGTTCAGCAGCCTTTTtGGTGGCTGATTTACTTTTAGCTTtCTTCTTTGAGTCCTTGtCATCACcGACTTcGGCTTCACCATTCCCTACAATTTCTTGAGCGTTTCCATTCTCCTCTAGTTCTGGGGATTCTGTTTCCTCCTCCTTTTCACTTTCCTC

>contig00125

TGCATTTTCGTCGTCAGCTTCATCGTCCTTGGCTACCTTTTtACCTCTTCCTTTTTTCGAACCAGCAGCTTtCGTTTtCTTTTTtGTCTTTGAATCGCTGACTTCCTCCTCTTTGCTTGGCTTATCATTGGCTTGTTTTTTtCTCGAGTTCTGGTTACTTGTTTTtGACCTTGATT

>contig00126

AAACATTCGAGGTTCGAGCGCCAACGTGCAGTCGCGTGGGAAGATAACAAGCTCGTGATCCGGCAGACAAAATTGTATAAAATACCGGTGAAACGCGATCGAAAGCTGCCAAAAGACGCCGTGCAAACTTTCTTTCGGACTTAAAACTTTGGACCAGATCTTCATCTCAATTTCGTCTTCGGCAGTGCACCGTGAAGAATCTAAAAAATCATCTCGAGTCCGGCGCGTTTATCGCTAATTTCAGTCCCGAAAACGCGGTTGATTACAGAAATGCCTCCGAGAAAGACGAACAAGCGTGCCGACGAGGAAGTTGCTGTCGATACTCCTGCTGAAAATACAGAAGTATCTCCTCCAAAAAGGAAGAAGATGACTAAAGCTGTCTCAGAGAAGTCCGATGAGGCAAGAGAGCCCCGATTTCCCAGGGCGGCAAAGAATAAGAAGCCAGAAGTCACGCCAGAGGAGCCGGTAAAGAAGACCAAAGCGTCGTCGGCTAAGAAGAACGACGCCGCCAAGAAGTCTACAGCTCCAAAGTCTAAAAAAGGCAAAAGCACTGACGATGATGAGCCGATAAACGACGAAGAGGTCGATGCTGCATCGGATGAAGCTACCGAAGTTTCCAAAAAAAAaGCTCGCGGCAAAACTACAACGAAAAAGGCGGGCGTCGATGATCAAG

>contig00127

TTCGCAGTCAATGAACATGGATTCCCTAGCCATTCGCAATATATACCATTAGGGACGACGACTTaCAGGGATATAAATTTTCCATACATAAAATCGATTACTGCAATTGCAGTGAGATGGGATCGAGAATACAAAGAGACCGATTTTTACAATGAGTTGCA

>contig00128

ATTCTACTGAAAATATTCTTAACTTCAAGTCGCATTAAGAGCGTTTTGTTGAATCTCGGTAAAAGAGGATTTCAAGTTGGCCTGTTCCAGTTGAAGTTGATAAGCGTGCAATATTATTGTAATAGTTCCAGTAATATTTTACGGAGTACGTCAGTCAAAAATGTTTAAGTCAGCTATAATATTTTTAATCGTTTTCACTTCATTAGTTGTCTCCGGTGACGAGTTTTACGCG

>contig00129

AATTGCGGCTATTGCTTAGGAACAGAGGATGGAAGTTCCTGTATTTCGTGTGCAGAGAACTGTATTGGTTTATCCAAAAAACTCTTATATTACATCAAGGACGTTACATATACCGAATCCGAATTACAATGCGGACAAGATTGCAAGAAGACCAATAATTGTAACTAATAGTAAGTGTGACCGTCAAAACATCTACCCTAAGGTTGACTCTACTTCAACATGATGCATGCCAACCACAATGAATTATTtAA

>contig00130

ActtGGGgATTCTATTAAAGATATTCTTAACGTCAAGTCGCATTAAGAGTGTTTTGGTGAAACGGGGTTTCAAGTTGGCCTGTTGCAGTTAAAGTTGATAAGCGTCCAATTTTATTGTATAAGTTCAAGTAATAGTTTGCGGAGTACGTCGGTCAAGAATGTCTAAATTAGCTATAATATTTTCAATCGTTTTTATTTCATTAATTGTCTCCAATGACGCGGGACTACAAGATT

>contig00131

ATTGCAAAAATTGCATAGAATACTCTAAAGAAAGCTGTAAGAAGTGTGCAAAGGGTTGCGCTCATACATGGACACCATTCAAACATGTTATCACAGCACAGGCAACATATACCAAATCCGAACACAATTGCGCAGAAAATTGCAAGGACGAAAGGTCTTGTCCATAATACCAAATGTGACTGTCAAAACATCCACCCTGAGGTTGACTCTACTTCAACATGATGCATGTCAACCACAATAAATTA

>contig00132

TTGAaGAACTTtCGAAACCAAAAaCAATCCAAGAAAAAAATTaCTGAAAGCCAGTGGGAGTAAAGAAATAAATAATGGATTACGTGTCCCAGGTGCAAATTGGGAAAAGTATAAAACACTTaTCACGGAGTCAAAaTTGAAAAATAAGGATTCTGGAGAATCGACGATGATCAAATCGCCTAGAAAACGTCAGAGGCCTAAAAAAaTCAATAATCGGCTTGAGAATTTACAAGTCGACATTCAATCGAAAACTCCGAGACAGAaTGGGACTGAACCAAGCGGGGAACCGATTTTAACGAAACAAATAGCAATGGACTGCGAAATGGTGGGAGTTGGTGATGGT

>contig00133

GCCAAGCATGTAGAGCACGCCTCGTATCAGTGAATGCAGATAAAGAAGT

>contig00134

TTAAGATCTTGAAATTAAGTCCTTGACACTATATAATATTCGAAAA

>contig00135

ACTCTTTATTCAAACGACTTcAtaTAAtGtGTATAAATTTtAATAGAGTTTTTttATCAGTCCAATTTGGGTtCCAGAATACGACCTTTGAACAaTGGGATATCGGAAATACCGATCGGgTATGAAGACTCATCTTTaTGGATTACAGAATCAACTAGTATAAAGAAAAATGGCCTGTTGACTAGGAACGTTTTGGGCGTTGTTGGAAATGCAGAATAAGCAAAAACTCTAGCC

>contig00136

ACCAACCATGCATCAGAAGGTCAAAaTCAtGtATGGAGAACTTCCAGAACTGAATGCCAAATTCGTGGAAATTCCTTACGTGGGTGACGAAGTTAGCATGATCATTGTTCTTCCTGATGAAATCGATGGCCTTAGTAAAGTAGAAAAGAAACTGGAAACTTTCAGCCTTGACAAACTCAGACAAAATGGAGCACTTACTGATGTTGAACTCTCTCTCCCGAAATTCAAAATCGAAAGTAAGATAGACCTGAATGATGTCTTGATAGAAATGGGAATGGGTAAAATGTTCTCCAACGAAGCTGACTTCAGTGGAATTTCTGAAAGTCCACTCAAAGTGAGCAAAGTTGTTCAGAAAGCTTTCATTCAAGTTGATGAAGAAGGTGCTGAAGCTGCTGCAGCCACTGG

>contig00137

TATGACTTTGAGTAGAAACTTGATAATTAGTAACACGGTTACATTCAATGCTAATTATCCGTTCTTATACATAATTACGAGTGGAATTAATACGAGTCTATTTACTGGTCGTATAATGAATCCAaTAGTTTCTTAATTCAACAATGGTCATAAAGGTTCGATATTTATGTTAACATCTGTGAGAGGGATGCCGACAAAaTTCAAAGCAGACAAGCCTTTCa

>contig00138

CTTGAGTTGCTGTGGCTGCAGTTGTCGAAGCCTCTCCTGATCCAGTCGTTGAAACAGAGCCGCAGTCCACCTGATCTTCCTGCTTACCATCCAATCTGTGTTGCTTCAACAAaGTGCAATCaCCtCCcTCGTTTGTGTCTAGGTTATATACATACAAGTATCCATCCGCACTGGCAACTAGCAGCCTTAAAACTTTCTGTATCGTAGTGATGGCACAGACGTTTTTCAAACCTTGAAAAGGTaGGTGAACACTAGCAAAGGCACGCCCCTGATTAAACACGTCGGTGACCTGTGAGGGTAAGTAGTTTGCGGACGCAGAGACTGCCTTCGTCAAAtATCCCATCCAACTCTGAGCCTCTTCTGGCTGTTGCTGAGGTGCTTCCTTGGGTTCTTCGAGCTTGAAAAT

>contig00139

ACttAAAGttatCGAAGAGAATAATTGTCACAAAAGATCTTCTCAATTTCTTTTGAACTTTCAAAGTTTCTTTAACTGGAAGCTtcaGTAACGACTTGAGGATGACGTAaTGCTGAACAAAaTTCCAGCTAGGTCTGCCTACCTCTTGTCTTCCGAAAAATCTCTTGGTCTTCTGATATGGGACATTTCTTTATTTCATTCTGTTCTTTGAGTAACAGGTGGGAACTCTGTCTCGTCATCTAGACGAAAAGCTCCACCTCCTTTTGGTGGACTTTCGGTCGCAGCTATCCACTCGTGATACTTCTCCGATTCTG

>contig00140

AAAATGAAAAAAAaCaTaCTGTCGTTGATTTGCTTCGTCAAAGAGAgaCACAAGAATCAG

>contig00141

ACAtGGGgACTGATGTGGGGAAACTGAGAACGGAAAGTATATGGGACGTATAAACGAAACAAGAGATATATAATAAGATCGAACCCT

>contig00142

GAGGGAACAGTCACTGATATCAACGTGAAGAATAACAGCTGTTATCGTTTTTCTAACCAAGCAACTATACGCACTTTTGACGGCAGTCGCAGTTTTCCACACAGAAGAATTAATACATTGCCGAGAATGAAGTATTTGGTCCTTTTACTTGTTTTTGCAATTACGGCTTTCGCTAATTTTGTGGCAGGTATGCCTCGCTCAGTGAAATATTCGGATCCTATTGCACCAAAGCCTGAGATGCCTTATGTGGATCCTTACGCACCATCGCCAAGCCTTTCGCAACACGATCAGGAaCCTAAAAGTGATGATTGAAGATTTCCAGAGAAAAAaTGTAGAATGAGCTGAAATGAAGAAAATGGTTGAGATAAAAATGTTAATTCTTAGAATAGATAATTAATAATTAATTATAAATAATTAATTAATTCTTAAGTTAGATAATAATGTTTtA

>contig00143

CCGTTTAACCAGTGTTAAAGAATAAGTCGGTGATTGAATTAGCTATAAAGTATTTTTtCTAGTTTTTCTGTGAACTTTAAGCAATAATGTCCGACAAGAAAGCATATTCGGTTGGCCCACATCCATCAGGAGGTTTCGTTCCTGCTCCAACGCAACCAGCTACCTATGGTGTTGCAGGTGCTTCACCCTACGGCGTCTTTtCGAATCAGCCACAGCTTTATGCGGCGCAAGGACCTCCGCCACCCACATATGATCAAACACTTACACATCCAATG

>contig00144

TTGAACTGCCAAGGGATCTGTCATTGCGAAGAGAACTACCTGAACTTCGCTTCAAGTCAAAAAA

>contig00145

TGTATCAGCCGATGTATGGACAAGGATATCCAGGTGGATACTTGACTGGTTACCCAACTGCTTATGGTCCTCTGCAATATTACCCACCACTGGCTGCTGCTGCAGCTTACTATCCTGCCGCTGCTATGCAGCCTGCGCCAGTCAGGCCCACaCTT

>contig00146

ACATGGGgaCACATCTACGAAACATATTAGTTCGGAACTTTAAAGCGCAAGTGAAATGTTATTGAAAGCTCTTTTGTTAGTTACCGTTATTTCTACGGTCGCAGCTCTAA

>contig00147

ATTCCAACCTGACATTATTAGCAGGTAAACTTTCAAAAGCGAACGAGGCTGAAACTCCGGAAAATTTCATAAAACTAATCTCTGCAGTAATATACAAACCTGTTTCAACTGTGAAGGCTACTGCAACCTTTGTAACAGGAAAATATCTCTTAACAGCAGCTGGATTTCTCTATGATACAAAGTATAAAAAAaCAGAAAAAGAAGATTCTGGGAAAATCCTATCTATGTATTTAGCAATTCGTACCAGTTTTGTAGATGATATATTACAAGTGGCAAAAATTTACAACAAAGAAAGAGCAAAAAAaGATGATTATAGAAAAGAACCTGCAATCCTACAAGTTGCAGATCAAAGTAAACATTTAAAGGAAGGCTTTCCTGAAATACCGAAATCGCAACCTGATATAGGATCACCCGTAGATATATTTGGAATGCTAATAAAGGATAAAAAaCATACATTTGTTGTATTATCGTTTTCATCTAAATTTTCACCAAAAAGCCAATGCAATGCGATGACGAATAAAGGTGAACCAGAAGATCCAAATTCTATTTGTGTCATCTGTGAAGAAAAATCAAAACCTAAAATTACTACAGGGTTTGATATAGGTATGCCTATCTTAACCAGCAAGGAGATAGTAGGAATGTTAAGGAAGATGTCAGGAACTTTGCACACAAAGTTACTTTTCGTCAGt

>contig00148

ACTGCTGGACACAGTTTCTGGATTAATAAAATTTTATTCGGCAAGGGTGACGGAAATAAAGGCAAAAATGAGGACGAAATGGAGGTCGTAGATGAGGAAACTGCAGTTGAAGATGAGTAGACCTGAAAATAATGAAACTAATGATGATGAATAGATGGTGGGAAAACTATAAATTTACAAAATTAAGAATAAATAATTTCAAGGTAGTGTTACGTCGTGTAAAAATAGGTCTCTGGGAAAAAaTATTTAATCATACTTTCAGAAATTGTAATATTATTTTTGATAATTTTGTCCCAATAAAGGAAGGTCATTTTTTGAAAAAAaTATTAACAACAGAAATCATTTGTTTAAAAATTTTtATTTCTGACCAAATTTTTTtATAAAAaTAAAAaTAATTGTTTTtAAAATTATGGGGTTTTATAAATAGTATTTTCTCCTCTAATGATAACGTCAATAATAGTACTGGAAATTCTAAAAGTATGGTTAAATTATTTTCCTGCGATCTGTTTCTTATCAAGGCcAATGCcTTAAAAaTtCGTTTGCTGATAATAATATTATTATTAAAaGTGTA

>contig00149

CAGTCGAACCTCACATTATTAGAAGGGAAACTTAAAGATGCAGACAAGGTCACTACTCCGAAAAATTTTATAAATCTGATCTCTGCAGTAATATACAAACCTAAGTCAAATGAGTGGGTTACTGCAACATTTGTAGCAGGAAAATATCTCTTGACAGCAGCTGGATTTCTCTATGATAGCGCTTATAAAACAAGTGAAAAAGATAAATCTGGGAGAATCCTATCTGAACATTTAGCAATTGGTACCAGTTTTTCATCTGATATATTACAAGTGAGAAAAATTCACAACCAAAAAAaGAAAaCAAGGGTGATTATGCAAAAGAACCTGCAATCCTAGAGGTTGACGACCAAaGTAACCATATAAAGGGGGCTTTCCTCAAATACCAACATCGAAACCTAAaTTCGACACACCCGTAAATGTATTTGGATTGCAATTAAAGAATGAAAAATATAAATTTtCTGTATTATCGTTTACATCTCAAATTAAACCAAAAGGCAAATGCAATTCAATGTTGAATCAAAATGAAAAGGATGATCTGAAGTCTCTTTGTGCCATCTGTACAGCTAAATTAGAGCCTCAAATTTATGAtGGGTTTGATAGAGGTATGCCtATCTtATCCaGCATGGAGaTAGTAGGAaTGTTTAGGAAGaTGTCAGGAaCTTTg

>contig00150

aCTGATCTATCGGTTTTCTCTTGTTTCACATGAACAGGATATTTGTCTACTGGTTTGTCAATGTATGGGAGCTTATCACCCGTGTAAATGAAAGAGTGTGAGTGACCTCCAACAATTATGTCAACATGGGGACATTTTGCAGCCATTATTTtATCAATATCTAATCCGCAGTGACTTAAAaCAATGATAATATTTATTCCTTGATCTTTtAGTTTTTtGATTCAGCTATCACTGTCTCTACTTCATCCAAAAAGCTTACATTTCCTGTATTTGATAATTGACTCGTGGTAGAAATAATTACACCAATCACGCCAATTTTTCGGCCTCCTCTGGTAaTTATAGTGCTGTTTACGAAAaGATCCTTCAATGTTGGTTCCAGAGTGATATTCATGTTTGCTGCAACCACAGGACAATTtATTGCTTCCAAAAatGGT

>contig00151

GTCTCCAGCATTTAG

>contig00152

CTTCACGCAATTTTTtACTAGCAGTCTTGACTCTGGCAATACCTCCAATGCATTCCTCTTCCTTTGAGTCATCGCAGGTACCGGACGTAGGTCCGATTTCCAAAAaTCTTGCATGAAAGTCGTTTATATGAATCACAGATAGCTCGAATAGATCTGATCGTCTATAATGCGTACTAGATGTTGTATATAATCCATCACAATTTTTAAAGGCAAATAAGGACAACCACAAGATCACGAATCGACCAAACATCTCTCTTCAGAACTGTTAGAAGACGTCATCAAATTTTTTtGATACCCATTGACAAGTCGACACTCGCGTCGTGAAAGGTCGAATAAGACTC

>contig00153

TCACACTGTTAGAAGTTTTtGCGGTAATTtCCTGAAACACGTgACTGGAGTTCTATTCAGTTACGGTTTCATGAAATTGATATAAAATTTCAGAAaTTTCGGTTACTTTTCAGTAAaTTtAAGAAaCAATTTCTtAATTTtCAGTTACCATGTGTTGAATATTCTGATACTTGTGTCTGGAAATTTTGAAACTTGAAACAGGAATAATGTAACGTTTTGGGTAACTTCAGCTTTATGCAGTTTATTGTACATGTATATGAAAATTTTGCTACATATAACTGAAAATGTTACAGTTTTATATAAGTTTATGATAAATTCTATAAAAATCCTGTTACTGTTTAGTAGTTTTAAGATACATTTTATGAAGaTTCATTTAAATTTCATAATATTAAGAAAATTTCAGAAATTTCAGTACATTTTTCGCATTGTTAAGATACCTTTACTCAAAAACATCATAAATATCATCAAATTTTACGTTCCCTTTGTAACATAAAAaTTTtGgAaaTTTCCGAACATTTCTAACAGTGCATTTTAAGAACAGAATTCACTTAAtATTATTTACTgAaTAACATTATACTtAATTCTAATATATAGTACGAaGATTATCGATAATATGAATCATAAGAACACGTTAAAaTACTTATTTTGTAAAaTATTATTGTATCCACGCAAAAACACCAAAAaCTAATAAATATAGCCcAGAAACGTCAAGCAtcGTtAGTAACCCCCTGAAaGAtGTGTTTTACTTTTTCACCTCCAAGTGACTTGCTCTTTCACGTTCAACATAATTTGTTGTAAAAGGATTTTGCTCGTGATAGGAAATCATTAAAGTGTCTACTTCGGTGAAATCAAACGTTCGCAAAGACTCCCAAATGACAAATTTCTCCATACCACCACTTCCTTCTTTAATTATCTCTCTTTTAAAGGAAAGCTTTCGAGAATGTGTCTCGCTTTTGTATGGCCCTCGTTCGACAGAGGCTGCATTATAACTGTGACTCGCTACAAGGATGAACTCTTGTTTAATTTCAGATGCTCTTATATATCCGCAACTAGAAAACAGGGGTGATTCAGTTTtGCTGTTCCAGGATTCATAAACGAAGTGAATAAATGAACCATAATTCACAAAAACAATCCGGCTTGAGTATCTAATGTGTTTACGGTTTTCATAATCATAAAATGTTCCATTTGCTTCATTTTCCCAGGTTCCTTTAAGAAAAGATATAAGCTCTAATCTTGAATCTGGGTCATTGCAATCGCTTAAACAAGCACTGGTATCTTGCGAAAAAGT

>contig00154

GATtAaGAAAAAaaTTCtAAtGGATGACATTTtAACAAACTTGGAAAaCTTTTAAGTAATACGTCGCGAAGACTTTGCAGtGGG

>contig00155

GCCACTGCAAATTCTTCGCGACGTATTACTTAAAAGTTTtCCAAGTTTGTTAAAATGTCATCCATTAGAATTTCTTTCTTAATTTTTTtGATCAGTTTTTtGCAAAATACCAGTGGCGATTCCAATGACAAAGATACAAGATTAGAGCCTATATCTTTTTtAGAAGGAACCTGGGAAAA

>contig00156

ACCgaCAGGAAaTACACAAGTCGTCCTGGTGAtCCTTtATTTTTCATGAATCGTCTTATAGGCATTTTATTGGAAAAAaGCCGAACATGCCATCTAACTGCCTTTTACTACAGATTCTCCGTTCCAGAAATTGACCATTTGCTCACTCTATAATAGGATTCTTAAAAAAaTGTAATTGACAGtAATTTTTtATtACTAACAATAATTTCAAGCAATATCAAATACTGTTCTTTCTtGACGTCAGACATAAGAGCTTCTAGATAATATAACGGCATATAGAATTATTTGCTTATTTTtCGACGACCCATAACCTTGATAATTTTtAgAaTTTCAATCTCCAAACATACAATCTtACAGAAAATTCAGGAGCTCAACATTTTTCCCCTAtAAAAAAaCGTCGtATCTGATATTATTTTtGAAAAAAaTGCTATACAAAATTGTCTAAATAATTCTATGTGCCCCTATACTATCTAGAGGCTCATaTGt

>contig00157

AACAtGtAAAAaTTtATCAACAGTTTGTTTTGGTTTTGGGTAGTTATATAGAATTCCTGCTAGTCTGTTATTTTGTGTtACAAACCCACCTAGGCACGCATCATCCAGATTCACCAAACTGTTTGCCTtAGGTATCATAAGTATATCAATACTATGCTTTACATCCTTACTTACAAAGCTTTTGGCAAAATTTTCACTtCTAATTCCcAGGAACTtGTTCCACATCACTGCCcATCCGTCTTTTACTATTtGAAAaTGTTGtCCGTAAGTAGCCcTTTCCGCTGGAATGAATTGCAAATCTtGCGgTATTTTtGTtACGTCGAGTTCGAAGATtACGATTtGCAacAGGAGGATGCAGACCTGCAGCCTCTTTtAACCcTtCATTGCTGATTACGCTTATCACGTtATGTAAGAACTGCTtaTtATTtATATtAGCATTTTGTGCTCCAACTCCAATATAAAGTTGTCCAGAtGtAATTTTTTtGTtAACATATTGTGTTaGAGGTTCTTTGAGAGTGATAGCGCACCTTGGAGTCACTAAGCCCGTgCTCGATAGTAaTTCTTTTGTGTCTGTtATGAAACCTAAtGATTTTtCAGGCTGAAAACTTATCTTCCTAGATCCAGGCCcTGTTCCGTCTAATGCAGATTCACAATGTGGGACTGCTAAAATaGTGAAAAATACAGCAACAATATCAAACTTCaTCTTCTTCGTTTAGTGACTCACAAGAGACT

>contig00158

TTTTCAGACAAATGGCTTCTTTATGATTCTCCATATTTGCaCAGcGATAATAaTCCGGTTTAT

>contig00159

AGtATCTTCAATCTTCATGAGTGATATc

>contig00160

ttAGCAAACTATcgCcAACTATATATCAAGTATTACTTAGAAGTTTATT

>contig00161

TGAATTATATTTTtGAGTAGTtCGGCAACTAacGTgTAGAATGACTTAATATACTAGATGAAAaaTAaTTAAATGACAACTGAATCCAAGCTtaGGaCAAgAaTTTGCTACTGAACAAAAATTGAACAACTTAGTCTGGAAGATAATTTTGTGATtacATTTTTTACCATTATTTtATTTTttCGTAaACAATTTTACTTtGCCAGACGAAATaTAATtGTTTCAGGGAaTTAAAAAaTTtGATTGATTCTAGGTTtCTACAtgTTTTCACTGTGAAGTAAAAaTGTGGTAAGAACCCAGAATTTAATGTtGTGTTGActtGCATTGTTTGAAATCACTAAAGTATCATTTtAGTTTtGATTGTAGcTTTTTCAACTCGACGagAAGt

>contig00162

ACATCAGATTtCaGAAAaTAAAATATTTtGTCACAATCAGTGCCTTGTAGAATATAGAAGCCAGCAAACaTGCTGCCGTCAAATAAAAcACCGCCaTACATTTTCAGACAAATAtCTTCTTTAcGATCCTTTaTatcGgCACAGaGATAGGAATCAGATATATCGAAT

>contig00163

CCCGCGGCGATTAGAGCACTCaCGTAAGTAAaGCCACAAGTTTTCGTAGAGGcAATGAGGCGCTTATTtACGATGAATCCCACACATTTCGTCACcTtCTCCATTTtGAACTTTACAAGGAGTTTTATTTGATTATCTTCCGCAGAAaCTGTCTCTTTTTTCTTACCAGTCGTTGCAGGTGAAGCAAACACTCTAAGATTTTGCAATAAAACGATCAAtATAATAAGTAAGCCAAGAGCAGTGGGTGACATTCTAACAAATTG

>contig00164

AGTTTACGTtCGTTCACAGTGTGATTCACTCCAGTGAGTTCTATTTATTTGATTTACAACTCTTATTGGGAGTTTTtAAGTGATCAGGATGTCGTCTCGCAAAACAGCTGGTCGTCGGGCCACTACCAAGAAGCGTGCACAAAGGGCGACCTCCAATGTGTTCGCTATGTTCGATCAGGCTCAAATTGCCGAGTTCAAAGAAGCTTTCAACATGATTGACCAAAACCATGATGGCTTTATCGACAAGGAAGATCTACATGACATGCTGGCCTCTTTAGGAAAGAATCCAACTGACGAATACTTAGAACAAATGATAAATGAAGGTCAAGGTTCAATAAACTTCACCATGTTCTTGACACTTTTCGGCGAAAGACTTCAAGGAACTGATCCTGAAGACGTAATCAAAAACGCATTTGGCTGCTTTGATGAAGAAAATAAGGGTCACATAAACGAGGAGCGACTTCGTGAACTTCTTACAACGATGGGAGATCGATTCACGGATGACGAGGTGGACGAGaTGT

>contig00165

ACCGtgAAgCTCCGATCAAAGGATCAATGTtCGATTACATCGAGTTCACTAGAATCTtAAAACACGGAGcAAAAGATAAGGATGAACAGTGAGAACTTCTAAAAAGGTTGCCGAATCTGAAGAACATCGTAGGCAATATGGATTTCCCGATGTTCACTGCCAGAATAAGATTTTTCATCGACTTtCTTAAAATCAGATTAAGCATTTCAGAAATTATGGAATGGTTGGATATCCACAGAATACCTTTGATTtCcTTATTTTATGCAGTTAATTATatCACTGCTGTATTGCAGTTCTTTTTtATCCGTCCcTCATCATTCCTATCGGGTGGGAACAATATTtaTGTCGATAAGTGTAAGAATGATGATTGAGGGACTAATAAGCTGTTTCCAGGACTtAAAAAGTAGTTTAAGAAACTTTATATGAGACAACTAAGTTATTtCCAGAACCCTCTGCTATGATTTACCGGCTACGTTGTGTATAAAAGCGAACTTATATAAGTATTCTACAATTATTCATATACACTTCGACTCATGTTTACCAAGGAACTGGCATTTGTATTTtGATACaTTaTaGAAGt

>contig00166

GGAACGGAATTTCTCTTTTATGACAAATTTAAGAACAAAGAGAAAGATAATTCTGGAAGAATCCTACCTAATCATTTACAAATTGGTCCCAGTTTTCAAGATAATGTATTGCAAGTCACAGCAATTCACACCCAAGCAGGTCTACAAAAGGCTGATTATTTAAAAGAACTTGCAATCCTAGAAGTTGAAGATCAAAGTCAATATTTCCCGGGAGGCTTTCCGCGAATACCAACATCGCTACCTGGTTTCGACACACCTGTAGATGTATTTGGAATAACAATAGATTATAATCAACGTAAATATTCTGTATTACACATTCCATCAAAAaTTAAaCCAAAAAAaGACTGCAATTTtCAAATACTTAAAAaTGAAAAaGAAGATCCGAAGtCTTTtGTGCCAGCTGTGAGAGTAAAAAaCAGCCTTC

>contig00167

TATGCCTATCTTAACCAGCGAAGTGATATTAGGAGTGTATAGGCTTATGTCAAAAGTAGTC

>contig00168

AATGGTGgCGAAAATAAATGCGTAAAAGAGGTCAAAAATGGGGACGAAGAAAAAGACAAAAAAGAGGTCATAGATGAGGAAAATGAAAATGAAGATAATAATGATGGTGATGCTGATGCTGATGATGATGTTGAATAGATAGTGGGAAAACTTCAAATTTACCAAATTAAAAATAAaTAATTGTTATATTATTTTtAATAA

>contig00169

GTTCTTCTCGAGAAGAAGATTTTTCACAGCTTGACTATTTCCAATTATGAAAATTAATTTCTCATTGCCTTGAATCATAAGAAAaTATTTTTTtGCAAATAAAAAaTTCAAATCAACAAAATTACTTGACATTGGTTTtATTGGATTCAAAAAtCTAACGCaGTTAATTGAACGCAATCTTCTTTCCCAGAATGTAAGTTCTTATCCATTGAAACCTCATTAACCTTGGGAAATCGGTGTTTTtAAGATTATCTAAATAAATGATTTCTGTATGTTAAAaTTTGTTTATAAATAGTCTTTTTtCCTCTAATGTTAGCATCAATAATAG

>contig00170

TGTCAAGAGAAATTTtCCTTTTACAAAGGTAGCAGTAGCGATTGCAGATCCAGTAGTACTGTATATTACTGTAGAGATTAGTTTTATGAAATTTGCCGGAGTTTTGGCTTCTGCTGCATTTCTAAGTTTACCTTCTAATAATGTGAGGTTCGACTGTAGAGCTGCGACCGTAGAAACAACGGTAACTAACAGAAAAGCTTTCAAAAACATTTCACTTGCGTTTTAAAGTTCCGAACTAATATGTTTCGTTGATGTATCCCcaTGTAaCTaCTAGaCGTTaGAAtAACCAaCTAGACT

>contig00171

GATGATGAGGACAGTCCTAAAACAAAAATTAAAGTTGAAAAGAAGCTATTAAACGAATCTACAGATTCGAATTTATCCGAAGAGGCAAAAGAGGAAAGGAACAAGAGATCTATTTATGTTGGAAATGTTCCTAAGGAGGCAACGCAAGCTGAATTGGAG

>contig00172

AAAAAaCAAGGGTTTTAAAAGATCGCACTCTTCAAGCTTTGACAAATCTCAGTCACCC

>contig00173

AAAAaTAAAaGATGAAAATGATGTCGCTACCCATAACAGTAAGAACGCAGAAAAACGAAAGGAGAAGAAAAACAAGAACCGCTCGCCGGGCCAAACTCAAAGCTCAAGCGAAGGATCTTTAAGTCCTCAAAAGGATCAGAAATCAAATTTCCAGGGTCAAAAGGCTGATCAGAAAAAAAaGCAAAAGTCCAACAAACAGGATAAGAAAAAGAAGATTATGGCTGAGAAACTAATGGCAAAGCCCAGTAAAAAGATGGCTGACTAAATTATTACAGTTTTTTAATTTAATTT

>contig00174

TGGTATATATATATATCTAAATATATATATATAtatataGATATATAaGTAAAaCAAATATTGATAtAATTTAAT

>contig00175

TTTTTAGTCAGGATAGAGTGACGCGCGATGTGTTATAGCATTGCACGTTAGAATCGTGGCTATTAACGTTCAAATATTATTGTTTCACACTCCAGAATACGTAAATGATTTGTAATTCAGCATATTCGTATTGTTTGTTTATCCGCTTTTCTTTGCGTTTCTTTAACGGCtAACTTTTtCAACCCTATTTTTtAATTAaTAATGATTTATGAACAATTATGT

>contig00176

aCTCGAATTAGCCTCTGCAGATTAAGACTACTGTATTTAACGAACACGGAATTTATAAGACAAATGACCAGACTACAATTTTTTtAtGGGAAaTCGACTGAACaTTTTCGTttAAaTTCATAaTTtAATAAGTCTATCTGCTTTATAAGAGGCTTTTTCTTTTCAAAATAaGTTTAaGGTAC

>contig00177

GCGTGAAAGTAAAGTTTGCGTTTGTACAATCAAGGACTGGTACTGAAACTTTTAGTATTTTTtGGGTTGTTTAAAAaGTCAGATTTTTTtaCTGAtAAAAACTTGAAACCCTGAAGGACTCAATGATATTAAAATTAGGAaGGGGAGTAGAGCGTGAATATTTtGAAGCAATACCAGTCCCTGATTTTtATTGCAATGTTGGTTGAGTTTtGTCTTGTtcTtATAACTTCATTTTATtATTTGTACGACCATTTGTTACGCGAGTAGCTTAGTGTAATATCCATTGTATCCTACTGTAAaCACTGGCCATATGT

>contig00178

CCCGACCAAATAGGCTCcTtCAtCcATGGAACCTGAAGCTAATGCAAATTtACCAAGTATTACTAGTTAACATATAATTTTTCATTATTTAGTTTTTtATGTAAATAAACTGAAATATATTGTAGTTTACGAAATTTCTAAGTCAACTTCGAATCCTGCATTTAAAATGCTGGTATAATAAGGAAAaGGTAAATGCTTAATTCGTAACTGGGTGATGTGGTTAGCTAATATCTGTTATCTGCTACTTAGTTGTCTGCAAAGCTGATGATGTTTTTTtCTTtCTTGCTGAACAATCCACAAAATCCCATCTTTGGTTCCTCTTTTtCTAATTTAATTAGACCTTCTCGTTTATTCTTATAATTTATGCATAGAAAATCAGCAGTATCACCGTCACAGAATATGGATTGACAAAATGCGAGAACATTGTTCTTTTGTAATAAAATCGCAGTATCATCTCGCTTATAGCGGTCGTtCACAAATACGGATTTTCTAGGAAGTTCTGCTTCTATTtGt

>contig00179

CTACCTTCTCATCTAGGATTTCCACCGGATCAAAGCCAACCTCAAATTTGCCCTCAAGTTTTTTtACCAAAAATATCGCCCAAAAACCTTTATAATGCAAAAGATCTTTTACTCCACTCCCATCAGAATTAGACTTTACGAAGATTTTTtCACGATAATTCTTTTCTTCCAATAATTTTCCTATGGAAATAGATGAACAATAAGTGAAGACACGCTGGAAATCGACCGAGTATATGTACCCGATACAAGCGAATGTACCATTCATATGTATAGAAACAAAGGAGCTCTTCAGTACGTCATCACTTTTTTCCACATCGGTCATCTGCGATTCAGTTGCCGAAATAGGCTTTTtAATTAATACACTTATAACTGCGATGAATAATAAGAAACTTTTGAAAAACATTTTACCGAATTTTTAAAACTCCGTATTAATAt

>contig00180

AAAAGTTAGCGCCATcTT

>contig00181

cGCATTTTctAGATtCCAACAATTATCACTGATGAGGGCctCGATCTGAGCCGAAACG

>contig00182

ACGAAAATGACATTACGGAAACGGAAGAAGATGACGATGAAGAGGAGGAAGAAGTCAAAACTGCTCCATCGATAAAATCCAAGTCATTAATGCTTCCTACAATAAAAACTATGTCGCCTCAGATAACGAAGTCCCATTCTTCTCCGAAAATGAGTATCGGCAGTATAAGAGTTCAGAATTATAAGGTTGTTCAAAGCCCTAGTCAGACACAGCATGTTAGGAAGCAAATATACAATAACAACGTGCACGTCAATGGTAATTCT

>contig00183

ctcTCTTCCGCAGTTTCGTCACCTGAACTTTCAGATTCCTCAGCTGATAAATCACCGTTGCTTGCGTATTTCATTtCTCCCCGTTCCAATAATTCCAAGTGATCAGGATAGATCATTGCCTTTTTTAtGGCAAGAGGAtCCTTCCTGGACCATTTTtGGACTTCCTCATCCATCTtGGCCACCTTATCAGGATTTATCGCCCATAAACATCCTTTTCTCTGATTTCCGTTCCCAGCGGGTTTTtCGATTTTTTCAAAaCATTTGTTTAGGGAAAGGTTGTGCCTTACTGAATTTTtCCAACCGTTCGGCGCTGTCTTGAAGTAAGGGAAGTGTTCGCACATAAAGTTGTAGATTTCTGAAACCGGAAGGGATCCTGTTTGACTATTTTTTAAAGCCATGGCTATTAAGCAGGAATACGAaTAAGCAGGCTTGGGATAAGG

>contig00184

TgAATtCTTCATAGAACTCTGGATAAAAaTTACTGATTTAAtCCGAGAAAAGCTCGTCcaGCAAATTCCGTCACAAATCGGTTTCGAGCACTCTCGGAGCAACTAGTTCTCAGCAAGCCTTGACACCGAAGGAAGCTTCGTAGCCTCCTTTtCCTtGCATTTGCGTTTGAATGgCAGTAAGGtGAGCACTGGCCATGtCCAAGTCACCGCTACCAATATGTCCAGTGTGTTGAAAATtcGTTGgAGCTCCAATCATACTCCGGTCTATCCTGAGCCTCTGCtGTGGCCTCTGTCTTTTaTTtCGAGCTCCAGGTCCTTGTTGAGTCAAACAaCATGTGAACCACTGCACCCAAACTTCTCCAGTTCCTGCCATCTTGGtAATCTTAATCTTTGTCGTCCTGAGTCGAAATTCAGTGACTCTTTGATCGGATCTTGTTGATAATAAGAGGTCCACGTGATATCAATTCCAATAGGAGCGTCTTAAaCTCAAAGaTATtAAAAaCACTTAGATTTtCCGTTGAGCAGAACACACTtATGCAATTTGATTAAATTAGAACTTTtGATTCTAATCGGAACTTTATTGTTCTTGTTTTCCACTCACTAGTTAATATGTAGTTTGGT

>contig00185

aCACCtCGCGGCGCGGGCGCGATACCGCTCAGCTCGAATTTACCGAGTAGGTTGTTGTCcTTTGTCATCGCACGCTCACCTTCATACACCTGAATGGAAACGCCAGGTTGGTTATCGGCGTAAGTGGTGAAGGTCTGCGATTGTTTGCATGGGATACGTGCGTTGCGCTCAACGATGTTGGTCATGACTCCACCAGCAGTCTCGATaCCCAGAGAGAGAGGTGCCACATCGACCAGGAGAACGtCCTGTAGAGCAGAGCTCTTTGAGCCTTCaCCGgTGAGGATGGCTGCTTGt

>contig00186

TCTTAATTATTGATTGAACTtGTCGCGATTATTTGACGATGAGACAATCGATGCCCACTTTAAGCTTCGAGATCATCGTCGATATTCTCATTAGTCGAATATCTTGTGTtCAGAAATTTTTGCTCAGCATAATATTTCTTATCGATTTTGTTCTCAGAGCTTATCGTGTGAAATTTGAAATGCTCGATTGACAATGCGTGTGTTATTTGTCATGAGAGTGTGTGAGAGAGAG

>contig00187

ACTtAAATCTACTAAGAATTTTttGTGgTGTGCATAAaCACTAAGTCaCTTCTCTTGAAGCTTAtAAACCAaTTCATTTtACTCATCACTTTTTTGAAGATTGCTTTG

>contig00188

GATgTAAATTATAAACTATTAGTTGAGCGGTTCGCCTAAATAATTTGGATTTAGCCAGTTTATCAACAACATATTCATTGTTTGCAAAGTAGTGTAATATATTACGCAGAACCATGATTAAATAAAATCATGTAACTTTtGTCGAGAAAGTATTTATACTTTAAACATCTCAAATGCACACATGTTACGATATTTCAGTAGAAGTTCTCTCTTCAATCTCAGTTGCTTACGTAGA

>contig00189

AATGAAAATGGATATTGTGCAAGTGATTGATGAAACGTCGAAGAAAAGTCAATGCAGCGTTGATGACTCAGAAATTGAAATCTTGCCACTCATCTATGAAATAATGAAAAG

>contig00190

TACGAAACGAAGAGGCTAGAGGTTTAGAAGATAAATATACCAGCCTCGCAATAA

>contig00191

AACAATTATTATGTCTCA

>contig00192

AGTCTGTGAAGATGACGTTCTTGTGTGTTGTAGTGCTGCTTTCGCTCTTAGCTTACGATTTCGGTGAAGCT

>contig00193

CtGATTCGCATGCTGAATGTATTAACATCTTTAtCCAGCTGGTCTAGCAAAGCAATACTCTGGATAATCATGTTGTCTACtCGATTGACATTGAACTTAACTTTTGCCCTAGAATAACTGTGACCTAAACCAAGTTGAGCGATTCCAGAGCTTTTTGAAGTGAATCCTTTCACTAAACTATGAAAAtGAAaTCTAATCCCTCTAATGATTTCGGgTATAGCACCCGTGTGATCACATTTtATACCAaGAGCTTCGGTAATACTGGCTCCAAGTTTCGCATCAGCAACTCCGAGtATTACTTTTtCTTTTTTtCCAGCTTTTGGAaCACACGAATCTAAAAaGAGTGCTAGATCTTCTGGAACTATTCCTTCAGATATGCTATTCACGTTCTCTAGTGCAGTCAGCGCAG

>contig00194

AAACTGTTGCAGAAcTtAGAAGGCTGTACtGAACAGAGTGAAAATCTAAAAAACTAGTTTGTTTCGAATTGCATAAAACAGTTACAATGTTTCATATCTTCCTGGCTTCGTTGATCGTTTGGTTTACTTCGGCACAA

>contig00195

GGCGTGAAATTGGATCCATTAAAAGCCTTAGCTTACATAGCCCATCGAGATGAGACTGGAGAAAAATGTATAGGAGGCGTGGCCTTATTAAATCGGCAATACGCTTTAACAGCAGTTAAC

>contig00196

TGTACAAACGTGATGCTATTCAACATAATGTGAAAAACGTGAAGAAAATTGAAAGTGATGACCCTAACAATAACTATCTTCTTGCTATTTT

>contig00197

TTTGAGTTCAACGATGGT

>contig00198

ACATcgAaaGCAGAGATCAGTCTTCAGTCATAGTTTCTCAGCACAACATTCACAGTTTCGCGAAAGCTCTCACTGGAATAAGAACTCTGAGTTCATCTCTGATTGAAAAATTGTTTCTTTTGATTAAGAAACTATTACTCGACAACTGGAAAGGCAGATAAGCTTTGAACAAGCCGAAAAAATGAGTCGTTTTACTTTTTtGATCGTGGCCTTGGCTGGACTTGTCGTTTCCAGTTACTCCGCTGAAATTACAAAGAAGACCGACGAGAATATCGAATGGAAATCAGCCTCGTCCGTTTACAACTTCCACGCAAATAATATCGACGAAAATGACGTGGATCTTGCTAGCTACAAAGGCAATGTTCTTATTGTTGTTAATGTCGCCAGCAATTGTGGATTAACCGACGCTAATTACAAACAATTGCAATCCCTCTATGAAAAATATTCAAGCGAAGGTCTCAGGATTCTTGCCTTCCCGTGCAACCAATTCAATGAACAAGAACCAGGAACTGCAGAAGAAATCAAAGAGTTCGTTAAAAGGt

>contig00199

TGTTTCATTAAaCTAGAAAaTTaTAAaTTTCGTCAGTtCGCAAATTCGTTAAAAAAaTTATTCATAATAATtAACTCtCCTGTGCAAGCTTGTTAAGATATATCAACAAGGTTTCTTCCATCTGaTTGGGTTCAGTTTTAGGAGTAAaTCTGGCAACGACGTTTCCATCTCTGGAGATAACAAATTTGGTAAAGTTCCACTTGATATCCTGGGTAAAGTTTCCAGCCGGGTTAGGTTGAGCTTTCAACCACTTATAGAAGGGATGAGCGTTTTCTCCATTGACTTCAATTTTCTTAAACATGTCAAAAGTAaCTCC

>contig00200

ACTgTTCtGtCTCTGTGTCGTTTTCATTTTCGTTGTCaTTGTCAATCTCATTATCGAGTTCATTATCAAGTTCGTGTTCAAGCAATTCGTTATCGTCTTCGATATCGACTCTCTCTTCTTTGATTTCATGTGTTGGAGAGAAGGAAAaaTGACGTCTACTACGAGGTGATGGACTaCGAATTGCCATATGAGGCAtCACTGAGTTCGGGTTCACCATAATCGAGGCTGCATCTCCTCCACTGAGATCAAGGTTGAAATTACTGTTGGAACTATTGCTATTTCCATTAAGTAGACTCGTCGGACCACCGAACCATCCATTGAGGTCACCTTGAGAGCCGACAGGATCATCGTCCAATTCTAGAGGCGATAGATCTGTGAAGTTAAATCCTAACTCTGTATGTCCGCCGATGACACCCTCAATTTCGCATTTTATATCAACATCCAGCATCTCTTGAAGAGACAAGCTATCTGTTCCTAGATAATAGTCCATCGTGAGCCAAGGGTCCCTCGTCGGTCCCTGATCCAGCCCAGTGCTGTGTCTACTCTCTGTATCCAACATTCACACGACGTTCGTTCGACGAGTTGCAAAATACGTCGTCCTTGCGGCCAAGCACCACTTTCACACATATATTTACAGGGAAATTATGCGATTTTCGGCAGCGACCATGTATAAACGGACACCGACACAAGGCCCCcTTTACGGAACT

>contig00201

AaCCGAATTTGACCTTCTC

>contig00202

ACGAGCTttGGACGCtGTGGAAGTCAAGGACATAAGTATAGAATCGTTAGGAGATGCGGAAGCTTCAGCAGAAGGTtCGaTACTTTCTGCTTGGTTTTTCCAAGAATAcAAAAATCAAGAGAAAAAGAAGGTCTTTCCAAAGATTTCTCTCTACGGAAaCGAAGGAGAGAATGAATGGAAAGTGGGCCTGAAAAAAGCACAGGCTCAAAACTGGGCGAGAAAaCTCTCCGATACACCAGCTAATTTGATGACCCCAACGATATTTGCTGAGGAAGCTTCTAGAGTTCTTGGTAACTTAGGAGTCTCTGTTCAGATTCA

>contig00203

CTTTCAGGCCTGTCTCTAAGTTCTCGACTAACTGGTCTGTCATGTTCTGTTCTGTCGCGGATGTGACGAGGTGTTCCTTTACTTGGTGGATGATTTTCCCGAGATCTGAGAGGCATTCGGT

>contig00204

ACTTGAATTTCTTGCTTAAGGTCTCGAaTTTCATCCATtaGTCTTTGCACTATAaTTTtCAAGCCATCCTTTTCGTCTAATTCAGATTCGAGTAATGCGTTCTTCTCTATTTCGAGGTTGAATTTGGCTTCTATTTCTTCTTCGGTtGCTTTAATAATTCTATGAGCTCTTTCtAAATCGTCATTTTTTtGTtCTAATTCTCGAATATATTTTATTTGCTGCTCTTGGAGAATCTGTAGTTCTtGAGCTCGTGCTTGAAACATAGCACAATCACcAGCTTGTTGaTCCTGTCGGATTCTTAATTGTTCtACTTCAgTGGCAAGCCTCGAATTCCTCTGTTGCAGTTCCCTGATTGTTTTTtCTGCTTGTTCCAAAGAGGTTTCTAATTCCTTTTCTAACATTTGTGAGTTTTCTTGAAATTCCTCCAGTTCGCGTTCTATATCTTCTTTTCTTTGATGCATTTGATGTGCGAGGTCCATCCAGAATTGAATCTCTTCATCCTTACTAGCAAAATCTGGAGTATCAATaTCCATCGTTTCAACAATGTTAGTGCCAATTTCTCAATCAGTCCAAACTCTTCAGTTTGCGAATAAGTTCGCAAACCGAtCACTTGACATTAAAACAACACAaGAGTTCCTTTTTtCACGACaCaCAGT

>contig00205

ACGTCGAATTGCCAAATTTATACAAACATTGTGCATCGTCTATACAATGATCGCCCAAACGTCGAGCATATTTTAGACAAAATGTATTGTCCTGATTTCCAGTGTAGTCGTCTATGCAATCACATTTCTTTGTTCCTTGATCGCATTTCGCATAGATAAAAGGGCAATCTTGATCAGTTTCACATGGTTTGCCTATGAAGGGTTCTGCATCATTAAAACTTCtCGCTTGTGCGTTTGATACAACTTCAGCAATGTTCACGGGACACTCTATCATAACTCCACCTCTAGAGCACCGGCAAGTATTTTCATTGCACGAGGCGTTTGGTCCAAATTGTCGTTGGCATTCAGAATCTTCTTTGCATGGCAGAAGTTGTTTAGAAAaTGAATTTGc

>contig00206

ACAAATAGATATTCTGGATACAGCAGGACAAGAAGACTATGCAGCTATTAGaGATAATtATTtCCGTAGTGGTGAAGGATTCCTTtGTGTCTTTtCTATTACAGAAGATGACAGCTTCCAAGCCACACAAGAATTCAGAGAACAAATTCTCAGAGTGAAAAACGATGAAAaCATTCCCTTTtATTAGTGGGGAACAAAaGTGATCTTCAGGAAAAGaGAAAAGTATCGTTGGCTGAAGCACAGGGCAGATCACAACAaTGGGGTGTTCCATATGTtGAGACGAGCGCAAAGACGAGAGAAAACGTCGATAAGGTGTTTTtCGACTTGATGCGTGAAATAaGGTCGCGCAAGATGGAGGACAAATCGGCGAGTAATGGTCGTGGAAAGGACCGTGCGAAGAGGAAGAAAAaGAaGT

>contig00207

GGAGGCTtCAACAAAGGTAGCCAGGGTCCAGAGTGAGAATCCTAACCAGACGAATACCGACTGTTAATTTAGTTTtAGGTTTAGTTTtAGTAATGATAAATCGTATGTAGCAGAGTTCTAACAATTCTGCGTGCGCATTAAGTGCACGCGCACGCATCAATCATATCCGATT

>contig00208

GGATTTtGGTTACTGTTAGCGCTAGTAAGCAGTTGAACCcACCCAATGACATAGTAaTTAAaTCTGGAGATCAAGCTGTTCAAAATGCTTTGCCACAAaGTATGcAACTAGACGATATCAAAGATCTTCATCCAGATTCCAGGTTCAAAATTGTATATTtGAAATGCAGTATTACACTACTATCGTCTCCAAACTTtGAGTATTGTTACGTTCCtCCAAAAGAGAAACTCGAGTTT

>contig00209

GACTTGTGAATACGTAGTTTTttGAAATACGGTGAGATGTTTTTCAAAAGTTTCTTATTATTCATCGCCgTTATGAGTTCAGTACTAGAAAAATCTATTTCGGTAACTGAATCTCCAAAGAGCGATCTGGAAGGAAAATTAGCCGCAGAGGAGAGCTCCTTtGTTACTGTGTATAACAATGGTTtATTCGCTTGTATCGGGTATATATACTCGCTTGATTCTCTACGTGTCTTCGCTTTtAAATCATGTATTAGTGAAAcaGAATTCATAAATAATAAGGAATATCGTAAaGGCATCGTCGTAAAGTCTACGTCtGGTGAGAGTGG

>contig00210

ACTCCAACTTTTATTGTCATATTTTtGCAAAATGAACTTGGGAGCCAACTTCAGGTTGGCTTCAATCCGACAGAAGTCCTAAATATCGGGGAGAAaTTCGAAATTGAGGGATGTGCTATGATAAGATGGgACAAGGTGGATGATAAATTTAACATGTTCTACCGTACTGACGGACTAGTTTGTGTTGATGTTAATGAATTTATAAACGCACAATTACCTCATAAAaTCATAGATGTATTCTGGTCTGGATCCATTAAGGGCGATTATTCTGCGATTTTATTAAAATACAATAATGTTATCGC

>contig00211

ACTTAAAtAtATTGCAGTTCtCGAAATTGAACAGACAATTTTGAaTCATTCATTTAAaCTGCTGGTCCAATGAGAAGAaGTGAACGTTTAATTCGTAACTGGgTGGTGCAGTTAGCTAATAGTTGTTATCTATTAGTTAGGCGCCGGGAaTCGGGgTGCTGTCATTTATGATTTAAACTtGAGAAAGTTTCCAGCAAATCCTCTCATTGATTTCTTTCTTTCTAaTTTGATTAGATCTCCCcGTTCTTTTTtATAACTATAGCaCCcAAaTTGAAGATTTTtATCTTTATCAACAGAACGTACGTAAGTACAAAAT

>contig00212

GAATGCCATCAGCCGAGATATTAGGTGGGTTATCCAAAATGTTTAGTTCTAAAGGAAACGTAGACAAATGCTTGGTTTCCATCCAGCTAGAAAATAATCATTATTGTTCCGCATGTCGAGTTGAAAAGCTATATGCTTTAGCTCCTAAAGATTGTCAGATACTAACGCCAAACTATAAGGATAGAGTCAGAGTTA

>contig00213

ACGGcTGAAAGTAAatCTGTAGGCATTGAGAAAaTCATAgATATTCAAGGCTGTAAtAAacTCGTTCTCTTGAAGTTCTGGGACATTCTCGAACTAAATGATCCTGCTAAAATAATTTTTtCTGCAAAATCGTTTGGGGAAAATGAACCAATAAAATGCACTGTAATCAATTGGAAGCAGCCGGAAGGTTTTGCCAaGGAGGAGTTGGATAATGTATATCAAAAAGAAaGGAATTATTTTtCACAACAGCGCCACGAAAGACTGTTGCTAATAGTGATAATAATCTTGGTAGTGTTCTATATATCAGTGATGAACTTCTAGGAATGGGCATATTTACAATGAGTTCTGATTTTCATTATGTCCATATTCCAAGTTTtCGTGATAAAaTCCTCGAGGCACGGAaTGCAAaTTAACGTTTTtAATtGTCGGGACAG

>contig00214

AATATACAATCTGCTGCAAATCTTTACGCCACGCATAACTTGCAAGTTTTTCAAGTTTGTTAAAATGTCATCTACCTTAATTGTTTTAAGAATCTTATTAATTGCTTCATTACCAAATGTTGAAA

>contig00215

ACAATTGGTCATGTAGCCCATGGAAAATCAACTATTGTTAAAGCCATTTCTGGAGTGCAGACTGTTCGTTTTAAAAatGAATTAGAACGAAACATCACAATTAAATTAG

>contig00216

TATGCGAATGCGAAGATTTATAAGTGTGACAATgACAAGTGTCCAAGACCAGGATGTTACATCTCGGGTGGATCTaGCAAAGACGATTCTTtCCcTTGTTTACGTCCAGTATGCACTGGCCGTTTTCAATTGATCCGTCATGTTTCATTCGTAGATTGCCCCGGTCACGATATTCTCATGGCAACTATGCTTAaCGGAGCAGCAGTCATGGATGCTGCGCTGCTATTGATCGCTGGGAATGAATCTTGTCCTCAACCACAAACATCGGAGCACTTAGCTGCAATTGAAATAATGAAATTGAAACaCATTGTAATCTTACAAAATAAAATAGACTTAGTAAaGGAGGGTCAAGCAAAAGAGCAGTATGACCAGATACAGAAGTTTGTtCAAGGAACAGTAGCCGAAGCAGCTCCAGTAATTCCCATATCGGCTCAGTTGAAGT

>contig00217

TTCTTCGTATATTCATTCAAGTTACAAATATTAAATCACACAAAATATTCGCTTCAGTGATTAAAAAAAaGAATTGACTTTAAAAACCGTGATTTTTTAAAATGTCGTTCAAGAAATTTTTTtGGCTTTTATTGGGCCTCGTAATCTTTTCATCAGACGTCTGTGCATTTGGGGACAGGAAGAGGAAGAAGAAACAGAAGGAGCAAAAAAAaGTTGAGGTTGAAAGTCCCTTGGTCTATCAAGTGACAGATGAGTGTCCAACGGATTGTGAATTCAAGACCTCTCAGACCCCAGACTGTAACGCCGTTTGTGAGCTTCGTCAATAAAAGAAATTGGCTGAGCATCAAAGTTACTAAAAACAAGTCCACAAATGAAAACAATTGGATCGAAAAATTATTGTACAATATTTCAATTATACCAATGTTCAATTAATACTATTATTACTTAAAATTACATTAATAATT

>contig00218

GGgAaTAATCCAACACACGGTAAACGGCCGAGAATATAAGAAATCTTATCTACGCGTCCCACTTTTCCTTGTTTTCCTTTCTATTGTTTCCATCTTCCCACTTTTTGCAAGTAGCATATAAACAGTTCAAAGTTCCAATGCTTCATTC

>contig00219

TTTTTCCTATATACAGTCAGGTTGCAAATATTAAATCATATAAAAAATTCGTTTTAGTAGTCAAAAGTAAAAATTGAGTTCAAAATCCGTGCTTTTTCAAAATGTTGAACAAGGTGTTTTTCTGGCTTTTATTCGGCCTCGCAATCTTATTATCACTCTCCTATGCAGCACCTACAAAGAAATACCCTGGTAAAGTCGTTCCTAATTTTCCAAATCTGCCCTCTAAGCCAAAACCAAAGTGTAATTGGGAGTGTAGAACAAATTCAAAAACACATCAGAATACATGTAAAAGAATATGTAAATAAAAACCTCGTTCCCAAGACGATGTAAAAAAGAATCCTATTCTCGATGACAATCAAAATTATTTTACCATATTTCCATGCCTTGGACGTCACATACCTAATGAAAGAAATTGGCTGAGCTTTAACTTCACGAAAAAaCTACTTTAATAATTCATGTGACTTTATTGGCTTATGTATTATCTTCCGGGTTCTTAATGAAATAAAATG

>contig00220

ACCTTTTGAACAACAATGAGGAAATTCAACATGTTGAAAAAGTTCGACGTCGTCAGCAAGTTTGATGAAGTGGATAAGTTTGGCAGAAGTCAGAGGTTCATAAGAACACCAAGTCCACAGGCGAGTGAACCTGCATTATTGTGTAACTTTGACGAAGGTGAAGAAATTTTACGGACAAaTTATGAAAGTTTAAaCCAAaTTCCCCATTCTTCTACACGTATTAAAaGC

>contig00221

TACCGAGTCAGCATTGCACATACATATGATAGAAaTTTCGCTACCCTCGTTACACACATTTAGACGTATGCAACCATGCGAGAGCGCTTAAGCGTGAAAAAAaTCCATATTTACGTAAaTCTATTACTTTTTATAAATAACGaCAAAGCGATCGCGAAGTTGAATTGTAATCTTTAATCTCAAGAAATCTGAGTCTGAGTGTTCAGAAGAAAGGAATTTTCTCTGAAtACTCGAATCAATATTGAcTTtCTTGGGAAGCGAGATCGAATGATCGTTATGTATAAAAGAACTCTGAATTAATCTCtGTATAAGTTAAAATACATtCCTGCAAGAGAATCTGGTAAAGATAAACATTCGAGTCCTGTTCACGAAAAGTGTGAAACATACTGTTTGt

>contig00222

GTTTCGAATCCTAAC

>contig00223

CCACACGCTTCCTGTATTGCTTCTTCTAAACTTCCAGTGAAAAATTGCGGGTGACTAaGGCCATATCTCTTTTtCAAATTCCTCAGCAAAGTGTAAGGTGCCGAAAGTTTCGTCATCTACATTGCTTGGCATGAGATGCT

>contig00224

GTCTCCTCAGTAGCGTTGCTTtCCGTTGGTAGCTCAGCAACGCCTTCAaCAATGTTATCCG

>contig00225

ACATTttGgAGACTGGTTTTTAATGGCCGAATTCTAAGAACTAGGGAATAGGATTGTAAAGACAAAATGTCTTTGACAAACTACTCCAAAATGTTGAAGCAAGTTGTTGGATTAAATTGGACTCAAGTAATTCGTGGTCAAACCTATGCAGCATTTCGCAGTAAATATTACTACGTTAACAAAGAAGAACCTATGGGATGGAAAGACATTGGAGAGAGAGCAGTTTTCAAAATGTTCATCACCGAACTTTGGCGAACCATGGCAaTTGCTCTTTCTCATTTCTTTAGAGAACCAGCTACTATTAATTATCCCTTTGAAAAAGGACCTCTAAGTCCCCGATTCAGAGGAGAACACGCGCTTCGAAGATATCCATCTGGAGAAGAAAGATGCATAGCTTGTAAATTGTGTGAAGCAATTTGTCCAGCACAAGCAATTACTATTGAAGCAGAAGAAAGAGCCGACGGTTCAAGGCGGACAACAAGATACGATATTGATATGTCCAAAtGCATCTATTGTGGATTCtGTCAGGAAGCTTGTCCTGTGGACGCAATTGTCGAAGGTCCAAATTTCGAGTTCTCcACTGAAACGCATGAaGAAaTGTTGT

>contig00226

TtaTTctGtGTgACTGAAaTTTTGTTGCGTGGAAGAATTATGACTAGCTATCATGTTTCTGTCCTTTGATTCTAGCCAAATTTTATTGTTATTTCTTGGACAAAACTGTATTTTGCCAGTCGTGTTTCAATGTTCAACGGCTTTCTTAGTTTGAAATTTTCTGATCATTTtAATTTtATTCATTGGGTAATTGATACTTTTGCACAGCCTTTAAAAAAaCGACTGCTACATTTTAAAAAtCTTTTCCTGAATCAATTTGTAAATATAGCACATTAATTTTATGATAATATAAGATAAAAaTGACTCCAATTTAATGAAGTGAAAaTTATTTATCAAAAAatAAGTTTTTTTtCAAAAaGTAACTTCATAACTAATTATTtATAGACAATAATTTTTTATTTtATACTCCTTGATTATAAATTATGGATaGAAAAATGTGAAAATTTTGTTCTATTGATATTTTTttCACATAACAATATTTATTTTATAAATAaCTTATATTTTGATT

>contig00227

ACGAAAGACCGAGATATTGAAAAGACTTCGCCTGGTTCGCTGTATACACCTCAAAGGATTCAAATCTTAAGTCAGGATTTGAAAATCAAGAAGTAGACAGTCAGTGCATCAGTGCTAAGTGCCAAGAAGAAGaTAACTtAAAATTGTCGACGATAAAGCATCGtGGAATGtGCGGACAaTAAATAAAAATACAAAAAAGGCACTGCTCCATATgCACCGCGGGcAAAACATGATTCTCAAAAAAAaGaGCGTGCTAGCACACAGGAAAATtGCGTGgATAGGATTGAATCGtGTAATCGCGACTTAGAGATGTTTACGTATACTCGGTTAAACAGTAAACCTA

>contig00228

GAGAGCACAATAAATaCGTAGTAATACTTT

>contig00229

ATCATTTTGAGAAAGAGACGCAAAAAATGTAGATAACAGTGGAAAGCGAAAAAAaTTAGTTgaaaaGTCTGATGCTGCTGAAGAATGGGATCAT

>contig00230

AATACTACTAAAATGTTAGCATA

>contig00231

ATGTACTCGTTAGCAAACTCGTGATTATTATTGTAAAaTtAATTTTtATGCAATAAaGAAAAaTTAaTTAGCTAGTTGAATAAGAAGCTTGCTTTACGAATCTCTCCAGATTTTCAATTATTTTTtCATAGATTGCTGAAAGGTTTAAAAAaGTAAGAGGAAAATTTGATGCGCGCTTCGATATACCACCTGTGCTTATTCCAAGAAGTTGGTGACCTGAGTATAGAAGGAAACCAACATCATTGATAACATATATGTATTTTGATTCTGGTTTAGTTGTATAAATAAAATTATTATCTTCAATTGCCTCAACATCTTCTTTAATAAATTTGTCTTCGAAACCAATTGCTAATTTGTCTTGCTTTCGAATGTCCGT

>contig00232

ACATTtCTTtCTTTtATTCAAGGAAAaGGGACTGTCATCCAGTTGTATGCTGGTAGGCACTTTAGCCATCGATGCCTTTATCTCTATAATAAGAAAGTCGTCATTCCAGTCACATTGGTGAACGGTTTTAATGCCTGATGTCTTTCCAGAGGTGATTAAATTCGTCGTTAGAATATTATATTTGATGGTTACACCATCTTTTAATTCGGGAAATATTtCTGTaCAACTTTtAGTAGAAAaGAAAAaTATAGTGTGAAATCGGCATGCGTtACAGAGAGGATAACTGTTTAGTGTGACGGCAGCTATGCTTCTATCCACGCGGTTATACTTATGaTATGTTAATGATaTAAAAGAGTCCTTCTGGATTAACCACGTATCCTTTGTTGTATATGATCGTTGAACTGCTACAGCAaGAACAACGGCGAAGAACAAAACGTCTTTtAAAAACATTTTACTGAATTTAAGAAATTTCAAACTGATATC

>contig00233

AtGGGgAGTCGCACCTTGGAAGCCAAATAAATTGGACTGCTGGCAGTTTTAAAGTCTCTCGATAACTATACATGAATAATTTTCTAAAGTAAAGACAAATTCTTAAATCGACAATTTTCTGGTGCGATTGTAAACTTGTTAACATAAAAGTTGAGAAGATGAGTTTGAAAAAaTTAATTTGCTCTCTTGTATTTTTGACGACACTGACAATGAGTAATTCAGAAGACCTAAATGAAATTCTGAATAACGAAGAGTTTGTGCGGAAGCAGATAGACTGTGTCATGGATTCTGGATCTTGTGACCTAATTGGTTTtCAAATTAAAAGCTTGATTCCGGAAATCCTATTGAATAATTGTAGAAATTg

>contig00234

TTTATTTATTCAAATGTCTAGTAATtAATTTTTAatAAAAaTGCTTAATGTCATACACAATAGATAATTTCtACTTTTTTttAtAATCAATAATGCATTCGAATTCCTTTTTGCTCAATAAGCgTCATTACAAAGAATCGAACTGCATTCTCACACTCGATGCATACCAATGTTATTAAATAACGTCAATAAAATTTACTTTCAAAAGTTTTACCACTAGTAATTAAATTTTTGACCTATAACTTCAATTTTTTTGGACATATACAATTTTTAAAGtAATtGATATAAAaTATCCTTTTCTGTCGCTAAACAATAAATTTTCACAAGCCCATAAACGCGATAAAATTtGATTAGACTAATGTCACAATAACTAAAAGTGAAAAaTGTCACATGTCCCAATCCAATCAATCCTCCAACATATAATCCGTTTtAGTTTCATCCATACTTCGCGGAGAATAGCGTCGAACAATCTCTTCATAATCTTTTGGATAATTTCTTTTGaTAAAAaTTACAATTTGTCTGGACATACTTCGTTgAAACGGGGT

>contig00235

ACCTTTTGCGGGCTAATTCCAATTAGAGGAGTTCCAAGGTGAACTCCGGTTGCTCGTAAGATGCGCCCTGTCGTCCATGGAGTATTTTCTATGGTGGGTCGTATAGCAGGCGTTACGTCGACCATAGTCAAAATATCTGGCGTTTGACGATAATTTACAAATATTTTTTGCTGCTGTGGTAATGCTtCTTCTTTATGCAATGCAGACAAATATtGCTTAgaTGTtAaCGGGTTTTTtACTGAAtACGACAATTttAagAatCcAataTtgTtcatAaaGgtatAactaCcGTCATAATACCTATGAGGTATAAAGGTTCCTGCAGTAGAAACTTCTTCTAAAaGATTTGGACGATCTTtATtCCCATATTTGATCTGAAAAACTCTGTTGTCATCGTGACGGAAAaCAGGATCATAAACAGTTAGGAGGCACGATGTGCTTATGAGAATTCCGTTAGCCAAATGATTACTCTCATAGTATATATAAACTATAATATCGTGTAAGACAGTATA

>contig00236

CGCATTTCCAGCTGATACAATTATACTGACGATAAATAGTAGAGGACTTTTGAGAAACATTTCAACGGATTAAAAaTTTTCCAACTGaTAaGTTTCC

>contig00237

GACTATTTATAAaTgTGAATtGCATCTGTGGTAATTGTTCTACTGgaTCGAAAGCAAGCAAATATTGAtcAAATTcTAACGTGTCATCTACTGGACGCGACAaTTTTAAGAAtCCAATATTATTGCGATAAaGATCATCATTATCAATTtCTTTATGAGGTTTAAAGTTtCCTGCCGTAGAAACTGTTTtCAACTTATCTGGTCTACATTTtCTTCCATATTTGACCTGAAAAACTCTACTGTCACCATCTTCAAAAACAGGACGAAAAaCAGTAACGAGGCTCGATTTGCTTATAAGAAGtCCGTTAGCAATATGCTTATTCTCATAaTATATATGAACTATAACATCGTCTAAGATATGAAT

>contig00238

ACTATCAGTTGTGCCACCaCCTTGTATATATGTTTGATGCGTAGTTAGTTGATCAGCTTGTATGCTTTTATAAAGTTCtCGTATTTTTAGTTGAACTGGATGCGATCTTAGTCTCAAAAGTTCCAAGGCACGTGTTtgTAATTCGGTTTtGCGGCCTGATTTGTTtCGCCCAGCGAATCCCAaTAGCATCTGTAGTTCAGATACTCGAAAaCTTAACACCATATTCT

>contig00239

CTAATTCTTCTGTTTCCGCCATCTTCTTTGACTTTTTTtGTGGAGCCGTTTTGCTGGTGTATTTGGACTTAGTCCACAAAATATTCCTTGATGTTTTTCGGACTTTTCAAACCCGCCAAATATCGAGCTTGGGCTGAGTAACGTTGACTATAATTAACTCTTCCCTTTTTAAATAGCAAGCTTTCGGTGTTACAAATTTCCCAGTGATAGTAACTGTTTTTTtCAGACTGTCATCGAAAAATATTTTTGTTTCAATTGAAGCGCGTTAAAGTAAGTTTATTTACTTAATTGTCCGTTCAAAAAGAGCGGAATTTCCAAGATGGCCGCTTGCGTTGAATTTGACTAGATTTCTCACTCACGCTGCACGTTACTCCAACATCCGATGTTGCTtcATCACTAGCCGGTAGAGGAAT

>contig00240

CGAGACCGATAGCGAACAAGTACCGTGAGGGAAAGTTGAAAAGAACTTTGAAGAGAGAGTTCAAGAGTACGTGAAACCGTTCAGGGGTAAACCTG

>contig00241

TGgggACGACCTCAGATCAGGCGAGACTACCCGCTGAATTTAAGCATATTACTAAGCGGAGGAAAAGAAACTAACAAGGATTTCCTTAGTAGCGGCGAGCGAACAGGAAAAaGCCCAGCACTGAATCCCGCGGTTTTCGCTGCAGGGAAATGTAGTGTTCGGGAGGATCCATACATCCCGTGACGTTGAACTGCGTCCAAGTCCATCTTGAATGGGGCCACTTACCCGTAGAGGGTGCCAGGCCCGTAGTGACCGGGACGCGTCTCGGGAGGATCTCTCCTTAGAGTCGGGTTGCTTGAGAGTGCAGCTCTAAGTGGGTGGTAAACTCCATCTAAGGCTAAATATAACCA

>contig00242

ACGAGGGCGGATTTTtCAAGGCTCATTTACAATTTCCAAAAGAATATCCCCTTAGGCCTCCAAGAATGAAATTTATAACAGAAATATGGCATCCAAACATCGAAAAAAaTGGTGACGTGTGCATATCAATCTTGCATGAACCAGGAGATGATAAATGGGGTTACGAAAAGGCTTCGGAACGGTGGCTTCCAGTCCATACAGTTGAAACAATTCTTATAAGCGTCATTAGCATGCTCGCCGATCCAAATGATGAAAGTCCTGCAAATGTAGATGCCGCCAAAGAGTGGAGAGAAAGTTATACGGAATTCAAACGAAAGGTGGCAAGGTGTGTGAGAAAAAGCCAAGAGGAGTGTTTGTAGGGGAATAATTAATATTCAAACGGAAAGACTTATTTAATTCT

>contig00243

ACTtAGtGGCACtGTTTTCAGTTAGCATTAAGCTGAaCTTCTTTCTCTTTCGCACAGTGCTTACGGCTTCCcTATACCCATCGAAGTCGCC

>contig00244

AGTAACTTTCCACATATTCGCCATCATCATCGAACATTAAAGCGCTTGGTTGGTAACAGAACGAGTTTATTATAATTTTATAAAAGTTGAAGATACGCTAGAACATATTGTGGATTTCGAAAGAATTTTGACAATTTTTAAAACATGTCGGGTGTTGAGGACGGAGAACTGAAGGGTGGACATCCTCCTGCAGTGAAAGCTGGTGGTATGAGAATAACTCAGCATAAGCTGCCTAAGGAGGAACGCGAGGTCAAACCTGTGAAGGAAGTCGAGGAATATAAAGCTTCCTCTAGCCCTCCGAAGGCGCTGATAATCAGTGGATTTCAAACAAAGGGTCACTCGGACTTTCCTCCGGAAGCTGTGCAGAAGTTTCACGAAAAGCCGACTCCATGTCTTGATCCTCGCCGTAACACACATAATTCTCGGCCCATTGTAATTCAGCAGCCTAGAAAGTAAATCTTTCATTTTCTTCGAACAGGATGGAATGAAAGGAGG

>contig00245

TGATGCtAAGCGTGAGAAGTCatAAAAATATTTtGTAaTGGATGAGtGTAGATTACAtAtCTACTTAAATTTATTCTctGATCTACATATATGTGATAGATCCATTTAATTACTtACTGAAAATTTTGTTAtATACTATCTTTCAATTTCTTTGTTTTTtGCAGGCTTCTACGAGATAtATTTTtCGAAaGAAAAATTCGAAAAACAATATTGCTCAGTTTGTATTCTTATCATCTTATTGTTATTTCTAGTATTtACTACAaTTCATTaGTTTtAAAAAAaGTaTAcA

>contig00246

ACTTGAAAACAGGTtCAACAATTCCCTTTtGAtAAtCAAtAAGtATtCCTtCAAGCTTGTCATCTTtAAACATAAGACTGCCATTGTcTTCCTTTGATAGcACGTAATTTTCATCCTTTAATGCCACGTAAACAATTTTAGAGGATTTTTCCAaTCTCTCAACGACCAATTTtATATATCTATTTGAACCAGCATATTTTTCTATTCCGTTATATAATACCATTGTATGTTCAGCAGAGTATATCTCCTTTGCATATCGTGGAAAGTCTTCAAGTTTTTTAAAAaTTCCACTGGTTTCTCC

>contig00247

TACATGCTGGGTATTACACGTGAAATAGAAGATTGTCGACGCTTTTTCaTTACATCACATCCATATGAACAGTCTATTGAGGAAAGCAACGCTATTGAGGGATTTCAAAGCATTAAAGATTTTTTAGACGATACTAAAATAATTAAAACGGAGGACATGATAAAAGGCAATACCAACCCTACTGGTCTGGCAGTTGCAAAGTT

>contig00248

ACTTGAAAACAGGTTCAaCAATTCCCTTTTGATAATCAaTAAGTaTTCCTTCgAaGCTTGTCATCTTTAAACATAaGACTGCCATTGTCTTCCTTTGaTAGCaCgTAATTTTtATCCTTtAATGCCACGTGAACAATTGCAGAGGATTCTTCCAATCTCTCAACGACCAATTTTATATACCTATTTGAACTAGTATATTCTTCTATATGGTTAGATAATACCATTGTATGTTCACCAGAGTATATCTTCTTtGCATATTGTGGAAATTCTTCAAGTTTTTtAAAAATTTCACTGGTTTTtCC

>contig00249

ACCAGATCTAGCAGgCTCAGTAAAGACTAAAGCAATgAATTACATAAAGAaTAtAGGTTATATTTCAAaGAATAtCATATAAGAGTTCAAAaTGGGCTATtACTTGGAGAACCTGACTGCAATGGTCCTATACATAGTTAT

>contig00250

CCTTCAtAGTCctGACAAGGACCAAAACCctGGTCGAAACGTCAACGATTTATGAATGAACATCTAcACAAtACCGGAAAGGCCACctACATTcTtCTTTAAACTGAAATACCGGTAATACCAAATTTGAAAATTAATATTTCATATTAATTTTTATACTTGAAATAAATTGATTCTATCATTTTTCACTCTCTGTATCACTTTGATAAGGAGTCGGTGCAGTATATTCTGCTATTTTTtCTTCCATTTTATCGATAATAACTGCCTTGTAAGTTtGAAAATTTTCAAAAATAATTTGTTGACCATTATTATAACTGCCCATTGCAAGAAGTTCAAATTTATAGAATAGAACGCTTCCAGCATAGCC

>contig00251

GAAGTTGTGGATTCTTAAAaTTGTCTAAAATGAATCTTAAATCGATTCAGTTACTCTTTTTtGTTATTGCAACAGCATTTATAGCCGGGGTGAATGGAGAGTTAATAAAGGTCTTGAGCGAAGGTTGCTATCGAGCGCACTGTTCAACCCAAGTATTCTTtCTCCAAGGTGCTTGTACGACTTCATGTCAATTAAaCAATATGTATGATAGTAAAaCTTGCGGACTGAATTCCAGTTGCAAAAAaGACCCTGATAAaTATTATGTGTGGATTCAGAAGCCCGGGTCACGTGAATAAGTGAAAATAAATAAGAAAAGTCACAAAAATATTtCAATAAATTtGTGgCATTtAATtAATTTTtCCAAAAcGTATGTATTtAAAAaTTCTAAAATCAATTAAaGATTaTTtGTCTCCTGCGACATATTtGTAATT

>contig00252

AAGTTGTGGATTCTTAAAaTTGTCTAAAATGCATCTTAAATTGGTTCAATTACTCTTTTTtGCTATTGCAATAACATTTACAGCCGGGGTAAAAGCAGGTGATAAAAAGGTTCTTCAGCAAGGCTGCTATTTACAGCACTGCTCAAAGCAATTACGTGAAAACCGAGATATTTGTGCGAGGACATGTCAAATAAGCAAAAAGTATGATGGTGAAACTTGCACACTGAATGACAGTTGTATAAAACACCCTGAAAACAATTATGTATGGGTTGACAAGTATGGTAACCCTGTATATTCCTGAATAAGTGAAAATAAATAAGAAAAGTCACAAAAATATTTCAATAAATTTGTGACATTCAATTAATTTTTCCAAAATGTATGTATTTAAAAATTCTAAAATCAATTGAAGATTATTTGTCTCCTGCGACATA

>contig00253

ATCATGGATGAAGAGTGGGATGCAGaTAGTTTTGAGGCCCATAAAGTTGACCTCGCTaTTAGGTCAAATAAaTGGGAGGGTGAGGATGAAGAAGAAGAGGTTAAGGATAgTTGGGAAGACGTTGAAGAAGAAAaGAAAGATGTGGAAAAACCAGCTGAAGTTCCAAAGTCGAAGAGTAAGCCTAAGAAGACGTTGGCCgAAAAAATCGAAGAACGTGAGAGAAGGGCCCGCGAAGAAACCGAGAGAAAAATGAAGGAGAAGGAAGAGATCATGACTCCCGAGGAGAAAaGAGCAGAAaTGTTGAGGCGGCAAAGATTGCAGGAAGAAGCTGACTTGCGATTAGCAATGGAAACTTTTGG

>contig00254

AAACAGAACAAGAGATATATAAGAAGATGGAACTCTGAGGGAACAGTCACTGATATCGACGTGGAAAATAACTATCATCGTTTCTTCAAACCAAGCAACTATATACGCACTTTTGACGGCAG

>contig00255

TTGCAGTTTTCCATACAGCAGATTTAAGATTTTGCCGAGAATGAAGAATATAATCCTTCTACTTCTTTTTGCGATCATAGCTACCGCTAATTTTGTGACAGGTGTAAATTTCGAACGAGACCGTGATGATCCTCTCTCACCAGAGCATGAGTATCGTTTCCATAATTATAACAAATATAGGCACAGACCTGCACCACATCACGATGATTCAGATGACGATAGACCTCATTGAATATTGAATTGATTGAATATTTTGGGAGAAAAAAaGTAGAATGAGAGGAAATCAACAAAATGATTTAGATGAAAATATTCATtGTtAAAGTTATTATATGTGTGTtGTGA

>contig00256

TCACAGTTTTCCACAACAGATTTAAAAAATTGTTGAGAATGAAGTATTTGGTCCTTTTACTTCTTTTTGCGATCACAGCTTTCGCTAATTTTGTAGCAGGCGTAAATGTCCCATCAGAGCAAGAGCCTGGTCCCGATGACTATGTAGATCATTGGCACGAACCTCCGCGTCACCACAATTCAAATGTCTATCGAAATCATAATTAAATAATTCGGGATGAAAAAaGTGGAACGAGCGGAAAACAACAAAATTATTTAGATAAAAGTGTTTGTTATTGAAGTTATTATATGTGTTAGCTTAACATCTCTGACAAATCGTTGATGTAATTCTATTGAAATAAaCTAaTTCT

>contig00257

CACCAACGCTAATACTATATATACAT

>contig00258

GACAAAGATGACACAGTCTTAAGCACAGTGAGTCGGTTAAATAAAATGTCAAGAAATAGTCCTCCACATATTTTCTATTGTT

>contig00259

AGATAGGCATGTAAGAGATATGGATCGAGAAAATAATTGGAGAGCCGTAGAAGAGCATTTTAGAATACTGAACTCTAGTCTAAGACAGGAACAGCCAATCAGCGATTCTAATAACGAAAGTTCGGACACTGAAGAAGCTGATACATCCTCAGAAGTAGAAATTATAGATTACACAGGGTCTGATATTGCAGATCACGAAATGTCTGCTGATGTGACTGACGGAGCCTCTTCGCCAAATCAAaCTACAGCTGGAATCCCAGCCTTAAGTTCAGATGAaGACAAAaGGTATTGTTGGGTCTGTTtCGCTACGGACGAAGACGATGCAACAGCCTC

>contig00260

ACGAGATACAACGTGTGTTCTCGTCCTTGGCAAGATATCAACGTCGATTCCTTCGTCAATTATTTtATGCAGTTtGCCGGAAACTTCTTCaGAAATtcTCTTCAAATCAAACAGAACATCTAGGGAATATCTACACCACAAGCAAATCCAAACTTCCGTTTCATCACTTAAATCGTCTTCAAAGAGATAATAAATCTTGTCGAAAGCGTCGTTATATTTATTAGTCTTtCtcAGTTTAAAAaGATAACCCTCCTTACCAGGCTTTtGGCAAAAaTTACAGTTGGATTTCTTAACTTTCTTAAAaCTTGTTTGATCTACCTTCTTTTtATAGTTTtGGACAAAATCACAGCATTTATCAATCTCATAGGCGCATTTATGACAGAGCTTGGTCGATAGCGCATCGTCCTTAGAAGCCTT

>contig00261

GACGACTGTTCAGGAGACTACAAGAAACTATTGCTTGCTCTTCTCGGcTGAATTtATTTAATtATTCCATtATTTCATATTAATTTGTATAGTAAATTGTCGGATAaCAATTATA

>contig00262

ACCTTCAATGCTATTTTGATTACAAAAAGTTTCCCTCAATTGCGCAGAATTTTCAAAGAATACGAGCGTCTTTCTGGAAACAGCATTGAAGATGCTGTGAAGAGAGAATTTTCTGGCTCTTTGGAAGATGGTTATCTCGCAGTTGTAAAATGCGCTCGTGACAAGACTGCATATTTCGCCGAAAGATTGCACAAGGCAATGAAAGGCATGGGAACTGAAGACAAAACTTTAATTCGCATAATCGTGGCTCGATCAGAAATTGATTTGGGGGATATTAAAGATGCATACAACAAGATGTATGGACAGTCACTCGCTGCCGATATCGAT

>contig00263

AGCGATTGCAGTGACGATTTGAAAAGGCTCCTGATTGCACTTTTGAaTTAaTTTAAaTTCGTGACACATCGTTCTCTCTTACGTGATTGTATTGGAGGAAaGAAAAaGCTTCTGATTGCTATGCTGAATTG

>contig00264

ACTCGAAATCTTGCAAATAATCTGCTAGATAACCTCGTTGATTAGGTAAATTCGTTTTCACCGTCGAGATGGCACATTCTGAAAAATATTAGCTAAGCCTACATTACTTTTAATAAAACTGTTTATAAATAAAACAGACAATTTTACACTGGCTAATATTTTTCTTCTTGTGAATTTTCGCTACGAAAGTCGCCTCTGCACGCTAAAGTCACATAAGGGCAATCCCT

>contig00265

CAGGAATTTGTTAAAACAAGAGGAaGATACACACAATATTTTCAtCAAGTTTTTTTAAAGaTCACCctGAAAGCTTTTTTtAGCGATCATGCAAAGGCAAAcTTCAAGCGATAAtGATCTGAGATGAAAGAATGAAAATGTGATGTTTtGATTTTAATtAACGAGATAAAAATTTTATTtCACTCGATTGATATTACGATCAGGCGCGaGAAAAAAaTGAAaCCGGAATATACTTTCCTACGTAAGTTGTTAATAAAATCTTTGCATAGAGCTACGACTAAGTTTTACGTGTATATTCAACTTATTGATTTCTCGGATACTTTAATCCAGGTGCTCATAGGAAATTCATTTtGTTGACAATTCGCGATATCCCAAGCCGTTGTAAAGTCGCGTGAGAAGCcAAAACATATGAaGGCATTATTTGTGTaGGT

>contig00266

ACAAGGTGCTAAAGAATGTCCGGACAGAaCCATGTTTTGTATTGGAAGTTGTTCTCAAGGTTGCAATGACGAACGTTGTAAGCCAATCGTAAAGGACTGCGTTCGTATTTGTATAATTTGTCCTGATGGAAAAACACATTGCTTTGACCTAGGGAGAGCACTTAGAGCTGCTATTGGATGTCTAGATGgCGGAGAGTGGCGCAAGCCAAAGGAAAaTAAACAAACTTGTCTAATGCGAGCCAACAGTTGCAACCGTAATGATTGGGGCCCTTAGCAACATTAATCAGTAACAACTTAGCAGACATTCTCTTTAAATTTGAATCAGTAAAAATTTTtgaTgaTTTCAATTaGTAACTGAATTCTTACTTTTAAATCt

>contig00267

aCGGCCTCGCCATGGCCCTTtCGTCTTTGGGTCCCATGCCACCACCATCCCTAAGTTATCCTTCATCACTGGCAGGTGGAAGACCCTATGTCTGTAATtGGATCGCTGGGGAGTCTTATTGTGGTAAACGATTTAATaCTTCGGAAGAGCTTTTACAACATCTTCGAAGTCATACCAGTTTGGCAAGTAGCGAGCATGCAGCATCCGCTGCTCTCCTTGGTTCTCATCCACATCCATTGTTGTCACCCTCGGCTGCTCTTCATCGTGCAGCTGGTGGAACCTATCCTGCACCTTCTCTAAGTCCGCTCAGTGCAAGaTATCaTCCGTATGGAAAACCACCAACTGGACCACTTCCTGCCTCCCTTGCTGCATCACCCTACAGCGCCTTTAATGCTCTGGGACCTTATTATTCGCCGTATGCAATTTATGGTCAGCGAATCGGCGCGGCTGTTCATCCTTAAGATGATAATGCTACAAGTTGGGTCTCCGTCGGATAG

>contig00268

CCACAAAGGCAAATCCATTCTTCATAGCAACCTCCTTGATTCTACCATATTTCCGGAAGAACTTCTCAAGGTCGCGCTCTCGCACCCGGTATGTTAATCCACCAACGAATACTCGGGTGCTCATTCTGAAAATGTGTATAAAC

>contig00269

ACGTAACTTCACCAGCTTGTCTCATGTAATCCTTTAAaTCCTGCCAGCTGACACGACTCGAAAGATTCTCTACGGTAAGACGATACTCGGTCCGGGTTGGCGGTCCATATCTGGGCAATGATTGCTtGTAGCTAGATGCAGTCCTCGTGTTtCTGTTCACACTATCTCTGTCGTGCCGCATATCGTCTCGGTTGTCTCTCCAGGAGCGTGAGCGTCCGTAGCCACGGTCGCCTCGCCTCCCTGAAACACCCCGTGCTATCTCCACCGCCACTCTCTCCCCTAGAAGTTCCTTtCCGTTTAGCTCGTAAACTGCATCATCTGCGTCCCTGTAGTCATCAAACT

>contig00270

ACAGGTTTATAAGGTTTTtCGTATTTTTTCACAAATTCTTGCGATGACATTTCTGATTCTTCGATTCTGTCTACATTATCTTCTAACTTCCAAAACGAATCGAAAGTTTTAAAGTAAGCATGTTGCATCCAAGCAGCTTTATCAGCAAGTTCAGGTCTTGCTTTTCTTTTCACCT

>contig00271

CTTTGATCCTCTTTTTtGCTCGGTGGTCTAACTTTAACTCGTCCGTCATGTCTCTGATTGTAAAAAaTATTAACTTAAAATAATTTCAACGTATTTACAAGATTAAATAGGAAGCACTTCTAACACCTAACGTCACGTTGCTATCTCACAGG

>contig00272

TTCACACTCCGCAAAACTGAGTGATAAACAATATATTGATATGCAGTTTGCGAAAATTGACTGTCTTGATAACTGTTAAAAATCCAATTT

>contig00273

ACTTGGCtGTtCTtGtGACGGgCCCTtGGCTtGGgAGGCCTGCTCCTTTTGAGAATTTGTGCTTGAAACGCAGGAAGCATCTTTCAAAGCTGGTTGACCAGTATATTGAATTGGAATGACTCGTtCATTCTCTTTATCTTCAAGAGAATTCTTCCGAGGTGCAaGAACTGTTAAAACACCATCGGAAGAGATGCTAGATGAAACCTGATCCATATCGCAATTTTCAGGAATCTGATACTTTCTGACGAATTCCCTAGAAATCCAACCATGTTCGTCCTGTTtCTCCTCATGTTTTCCTTCGATTACGATCTTCTTGTCGACCACTTTAACAGtAATTtCTTCAGGGCGAAACTGTTGGACGTCGAGAGTGACTTGGAAGTTATCTGCTTCACTAGATCTTtGTGCGATGGCAAGGCTCCTATCATAAGGATGATGGCGGTAATGCGCCAGGCGACGAATATGGGGTCTAGTTCCAATGCCAGAGTATGGTGAAATTGGTCGCTCCAGGTCAGTAAGATTGATCCCATGACCAAAGTGTTGGTCAAAAAGCCGGTGCGGACGATCCAAACCTTCCCACCAGTTGGAAAAAAGTGTCGGAAATAGGGACATTCTCGTCTAACCTCACTTATC

>contig00274

AATCTATTTCGGCAACTGGATCTCAGATGACCGATGTGAAAGAAAAGAAGCCGCACAGAAATGCTCCTTTGTTACTGTGGATGTAGAAGGTTCATTCGCTTGCCTCGGGTATATATACTCGGATGATCCTAAACGTGTCTTCACTTTTAGATCATGTATGTCTGTAAAACGGTCATTAAAAAaTAAGGAATATCGTAAAAGCATCCTCGTAAAGGCTACGTCTGATCAGAGTGGAGTAAAAAaTGTTTTGGGTACTACACATTTTCTTGTCCTATTTTTGAAAAGGAAGCTTGATGGCCAATTTGAAGTTCAGTTTAATCCGAGGGAAGACTTAAATAGCGACGAGAATGTTCAACTCGAAGAATGTGATATGATAAGATGGGAAAAGGaGGAAAACAAAATTACCATGT

>contig00275

ACTTccATtATTCGTTTGCCAAGCCGTCTTGACCAGGTCTGTGGCGTCTATACTAGGATTTAGCATAGATGTGGAGTCGGTCACGAtGAGAAaGGAATCGATTCTtGAAGGATCAGCGCtACGCGTTGGaTCATCTTCTGAACTAGAGCCATCaTCACTACCTTGAGAATCAGTTCGATCTCTTtCCTGGGACgTTGAATTACCCACCGTATCGAGAATGCTTAAGGTGAAGCTATCAGCTTCTTGTGCATCATAAAaCACGTTATCCTCTTCGTCTTCCGATGGTGAGGTGTTCACTAAAGTAT

>contig00276

ACGTCTGTGTTTATGATTGAATTTTCCACTGTGCGTTGATTTTTTTTCtCCAACGTGACCATTTGTAACTTCTTCCGTGGGAaCCTTTGGAATGTCGTTCTCTGGCACGACGGGTCCTTCGATATTATTCAAATCGCCGTTGCCTAAGTCTCCTGCCTTTGCGTTtCCTTGGAGTTCGGTTt

>contig00277

TTTGGCGCGCTAACACTCACACGCAAAATGCGAGCAGCGTGAGAATGTCGCCGTGGTCAGAAGCCGTTGTTAACGTTTtGGGGAATGAGCGAAAGATTCTGTTTCTAGAAGGTCGAGAGTAGCAGCGCCCGATTAATTCACACATTGAacACCCTATCTGCGCATCGCTTTGTCGTCTTCAAGGCGCGTGCTCAAGCGGAAAAGAaCCTCCGATGAGCTGAtCTTCAAAATCTCAAAAGCCCTCGAAA

>contig00278

GtGATATTAGTTGTGATTtGAGATCAATTATTCAATACTCCACGATAGTTTATtGaTAAAACTTAATATATTTATTTTATGAGACTGAAATATACTAGAGTTGATCGGGGAGAAGTAATAGCAAAACTCGATGTATCTAATCCATGTTTTATAGAGGAAGGAGGAGATTCGAGACGAATTGGAATAAAaCTATTTTTACATTGAAACAGAGAAGTAATTCTTTTTATTTGATACTCGTGCAATGTCTATTAATGTTATCCcTACTTTTCAG

>contig00279

ACTtCACATCTCCCGAACCAtCATCGGCGGCTtCAAGAGCAGCAAACTGCGTGAATATGTGGCTGAGAGGTTTCCTGCAAACGTTGGCTAGGaCGTTAAGTCGACCTCTGTGTGGCATTCCCATGACTATGGACTCAACACCAAGTTCTGTTGATTTGTCAATAACTTGCTTCATTGCCGGGATCAAAATTTCGCAACCTTCCAATCCAaaTCTCTTTtCTGAAGTCCATTTTTtAGCTAAGAAGGCTTCAAACCcAGTGGCTCGAGTCAaTCTCGCCAAAaTAAGTCGTTTCTCATCATTAGTAATTTCCATAATTCCTGGAGTTTCCATTTTTtGTCGAATCCAATTACATTGCTCAAGGGAGTTGATAAACATAAATTCGACTCCAATATGTCCGCAATATGTTGCCTCTAATCTTTTTAAGATTTCACGTAAAGGAAGAGATTTTTCTTtACCACCGATGAAGGTGGTGGACGGCAATTTGAATACTCTGTCCATGTCTGCC

>contig00280

ACGTCtAAAAAaCAGTGtGaCTGTAAaGAAAaTTATCACGAaGCTCTGACTCAATGCTGGAAGGACGTTGGATACGAAGAACCTTGTGATGATaGACGAGAATGTtGGCACCTGCATGGGCATTGTAATGAAACTaGGAAGGTTTGCGTTTGTGAGGGAAACCGAATTATAAATTATAGAAGAACATTGTGCCTCGATGTCGTTGGACATAAAGATGTGTGCTTTGAATCTGTTCAATGTAAACCGAATTTAGGGGAAGCTGAATGTGTCTCAGGATATTGTTCTTGCAGTAGAGGACATCATTACGTTCATATGTTGAGGAAATGCATCCCCGACAA

>contig00281

ACTtCATGCATACATGTGATTGATTtAATTAAATATTAACGTTACATTATTCGtGTGATCAGTTCCAATATGAAaCACTTTCTTAAACTTAATATAtAAAAtCAACATATTAAATAAAAaGTAATTAAAAAaTAaCAAAATTGACAAAATACAAACAAAACGCGCAACGTGTAAAACACTAACTGAGTTAAAAATAGTTCGAGTAAACTTAATTATACAATACCGGGTAATAATTGGTAATTTTTTGGCGGAAAATAATTTTTCTTCACCGCCTCCCGTTATAATAAAAaCGGAAGAAGTGTCACAAGAAATAGAATTGATGAGTATAATCCAACAGCTGAAGACGCGTTC

>contig00282

AGCTTCAGCGACAGAGGAAAGAGACTGAAACTTCAACAGTTTATTGCGAAAAAGGCTGACGCTCTATATGACAAATCAAATTTGGAAACTACTGCTGAGCCAATTACACAACAACTTGGTGATGAAGAGTTTTATGCGACTATGCCAGGACTTGATTCGTTCGTCACAATGGAAAAATCTCAGCGCATTAGAAACTTTTTAGAAAGTTTAGTCGTGGgCGATGTGATTTATGCCCTAGTGATGGGAAAAaGTGCTGCTGGACTCCTTCTAAAGATTCTTtGCAACTGTAGTGATTATCCAAGAGTGGTTTCTGATCTTGGTGTTAAGGCCTTGATATTGAACACGGCCACAGTGCCGGCGGTGGACAAGAAGGGTGTGACTCGAGGGT

>contig00283

ACCGATCCGCCCACAGTTATTCGTAGATCGTGACTCGTGTAGAGTTCACGGAATGTAGTGAGATAACTGTTTCATTCAATTCAACTTGTTCTTAATTTCTACGTTTTAATAACATCGGAACGTAATTATATTAACAAGTATtACAGGTTAGGTTGATCAGCCGATATGGAGTCTATGGATGCGCGTCTCGTAGCGCAAGCCTTGAACTACCATGGTCAACAGTTGCAGAAAGTTTGGGAGAGCGAGCGCAACGAAAATGAACTTGCAATGCTTAACCTCAAAGAACCCAGTTTCGAAATTTATCAGCAGAGACAAAAAACGCTC

>contig00284

AGTTCAAAAATGAAGGACGTTATAGACGTTTTGGCCATGCAGAGAGATAAAAGAAACACCTTACAAAAGAGTATTACCATCGATTACAGCGGTGCCAGTGATAGCTTTACAGTTTCCAGATTCATTTTTGAGTGTGGATACAAATTAGAAGCGCATCCTTTGACGATTGCAACTGCTGCAATTTTATACCATAGGTTTGTCAGAGAAGCTACTGTTAATGGATATGATAaTtACCTGATTGCATCGACGTGCCTATATCTTGCAGGCAAaGTAAAAGACGATGCGTTAAAAATACGTGATATAATGAACGtGGCATACAATACTCTGCATAGAGGATCTCAACCCTTAGAACTTGGAGATCAATATTGGAGTATGCGAGACGCTA

>contig00285

ATACATTTTTGATGGCGTGTATTTTTTTCACCGGTGTTTGTTAATGAAAATAGTTGTTTGATTGTGCTTTAAAAATAATATTTTTGGGTATTTTTGTAATGTATTAAGTGTATTTAGATATTCTTTATCAAAAATTGAAATGAACCCGACTGAACCCAATAGTGCAGAAGCTCTC

>contig00286

AAAATGTTTTTGAAAAATTTTGTGTtAaTTTTTGCTAtcGTTGCTGTAGAAGCTATGAATcAAacGGAAAaGACATCAAGATCGATTGCAATTTTCCTCAGTTTCAAAAAAAAaTCTATCAATAtgTAGTTCTATTACAAGACAATAAAAATAAATTTAAGGGCACAGGATACATAGTGACTGCCAATACAC

>contig00287

cttGGGGATCATTCGAAAATATACAAACGTCGAAATGAAGTTGTCAATTATAAAACCCTTCTACTTCCTCTTTATGGCTTCCCTAGTTAGGGAAATTACTTGTGGTGGCAAAACTAAACGATTCATGTCAACTATGGCTAAACAATACATGATGGAAGCAACAGAAACA

>contig00288

ACGAAAAtCACGtCCAAAGGAATGgTCtCcAaCtCCAGgATATGAATCAAaTCCTCTGGTATGGTCTCCAACTCCAGGATATGAATCAAATCCTCTGGAATGGCCTCCAACTCCAGGATATGAATCAAATCCTCTGGTATGGTCTCCAACTCCAGGATATGAATCAAATCCTCTGTTTGAATTTGGATATCCAATACCACTCATAGCAAATCCACCTTGTTGATTTCCTCTTCCAATATGTAGATTATATCCATTATCTTGGGCTGAACCTCCACTATATGCAAAAGAGCCACTTTGATGGTATGAATTTGGAGAATATGGAACAGCGACCGGTATTGAATCTGTTCTGGTCATTGAAATAGAGGACGTAGATGCAGGTTGTTGATTTGAAGTAATTGAAACAGTAGGAGCCATTGGCTGCTGATTTGGAAGAACTGCAACGCTGGGAGA

>contig00289

ACAAAGCTGAGGCTTTAATACGAATAGTCAGATAAAAGATTACCTTtCAAATATTTTtAGTTTCCGTGTATCTATGAAAAGAATAATTTTCTCAAAATCTTCGAGGCACGCAGTTCAAaTTTAAAtAatACCTTCTCATTTATGAGAATgTAAAAaTAAGCTTATTTTCCTgAATAGTCAGTCATTTTTTCCTAATTTCTATATACTGTCTCGCATAAATAATTTCTATTTTGGTCTACAACGTAGGCTATTGCAACATAACTAATCACTTTAATCAAATTAAAATAATTATCTGAAGCTCACAACTCAATAACTTGTGTTTTTCTTAATTAACTAGGAATAACTACAATACTAGCGACTATAAATAAGTCGGACGAAAAAaCAAGAAACGGCTTAGTTACTATGACCAAAGTATTGTATTAAAATCCATCCCGAGCGAAT

>contig00290

ACCATAATCCtCAATGAGCCCGATTTATATAGAGCCCACATTTTTAACAAGGCAAAAGCTAAAATTCATTCCGATtAGTTAACTTAATCCAAAaTTATTCGTTTTCATGGGGgCCTATTTTttCAAaCATTATAATTTCTATGCTTTTTttAAGTAATTAGGAATTACGCCATAAAaaTGAGTCCAAAAATAaTACCCTACCCTGGCGAAaGTATCGGATAGAAATTCATTCTG

>contig00291

GTCTTAAATTTtCATTGAGTGGGgCTTTtGCCGAGGTATGTAGCTAAAGTATCCGAGGCTAGTGTTGAGACCGGTCGCCCCGGATCTTCTGTCGTTCTATTTtCAGGCAaTTTtATCACAGAAAaGGACCTTATCACTAACACGCGTGAGTTCTGACGCGAtCGACAAAACGAATCTCGGAGTAGGAATCGTTCAGT

>contig00292

ATTCGGaGGGGAAAGgCAGCGACGAAaCAGCCATCGCATGTAGCATTCGGGTaTCgaCAtGATAAaGCCaTCCATGAAGATGGGGGTGATTGCCTTGATAGGGATATGTGTAATATtCTACCAaTACCATCTATCAaCTGGAATAAGATTTGACCAGCTAaCAGCTTTCAATGCTGTTGGCCCTTTGGACGAGTCATCCAATCTGCAACCTGGAGAAGAAaTGGTAGCTTATACTAGAACACTCAAAGTCACAGAGGAAATGATCCATAACGT

>contig00293

CCAAGACCTGTGCAAGCTTTTAACTACTGTATATCACATTCTCCTTCTAAAatCATCTCCCTTTTCTATACCGCTGCTGATACAGAATATGTATCTCTTTAAGTCTCATGTATAACAGTGTCATATCACAACGTTTCATTTTCAAGATTACAGTTTCTAGGTCATTAATTTCAGTAATCTTGTTGACCATTCCAGGTTTTAGAAaTAACCAGTGTCTTGCCTCCTCTTCAAGGAGTTGAGAATTACAATCAAAaCAAATACTACGAGACTCAGTCCAAGTCTGCAACCTTtCTTTtAATtCCTCCATCTGGACCAGAAACTATTAACTTTtCCTTTAGGTATTGGTATTTTCGTAACCTGCATGCTGCATAGTCCCAATATCCAAATGGAATTTGTGACAACTTGGCATTCACAATGCTCCATAATCCCcAGAaTAGGTGACTGGCTAGTGAAAAGAGCTTAATTTCTGCCATGATTCGGTCTTCTTCAACTTGGCCCTCTTTACCTAATGTCTTCAGGT

>contig00294

ACATATTGCAATCGTTCTGATCTTGACAGGAGTGTCAGAATGCAGTCCCATTAAGTCCCATTTTGAGAAATTTACGCTGATATTAGTGATATTTACGAAAGCTCAAAATAACTAGGCACTCCAGCACTAGACAAACATCTAGTCATCCTTATAAAGGCTCTGTCTTTGCCGTCTATGCAAAGGTCTTCGGCTGTGACGTCTAGTGATTTTAAGATTTTTATTATTTCTACTTGATTCAACGTTCAACTGCTCTTTTTATAAAGGAACCCAAACAAGCCCAAAGCACCCTTCTCTTTTGGTCAGAGATATTGAT

>contig00295

GAAATAATTAAATATGGCTTTTTTGGGAAAAGTAGTCTTGATAACAGGTGCTAGTTCTGGAATTGGAGCCGCAACAGCAATCTATCTCTCAGAGCTGGGAGCCTCTC

>contig00296

AAGCAAGCTTAGAAATGAAATCTAATCGCCGATCTAACTCAAAAAATGATTTTGATCCTGCCAAATTAAAAGTGATAAGAAATTTGCTGAAACCCAATAGATCTCCAGATTCATCAGTAACACAAATAATCGATGACGTAGATGATGATCCCGAAAGATTCAGAACTGATATTCCTGTGGATGAGGATGAGAACTATTATCCCAATAGTGATTCATCCGTGTGGTTGAGATCATGTGAACAAGAAGTAATCGAGCCAATTCATGGAACAATTACTGGAAATATTCCAGAATGGTtGAAAGGATCTTtATTGAGGAaTGG

>contig00297

ACCTCTCCTCCGAGAAACCCTTTTTATCAAAAACCAAATCTAAATTCGCAAGAGAAGCGAGTGTGTGTGATGCGTTGACGTTGGAAGGAAGAGATCCACCTTCTCTTTGTCTAGCCGAAACACTAGGTCCTGTGTGTTTCTTGCCTTTTCTCCtACcACCTCCgTATGTGTGACTCGATCCAGAAGTCAGGTATGCGAAGCTGCTATCAAATTCAGAATTTATGGgTTGCGCGaGATTTtGCAAAGATCCTCCGTGGGTTAATCTCTCAGATACGGAGtCCCTTTtGTGTCGGCGACGATTCGAGCCAGGGAGACAACGAGAATTTGCTACCGCACGACGACCTTCCTCGGTTTCATCTGCTTCTGCCTCTTGAAGAAGTTCCTTAAAGAGATTGGATTTTCCTTCACGATCTTTGGGACGTTTGTAAGTTATGGCTTCGTCCAATAATGCCCTTAGTTCTCCTTCATTAAGAGTAGAAAGAGTAGCAGGCTCCTCCT

>contig00298

GTTtATTATCTCCAACTATATTACACGGAATACTCTGAGGACTAGGAATATCATCCACAGGTTCATTTCCCGAAATGTTTAAAATATCACTACTAACATTCGTCTCTCCATTAGTTTCACTCGAAATTTCGACAGT

>contig00299

ACGGTTCCATCTTCTCCATGAGATTTATCAGACGAAAATTCGTTGAAATCTCCATCCCGAGGAAGTTTCAAATCTTTTTCATTTTGCCCAAAGGAATTACTAATTGGAACATTGTTATAACCTCTGTTCTGATAATTACTTTCACTACTTTCGTGATTTCCATGCGGAATCTTTCCATGCATTtGATTATATGCTCCATCgACACGTCTGAGTGCATGCtGTATATCATTA

>contig00300

TGATCTTGATTGTCATATTTGAAGCCGCCTTGGTTTTTTtGCAAGGTGTCTACTTGTTGTTTTATGTAACCGCAGTGACAGGAAATTTGTTTATCCCAGTCTCCTTTATCCATTTCGTTGTCAATATCGGGGACTTGAAGAGAGTGTCTATTCAAAACTTTTGTCAAATCATCAAAA

>contig00301

ACATAGTtGTTGGTTTCTACTGGTAGTTACTGAGATGAGGTGAAAAATACAGGTGGAAGTGAGCGGAAGTTATTTATATTGGTTTGTATATACAAATTACGTAGACATGACTGAGAAATCAAAATTTCATACCAATGGAATTGGTCTTTATAAGCCATTTGGAGAGATTAGAAAAGTGACAAACGTCCCTAATTTGTGGAGACGACTTGAATTTAATGAATGGACATTCAGTTTACTTCTATTCATTTTCTCCCTGATCATCGTAGTTATCAAATTGGCTGCTAACTAC

>contig00302

GGATACATTAACCACTGGCTACTTATCGATCGAAGCTTTATACCAGAAGCCCTTAGCACCTACCAGTTGATGGAATTTGATGGGAAGAGATTAGAGAACTTCAAAGACAGTATTAACGAATTACTTGAGAAATACGGTTGGGTTATGAGAGCTGTATTCAGTGGCTTGGCGACTATGGGTTTTACATGGTTTATTATCTACAAGGACAGCAATATTCCCGGAATCAACCCACCTTCACCTTTTCGAAATGCTAAAGATAGATTTACGAAAAAAACTGGAATCCCGaCCaaTTaTTTAGTTGGAGT

>contig00303

GACCTCCAGAAATTCTTCTAGGAACGAAATTATACTCCCCTGCCGTTGATATATGGAGCCTTGGTTGCATTTTCGCAGAAATGGCGACAAAGAGGGCTTTGTTTCCTGGTGATTCAGAAATAGATCAGCTGTTTAGGATATTtCGAACACTAGGCACGCCAGATGAAACTGTTTGGCCAGGAGTCTCGCAACTtCcAGATTACAAGTCAATGTTtCCTCGTTGGGATGCTCGGAGCTTAGACGAAGTTGTCTCTACCTTTGACGACAATGCCAAAGATTTACTTACT

>contig00304

AGTTAAAaGTGGAGTCTCATTTCATCAACGACCTGGGATTGGACTCTCTGGACCACGTGGAGATCATAATGGCGATAGAAGATGAATTTGGTTTCG

>contig00305

CCACAATTGTGTATAAATACTGCAATTGTCTACATGCACCGATTCTTCATGTTCCACTCGTTAACCAAATTTGACAAATTCAAGATTGGAGTAGCAACTCTCTTTTtATCAATGAAAACAGAGGATAAACCGTTGAGAATGGAACACGTTATTCAGCATGCTCACGTATTG

>contig00306

ATTGCCATCATGTAAAGCAGACTTTGCCGGTCACATAACCTATAGGCAATTAAAAGTGAAAAAaTATTGTTCGAGGAAACGTGTGCAGTGTTAAAGATTAAAGAATGGCAGCTAATACAAAgtGGATGTTCACCAAAGAACAAATTGCAAATTCACCAAGCCgAAAaTATAACATTAGTCCAGAAATTGAGGCGCTTTATAGGCAACAAAGTGCAAGTTTCATCCAGAAACTTGGAGAATCGCTGAAACTT

>contig00307

AGTCCAATACTTGGTGGCAAATGTCTTCTAGAAGATCCATAGTAACATCTTCAACGAACATGTCCCACCATCTTAAATGTTTCGGTTGTCTTCCACTCCAGTCAACGACTTCAAATTTACTTAATTTTCCTGCTAAATACATAAGTGCTACTGCTATTATTTCTGGTTCCCACTGAAGGCATAAGGTTGTGCAGAGACTGTCGTTCACGAAGGTCCAGGCCATTTGAACCATCTTTTGTAACTTATTCTTATCACCTTTTAAAGACTTAGCGTATTTCAAAAGATAGCTGTATGGATGTTCGACTTGAAGATCAAATTTAATTGTTTGCAGTAAAATCCTTTCAAGTGTCATTACTTCTTC

>contig00308

AGGTAATAAAGCTTCT

>contig00309

AGTCTCCATGTTTCTACCATTGAGTTGACAAGGCCTGTGCAAATTGGTGCAAATTCAAATGACAGTTTTTTCTGGTTTGTTAAAAAAGTATCCCGGTTCCTGAAATATCAAATCTCAAAATGTTGAGACCAAAGGCTCTTACTCAAGTTTTAAGTCAGGCGAACACTGGAGGTGTCGAAAACACCTT

>contig00310

ATTACTTAACAGAGATGGTGGTCTTCTGGCATACAGTGGATACGGCGATAAAGATGCAAGAGTTACAGCTGCTATAACTAGTAGTATTTGGTCTGCTTATGAAAAAAaTGGGCGTAATGCTTTCAAGGAAGATGAGCTTCAATTCGTGCTAATGGATTGTGTAAATGGAAAAGTCGTTATTACCGAAGTTGCTAATTTGCTTCTGTGTCTTTATGCGAGGGAAAACGTTGGATTTGGGCTTCTACGAGAAAAGGCCCAAGCATTAGCCAAGTATCTCGATCAGCCATTAAAAATGATAGCCAACATGCCATAAAAATCTTtaTTTtAATTtCGAACCGTTCATTCTACATAAGATTTtCCTGATT

>contig00311

AtCTTTTAATCCATTAGGGAAAaCTCGACCTTTACGCTCTTCAGCCTTtCTGAAATCTCGCATAAATAATAGGAAACCGTTTGGCTTCTGTTTTGGTGCCATTTtACCCTTtGTTCCTGATATCGCCCTtCAATGAGAAGTCAAACTTTCTTTTACAAAAGCGAATTTAGATAACCGGGAAGCTTCGTTGAAGATATATTACCCCCAGTGTCAAAATATGTTAAGCAGGTTTAGCAGATTAGCGTCCAATTCAATTGATATATATCATGTAACTCAATGCTGTTCATATGGTTCCTTTCCT

>contig00312

aCATGGGGgcATTCTTGGTTAAAGTGTCCATCAGAGATCTTCTAAGAACTTCTAAAAaTGACTGGTCGAGGAAAGGGAGGAAAaGGATTGGGAAAaGGAGGAGCGAAGCGCCATCGTAAAGTT

>contig00313

aCtATGCGAgaggATGTTTagCCGCCaAATCCGTAGAGGGTACGTCCTTGACGtttcAGAGCGTAGACAACGTCCATGGCGGTGACAGTCTTCCTTTTGGCGTGCTCAGTGTAGGTGACAGCATCACGAATCACGTTTTCGAGGAATACTTTTAGCACACCACGGGTTTCTTCATAGATTAAACCTGAAATACGTTTAACTCCGCCACGACGAGCAAGACGACGGATGGCAGGCTTGGTTATACCCTGGATGTTATCACGAAG

>contig00314

CGGTGACGGTGGTCGTCACAATTGGGTTTtATCACATTATATGATCCCATTCTTAGGTCTT

>contig00315

GACTTTAAAAaTCAAGCAAaCTCTATATCAGCTGAGAAAAaGAAAAAaGACCCcAAaTGTGTtGCTGCATTTAGCCcGAAACTtGGGGTAAAAAAGCAATGGGgAATTGGAGTTATTGAAGCTACAGGCTTAAtGCaTTTATCAAACGAAGAAaGAGCTTGTATGTTTGAGCCTGTTGGACCACAACCAGTtCAAACAGCTCAACCAACTAATAAATTGAAAAGGAAACTCTCTACGAGGGgAaCTAAGAATTCGAGCGAAaCTCCGACACCTGG

>contig00316

ACGCAATCTTCGCATGACCAAATCACACAAAAtCAAACtGCAAaCGTTtCCGtGCACCATGAAGtGGACGAAGACTCCAGTGAAGACGAGGAGAATGAGTCtGACGGCGAGTGCGATGGAGATGATGGCCTACCTGTAAAaGAAGAAAGTGAAGACGCTAGTAATAGGACAGTAGAACCCACAACTTTCGTCAACGTATCTTTAGCTTGTGATGaGgCcGGCCCATCGGGACTTCAGCAaCAAAAGATCaCCGAAATTCCTGAGATGGCGATATCGCAAGTAGCCAACACTGAAAaCAAGACTGGG

>contig00317

cgTTATCTCGATTGTCGAATTGACTTTTCTGTAAAAaCTTTCAACGCCGTTaTCAATCTCTGCAAAGAATTCGGCATCAGACATCCGGAAGAGCTCTCG

>contig00318

AGTTAAGAAACCGCCACCAATAATATTCACGGATCCTCCTGCGGAAGACGCTTTCCCCAAAAAGCAGCCAAAACACCCTTCAGCCGAGGTCAATAATTTTCAAGAAAGTGAGATTGAAGTATCGGGTAAAACTTCTCGTAATGCTTTTCATGGAGGGCTGGTCGGTGCGGATAAAGACGATTCTAAATAAAAAGACCATTCGGAACCAGAAGACCATCACGATCTTCACCATTAAAGATTGTTTAGAGATAAAATTTGAAATACGATAAACTAAAAGTGACGGGTTTAAAAATGTATCTGCTGTTGCAGAGATTATACATGTAT

>contig00319

GTTTTCTCAAAAGGAAGTTGAGACCCTGTGCCTATTAAATGATTCTAAAtGCAATTTGATACTTAATTCTAATTATATTAAATATGTCTTTTAC

>contig00320

GCCTTGTCaTTTCCGCAAACCTTGTGGTCAGAaGGTATCAGCGCATGCTCGCTCGCTATcTCACCGGCGCTTTAATCCACATAGACGTGGTTGTGCAAATTTGGGCCCTCACCATCATCATCATAGACACTATCTCCAACGTGAACCtCGTTTCTCCCACTATAAAGAAGAATCTGCAGGGAGGAAG

>contig00321

ATAACGATTTTCCGACTCGTAATTCTTATGGACGATATAAAAACGGCCTACATTATTCACAGGATCGAAGCATAAGCTATGCTTGGGCAAGAAATTCACAAGGGGGATCAGGAAAAGGTTGGCAAGCAGGTGCACAAAGTATATATACTACATCTGCAATACCACAAATCAGTAAAATTATTGAATATTCCAATGCATTAAAGGATTGTTTACCAATCATAGATAAAAAGATTCAATTTGTCAAGTTGAGTGATGACCTTCGAAAGAAAATTATAGATTCGGCTGGCCGGCTGTCATCTATTTATACGAATTTGCTGAAAAATCTTAATGGAAATCCTTCTTTCCAAGGTCAAGTACCTTCTCTACCTTCTCTACCTTCCCTATCTTCTCTACCTTCTCTACCTTCTCTACCTCCTCTACTTTCTCAACCTTCTCTATCTTTACCTTTTATGTCTCATACTTCAGAAGGAAaTGTACCCATTTTAAACCTACCAAATGCAGTGAAACGAATTGGATCAATTAACAATTGTATTTGGAAAATTTTTAATGCAATGAAAAATCAGCACTATTCTGGATTGACACCAGCTCTTGTGAATAATATCAATGATCTTAGCCCCTTATTGGGAAGGCTTCTGAGAAGATTGCGAAGAATCGATGATAATGGTAACGGTCGTATAATTAATCCAAATATCGGTTTACCAAGTGTTCCGAGGGTCGGCATTACAGGTGTACCGAATGTCCAACAAGACAGCTCTCCAGTAGTACACCAACCGATCATTCCAAGTATCCCGAATCTGCAAAACGTCGGCATTCCAATGGTACAGAAACTGGGCACTTCAAGTAGCAGCATGCCTGGATTATCTAATTTGAGTGTGAAAATGCACGCATAATTATGTATTTCGTCACATTAATTTTtAATGCTAGCAACGTTTCAAAAATACTTtAATATAATTGGAATATACTAT

>contig00322

AACCTCCATTCAGAGAATAATCATTACTTCTTTGGTGATATCCATGAGAGTTACGAGTCGGAAAATCGTTAGAACCTCCATTCATAGAATAATATTTACTTCTTTGGCGATATCCATGAGAGTAACTAGTCGGAAAATTGTTAG

>contig00323

AGTTCGAGTGTAAAGGTTGTCTCAGCTTGTCGTTCGCCAAGAAGATTGAATCAAAGCCATCCGAAATGGAAAGGACGCCAACAGTGACGCTATTCATTATCTTTGCAACTATTTTCGCAAAATGTGATTCCAATCTAAAATGCTACGAATGTGTTGAACACCTGGGACATCCCCTCTATAAAAATTGCTCGACACCTTTAACTCATAATGTGAGTTCTGATCTTTTATGTACAACCAATTCTTCTTGCCACGCTGCAATTTTCCATAGAGCAGAAAAAaTCATGTTTACGCTTCTATCATGTAATGCGACATTCCATGTGACTTTTCTTCCCAGGGATTACTATAATAATACTGACAATCCTATTTCGGAAAATGCAACTTTATCAATGTTCTATTATCAATGCAAAGACGATTTTTGCAGTAGCAATGTAACATCTCTGTATGAGATCTATCGTCAAAAAGAGAAAAACAATAGTTCTCGACTACATCtGaCGAGGATAACATctaCAATTGTATTTTtAACAATGTTAACATCATTGCTATCTCTTCAAAATGTGTAGTCAATTTCACcGt

>contig00324

ACTTTtGtAATTTTTATCTTAAACATAaTCAACATAACTACATTTGTCACTTGTGTGTAAATCGACATGCCTAAAAATGaaagTTTAagCTCAGAACTTTTGTGAAAACGGTTTTAGTTCGATGGTAAACaTTGGAAGTATCGCGAAaTCCtATGTAAaTTATAGAGGAATCTTAGTGCCATCTTTAACTGTAAAGCCTTTCTAACAGTATTTTtGAGTTGAAACTTTCGTATTTAAACGTGTCGAAATGATACGCACTTTGTtATGAATTTTATATTATtATACAAAaGACGTGAAATGGAAAGATGTATATTCCTAGCAGTCGGTATTATTtACATTCTCCTTTAAGGACATGTGACACTTTTAGTTTGAAAATAATAGAAATAATTAAATACAAATTTGAGaGAAACTAGAGGT

>contig00325

CATGCAAATTGACGTAGATAGCTTATTTTTAGTTTTGACAATGAAAAAGGTTTGTTTATCTTGCATTGGCAGTATAACTTGATAAATTAAACTTTTTTAAATATTCCAAGCAAAAGTAGGATGTCTACGTTTATTTTCATGTAATCCATCATTTCTCAAATTTTCAAGACAAAATCGTCTTCATTTCGGTAATAAAGATCTGCAGAAAAAaTAAAATTTACTTAATTTAaGAGCATGATTaGTTTGCTCTATTCAAGAATGAAATTTACTTAATCTAGGTAAAATTTAaTTGATCTATAAAGCACATATTTGATCTAAGTAAATTTTTCTGAAATTAAGTTCATGTCTTTGTTGAATAAAaGCAACTAATTATGCTCTTGGATCAaGTAAATTTTATTTTCCGTGTAGAAACTTAAAAATCATAAAATTTGCTAATTTTTtAagTGTCACATGTACTAAaTACAACTTATCTTTTTtAtGTCTTtATACAACATTTAGTAaCTAAAGAATATGTA

>contig00326

ACTTgAATcaGAAAGTGTATGAAAACTGACACTAGACCCCCcAGCACCTTCTCCAAATAATGTCACTCGATTTGCATCACCTCCAAATTCTTTGATATTTTCTtGGACCCATTTTAAAGCCAAAACCTGaTCTTTCATGCCGAAATTGCCTGGAGCAGCAGAATCTCCTGTTGaGAAAAATCCAAAAaTTCCCAAACGATAGTTCATAGTTACTAGAATTACATCCTTGTCTAGAAGATAATTAGCACGATAGTCATCACTGCTACCAATAAAGAATGCACCTCCATGAATAAAAACCATTACTGGCAGCAATTCGGAGTCCTGCATTTCTGAACCAAATTCCAAGCGTGGTGAATAAACGTTCAGGTATAAGCAGTCTTCATCTCCCATCACTTTGCCAAATATATATTGCAAACATGGTTTTCCATATTCAGTTGCTTGCCGTATTCCCTCCCATTTATCTCCGGGAACAGGACTTTTGAACCTGAGATTTTTtAATGGGGGCTTAGCAAAAGGTATCCCAAGGAACGCTGAATACAGTCGGCCATTTAAGGATTCCTCGACGGT

>contig00327

GTGATCAGTGTTCAACTGTTCAACAATCTTGAGGGTGGTGTTCGGCGTCATTCAGCGTTCGGCGACTTCGGCGTTGACTTCTGACCGCATTTGAGCTTGAGGTGCAGTGTATATATATGTATATGAAAGACAGTTAGCTTGGTGCAATATTTCATTAACGATTTTTTGGAATAAGTAAAACGGCGCTCGTAGAGCCCAAAAGTTTGCAAATTTGCAACTCCACAACGTTCAACGATGGAGGTTTTCACAAATATTTCGTCCACTTTCTGGTCACCTGACATTTGGTTACCTCCGAACACGACGTGGGACGACATCGAACCCAATTTGACTAATAAATATGCAGACCATAGGCATTTACTATATCCAATTCCTATGGCCTTCATCCTCTTGGGGATCCGATACGCTATGGAAAGGTATTTTTTtAGTCCACTTGGGAAGTGGCTAGGAATAAAAaCAACAAGAACAAaGAGAGCACAGCCGAATCCAATATTGGAGAAAGCCTATGCCAATAAACAAATTAAACATAAGCAGATATTAGCCTTGGCGAAGCAATTAGATTGGAGTgAaCgaCAAGTAGAAAGATGGTTGAGGTTACGGCGTTCTCAAGACAGGCCTTCAACTCTGACTAAGTTTTGTGAAAACAGTtGGAGATGCTTGTATTATACATATTCATTtATATATGGTCTCATCAtCTTATGGGACAAaCCATGGCTGTGGgACATTAAAAaCTGTTATTACAATTACCCTTATCATCCAGTAACGAATGACGTGTGGTGGTATTACATGATATCCATGGCATTCTACTGGTCTCTAAGTTTTtCCCAATTCTTTGATGTCAAACGAAAGGACTTCTGGCAAATGTTTGT

>contig00328

ACATCGATAGAAGCACCTAAGCTACTACCAATgTTCCCCGCATATTACATTTTCAACTCACTACTAAGTCTGCTGATGGTCCTTCACGCGTTTTGGACCTGGCTAATACTAAAGATAGCGTATAATGCCTTTTATGCTGGTCAGATGGAAGGTGATATTCGAAGTGACAGTAGCGAAGAAATTTCTGACACTTCTGTAAACAATGCGTCAGTCAACGATACTCCTAATAATATCAGCAGGAATAATTCCAAACAAAaGTTAAaCTAAAaTATTCTTATTTAAAAaTTAATTTTCATAACTGCTGAATTAGACGCTAGTTTTTACGGTGAAACTCTCAGCGATTTCATTATTTCACTAAAGCTCTAAGTTATATCTCTTtATtGG

>contig00329

TCACAAaTGTAGTGCACAaGaTTTTGtAAAAACGTGATGTCGTCAAATCTTACAaTTGCCAAAAACAaTACCGGTGAAATAtatAGTTTAGTTTTTtAGAGTTTTTAAATTCAAGTATAGTGAATATtGTAAAaTAGTAGCACATTTTTAATtGTCTGTTCtAAAaTTTTCGTATGAATCTTATATCTTCTACGATATCTTGAACTTtAGATTTTATATAaCAGAATATtCATAaTATTAATCTATCATTATATTACAAATAGTGtAAGATAAGTAAACTTTCATCTGCTCTTAAAaTtATCgCATACAAAaTAACTTGGGACTTGAAAtCAACAAAaCAGGTAAAaTGATTACAAAATCAAAGAATAATAATGAACCTTAGAACATAAGTGAAGCGTTATTCAGTAATAGACTAATAAGCGCATTGTAGGTTTTTTAAAAAGaTAACCCTATTGCCATAAACTCATGCAATTATCTCGTTGCATTAaGAAaTTCCCCAGCGCTGGgCATCGATTCATAACGCAATAGTCTTAACTTATAAATAAATAAAAAaTAAATgATTTtAaGaTGCTGTCGaCGTaTTAAaTTTTTGCTGGATAATTGATGTCTTTCGCTGAGGT

>contig00330

aCCTGGTTCGGGAAGATGAGGCCGGACGATCTTGTTCAAACTGAACGAAGGATGACGGAGACGATGTCGGAAACATCTGAGGGCACGGGAACGACGAGCGTCTCTGGGAAATCCAATTTCGAAACTGAGATGCAAAACTTTGCTACCCTCTTAGGCATTGCAAATCCCGAAGATGTGCATCAGGACCGATTTCGAGTAGATCGACGAAAATTGGAACACATGTtGTCCGATGACAATCAAGCTCCAAAGGCTGCCGACTCATTTTTtcAGAAGGTTATGGAAGAAaCGAATACTTTtGTCACAtGGCCTTCCcGACTGAAAATTGGGGCCAAGTCGAAAAAaGATCCCCACATAAAAGTtGCTGGACGACTTGACGACGTGCGGGCTGCGAAGGAAAAAaTCATGCAAATTTtAGACACGCGGCAAAaCAACCGAGTAACCATGAAATTGGACGTCAGCTACACAGACCATTCTCATATTATTGGCAAaGgCGGCTTAACAATAAAaCGCGTAATGGAagAAACaGGTTGCCACATTCATTTtCCGGATAGTAACCGTAGTAaTCATCAGGAGAAAAGTAATCAAGTCTCCATTGCAGGAGAAATGGAAGGCGTCGAGCGtGCTCGCGCTCGAGTCAGGAATTTAACTCCTTTGATCTTCTCGTTCGAACTGCCGATAATGGGTGCGTCACAatCGATGCCAGACtCTACGTCGCCATACGTGGTAaaGATtCAGGAACAGT

>contig00331

ACtATtGGGATCCCTCAAATAAAACCTATAAATTGATTAATTTTAATAATTTATTTTATTTTAAATTTAATTATAACATTTTTATCAATATACTATTTTAAAATTAAATTACCGTCAAATAATAAAATTAATAAATTAAATAAAAATATAAAAATTATATGATAACAAATCTATTTTCAATCTTTGACCCATCTACAAATTCAATAAGAACTTTAAATTGAATCAGAATATTATATTTCATAATATTTTTACCCAATCTTTATTGAATAACTCCATCAAAATACAATTATTTATGAAACTTTATTTTAATTAATTTATCTAAAGAAATAAAAAATTCATTAAATAAATTAAATAATTATTTTAATATTCTAATATTAAATTCATTATTTATATTAATTTTATTAAACAATTTTATCAGATTATTTCCATATATTTTCAATACAACAAGACACCTATCTATATCAATCTCATTATCATTAACTATATGAATTAGATTTATAATTTTCGGTTGAACTAAAAATTCATATAACATATTTATTCATTTAACACCTCAAGGAACCCCATTTATATTAATACCATTTATAGTAATCATTGAAAGAGTAAGAAATTTAATTCGACCATTAACTTTAGCTATTCGATTAAGAGCAAATATTATTGCCGGACATTTACTAATAACTTTAATTAGACAAACAATAACAAACTTAAACTTAAATTTAATTATTATAATAATTTTAATCCAATCAATTTTAGTTATTTTAGAACTAGCTGTATCATTTATCCAAGCATATATTTTTtCAATTCTTAGATCACTATATTTAATTGAAACAAATTAAAATTATTTAAAaTTAACATgAAAAAaTATTTTTATAACCATCCATGCCACTTAGTTACAATAAGACCCTGACCAATACTTACATCATTTAGATTATTTATTACAATAATTGGAACAATTAATATATTCAATTATTACAAAATATCCTTAATATCAATTGGTACATTAAGAATTATTATATGCTCATATCAATGATGACGAGATATTATTCGAGAAAGAACATTTCAAGGATTACACTCTAATATAATTTACAAAAGATTAAAATTAAGTATAATATTATTTATTATCTCAGAATTACTATTTTTTATATCATTTTTTTGAAGATACTTAAATTCATCCCTATCCCCAAATATCGAAATTGGTAATATATGACCACCAAAAAATATTCATCAATTTAACCCATATGAAATTCCTTTATTAAATACAATCATTTTATTATCCTCAAGAATTACAATTACCCTAAGACATTACTACCTAaTAAAAAAAAAaaTAAAaTCAATTATTATTTtAAtAATTAC

>contig00332

AAGAcAAaGAGAATAGTGTTGTCAAATTGTTGGGAGGTTCGTTCTTTTATCAAACGCGAAAATCCAAGATGTCTAAGTTAATACATATACTTGGTATATTGTTAATTTTTACTGTTGCTTCTGAAGGTAAAAAAAaTCGTTTGCTACTATCCGGACTGGTCGCAACTTCGTGAACCATCAATAAGATTCGATATTAGTGACATCAACACCGAAATTTGCACTCATTTAGTTTATGTCTATGCCGGAATCTCTAGGACCGGGAATGTCAAAATTTTAAAGCCACAAATTGATATAGAAAATCATGGTTATCTAAGATTTACAGAGCTTGGAAGGAAATCATCACTACCGACATTAATTGCTGTAGGTGGATGGGAGGAGGGTTCCGCTAAGTTTTCCAAAGTAGCAGGAAATTTCAAACGTCGAGCTAACTTTGTTACAAATGCATTAAATTTCGTGAAACAATACCATTTCAATGGTCTTGAACTAGCTTGGTCATACCCTGCTCAACGGAATGGAAGTGAATTGGACAAAGAGAACTACGTTACGTTATTAAGACAACTTAAAGGAACATTCGAAAAGGAGAGACTCATCTTATCAGTTTCAGCTGGAGTATCTAAGTA

>contig00333

ACAGTATTGgATATCTCTtGGTGCACCACCAGAAAAATTAATATTGGGAATGGCTTTCTTTGGAAGATCATATACTATGACAGACACAAAAATAATAGGCAGGGGTGCACCATTTAAAAAACCGGGGAATCCTGGTAGATATACCAAAACGGAAGGTTTGCTGGCATACTTCGAAATATGTTCTTTGATACAGGAACACAATTGGAAGGTCATTTACGATACACAACAACGCGTTCCATATGCCTATTCTGGAAATCAAATTGTATCTTATGACAACCCCGAATCTTTGGCAGTAAAagCACAATATGTTTTGGATCaCAATCTTGGTGGAGTTTCGGTTTGGACTGTAGATTtCGGCGaTTTCAaGGgAATCTGCGGACCAaCATaTCCTCTTCTTAAaGCAATAAAaCATATCTTAAAAGGTtAAAAAGTTTGTaGCAGTtAAAGTgtGTtGTGTCAAAAAaaTTTTAAGTGTGTAATTGCTATTATTATATTTTTCTGATTGTtA

>contig00334

ACTGAGAGCTTATTGAATTGAGTTTTTTTtGTGAGAGTCATTGGTCGCGCGCGTTTAtAAAAGTTCTGCTAGCGCAGTTTtCAATaTTTCGGATCTAAATTTTtGAaGAAAAAAAaGAATCGATTTTACTGGAGTTACGGAcGCTAAAGCCAGATCGAAACGAGAGTCTGCTAGGTtCGTTTCTCTATTCaCAAAaGGAGGAAAGATTTGAAAATTTGTCAGAGCGGAAAAatACGGGTCCGGgTtCTTTTTTTTTttACcACGCTCGGGCCAAGTGGTATTTTCATAAGTGCAAGAGTCCTGTGCGAAAATGTCTAAAATTGAAGTAGGATAAAGTTGTTACTTTTAGTAGGCAATATTCTCGTTGAAGTCAAATACCACATACGAGAAATATTTGGAGTGCATAATTATAGATGTAAAGAATGAAAAGCGTGCTTCTACGAGTATCGGGTATTGAAGGGAACATTTAGTCGATGCCTATGGTTCCTGTCTGGTAAAaTTTGATAACATATTATTAAATTTtCATACAGAATTCGTTAAAGAGTGAATATGTTGTGCCTGAAACTGCCTCTAAACTCTCGAAGATGAaCGCCAAGT

>contig00335

ACCAAGGGgAAAaTtAGAAATGCGCaTCCTGTCGCCAGTaGACTAGGAGTATACGTCATTATCTCTTGAATTCtAAACTTTATTtATTTAAgaCGATGACCGtGTTGTGAGTGAAGAGGAAAAGAATTGTTTtACTTTtATaCATTTACTAGATGGTTTTGATTTTTGAACGGGACGTGCCAAGCCGTTCTTCACACGCCACTTTTCTAGGTCGAATGCCATGAGTGGAAGGCTGAGAATCTCAAGGGAAAGGGAAAAaTACCTAAAAATAAGAAGGAACTTATTATACTAAAATATAATTAGTTATTGGAGAATGCCGGACGCATTTATGACTCAAGCAACTTGACATATAAACGTTTCCCTTTTTCTTATTTAAGAAAGTCGTCCGTGCGATCTAAAGCTTTTGTCTGCTTGTGGCACTCGACAATTAGTGAAAAGGATCTAAGAATAAAAAAaGTGAATAATGCATGATTATATTCCTCTCGAGTAATAAGCAAAAAAaTGAAGAGGGCGTAAGTCGCTAATCATTCACGACTGTTATTTTTGTATGGCTCTTATGGAGAGTCGCACCAATATACATACATACTATTTAGATATTTAGTTGAAATACTATAGGAGCGAAATCCTTCCTAGGCTGCGCGCGTAATCAAGATGATAATAAACGGAATAAGGAAATGATAAagTCGCTGAAAaTGAATGCAAGACAGAGAGagAAATAGCGCATGTTTTATGTTTGATCATAGATACACTTATACAATaCAAAAAAAGGGAGAAAAAagTAGATATAtACAATGAGcGCGATTTCTGACAGTGTATGTTTTGG

>contig00336

ACAAAGTAAAAaaCACTATCAAGATGTTTGAAGCACGTTTAGTtCAAAGTGCTGTCCTGAAAAAGGTGATTGAAGCGGTTAAAGAACTTTTATCCGAAGCCACTTTCGAGTGCTCGGATTCTGGAATGCAAGTCCAAGCCATGGACAATGCCCATGTTTGTCTTGTTTCCCTTAATCTCAGAAGCGATGGATTCGACAAATACCGCTGTGATAGGAATCTATCCATGGGGATGAGTATCACGTGTATGTCAAAAATTCTGAAATGTTCTGGAACCGACGACACTGTAACCTTGAGGGCTTGGGACAATCCAGAGTCAATAAATATCATCTTCGAGTCTCCAAACAAAGAGAAATTGGCCGAATACGAAATGAAACTCATAAATATGGACCAAGAGCATCTTGGCATACCCGAAACTTCATACTCTTGCACAGTAAAATTGCCATCAGCCGAATTTGCGAGGATCGTCAGAGACTTGCAAACTTTtGGTGAATCAATAACGATCGCATGTTCAAAAGAAGGAATCAAATtCAGTGCAGCTGGTGACATTGGCACAGCAAATGTGAAATTGGCCCAAAcTGCAGATGCAGACAAAGAAGAAGAAGCTGTGACTATAGATATGCAAGAACCTGTGAAACAAGCGTTTTCTGCTCGTTaTCTCAATAACTTCGTCAAGGCAACTCCTCTCTGCAACCAAGTTCAATTATCCTTGTCGAaCGACGTTCCACTTGTCTGCGAGT

>contig00337

tATCCGAAATTGGCCACATTCGATATTATTTAGCTCCTAAAATTGAAGATGAGGAGGAAAGTTAAGTTTTAATAATAATATTAATTAAAAAaTCGTTCTATTTAAGTTTCATCGTTCCACTGATAATTATTTATTCTAACTTCTTTGCTTGCGTCTTGTATTTATATATAAATCGGAAGCAAAGAATTGTGTGGAAAaTGGATTGAATTTTCAGGATATAATGTATTTATAATTAGTTAAAAATAAGAGATAAAATCTAGAAAAATGACAACATTTTAAAAGTAAAGTGAAAAAAtGAAATTTTTAAATTATTCACGATAATTACATGAATATTTGTATGGATTCGCGTATGTAATGCCGAGTAGAATACATTTGAAACTTTTTtGaTAGACCTTTCTAATTTATTTTCGGATTTCCAATACTTGATATACAAAAaGGGAGGAAAAaCTGAgTCATTCTGATAGTTCGAAGTCGATATATTTAAATGTTACATtCAATAAAAGATATTTGGCTCGTCACAGTATTAGCACAaCATCAAATTGACGCAAATTTTTGTAGCAAAGTGATACAATCACGACGAATAAGAGAAAATGCTCTTAACTCTTTTAAATTATTTATACACTTTCATCGT

>contig00338

ACCATAAtAtGAAGCATGGTGTTGTGGCGAGTAAAGAGTTGGCCGATTTTTTtCGGGAACGTTCGGCAATCGAAGAAAaCAACTACAAGCTTCTCACTAAACTGGCAAaGCAAGTTAGCAGTAGTAGTTCTATGCAaGgAaCATTtACACCTGTTtGGGTGTCATTGAAAGGTGTTTTGGAAAAATTAGCCGGACTGCACTTGCAAATTGCACAGAAAGTCAGTGATCTTATCAAAGATGTCTCCAGATATTCAGAAGAACTCCATAAAAAaCATAAAGCGGTAAAAGAAGAAGAATCGTCAACAATGGAAaTTGTGCAGAGTATACAAAaTGTAACGGTTACATTGCAAAAAGCTCGCgAaCTCTGTTTTCAaCGTGGTTTGGAATTAGAAAAGCTAAAAAGGGATAATGCGAGCCAACGTGAACTTGAAAAAGCTGAAGTAAAATTCAAAAAAGCaCAGGATGACTATAAAACTTtAGTCGAAAAATATAGCATTATTCGGAATGATTTTGAGAGCAAAATAAGCCAAGCTTGTAGGAGGTTTCAATTCATCGAAGAAACACATTTAAAACAAATGAAAGAATTCTTGGCGATTTATGTTGACGTTCTTCAAACAAaTCATGAAGAAGTAGGTCGAGTGTATGCAGACTTTAAACGTCATTGTTTAGATATGACAGTCGAAGAATTGTTACAGCAATTTGTGCAAAGTAAAAg

>contig00339

GATTAATTGTAATGCGCTACTATATTtGCCGTCAAtCACTGAaTTGAATATATTGGgAAAaCACTTTTTTtGTCTtAATTTTTACGCTTGATTGACCTGGAATGTGGATTTCGACCCCTGGTGGgCTCGAGCGTAATA

>contig00340

TTTTTACATATTTCAGAAACATTTTATGTTATTGACGAATTTAAAATTCAAATTACtGACGAAGTTAAAGTCTTtCAGACAAAAAGAAATCAAGTAGCTTTTTCTAATTCAAAAaCTTTtAACTACTATTTATTGCCAAGAATGTATGCGACCTCTTAAAaTCGCTTATCAGCATTAGAAAGAATTTCTACTTATAACTTCTGACGCtCTAAATTACCATGGAGTTTtATGGAATTTCAaTTTTGAACTTCGTCAGGTTCTTTTGTAAGAGAAACCATCAATTAAGCAGAGAAACAGCTCGAAGTAAAAATCAACATAAACATTATTAATATCATTGCTTAACTTAGCAATATAATCCATAGAaCTtCTtAGTTGATAAGTGCTGTACGCTTGGGTCCGACGTTTATCAGGAAaTCCTTATTTtAACTGATAAAaTATATTGGAaCTAACGTTTtGCAATCTTTCTTCACCATATTCAATTTTCGATTTCTTATACTTTTTCAaGCCAAACTTTATTTTAGATCGGTTATTTTTCACTTAGTCTCGTAGTTTCAAATATGTTtGACCgAAaGTGTATTAAGTATTAATTTAaGTATTCCCTTAGTTTAAgaCTACTTTtACGAaGAATaTTATTGATAATATGAGCTTGAGTATTCATTCAAGCTCATATTTTTCGAATTTtAAAAAaTTTtGACGCACATCAATTGTATCCACATAATTAACATTTGTCTTGTTTATTCGAGATAGTGTGtGAGACTTCGTATTAAATATCTATTGTTATTGTTGctGCTAAAAGTATAATTATTTTCTTAGCTTATTATTAAAGAGGTAGATATCATACTCTTGGTAATTCCCAATCAGTTGACTTTaGAGaGATCTTGCTAATAAaTGCGCTCTTTAGTGTTGACTTGATtAACAGAcACCaCTAAAATG

>contig00341

TTTACTGTATCTTCTTAAAAAGTGAAGAGGTAGTGGTTTtCCAAAAGAGTAGTTCAATGTCCTTACAATAAGCATCTCCATTTGAAGGATTTCCATCTTCGAGTACGCGTTATCAGTAATATAAACAAAaTCGCTAATATCTGGAGAGTACaTCTCCTCGTATTTGCTGGCTATGAACATGGCAGTGACACCTACTAACTGCAATCTCTTTCTATCTATCGACCGGAaCGACTGCAAGAAGCGATCTATGATTGAGACGGTTAGGTAAAGCGTCTCTTGCATCAAACGAAATTGTtGATGCACTTCGACTAACCAGTCGACGAGT

>contig00342

ATCTCATTTTtGGCGTAACTTCTTGTCCTGAtAAAAAaCCT

>contig00343

ACTTCTACTTTTACCACCGGCTTGACAACCTGAACTGGTGGTTTGTCCAATACTTTTtCTGTTACTTTATTGCCTTCCTGTTTAAGTGCTGGCTTCTTCTGTGCATTGGTGGGCAGTAGACTAGATTTGTGAATCGGCTCTACGCCTCTGTGAATCGTGACTTTGTTTCCAATTTCTCCCAAAGCGGCTCTCCGCAATGGTTTTTGGATTCCAATTCCAGCTGATTTTGTATTCTTGACATTTTCTTGGTTTACGATACCCGTAACTGTTATCCGATTTCTCAGCGCCATCGTCAGCTTGTATCTTCGTGAAAATAAAAGTCACTCAAGAAATCGTCTTGCAACGGAAATGAAGCCCACCTTCAATTTCCGAACAACGGAATTTTCTTGATAACGTCTCTCGTTTAAATTTGTTTAGTTGCTGACTgCCTGACTGTTGCAaGACGAGAG

>contig00344

ACTCGtgaTAAACGAAGGATtACaTCGAAAaCACAAAGAaGCTCTTGCtACTGTCAGGAATAAaTATAGTCATAAAaTTTTCTTtGAaGTAGCAAAAGTGCCaTtGAAGgATGTTCTAGACGTATAACTAGTATTTAAGTATGTAAGTATGAaGAAAATAGACGAAaTGCGCGaCTGATCGTATTGATCGGCCAAGCTTGTTGTTCATTAAATCtAAATTACGATGATCTAAAAAAaTGCGACTGGgACGAATTATCTACCGATGAATGCAGACTATGTCTGATGTGATAAaCGACTGACGCGAATGGAAATGCAAATTTTAACAAATATTGAaTCGCAAAaCTCTAGAGAAATAtATGACTGAAGCTTAGTCGCAGTAGATGAAAaTAGGTCAGGAAGGACGTAGCGATCTTTATTTTTATtATTtAtGAAtACCATTAACCTAATCTTCTGcAGCCTCAGTATTCTTAAGCTATTTTGATTTTtGTTTGAAAAATTTATTTTAGCGCCaCGC

>contig00345

ACTCAGAATCCTTTATAGACATTTTTTTTtAACTtAGACCTTTTtAAaCATTTGTCACCGTCCTCTGTCCTTTCGCGAATTtATATATTTGAAATAAaGTtGAAAAAaGACAtACAaGTATCGAAAGTTTAATAAATgTGACATTGAATTCTAAGAAAAaTGACGCAGTGAATTGAACGTTATAAACTGATATtATAGCTTACAAGTGTGGCGCATTGTTATGCATTTTTGTTGCACCTTATACAGAATGATAATAATGGTAAACATTATGTCACCAGCGCCAGAtAT

>contig00346

AAAAAAtATAATTCATTTtGATAACTTGGACAATAGTTTTTATATAACACGTTATTCATTACGAGTTTCAATCACGATAAAaTAAaTTTaCGTAAATTCTCGTGATAAGAATTTTCActGAGAACATTGTTCAAaTTTTATAAACCGCAAAATCGTAATTTCATTAATCTTTACTGTAAaTTATTTCAAGTTtAAATTTTAGATTCGTTAAGCTGTTTTCACAATCATGATTCAATTGAAAAAAAATTACTTTAGCCATAAACAGTTTTTATGACCTCCTGGTGAACAAATAGATATGAAAAGGTTAAAACGCTGGTTGAATTATTCGAATGATTCAGATTTCATGAAGTTTTTGAGCCGTGAATTACAAAGAAATTCAATTGGCAGAGTGTGGGAATGAGTTCAGAGTTG

>contig00347

ACGAATACTTTCAAGATGATGCGAGAAATGTGTCTGGAaCTCGACATCCCTGGGTCCTGTTTAACAGAACAGGGAGACAATTAGCTCTAGACCTTTTGCACTAAATATTATATTAGAATTATTTTGCGCGCTTTGTTTGCCTTTTTTCTTTCAAAATATATGACAACTAATCCCCAAACTTTTGAAAGAACTGAGGCAAATAAGATTAAGAAATAATATTATTTATTTGTAAAAATGTTTtAAATTTAGTGAATCTATAAGTAGGACAATTTTTAAAATCAAAAAACGATACTGAAATGTGGATGTGAAACTTTTCGAAAATGCTTTACTTTCGAATTACAAGCAGaTGTTTCTAAACCGTCGCTATTAATTGATCGACTACAATTAAGGTAAATTCTGAAAAAaGCATTTTACAAAAAGTTTGCAGACAATTTTtGATTTTaTATTTCTTTTAAGGAATCCTACTTTGTAACAAATGAATCGTTAACGATTTATGCAGATATATATCTtACGTTGCTGAAC

>contig00348

AGAGTCCGTAATTACTTCGTGTGAACGCCAATGAATTGATATTTATTTAATAGATAAAATGTCATTTCTTGTAACGAATATACAAACGGCAGGTAGATTATGGCGTAATGGTGTAATTAAGCCGGTTATATCTTCTAGAGGAATTTTCAGTTCTTCCATTTGTTTCCAAGAGGAGTCAGAAAAGTCAAAAACCGTAAATGATAACGTCAATAATATCAACCCTGATGTTAATCAAACCAGTTATGTAAAGAAAGACAGATCTGTAAAAATTCCAGTCGAAACGAGCATTAAATACATGGAAAGCGAAGCTTACAAAATAGCTTATGGAGATACACCTGTTTGGAAATTGTATAGGCGAAACTTTAAAGGTCAATTTGTGCCAACAAAAACTAGAAAAACATGTATTAGAGCAGGTGTAGTTtCAACTGGAAACCCgTGTCCGATTTGTCGAGACGAaTACCTGGTTTTGGACTACaGAAATACTAAATTAGTAAACCAaTTTATTTCGAAATTTAACGGAGAAATATTGAATTGCAAGGTCACCCATATTTGTCAAAGaCaGT

>contig00349

TAAAGGGGCATCAGAAGCGTTACAATtAATTTGTCTAGGGTtCTTtGTTAGATCAAATTGGATTCTTCTTaCAAAAAaCTGTGCTGATGAGTTGGAaGCGTATGGTGATACTATAATTAAATCTGCAAGTGACGATATTCAAAAAaCTTtGTCAAAAGGATCGAAAATAAAAAaTTTtCAAATAATTGAAGCGCAGTCTAAATTCAGCATTGCATACTTGGAAAaCACTATTTCCGAATCATCGtCATCAAAaTTGGATTATGTGGAGCGCCCTTCATTAGGAGAGAAGGAATTTTTTTATGGAACATTGGTAGGATGGAATGAGCAGAAAAAaCATCCAGAGACAATGAATGCTAAAATAAGTGTTAGcaGAaTTTatATATCGCTGGATAAaCaCCTaGAAaaCGT

>contig00350

GaGTACATGAAAAGCAACCAAACGATTTACACTAAGCAATTTAATAAAACGAACGAATACGAAACATCCAACATGCCTGCAATTGGAATTGATTTGGGAACCACCTACTCCTGCGTGGGAGTCTGGCAGCAGGGCAAAGTGGAGATCATCGCCAACGACCAGGGCAACAGAACAACACCCAGCTATGTTGCCTTCACAGACACAGAACGTCTCATCGGCGATGCTGCTAAAAACCAGGTGGCGATGAACCCGGCCAACACCGTGTTCGACGCCAAAAGGTTAATTGGTCGAAAGTTTGACGACCCCAAGATCCAGAGCGACCTGAAACACTGGCCGTTCAAAGTCGTCAATGAAGGAGGTAAGCCAAAGATCCAGGTTGAGTTCCGCGGTGAAGTAAAGAGGTTCAACCCAGAGGAGATCAGTTCGATGGTCCTGACGAAGATGAAGGAAaCAGCAGAGGCGTACCTCggtACAAACTGTtCGAGATGCAGTAATCACAGTTCCGGCCTATTTCAACGACTCACAGCGTCAGGCCACCAAAGATGCTGGAGCAATCGCCGGAATAAATGTTCTCAGGATCATCAATGAACCCACAGCCGCGGCCCTTGCCTACGGTCTTGACAAAAACCTCAAAGGAGAGAAGAACGTCCTTATTTTCGACCTTGGCGGCGGAACCTTCGACGTTTCCATCCTTTCCATCGACGAAGGCTCGCTCTTCGAAGTGAAGTCAACAGCTGGAGATACTCATCTGGGTGGCGAGGATTTCGACTCAAGATTGGTAGACCATCTGTGCAAGGAATTCGAGAGGAAATACAGAAAGGACCTGCGACAGAACCCGAGGAGTCTTCGACGCCTTAGAACAGCAGCTGAAAGAGCGAAACGAACCCTGTCTTCAAGCACCGAAGCCACCATCGAAATCGATGCTCTATATGAAGGCATAGACTTCTACACCAAAGTCTCGAGGGCTCGTTTCGAAGAATTATGCGCAGACCTATTCAGGGCGACCTTGCAACCTGTGGAGAAAGCCTTATCCGACGCCAAAATCGACAAAAGATCCATCGATGAAGTCGTCCTCGTTGGAGGTTCAACCAGAATTCCGAAGATTCAGAATATGCTACAGAACTTTTTCTGTGGTAAACAGTTGAACCTTTCCATCAATCCCGACGAGGCCGTGGCCTATGGAGCGGCAGT

>contig00351

TCAGACACGGTTGTGgAACCTTACAACGCCACTCTTtCGGTTCaTCAGCTCGTAGAAAaCACAGACGAAACcTATTGTATTG

>contig00352

aCGACATTTGCTTCCGCACACTCAAGCTCTCCACACCTACATACGGTGACCTCAATCATCTTGTATCTCTCACAATGTCCGGTGTCACCACTTGCCTTAGGTTCCCAGGTCAGCTTAACGCTGACTTGAGGAAACTTGCAGTAAACATGGTtCCCTTCCCACGACTCCACTTCTTTATGCCAGGATTTGCTCCATTGACCTCTCGTGGAAGCCAGCAATACAGAGCCCTTTCGGTCCCAGAGCTTACCCAACAGATGTTCGACGCCAAGAACATGATGGCTGCTTGCGATCCAAGACACGGAAGATACCTCACGGTTGCAGCTATCTTCCGAGGAAGGATgTCGaTGAAaGaGGTCGATGAGCAAATGCTCAACATTCAAAACAAGAACAGCTCGTACTTTGTTGAATGGATACCTAACAaCGTAAAGACAGCCGTCTGTGACATCCCACCCCGAGGACTTAAGATGTCCGCTACCTTCATCGGAAACTCCACCGCTATCCAGGAGTTGTTCAAGCGTATCTCTGAGCAATTCACTGCCATGTTCAGGAGAAAGGCTTTCTTGCATTGGT

>contig00353

aCACTGGAGAGGGTATGGACGAGATGGAATTCACCGAAGCTGAATCCAACATGAATGACTTGGTTtCTGAATACCAACAATACCAGGAAGCTaCCGCCGATGAAGATGcAGAATTTG

>contig00354

ACAGAGCGCTACGTCTCGACACAGGAAATTTCTCTTGGGGATCAGAGGGCCGTGCCCGCAAAACTCGTATCATCGATGTTGTCTACAACGCTTCTAATAACGAATTGGTGAGAACCAAAaCTCTCGTCAAGAATGCAATCATTACGATTGATGCGACGCCATTTAGACAGTGGTATGAAGCCCACTATGCAATTCCCCTTGGCCGAAAaCGAGGCGCAAAACTGTCGGAAGCTGAGGAAGAAATTTtGAACAAGAAGAGGTCGAAGAAATGCGAGGCCAAaTATAAGGCGAGACAGAAGTTTGCGAAAGTTGAACcAGCTCTCGAGGATCAATTTTCTACCGGAAGAGTTCTTGCGTGCGTTTCCAGTAGACCcGGACAGTGCGGTAGAGCCGATGGCTACATTCTGGAAGGTAAAGAACTTGAATTCTACATGAGAAAGATTAAGAGCAAGAAGGCGAAGTAAATGTAATCGTCGAAGGTGAATAAACTTAT

>contig00355

AtAGACCAAAaCAtGCATTCCCGAAaTtATTATACGAGGCAAGGCCATGCTCAGGCTATGGGACAACAGCAACAAACTTCGGTGCCCACAGGgAAaGAATTGCCTCCTGCACATCAAGCATCCTTACCACAGCCGCAAGTTGCTGCGAGAGCAAATCCTGTATATCAGCCTCCTGGATATACAAGCGTAACACCACAGCAaCGTGCAACAGGAGCAGTTTATCCCTATCCATATCCACCGAAAGTGCTACCGATGCAATCATCCTTGCAAATGCAACAACCAAATCAATATCCAGTGCATCCAGATGTAAAaTTtAAAAaGTTGCCGTTCTTTGATCTTCTCGGAGAATTATTAAAaCCaTCTAGTTTGACGCCGCAaGGGacGaTgaGGATGCAAGAG

>contig00356

ACAAATGCgTTTTTGCTTGcAAGAAACATCTTGTGAACAAGAAGATTATTTtCCTCCTAGCATAGCTGTAAAAGTAAATGGAAAAATaTGCCCCTTACcGAATCCAATACCCAcAAATAAACCAGGAGTAGAACCAAAAAGACCaCCAAGGCCGGTTAATATTAGCCCATtAGTAAAaTTaTCTCCcACTGTAGgTAaTCAAaTTCACGTCACATGGTCTGCAGATTACGGAAGGAGATATGCAATTGCCGTTTACCTCGTGAaGAAGTTGTCCaGCTCGGAaTTACTGACGAGGCTGAAAAATCGAGGTgTCAGACATTCTGATTACACCAGAGGATTAATCAAaGAAAaGTTGAACGAaGATGCGGaTAGTGAAATCGCTACAACATCTTTGAGAGTGTcGTTAGCCTgTCCTTTaGGgAAAATGAGAATGAGCACGCCTTGTCGTTCTTCTACAtGTTCCCATTTACAAtGCTTTGACGCCTCtCtGTTCCTTTtAaTgAACGAaCGCAAACCAACCTGGAGTTGTCCTGTCTGCGACAAGGCGGCGTTATACGaTAaTCTCGTAATTGATGGAtACTTTCAAgAAGTTCTTCATTCTAACAAACTCTTGTCAGATGTTAACGAGATTCAATTACTTCAAGACGGTTCTTGGGAAAACTTAGT

>contig00357

ACTTGCTAATCGGAGAACAAGATACATTAAGAATGACCCGTGGAAACCAACGTGAACTAGCCCGCGCAAAAAACATGAAAAAGAATCAAAAAAaGGCTGCTGCCGAACAAGAAAGTAATAAAGGTTTAACCCTAGAACAACGAAAAGCTCGAGATGCAGAGCGAATGAGAGAAAAACAGCTGAAAAAACAACAGGATGAAGGAATGAAGCCAGTAGCGACATAATTATATATAATTGCAGAAACGCCTGACGAAATCAACACAAACCTAACTTGAAAGATTAAACGTTATTATCACCAGCACTGTTATAATGAATCAAACATTAGTAAATTTCATAAATACTTGT

>contig00358

TTTGGTGACaGTCTT

>contig00359

TTGCAAAAGTTCAtGCGATAAaCTCCATCTCTAATGAAATATCACGATCCAAGGAAGTTCCTTCCATCAGAGAACTTTTTTCTAGTAATGAAGAATCTAAAGATTTCCCACGTCTGAATGAAATTCCTTTTCCTAAACAAGTTTCTGGTCCTAAGAGTTTTTCCCGTCGCAAGCAAGTGTCTCAAACTAAAGAAATGGTTCGTTCCAAGAAAAAAaTTTCGAAGCCACACGTCTGCCAAACTTGTGGTGTTAGATATGCAGAAGCTCGCTCATTGGCTGGTCACTCTTTACGGGAACATGGAATTTTAACCGAATTACATGAACGATGGTTAGCACAACAATCAAATCCCAATGATTACACCGTTGAAGATCTACTTAAAaGACATTCCTGTTATTCTTGTGGTAAAAGATACAGTTGCATGAAAAGTTTGCGATTGC

>contig00360

GAACCACTgAGTGACTTGAAACGTATAAAGCAGTGTGAAAACTAACGCAAATAATTCATTAAAAATTTCTAAAAGTTGTTAAAATGTATCGAGTTTTTGTTTTtGTTGTGGTCATCTGGATCACTTCAATACAACAATCACATGGTGCAACTACTACACTGAAAGTTGAAAATGTAAAATTCATGGGTCTTATAAGAAAGACAACAACTGTTTCAGAAGTTCCTCAGCTCCAAACAGTaGTAAAGAACATTTGCAATGCATTTTTGATATCGCCGAAACTTGTTGCATCTTTAGGCTCTTGTACAAaTAAAATGGAAAAAGAGTGTGGGGAATCTTATGAAAATTGTAAATTTTtAAATGGAAGGAAAGAACGAGATGATTTTAAAGCAACCTCGTATGATATTGATCATGTAAAAAaCTTTGCGAAATATAAAAAGCCAAAaGACGCCAATATTGACTTTGGACTTATCATACTCAAGCTATTAGAGGAATCACTAACACCAAAATTTTTTCTTCCAATACTAACTGAAAAATTTCAGAGAACAAGAACATTTACTTTATGCGGATTTGAATATTTTGGAGATAAATTTCATGATAAAGAAAATGGATTATGGAGCATGGATATTAAACTCAATGCTAATGAGCAGTGTGAGAACTCATTTCCAGGTTATAGCTTTGATAAGGAATCGGAAGCGTGCGGAGGCTCTAAAAAAAaCTTGATTCATGAATTTACTGAAGATGACAATGGTATTCCTGCGCTTTATAGAAATCAGCTTCAGGGAATCGTTAAACGTGt

>contig00361

AAAAATGGCTGCGTGGAGCAGTATTCTTCGTTCTACAGGACAAAAAGT

>contig00362

TACtATTTaCTTCAGAATTTCAAGAaGACATATGATCGAACTTGCAGAAAAAATACCTGGACCTACtGGACTTCCTCTTCTTGGAAACGCCCTCGAATTTATGGGAA

>contig00363

GCtGGATCCAGTTTCTTCCTGTCCATGAtGGGTTGCCACCCAGAAATCCAAGAGAAAGTTATCCAGGAACTCGACGAAATCTTTGGAGACAGTGACAGACCTGCCACCTTCCAGGATACTTTGGAGATGAaGT

>contig00364

ACCATATATTCTTCACAGTGGTTTTCGCTGTGCTAGGTATTGTTATTACAGAAGAAGAAGAAGCAGAATCCGgAGGTGCACGATTGCTAATTGCGAAGCAAATTCACAACAAATATCTTGTTGAGAATATGGATGTTATCGTTAAG

>contig00365

TGGgATGAAAATTAAATTTTATAATAATAATATTAATCTAATTAAAAAATCAATTATAAaTTTaCCAaCCCcATTAAATATTAaTATTAtATGgAaTTTtGGTTCAATTTTAGGAATAATTTTAATAATTCAAATtATTTCAGGATTCTTTCTTTCAATACATTAcACTCCCCATATTGAaTtAGCATTCAATAGGgTTATTCATATAATACATGATATtAATTACGGATGAATCATACGATTAaTTCAT

>contig00366

CCACTTTAAATCGATTTTATTCCCTTCACTTCATTTTACCCTTAATTATTACTTTAATTGTAATAATTCACTTAATAACTCTACATAAAACTGGATCAAATAACCCAATTGGTCTAAATAGAAATTTTTATAAAATTCCATTTCATATTTATTTTACAATTAAAGATATCATAGGATTTTTAATTATAATTTTTATACTATTAATTATATGTTTATTATACCCTTATATTATAAGTGACCCAGAAAATTTTAACATTTCAAACCCAATAATTACCCCAA

>contig00367

AAATTGTTTATTAAAAAGTTCTTAAAGCATTAACAATGTTTCGTATCTTGCTGGCTACGCTAGTTGTCTGGAACGCTTTAGGAGATGAATCCAGTGTGAAGGAACCGAATCAATTAAACGGCAAGTTGTGGGTATTAGATACTGCAAAAAATTTTCTCGGACTTGGAAGCAAGTTTTCTCCGGAGCGGTCCTTAGCTTATTTGATGTATAAAGAGAAAATTGTATCAGGTGCAGCCATAATTCATGAGCTTTACGCTTTATCTATTGGTAAACCCCTAGAACATTTTGAAACTAACCCCAAAAAAaTCGAGGAGCTCAAGATTGGAATGGGAGCAGTAACGTTTGATCCAAAAAaTGATAATGTTCATCACAATGTAAAAAAaGTTGATATTGGTAAAATCCCTGCCGAGAGCCCAGAAAGGTGGCTTGCAATCATAGCCTTCGAACCCAAGGatGGTAAaTTACCAGAAGAGGTAAaTATTATCGATTTGCCAACGGTCAGGtATTTCGAGTATTCTGAACACAGAGAAGAAGAGTTTtCAGTTGGATGGGCCTGGGAGAATAATGAAGTCGTCATGGTAAAAAGT

>contig00368

ACTGATGTAAAAaaTAGATAAGGACATACAACTGGATGAAATATACTTCGCTGGGTTCTTAACTTACAAAAaGCAAATTGCAGGAATGATTATTGGCTTCAAGCAAGTGTCTGAGAAAGATACAATAATTAATGTGAATTaTGCTGATATTTTGGaTTaTTGTTTaTTCATATTGCAAACAACTAGTGATATAGAaCTAAAaTGTGGaCCACATATTCCGAATTCTTCTAGAATAAAaGATaTGCATGATTATTCTTTCTCCACTACACGAGGCGAAATATCATTACATCGTAAAAATAATGCTGGCAAAATGGAAAAAGTTATTAATCTAAATTAACACAGGGAATTTTTATTTTCCCCTTAAGGTCATATTACTAAAAATCGAAGAATAGAAAAT

>contig00369

ATTAAATTGGTGTATACAAATAGTTCATTtAAATAAAATAACAACGACATTTCG

>contig00370

CAATTTtGTAAGCATCGTCACACTGGATTTGGTTGTTGCTTAATGTTGACTTATAAATGAAaCTAAATAAGTACCTAATCACATGATTCCATTATAGCTCGGATTTATCACTAGTTTCTGATTCTTCCTCGGCTTCCTCTTCTTTCTGCTCTTCCTTTTCTTCTCCTTCTTTCTCTTCGTCTTCCTTCTTTACTTCCTCCTCTTCTTCTTCTTCAATGTAATCTACATCATCTACACAGTATTTTGCTGTTTCTACACAAAGTATTTCACGGATTTTCGGATTTCCACTTTGGACAAGAGAAATAATGGGCTCTTCAAATTCATCGACTATGGATTGACACAAGAATTTCAAGCTCTTGTTGAGATCTCCGTCCTGGATAATGTCGACTCTACTTATCAATGGGTTCAATCCACCAAAAGCATTCATGAGATTCAAGAGTATTAAACGTCCTGACTTCTTGAACGTAGATTTCGCATAATTATCCAGCTCCTTACAGATCGTATCCAAAGTATCAGAAATATGAATCTCCGATTTCGTCAATGGGACCTTCTTGTGTACAGTATTTCCTTGGGCATCTAGACGATATTGGCCTACTTCCAGTTCCTTTTTAGGGTCAAGTTTTTGTAAATTTTTCATCAGTTCATCTACAGTTCTTCTGCATACCAAGCATCGTAGTTCCTTTGGATCAACGTCAGCTGCTGCGAAGGCAGCAAATAGGAATACACTTAATAATATTGAAATTTCCATAGTTTTCGAATTCTGGAACGTGAGACAaGGT

>contig00371

ACATCGAGTATGAGTATAGTCCCTCTGTATTTTAATAAATTGTGTAGAACGAAATTGTGTAGATAATTTCTATAACACTTTGCAGTATGAGATTAGAGTTCTGTATAAAAATTTGCAGTTAGGCCTAACTATTTTTTCTGGTAAAGGACATGTGATATCAAAGACGCTACGAATAAAaTATAGCGGAATTAATATACAATCACAGTATAATGGTAGTTTAATGACATGCATAGCACTAAATCAACTGTTGGACTTTTTACACTTTAATCAAGCGTCCTGCACATGGGTTTTAAACAAGAAATGATTCAGTGAACTTTCAGTAGAGAAATTTTTtCAATTTGtATTTTTAATTTTTTTttATGTAATATCGGTTTTACGCGTTCTATTAATGTTCAGTTGGTGGTTTTACCAGCAGCGACAACGTGTATTGACGTTCAGAAGCCAGGTCGAGGACCTGGTGGAAGAGAAGTCCCTCAAGCGTTATTGCTGCGCAATTCAGGTGTCCTGCAACAa

>contig00372

ATATAATAACTCAATaTAATTAAAAATGCACTCAAGTTTTTGCATTATTTtCAAAATATAATAAATAAATTTtGATGAaGTATATTAtAAAAGTTAGAACTTTTTTCGTAACATGGGATCAAAAaTAtAAGTTTTTAAAATTTATTTGAAAaTTAAGTTTtATTCCAAGATCCAATTAGAGTAATTCTTCCTTCAGTACCATAAGTGATTGGGCTCTTCATTTTAATAAAGGCCAACAGAACATCTAAATCTATGGGTCCGGTTTTGTAATTACGCTTTTTTtCTTTTATTATAGTATGATTATCACCTCCATTTGTTAAAAAAGATGGTACCACAACTTTGTACCATTCATCTGGAATGATATCATCAAACTTTGGTATCGGACAGGCTTGGCATCTAACTTCTGCTTTTACGACTCTAGAGTTATCTGGATTAGACAAGTTGTAAGTGACTTTGAGTCCAGAAAATTGGAGGAAATTTTTGCCCACAAATGTGTTTGGATAGTAACTTACTAGGACGGCTTTCTCAAGTGTCTGTAAAATATCACTTCCTTTTAATTCGATTAGATCAAAAGTGTTACTAAAAGGTGCAATTGCAACAACATCCTCATATCTGATAATTGGAGGATCATTACTGATATCACCTCTAATAGCACCTGCATTTATTATAGCAATTGCTGCACGGGTCCAATAATTCTTATTTTCATCTTTCCCAACGTAATAATCCACCATTGCATCGGCTATGAAATCCCCCAAATTGCACTCTCTGGATCGACATGTGTTTAATCCTTCAAGATAAACTTTAGTTGAACCAACTTCTCTTTTACCTAGTTTATCTGCTCTTTTCTTCCATGGCTGAAGATCTCTCAATATTTGTGGGTCCTGATCGAATAAATGATCCAAAAGAATTGGATCTCCATCCCAATCAACTATTTCATCTTCGTCATCGAACCAGGCAGTTAAATTCCCTATGt

>contig00373

AAAAAAaGGACAaTTTtAAaTtCTTAAAaTATTTTTCCAGAGTCTTCGAGTTAAAAATTGATTCTCCAAGCAAAGTATGACAGGACAAAGTTTTTATCCTTGTAGACATTTTACACCAACTTGATTCTTGTTGATCCTTTGAGCTTGGGGTTCTGAATTTTTtATAATCTCTAGCTGAATTTAAAATCTCGTTTGAGAATAAATTtACCACACGGAGCACTTTTtGCTAGGATTCATTTTGGAGCCCACTGGACACTTAAAGTCTTTTGCGAAATCTGGCATGTTGGAAAATGGACCCAGAACTCTGAATTGTCCAGGACTATGAACTCCAGTGGTGATGCGAAGTTTCAAAGTCTCTGGTCTGTATTTGCTACACCACGTGGTGGCAGCACTGATCCAGAAAAGTTGAGATGGAGAATAATCTAATCCTGGCAACTtGGgCTCAGGACCATTTTGTTTtACCCAATTTtGGTAGGCCAAATACGATtCTTTTATTCCTCCATTGTCTGCGATATTTTCTCCTtGTGTGTTTACACCATTTAGGTTTAATCCAACTTCTTCAGCTGTATAATTTCCATACTGGTGAATAATGCACTCAGCTTTCAAGAGAtATTTTTCTTTGGTCTCTGTCTCCCACCaTTCCACCAGATTACCATTTTCATCAAACTGACGACCTTGGTCATCAAAACCGTGAGTGATTTCGTGGCCAATAACGAATCCAATTGCACCATAaTTCaT

>contig00374

ACAAATCCCAaCGCTTCCGAGCTGGTAAGGGTAAGATGCGTAACCGTCGTCGCATCCAACGTCGTGGACCTTTGATCGTCTATGGACAAGATCAGGGAATTCGCCATGCCTtCCGTAaCATTCCTGGCGTAGATTTGATGAACATCAaTAAAATGAATCTGCTGAAACTGGCCCCTGGTGGGCATGTTGGACGTTTCGTTATCTGGACAAAGTCTGCTTTTGATCAGCTGGATGATCTTTACGGAACGTGGCGCAAGGAATCCAAACTTAAGCAGGGATACAATCTTCCCTTCCCCAAGATGGCCAACACTGATCTCTCCAGGCTTTTGAAGTCTGAAGAAATCCGTAAAGTTctACGAGCTCCTCAGAAGAAGGTCGTTCGTAGCGTGAAGAAACGTAACCcGATTAAGAACACCAGGGCGATGTTAAAACTAAaCCCCTaTGCAGCTGTTACGAAACGTGTGGCTATTCTTAAAGCCCGGAAACTACAGT

>contig00375

AGAATGTTCTAGACAGTTCTAGAAGATTGTGGAATACACTGTACTGAGCATACACACCTACAAATCAAAAATGTTCCATTGTATATTTGGGTTACTAAAACTTCCAATAATTCTTATAAAGTATTTTTTCTTTAATGAAGATGACTAATTCGCTAGTACATAAAAGTTTGGAAGAAGTAGTAAAAGAAGGAAGAGTAGAAAAAATGGAGAAAAATAGAGGAATTTTAGATATAATTCCAGCTAACCATAGGTTAAGCTCTAATCAAAATAAATCAGCCTGAAGTTCTTTTTAACATTGGCCATGGAAAATGCTGGAATCGACGTCAATTTCGCACCTATGTACCACCGGATGTATCAGGATATTTTGACAAAATTTTGTGGCCAGGGTTTTTGAAATACAAAGAGAAAATCAAGAGTAAAGACACATTTACGACAATTAAGTTTTTtAATGGAACCAAAaGCCAAGAGGAAGTGTATAGCATAATTTTGGACGAAGTTGAATCCATATTGAATTAGTCTAAATTAACCTCAATCAGTTTtAAaCTTtGGGtATATTAAAAATTCCAGATGAaTTTGGTTCGCATTTTTCATCGCAATCTTTTTCGATtAATgTTACAAAAGTTCTTCCCACAGTGTAATCTGTAGTATTTt

>contig00376

ACCGTCCGCACTTCGACGTGTGTAGAACTTACGGGGGATTATTGGGAGATCAATATTTCTAATGAAAAAAaCATTACGAGTCATTCATTTTCTGAACGCTTAATGTAAACATCATTCAAATACAGTGCTTAAC

>contig00377

AtGAAACTATATACAGGAAACCTTATGAGAAACTATATAGGATCCAAGCTCAAaTTACGATATAGATTCCCATATGGTTTCCTATGTAATTTTTAGATAGTTTCTATATaGTTTCTTATGATAACTTTGAAaTTTCTTATATAGTTTCTTaTagaaTTTTGTATATGgTTTCTTAGATGAGATTTTtCGATTtCTTTACACATTTTTttGAAAATTCACAaCAAAATTGCACTTGCTTTTTCtGAAATCAAACGTTTTAAATGTAATTAATGTTATTAATCCAGTAAGAaGGAAAGAATTGAAATTTTAAaTAAaTGTAATTCtCGTTTTTTGCACATTGGAATTCGGTCAAATTAGTCATCATTCAAATAAATTCATAATGACAGATTCATAAAAAaCAAAATGCAAATTTGGACTCTTACAAATTGAATTGAAATAAGAGCGCGTTAAATGAACTTTCATTGACAAATTGAAAAGTTATGCATTACTTGAAATTTAAAATAGCAAAAACTGCTTAAGACCATTCGATGTGATTTCAAAGAAGGGATAAAAAAAaCTATCCCAGACAGATTTTGTTTATACTGCGCTCATTAAAATTCTGCAACTACCTTTGAATTGTCAAAAAATTGAGTTTTTTGTATACCTCATTTATTAATTTTGAATCTTCTGTATTTTGAAAATATTTATCCAGGCTTTAATAATAAATTGACCTGGAAAAAATTACAAAGCATTAATTTTTTtCACTGATATAGGTGTAAAAAGTGCAAAAAATGGTTTGATAAATTAATTTATATTTGTCGTAAAATGAAATTCAATTCAATTCGTTCTTTCTTACTATACAAAGTAAGGAAAGCTATATTTATGAAATAAGTAAAATTATTAAATTACTTATTATTATGCTTTAAGTTATTATTCTTTGTTCATATTATCATTATTATTAGATATTAAGTATAACATAAGCAACAAATATTCTA

>contig00378

GCAACAAGAACCTACGATGATAAATGTGAGATTTACGGCCTCACATCTTCTTTGCAAATGCTATGCGGCTGCTAACAAAaTTACGGAATCG

>contig00379

CGCTGTGACGATACGA

>contig00380

ACAAAAGCGTGGTAAAaTTTGAAAaTTACGTAGCGAAATTTATGCAAGTATGAAAATTAAAGGAAAaTAGTGAAATCATTATTTtATAGATTTTATAATGTTATTGTAAATTGCACTAAAATTTTCTACGTCTGTTTTGTGATTAATTGAGGAATTTTCTTTTTGAATGTAAATCATTTCTAGAATTAATCTTTTTTtAAATTGTTTTCGATATCTAAAATTTTTGTATTTTtAAAATCAAAAGAATGCCTATAGATAGTAGCATGTTCTGCAAGTGTGGTTTTATTTTCTTTATTTTTAACTGATCTAGCATGTTCAAGCTGTTCAGAAGAATCCTCTCTAAAGGATGAGGATAATAGTCAGTGAATAAATAGAAATGTGAACTATTTCTCAAAAgAATTGCTGGTAaGTGgCGATaTTTaTaCATaTTTCTTTGgAAaTTTTATTaGATACTTTTT

>contig00381

ACAAGCAAAGGATCCACCATCGCACtACTTAGCGAAACTACGAACTTATTTAGATCCAAAaGCATCCAGAAGCCATCGA

>contig00382

ACCGAAGAtCGACGtCACCTTCGACAtGGACGCCAACGgCATCCTTCATGTAACAGCGAAGGATACAGCTAGTGGTCGTAGCAACAACATCCGCATTACGAACGACAAGGGTCGCCTCTCTCGAGAGGAGATCGATAGAATGCTGTCGGAGGCCGAAAAATACCGCGATCAAGATCGAGAACAGCGGGAGAAAGTCGAAATTCGCAACAAAATCGAGAATTACATCTTCTCGGTTAAACAAGCCGTCCAGGATTCTGGATCGAAACTCACTGAATCGGATAAGAACTCGGTTCTAGGCTTGTGCGAAGACACCGTCCGTTGGCTCGACAACAACACCCTCGCCGAGAAGGAAGAATACCAACACAAATTCGATGAAATCCAACAACAATGCGCTCCAATTATGACCAAAATCCATCAAGGAGGTGCTGCCCCTGGAGGCCCACAGGACTGCGGGAACCAATTCAGACAAGGCCAACAAGATTACTCTGGACCCACGGTGGAAGAGGTGGATTAAGAACAATAACATCAGTTCACTAATTAAGAACAATAACATCAGTTCACTAATTAAGAACAATAACATCAGTTCACTAATTAAGAACAATAACATCAGTTCTTTAATGAAGAACATTAACATCAGTTCTTCAAAAAATCCAGTATTTAAAAGAAATTCCTAAGACTGATAATTTATTTTGGTATTAAAGTTGTTTCAGACCAAACGATGATATAAAGTATTTAATATTTTTCATGTCAGGGCTGGATGTTTCATCGACCGGGTAATTTTATTTGATAATCTAAAAACGGGTGATTTGTGAATTACAATTGATTTTTCCAATCAGGTGTAATTCACAAATTGGAGTTTCAGATTGTTAGGTAAAGTTATTTTGGAAAAGGAAACACCTAGTCCTGTAATCAGTTTAGATGTCTTTCTGTAACTTTATCCAGATTTTTGCATTGTTCAACTTATAAGTTGTAATAATGTATTTTTtCTTGATGATGTTGTTATAGACAGGTGATTAAACTGGTTCTTTTGTGATATAACGTTCGTAAATTAATAA

>contig00383

ACTAAAAAaTAGCCTATTTTtAACACGATGTTtATTTTGGAATACAAATTAGATTGGGCTGAAGTTTCAGATGTCTAGTCAGAGGAATTTTGAAATATATTTTtCCCCACTAGAAACAATTAAACTAATTTGAAAAaGAAAAaTTAAAAaCtATTTTTTTCCTAATTTTAAAATTGTTTATTGTTTACAGAGTCTTGAAAACTATTAGTTTCGTGTTTTCGAACGTTTTTGAATTGTCCCTAGTGAAAAAAAAATTCAAAATTCGTCTGACTAGACATCTAAAAATTCAGACCATCAAATTAGAATAAAATCGGGCTTTTtGTACATGAAAAGTTTTTAATTTATTTCAGAAAATGGCCCATTATTTACAACAAAAATTGTTTTTAAAAAaGGAACATATTATTAAAGATAATTATGTGATTAGTAGACTATCCCCTCAAGTATAAAGAATAATAAGCTTTCATCACATTTCAAATGTTGAATTTGAGTCCTCAAATTTGATATTAATATACGATTGGGTAACATCCAAATAGCTGATTACAACAGTATGGATAAGTATAAGACGAGTTTCCCAGCGCTATCACTGTCAGGTAATTGTCTGGGCATCGTCCACCTACTTCTTCGCACCCTAGAAGATTTTGAGAACCATCTGGTAAACAGGTAACTCTAACACATTCATTTGCTCTTTGTGGATAATACCAGGATGCATAAAAAGGACAACCATCATCATACAAAGGAGATTCTTCCGAAAAAGACACCAGTAACAAAGCAAGAATCAGAACTCTGAATTTCATCATGATGAGTTATAAACCTTCTTCTAAATTCAAACAATTTatccAAGCTGAAAAATTGTTCGACATATATAGAACtcccc

>contig00384

ACTGTTTATTCGGGAGGTTGCTGTCGTCGAAGGTGATGCCGGAGGAGCTGAAGGACTGTGAGTTTGGAAAGCCGAATTATCAGTCGTTGTAATTTTACGTTCTTGGAGGGAATTCTGCTCCCTTTCTTCAGCCAATAAGGGTAATCCAAGACCCGCTCTTGCCCAGTTCACACCAGCTAGTTTTTCTCTGAGAACATCGGCCACTGGAGAAACTAAATTCACTTGTGGAAAAATTCGGGATTGGCATGATGGGCATGTGTATCCTGCAGGTGCTGTCGTAGCT

>contig00385

TCGTGTATTTAAATCTATATTATTTACTTTTtAACTTTAATGTAAAACACTTATCAAGTAATCAAACATTTTCAAATGATGTTTTTACTCTGCCAAGATATAAAACAATGAATATTGTTTGATTTTGTGTCATTTAATTGAAACAAATACCTAAATGTTTAAAAAGGTAGTGCATAAATATTGTAATACAAATTCTTAATCCATGAGATAaGTAAAAAAGCGTCAATAGACATAAGTTAAACTTAAAGAGAAGGGAGCCTATGAAAGAACTTTATCTTCCTGTATGGTTGGACTGGTTGCGGAAGATACTGCAGATGCACGTCTAGAAGCTTCTGCTTCTCTTAAAGCTTCGTCAAAACTCTGTGTGGAGTTGGATCTTGAAGGT

>contig00386

AAAcACTtGACTCTCTTTAACATTACGAAaTCCCTGGTTGACAtCGTCGGTTAATTCTTTCCAGACGCTGATACCCAACTTTCTTTTCAAATCATGTGAAACTTTAACTTTACTTGATAAAACGTGCCTTAGTGTTTGAATTTCCTCTTCAACCTTGGCAAGTTCTGCACTCCACTCTGCCTTCTGTCGTTCACGTTCTTCAACTGAAAGGCCTTGTAACTCATTCGTAAAGTCTTCTGGTGATGGTGACATAGACCCCCcTGAATGGAGAGAAGCGTCTTCTCCCGTTGATACAATACTCATTTCTCAATTTTTTtATTTGCTTCTTGTATTATTCCCAAAGAAGGTAAAAAAAGTGTAGCGATTTTTATTGGTTTCAAGGATTCCACTTTTCACAAAGGAAGACTAAATGTTTTAACCCATAAAATGTCACCTTATTACATGTATGCCTAAACACATGGAG

>contig00387

ACTAAATTTTCTAAAATTTTATCTTCATTTTACGACACTGTAACTGAATTACGGTAGAAATTTCTAACACTGTTCAATTCGCTATCTTATAAAGAAATGTAAGTTTTTATATATTTATTTAAGTTGAAAACATTATGACATTATATTTCAGAATTGAATCTCTTCCTTTTGAGCCAAGTTGACTTATTTAATTTCTTTCAAGCAAATATTTCaCAAACTGAGACAATCATTTCTtCATAAAAaCAAATATAATACATATTCGGAAtATTCTCTTGAATCCAAGAAAATTAAGATTtATTTTTCTATTTCTGAATCTTTGACTGTTTTCAAACCGATGTAAATTTTGTTCtCACGGGAGTTAGCATATTATTtATTTTGCTTTTTTTtATCGGCATtGATGAAAaTATCAGTTTTATCCACATGTTATTTGTTTAGAAGAATTCTATTTTTAAaCAAGAGTGTGCGTTTAATGAAGTTCATCTCATAACCCTGAATTTTAAaTTtATGATATGAAAAaTTATATCTAAATGTTTTTtGTTAGACTTGtAAaCTATAAACGGTATTAATTTATGAAATATTTGATTTTTAAGTTGTTTTTTTGATACGT

>contig00388

TGGTCtCTaCTATctCACAAGAGTCACGTGCCAAAAaTCTTtAATGCTATTACTATATATTtACTtGTATTATTATtATTATTATTGTTATTATCAATATGCTTATTTTTATtACATAAATCaTATCGTCTATGATTTATTACATAATGTATTTATATATATTTATATATCAATAACGAaTaCCGCCCATTACTACTAGAATTTCAAACGGGAGTTCTCTGATTAACAGGCCTGATCTTCATCCTTtATGTTTCCATCCAAAATAGGTGAtATATCATCGGATGCATCACAAGATATTCTTTCTATTCATTCACTCAAATAAATgTTCTTGATTCATATATCACCTTTCACTCTTCGCAGtAGTTTTtCTTCATGTAAGTTCCATTtATAGTCAGTTtGGACACATAAACGGTGTATTTtACTtCTGTGTAaTATGG

>contig00389

GGgcTaTGTTTCCAGATGAAGCAAACGATTTCAAAGACTCTaTTATCAAAGGAATATCCAaGATAGAAAAAGAAATTCTATATAAAACGAAAAAGGACTACCACAAAAGTTACAGTGATATAAGAAAATTtACTGAAAAGGTTGACAGGTTGGgAAaTaTGGAAAATCtGATTCACAAaCAGATACAACAGCTGGAAGAAG

>contig00390

ACtCACAATATGCTTTTATCGTGTAGGtGTCGTTAAACGCGGACTGTTTGCTGAATCTGCTTTTCTGTTGCACTTTTCGATGATTACTCAACCAGTTCTTGTGTAATATTTGATGATACAAATGC

>contig00391

GATGTTAGATAATAATAGAAAAAatCATTTGAAAACATTTTtAGAATCCAGATCATGAAAACATATAAATCTGAAGTCAAGTCTATTAATGTTTATAGTAATTTTTCTTGAAATTCAATTAAGAATGTATAATTAAAGAACATTATAGAATAGAAGAGGATAACGAAGAAGAAACGCAGCGCCAGAATTTtCCTAGACTAATACTtACATCAGGTACGCGGTTGCGATTTCCGCACCGTAAAGATCGATCGAAGCAGACCAATGCCCTACTAATTAACTCTAATtAGTTTTAGGCAAACAAACTGT

>contig00392

CACAATTCATCATCTTGTCCAGGTAAGACAATAGGTTCAGTTtCCAT

>contig00393

AGATTtATCTTTTTTATCtCCAGCATCCGTGGATTTAGGTTCAtCATTTTCaGTTAACTTTtCGACtATTTtCTCACATTTATTGACTATATCATCATTTCTTATTGAATCAGCTGTATTTTGACCAGGTGTGCTAaTAACCTCCTTGTCGGGTTTCGGCGAACTTTCAAGATCTGATTTATTCTTCTCTtGATCGGTGGCTGTTTCACTCGCTGTAgCGTTCACAACATCCTGTAAATTATTtGCCTCACTTCCTAATTTTTCAAAATTTGCTTTGCTTTTTGAAATAGCTTCAGTCTCCATTACTTCCGAATTATCGGTGAGAAGATTATcgCCaCCgAATGGATCTTCAGCATCCATtGAATGATTCTCAATCTGAGAATCATCTTGCTTGtCTTGAGATATTTGT

>contig00394

ACTTAAAAGAACTTCAGTCCCGAGATAAAATCATCTGCAAAAAACTCGGCACTAAGCCTCTTAGCCTGAACAAAGAAATACCTTCTGAGGTAGATCTTGCAAAATTTAATGACTACTTAGAAGTTCAGGAGCGTGAGAAGGTTCGTTTGGATACCATGTTCAATGATTTGCGAAGATCcGTAATGAAATTAATGGCTGAATTAAATATATCACCATCATTGGACTTCGAAAGACTTGTATGCAACGATTATGATAATTTCGTATATACCGCCGACAACATGTCGAAATTAAAaGATATGAGAAATGATCTGAaGGCTCAGGTAGAAGATGCAAAGCAACAAGCTGCCGAGAAACGAGAGGAGCTAATAGCTCTTTGGAGTTACTTAGATGAACCAGAATaTTTCCGAGAAGCATTTTtACGTGAACACTCTGGTCATAGTGTTACAACCATAACTACTCTCAATACAGAAATCAAaCGTTGCAAGGACAAGAGGAGCCAGAACATAGGAAAG

>contig00395

TTTGGAAATTGTGCATGTTTAGCGAAGCCCAGAAAaGaTCCTTCAtACCTTTCTACTCACAAACTTaCACAGAAGATCTaCTTACGCTTCACGAACTAGAAGCAGAAAAaCTTAAAAaG

>contig00396

CAAATGACCCTGACCGTTTCCAT

>contig00397

GGAGAGGtATTTACCATCAATGGCGTGGCAG

>contig00398

ATTTTtAATACAAGATTTTTGCTCGAAAACGATAAAaGTTTAaCAATTTTTtAATTTTTACGTCTTTCAATGGAAATGAAGATTtAGTtcACTTTGTAAAATTCAATGCATGTaTTTGTATTCCTTGTTTTtAAaCtACGCTtGGAATTtATGAATCGCTTTTTAtCAAAGTCTTATGCTAAATTCAGTTCATTGTATCTCTTTTGTAAAAATtCAGTTTTGGCAAACCaCCCAATGACGGTGGAaGAAC

>contig00399

AAAAACGTTTGCCGTTGGTGCTGAATCGCTGATGTTGATAAGTTCAACGTCAAAAAGCAAAGTTGCACCACTAGGAATAACGTTGCCAACTTCTCGATTACCATATCCTAATTCTGGAGGTATGACCAATTTTCGTTTTTCTCCTACGCACATGTTCAATAACCcTTGATCCCAACCTTTGATGACTTGACCAACACCTAATTGGAACGTGAATGGTTGTTcTCTATCCAAGCTTGAGTCAAaTTTGGT

>contig00400

TCTACTaTcTAGGtGgaaTCACCGAAGAAgAAATCAGGAAATCTCCCTTCGTTGAACGTCTGATCAAAAaaaGGATATGAaGTATTGTTCCTGACTGAAGCTGTTGACGAACACGCAATTGTTACCATTCCTGAATTTGAGGGCAAGAGGTTCCACAATATTGCAAAGGAAGGATTCGTTTTGGACGAGAGTCCAGAAGCCCAGCAGAAGTTACAAGAGCTGAAATCGACTTtCGAACCCCTGACCAAATGGTTGGACGATGTCCTCAAGGATTACATCAGCAAAGCT

>contig00401

AGGGCGAAGAAGCCGATGAAGATAAAGAAGATGAAGAGCACGATGAGCTTTAAGTGCTTTAAAATAGACTGATGTAAGAATTACAAATGATGTCAAATTTAAAAAAaTGATGATTTAGAATGATCTCTAATTATTGTAAGAGACTTATTTTTCTTTTCCATAGGCTATACAATGTATAGTTATTTGTTGAATTTTTGAATTAGGTTTGCAACTAGTTGTTGCGAATGTTCAATCTTTCATATACAGTCATATTACAATGCTTtATACTCTTTGT

>contig00402

TtGACGACGCTATTGTTTTTGGCTATCAAACTGTTACCTTAGTCAACAGAATT

>contig00403

ACCGGAAAGACCACTGAGGAAAACCcAAAGGCCATCAAGTCTGGAGACGCGGCTATTGTAAaTCTTGTCCCCACAAAaCCTATGTGCGTTGAGGCCTTCCAGGAGTTCCCTCCATtGGGACGTTTTGCtGTTCGtGACATGCGTCAAACCGTGGCCGTCGGTGTTATCAAGGCCGTGAATTTCAAGGATGCCACGGGCAAAGTAACCAAGGCTGCCGAAAAAGCCCAGAAGAAAAaaTAACTAATTtCCGCGCGAACGACCTGCCGAATAGTGAACACCGGCGTATAgTAGTTAAAaTAGTTTGTCTTtCTTTAAAaTCCTTGACAAGGATGAATCGCATTTCACTACTCGCCTCGATGGgCCTGTTGGCATCATCGACGAAGGCGAGACGATGGACTTGgAGAAGAAATGCGAGTGAGAAAGAAaTAAGGAGGCTTGTGTTGCGCAGAGACAAAAGCGTTGAGCTTCCCAGTTTCTTtATCAGTCGGCTTTTCCATCTTtCAAAACGATTACTAAGACATTTTTtCCATCGAAAGGAGTTCCGTAGGAAaGATCGCGTGGAGGAAGGGCAAACTACGCAAGCTATTATtACGT

>contig00404

CACTTTTtACtGCTCAAACTCTTGAGACACTaTTGCATAaagTCGTGACTTTATTTtAAAGTTTTGGGATTTTTtCGAAaTAACATCCACCTATCTCGACAACCTAaTTTAGCTCGGTTtAATTATATTTTATAaTATTATACGAAAaTATAATTAAaCCGAGTTAAaTTAGCTTGTCGAAATGTCTGGAtGTTATTGCGaaaaaaGTCCCAAAaC

>contig00405

ACGTTCTTTTAAAGATGCGAGATACTACACCGTTCCAAAGAAGGAAGCGACTGAGTTTACGAAAGATTTGGTAAATAAAGTAGAAAACAGTATCCTTAAAGTAGCCGTTGAAAAAGCTTTGCTCTTTCTTGAAAAGAATGAGTTGCCCCCTCACTGGGACAAAGATTCTCTATTTGGAAATTCTGTCTCTAGCGGAAATAGTGATTCTGACGGTGACATTGATTCAGACAGTTCTGACGATGAGCCTCGTGAGGAAAAGGACCATTTCGTTGCCCAGCTTTATAAATTTATGGACGATCGCGGGACACCGATTAATAATTGTCCAATGATTGGTGACGAAGATATTGATCTCTACAGACTTTTTCGTGCGGTTTATAAACTTGGAGGCTACAATAGAGTGACGAATCAAAATCAAT

>contig00406

CGAACTTCAATGATCGTCAtGAtGAGATCAGGCGTCACTGGGGAGGTGGTCTTCTTGGTAGtAAATCTACTGCCAGAATAaCTAAACTCGAAAAGGCGAAAGCtAAGGAACTTGCCCAAAAGCAAGGTTAAACGTTTGGAGTTGaGTaT

>contig00407

GAGGCAGCCAGCCGTGTCGCCtACAATCTGAAAGTTGTCAaCAGTGGCAAGCATTtAGAAGTTTTCAGTAAATTAGGAAAAATCTCAAGGAACGTGtGTGAAAATGGCGGTATTGTTCAATTAAATCCCCTCAATCTTTGATGAGTGCAAGATTTttCTtCACGGACTAGAAAGATTGTAAAATATTAAGAAGAAaCATAAAaTAATAGGTATTACCAATAGTAATAGTAGTATTGAAGATATtGgCCAGAAAGGACAATACCTTCAATACTACTATTAaGAAGCAACTGATACATCAATATgAAAAGAGATGAACCT

>contig00408

ACGGCCCtGGCGTTAGGTTTATCTCCGATTTTCCCAAGATTCATAGACTGTTGTTGCTGCTGGTGGCCAACTTGAATACTCTGATCTGTCGTAGGACTTTtCCTTGTGATTAaGgAAGCAGTTGGAGTTGTTGATCGAGACCCTCCGCTTATCAAATCTAAGACACTTGGCTGTTTGTTCATTGGTCCGCGTGGATCTCTGGTAGGCATTCCCATtACAGGACCCATTGCGCCCATAGCCATACCGTAGGGTGCTGCGTATTGACCTCCCATTGGACCTATTGGACCCATTtGACCCAtGATTGGATGACCGTAGCCGGGTTGGGGTTGATACGATTGTTGACCCATTGTTGCCTGCACTGTCATCTGtACAATCGCTGGATCATTCGGTATGGTGCTAACATTTTTAACGACTTGGATATCCTTTATGTCGGTTCCACGGAATAaGATGTATTCGTAGACCTGGTTTtGAGGTGAAaCAGGAAaTTGTGTTTCCCGGTCcTCCGTGCCAAACGACCTCAC

>contig00409

TTTtATttAATCAGCATTTCAACGTCATTCAAATTTCGCTCTTCTCTCTATTGCATGTTCATAAATTGAGTGTCGCGAAGCGTCAAAGTTGCAAACTTGATTTGAAAAAATTGATAAGTTGAAATTAATAATGTTGATTGAAATAATAAAAATATTTATCATAAATATCCTAATATCAGGACTTGAAGCTCGGACGCAAAAAGAACCATTTTGTGACGATGGAAAGACTAATTTAATCCTAGACTTGCCTGATTCTTCAGATGAGCATAAATGCAACGGAGAAGTGATACATCCTAATCATAACATAGATGCTTATTCTTATTGTTTAGATAAATTACCAATTCATGAGGTTAAACACCATTGTATGAATGAAAGTATTACATACAAATATCCCGATCCTTTTTGGGGAGATAGCCGAATGGTCTGGCCCGCATATGGTGAATACCGTTACTGCCCAAGACAAAGATGGGTTCATGCTTTGGAAAACGGAGCCATTGTAGTGGTCCGTCACCCTTGCGCACATGATAAGGAAGTAGAAAAATTGAAAAAaTAGTCAAAAAaTGCCTCAGGCGGCATATAATTACCGTTGTTAATTACCATAATACTGATAGGCCCCTGGAGTTATTGAGTTGGTTATGTCGTTTGAGAATGtCTTTTGTTGATGACGAAAGAGTTCAACAATTCATCGTTGACCATGCTCTTAAAGCCCCTAATCAAATCGCAGAAAATGGCCATTTTGATGACGGTCTTATTGAGCGTTCAAAAATAGTCATAGGCTCAGACTTCTATGATTCCAATTTATGCCCCGAATAAGAACGTTAAAAACATTTATGACAGGATGATGTAATCTTCAAATTGATAAACCCTATCTAGGAAATCATATagAAAACTatagT

>contig00410

ActattGGGgAcATTACTGACCAGGACGTCCGACGTCGATGGGTGCGGTCAGTTCCTTTACAAGCATGTAGGCTTGTGGCGTCATGTATCAAATACGTTACCATTTTTAGTAAAAATATAAATTTAATCAGTTTTTGAGGTGAGTGGAGCCGCTGTATGATTGTCTTCACAATAACGTAACTATTTAAAAATTCTAAGCAAGACAACCATCAGACTCTCATGGAAAGGATAGGGTGGACAAGCCTCTGAACCAGAAAGCATTCTAATATTTTCAAGATGTCGTAACCGTAAATGGACAGAATGTCCATAACCATACATTGCAAAAAATAATAATTTGGCAAAATTCAACACATCTTTGATTTGTTCTTGATTGAATTTTGAAACACATGCATATATACAGATTTATTCGACAACCTTGAAAATGTTGGTATTGGCATTTGGTGAATTATATTATGAAAAATGGAAAGAACTAGCTATGGTGTATGTCACGGAAATTATAGACTCACGACATAAGTGGAGATTGCTATCCTGACTAAGAGCAAAACCCTCGGAAAaGATTTCCTCTATTGCGTGCTCCATTACATCCTTTAACTTTTTCACAATCACTTTGTAACCGTATGCTTTTAAAAaTGCGTGTGTTTGTTGAAATTTCATTCCTCATTTTAATTAATGTTTGAAATTACTTCTCGTATATAGAGATCACTGGGCTTTtCTTAGCCTTTATTTATTCTTCAGTATTGACTGATAACTATAGAGTTCTTCTATTTGTCCTGAGGGTCAAAAaCTTTCAGGATAAGTAAATAGAATTGaCCTTTAAAaTAAGCGACAGCTAAAaGCGT

>contig00411

AGTATATTTTCTTCTCTGCCCTTGAACGTTCGGAGTATTTTGTTGGTAATCTTCGCGTTATCCCGTCCCCTAGTAACAACTGACAGATTTTCAAACTTCCGGCATAATGTCGAATCCAGTGAGTCCATCCTTGAAAAACCTACCAAAGGTAGCTGTAGACTTAAAGAGTGAATTGGAAGGCTTCGATCCCAGCTGCATGAAAAAAGCAGCCACTACGGAAAAAAaTGTCCTACCCTCTGCTGAAGACGTCGCCGTGGAAAAGACCCAACAAACCTTAATCGCTGGAATTGAAAAATTCAACCCTGCAAATTTGAAGCACACGACGACCCAAGAGAAAAATCCTCTTCCTGACAACGATGTTATCCAACAGGA

>contig00412

AACGAGAAATCAGAGGAGTGATTtGTGATTTTGCTACTTTtATATATCGCATAATATTTtATAATGTTtACCGATATGAAGCAGGCGAGCGTCCTGAcTtAATGTAATTTtATGGACTATAATTAAACTAGCATCTCAAGCTTTCTTTCGTGCGAATAaTAAAAATTGCAATTTGACTCTGTTCGACGATTTTACGCAAAATGTCAATGAGATTATAGGTTTCGAACAGTTTCCTAGAAaCGATAATAAGCGAGAAAAAAGaCACTCTAGATTCGATATACCTAGTTTATTTTtGCATCTCTCATGTAATTTGTCATAAAGATTGTTCGAaTTTCGTGCTGGTGCGTAAAaGATAaTTGTGCAaGT

>contig00413

GATTTCTTGCAATCGCTTTCGTATGAAGAGGATCAGTTTATAGTTTGTACAACATACCAATTGGAAATATACGTAAAAATAAATTTACAATAATCAGTTGTAAAAAaCAATAACGGTCATTTGATAGTCTTGTGTTGTTATTAATACACGCAGATAACAAAATGTTGCGATTAATTCTATTTTTtCTCTGCACTGCATCTTTGGCTGCCACTTTTCAAACGAATAATAACAAAAAGTTGAAAATTCCGAATGTCTTCAATCTGAGGAAGTTAAGAAAGATTTGGTATGTTTACGAAGTATATGAGGAACTTGGTGATTTTTTtATAGCCCCTGGAACCTGCAAGATTGTCAATTTTAAAAaCCTTATAAATAAATATACCTATAATATTTCTATTAGCCAAAATCGCAATGGAAAAGTCGAAGAAAAAGAAGGCACCGCTACACTGAGTGGATCAGCGGATGGGTTTCCTCGAATGTTGCTTATATTCAAACCAGAGACAATACAATATTCATTACTGTTTCTCGGTACAGACAGTGAAACCTATATCGTGTTTTCCTGCATGAAAATCAGCTTAGAAACCGaCAAAAAaGAGgTaCTTtGGGTTTTGACAAATAAGAAGGAGACTACAATTAAAAATGTACCTGTCAAAAAAGCCCACGAGGTTCTTGCAAAACTTGGAATCAGCATTGAAAAAaTGAAAgTGGTAGATCACAAAAaTTGTTtGCCAAACTCTGCAATGATAAAAGGCaCTAGAATGGATGAACGATTAAAAAaCGGAAaCCACGT

>contig00414

CAAGTGTAAAaTaCAGAAAaGTTTGTCCTCGAAGTTACAGAAACGTGGAATGACATCTAATAaCATACATCTATTGATTTACAATTTTTAACCACCACCATTAAATAATATTTCACATAATTCTTACTTAATAATAAACGCATGCTCCCAAGAtAACTTAGGCCTCGTTCTCCTAGGAGCATtAGTGTATTAACGGTATTCATATGTGCTCTTCATATaTATTACGATTAAAGCATTTCTCAAaCTCTTGAATATGATGGTAGAAGCTGAGGgATAGCACGAATAGAGGATTATACTTATTGTCCTGTAGACTCTGGAGTTAATAACCATTACTAGCAGCGAAAaTGAATCTATTAGTTTGAGTATCACCTTCTATCTCTAAATACTtGACAAAAaTGTCAACTTtCGAACA

>contig00415

CGATTTTCTCAGTCTCTCGTGAATTCACCGTTTACAGTTATTTATACACTGATACTGGATGAGACGAAAGATTCTTAACTACTGAATATAGATTTTACTCACTGAACGATCATTACGAAATTGTATCTAGCAATCGCATTTCGGTGTTAGTCTCATCCTCTTCTTCCTCCTCCTCCTCATCAAAGCCAAAGCAT

>contig00416

CAGTTTtGGAtCGCGTTGCGATGCGtGTGATGCAGATTtGCTTCTCGTAGTTGTAGTTtGCAGCTTtAaTtCCTCcATTGAaGCCTCTTCTTCTTCTACTTCTGCTTCTTCCTCTTCTATCTCCTCTTCGACTTCGGTGACGATGCTATTTTCTTGGCTCAGAGGATCTTGCATCACTATATCTCCATCCtCATTAGCCACAACCgCCATTTGCTGCTCCGTtCCTtGGTTATCTGCAAGCTGAATTACTTCCaGgACAACGTATTGCTGGCCATCGCTTCCTTCAACAGCCATCATGTCTTCTTCATCCTCTtcTTCCTCTTCTTCTTCATCATCGTCATAaCCTGTCaTGACTTCTACTCTTTGACCGTCGATGATTTGGATTTtCTTTTGtCTaCCGATTTtCAaGGCTTGCTGTTtCTCTTGCAAAGATGACTCTGGGTCATGT

>contig00417

ATAGTTTGTTGAAAGCATTTTGAAGTAATTGAAATGATTCGAATTTTGGTGGCTTCACTAATCGTCT

>contig00418

aCctcTAGGACTTGAGGGTATATTAGAAAAAGAGGGCTCAGAAATAGGTGTTACTTACTACGGATATAATAATAACTACATAGGTGGGGgTGTAATACACAATGATATACTTGTTGGAGTTGTTATAAACTGCTATACAAATAACGCTGGCTTTACTGCAGGAAATACCATGGCAATTAGCCCTGTGAAAAAGCAATCTATTGACGTGCTGCTATCGTAAAATAATGTGAAGTTGGAGTAGTTGGAAAATGGAAGAAAATTAATTACGATAAATGATTTCGAAGACTAAAGCACGAAAAAATGTTTTTCCACTCTTCAAATCTGAATAAGTGAAAAGTGAGCCTGTAATTCAACTTAGTAGACACTTCTACTAAATACTAATGAAAACGACTGATGATTAGTAAAAAAaTTTATTAAaGTTAATTAGtGGCTCACTTCTTACTATTTTAGATTTAAAGAATGAATTTtCAAAACTATG

>contig00419

ACAtGGGgAGTTGCTACTTGACACGTATTCAAGAAAGATGTTCAAACCAAATCCACGCAAAATGAAAATTTCCATAGCAGTCCTACTCTGCTTTTTTATACTTGCGGTTTGTGCTTCCGATTCTAATGGGACACCTAACAACCAACAAACTGAGAAGATAATATTTATCAATCCAAGAGAATTGGAAAAAAACTTGAACATTATAAAAATATGCTGAAGAGTCTTGTGGTTACTGCATATGATGAACACAATAATATTATATGTGGCGGGAGCATGGTCACGAAGAACACGGCAGCAATTCCTAAACATTTTTGGAATGAACACAAAATCGTGAAAATTCTACTACTCGAGAATCTCGAAAGATTGGGTACTCATCAAGTATCGGATTTGTCGGATATGGTGCCCGAATTAGTTGCGGCTGAAATTAAAATAATATTTGTAAAAGACGTTGAGATTTATATACATGATGAATATTTAAAGGAATTATGGAAAACAGATTTGAAAGTTGGAACAAATGAATTGGCCcATGGAATAGAACTTAAACCGGATGGACCAACTAAACTAAAGGAATTTTTtGTTACTAAATTCGAAGGGAAGTCTATTATTGTTACTTTGCGTACTGGCTTTAACGAACCTAATCTGCAAGGTGAGCCCTTATATTACAAAAACAGTATTTTGGGATTATACAACAAAGCTATACCAGGGGGTACTGAAAAGATGTATGAATACATCTTGGTGAACAATGCTTCCCCAAAATATCCTTAGAAAAaCAAAATCTGGTTCCAGCAAAGAGACGATTGTAACTACCTTTCAGAGTGTGATAATGAATACATATTTTACATGAAAaTTCGAGTGAACATGAACCTTATTTTTTtCAATG

>contig00420

GCAATTTGCGCATTTTtAGTGATCATtCCCTGTTATGAACGACCCTATGAAGTTGGTATGGgTGCACTTATTACCTTGTCAGGTATTCCAGCCTATTTGTTCGGCGTATGTtGgAAAAaTAAaCCAGCTAAGTtCCAGGAAATTAAtACCAAAGTTACTCATACAATCCAGAaGTTATTCATGTCAGCGAAAGAAGAACATGATGATTGACGTAG

>contig00421

ACTAttGAAGAACTAACCAGCTATTGTGGCCCTTAAaTtGCATCACATGTCTTTtATTATGAAACATTTTTTtCTaCTTTCACTTCGAAACTTGATACGAGCTCATCCATTAAGACGAAAAAACAATTTATAAACTGGATTACCAATAAAGGAAAAAACATACGAAAAAGGGAATAATGGAACAATATAGTAAAATATTGATTATAATAAATAACATTATATTATAATAAATTTGAATATCGAGGTTTCCAAAGTTATTGATACTGAATAGTTTTCTATTGATTTATCCTCATTccAAAaCGGG

>contig00422

GTGAAACAAGTCTGTGGATTAAGCAAGGATTGACCCTGAAAAGGaTTATTGCGAGAGTTTAAAAAAaCCTAAACATAaTTCAAACTTGCAAaGCAATCAGTCGTTTACATCCAGAATAATAAAAAAAAcAAGAGAAAAa

>contig00423

GTGCGAGCAGtGTTTTTTTtAAATG

>contig00424

ACTTGTAATCTGGGTGTTCCTTCATGTGTAGAGCCCTTAATCTTTTTGCTTCGTCAATGAAAGGTCGTTTTTCACTTTCATTGAGAAGTTTCCACTCAGCACCTAGTCTCTTGGAAATTTCAGAATTATGCATTTTCGGATTTTCTTGTGCCATTTTTCGACGTTGCCCTCGGGACCAAaCCATGAAAGCGTTCATCGGCCTTTTGATGTGTTCATTGTGAAGTGATTTAGATGACATTTCGATAATCAAAATACTAACTAATTAACCGTtATTtCAATGTTATAGCACCCTTCAGGAAAACTTTGATAACCGtCTTTCTTCACCT

>contig00425

TTTTTTtAATCCACGAAAGAACTGTTTATCATTAAACACACGGAAACTCCTTTTcccATCGTTGCGGTATACTTCTTTATCAGCTTCATGGTGTCACCAAATACTTTtAGTTTTTTGtAACCTTGATTTCCAATGAAATGTTTTGCAAGAAATGTGGAATAATTTCCTGTTGAAATAAAAATTTGAAGAAATGTTGGACGTTGAAAGACGCGAACTTTGTTGATTATTTTTGGATAAATATTGGAATGTTAAGGACAAGCGAAACGCGTAGGACGTCACACTGACGTCGTCAGGGTGAAGT

>contig00426

ACCACGGAGAACATtGGAGAAGGCtCCTTtGAAGAAGGCTTtAGGACCTTCGCTCTTAGCAATAGTTTtCCAGCAGTGgaTTGTGCCTCTGTAGAGACGATCTTCCTGGGTTCTACCAGATtGCATCAcCATACGCCTACGCACAGTGTCGAAGGGATAAGAGACAATACCAGCtATGGTGGTGACAGTCTGGGCAATACCCCATGAAACGAGGAaTGGAGTCTTCTTTGGGTCAGGCAACATACCACGAGCGGTATCATAGAAGCCAAAGTAAGCTGCACGGTAGATAATGATACCCTGCACCGATACGCCGAATCCTTGGTAGAGTCCACCGATTCCATCAGCtctAAAGATCTTTGTCAAGCAATTACCTAA

>contig00427

TTGGgTGGGGAAGTATCTGATAACATTGGCGAAGTTtCCACGCCaGAAaCTGAGGAATCCTTGTTCTCTTGGGATACGGACGAAGCAATCGACCATACCCTTGTAACGCTGGTCTTCTGCGATTTGCTTGGAGATGTGTTGCACCTGGAGCAGAAGTTTGACTCGCTCAATTGGTGCTACGGCAGTTTTGGAGATCGCAGCAGAGATTCCACCGGCGAGGAAATCCTTGGCAAACGCCACGGGATCCGTAAGATTAGACATTGTGTGTGTTAAGAGGTGTGTATGTGTTAGGGAACGAGAGGATAGGAGATAATAGACTCCTGTATTGGACGCGCGCCGGACGACGAGTC

>contig00428

acAaTCCGTGGAGATTTTtGCATCGAAGTCGGACGTAAcATTATTCACGGTTCaGATTCTGTTGAATCTGCAAAGAAAGAAaTTAAacTTTGGTTCGGCGATGAAAAGAAGAAAGAAGTTATCGACTGGGCTTCcTGGGCAGAGAAATGGATCTACGAATAGGgTGATTAATTATTtaGCTACGCAGATCAACTaTTTTCATGTTCCAATTATACAGGAGTGCTTtATTAATGCATTCCaCTGTCAATTTCTtGACTAAAAATaTTACCCTTtGGATATTAGTAATTGTATATACAAATAAGCTGCCTCGTGGACTGCAACCTTCAGTTTAAAAAAaTAGAACTTACCTGCAAAaTGTATATCCAGCTTTtACTTGAATTACAAGTTTTATACTGTT

>contig00429

AATGGTCGCCGTAGCCGTCCGGTTAAGCATCGGTTAAAGTTTTTTCAAAAAAAAaCATTAATTTTTACGACCCCATTGTCTCAGTTAGTGTCGACGAAGTTGTTTTAGGAGGATACTTAAGCATTCACTGTCTGAGAAACGAAGATAAAAAaTGAGTTCCAGTTCTTGTTACAAATGCAACCGCATGGGTCACTTCGCCAGAGAATGTCCACAGGGAGGTGCAGGAGGTGGTGGCGGTCGAGGTGACAGACGTGACCGTGAT

>contig00430

GTCACAAGCCTGGTCACATAAGTCGCGAGTGCGACCAAGATGATaGGAAGTAGGAGATAAGTGGAATCCGTGCCGCTCGTTTAACGATAAGCCGTTTACCGTtGCGtGCACCACtACCAGGGATTGTAGCTaGGTAGGGATGTGCGGACATATGTCTCGTTGATGCGTATAATCGTTAAAAAAaaGATGCGTTTCGTCATAGTCGTTCGGATTCCCATATCTCCATGCCCAAATCCTGGAaCTCCTTCTCCCTTCTCCTCTACCCATCGTTGTTCTCTTTGCAAtGGTTTTTTTttCATACGATATCTAAGTTTTtATCGCTGTTACACCGGACCATAGaCCACGT

>contig00431

TtGCCtAAACTtCCAAGAGAACCtAAtGtGCTTAAtCcTTGCAACGAACTGAGTCCCTGAACACTGgAATTtGTAGCCCcAGGACTTGCCATTTTTCGAACATCAATGCAATCCTGAGTTTCCGAA

>contig00432

ACCTATCGtCTtCGtCTTTCAATAAAGATCTAAGTTGATCCTGTCGTTTtCGTAACTTTTCAGCTTTAAGGGTTTTCTCGAGATACGGCGATTTTCGGTTCAAGTTCCAGAAGTTGAAGTGCTTAGCGATTTCACTCTCACGTTCAAAGAACCTTGCTGTTTGCTTGTAGTAGTTGAATTTAAGATCAGCTTCGTGTCTTTTCTGAGCTGCTAACTGATATTGTTTCTCTTGGAACCCTATAGTTTTCCTATCTCGAGACGCTATACAGCACAATATACAACTGtAGTTTTTtCAAATACTCATTGCATTAAATTCACTGCATCTCCTTCCAATCTATTTGAAGAAAGTGCGTTCTCAATTTCTTGAAAATGCGCAATAATGCTGTTTTTATGCAACTACCTACTCAAAACAAGCACTCCCGTTCGATTTCCATTTAAGTTGT

>contig00433

TAAAATTGATAAAGGAGAGGAATATtCCCAAACTGATTCATTTGCACTGAaGTCACTAATCTATCCTGTAATACGTGATAATGATATTAAAGGTACTGGAATATTTGTGCAACTTAACTGCTTCTTAACAGTAACTTCCGTTTtGTATAATAAGAATAAAAAGAAGGGAACAACTGTTAAGGAAGACACGCTTGGAAATTTATTTATTCGTATCAGATACCATACAACCACTGTGCTAAAAGAAGGTAATATAGATGTGATAGACATTCATTACCCGAATCAGGATCGTGCTGAAATGCGTAAAAATGATCCTGCAATCCTAATAATTAGAGACGATGATCGATTACCGAAA

>contig00434

ACGACCGtAAGtGGGGATTCCAATCACAATTTTTCCACCTGGGGTTCCGTTCTCCAACCAGTATCTtGTTtGGTAGTCaaTGTTaTCGTCAGGAACTCTTCCATAGCTTTCATAGATTGGAGCTGGGTAATCAGCCTCTTTCGGGTTGCGTtCAGGAGTTTTCTGGTCAAAAGTCATCAGGTGAACGGCATCGAGGTTTGATGACAAAAGTCTAGCATCGTAGTAAATGCTTGAATTGACGTGGGGCAAAACTGTAaGGgTGAGAGCCTtGTTtCTAGATCTGAGTTGAGTCTTCAAGTCACGAACCAGAATTGTGAAGCCATCACGATGTTCTTGTtCTTtGTCATCCTTGAATTTtCCATAACCGAAAGTCTtCTTGATTCCATGCCAAATCGAGCTGAAGGTCCCGCGATTCTTCTTCACCTTAACTTCAGGAAATTGCCATGCCAAATCAACTCCATCGAATTCATAATCAGCTAAGAGGCGACCCACCGAATTGATAAaTTTGGTCCTTGCTTCTGAAGTCTCGGTAACAGTTAGGTATTTGTGAGTTTCTTCAtAGGGATCAGCATTTCCACCGATACCAAGAtAAACTTTAAGATCGGGGAAATTCCTCTTCAACTgaGTTACTAATCTgTAAAAAGCATATCCAGCTCCTGtATCTAAACTTGGATTTAGAGGAACgACATCAAAACTATTTGCATCAATTCCAGCAAATCCATAGATTAagTAG

>contig00435

ACATACTGCGGAACAAAATGTTTGAAGCcGTTGATTTTTCtGAAGATGAAAAGCTGCCTGAAGACGAAGAAGAAATTTTAGTTTATGTTGAATTTCCAGATTGTGTGGAGAATAATATGTTTAGTAATGATAGAATTAAACTTGATATAATTGGAATTGATACCGAAAGCCCAGTAATGCAAaTTAaTGGAAAaTTCTATGAGGGAAATTTCGAAGATGTGATCGGCACTTACATGTTCTTtAGAAAAGATAACAaGTCTACGGTGGAAgATCCAGTATTCGAAGTAGCTCCAACTGTAAAATACCTTACAAAGaCTAGAAaGGTTTTAAAAATGCAGAGAGTGTTCGTTACGCCTAGAACTGAAGTTTTaGG

>contig00436

AATtGGtGGACtGAAGAaGTtAAaGAAAGAGTCGTTTtGGACGGTTTTtGTTACTGTGCGCATGGATCCCcTAGACTTGTGCTTCTGATtCTTCTTGATCGTTTTGACAGTTACATTCTTGCCTTTACGCCAATCAATGACGCATCCCTTGCTCTTGTAGATTTCAGGGCCTTCGAAACTGAACGGATCATTCTTCTCCGGTtcGCACTTCATGATgTaTTCCTtCGTTAAAACGGAaTTGGTAAAGTATTCATTTGGTTCAAAGTGGAACTCCAATACAAAACccATTGgaTCTTTCTCTAAGAATATCACTTTAACATCATACAGATGTTTAAGAATaGGTTC

>contig00437

AAAAACTTGGCTTCGATGTTCGTtGTTTTtAATTGAAGCTGTTTCAAGGCCTTgATTCGTCtCTTCACAGGTGCCGGAAGCAtctgTtgcgacAatgcgAaaTtAAaTCgcTCCATTTTGCGTGGGTCAAATCCTTCGAATCGATCTTGCAAGGCGGCTAGGACAGCAGGTCTCCGAAGAATCTGAGAAGTAATGTTAGAAGTGCGATTATCATCCTCATCTTCTACAGAATCCATATCACTCTGaTcaCcA

>contig00438

ACGTAAACACCGGCCTATCCTCGATAGTAACATTtGTtACAATTACAACTTTGCTCCGCAAATCATcTAAAaCTGTTagTTgAgAAaCAGTTTcTAATGCATGACGAGCTCCAAAGTACGTGTtGGCATCTATATTGACTTCTACCTTtCCGTTTTtATTCTTAAAAaTGTTTAATTTATAACTTTCATCAGTGCctAAAGTTATTCGGGCGTCATTAGATTTGATCATAATACGCATTTtCAGTGCTTTGCCGTTAGTTTtGGATTTTTtACAGGGAAGATTCTGAACGTTTTtCTtAAATTTTTCAATATTCATTTTCATTAGACGACCAACTTCACTATCTGAATTAATTATGGTGCTTGTTATTTTTTtGGGGTCCAAATGAACTATTTTTTtACCAATGGAATAGGTTGTGGGTTTAGGCCAAAGGGCACCAGCATCACCACAAAGTATTTCACAGATTATGAGAGAGTTTGGGTTTTTGACATCCGATGTGATTTCCGATTTTGTACATAATCCTTTATGGCATTTGTAGTGCCATGGTGAACTGTAGCGTTCGATAATCAAAGCATCAAGTGAATTCAAAAAATGAAAGAGGAAAATTATAATTAAAATTCTCATCGCGATGAAGAAGGTTCGCGTCCCGATCAGGGTTATCACAATGAAAATTATGTTCTGCTCTTGAGTTCTCCCAGAAGAGTCCAGTGTAGTCAATGACGTCACAGTGTAATACTTCTAAGTGATTTTCTCTGACT

>contig00439

TTCCTCCTCGCCCTCTTCATCTTTTGCGTCCTGGgATTCtACCTTTTCAGCCTCAGCATCCGACGCACTTGCTTCTAATTCTGAATCTGTAGGCTTTGCTTTAATAATTGTAGTTTTTACTCTTGGCTTATTCTCTtCAATCTGTTCACTTTCTACGCTTGATTCTTTGTCACTGGCATTCTCAACTGTCATTTCCAGACCATCCACCACTTCCGTGCTCTTACCGAGAAAACTTTTGCTTTTTtCTTCGTCACCTGATTTTACTGTCTCTTCGGGCTTTTCTTTCTGGCTGTTCTCATTTTCTGGCTTCGCTCCGTCCTTATCTTTAGCTTCATCCTGATTTACGGGTTTGGCATTGTCATCACTAGCCACAGAATCCGGAGCGTCCTGGCTTGTTTCTATTTCATGCTTCTCGTCCTCAGATTTCTCTTCCTGAGACTCTTCTTGTGTTTCCATATCAGCACTagatGcaTtat

>contig00440

CtGAGGCTtCtAAAAAaCAAAAaCtGTTTTtGAtGtCGtGGtGtCCTGACACAGCGAAaGTgAaGAAGAAGATGTTATaCTCTAGTTCTTTCGATGCATTGAAGAAATCATTAGtCGGTGTGCAAAAATaCaTTCAAGCAaCGGaTCTTTCGGAGGCTTCTGAAGAAGCTGTAGAAGAAAAACTCCGaGCCACCGACAGGAACTAAGCACATAAAAaGTaTTCAATCTGCCCGATACTTATGAAAGGAaCAAAaCTCAAGTGGTCAACAAGCACAGGAATTCTCTTCCTAAaGAATGCTTCGCGCTGTCACGTCGAAGATTCAACTTTTTACATATTTTTCATCATAAAGATGCTTTtATTGCTGCATGTATTAAAGGGTTGGGgAGGGGGGgAAaTATTTaTC

>contig00441

ACTAAAAAaTCTTTAGAGAGACTCTATCTCCATTGAATCTTCTTTACTGTCTCACAAAAGAAAAaCGTTACAACTTTTCGAAAaTGCTGGTGTCTAGGAGCATCATTTCCATTTTGAAACAACCTGTTATGGTCAATAGTATCATAAAAAGAAATATTGTCCACGATCTGATCTGT

>contig00442

ACAAGACCGAGAGTCCGATTGTCTACCCTTGAGAAGCTGG

>contig00443

ACATCGCCACTAACATCAAGCATTACAGAGGAGACCAAGCAGACTAAAAAGCTATTAGATAATAAAAGATAATTCTTTATTGTAAAGTGTAGTATCAACTGTAAGCAAATTGGATTATAAGTTTGCATTAAAaTATTTATTTtCaGG

>contig00444

AAACAGCTGATTAGTTTTCCTTTAAGAGCACATGTTTATGCGGCGGGTGATATATAATTTTCCTCCTTTTTCGTAGAGTTTCACAATTTTTTtGGTTATATACTGCTCACATAAAGAACATACAGTTTTCTTTTAAACAAAATTTTGATTTTCTGAAACATGAAAAGTTTGTTTTAGTTTGCATTTTCATCGTGTTTACAAGAAAAATGGCTGACCGAGATAGCGCTTCCGAGAGCGATTCAAGCTCGATTGATGGCGGTCCAATTATGATGCCACCGtCTCCAGTCACTCGAGAA

>contig00445

AGAACAAGACGCCGAGATACCAGAGTCTCACCGCATCGTTTTCGACGCAACAAAGAAATTATCACAATGAGCAAATCTAAAGATTCCCCTGTTACGGAACCAGAAACTGAAGAAGAATTCAGCGTCGAGAAAGTCTTGGACAGGCGAGTAGTGAAGGGCAAGGTTGAGTATTTTCTCAAATGGAAGGGGTATTCAAACGATGAAAATACTTGGGAACCAGAAGAAAATCTTGACTGTCCAGATCTCATCGCTCAATTTGAAGAACAGAGAAAGAAAAAGGAAGCTGCTGTCGCCGGAAAACGGCACGAAGAGAAAGATAAGAAAAGGAAGAGTTCCTCGACACCCACTCCGACACAAGCCAAGAAAAAaGTTCCTGAAGAAAAGAAAGCCGAAGGATTCGATAGGAACTTGGAACCTGAACGAATCATTGGTGCCACTGATTCAAGCGGTGAGCTGATGTTCTTGATGAAATGGAAGGGAACTGACGATGCGGATTTGGTTCCGGCAAGAGATGCAAATGTTAGATGCCCACAAATCGTAATTAaGTTTTACGAAGAGAGACTGACCTGGCACAGTCCAGCGCATGACGATGAGGGTTCCACAAAACCAGATCCAGAGTAGCCCCCaCTTTtCCAGCAAaCTTACTCTGCATGCTTTAGAATTTGTCGCTTtCAAGGCGCCAACTAAATCGTGCACTCTGAACCAAGTTTAATGTATTACCTTTAAACGAATGGGCACAAAAACAAGGCGCTTCGATAGAATTTAACATTTGACCATTTTtCACGAACCGATAAGTGTATTTTTAATTAGTTTTtACTTTACATGTAtATTTTtGAAaGATTtGTTTACTCAAaTTTTGAaTTtCTATGAAaCTTTtaGTTTCTATTACTTGAAaTTAAagT

>contig00446

TTACAGACGGTGGAAaGTATACAACTAATAATGGAGTGCATC

>contig00447

GCATTCTCCTATTCCTGGgTCAGAATTAACGAAaGAACCTTTCAGATGGGATCAAAGGCTATTTtCATTAGTATTAAGG

>contig00448

CCCCGATCCTCCACCATTAACTAATGAAGCTCAGCATCTTGACGATGATGAAAATGACGATGAGAATAACCCTATGAACGGTCCACGAAA

>contig00449

ACtttCCAaTCGGAAAAACTGATCCAGTCGTATCTTTTCCTGTAGTTTACATGCAAACTCCTGATCATCCACTAAACTaCTCAACAAAAGCCTAGATTCTAGATTTTTTAACAaCATATTCTACTTACTTAGTTTCTCAAAAAaaCCCGCAGTAATGaCCGTTAATGATACGTTTCTTTACTGTTAATCTAATCTAAATCTTAAAGGACTCACGAAAAAaGCAAGAGTTtAAATTTTCTACAtATTCAAATTTATTTTGTGTAATAAATATTACTTTGATTCAGATTCTTAAATAAACGTATTtAAATAACTCATAATCAATTGTATCGTAATAAAACTCACATAGATTtAAATAGTTTtATAATtCTTTTTGCAaTTAAAaGACTtATAAAAaTTTCtAAaTTAATAGAAaGATGAAAATAAATCCATGTGAGGTTTGATGTGGTTTAAATTGACTAATTGATTTaTAaGATTTtaCCAAAAGTTCATCGCCTGTTCAAGAATGGCTTTCGCTTCTTGATTATGTGATCCATTTGGAAGATAGGCAGTAGTTAATTCTCGAAACGCGAGACAAACACGGCGTGAATTTATATCTTCACGCAAGATGGCATGTTCCCAGGCATACCTtGgtgcTCcATAAaGAAtAAGTAAAGATTTTtCAGGCATTGGAATCCTCACTGTAATATTTCCACAATGAGGTTCTTTtACAtGATTCtCGTCTTCATCGATAAGCTTTTGAGCAGAAACATAATCCAGATTGTATCTTGTTTtCAGTCCTCGATAaGGAATCATAGTTAAAaTaGAATCTGAAATTACGTtGACTGTTACGAtCCTCTCTCCCCAAATCCAACAATCGTCTATATGAGGATCTATAGATGCTCCTCGTTCAGGATTATACTCAAGAGT

>contig00450

TtGGGgAGAAATTATCAGTTGGGAAATTTTAAATCTGTCGAAATGTTtCTTAAAAGTGTTCTATTATTTATCGTCAGTATAGCTGTATCAGTTGAAAGTGCATATCCAAATAAGGTGAGCATCGGCGTAAATATTACAGTTTTTATATGCTATGGCGGTGAGCACATTGCCAACGGAATACTTATAAGTCCACGCGACGTCATGACTCCAGTGGTTAATTCTATTAAAGAAAACGACCGAAGAAAGTTTATCATTAAATATTGGGTTGGAAATGGAGATACATTTTCCTTAAAAAAATTGAAACAATTCCAAATTTAAAATCTTATGCCGGACATTCTAAAAGTTCTAATCACAAACATTTTGGATTCTTGAGATTATCGCAACCAGTTGAAAATGCGTTACAAGAACAAGATTATTTGGATGGTTTGCAACAAACTGCGCTGCCAAAAATTGATATCAAaGGAAAAGTATtGGt

>contig00451

ACCGGTTACGATTAATGTGATGAATGATAAATGGAGTTTTGGAGGCAGGATTTCCTTAAAAAGCAATGAAAATATTTTACCTGGAACTCCTGTACTTGACATCAATAAAAAAaTAATATATGGACTTACtCTACCAAtCATGAAGAAAGAACCGGTTTTAACGGTACCGTTCAA

>contig00452

cTctAGAGATTATCAAGATTACATGATGATACAGGTCATAGTaCACCAAATTAGGTTTGATTTTAAAAAaCTAAGAGAAACATTAGAAAACGAGATTAATCCTTGATTGGTTAAAATTCTAATTTCATCCTCGGAATATGTAACAAaCTAGGTGATAAATGGTCCAAGTAATTAAGAGTATACGATCAATATTTTTtCTTTTTtAtAAAAaTCGTCGTGTTATCACGTGGACAAATATGGTGCGCTGGATTTATTTCCAGTTAACATTTTCTTGTCCACGTGATCAAGTCAATAACATGAATTGTCTTtCCAAAAAAAaGAGAAAACAAAAAaTAGTGGCAGCAGGGTCTTATTGATGAAAAAGTTATCCATGATATATAAAAACCTAGAAGTCTGAAGGGATTGAAAAAGGTCATGAGTCCTATAGAAAATTTCTTGATTTCCGCTCAATTCTCTTCCTCCTCCTCTTCTTCTTCCTGTTCGTCCTCTTCTTCCCcAgT

>contig00453

ACTTGGGCAAGAAGATGATGTTGATTCGGTGACGCGAAGGACAAATCGGTTTGTTTACGATGGGATC

>contig00454

ATTCATAACTAaTCgTATTTGTTCCGATCGTTATTTCCCcGATTCCGATCGAAAATGACGACAATGGGTGTCACTGTCTACTGCAACAGAGTCCAAATCCCTGGGAATGATCGGGAACAGTTGATGCTCTTGCGGACTCTGCAGGACAACGAGAGCAACATCCTTGGGAACAGATCGCAGGTGATAGCGCCTAAGAGTGTCCTGGTGaCAAATTCGGGTGTTCCGATGAATaGTGGTGTTGTGGTGAACGGTTCTATACCTAaTAGTGTTGCTACGaGTGTTGTTCACCAGCAAGTGATGAAtGTCCTTCCGCCTTATGACCCAACGAGGCTCAaGAAaCaTGGTAAAAACGGCCAaCCGCCACCAGTGGCAGTTGCAAGGCGCAACGCCAGGGAGAGGAACAGGGTGAA

>contig00455

ACTCCACCTTCGAAGATTCTCTTTTTAGGCTGTTGTTGTGTATTCTGTTGCTGcTtCTCTGGCTtATTTTTTTCtGCTTACtCATTTCTTGCTTAGCTTCTTTtCCATTCACTATTTTCTGTTTATCTTGCTTCAGAACTTGCTGATTTTGTTTCTGCTTTtGTTTTGACTTGGTGACTTCTGGTTCGGGTTCATCATCGCTTTCATCGTCTTCATCCTCATCGTCTTCACTGTCATCTTCACTTTCATCCTCAAGATCATCGGCGTTCATAACTTTAGTATCATCTTCGTCAGAATCAGACTCCGCATTTAAATCATCCTCAGAAGAATCGTCATCCTCAGTAGATTCTTTCCTGAGTTTCTTATTTGTTTTTTtAGAGTATCAACAGCTTTTCGTTTTGGTTTGACAAGCTGAGGCATTTCTTCTTCCTCATCTTCTTCCTCTAAGTCGCCAAAATCGAGATCTTCATCGAGGGTTAAGTAACCTGTTAAATGT

>contig00456

ACAGGAAGTTTACGTGGCTCAACGACCGCGAATGCTTTACTTTCTGATGGTTTGGCCTCGAGCTTAGCATGTAAACCTCTTtGCGAAACTTGCACTGGACACGCAtcTCAAGAGTCTAGTACT

>contig00457

ACCAACACCACTTtCCTTCCTAATGGAGTATTGCACCAGTAACAACCTCTCCGAGAAGAGCCAATTTTTAGCAAtGTATttGTGCGAaTTGTCTCTGCTTGAGGCAGATCCTTACTTGCACTTTTtGCCGAGtCTTTTGGCCGCTTCGGCTGTGGCTTtAGCTCGACATACCCTGAATGAAGAAATCTGGCCACACGAACTGGAATTATCGTCAGGCTACAGTTTGCAAGATCTGAGAAGCTGTATTATCTGtCTACAAAGGACTTTtCCACAATGCTCCCAGCATACAACAACAAGCAATACAaGAAAAATATAAAaaCAGCAAATACGGACACGTTTCCCTTCTGCTGCCACGGAACAGCTCTGTATTCGACTGTGAGGAAGATCTCGAAAGTGCCTAATAAAAaGATAATTTCAGGATCGAAGAAATAACGAaCCTCCGTGATACGgAACGTCTTAAGACACTAGGA

>contig00458

cAAAGCAGAGTGGAGACTTGGAGAGTTAAGTTCGCCTCACTTCGTTCCTGGCTAACGTATTTACTAGACGTTCTTCAGAGTTATTTCTTCTTCTAAAACCTTGTCAAGTAAAAaTAACAGCATGGTAGATCAAGCAGTATTGGACAAATTGGAAGCTGGCTTTGCCAAGCTCGCTGCTTCGGACAGCAAATCTTTGCTGAAGAAGTATCTGACGAAGGAGATCTTTGATCAACTGAAGACGAAGAAAACCTCCTTCGGCTCCACACTTTTGGATGTGATTCAATCTGGTGTGGAAAACCTGGATTCCGGAGTTGGAATTTATGCTCCAGATGCTGAGTCTTATACCGTATTTGCTGACCTCTTCGACCCCATCATCGAGGATTACCATGGCGGATTCAAAAAGACTGATAAACATCCTCCCAAAGATTTCGGAGATGTTGACACACTTGGAAACCTTGACCCAACCGGAGAATTCATTGTATCTACACGTGTGAGGTGCGGACGTTCATTAGAAGGATACCCATTCAATCCTTGTTtAACTGAGGCCCAGTATAAGGAAATGGAGGAGAAAGTATCCaGCaCTCTCTCGGGACTTGAAAACGAACTcAAGGGT

>contig00459

TtTTAAtAATTCCGGTTAAAAaTGTTAAAAATTGTAGTAAACACTTTTCGGTTGTTTtGATATTTTATTCCAATTAaTGCAATAGAATTTTCTaGTTGATATCaTCGCAATTTTTCTAGTAAAAAaTTTATCGCTATATTCCGGTAAACTTAACTAATATAGTCTaTTTAACATTACAATATTTCTAGTTAAATTTACTTTACCAGTTGAATAAAATACATATTTATTGTTCAATTCATTTACTTTGCTTATATTATTATTGAAAaTGTGATTTTGAaTAGATATAAATTTACTAAAtCTTCAAATTTATTTACTTTGAATTTTTCTTTTAAGTTATCTAACCAGAATTTTATAAtC

>contig00460

AaCCGATTAGCTTCAGTAAaTTTAACCGAACCTTTTtCTGAGTGTAGCCGACTGGAAa

>contig00461

CCACATAATAAGTGTAATTTATTATTATAAAACTTTCGATTCATTGCGACGTCTTTAAACCTGTTTAGAAAAGGATTGGTGCCACTGTGGCCCTTGTATATTAAATTTAATAATATTTTtCATAGTGTCTATACATGT

>contig00462

ACTTAAAGTTCTAAACGTTCATTGGACGATGAAAAATGTTCTTTTTGGGAATAAAATGATTAGTTTTTGCCCCAATTAATAAAAAAGTAGTGTATATTTCATGAGAGATTTTATTTTTGAAGTGCTCTAAATAAAAACGTAGTTAATTTTGTTTTGAAAACCAACTTGTTATCGTCCAATGAACGTTCATTACTATAAGTAAATCTAACACATGATAATCAAATTGTTGCATATTTGTTCTGAGAACTTTAAAAAaGGAAATTTATGTAATAATTGATATGCTCTTTAAATCTGAATCAGTCATAATCTACTGATTATC

>contig00463

GACGTTTATCACTATCGAATGATCAGAACGTTGCAATGGTTTTtAATCGCATAACGCGGCCGAACATGTTAAAaCGCCTGAAATTCACATTGAACaTTCCACGCTCTGCATTTTATAATTCTCCCTTTTTTtGTTtGCAAAGCGCGAGAGAATTTCTGTATACTGTAAATAGTCGAAAGTaTAAAAaTACAGCAGCaCAAAAGCACGTTTTGCACTAAAaCAGAAGTAATGTTAATAATCAGTCTATTAGGTaGAATTAATTAATTAATTAATTACTTAATTAAATTCATTaGTTAATTG

>contig00464

ACTATAAAGAAGCGTCAGACTAGAATAGATGAGTATTTGCGGAAAACTAAGCCAAGTTCTGGAAAATATATTGATGAAATTACACCAAAACCAGTAAATAAAACGCCTGCTGTAGTTTCGAAACAACTGAACCCAGAGGATGTGAGAATTGAAGATTGCGTGGATATTCTGGATAAATTGTATGGAGATGAGTGGAGAGCCAAAGCTGATGTGATTCTGTCGGAACCCCGGAGACTTAAAGTTGCAAAAAAaGaCAGAGGAGTGCAGACTGAAAAGAAAGTAAAGCCAGGAGTGAAaGGAAGATTGATTTTAGTATCAGATAGTGAAGATGAGGTTGAGTTTAGTCAATTTGTGAGTAATATTAAACCGACTCTAAaTTCAACAAGGAAAATAGGACCTGAGGGATCACAAACTTCGAACTCAACTAGACAGATTAGACGAGAGAGgACAaGgAAAAAGATaGCTTCAT

>contig00465

TGATACATCATCAAGTGAGGGTAGTGGCC

>contig00466

CAAGCTGTGGATGGAATCCGGATTAAAAaCTTGTGAATGTGGCATCGACTCTGGGCaTCGAAGAAGAACGTGGAAGTAATGAAATGAAATTaTAAACAGAAGTTACTTTtGGTCGTGtAAGaCCATTGGCATTATAACTtCTCTACTGTCTCCAACAAGTGGATCCATCACTACCGCTGGAAGCCGCCCATCGCCTATAGGATCCCAACTGTCACCTAAGATTTCCTTTATTTCATCCATGATATTTTCACCTtCATACCACGACTCCTCAGTTCCGATGTCACTTTCAGCAAATTCTAGACGATGAAGCGTATTCCCACGGAGTCTAGCAATTGCCAATAAATTTTCTTGTGCGTATTTATGTCCAATAAAAACTATTTCGACCAATTTAGT

>contig00467

ACTTTCTACAGCTGATTTAGTAATCGAGGTCTGGGGTTTTGGTTTTTGCGCTTCGCTCTTTGGTGGCGTTTCCTCCACCTTTTGTGATGgTGCTGTTTTACTTAGAGTAGAGGTTGGAGCAGCGGTCTTTGCTTCTAATTCTTTAGATCCATTATTAACAGGTGATTTCTTTTCCTCGGGTTTACTTTGTGCCGGAGTGCTAACAGCTGATGTGTCTGCTTTAACAGCTACAaGTTCTGTTTCGGATTGCGATtCTGGTAGCTTTTTTtCTTGTGTTTCTTGTTCCTGGCTGGGATTTTCGGGACTCAAGTTGTTATTTCTGCTAGATTCATTCGGCTTCGCTAGAACAGGCTGTTCTTGTTCGGGTTCGGATTTGCCAGAGTCTACGCCATTGTGAATGACTTTCTCGGACTCGgCTTCCTTTtCTGCATTGTtGGTAGAATAAGTAGATGGCGTCTGATAGACTGGAGGTCTGTGGTCTTGCACTAGACTTAGAGGCTGCTGGGgATGGTTCGCTGGTTGCAGCGTAACGTAaCTTAAaCcGCTACTGGTTAAGGGTTTCATTCCACTACTGGATTCTGATACTGGT

>contig00468

ACATGGGgAGTCAGCATCATTGCCTCTTCATTGACTTCAGAGCTTCTTACTGGTTTATTAAAAATCAGAAGATGTATCGCTTCAGAATGAAGTTTGCATTATTGACTGTCGTATTAATTGGAGTTTCTGATCTTGTGTGGTCACATGAAGCTCAGTTTCCATCGTCTCTTGATGAAGAAACTCCTTCGAATTATCATTATCAACATGCACATCAGGAAGAATACGAAAATTATTATTCACAAGGTAGTTCAAGTGATTCAGCCAGTCATATTGAGAAATATAAGAAAGAATATGCACATGAATATCAGAGTGCTGGAAATATGAATTATCCGGAAAAAAGTACTTCCACTTATGACAAAGGAAGTTACTCAAATTCCGAAGGCAAAAGTTCTGACGAATCTTCGAACGAATATTCAGATGCGGGTCAGGAAAAAaGTTCACAGTACCTTCAGAATGGAAATGCATACGCTTCTCAGAGAGGAATAAGACAGAAACATCAAAGTGTAAAAAaTAATGGTTATCAAGCAGGAAATTCAAACAATCGTCGGCAACAAAATGCATATAGATTTAGGAATGGAAAATCCAATGGTCTaCAGAATgAAAAAaTTACGGGCAGAATGGACAGAATTATCAAAATAAACAATACAGTAGTTATCAAGCAGGAAATTCAAACAATCGTCGGCAACAAAATGCATATaGaTTTAGGAATGGAAAATCCAATGGTTTaCAGAATgAAAAAaTTaCAGGCagAATGGACAGAATTATCAAaGTTTAAAAaTAATGCTTaTCAAACAGGAAAATCAAACAATCGTTTGCAACAAAATGCACATAGATTTAGAAAaCCAAAATCCAATGG

>contig00469

GCATTTTTAGATCCAGGCTTAGGTCCAGGTTTAGCTCGAGGCTtAGATCCAGGCTTGGCTCTTGTAACGAAaTTTCTTGTTAtAACTTTACCGTTTACAGCCGCTAGTCTTGTTCCCGGAGGCTTTAAaGCTGCGATCTtAaCGGGACTAGGTTGTtGaGgTCTGGGCGGTGGATCCTGTTCGAGAAaGGTCAGTAGATCGAAGTATTCCCATCTTGACGTGAACTGTCGGCCTGCCTGTGCTGCTTTTCTCATTGAACTTTtATCTGTTCTATACTTTTCACGAAGGTGCGTCCAATCTCTCCTTGGT

>contig00470

ttttCACaTAAAAAaTaGAgTT

>contig00471

ACATTATAACTCATACAaTGAGACCATTATTTTATtATCAACTGAtCATGTTTTtCTTCATCCCATTGCGTCCTGATAGAAACATACAATGTGAGAAaGCTACCGACATTAAATTAATTCGGGTTtCAATGGAAAAATTGTTCATGTAtATATACAAGTCATGCGAAAAAaTAAAAaCTTTATTTGTCCtCAAACTACAAAaTATTTGCCAAAAAAGAACATTTTAGCACTAGAGTAGATATATAAATTtCCATATACAATTACAGCAAAGTAATTT

>contig00472

aCTAACAACTCCGCTGGCGCTTTGAGCAAaTCAGGCTACTCAAAATCCTCTTCTGGTCTCCAAAAAACGATTTCAAATCTCAAATTTTtGTTCCCTCCTCAAATTTATTCAGATaTTTGAtCTCAGAAGTTTCAGATTAGGAAAATATATATTCATGATTcTTTtCTCTACCATAAACAATCTTCTTGTAATTTAAAACTATAATTTCTCAAAAATCTGAACAGATACAGGATTTtGTTTTTGAGATATCAGTTTTTGGAGACTTATCTATTTATTCTGGTATAAAAaTTCATTTAATGTTGTAATTTGTGCGAAGTTTCACTTTGAGgTATTTTAGCAGTGATTAGGATTTAGGGTCGATATTAATTAATGTTCCCTGATATCAGCGCACTTCTATCGTCACCAGATGGTGCAAATATCGTTAGTTTCACTAGTATACATTGTGGATTTCATAAAGTAAACGCTGTTTATCGCAAAGTCTACTAAACTTATGGT

>contig00473

ACAAaCAATtCAATGAAAGGTTTAACAACTGTTTACGTTGCAGGTTCCTTTCTATATGATAAGGATAGACTTATTGGAATGGGCTTATCATCTTCAAATCTTCAAGGATATGTTTTTGCATTCGTAAATATTGGAAGTTTTAAAaGTGACATCGATGATGCACTGGCGAAATTCCCTGAaTAAaCAATGTTCTAATTTtAAGAAgaTGCACAACTTAAATGAGTGCCAAGAATATATTTTtAACGTAAaCCATTTTtCATTTTtCTtAATGCAAGGTGAAGTCAAGAATATATTTtAAAaTTTtAAaTATTTTTtAAAtATAAGAtGGTtGTGTTTTTACATtCATAATATATTCTGTTAAAAATATTACGCTGTGTtaTATTGTAAa

>contig00474

ACTATATTATATTCCCTGATAAAAAT

>contig00475

AATGTTGTTGATTTGAAAAATAAACTTCAGGATAATTTATCAATTATGAAAATCAATGAAGAAAAGCAAGCACAAAAACTCGCAGAAGACGAAATGGGAATTCAAATGCTCACATCAAGACTTTCAACTCTGATGGAAGATCTCAGTCGAGAAAGCAGCATTTTAGCAGAGAAGTCGAATGATTTAAAGAAATACAAAGAAGAACTGGAAAGTCTAGAATCTGAAGTAAAG

>contig00476

ACTGtCGCCCTTCGAGAAATCAGAAGATACCAGAAATCAACtGAATTGCTGATCAGGAAATTGCCTTTCCAGCGTTTGGTTCGTGAAaTAGCTCAAGATTTCAAAACTGATCTTCGATTCCAGAGCGCAGCCATTGGTGCTTTGCAGGAAGCCTCCGAGGCATACTTGgTCGGtCTTTTCGAAGACACAAACTtGtGcGCCATTCACGCAAAACGAGTCACTATTATGCCGAAAGATATCCAaTTGGCTCGaCGAATTCGTGGCGAGCGTGCTTAAATCTTTCAGTTTAGACTTtATTTCCATTtCACTTCTTTCTCATTTTGTCGAACATCATTTTtcTtAATCCACAATGCAATTCTTTATGaGCTCCTTCCGCAAAAATATTTTATTGTTGTTTTCACTGCCAAGCACTCCGAACGTTAGGCGAAAAATTCCAGATCCAGATCAACTGTTTGGCAGAGGATCTTCAATTTTTAGAACAGGAATGTATTATGAGTTCTAATCTTTAGTTTGTGGTATTGTTAGCTTAAGCTCATGTTACAGTTCTGAAGCCGCAGGCTTTAGTTTCGAATTTtCCGCGTCGAATTGAGTTCTTACAGACCTGTTGTGGCGAACCATTTtATTTTTACAATTTTATTCGAGATGCAAATTAAATAACACCAAATATCAGTTGATCATTATGTATTGCGATAGAAGAGCAGTTATTATTTTCACTTTTTAATTGaCGAAAACGTCTAACTATTTGTTTGAAGTTtGtGTTATAAaCATTTCTAAGAATTGAtCTTTGATTGCAATACTTtCTCTGTTGGCGCGAAAGAATCGTTTTTAAAGCTGATTCGaCGGTGAaTCTGAAAaTGaTTCTTA

>contig00477

ACTTTATtCAAAGAACTACTCAGTCAGGTAACTGATGATCATCCAACAATAATATTAAGTGGCATACCTCTCGATGATGTTAGAGCAATTGTCGAATTCACATATCAGGGTGAAGTTCATATTCCCGAAGAAAATATTGCCAATCTCTtAGAGGCTGCACGTTTTTtaCAAATAAATGGTCTGATGGAGTATGACGCAAACGATTCGGACATGAGATCAAaGCATTTTAACGACAATGATGATGTCTTTCAAGAATTGAATGATATGGAAAaTAAAGTATTtGAAGTTATAGAAAAAaGTTACGAAGAATCATATGAACAATCGGgACAAAGTGCAGCAGAGGAGAATTCTGTGGTAAaGAAAAGAAaGCGGCGTTTtGCTGTGAAAAGGGAATACAGCGATGAAATGCTAGCTACTGCAATTAaTAATTTACGTGATGGTCAGACTTTAATTGAGGCTTCTACCAAAAaTAATaTTCcTCGATCCACTTTATACATGCGAGCTAAATCATTAGGAATTAATTTAAATGCATCAAGAAGCGAATACCCCGCGGAGTGCATGGGTGCGGCGATAAAGTCTGTCATTGGAGGATCGAGTTTGCAACAAGCAGCAGAA

>contig00478

AAAAaTaTGCAACTTtACAAAATGCGAAATGGATATGAATTaTCTCCAAAAGCAGTAGCTAGTTTCACACGTAGAAATCTCGCGGATTACTTGAGGTCTAATTCTCCTTACTTCGTAAATCTTTtGATAGGTGGGTATGACGAGCAAACTGGTCCAGAACTGTATTTTATAGACTACTTAGCATCGTGTGTAAAAGTTCCTTACGCAtCGCATGGTTATGGAGGA

>contig00479

ATCGTCGTCAGGAAaTAGAGAAAGATCTATAGGATCATCTTtAGGTCTGTCTTTAGTGTTTTCATCGTCTGCGGGAGGATCAGGAtCATATTGCCTCATATTGGCCATGAATTCAAGGTGTTtCTTTtCTTCTTCCATTTGGGCTACAGCTTGATCACTAGCTTGTAATTTTTGTTGTGTGCCAGCTAATTCATCCCTTAGCCAGGCATTTTCCTGGCAAAGCCTTCTGACTTGCGTTCTGaGTTTtGTTTTtCTGCTTCTACCATTtGCAAGTGATTAGCGAGGGCCATCATTACCTgaGCTTCACCCAGACcTAGCTCTATCATTTCGATGTTTTTGGACAATAAAGCCGCTTTTTCACGTGCCTCAGGTGCATCCTGGGACTGCAAACtctGgagAagtCcactgtgTtcAactctcAaagTttCcAaaCcTTGGGCAACGGT

>contig00480

ATAAACCCTATAAATTTTAATTGCTTCACGTTTtGTTATCTGTCTGAAAATAAATCATAAATATGACACTATTTTtGGAGGCTCGAAGGTGATGAAACCCGTAAaGAA

>contig00481

ACAATTATTtATCAGTCAATTTAAtAGGAAAGaTGATCTGAGGAGTCTAAAGCTAATTGCCAGCTATAGTAAATTCATTTCATTTTCtCATTAACTAAATTaCTTGTCAATACAATGACAAACTTACGATTGATACAGTGGACTAGATTTCTGTTTTGCTAGATCAGTCGCTTTCTTCGCCACACCTTTCACTTTCTCAGCTTTGTCATGTAATGTGTGCTGTAgATCTTCTGCAGCATGAGTCACCTTGGTAATAACTGGTGTCTCCATCCTTTCAGCAAAATTCATTTGTCCAAGAACAGGTTTtATACaCTGAGCAGTGTGAGGAAACATATGCTCCAAAaCATAATTGAAACCACTGGACATTTTAGCACAATTTTTtCTGAATCtATCAAGTCCAAATG

>contig00482

AaTTTACTGATAGTCGAACCTAATTTTGAGAATGTGCCTTCTTCTTCTGCTTCTTCGACAGCTAGAGTtGTTTTTTCAGAGACCAATTCTACGTTAGCTAAATTAAGAATTCCACTTTCGTCCATGGAAAaGTGAGTTTTGATGCCTTTGATTTCCGCCTTTtCTCTTgCATGCTTCTCCAAAGCCTCGGCGACACCACTTAACTTCACAACTGATAAAGTCAAGTTACCAAGAGCCgCgAcTTCTTCACTTGGTAGATAGTCCAGATCAGCATAGTTAAtATTGAACTCGAAATCTTCCGTGTGCTTATTAAAaGTGATAATCTTCTTCTGtGGATAtGCATTCATTTTACTGAACAAtGTTCTCTTTACCTGCTTAACCTTATTGTtATTGTTCCTGTTAAAGATAACTTGTATGGGGaaCaGT

>contig00483

ACATCCtGATAATGAAATTGAAaTCGAGCCTTTTGATGTGAAAATTGCAAAGCCTAACCAAGAAACTGCCCCcTGCCGTCTTCAAAAAAaGAAAaGAGGTGTTATCACAGCTTATCAACATCTCCAGCGTCGTTTTCGAGAAACGTATGCTTCTTAACAGTGGATAAAaGCTGTGGCCCATTTTAAGGATCCGGTTACGTCTAATCAAAGTTGACGCATTTAAGTCCGTCTTTGCATTTGCAGCTTACTAGCTGCTGAGGTCAATATGAAAATTTCGAATTTCTTTCAGATCTTGTGAACGTTTTGGAGACCATATTACAAGATCTTGTGCAAATTTTtACAAaGAcaGACAGTTGTATgTATGATACGAAGATCTCAAATTTCCGACCGTTCGGATGATTCTAAAATGCAATTTTGTGGACCTAAATATCTACGATCACTATCGaCTGAaTAGAGTtAATTTTtttCTAACtAGATTtCATACGAAGCTGTTCCATCCGATGAAAGCTATCGCTTtCTTTtAACAGTATGAAGTCCTGCAATAATAAACAAATT

>contig00484

GACTTTAACAAACTTTTAGCAAAATAGATTTGAAATCGTGAAATAAGAAATCACTGAATGAAAAAAATCTAGGAGGATCCGTAAATTTGATGGGTGGAAAATTCTTTGACGTTAATTTTtCACAGCTTTATCTCTCATGAGCACGGATCTTGTTTAATAAGCCTAAAACAGATCTTATTTAAAAATCTTCCCTTGATTAAATTGACGTCCCTTTTtGTCTGTTCCAGTCCAGCTAAAGTATTGTTTCACTAAGTCCTTAGTCTCTTCTTTAGTTGGATCTAATTTTGTCCAGTTGTAAGACTCATAAtCAATTTGCCAATCTGGGCACAACGAGAAAGCGAGATCTTGTCCACGCCAAACCCAAaTGCCactGATACTGCTGTCGTTGTCTGtaCCGAAtAGACATGAACTAGCAAATGTCGCTTTTCTCATTTTATCCAAACGTTGGAACATTCCACTAATTAGATTACAGGACATAAATACCTTCGTGAGTTCATCGTTGTATTTATATTCACCTAACCAGATACTGTAATTTTCCGGATCAAATTTCTCCCAGAAGTAGGGGATGGATTTAGATTCATCCTGATTAGAATAAAACCGCTTAAAGTCTTCCATATCGAAAGTGCCCTTAGGCATGGCGTCAAATGGATTTGAAGTCTTTGGCTC

>contig00485

ACAGTATCCATAAAAaGaCATAATCCATATAtAGATATtCTACAGTTTGAAaTTTTtACACATTATTGCACGAAACTCACATGTCTTGCAATGTCTGGTCGATTTATGGAAATTTTGAGATaTTTAACTCGgCGACCTTCATGTCTTAGAGACATCCCTACAAGTTTtCTGTTTTtAAACAGAAGCGAACCCTTGTATGTaTTGTCCGATTCATCTAAATCAGCAATCAATTCTTTATAAaTAAaCCCTGCATCTTGTGTGACGCTAATTACATCAAATTCATAACGCTTTTCCcATTTTGTTTCTGTAACTGTTCCCAAAGTCATATCATTCTCctCCAAaGGAGTAATTAGTTGTtGAAGACTAGAATATGTTAGTCCCCGATTTGGCTtCAACTTTATAATTTCAAAGGTATTACAATGATAAAGAGTATCTTGTTTGTCCATATAACGAATATTTTtATTTAGTCCTCTTGCAATTTCGAGATGAaCCTTGACATCATCATATATATTTtGAAaTAATTGAGTGCAACTAAGGACaGTTAAGATGAAAACaTTaCTAATtAcGGCCCCGAAACATAGGATTATTTTTtGTGT

>contig00486

ACAGCGGCTTTTtCTTCATTCAACCACAAAGTgTGTAATTCTGTgAATCCtCCATCAGCGATaTTAAaCATAAACTTGCGCTTtGGCTTGACTTCCGCCTCACCAtCTTTTtCTTtAGCGTCTTTAGTATCTTTCTCTTCTTtCTTTTCtCCTTCTTCTTCgTCATCTTTCACTATAACCACATCTTCTTCAGCCTCTGCTTTAGGTTTGGCATCAGCATTTTCTGTCTTTTCTTCTACCTTTAGAGGAGACTTCTCATCTTTTTCACTATCTGGTTTCTCCTTTTCTTTTtCCTTTTCCTTCTCGCTGGTAtCcATCTTTGTCTCCTCATCTtCCTTCCTAGCCTCTTCGCCTTCTTTATCCTTTTCACCAATCAACTCTTCCTCTTCCTTGGATTCCTTGTCTTCGTCCTTTAATTCTTTCGCTTCAGAGGTCTCCTTCTCACTGGCTTCTTCCTTCTGTTCCTTGACCTCAGTGGTTTCATTTACTTTGCTCGGATCGTTGCTTACAGCTCCAACAGCATTAATCGGAGTAGGAGTTGCAGCAGGACTAGGACTGGGAGCATTAGAAGTCGCAGGAGTGGCACTAGTGCTGCTAGTTCCAGTCACCGcATCTCCAGTTTCCTGAGCTTTCACTGGTtCCACtGGTTTTCGAATCATCTCTGGCATAGAGTAATATCCATTAAtGTGCTCGAACTCCTGGACTTTCTTCCTAATCAAAGACATAaCTCCAATCCTCGTTAGAACATGTTGCCTACTTAGACCCTCTCGAGGCACTCCGTCAGCAAACGtctCCgCATTATCAGCACCAGGTTCGCAAAGGTGGCGCATAAATAGAGAAACGT

>contig00487

ACTACAACACAACAGAGCAACcAAAACAACTCATTGAGTCCACAACATGATTCCAACACCTGGTTGGATGGCCTCAACAGTCCACTCATTCAGGATGAAGGTGACAACAAAATGCTGAAACTCAGATTTGATGTTTctCAATATACACCAGAGGAAATCGTCGTGAAAACAGTCGACAACAAATTGCTGGTCCATGCTAAACATGAGGAAAaaTCAGAAACAAAATCAGTCTACAGAGAATACAACAGGGAATTCCTTCTGCCTAAGGGAACCAACCCGGAGAGCATtAAATCCTCCTTGAGCAAGGATGGCGTCCTCACCGTCGAaGCACCTTTGCCAGCTCTTGGACAGGgCGAGAaGCTCA

>contig00488

ATTTtAAATAAGAATTCGGCAGCTATGAATGCGAAGGAAGGAGGTGAATTAGATCTACAAAATTCTGCTTCTTAAAGTAAAAGTAGATtATGAaTTTTGTTCTTTTTCTTTACTTTGGCAAAGTTAATGTGAAATGTAAATTGTTTATTTAAAGAACGTATTTTCCAGAGGAAA

>contig00489

AcAGGGGAGAAGTAAAtAtCtCCcAAGACCAATtGGCAGCCCtCCTtAAAGCCGCAGAGTCTTtACAAATCAAAGGCCTCTCGGAAAGTAGAACTGGTGGCAATGGTTGCACAAAACCGGACACCAGGCAAACAAAAGTCAGCTCACAGTCGACGGCACCGTCGTCATTAGACATTCCACATGCGTCTTCCGGTCTCACTATAGAAAAGAACAACAAAGTTCCTAGGCAGAGTTTGGCACAGAGCTCTGTAGGTGACCTACCTGAAGACTCTGCAAGCCCCTCTATCCCCAAAGGTCTATCCTCCAGGGAAGGTTCCCAAAGTCCAGTCGGTAGGAAAaGGAAGAGAAGAAGGAGAAGCATAGGCGAAGATACTTCTGTAGAAAACCACGAATCCAACTCTAGTGATATGACCCAACAAGCCAACGTTCCGGCATTGGGAATTGCACCTGTTGCCGATGAGAAATCCCATGCCGACCCCTCCGACTCGCTTGGAAGGTCGGCTCTCATGCAACAGTTAACGAAACCTGCCGACGAGATGCTTCAGCTTCCTGTTGAAAAaCCCGAACCTACCGAAGACATGaTACAaCCAAaGTCTGAGTATTtGGACGACCCGGAGGAGAGTGtGGAAGACTTAACGCTTGACGATGACATGAATGACCTCAATGAAATGGAACAAGATAGTAAtAGAGCCGGACCAAGT

>contig00490

ACTGTGTTTTGGGTAGTAGCACATCGTTGAACCGGTGATCTTAGAGTGCTGCTTGTGATCATGAAGTCTTTTATTGAATATCCAGTTGAATGTGAATATCCTATCGAGAATCTTCCTTATGGTGTTTTTTCAACCGAAGATTGCCCTCAAAAACGGATAGGAGTTGCTATTGGGGATCAAATCTTGGATTTATATGCAATCGCTAATTACTTTACTGGGCCACATCTGGAGAAAAAACAAAaTGTGTTCCGCGAGAAAACCCTCAATGAATTTATGGCTCTGGGAAGACCAGCCTGGATTGAAGCAAGGAAGACTTTGCAGCGCCTATTGTCACCCGAAAACACAACTTTACAGGATCCACATATTCGTGCAAAaTCCTTTGTTCCTC

>contig00491

CTACAATGCACATGCCTGTCACG

>contig00492

GGTGATTATACAGATTTCTACTCATCGATTCATCACGCCACGAACGTCGGTATTATGTTtCGAGGAAAAGATGATGCCCTTATGCCGAACTGGAAATATCTTCCGGTTGGTTATCATGGTAGAGCAAGTTCAGTAGTAATTTCTGGAACTCCTATACATCGTCCACGAGGGCAGACAGTTCCAGTTGAAGGAGCAGCTCCAGTTTATGGACCTTCGAGAACACTAGACTTTGAACTAGAAGTAGCTTTCTTCGTCGGAGGTCCACCTACCAAATTGGGTCAAGCCATTCCCGCATCTAAAGCATACGATCATATTTTTGGGATGGTCGTTATGAATGATTGGAGCGCACGTGACATTCAGAAATGGGAATATGTTCCGCTaGGTCCATTCGGaGCTAAAAaTTTCGGT

>contig00493

AGTTTGCCGGCGtCTGAAAAGGAAAGAGGaTcAGtaCATTtCGGTCTTTCATTATTGCACGAATTCCCAAAACTTtGGATTCAACTTCTTCAAAATGTCTTTTCTTGGCAAGAAGTACAAACTCTCCCAGAGTGAGAATTTTGATGATCTCATGAAGGCTCTCGGTGTTGGTCTTGTGACCAGAAAAATGGGGGCTTCTGTAAGTCCAGTTGTGGAAGTCACTAAAGATGGTGACGAATATGTAATGAAAACAACGAGCACCTTCAAAACTTCAGACATCAAATTTAAACCTGGTGTAGAATTTGACGAAGAAACAGCTGATGGCAGGAAGGTAAAGAGTGTTATCACAATAGATGATAACAAAATGATACATATTCAAAAAGGAGAAAAAGAGACCATCATCGAAAGAGAATTTACACCAACAGAAATGAAAGCAATAATGAAGGTGGATGACATTGTGTGCACAAGGGTTTATAAAGCCCAGAACTGATTTTAACACCAGAGAAACAAATTTCAATACCGGAAGGGTGTTCTACAAATCCTCTAAGCTCTATATGAGAATCTCAAATGTATTTAATGAAAGTCTTGAAAATTATAATTTTGATTTCAAAATGAATTGTAAAATTTTTATAACACTTTTCACTGTATTTTTATAAATTAGAGTATTCCGAGTTAGAACATTCCTTATATTTTGTCACATAATTTCGTGTGGTTTAGTTAAGATTGCTTTATACAATAAAATTCTTACTGAATAAaTTTtGTAaTTAAAaTGTATTTTGAaTTAATCACATTGCAAACATTCTGAGAAATGTAAAAGTATTTAACATGAATAATAAAATGGGT

>contig00494

ACATTtGAAACAACGAAGCAAACAAGCTGGAGTTCAAACTGCGATAGAAAACGATTACAAACGTTTTTAATATAAGTTATATTTTGTGAACAATTTGGAAATCAATTGGCTGAAACCATGAGGTCGAGAATGTCTATTATCCCCAAAATGATGTCACAGTGGTGGGAGACATTAGAGCGGCCCCATCGAATTATGGATCAACATTTTGGTGTGGGAATGTCTCCGGAAAATTTTTTGAGATCATCCATATTTGACAGAAGATTTCCTTATGCTTATTTCCGACCTTTCGAAGAGATGTTGCAAAATGAGTTAAGGGAGGATGCAGAATCGAGAGGATATTCTGTTGTCAAAGCTGATAAAGACAAATTCCATGTCGCTTTGGATGTCCAGCAATTTAAaCCTGACGAAATAAATGTAAAGGTTGTCGACAACTGCATCGTCGTCGAAGGCAAACACGAGGAGAAGAGAGACGATCATGGTTACATAGCAAGGCaCTTTGtCAGGAAATACGTAGTTCCAGATCAATGCGATCCTGAAAAGACGACAAGTTCGCTTTCATCTGACGGAGTTCTAaCTATCACCACtCCCAAGAAGCCAGAGGCTATAGAGGATAAAAAaGCAACAAACATCAAGATTGAGCACACTGGAAAACCATCCATAGAAAATAAAGAACCAGAGGAAAAGAGAAGAATTTCTGCCAAaGGCTAAACAGTGtGAACAATTTGCACAAAACCATGTAAACAATTAGTtCACATTTTCGTGAtACTTTGTTCCTTTTAAGACTGTTCATATGACCTCAtCTTTTGTAATTTTtGGCATCATaTaTATGGTTCATaTAaTTTAGT

>contig00495

AACATTAAGCCAGGCACAATAA

>contig00496

AAAAAAaCTAATGGTGGAAAaTTTtATAAAAAATaTAATCGTTGATGAGGTTATAAGTTGACTCCTAAAGTCAAGACATAACCTCAACAATGATTGAAAATTATTATTAAAGGGTTGATATAAGTCCTATTCACAATTATATTTAGGATAAGCTTTTtATTTTTtCGTTTAGATGTTAATTCTTGCGCTTTGCTTGGATTCAGTTATCATTTATTTATATGCAAAGTAaTATTTTGTGTTATTCTATACACTAGTTGTCGAACTACCTGAATATAATTAATTCAGTGAATTGATGTTTCAATTATAA

>contig00497

TAACCTTCAGAAGTTGGTTCACTTTCGTAGTAACCTTGTTGCCATGCACTGTAGCTGTTCCAGTAAGAAGATGTGTCGTAGTAATTGTAACCTTCCGATCCAAGATTTGAAGAAtAATCAGTTGAAGATGGCGGAGAAGAcctaccTGATTTACTCCAAGGCCTTGGCACTGCGTTACAGATTTTTAAAGATTTGGTTCCTAAACCTCTATAACCGTTCATAGTGACCAAGCAGTTTTTCTGTTCTTCTTCGTTCGCGAATCTGACGAATCCATAACCTTtGCTGAATCCAGAGCTGTCAAGAATCACTTTCGCTGTTCGGATTgAGT

>contig00498

ACTGAGTCTTTTAGGAACTAGTCaTTTCAAACATTtATAAAAAaTaTCTGAATGGAAAAaaTGTTGCCAAcATTTGTAAACACTCGTCCAAAATCAGCTTCAGATTTATTTATGAGAAAGGAAAGTGAATTAAGAGCAAGGAAGGATTCATCAGAAATGAATCGTTATCGAACATATAATGTGAAAGATTTGAAAATGGCCAAAGCTTACTTGCCACAAGATGTGCTTCGTGAATTTAAAAAaGGTTTCGAAGCATCTACAAAATCTCCTTCGGTTtCAAAATCCCAAAaTTTtGTAAAAAGGTTCGTGGCTGCTTtGGAAaGTAAAAAaGATCAAaGAAATGGTCACTGtGTTGCGGACGATGAATTTTTGACAAATTGTTATATatCTCCGTCAAAAAaTTCtAAAAATACAGAAATTAGGAAAGACTGTTTTGAAGATGAATATACTGCTAATATtGAAAATTTTAGTCCATCAATTtCAGAAGGATTAAAGCAATTAAACATAGATGGTGTTGTGAAAAACGAAaGCATAGATTATTTTAATGAAAAaTCAAAAaTCGCTTTtGAGGCCGATGATTTtATGAAAATCTgt

>contig00499

TTtGTAAAAAGGCGTGCAACTTTTTGAAGTAAATAGTCACAATTATAACGTAAAAATGGCTTTACACGTTCCAAAGGCCCCTGGAATGTCCCAGATGATGAAGGAAGGAGCTCGATACTTTtCTGGTCTTGAAGAAGCAGTTTATAGAAACATAAGCGCCTGCAAGCAaTTCTCACAATCCGTTAGGACGGCTTATGGCCCGAATGGAATGAACAAAATGATAa

>contig00500

CCAGATCGAGGGCGATATCACCAAAAAGACAAATGCTAAGATCGCGGTCTACACATGTCCAGTCGACATCACCCCCACTGAAaCCAAGGGT

>contig00501

ACGCAAGTCTTGAGAACTATTAGTCTACAAAGTCCATCAACACCAGGACAACGCTTGGTAACGATACCGTTGCAAAATGCAAAAGTATCATCGGTAAAATCTGGTGACACATTGATGACGAAGACCATTCAGCTCACTTCAGCTCAAATGAGCGATATAAAACAAGCAATAGCATCTCAGCAGCAGCAGCAGGCTAACAA

>contig00502

TGATACAGaCAGTGGAGATTTCATT

>contig00503

ACCAAGCTGACTGTGACTaCGTCACTGTTTTTCGAGACAGTGATTGCACGTAGAATAACAGAGAGAAaTTtCTTTTATAAGGGAGGAAAGTCAACTTtACAAGAAGACAAGAGTAaTGGAACGCGAGTCGAATCCTAtGCAAGCTCTTTGCCGCAGTGGCTGCGGATTCTATGGTTCCCCAGCAACGGACGGTCTCTGTTCACTATGTTACAAGGAGAACTTAAaGAAAAaGCAACAGCCCCCTGTaTcAGCCACTCCTGTATCAGCCTCGCAGACCTCCTCCGGTAACGctGGCACGTTGCAAAGCGGTTTCGGCAATCCCGCTTCTACAGGAACTACCGCACAGCCCACCATTCCTACCATTCCCCAACCCACAAcAGATTTACCTGGCCCCAAAGAAaTAAGTAGAGAAGATCAGGAAGATGAGATTGGAGTCAGCAGTGGAGTTGCTGAAGGGTCAATCAGCAGTGGaGACGTCGACGACTGTTT

>contig00504

ACATTTTCGGAAATTAAATGTAAGTTCACACATTTCCTATGACCATCCACTAAGTTTTtGTCAACTCATTAGAGTTTAAGACTATGTACGATTTTGAAATTGTTTGACTGTGGAAGTTAAAATTAATAGATACTTCGAAATCAGCGATAGTTCAGCTGCTTCAAAGTTATGTTTAATAGTATGATTTTTATAATCTTATATTTTTGTAGTATTTGATAGCAATGAATAATTGTCAAGTTTTATTAGGTTGATATTTGAACTATATCTAGGGTTGAATTCTCGTATTCTATACTTCAGACATTCACATGTCAAACTGTTTAAAGGTCGGATCATAATTACTGACAATTTTCTTCTAAGACACCAGAAAGATTTTTAATGTGCACTTGAATCTTTCAAACTTTCACTTCACTTTtCAAAaTTTTtCAAGGGTCTTTGTAAATTTGAGAACGCGAGTTGAATAATAAATATATGTAATTATAATATCGAaGTAAaGTGATACTGTAGTTAaCAGGTGCGTTTTCACTTCATTAATGTATCATTTtAATATTAATTTTtGCTATaTAaTAaTAAAGT

>contig00505

TTAaTTGAAGGACGGAaCTTAaCAACAAaCAGGACAATCAAtCAAGGCTGGCTAGAGCGATGTGGGATGGGAAATATCCtGGACATTCTtCCAGAAGAGCCCCGACGACTCTCTGGTATAAGTGATTCTGGAG

>contig00506

aCGAACGTGGTTGGAATTAAAAaCAACAAAGTGATACTTTTCAAAGATAAGACTTCGATCATGGCTATTACTGCGCCTGCACCGCATCTACCAAGTGTTGACTTTGTGAAGACTAAATTACCAGTGAATCATCCACTAAAATACATTGGCATTGACAATTCTGAAAAAaTAGTCAACTTAGATGTCAAAATTGAAGGTGAAACAGCGTGCGGTGCACAATTTGTAATAGAGGGACTACATTATTGTGCATCTATAACGATCTCCCCAACTCCTTGTCTGAAAACCCAATGCGGAATTTTATTTAACGGCGAATCATTCGCTGGAATCTTtGTTGAaCAAGGCCAACACTGTGCAAAAACATTTtATTTtCTCAGAGCTGATGTAGTTCACAAAGTTTATGATGAAGCAAAATCCCAATTAtAAAGATaTTCCAAAGTTTTTAACTAGTGCAGCacAACACAaCaCTAAAGTCT

>contig00507

ACCAATTGGTAGAGGTCAGCGAGAATTGATCATCGGAGACAGACAGACTGGAAAGACTGCTCTCGCTATTGACACTATCATTAACCAAAAACGTTTCAATGACGGTGGAGATGAGAAAAAGAAACTTTACTGTATATACGTAGCTGTTGGTCAAAAGAGATCAACCGTCGCTCAAATCGTGAAGCGATTGACGGACAGTGGAGCTATGGGCTACACCATCATCGTGTCAGCTACTGCCTCCGACGCTGCTCCGCTACAATACCTAGCTCCATACTCTGGCTGTGCCATGGGTGAATTCTTCAGGGATAACGGCAAGCATGCTCTGATCATCtACGACGACTTGT

>contig00508

ACTCTCGTCTTCTGGAGaGAGCAGCTAAGATGAACGAaTCTCTtGGCGgTGGTtCCTTGACTGCATTGCCAGTTATTGAAACTCAGGCCGGTGATGTATCTGCCTACATTCCCACCAACGTCATCTCAATCACTGACGGACAAATCTTCTTGGAAACCGAGTTGTTCTACAAGGGTATTCGACCAGCTATCAATGTCGGTTTGTCTGTGTCACGTGTAGGTTCCGCTGCCCAGACCAAGGCCATGAAGCAGGTTGCCGGTTCCATGAAATTGGAGTTGGCTCAGTATCGTGAAGTCGCAGCTTTCGCCCAGTTCGGTTCCGACTTGGATGCTGCCACCCAACAACTTTTGAACCGTGGTGTCCGACTTACGGAACTCTTAAAGCAGGGACAAT

>contig00509

ACTTTTCCACGTAATGTTAAAGTTTTCACCTAGAGTCAATACGCAAATTGTCTCAAAGGAGAGTGGTTTGGGAATTAcAATTAAACACTATAATAATAATCCTCAATTTCAATTGGACTCCAGTTTTAACGgCcGAAAACCGTGTTACATTCTATCTAACAAAATGATTGAAAATTTTTCCAATATGACAAAAGTGAATTTGATCTTTTTCGGATTATTGACGTTCACCAGGgCTGTATATGCAGAAAATGTGGTACCAGAAAATGAAATGTGGGAAACTCACTGGTCACAAACAGCACAGGACGCCATTGTTGAATTCAGAAAAAaGCAAAaCCTACAGAAACGTTATTTCTTGGGGCCTTGATTCATTCTATGTATGTGATAACTCCTTATTCTATTGCTGAAAAATACTGTGTCAGAAAATAAaTTATTTtGCTCGGGTGGTCAATTTTAACCATGATATTGGTATAAAaGATGTAGCCATTCTTAGAGAAGGGTCCAAATTCTCATTTATAGAGTTGGATCAAGCCGTTCACCGGAATGAATATTTACGAGTATTCGCTATAGAGGGCGACGATTTAGATCACAGTGGAATCTTATTGGGgAGGACTCAGATGGGCGTTCTCATTAAAGGTCTCCTCACTAAAATTCACAAAACCGGAAATACTTTGAACACAACAAGATATCGGACCTGCGTAGGCTCTCTACCTATTATACGtAAGGGCAGTCTCATAATAAttcAAAGACGGCTTGCAGGATTTTCTGACATAAATGTTAatGCTA

>contig00510

TtGcGtgAGAGCTGATTCTTTCAATGTTAGAATACCAATATGGCTAATTAAAGTAGTGCCAAATGTTTTTAATTACAGTAGATAATTTTGGGTAGAACACCGATGTGAATAGTCGCATTCCTTGTGTTAGCTCAAGACATCGTGTGTGAAAATCATTTTAAAAAGTTTTATCGAATTATTTTCAAAGTGCTGTTATTCAAAGTTATTCAGCAGAACAAGAGGTAACCCCCCTTCCAGCTACGGTCTTGCAAAAAaCAACAAGACTATCAAGGAAATATACGTGGTTTTCTTCTGTAGGTCACACGATAAATTGTAGAAATGAAGTTCCTCACGGCTACGATTCTTTGTGCGTTTTTATTTtCTCCTCACGCTCAAGGAAAGGAGAATTCGAC

>contig00511

CCAACTAATCGGGAAGATGCCTCAGACAAaTGACATGGAAAAGACCTTGGTTTATGTTGACTATTGGATTGACGGGAATCTGGCAGAGACTACCCCTGGGGTTTTtATTTCAGAAACAATAATCATAGCTCACGCAAAAGTAGCAGAGAAAAGTGGATCGGATAAAAaTACACCcGGAACAATTTCAGTCCcATGTATCAATGTCAGAAGAaGATATGACGATGATGTTTtGATCCCGACAGCAGTAAATATCGCCAACTCCATTGCTAAGCTAACAGTCATGGAATCAGaCAAAAATTTGTATGCAATATCcTACCCcGTCAAAGAAGCAAAGAAACACGACTtCAACCTCTTAGTGGGTTtGAGCAAGGATCATAAAaTCAGGACTATGTATCCATATTTGCAAACATTTCCGGGGGGgT

>contig00512

ACTCaCGAAAaGTAACTtCGTGCTTGAtAATGGTGGCGTATTCCTAAACATTCCCAATATCGCTTCGTTGGTTAAGATAGGCATACCTCTATCAAGCTCTTTAGTAATGGCAGAtATTTTtCCAGAAGAAGAGACGGCACAAAGAGACTTCGGATCTTCAGGTTGACTTTTCAACGGAAGCGTTGAGCATTCGCCTTTTGGTTTTATCTTAGATGAAACCGATAATTCAGAAAaTGTatGGTCTTtATCCTTTATTATCAAtCCAATTACATATGAGGTTGATTGCATTTGAGGCGGCGATGTTGGTATTAAAGGGAAGCCTTCCGTCAAATATTCACTTAGGTGTTCAACTTCTAGGATTGC

>contig00513

GtAAATTTTTtCCACTTTtAAtAGATTATCTGAAAACTTAGT

>contig00514

CCGGATCTTTATGTTCTGCATGATAATCGTCATCAAAGAGAAATCCAGCTGCTGTCAAGAGATGTTTtCCTGTTACAAAGGTTGCAACAGCCCTCTCAGATCCAGTGTAATCATATATTACTATACAGATTAGTCTAATGAAATGTGCTGGAGTCTTGTCCTCTTTTGCATTCTCACCTTTACCTTCTAATAATGTGAGGTTCGACTGTAGAGCTGCGACCGCAGAAACAACGGTAACTAACAAAAGAGCTTTGAAAAACATTTTACTTGGGTGTCAAaGATCCGAACTAATATTTTTCGTAGATGT

>contig00515

ACTtGCTTATTTGTCTTTTAAGAAGCCAAGTCATGGAGGTTCTTCCTTGCCCGGTCAACGTTAATTTCAGCAACAATAGAGCTGGAAAAGTGACAGATGAACTCGACGGAGCTTGTCAAGCTCTGGCGAAACGGTTGGAGGTTGAGCTGAGAGCAGCAAAGCATGCTCAGCTTGCCTGTGGTGAAGTTTTGCTaCCAGCAGACCTCCTACCTAGGATAGCTGAAGAT

>contig00516

TTATGCGCAAGTTGATTGTCGATCCAATTTGGAGATTCGTCGCAGCTTGTTCTCGTTCGCAAAGATGAGAACTCTCAGTTGATGTATTTGATAAAAGAGAAGATGCTAAGGGACAAAGCCGCGAGGGAGAGAGGAGAGAGACTGCGGATGAGATTGGAAAAAGGCGTAAGAATTTTTGAAGCTCCTGAAGAGCATTTTTCTCGAATCTGCAATTAACACCTTCAGATTAATGTGCGACCTTATCTCGCGT

>contig00517

ACGCTCACGACCTAGAAATGTCCCcGTTGTAACAGGAAGCAAAGgTCTTCAAAATGTAAACATCACCCTTCGTATTTTATTCAGGCCTGTTCCTGAATCGTTACCTAAAATTTACACAATACTCGGTGTCGATTATGATGAACGAGtGTTACCTTcGATCACGACGGAGGTTCTAAAGGCCGTTGTCgCCCAGTTCGATGCAGGGGAATTAATCACACAACGTGAACTTGTATCTcAAAAaGTCAACGATGAACTTACAGATCGTGCCTCGCAGTTTGGATTGATTCTAGATGATATTTCCATTACACACTTGACGTTCGGAAGGGAGTTCACTCAGGCCGTTGAATTGAAGCAAGTCG

>contig00518

ACTTCCtCCAtGTGACAATCCAGTAATTTCTCCATATTTTCTGGTATCTTCTCATCTTCATTCAACACGAAATGAACTTTTTCTATGCTGGgAGCGACATCCAATTTATTAGCAATCATTTtATAATtATCAaGAAGAAAACATAGTCGATAGTAGTAACACACAGAGGCATGTCCTGAATCATTTTtATGTTTTTCACAGAAGCTTGCTTGAGGACAGGATTGACAATTTGAAAGAACCCTTGGGTTGCTTtCAGAGCAAACAACACAAATTCTCGGCTGCAGAAACATCAGTTTCtCAGCAAAATTGAGATTTCTATGCAGTTTtCTCTCGACAAGCTTTATTAAGTTTtCTTTCATTTCGAGCCGAACAGCTGGGTCATTAGTGGTTTGCAAGAAATCGAACAAGTTCGTTGCATTAAAGGCTTTCAATAAATTCTTAATTTGCTTACACAAATCTTTGTGATATATCCAGTGCTGATTCTGATGCTCCTTTCCACAGTAAGAGATCATAAAGCAACGGCCACATTTTTTCAGCCTAATTGAATTCATTTCTATAGAGTTACACACGTGGCAC

>contig00519

ACTCAAACAGAAATAAAGGAAACAGGATGGACTAAACAGAAAAACTAGTAGTTCGATCTGCTTTGCTTGGAAACCAT

>contig00520

ACtCGCCAACTTTCTTTTCAAAGAAATTTGTTTTACCTTCAAGGGAAATGTGTTCCATAAAGTCAAATGGATTTTCGGAATGGTATACCTTTGGACATCCCAATTCAAGGAGTAATCTGTCTGCTACAAATTCGATGTATTGGCACATCAGCTTACAATTCATTCCAATCATTTCTACAGGTAAGGCGTCCGTTAAAAATTCTTGTTCAATTTCCACTGAGTCTTTTATTATCGAAATTACTCGCTCTTGTGATGGCTTCTGAACGACGTGTTTGAACATCAAGCAAGCGAAGTCACAATGCAATCCCTCGTCCCTAGAAATGAGTtCATTACTGAAaGTGAGACCAGGC

>contig00521

AGATTTtCAATtGCGTTAAACAAAAAGTCTTTCTGTTTTTtGATCACTGATGTAAGTCTCAATAAGTAGCGAATACATTTCTGAATGAACATTTTCCATAGCTACTTGGAACCCATAAAAaCAGCGAGCTTCTGTTACTTGAACTTCTTGACtAAATCGTTCCACAAGATTTTCATTAACGATGCCATCGGACGCAGCGAAAAAGGCCAAAATGTGTGAAACGAAATGACGTTCATTATCCGTCAATTTGTGCCAGTCAGGCAAATCCTTAGATAAATCTACTTCTTCTACTGTCCAAAaTGAaGCTTCAGCC

>contig00522

ACTAtCAAATATTGATCAACATGTCGACCAAGGAATTAGGGAATCCTTTGTTTCTTTTCAAGAAAATAATAGGCACAtATGTGGTGGGGCTCGAGTGAAACCCACACTAGTTTTAATTACTTCAAAATGTATGGAAGCAATTGGTAATATATATTGTATCATTTCCGTCGTGATTCCTGCTCTTCCTGATGATAGGCGTGAGGGTTATATAATTGATGACATTATACCCTTGGAAACAAGTGCCTTTCTACTTGTGACTTTGATATTACCTGAAAATGAAAGATCGAGAAACGTAGCAGTGATTCCTTTGCCAGAGCGTGTTCCTTGTAATGGAAGGGAAGGAGTAGTAATAGGATGGGACCAGAGTAATAATACATATGCAGAGGAAGCTGTCAATAACGTTCAATATGATACTGATCGACTTGCTGTATTCGATAATTTTTGGGAAAATATTGATACATTCTCTAGTcAAT

>contig00523

ACttCTTttCAGtCTtCGTtGtATTCCCGAAAGATTTTCTTAATCGACTTACGATAACTTGAGGTTTGGGAATTCCAATTTGAaTGTAAGTTTTGGGCGACTCAAACgAACGaCTCACCGTAGGGATTGTGATATGTGAGAATCAGAATGCTCCGAATATCGAGCGGTCCGAGAAaCCAGGCTCGCTTCTAAGTAACGTAATGAAaTTGAGTTTTAATCCTCGCGGTGTTCTGGACAATGATTCGCTTTTCAGTGTTCGAGACCGAGAAGTTACTTTGAACTTTTTGAAGGCTCTTTTCACTAATTATCCCTGATATATATAATAGAGATCTAAGCTCaGATTAAAAAGTTTtCAAGTTTGAAGCGACTGGGAATTCAAAAGTGTTTtCTtCTTAAAATATTTGTTAGTTTAAATTTtAGACTCGAATGCCTCTGAATTCAAAATCGTGAATAATTGAACCCCTGGTGATCAAAATCCTTTTGATCGCAAAATGTTCAATTCTAATTAACAGGCTTTTGAATTCACATATATTTtGTTTAATAAGTCTTTGAATTTAACGCGCTAAAGTTAATCTTATGTTAAAATAAATGTATAGCGTAttttATTCTGAAAGTTTTTTAAGTAAGCTTTAATTTTCTATCGGAATTTTAAGATGAATGAAaGCTTTTTGTCAAATTGTAGTGACTTTTTCATTGCTTGCCCTCTGTATATGCTTAATTTGCAACaTaTTTTAAAAaTAtAAGT

>contig00524

ATCAAGTTCGCCAGTAGAAGCAGAACACGAAGAACGAGTCGTGTAACTTTTtCCATTtCCTAGTTTAAGAACTTGAATAAGTTTTTAACGTAAGACTAT

>contig00525

GGCTCGTTCCTTTTTCTTTCTTCTCGAATCGAGAAGAGTTCGAACACGAAACACTGGGCACGGATACACTTCACAGAAAATAATAAtAAACACCTTATAATTCCGGTGCACGA

>contig00526

AAACTACATCGGAAaTATGCTTACAAGATTCTTCTCGACGTCAAGGCCTTGTTTGAATCACAACCGAGTTTAGTCGATATCACAATCCCAGATGAAAaTAAATTTACGATCTGTGGTGACATTCACGGTCAGTTTTACGACTTGCTCCACATCTTTGAGCTCAATGGGTTGCCATCAGAAACCAACCCTTACTTATTTAATGGAGATTTTGTAGATCGAGGTTCTTTCTCAGTCGAGTGTATTTTTACCCTCTTCGGATTCAAACTCCTTTACCCGAACCACTTTTTCATGTCTAGGGGCAATCACGAATCTGCTACAATGAATCAAATGTATGGCTTTGaTGGCGAAGTAAAATCAAAGT

>contig00527

ACAGAAATAGTATATCGACACCGTATTTGCAGAGTAACCAAAGTCCTAACACTATTCACAGTAGTTTAAGACCAGTCAACACATCTGAGAAGCGACCAAGCTTGCAGGAGAATAGTTTAGAGCAATTGCAAAGAACTTTGCAGCGT

>contig00528

TGGCTGTTCATATATATTACCGACCAGAGTCATTATTGTCTCAAGCCACAATTGAGACAAAATTGTGTTTCTCTGATGCTTATTCAAAGTAATTAGCCATCTTCCCCCATTtACATTtCCTGGATCTTCCCACATCGGACGTATATTTTCTTTAAAAAGACTATAGTCACaGCCTGGaTTAAGCAATCCGGCATCTGAaGTATAATGCTGGATGcTCCAAAATCCCTCGATGGTGTGAAAAGT

>contig00529

ACAGCTTAATCTTACGGATTGTGTTTATTGCACTTTGCCCAATGTATTCGTCGTtCCTGTAGAGGGCAAGGGCGTGACCTGTAAAGTCCTGAGTGTTTTTGtCAAGGCTAAACTTATTATAAAGAGCGCTCATGCTATTGTTGAATGGATCGAAACCATCCcAAGTCTTGGGATCATCTTCTTGCATATTcTGCACCcAaGTAAGGAAATTCTtGAAGCGCCTTTTCTCGAACATACCcATCAAATCACTGGACAGTGCTTCAGATTGATCTATTGGCACTTTGGAAATTTt

>contig00530

AGCCTTGTAAaCGTAGGAACCTtCGATGGATTTGAACTCCAAGTATCGCGTGACTCCTGTGTGAATCAGAAGCTTCACAAGGATTCCATTTGCCATCAAAAaCTTGGGAATTAGGTCTACGTTCCAGTCACGACCTCGGCCATAACTCTCGTCTGGTGGTGGAGCTTTGAATTTACTAAAGAGGTCCTCCAAAGGAGTGATGGAGGCAGATTCACCACCGT

>contig00531

ACCAAGGACCTGCTTTCATACCCTTTATtGATTCCTTGCCCTCGCTGAATCGAAAAaGCAATGGGCCATTTATAATGCCCATCGTTGATAAATACAAAGACATGGGAACAGTAGTTATGGGTAAAGTTGAAGCTGGAGAGGCTAAGAAAGGACAACCTCTTCTTGTTATGCCGAATAGGACGGCAGTAACGGTTGATCAATTGTGGTCTGACGATGAAGAAGTGACTTCTGTTGGGCCAGGAGAAAATGTAAAAATTAAATTAAAGGGCATAGAAGAAGAAGATGTAAGTCCTGGTTTCGTCCTCTGCGATAGTAGCAACCCAATCAAGACGGGAAAGATTTTCGATGCACAAGTAGTTATCTTAGAACaTAAAaGCATTA

>contig00532

CTGTGCGGGATACAGTGCTGT

>contig00533

TTGGCAAGGTGCTGAAAGTGGTCGAGTAAGTTTGCTCAAAGTCAACTGCTGATCAAAAGGCAAGCTCTTAATATTTATAAaGGAGAAGTAAAAGCAACGACAGCAAGCTGAACTTACGATGCGCGAAATTAGCCGAAGAAATACGTGTGAATCATTGAGTAACTATTTCAAAAAGCCTCATAGA

>contig00534

GACAGCTGCCTCTTtGCTGCCTAACTTCTACaGAGTTTCATTAAAAGTAAaCACTTCGCCAGTCACTCATCATGAAGTTAACAAaCTTaGATGAACGTATCCACGTGCTGGACAACACTTCGAACGTAATGACCGTCATCAGAGACATTGCCACCAGCGGTGTTCAGGAaGAAGCTTTTTACGTTCTCGATATCGGAGACATTGTGCAGAAACATCAAATTTGGAAGGAAAAACTACCTCGAGTAGATCCTTACTATGCCGTAAAATGTAACGATAGTCTGACAGTGTTGGAAGT

>contig00535

AGTCCGTTGGAATAGCCATGAGTTCCTGAGATACGTGTTCCTTTGGCTCtGATGCAATGAATATTtACACcAATTCCTCCGGCAGATTTACTAaTTAGGGCACaTTTCTTCAaTGtATCGtaGaTGCcTTCGaTaCTaTCTtCGGTCaTCGCCACGAGGAAaCAGCTCGACATTtGTTCCcTCATGGTGCAGGCAGAGAAGAGAGTTGGtGAGGCATGAGTGAAGTaTCTTTCTGACAAaTAATTGTAAGTTTCGATGGCCTTCTCAATGTCATCGCGATGAATAGCAACTGCGACTCTCATCAACATATGTTGGGGTCTTTCAACTACTTTtCCATTGATCTTCAGTAGATAACTTCGTTCCAGCGTTTtGAAGCCGAAATAATTGTAACTAAAATCTCGGTCGTAGATTATGGCAGAATTCAATTTTTCCGCGTTCCGTTGAATGATATCGTGACACTCTTTATTGATCATCGgCATGGG

>contig00536

CCTTTTTAGTTTCTTTATGGAGATtCGAAACTGCAATTCTAGCCGCCAGAATTGCGTAATCCGCGTGCTTGGTAGTCATCGTTGCGGCAGTTTCTGCAGCAAGATTATCTAGCTCAACTGTGGTCACTCCAGAGTAAAGACCGTTGATGACGCGGAAAGTGATTGCCGGTGGATCTACGTAATCCATATCCAGACCGTAGCAtAATTTTTGTATTCTTGAAGTAATTTTGTCAAAATGAACCTCCTCCTTGAGACCATTtcGTttCaaGACGT

>contig00537

ACCAAGGAAAAAaCCAGTTTTGTCACCTAATGCTGaCCCGTCAATTTTTCCAGAGAATAATGTTAAGCCTAAGCCAGATGCTTTTATACTACTAGGCCCTGAGATTCTCGCACAGCAAAGAAAACTTTTTCAAGAGAGTAGAATTAATTCACAAGATAGTGTCAACGAAATTAATGAAACAACAAAATTAATAGAACGCAATTGTGAAGGTGAAATCAGCATAAAAAGTAAAAaCCAACAAACAAAGAAAGAGCCAGTGAAACGAAAGATTAAAGTTGTCGACAACATTAGCACTGAAGACATTAAAAGTCCAAAGCAAAGAAAAaCT

>contig00538

ATGTCGCAGCCCATATATCATCAAATTCCAATTTCTTTCCCAATTTGAATTCAACTGATGCAGGCACTAATAATGGTCGCATCCCATGACGGTTGTGCCGGTCCGCACTTCGTGGCGATATACGCCCAAGCTGTCCTTGCTCTCCGCAACCACACGTATAAACACATCCATGATCGTCAAGCATGACGAGGTGATCAGCACCAGACGCTAATTTGACTATTTTCGCAAATGGTATCATCTCTACCGGAAGCTTTTCGTTTCCATTTATAGTAAGTCCCATTGCACCGTGCGAATCTCGAAATGaTCCCcAAGCAAACACACGGCCATCGTCCAAGAGTGCAGCACT

>contig00539

ACTATtCGATTTTtGACCGAAGCCaCCATAGGaGGAtAtATCATAATTTTGGtCGGCTTATTTGCTGGAGGACTTAtGGGAACTCCAGTGAATCGTCGCGTGGATTTGTTCTACAGTTTGATTGGTTGCGCTCTGTTCATtGtCATTGGAGCCCTAAATATCCATGTGTTCAACAATATGTGGGgTAGAACAAGATCTATGCGAGATACAGGTCTTTCTAAAGGATCATTGGCAATTTTCGAAGGAGCTCTGTTCCTTATGGATGCCATTCTTACCTTCAGGGGAGAGGCATAAGAATCTTAATTTTCACTTTTAGGGACTACTCGAAAATCCAAAAGAAATGCTGACTCCATCTTATGTTGATGTTGCTGATCTgAAAAAaTTtCAGATTTTtAAGAtAACTATtaTTTTtCGATAAATCACATTTTTtAGtAGCAATGTATTTTGCTACTAAAAGGCGTCAGTTTACTTTtCAAATCTATTAAAATCAGAAAaTTtATTtCCTGTGTAAtGGATTGCATTTGGCTTTTTTTGTTCTGCTCTAAACATaGGAAAACCGATTGAAATAGTTACTTtATACAAATAtCTTCCCATGGGAAAGCATATCTAAGCTCAATGAATTTtCAGCGCTCTAGTTTtCTAATGTtCCTCAAGTTtgAaGGTTTtACCcAGACCACGTGACAACAAAGAaCAAATAGAGGAGACAAAATGCGT

>contig00540

GTGTAACTTCCACCTGGTGTATTACCCGACTCATCGAGAAACGTCAGaCCAaGTTGTATAATCCTTAGTAAATCCACATTGCAGCGCAAAAGCTGATATTGGTAATCTGCACTAGTTCTGAATTCTCCGATAGGCCTTGCGACAACTCCAGGAAATTCTGTGTCCATTGCTaTaTATTgataCTGT

>contig00541

CCGGAAGAAAATGATCAACAATTTCTTTTCCTAATCAAGTTTCCATAATAATAGTTTTACACCcATAGAAAGGATTCAAAATAACATAGATTTTTTCAGTGATTCGAAAAC

>contig00542

TCCGGCAGGATACCAACAACAACCTGCTGGGC

>contig00543

CGATTGCAATGGGTATCAACTTATCTACCCTAGAAAAAGGACTTCCAGAAGGCACTCCAGTAATAAGGGTGATGCCAAATACTCCAGCTTTAGTGGGTTGTGGAGCTACTGTTTATTCGAGAGGAAAAAATGCCAGCGAAGAAGATGCTCAACTGACTGAAAAATTATTCTCTGCTGTGGGTATCTGTGAAGAGGTTCCAGAAAATATGATTGATCCTGTAACCGCATTGGCTGGATCTGGACCTGCATACATTTATATGGTTATTGAAGCTCTTGCTGATGGTGCTGTAAAAATGGGTCTGATGAGGCCACTCGCTTATAAACTAGCCGCACAAATGGTTCTTGGAGCAGGAACCATGGT

>contig00544

GACGCTAGCCGTCCCAAGCCGTCTTGACGCCACGTGTCTTGCTATGTGCCTCATTTTtACACACAaCAATCTACTaTCTATAGATCAATAaGCCTCGTCAGCATTGGTCCTCAAGTCTGAGGCAGaGGAAAACCGTTTGTCCAGTCTGACTGGAGATAACCTGGGTTCTGAGGTGAGAAGAGGTGCTGGATACGAGGACGCAGCCG

>contig00545

GCTTTTTGTCGGCAATGCGGTCATTGTCTACATCATcTTCCAATAC

>contig00546

CAGGAACGCAAGGAGAAGAAGTAGC

>contig00547

GAAGTcAAttCTTCGTGATGCTTTAAGCGATCTAGCAATCCATTTCTTGGACAAAATCAAGGTTATGGTTGTAAAAGACATTGAACGCGAAGACATCGCTTACGTATGCAAAACTCTAGGTTGTAGACCTATcGCTTCCTTGGAtCACTTtACTGCCGAAAaTCTCTCTAGTGCTGAATTGTGCGAGGAAGTTATGACTGGAAaTTCAAAATTCGTGAAGATTACTGGAATTCAAAATCCAGGCCGCACCGTTACAATTGTTGTGCGTGGAAGCAACAAATTAGTTTTGGACGAAGCAGAAAGATCATTGCATGATGCCCTGTGCGTAATtCGTTGTCTGGTTAAGCAGAAGGCGtcGATCGCCGGAGGAGGTGCTCCAGAAATTGAATT

>contig00548

AGAATATCTACTTCCAGGTCTAGAATGAACAATAGCATAGTGATCGCTATTCGTTGATATGGACCTGGTGGAaTtCATTGTGgAACCACCGGAAGCATAGCCAGGTTtACCaTTCAAAGAaTATCTTGATCCGTATTGAGATCCGTGCCAACTTCGATATGGATCATAATCTTCAaTGATCGAAGGTtCCTTTtCTATTACCTCTGTTCGAGTGCAGGtGCATGACATCGCAGAGCAAAGACATAtGTTAATAATAAGCATAATGAGAAACAAAaTACCAaGGGCTACAGCGAGCCATAATAGCCATACAGGATGGACGCTACCATCTTCGTAAGACGATGCTaGT

>contig00549

ATTAACAAATATGTCGCGGGGAAGTAGTGCTGGTTTTGATAGGCATATTACTATATTTTCGCCTGAAGGAAGGCTTTATCAAGTGGAATATGCTTTTAAAGCTATTAATCAAGGCGGACTCACATCCGTTGCAGTAAAAGGTGTCGACACTGCTGTCTTTGCTACTCAGAAAAAGGTCCCGGATAAG

>contig00550

ACTTTCAtCGTCTGTCGAGAGTTCACTTATtGtGAAGGATTGAACATTGTCTGTATTATTACTTGGAGGAAtCAGAAAGTATCCTGGATTATTCATGAAAaCAGGTGGCACGGTGATGAAAaCTTGGTAGGCGCTGTGACACCAGAAGAAAACGCAtCCACTGAAGGTGAGAGCGAGTAAGAAGATGAGGTAACATGACGAaGATCCAATGGCTGGTAGCCGCCTCTTATCGAGCATGCTGACAaTAACGTTtCATTCCACTCATTCCAGCCCTTA

>contig00551

CTTCACGCATGCTTtCTTGACAGATTCCATCTCGAAAGGCTCAAATATTAAGCTCATTTACACAATATACTAAATACGAAGATGGAAAATAATTTAATAATCTATCACTATCATATGCAACAAACATTCCGGACTAAATAACACATTATTTTTTCTATGTTAATGGAAAATCTTTTGTCAAAGAACACGAGACATGACCAGACATGACTCTCACTTGCCGCTACGCGTGGTCTAcaCcAaT

>contig00552

ACGTCTTTtACTGTCAAaGGGACACTCTTGCTACAGACCCAGACGTGATGGAGAACGAAAGCGTAAATCTGTTCGTGGTTGTATCGTCGATGCTAATCTTTCAGTATTGGCACTTGTTATTGTCAAGAAAGGAGACCAGGAAATTCCGGGATTGACCGATAACAACATTCCTCGACGTTTGGGTCCTAAAagAGCAAGTAAAaTTCGTAaGCTCTTTAATCTGACCAAACAaGACGATGTTCGTCGCTTCGTCGTAAAGCGCCCAATCCAAAAGGAaGGCAAGAAGGAACGCTTTAAGGCACCcAAGATCCAGCGATTGATCACACCACAAACGTTGCAGAGGAAAaGACATaGgATaGCTCTTAAGAAGAGGCGTTGCTTGGCTCGTAAaGAACaGGCTGCCGAATACGCAAAACTATTGGCTCaGAGACAAAaGGAAGCGAAAGCTAGGCGACAGGAGgAACTtAAGCgAaGGCGAAGTGCTTCCATGCGTGATTCAAAATCCTCTAACCAATCCGCTCCTCCCGCGAAAAaTGAACCgTCGTCA

>contig00553

TTTTACTACGGgCgaCAAAGTGTTTGCAAaaGTTCGAGGTTATCCGCCATGGCCAGCCAAAaTAATAGAGATCAGCGACCcAAACacGAAAAaTGTAAAATaCAATGTTTTCTTCTACGGCACTCAAGAAaCaGCTACTTGTAAATTAGAAGAACTTTTTGAATATGCCGAGAACAaGGAGAAATTtGGCAAACCGATTAaGCGAAGATACTtCCACGAAGGTTTACAGGAACTTGAGCAGGAACTCAAAAACGATAAAACTCTTGTATCTTCTAATAAAGAGAAAAAGGtTGCAGCACCTGtAGTAGCATCACCACCAAGAGaTGAaGAACCAAaTAAAAAaGCTGAaTTGgAAaCgAcAAaTGTGGACAATGTTAGT

>contig00554

CGCTTTGGTCATTGATGAAGGA

>contig00555

GGATGTTCCTGAGCCAAAAAAGAAACGAGGACGCCcAAAAGACATGACCCcGAAGCAAGAACCAGCTTtAGACtCCCAAGGAGAAGAAACACATGAAAAaGAAGTGATAAGTCGTAGTGGAAGAAAAaTTAAGCCTAAGCGCTTCGCCGACTTTtCAAgCTCAGATGGACCCGAAACACCTCTGGAcAAAAaTG

>contig00556

ACCTCTTCATACCTTGTTTGATTTTACGTATTCGTTTtCGCAGCACCATATTCGACTGAGGCGGAGCTACGTTCGATTCCAGAATG

>contig00557

AGtGGAAAACGCGGTTTATCAGTGTTTAGTGGAAAATATAAACTTTCGGTATATAAGACATCA

>contig00558

ACtCAaCAACAGAGACAACCGGCCACTCCAAACtCTCAAGGATCATTCCAAAaCCAACAGGGTCAAAACCAAAaTTCGTTTAAGGGCCaGCCGCAGCAGACTACTCTTCAGCAGCAGGCAAaCCAGGCtAGGATGCAACAGCAGGTCAAAaaTCCCCAGCAGCAACAGCAAACTCAACAaCAaCCTCAGCAGCAGCAGCAACAaCCACAACAGCAGCaGCAACAACCACAACAGCAGCAGCAGCAACTGAAGCAACAACAACCAGCAGTTGGGACTCCTCAGACTCAGCGCTTGAAaCCCATCCTGAAGCAGTCACCTACGACCCCTGTGGCTACACCTCAGGCAACTCCTCCTCAACCACCACAGATTCAAACTCCGGCTCCAACTCCTGTTGCAAaCAATGGCGCTGAGGTGAAAGATGGTGAAAATACGGAAGGAATGGAAGTTCCAGAACAGCCAAGATGGAGACGAAAGACTCCTGGATTGAAAGTCAACCGGAAACTGAAGCGCTTACGCATGAACCAACGTCTCAGAAGAACTCTTCAGCCTAAAAACGCGATCATGGTCCTCAATGAAATGAAAACTGGAGTGCAGTTCACCTTCCCAGAAACTCAATCTTCTTTGACTAACTCTCTATTCCTTGTTCATGCTGAGATTGACGGAAAGACTTACGTCGGTCAAGGTCTATCAAAGCCTTTAGCTCGACAGAAtGCAGCTGAAAACGCTCTTAaGTGTCTTCTTCTtGAAAAAatGACCGcTGCTTCTTtGAAaGCTCGtAtGGATGCaGAaTCGGaCGGAGT

>contig00559

GAGTTGGCTGACACTTaTTACTTGTCTACCTAAaGTTAAAAACTTTGCAATACGCAAATTGTCTCAAAGGAGAGTGGTTTGGGAATCATAATTAAACACTATAACAATAATCTTCAATTTGAATTGGACTCCAGTTTTAACAGCAGAAAACCGCGATACATTCTATCTAACAAAATGATTGAAAATTTTTCCAATATGACAAAAGTGATTTTGATCTTTTTCAGATTATTGACGTTCACCATGGCTGTAGATACAGAAAATCAAATGTTGGGAACTCACTGGTCACAAACAGCACAGGACGCCATTG

>contig00560

CGtCTTAaTCTCAAAGAATACTATAAatATAAAAATGTATAAAAATTAagAaTACAGAATTTCTACCTTTAAAAGTTACTCaGAATTCAAATtTcaTCG

>contig00561

GCAAATGAGTAGAGCGCTTGGCTTCACAAGTTTGTTGGAAaCTAaCAGGCTtATTTtCGCTGTCCACATCACTGAAtCCCATctgTTCtAGTCGCTTTTTACGATaAaTTCATAGTGACGACAGTGTGTTTTCTCCCTCATGTCTTCCATATTGGTCCTGATAAGCATTTCTCGCAGTTTAACAAAATCGCAGTGCGACtCGTTTTCCACTTGAACTGTGCCCCATGGATATTGACGGGATCTCATCATTTtATTTCCAACTCGAACAAAGTC

>contig00562

CCCGCGTCAGCAGTTTATCCATCTAAAGATGAAGATGGAAATCCACTAGAAACTCAATATGGTCTTTCcACTTATAAAAACCATCAAACTCTGTCCGTTCAAGAAATGCCTGAGAGAGCACCAGCCGGTCAATTACCACGTAGTGTTGATATAGTTTGTGATAATGATTtGGTGGACGTTTGCAAACCAGGAGaTAgaGT

>contig00563

TTTTTGTGTCGATTTCGGCATGACAGT

>contig00564

CAGATGGAGGTGGTTCATAAGGTGGAGGAGGTCCATTTTTATTCATAGTGTTTGCTTCCGCCAGACAATAAGATCACGTCAACAATCGCACAATTTCAATTGCTTTTTGTGAAGAAATTCTTTGTTTTTCAAAACAAATATTTAGAAAATTTATCACTATTCAACGATTGACCAC

>contig00565

TCTTTGGATACCATCCAAAAAATGCTACATAACTAACATCGCTTGTCTTCTCCATAATAAATACGATAACAGAAAATCGAAAACCTACTTTGCAAATGGAACGGAATTTGCAATTCGATACGGGAGTGGAAGTCTCTCTGGTTTCTTATCCACTGACACTGTTAATGTAGCTGGAGTTGATGTTGCCAATCAAACTTTTGCTGAAGCGATGAGCGAGCCTGGATTAGCATTTGTTGCTGCTAAATTCGATGGTGTTTTGGGAATGGGGT

>contig00566

AAAAAACTGGAAAAAACCATTAACCTTGTTTTGAAGACATCTTATGACAGCTGTCAGATAGAAATAATATGGTAGAAAGTTGTGTTCACAAAGAATAATATTTCTCTACATTTCTTCGCAACGATTTCGAGTGATTTTACGGAGAGTCATTAGTATTCAGTTTGTTCAGACTCTAACTCAAAATATTACTTGAGAAGGATGACAATGGGCGATGAGACTCCAGTTACATCACTTA

>contig00567

ATTTAATtGCtGCGAAGTGCTATTTTtACCACTCAAGaGCCTACGAACTAACCAATCAACTtGACAAGATCAGAAGCTTCCTGCACTTACGACTTCGCACAGCGACACTTCGTAACGACTTTGAGGGTCAAGCGGTGTTGATTAaTTGTCTTCTGAGGAaTTATCTTCACTATAaTCTTTACGACCAAGCTGACAAGCTTGTGCTTAAGTCGaCCTTTCCCGAAaCTGCGAGCAATAATGAATGGGCTCGTTTTTTGtATTATCTAGGCAGAaTCAAGTCtGCAAGACTtGAGTATTCTGCAGCGCATAAGtATCTCGtCcAGgCGATGCGCAAGgCACCACAgACAACCgCCGTTGGTTTtCGgCAGACTGTGCAAAAACTCGCAGTAACCGTAGAACTTCTACTGGGTGATA

>contig00568

CAAATCTTTCGCCAGGCGGCATTGCGACGTGCTCTGGCACCTTACTTTCAG

>contig00569

ACTCTTCAGGTGATCTCGAAGGATCGAAAGATCTCAGCAGCATCTCGATTTCCTTTTTCATAGCTACAAGTCTTTCCTTCTCAGCTTCAACATCTCGTGCGAGTCTCTCCTTTCTCTCTATTTGCTCCATAATTAATTGTCTCTGACGTTCCGTCACTTGGTTTATAGTCAAAGGAACTAACTCATTGCTCGTCGTTTCCCTTTCCTTTTCTTTCTCTGGTGACAAAGATGGCGATCTAATCACATGAGGAGCCAACGGAGTCGTTGGCAGACTTGTAGGCGAAAAAACTGAATTAATGGCGGATGCCAAGGGAGTCGTCGGTTCACTCATTGTCATCGTCGGCCTCGACAATTGACTCGGAGGTCCTGCAGCTCTTCGAAGACTATTATATTGACCAGAAGGTATGAGACTGCTGGGTCTCGTGGGAGCCATTGGAGGTCGAaTCCTAGCGGCAGCGACACTTCCAACGTTTCCAGGACCAATTCTAATCTGCACTCTGCTCT

>contig00570

GGCGTTCCGAGGACGGTGGACGTaGAGTTAAACTAACAGAAGTAAAACTCCGaGTCTGATCGCCCTGGATCGAAGGATTTACATTCATGTAGGGTTCCGTATAaTGCGGTCGAGGTTCGTAAATAATGTCGCTTCCTCTAGGtATCACTGGTGGGGATCCAAATCCAGtGTTTTTGACAGGACTGCAGGCCA

>contig00571

ACCATCGAGCAAAAAaGTTCAGTGGGCTCGTGAGCATTTGGAAAAGCCAGTGCCAGTTTCTAACGTCTTCGCACCTGATGAAATGATTGACGTGATTGGTGTGACCAAGGGAAAGGGATACAAAGGTGTTACTTCTCGTTGGCACACAAAGAAACTTCCAaGAAAaaCCCaTAAAGGATTGAGAAAGGTCGCCTGTATTGGAGCTTGGCATCCAAGCAGAGTTTCCTTCACGGTGGCCCGTGCTGGTCAGAAGGGATACCACCATCGAACGGAAATGAACAAAAAGATCTACAGAaTTGGTCAGGGAATTCACACCAAGGATGGAAAGATCGTGAAGAACAATGCATCTACCGAATATGACCTTACGGAGAAGACCATTACACCGATGGGA

>contig00572

ACAtACGCACCGCCGCATCGCACGTAGCCTGACTGCGTCTAGTGACAAGAATCCATTTATATCGCACCGATCACCATGGCAGAGGAATATTTATACGGAATTACCCTCGAAGGCCCTGACGCCTCCGAAGTATGGGACCCAGAGCACAGGGCAGAAGACCCCGATGGCACCAGTCAGCACTTCGGTGGCGACCAAAAACTCATTATCAAAATGGCACTCTTAGGCCCAGAAGCTAAGCCAGGTGAATTGAATGTTCTCGAAGTAGAAGCCACAGGACTTAAAGGACCTATCAAAaTTCCAATAGCTTTACTAGAAATGGGAAAAACGTCACAGATCATTCTCGATTTAAGTTTTCC

>contig00573

CACACATTGAAGAGTTCGAAGATATGGAAGATATGGAAGATGAGATGGAGGAaGAAAACATTGATGACGAAGATGATGAGAAGGCACCACAAAAGAAAAGAAAGCATTCTGCGGAGGCTAAAAAAAaTGGcACCAAACGCTCAAAGAtGGACGAAGCAGTAGATAAGTGATATGTTAGGAAATCCATTCCGTAATATTAATTTGACTTACATACATGGATAAGTTTtACATTTATTGATAAATGATAAACGTCACATTAAAGCATCAAAGCAGCAAATCTGATCGTCATAGAGGATCCTAGATGTATCTCTCGtCTATCCTACGTCTtAATTTAGATTTGTTACCTCCA

>contig00574

ACCCTtGtGCATTGGCCCCTAAACATATTAAAaaTtGTTTTtGtATTTTtATTtaCGAAACACATaTTtCAAtAGATCAGTATAATCTGTGTTTtGAAAaTCAAAaTATAAAAaCAATTTTTtCTATAGATGCAGGCCAATGCGCAaGGgTTCAAAAATTGTaTGTaTCGTTCTTGCaTCAGGGCCGTCAATTAGGGGGgCTGGaTCAAATAACTATATaTCCCCTTATTCGATCCGgCACCtCAATtATACATAATGCAAGTTTtAAtCATGTGTCGTTaGTATTTATTACAGAAAaTCCAAAAGAAaGAATAATCGGACTGAAAaTGTAGTTATTTATGAaCATCGTATTGTTTAAGAATTGACAAGAGCGTTAATTGtATTTTtCCTtCTCAGTTATTGAGCAAACTGATCAGTTTATGAGACCGTCGATTGCGATTCTG

>contig00575

AGTTGGTACTGAAGAAGTCGAAAGAAGCACAATGAAGTTCCTTATTGGTTTTTTATCATCATTTATTATATGTATTGTTGGAGTTTGCACAGCTATATCAATTAAGGAACCCGTTGCGGCTGAAATAAAGGAAATATATGGATTACATTGGGATGACTTTCAAGAACAATGTAAAAGTAGTAATCCTGTCCTCTTTGATGAAATTATTGAAATGGAAAAGCAGTGTGCGAGAATTCTTTCTGAAACTACACGAGACACAGTTGATAAAGCGGTAGCGACGCCTGAAGTCACTGAAGATGATTGGAATGAATGGATTGACGACAAATGTTTTTtCAATGATACTTTAATAGACAATTCTGTAAAAATAACTGAAAAAGTTAAAGAATGTTTAGGCTACACGTTGGTTGAATCAGATAAAACATCAACTCCAGAACAACTTCTAGACAGAATCTGCAACACTATAAAAGATTTGGTC

>contig00576

ATACATTCCGAGACCTTTGCGCGCCAAAGAAGCCTCGAGACCAAAACTACGAGGAAATTGTTGAAATAATAAAACG

>contig00577

AGTCGTGAATTGAAAGAGGCTGCTTAGCTATGTCATTTTTCGCGGATCTCGGGAAGATTTGCTCGTAAAACGGTGAGTCTGG

>contig00578

ACTCAAGTGATATTCTACTCCtAATTAGAATTTCTTGACCACAtAGGTTGAtGTTTTTCAGTAATGCTGGGGTATAAACGACGCTATATAACAGTAAGGATAACGCAGAGAATCATTTAAGGTTAAGGCTAATTCGTAAATTAAGGTTAATTGGTAAAAAAGCGAAATGGAATCGGTTtCTGATAAGCAAGTTAAATCTTCAAAACTCAGGTAAATGCTGATATCTCTTGAAGgAGCAATGAGTTCTTGAAGCGTCTTTCAATACAAAaTTAGAATAAACTTTAAAaCGCTGTGTTCATATCCAAGATGATAACATTATTCTGTTCCAAaTTAAAAGGATAAGTGTTCCAAACGATGAGTAGAAAaGTTACACTTCAATCAAATTTAGTTAAATTTGCACTTAGAAAAGTCCATTTCTGGATGTTGCTCCAT

>contig00579

CGAGTCGCAGCAGTTTCATTCTGCAATTTGCAACGCTTCAATTTACTGATTGCTTTACTTtCCTAGACGAGAAAACTGCTAAGAaTAaTTGGAAaCTaCCCtGTTTTGCAGATGAATCCGATCTAACAAGTAGAGTtAGAAAACTTTGCGCTTGGACCACCAAtACATCCTGGTAGCCCATTCAATGGGTGGTGTTTGCAAAATGCTGTTAATTAATTCCGCTGTAAGTCTGATCCGGTATGTGATGGTTGTCGGATATGCGGGCGCATGTCGCCTACCATCTCTACAATGCGAGGCCTAGGAGTGATATGGGGTGACGTAGGTGGACGGGAACAATCCTCGCCTGTTTCCGATTTCGCCATGCCACCAATGTTGATCCGTGCGGTCTGTAACGGTGATCACATCTCCACGTCTGAATTCAAGCTCCCCTGGTTCTTGTGGTGTAAAATCGTATAAAGCTTGt

>contig00580

AAtGATTATACAAGGATTGATTTATGAATTAATCATAAAATAATGTTCGAACTAAATTTAAATTCGAACTCGAGAGTATCCAGACGGCCTAACTTCAAAAAGTTTTCTCACAAAATAAACTTGCACAACCGTCGTTGCAATAATTACAAGCAACTGTGCAATTGACCAATTTTGAACGTATGAATTATTGTCCAGAAGCAAaTTTAaGTCTCGTGCTTCGCGACCACGACTCATGTATTGAATCTGCAGCATTTCGTTAATGTTTTTCtCTACATGTATGATTGAATTCGTAAAATTTT

>contig00581

GTAGTTCTCTTGTGAAGTGCTCCCATTGATCG

>contig00582

GATGGATTTCTTACAGCGAAACCAGCTTTTCCATCACCTCCTCTAAGAACCTGGAaGCTGACGTAGAAAGTGGCACCGGGATTCACATATTGAAaGTAACAATCCTCCTTTCCCGGgTCAATGTGCACCTTATAATCCATTGCCACAGCCGGCAAAGTTTCATGCCACGGCTTGGTAACTTCGGTTTTCGCCAGGGTGACTATCAGACCCGCGAAAAAAAAAaCACGCACGACTAGAAGCATTGCATGAATTTTGTGATCATCACGAAaCGAaC

>contig00583

ACTGGAAAGGtATATGTTACATCGTCTCAATATTCTTCTGCTCCTTCTCCTTCACCTTCAACGGAGTCCATGCCGACTTCTTCATAATCTTTTtCTAGGGCAGCCAAGTCTTCACGTGCCTCGGAGAATTCACCTTCTTCCATACCTTCACCCACATACCAGTGGACGAAGGCACGCTTGGCATACATTAAGTCAAACTTATGGTCAAGACGAGCCCAAGCTTCAGCAATTGCTGTAGTGTTAGATAACATGCAGACTGCACGCTGAACTTTAGCAAGATCGCCACCGGGAACGACTGTTGGTGGTTGGTAGTTGATTCCAACTTTGAATCCTGTCGGACACCAGTCAACAAACTGGATTGTGCGTTTGGTCTTGATGGTAGCAATCGCAGCATTAACGTCTTTTGGCACGACATCTCCTCTGTAGAGCATACAGCAGGCCATGTATTTTCCATGTCGAGGATCGCACTTGACCATCTGATTCGCTGGTTCAAAACAGGCGTTTGTAATCTCAGCGACTGAGAGTTGTTCGTGATAAGCCTTCTCTGCAGAGATCACAGGtGCGTAGGTTACCAaGGGGAAGTGAATTCTTGGaTaTGGT

>contig00584

ACTTCATGGCTAGATGTAATCTGCAAAGTATTTTGCTATATCAAGAAAGAAAaGAAGAGATCGATGTGGATTCAAAaTGCCAAGGTGGGTTCATAACAAACGAGGGAGGACTCGGAGGAGTTTTAAGTAGCTCTATAGAATTAAACGTTAAGAATGAAATAGTGTTCTATATTGTATGGTTGC

>contig00585

ACAAATTtGGAGAAGATCATTCGACACACCaCCACcACCAATGGAACCAGATCACAAATTTTATGACAATATTGTCAAGGATCCACGATATGCGAATGGACCCAAGCCTGAAGAATTTCCAATGTTCGAATCGCTCAAACTCACCATTGAGAGAACTCTGCCTTACTGGAATGATACTATCATtCCACAACTCAAAGAaGGAAAGGAAatAATTATTGCTGCTCACGGAAACAGCTTGCGAGGCATCGTTAAACACCTTGACCAGATGAGCAATGACCAAATCATGGGACTGAATCTTCCGAcTGGTATTCCATTCGTATATGAATTGGACGACAATTtCAAACCACTTGTTTCGATGAAATTTCTCGGAGATGAAGAGACCGTAAAGGCAGCGATAGCTGCTGTTGCTGCCCAAGGCAAA

>contig00586

ACTCtGCGACTCCTTTTGAATGTCTTCCCAGACCTAAATCCCTCTTTCTTTCTTTTAAATAGCGAACtCAAATTCTCAACTGATAATCTCCCaGAATTTGGAACTtCTAAaGGTTCACTTTTGAGGTCCAGTCGTTCTATTCCAACATTATCAACACTATCTACGATTTCTCGTTTAATTTTTAGTTCAAGAGCTTCCACACTTAAACTATTTCTTCTGCTTGATTTTGGAGTCTTAATCCGCTTTCTAGAATATTCTAAACTTTTTGACCGATTTTTTTtATtCTTTccTTCAGCTtCGACTTGCTTCCTGGGCcTTAAAGACCGAAGTGGTTTTTCATCAATCTCGACTTTtACTTGAACGTCGCTGATGATTTGAGGATTAGGTTCAAAGCTTTTCGACGTCGTCTCAGAAGAATCTGAAGCGGGATTTTtCTCTTCGTCCCGACGtAATCAACGGAtCGTTTtCTGGGTCTAAGTTTCCTTGAAAGGCTTCCGGGTTTCGATAAAGTAGATTTTGCAAATAGAGGATCTACTTGAGATTTCTCTGATCGCTCAaGAACCTTCTGGGgATCCTCAGAAAaaCCAAaGAGGTTCTTTCGCTTCTTCCAGAGTATCTCAGGCTCTTGGGGTTTTTttGTtCcGTTCAAAtGAAATGGGAGATTGGATCTTTTCCTAAGGTCGAGTTCAGAGTGCGtGCAGTCTTTAGGGATAGcTtGACATTTTGGGTCAGCTTtATCACAGTAACATtGACAATGAGAAGTATTCGCAAAAGACGAGT

>contig00587

CAACTCGTAAGGCGTTAGTTAATAGTTCCCTGCACTTCAACCTCACGGCGTCAGTTATAGTTGGAGCTGGGAAAGATGTTTGTTTCCTCTTATCATCTTTATTGTTTTTTtCCTCCTTCATCTTCACATCACCATCGATCGCATCTTTGTCTTTCTTTGAATCATTATCATCCTTATTTCTTTCGGATTTG

>contig00588

CTtCTTGGAACTGCTAGATGAACTGGAATCCTTACTTTtATCTGaTGAtAAACAC

>contig00589

AATTTTTAATtAAGGTTTTTGATAAGGACATGACTTCCTCATCTTGACTCGATTTTCGCAAAGCGTTAACAGTCATTCCGATTCTAGTAGTCGTTAGAAGTTCCAAATTCATAGGCAACTTCTGTAAAGTTTTAAGAAGGTCTAAT

>contig00590

AAATATCCACAAAAAATTGATCGAACCCCTt

>contig00591

AGTCTTGCGTTGTTTATGATTGCTTACAGATCTATCAATTTTtACTCCCCTTTTTTCTATCATTATCCTTATAGTGTGTGCATATTCATTATGCTATAAAATGGCGATGGGCGCAAGATTTTCTTCTCGATAAACTAAGAGTATACAGGACAGTTTTTTAAAACCAAGTAAAGGGTAGTAAACAGACATTTTAAAGAACATTTTATCAAAATGAGTTCAGCAGTAGAGAATGGAGCCGGCGATGGTGGCTCTAAGAAAAAACTATCGAAGGGATTTACCAGAAAAAaTCGCAGTTGGAGCATATTTTGCTTCGACCCGACACTTACATTGGTTCAGTCGAGTTTGTCACGGAACTGATGTGGGTGTATGACAAAGAAAAGAAaTGATGATCCAGA

>contig00592

CAAACTATGATGACGAAGAAGAAAAAGTAACTGGTGGCAGAAATGGTTATGGAGCAAAATTGTGTAATATTTTCAGTCATCGCTTCACAGTGGAAACTGCCTGTAAAGaGT

>contig00593

aCAGAATATCATCAAATGCAGGGAGGTTtgTCTCTTACCTCTTTtCTTACAAATCGTTGAAAATATATCTAGTtcAGACTTTTtCGGATTTtGACACTTTTTtCACATAAGTTACCTCAGAATCGTGAGGAGGtGGAGGTGTAAAAATAGGACTTCCATCTTCATCCACGTATTGGgCATACTTGAGTTCAAAAaCCGGTTCGAAACCCAATCgCTGACACAATTTACCAGTTATTGCAGAAGAGCAGTCTACTCGTATTAAATCAAAACCTCTGGTCTtACTGATTTCTATCGCTTTTtCAAAAAGGGCTTTTGCTactCCCTTTCCTCTCCAATTACTATCCACAGAAATTA

>contig00594

ATTTTGACAATTCaTTATGTAATCCGGCTCTTCATTAGGAGTAGGGCCCATCATATCATTCAAAATGACACCAATGATCGATCCATTATCAGAAACTGCCATAAAACTTAAATCTTTATTCATAGCAGACAAGCTATATTTCTCGAGCTCGATACAAGTGCTATTTTCACCATCTGAGATCAGTTTAATAGTTTGATTTAAAGGCTCGTCCCTGAAAAAGAACTTTCTCAAAAAATTGAGT

>contig00595

ACtAcaGGAGTAGTAGTATTAGCTGGTGCTCTTATGGAaCAGGCCCAACAGTTACTtGATAAaGGAATTCATCCgATCAGAATAGCAGACGgATTTGAAGTAGCTGCCcgaTGTGCAACAAAacATCTCGTCaGCATTTCTGAAGACTTTCCAGTCAaCCCCAATAACTTAGAACCTTTAATCAaGTCCGCGATGACTACCaTCGGATCTAAAaTCATCAACAAATGCCATCGaGCAATGGCAGAAATCGCCGTCAATGCAGTCATGGCAGTTGCtGATTTgAAAAaaCGAGATGTAAACTTTGAACTTATCAAAGTGGAGGGCAAAGTCGGTGGCCGATTGGAAGATACCGTGCTGGTTCGCGGAGTtGTTATTGATAAAGATTTCAGTCATCCTCAGATGCCAAAGAGGTTaGAAAaCGTAAAaTTGGCAATCTTGACCTGTCCGTTTGAGCCACCGAAGCCCAAGACAAAGCATAAGCTCGAtGTCACTTCCGTCGAagACTATAGGGCTCTACGCGCATACGAGAAGGAAAAGTTTGAAGAAATAGTAAACAGAGTTAAGGATGCTGGGGCAACTCTAGCTATTTGTCAATGGGgCTTCGATGaTGaGGCAAaCCATCTTCTTCTCCAGAAACAGTTGCCTGCAGTAAGATGGGTTGGAGGCCCAGAAATTGAGTTGATCGCTATTGCGACCGGTGGCAGAATCGTTCCACGATTCGAGGAACTGACACCAGAAAAACTTGGATTTGCTGGAGTTGTGAGGCAGTTGTCTTTCGGT

>contig00596

ACTtGGCACTTGCTGTTTTTAGAAAaTTGTAGACTTCTTTGTTAGTTTCCTTGGCACGTTGCAAGTCTTGTTCACCTCCAATCTGGgCTTCAATtAAGTGATCGCAGTGAaTCGtGGAAGGAACGgcAACCTTAGGTAGACCAGAACTAAtGAATTGAAGCAtAGCCATTTGAGCTGTAGCATCTTGCATAGCCACGCGATCTGGCCGCAATCGTAGATAACTCGTTCCTCTTtCGACATCTTGCTTGTTAGGTTCATCGA

>contig00597

GACAATTATCAGTTGGGAAACTTTAAATACGTCGAAATGTTtCTTAAAaGTGTTCTAATATTTATCGTCGGCATaGCTGTATCAGTTGAAAGTGCATACCCAGAAAATGTAgTCATCAGCAGAGATATTGCAGTTCGTATATTCTATGGCCAGGAGCATTTGGCCAACGGAATACTAGTAAGtCCACTGGACGTCaTGACTCCaGTGGTTGGGTCAATTAAaGCAAGTGaCTCGaGAGTGTTTACCATTCAATATGGGATTGAATATGGGCAACTAAGTTCATTAAAGACAGTTTCGACAGTTCCAAAATTATTAAATAATGGTCAATATGATATAC

>contig00598

TTAAACCGATATATAATCAAAACGGATATGGAGGCTGGATTTATCTCCCAGAAGCTATGAATATTGAACCTGGAACTCCTGTATGTAACATCAACAGTAAAGCAATATATGGACTTACTCTACCAATCATGCAGCAAGAATCGACATtAATGATCCCCTTCAATTTAATTTCCGACATTTATGGAGATATCAAATATTATTTTGAGGTTCTTCCAACTGGAATTAAAAAGACAAGGGAAAAGGTAAaCGTTTCAGCTTGTCCTGCTTTtCAGCATC

>contig00599

ATTCACAACTGGAGCAGAACTTTCTCCTATTTGTGAGCGCGTAACTCTACGGATAGCATCATCCACGTGAGAATCATCATCACTCTCATAATCATAAGGATCATATCCAGGTTCACCAGGAGCAACCCGTGTTTTtCTAGGCTtCTTTTTTtGTTGACTGATTTGTTGCCAGATTCGTCCTGTTCACCTTCAGAaCTTCCATAATAACTATCACGTCTTCTGCGCTTCTTTTCTTTCTCGAGAATGCGCTTAAAGTATGCGAATTGAACCAaTTCAATCGCTGCGTTTGCATCTTCCT

>contig00600

ACAgTGaaGtGgAAATTTGATAAAATGGTGATTGTAGAATATAAAAaTGATGGCGAATgATGATTCTGTAGAAAAGAAATTAGGTTTAAGTGTGATTTGGAATTATAAAAATAATGGAGAATGATTTTtGAAAAAAATCGGAAAAGTaGaTA

>contig00601

acTGtAAAGCTGTCTTTACGACACAAGTGTTATTAATTAGGAAATAATATGTTACGGAGGGGCTGATTACACTTCGATTTGAATTTAaGCACTAAGGTCCAATGCGAATCCAATTTTATGACCACCAGAGCCAAAGTTCTTGCCATCGATGTTCGTAGATAGAGTTAGTGTCACTCCTTCGCGGAGCTTTTGCTGATATCCCAATCCAATTTGGAGCTGAGAGTTAACCTTGGCCCTAACACTAGCGTCGTTGTCAAGGTTGTATTTGGTTGCAATTCCAAACTGAGTTACATTGTtACTGGAGTtCCAAGCCAAGTTGATGGCTCCCTCGAGTCCtGGcTTCACTTtATGATAAATAGATCCACCGAAATCGCGGCCATTGTCAACAGAAGTGTAAaGaGCAAAATCAGTATCGGAGAATCCAAGTGCAAAGTTATTTTTAGTGATTTtGcTCCTCTGAGTgTCAAAaGATGTTTGAGATCCAGCAAACCAGCCTTGATATCCTACAACCGAAGATGCGCTTATTAGTGGACCAGCTGAAAGACTGAGATCAAGATCAGCCGTGACTGATGCATTTTCATGTTTATAGACGGTCTTTAGCTTTCCAGTCTTACTCCCAGTTTGAGGTGAAAATGTGCAACTGTATGTAAGCTTCAAGCCTTGCAAGAGTTTGTCAGCGATAGTTACGTCGGTTCCAAGAGTATTGTCAGTGTTCCATTTCTCAGCAAAAGTTAGTCCATATTCTTTGATTTTATATTTAGTCTCCAAAGT

>contig00602

ACCGAGGATGAGGTTATCATGGCTTTGCACGACAGCGACGGTGATTtCAATCGTGCTGTGAATGATCTtCTtGAaGGCGTAAGtCCAGAGTGGGAAGTCAAAAAGAAGAAGGCTCGCCAGCCCAGTAGCTCGAAGCAGAGCACTGAACAACCCGGTGGTCAGGATGAGGGTGGCgAATGGGAagATAGAAGGACTCAACGTGGAGgaGgaCcaCcacgtatgcgtGgacgtgctAatcatgacAaCcggcgcGggagAGAAAaCAAGGAAAATGAAaGAAATATtGAAGAGAGTGGACGAGATGGTGGTTATGGCAACAGAAGAGGAAGAGGTGGCCCTGGAAGATCAGGACGAGGTGGTCGGGGTGGaGGACGAGGTCTTGGACCTCGGACCTTCGCCAATCGTGGAGATCCATCGAGTTCATCtCACAGTTTCAATCGACCGATCGAAACGTGGACCGGTAGCGAAGAACaGCAACAAaCTATCcAGGAACCTAAAaTGgAtGCCTGGAATAaTTTGGAGCCTTCTGAAGATTGGGACAATGAGGAATATACAGGTTCTCTCGCTGACACTAAAGTGTTCACTCCAAGCACGACTATGACTGATGT

>contig00603

ACCGGCAAAaCTGCAATTGCTATGGGTCTTGCTCAAGCTCTCGGACAAGACACGCCGTTTaCCTCTaTGGCAGGTTCAGAAATATACTCCTTAGAAaTGAGCAAGACAGAAGCTTTGACTCAAGCCATAAGGAAGTCAATCGGTGTTAGAATCAAAGAAGAAACTGAAATTATTGAAGGAGAAGTAGTCGAGATTCAAGTCGATCGACCCG

>contig00604

TTGGAAAATGATATGGCCCCAGTCGTTATTATGGCAACAAATCGAGGTATTACAAGGATTAGAGGT

>contig00605

tGTtAGAATTtCGAtCAGATGAAGACTCACTGAGGATATCCCAAAGAATTTTAATTACAAATATTGCTGCAAATATTTTTtACACTCAGTGTAATAGTGACGAAAAGAAATAATGCGTGTAAAGTCAGTGCCAAGAAGAGTTAACCCGAGTAGAATTCAGAAAAACTGCATCGTAAAAAaTaGTGAAATGTGGATGGAAATTTTAAATGCGAAAGACGATAGTTTCCAGGATCTCAAGAaTCCAACTGGaTTGGATTTATCTACTAAGAATAAGgttcttcagTATAAGGTGCAAAAAATAaGTCCACAAGCAAGTCCaGAAAAAaTAGAACCACAAATAGAAACAAATCTAAATATTACTATTATTCCTGAAGTCAATGAAACTCAAAATACACACAAGGAAATTCTGTTAAAGGTCGAACAACAGAACAAATTCAAGGACACATTTTTG

>contig00606

GAGTTTAAAGAAAACTTCAGCAAGCTTAACATTGAATTGAAATTTAACTCTTATTTTTTtCTGTGCGATCTGAATTACTATAAAATATTTCTGAATACAGGAAGTAACAAATTTTATTGTATAAATACATTTTCAACATTTTTGACCTTAAAGTGGTTCTTCTTTATTGGCAGCAACTATTTGCGCTCCTGCAATCATCTGAGGCTATTCTCCACGTTTGGGGTTACAATTCAGCTTAAAGTGTCAGGATATATCAATCATCAACTCCAATGTCAGACATATACACAATGCAGCGAATTTTGAAACAACTTTTTtGTGGATTTTTAATTTTAACACTGATATTAGAAAGCCATTCAGTTCTTCCATACAATTATGGAATGCCTAATGAATGGTGCGATGCTTTTAAaCAATGTTGCGGAAGTAAGCAGTCACCTATAAaTTTGGAATTACCTGAAGAGACTGATGATAAAATACCCTGCATTTTTAAATTTGAGGGCTATGAACAAaCACCTAAAGGAATGGATATATCAAATCAAGGAAaCACTGTGGAAATTCACGCCACGTGGTCAAAGAAAGCTCCATATGTCTTTCTAAAaGAAAAaGAATCTAAATATATCTTCAtGAAAaTCCATTTCCATTGGGGTGAAGATGACACTAAAGGCAGtGATCACACATTAGGAAatAAAaGATTtCCaTTGGaGaTGCaCaTGTTGCATTATAATGAAAAGT

>contig00607

acaTtATCAACATCATCTTCAGAAAATCCTGCAACCTGTCCTCGAATTATTATGAGACTGCCCTTGCGTATAATACGTGGAGAGTTTGTGCAGGTCCGATATCTTGTTGTGTTCAAAGTATTTCCGATTTTGTGAATTTTAGTGAGGAGACCTTTAATGACAACGCCCGACTGAGTCCTCCCCAATAAGATTCCACTGTAATCTGAATCGTCGCCCTCTATAGCGAAAACTCGTAAATATTGATACCGGAGAACGGGTTGATCCAACTCTATAAATGCGATTCTGGACCCATCTCTAACAGTGTCTACATGTTCTATACCAATAACACCGTTAAAAAGGTTCACCCGAGCAAAATAATTtCTTTTATTACACAGCATTTCTACAGCAAGAGAATAAGGAGTTATCACATAATAAGAATGAATCAAGGCCCCAaGAAATAACATTTTTGTAGGTTCTGCATGTTTTCTGAATTCAACAATGGCGTCCTCTGCTGTTTGTGACCAGTGTGTTCCCAACATTTGCTCTTCTGTTTCTATAGCCATGGTGAACGTCAATAATCCGAAAAAGATCAAAATCACTTTTATCATATTGGAAAAATTTTCAATCATTTTGTTAGATAGAATGTATCACGGTTTTCTGCTGTTAAAACTGGAGTCCAATTGAAATTGAAGATTATTGTTATAGTGTTTAATTACAATTCCCAAACCACTCTCCTTTGAGACAATTTGCGTACT

>contig00608

ACGCtGCTAGAAGCAAGAAGACCGGATAGATAAAACAAATAATTTCTACCTGTAATATTTAAGTTGTTATAATTACGGTTTTTATTCATTAAATATGACTACAATTATTGAAATGTCCTTAGTTTGCAGTTTTCTATCTCTATAATCAACAAGAAGCTCGTGATTGAAACTCGATACTGAAATTAAAGTTTCAATGAtAAAAAATACaTTTCATGTTTCTAAAAGAGTTTATACATTtCTTAAGTTTtGTTTTTAGTaTCCTTCATGCTTtATtGAAATGGAATTtACAaTGCTTaGTCGCCAGCTTCTtATTTtACCTATGGGTCATTCTATTAATTCGTCTGATCTCAGTTgTGGGTATATTTTtATAAAATgTGAtACGCAAAaTGTTAACTAAGAAAAaTTAAAAaCACAAAtGGTTTTTTtATAaCAATTATTTTATGTAGTTAGTTCCAAGTAGTTTTTtCcAaGTCTGCTCTTtAAAAATtATGAGATTTtGATAAATGTAAGTTCACTTtGAAAaTTTTtcAGATTTCACCATCATAAGTTTCTAAAaTTAATCTCACATTTTGTATTACTTGGGAAACGTAAATTGCAAGAATCCAATTACTTTTCAAATCGAAGATTTTGTAGTCTTCAAAACTCACTTAATGT

>contig00609

AAaCTGTAGTAGAACAGGAATTAAAAAAaGTTCTTTCAAATTGTAATTATTGAATTGCAACATATAATTAAAAATGGCAGATTCCGAACTTGATGCAATTCGTCAACAACGTTTGGCACAATTACAATCTCAATACAAAGGAGGAGATGCTGGAAACCAACAAGCAAATGAGGAGAAAAGACAGCAAATAGAGGATATGAGACATGTAATTCTTACACAAGTTCTTGATCAATCTGCAAGAGCAAGATTAAATACTCTTTCTCTGGGAAAGCCAGAAAAAGGTAAGATGATTGAGGAAATGC

>contig00610

CCAAAAAACCCTCGaTTTTTCACTGTTTTTGTTACACAATCCCATATTCCAGAaTATtCCTTCCcTGCACCTGCTTTtCCATCCAGTtGTAATTGAGTTTTAaCATACTCCGTTGGATATGTTATGCAGATTTCGATtCCTCCAGTGATTCCACCGGCAATAATTCCTTTGAGTCCGATATTTCCACTGGGTGTAGCTGCTGctGCTTTATTCTGGAGCCATGGCCTAGATATGAAGGGATTGCCAGAAACACGGGATCTTTGCCATAATAAAGATTCACGT

>contig00611

CGtCGCTTAGATTCGTCCTGATTTtGGTAGATGATGCTTCAGAGCGAATTTCTCTGAGAATAACATGTCGAACACTCATGTAAATAAGGAAAaGACGTTCAACTATTTTTTtCAAAATaTGTTCGATTCGGACATTCACTTTTTtGGCGATGGGGTGAGACTTGGaGTTATCAAATGAATAAaGTAGATATGGAAAAaGaTTTAAATTCTGACAGCAGTGGCGAATTTtCTGCATGGAATTATGAGGAAAcAGTGATGGCGGTCCACTATTCGATCCGTCTTTGAaCATATCGGAAACACGTCGAaCCAAAGTTAaTCTTTTCTTGAGGACAGTTTTCTGTtGAaTTACTTCAGTTTGACTCaTCTTGGATGAGTTGATGATCGTAGATCTTTAAAAGATGATAAATCTCGTCTGGTCTGATTATCGCCTCTTAATAGAAGTGATG

>contig00612

GAAGATAAGAAGCAACAGG

>contig00613

ATAATTtCTTGGCTTATCATCTTtACTTTCGCTCTGCAACCGTCTCACAGCATTATTTATCAAAACCGAACGACATAAaCTCGTTTCAGGATCTTCTATGCGTCTAAGTTTGCCTGCAGATATTTtCAAAAGCCTACGACGTTCTTCTCGAGCTCGATATTTTGCGGATATcaTAGTTGGCCTGCTTCTACGAATCACTAAGTCCTCCTCCAATCTCATCTTTCTTTCTTGTAGAAAGTAGCGATCCTCCTTGCTTGCCTACGTCCTTTAGTTACGGAGGCACGCTGTTTCTTTGTTCTTTCCTTCGTCACTATTGTGGCTGGCCGTGCTTGCTTGGTGAGTCGTTATTGCTAGCGGaTAACGGAACGATAATCACAGCACGCGTCAATGGCGTCGTCGTCACCTGCACTTCGT

>contig00614

ACTCGtCGgAGGATCAACTAGGATTCCCAAaGTTCAGCAACTCGTAAAaGAaTTCTTCGGTGGAAAAGAGCCATCCCGAGGTATCAATCCTGATGAAGCCGTTGCTTACGGAGCTGCTGTTCAAGCCGGAGTTCTCTCTGGTGAACAAGACACTGATGCCATTGTTCTTTTGGATGTCAATCCTCTTACTATGGGTATCGAAaCCGTCGGTGGTGTGATGACCAAACTCATCCCCAGAAACActGTCATTCCAACGAAGAAGTCCCAAATCTTCTCCACAGCCTCTGACAATCAACACACAGTAACCATCCAAGTTTACGAAGGAGAACGTCCAATGACGAAAGATAACCATCTTCTTGGAAAGTTCGACCTTACTGGAATCCCACCAGCACAGCGAGGAAT

>contig00615

ACTCACAACGGTGTATTTCATTGCGATGAAGTTTTGGCCTGCTACATGCTAAAAAAATTGCCACAATATAAAGATGCTACGATTGTAAGATCGCGAGATCCCACGATTTTGAAAACATGTGATATAGTAGTCGATGTGGGAGGAGAATATGATCCATCTAAACACCGATaTGATCATCACaTGAGAGAGTTCAAAGAAACTGCAAGTTCAGT

>contig00616

AcTtGTATtGTATGATGAAAtCAAGAACCTTCTTTAAGAAAGAGACAATTAGGGTCCTCACCTCATTAACATTACCAATCACAGAACTCATAATCTCCATTACCAAATAAATTCCCTCATCTGGTATGAAAGCAACAGTCTCCACACCGAGTGGCAGGACATAGACTGTCTACAATTTCATAAATAACTCGGGCTGAGAGCGATCGGTAGCGGCTCGTAAAACGCGCGAAACTTGGCAAAAAGGaGCTCGAGATgTATTTGAGCTTTTTAGGATAGAATTCGCTTAATTTATTGCCGGATTCAGTATTTTCCTGAGCCTTTGGGAACGCGCTATTCACTTCATCATTCCTACGGCGCGCGCGAAGGGGCAAAAGGTTAAAGATATTTGTGAAATTGAAAACACACGTCGTTTGAAGGACTACCTGATGGGCGTGCAGGACAGGCCGTTCAGAAGAAAGTTAAAACAGACGGCGTGTGTTACTCGGGTCAGTGAAGCTGATTACGTGAACATCAAACTAAGGAAAATGAGTCAACAATAATTGAAAGCAAAATATTAAATTGTATTGAGAACGAGAGGGACGCGCGGCGAAAAa

>contig00617

GATGTGGTGAAATTTGTAACTCtGGTTTTTtGtAATTTTTGCGTGAGTAGAAAACATCCTCGACATCATCGAAACCAGTGtAACGCTTGACAAGATTTAACAGCGACTCTAAaCATTGCAATGGCGTAGTA

>contig00618

AGTCGCGTCCTTTCCGAATTCCAACTCAGATCGTTTGGGATATTTGAATCGCGTGTGTTGTAGAATTAGTTTCATATAATTGATATTATTTATTAAAAaTGCGAGAAATTgtGCACGTACAAGCTGGCCAGTGCGGTAATCAAATCGGAACCAAGTtCTGGGAAATGATTTCAAATGAACATGGAATTGATCCAGACGGAAAATACGTTGGAACTACAAGGGATCTTCAATTAGACAGGATAAGCGTTTATTATTCGGAAGTTCTAGGTGACACTTATGTACCAAGGGCAGTATTAGTCGATCTGGAACCTGGAACTATGGACGCTGCAAAATCAGGATTATTTGGAAAACTCTATAGACCCGATAATTTtGTTTATGGTCAAGGTGGTGCAGGAAATAATTGGGCCAAAGGTCATTACACGGATGGAGCTGAACTCTCAGAAAATGTCGTCGATGTTATCAGAAAGGAGGTGGAAAACTGTGATAGTCTGCAaGGTTtCCAGGTGTGCCATTCTCTAGGAGGCGGCACTGGATCAGGAATGGGAACACTATTGCTTTCAAAAaTAAAaGAAGAATTTTCCGACAGGATGCTTTCATCGTTTAGTGTAATGCCAAGTCCTAAAGTCTCAGACACAGTTGTTGAACCGTATAATGCAACGTTaTCCACACATCAGCTTGTAGAAGTCGTTGATAATACTTTCTGTATTGACAATGAGGCTCTTtACGACATTtGTTTCAGGACACTAAAAaTTACaTCACCCGCGT

>contig00619

GAAAAACTGAGACAAACGCACGTTTGCACGGACCGGCTGGAAAAGAAGCTAGTGAAATAAATTGATTAAAATGCCGACTTATAAACTTTATTATTTCAATCTCACCGGCTTAGGTGAGCCGGTTCGATTTATGTTGAGCTATGCAGGAATTCAGTTCGAAGATATTCGTATCAGTTTCGATGAGTGGCCAAAATACAAATCTGATATGCCTATGGGTCAAATACCAGTTTTGGAAATTGATGGAAAGAAATATAATCAATCGAAATCTATTTGTCGTTATATAGCAAAAAAaTCAAACCTTTATGGTTCAAATGATCTGGAAGCGCTGGAAATTGATGCTGCAGCTGATAACATTGATGATCTAAGAATACCTTtaTCTCAGTAtcATtGGgAAAAGgATCCGAACATCAAagCAGGTTTAGAGGAAAAGGCCATGGAAAAGT

>contig00620

ACCAGTGATTCCTATGATTGAAAGTCCAACGCCTGCAGTTCAGGTAAATGGTTCTGCCCCTATAATAATCAAACGTGAAAGTAGGAAATCAGATTCTGAGAAAGAAGATAGGGATAAGGAAAAGCGTCCCAGAAGTCGCGGCCGTATGCGATCGAGAACAAGGTCACGCTCCAGGTCATGGGAGAGAGATAGACGGCGATCCAGAAGTAGAGAGCACATTCGTCGTGATAGGGAACGTGACAGGAGTCGTCCTTGGAGGAATAAGTCTCCTCCTCTCAACACTAGGAGGCACGAAAGAAGACGAaGTAGAAGTCGAAGCACGTCACCCTtACGACAGCGAATACGCGAGGGACCTGAGCTTAGAGATCACAGAACAAGATTTCGAAATAGATCTCCTaCTCCGTTGAGATCTAGGTCGAGGTCTAGGTCCAGGtCAAGATCTATAGAACGTAAGAAGCTGGAtCGTTTAGAGAGGACAGATC

>contig00621

GTGaCTATGTGTTTCTGTTTGTGGCTTATCGTCTTTGACTCTGTCCTTTAAAAATTTATAATTTATCCCTTGTATAAATAAATTAAAGAAGAAAATAGTTTTAAAAAGCGTGGAacTTttaGggaTACAATGGAAAGCTCA

>contig00622

CACATTTACCTTTtGGGATTATTCATTCATGATATAGTGACTTATGAATATATTCTGACTAAtAATAATTGAAACTTGCAATGTTTCACAAACTGTCTCGTATTTCAGTTTGTtGTCGAAAAaTAaTTGTATATGATAAtGCATGAATTTtGTAAAATaGGgTaaTAAAGT

>contig00623

aCTAAGGTTAACGACGTTAACCAAGTAGTATTGTGGGACAAAATAATTTtACGtGGAGAtAAGGCAGTAATCGACAACAAAAATaTGAACTCCAAGTATTATTtCTGGGATGATGGAAACGGTCTCAGAGGAAACCAGAaTGTTACTTtAACTTtGACTtACAATATTATTCCCAATGCTGGATTACTACCGTTCGTAGCagCTTACGGgTCTCACAAaTTCGCATTCCcATCAGAATACAcAaCATCACGAGTGTAAAaGCTCTTCTACAGCATAATG

>contig00624

GgACTGGCGAATTGTTAATCAAACATGTCTACCAAACCGTCTATTGATCTTAGGGCGGAATTGGCAGAACTCGTTAAACGAAAAGCAGAAATCGCTGACACATTGGCCAACTTAGAACGACAAATATATGCATTT

>contig00625

TACCAATAGTAAAGCAGAt

>contig00626

ATTATTTAAATGTTTTTtGAGTGCATTAAAATGTTTTATAATACTAGTATTATTTTAATAATCATCTCTATTCTTTCATCAATTTTGATTATACAATCAGTTGATGGAAATCCTAGCAGTAAAGAAAAaGGTTCAAaGTTCAAAaGGGGAATTATAAGGTTCTTTAGACGGGATAAATGTGAGAAGACAAATTCTGATCAGCCCGAACTTGAATATACTGAGCACCTTGAACATCCGGGTGATAAAACATATTGTATGGATCCAAAAGACTTTGAAAATTAAAAAaGAATGGCAATGATGGTTCATATAATTATTTTCTAAACCAACGTTATCCATATAATCCAAATCAAGGTTATTCATATATTGAAAACCAAGGTGATTCATATATTGAAAACCAAGGTGATCCAAATGCAGGAACTTCAGAGGCCGAATTTACATTCAACGGAGCACCAGTGAATACTTTGACCCCTATTAGTCAAGTTAAACAATCTAACCCATGTTtCAAAAAGTGTCTTAAACCATGCCCCAAAGCTTCaGTTTtAtGT

>contig00627

GTGCTTGCGTTTTTtGCCTCTACCCGTTTGTTtCTCGCCCTTAGGCGGTATGTAATTCTGCATTTCCGTGTCGTATCTCTTTTTGTCTTTCTCTGCCATTTCATGGAAACGCTTTTTTCCTTATCAGACATAGTCTTCCACCTCAAGGCGCACTTTTTTGAGAATTCCTGGAAAaCAATGTTTTCCTCGGGGTGTTTTTTCTTGTGCTCTTGACGACACGTTTGAACGAAGAACGCATAAGCGGTCATCCTTCCTCTTGGTTTCGTTGCCTTTCCTCTTGGCATTTTTtCTTGTTGCAAACTCTGCGCGTTATTTTGTTGAAGGTTATTCTGTTGCTGCGTTAGCTGTTGTAACTGATGCTGATCACGGgtCTT

>contig00628

ACAGAAAGAGGAAGTGCAAGTCTCGCTTGGTCCCCAGGTTAGGGAAGGCGAGATCGTTTTTGGAGTTGCACACATTTTTGCCAGCTTCAATGATACGTTCGTTCATGTAACTGATCTTTCCGGAAGGGAAACAATTGCCCGTGTTACTGGTGGAATGAAAGTCAAAGCTGACAGAGATGAAGCTTCTCCTTACGCTGCTATGTTGGCCGCTCAGGATGTAGCAGAAAAGTGCAAATCGCTTGGAATCACAGCGCTGCACATCAAGCTCCGAGCTACTGGAGGAAATAAAaCAAAGACACCAGGCCCAGGAGCACAATCTGCTCTTCGAGCTCTGGCACGTTCCAGCATGAAAaTTGGAAGAATTGAAGACGTtACTCCAATTCCTTCCGATTCTACGCGCAGAAaGGGTGGTCGTCGTGGTCGCAgATTGTAAATTTTTtGCtGGTATTGGTTTtACAAATTC

>contig00629

ACTAGCCATTTATACATTTTCAATCTGACGTTtCGTTtCtCtCTtAAtACATAGTTACaTGTTAAAAGTTACAGTGAAAGTGAAAaTAACATTTGTATTAATTATTTAAACGGAAGTGATTaGTTTCTTCACaGCAAGAAAATGGAAATGTGTAGTAGCAATTTTGAATCACCTTTGAACGATTATTCATATGAAAATGTTGTGAAAGAAGCTCCTATTtCTAAAGGATTGACGCTAAATAAaCTAATTCAaCATCTGCACGAAGCATTCAAAACAGATTCTGTTAATATTGACAGAGTTCAGGATTTAATGTCCGAATACAGAAGTAATCCATTGGAGTGGAAAAAaTACGCCAAaTTCGACAGATACAGGTATACAAGAAATCTGGTGGATGAGGGAAATAATAAaTTCAATTtGATGGTGCTTTGTTGGGgAGAAGGTCATGGATCAGCTATTCACGATCATGCGAATGCCCACTGTGTAATGAAAATGCTCCAAGGGGAGTTATGTGAGACACTGTATGCGTGGCCCACGACGAAAAATAATGAAGATTGTTTCGAAACGTCACAAGAACTTCGAGTAATTAAACAAAGCATCATGGGTATAAATGATATTACTTATATAAATGACAAAATTGGACTTCATCGTGTGGAAAAT

>contig00630

AATCGTATTGTTTTCGTTTCTCTACATCAGTCAAAACGGCAACAGCATTTCCAATGGCTTTGAAAGCTTCTGCAGCTCCTGGAGCTTTATTTTTATCAGGATGTAATTGGAGAGCTAGTTTTTTATAGGATTTTTTTATATCACTATCTGTCGCTTCTTTAGTCACTCCCAAGATCTCATAATAATCTTTGCACTTTTTAATTCTTTTCACATATTCGATTTGTTCTTTGGTAAATTCAACCGTAGTCTGTCCTTGAGTTTTttCTTtGGTAGCAGTTTGCCGTTTTCGAAGTTCAGGTTCAGATTCAGGTTTCTGGTTTtGCTTTGAAAGCATTGCgACTCTGGTTAATAAATCTTC

>contig00631

CGACGGAGTCGGCGTCTTCTACG

>contig00632

ATGTCTGCTGCATTTTGAGTGGTTTGAGTATTTTGCGTATTTTGCGTATCTGGCATTTCTTCGTCGTTATCATTCATTTTAATTAGTCTGAA

>contig00633

ACAGAAGTAAAGAGCGAGATGAAATACGGCTGAAGCGACAGGCTAAAAATCGTGGAAATTATTACATTCCCGGCGAGGCTCGTCTCGCTTTTGTTATACGAATTCGGGgTGTCAATCAAGTTGCTCCTAAaGTCCGGAAGATCCTGAAGCTCTTCCGCTTGCTTCAAaTAAaCAATGGAGTGTTtGTAAAGTTGAaCAAAGCAACTATTAACATGCTTCGTATTaTTGAGCCCTACATCACGTGGGgTTATCCCAACTTGAAGTCTGTTCGAGAACTtATTTACAAGCGAGGGTTTGCGAAaGTCAACCGTCAACGAATTCCAATCACCAGCAACGCTATTATCGAAAATCAATTAGCAAGAAGCAACATAATCTGCACAGAaGATTTAaTCCaTGAAaTTTTCACAGTCGGTCCAAaGTTCAAGTtGGCCTGTAACTTCCTTtGGCCATTCAAGCTTAATACTCCAAACGGAGGTTGGCGCAAGAAGACAAACCACTATGTC

>contig00634

TTCATTaCCTGAAGCAaTGTGGagTGACGTGTGCTTCATGTGATGTtCGGgTGgaCCCTCgCgTTGGCTCC

>contig00635

AGCGTATTCGTGTCAGTGGTTGAGCATGCACGAAGTGTATGCACTTGGCCGTTATTTTGAGTGAGACAGAAAAGTAAaTTTGTCCGTTTTCAGACATGAATTTCATCTTCGTCGAATTCAAAGACACCGTGAACATTCCGTGATAAAATCCACACACATCGCACCCTCTAAAATC

>contig00636

CCTCAAAATTTTGGGAACTATTAACTTCTGCAACTCGAGAGTTTTGCGATTCAAGTGCGTCATTAGTAGATTCACCATTTATTTCAGCACTTGAATGATTTTCCACGTTGTTTATATaCGAAGCATCCGTCTCTAGTTTTGTTAGGACCTTTTTTCCGTCACGTATGGTGATTTTGTAGACAGGTATTGACGATTGTGCAGAGGATACAACGACAAACGATTGTTTTTTCTGCAGTTGTGGAGAATTTGCTTGACATTGTGTTTGCCCAGAAGGCTTATTAACTATTGATGATGAAACCGGT

>contig00637

ATTCGCTGCAGTTTTTGTGGTGTAAAAGGTGCCTTTTAAGTTtACTTTCAACACTTCATCAAAGTCATCATCGCTGAGCTTCAGAAGGAAATTGTCTCGGGTAATACCAGCGGAATTAACTAGGACGGTTGGTGCACTTGAGAAGTGATGCTTACATTTTTTGAATGCAGTTTCTATACTTCCTAGTTCCGCGACATTCATCTCCAATGCTATGTGTTCAGAACCTTCCAAAAACGAGGCGGTTTGTTCTGCTG

>contig00638

ATTTTTATCAGTCACAATGACTCGAGCACCTTCTCTTGCCAAAATTCGACACGTAGCTTTCCCAATGCCGCTACCGGCGCCT

>contig00639

TCCACCACTaGGAAACGAAAAACAAGATCGTCCAAAgACGATAAAAGCGATGTTGAATTTAGTGATGCAATTGATCTCGATTAAaTAGCTCGAATCTaCTTTTCTCGTCTATTAAACTACAC

>contig00640

ACGTttGtAGGAGTAAATCTtGACGCTTACGATCCAAGTGCCAAGGTTGTTTCTAaTGCTtCCTGCACAACCAATTGCTTGGCTCCTCTTGCCAAAGTTATCCACGACAACTTCGAAATTGTCGAGGGTCTGATGACCACTGTTCACGCTACGACTGCCACTcAGAAGACAGTTGATGgACCTTCtGGAAagCTATGGCGAGATGGTCGTGGTGCCGCACAAAACATCATCCCAGCTGCAACTGGAGCAGCCAAAGCTGTTGGCAAAGTAATTCCAGCCCTGAATGGAAAATTGACCGGAATGGCTTTCCGTGTCCCAGTCGCAAACGTGTCTGTCGTTGACTTAACTGTCCGTCTTGGCAAACCAGCGACCTATGACCAAATCAAGGCTAAGGTAAAGGAAGCTGCGGCTGAAGGTAGCCCACTTTCTGGAATCTTGGGATACACAGAAGACGATGTCGTCTCCTCCGACTTCATTGGT

>contig00641

GTTTCTCTTCAGTAATATAACTGCTACTTTTCAAGATGCCGAAG

>contig00642

CGATGATAGCAGTGCCAGTGACAGTTCTACGGAGGCTGATAAGAAAGGAAAGAAAAGACA

>contig00643

ACGTTGATGATAATGGAGACATGAAaCCGTCTAAGAAaGGTaTATCATTAAaTCcAAAaTTATGGAGAAaTTTTatGGAAGTTGTCCAGGAAGTCGACGAAACTGTTAAGGAAAAaTGTTAAaGGCTTTCATTACAAaTaTCAGAGACATTGCGAATTCGtGTGTATATATTTTGAAGTAAaGTaTAAAGTCTTtGGCGATTACTATTTATTGAAACGTTCGTATTTTCCGTTGAATTGTTTAAAA

>contig00644

GGTCATATTTTATTTTGATTATTTTCAATCTAATAGCTAATCCAATATAAAAATACAGAATAAGAGTTGATGTAAATAGCATTCCCCATCTGAAAAAATTTCATTATAATAGAATTACAAAATTCTATCAAAATCCTTAATGGGAATATGCCAATTGTTATTCAAGAAGATCAAATAATGGCTGAACAAAAACAACAATCAGCTCTATGTTGCTATTCAGAACTGTCATCATATGTTCCTTCTAAAAAaGATTTTTtACTCAGATCCATATTGATCGTCGATGGATGAACTCCCATCTTTAGACATTTTTCAATTGCTTTTTGCCTCTTACGTTCAACTCCTTCATGTATCGCATATTCCTTGGAAGCAAGGAGATTATAATACTCAATTTTTGAACTGGCCATCTGGTGAAAAGTATTTTCAATCTCCCTAGCACAGCTTATCAAATCTTCTATTATCGTTTCTATATGCATCAAGGCTTCCGGAATGGAAGTAGAAAATACCGCATGGACTATTCTGtGAACCATATGTTCCCTCATAAGTTTTGAACAATGCCACTTACTCGaCTCTTCGTTTGATGTAATTTTCTCTTCATTTGGTGGAAaCATCTGTGGAAGTGTTTTACTAACCATGGGTTGATTATTAATTGCCACCATTCTTAACATTGCTGATGCACTtCCAGTAAATTGGTTGCCTAaTATATTATTaCTTCTAGCTGCAGCAAATCTCCTCGCATCCGCTTGTTTTAAaGGCGT

>contig00645

TGGTAACCTGGTAAAGGAGCGTATCGTGCAACGGGTGAGTTtCGTATAAATGCGAGACTCTTGCCTGTCGGCCGTTCCGTGGTTTTGAAATGAaTTtaCGCCCaGCAAATACTTCaTTGCCGATGTCTCGACGGTGGACACTTATaTTAAAATaTAaGTGTAaCCACTGATCGTGTTCAGAGGCCGATACAAATCACTTTATTGTGATTTTATCGAATCGTTTTGATATCGATTAGAACTGTTGAAATTGATATAAATTGTAATAAAATGATTACCCTGAACGGTGGATCACTTGGCTCGTGGGTCGATGAAGAACGCAGCTAATTGCGCGTCATATTGTGAACTGCAGGACACATGAACATCGACATTTCGAACGCACATTGCGGTCTACGGATACAATTCCCGGACCACGCCTGGCTGAGGGTCGTTTATAATATAAAAACTGCTTATCTGTATGTATATTTGAAATATTACTACATATATAACAGATACGAGCGAAATAATGAATGTTCGTTGTTAAAATTGTTTGCTGAAGGATGAAAATAAAATTTTTtATCAGAAGTAAGCAAAAGCAATATAAACAGCGTCATTTGAAATAAGTTGAGATATTATTATCCCTGAGACAAAACCCGTCGTCTGGTCTGTTAAACAAGCAGACATTTGAACGCATTTAAATCGTTTGTGTTATACACAATTGTTTATACGCAATCGTAGACTGGTTGTCGAGTCCTGG

>contig00646

CACTAACGAAACATATGAGGGTCAGTTTGCGAACGGAACTCAAATCCCCTACGATGCTCGCTTTAGCTAAAAAGAAACAAGCTTGAAATGTAATTATACAAGGAATATTTTTAATTTAAGAATAAGGGAAGCGACTGATGATCTTTTGTGAATACCTAAGTCTATATTTCTATGCATGTGAATTTGACTGCATGCACAAACTGCACACGAGA

>contig00647

ACAGTTCCtAtAGTTTAGAAAGTGTGTAGAAGAGGATTCTAACAGAATTGAAGAGGTtATaTTATTGTCATCCTCAAATAAAAGAAGTTATTGGAAAATTCACAAAATGGCTGACTACTGGAAGTCACAAGGTAGAAAGTTCTGCGACTTTTGCAAGTGCTGGCTAGctGATAACAAGCCTAGTATTGAATTCCACGAAGGAGGAAAAAAACACAAAGAAAATGTTGAAAAAAAaCTTAAGGAAATTCGAAAAAaCAGCGCCAAGCAGGCGA

>contig00648

ACTAGATGTaTGGTTATGAAaCG

>contig00649

ACCAAAATGAATTATTGGGTGGATAGTCGTTTGTGTAGACCATATTAATGTTACAATATTCACCCCATGTCAAGGGAGAATCCGCAGAAGATACGAAATTGTAAA

>contig00650

TTTTTTTTtCtCACATAATCTTCTGCCAGTGCT

>contig00651

CATGTCCGAGGTCCATAAGgTAAGATTGTCCCTGaGCAACTGCATAATGAGGgTGGAATCCTTGTAACTCTCTTCGGAAAGGGTGTCTAGTTCTGCGATCGCATCGTCGAAaGCAGCCTTTGCTAGGCGACACGCTCTGTCGGGACTGTTCAGGATTTCGTAGTAGAACACTGAGAAATTGAGCGCTAGTCCTAAACGAATGGGATTAGTTGGTGGTAGGTCCGTCATCGCGATATCACTCGCTGCTTTGTAAGCTACAAGGGAATTTTCCGCAGCGTCTTtCCTGTCGTTGCCGATCGCAAACTCAGCCAGATAACGGTGGTAGTCTCCCTtCATTTtGTAATaGAAAACTTTGGATTCGCCCGTTGACGCACAGGGGATTAAGTGCTTCTCCAAAaCTCCAaGTATGTCAGCACAGATATCTCTGAGCTCCTTCTCCACCTGAGATCGGtATtGCCGAATCATCTCCAACTTTTCCTCTACGCCTTTATTTTCTTCCTtCTGCTCAATACTCGAAATTATCCGCCATGATGCCCTTCTCGCaCCAATTACA

>contig00652

CGATGCATTGTGGATCTGCCTAATCTGTGGACATATCGGTTGTtCGCGTTATCACCAAGGtCACGCGTTtGAACATtACCGTGACACACATCATTGTTACGCGATGGAGCTTGGGAACAATCGAGTCTGGGATTACGTCGGAGATAATTtCGTCCATCGTCTaCTGCAGG

>contig00653

AATGAGGAATGGTATTTATTTTTACAATCACTGTTTTATACCTATATTTAGAAGATATTACATAATAATAGAATACAAATTAGGTTTAGTATTTGTTTCACTGAAAAGTTACTTCTTAGTGATCCCAATAACACCACAAGCTAAACGAGCTCCAGCGTTTCCAGTAGTTTTAGACAATTCGTGTCCACCTTTGCCCAAATCATCTGGATCAGCATGGACAACGAGTGTTCTTCCGATTATGTTATTGCTTCCACTAAGTTGAATCTGGCTGTCTGTTATGTTGACTTTGGCCACACCATCGTCGCTGGCTTCGACGTTGCCTAAGTCTCCAACGTGACGAATAGTATCAGTTGGTCCACCATGAtCTTTtCCAAGGGGATTGAAATGGGGACCAGCACTTGT

>contig00654

TTAGAAAAtCAAATAAGAGAAG

>contig00655

TTGTCCATGCCATAAGAAGCGGAGAAAAACCTAAATTTAAAAAGAAATCTGAAAAAAAAaTCTTGGACTTGGTTTCTCAATATGAAGAACTAAAGAATTCTGGAAAACTTAAGAAGCATATTAAGAGATTACGTCGGAAAAATATAAATAAAGAGAGAAAAAAATTTGAGAACTTTGATTAAAATGATTATTTCATAGCGCCTCCTTTGAAAATCCGATTTTCGGT

>contig00656

ACGTTgCCGCAACGTATAACGTCGATGCTGAGAAACAAGCTCCTTTCATCAGAAAATACCTCAAAGCTGCAGTTACCAAGGgATCTCTGGTGCAGACTAAAGGAAAAGGTGCTGCTGGGTCTTTCAAGTTGGCTGGTGGAAAAGCTGAAGCTAAGCCAAAATCCGCTAAAGTTGTTGCGAAGCCAAAGGCAGCTGCTGCTACAAAGACAAAAAaGCGGCAACTTCGAAGAAGGCTGCTGCTCCAAAGGCCAAAACTGCTGTTACTGAAAAACGGAaGACTCCTAAAGTTGCTGCTAAACCGCCAAAAGCAAAATCCACAACGGCTAAACCTAAAGTCAGCAAAGCTTCCCCTAAAAAGACAAAGGCACCAGCAGCTCCAAAAAGAAGGCTCCAGCAAAAGCAAAAAGACCGCAGCAAAGAAAGTT

>contig00657

CTCCACTACTAGCTGCTATGTCCAGATGAGAGTAGAGCAGAGGTATTTTTGACTCTATGCCATGCTTATCGAGACCTGATGtAATAATTAGAAAGGCTCCTGGCCCTTGGTGCCCTCTCTGTAATTGGGAACTTgaTTTGCTGACTCCCTGAATAACGTCATCGCCCTCGGCTCGACCTTCATGGAATTTGAAATCCTCTC

>contig00658

CGCCCACGACCAACGGGGGgAGTAGGAGAAATCTCTGATGATGAAGTTGAAGCAATAAGAGAGGCTGCTAAAGA

>contig00659

AGAAGTTGATCATTTGTCAATTTGATTGATTTCTAATCGTTAGCCAAGAAGTTCGAACTCTTCGTCTGATGTGTCGTATTGTCGATCGAAATGCTCAAATTCGAGAGCAGTGGAATCTGTTGCCCCTAAAGCTTCTGAGACCATGACATCTTCTGCCGGAGACCAATGAGCGGGCCTTTCCGCATACAATTCAAAAAGCGGCTGCTTTTCCGATGAAATCAGTTTTTCCTGCATTAGTTTGCACAATGATTTAAATTCCTTTTCATTCTCACaGAAAAAaCAAAGTGCGACTGAGGgATCCATTCCACTAaTTGGAATACGACTAGCGATTTTGCAATGATACGTTGAATCGAGATCAACTtCATTTTCATCCAACTTTTGATCAACACATCCCGACCTTTGT

>contig00660

ACGAGTTCTGTCAATTGTCAGCTGGTGGATCGGTAGCAGCAGCTGTGAAGCTCAACAAGCAAGCTTCAGAAATTTGCATAAACTGGGGAGGTGGCTTGCATCACGCCAAAAAAaGTGAGGCTTCCGGATTTTGTTATGTAAACGACATAGTTCTTGGCATTTTGGAATTGCTAAAATATCACCAGAGGGTTCTGTATATCGACATCGACGTTCATCACGGAGATGGAGTCGAAgAGGCTTTTTATACCACCGACAGAGTCATGACGGTTTCTTtCCACAAGTATGGAGAATACTT

>contig00661

CTTGAGctATTCCAGCAGATTTGAAATCGTAGACAACATGCTCGATTTTTTGTCCATTAGTGCCATTCCAGGTGATTTCAAGCTTCCCAGGACCAGGAACTAGAAAATCAGTTGCTTTGTaTTGATCTCCaTGAGCGTGCCTTCCAATAATGATCGGTTCAGtCCAGCCAGTAACCAATCTGGgAATATtCTTGCAAaTAATTGCCTCACGGAAGACTGTGCCTCCTAAAaTATTtcTtAtAGTTCCATTCGGACTCTTCCACATTTGCTTCAGCTTGAATTCTTCTACTCTTTTCTCGTCCGGAGTAATTGTGGCGCATTTAATACCAACGTTGTATTTTTTAATTGCCTCAGCACATTCTATAGTCACCTTATCGTCCGTTTTGTCTCGGTTTTCCATTCCTAAATCGTAGACGTGAAGCTCAATATCCAGATAAGGAAGAATTAATTTTTCTTTAATTGAGTCCCAGATGATTCGAGTCATCTCGTCTCCC

>contig00662

GGTTGTCATCAAAAaTATCAAAAACAACGACCAGAAAaCGCTTTTTTGtCCTACTTTAATTTATTTATACCAGTTTAAAAAaTACCAATAAAAaTATTTCAAAaTGACTGCTCCAATGGAGAGAATTCAGATTCTCGAAAAAaTCGAACAGGATATCATTCTCTGCTTGC

>contig00663

TTTAATTCTACTGAGTCACTTtGtAAATGAACGTGTCTCACAGAATTTAATGTTTATTTGGAAATCACTATTTCTGCAGAATACATTGCAGATATTCTTTGGGAAGATCCTGCCTTCTTTTCCGTTTGCTTTTCATGAAAGATTCCAGCCCGTCACCATAAAAaGGGATATGAAATCATGTTTCACACATTCGTTAAATGTCTatGGTGAGAATTTTCAATTCGATGAGAACAGCAAaGATCAGGAaCATTAaaTACATGCACAAACATGCGATTCCCACTCTTTtATTTAaCTG

>contig00664

CTGAATAAAATCCTaTGACACAAACTATTATTGCTATAACACTGTAGGTAATTACACCAGAAACAATCTGGACATCCTTTCCACTTAATATCACTTTAATCATCCAAGGCAGTCCCAAACATAAAAGAATGTCTAAAGTATTCGCCCcTAAAGTATTGCTCATTGCCATATCTCCATTATTTTGCTTGgAAAGAATGACGATAGAGACCATTTCCGGCATGTTCCCTCCAGCTGATAGAAAAGTAATTCCCATGATAGAATCTGGGATCCCGAGAGTATCCCCCACTACAGTAGTCATCCAGGAAATTAAGTATGAGGATATGGCAATCCATATTACACTCATGAGAAATGTGACTGGATACCATTTACGGAATCGTTTGTATCGAGTATCGGGAATTGTAATAAATAAGAGAAATCGAACAGGCCATGTGAATAGGT

>contig00665

ACtGTTTTTGAGAAGAATGGCAAAGGTATATAAGTTtATTGAAATCATGATTAGTGATGGAGTTTTTtGTCAaCGTAAGTCCATCAAACAGAATAACGTTTCTTTCGTATAAAAGAGACATCGCTTCAATTTCATTCCTTCCTCCCCATTCTGTAAAGCAAGCCATTTGCTCTAAATAGTTTTCGAATGGAACAGATATGTTCTTTTCGAATAAGTGACAGTTTTTCCTCATAAATTCTACACATTCCCCTCTTACTCGTATATGAAAATGTTGCGTTAAGTATACTTGTTCACTAACTGCTCTAAATAAGCAAGTTGGATCTTTCGGGGCGTGTTTCCTGAAAAAACCTTCGCTCTGCAACCACTCGTCTATCGGTTCAGCCACACGCTTCGCAGGCTTTTTAGATATTACTTCCATGATTCTTGTTTTCTTTTGTTTTCCCCCGTCACCGTATCTTCAAAACTAAATGGAAAATGAATCCTGTTCTTCGAAATTCACAATAGAACAGAAAATCGAATCGAGGGCTGAACAGATACACGCGTTTCCGACAATCAACGTTCAAAGAGACT

>contig00666

acGTCTGGATAATATTATTAtGtAAGtAGGtGTtACTTtGTtAAAAaGTtGTTtCtCGCCGCTGTTGAGATGTTGCTGTTGGCTTTGTATAATTGTTGACTCGTTCTCTGGTTTTTCTTACCAATGAAAAAGTATCGATTGGCTAAGCGGGTAGTTAGGTCGTGAACGGTTGGAACTGAACACAATTATTTGTCGAGGGCAGTGTTGGTTTTAATAGCGATTACAACTTCTTCGCCGCAGGCTTTTAACACGGTGCACAATAATTCTTTTCCcGCCTCAAAATCGGCACGTAACTGTGCTCCTAATTCTCCGTCGGGTATCTTTAGATCCTCGCGGAGGTCTCCGTTGTCGGCCATCAGGCACAAGTAGCCATCGTCGGATATATCTGTCAGCTGATAATCTTCCCGCTTAACGAATGGCACATCCATGTTGTGTGTAGAAGGGCAAATATCTTCGTATTTCTTGGAGTTAAATATGTCGATACCCACAAGGTGGACCTTAGCATGGCCGTGTTTTCCAGTCTTAGAAGTGGACATCTCGACAATCTTGCAAGGACGAGATTTTAACATGACGAAACCGTTTTTGCGAAGTGCAGAACATTGCATAGGATACGTCACAGAGGCTCCAGAGTCTCCAGTCTCGAAGTGAGTGTCCTCAATGTCTGCCATCTTTGATCTTAGAACTGATGTGATCAAAATGGATACTCTAACCAGAAGATCTTGCTGCGACT

>contig00667

aCATCGACAATTCTgAGGAATtATCGCCACCAGAAAGTAAAAGATTGCGTTTAGACGATGGCTGTAATAACAACCTAAaTGCACTATCTGAAACATCGAAGAACAACAGCACGCATtGGTCATGTtCAGGTGACACAAGTCTTGTGGAACCGGAAATTTTGGAGGACATGGGCGTCAGTATGCTGGAAGACGAGAATAGCTGTGAAAGTGATCTAAAAAaGTTGCGAACGCAAGACCCTTCGCCAACACTTACTGAAAGTAATGATGGAATTCTTGATGATGAGAATACGGTCTACAGCAGACGAGATTCTATGTATAAaCATTCCAGCTTTGAAACAGTCACAATGGATAAATATGATAGGCTCGAGGAATTGATAGATTTGGACGATCCAACAAGTTTCTCCACTGCAGGAAaTGAATCCGGATATGGTTCGAGAGCAGAAATTGAAATTTTTGGAGAAGCTAAACAGAGTATTCACAAGGAGGATCAGAATCCTACCTCAGATAAAAGTAATTTAGATTTACAATTCGGTTTACGTAGGAGAAAAAAaCCTTCAATCGTAGGAGAAGATAAATTTAACAAATTATCAGATGAGATGATCTTGATGATATTAAAATGGCTTCCAAAaCAATGTCTGGT

>contig00668

ACTCCGATGTCTCTTGCTACTGAAGCTTTACTTTCGCCACGATGGACCCTCTCGATGACTTCTATTTTCTCGGCTGGTGATAAGTATTTCTGGGGCCGCTTACTCACTTTCATAAATTCCATTTTCATTTCCCTGTTCTCCTTCATGCTCTCTAGATTATCATCTTCCATAATCCTGTCCTTCACTTCATTTGAGTTCTGGTTGATGTCGATCTTCAGCGGATTCAAGTtATGGATAtGGAAGTCAGAGACTGGCATTTTCACTATTCTTaGATCCATCGtAGTAGCTCTCTAGTCACGCGATTCTCaTAAAaCATACACTTTGAGAGTGATAAaCACCTTTG

>contig00669

ATTGTGATTGATTGTGATTGTAGTCGTTGGTAGCTTGCGTTTGCACAGCTGCGATTTCAAAACTTCACATTTCTTATGAAGTTATGAAAACAACTGTCATGAAATTAACAATGCTTCGAAAAAGTAGAGTCAGTAGAACGGTGTTGAGGTCAAACTCCTAGTAACCACGTGACAAGTCAGAGCTTTTTGGGTCCAATATTCAATCATATCGCAATCAACAATCCACCTAATTCCGCAGCTGTTTAATTTTtACGCAAAGAATACCACTTTGACTATCCGTAAACACAAGGAGAAATTGCTCATTCGTATCGGCAAATTTTGACGAAACTCGCACTTCACTTTTTCTAGCATATAGAGAAGATCAAGATGGGATCCGCTAGACCACAAGATAATTCTCTTGGGGGAGgCATTTCCTTGCCTAAGTGGATGAAGGGgAAaTtCGAAAACAaTTCGATTTCGATGAGAATGCATTTAGCCCACCTTCCCATGACGATAaTTTCTTTtGCATCCGCTACACAAAGAACCCGGTGATAGAAAATGCCTCTTCAGGATTCACATCTATCTCTGATGTAATAAGTGGGAACACAGAGACCAAACAGGTGCcTACTGTTCAGACAAATTTATCAGATTTCAAAGATACAAAGCAAATTGaTTTCAGTAAACACCCAGAACCTTGCCAACCaTTCGTTCCTAGTGCTGCTAACAAAGGGAaTT

>contig00670

ACTCTATTATTGACTTCTTGACATAAGTGAGGGTTTATGAAGTGTAAATAGTGTATAAAAAGTTACTTCATATTTTGCTTTAATGCTTTATGGAAATAAAATGTAGATGTCTGAATTTGAATAAGAATACATAGTAGGATTTGTATTTTTAATACTTGATGCTTAAATCTATAGAATTTTATATTATTTGATACAATTTGCAAAGAATTATTGGAAATGATTACGGTTATTCTACTAtCATTGGTTGCTAATTTGGAAAATCGACTGGACCGATGCAATTAATAATGTCATaGATCtAtAATTTtACTTTTtATGtAAAaGTAAAAAAAaTaCTAGAACA

>contig00671

ACTtAGTTGACCGATAAACtGGGAAtAAGTtAGCACAAAAatAAATTTTTttATTTtCtATTATGCTTTTTAAGTCCCATTCGATAATGTTTAAATTCTGTAACAAACACTCTTTAAACATTAAGAATAATATGACAAATTTTCATTTTTttATTCTTATTCCTTATTAACTCAGATTCACATATC

>contig00672

CGATTAATATAGTAAATCACGAAAGGAATCAGAaGGATAAACTTGATCAGATTTCAATCATAAAAATAtAATTTTCATTTCCATAAAATTTTCTGATAAAATACTTATGTATGAAAAaTTCATCGTCTGACTCCTTCACAAAaTGACATATTTGTGTAAaTGCGGCGAGGCATATTGTGAGATTTTtCCCcTAGTCACGCTTAAAACTGAGTTGAAAaTATAAGAACTTAAACCTTGAATATATAAAGCaGTAaCTaGGAAgAGT

>contig00673

ACTTTCGCAAGAAGTTATCCTGACGGGgATATCTACGTCTTTGGTTtAAaTAATTATCATCAAACTGGTTTAACCGAAGGAGACACACATTTTCATCC

>contig00674

TTTTCTTAACTGACCATCTAGCaTTGTTTTTAAATGCTTTACCCATAACTTTGTAGAAGCTTTCTCTAGCCTCTTTTTCGTCCGGCAAaTCATCGTTAAAATAGGGATCAATACTGTCGTAACTtCGTCGTTTAACAGGATTACCTAAAGTTTCCCAAGCGCGCGTTATGCAAGTAAAaTAATCATCATCTGGACGAACATCTTCACCCATTGCCTTTCGTTTGTCAGGATGATGCTTCAAAATTTTGTGTTTGTAAGCTCTTTTAATAATTTCTTCGGTCGCTCTATGTCTAAGATTTGTAATTCCAAGCACTGCATAGTGATCTTGATCTTTCCATTCCTTTGGATCTAACGATCGTAAAAAATCGAGATCATCTTCAAATTTATATTGATTGATGTCTTCTTCATCAGAAGATTCTTCATTTGCATTTGAGTTCCGTGGTAAGTTAGGTTCTAATATGGATTTTAACCAAGCCTCTTGGGATACATATAAAATTACAGCACCTGCTGTTGTTGCCTCAGTCTTACGAAGAGTCATAGTTGAATTTGAAATTATATGATCATCAGATGCTGTAGTTTTCGCCATGTTTCTCACTGAACCAGAATTTAAAAAAACGAACAATCAGTGCCTCAATAATAAAACGAAAATTATGAATGCAAACTACCACGCCATGAACATTTTCCTTGATTTTCCTGTGTGAAG

>contig00675

ACTTATGGTAAAAGCAGTCCAAGTTTCAGAGTTAATTTTATCAAAGTGAAAAAGATTTTGTTTTTATAAACAAGGTGATTGCATTTATGTAAAACTTTGAAAGAACAGAATTTTAGAAGTCTGGCAAGTGAACAACGCAAAATCACATGTAGTTAAAAGTACATCTCTGTGAATTAAAGATGTTAGTTGAACGTATTAAAATTGTTTAGATAAAAACATTTTTACTAGATCCACTTAAGTAGATCAGTTTCTGTAGTTAATCGAATCTCACCGAACAACTGCCATATGCCATTTATTGGTGTCTAATTAAACTCGCATTTGCTGACCTGCTGTAAGCACTTGTCGTACAAAATTTAACGGGGTCTGTAATAATATTCAAAaCCTACCTATATATTAGAACTAATGTCTAGATGAAAATAAATATTAATTACCGACTAACAACAGAAATACTTGTACAGGATAATATATATTGTGTAATTCAAT

>contig00676

gCAGTAAAAGATGCCTATGTGAACTGTTGTGTAGACGCGGCAAAGCGCGTAGTTAAACCACTTCGAAGAATTTACTTGTTTAATATAAATATATATTTATCACCGAATAGTGAAGTGAGAGTTTGTGAACTTGAAAGAAAACAGATTTGATGCTGCTTTGTGTTGTTACCGTCACCGGTAACAGTAGACTTTCAACTAAATAGCATTAGTGATTTATGAACGTAAAAGCGAAAAAaGTAGAATTGGTTGGCCGGTGATAAGATAAAGAAAGCAAAAATTTGTGGTGACGAGTGAAAGAAGGCTTTTTGCAGCACAGTTTCCTGGACAAGAGGCACATGCGAAAACTCAGCATGAAAGGCAAACGTAGAAAGTCTGAAACGAGCGACGGCGAATCAGAAGAAAATGGCGTCGCGTCGGGCGTCACGCGGGCTTCAGATAACGAAGCAGAGGAGACTGCTAGCGAAAGATCAGAAACTACAACACGTAAATCTAAATCCAATGGCAGAAAACGTTCATCGCGTCGAGGAAAGGCGGCTGAACAAGCGGATGAAACAGTTGAAAACGAGGAGCAAGAAAAGCCGGCCGGAAaCGAACAAGAAAACGGGACCGCTAGCGTCAAaGGGGGTGGTTCGAAACGTCGCAaGAAAGAGGAAAAACCTCCAGTTCCTTCaCAAGAAGAGGAAGATGACGAATACGAAGTAGAAAAaGTGGTTGCACagcGT

>contig00677

ACTACAGTTGGAATCACGATtGATTTTCACGCAAGTACTACaGtGAAGTCCGTAATAACTTCTGTACATGGAATAATGAATAGTATTCCAATACCTTTTCCACTACCAAATTCAGACGAATGTGCGAATTCAGATTCAGGAATTACTTGTCCACTTGAAGCAGGTGAAATATACACGTATCAAAGGATGTTGCCAGTAAGCCATTTGTATCCCCCGGTCCAAGTTAAAGTGAAGTGGGAGCTTCTAAATGAGCTGAGCGAGGTAATAGTATGCGTCCTCATTCCAGCAAAAATAACATAATTAATTGGTGAAGAAAAAaCTTCAGCTATTCAAAATTTGAACTTATTTATATTTTACAAATATCACTGTTACAATGTTATACGCTCACAGGGCTAAGTGTTTTATTATGAAAATTTGAAAAAGTGTCACAGAGTGAAATCGATATATCGAAGCTCGTGATTTTACTCATAAAGGCTTTATTTtACTATCACGTATTTtAAaCGACGCGTGTGGTTCATTAAATCTACGAAAaGCTTTGATTTTtACACCGCTTGACATAAAAatATCGCATGTATGTATTTG

>contig00678

ACTCAATCGTGTtCACTAACTTAGGGGATCCTCGAGTTTAAAAAaTATTAGATGAGAAAAaGCTAGAGGAATAATCGTGCCACTGCAAaGCACGGAAaTCGGACAATGATATCGGGGCATTTAATCTAACAGACTGATAAAaaTAGGTTTtAATtAGCGCTTAAGAATAAGCTCTAAAAAGTCTGAAGCaTCGCGTAAGCTTTCTGATTGAATCAGAAAGAGAGTAGTCTATTTTTCTGAAGATATTTTTAATGGGCAATCGAGAAGGCATAAtaagatGTATTTtCATCTTCAATTTGTTTAAAgTGGGCCTCCATAACTCGGAGTAAAAAAaTAATGCTCAAGTATTCGCGTAATTTAAAAaGAGGAAAAAaGCAATACCTTGTgaTATTAACAGATATAATTTGTTTGCTAAGGTATAATAATTATAATAGTAATAATAATATATGCATAAG

>contig00679

ACTGTGATTGGAAATAGTCGCATAGCTGCAGCTGTTGAGCATGAACTTTCCAATGGTTACAACAGCGGtGATGAATATACCGGCCGAACTGGCAATAATCTTACAATCGCCGAGTGGCAAGAACGCGATCGATGGTTTGAGAAACGTATGCGAAAACTGGGCTTTATAGTTAAGAAAATGGGCGAAGACGGGGCTTGTTTATTTAGGGCAGTAGCTGATCAAGTTTATGGAGATCAAGAtATGCATGGAGTCGTTAGAAAaCACTGTATGGATTACATtGCTGCAAACCAAGAATTTTTCTCACACTTtGTGGCaGAaGACTTTACCACATATGTAGATAGAAAaCGACAAGAATatGTGCATGGAAATCATATAGAAaTGCAAGCCATGTCTGAAATGTATAATCGAAgTGTCGAACTTTtCTGCTACAGTGATGaGCCTATCAACaTCTTtCATGGAGCACATGAAACAGACAATGAGCCAATACGATTGTCATATCATAGAGGTAGCCATTATAATAGTATAGTCGATCCTTACAAAGCTACTATTGGTGTTGGCCTTGGTTTGCCATCTTTTACCCCTGGAGCTGCTGATCGCTCCTTGATATCTGACGCCGTTCGTCAGAGTGAAGATTTACATATCGAACAGACTATGTTGGAAGATAAAATCAAGGCGACAGACTGGGAAGCGACAAATGAAGCTATCGAGGAACAAGTAGCCAGGGAGAGTTACTTGCAATGGGT

>contig00680

ACCTtGAAGATAGAACTGTTAGATTGCAACTaTGGGATACTGCAGGGCAAGAACGTTTTCGTtCATTGATTCCTAGTTATATACGAGACTCTACTGTGGCAGTTGTAGTTTATgaTATCACAAACGCGAATTcTTTtCACCAAACATCAAAGTGGATCGACGACGTAAGAACGGAAAGAGGTaGTGATGTAATAATCATGCTTGTTGGAAaTAAAaCCGACTTGAGTGATAAACGACAAGTGTCTACTGAGGAAGGCGAAAGGAAAGCTAAAGAACTAAACGTAATGTTCATTGAAACTAGTGCTAAAGCAGGTTATAaCGTTAAGCAGTTGTTCaGAaGaGTAGCAGCTGCGTTACCGGGCATGGACTCCACTGAAAaTAAGCcTCCAGAAGACATGCAAGAAGTAGTTCTCAAGGATACACCAATTGAACTGAAAtcGTCGGAGAGCAGTTGTGCATGCTAAATCTACAACGGTTCTGGT

>contig00681

ACGGGACAAGCTTGTGTTTTGTGAGTGAGTTGACTCTTGGATGACGGATCCGATTCTTTTGAAAAGTTCTGTTTACTACTTCTGTTTACAAATTTCTGATTAATATCAATAATAGAATATATTATCAATGTTTATGTGATTTTGTCAATAAAAGTTTGAAGTGGCTGAGAAATTTTTGCTTTGACGACCTGCGAAATAAGGAACAATGGGTTTTGAGTGTGCTGTTCCAAGTTGTAAGAATATATCAGTGAACACAGGCGGAGGTAAGAGGGTTTCTTATTTTTCATTCCCAAAGGACGAAAGGTTGAAACACATTTGGATAAGGAAGTGTAACGGAAAAACCTCCGACAACTATGATGAGGAATACATTTGTTCAGATCATTTTAGAGAAGATGATTTTGAGCGTGACCTTAGAGGAGAATTATTAAATATGCCGATTAAGAAGAAGCTAAAACCTAACGCAATACCATTTCTACTCTTGGGTAACAATGATGATGTGGGACCTAAGACAGTTACAGATAGGCAAAAACGA

>contig00682

ATGGGGGTAATGGTAGGTAGTATTATAACTATGAGATTTATAGGTTATATAAGAAGTTTAATTATAATAATTAGACATGGGCTATGTTCATCTGGTTTATTTTGTTTAGTAAATATATATTATGAACGAGTTTATAGACGAAGTATATTTATTAATAAAGGGATAATAAATATTGT

>contig00683

ACCTTCATTAAGGTTGATATGATTTATAA

>contig00684

ACCAGATAGACCTTTTttGGCtGTCTAGTATAAACCTGTATCTCAGTTATTAATTTATGAATAACTTtAAATAATtAAATGCAActACCGAtATCAAGTGACCGATTTGATAAATGGCGTTAAGGCTAAATAAATTTTGAATTGACaTACTACATTAACATACTATATGTCTAATTAATATAAAGAaTGCATTTGAAAaTGaTTGTTTttCtCCTGAGGCTCTTTAGGGTATGTATGGAGTCGTTAATGCAATGTCGGCAATTGCAATTACGGgTGTGTAAGGTCATTCGGAAACT

>contig00685

AAAATGAATTTTTATACCACACTGACTTTTTTCAC

>contig00686

ATTTTAGTTATTAGTTTttAAATAATcTtACTtActGTAATAGCAGTTGCCGAAGTTACCTCACCCATTTAAAaTGTGGCCTTAAAATATCGAGTAAGTATCAAaGgAATGATGTATGAAGGGCTACTGCACTTTTCGgaTTGAATTCAAaTTATATAGTTGATATTGAGATTAATCtAAATACTATACCAATGCATTTATCTTGCAAATGTTATACGGTTTCCGAACGTCGTAAAACAGCAAAAATCTAGCTTTTACTATTCTACCAGTGTTTAAAACGAtGAAaTCATATCCAAAAaTTTACATTTttATTCCAAAATATTGAATTCTACGATTGATTTGCCTGCGAATAGACGGAAGACGCGGCTTtCAGGT

>contig00687

AACTCCTGCTGGgACGTGACGAGCACGTCCCGAAACGCTTGAAAAACTTTCACTCTCGAATTATTTtAGTTTTACGCACTATACAGAATAACTTATGGAGCACaGGTTCATTAAAATTC

>contig00688

GGTCGTTTCTAACCTGTGCTCAAAAaTt

>contig00689

CTAGGGACAGAAAaaCCTTATGTGGCAGTTTGCCCTGTCAAACCTGAATCCATTTCAGCCAAGAAGGACGAGAaTGAAGACGACGATGTTGATCTGTTTGGGtC

>contig00690

TTtAGCATACGGCATCCACAAaCTCCAAATCTCTTGtGTTGTTGaGGACGATAAAGTATCTGTTGACTGGcTAACAGAAAAAaTTCAGGAaCAAGAAGAATATGTTCAAAGTGTCGACATAGCTGCATTCAACAAAGTCTAAACAAATTTTAAATAAAATGTTTCTTTTTTTGGAAATTTAGTCGTGATAACTACGAAAAGGCTTACTGTGCCATTTTTTCATTCCATTAGCAATACCTATGCGAGTATACAGACTAAATAAATTCAATGATGCTCCATtCAAGTCAGTGTAAAAGAAATTtAAAGGAAACGTATGGAAATAATATAGAAGGAGATTTCAAAACAAAGAATATTTGGTGGGAACACAACTGACATCAATTATATTTGAATAGATATCCATGAAGTTTTtGAAATTTtGTTA

>contig00691

ACGCGACTGTAGTATTACACGGGATCTTCAGTGTTCTCTTCAGTGTTGAAGAATTTTCTGTGTATCTATTTAATAGTGCTTCCGTAAATATCCACATTAAACCCATAAAAATGGCTGATGTCGAAGCAAAAGAAACCAAAGTTGTCAGCACGCCTGAGAAGAAGGCATTGAATGGCGATGCAGAGGTTGAGAAGCCCGCGAAGGTTGTGGAGGAAATTGACGAGGACTCAAAAACGTCAGAAACTGATGCTGTCGCAGAGACCAAAGAAAATGGGTCAAGCGAAGAAAAAGAGGCTGATGAAGTAGAAGAACAAAACGGACACTCCACAGATGCTCCCGTCGACACTTGTTGTATAAAGAGGAAGTCTGTTGGAGCTGATGTGACCGACGAGGTGTCGGCGGATGGAGCGAGTCCAGAAAAGAaGCAAAATTGGAAGAGAAACCAGCTGAGGCTGAGGCTGAGGCTGAAAGCAATGGATCAAAGGTAGAGGCTGAAGCCACTGCTTAATGTTCCGTAGTCCACTCAATCCATAGACCTGATCTATAGACACAGTTTCACCATCTAAaCGTGATGTCAGAATGCAACATAATCATCAGTGCATCGCCATAAAGACCGAGTGCCGATCGCAAAaCTCTGATATTGTTTACGATTACGTTTTGTGTTAAGAGTGGCCGTGTGTATGTTAAGTTCAAGATCTTGCGCATTATAGCCTTAAGGCGGTGC

>contig00692

AGTATaTCTCTGACCAGCTAACACGAGATATGTGTAACGTGCGCTAATGTTGAATGACGgTTATTAAaTATGAATAGCAATACAATtATTCTCTTTACTGTAGCGTGAAGAGAAGGTGATTTGTGGAGCTTTATTGTAAACAATTCGAAGATTTTCCAAGGAGTCTTTATAACTTGTGATATTTAGTGCACTTCTTAATCGGTCGGTTGTGAATCTAATATCATAAACTACTTTGTGTTATCAAGCCGTGCTAATTATTTCTAATAATGTATGTAACACTTTATCAAGAATACGAAAACTATGTTCCTAATTTATATACAAGTGATTTATTGATATTATTCAGAAAATCAACGACTCCAAAACAGAGAGTTACTAGAGATAAAGGTAACACGTTAGAAAAAGCACTCAATAAAaTTGGTCGTGAGGACATAGTAAAAAaGTGCATATTTAACGTTGAACTTGTAACCGACGACGTTGAGAAAGCTGTTGCGAGAGTGAGAATTGACCAGCCTGGATTCGATTCTCTTAAAGAAGAATTGGGACCTTCAAGGGATACTTCCCTTAGGCGAGATGCCACTTTGGACCCTAACTTTGGTCCAGaTTATGAAGAGCCTGATAGAATGAAGGACTCTGAATCTGTGGAAGATCTTACAATTACTGGTTCAATCCCCCAAAAACCAACCAAGCAGCACGTCACAATCACGAACGGCACTGTATTAAATGGT

>contig00693

TCTGAGTAATAGAAAAATCTACTCTTATGCGCCGACCATCGATTTCCATT

>contig00694

AcTTTatCAtCTTtAtATGGCCCCAGATGGTTTTTGCTTTGAtAtGtATtGtGGAAAtCAAGACTTGGATGATGACCCTAAAAGTCCTTATTACAATATAGATCGTAGGGCAGAACAATTTCTGAAATTTGTGAATAAACAGGCAGAGtCCTACATAACAAATAACgtGATCaTTACCATGGgAGGTGATTTtACTTACCATAGAGCAGACCCTTGGTTCATAAATATGGaCAAACTTaTTAAaTATATCAaTCAaCGCAATGGgTCGACGTATAATGTGATCTATTCAACACCATCCTGTtACTtGAAaGCTGTTAATGAACAAGATATAaTATGGCCAaCAAAAAAaGaCGATTTCTTCCCTTATGGCAGTGATCAGCACTCGTATTGGACTGGTTACTTCACTTCTAGACCAGCACTTAAATTCTACGAAAGACAGGGCAACAACTTTCTACAAGTTGTAAAaCAACTTTCGGTTATTCACGATCAACGCGACTCTTGGAAACTGGACGATTTCAGAAAATCAATGGGCATACTACAACATCATGATGCTGTTTCTGGAACTGAAAAACAATTTGTTGCAAATGATTATGCTCGAATGTTGTCCACTAGCATGATTTATGGGGAAAAAATGTCTGCTGACGCTTTGAGGACATTATTGCTGCATAACCAAACAACTAAGGTTAATGAAATACTCGAGATACATACATGCCGGTTATCAAaTATCAGCTCTTGTgaGAaGT

>contig00695

CCATAATTTCGTGTTGTTCCAAATTATGGGAAtCCAAAGCACCAACGTCAATTATATTtGCTGCTGTTTCGTgAAGAATTCTATTCAGTGCACTCTGTTCATCAGTTTTCTTtGGAACTGAACTCGCATACTGATTAACGTAATCGTCACTATGAACACGAGGAATGTTGGAATTGCTACTAACTGGATCCACCAGTAAGTGAGTTCTTTCACTAACTTCACTCTGCGGT

>contig00696

TAATTCTTCAACGTAAAAAGGAGACAAAGTtGGCTtCTTGGgTGTAAT

>contig00697

CGACTTCGCTTTGGATTTGGATTGCGACCTGGACTTtGACCtCTCAGCcTTGGACCTAGAACGAGaTCTGGATTTGGACTTTGACTTAGATTTCGACTTTGAGCGGTCTCTTGATTTGGATTtGGATCGGGAGCGACTGCGTTCATTTGGAGATTTGgATTTtGATTTTGATTTAGACTTCGAGTGAGCCCTGGATTTAGAGCGACTGCTGCGGCGACTACGgCTTCGAGAGCTGCGgCGACTCCTTGAaCGGGAGCGAGATCGACGGCGTGATCTGGACCTGGATCGCGACCTTGATCTTGAACTGCTCGACCTTGAACGACGGCCTCGTCTCTTGTCCTCAATTAAACGGATTCTGCGACCATTTAGTTCGGTGTCGTCTAACTTCTCAATAGCGTTCTtcAAGTCTGAATATGTAGCGAATTCTACAACTCCTTCATTTCTaCGTTGTTTGTGtGCaTCTGCGT

>contig00698

TAATGTGGCCCACCAGCCTTATCATTTGTTCCAGACATTCTGCTACCACCAAATGGTTGCTGTCCAACAACCGAACCAGTAGATTTGTCATTTACATAAAAGTTTCCGGCAGTGTATTTAAATTCCTCAAGTGCTCGTTTCGCCCAAACTTCGTCCTGTGCAAAAATCGCCCCAGTGAGCGCGTAGGGTGTGGAAGACTCAACCAGTTTCATAGTTTCATCGAGTTTCGAATCTTTGTAAACATAAATTGTGAGAACTGGTCCAAAAATCTCTTCGGTCATGATCTTATCCTTTGGATTTTTTGTCACAACAATCGTCGGTTGGATGAAGTAACCACACGAGTCATCATAACCGCCTCcACCGATAATTTCTAAGTCAGAaGATTTTTTtGCGTGTtCGATATAGCGTGTGAttCTTtCAAACGCCCGATCGTC

>contig00699

AcAGATAATATCAGTCAGAATGCCAGTTCCTGCAATCCAAAAaCCAGCGCCTGTCTTCAAAGGCACTGTTGTTGTTAATGGACAATTTAAGGATaTTTCGCTtACTGATTACAAGGGCAAaTAcGTTGTTCTATTTTTTtATCCACTTGATTTCACTTTTGTCTGCCCAACTGAGATCATCGCATTTTCTGATCGTGTAAAGGAATTTCGTGACATCGGCTGTGAAGTCATTGCCGCTTCAACGGACTCACACTTTAGTCATTTAGCATGGGTCAACACTCCTCGAAAACAAGGAGGACTTGGAGAAATGAACATTCCTCTTCTTGCCGATAAGAGCTCAAAAATCGCCCGGGATTATGGAGTTCTTGATGAAGATAGCGGTATTCCTTTCCGTGGTCTTTTCATTATTGATGACAAGCAAAACCTTAGACAAGTTACCATCAATGACCTACCGGTTGGAAGATCTGTGGATGAAACATTGCGTCTAGTCCAGGCATTCCAATACACCGACAAACATGGAGAAGTGTGTCCAGCTGGCTGGAAACCAGGAAAgAAAaCCATGAAACCCGACGTCAATGGATCTAAaGAGTATTTCAAGGACGTCTAAGAACTTTGATTtATTAATCGCGTTCTTAAAAGTGCAAAATtGAATCACATGTCTTTTATTCGACAGT

>contig00700

ACTtAACAAACTCTTCATGAAACATATAACTGATTTAATGTCAACTGGTGAAGTAGCTCCACAGTTGTTAGGTCGAGGCATAGAAATTTCTAATGTTAATATCACAACCGATTTTAAGCTTGTGAACGTTTACTGGTTTT

>contig00701

GGTTTCACTTGtGTCTGCACAGCATtGGGTTCTAGGTAAGGTCCAATACGTtGAAGGGTTTTGGCAACGTTGAACCtGACGTTAGCGACGTTGTCGTTCGCCATCGCCAGAACTGTTGGAAGCATCACTTTTGTTGTAATTTCGGGGCCACAAACTTCAGCTAAGACATTAATGCAGAAAAGACATGTCATTCTGTGAAGATAATTTTGGTCTCTCGACATCGCCAGCACTTTGGGGATAACCACTGAATTTTGTGCCCACTCAGGGCCGAATTTTTCAACTAACTTCTTCAAATTCAGAGTAGCTGCTTCCCTGATAGCATAGACATGAT

>contig00702

GCcTCTtCCtCATTAAGAGTAGAGTTGTGTTCACGTGTGGTTGTGTCTCCTTTTTGCCACAAAATAGGTCACACAACCCACTTCCAGTCGTGAATTAACTCACTTATAACTAATATAAAAAaTCCAAAAAAaaTATAAAAaCATTTAAAAAaTACAACACAAAAAAaTCGCAAAATCGTAGCTGAAAACCGCGAAACCCGAGGTAAAATAAAAAaGGTGTGGAACAAAAATACAAaGTGAAAATATTCTCGTTGATTTGCGTTCACGCGTGTTAGATAAAAGTGCGAAAGCCGTGAAAAACACGGAAAAGTGCAAAAGGCCTTAACAAGGAAGCTACAAGGAAGCTGAAAATTCGAGTATTTCCTGACGACTTAACGACGTTGCCTCAATACCGTAAAAATGAATCCTGGAGCGCCAAATTACCCTATGGCCTCGCTATACGTAGGAGATCTCCATTCGGACATCACCGAGGCGATGCTTTTTGAAAAGTTCTCCTCTGCTGGTCCTGTTCTTTCGATTCGCGTCTGCCGTGATaTGGTCACCCGACGCTCGCTGGGATACGCTTACGTCAATTTCCAGCAGCCAGCCGATGCTGAACGTGCTTTAGATACCATGAACTTTGACATGATAAAGGGAAGACCAATCCGTATTATGTGGTCTCAACGTGATCCTTCTCTCCGGAAGTCTGGAGTTGGTAaCGTCTTTATTAAAAACTTGGACAAAAATATTGACAATAAAG

>contig00703

ACTCAACAATTCAGTTTTCACTGTGCTCCGGCGTTGGATGTAGACTGGCAAACGAACACCTCTTTTGCAAGTTGCTCCACCGATCGgTGCATTCATGTGTGCAAaCTGAATGTTGATAAaCCCATTAAAaGCTtCCAAGGACATACGAATGAAGTCAATGC

>contig00704

ACAACTTAGTtATGACTTAaTTTTCTCATTTTCAATCAGTTATTTTTTtGTGTCATGACACtATTCTTCTTTGCATATGTCTTAGACACTTAGTGAAATGGGTCATTTTAtGATGAATTCAAAaCTTCATAGAATACACATTGATTGTGTgaTGATATAAAATTACAATATATGTCCAAAAaCtCATAGACATTTCATGAAAAGTCTAACATCCTTGTGCACGTAATGTCATCAGTTTTGTAAGCAATTCAATGATGTAAaCAATCATAACTCCACGTTAATCAATTAACGTCTAATTAAATCGAGGTAAGCATTTTtGGACCCTAAAGCCAaTTTTTTCCAACAACTTACCATTTTGTCAAAAATGAGGGTCAATACAAcTtACACTACTTTtAATTATTCTAAATCACACATCATTTtATTAGAATTAtAAAATCTTAATTATGTTTACTT

>contig00705

ACGAAAAATTTCTAAAGTAAATTTCTTTTTATCAGAATAATCAATTTTTTAACTTCGAATAAATGTAAATTTATTCCAAGATAATAAATTTTGTGATTTCTGGTGtCTCTAAATTCAAATTCAGAATTCATTTGGT

>contig00706

tAtGAGATCATCGAGATCGCCGACATACACCTCGCACTGAGCATGAAAATTTaTTTCAAGCGCGTGAGAaTTTACGtCTTAACTAATTTGGACTTGATACTTAAAAaCGaTAAACCTATAGAAACGTGATTCGCGATTTCCTTGTCTTTCCTCTAAAaCCTGGGACTTCTCAATTtCGAGTAAAAaCGCTCTTCTGTAAGTTCTGCATCATCACATTTACTTAGATTACACGTTAAACaCTGAATATGTTTCCCTGAATAAAaCAACGTCGATCTGTCTCAATATTTACAaGGCgCACTCGAAATAATTCTTtCTATTGTGATTtGATCCTTtAATTTTtGGAAAaGtAatGAAAaCGAaCTTAAGCTTCG

>contig00707

GTATCGAGACAAATTTTTTCCtAGTCGACACTGATAATACTTATCTTAAGTATGCC

>contig00708

GAAAATCAATCGATAGC

>contig00709

ATCTTATCATTTTGAGCTTGTAATTCGTGAGAGATTCTCATCACTTCACTCTTCATGATTTTtATTTCGTTATCCAATAATCGTGTCCGCGAGACAATCTCATCTGTTGACATACGTAAAACGTCTT

>contig00710

GGGATTTATCTtCAAGAGTAGTCATTCCGTATTACTTAGGTTTCTGGAAATGAAATTATTGATAAATTCCTTATTAATTAGTCACAAAAAaTATGAGTTAAGTAAATTTCTATGATTAGAAAAACTGgATATTAATAACCAAAAAGGATCAGAaGAAaTTTtCTTCTgCCGTTTTGCcAA

>contig00711

ACCTCATCACAGTTGTTTGTGGCGTAGGCAGTGGCTAGTTCCTGATAAGCCTGGCTCAGCCGCTTCATAAGTCTTGCAGCAGAATTCGTGAATCTAGGCAAGAATACCACCTTACCGTTTAAAATTAACGAGACAATAGTATATTTTTTGTAAGCCTCGAGCATTATGTGACTGACGGCCATTGCTGGAGTCGTCACACAGACTTCAAAAAAATATAAGGCCCTGTCGTAGTTTTTTAACGCCGTGTATATCATGCCACCGTAGTAAAAATAGAGTAAAAAATATTTTGTGTCCTCGTCAGACGAAGAACTAATGCTGGTGATATCGACATCCAGAAATTCCAGTGCTGGCTTGAAACATTTtGATATAAGGCAGAGCTGGCAGAGATcAGCGTGAATCGGTGTTAACTGACTGTCGCAGATTtGAATCTTACGTATCGCTCGACAC

>contig00712

CAAAACGACTATGACAATGATCTTGAGAATTTGGTGCCAATTAGAAACACACAAAAATCATTCATGAGTGATAAGTCTCAAATGAATATTAGCTTAAGT

>contig00713

TGTCTAAAGAAGAAAGTGCCATATGTGAAATTGAGGGTGCCATTCTTGAAAAaCAGACTACAGTCCCAGAAGAAGTTGGAGCTaGTC

>contig00714

GGCTGTAATGATGATGTTAATGCGGCGATTATCATACCGCGGGAAGACGGTGTTATCAGTGTTtGTGATGACAGAACTGTGAGAGTATGGTTGAAACGTGATTCTGGCCAATACTGGCCGAgTGTATGTCAGTATATGGCCTCAGGAGCTACAGCAATGGATTATTGTGTCGCAACCCGGCAATTGTTtGTAGGCCTaGAAAaCGGTTGTATAAaTGAGTTCATAATAGAACAAGATTATAAtCGAATGACTCCTATGAAaGAATTCTTAGCTCATCAGGCACGAGTAACAGGAGTT

>contig00715

CcTTTCTTTTCTTTTGGATTACCGAGTGCGTGTATGTTACGCGGGTAAACAAATTATAAATCGCAGCCATGGTGCGTATGAATGTTCTCAGTGACGCTTTAAAATCAATTAACAATGCCGAAAAGCGTGGAAAAAGGCAAGTGTTGCTGCGACCTTGTTCAAAAGTTATCGTCAAATTTCTAACTGTCATGATGAAGCATGGGTACATCGGTGAATTTGAAATTGTAGATGATCACCGCAGTGGTAAGGTAGTCGTAAACCTTACCGGCAGACTAAACAAATGTGGAGTCATTTCACCTAGGTTTGACGTTCCTATTACGGATATTGAGAAATGGACGAACAATTTGCTTCCTAGTCGTCAGTTTGGATATGTTGTCTTGACAACGAGCGGAGGTATTATGGATCACGAAGAAGCTAGGAGAAAACATCTTGGTGGAAAAATTCTCGGGTTTTtCTtCTAACTTGAtACAAAACAAAaaGTGTTTCAATATTTAAGGCTTACAGAATAAAGTCAATAAaTGTTGATACAGAATTACT

>contig00716

ACGATTTGGACTTCAAGAACGAAAAATCTGATCCTAGCACTTATCTCGAGCCAGAGGCTTTGAAAAATCTTTATTTGAACTTCGTTAAAGAATTCCCGATCGTGTCCATTGAAGATCCTTTTGATCAAGACGATTGGGCTTCTTGGACTTCAATCACTGCTTCTACTCCTATCCAAATAGTCGGTGACGATCTTACTGTTACGAACCCCGAAAGAATTAAGACGGCGATTGAAAAGAAGGCTTGCAACTGTTTGTTGCTG

>contig00717

ACAaTGGTGTCACATCGTtCGGGAGAAACAGAGGATaCGTTCATTGCCGATTTAGTCGTAGGACTCTCGACTGG

>contig00718

TTtACACCAGATGGAATTTCGACGTGCTCGATCCCATCCTCAATGTCACCTCTCATTCGTTTTGGATGAAAGTAATGCATTAAATATTGTCGTCGTTTTTCCTCCATCGCTTGCTGGGGTATGTGTAGATCTGGCCGTGTAAAAGACCAGGCAGTCGCTCTCTCGCAGACATGAATAATGCAGAATGATTCTGCGCCACACCAGTCTGCGAGTCTAGAAAAGGCATCCGTAACCTGCGTTCCCTGCATAATCTGGTGTTGTAATTAGAAGAATTTTCAATGGCTTCCCTGTATGCGGTGTCGTTCAAAAAACTTTCTATTTTACCTAAATTAGATTCGTTAACTACTTGAATTATGGAATGAACACTCGGTGGAATTGATACAGCCATCATTTTTACGAACAACTATCCATACTTGGTTACTTGGCTGTCCTTAGTTGTTGCTCGTTTATGCTGTTGGCTAAGTTGGCTTC

>contig00719

ActaTTACtAAtACCGTCTTTAAGAACaGAGTGAATGATATCTAGCGAAAGGCACTACAATAAaGCTATTAGAATTTTAatAAaCAAaTGTCAATAAATAAAaTAATAGATTTCTTAAAATTTGCATTCTTAAACTtCAAATGGTAATCTCATCCCTTAAAAaCTGTGAAATTCATCCAATTCTCTCTTTACATTCATACCAaTTTGTTTTTAATAAGAAATCCACAAaCTTTACGGAATTACCTTTCGATACGCATTAATTCAAAAATGAAAAAaTAAAaTCATTTtACTGGAAACTGACTAaCAAGCAGTGAATaTATGAATGCTAAAGCACACTGACTGTCGAATATATtATGCAATTTAGCGATTCCAGCCAGAACTAGCTAAACCAGCGGCCTTGATAGCTTCAACAAGATGAtGTTCGTCCTGTAACTCCGTTTtAGAGAAAaTATTATGATACAGATCAGATaTACGATCTTTATCTTGCATAGCCAAATTTTTCCATTCTTCTTCCAGTTGATGAAGTAGAACCTCTCTCTGTTCTGGTGGATTCAATCTAGTGATATCCTCGTCTTCCTCAACCCACTTCTCCCAAATATCTTTTGCATCTTCATTATAAATGGAAGAATTCTGGCAGATTTCTTCTCTTTCAGCAACACGGAGTGTCCTCAACTCTATGCTCGCAAGGCCACCTTTCTTCGCCTCATCATTTAAATGTAAAtCAAGAGTGT

>contig00720

AGCAGAAGTTCACAGATTAGTAGTAGTCGATGATGATGAAAAAGTGATTGGtATTATTtCTCTGTCAGATTTGCTGTTTTACCTTGtCCTTAGGCCTTGTGGAGAagATAGTGGTaGCAATAAAGGCAGTTCCATTTCACTGCGCGCACAGGATTCCGTCACGTCGAAACCaCCTAGCTCTGCGAATTCAGAGTCATCGCTCGCTGATG

>contig00721

TGGGCAGCAATCAGTCTTTt

>contig00722

AAAGACTTAAaCTTtGAGGTATTAAAaGCGATGGTCGAGTTCATGTATTGTGGAGAAACCACTA

>contig00723

GCCAGTCaCATTGTCGACTCCAAaCATGACAATGGAAGCCGTCTCAAGATTGACAGCCCcAcATATCACAGTCAAaGATTCATCCATATCTGGAGAGAAAAAaTCGCTAGACG

>contig00724

GAAGGTTCGTCGAAAGCACAC

>contig00725

ACCCCACACTCACGACGACATTGGTTGGGTGAAAACaGTCGATCAATACTATCTTCAAGATGTTCAGCATATTATTGGCTCAGTAGTAAAATCCTTACAAGAGAAAGCAgAAaGAAGATTCATTTACGTGGAAACATGCTTTCTATGGAAATGgTGGGTGGAGCAAAACGCAGAAaTGAAAGCTaTAGTTCGATCCTTGATCAACGAaGGAAGGCTAGAAATTATTGgAGGTGCATGCAGTATGAATGATGAGGCAGTGACCCACTATCATTCCATCATCGACCAATTTACTTGGGgCTTAAGGAAaTTGAaTGAAaCTTTTGGTGAATGTGCACGGCCTAAAATTGGATGGCAGATCGATCCTTTTGGTCACTCAAGAGAGCAGGCAtCAATTTTTGCCCAGATGGgaTATGATGGTCTGCTTTTCGGTCGTCTAGATTATCAAGACAAAGCACTACGTATGAAAACTAAAaCAACAGAAAtGATCtGGCGTGGTAGTTCCAGTTTAGATGCAAGCTCTAATTTATTCACcTCAGT

>contig00726

GTCCCACATCACTAACATTCCTTTAATTTTtGGT

>contig00727

TTCTTCGGTATTTTAGTTTCGTCCAGTTTGAAGATTACGATTGCAATGAGACGGTCATTTGGCGTATCCTTTTCTTCATTGTTGTTGGTTACTCTGCTCACACTATGTAAGAAGTTCTCAtATTTttCTTTAGAAGGCTCGATTCCAATCCCAATAAAAAGTGTTCCAGCTGCTATTGGTTtGTTAACATAATCTTTTAGTGGTtCGGAAaTAGTGaTACCGCACTTCGGAGTCACTAAGGCCACGCCACaTAGT

>contig00728

TATTGCTGATTCTATCGCTTtGTcAGTGGGTCAGGAATATGAAGTGAAGTTGCAATGTT

>contig00729

ACAAGACTTCcTTTCCTCGCCAACTTGACAGGAATTGTTCAAGATGGGTCGCCGACCAGCGAGATGCTATCGGTATTGTAAAAACAAGCCCTATCCAAAATCCCGTTTCTGTCGAGGAGTCCCAGACCCTAAGATCCGTATCTTCGATTTGGGAAAGAAGAAGGCATGTGTTGAGGACTTTCCACTATGCGTTCATTTAGTTTCGGACGAGTATGAACAGCTAAGTTCAGAAGCCCTGGAAGCAGGTCGTATCTGTGCCAACAAATACATGGTCAAAAATGCCGGCAAAGATCAATTCCACATAAGAATGCGGCTTCATCCATTCCATGTTATCCGAATTAACAAAATGTTATCGTGCGCTGGAGCTGATAGACTCCAGACTGGAATGAGAGGAGCTTTTGGAAAGCCTCAAGGAACCGTAGCTCGAGTGCACATTGGCCAACCGATCATGAGTGTGCGTTCGTCTGACCGTCATAAGGCTGCTGTTATTGAAGCTCTCCGTCGTGCAAAATTCAAGTTCCCGGGTCGCCAGAaGATCTACGtCTCCAAGAAATGGGGATTCACAAAATACGAACGCGCTGAGTATGAGAATTTGAAAGCGGCTGGCCGCCTTGCTCCCGATGGCTGCAACGTTAAATATCTACCCGAGCATGGACCCCTCGATCAaTGGAAAAAATACAGGGAAATGTTGCCTACTGTATAATTAAACTGACaCTTTAGT

>contig00730

ACGTAtAAAGAAATaaaGTCGAAAGGGGATAAATACAAATGCTAAAaTTGAATGAtaGCTATAAACTGtAGAATATCATTGCAAAAGCAGTGGAGCAACGaGATTAAAtATAAGCCATGTAATAAAATTACAGCCCCAACGAATAATGaCCTTGTTCGGATTTTTtAGTTCAAATTTTAGTTTAATTCGAGCCCATAGGAGTAACAAAATGTAAATAGATGACTGGAAAAACCATTAAATATAAAAAaTAGCCGCACTTGGTCTTTCATTTCCAAGTTCAAAAAaTAATTTtGACTAAAGGGAAGTAACTTCAAATCGTCATAATTTTTATGGTTCGAAACTaGAAAAATGAATTGAAAAAAGCGATCTTCTAGTCAAAGTGAAGGCAGCAAGTCTaTCATCTGTGATGTTTTAACTTTATAAAGTATTTTCATCAGAGTGCGGATCCATtGCCAAAACTAAAATCTTATAATAATTTATCGCCGATACTCGAAAGTATGTCTAATTAATATTAACAATTTATATTAGTGTAAAATAAAATCGTGAATATAGTCAGAAGTATTATACATTTTCACACCAAGCTGCTTTACAATAAACGATCCAACTTAATATTGGATATACAATACGTATTaGTAGTATAAtATTTTTGTAGTCATCtGCACCTCCTCAAATTtACAAGGGGGgTGTAGAAAaCGATTTtaTATTACAGATTCGAGTCaTGCCCT

>contig00731

ACGACCAAACGCtCAGACCGGTAGCTCTGGTTTCGCCACTATGCAAAACCAATTCAGAGCTGCGCCTCGTGTTCCTACTGCTCAAGCGGGAGCTATGCGCAATGCTATTTCTGCCAGACCCATCACAGGTCAACAAGCGGTGGGTGGAGCAAATATGCCAGGACGTTCGATGGCCGGTCCAGTGGTTGGGgTATCTCCTCAGAACCGTCCACCAAACTACAAATACACCTCCAACATGCGCAATCCACCTCAACCGATGGCTATTCCAGTCCAAACTCCGGTTCAGCAAGCGGTCCACATCCAAGGCCAGGAACCTCTGACTGCCTCTATGTTGGCAGCAGCTCCTCCACAAGAGCAGAAACAAATGTTAGGAGAGCGCCTCTTCCCGCTAATTCAATGCATGTATCCACAACTTACTGGCAAGATCACCGGTATGCTTTTGGAA

>contig00732

TATGGAAGGTGCAAAAGGAACCTTATAtGAAGGAGAACGCTTtCAATtACAATTCAAATTCAGTTCCAAATATCCGTTCGATTCGCCCGAAGTGACTTTCATTGGAGGAAaCaTTCCcGTTCACCcTcATGTTTATAGCAACGGTCATATTTGTCTCTCTATTCTCACAGATGATTGGTCTCCTGCCCTGAGTGTGCAAAGCGTGTGCTTAAGCATCGTTTCAATGTTGAGCAGcTGTAAAGAGAAGAAAAGGCCACCGGACAATTCATTTTACGtCAAGACTtGTAACAAAaaTCCAAaGAAGaCAAAGTGGTGGTATCATGATGACAGTGTGTAGCTATGCTCAACTGGCCAAaGTGACTATGTGAACTGTCGTCTTCAAGACAAT

>contig00733

CCACTGAACTGGGCCGGATTGCAGTTCACCAACCACAATTTGAACGTCGTAATGTAAAAaTATTGGCTCACTCAATTGACGAATTGAAAGATCACGTAGACTGGGTGAATGACATTAAGTCTTATTGCCAAGATATTCCTGGGGCTTTCCCATATCCTATTATTGCCGACGGTAATCGGGATTTGGCAGTAAAATTAGGAATGCTAGACGAAGAAAATCAAAAAGATCCAAATTCCGCAGATACTGTCAGAGCTCTTTACATTATTAGCCCTGATCATCGTATTCGTCTTTCTATGCATTATCCAAAATCAACCGGTCGAAATGTC

>contig00734

GTtCtCGGTtGAAAACGtGGAAATGTGGAGTGTCATTGGCGTTTTTCTTTACGCTTTCGTGGCTTTTTCCAACACATTGGATTCCATTTATCCAATATGGCCACAAGATAAAaCAAATACTAAAAATTGCAaCCTGAGAGTTACTTTTATGGCTGG

>contig00735

ACTGGAGCTTTCATAGCTCGTTTTCATATTTTAACAGTAGCGTTGTGTCTCATAAAATTTTTtACGAATTtAGGCCTTCCTTTGGAAAaTTATCATGCTCAATCTGGGAAAATGTTAACTCCTTTTGAAGTGTTGCGATATCAATTTGAAAAAGTCGAAATTCACAAAGACTATAATTATCGGGATAGGACGTTTCTTTATAACAATATAGGAGTTATAACGGTAGCGCCCGAAGATTCTTCACTGAgTTCTGAAGTGAAATTATCAACGTTTCAATACACATATGATAGAATAGATTCCAAAATCGGTGCTCACGTAATTGGTTGGGGATATCACCGACTTtCCGCATTGAGAACCGAAAAGGAATTATTCTCTGCTCATCTTAGAATGTTTCCATATGCCTTCTGTTCCcAAATTATTGAaCCCAAAAAaaTGGATAAAGATAAAATGTTTtGTGCAATTAAATGGAaTCCcAACGATAaCATtCACTGGGATACTTATGTGGACcGTGGAGCTATTTtAaTGAAGGGt

>contig00736

ACCTTGTtGAGGTCCGCATAACACTtCTTTACTTTTtGACGATTCTCCTTCTTCGACGACTTTCTGCAGAGGCAACTTGTGTATAAGCTCTAATAAATTTTTAATATAAATTTCTCGAGGCTGTCTCTTTGGGGTAAGCGACGGAAGGCATTTAAACGTATAATACAAAGCGTCAGGATAGTCAGAGATTTCCTCAAATCTAGCAATTAAATTAGCGTTGTCTTTTtCCGGAGACAAAAGCGCCATCAATA

>contig00737

ATAATTAGTCTTAGATATTTAACGTGAATTTATATTTCACCTAAAGTAAAaTATGGCTTACCTTTCTCCTGGAATTGCTGATTCGGCGTCAAAAGATAACCTCACAAATGGATTCAGTGAGAAAaCAGAATACAAGTTATATGTGACAAACTTGCCTCCAGAAcTtAACGAGGATGGCATACGACAAATTTTCAATTCCTAtGGAGATGTGACTGGCATTTTGTCTCCTCAAGAAGCTGGATGGGCATTCGTTACTTACCGAACTCACCGTGAAGCATtGCTTGCAATCGGCAAACTGGACGAAAAAAGTCCTCTACaTTTGAAAGTTGAATTTTCCAAAGAAAGGCAGGCAAAGAATGTAAAAGATTGGAAAAAGCAAAAtGTGTCTCAATCTAACATaGCAGTTGAAACTGCAATAGCTTCTGACTACAGAGACTCTGGAGGATTCAGTCAGCGTAATAATGTATCCCAGAGAaGACTTGAAGT

>contig00738

CGTCAAAaCAGTATCCATAAAAaTTAATTTtATTTtAAAATAAAAaTTCaGAGATTTTCCAATATTGAaGCGAAGAATTTAGGAAAGTTTCGCTGTAAAaGTTACAcAaCAGAATCTCTTGTATATTTGTTCCTCGATCAGACgACGtATTTTGAAGTAAAATtATCTCTGTGAATATaTaGA

>contig00739

AAATAAGAAAAATATGGACGCTGTCAGTCATTTTGATAGTCGA

>contig00740

AAGGGGATTCGCAAGATAACTGTATGCGCAGTTATTTAACTGCTTCATACACTTGCTAGATCCCTTAAGGCTTGAAATCTTACATATCAATATACAAAAATTTTATTTTTCCGGAAAGTGAATCTAAATCCACGA

>contig00741

AGTTtACGAATGGATATTATTGAAACTACTATAAtAGTGTGTGTGTGTAAGTGTGATGAAAaGTGAATTTtATTTTACTCCCAAAACTATTCTCTGACTGATTTtCGGAGtAATTTTTCGAAAGTCCTTCAGAAGAACAACAaGTaTTCCATTTGAtCAACAAGCTGCTACACTTGACTTTTTTGGACTtCTTGGAGAgAaTTGCATTGAAGGTGGAAGAGAAATTGGCGACGGAAGTGAGGCAATTAAGAAAAAAaTATAGAATGAAGAAGTGAGGTGAGTTCGTGGAGCAACGCGAATCAACTCACACGATTCGataGAGAGAAGAAACCGAAGTCGAAGGAAGGAAAGAGGCGATTGGAAGAtGAAGAGCTTGCTGTTTCCATCAGCCTTGATCTTCGTCGCGGTGTCTACGGTCCTCCTTTtAGCCACGACAGTGTCGACGGCCCCGACAAACACACACATAA

>contig00742

ACAGTCTAGAACAatATGGCGGATCTGCAGGGAACCCcGGTTGCTCAAGACGCCCTGTCCGTAGCTCAACTTGAACTAGCAGGGAATCCAAATGCCTTGGCGTTGCGTAGACCATTTGATCCAGTGGCACATGACCTGGACGCAACCTTCCGTCTCACAAGGTTTGCTGACCTAAAAGGATGAGGGTGTAAAGTCCCTCAGGAGGTTCTTGGTAAACTCCTCGAGGGACTACAGGCTGACGATGGTaGCgCCcAGGATCATGAACATGCCCACTTTCTGCACATGGCTATTCCACGCATTGGCATTGGTATGGATTCTTCTGtCACtCCTTTAAGACATGGTGGCCTAAGTCTAGTTCAAACGaCAGACTTCTTctATCCTCTAGTCGACGATCCATATATGAtGGgTAAAaTCGCGTGCGCAAATGTCATCAGTGATTTGTATGCGATGGgAGTAaCGGAATGTGACAATAtGTTAaTGTTGTTGGGaGTCAGT

>contig00743

ACtGTGTCTAGtAGTTGTTTATGGACTTCTTCAACGTCTTGACTAGCATCGATtaCCtCCCAGTTTTCGCTTTTCAGTTTGTAAAaTTGTCAGCTGCCTTGCGCTGCAGTTCTGTTTtCTCGAACCTCtCTTGGCCCCATTCACTTCGAGAGCGCTGAGATTCCTCGTCGATGTTCAGAAAAGCTACGAGATCAGGACTTGGAAGTCCCTTGTCTGCTTtCTTGcACCATTCCAGATcTTTTCCCGTATTTGCGGCTGTGTAAGCTGCTCCAGAAGCAGCATATCTATCGACTATCAAAGTTTTCCCAGAATTAAGAGTTTTTATCATATCGTTTGCTCGTTCCCATCGATTTtGTGAAAaGAGTTGATGAATTTCTTCTGGATCCAAATCCAATTTATTCGATAAATATTGATTGATTTTGAAACCAATGACTGTTGTTCTATCTGGAAAAGATATAGCTTCTGCAGGAATTAAGCGATCATTCAATGCTTGTAGGAGCA

>contig00744

ACGTCGAATCTAAACCCGTGaCCTGCAAaTGTGGACACACCTTTTGTTTTCACTGTGGTGAAAACTGGCACGATCCTGTAAAATGTCGTCTCCTGCGAAAATGGATAAAAAAGTGTGACGATGACTCAGAAACATCAAACTGGATCGCCGCCAATACCAAGGAGTGTCCCAAGTGTAACGTTACGATCGAGAAAGATGGTGGTTGCAACCACATGATTTGTAAGAACCAGAGCTGTAAGACAGAGTTCTGTTGGGTTTGTTTGGGACCCTGGGAACCACACGGATCAAGTTGGTATAGTTGTAACAGATACGATGAAGAAGATGCcAAAACTGCCAGGGATGCGCAGGAAAaGTCTCGAGCTGCTCTTCAGCGATACCtGTTTTACTGCAATCGCTACATGAATCACATGCAGTCTCTtAAGTTTGAACACAAaCTCTACGCTAGCGTTAaGGAGAAAaTGGAGGAGATGCAGCAGCaTAACATGTCCTGgATtgAA

>contig00745

TTAGCTAAATGCACAGACTGCAGAATTAAATGCATCAAATAAAAGTCAGCTAGAAATGCTGAAATTTAATCTCAGATTAAAATTAATTTTGAATTTAATCTTTAATTTTGCTGGAATGGAACTGACATTGCGAAGTGATGATGCGGCATGAATAATTAATAAAATGAGAAAATAAACTCATTTTAGGTCTGAAATCTTTGTTGCATTCAAAAGCGGAGTAAAACACaCaGAATT

>contig00746

ACGAaCAGATGGTTACGCTTATGAGATGGGGAGCTCTTATGGATGTAACAAACGAAATCGCTAAGGAGTATGCTGTCCAGACTGTCCAGATGCCAGTTATGCAAAAATCTGGGAGTGGATTTTCTGTTGTTCCGAGtCAaCATATAAATGAGAAAGGAAGAaTGTAGTGTGATCACCCTGGTAGATTTCACTACAGATCTCACTTATATAAATCTTTTtAGATTCAAGAAATTCTTCAATAAAaGTTTCGAAGGATAGAATTATGATTGTATACTTTGTTTGTGTAAATAGATTTAGTTATATCGTGAGACTATGTATTTCAGAAATATGATGTCTACAAAGCGCGGATAAACCAAgAAaTACATGCCTTTAAAATAAGCGATATCTAAT

>contig00747

GATTGACGAGAAGGAATTAGTAAAACTAGCAAAGCGTTTAAAAAAGGAGAAAGTGAATGTTGACATTATAAGTTTTGGTGAAGAGACAATTAATAATGATGTTTTGACTTCATTTATTAACGCTCTTAATGGAAAAGACGGAACTGGAAATCACCTAGTGACAGTGCCACCAGGTCCTCATTTATCAGATGCCTTGATTTCATCTCCGATCATTCAAGGAGAAGATGGCTtGGGTGGCGCTGGAATGGgAGGCGCAGCTTTCGAATTTGGCGTTGACCCTAACGaGGATCCAGAGCTGGCTTTGGCTCTTCGtGTTTCTATGGAAGAaCAAAGGCAGAGACAGGAAGACGATGCAAGACGAGCCCAGGCTAAAGATGCAACGGCAGAGAAAAAGCCCGAGACGATCAAGGAAGCGCCAAACGAGGAAGCAATGTTGAAACGCGCACTAGAAATGTCTCTAGAAGGAGGTGAAGAAaCgACTCCAACTTCGGATACAACGACTCCTAGCTCTGGAAACATGCCAGATTTTGCTCGCATGACAGAAGAAGAGCAAATCGCTTTCGCTATGCAAATGTCTATGCAAGATCAACcagAGACAGATCCACCGAAGGAGGAAGCGATGGATGTAGAGGAAGATTATGCAACGGTGATGTCCGACCCGGCATTTATACAATCTGTGCTGGAAaGTTTaCCAGGTGTGGATCCTCATTCGGAAGC

>contig00748

CCCCTtcGCCTTTTCTGTCAACGGTTTTCGTGAACGCATGCTTTTCAATCCATTGTTTTTtATCGTCCTCAACTGCCTCCGGCGCTTCTGTATGTATTTTACCATATCACTGGGTTGAACTTTTGTATATCCCATGAAGACAGCTCCTAAGTTGAATCCAGCTCTCATGATTCGTAATAGAATTAATCCTGGTAGGAAGAGGATCCACGGAAGTGGTCTAACAAGTTCGATTTCACCAAAGGTCTTTCCATTTTCGTCTGCTGCCATTTCTTGGGCTGGAAGACTCCATTGAGCCGATTTTTCAACCACCCACAAAATGCGGTCAATCAATTTGAGAAAAGTTTGACCAACAGATACACGTGATAATAATTCAATACTGTAAATTACTAAGCCTCCAAAACTATCGGAAATTCTCGTTGACATCGATATTGCCCTTTTTtCCTCCATCATTAATTTActgTATTTTCTTAAGTCAATACCAAATTTTTCGAGTCCTCGAATGAACACTAAACACAACTCGAAATACGAAAGCATTCACTTACTCAATTCATCAAGACGgCCGGCGTCAAACT

>contig00749

AAATTTTCTACTAAaGACAGctATCTTATGACCTGGGAGCCATTCATCGCAACaCCGAAAaTTCCTCAAGGCTCTCCAAaTCTCAATATCTGGAAATCCGAAAaTGGGgAGCTAGTGAAAAGCTTCATACAAAAAAACAAACTGATTGGGAACCTCAGTGGTCAAGCGATgaGAAGATCTGTGGAATCCTAGTCAATAATGACGTCGTTTtGTATGAGGCCGTAAaTTTCGAGAGAACTGCTCACAGAATAAATGTATCCAAAGTAGCTAAATTCGGTATAGCTCCTGGAGGACTTCCTTACCACATTCTTTGCTATATGCCTGGAAAAGCTGGCCAAGCATCTTtCGGTCGATTGTATCAATATCCGAAATTtGaTTCAACTCAGGCATTAGCAAG

>contig00750

ACATGACTTCGAAACGTCGAATAtAaCTCATtACTaccTATTATTGCctGAAGAAGtCGTTTTTtCcACCCTTCaTAcTTtCACAcAATATGGATTcTCAGACgAgTTtAACATCAACATCATGATCTTTGGAAATTCTAGTATGTTTTACATATGAGTGCAGTGGGTCTGTCATTGGAGCTCAATGTGCATTTATATGCCCAAAAGGAGGATGCGCAGCGCGTCACGATATCCAACCATAGAGTGCAGGAGAGCACACGAGGGGAAGAATGATCCGCAGAC

>contig00751

ACTTTGGAGATTTATACGTTGGTGAGTATGCATTGTAATATAATCAGACCTGTAAAATGATTTTtCTTTTTTttAATATATATTtCAACACAACAGTTtCTTAACGTTTATCCTTTGATTGCACTTACAAAATCACGACAAATGACCATCACAGATGAGTAAATAGTAATACTTAATTTAAATTTTGCGCCACCGAATGAaCTGTCAAACCGCGATATTCATCtAATATTGTGATTCGCCATGAAACAaGGgATTAAACATGTAATAGTGTTTtGTATTGAAaT

>contig00752

ACtaTtGGGATGTGTAATTGATTTTATATAAATCTTCAAGATTCTGGGTCTCCTATAATGGAATTATTAATTAAATTTCATGATTATTTATTATTAATTGATTTAATGATTTTAGTTATAATTTTTTATTTGATTTTAAAAGTTTTATTAAATAGGTATATTAATTTATTTTTAAAAAATCAGTCAATTGAGATTATTTGGACAGTTTTTCCTATAATTATTTTAATTTTATTAGCATTACCATCTTTAAAGATTTTGTATTTAATAGATGAAATGTATATTCCTCTAATATCTGTTAAATGTTTAGGTCATCAGTGGTATTGAAGTTATGAATATTCTGATTTTATAAATTTAAATTTTGATTCTTATATAAATAAAGATTTATTAAATATAAGATTTCGTTTATTAGATGTTGACAATAATTTAGTTTTGCCTATAAATGTACAAATTCGTTTATTAGTAAGTTCTGTAGATGTAATTCATTCTTTTACTGTTCCTTCTTTAGGGTTTAAAGTTGATGCAATTCCTGGTCGTATTAATCAAGTTAATTTATATATTAATCGTCCAGGTTTATTTtaTGGGCAATGTtCAGAGCTTtGTGGGACTAATCATAGGTTT

>contig00753

TAACTTCTTCAAGTAACATTAAGATCTGATTCTGTAGAATTtCTTTTAATCTTCCCAAAAaTTTTGTGTAATAATCTAGGTTtCTTTTTtCTCTTTCACCTAATCTGAAGGCAGTTTATTTtAATACACAAACAGAATGGAGTTCGCTATACGTTTTTTCATGGTATTGTTGAAATATATAGCAATAATTGCGCAACCTATATATtGGTTTTTGAATCGCCAGCGACCACCAGATATTCCACCTATAAAAAATCCACTTCTGTTTCTCAGTGCTATAGAGCTTTCAAAGAAAATCAGAAACAGAGAGATTTCTAGCGAGGCTGTGGTAGAGGCTTaCATAGAACGTATAAAAGAAGTGAATCCTCAATTAAATGCTGTGATTGAAAATCGATTCGAGGCAGCAATTAGAGACGCAAGAGCTATTGACAAAAaGTTAGAGGCAGGAGAAGTCAACATTTTCAAATTAGAAGAAGAACAACCACTTTATGGCGTCCCATTTACTGTTAAGGAATCGTGTGGCCTGAAAGGATTGAGTCAAACaGGATGT

>contig00754

ACTGTTGTGGGAGAAATTTTCATTCTTGTTCAAGAGGTCCATATCAATGTTTACATACTAtACTTTTCAaGAAACTATtGGATTACGTGGAATGGACGTTAGTCTTGAATTCATTGGCAGTAAAAGTGTTGCATTATTATCTCTGATAAAATCAAAAAATTCATTAAACTTAACCTATCACGAGACAGAAAATTCGTTAGAGGTGATAAAGATGACTTTTCATACATGCTAAAGATTCCGTAG

>contig00755

GAAGAaCTTTTaGCGTTGCTTCTTtACGCTGCATGAaTCGATTTCGCGATGTCGACACCCGTCAAAGTTTTCACGAGCTCAGGGACGCGAATCACGATGTtCAGAACCTCTTCTGTTAACTTTTCAGCTCCCAcTGTGCCATTtCCACTTGAAaCcATTGTGATTTtCTTCGCCTGCGAAAGTGgTGCAGCAACTTCAGCAGCAACCCTGGGTAGTGTTtCAAGCATCATGTCGATCATAGCAGCGCTCTTGTATTCGTTCATAGCAGCAGCTTtCTTTGCCATTTGTTCAGCTTCAGCCTTTGCTTTTGCGTCAATGGCATAAGCCTCAGCATCTCCACGAATTCTAATAGCTTCAGCTTCAGCCTCTGCTTCCATAATTAGTCGAAGGCGATTaGCTTCAGCCATTTTCTCTAATCTATATTTCTCAGCATCAGCAGGACGACGAACAGTAGCATCTAA

>contig00756

AGTTTGGTCTCCTTATTATTCCTTCGTGGAAAAGTCTCACGAATTGCGAATTATCTTTCAAGTTGCTTTATGAGCGGCTGTATTATTAGATTATTTTGCGATCGTTTGTGTAACAGTGGAATTTTTCGATAATAATTTAATCAAATTTAATTAATATTTTTGATAATGCCTGTTGATATTCCTGGATTTGCTTATGCAGCAATTGTCGCAGCCGGTGGAATTATGGGTTATGTAAAGTCTAATTCAATTCCATCTTTGGGAGCTGGTTTGCTTTTTGGATCTTTATTGGGAGTAGGTGCATATCGTATATCACAAAACCCGACTAACTATGCACAACTTTTGGGAACAAGTGCAGCTCTTGGATGCTT

>contig00757

ACTtctATCATCCCtcTACACTCTCAGCTGCCTAGAGAAGATCAGCGAAAGGTATTTGAACCGGTTCCTCCGcATGTGACgAAGATTATTCTGTCTACAAATATTGCAGAAACTTCcATCACGATTAATGACGTAGTCTACGTGATTGACTCCTGTAAagCtAAAATGAAGCTTTTCACgtctCAcAATAACaTGACCAACTATgCTACTGTCTGGGCCAGTagAACTAaTCTtGAACAACGCAAaGGTCGTGCAGGTcGTGTCAGGCCTGGTTTTTGTTtCCACCTTTGCTCCAAAGCCcGATACGCTAAATTaGAcGAgCATATGAcaCCAGagATGTTCcGAACTCCATTGCACGAGTTGGCTTTAAGTATTAAGTTGCTCAGGCTGGGAAGCATTGGCCAGTTCCTTTCTAAGGCTATTGAACCACCTCCTATAGACGCTGTGATTGAAGCTGAAGTTATGcTGAGAGagATGAAGTGCTTaGATAAAAATGAC

>contig00758

ACTAaGAGATTCCCTGGTTATgATAACGAAGGCAAATCCTTCAATGCTGATGTTCATCGACAACATATTTTCGCACAGCATGTTGCTAATTATATGCGAACTTTAGAAGAAGAAGACGATGACGCATTCAAACGCCAATTTtCTAAATACATTCAAAATGGAATTACAGCTGACAGCATTGAGAGTATTTACAAGAAGGCACATGAATTGATTCGTGCAGATCCcGAACACAAGAAGGTTAAGAGAGATCATCCACCGATTAAGAAACGgTGGAaTCGTGCAAAACTCAAGCTTTCGGAAAGGAAGAATCGTGTCGCTCAAAAGAAGGCGTCCTTCCTCAAAAAaCTCGAGGAAAGCGAAGATTAATGAGTTTAACTGCTcTTTTTTCCGACTTTACAGCGTTTtACCTGCGGAGAGTTTTtACATTGTCTATTTTTTtCCtAATGTATTTtACATTCTTCGTAGAAGAAACGCTGTTTCGAGGGCGTAAT

>contig00759

ACCAGTTTTTTAAaTGAATtCTTACAagTGgCAGAAATTCACAACGTAATGAAATCAAACAAGAATGATTATAAAAAaGAACCTGCAATCATAGAAGTTGTTGAAGACCAAAGTAAATATTTGACGGAAGGCTTTCCTCAAATACCAACATCGCCACCTAAATTCGAGACACCCGTAAATGTATTtGGATTGAAaTTAGaTTATaGCAGCCATAAATTTTCTGTATTAACGTTTTCATCTACAATTGAACCAAAAAaCGATTGCAATATGCTAAaGAGTAAAAaTGAAAAAGAAGATCCGAAGTCTCTTTGTGCCGCCATCACGGCTAAaCCAAAGCCTTGGATTTTTGAAACGCTTGATAGAGGTATGCCTATCTTAACCAACGAAGCGATATTGGGAATGTTTAGGTTAATGGCAAAAACATTGGAAAAAAAaTACTTCTCGTTAGTATTGCTGGGCACAAAGACTGGATCAATGCCATTTTATACGGCAAGGGTGACAAAATAAaGgCGTAGAAGAGGTGAAAAATGGGGaCAAAaTAGAAGTCAAATCGGgATCATAAATGAGGAAaCTGAAACTGAAGATAATAATACTAATGATGATAAATAGATGgTGGGAAAaCTATAAATTTaCAAAATTAAGAATAAATAATTGTTATATtATTTTTGAtAATTTTtgTTtCAATAAaGAAAAATCATTTCTTtcAAAAAAaTT

>contig00760

GGTGTGGGAGTGTGGAACACCAGGAGCTGGCCCGAAGTGTCTCACCGCTTCACGGGCCTTACGACGTCCCTGCATAAGGACAGTCTTTTTtCCAGTTGGAGCGCGCAGGGCCAACTGATCGAAAGAAATGATCTCACCTCCATTTTTCAGGATGCGGAGTCGTGCGGTTTTTGTTACTCGAAGAGCACATACCGTCAACTTAGGGACTTCGAAGATCCTGGAATCGTCAGTGACAGTGCCAACAATAACTGCGATGCAGTTTTCACGGCCTGGTTTTTtCATGAAACGAACAATTCGTGCTAGCGAAATTGGTGGCCGGTTAATTTTGCTCATGAACAGCCTCTTGAGGATGATTTTATTAAATTTAGCGCTTGTCCTCCTCGCCAAGAACCGATAAAGCTTAACGAGAAGTCGCAAATACACGTCTTGTGACTTCGGTTCCGTTCGGCGGACCTTTCGGTCATGTTTATGGTTGATGTCGATACCCATTTTGACAAACCGGAAAAgAG

>contig00761

CCAATAACGTATAGCATTGAACTGGTGACTGTAGATTCATGGAATGGATGGCTAAGTGATTCATCATTACAAAAAATCCTCGTTGATATGGTTTGCCAAAAATGTAAAATGTCAGTGCAATTAATCCAGTTATGAGAAGGCAGAAGAAATCCAGAACAATTTTTCGCAACATAATCCTTGAAGATCGATCCATTGTGGAAATATACAAAaTAGGGACCTTTTTGCTTCTAAATCGTCTTAGTTTtCTTAAAAACAAGGTTAGGAAATGCCGGAAGAATGAAAAAaCAGTTTGCAACGCGGTTGACAGCGCGACGGTGTATAAATATTACGAGGAATtGAAAATATAAACGAGACGAGAACCATTGCCTTGAAAGGCTCACTCTCGACT

>contig00762

GCAaCCTGAaCGATTGGTCCTATGCTTGCCTGGTTCCGCGCCGTCGGAGACCGAGTAGGATCTTTCA

>contig00763

GACtCCAATATGACAAGAagTCACCGCCTAGCCTTAATCACAAAaCAgAAaGAtCCCCATTAAAATAGTAAAAAAaTGTGACAaTACTGTAACTGATTCTTATATTATGAAATATTTTTATAACTAC

>contig00764

ACGtGtAAAAAaCACTTCAACTGCCATAAATCCATCGTAATTATAAaTGGATACTtCgACTTTTAACCCGCAACATCACTCTGAAATATATGTATAGAAcTCTGTCTCTGTGAATAGGTGTtgTTtCACTTtCCTTgAAAAaCGCGACAAagTAGTAAAGCATTtaGCTGAACgTTCACaCGTGtATTCTTaGGCAAAAaaTTCGGAaTTTTTTtCAACAACAATGTGTGCTTGAtGTTtATTTCAAGGCATTtAaCTCCGATCGTAGATTCAAGCGGTCAATCATCCTTCGATTTCGGTTCTTCGTCGTTCTTAATTTCTtGCGATTCTTGCTGACTTCCTTCTTCTTCTTCATCTGAGTAATTtGTTGGAGCTTCTCCtGGCTTCAAAAGCTTTCCAACGTAATCATACCTCTCTTtAAaCTGTTCTTCCCATTCCCTGACTGAATTCATTtCAGCAGTGTTTAAATCACTCAAATCATCATATTCGTCTTTTCCTGGCGATACTGAAAaTGTGGCTAAACCTCTACTAGCATCACTGCCACCAAATGCTGCATATGGCCCACCAGGGCCGTAAAATCTTGAACCTCTTGTAACATCATATACACTGCCGTTGACTGCAACAAGGACTCTACCATCAGGTCCTGTtCcATCAtAGGGTTTTAGTTCTTCAAGCGTAAAATCTCGCCTCAATTTTGGCAACTCCTTAATCTGT

>contig00765

AaaaCggggAGAGACGTAACCACGTGTCTAGTTTAATGTTCTTGTAAGTTTTGACTATTTAGCAGATGCAAAAAGTTGATGATCTATTAAAGTTTAGTTGTGAGAAACGTGTAGAATAAGTTTATCCTGCAATTCATATCATCATGTTACAAAGTGCATTCGATGATTtAAAACGCATGAATAAGCGGCAGTTCCTTTACCAAGTACTGAGTTTTGGCATGATCGTTtCATCAGCTTTAATGATATGGAAAGGCTTAATGGTTATTACTGGGAGTGAAaGCCCAATCGTTGTGGTTTTAAGCGGTAGTATGGAGCCAGCATTTCATCGTGGAGATCTTCTATTTTtAaCCAATTATG

>contig00766

ACAATTCCCTTTGaGATCATTGTGTCAATAGGTGGATTTTTCTCGCGACTTATCATTTTTCTGCAGGTCTGTGTTGCcTGGAGGACAATTGTTTCATCTGGGGAACTCATTCCTGAAACTACTTCATCCACAGTCATATTAAGAGGTGGAGATTTCAATTCACCCTGTGGACTGAGTGCTTCACATTCATCTTCATTAAGATTACGGCGCTTTAACATCTGATCATCCTTCCGTGCTTTACGCAACTCTACGCTTATCTCGTTTCTTCTTCTGCGAGCCTCATCATGTTTGTTGTTGAACTTAAACTTtGCCATTCGCCCTGACGTGTCTATTTCTCGAGCCGGCATTATGACTTTTGGAAAAATATAAAAaGTAATCAAATTAAATGAAAAACAGTATCAATCGCGACCCGCGCGATAAACGTGAAAATGGCCGACTGTAGATCTTGCTATTCGTGCTATTCGCAAGGATCAGTAaCGATC

>contig00767

CTATATTTATCTATATCGGCA

>contig00768

AGATCAGCGTTAACATAACGATAGAGAAATTTGATATCTCGGGGTAGTGTTCTGATGATGATGTAAAGTTTTCGATGAAACCTGTAGATAAGGGGGATGAGTGCGGCTGCCAGGGCAATCTGTGCAATCCTGGCAAAGGATCCAATCCTGGAAGTGACGCCGGCCGCCAtCAGGCCGACGATCGCAAGT

>contig00769

ACGCAAGACTTTGGGATGAGGTTGCTCTTAGAGAATTCATCAAGTCCTGGAAaCAAAAAACTTTTCATAAAGCTAAAAAGTaTCTTCTTGGAAGTATAGGTGTCTCAGCTTATAATTGGGATAAGGAGCGAATATCTGATAAAGAGATGCAGAGCTACAGTCAAGAAaTAGATGAAATTTATAAATTACGTGATTCTACGGTCAAGTGTtCACAATGTCATCAGCGTCTTGTCATAGATGTCACCCAGATAGGAATAGATTACTGCTATTGTCAAGGAAACAGCCCAACTGTAGCTGTTTTCAAAAAaGATGAATCTtGGGAACCATTTATTGAAAGGGAAGACATGATAATATGGCGTAGACTAGAACCTGATACCGGACTATATGCTTACAAAGTGTATGGATCC

>contig00770

ACCTGATTTTTTAATTGTTTTAGAGCATCGTATTCTTCAATGATATCCTTTTTTGCCATATAGTTCAAATAGTCCAGAAGAGCGACAAAGGTCAAATCAGCCCAAGAAAGAGCACTACCGACGAAATAACCACCGTTCTTTTTCACCTGCTCGTTTAGACGTTTCAAAAAATaCGGGATACGTtGATTCATCTCCTGTAGTTTCtGTTCTTTGGCTTCAGGATGATTTTCATAATTGTAGGCAGAAATTTTTGAACGAAGATCGTGAATGGTATCGACTGTAGCATCAATTTCATATGCTTCGAAATCATTTTTTCCAGCAAGACCGCACTGCTTGGCAAAATAACGACAGATTGCAGAGGACTGATGCATTTGCTTTCCATCAACTTCGAGAACGGGAACTTGGCCATAGGGCATTTTGTCTTTAATATTTGGCCAGTTGTCTCTATCAAAACGGTCATCTTCAAATTCAATGTCAGCGTAACTCAGGACGAAACGAATAGGTTCAGCCAAAGCCTTGACAGGGAAGTAAGTCAGTTTGTAGTTAGGCATTTTTCCTTTCTGTTGATCTATTGGCACAAATAAATTATAGACTGCACTTTGCTGTCTGACGAAAATATTTGAGAATATGTATAAAAACACAAATTATCAAATTTACTACTGATTGCTACAGATCTGCTGATCACTaCcAATCACT

>contig00771

AGTTGCTTTGAATGGATTGGAAAATATCCTGAGATTAGGGGAACAAGACGCTACAGTTCACAATGGAGTGAATCCTTACGCAGTTCTTATCGAAGAATGTTATGGCCTAGACAAGATAGAATTTCTCCAGTCCCATCAGAATTTGGACATCTATCAAAAAGCTTTCGACATCATCGAACGTTACTTTGGTtCTGAAGAAGAGGACACCAGAATCGCTCCTTCAGTTGATGCCCAGAGCCAACAATTttCAATtCACAACTCCGgATtCTtCTCAAATTCCGATGCAAGGCTTCCAGTTTtAATTCGCCAATCCTGATATTC

>contig00772

ACTTCGTCATCTGCTACGGGCTGCTCTTCTGATGTAGCGTCTTGACCTGTGTCGATAGTTGGTTTCTCCACTGTTTCAAAACTATTGCTATCTTCGTtGAGGGgAATTACTTCTCCATTTGATAAAGTGCTAATtaGGTTTAACATACCTTCGCGATCCcTAACAaCAAGTTCCCAGTTTTCTTCATCCAAATCTTCACGATAAACTCTAATGTTaCATTCCTCATCGAGTTGACACCAATACGACAATCCAGTTTTGTCTTTTCCCAAGGGTTCTAATCGCAGCTCATTAGCTGTTAATTTATTCACTTCATTTTTGAATTTTTGATTGAAGTCGAACTGTGCCTCCAGAAGAACCTTAAGTATTTGTAGCTTCACACCAATACGTGCTTTCTTGTAGCCAAAACGTTCAAGTTCCCAACCATCCTGATTCGAAtAGGTATGGC

>contig00773

ACtAAAGGCtGGGTtGAtACAATTTTATACGGCAAGGCTGGTAAAAATAAAGGCGTAGAAGAGGTCAAAAaTGGGGACGAAGAAGAAGACAAAAaGGAGGTCATAGATGAGGAAAATGAAACTGAAGATAATAATAATGGTGATGCTGATGCTGATGGTGATGTTGAATAGATAGTGGGAAAACTTAAAaTTTACCAAATTAAAAaTAAATAATTGTTATATTATTTTTAATAATTTTGTTTAAATAAaGAAAAaTCATTTCATTCAAAAAAaTTTTTACAAACATAAATTGATCATTTGAAAAATTTTATTTACTACCAAATTTATTTTTATAAAAaTAAAAaTAAATGTTTTAAAAATGATGGGGTTTTATAAACGAGAACTTTTTCTTCTCGAGAAGAAGATTTTTCACAAAACTATTTCCAATTTTGGAAAATTAATTtCTCATTGCCTTGAATTATAAGAAAAAAaTTTTCTGCAAATAAAAAaTTCAAATCAACAAAATTACTTGACATTAGTTTAATTGGATTCAAAAaTTGAACGCGGTTAATTGGACGCAATCTTCATTCCCAGAAAATAAGTTCTCATCCATTGAAACCTCATTAACCTTGGGAAATCGGTGTTTTTAAGATTATCTAAATAAATGATTTCTGTATGTTAAAATTTGTTTATAAaTaGTCTTTtCTCCCCTAaTGaTaTTaTCAatAAtagT

>contig00774

ACTCTTCTcTGCAGGCTtGTtAACAACATtAAACCtGACtCCGTTAAAAAaaTCAACCAAACTACCTTGGCCTTCAAATGTATGGAGAATATAAATgcATTTCTGGAAACTGCCAAaGCTCTTGGAGTTCCAGCCCAAGAAACTTTCCAGACCGTTGATCTTTGGGAGAGACAAAACCTGAACTCTGTTGTCATATGCTTGCAGTCTCTTGGTaGAAAGGCTGGAAACtATGGAAAGCCGAGCATCGGACCAAAaGAaGCTGACAAAAaTGTtCGTAACTtCACGGAAGAACAATTGAGGGCAGGACAaGGAGTGATCAGTCTTCAATATGGTAGTAATAAGGGTGCGAATCAAAGTGGAATCAACTTTGGAAATACTCGACACATGTAAAGCGATAAATTCCTTTAATTTTTtAAGTTATACAATTTTCCCCTACTCTTTCTTTCAACATAATCGTAATCAGAGATATACTATAACAGTGTTGTTTAACTTCACTGAAAATCAGATGGAAAATGGTAGAGAAAACACTTAGAGTCTATAAAAGCATTGTTGAATATTCAATCATAAATTAATAAGCCAGATATAAATCTAACTCACATAAAACTTAATGGACTCGAACCACGGTATTAACTAGACTTTCAGTTCGTTTGTTATTGAAAAAAaTTTATTAAATGTTGTATGGGACACATCTTATTTAAGAGTAGaTaGT

>contig00775

ACAgTAATATTCGGGTTACCGGCAGGATTTTtATCCTTGATTTGCTACGGCATTTGCTGTCCCGACATTTTAGATGCTGATGACGAGGAAGAAGAAAACGAAGAAATAAATCATATAAAGAAAGATTAAATTACCCAATAAGGAAAAACGTTATAaTGTAGAAAGACTGTTCACAGCCACGGTTTAATTATACGTGTTGTTCGATTCTGGACTTAAGTATAATGCTTCGCAATCGTAAAC

>contig00776

ACTGCGAGTATTTGACTGGATCTTTTTCAAAGACTTCgTAAGTTCCAGATTCAAGATTATCCATTAaGGGCTGCAGCGGACATTGTAAGAAATCCtCGTAaCCTCGaGCAtAGCGTTCAATCGGACCGTCGTTCTGCCAAGATTTCCAGAtATAATCAAGATAATGATAATAGAAACTGATaTTtCGATAGtCGATTAGCACCAGTAATAATGAACTGCACATTCAGAGAGCAAAaT

>contig00777

CACCTTACAGGCTCACCTATCCACCTGGTTATCTCTTCTTCGTCAGGAAGATCATGGC

>contig00778

AAAtGGAATTAGAAGCATTtGCAGCTCGGATAAGTGACCtCTtACTTTtGCGAAGCTACA

>contig00779

ATTCAATTCATAAGAAATTGTTGAGATATTAT

>contig00780

GTCTCATCCTCGTCATTAAGTTTAaTATTCTaGAGAAGTA

>contig00781

TtAAATTTTTtAAATTACTaTAAAAaTATTCCAAGGGCTTTCAAAAAaTCAGAATCAAATTTTtCATCAGTTTtCAGTATCAA

>contig00782

AGTCGAAGAAATTACAAAGTGACATTTTACAACGATGCAACTCTTGCCAACGATACTATGCTACCATGGCTCTGGGTGTCGATGTCTGGAATCGACAAAAAGTCGAAAACTATGCTGCAAACAAATTTTCACAGACTA

>contig00783

ACGtAACCAGATTGAAGAAAaGAAAAGGATGGTAAAGGCTCTGCAGGCCGGAGTATCGCCTGAAGGGCAGAAACTTTTCATAGCAATTTCAAAAACCATTGATGATATTACGTGGAATGGACAGAACATCGTCGTTTTCAACACCGTAACCATCATACCGCCATATAAAGTCGACAACGTTCATAGCAAAGTTGACGATCAAGCTTACATTCACGTTAAAAAaGTAGTGGAAAAACATATGAAGGACACGGCAACGTCATC

>contig00784

ACAACCAATtCATCCTTTTtGTAATGGGCGTGCAGTTACAaTTCAAGGAAGTCCAGTCTTACATCTAAAaGAAGACCTTGCATTCTTCACGGCTCAAGATACGACGTCTAACACGCCATTtCTGACTGTtGTAATGAATGGATTTACAAATAATTATCCACTTCAATACTTTGGGTTTGAATGGGTAAACACAGGAGTTGCTGACAAAACAGTCATGTTAGATGTCAGGGTTCAAGATCTTAGCGCCTGCAATATTCATGAGACTGGAACAGTCATTCATCAGTGTGC

>contig00785

ACGATGATTCGTCGACGTCCATCGACGGTAAGTCCAGCAGGTCTATCGAACTGTCCAGGACCAGT

>contig00786

tttAGGTAATCGGGAGATTGAGGTGgTCAGTGGTGGAATCAACAAAaGCAAATCCAACAATtATCGTCTCGAGAGTATGGCGCTTCCAAGTAGAGCAAGGGTTTATACAGACGTTAATTCACACAAATCCAGAGATTACTGGGATTATGAGTCATATGTCGTTGACTGGGGACAGCAGGATGACTATCAGTTAGTAAGGAAGCTGGGACGCGGAAAGTATAGTGAAGTCTTTGAGGCTATTAATATCACAAATAATGAAAAaTGTGTTGTCAAAATATTAAAGCCGGTTAAAAAGAAGAAGATCAAGAGAGAGATTAAAATCCTAGAAAaTCTTCGAGGTGGGACCAACATTATTACGCTGCAAGCAGTTGTTAAAGATCCAGTTTCAAGGACACCAGCATTGATATTTGAACATGTCAACAACACAGATTTTAAGCAGCTGTATCAGACGCTGACAGACTACGACATAAGATATTACCTCTACGAATTATtAAAAGCATTGGACTATTGCCACAGTAtGGGCATAATGcATaGGGACGTAAAGCCGCACAaCGTTATGATCGATCATGAAAaTCGAAAACTGCGGTtAATCGATTGGGGTCTGGCTGAATTTtACCATCCAGGACAAGAGT

>contig00787

aCTTGTTTTgCTCATGCACCTACACTTGAAGATATTTCTTTGTCAACATTATgTCAAAGCATtATCTTTAAGTAAATGTGTAAACACAGCTGCGGATTGAGTATCAAACTCAAGATGATCTAATCCCTATATAaTTtCTTCACTCCTCCAAAAAAAAaGCCTTTttCAAGAAAAaTATATTCCATATAAGAAaTtGAGTtAAAAAAaCCGTGTtAATAATAAaTTGATTTACAaTtATTCATTTTTTtCGATTAATTCCGATACGAAAaTTTCTCGAATGCGTCAAGTGTTGCAGGTAAAAAAAaGATCTATTGTGCATGCGCAATGGATAACACTAAACAGCGATCCATTCCGAATGCGTGTTCGACTTCCAAAAATCACTAAACAGATTTCCAGTCAATTGAGTCGAGCAAGTTAGAGTATTTTTCAAGGATGGAGTTTCTAAAATATTTTtCAAGGGTATTTTTTGGCGACCATCTTAGAGTTTCACACATGAGGATTAGAGTCGGAGAGTAGCGGGCTTGCGCTACTACACCCAATAATTCTATTCAAACGTTGAAAACCCATTACTATTTACGAGAAaCGAATtATTATCTCCGATCCATCTGtAGTTTTTTTttCTTTTATTtAACTACCTCCTtCGAAAaaTATT

>contig00788

ATATACCTAAAAACCTTTTTGCTGTCATACAACCTTATAGTTAAACATGGTGCGTATGGAACAAGAAATGTCCTCGAATACTATTCTAGGAATAGTATGGCAGTTAATAGCATCAGTTGGCTGGTATTTAGTTGGAATTGCAATCGCGTTATATTTTGCAGCATCATCTTTGCAACAGAAATACAGAGAACGAAAATTAAAAAaGGACGAAGAGGAATATGCtGCAAAGTATCACAAAGATCCTGATATTGTCCATGAAAGATTATTTGCGGTTGAAGCAGCACGACAGCGTATGCAAGAGAAGTACAATAAAGATACCGAATTAGCTACAAAAAAaGAAGAAGAGTTAAGGC

>contig00789

TtgTtAtATTGTTTCAGAATGGTTTACTtACAAGTGAAATTAGTAAAAGATTTCATAAAAaTTaCAGTGTTCACTTAATAATCAAAT

>contig00790

TCAACTTTCCATCTTTATGAATTATATTCATCCCTTTCTTCAATCACAGAATCTAGATTACAGGATATTTGTCATAGAGCAAAGTCCCGTGCGAGACTtcAATAGAGCAAAATTaTTTAaCGTCGGATTTACTGAGGCCACAAAAGTCAATGATTTTCACTGCTTTATCTTTCAGGACATTGATCTCATACCACAAAATCCAGACAATATTTATG

>contig00791

ACAACAGCTGGACCCTTATCGAATCAAGAAGTGAAAAACGTGAAACGTCGTGAGTGTAAAGTTGTTTTAAATATGTCGCTTTAGAAGAGGCTTATTTTATTTTTCACGTTACACTGTCCAATAATGATTTCACTGGCAAGCTTCCTCTTTATCGGTTTCTTCGCCAATGCCGTTTTCACCTA

>contig00792

AACAGAGGCCGACCATTGCTCCAGATCCAATAGCGATTACCTCCGGGACTCCATTGATAGTTCTTGCCGTGACTTTATCTGTTTGCTTG

>contig00793

TCCTCGTCAAGAGGAATGGAGAGTGTGCGATTGCTGATTTTGGGCTCGC

>contig00794

TCCTCCTTTACTCACCACAGTAGTGTTGAAATGACAGCTGCAGGTCCAACATTACCTCCGAAGAAACCACCACCACCCCCTCGATTGAGGTCTTCCATTTCTGGTGACTCGCTCAGAGAGACACTCGGAAAAGAAATGCCTAACTTCAAAGGAAACTTGCAGAATCTGTCGCTCGATGAGATTAGACGAATGAGCGACGAGGACTTGAGAGATATTGGGTTAACACCGAATGCAGTTGGACAATTAAGGAACATTATTAAAAGT

>contig00795

AAGAGCTAAAaGACTaCCCAaCTTAAGGATGCAAaCTGAAGGGGATCAAAGCGAGATTCCGAGACAAGAGAGAGTTGATGGAGAGGAAGATTTCCCTTTGGAAAaaGAAGAGCATTCATCTCAAaGAGAGGTTCCTTCTCAAGAAAATTGGGAaTCTGATGGTGGTTATACCCCcTGCAAAGATgaGTTACCCcTGAGGgAAAGACGAAGGAGAACAGTTtCGCCATTtGATGAAAGTGGATTGGAGCCAATCACTCCTACGAGAGACAAAAATGATGATTtGGATCGTTGCAGAACCCCAGCAGCATATGCTGGCCTAGGCACTGAGGCTATTTCTG

>contig00796

TTGTTTGTAAGTAGGTtAGAAACTGAATCaTTGCAAaGTTTTCATGTTGTAAaGCtAAGTTATAAAACAGATAGTGAAGTTCTTGAAAAtCAAAaTTCTTCCTTCATCTTCATGTCTAATGGTAATGTTAGTTTTTGTATTCATTCACTTTAAGATAAATCGTCACATTCGATAGATGTTTAGGGATATTAAAGAATTAGTTTtGGAAAATAGAGATGAGATTTTGTCTAaGAAAAaCAAAACTTGGATAGAATTTGTAAGAATTCTGACTTTTAAGGACTTTTTCTTCTGAGGCATAATATGAATGTGTGCATTTTTCCAACGACATGATAACGCCGATTTTTGGACCTGATCTTGAGAAGATGTTTGCGCTTGTAAACCTAGTATTTTTCATTATATAATTTtGAATTtGGGTAATATTGCTGCAGGaCCCCTAAATTGACAAAACAGCAATTTTCATTCCTTTTTTtATTATTTAAaGCCAAATAaGAATTGCACTCAATTtCTACTTAAATTAACAAATATTAATGTCTATTCGGATGTAaTACTACAGAACTATAATTAGATTCTAGATATTCAAATGTAAACGATTATTCCAAATTGTCGATATTCCTTTAAGAAAAATTTCTGAAGAGTCATTAAATTGT

>contig00797

ACTATATAGCAAGATCAAATGAATGCTTtAGAGTTAGCTGTTATGCGGATGGCAGTCAAAATGTTTTtGGATGCGAATCAGTAAGTGGACGATGTCCAGACAATTACCTGACAGTAGTCGCGCAAGGAAACGCTTCTTATAGTTATCCATACTGTTGTGATCAGCTATTTGGTTGTTATCCAATCTCGTATATAAGATACACATATTATTGATTTTATATTTAAGATTCCATATCAAATGTCGCTTTCCGTAAAGAAAAGGCTCTAGTGCGGAAAAAATCGATTTTCAGATTTTCAG

>contig00798

AGTTCGATTGGGTCGTCAATGATCGTCAGGTATAAAACCGAAAaCCCAAaTATAACAGAACGATTTCAAAAAaGGAACTGTTTGGACGCGAGACGCGACGCTCAGTGTAAACGTAATTTATTTAGAACTGGAGTATTTATTTTTCGTTTTAATCTTATTTTTGAGTGATAATGGCTACTGACGACTCCGAACCAGAACAGACTGATCTCACAATTGTAGAAGAAAGCTTGATGAGTTTCGAAAAACTAGACCGAGCATCGCCAGATCTCTGGCCAGAGCAAATtCcAGGTGTAAACGAGTTTGTTGCTCAAAACTCTCCGCAGACAGAACAACCATATTGGACTTCAGCTCTCACAGCTAGTGAAATTAATCAGTTACACCAACTGGGAAATCTCACAATGGATTTACTTATCAAAGAAGTAAAGAAACTTCATGATCTTGCTTATGAACTTGGAGTGGAAGAGGCAaGAGAAATGACTCGTGGGAAATA

>contig00799

ACCACCATTTTtGCGACTTCTTCAATCTGTATGCTTCGCTGCATGTAAATCTCACACATCCAACAGGTTTTAGTAAAGATAACCACATTATGATATGGGAAAGTTACAGTTATCGTTCAATATTCCAAGACACTTTTGAAGCATTTACATCCAATTCTATATGGGATTTAAAAACaTTCAGaGGgAAGACAGTTTGCTTCAAAAaTTTAGTCTTTCCCTTGTTACCGAGAATGATCTTtGGATTGTaTTaTAATACACCACTTATTTATGGTTGTGAAAaGAGTGGTCTTTTTAAAGCTTTtGGTGATCATGtCTTGCATCATTtAGGCATcACCcTACAtGAGAGAAAAGACCCAAAATTGCGCGTtACTCTACTTAGCAGAGACACTCAATACAGGAAGATTCTAAATGAAGATGAATTGGTG

>contig00800

ACCTTtCGTGGAAATCCAATAACTCTTCAACTAGACCCGCTTCCAACATTGAATCTACTCTCGCGTCCAGCCTCTTGTCAA

>contig00801

TTcTtGGGAAAaTGAATTTCTAGTGCGGTTtGGAGTAAGAGAATTGAGATTCTTCGAAGGAGAGTGAGTAGATTTTGGAGATGGTGGGAATATTATTCGTCTAGGAGAAGAGCGAGCGGCTCGTGTGACGTGTTTtAGAGAGCTGAaTGGAGaGAGGCGCTTATCgAAATAACCAGAAGCAGGTTTTAGACGATCCCAGGTGAGATTCATGAGAGGAATCTGAGGTGAGCCCTCTGGTGAGGCTAAAGGAGAGACGAATTGTCGTCCAGGTGAACTGTAGGTGCTACTCGGGCTATTATCGGTGAGAGCAAGGGGTGACATTGGTCCCAGATCAGATTCATCGCCTGAATATGAATCATTCTCTCGCTGAAGGACTGGTTGTTGAAAGAGACTGCGTTTAGTCGAACTCTCTTCAGCTCCGAAAAGTCGCTTTTGGGCTCTGCGAGGAGTGCACAGCAACTCCTCTGAGCCTTGACCACTGTCGCAAGAAAGCATTGATAGACTTTATGGCTCAGAGACAGTGTTTTATTCTGCTTGCACACAAATAAGTAGAAGTTTATTTTCACTTAAAAACTACGAATTAAACGCGTTTTAACTGATATTTGTCTCTTCAACTCGACGCAGTTACAAACCGTCACAAACGTTGTTTTACCTCCGAACCACTCCaaCCT

>contig00802

GTCAACATTAGCAaTGCCTATGAAG

>contig00803

ACTTACAAtGTATATATTTACAAAGGACAAAGGAaTAGGAGATTTAATAATAAGTCAGACCAGAAGTGGTGCAGTTTGCGTCAACGATACAATAATGCAGTATGGTGTTGATGCACTtCCGTTTGGAGGTGTTGGTGGATCTGGCATGGGAGCCTATCATGGAAAGTTAACATTTGATACATTCATACACAaGAAAGGCTGCCTtCTCAAAACCTTTAaCAAACTTGGAGAACGTTTAGGATCAGCTCGATATCCACCCTACACAGA

>contig00804

ACtCAAATTCTGTGAATTAAGTCCTATACCCATTGAATCCCTAGATTCCGATCCCTGTCCTGAGAAATCAGACGTCGAGGGGACAAACCCTATCCcGGAAGGTTCACAGGTGCCAATAGTTGTATGCTCTGTTTTCTCCGGGGACAAGGAGCCTCCGTTCGGAGCaGGCCCTGTTTTAGAAATAGTAGATGTAACATCGTCTAATAACCTCGTTTCGTTATCCATCGTCTGCaCAGACATCCCCACCGTTGAAGCCGCACAGTCCTTGACCCAAGAATCCATCTCGACCTCGTGTGAAACACCACCTGGTGCATCGTCCTCAACCGATCCATTTACCATTCCATCtCCGTTTtCGTtCCTTCTGAAAGTTGAGCCcGGTtCCTtCGTtGATACGACCAAAaTGTTTTTCTCGATAGCACGCATAAATTTATCGACTCGATTGTATTCTTtACGTGGCGCAGTTAAGAGTTCGCAAATTCGTTGGACAGTAAATGGTGCATTTGCAAAAGACTCCAGTCGCTCTAAGAGAT

>contig00805

ACAGGCAGTGCTTgCTTTAGCTGCTTCATGGACTGCAAAAAaaCTTGGAGGACAGAAGTCTAACGGGCATCGTCGTCGACAGT

>contig00806

CATTCAAAGTCTtCTAAGAGAGCGCGAAGTaGGAATTCCACCAGAACAATCGTTAGAGACGGCAAAGACGATCAAAGAAAAATATtGTTACATTTGTCCTGACATTGCGAAAGAGTTTGCAAAATTTGACTCTGACCCTAGTAAATTTCAGAAGTTTGAaGgCGTGAaCAATATCACGAAGCAACCTTTCACAGTCGACGTCGGTTATGAAAGATTTCTCGGACCAGAAAT

>contig00807

AAAagAGGTGCGGAAACTGAAGTTGAtAAATTtGTtCTtATTAGTCACGTGACCAGTCTGGCACAAGGCTTTACGGGGATGTGATGCTTGAGAAATTAGTGAATTCGTGAGGGCAGACGGATCCAGTTTCTAAATGCTTATGTTCAAAaTAATTAAAATTATCAAATAAAAATGTAAGTAAAGCTAAGGCCCATGCATACCAAAAaTAGGCTATCTACATCCATTTTAATTTTTTTACtATTTTCCAAAaTTTTAAaCATAACAATTTTTtCATTTTGTTCATAAAAATATAAGAATCTAAAATCAATAAATTTTGCTAATTTCATAAGCATCACATACGCTTAAGGTAATATACGTATCCATCCCTGCCACTCAGGGAAAAGATCTGGACTGAAAACTGATTCTGATTAAATATGTAATTTTTAGTTTCCTTGGTTTGAATGGTTATAATTAAGTATAAATAAATTAGAGATATGAATAGGATAATTGGCATCATATTtACGTAAAAGATATACTAGTTTTGCATTCAATAATCAGATATGAATGTATACGAAAGAtAGCGTAaGGCAGGATAAATAaTTGTTAGT

>contig00808

ACTTACTGTTTATtATtCACGTTAATAaCACACCAGCTATACTCCAAA

>contig00809

GCGAGGGGAGACGTAGAAATTACAGAAGCTGATCCTCAAGGACGTTATGTTAAGTTAACGAACAAAGGAAACAAGGAAATTGTTTTAAGTGGCTGGCAAATCATTCGCAAAGCTGGAGCTTTAGAAACATCCTTTAAGTTCCACAGGACCGCAAAAGTCGATGCTGGCGCTACTGTAATGGTTtGGTCGTCAGACATTGGAGCCACTCATGAACCTCCATCTAACATTGTCATGAAAGGCCAAAAaTGGTTCACCGCAGACAACATGACGACTTTGCTATTAAATAACGACGGTGAAGAAGTGGCCACGTCTGAaCGTAAAAGGCAGCATTtGTCTACCAGCTTGTCTCGGCATAGAGAAAACCTGGGGTTTAGACCATCAGAGGAACTTCACCACCAACAGGgTGATCCaCAAGGTGAAGATCGTTGCCGCATAATGTGAAGATAATTTTTAGAAAAAaTAAGAGATATATCAGATTTTTTTAAAAG

>contig00810

GCAAGAGTCGTTTAAATATCaTT

>contig00811

CAGAATTGATTGATCCTCTCGATCTTGAAGCATAGAACGGTAAGCAGTGTCCGTGATGGCGAAAACGTGTGGAGGAACCTCGTGCCTTTTGATTCCCTTATACCTTTCCATAATTTTTTCTGTGTAAATTGGAAGTCTCTTGTATGGATTGACAACGACACAAAAGAGTCCGGAGTAGGTGTAAATTAGTCCGGAGTAGTAACGGTCCTTGAGGTTATGTAGAACGGAGGCCTCGTTGAGGCAGGTGAGTTCGGCCATGTCCTCGACCTTGTCAAaCTTtGGTGGATTCATCTTCTGGATATCATCCTTGGGCACGAGAACTCTCTTACCGGTTTCTGCAATCTCTACTTCGACTTCGTCCCCGCGCTCGCCTTTGATTCCAGCCGCGACAAACCCCTGGGATTCGTGCGGCACCCATACCAGCTTCTTCTGGGTCCATTCTGCCTGaGTGGCCGGGTCGTTGAAaTTGTTCCTGTCCACaGAGAGGATACGCAGCTCCGGGTCAGAGCGATCTACCCTAGAGTCCTCCGCCATC

>contig00812

GCTCCTCGCAGCCGGGCCAGAAACTCTACTTCACCACAGCACGTTTTTTCCGTTCTTCGAACAGTCCTTTTCGCGAACAAGTTAA

>contig00813

CTTCCTTGGAGTCTTCATCTTGTTGTGGTATTTCGTCTTGACCCCGAATTCTAATGTTGTGCTTCTCCTTATATTCATTAATCTCGATTCCTTTCCTCGACAGCTGCTCGGTTAAGGTTTCTATAACTTTTGATAGCTGATTTTTATTCGAAACTAAAGCTGGCATCACTTCTCCAACTGTTCTTTCACAAAGAATACCTCCAATCATTCTATAACACTTTTTCTTTGGATCCACATTCTTTAAAGTGTCTATAACAATCTTGTGCTCATTCAGCTCCATTTCCATTTCGGACAATTTATTAGCCATAAGTCTTTGTTCATTTCGAAGAGTTTGAAATCCTGCAAATATTTCTTCATTGGATAATCCACCTTTAGAAGAGGATTTACCTAATTTCTTTTCACTAGCCATTTCTTTGGCCGAATCTCCGTATGT

>contig00814

GTAGCCTCCATCTGCcGTTAAGCATTGGgTGAAGGAATGCTCGTCAGATTCGTTCAAACACTCACACTGTGCCTTTGTTaTAAAGGTGATTAAGTCCATATGTCCAGCAaCGGATCCCTcGGAGTCATCGCcATCGCCACTTCCAT

>contig00815

GGCTAGAATTCTCTCTCCTACGGCGAAGtCCTTTGGTTTCATTTCTCGCGATTGAATTTGTAACATTCGTAGCCTGGGGCTGTCCATAGGAATCCACTCGTCAAATCTAGAACTCCATTTGTCGAAGTGAATCAAGACTTCCCTTTCAACCCaGTCAGTCTCAaCTaCTCGTGCAGAATACCATTTCTCGTTAAAaTCCTTTGCTTCAAGTTTATCTCCAGGAAAAAAGTTTAAATCATTTCGATTTACGGCTTtAGTTTTGAGCTCATCTACTTTCTCAATTTTATTTTCACTGTGTAGTCTGATGTTATCGACTTCTTGGTGAAAATCCGTAGCAGGATTTTCTACTGTCATGGGAAGTTCATCAACTTCTGTAGCACTATTAACCGTAACTGGT

>contig00816

ACTtCCATTTCCACCGATATTACCATTTCGATGAACTCCACCGGAAACCTTTCTCTCTCTTTTGAGCAAATGTGCTGGGACATACGTATGCAAATCCTTCCTTTTCACATGCTTAGCTTCAATGAACATTCCTTCCTTCAATAGATTGTTTTGCTCTGCTTGTCTGTAAATCGTATCAACAAAGTTTTTAATGTCATAAGTAAGATCTACATTCAAGTTTTCGCTTCGCGCAAATAaCAGTCCGATAAACCACATAGAGCAGTGCTTTTCTGCTACGTGTTCAAGAGGTGAATACGCTTCTGGGTTGATGTGTGCCAAGGTGATGTGTGGATTCCTCTCTAACTGACCAATGAGGTGCCGTATTTTCGACTCCACAAGCCCACACCATTCAAGTTGATCCTCGGCCTGCAAACTACTAGCAaGTAATACAACATAATGTTTATATTTACTGAAGAAGTTCGGAGGTTC

>contig00817

ACCTTAATTGATCTAAAAAGCTTCAAGTTTATTATGAACCATGACATGTGTAATAAGTCACAACCGCTTCTACTAATAATGATACATTCCGCCCCAGGAAATTTGAAAAAAaGGAACGTTATTCGCGAAACTTGGGGTCAAGAAAGCTCATTAGTTACCACCTTGTTTCTCGTTGGCTGGTCCGAGGAGTATCAAGTGGAACTAGAAGAAGAGAACATGAAATATAGAGACCTAATTCAAGGAAaTTTTTtAGATGTCTATAGAAACATGACTTATAAGCACGTCATGGCTTTGAAGTGGGCCACCTATCATTGTGCTAGTGCCAAATATATATTGAAATTAGATGATGACGTATTCGTGAATATCCCAGCTGTGTTGGATTTCTTGAAGCATAATTTATCACCTTGGGGAGCTAGACGTCTCATACTCTGTGATCCTCTTtCGGCAAAAGTGAAAAGATCCTACCGTTCGAAGTGGAGAGTATCCCCGCGAGAGTATCCTGGAAGACATTATCCAATTTATTGTGCTGGATGGGCCATCTTGTATTCTCCAGATACTGCGTTCTTACTATATCGTGAAGCTCAAAAAGAACCTTATTTTTGGATTGATGATGTGCATATTACTGGAACTATAGCTAGGAAAATTAATTTAACTCAAGTGTCACTACACTCCTTAGTGCTTACGAAAAAGGAGAAAAAGT

>contig00818

TACAAGCGATCAGTTTTTAAGAGATAACTTAGAATGGTTAGCTCGAGCTACTAATTGGGCAAAATTGACTGCGACCGCTTCTTTAGGAGTAATTCACAGAGGTCATGAGCAAGAAGCTTTAGCGTTAATGCAGTCTTACTTACCACGTGATACTGGTGCTGGAGCTGGTTATTCAGAAGGAGGTGGTTTATATGCACTTGGTCTTATCCATGCCAATCATGGCGCTGCTATCACAGATTACCTCCTTGGCCAATTAAAaGATGCACAAAaTGAGATGGTTCGTCATGGAGGTTGCCTaGGCTTAGGGTTAGCTGCAATGGGCTCTCATAGGCAAGATGTATATGAGCAATTGAAGTtCAATCTTTATCAAGACGATGCGGTGACTGGTGAAGCAGCAGGTATCGCTATGGGGATGGTAaTGTTAGGATCTAAGTCAACTCAAGCTA

>contig00819

AATTtATTATtATATTTTTCTATTTCGGTGTGGAGAAAAGAAATTTtGGATTCAACGACCTACAAGCGAGAGCAAaCCTCGAAAAAaTTTTTTGTTTATATTATATTTTAAATTTTACaTCTCTAAGGCGAAAAaaTTTCATAAgCTTGATACCTGTTTGCCGATAACAAAGACTTATTTCTAACAAAATGTTATATGTCTTATTTGAGATTACTCTTAAATAATAGTTTTATAGTTTTTTAAATATTGCGTTCTCAACTGATACTTGAATTTTtGTTTGTCAAATAGTTTtAAGTtGATCATATATgAGTAGATAAGAAAGtAATTACGAAAAaCTTATCTAGAAAGGAAATTTGTCTGGATTTGAATCTTAGCTCACGTAAAAGTTTATTCTTTCGCTTTACAGAAAGATGATAATTGCCTTGCGCCAT

>contig00820

TTACTGTAGAaCCAGACGAAGATTGGAAATCGGTAGAAATACCAGACTCTACATCTG

>contig00821

TTggAAACACTGTCATTCATCCTATCAAATAaGATTCAAAAAAGACGGGCAAAACTCAAACATGTATAAACTTAAATGTGACTTGAAGCAGAaCAAATATAAATGAATTCTAACAAAGCGTTTTGGAATATGGATTGTTAAACaCCGaTTCGCTCATGATTTTtCAAaGGAaGAAaTGAATCTTGAGTTATTGGACTTCTTCGTCAATCACTTTATTAGTATTTCTATTTATCAAAATGTTCGTATTGTTTCTTAAGCATCAAAaTTCCCCACAAAaCTTAATtACACGAGCTCTGTGAtGATGAGAAATGCGGCATTTtCACGAGTTTtCTTTtCGAATATTAaTAAAaCGAaGTCTAGATTGCGCTtCCGCaCATAGTTAATATCAAaTACGTTCGCTTCGTtCCACCAATAATTAtAATTCTTCAAATaGGTCGTAAaTTCaTGACTAaTTCAAcTGTCAAAAaGCCGTCACTTGGGTTACATTTCTGTCAaGAGATTTtACTTTCCCTACTTGTTTATCTGTTGTTTTTTtAtGTATGCGATGtGCTGTGTCTGCCTTtATGTGCATACACGCAACAACACCTGCGCGGAATCGGCGTTGCTATACCGAAGGAATTAGATTTTGGAAAGAGGGAAAGTAATAAGTTACTGAAAAAaTTGAGTGTTTGAACTGCAATTAAGTATGTTCAGTCgA

>contig00822

GAACTGAATGTTTTAGTGAGTTGAAGTATTTTTGAGTTTCTATCAAAGAGTAAAAATGGGTGACGATTTAATTCGTGTGGCTGTTCGAATAAGGCCACTTGTCCCTTCAGAACTAGAGAAAGGATGTCAAGAATGTTTAAACGTAGTTCCAGGAGAACAGCAAATACAAATAAAACCCACAGACAAAGCATTCACTTTTAATTATGTTTTTGATACCGACATTGGACAAGCAGAATTTTACAATACTGCAATTAAAAAAaTGATCACACACATATTTCAAGGTTACAACGTAACTATTCTCGCTTATGGCCAGACTGGAAGCGGCAAGACCCATTCTATGGGCACTAACTATTCCGAAAACaGTGAAAGCGATGGCGTTATACC

>contig00823

ACCAGAGCCTTtACATTTCCCATTGTTGCTGCAAaCGTTGGTCGGAAATCCTGGACaTTTttCACATTCAGGTCCGAaGTGATATTGCGGACAACAACTTTttACTTCTTCTATACAAACGTAAGTGTAGAGATCAGGATGAGTCTTCTGTTTTttGAACCACCAGTCTTCAAAaaCaCCTTCTAGTTCCTCAGCTAACCCTTGGCACTGTATTTTTCCGCGTTCAACGTCCTTACAAAGTTGTtCTTGGATTTCtATAAAaCGCAATTCACTACGAGCATAaGAACCTAATTTGTCTTCTTCCCAGGCGCTGTCACCtCCTTCGAATTTTCCGCGTCTCGTGGATTCGATCCCTTTGTTGAAACTCTCGATAAGTAATTTGCAAGCAGCAcATGGTGGAAGTCGAATAATTTCTAAATCTTCTTTACG

>contig00824

CTTCAATGTGTTGCAATTTTTtATCCAAaTATTCTTCAGCTGTCTCATTACTATTCTTTCCACTTGTCTCTACAATTTCCTTTTtGCTTATGCTACCAGTAAAGTCAGGTGTAACCATCATtGGACTATGAACTGAGTTATCTTCGTTAAACAATGCTTCATTAGATTCTTCATGTTCAGAATGTCGAGACTTCTTTTttCTAtGGTTATTTtCATTTGCAAAGCAATCTGACGAATTATCAGTATTTTCGATTTCAAAAACTCTGCTTAGTTTCCTTTCTCCTtGTGAAAAACTTGTTGCTTCTTTCAAAACTTTAACTTCATATCGGTCAAAATCTGCTCGAGGTGGCACGCACTTTttCACCCATTCCaCTATAAAAG

>contig00825

ACCAGAACAGTTGGTATTTCGCCACACTCGTTCTCCACCTTCAACTTCCAGGAGGGGATGgCATCGAAGGAATCTCTATCTGTAGCTGAGTAAGCCAATACACATGCGTGAGCTCCTCGGTAATAAGCAGCCGTAATTGCATCGAATTCTTCTTGACCTGCAGTGTCCCAAAGCATCAAACGAACGTCCTCTCCATCGACCTCAATCTCTCGCTCGAGAAAaTCGACTCCTATCGTTTTCTTGTAGTCCCTCGTGTAaGTTCCTTtaCAAAaTCTTtGGATCATTGAGGATTTTCCCACAGCACCATTGCCcACTATTACCACCTTGAGGgATATCTCGAGCTCTTCCTCTCGCATTCTGACCACACCGTAATTAGCATAATAATATCCCACTAGAATTTCCTTCGCCGCTTCTGCGACTCTTCGAACGGAAAACTTATGCC

>contig00826

ACTTTTTtAACAATTTATTTAGTGAAAGATATATATTTTTCTGGACAATGGCAGTCAAACAAGCATCGGCCTGTTtACAAACGATAAGCAAAATGCAAAGgTTTtCAATGTCAACTGTTAGCAaGATTGGAAATCGTGAAATAGTTGGATACGGGTTGAATGGATCACCTTCTTATcTtGatcgtCtAGAGCGCCCGATGCCAGCTATTCGTTTCAAGGAAAaCACTCCTGAAATTCAGGcCTTAAGAgaGAaGgAGAAAGGAGACTGGAAGAAGCTTTCGATTCAGGAAAAGAAAGCACTTTATAGAGCAAGTTTTtGTCAAACTTTTGCTGAAATACAAGCGCCAACTGGAGAGTGGAAATCTATCATTGGAATGACTTTGATAGGCATGAGTGCTGGTATCTGG

>contig00827

ACttAGTtAaTTTTCGAGCCTGTCGGTCCATCAATGATGTTCTAGTGCGCGTTTTTTtCCAACTATAGTAATaTTTCACTAAGCTAGCAATGGATTTATCAGGAAGCATTTGGCGAATACGGTGGAAACTTTtCCCGTGAAATTGAAaGGCTTGTTCGAaTAAAACTTtATCTTCAACGGTCCATTCATCGGgAAAAGGAGTGAAATTTGCTAAATCCAGCACTGCACGTTCtAAATTGTGCTTGTGCcAAAATAACATACCTAGTGCTTGTTCTCCGTTATAGCCATATTTTtCTTTTGCTAATATAATATATTCGTCCAATTTTtGAtCCGGAATTTCTGTAGTTGGAGACCAAACGAGTAATGCTCTATCGGGACACTGATCCAATCGTCGTtCATTtAaTGGAAtAAATTCTGGAACAATCACTTGATAATCTCGGCCAACACGGATTTTTTCGGCAGGAACACCTTCTTCGTCGCTGGAGGAATCGGTTTGACCATTAGGACTAGGGCCTCTGGATCTACGACCaTTTCTAaGaTTCTCGGAaTTTCTTTCagaCTaGT

>contig00828

ACATTATTTtCCGAAAGGGAATCGTCACTTTTTTtaGAAGCCTGCTCTTTTtCttCATATGCTTTCAGTCTTCTTTCTAATTCCAAATTTTTACtCTGACAGATCACAAGCTTTTCTTCCACTTTTTtCTtCGCAAAAGCTCATCAGTCAaTAAGTCTCGTtCTCGAGAAATGGAGGCAACaTCTTGTTGCAATCTCTTCACCATAGATCGCATTTCCTtCcGTTTGTTGGTCATATTTGCCAACTCCTGTTTCATAACTGCTATGTAGGTAATCAAAGTATTAGGATCTCTTGTCTGTCTGACCATATCGTTGACGTTTTCGAGGGCAGAATTCACTAAAGTTTGAATATTGCGGAATAGGTCTACTTGTTCTCTTAATTTTtCTAACTCTTTTTGTTGTGCTTCTGCTTGCAGAGATTTTtCTCTtAAATATTTtGCCTGTTCTTGtAGTGCAAAAATAGATGAGTTtCTCGATTGTATATCATTTtCCAATCGTCCAACCTCTTTtCGGAGACCATGAACTTGTTtATCCAAAGACTCTGCTTTCGACAAATAGTGATTTATGTCTTTTtCTTTTAACAGTAGCTTGAACTCTAGACTTTCGACTCTGTTTTGAAAG

>contig00829

GCGAGAACGACACAAATATGCAGCTGCACATTCGTGGACAGGAAACTCGTGTCATCGAGTGTCAAGATGACGATACTGTGGCACATGTCAAGACTAAGATCTTAGCCCTTGAAAACATCGAATCTGGAGCCGTTTGCATTTCTTGTGCTGGAAGCCCATTGGCAGACAATGTTTTCGTTAGAGACCTTGAATTCGATACACTCGATTTCAACATCCCACTTCTCGGAGGTAAGGTTCACGGATCTCTTGCTCGAGCTGGTAAAGTAAAAGGACAAACGCCGAAGGTAGAGAAAAAaGAAAAGAGTAAGAAGAAGACCGGTCGTGCAAAAAGGCGTATTCAGTATAACCGTAGGTTTGTCAATGTTGTTCAAACTTACGGACGTCGTCGGGGACCTAATGCTAATGCAAACTCATAAGCATTTCATTAATTTTTCATATTTTATCTGCTTTTtGTATTTgTAGT

>contig00830

AAGtAAAACCTATGGTAGTCGTCGTGACTTATACTTGCGATCAATGTGGGGCTGAAACTTACCAACCTGTGCAGTCAATGAGTTTTATGCCTCTTCAGTCTTGCCCGAGTGAAGACTGTCGACTAAACAAATCTGGAGGAAGACTCTGCATGCAAACTAAAGGTTCCAAGTTTGTCAAATTTCAAGAAATAAAAaTGCAAGAACATAGCGATCaaGTTCCAgTTGgACACATACCTAGGTCACTGACAATTtACTGCCGAGGCGAGACTACAAGAAaTTGTCAACCAGGGGATCACGTTATTATCaCTGGAGTATTTTtGCCACTTATGAAAAGCGGTTTCGCTGCTGAAAGTGCTGCTGGACTTCTCAGCGAGACTTTCATTGATGCTCATaGGGtAGTTtGTTTGAgCAACTCTCAAaCAACAGACGAGGAATCCTCAGAATTAACTgATGCAGAATTATCTGAACTTAGAAGGCCAGATTTCTATGACAAGTTAGCCTGCTCTATAGcTCCGGAAATTTATGGCCTCGAAGATGTGAAAAAaGCGCTACTCTTGCTTCTCGTTGGTGGAACTGACAaGAAAAAAGGAGACATTAAGATCAGGGGAAACATAAATATCTGCCTTATGGGAGATCCTGGAGTCGCAAAATCACAGTTACTTTCCTTCATAACTAGGCTCTCTCCAAGATCTCAGT

>contig00831

ACCAATGCTTCGACAGGTGAAATCCCGTCCTTGGTGAGCACCAAAACAAATTGCTACAAGTTTTTCACCATTTCTAAATATGGGCGCACCAAACCATTTTATTTTAGCAGAATGCAAAGGTCTCTCTTCTTTCGCCGAGAAATA

>contig00832

TATTATAAACAAATTTATTTTCTATTTATCTTGAATAGTTATAATTGTCAACACTTGTATACAGCTGTGCAACATGGAATAGTATTCTGCATGTAGTGTTTACCTTGTGTTATCATTTATTAGTGAAATCCTTACTAAATTTATATAAAGTTAATCCACTATGGCAACTGAGAAAAGTAATCCAAAGGACGTAAGTCTTGA

>contig00833

GAGAGAAATCGTCAAAAAGC

>contig00834

ACTCTTTTtGTTCAAAATaTACAGCAGCGATaTTTAACaTGAAAGTAATTTCAGTTGGGTCGAGTTGAACGGCTTTGTTGTAATGTTCAAGAGCTACTTCAAAGTTCTTCTTTTTaTAGGCTTCGTTCCCGAGGTTTTTttCAGCAAGGGCTTGTTTCTTTTCTGGAGAAAGATTGTCTTCTTCCTTGGGTTTAGGCTGCTCTTTAGGCTTTGGTGGTTCTTGTCGCTTCGGTGGATCTATATCCATTGGTTCTTCCATGTTTATAttCATTtCAAAAAGAaCACCAaGGGTCGTCATTATtCTTTGGTCCTGTAAaTACGTTCCTATGAGTTTtGGATTGGCACGGATTTCCTGTATCAGTTTCAaGTATTCAGGgTCGTCAAGATAGGCTCTGGTTCGTGGATTGTTTCTGAGCTTCACAAaGaGGtcGGGAGTATTAAAAGGATTCGTCATCGAAGGGGCTTGCTGCGCTCTAAGATCTGCTAGACTTTCCTTGATTtGTGTATTTGAGGGATCGAGTTGAAGACCTTTTTCGTATGCTTTAATGGAATTTTCTATGTTACCCAAATATGCCAATGCGCTACCTTTTCGTGAATAACCTTTAGCCCAGTTGGGTTGTAGTtCAACCGTCTTTTCAGCaTCCTCGAGAGCCTGAGtGTATTTTCCTGCCTTAGCATAAGCTGCAGACCTATTGC

>contig00835

GGCATCAGTTTCATCATatCAACAgC

>contig00836

GGTTGAGCTTCCATAGATCCAACGGATGGCGGACcACCAGCATCCATGCTGTCTTCACCCACTCCTACTCCTACTCCTACTCCTACTCCTACTCCTAATCCTATTCCCATGCCTAGCACATCGGGTTCCAGCTTCGGAATCGgTGAaGAATCCATTtCAAaTGATCCAGTTGCACCAAGTGGTTTGATTTGACTTtGCATTTGAGCCACTTCACTTGACACCTTAGCTGGGTtGATAGGCTTGCGGAAGAAaTAAATCTCGT

>contig00837

AGACCGTCCTGATTTAGTTTTCTCATTAAGTTTTTTGTTTCATCGTAAGTGGACTCACAAATAAAAACATCGTCCTCTGGAATTTCAGTTGGACGACTGCAAACGTATTCGCCGTAATCTAaGT

>contig00838

ACAAAGTATCGCGAATGGGACAAGTATTGCTTGAGaGTTTATTCAATTTGTCGAAATGTCACCTACTGCTATCAGTTTATTTATTCTACTGATATTTTCGTTGCAAAAGGTTGATGGTCATATTAAATCTGGGCAGACGGATGGAATTAATTTGAAGAATATCATGGCTTCTTTCTGGAGAATTCAAGAGGGTAAAGATGAGTTAATATTGATATGTATGGGAGTCTTTATAAGATCAAATTGGATTTtGGTTACTGGTAGCGGTGGTGAGCAGTTGAACCCAACCAATAATATAGTAATTAAATCTGGAGATGAAGCTGTTCAAAAGACTTTGTCAGAAAATATGCCAATAGACTGTAAGAAAAATCTTCATCCAAATTCCCAGTTTCAAATTGTATATTTAAAATGCAGTATTTCACTACTATCGTCTCCAAAaTTtGAGTATTGTtACGTTCCTCCAAACGAGAAACGCGAGTATGAAGGAAgAaTAGTAGGATGGAATGAACAGGATGGATGTCCACAGGAAAcTACAGGTAAAATCATTATTTGGtCTcAACAAGCAAGGCTGAAAGGGACAaCCAAAATCTACCCAAGATCAATTTTCCCCGATGGCACTACAGTTTtGAAGGATAATAAtATtATGGCATTTGCTGGTGGTGTtGGAGAAGATAGTGATGGATTAACCTTGT

>contig00839

ACCAAAATAGTAATGCGTAGTGAGTATTTTACCGAACCACCGTTTTATACACACATTGCTTTCTTTTTTTACGCGGGTGAAAGTCCCTTGAGCTGTTTAAAAGCGCTATAAAACTACACGAAAATTTATTTCTCATCTTTCTTAAAATTTTCCAACGTGTAGAGCTTACTTTAAATTAAAACTTGTAGTATAAATTGTGAGAAATGAATAGAAAATATTTAAATTAAGAGCTTTCAAGGCGATAATTGAGGGAATAGGgAATTAAAAGTATTACTTCATTCCTTGTAAAAAaGaTAAACGTCAAGAGGCATGATATGATTAGCGAAACAGTTTTGTtCCATTGTAAAaTATTCTTTACGATTTATTAAATCAATTACtAATTTATTTCTAATAATTTtCCTtCGCATTATAAACTCTGCGGGAAATTTCAATTGAAAAaCATCAGAGCGTCTTTACTGTAATACACGACGAAGGAAaTTtATCTATATTGTAATTAAACTGTATAATAAGATAGAGAATCGAGAGAAAGAGGGgaGAGAAaGATACTATAaTGTTATGTTGGACTAGATTGTATATTTTGTGATACGAGGGGACTGTTGAGAACTGGAAGGTCTTTGTTAGCCTTCAGAGAGAAAGGTaTATACAGAtATATATTAtATTACTGTTaTTCTATCGTTtACGAGTAAAAAaGCGT

>contig00840

CGTAAAGACCGCTGTAAGGGCGTAGAGAGCAAGCGTCGTCGTAACAGTGAAGAGTTGAGAGtAATGAAAATGTGAATACCAaGCAAGTCCCAAAaGGAGT

>contig00841

TCtGTAGTTGCAAGGGCTACTAAaGAAGCATTTTCAGCCcAATATACATGAGTTGGTTGTATATCTATACGTCTTACTAGCTTCAAaCTATCCCAaTCATAAAATGACAAACCCGATCCAGACGAtaCTCCAAGCATAAAaCCACCAAAAATTCCGTCTG

>contig00842

TCCACATACGACTAGGAATCTGCCGTTAGGATTGTGCTGAATTGTTTGGGGATAAaTTtCACAAGCACCCAT

>contig00843

CCTCCGCCACCATAATAATCATGTCCATAACGTGGTCTATCGTCCCAATCACCACGCATTCTTTTCATAGGTGGAGCCATTTCTTGCGACCGAGCTGGGCTATAACGGTCACGACCACCGCCTCCACCACCTCCACGGTATTCCCGATAATCTGGTCTCGCTCGAGGGCGGCTAGACCATTCTCTATCAACCCAATCATCACGTCGTCGATCGTCTCTGCGGCCTTCTCTAGAATAAGATTCAGTCCTTTCACCTCTGAATTTATCCCTCCTTTTtCTATCATATTCATCATCAGAATCTGCCATT

>contig00844

GTGGCCCGCAAATGTAGAGCTTTTCTCCCCACA

>contig00845

ATAAATCTCTCTAATAATGGCCACGTTTTCATCCCTGTCACCAGCTTCACCTACTTC

>contig00846

ATGTAAAAAaTCTTTTTTtCCAATATGGAATGTATGAAC

>contig00847

AGTTTCTAAAAGACTTAAATTaCTTACGCCGAGGTGAGAAaTTGTGATGATGTGTCACTCGTATGTGAaTATCTTGCGTTTCGCCTGCAGAATTAATAACGAAaCATTATCAGAAATTTCAaTTTAAaGTATTTtCATTCACCCGGAACAATAACACCAAGAGATCAaTAATTCTAAAATGCaGgCGAAAAaaCACATTCGATTTTtAGATTTTAGAGCGTTTGTTAAAGCTAGAAAACACTTTTAGTTATTATtcACAATATTATTATCACTGATATTATCTATATGTATAGTCACATCTTGCCTTGACAAAAAGACAACaCTAaCAaGAcAATGT

>contig00848

ACCACCtAtCATTTTGGTAGACAATGTAGAGTTGCTGATTACGGTTTTACTAAATTAATAGATTTGTTGGAAGCTCTTACCCACaCTGTCCAGGTGATGGGCGAGGGAAACAAACGAGTAGTAACTCTTTCACATAGAGCCCAAGTTCGTCGCTTCACTTCTGATTtGCTTCGCGTtCTGAAaGCTCAAGCTAGCAAaCAAGTCGTCCTTTCcGAGTTCCCTAGCGTCTATGCCAGAGTCGTGGGGAAGCCATGGgATGTTGTTGACTACGGTGTTtGTGAAATAGGAGATATTCTTGGAGAAGTCTCAgaGAACACAGTCGtCGTTTCTAATTATAATGACGACGATCAGTTGATCGCTATTCCCAAACGAGAGCAAACTCCAGAGGAAATAGAAAGAACCAAGCAGTTtGCTGTTGAGGTCGTAGAACTTTTACGTCACGCGCCTCAATGTAGAATGTTATTCAACAAATTCGTGCCTTCCTATCATCATCA

>contig00849

ACAAGGGACACGTGTgCTTGATTGTCAACGtGGCTTCAAAATGTGGCCTtACAGCAACTAATTACAAGGAGCTTAACGAACTTTAtGaTCAATATTCaGAGTCAAAAGGCTTaCgCATTCTAGCTTTTCCATGTGATCAATTCAaCGGACAAGAACCTGGCAGTGCTGATGACATATGCAGATTTGCAGATCGTCAAAAGGTAAAATTTGACCTATTTGAAAAAATCAATGTCAACGGGGATAGTGCTCATCCCTTATGGAAATATTTAAAGAAAGAGCAGGGAGGATCTCTAGGAAGCTTTATCAAATGGAATTTTACGAAATTCATTGTGGATAAGGAGGGTAAAGTCGTGGAAAGACATGGTCCAAATGTGGACCCCAGTAAGTTGGCAGCTAATCTTGAAAAATATTTTTAAA

>contig00850

ACCAGtgtCCAtCAGCAGTTAAAAAAaTTCCCGGTCGAAAAaGCTCTGCGTCATATGAACTCATATTTTTGGCTACTGATCTACCTAGTCTAGGCTACCAATGCTTTTATGTCAGTAAAAGAAGAAAAGAAACTGAAATAGATTCTAGGAAATCAAAAAAGAATACACATTCTTGGTTCAAGGGTAGCAATGCATATGAAATTTCTATCGAGAGAAATGGTGAAGTCGTCATTAAATCTACACTTGAACGTATTCAATTGAGACAATCTTTCTTTtACTATGAAGCAGCAAGTGGCTATAATAGAAGACCTGAAAATCGTGCATCTGGTGCTTATATATTTAGACCAAaGACACCTAACGCTAAACGTTTTtCTTACACAGGAAATtATCGAATATTTCACGGTCCATTAGTGAAAGAACTTCAAATGACCATAAaCgATtGGgTGAGCCAGGTTATCAGAGTTTACAAtGGAGAAAATAGAATTGAaTTCAactGGCtCATtGGTCCAGtCAAtGTCAGCGACAACATCGGAAAAGAAGTtATAACCAGATACACCACTGAATTGAAATCAAATGGAGAaTTTTtCACAGACAGCAaTGGCCGaGAAaTGTTGAAaCGAAAAaGAAaTGTGCGATCAACATGGAATGTAAaCCTTGTtGaGaCAaTCGCGGGAAaTTACTATCCAGT

>contig00851

ACTGCAAATTTAACCCAACGTATTACTTGAAAGTTGTTCAATTTCATTGAAATGTCACTTGTAAAAATTGGTTTATTTATCCTTTTGATTGCCATACCGCAAAATGCAGAATCTTCATCCCAAGGAGGGGAAGAATCGGATCTAAATAATCTTAACCAACAAAAATTGCAAAGAGCTTCAGGCATGTCGCAAGATCAACTGCTTGAATATTCTGGGAAAGGAAAAGAAGTACCCTCTGCTCCAGTAAGTACAGGTTCATCCAATAAAAAAaGAAAAaCCAaTAAATGATAAAGGAAAAGAGAAATCAAGACGGGAGATGAGGAATGCGATTAGAGAGAAATCTAAAAACaGGAAAGCTG

>contig00852

ACAATCTTTGCTTGGAAACCTTTGCATTGGAAATTCTAAGAAaCGTTCTGCTGAGGACACTTCTGACGAAGAATCT

>contig00853

AGTTTtAGAACAACGTCACCTCTCTCCACTTCTCTTAATTTAatACGTTTTtGAaTGGGTCCACAGGAATCACTGCTATCTCTCATAATGTCAACAACTCGAATCTcAGCTCGACCCATGAATTCATCAGGACTGTAGTATCCTTTATCAAAAACcGTGATACATAATGTGTCTTCGTGGAGATCCTtAACTtGAAATTGCATCGATGCATCCCACAGAGGACAaTCTGTTCCAGAATCAACAGATGTTCGTTCCTCCTGCG

>contig00854

ACtCGTtCTCTTATCGCATCATCTTAAATTATCTAATCCGATAAGCTCTTTTAAATGTGGTCCGTTGATCACAATCTCCaGAAGACAGCCATTTTTTTtGGtGGCAGATTATTTAAAAATTCATATTTTACATTGTCTCATAGAATATCAAGTCATCAACCAAAATAAGCTCCGCAGTGGGAACAACGTCGAtttttcAATAGGAGACAACATATGATTCCAAGAGGAAAGAAGAAGATTGCACAGAaGATtCCTAGACACGTGTAATCATCCTCTAGAACACCcA

>contig00855

ATATAAGAGTAGATCCCAGACAGGTCGCATCAGCATTTTGGTCGTATTCGAACTGAGGACCTCTTCGCGTGGTGTTGTTTGCCTTCAAACCTTAACGAAGATTGTGTCGAAATTTAATGTGAGACTCGAATAATTGTTACTCAGCCACAAACATTTTTCATTCGACCATGGCTCCAATCCAAAACACAATGTTATCGGATATGGATGAACTTCCGTCCATCAAGCTTGGGGACTTTGTCCTTGAGTTCGAGCTTGGTCCACCGAGTCCTGAAATTCAGGAAGTTGCGAACAAGGAACTTAGAGAATCTCCAGAATTGCAAAAGGAAGCAATGTCTCGACTGAAAGAATTGCTTAGAGCTGAACCTGACCTGACCTGTCCATTGGACAATGAGGCCTGGCTCATAAGATTCTTAAGGCCATGCAAATATTATCCAGAGTCAGCATGCAAACTAGTGAAAAATTATTACAGCTTCAAAGTCAAGCACGCAAACGTCTACACAGGCTTGAAACCGAGTAACGAGAGGAACATTTTTGAACACAACATTCTTACTGTTTTACCTAATCGGGATCAACAtGGACGGCGGATTTTAATTATAGAATTAGGGAAAAAaTGGAAGCATAATAAATGTAGTCTTGATGAAGTTTACAAGGGGTGTGTGT

>contig00856

CCTTCTTTCGCTCACTGTTCGTGCTGAGTCCTTAAATCTCAGTTAGGTCTCTACTATCACAAAGATGGGTAGAGAGGACAAAGCATCTTGGAAGTCAAATTACTTCACCAAACTTATTCAACTTTTGGACGACTACCCAAAATGCTTTATTGTGGGAGCGGACAATGTTGGGTCGAAGCAGATGCAACAAATTCGCATGACCCTGAGAGGAAGTGCCGTCGTTCTCATGGGAAAGAACACCATGATGCGAAAAGCTATTCGTGGTCACATAGAACGCAATGCCGCTTTGGAGAAACTTCtCCcTCACATCAAGGGAAACGTTGGCTTTGtATTtACCCGCGGTGACCTGGTCGAAGTGAGGGACAAGCTAGTAGAGAACAAAGTCCGTGCTCCTGCGAGGGCTGGGGCAATCGCTCCATTGAGTGTCATCATTCCCGCTCAAAACACCGGTTTGGGACCCGAAAAAACTTCCTTCTTCCAAGCATTGAGCATTCCGACCAAGATTTCAAAGGGAACTATTGAAATCATTAATGATGTGCACATCCTGAAACCTGGAGACAAAGTGGGAGCTTCAGAAGCCACGCTTTTGAATATGTTGAACATCTCTCCCTTCTCATACGGATTACTCGTTGAACAAGTTTATGACTCAGGT

>contig00857

ACTTCAAGAAGCAGATACGCTGAATTTaTTAAAATTTtGTGTTATACATAATTATTGTAATCCATGTTGTAACATCAGTGATGAACGATGGATACAAAGCTTGATAATTTAATAAAACATTTTCAGAAATAATTAAATGCAGTCCTCAAATTAAAGAGCTTAGATGTTCCTATATTCCTGGCTGAAGCTTGATCCACGAAGAATGCTAATGAAGACTTCTTTCAATGGATTAGGAACTCCTATATCTTGTTACTTATGACTTGACTTTtCCTGATTGTTtCTTCTTATGTTCGGTGTTTATGAGTTTAGTCAGGATTTTTCTAGAATGGCCGCACGGATCGAAATGGTTTGTATGAAGATCTGCTTCGCGTTTGCTTCTTAATTTCCCACTATGGCCACTGAACATGTGCTTGTCATAATCATAGTTAAAGTGCTCGTACTCGTCAAAATGTTCCTCCGACATATTTCACGAATTGATTATTTCAAGTTGTTGAAAGCTCAGTAAAGTTTGCGCTTCGAATTCTTCGAAAGATGCTCAGTTACTTTCAGTTGCTTTTGTGTCAATaCTC

>contig00858

ATACATAATATTACtGGCGATAATGGCTTTGTTGTCCTTt

>contig00859

CTcTTtGACAAAGTaGCGACTTCCTtAAGCAAGCCAGAAATTTTtAATTGCATTGAGGAAATTTTCGCATCATACTCTTTTTCGGTTGTCTTCATTTTACTGCGCaTTTCGGCCTCAGATGTGAAATTCTTTGCCTTCAGTTCAGTTATCTGTTTATCCTTAGTTAACAATTGTCCGTCTCGTATACT

>contig00860

GATTCCGGCTCAGATTGCGGTGGAAGTTTA

>contig00861

ATCGACTCTCTGAGGTCTTTtATTATGGCCTTTTAATTTCTGTTCTTTATGCTTTGCTGCTCGTTGTTG

>contig00862

GAAAaTTGTAAaTTTTtAAATGGAATGAAaGAACGAGATGATTTTAAAGCAACCTcGTATGATATtGATCATGTAAAAAaCTTTGCGAAATATAAAAAGCCAAAAGaCGCCAATATTGACTTTGGACTTATCATACTCAAGCAATCAGTAGGATCACCAATACCAAACTTTTATCTTCCGATACTAACTGAAAAATTtCaGAAAACAAGAACATTTACTTtATTCGGATTtAAaTaTTGTGGAGATAAATTTGATGATAAaGAAAATCGATTATGGAGGTTGGATATTGAACTCGATGCTGATGGGATATGTCAGACCTCAATTCCAGATTATAACTTtgATAAGAAATCGGAAGCGTGCGGAGGCTCTAAAGAAAACGAGATTCATGAATTTACTGAAGATGACAATGGTACTCCTGCACTGTATAGAAATCAGCTTCAGGGAATCGTTAAACGTGT

>contig00863

ATATTGGTCACCGGTGGAACCGGGTTAGTTGGTAAAGCAATTGAATCGGTTATAAAGAATGATAAATGCCCGAATGAAAAATGGATTTTTGTCGGTTCAAAAGATGCAGATTTGTGCGATAAAAAGAGCACAGAAGAGTTATTTGAAAAATACAAACCTACTCACGTTATTCACCTGGCCGCAATAGTGGGTGGTTTGTTTTCTAATATGAAACATAACTTGGATTTTTTGCGGAAGAATTTGCAAATCAATGACAATGTCCTTCAAGCATCCTATGAGAAAAATGTAATTAAAGTTGTATCATGTCTTTCAACTTGCATATTTCCTGATAAAaCTACGTATCCTATCGATGAAACTATGGTtCACAaTGgACcTCcACATCACTCAAaTTATGgTTACAGTta

>contig00864

ACGtAAAATAAATAGTCACAATATTAAGTTTAGATTTTCGTCAGACGAAACATTCTTGAAAGTCTGTTCAAAAATTGGAATACGAAATACGAGGCATCAATCTCTATGGATGCCAGATTTGAATTAAAATAAGACCCGGATCGTTTATCTTTTCTGTTTCTTGTGCCTGTGAGAGAATGTTCGGTTGTGAtCCATTTTTGGGGCCTGACACTAGGAGCTCGCGTTCcTCGAGATGCTTTGCACGACGCGTTGTCCCCACCAAGTCTCAAGATTAAATGGCTGGAAGTCTTTAAGCTGCGGATTAGGTTCCCGTTCCTGATAATACGTAACAGAGGCTCCATTTCTGTAaTTtGACGAATTGCAGGTGTCcAATTCTCTTGACACAGAATTCCATGAGTCGTAAATGTATTTGATTAGGTCCAAATGCTGGGAaGTGGACTCTGTTTGTTGCCTTGGAGGGATTGAGGAAGGCCTCTTTCCATTCATTGCaGTCATTTGTGGTCGGCCACTCATTTTATGGTCGTTCAGCTACAGAATCGTGTGGTCAACTGCTCGACTTACTCTATTCTCGTCAGGGCGGAGAGTAGCCGTCAGTCAAAGAAGCGGAAATATCACAAAACGCGTAATTAGTAAGATACGTCACTTTTTTCGGAAGAAATATATATATAAGTgCCtCGTTGACAACGCG

>contig00865

CAATACTCGAACTCTCTTTtCACTTGGCAACCACTCGGCAATAGCCAATTGTGATTTACGTTCCACCCTCTCTATAATCAATAAATCTTTCCTGTGCTGAAGACCTTTTTCAACCTGTTTTTGCTGCAACCATGAACCATTAGGCTGAAATAACTTTTTCCCAGTTTTAGGCAGAACCTTTAAAGATTTTTCATATTCATCCCAAGCTTCATATCGAATTCCTTTGTTCTGCAGAAGGCTTTCACCCTTTAAAAAGTTGTCGAAAAAAGACTCCGAATTTTCTTCCATTATTTCATATTAGTCAACATCTACTTATTATTGTTTTTCTCCTTTTCCGCGTTGACCCAATGCGAAACTTAGCGAAACACTCAAAAATGTAAAAG

>contig00866

ACGCtCtCCGTtAGGAGCTCGCCCCAAAGCTCCTCTATCGTCCCAaGCACATATAATTATCACTCCGGGTTTTTtCTGAATTTGATTTACGTAATCTAAAGTATAAGTCATATTTTtATCCCCTaCAGAGCTTATAAATCTTTCtCCGATGAATACTACATCAGCGTATGGCACCAATTTAAaTATTCCCGGtCTTTTGTtATCTAACTCGATACTAATAACAATAGGACTATGCGTAATGTCTGTGAATTTACTTTTtAACCTTCCATTAtGATTATGTATAGCTTCCATCATATCTATTACATtATCCACATTtCGTCCTTCAAaGtGAATCCAACTATAATCATCTAAGTTAaGTTCTTTAAAATCTTGAATAGTTAGTTCAGGTAAATCAGGATTAAAATTCCACATTGTGCGAGACAAGTTTTttGAATTTATTAAAaTAGAGGTAGTAGGACAAGTAGTATTGTGCTTGATAGGGCAATGAGAAAAATCGATATTGTGTTCACTCATATCCTTCATTAGCTTCTCTAATCTTGGCTCTTCATCAGAGAGAGTGCCTAAGAATTCAACTGGGACTTCAAATTGTGAAAGCACTGTGCATGTGTTTGAGGCATTTCCACCTCGTTGCCATCTACACTCCACGCATTCAAGTTCAGCATATTCTTTTGGAAATTCTCGACAAACT

>contig00867

ACTCCAAGTATCCACATGTCGTTTCTTTTCTCTTTATTGTTTCTCCACTTATCATTATAGCATTTTATCCAGGGCGAATCAAGTCCCGTCACTAAATTCTCACAGCTTAAAAACCTGAACCATTGATAAATGTTGAACTTTGATCAAATATTTTAGACTTTTTAAAGAATAATTTCAAAAAATTTGGACAGGGTTTAAATATTTTGTCCAAGCAAATATAATAAAAATATATTGTTACAAATATCGTAAAAACTCAATACCATTTTTTAAACCAATATTGTCTTTtACTTCAAATTTTACAGTATGAAGAATTTaTTTGCTTTATTATTATTCTTCATAACATGGATTCGCCCATGATCATTCAATACCTTGTATATATTcATTATAATGACTTAATTCATAAaTCAAGAAAGCTTTCATTTATTTTTGTAAAAaCCCcaCAATTTtGAAACTTTTATACACAtAAAGGACTTCAAAAGAGATTTTAGATATAAATTCCTATTTTATTTGAGATTTAATCCAGCTTTCACAATATGACTGATTTttATTTtACTGTTATATTTTTtCATTCACcAAGCTTTTCCTCGGGTGCATAAAATTTACTCCGGCGTTCATCCGTATGTATACGGATAATGTGATGAAGCAAATCTAATGCATTTAGTGATGCCGAAaCTACTCTTATTaGT

>contig00868

tAgaCtAAtAGTTTTCGAAtCCAACTACTTATACACATTCACTTCTTtCGGACGAACTGTTTCGACGAAGGAAAAaaTATACTTtCCctAGAATTACTATtACCCAATACcTCATAAGCTAGTGAACACAGGGATaTTATTAGAACGTCTcAAAAaTTTCAATATCaGGCTTTTTtGTCTTAAAACATTGTATGCCTAAAAGACGACTaTCAtACTtAATACCTCCTCTGGGAaCAAAACGTCTGACATTGAAGTCTGTGAGACATTTCATAGACGTCTCGAAGACGTCCAATGTCTTCGTATCCACTGAAAaCTTAATACAAACGCAACGCAAAATTTCACAACATCAATAACTCGTTCTTAAAAGGTTTCTTGATTTAAAGTAAATAATCTTATTATTGATACGCTCTTAACAATAAATTTCTGtAAAaGTGAGTGCACGTCTTTTTCAGCCTCTTGGATTACAAGTAATTAACAATGTTACATATAATACAAGTAATAATGAATTAAATAGaTTATAAaTACTGAATAATGCTGTGATAAAAATATGTTCAACAGGATTGATTtACAACATTTTCTATTCTGACATTCAATAATAAAAaCAATCACAAACCTCGTTTCGACGTTGATCGGTAAAATAGAGGAATGTATGACACATAaTGGACGTTATCTAATGTTTAAaGGG

>contig00869

ACGCACTTtGGACAGTTAACTCCAtGCAATAcTCCAGCAACGCCACCAAATTTTCCAGACGCATTACTAGcaTTTTCCAAAATGTCGGCtGgAGAAAAAaCCCCcTcGGGgATGTCTCCAAGTCAAAATGGAACCTTTTGTCAGCAGCAACAGCAGACAGCGGCACAACaTCATGCAGGTCGATGTAGCGTCTCTGGCAGCTCACAACAGCAaGCTAAAaCTCAACAGCAATTCGGCCTAGGCGAGCCACAGAGATAAAAAaTGCTGCTTTTtGCTTTtGCACTGTGCGATTCATCGATCGCAAGAAGACTATTGCGTTGGCGCGTTCCATAAAAAaCGAaCGAAGTATCGATGATCCGTAAATGCTGACAATATTTGAAGGATAATGCCTAACTGATCGTTtGATCGGAAaCCAAGACAAGGCGTCGATGGAATGAGCTTGATGGATCTtGTTTTtCCATCTTACACTCAGATGTTtCTTTGGGGATTAAaGAAAAATCCTATCGGCCGCTGCTTCTGCTTTTGGCCAGAATCCGCTAGCAATTACGTCCTTAGATATTTTTATTTCATGTCCTCGCACCGATGGGTTTAAGAGGATCCAAACTGGCCAACAAGTTGTTTCGAGAGCTCTAAGGCTGTAATTTCAATAAAAGACAATGAAAGATGGCGGtATCGTTAGCAATTAA

>contig00870

ACCAATCCCGATCGAAATGATTGTCGTGGTTCTGGGAACGGTGTCATCGATATATATGAATTTGGCAGAATCTTATGGTATTATAACCGTGGGAGATATACCAGTGGGATTACCAGAACCAAAACTGCCACCTTTTAGGTTACTAGGTGCTGTAGCTTTAGATAGTTTtATTATtACAATGGTGTCCTATACGATATCAATGTCAATGGCATTAATATTTGCTCAAAAATTCAACTaCGAAATTAACGCCAATCAAGAATTATTtGCTCAGGGCCTGGGAAaTCTTGTGGgATCCTTCTTCTCCTGTATGCCATCTTGTGCCTCGTTGTCCAGATCATTAATTCAACAAACTGTCGGAGgTCAGACTCAATTAGCAAGTATTATTtCTTGTAGCATTTTGTTGAGTGTCTTACTTTGGATtGGTCCCTTTTTtGAACTTTtACCAAGATGCATTCTTGCTGGTATTATAATAGTAGCACTGAAAGGGATGATATTACAAGTTAAAGATTTCGTGAGATTTTGGAAAATGTCAAAAATGGATGCTGGCATCTGGATGATGACTTTtCTCAGTGTCGTCATTTtAGACGTTGAATATGGACTTCTTGTTGGTaTAGTTTtATGCATTGGAAATTTGAtGTCATTGTCAATGAGACCCTACACTTGCAAACTAGCTCTTGTTCCTGGT

>contig00871

ACTTCAATCCCGCtGAATGCTGAATTGACAGGTTTCATTTGATTGTGTAGGAAGTTGTCGGATTGAACTGGATTGAAGGTAAGAAAGGTTCATAAGTCGGCAAAAGTTAACAAAATTTGGTGCAATTTGATACAGCAAAGAAAATGGAGATTCTAAAACATTCCGAGCAATTTGAAGAGTGGAAAAAAAaGGCCTTTCTAGGTGCCCTTCCAAGTGGCTTTTTAAGAGTTGTCAATGTTAATCCTCAAGCTCAACAGGAAGCTGCAGATCAGCAAGCAGCGTTGGCACTACATCAGGCTCAGATGCGAAACATGCCAACTCAGGCTCATGATCCAAGAGTAGGAAGACTTAGCATAACAATAACTCAGGCTAAATTAGTAAAAAaTTATGGAATGACCAGAATGGATCCCTACGTAAGACTTCGTGTTGGACACGCCGTATATGAAaCACATACAgCAaTAAACGGaGGAAAAAaTCCACACTGGAATAAAGTTATTCAATGCCTACTGCCTCCTGGTGTAACGCAAATTTaTGTAGAAATATATGATGAATGTTCATTTACAATGGATGAATTAATCGCTTGGGGACACATAGAGATTCCCCCTCAGGTGATTCAAAAAGGAGAGACCCATGAGG

>contig00872

ACTTTTCGTGAATGCTAGTGAAtGCGTTGGTCGTGGACgCAATTCTCTACCGTTCTCCGGAATTTGGTGAACAATCAaGAAGATTCTTGGGATCAATCACGTGCATTCCGTATAaTAACTAAGAGATGTCAGCCAAAGCGATCCGGGAAGCAACCGGGAAGGACTTAATTAATCGGAAGCTCACACCTGGGACAAAAGCAGCGAAATGTCAGTTTGTAACAATAACAGAAACCACGAACTGGCCACACGTGATTGCCGAAAaTCCTTGGTTAAAAaCCGAGAAaTTGGTTGTCAAACCCGATCAATTAATCAAACGTAGAGGAAAATTAGGTCTTATCAAGGTCAATGCAGACCTTCAGACTGTCCAGAAATGGATCGCAGAGCGAATGAACAAAGACCAGCAGGTTGGAAAAGCCACTGGAAAATTGAAGACTTTCATTATCGAACCCTTCGTTCCCCACAAACCGGAAGAGGAGGCCTACGTTTGCATTTATTCACATCGTAACGCGGATACGATTTTGTTTCATCACGAAGGTGGAGTCGATATTGGAGATGTCGACGCAAAAGCTTTGAAGCTTGAAGTTCCAGTTGGAGCTGATCCTCCTGATCAGAAAACTTTAATTAACAAACTTCTACAAAATGTTGCTGATGaCAAAAAACCAGTGATCGCAGAATTCATCCATTCA

>contig00873

ACGACAATTACGAACTAGATGtGAAAACTAAAGAGAATGTAACTCCAGAGGAACGTAAAGAAGAATCAGATTTACTGGACGAGTTTCTGAAGTCTGAGGTTCTTCTTGGTGCCATGAAATTTTTAGCAGAAAAAGGCTACATCCCCAACGACGAATATGAATTTAAAGACACTTTGAAGAGAATTTGGTTTTCTCAATTCAAGAGAATAGACGGGGATGCTTCTAGTTCTGGATTCGAGACAGTCTACTTAGCTGAACAATTCGATTCCG

>contig00874

aCTCTCTATGGATTGTTGAtAGTTTAGTAACTACTTTTTTAATTAATGCCAAGTATTATGTATTTGCCAAaCATaTACGAAGCTCAACAAGAACGTTTTGATTCTACATGGCCATCAAAG

>contig00875

TAAAGACTCTGGATTTGCCATAGAACCTTGCTACAGGTATTCCTtAGAaGGCCAAAAaGgTGCAAAAaTATGTGCAACTCGCAAATGGCTGAAACATGATAAAATCCCGTGTCTtGTAGGATGCATTGCAGAACTAACCGAAAAGGAGGAAGCTGCTTTACTTCAtCCTGGAAAAAATGATTTTttCAGTCAtGTTCAGCTGTAGGAAAAaTTGTGCTCAGCTTTGGTTGGGCCCAGCAGCTTACATTAACCACgATTGCAGGGCAAATTGCAAATTTGTAGCAACCGGTCGAGATACAGCTTGTGTAAAAGTCTTACGAGATATAGAAGTAGGTGAAGAGATAACATGCTTTTATGGCGAGGACTTTTTTGGAGATGGGAATTGCTATTGTGAATGTGAGACTTGCGAAAGACGTGGAACTGGTGCATTCGCTAGCCAAAAACCGGGAGAAGATCAATCTTCTGGTTATCGTCTTCGGGAAACTGACAATCGTATAAA

>contig00876

TGGAGAGTTTAGATTAAGTCCTAAAGCTGTCAAGTCTTGTCCTAAGGCTAACgAAaCGAGATTtGGATCCGTTtCtGCTGCTCTAATAAACGTCAAGAGCCCTACCATTCCAAATTGATCTTtGACCATACTAGATGGTATATTCGTTACTTTACCATCAGGAGAAGTTTGGATgCCtCTTTTAGAAGCTTGCGACTTTTCGGATCCTGGAGCTCTGTTaGCTTGCAGGATGTCTTGACTAATTTCTGGACCAAGGCCTACCGACAATGATTTTtCTCCAGGCACACCACCTCCAGGAGACGGTCCTTCCcTACTTTGAGTTCCTGGAAGGGCaGGAAAATCTTCCGAGCTCAT

>contig00877

ACCCAAATCCTATGAATCCTGCTGTAATTGGGACGGACGTCATTGATAGAAAAGTAGTAGATGGAGTTTTACACAcGCACAGACTAGTCTCTAGTAAATGGGGCTTTCCAAAATGGGCACAAGCTTtGATTGGCCATGCAAGTATCTGTTATGCTAGTGAACGAAGTGAGGTTGATCCTAaTAACCGAGACATGATTCTCAAGACGaGAAaTCTTACCTTCTGTAACTACATTGcGGTTGACGAGACTGTAAGATACTTACCTCATCCACAAGATGCGGGCAAAaCTCTGTTAACTCAAGAGGCCGTGGTCACGGTGCAAGGCGTTCCtCTTACTCACTACAtGGAaGACctCTTGACTTCGAGAATCTCCTTCAATGCTGGAAaGGGAaGGCAAGCTATGGAGTGGGTAATTGACAAATTAGAGTCAGAAGTTAAAGACTTTGCAAATAGCGCAGTGAAGTCAACAGATGAaCTCTtATGTCAAaCGCGACGTCAACTTGATGATCTGACATCAAAAACAAAGCGAAGCTTGGATGATCTTCAAAGCAGTGCAAAGAAATCTTTTGACGGAATACATAATCTAACAACACCACCATCATCTCAGTCCATGCCCAAGTTATAGCGCTGGTTTCGCACTAGTTTCAATCTATTTAATTAATATTAGCAGGCTTCCTTGGAACGATT

>contig00878

TtAGTTTGCTGGAATCGGTGgAAGAGACAATATTGGATGgAATGCCAGGCTTTGCGAGCGAAAGTTTTtGATtCAAGTCCAG

>contig00879

ATTCTGCCCACTTTGAACTGCTTGAATCGTtGTtAAA

>contig00880

CAACTTCCGAACATCATACGGTTGCTCATGTCgCCt

>contig00881

ACCTTATTtGCCAGAAGCAttGAGAAtGtAtCCGGAtGCTTtGCCTTCAAGGCTCTACGCTGAAGGGTCTGGAAGAAACATGAGCCTTTTtCTTCtCATGAAAAGGTATGATACTACTTTGAAACAGTATTTGAcTAATCGAAATATTTGCACGAGAGAGTCGATTTTGCTGCTGGCACAATTGCTTGAAGGAATTGCCCACATGAATGCTCATGGCATTGCTCACAGAGACTTGAaGAGTGACAaCATtCTTTtAGATTtATCaGAAGAATCTGACAATTGTCCATCGCTGGTAATCACAGATTTTGGTTGTTGCTTGGCAGACAAAAATCATGGGTTATATCTACCTTATAATTCACACGACATGGACAAGGGCGGAAACGTAGCGTTAATGGCGCcTGAGGTTGTAaCAGcGGTGCCAGGACCTTTTACAAACATAAACTACACGAAGgCAGACCTTtGGACTG

>contig00882

tggATATGGAAACCAACACGGTGGCTCTTTTGCATATAGTGGAATTTCATCCCCAGGTCTTGGACATGATTTACATACTAGATATGGAAACCAACCCAGTGGCTCTTATGCATTTGCTGGAACTTCAAGCCACATTAATTCTCATAGATCATTCGGAACTTCAACCTCGCATGCACACAGTTTTAACTATGGTAGCCAACCATTCTCAAGTTCTTCTACTGTTACAATTAATGGAAATCAACAACCCGAATCTACTTCAACTTTTACAATTAATGGAAATCAACCAACATCTACTTCAACTGTTACAATTAGTGGAAATCAACCAACTACTTCAACTGTTACAATTAATGGAAATCAACCAACATCTACTTCAACTGTTACAATTAATGGAAATCAACAACCCGCACCTGCTTCTACTGTAATTATTGGAAGTAATACCCCAGTTTATAAAGATCAATCATCTAAGGCAAAGTTTAAaCGAATGGTATCTGTTGGTGTTATCGCAGCTTTAGACAAAAaGAGCCAAAAGTCTTAAACTTTTACATTGTCGTTTTATGTTTGCTGCAGGTTCTGCAGATTTGCTCTTAACACGCATGTTGTATAATTATAa

>contig00883

ACCGtCCATGTAGGCATCGACAATTACACTCAGAATCCCACCTTTTGGCATACACGGCTTTCCGGTTCTCGTTCGACCACC

>contig00884

TGATGTCGTTGCTCAAGAGAATTAAaGTAGACATAaTaTAaTCCAAGtGACTTCTGTGCAAAGGAATGAAAATTAACGGCAAACCAGTGTCATTTGCCTCTTTGAGGATTTCAATTTGAGAAGGTTG

>contig00885

AAttCCtGTTTTCTCTTTACAACAGCACCAGTATTGTAAGCAGTATCGATCAAAATTGTTGACAAAGTaTCCGGCAAATGAACTTTGGTGCCCTTATCTTtCGCAGCTTGTAGTAACCAATTCATTCCcAAAAaTAATGACCGCTTAGCTGTCACTGGATGAGGAACCTGGTAaTAAACACCTCCTTTTCTTAACTTAACTAGTTCTAAAACGGGCATGGCATTATCGACAGCTCGATGTAAAATTTTTACTGGATCGAGTTCAATTTCtGATCGTTCTTGAAGAGTTTCTGATTTATTATATTTCTCTAGCTGAATCCTCTTTATCTTTGTAAATGTTTCGTGCAATAACTTTCGAGCTAAAaTTTTTTCCCCTCTCTCATGATATAATTTGTAAATTTGCAAACTTTGGTATCGTGAAATTCGGAGCTAGTTTCATTTGTAAATGCAGGCTTTACAGGTTGAAGAATTACGTTTGCCAACTGTCCAGATTTCTCAAGCTCTTCTTGATCACCTTTCCTATAAATTGGTTTTATATACGTGGATGGAAATACGCTGT

>contig00886

ACCTtGtAACTTCTCCTTTTTttAAACTCTTTttCCATTCACGACTTGAATTCATGTCCTGTGAACAAGAGGAACAAAAAGCTAAATGCGTTGCTTTTGGTGATTCTTGATCTGTTATTCATTTATTAAATCAACGTGACTTTttCACTGTGAGTTCGCCAGAGTGAATTTAAAGTTGACCGCAGTCTTCAATGATGATTTTCTTTGTGGTTTTtCCATTTGGAGTTCCGTAACTCTCCAGTTTTTTAACTACGTCCATACCATCGACAACGCGACCGAAAACGACGTGCTTGTCGTTGAGCCACGAAgTCTTAACAGTAGTAATAAAAAACTgAGAGCCGTTaGTGTTTGGCCCAGCATTTGCCaTAGACAAGATTCCAGGTCCGGTGTGTTTCAGCTCAAAATTCTCATCTTCGAACTTTTcTCCATAGATGGATTTTCCTCCAGTGCCATTATGCTTAGTGAAGTCTCCTCCTTGCAACATGAAaTTAGGAATCACACGATGGAAaGAACTTTtCTtGTAaCCATAaCCGTGTtCTCCTGTGCAAaGAGCACGGAAATTTTCTGCCGTCTTGGGGACGATATCGTTGTTCAACTCCATGACAATACGTCCCACTGGCTCGCCATCAGCGGTCACGTCAAAGAAACATCTTGGCAAACCCATTTTGGTTGGTTTCTAGT

>contig00887

ATtATTAaTtcAAaGTTATAATAAaTTGTCAACATGtGGAACAATTtCGATTCCGGTATGCACTCCTCAAACGCCGGCTACATGGATGATAGCCGAATGGATACAACTATTGATGATGATAAGAAGGGAGGTAAAAGGGCAGATAACCTCGTGCCTGTAATgATTGGACACATAACTTCTTGTAACCAGGAACTCCTTTTACATGGTTCACAAGTCCGAGTTGTCACTATTGTTGGAATAGTTCGTAATATTGAACAAGTCACCACAAAAATTACTTACGTTATAGAAGATGAAACTGGGAATGTCAAAGCCATCAAATGGAATTCTCCAGAGGACGAAGCCGAAGGAAAAGAACTACCACTAAATATTGGCTCATACGTTCGTATCTTTGGACTTCCTCGAGAACAGGATGGAGAGAAACACATTTTACTTCTTCGAATTTGTCCTATAAGCAGTTTGAGCGAATTGGTAAACCATCTGCTGGAAGTTGTTtATGTTGCACAaGCTGCTGCTGCACGCCATCCCATTCAGAAAATTGGTCCTGTGTCTGACACGACCTCGGCTATGCAAAACCTTGAATTCTCCGGCATGGACAGAGATCAGGCTATTGTCTTTAATTTCATAACAAGGGATGAATCCGAAAATGGAATTGAAAGGGATGAAATAAAGAGGAAAGT

>contig00888

ACGACCTTTTAATGTCTCTAGAACCAAGAGCATGCGTGGGTTGAATCCTGAGGATATAGATCAACTACTTACTATTTCTGGGATGGTTATTCGAACTACTAACCTTATTCCAGAGATGAGAGAGGCGTTTTTCAAGTGCATCATTTGTTCCTTCACCACAATGGTCGAAGTCGACAGGGGTCGCATTGCGGAGCCTACTGTTTGCACTCACTGTAATAATAATTTCTGCTTCTCTTtAATtCACAACCGCAGTCACTTTTCTGaTAAGCAGATGATCAAATTGCAAGAGTCTCCTGATGATATGCCTGCTGGACAGACTCCTCACACAGTTTCTCTCTTtGCTCATAATGATTTGGTGGATGGTGTTACACCTGGAGATAGGGTTGCTGTTACTGGAATTTATCGTGCTATACCGATG

>contig00889

TTGGCTTTTTttCTTTAAATTGTAAAaTTTTttCATTTttGCAAATTGTAAAaTTGTATTAATGCAATACACGCAGTTGTTTGACCGTTGACAACAAAaTTTCTtCACGTAGTGTGTTAAGGCAAGTGCAAATATTGTTGTGTGCTCTTAACGATAGCGTGTCTAGTTTAGGGACAAATTACGCCGGTAATTATTCAaTCTTTTACAATTTTATTGGAAAAGAACCAAAAAATCAAAGTGTAATTTAAATTGAAATTATTTGCAAAGTAGGGAAAACGAAGATCTTGGCTAAATATAGAAAGGTCGATAGTTtaTGATTTTTAAGTAAAATTTACTtgATATtCTCAAACATGTTGACCTTCCTATGAAGAGCAAaCACGGTTTGTCGTGCATACAAGTATTTGATAAATGTATTGATTTTTCGAAATAAGAACGCgaGCAATATTGTGTTGCCGCAGTGCCTAGAAACTTCtCGAATTCCCCcTTTATATTATTTTTTtCtAAtCAAATTGAGAAGTGACTAGTAAAATGTGtATTGCGTTTtGTGCACGCGCATAGTAGTGTAGGTAATAAAAAATTGTAAATGATATTTTATAATGAGACGCGTTTTCTTCTCGCTGATAACGCACCATTTTTTCTCAGTTATACGTGCTCCAAACGATATGGGCAGTTTGGCTGATCT

>contig00890

ACCCAGATAGAATCACATTAATCCGAGGAAaCCACGAGTCAAGACAAATTACACAAGTTtaTGGATTTtACGATGAATGTTTACGAAAaTACGGGaGCAATACAGTGTGGcGTTATTGCACGGAGATTTTTGATTACTTATCTCTGTCTGCTATAATTGATGGAAGAATATTCTGTGTTCATGGTGGATTATCTCCAAGTATACAGACACTCGACCAAATTCGGACTATAGACAGAAAaCAAGAGGTGCCCCACGATGGACCAATGTGTGACCTCTTGTGGTCTGATCCTGAAGATACTCAGGGTTGGGgTGTATCTCCTCGCGGAGCTGGATATTTGTTtGGTaGCGACGTCGTAGCACAATTCAATTCTGCTAATGATATCGACATGATCTGTCGGgCTCATCAGTTAGTGATGGAAGGATACAAATGGCaTTTCAATGAAACTGTATTAACCGTCTGGTCTGCACCTAATTACTGCTATAGGtGTGGTAATGTGGCTGCGATTCTAGAATTAAATGAACATCTTCAGAGGGACTTCACGATATTCGAGGCTGCGCCACAGGAGAGCCGAGGGATCCCGAGCAAAAAACCTCAAACTGACTATTTTTTGTGAATGGAAGGATATTTTATAGCCAATTTTCGTGGcTTTATGGACTCTAAAGTTTTAAGATTTTCAGT

>contig00891

ACTGAAGGATTGGTTCGAGGTCAGTCTGTCTTGGACTCCGGATACCCCATTcGAATCCCTGTAGGTGCTGAGACACTGGGACGTATCATCAACGTCATTGGTGAACCAATCGATGAACGTGGACCCGTGCCCACTGACagaCTCGCCTCCaTCCACGCCGATGCTCCTGAATTCGTGGAGAtGTCTGTCGAACAAGAAATTCTCGTCACAGGAATTAAAGTCGTCGATCTTCTCGCTCCTTACGCCAAGGGAGGAAAGATCGGTCTTTTTGGTGGTGCTGGTGTAGGCAAAACTGTATtAATTATGGAGCTGATTAATAACGTAGCCAAGGCCCACGGAGGTTACTCTGTCTTTGCTGGAGTCGgAGAACGAACTCGAGAAGGTAACGATCTTTACCAGGAGATGATTGAGTCTGGGGTTATTTCCCTGAAAGACAAGAGTTCAAAaGTATCGCTCGTATACGGACAAATGAATGAGCcTCCTGGCGCTCGTGCCCGAGTTGCCTTGACTGGTTTGACCGTCGCAGAGTATTtCCGTGATCAGGAGGGTCAGGATGTGTTGCTCTTCATCGACAACATCTTCAGATTCACCCaGGCCGGTTCTGAGGTATCTGCTCTActGGGTCGTATCCCCTCAGCCGTAGGTTATCAGCCAACTCTGGCTACTGACATGGGT

>contig00892

GACGATGAAATAAAAGTTTGGTTATGATAATTACAATAATAAACAAATAAACTTACTATTCCATATTGACAGT

>contig00893

TTCACGACGTAGATTTTtGCTGAATGACCTTAGGACTAACGCCGAAGCTTGCAATTTGAaCTAAAAaGGCCTCAATAACCTTCCATTtCATTTAGCCGTTCGGCTTTACCAGCTTGAAACACTGAGAATGAGCTTCCAGGATTAACCGAACACGTTCGGTTACG

>contig00894

TCTGCGGATCATtAATGATTTCGTTTTTTTtCtAATGGGACGATTTTTACAGCATGCAATCGAGGAACCaTAGATGtGGGAGGATAAAGAGTTTGAGCTTCAACATCATCAATATACTTTTCAGAATCACGGATCGAAGCGTAGAGAGGATCAaGAGATAAAAAaCctATGAAGTCGACAACTtCGTTC

>contig00895

CCATTATTTCCTCATCAGTTGTTTCAAGAGATCTTTTACTAACATTCTTTGCAACGTTACTAACATAAGTAGaTGGT

>contig00896

ACCAGTGTTTACCCATCAGCGGATCTTTCtACCTTTtCTtCCCAAGGTGATACGACACACAGCGGCATGGCGTGTGATAATAAAAAaCAAaTGTTCCCTCCAAAACTCGAGGAATGCGGGAACGTTTCTAGCAAAACGCCGCAACTCGCTGAAAGTCCCCAAAGCAGTGGCGAAGTTTTGAGTAGCCCTCGAAGTCCAGATTACCCTCCAaGGGTTTCTAGTGATCATAACCAGGTCAGCACTTTTATCCCAGTGAGTTCATCGTCAATGCAaCCTCCAAAAATCTCTATGGATACTGCGAAaCTTTCAGTCACCAcTATCAATaGTGAGATTAAAaGTTTCCCTTCTCCTTCAGTTGATAAAATTATTTGCTCTCCGGTGAaTATCATTGAATTACAAaCAAGCGTCAAAatGGAGATTGATTTGGTaGAAAGCGTCAATaCCAAAGTGGAGAATACAGATGGAATTTCAATTAAATC

>contig00897

ACTGCAAGGGCCACTTGGTAAAaTaTtAATTTAGCATTTTTCTCTGaCAAACCGCTTtGAGTTTGCCGTATGCGATCAAACAATTCCCCACCTTCCATAAGTTCAAGAACGATATAAACCGAGTGAGGTGTATTGAAAATTTCCTCTGTTTTGATCACGCAAGGATGACGCAaTTTTTtCAAAATCTCAACTTCATTCATAATCTTTTTtGGATCGTGAGCAGGATTGCTATTCATTGTTGTCAGGCCTTTCTTAGAGATGATCTTCATAGCGAACCTTTTGCAACCCACCTTtGTGAAAACAAGTTTAACTtCCCcACAAGCTCCAGATCCTAGTTTTCGCGAAACAGCGTATTTATTTTttATTtCAGGAGGCAAATCGTGTCCTTCATATCCCTGAACATTCATAAAAATGTAAACTGCAAATTTTGCCTGTGCAAGGGAAATGACATCGTTATTTtCAAGGACGACCCTTCTGTTTTTTCCAACTTTtGTTTtGTTAACAAAAGTCCCATTTTGACTTAAATCTTCAATATAGATAACAATGTCCTCGACGTTATTGGACCCTTTAATTCTTTCTCTTGTGATCTTAAAGTGCATCTTAGATATGCAATCCACTATTCTTTTTTCCAAACCTAAaTGGGTCACACTTATTTCGCATGATTCAgCTCGTCCCAGTG

>contig00898

ACTATAGTATAATTATTTAATTTTAAACAGACAAATAAATTTGAATTTGCTTTTGAGAAAAACTCCAAAAGTTCTTTATATTCAAGGATTCTTTGCCGTCTGATATTCTGTTTGTTTCCGATTAAGAGTAAAATAAGAGTCCGAAGGAATCCCTTAATGTCGCATTATTGCAACTAAAAGATTTTAGTTTttCtACACAACTTTTGTATCAAAAATATTTTCACGtAATTtATTTCGGAACcTAATGAATCGATTGAAAAATATTTCGCACATGATAACTTATTCTTTTTTtGATTTCGACAAAGTTAATCGCATGAAAaTAAACTTTATTTCTCTaGGAAAaCTAGTGAAATTGGTGCAAAAATTCCGAAaGGACTTATCCGTTAAGTAACAAATAGTAGGTAAATGAATTATGCtaTTTGACTGAATACCGTTAGAAATGAATCTTTCGAAAAGCAAAATTCATAGTAGTTGTAACAAGAAgaGAATTACACCGGAGTTGCTCCGCGAAATTGGTTACATACACGAGGTGGCCACGAGTGGGTAACAGAATAGAcTATGAGTGCAAAGAAGACAAACTTATGTGAAAGCATATTGCTGTCATACTCGCACTCATAGCCCTATCGTGGCTACTCTGTTTATTCATAGCGTTTGaCATTGCGtCTTTTtaaaTACGT

>contig00899

ACTTATTAGTTTGTAATATCCCCGATTCATGGTGAAATTAAATTTCAAGGTCAATTTCGATATGTTTGGACTCGTGAAAATCAAACTCATTATCACTGTCTTGCCTTGACGTTGAGCATCGGAACCACGAGAAAGCGAACTTAATTGCTCAAAACCCTGGCCTTCTTCAGTCTGTATTAATGGATATGGACTGGCGTAGAATAATACACtGGATTTGTtAAAGAAGACACTTGTATCATTTtCAACGGACCTTTTtACTCgATCGGAGCGACTGTAAATACAAGATCTTCCAGTAAGAGCTGCGACCACGTTAGGGCTGGATTCTCGAAGTGTATTATAGACTTCTGCAATTGATTCAAaGTCTGTTGCCTCAATTATCTCTAATTGATTCTCACCATCTGTTGAGTTGTATGAAGGCAAATTTTCCAATACAGAAAACGGAGACTCGACCGCTGGGAGATATATTGTG

>contig00900

CTTGAAAAaCTGTCCGGCAAAaGCAGTTTGCCGGATTCTCCGATTCTCCACAGCAAATCAGTAAAACCCATTGATCAATTAGCTGCCAACTTAGTGAGGATACAGTCTGAGAAGTtAGAAAAGCCCACAAAATCTCTTGAGAAAaTTGCaGAAAATCTTGCGAAGTCTAACCAAGCGGCCTtGAACAACGGAGAAGATTtGCCTCAGGATTACTGCGCTCGTAGCTCAGATAAATCCTTGAGAAGTCGAAGAGGTATAGACTTATCGACTTCTCCTCGAGGTTGGGATATGGCGAACGACCATAACAAGCCAGTCGATTTCTCTGGCAAGACTTCCAAACCTTCAGATCTCTCAAaCTATAGATCCCAGGACTTtCAACACAAGGAAATGGATCTgaG

>contig00901

TGGATCTACATCATCGTCAGTCGCAGATTCCATACTGTA

>contig00902

GGAGATAGTTGTAAGTTTCTtCACGACCGTGGtGACTA

>contig00903

ACTTGCAATCGTCATTCTGTATTCGCACAATTTTACATTTTTTAAAATAATTTATCAATTATCTTAAAATCATAACGCGTGCCCCGTTTTCGTATGTTggACAAACATAAGCCAAGTGCGCTAT

>contig00904

ACTTTGCCACTTTACTCAAGATTTTTCCTCTAGAAATAAAATACCTGGAAATTTGATCAAAATAGGCTGCAGCTTCACTCTCCACAGCTTGGACTTCAGCAAGGGTATCTTCTTGAATAGAGACGCCAAAATTATTTCCATCCTCTATTTTAGGAATCATAAAAGAGATCCACATTTtCAAAAGATTCGAATCTtCTAAAAGTTGCCTAATATACGgTTTTACTATGTGAATTAAATCGCAGAGAGGTTTGTTGCATGGAACTAGTCCATTCGGAAGTATCATCAcTTTtGTGCCGGAAACATCTTCTAATCTATGAtCTAATTtGACTCTTTTCATCGGTGGTCcATCCTCGGAATGATTAAGGAGTATCGGATCTGGAATAGGTATATTCAATTCTTGATGAACTTCCGATAATTCACGGTTACGAAaTCCTGGTGTTTCTAATAGTTCATTTAACTTTACAaTCTTTTCAGGAAATCCTTTTATAATGAGTTGCTCAGCCTTTGACTTTAGCGAGTCTTTAtATTCTTGAACC

>contig00905

ACCTGATCGAGGTTATACCCGACACAACGCAAGATTTCCTCTCTCGAGATGTCGACCTTCTCGTATCACAAATAAAAGAAAACCTGAGGCTTtCGAAtCTGAAAGCCAAGTCGAGTATCAGCCATAAAAACCGGCCATCGCCTTACCGAaTACCATCTCGTTCCTGGGCAGATCCTGCGAGTAATTGTGAGATCtGTGGAATGAGAAACTCGACCCAGCACGAACACCATCGACATCAGCTTCATCGAAAAAGGACGACGGCCAGGGATCAAGAGGATGATCCTTATGAATTATTACAGGAACTCCTTAGGgAAGGGgTTTGATCAaGGAAGCTGTTAAAaGACTCCAGGAGAATTTTGACGATTTTGATAATTCAACTGTCGAGGGATCCgATGATGAGGACGAGAATGATGAAGAAAaTGTTAGACAAAGGTTGACAGCCTATCAGAGAAAACCTTATTTTTACGACTCCGAGGACGAACCTTTACCTCCGGTTTATCAATCCTTGGATCTGTGAGTTTAAGTTTTTGCAAATTGAGACTATAATTCTGAAATTAGGTGTTTAAGGATGTGGGGAAGTTGGGTGGGAGCGGATGACGAAATATTGCTTTCGGCTTTCGCCTACTTGGCGAGTGAGAGTCTATACACGGAAAAGGACTTGTGTTTGTGAGGCTT

>contig00906

AAAGAATCGTTTCCAAAACGCCGAAATATTGGCCTGCTGAAAAAATCTATAGTAAAGGTTTTCGAATGTGATTTTTGTAAACATTCTTTCTACAAGAAAAGTCAACTACGTTTGCACGTTGCTACACATCGAAACATATtCTTTTGCGGTTCTTGCGAAGCACAATTtGAGAGTTATGTGGAGTTCCAGgAACACGAGAAAaGTCACATTCCTTtAACCACTGATAATTATATTCAtAAGCCTTTtCGTCTTGaGCTAAATAATTCGAAAGAAaTTCAAGTAAAAAaTaCTCCAAGAGAGGAAGTAGAACTGGTAAATAAACGAGATCCTCGAGAAAaTGGAATtGAGGATAAGAaGAATCTTGAGTCAGAGACACCAGACGAAAtaTTTTTTACAGAGTCTTCGATAAAAGAGGAGAATGACATGGTTtACcTCGACGGTGATTTGACGCATCCGAACTTCA

>contig00907

ACCACtCTCATCCTGGCTTCGGTTGTTGGCTTTCAGGTGTCGATATTAaCaCGCAACAATCGTTTGAGGCACTCAGTGAAAGAGCCGTTGCTGTTGTGATCGACCCcATTCAATCTGTGAAAGGCAAaGTTGTTaTTGACGCTTTTAGATTAATAAATCCTAATATGATGGTTTTGGGACAAGAGCcTCGACAGACGACCTCTAATTTAGGTCATTTGCAAAaGCCGTCCGTTCAaGCTCTTATCCACGGTTtGAATCGTCACTATTACAGCATCAGTATTAACTACCGGAAAAAcGAATTAGaGCAGAaGATGTTGCTGAACCTCCACAAAAAaaCGTGGATGGATGGTCTCACGCTTGCGGAGTATTCAGAACACAGCAAACTCAACGAAAATATAGTTTCTGATATGTTGGAGCTCGCAAAAAaTT

>contig00908

ACTtCCTCATATAAATATTTTCAATAGGTTCAAATGGTGCAAGAAATGTTTTCAGTTTCTCTATTGTCATTCCCTGAAGTGGAAAACCTTTAGCGTAAaCTGTCCTGGCTTCTTGGgCcTTCCtGTATTCTTCATCGTAGACTGGAAGTGGAtGATTAAGGGAACGACGAATTTTTTtCTtGTCTTCGGAAaTCTCAaTAAGTTCACTAGATTCAAGAGCTTtAACAATCGTGTCCACATCTTGGCTCAGCGATGCTAGCATTTtAAATTTCAACATTACCTCCATTGGAACCCAACCTTCGCTTAATTTAGTCTGCTCAATCAAAAaTTTGTCTCTTTGCATATTTACGTCTCCAAAGTAAAACTCG

>contig00909

ACCCAGTTATCAGAGACAATGTAGAGAATGATCTGGCGACTTGTGCCCATTGCAATTACAGCTTTTGCATTTATTGTCGTAaGgTTtATCACGGAGTCTCTCCCTGTGAAATGTCTAGCAAGGAAGTCGAAAATATAATCCATCAATATAAAAGTGCATCTCATAAAGGG

>contig00910

GTtCCtAGCGTGTAGAAACTGAAACTTGCCAAACTCTAATGGTGTTATCGGAGTAACCGGCAAATAAAGTTTGTCCATCAGTAGACCATGCCAGAGACAAACATTGGGGAGGTTCGGCTTTGCTCGTTGCTGAAACAATTTCTGGTTTCAGTTCTTCAACCATTTCTTTGCTTTCAAGATCCCAAATTTTAATGTAAGGTCCGAAaGCGGCACATAACCAATAACGGTtGGgACTGAAGCACAAAGCAGTTATGATGTCATTGTGGTCTAAAGTGtGCAAGTGCTTGCCATCGTTCAAGTCCCACAACATAGCTTTGCAGTCCTTGCCACCAGAGGCGCAGAGGGAACCaTCTGGAGATACAGTGACGGTATTCAAGTATCCAGAGTGTCCATTGTGATTTAtCTTTAGTTTGCAGTTAGTTAGATTCCACACCTTCACTACTCTATCCCAGCCAGCaGAAACGATGATGGGATTCGAATGATTGGGaGAAAAaCGCACGCAACTGACCCAATCAGTGTGTCCGTCAtCTTGAATCGTGTATTTGCACTCTGCCAAAGTGTTCCAGAGCTTAATAGTTTTGTCTCTTGATCCAGAAACTATTTGACGATTGtCCACTGAAAAaGCCACGCTCAAGACATCCTTAGTaTGaTCTTCGAATCTTCTAGTAGT

>contig00911

CGTCCTtAGCTAGAGACGGAgTAaTTGATGAaCCTAATTTGACTGTAGAGTAtATTGGGACAAaCTATGGT

>contig00912

AACtGCTCGTCAGGAAATG

>contig00913

ACTATGGCATCCAACTGCCCTGAACGTATTGCTCACAGCCGGCTCCGACAATTTAGTTATCATTTGGAATGTTGGAACTGGCGAAGCCCTAGTGCGAATAGATTGCCATCCAGATGTGGTTTACTCTGCGTGTTGGAACTGGGATGGATCCCGACTAGTCACTACGTGCAAAGATAAGAAAATCCGCCTCCTGGACCCGAGGTCAGGTAAAATATTAGAAGAAGCTGTTGCACATGAaGGAAGCAAAGCCACGCGTGCCATTTTTCTCAGGGgAGGATTGATTTtCACGACTGGCTTCAGCAAAATGTCGGAAAGGCAATATTCATTACGAGCTCCTGATATGTTGGCAGAGCCAATAGTTATGGTAGAACTAGATACTAGCAACGGAGTAATGTTTCCTCTTTATGATCCTGATACGAATTTGGTTtACTTGTGTGGAAAGGG

>contig00914

AATAATCCTTGTTGGTGTCTACGGCTATGAATGTCAGCTAAAATCTTATAATtCAGTGAAAAaGTGAGGAAAAGTTGTGCAATATTATGTGATAAATGAAGTTTaGTAACT

>contig00915

CCTTGATGAATAAAaTGGACGAGGCTAGATA

>contig00916

AGAGGCAGCCATTGTAAGCATATGACGCCGCGTATCGCACTTCATTATTATTTTCTGAACTGTTTCTTTGCAATTACCATATTAACGAGCTCTGGATTATTGTCTAAATCTTCTTTCTTCAATTCGGCGCTTTATCTGTAGATAAGATTTTGAAATCGTAGTTGTTTCAGTGTTAGTTTCTTAACAGAAAAGGGTGACTGGATACAATATTTATCAGTGTTGAATACCTTTTCGACTAATTTTTAAACACCTGCATCAATATGAGTCAAGAACAAATTAAAGAGACAACTCAAGCTGCGGAGATGACATCGAATGATATTGAAAAGATTGAAGAAGCAAAATTAAAAGCAAAATTCCCTAACACAATTAACAGACCAATAAGTGGTCACTCTGCTTTTCTACAGAAACGACTAGCCAAGGGGCAAAAGTATTTtGACTCTGGAGATTACCAGATGGCAAAACAAAAaTCAGCAGCGAAGCCAAAaCCAGCTGGGgTTTtgCCAAcGGgCGATGCTATTCCCaCaCCTGAaTCTG

>contig00917

CCTCTGATCGCTGAACTACCTCGCATTATCCTTGCTCGAGAGCGAGATAGTCCTGCTCGGCTTCCACTTCCACCACCTCCGCTtCCACTT

>contig00918

ACTTGTTGGCAAATAAGGATAAAGGGTCTCGTTCTCTTAAACTAGGAAGTTCATTGAAATGCTGACACAGGCGTCATGTATGACGAATCGACGACCTCCTCTTGGACAATCTACTTGCCCATTCGttGAATAGACCATTCTTGCTGACAAaGGGGACAACGATTGTTTtGCTTGACCCAAAGTGACATGCAGCAATAATGGAAGGAATGATTACATTCTCCCCAGACGACCACGCAGTCCTGCCTTCCATAAAAGTCCTTTtACTTtCAGCTTGGCAGCGCAGGCAGGCATCCATAACTtGAACTCGACAAATGGCACAAGTGTCACATTCGACATCCCAGCTCCACATGGCTACAGCATTCCACTTTTtCAACGtGAATAGTTTATCAGATTTCATATTGTCACCGTCTCCGCGATCGGGATTATCTTGCTCTGgaTCTGCCA

>contig00919

AAgCAaTACTGATTTAAGAGACGCATTTTTGATTCTTGCATTGTCCaTGTGTATGCTTTTTGAAGTATTtGGTCACTTAGGTAGTCTTCCATTATCaTTCAGTTGCGTTGTCTAGTAGAAAaTAAAAAAAGCTTtGCgATTCAAGGTGTAGACGATGATGGCAACACAATCTGTGTCAAATATTtGAGTGTATAAAAaTCGATTGTTCTTctCcAACAGCTGTATATTATATTGTAGGTCCaTATTTGTATtGTAAATTATATTTTATGTTTAAAGAAATCGAAAACCTACATTTTGCGTTGCGCGcGgatATTAATtGTTGAAAGCACCAAaTGAGTTTAAATCATATCCTACAGTAAGTATGTAAGTGTCACTGTAGTTAAaGGGAaGGAAAATTGTATGTATACAAATTAAAATTaCATACTTGCAGCAGGTGATGTTTaTTCATATTAAGCTCTTCGCGGACGTCCGACGTGTATAAAaTTTTCTGTTTTCGTAGTCTTTCGCTTTGGGT

>contig00920

TGTTCTACAGGTTCTGATCCTAGAATACTGCCTCATTAAGCAtCTCTGCATGGTTGCaCTGCAAAAAaTGTTTGAAATTTaGCCCACTTTCtAGTGGGGAATGTCTCGTGAGAAGGTATATAGGCAAAATATTTTTGTGACCAAAATCGATCATTTTtCTCAGAATTTTGTCAAaTGTGCCGATAATATCAGCTCATTTCCGAAaTGTTAGGGAATATATTCTATCGGTTAAGAAaTTaGTCCAATATAATTTGAaTATATATCCGACTATAACCAGAAAaTTTTTCGAAAaGTTAGGGTTATTAGCTGGCACACTATTTtcGGAAATATTTTCGAAAAACTTTTTACAGTtCACATAaCTTGGAACTAACATTGAAACACaTGTGAGAACCCATACAGGTTTTACTTCTAAGCA

>contig00921

GCATCCTATGCAGCGACCCACATGGGTTTCTATCTATTTTACTTCACACGCACCTACATtATTTTTtAAATTTGATATATGTTACATATAATAATTATACAGAATTATTCGAAAAAGGTGCGCTTGCTCATTATTTTTTGAAATATATATAAATTTTCTCCTATAGAG

>contig00922

ATTCAACGTAGGTTTAGTATTGG

>contig00923

ATAACGTCCCAAAATAATTCTTCAAAGATCCAGGTTGCATACATTAGCACTCGTGAAATTCGAAAAAaCCGACCTCCACGTCCTGAGCCTTTTCCCTACAAAGAAAAGAGATTTGGTCTTTGGGCACAATT

>contig00924

TTtGATGACACAATATTCCGATTCGATGACAATACCAAGATTAT

>contig00925

AATTACAATTTATAAATTCAAATTCAAAATTTCTTTTTCATTATACAAATTAATAAATCATTTGATACGATTTTTtCGAAAGTCGTTGCAGATGTATGTTTTTGCACTTtGTCTTTTGCAAATGCCCATGACATTCACTGTAGGATCACTTGAGATGAAATGGAGAATTTtCACtGGATCGAAGTGTTGATAAAAaCTGGAATTTATACAGCCTtaGACACCTGtGTTCCAACTTATTtACATAaTCCCTAATCACGCTGTCCGGGTTGAtAGAGGAaTTAAaGTTTtaGGTTaTGtGAaCTAGTTtGCTCCAGGGTTCGTATTTTGtATGAGCGTTATaTTATCGCCCTtGAGCATTATACGGCCAAGTTGCTTCCTGTTTTtCGTTTtCACATAGAACTCTTCTGCATCATCTAGCACTAAGTTCATATATTCGTCGAAGCCAACAATGTGTCCTTCGATTCGTAGGTTAACGTTTtCATATAaCCAAACTtGAACGCGCGATCTGTTCTGCAGGTATCGAAAGATGAGGTTAATGGGTTGCACCATCACCTTTTGCACCTTCTGaGGTCcTTTGT

>contig00926

CGACATTCAGTGACTCGCATTTGTTGAGTATGCTCTTAATATTGGTTATATAACTATGATCGGGAGTGACCGAAGCAGAACGCGAAGCTTTCGAATTACTTCCGGATGACGAAAGACAGTGCGAAGCATGTAAAACGACCTGCTTCCTAAGTGCTGTAACTTGTTCGTGTCAGACTACCCAACTTGTTTGCTTGAGGCATTTCACTGATCTTTGCGAGTGTCCACCAGAAAAGCATACTTTAAGGTATAGATATACTCTAGATGAATTACCCATCATGTTGCAAAAGTTGAAACTCAAGGCCGAGTCCTTCGACTCATGGGTCACGAAAGTCAAAGAAGCAATGGATCCTAAAGGCGACAAAATTGAGCTCAGTGAGCTGAAAGATCTTCTAAATGAGGCTGAAAGTAAAAAGTTTCCTGAAAGTGAACTACTTACTGCCCTCACAAAtGCTATTCAGGACGCCGAGAAGTGTGCGAGTGTCGCACAACAATTGTTAAATAACAAACAACGT

>contig00927

ACTtGTTTTTAACCATTCACATAAAAGCTtATAaTTTaTAAAATACCTAACAATGAATTTTTGTCTTTAGAATAACATAGAGGGTAACAAAATTCTGCATTTAATGATTTAATTTTAGTCAATTATGAACGAAGATATTGCGAAAAAAGTCAAGATTTCAAAGTTTTTTAGAATTCTAAACAACTCTTGAGACAATGAACATTTTTGAACTTTTCTGAGCTTATTATGAAGGTAATTCACATAAACTATTAGCAACGTATCAAAAATATTTGGATAGATG

>contig00928

GTTGTGAGAATACAAAAGATTCTCATACTTGAAAAATCGATTTTAAAAATT

>contig00929

CATTAAaGACAAATTTATTATAATTTACTTGTTACATTACTGACATTCTaCGTAAATCTCATTTTGAAATGAGTCAaGAAAAATAAAAAAaTATGCATTATTTCAGTTtATTTtCATTAATAATGGACTAAAACATTTATTTTGCACAGCaGT

>contig00930

ACGATCCGTTGAACGATTTCTTGGGTCTGAGTTTTTTTCATTAAACAGGGCAGCTTTCGAAATTTTCGGTTATAATAACTAATAAAAGCAAAAAGAAGTTTtAGAAATTTCAAGATACAGGTTTTTCGATTTGACGCTAAATGCAGCTAATTtGTCGAATTGACATAATGCAAAATTtCgCGTTTGACGATTtGAAGAGAAGAATATAAAGGTGACgAGAGTTtaGAAATTTGTTAATTGAGAAATTTCGAtCGCTGCCCTGGAAGAaCGAAAGTAAAGCTCTAAAAGACAGTTTTTtGGTTTtAAGATCTTTtGTTTAGtATAAACAGCGAGATATGATAAAaGAAATTGTCTAAATGGAAGCATtAGAAGTTTTTTtAtACTaCGCACTAAATACTGTAAGTGTATAATGAACGACTACCGGTCGTTGTAATACTAATATATTATTATAATTACTTAATAATGTTAGGAAAGAAACTATTCGTtGAGATCGTTGTATTTTGtAAACCTGGAGTGGGACTTTGTTGATGTTTTACCTCGAGGCGATGCATACTTAACAAACCAAAACTTATTCAAATTTCACTCGTTCTGCCTGTCGTCGTTTGTATTTGCCTTTAAATATTTCAAGCTATAAATACCACACAATCTTCTATACTATTAAAATAGT

>contig00931

ACTCTAATACTATAAACTCGGCCGAACGAAaTTtCCAAAGGATTCACCGACTTTCTTCGTCAGAAAGAGACGCCTGCGATTTGGGCTTGGCATGTCGTCTATGCATTGATTCCATGAACTTtCTTTCGCTAGACCCTtGATTTTTCTCTCCGTCTACACTTAGATGCAAATCACACCTTATAATAACCACTCCAGTTGGATTCACCATAACTTTAATTATTGACGACGGCTTCAGGATGCCGTATTTCTTATTTGCATACAAATCCTCAACTTGGTAACCATCAGGATAGTTCAAGCCGAGCTCTTCCAAGCTAACAGAAAAAGCAGAGGGAGTTCCATCTGTTCTTCTGTTCAGGAACACTATGGCATATGAGTAATAATTTTCTTTAGTTGGGGTCAAAGGTTTTAGCCAAATTTCCTTCCCCTTGTCCTTCTTGAATCTTCGACCTTGAATTCCAAGCGGATCTTGATCAACAGCAATGATTTTTtCATTCTGTAAAATAGCTTTATATTTTTCGTCAATATGACGCAGATCAACTGACATCAAGAGAGGAGCAGCGAGGATGGCCCAAATGGCCATTTGAGCCTTTGATTGCTCGTAACTTAATCCGAAGTTCCCAATAATTAACATATCAGGATCATTCCAGTGTCCAGGTCCAGtGT

>contig00932

ACCGAGTATGATATTGAACTGTAGTGTAAGATAGAAAAAAaGATAGCTAGACACAGCGCCTGTGATGTAGCTAGATTTGGATGCACGCCGATTAAACGTATGCAGGGCCCTCGTCGCGCATAAAACTAATTTTGCGCTCCTTTTCTCAATCTTTtGAAGTTCTGTTAAATTAGTCGATCTTTATCGTCGgTGGTTTGATTTTTTCGTGGTTTAATTCTCTCAACTTTGAaTCCTTTTttGAGAATTTTTCGTTtGATACAAAATTGAAGATAAGGGAAATTTAAaTG

>contig00933

ACTTGCAATTAGCTCTTCTTGTTCTTCTATCTGTTCCTTCAGCTTCTCAACATACTGAGATTGCTGATTGATTTCCTCGTCTTTTTCGTCCAATTGTTGATAAAGTCTTTCCCGTTCATCTTCAAGTTTTTgCCTCTCTTCATtAGATAGAGAACCAGCCATAAGACCACCTCCAGGTGTAGCAGGCATTGGGCCATCGTCAATTTTTACTtCAACTGAAGCAGTAATTGGAGTAGTGACATCTGAACTCTCCACtAAaTTAaCCTGTTCTTCTGGTTTAACAGTTTCACCTTGCCGCCATCGTGACAATTCAGCTTCCAACTTTTCGACTTTAGCTTtAAATTTAGCTGCTTTCTCCTTTtCTCGTTCGTAGCGTCGTTTCCATTCTTCTGCAGTTAACTCTTCATTgaCGCACACAACGTTCTTAATAGTCTTGGCGCGTTTTCCAAAGTCTAGCGTGGATTTGGATTCAGACTCATTAAAaCTAGCTGGTGAACAACATATGATAATTGTTGTTCTAGCATTTCC

>contig00934

TAACATTACCAAGCGCCGATAAAGATTTATTTATATTTTTC

>contig00935

CGtATGTGTAGCCTACTCAAAGAAATCAAGAGAATCTGTTTCTCAATCTAATAACTTAGGTCACTTTACATTATATACTTGCAAAAAaaTTtaGAAaCTTAAaTTACAGTATACGTAAATTATTCCAAAAaTTAATGATTCAGTCTCAAAAATTGAAGGTGAAAATTGTGTTCATAAATTGAAGAAAgAAaTTGTTTACTTCTTTTCTTCGGACTTTTTCTCTTTACTCTTTTCCTTCTCGCCCTGTTTCAAAGCCGGCTTTCCAGTGAACTCTATCTTGACAGGTTTCTCACCCTGGAGTTTAGGTTGTTCTTTCCTTGGAGCTACAATGGTCAGGACACCATCCGAGGATAGATTTGATTTTACCTGATCGATGTCACACTGTTCAGGAATTAGATATTTTCTAACAAACTGCcTGGAAATCCAGCCATGTTCGTCCTTCTtCTCCTCGTGTTTGGCACTTACTACAACGCACCTATCAGTGACCTTTACTTCAATTTCTTCAGGTTTGAaTTGTTGGACATCAAGCTGT

>contig00936

TGTATCTCATTGCATATAAATATGTATACAGTGTCAGAATATGTAACTAGACGTGTCTATAAAATAAGTTCAATAGCTTTTCTTTTTGGCAGATTGAGAAATTTTATAATTTGATTCAAGTTTAGTGACTTTATTTTAGTGACAACTTCTATCTTTAGTTGTTCATTGTGCAAGAAaGACTCTTGCATTGTATCATTTGTCATCCGAAATCTACTCCGGTGTCAAGAAGATCCCTTAACATGAATGATGCCTTTTttGGCAGTATCCTTAAAATGTGACCAAGATAAAGCAAATATCCAGGTATGCTGAATTCCGCATAATtCCTACGCACACCGTCCAAAa

>contig00937

ATGCATGCATCAGaTAGAGGAGAATTGTTACAATAGCAGAGACGACGAATGGGTGAATATTTATCACCTTGGTGAACTTATCACCTACaTGTATTTTTTCCACG

>contig00938

CTGGAAaGGTTTtATGGCGATACCTCCTTTGTTTTtCTtcTTCAACGTCTCCGTCAGTtGTTtCACAATAAGATCTGTTACATGGTCAACGTGTCGACCCCCcTTTGTTGTTGCAATACTGTTtACAAAaGACATTTGTTGGAATCCTTtATCAGATAAGGTCATTGCCACTTCCCAACGCGCTCCGCAGTGTTCATAAGCGATTTTTAGAGGATTACCGACGTCGTCTTCTTTtCCTTTAATATAAAGATCTATGTAATCTTTAAAATTCTTTACGGGAACTCTATGACCATTGAGGTAAACTTTCACTCCGCCCGTAGAGGCAGCTACGTCAAATGCTCGACGAGACATAAGAGCTACGATGTCGTCATCGaGAgaTTCcATTTTAAATTTtGATAAaTCAGGCgaGAAAGTAACTTTTGTGAAATCTTCTCCGCAAAATTCTTTGATTCTTGGCTCGCTTGCTTTACCCATGTTGTTACCCCAAGTCTGTTTAAGACTCC

>contig00939

AACTtCAAAAAaTTGCATTGAAATtGCAGAACTTTTCATAAGGCTTACGTTATCCGTATGCAAAGACTTTtGAAGCCTAGGCATTACTGATAGAATTtGGTGCAGAAATAACTAGATTCGGGATGAaTCTACACGTAAAATCTAAAGTTCATAaCTTTtaTGACACGACGGTGACACATTAATTAAATGTCTTGTTCAAAGAGTCTGATAtCAACTCTGCTTTTGATGATAAGATAAGGATAACAAAAATTGCTAGAGtGAGATTCGAACCGATTAAGATTATCATAGTGATTGCGGCAGtCCGTTTCTCGACCTGCTCAAGCAGTTTTGACATTTAAAAGGACCCTTATGTTGTTAtGCATATGAATTTtATATCGAAATAATTTCATTaGTATGGAACATTCGAGAACAAATATGACACCGATTCGCCGTTATGTGTCCTCCTCACTGCTTCGGCGAACATCATCGAAACGTcAATGCACTGAATCTTAGGGCAGTCTTTCAtGTGACCATCTTGTGGAATTGTGTTAGTTACGACAACAGCTTCGAAGCaGGCATTATTGATCCTGCTAATAGCAGGGCCACTAAATATTCCGTGAGTTAAAATGGCATAAACTTTAGTTGCTCCTGCTtCcAATAATTTTTCAGCCGCGTGaCAAAtagT

>contig00940

ACTTATCATCTGCTCCAGATATCAAGTATGGCTTGTCACAACCATGATAGTAATCTACACAATTCACACCCTTTTCGTGACCTTCAAGTGTAAAGTTAGCAGTTGAGGAACCCAGCTGCCATACTTTTACTGTCCTATCCAAAGAGGCACTAGCAAAAGTATTATTGTCTTTTGGATTAAAaCAaTCTGCATAACATAATGTGTGTgCCCTTCAAAAaTTTGCTGACAAAGCCATGATTTTtCCCAATTCCAAAGTTTtATTGACATGTCATCACTACTGGTGaGTaTAAAaGATTGCGTTGGATGAACAGCAATACACCTCACATAATCGCTGTGTGCGTCGAaCGAGTGAaCTCGTTCTAAGGTATTATAATTGAAAaTTCgAACTTGCATATCATCGGAACCAGTGACAACCCAATTCTTtCTTGACaCAAATTTTGCAGCCCTTACAGGTAGgtCGCATACTTCAAAAGTTTTtACCAaaGTCTGCGTTTCATGATTCCAAATATTCACATTACCCTGATATAAAGAACAAAGCATCCAAGGTTCCGTAGGATGAAGGTCCACGCTCTTTACTCTGTCTGATCTTGCAGTCAATTTACGTTTGATGTCTAGACGTAATGGCATTTTGAGTgTTTgCGGATTtGTTTACTCTCCAGGAaTTCG

>contig00941

ACTTATTTTAATTAGTGCAAAATAAACGCCAGCTCCTATAATGTTTAAAGTGCGCATCATACTCGAGAAATAATTACTCAGCATTAGGAGACATCTTTTTTAAATTTTAACCATAAAATTTAGTATTAAAAATGCAATTTTTTtGAATTGATGTGCCTGAATCTCATTATGGTAGCTGGAATTTTAAACGTTGGGGCaCATTAAACAGTGAATGTTTATTGATGTGGCAGAaGAATCCGATTAtAAATTtGAGAAATTGaTTTTtATAGATaTAAGATATTTtACGGAAAaCAaTTGTTACCTGCACAAATTTATTtATTTTTTTtGTTtGAAAaTTGTTTTTtATtGACTTAATTtACGAATGCTCATGTGAATCAGTATTACAAAAaTGACCGTGTCTTGTTTCATTTTTGCGTTATTTACAAAGG

>contig00942

ACTAGtCtCTtGtCCAGCATATTTCATTTTTTGACGaGACCAGCCTAAAGGGAGAGTAACACTTCTTTCGTCGTTAATTATAATTTCGAAAaGAGGAGTCTCGTCTACAAACTCCTCTAGAACTTCCaGGATACATTCTTGTGGATGATTTTCTACATATGATTCAGTCTGCTGAGAATTATTTCTCACTATCTGTTTACTATTTTCAGTTTCAGGCTGAGAAtCATTATTATTATTTTTATAGTAAGTTCCCCCcAATTCAGTCTCTAAGAGATATtCCTTTgTTACAGAGCAAACAGGCAGTTCACGGTGTAAATTGTTTTTAGATTTCACACTGCAATTGTCATTTTtATCGCAaGTGTCCATTGGTTCGCTACTTtCGAGAGATTCTTCGACTTCAgAAGCAGAGGGCAGGTCAGTTGTTATTtCTGGAGTGaGACCTAAGAATAGGGATGGTAAaGACCcTTTTtGTAATCTAGGCTTTTTGTAATCAGCACTCACTCCTAGGATTCTACCATCAGGTGCCCTCAATTCTTTCCTCCAAAGGATATCTTCCTTTGAAAAATGTTTGTGACACACTCCTTGTCCTGCTTTTATGACAATATCTTTTCTCAATAGAGCACGTTGCCATAGTTCTTCACCATCTTTGGGCACTTGAAAAAaGT

>contig00943

TgATTaTCtAtATCAGCTAGGTTtGATAGATCTTAACGGTAGAGATGCATTTCATAGGTTTGAGAAGAAaGGTCGTGATTTAATTGAACAAAGAAAGTTTGATGAAGCTGCTGATGTTTTTgATACGCTTCTTAATAATGACTTCGATGAAACACCTTCGCTTTttACGAATTTAACTGGTTACAACTTTTACTTTAACTATTTGCATACGACAGATTATTCTGACTCAGACTCGATGTCCGAATGGATTCAACAAGTTGATATTAGACGTGCTATTCATGTTGGAAATtGCTCTTTtCAtACTGAAGATAAAAAaGTAGAAAAATACCTTAAAGGTGATATtATGCAATCAATGGCAGTGTTAGTGTCTGACCTTCTTGAACATTACAGAGTTCTTATTTATAATGGTCAGTTGGACATTATTGTTGCATAT

>contig00944

AAAATGGtcAGGTGCGCAAAAaaTaCAAAaCAGCTACGAGAAAAaTATGGAAaGTAGGAAaTGAAATTGCGGGGTATACAAAGACTATTGAtAACTTGACAGAAGCTCTTGTCAGAAATGCAGGTCaTATGGTCCCTGgAGATCAACCAAAATGGGCtCTTGATCTAATTACGCGATTCaCTCGTGGGAAACCATTCTAAACATT

>contig00945

ACTCGCTCGTtCAAAtCTtCtGtCtCTTTtGCGATTTtGCGCCTCTCTTAAACGATGATGTGTTTGGTAATCCAGGATACTGATAAGATTTTTGTGGATTTCTTGGGCCGACGATtCcATCTGAGTCATCACAGTtACGTGTTCGCCAATTCcAGGgAGAGGATGTTCCTCACCcACTTGgAAATCCATGTAGACGATTTtGTGGGAAAaCGTGGAAAATTCaTTACTAAAaCAaGCTTTGTAGACGCCTGACATTGTAGGTGTGAAAGTATGAGAATCAAATTGGGTCTTGACTTGACGATAAATGATTtCCTtATTGGGTGCTTCTAACGTGACaTCCACATCATACTGCCCTCCAGTCACCACCTGGAATTCTAGTGTAGATGTAACATtCTTTccTATTTCTTCGTAAAAGCATTCCTTGGCATTGTCTGGCAATTCGAAGGTTAGCTCTATGCTACCTGCACGTAAAATGGAGCCAAGGAATGAAACAAAAAGGAAACACGCAGACCAGTAGTCCATTCTTCACAATTCAAAAGTTCTTTTGTTAATTAAGACACATTTAAGTCCATAAAAaCACCAAGCACGACCTTTATTACCTTTGCTAAAGCCCGGT

>contig00946

CAACGTCAGTGAAAAAAaTGTAATCTACTGTAATCCACTTTTCTTGATTTGTCGTAGCTTCTAGTTCTCCATGTCGATTCACATGCTTG

>contig00947

TGTATtCTTTGGAATAAGATATTGAAATGCTGACACCC

>contig00948

TCCAGCAGTTCAGAAAAGAAAAaTACCGAAAAGTAATTGCATCTAAGAGAAATTTtACAAATAaTTAAAgtATGTATTAAAATCTTGTCCTTATGAAAAAGACTATGCTGTTTGCCAATTAGAGAAGAGAGAATGTTTTTACTCAAACTTTTATATCTGCATTTATACAAATGTCCATTAAATTTAATATTTTATAACTTAATGACATTTAAAATTTGTCTTTGGACTTGATGAAAAGGATTTTCAAGAAAATTGTCTAAAAAaTTATACAGATTACAATTGAAATATGCTCATGATAACATCAGCGAATGAAAAAAATTATTGCTATTCACTTATTTGTTATTAATTTTCGGTTAGACAAATATAATGTTCGAATATCAGTAAATTGTTTCTTTACCTAATTTCTCTTGAAaTTTGTAAAaTGTTGACCAATGGTGGTTTTAATATATATCAAGGTATCATATCAGCCAAT

>contig00949

GGAtATTACAGGCTTCGATACGTGATTCATAAGCTGACGCGAAGTTCTTTGCCACGTTTTGCTTTGAAGTTTTCTTCAGTTTATTGAAATGTCATCTATTAGGATTATTTTGTTCATTTTTTtGATTGCTTCAATTCAAAATATTGGTGCAAATGACCCATATATTGATAGCACTGTTTCTCTCTGGCGTCTGGACAAAGATGGAATGAAAGCAGAAACATTTTTATGCGTAGCGGCTTTAATTAAACCAAATAGCATATTTATTCTTGAACATTTtCTTAACACGCTCCAACCACTCAAAACCATTGGCTTTTACACTGGATCCGAAAaGAAAATTTAGGCAGAATGGAGAATTCtGTtATTGTAGACGCAaTAaGAcTtACATTCATGCGGCAATCCTGCATTCTAATTGTGAAACCCAATATTCGGCTAGAACCGATTTACAAAATAAACAACCCCATTTATGTGAAAAGTCACAGTGTGGAGACAGATTGTGGATTTTttATTGGATGGTATAAGAGAGAGCCCCAAAAAAaGAAGGTTAATTTCCAACGTGGAGAAAGGATTCAATTGCTCAATcGTGATGGAATAAGCAGTGAATTATACCCGGTGGgCAGTTTTCTAATAAAGAAAGATTTAATTaTAGCAATTGCTAGT

>contig00950

ACAGTAAATtGAAGTTAaGTCTGAAGGTTTGTGTATTATGAAGTAGTAGTGATG

>contig00951

CGAATGTAAGAGTCTCGGGAAaTATCATCcTCGACCTTGTCCTCCTCTTtGAGGGATCGGAAACAGCTGCGAATGAGCGACGAGAATCGATTCCAGATAACAGGGGTAAAAaGAAATTAAGCTGATCGTTAAGTTTATGCCATAATCAATTCTTCGTTTCCTTGTTTAaTTATTACAAaTTGAAGTAATAATtACCGGGATATGAATCAGCAATTTACAATATGAAGTCGCAAAACCcTGTtCAAATCCaGGAAAAACGAAACCACATGGTAGACCcAACGGAGGAGGCGAACGAGAAATACGACGAGTTCGATACAATAGTCATGCCACTtGCAAGATCGCTCAGCAAGAGCCCATTGAGTGACGAAGGAATCGAGCCTGATTCTGAAaGAAAAaGAGCTATGGTAGGTCGTtGTTGGAGCTTAGATTCGGCTATTCCAAGCGACGAAGATATCTcTCAGGGTGtCCGACAACAAAAGAATAACAAGCTGCAAGTTTCAAGATGTTGCAGTTCAGATAGTGCTGTTCTCAGTGACGATGACCAAGTTAAAGGATGGGATAATAATATTGCAGGAGAGAACATGGATTACGAATCCAGTGAAGGAAGGCCGAGATATTGGaGAaCTCCTaGCGTGGTaGTGaGTGaTTaTTCGGATTATTCGT

>contig00952

aCAGTTTTGTGAGACCGTTAGTTTAAAACGAAGATGTCAGGAAGTGGAATTTGGAAAGGCCGAAGTTGCATTAGAGCATTACTGATGCCGGATCTGAATATCACCaCGGGTGAGGAGAAGAAAGCCCTCCCAGAAGCCATCGCTCCaCAGGAAaTTGCCCAACAACCACCAGAGGTGCATGACTTGAAAACACTGACCCCTATTCCAGAGGCTGCATCaGATAGTGGATTTTCAATGAaGGAGTTTCTaCAGCAAGgCCaGgCcTTCGTGGTTCAAAAGGATGTGGGCAGAGTGAAAAACGCTGCCACAGCACAGACAATCACTAAGGCAGCCACCAGTCCAGCGGACCTTATG

>contig00953

CTTCCTCAAGTGGTCCGTCATCAATAAGATCCTTTACTACTTCTCCGTCATCATCCTCCTCTTCATCACTCTCCTGCATCGCTTTAAATCTCTTGAGTTTCTTTCgCTCATGcT

>contig00954

GTGCTTCAATTtCTTTTCTATTGAGTTATACAATGCGAAAAGTCTaGAAAGACTAGAATTAGAATATGCCGACTCAATTAGAGAAACAACCACGC

>contig00955

TATCAACTCATTGTCGGTTTATTTGTTGAAAGAGTAGCAAGTGTATAACTTTGATTATAAAAAGTCAAATTAGTATCATATAcTtAACGTTTTTTtAAAtGTGTTGTTTTTtATTAAGCATTTAGGCTTTGTATTTAAAGTAAGAAAATATGAGTTATTGCATTTAAGAGGTTATATGGAAAGTGCTTTAAAAATTAGAAACTTGAATAAATATGAGTCTTTCTTTTGCAATTGGGATCTTTGCTTTTGTGTTTCTAATCATCATTTCGGTGTTGAGACAATTCTGGCCAATCCGTCTGAAAAATGTGGAGAATAAACACGTTGTGATAACTGGCGGTTCAAGTGgAATTGGAAAATGTGCAGCTCTGATTGCCGCCGAAAATGGAGCCCATGTGACTATCATAGCGCGGGACATTCAAAAACTGGAAGCTGCAAAACTCGAAATTGTCCGAGCATGCAAAAACAGAGATACACAAAAAGTCGAGTATTTaTCTCTGGACGTGAGTAAAAGCTACACTGAGGTGGAAAAGTCATTGAACGATTTAGAAAAGTCCATGGGACCAATTTACATGCTGTTGAATTGCGCTGGCACAGCAATTTGTGGCAAAATCGAAGACACTTCTATAGAAAGTCTAAAATTAATGCTCGACGTCAATTTCCTTGGT

>contig00956

ATCTGTCAAAGGTTTGAAAGGTTACATTTCATTATATAATTTTCTTTCATACTTTGTTCGCAAATATAAACAATAAGCAATAAACTCATTACTAGAATCGTGTTTATGCTTCATAGAAGCTTCAACATAATCAGGCAGTTTACGAATTCAGTGAAAAAaCCAaCGAAAATGGTAATTCGTCATCATGTAGAAGGCTACGATAATTTCTTAAATTTTATGGAGAAATTCAAAGGTGATGGGGTTGTATACGTTCTTTTTAGTGGAAGCAAATTGCCTGATGGAAGAAGCTGGTGTCCTGATTGTGTAGAAGCTATGCCACATATCGAGGAGGGAATTAAAGCTGCACCGGAAAATTCGCATTTTGTTtATGTCGAAGTTGGTG

>contig00957

GTtATGAaGAGTTTAGGAATTATTGTAGATAGATAGCAGAAATTATTTAATTATTACTGCAAAaGATGTAACCTAGGTTTCAATTTATACTGCGGtAATCGTGATCTTCGAtAAATTTTtATTTGTTCCATTCAAGGCAACGACTAAATA

>contig00958

ACAGATAAAGACCCTGCTCGGCCAAAaTCTtCAGATTAGGGATTCATTTGTTGTCATTATTGAAaTtATAGTCGACGTTTTtC

>contig00959

GTAAAAAAAGTTAAAaTAACTACAACAGTATGTATAAATAAGTAATTTaTGCTTTtAACTTtCAAAGCAATTACAATATTtACCTATATCACGGAATtACTGGTTGATAAAGTGCACGTAAAATCGATGTGATAGTAATTTAAAATAATGTTTCTTTTACGGTGGACACATTTCCTTGTAAAATAAAAATAGGTTTTCAATTAAAAAATAATCAAACCTTTTAAAAGCACAAATACAGCTTGAAAATTAAAATTATGGAAGTTTACAACGCACTTTAATAAATCTACGTCGAGTGGGAAGAAAATCACAAGAACTTAGAGAATATTATTATGTTGTAGCATAAGACGTAGCAGCTAAAAATTGGtAAAATTCGTTaCACTTCTGCATATACAGT

>contig00960

AGATCACTTAGTGATCGAGCAATGTTACGTGGATCGTGATAGAATTACATCACCAGCTACTGATGTGGACAACTTACCAGATTTCGCATTGAATAGTATGGAAAGGCGACAAAATCGTCAATCAAATGAATCTAAAAGGCACCAAGGTGAAGGCGACATTAGTTTTCCATACCTTCCTGATTATTtAGAAAGgAGAAGTCGAAaCCCTGTCTTGTCGAATTCTGCTCATTCTGACCCTCTAGATTTGCCtAATTTTAACTGCAaCGAAAATGCTAGTGGATCTGATTCTCGAAATTTtCCATTTGATCTGCAAATGCCTCCAGCGGAGTCACACAACCTGCTTGCGAATGGCGCGTTAAaCGGAGCATCAAATATTGTTAAGGCGCTACCAGACTTTTTGAGTGATGGGCCTA

>contig00961

ACTAGaCTAATTTTtATTTTTCATGAGTCAATAGTTTATACTTCCTTTTTTGCTTTGTAGTTTCTTTGTTTCTCAAGTAGGAAACTTAAAATAGAGCTTAGCAGCTTTTTtATAGaGTAGAAAAaTTAAAaTAGAAATttCTCAATGAAaGAATaTAAAAAAAAAaagTTCTGATTAAAAaTtAaGTCATCTAGCCTCAGACATTGAGATTTGTAAATAGCGAGATAGATTTTTAAACCGGTTGGGCATTTTTGGCATTAAGAGGCTCCGAGGCAGCAGCTTTGGCTTTTGCTGAGCCcGTCTTTTtGTGCGACGTTTTCTTtCTTTGGACTCAGCTCCTGCAGCGACGAAATTATtGTGGAAGTATTCCTCGATTTCaGAGAGCTTTTtCCCGTGGGTTTCTGGAAGCAGAAGGTAAACAAAGGCAGCGGCTCCGAGGGAAaCTCCCGCGAAAAACCAGTGAAGACGATGAGgACCTCCTAGgATGTATaTCAaTGCAAAATAaCTTTGCACAGCTCCAAACATTAAGAAATTCGCCATGGAATAACTAATAGAATGGGCGATATGACGAATATCAGTTGGAAAAaGTTCAGCGGTCATTGTCCAGGGGATTGTCAACATTCCAATCATCGATGT

>contig00962

ACTAGTTCATCTCAATCAGAAAGAGGATGGCCAACTTTAAAACAGCATGTTATTGACAATAAAATTAATGTTGGATTGTGGGTTTTGAGACTCCTCGCAaTTTTATTTACGATATTGTATTTCATTCCTATATTTGGGAATCCATACAATGCATATTACAAAGCATTAATCAGTAATTCAGCAATAAATGCTTTGCGATTGCATCAACGAATGCCACCAATCCAATTCACATCAGCTTTTTTtGCTGCCTTGTGTTTGGAAGATTCATTCCACTACTTGTTTTACGCATCAATATTTCTTTATGCTTCTCCAATAGCACTGGTTCTTCTTCCCATTTTTTtATTCGCGTTACTTCATTTCTCAAGCTACTCTCTC

>contig00963

ACTAGaGTTAACATTAGAATTCTGAGCTGAAAAAaTTGTaTGTTTTAGTTAAGAAAAAGGAAAaaCACTGAATCACAGCTCTCAGTCTATGACGTGAAATCTATGACAATGCTAAGTTACATAAACCCCGGGAGGTTTATTCTTCGTTTTtAAAaTAAaTGAAaTTCCAAATTTAAAAAAaTGTCACAGCAAAAAGCAAATTGCAGTTTtCTTCaTTAAAGAAAAACGTTCAACACGTATCCTTTGAACTCTtACATTGCACGAAATAGCGAATTTATTTtGTGTCTTaCAGaGAAAAAAAAaCTTAaCGAATATTATTTTTGGCACAGTATTCCTTTTttGGTCAATGTTTGAGTTCTTTGGAATTTGCGACTTGTTTACTAGAAAGCGCAAaTTTAGTTTCACTACTTATGTTTCAACTCTGATGCGTGAAAGTGAAAAATGAAAAGCTCAAGATTTTGAAAAAAaTTGGAATACTATTATTGTAATGTGtAAAGAATTGTCGATCATCGTGAAGAATCGTGTAAGAGGACATCAGATTCCAGATTCAATAAAAAATAAAGTCAGCCAGTGAAAATGACTAGCACCAATGAGTTAGGCGCTTTAAAAAAGAAATGGTATTCATATGTCTCGCGCCAATAAACTTTCTAAATTGATTGGGT

>contig00964

CAATTGGTCGTTGTCGTGCTGCAGTTgTTGGAGATCGCCTTTGGCTTGTCTTtCTCCACTTAACAATGTGTTGATTTGTGATCGCAGCTCCTGTTCCATCTGTCGACTAGATTGCAAGTCTGACTTCAAACGTTtAACGTCTCCTTCGAGCTTGCGACAAATCAGTAACCTTTGACAATATTCATCTTGTTGTTTTtGGTTTTCTGAtATAACGTCACTCGTGATTTTCGCCTGACGACCTTTACGTTCTCGCTGATTATTCgTCgTGG

>contig00965

ATGGTTTTAAGTTATAAGAGATCTCACTTTATCACAGTGCTTTTGATAATTATATGAGAAGTGACGAAAATCAAATTATTATTTAAAAAGAAAACCCAAACTCGTTTTAATATGACGGACAAGAATGATAAAGCAAAAGTAGATTTAGGATTGCTTGAGGAAGATGATGAGTTTGAGGAATTCCCTGCCGAAGATTGGACAGCGAAAGATGAAGACAATGAGGACATTAGTGTGTGGGAGGATAATTGGGATGACGACGACGTAGAAGATGACTTTAATCAGCAGTTAAGAGTTCAGCTCGACAAACAGAAGTCACAGCTCGAATCAAAGGACAACTGAATGAATCCATGAAGTGGAATTAAAGTTTTCATTTAAATAATTTAAATGTTCATCTTCATATTTCTTGATCATTCTACAGGCTATAACTTATTTCAGCGTCAAAAATGTAATTAATTGTATCTATTACATCGTTTTTGTGCTATTAAAAaCCTTAACATTTTTGATTTCTG

>contig00966

TTAAAAATCAATGTTTCAGAGACGgATATATaTtAAaTTGTGAGATTTATTCAGAGAAaGTTGAAATATAATAAAGT

>contig00967

TTGAATGTTTTCGATAGCTACTAGTTAGCATTATATCATATATAATTATCTACAAAGTTATAAATAAGGTTTGCAAAGCAATCTTTTATACAAAGTATCTTACTTTTAACACTAAACTTGGAGTTTTATTTTTGAATAGAAGTTATTTTTTAACAAAACATAATTATTTTTAAAAATAATATGAAATTGAGA

>contig00968

TTTAAAAaTtAAaTTA

>contig00969

CTCCATGTTGCTGTGACACGGAGCTGTTGATTTCGA

>contig00970

ACtCGAATTCGATATGTAGACAGTCTTCTATGCCGACTTCCATTTTAATAGGGTTGTTCATATCAGGATAGGAACTAAGCGTATGAACAACGAGTTCAAGTTCCCTGACGATGTCGCTTATTCTCCGGACGACTGTTACTTTGAGAAAGTATCTGAGACGAACGTTTGAT

>contig00971

ACGAAAaCGCTTTAGCTTATGTGTCAAAaGATGGCAAATTTCTAATTGGATCGACTATGTTACGCAAGAAaCGCGCCTTGACACTCCGTCTTCCTCATTTTAAAAaaTATACGCCCGAAACTAaTTGTGATGACCTGAAACTTGTAGCTGGGGCTTCTTCCAATAAaGAAAGTGGTGAAGTGTGCGATATttCCAAAaTaGAACCAGTCACTGAACTTGAAACTGACCCTAAAGAGGAGACTCGTTTAGTATTTATAAT

>contig00972

ACTTCTAagACTAAGTTT

>contig00973

AGATTGCTTGAAATTTTTAAGACCGCAGAATTAAGTAAGTATGAAACCATAACAAACTTACAAAaTGCTTCCAATACTGTTTAATGTGAGCAtCTGCTTGaTGCGGAGTCTTAAAATCAAaCTAGAAGAaTTtGGTTTtAACTAAAAaGATGAAATAGGTAGGTGCAGACCTaTAAAACTAAAaTATTTTGATGATAGTATTAAGTTGGATTTTATAAAATTTtATAAACTTTTGATTACAAGACTCGACGAATTCAACTTTTGCAATAACATTACCAAAAGGCATGAACAATCGCGTTTAATACTGAAACCTTTTAGGCCTGCCAGAATTATAATTTTTACTATAGCATATTGGTTTGATCTTAATACTTCATCTACATTAATTTTAATAATTATCCTTCaGACTCTATTACGATCCAAAaTTTtCTATTAACATATTtACAGAATCTCAGTTCACATGCTACAAaTAACATGTAGAAATAAGATATAAAGTTAAAGGACTCAGCTGATGgAAGtGACCAAACATTGTTCATCaTAaGATCATGAATGTTTAAACtGTCATGTGTtCTAATTCGt

>contig00974

ACTCAATaGGTAGGCCTTATACATGCATGTTCTTAATATGTAGAAAaaTAaGAATCACGTTATCATATTTTtACAAtACCGGAATTTTTTTCtCATAACTTTGAATAGTATATTTTTGG

>contig00975

GTTACTGTTTTGTATTAATAAAAGTAGGTATTAaTATTCCTATTTAAaGATATTGACAAATTCCTTATATTGAAGCAGTTTGAGGTGATTACAGTTAATATGGTCGTGGAGGTGGACCACCACGCATCATGCCAGGTGGTGGACCTCTCATTGTTGGTCCCATTGGAGGTCCTCCTCGACCCATCGGAGGCATGCCAGGGGgCATACCCATCATGCCTGGAGGTGGAGCCATCATTCCAGGTGGAGGACCGCCAACTGGCATTCGTTGCGGGCCTCCAGGGTGCATTTGTGGAGGAGCAGAAACTTGACCTCTGCCACCAGGCGTCATAATTTGTTGTGATGGACCACCTACTCCACGAACAGGTCCTTGTAACCCTGCaGGAaCACCAGCAACATTTGTAGGCATTCCACGACCTGCGGCTCTTCCCATTCCGGGGCCAGGTGCTGCGCCAGGaaTTGGT

>contig00976

AAAACTACCAtGCCAGGAATtCCTTCCACTAGTCCTTCAAAAAAaGGAAAGGAATCTCAAGTCAATTGCTCATATTCTTTCTATGAATGCACTCAACCTTGTTTAGAGGgATATTTATATTGTGGAAAACACGTTTTAGAAGATCCtAATTCTTGTTTTA

>contig00977

GCCAAAaTCCAGCGCCAAAaTTAGATCGTAGAGATATTTCATATTGTCCAGATCATATGAGAAAaGCTCAAGTGGCTCGGATAAAATCCACTTCTAAACATTCTTTACCACAGACTCCAGAAAtGTTGCTCCTAAATCTAAGCCATTATGTAAAGCCGAAAAATTCAGACAGCAAGGAGACGGAATCGAAACCAGGAACTCTAAAAGCTTTGGATCCGTTTtCTGAAATCGATGCTTTTAGAGTAAATACAAGTGTGAGTGATATTTTGGATTACGCTaGTtCCTCGGATAGCGACGTtGAACCCACAATAGTAAGTGATACAGTTCGaGGTCCATTTCTCGACGACAGTGACAACGAAAGCGTTCACAGTCCcAGGAAGACCCTTTAAAGCACGCTGGTATCTTTACCGCTGAAGAGGTAATCTACATCGCGAGAGAAAAaCTCATTAGACTGCAGTCTT

>contig00978

TATTAATtCTAATTTTT

>contig00979

ACCACTAGCAGACCATGTGAATACTTCTATTTGTCCTGGAGTATCCAAAATCACATATTGGTGAGTTTTTCCCGCTTTTTCAATAAGTTCTACCACTTCACTAAATTTTGTTGAAAATAGATTCAAAGCTGTTACTATACCACCGTTAGGACCCAAACAATACTGTTTCATCACTTCCTTGTAATTTACAGTATCTCGAATATCTATGTTAACAGGATAAGGGACTTCTAGGCAGGCTGGATCCAAATTGATTACATAGGGTTTATTttCCGTGAAAaGCTCAGAAACAATCCTCTTTAC

>contig00980

CACTTGCCAAATCAGGATTTGCGCTAGTCTT

>contig00981

ACCGTTTTTTGGCGAGGAGAACAGACTCTAAGTTTAACAAGATTGTGCTAAAGCGTCTTTTTATGAGTAAGATTAATCGTCCTCCAATATCATTAGCACGAATCGCCCGTTTTATGAAGAAGCCAGGCAGGGAAAATCGCACTGCTGTtATAGTTGGCACTGTTACTGATGATTCTAGGATTTTTGAAGTTCCTAAGTTGACGATATGTGCACTCCGTGTAACGGAAAAaGCACGAGCCAGAATCCTTAAAGCAGGAGGTgAaTTTATTTCTTTtGACCAGCTAGCTCTTCGTTCTCCAACTGGGCGAAAAACTGTGATGATGCAAGGACGTCGCAATGCTCGTGAAGCAGTGAGACACTTTGGACTAGCGCCTGGTGTTCCACATTCGCACACCAAACCATTTGTTAGATCCAAGGGACGTAAATTTGAAAGGGCCCGAGGTCGAAGACGTAGTtGTGGATATAAGAAGTAATCT

>contig00982

ACAcTCCTACAAGACGTGaTtGgTAAaTAAgAAGATCACCTACATTCAGATGTCTGACATTCTCaTTGCCAGATCTTCCTAAaTAAaCATCTACTACATTGGTCCACACTCTATTTATAATAACCTGTTGAGGAAAAGGAGCCACTATCGGTATAGGATTTGTAGCTGTGCTGGCAGATATGTTCTCAGTAATAACAGAAGCTGAATGTCCATCCTGAAGGCAAAAaCTCGAACGAGTCAAGTGTGGTATTGTGTTTAGTGCTGAAGATTGAGTTTCTAAAGTATAGACGGTAAGAGAATCCACATAATATGTAGCTTGATATGTAGGCAGCAATGGTCTTACGCCATTATTACGTCCAAAAACTGCCTTTCGGCCACAGCTGCCACAGCTTGTAGGAGCGATAAATTGTTTATTACCTATATAGGCTCCAATACATAAAAGGCTTGATTATCGCTGATTATAAACATTATACCCTTATATATGTCTTGTAAGTTATTGATTTCgTGACGAAaTTGCCTcATTCCTTGT

>contig00983

ACtGCTGTAGCTATGGTGTCAGCTAGTCCATGAAAATGTTTATTTCGAGCAACAAAGTCTGTCTCACAATTTATTTCCACGAGAGCTCCATGACAATTGTCTACGATTACTGCTATCAGACCCTGAGTCGTG

>contig00984

CCTTCTTCAAATCATTCTCGTGCAACTCCAAAGCCTTTTTACAATTAGAAAGCGTATATCCAGTTTTCTTGCGAAGTTTGGCAAGCAGTGATTTATTTGAAGCTtGCCAAAGAAAATTGTTtGTATGAAGGTAACGAATTATTTtACTGGAAATCAT

>contig00985

ACTTACGACTtACAAGTTTGGGAGTGATTGGAGCACTTGTTAAAACAGaTGAACAAGAGGTTATTACATTTttGTTAACCACAGAAATAATCCcACTGTGCTTGCGAATTATGGAAAGTGGTTCAGAGTTATGCAAAaCAGTGGCAACATTTATTCTGCAAAAAATACTGCTAGACGACAGTGGACTTTCATACATTtGTCAAACCTATGACAGATTCAGTCATGTCGCAATGATTCTCGGAAAAATGGtCTTATCTTtAGCCAAGGATCCTTCCGCACGATTATTAAAaCATGTtGTTcGATGTTATCTTCGTCTCTCTGATAAtCCCAGAGCTTTGCTGGCCTTGAGACAATGTTtACCTGATCAATTAAGGGACAACACATTTtCAG

>contig00986

AAGAGtCATCAAAaCTGTAGAATTTTCCCGACGAAACTTGTGCTACGGCTGTGTAGTGGCCACAATTGACGGAAGGTCCCATATGAGTGACCATAGAGGTGAGTCTATAGTTC

>contig00987

TTaCAGTGTTGCCTGTGCTCATTATAGGATACATTAATAAGACTTTTCTATCGGATATGTCGTCAGGAAATTTTGCGTAGACTACCcTAGCTTCATGAGTGTCCATGTCGCTTTCGACtAATATTTTACCAATCCTTATACTACGACAACAGTCTCGAAGACCCTGCTCCATCGCCTCACCACTTCTtACTATAGAAaCACCACAATTtCCTTTTtGGTATTTAAGGCCTTtGTATTTGGCACCAGTTGGCGTTGTtATCACGCATTTCGAG

>contig00988

CAATCCCAATATGGAATCAtCAATaCACTCGGTAAATAACGCCGCTCATCAATCACATTCAAAAaGTTtCAtATtCTAGCATTTAGaTTCTGAAATTTGAGTGATCGAAATTATTGAGATGAAATGGCGTTAGAAAAaTACCTATCGAACGATCGACACTCGTTTAGGtATTtAGCATAGCGTGCAGCGGCcTACCGAGATATAATATTAAACTTtATTtCGCAATAAAATTCAAAGTGGGACTAAaTTTTCGACTCTAGTCCAATTTGTGCGTTTAACGGTTTtATCGAAAGGTATTATCTTCAGGAATCTATGGGTTGATCAAATTGGGCAGCCAAACgaCGCTTGCCAGAGAAAGAGCGTTTtGTAGGAGTTGTGTCGACTTTATCCGCTGTTTCATGTTCCATAAAACTTCCACTTAACGAGCTGGTAGGACTAGTCCTTCCAGACCCATCTTTTTtAGCCGTGAGAGAAACAGAAGGACGTGGTAGTGCCGACGGATGTGGAGCAACCACCACATTCCGGTTTCCGGAAAAGTAAGGCGAAATTCTCGGATCTAACGGATTTGCGGAGGCAGGTAGTAGATTCTTTATGGGTGACAAAAGGTCAGAAGTGTCATCACTTGTTTCGATATGATTCCACATTGGCAACGTCATGGGT

>contig00989

AtAAaGAGTGCCGAAAGTCTGCCGTTCCCAATCATAATGGCCTCCTTGATTTGTTGCTTGCAGTGGTTTGCATATGGTTGCTTACTGAATGATCCGTTTATTCAAACGCCAAATTTTTTAGGAATTGTCTTATCTGCGTTTCAACTTTCTTtGTTCCTGATTTACCCGaCCAAAAGGACCGaTCAGTTACATTTCATAtAAGACACGTtACCAtCAGatGTTTtGAATGCtCCTCGGGAATTTtAaGTTtCATtAATACGTTtCCTGCAAAGAGaTTCTGTAAAATATATCAATTTCATCTTTGGCATTTTATAGACATAATTAATAATTCGTTTtAATtCTCCAATGGTTGCGCATAAGTGATGTGTGTGTATGTGTGACTGAAAGCATAATATTGCTGGATGAATGAGAGTGGTATTCTTCTGTATTTCTCGGTTGATTTAAATAGTTGTGAGCTTTTAATATTAATACAGAGAACATGTATTTATTAGAATGGTGGGATGCGAAATGGTTTTTGGGTAAGgAaTTGCTACTTTGCCTAAGTAGATTTAAACGGTGACAATCgCTTTTATGTTTAAAAaTTACTTGAAaCCTACTTTGATGCAAaCAAGTCTTAGAAATTTTTTCATTTCAAGTTAAACCAGTGAAaTAAAaC

>contig00990

AAATACGAAAaGATGGCGTCGCCTTTGGAAGACTTGGAGTTGAAGTTAGAGCT

>contig00991

AGTATTGCAATAGTTTTTGACGAAACTTGTGCGTGATTTCGTCTCACGCGTATTTtCTCATATGCGGAATTCAAAAtGCACTAAAAATTATTaTCAAAGTTTTCCAAAAAGTAAGGTTAAAGTTTGTGTAAGATACAGTGTGAAATGACTTTAGTTTGTCAGGTGTAGTAAGGAAAAaaTCCGTGACTGACTACGAGAGGATCAaGAaCGAAAAGTGTGCCCTCGGCGTCCGGAAGGCGGCAAAACCGGCAGAAAAAaTCGCTAAAAATCGGTAACTGACAAGATTTtAGAAGCGTGTTGGCATCGCtCGCATAACCTtCATCGGCATTTTTTtAGCGAGCTTTCTTGGCCGCTTAAAaCCAATTCTTAACACAGGgCTACCCTCAAAAAaGCCCTTTtGAGCTAAGAAAAaGTTTAGAAAAaTCGTGTGATTATAGTAAAAAAAaGAGGAAT

>contig00992

ACTAGCCATGGAAAtCGTAAGAAGAAGTAATTGAAGGAAGATTATTtGATGTTTttGTTGTTtAGATAGAAATGATGAAAGTTACAAATTTGTTTGATTTAACGGAAATCAGATGTTCTATTGTATCGatAAAACTCACAATATTATTACGTGAtATTTTtGAAAaCCCTTtAAAAGGTCTtCaGGTGTTAAATAGTTTgTCTGCGTATTTTTTTTtAATTCAACGTGACTTTTTCGATTAATAtCGAATATAAAATAAAAaTAaTCGCGTCCAGCCAACAaGAATTCAGATTTGTCTTCAATATGTAAGAtGTAGTGTCTTAGTTTAAGGAAAaTTTTAGAAACTTCATATGAAATTAAGCAAACTATTCGACCCATTTATCTTTTAAATCATTAATATGAGAAAAGAGCCATTTtCACCACCATTATTCTATCATCCTTCATAATCTAGTGTTAAAAAAaCTTtACAATATAaTTCAGGCCTTTGCAaGATTAAAAGaTGGATTTTTAtGAAAAATGTGTAGTTTCTACATTAaGTGTAAATGAGAAGAAAAGCTTTTtaTtGTTAGCGTGT

>contig00993

ACcACTTtCTGACATACTAGAATCATCTTCTGGATTAATTTCGGAATCGTCGACCaGCAACGCGTCTGGGGCAAGGAGTTTTGCTTCAACCGGAAGAGGCAGAACGTCAGCAACAGATCTTGACTCCATCGCCACATCCCAAaCCTGCTTCATCAGCTTATAAGTAGAATCCCTCGACAATAAGGAACAAAAAaCaTGCCTCTCATCTTCTGTGGCTACTGCCACCGCATTCGGAATGATTCTTGCAGTCTTCTCCTTGCTGATCTTCAACACTGAGACGGTGGGtATCAAAAGTTTGGTTACATAGCCGAAGACATTACTGTAGAAGGCAAAGTAGTTTGGCGTGATATAAAGATGACCCTGGAGTAAAaTATCACCCACcAGTGCACaGCTGTAGTAATTCAAAACTCTCTCGTCCGCTGCCACCTGGCT

>contig00994

AAGAACGTTAAGTCTTCTCCACCTGTGATCAAAAGACATTAATGGAGTCAAATCTGTTGACGGATTCCAATGGCACAGAATATCTGTCCGTCGAATT

>contig00995

TCTTGATCAGTGCGAGCTCTAAGACAAAAGTATCAGTGCTCGCTTTCATCTTCACTCAACATGACTGAGTTATTTATATTTTAAAAGTGATTGCCTGTAATGCATTGTGAAATTAGAGTGTATTAGTGAAAAATTAGAATATTTTTGGATTTTTAATTCAAAAAaTTGTGATACTTCCATCCCCTACGAAaGTGACGCGCTTCATCACATCTGGTGGAAAGATTtCCTACTGCTCCAAGAAAAaaTTAGAGGCAtAGGGATGGCTTATAGCAACAATGATTTAGGTGCCGAGGGTTTtAGACTTTTTGCAGGTCTTGACGATTTTGGAAGTCTTGGTGCTTCCGGTAGTTCAGGAGATATTGGGAaTTTTGGAATTTTCGGagCTATTGGTTCCAGCTTTtCTAATCCcTTTGATTTTGGGATGTTAAGATCAGAATGGATGAGGGAAGAAGAGTATTCCTCAAGGAATATTCCAAAAAaTGGGCTCTTAAAAaCATGTTCACCCTCAAGTTCGTCAAATGATTCACCTGCTAGTTTGTTTTCTGGACTGAAAATAAAAGCACAGTCAGAAGACAGTTCAtCAGCAAGTTCATCCAAAACATCACCTTCAAGCTTGTTTTGtGATCTGCAGaTGAaTGCaGAAaGAAA

>contig00996

TCACCGTGTGTAATAAGCTTTTGACTATCAGGAGTAAAAGCTAGAGTGTTACATTTTTTGTTCATTTTAAATGTGTTAATTAGTTCCTTTGTTACACTAGATAATACGTATATTtCACCCAGCCTCCCACAGATGACTaCATATTTTCCATTtGGAGAGACTTCGATTtGTTGCATATTAGTTATCCCATGCGGTAAGGCTGTTTTGAGTGTGCTACCACTCATTAAATCGTATGTAAAACAATGCGCGTTACTCAGTGaTCCTAAAaTAATTTGTGTCCCTTCTTtCAAAAaGCGAGCCGTGCTGATAGGAAaTTTTTGAAATTTCGCACTGTGTAaTTTATTATTTtCTCTGCCATCTACCTCAAAAACaGATAGCACTCCAGATGAACCTGCGACTAATGCCACAGTAGAAGTAGGATGAAATTGCACACTTGTTACGTGCGGTCCTTCATTATGAGTTTtCTGATTAATATCTGTTAGAACTTTCAGGTCTATTAtACCTTTTCGTAATTTCTCAACCTTCGGTGCCACTTTATAATTACTATGCTTAAAAACTTCACTGTCCGATCCATCGGAGTCCGCATCTTCTACATTTAGCTCGGCCCACTTAGGCATGCCAAACTCTTGGGAGAATTTATTTTGTAAAAAATCAG

>contig00997

GGGACtGCAAGCtACGTtGAATTGAAATGCTCGCTTAGAATtACGGCATAGCTTTtGTATCAAACGACCGATTGCAC

>contig00998

AACTATTTTAAAGTGTCATGTaTCAAAATAAAAGGCCTataCCTTCAAATTAAAATAATTAAAATGCTTGCCcATACTGTGACTGATATTTTCCCAATCaCCTATATCAAAATAAAAATATAAACCCTTCAAAAAATTCTATACACTTATCGAAAAAAGTCAAACTTAACGACGCTTGTTCATTCGCTATCTATGCAGCTcATATACGCAGTATGGCGTTTTCAAGAAGCAGAGTCAATAagaTACACAGCGCTAGAaTATCATACTTACTATTCCCTTATATCCTTTACGGATCGACACTATCGAAGTGTTGGGCTATTAATCATTAATACTACTTTTTCGTATGCGTATTCCAAATTCTTAGCGAGACCAGCGCAGCAGCCATGTTGTTGAATTTTACTAAATTTATATGTCCTGAAGTTTTTCTCCTTGT

>contig00999

ACCACCAGCACCTGGAATAGTTCCAATGGAAATTTCCGGTTGACCAAACTTTGCTTTGTCACCAGCATAAATGATGTCACACATCATCGCCAATTCGCAACCCCcTCCTAATGCATAGCCATTCACAGCGGCAATCACTGGTTtCTTGGCAGCTGCCACTCCATTCCAATGGCTTAAAAaGTTtCCTTTtATACAATCTGAATAAGAATTGTTTTGCATTtCTTTAATGTCAGCACCTGCTGCGAATGCCTTCTCACTTCCTGTAATGACAACAGCACCGACATTATCTTCTTTGTCAAAGTTTTTAATCGCTTCGTTGACTTCAGTCATTAATTTATCACATAAAGCGTTTAATGCCTTTGGTCTATTAAGGGTAATTAAACCCACATTTTTTTCTCTCCGGTAGTTTCTACTTTTATAAATTCATAATTCTGAACTTGTGTGGAGTAGAATTTGATGAGTGGAGTTATTTGTCCTCTATTTCTACCAATTTGTGATACCCGGTTGACCAAAAATCGGGCTACATATGTGTTCGCCATCTTTAATTTTTACTCAGAAAAAGACAGTGTAATTATATTTGCCGGTTTTGAAATAAACGTTTGGGAAGAGCAACAGATTAACAATcTGT

>contig01000

CAGCGATCAATTTATTAGCTTCTCGTTGGATATCA

>contig01001

ACCAACTACGTGTAATTTTTaCAATTTTAATTATTTTtCTGgAAATTCTCTATaTTtATTAATTTtCTTaTTTTTTCAATGATTTAtctATTGTTGCACGCTGAGAATAACATTGTTtCTTTTTTATTAtGgCGgTTTTCCATTTtGATTATTTtaGACTGATATATAAAGAAAATGAATACAAAATATACTTGATATTAAAGGCATCTCTTTGaCGTGACATTATCCAATACTTTTTGtCCTtCCcATTTTTAAATtGaTAaCTCAAATGGATTTTTAATAGAAGATAAACACAAAAGAAAGATTAAGCTTTGTTTATCTGCTATGGAAAATGTATTCGAG

>contig01002

GATATTAAATGCTTCAGGAGACC

>contig01003

GTGATGCTTCTATGGTATTAGCTCAAGAAAAaCTGGCTGGCGCTAGTCAGAAAGTTAAAaCTAGGGTTC

>contig01004

GTCCCAACCAACTGTTATAAAGTTATCAAAGCGATACAACTGTCGGAAATGTAaGCaCaTAaCTGT

>contig01005

AcAGAAAATTGCGGAAaCAGACTAGTTCAACGAGGGCCACTAAATTGCCTCAAAATTGTGGAATCTGAAGGAAAAGGTTCCGGTCTTtCCACCACTGAATTCATTAACAAGGGTCAATTTATCTGCGAATATGCAGGTGAAGTCATAGATATCGAAGAAGCTAAaCGAAGATtCGAGGAGAaTAAAAGAaGCATGAACTACGTCATCGTCATCTTCGAaCATGTCGGAAAACaGAAAaCTACGACTTGCATAGATCCAAAaTATTTtGGAAATATTGGgCGCTATTGTAATCATAGCTGTGAaCCGAATGCACGTCTTGTGCCTGTtAGGGtAGAAaGAGCTTTACCGCGAGTGTGTTtATTTGCATGTAGGGACATTGAACCTGGTGAAgAAaTAACTTTCCATTATGCGGAAGGGGTTgATACCAATGTCCATAaTCTAAGCGAAACACCaTGCCTTTGTGG

>contig01006

CTCCAAAaTaTAGTCACACGCTGCAATCTG

>contig01007

ACCATCTATCGACTTTGAAAGAGGCGAAAGAGGAATGAAGTCCAAAAACCTGATTTTTAGGAGATGAGACTTGCGCCAACCACGAGATAAGGAAGCTTCGCACTGCCATTAAAATCAGCTGCCTAGTAGTGGTTTACTTTTCCTTCCAATAGTATTTTGGATATTTGGAAGAATGAAGAGTTTtAAGACTGGGAGAAATTCTTACTATCACTAGGAAAAGATTATCGTAACTGATACCCtATAaTTTAATCGTTGTAAAATATTCTACCGTTTATAATTTGACACTATGGAGAtACTTTtCAGACCTAATTTAAAACCAAaCCTGATTCATGGATATTTATGACTTGgTTCCATGTTTTTtAGGACTCTTACAtACTTAATTAaTGAATAAATAAaGTTAATATTaTGGATTGGATTTAAGGTGGCTTGATCGCTTTTGAAAAAAaTTATAGACTGCTCTGAAACTTAGAGATTATCTTAATAAAATATGCTAGATtCATTTGGAAAAAAGAAATACCGTGGTTGACGATGTCATTGCCGAAAAA

>contig01008

TTCCATCAAGTGACCTTACCTCTCTAGATTCGCTTTCCCTCGGATATAATAGACCAGGAAGAGATGCTGGGGAAGGATCCTCAACTGCAGCATtAATTTCTGAAGAAACTCCGGAAATCTCTA

>contig01009

ACGTCGATAAATCAACGTAGATTAAAGCAATGGTTGTGTAATTGGTGAAAAAGTGTGGCTGTTGAGTGAGTTGAATTTTTAATCTATGCATATAATAACGTTGAATTGAATTAAAGTTGTAGGCACAGTTATGATATTTCACATAATACAGAAATGCAACTATAAACATTAATACTCACTGGACTTTTTAAGCTAATTAGTTTTTACAAAGTGATTGCGATTCAAAGATTCAACAGATGATTAGACATCaCATATTAATCTTAACGCTGATATAGTAACAAGTCTAACAACATtCAAGACTTATTATGATAAAATT

>contig01010

CTCTACAAGATGGCGGACACGGCAAAAGCTCCTCCCAAGCAGGGTGTTAAAAAGTTGAAAaCGAAGAAGGAGCCTAAAGATCCATCCAAAAATATTATGCGTGAAGTCCGCATTCGCAAATTATGTTTAAACATCTGTGTCGGCGAGTCTGGAGATAGATTAACTCGTGCCTCTAAGGTATTGGAACAGTTAACAGGCCAACAGCCTGTTTTCTCAAAAGCCAGGTACACTGTCCGTTCCTTCGGTATTCGTAGAAATGAAAAAaTTGCTGTACATTGCACGGTTCGAGGAGCAAAaGCCGAAGAAATTCTTGAGCGTGGATTGAAAGT

>contig01011

ACCCCTTTGTtACCTCATAAAAAATAaTTGACAAGTGGATATTTAAATAATTCTTTTttGtAATAAACATATAAGTAATTGGATAAAaTTGaTTtGTTTAAAAaTAaTAATTAGATCCTTAACCGCACATCACTTGACGAACAAAGTCTTCGTAGTTGATATTGCCTTGCGAATCTTCGTGACCCGCTAGTAATGTCTCCACTTCCTCGTCGCTCAATTTCTCACCAAGCGTCGTTAGAAGATGCCTAAGCTCAGCAGAAGAAATAAAGCCATTTCCGTCCTTATCGAAATGGCGCAAACCCTCAATAAAATCGTCAGCAGTATCAGATGTGCGTGCTTTGCTAATAGCTTGGTAAATTGGCAGGAAGACCTCAAAGCTGATTCTTTCGTCAGGTTTGTGCTGATGTGTGAATTTCTTCACGTCTGATTCAGTCGGATTTTGTCCCAGAGCGCGCAGAGCATCACCTATTTGTGCCACGTGGATTTTTCCATCACCACGGCTGTCAAAAAGCTGAAAAGCTTCCTGGAATTCAGCCAGTTGATCCTCGGAATAAGAAGCCATTTTACCCTTTCACGCCCGTTGACAAACGGTTTCTAACCTACGTTCCGTTCGTGTTTGcGATGTTACaCCCGTTACACT

>contig01012

CTACAGCTTATTATCGAGGAGCAATGGGAATAATGTTAGTTTATGACGTAACCAACGAAAAAGCTTTGAAAACATCAAGAATTGGATTCGTAATATTGAAGAAAACGCCTCAGCCGATGTCGAAAAAATGCTTCtAGGTAACAAATGCgAaCTCaCAGACAGGAGGCAAGTTTCGAAAGAGAGGGGAGAACAACTCGCAGTGGAGTATGGTATTAAATTTATGGAAACATCAGCTAAGTCGAGTATCAACGTCGAAGAAGCTTTTtAtACCTTGGCTCGAgaCATTAAGGCAAAAATGGAAAAGAAaTTGGA

>contig01013

GGCGATTGAACCACAGAGGAAACCTaCAAGTTGGCTGACACGCTGCTCTAtaCTCTGACCCTTCAACTCTC

>contig01014

AATACTGGTAACCGATGTCCcAAAGAGCAAGCTGCTCTTAGAACTTCATGATTTCCTTTtAACTCTCCACTTTCGACTGCTTGGACATACCTCAAGACGAGTTTAACTCTTGAGTGTAACATCTTAATTGCACTGTGTTGAGCAACAAGATGCTCAGCAACTAAAGAACTCT

>contig01015

ACTGTTTGAAGCTGAGAAATTTTACTGACACTCGGAGCGTTTTCATCTAATTTTGAAATCAACGAGGAATAAAGAATCCCACTCATTATAAAATGCGTATAATTATTTTACTTCTGGGTTTCATTGCTCTGGTTTCCTGCTCCAAAAGTAAGACACAAAATAAAGATTCCAATGAACCAACAATTACAGATACGGTATGGTTTAAGATAAAAATTGGAAATGAAGAGGCAGGAACTATAGAAATCGGTCTTTTCGGCAATACTGTTCCTAACACTGTAAAAACTTTATTGAGCTTGCTAAAaGACCCAAAGGTGAAGGATACAAAGAAAGTATATTCCACAGAGTTATCTCAGACTTTATGATTCAAGGTGGAGACTTCACCAACAGAGACGgAACTGGAGGCCGCAGCATCTACGGTGCTAAATTCGCTGATGAAAaCTTCCAaTTGAAaCATTCTGATGCTGGTTTTGTATCTATGGCAAaCGCAGGAAAaGACACAAATGGCTCTCAATTCTtcATCACTCTTAAGGCGACCCCATGGTTGGATGGCAGACACGTTGTATTtGGAAAaGTCACCAAaGGAATGGATGTTGTTAAGAAGATTGAAAGT

>contig01016

TTAAAGCTAGGTCACAATTCAAGCGACGCTCGACTGCTAATAATgtGGAGATCATCATTCCTGTTCCAAATGACGCAGACTCTCCGAAATTCAAAACTACAATTGGAAGTGTTAAGTATTCACCaGAGCAGAGCGCAATTACTTGGTCAATCAAATCATTTCCGGGGggAAAGgAgTATCTAATGAGGGCTCATTTCGGTTTACCTTCTGTTGTTAGTGAAGACGTAGAaGGgAAACCTCcTATtCAAGTTAAATTTGAAATTCCTTACTTCACAACTTCGGGGATCCAGGTGAGGTATCTGAAGATCATTGAGAAGAGCGGATACCAGGCGCTGCCCTGGGTAAGGTATATTACACAGAATGgTGACTATCaGTTAaGGACCAACTAGGGGGAGAATAGATGAACTAGTGACAAGAATGTGGCGATTCGAATGAGTGCAAaGATTTCGATGGGAAAGAGATATTTATATTAGGACTGGGCATCGATGCTCAGATCAGCTTTtAAAATAGTATTT

>contig01017

CGGAAAGTTATAGAGTATTTGCTGAAaCTAAAAAAAaCTAGTtAGTGTCAAAtCATCGGAAGTCCTAGTTTTTAATTAACGCCAGGACGATAaCA

>contig01018

GCaGGTAGGACGTAATTCTGACATaTATGTCTCGaTGGTCTCAtATACGCACCAGGTTGCTGCGAAAGGTTGGTtCATTGCCATGGTGTCCACGACCGTAGAAACAAACAACCCTGAAGCTGAGA

>contig01019

CTCGGTATCGATCTTCTCGGTCCAATCAAGC

>contig01020

ACAGGGACAAAAATTAAGACTGATATGGCCGTTTACAACCTTTTATTAGTTCGTAGGTGTTTTTCAATAAGTGGTTTCAGAAGTCTGTGGACATCTAGAGCTCGG

>contig01021

AAAATGCTTTCACTGAGGAGTCTAGTTCGAGTAGCGAAAGCGAAGAAGAATACGAAAAAAaTATCAAATCGAAGATCTTGAGTGCTTCATTAAACTTTGTTtATGACTACGGCTGGAGTCAACAAGCGATCAGTGCAGGTGCGGAATCCATCGGATACCCAGGAATTATCCATGGAATGTTTCCAAATGGAGGTGCAGACTTAGTCCAATACTTTTACTCCTCTTGTAaTAAAAAaCTAaGTAAAATCTTGAAAGAGAATGCTTTGGCGATCGAAGCTGATCCTTCTAAGCCTAAAAAAaCTGCAGAGTCTTATGTTCGAGAAGCT

>contig01022

AACCCTTTtGTAGGGTGGACTACATGCCATGGACATTCTGGAAGGGGGgCcgTCTTCTCGAAGAGGAATTCTAATACCGCTGGGAgCTGTCACGGAAATGGCCGGAGTCTTGTTATTGATGGCAAGGGGCTGATTTGATTCACTGTCACTAAAGTCCATTGAATTACTGAATGAGAGTTtCCTTGATTGTGGCAAAGGTGACATGCCAAAATCCTCAGCGGAAGAATCCAAAACATCCTCCaCATCGCATCCCGAACTATTCGTTCGACAGCTAATGTTGAGTTCCTCGTCCTCGATGTCACATGCGTCGAAATGCAGCTTTTGCGCGACTTCCATGATATCAAATTGTCACCATAAGTTGTCGATGTGT

>contig01023

TCGTCTTTTTAACTGGC

>contig01024

ACGCTTGGTAACAATCCTCTACAGGTTAGGGTGATACTAAACGATAACGAACCTTTGAATACTAAATACCGTGGTGGTTTTTtAACTTACGATAAAGCAATTATTGGATTACATTATGAGGTAAACGCTGTTTATGGAGATGAAACGATAATGCGATTAGAAGCTGTAAATCTTTACGTTCGTAGAAATTTTCTGTTACGTAATTCTTACATTGAAATCGCAACATCAGCACCTAATACACCAATATTTAATTCTGAAAATGAAGAAGATGAATGAAGACGAAATACGACGAGAAGTTGAAATTTTAGGAATAAAATAAGTTATACATATCCAAGGAAATTAAAGGTATAACGTCGTTTAGTGGTTTtGTGAATTTAAGGCTAaTtAAAAaTTGAATTTCAAGTTAAAAAaTAAAaCAAAATaGCACATTtAaGATAACATTCTTTCTGATTTGAA

>contig01025

aCtCTCGGGACGATAGAGACAGAAGGGATCCGTATGATTATCGGGAATACCCTCGAGACTCAAGGGAATATCCTCGAGATTCGCGAGATtATCCGAGAGATCCAAGGGAaTCAACTAGAGATTATTATTCACGAGATTCTAGAGAATACAGCAGAGAATATAGTAGTAGAGATaTCGAGATGAGTGATCCAgCCGGTAGCGGAAGGGCCATGGGCCGAGGTCGTGGGCGATCATCTGCAAGACCCACACCACCTCCAGAGAGAGACTCCGCGAGTAGCTGGGGAAGAAGACCTGgTGGTTCTGGAGACCCAGGAGCTGGATCTTCTGGAGGaTATTCCAGACCaGCTTCGGCAGCGAGCGCTGGGCGAGCGACTCAAAGGGTAGCTCCGGCTCATGAGCACCCATATGAATCTGGTGATTCTTCTCCTCCTgaGCCTGG

>contig01026

CGCGTCAAGCAAGGCGACTTTATGTTGGAAaCATTCCTTTTGGtGTTACAGAAGAAGAAATGATGGAATTCTTCAATCAACAGATGCACCTTTCTGGTTTGGCGCAAGCTGCTGGAAACCCCGTTCTTGCATGCCAAATAAaCCTCGACAAAAaTTTTGCTTTCCTAGAATTCCGTTCGATAGATGAGACGACGCAAGCAATGGCCTTtGATGGGATtAACTTCAAGGGCCAGAGCTTAAAAATAAGAagACCACATGACTACCAaCCAaTGCCTGGAATGACAGATAACCCGAGCATGAACGTTCCAGATTCACCCCACAAAaTCTTCATtGGTGGGTTGCCAAACTACTTGAATGAAGAaCAGGTGAAGGAGCTGCTGATGAGTTTTGGGCAATTGAGGgCGTTCAACTTAGTAAAaGaTTCGGCGACAGGACTCTCCAaGGGTTATgCATTCTGCGAGTATGTTGATGTCTCTATGACTGATCAGGCAATTGCAGGATTAAATGGAATGCAGCTTGGAGACAAGAAGCTCATCGTGCAAAGGGCTAGCGTTGGAGCCAAAAATCCAATGATTGGAGCTCAAGCACCAGTGCAAATTCAAGTTCCAGGGCTAACAATGGCAGGCACGAGTGGTCCACCCACAGAGGT

>contig01027

CGGAAACGTAACGGAAAGTGACGATTAACTTCTGGTGACATACTCGTGCTCCATTTCGCTAAGATACGAAAAAaGTTAAAGAAAATCTGGTGAaCCGTTCAGGGGATCATCAAAAAaGaTAAAAAaTaCGAAGAAGGATGACAATGTGCTCAGGACAGTTCAAACAaCATTtGTGGAATGGTTATAGCAATGTAAAGATTTGCGCAGCTTATTGTTGCAGACTAAATGTATGaTTAAAaGTAAAAaTGGATGAATTGTGATTTTGAGAGgTaTAAGTTCAAAGCAGCCCTTGTGCCATTTCAaTCTAAAAaTTGAAAaGTATTTtAACTGAATAGTTTATCCGtAAAAATTTaTAATTAGATTAAATAGATAGGATTACACATTCATGATGGAGCAAAATCACGTTTATACACCTGAAGCTGTCATGACTGATGTTAATATGCAGGACTTGCAAAAaCTACTCGAACATTTCGTGAAAAG

>contig01028

cTcTAGAATCTGTTTATACAATGGGATAGGATCGAATTCCCTATCAGGGTCTTCATCACTGTCGCTGTCTCCTAGGACTTTGTCTTCGATAGTTTTTTCGTTCTTTGTCCCTGTAGTAGGCTTGACCTTAACCCAGTCAATATTATCCAACCAGTTaTCTCTTATTAGCTTTTCTTTATTCAAGTGGTAAtGCCCTTCTTtATCAAAATGCCCCTCTTCGAGTTCCTCCGTCATATTAAATGCTGTGAATCCAACCCCTGTTTCCGGAGCTGAAGGTCCTTCTTCTAGCCCTTCAAAATCATTTTCATGCATAACATTGTATGATTCTTCTTCTGCTTCGTCGTCATCTTCATCCGAATCCAATGAATTTTTAAACGCTATTCCTGGAGTTTCTACATCTGTTCCGGATTCAATGTCACTTTCCTCTTGGAATTTGCGTTTAGGCATTTTCGTAGATTGATAATAAGCAGTTAAACAAAACTAATGTTTGTTTTGTCAACGTGGAACTCCTTTATTATTACTTATTTTACTGGTCTAATTG

>contig01029

ATTTCGTCTTATTTTTATGAGAAATAACGATACTATGTAAAGTTATCAAGGCAAACGTCGTAATCCAGTAAACT

>contig01030

TTCAAAATTATcgTCCAGCTGTATCACGTGAAAATCGAAAATGTTCGAGGAAGAGCTTAAATCAGATTTAACGGAATTAATAAATCGGTGGACTTGTCCAAATTTCGAAACACCGGAAATCAGGCCTTTGTTGTCAAAAAGtGAATTTTTGGAGGTTTTAAAGAACGTCATAAAAGTTACTTTTGCAGTGAATGTTGACTTTTTtCTAACTGCAAATCATCAAAATGATTGTTCACATACTTTAGAAAAAATTACTGATAGTAAATGCAACGTTGATCCTTCTGAAAGATATCAATCATTGGATCCACTACCATTAATAGACTGTGAATATTTGTCTGAAGAAGTGTGCAGAGATGGAGAAAATGTAAACATGACCATCTGTATTGAAGACGAAACAATACATGAAACTA

>contig01031

tGTATTACCAGTATAAATAAaGGTATGGAGGAGCAGGAATGTTAGAATGAAAGTGACGTTCGCACTCGCGGAAGAAACTGCGTTTGACATCTTtGAAACATTTGTCAATTCCCTTAAATTTCTTTTGCAGAGATTTTGTCTTTAAATTTTGTTTATAAAGTTGGTATTGTCTTCTTTAATTGTTGAAAATAAAaTTTCATATAATTGGGTTTTCGTAGTGAATTAAATCATATAATTCATTTTTtACAATTGAGTTTTGTATGAATTTCGTATTATTAATATTTTTACTATTTTtATTTTTTtAAAatAGCAGAAGTAATATTTCTTCTGTGAaGCATTCAGGCTCTCAAtAAAGTTTTTATGCTTTCTGTAAAGACATCGAATGCGTTTGATTCCGAAGAGTCCATAATCCTTCGATTACTCCGAAGTTCCGGGTGATTCTAATTCACTTGATTCAACAGTCTCTCGAAaTCAATTtGTCTTGTGATATAGAATAGGAATCAAaTGGCCTTTGCTGGTTTTTCCATTGAATTAAAATGTGATACAATTTACTTTGCTTTGTTTTGTAAGCAATAATCTTAAGGCTGACTGAATCGAGCGAATAAAAGTCATACACAGCCAAGTGTGAACGGTAAACTTTGCGCAAAaGT

>contig01032

TTTGCCTCCTCCTGCGCCTTACCTATATTCTTTTTGTATCTTATCCGCTTATTGCCGAACCAATTGGATACCTGGCTGACAGTGATTCCACATTTTCTCGCAAGTTCTTCCTTCGCTTCTTCACTAGGATATGGATTGCTCAAATGAgAGTAAAAGTATTCATTCAAAATTTCTGATGCTTGTTTGCTAAAGTTACGCCTCTTGCGTCTGGCATCAAGGAATCGACTCCTGAGGATCATAACAGCTTCACATGTCGACTGCTTCAATTGCATCTGGATACTAGAGAATTTCTTGTGAATGATTTGGACCATCCTCTCAATTtCCTTCGGCGTAaTTGGCCTTGTGCGGCTTTGTTCACGTAaCAGGTTCATCACATGAGTCGTGAACTCATTGCATGCCTGCTCGTATTTTtCCAACTCCTgATGaTAAATTTGCCTGATTTGCGCAAGCTTAGCACGATAATCCGAATGTTCAATaGCGTTGTCAGGTTGCCCAGGAGGTCCAGTAGCTGCTGCTGCGGAAGCTGAAGCCGCAGCACCAGCACCTCCACCcTTTTCTGGACCAGCTACACCTTCTGCTATCAACAtATTGTCTAACCTCATCAGCTGAGGATCAGGTGGTTCCTCCTCCTGTGTATTCcGtAaGGagaGT

>contig01033

CTCGAGGGCCATCAAGGTGATATCTTCTCCCTAGAATTCCATCCAGATGGG

>contig01034

AACTTCCATACAATCGCAATCGTGTCGCTGTCGTTCGATTTCGGCACTAGACGTGTGCGATACTAATTGTAAATTTTTTAAACGAGTTAAATAAGGAATGAGTGAGAAATCATGACTAGATCTTGAAAAATCGTGCTTGAATTTTCATCAATACTTTTCCtACGTCGCAAAaTCCGAGCAGACGTGAATTTAGTTAATCAAAaTCcGCTTAAAGTCCCGATATTGAGAACGACACGACTCGATAATTGCCAAATTCGAATCATTtGGCGTCTCGCATCTACAAaGTGACTTtAGACACAACCGTGACTTCAATCACCGGCTTGAAAAAAACATGCTTTTCGTATTTTTTCTCTGTGTTTATAAACATTAAGGACTGCGATGTGACGCGTCGCATCTCATCTTTCTAAAGGATGCACATTGAACGGAAATTCCGCTGTTCGAGATATTTATATTATTCGATTCCTATACGT

>contig01035

TCAGACTGAAAGGCTTCCTCCACCTGCATCAAAAAAaGCTCTCGAAAACTTAGAAGAAaCTAAAaTTACTGAAAAAATAAACAaTGCCCAGTATGTCTAAAGGAGATtGAGATTGGAACTGTCTCGAaGACTATGCCTTGTAAACATTCCTTTCACAAaGAaTGCATCATGCCGTGGCTGgAAAAGACGAATTCATGTCCACTGTGTAGaTATGAACTTCCTACAGATGATGAAGACTATGAAATTTATAaGAAaGGAAAaaGTAAGAGCAGTTCAaCGGGAAAAAGATATGGAGACTTTGCACGATTCAATtGTTTtCTTAGTGCtAATTTtAGGGAATtATTTTTtAAAGTGAATTCTACTGTGAATAAGAATTTAATGGTCAAAATAATGTGTTCAAGT

>contig01036

GGTCCATCTTGAATCTGCACTTCTtCTGCAATAGCCTTAGCTTCTtCcTCGGTAtGAGTAACGCcAATTAGATGCCGATATGCAACGTATTTtAAACTGTGGCATGCTGAGCATACATTTTtATACACCTCCcACCcTCGGCGCATAcTTGAATGGTCTAATGAACTGAATAAaCCACTAAAACTCCACGGATAACTAGGTGGGTGAGCTAATAACTCATCTGC

>contig01037

ACTCAGGGATCAATTCTTCGCAGTTGTCCATGATGAAGACTCTACGAACGTAAAGTTTGATATtGTTTTTtCGCTTCTTGTTTTCGAACAAGTCAAAAGGCATACGTCGaGGAaCAAAGAGCAGAGCTCTGAATTCTAGCTGACCTTCAACAGAGAAGTGCTTtACGGCTAAATGATCTTCCCAGTCGTTGGTGAGAGATTTGTAAAATTCACCATATTCGTCTTGTGTAATTTGGTCGGGgCTACGGGTCCAGATTGGTTTGGTCTTGTTCAACTCTTCGTCTTCTTCGTATTTTCTTTAATTGTCTTCTTTTCTTCTTTTCTTTGTCTTTGTTTTCCTCATCTTCGTCTTCACCAACATCTTCTATCTTTGGTTTTCCGTCGTCTTCCTTCTCTTCTTTTTtATCTTCAGTCTCAGCTTCTGCCTCGTCGTCACTCAGTTCCTTTTCACGTTCCTTCTGGACAACGAGCTTGATGGGATAACCAATGAACTGTGAATGCTTCTTCACGATCTCCTTGATTTTACTTTCTTCAAGATACTCAgCCTGaTCTTCCTTGATGTGAAGGACGATCTTtGTTCCaCgtCCAATTGGCTCACCAGGGTCAGgCCTAACTGTGAAGGATCCTCCAGCGCTGGATTCCCAC

>contig01038

ATACCGGATCCagTACCACCtCCCAGTGAGTGGGTCAACTGGAATCCTTGAAGGCAGTCGCAGCTTTCCGCTTCTTTCCTGACGACGTCTAAAACTGAGTCGACTAGTTCAGCTCCCTCAGTGTAGTGACCCTTGGCCCAGTTGTTACCGGCACCACTCTGTCCGAACACGAAGTTGTCGGGGCGGAAGATTTGTCCGAACGGACCAGATCGGACAGAATCCATCGTTCCGGGCTCCAAGTCCACGAGAATGGCTCGGGGAACATATTTGCCTCCAGACGCCTCGTTGTAGTAGACGTTAATTCTCTCCAACTGAAGGTCGGAGTCTCCATGGTAGGCACCGGTCGGGTCAATGCCATGCTCGTCACTGATGATTTCCCAGAACTTGGCTCCAATTTGGTTTCCGCACTGGCCAGCCTGGATGTGCACGATTTCCCTCATCTTGTTTGCTGAACTTTAGTTTATTTGTCGATTAAAGAAAACCGAAGTCTAGAAAATTGATAGAACGTAACACTTCACCAGTCAACCAACGAATatctCCCcATGT

>contig01039

CGATTTTCGTTtAAAGTTTCAAGTGTGTCTTGAAAAATCtGCACGACGAGATAAATaTATTGTtGTGGATTCAGAAGGAaCAaTAAAaGCAAAaTGCTGCATCGACATTGtCATTCGACATTTATCTGCGGTTCCTTCCAATCtCAaTGTTGTAGaCAGATTCAGCATAGATGTCGAaGCATaTCCAaCG

>contig01040

ACCAGGCCGAAAGTGGAAATAGTGACATTTTCCTTTATCCTGTAGCTATATACAATGATCCATTCCGAAGAGGAAATAATAAACTGGCTCTTTGTCAAACCTTTAACGCTAATAAAACTCCATTGAAGACTAATAAACGCTATAGTGCAAAAAaGGCTATGGATGCTGCGGCAAAaGATGAACCTTGGTtCGGAATAGAACAGgAATATTCTCTTTTGGATATTGACAaTAGGCCTCTTGGTtGGGgTAAAAaCAGTTTTCCTGGCCCACAAGGTCCTTACtACTGCGGCGTAGGAGCGAATAAaGTAATCGGTAGGGAAATTGTTGATGCTCATTATCGAGCCTGCTTGTATGCTGGAGTCAAAATATGTGGAACTAATGCAGAAGTGATGCTATCCCAGTGGGAATTTCAAGTTGGACCGTGTCATGGTATCAGCGCTGGAGACGATCTCTGGATTGCCAGATTtCTTCTTCaCCGAATTGCTGAAGAaTATGGCGTGGTCGTCACTTTGGATCCAAAACCTGTAGATGGTGCTTGGAATGGAGCTGGTGCCCATACGAATTTTTCGACGAAAGCTATGCGAGCTGAAAATGGTATCATTGAAATAGAGAAGGCAATTGAAAAACTCAGTAAAAGACATCTGGT

>contig01041

ACTCTTTACATTTGTTGCGGGAAATCCTCGGGAATCCATATTTAAGGCAGGTTGCAGGATAATCTTCTTTAAAGCATTGGGGTCTTAAGATATTAAAATACGTAATTCCAATTTtGGTAGCAATTGGAGAATGAGCTTTTTTTAGGCAGTGGTAAAaTTCATCATCACAATCACAATGAGATCTTGTAAaCAaGCCATCGTtaCGAAgaCCTTTGGCTGATCTACCAGCATTGATAGTGGATTTACAATGATCaTGATCTCTACAACaGGCaTCAGTAAcTTTAAaGAAACCGATTtCTTCCTCACtttctGATGAATCTCCAGCTCCACACCATAGTGTTCCTGGGTAGAGAGCTCTGAAATTATCTTTGAATAAATTTAAGATTTCCCCAATTCCAGACGAAGTCAATGCTCTTCCAGTAGTTTTCCCAACAGgTTTCAGGATGTTTGTGATAGGTTTTGTAACGTCATTTATGATTTTTTtGACACCGGAACTATTTTtAGTCGAATTAGATTCTGCAGATAAaTTTTCTAAACTCTTGGCAAGCGTTTTTAAGAATCTCTCAGGAAATGGAACATAAGTGGGAAGCTCATCAATTCTATTGTCCACGAAAGaTTCATCAACTTCAATGTTCGAAGATTCTGTTTC

>contig01042

TCGAAAATGCCATTCCACCAGTTCAACCAGTTCACTGGAAGACTAAAGCAATCAAAAAaTTtGCAACCTCTTTATCCAATTCCACCGATTCCTCTGGATAATTCAGGATTAACTGAAATTATGTACGACACAGCAAAAaCGAAACCAGTGAAAACTGGAGAAGCTTTCGCGGAAAAGTCTAAGATTCCAGAATATCCCCCTAGATTCAGCCCAAAGACCATTGAATTCATGAAAAAGTTaCAGGCTGACATAACAGCACCAGTGTACCTCGCCAAGGGAACTCCTGATAAGATTCTTTTtGGTCTTACGTGTGCTATTATTGGTTATGGTATGTTTGAATCGCTAACTTTTTGTtGGAAAaGAGCCACTAAAAGAATTTAAGTAAaCATGATCAGTAATGATGTAGATATAT

>contig01043

ACtACGATTTGACGAAAAAGTAGTAATAATAATAACAATAAGGTGTTTTAAACGaTGCTAAGACTAGCGCTAGAGGGTTCTTCGTTTTCATTACCATCCGTAAGAAAATTAATGGGGCGGTGGTGGGTCTGTTGATTGGTTTCGGCCATCAATAGTCCTAACAGATTTCCTACGTGCACCTCCCGTAATGCCAGCGGAATTTACACCACTtCTAACATAAGTAGTTGCAGAAGTAGATATTtGTTCTCTGATAGTtCCAGTTCCTGTGTTTCTAAGTGGGAaGGATGCTGATTGCTGTAaGACCATTGGCTGAGGTATCACAGTTTGCATTAAAGCTTGAACAaTAGATGTGTTTTGCAATGGGATTTCATTGGGATTAGCATGTAGATATGTATTTGATGTaTCtGTTGCAGTATTGTCAGTTGAAaTTCGAACACTAGAaGGTTGtAGTAGTTTTtcATTGCAATGTATAGGAGGCTGTCCGAATTCAGTTTCTTGATTTGAaGTCGAAGTTGCCGTATGTTTTGTAGCatGGTTTCTAGTCGAAATATCAGATCTATTTGTCGGCACATTCAGGTtGGATAAGAGTGATTCTGAATTCACTTGTTGCTGGTTACCTGTCACATTCATTAACGCCTGAGAAAGAGT

>contig01044

GTTtCTTGCCCAAATCTGCAGAGGCtGGCAGTGTTTATATCTTTGCCGGGTtGTTTCTGTtGTGGAGGTTGGCTGGGAGGAAcTGGAGTTtGTGGAGTAGAAACGGGgTTGCCACTCTGAGTATTTtGCTGCACCATAGCCACTTGTtGcTGCTGCTGCTGCAATTGCATTtGTtGCTGCTGCTGTTGCAAATGCTGTATCTGTtGTAATGCATTCGATTGCTGTTGCTGCTGAGCCAAAACCTGTTGGGCGTTCATCATTCCCATGGTATTGGAACCAACACCTGTAGTGACACCAGTTCCAAATTGCTGGGGATTTACCATTCC

>contig01045

TTGTTGCGGACCCATCG

>contig01046

ATTTtCGCTTTCTGATCGTCGAACCCATGAGAaTTCTTCTATACGAATCCCTATCGATTCCCCAAAGTTTTACGTCTGTTTTTGCTCTGACTGTTGCTGCTCGAGGTGTTCCATAAATCAGGGCCAATTCACCAAAaCTTCCACCTTCTCCAATGGTAGTTACCAACTCACCATTTACAAAAACTTCAACTTCCCCTTGATCTATTACGTAGAAATTATCACCTTCATCTCCTTGACAAATGATGGTTtCTTTTGGCGAGAATAAAACAGGAAACATGGCATCGAAAATGTCTGAACGTTCTTTCTCGTCGAGGTGAGCGAACAAAACATTTTTGGCGATTGCCTTGCTCAGAGCATTCATGGTCTTATAGTCTTTAGGCACAACCTTTTtAaCGTAGTTTGTaGCAtCTTCTTCGGAGACAGGTTCGGCGGAAATTCCGCCTCTTCGTCTAGGAACCTGAGGACCTTGTTGTCCTGCTGGCATGTCCTctGTGTCTTCTGG

>contig01047

AAATTTAGGATTGGTTGCTCACAGCTCTGAAAATCCAACTGAGCAAAAATTGGAGAACGAGCCTTCTTTAAAACCACAGGAATATTACCCAAAAGATCAGTCTAATGTGAATCTTCTTCAAGATTGTTCCAATGATTTGAATCAAGGGGTTGTTTATGATGATAAACTGAGAATAAAGTGCGTCAGAGAAGAAaTCGATAGCGTTCcAaGGATTCATAT

>contig01048

ACATTGATtGTTTATTCGAGAAAGGTGCTTGCACTGCTGATGCCCGTGCCCTTAAAAGATTCTTGCCTGATGCAATTGCGACCCACTGTAGGAAGTGCACAGAtATTCAAAAGAGCAATGCAAGGAAAGTTCTCAATCACTTAAGGTTTGAAAaGCCAGAAGTTTGGATAAAACTGATGGAAAAaTACGATCCCCAAGGCCAGAACACACAAAATTTCGAAATCTTTTTGAATGGAGAAaCGTCAGAAGAATACTTTTAAAGTCACAATTTTCCTAGCAAAATTTATTATTGATTTTTAATAATGTTGAAATTAAATTAAaTTTTACATTGAATTAATGCAATTATTACATaCTTGGCATAAAATgTTCGAaCAAAaTTAATCCATATGCTTCAATTTATATTTTtATCTTGTAAATTAATC

>contig01049

ACCATCATATAAACTCCTAGTAGTAAGGCAATAATTTCTTTAACAGACTGCCAAAACTTGGTATCGGATAATAATTCAGGAATTACAGAGACTGTGGCTATGTAGATGAAACCACCCGCAGTAAAAGGTAAAATCCATGATGTTGCAAAATCTCCCATTCCTTCAGcTAGTAATGAAaTGTATGTtCCCATTAACGCACcAAGTGCAGTTGGTAATtGTAATAATATAGCCTTTTtCTTGCTACAGCCACTTTGTATCAAAaTTGCGAAATCACCTATCTCGTGAGGCACTTCGTGCAATAATATTGTAAACGTCGTCACATAACCAACACTTtCTCCTGCTAAATAACTGGCACCAATAGCCAAaCCATCTGTAAAATTATGTAAGAAATCAGCTGCTAAATTTAAGTAACCAGCAATACTTAAATCCACATTTTCGTGGgAATGTTCATGCGTGTGAGCACTATCTCCTTtAATAATACGCACTAGTTTCTCtACAaCGAGGAATGTTACGATTCCTagCAAAaCACATAAACCTACGGTCATATCATGTCCAtGATCGTGTTCTTCGTGATTATGaCtGTGACTATGACCATGATTGTGCAAAtGAgAtCCGTGTTCTCCTTTGGAGTTATGtGAGtGAGGT

>contig01050

TGCATTATCCAATCCCACCATCACAATTTtGTGCTCTTCATTTCCGAAGAGACTCCATAGCTTTGAAAATAGAagTCCCATATtGGTGTCACCGTTCGTGACGTATTTGGACACTGATGTTTACTTTAGAGTTATCACAGTGCGATCTCGTTCACGATAAAAAaTCGTCGCACTTGACTTTGGATTTGGAACATTTCGTAATCAGAGGGTTATGTTCAACAAATC

>contig01051

ACTACTTATAGAACTAACAATTGCTATAATGACCTCTTCCTTTACTAGAAAACTGCCCACCGGGTATAAATCCTTGGTTATTCCATCACGATTGATAAATTGAATCCTTCTCTGACGTTTGAAATTAACCTCCTTTTTTTGGGGCACTCCCTCATACCATCCAATAAAATATCCATAATCACTGTCCACTTTCTGACATAATTTATAAaTGGGTTTGTTTATCACGTAAATCGGTTCTAGCTGAATAATGGGTTTCACAATTAGAATACAGGTTTCCGTAATTGATCCAACGCCTGTTGCGTCTACAaTAATAGAATTCTCCATTCCGTCTGGATTTTtCTGTCTGgATCCaGTGTAAAaGGCAaTGGTTTtGAGTGGTTGGAGCGTGTCAAGAAaTCGTTC

>contig01052

AaaaTtcGGATCGTCGTCAGAGCCGTTCATTTTATAGACACAAATTTCATCGTGTTCTTGCTTAATATCTGGAAAATCCTGTTCGCGCACGTCAGATCCAAAAAGTGTTGGCACGGCGTTTCTTCTAAGTCGCCTTTTTGTGGTGTGTAAATTTGTAAAaCTATCCCTTGTGAAATGATCACCACAAACAACTGTATAGGCTCCTGGCATAAAATTCAAGTTTGGAATTGAGTCCAGCCACTTCTTTAATAAATCAGGCTGTTTTAGAGGGAATTTGTGAAATGTTACAAGATGTCCAGGTTTCCATTCTGCATTACAAGTAGAAACACAGCATTTGTTGACCATTATTGGAAATCACTATAaTTTCTAAATACTAACATATTTGGCAAGAATATAAAATATCAAAAGGACAGATGAAaTTTATGCATTTAATGCAAACTAAAGTATCTAATCGCAAaCGACTCCAAACCACTTCAAACCgCtCCaCAAACACAG

>contig01053

TTAATTTGAAAGCACATTt

>contig01054

CACCGCAATCTAACTACCGCGACCTGCATTGCTTTtAATTATTTTAAAA

>contig01055

CTCTTGGAGTGTAAAAGATGAAACACATAGGAAAaGAAACTCTACCATCTGCGTGCTCCATTTTGTAACTATAAACAATATATCTGGGCTGGTGAGCAGGTATGAGATCTTGCACTTCATCTATTTGGATATCCTCGACAAGTTCATCGATACAAATCTTTTGTTTTTCTCTGTCCACTTTTAGTATAAGAGCAGCATTTGTTTGACTTTTTCGAAAACGAAACTTTTTAAGAGCATCCTTAACATCGTCTTCTATATCACATATCTTGTTGGAAGACATATTCCTGTATTGGAAAAAATTCACTGTCAGTCTTCAAAAATACGTTAAAATGTTCAAGACCAAATACGAATTGGTAGAATTGTTATTTGTCAAGTTACCAGTTGATCACAATT

>contig01056

ACCGAGGGGAAGTCTATGTGGAGCAGCAACAAATTAAAACACTTATGCAAGCTGCTGAAGCCCTACAGGTTAGAGGCTTATCAAGTCAGGGACGAGATAGTAGTATGAATGACACTATGACTTCCCAACTTGGCAACTCTACTACTCCCATCGAAATGTCTCCTCAGAAGCCGGACgaTGCATCTtCGTATAAAGGTGACGAGACATCATCgAATGACGCCAaCAGCATTAGCTTCCTCGAAACCACAaCAAATTTtGGATCTAGCGTtGCCAACTCTCCACAgCTCTCTACATCAACCGGACCTCAGcAAAAGCCCcTCACtCCAtCCAATTaCCTAGACGCTGAAaCGATGCAGGATTTGGAAAAACTCTTAACGCGGTtGAAACTACTCTGACTGAAACTCCTGGCATGGTGAAAATGGAACCcGATGAACAATTTATGCAGCAAGAAAAGCCTTATTCTATCAGTATGGTATCTGGAAaCTGTAA

>contig01057

CTGATAGATTTGTTAAACCAGCGATTCCACCAACTAATGTGAGCCCAGGTGTTGCTACAGCAGCTAATCCAATTCAAGCGAATTCAACTGGAGGGTGTCCAATGGATATTGCGAAGGCATAAGGCAACTACTTGTAATTATGATAAAATACTATCGCTATTTTTCAAAAAGATATGTGTGtATCTAAAAGAATCTACATGCaCATACAATACATATACACAAACATACCCACACACGCATGCACATGAGCAGATATATGGACATGATTACGAGATAATTTACTTTATCaGTAGATATGCAAGAAAAaTTGATCAaTACAAGATAAACTGCAaCTATTGTTATTTtCAAATTGATTAAAGTGTTAAGGtAAAAaTCAAGTGTGCAAATTaTGTATATCTCGGTGAAGTCGCTGAGAaTAAAAGGATAA

>contig01058

ACTCTCAATTGAGCGCCACTGAACCAGGCCGTGCTATTAGAGTAAAGGCAAATGATATGTTCGCCAGGAGTTTGAGATGTGAAAGAAaTTCGACCCTCAGAGCTGTAAACTCTTGAAaGAATTaGCTTCTCGTCAGGATCTTTCACTTCCACGTGCATTCCAATTCCAGGACTGCTTGGCATAAATCCCTGGCTTCGTGGGTCGTAGAGTTCTACTTTGTAATTCACTAAAATTGATGTTTCATCTGGAATTTCTTCTATGAAACATTTtCGCTCCGTTtCGCCAATGTGGAAGTAAAGACCAGACGTATATTCAAAAaCTaCACTGAGCATTAACaGAAAaCTTGAAAGTTTAAGCATGTTtGTTTCTTTGGAAAAaGTTATTCACAAACTATCTaTTCA

>contig01059

CTtGGAtGTTAAACAGCGTCTTAATGCCGCTGTCACAATCAGATCTCTCATACATCCTGACAAATTCATCATTGTTGTTGAACATGATTTGTCAGTTTTGGACTATTTGTCGGATTTTATATGCTGTCTTTACGGAGTTCCTGGAGCTTATGGTGTTGTAACAATGCCTTtCTCTGTTCGGGAAGGCATCAAtATTTTTTtGGATGGATTtGTtCCAACTGAAAaTTTACGATTTAGAGAAGAATCGCTTGTTTTtAAAGTAGCAGAAAGTGCCAcAGAAGAGGAAGTAAAGCGTaTGAATCATTATGAATATCCTGCCATGaCCAAAaCAATGGAATCTTTtGCTTTAAAAGTAGAAAAaGGTCAGTTTACTGATTCtG

>contig01060

AATTTACCACCAGACGAT

>contig01061

ACCTTCATCCTCAATTTGCGACAGATGTTATGAAACCATTAAGAATTGATGATATTATGGATCAAGAAGT

>contig01062

AGCATTTAGATTtGAGAAAGATGTTGgTTTCTGGTGAATGTGATACTAtGTGGAAGAAGTTTGTCTCCGTTAtCCCACAAGTTACcAGTCTTTTAAAaCTAGAATTGTGTCGATGTTCAGAGGTCGTTGTAGAAGAAGTAATTAAAAACTGTCCGCAGTTGGAAGTTTTGAGTGCTTTGACGATAAAGTGTGACGCCTTAAGCTTAGAGTCAGTAGGGAATCTTAAAAGATGTCATGAATTAAGACTGAAATCTGCCGGAGGAATGCAACTTCAAGGTGACCTTGCACCTTTACAAGAATTAaCCCAATTAACACATCTGAGCCTTACGTCTaTAAAaGAACTTGGCAAGAAGAAGCTCGACATTTTGCAATCTCTAGTAAACTTGGAGACTCTTGAACTAGGAGAgTGTtCTGATTTCCCTGAAAAATTCGGT

>contig01063

TGACGAAAGCAGATGTAGTCATCGTGaTTGGCAAaTGTGATAACTCTTTTGCTGTCCTCCTTCGGAACAGGAAATAAATATTTCAATATATCCAtCGTCCTCTTGGCTAGTTTCGTTTtAAAATTATGAAAAATGAGATGAGGATACTGCTCAGACATCGTTCCGATATCGGGAATATCGTGTCGCATTACAACTCCCGACATCGTGAAATATGCGGTTGGA

>contig01064

ACCAAAGGAGGAaGAaTCGCTGCAAGCTTGGCGAAAAGCTATACCcATAAAAGATAAAGTATTGAAA

>contig01065

GCCATCCTGGAATCGGTTTTCCCGCCCAGTCAGCACTTTCGTCAAATTCTTGTCTAAATGGTGCATGAGGATCAGGTTCAGCTAAAATGTATCTATATCCATCTTTGTTAAAAGGATGCTCTAGAGGGTATCCGTGTGCTGGGAGTTTTGGAGCTGCCAAGTCAGATCCTCTGCCTTTTTTTCCTGGTCCTGTTCCAGTTCCTTCTCCAGGAAACTTGCGTTTCGCATTGCGGCCaCGAaGACTTCCAGTGAGCGGGACTTTTAtCATAATTTCGTAATTtGGTTTTATCAGCACAAGGTCTGGAGTTGCGAGGCCAAaCAATTGACCATCAGTGTTATTCTCATCCATGGTAAAAaGGGTTCCTACGTCTTTCAATaGAGCTTTGTGAATTGTTGCATGCCAAGACTGCGTAACTCTGCGAGGCATAGTTGTC

>contig01066

ATCTGTAaTCATTGTAATTTATTCACATTTTtAAGGCGTGGGATTATTTtCTGAACATTtCTGTGTATTAaTTTATCAAaTTTTTaTTtAaataagAACACTATTGATCGAAATGTCTGACGAagAaCTTCCAGCCGGGTGGGAAAAaCGCCTCAGCAGATCtACCAATCaGCATTACTATTTAAaTACTTACACAAAaGAAAGTCAATGGGATaGACCTGATAAaCCaGCaGATCAaTCTGGAAATGgAACCGaCAGCGGTCCTGAAGAaGTCCAATGTTCACACCTTTtAGTCAAaCATTCTGGATCTCGTCGACCGTCTTCTTGGAGaGAAGAAAaTatCaCTAGAaCAAAAGCAGAAGCTTTAGAACTTATCAATTCTtATAGAGAGCAACTCACATCTGGAAAAGCAACATTTTCTGAaCTTGCTTCAAAATTCAGTGACTGTAGTTCTGCAAAACGTGGAGgCGACTTAGGACCTTTTGGACGAGgTGCAATGCaGAAaCCGTTTGAACAAGCTGCTTTTTCTCTAAAAGTTGGAGAACTTTCTCAGCCTGTTCACACAGATAGTGGAATTCATTTAATCCAACGAACTGCTTAAAGTCATTATACATAAAACCTGAACGCAGAATTATTACCAAAGT

>contig01067

AtGGGgAGACTCCTCGGaTCACCTCGGATCTTACGTCCAACAATTTAATTCTTCTTTCTGCTTTGGTAAAAAaTAAATTTAGTAATAATAGCTATTCTCTATAACGTTTGAAACATCCTTCTGGGCTGTCGCCTAGTTAGGAGAGCCAATATCTGTTCTGCGGCGGCTATGTGCAAGTCATACAGTTTTTAAGTTTTGGTCAAGAAGTTAAAATTATAGCAAGATGGTTTCTGCAACTAAGACAGGGCCTCAAGATATGCCTCCTCCGGGAGGCTATGGTCCAATACAAACTGAAAGAGTTAGAATAAAAACTGTCTTTACAGCTCCACGTATTATTGGCTTGATTATATTTAGCCAAATCGTCGGATGGACTATTTATAGAACAACTCACAAGCGAATTCAAAACCAAAAGCTTGAAaTGCGAAGTGCACGGTTTGCATTAATGCCTATGCTGCTTGCCGAAaGAGACCG

>contig01068

GACACAGGCATCAtAGACTCTTTGAGTCTTTCGACACGGCGCAAAATAAGAGTGTCCACTCGATTTGTCAATGCAGTTATAATATTGCGTAAACTCATCATAACAGGATTTCTTtATTTTtCTAAAGAAATCCAAAGTGCAGGATGTCACTTCTTTCCCGTAGTCTATGCATTGTCGACAATCATCTGGATATTCTGCCCGACATAGCATAAATTCATTGTTTTGAGGTTCACAATATTTTCCAAGCTGAAAAGCTGCGGCATGAAGAACTGGATATGTCAGAGGAACTTCTGGAACAGTTAGTTCTTCGTCATTAGGAAGATGATGATCATCCGTCACGACCATAGTTTTATTCTTATAACTTTACTTTAATTATTAACAGAAAAAAAaTCTTAGCGTTTTTAATTCTTCAGAGCCCAAAATCAGGAATT

>contig01069

ACCATAATGCATTCAGGCAACACATTATTGCCCAAATAATCATTCCAACTCTTATAAACTCTCCCTAAGTCATCAATATAATAAATAACTAATTTTTTTGTtCACTCTCTTGCCATCTTGGTTCTTAATCACATTCTTCAAGACCCTGAATATTGGAACAGGTTGCAGAAATTTGACCATATGCTCATCACTAAAaCATTTCCCAGTTTCGAAGATATGTTTTAAAGCTCTTATAGATTTCATATTAAATGTGACATTATAAACTAT

>contig01070

AGAgaCATCCTCAACGTTGTTCAGA

>contig01071

CCcGATCACTACAACGAGACTTGCATGTTTAAT

>contig01072

TATAGACTGTGATATAATTATTCGAAAAACTCAATCCATCTTCTCCAAaTGATCGAGTGGGTTTGATTCCTGCGAAAGGATTTTTTTCAGTGAATGATGGACTtGCAAGACTACCAAGAaGCTGAGTCATCTtCTTTTCTCtATTTCTGTTGTtAATTTTGTTCTtGTAGTTGCAACAAaCGgAGCTTcAaCCTTTGTCATCTTCAaCATCGATTCAGTCcaCATGTGAAATTTTTtCCcTTCGATATTATAAGCAATGgTAaTGTTCGGaGAAACAAAGgaTTTaCTGTCAACGTAATATACATAGGGtATTTtAATTACTTCAATTtCgAAaGtCTTGTTCTTCTCCTTACAGGTGCTGCAGATGTTAACGGAAGCTTTCATCTTCATGGATAGTtCTTTTCCAATCATCATAACTTTGAGGGTCGTGAGCTGCGGAATGAAATGTAATAGCACctCCAAGCTACCGAAAGTTGCTTCTATTGCATCGTATGCCCCTATGATATGGACGATTAACTCCGAGTTGTATTCGAATTTGAGTTTCTCCAGAATATACATGAGAGTTAGAGGTATAGTGTAAAGTTCCGAACATAGCATTTTCCACAATTCAATCTTGTTTATGGAAATCAAGATAGGTGGT

>contig01073

TCTAAAATATACATTTACAATTAAGAGAGGGAGATCACGTCTTACGCGCTACGCCTACCCAAATATTTACAAATTTAATTCTTGTTATATAcAaTTTAAaGATCACTTTGAATAGGCTCGATCGGCCTTGGAACTAACCTAACATTTGGTTACAtAAAATTCTCGAgTTTTCAACTCGATTCTACATACTGGgTTCAAAGTTGCaTTCAAAGACAGGTTaCGACATCATTTtCTAACTTCTCTTAACATTTGGAATTATTCGCATTTATAATTACTTCGActCGCATTAATAACATTCATAAAGTATGATCCTaGGAAAAAaTTATCGTAACCTAACTTTTGAATAATTTTACACCATTTCAAAAATGAATTTCGCTGCGCACTTCTACCGAGGAAaTACAAGTtCTGCACAtAGTTTCgTGAAAGCTACAAACCGATTGTTACTCtCCAATTGAAAACAAACTAAAAaTCGAGATATTAACATGTTTCAATACTAAAAACAATCTTTGCAAaTTTAAAAAaCGAAGaCAATAAATTAGAAAATATTCCAGATCCATCAAACTtAGTCTCGAAAATCCAACGACCAAGAAGTCATAAAaTTAATAAATTtGCCaTAAAAAAAaCATAAACCCGGAGAATcaTT

>contig01074

ACTCCTTACTTGAGACCTCCATTGCGTAATTGAGTCTTCACGGGAGCACATACGCTCTTTGCAGCGAGTAATATATCACTTTAAACACCGCTCAAAACAAAATTGTCCTACGTAGCCGCCCCTCATAGCTTTTGTAACgaTTTTGCTCAAATACTCTACGATtGTTTTGCAGCACCTCGGCCGCATCTTTGTTAAGTGGGTCTTCTGGATTtGGCTCCAGGAAAAGATACTGTAATCCGTAGACAATCGAGTTAATGGTAAGGACTGGTTTCCAGTCTTCTCTCAAAaTATTCAGGCATACATTTCCTTCGAGATCAATATTTGGATGaTAAACCTGCGTTTCGCATTTGACTTTtGGTGGTTCATGTGGgTAATTGGgTCCAACTTTAAAaCTAAAAaCGAaTCTCCcACCTCGATAAAaTCCCTCATCGGGGCAGATAATTATTTtAAAaCTTAAGAGATCATcaGGATCAGGATGATCCAACTGACATGTTTTCGGCAAATtAaGTTCGTTCACATCTTTCATAATCCTaaGTtGTGCTGCGGAAGCTTTCTTTTGTGTTCCAGCTTTTGGaGATTCTCCATCCTTTTtCGCTTGCTTTAAGGAAAATAATCTGATCATGTTAGGAAAGATGCGTCGAGT

>contig01075

tcaTttAatgATGgaGaCTGATGCTACGAGTTCTCAATCACAGTCAGATAATGAAGATAATAATGTCAGTTTCAATTCAATAGATGATCAtcTAGAAATTGTTAAACATGAAATTGAAATCAAAAGAGCTCACGAGCAACTtACGGAAGATTTATTTGGTAATGACATTATGATTCCTCATGTtACTCCAAGTAGTATTAAGGATCGAAGGGAGAGCAAAGAGAAAAGtAAGAGAGAGCTGGAGGAAGAAGAAAGGGAGAAAATGCAAGTTTTAGTATCAAATTTTACAGAAGACCAGCTGGATAGATATGAA

>contig01076

ACTGTCATTGCCACGACTCCTCCTTTACTGGCAGAATAGGCAGCTTGTCCAATCTGTCCCTCGTAAGCTGCTACGCTCGCGGTGTTGATTACGACTCCCCGTtGTCCATCCTCATTTGGAGTATTaTCACCTATCATTCCAACCGCTAGTCTCGTAaCATTAAAGGTGCCTACAAGATTtAcGTTtACGACTCTTTGAAAATCATCCAGCAAATGAGgCTTCTTTTtGTTGAAATTGTATACTTTAAAAGCAACGGCTATACCAGCAGCGTTGACCAGAACGTCTAGCTTCCCAAACTTTTCTTTCGTTGTATCAAGTGCCTTCTGCACATCAGCCTCAGATGTTACATCCATAGATGTGAAGATCGCTGAATCGCCAAGGCTCGAGGCAAATGAAGCACCCGAAGACGAGGACAAGTCTGCTATAACAACTTTTGCACCTAGTTTTGAAAATCTTTCTACTGTTCCACGGCCTAGTCCTGAAGCTCCTCCAGTTACCAGCACAACAGCATTCTTCCACATTTTTtATATGCAAAGAAATTTTTGTTACAAAAAAaTAAATTGAGAGCAAGTAATTATATAAGAGCTT

>contig01077

AGTTTtCTTTTtCCAGGTTTCTTCAAATTATCTGGCGAAGAATCCTCTGGTTTTGAGCTCTTAGTTGACTCGGTTGTTGATATTTTGTCGGAAGATTCATTGAAAGGTTCAGCTTCTtCTGTCCcAGAATTGCATAATGCAGGATGTCTtAATAACTCTTTCGATTTCAGcAACCCCTTTTGGTTCGTTCCAACACTCTCAaCTATCTCATtCTTTTTTtCTGATGCATTGAAaGTTTGCCCAAAaTTTAAATGACTGGTAAAAGTATTTtGCTTAATTTGCGATTTCCATAAGAAAGATTTACTTATTGGTAATGGAGTGCCTTCAATACAACTCGATTCTCTATTTCCATTAGTAATATTCATGCTTCGAGAGCTTCTTGATGTTGGAGAtATTTTTTtCTTCcTGTTGAAGGTTTTATAAAaCAATCTTTCAGTCTCTTCACAAGTGACATATTTTCTAGAGATTCTGATAAAGATTCTTCTATTTGAAAAGTTTCTACTCCTCCTGACTTTCGGTTTGTTCCAGCTGGGCTTGATTTtCGTTtATTTCCTGCtGGGCTTGAATTATTCGAGCctGAACTTGATCGATCTGTTCCTGGAGTTTtCTGGCTTCGGTTAGCATTCGATTTATACCGT

>contig01078

ACGCTTGTAATTTTACTACTGATTGCGGGAATCGCTTCACAACAGGATTTTATTTCGCCTTCACCGGACCAACTCATCGGCAAAATGAAGACTTTGATGAGGACTATAAGTTGTGGAGCTTCAAGTTCAATAGACGATACTGAACTGCTGCTGAAAGCGCGTGCTGTTGCTATAGTTCTCGAGGGCGGTGTTGTATGTAACGCGGTCGCTGTCTCTTCCAGGGATATAGCAATTCCAAACAAGTGTTGGAATAATTCCGAAACTGTATTATCTGAGTTGGAGGCTCAAATGCAACATCCCGGGAAaGATATTGACCCTGATATATATAAATTATGCTCCGTTTCAAAGGGATTAGGTTGTGATGAGAGTATTGACATAGTAAAACtAGAtAGAGACATTtATTACACAGGtAGTTTGAATGATAAATTCCATAAGCATTTAATGGgTAATAAATTCACAAAaGCAGTAGACACCTTCATTACTTATGGACTATCATTGCATCAAATTGAAGACTCTGAAAAGAAGCTGAAATTGGAACACCATTTaGAaGAAtATAgAAagCCGGT

>contig01079

GTATTTAATGACTGGTCGGGTTGAAGTAATGCGTGTATTTCAAAGAATCGAAAAACGCGGTCGAATTCAATTGAATGATCAAATTTATGAAAAATGAAAAGGACCATCTTTtGATAACCGAAGTTTCCAAATTtGTCATATCTTTATAAAACCATCCAACTATACGAAATTGGAACATTGGCGAATATCCaTTCTCCTAGCCCATGCAGCCTTATGAATTgaTCCTGTCGTCATGAAGCGAATAAGGACTTCgTAGTTACAAACTGCAtGAAACAAaCGGCTGCTTGTGTCAAAAATACTACACGaTcAGCAGATCGAGCAGCACTTAAAGTATTCCAtGTTtACTcAAACTGAGCAAaCCAaCAGTTtcACTCAAaCAGAACGAAGCAACTCTAGCTATAGTGACCAACGACAGCCACAACAGGTAGCGATGTTCGACCCACAGCAAATTCAGCAGCAGCAAACTGCTCAAGGTCAACAATCCCAGCAACAACAGTCACAGCAAA

>contig01080

GTGTGCATTTAAAAGAATTGGTCGGTGGAGGAAAGGGACCTGGCCATCTTGTAAAGATATGTCGTGCGAGACCAAACGTCAACAGAATATTGTTTATCTTACCCTAGATGCAGCCTGTGTTTTTtAATCACGGTTGATTCACGGTTTCGGTGTTCAATACGGTGGCGTTTTGATCCGTGGATAAAATCTGTGATTTTTAATTATGAATCGTGGCGACGGTCTTGTTCAACGACGTAGGGTAGTAAACAGTAGTAGCAGTGGAAGTGGCTTTGATCAGGAAGgCTCCAATGAGgCTGgAGGACATCCTGATAAActGTCAGCTCATGGCGTGGATGCTGACAGTTCTTCGCAAAAGGACCTTAATtCTAACCCCACCAATGATGAGGATTCTGATAAGGAAACTAGGTTGACTCTAATGGAGGAGGTTCTGCTGTTAGGACTGAAAGATAAGGAGGGGT

>contig01081

ACCAGTAGCAAaCATAAAaTCGAGTCAAaTATtCCAaTAAAAaaGTCCAACATCTCCAAATaCATTCTGaTTtCTCAGGAAGCTGGAAACAAATTCTCTTTTAAAAAACCtCACCCTAAATTCAACTCTGCCTTCCAGTAAGAACAGTGATGGGTTTGAGCAAAaGCTGAGGATTATGAGGAAGAGTCATCTGCCCCTGACTCATCGAAaCCGGTGGCGAATGTGGTGACaTAGGTTCCGGACTACTTCCCGGAGACGCTGTAGTTGAAGCCGGTTGGCTCATCAAAGGAGTCTGATTAAGATGATACCGAGGTTGCTGATTAAAGACAGCAGCCTGATGTTGATGAGCAGCCAAAGACCTCAAAGTAGTATAGAAATCATAAGGACTAGCCGTCGGTGTAATTCCCGGAGCCGTGATGCCCGCCGGGTAACCAGAAGGTGCCTGGAGTagCCTTTCCATAGAAAAGGCCCCGTGAGCCGGAGGTGCAGCCGTCGCAGGCAGTGCCTGAGGTTTGCAaGGTAAaCTCGGAGAAGTCGTTCCAACAGCACCAGGTAACAAATTAGCAGGATATCCAGCTGGAGTCGTATAAGCACCGCTGACTGGAGGATACCCAGAAGCTCTATGAAGATAAGGCAAAG

>contig01082

CTACAATTGCTAAGAGACCTTTAGTTGAAATTGGATCACGTTTATTGTCATCAATGGCACAAGCACTCAAAACCCACGAGGTTATCCCAGATGTCATCGACAAAATTCCTGTCGATGTTGTTGAAGTTACATATCCTAACAATTTGAAGGTTGAGATTGGCAAGGTTCTAACCCCAACCCAGGTTAAGGATCAACCTTCTGTTAAATGGAATGCTGAGTCTGGCTCTTACTACACTCTTTGTATGACTGATCCCGATGCTCCAAGTCGTAAGGAGCCCAAATTTCGTGAATGGCA

>contig01083

TAAATTTTGTTTAGCAATTATTATATGTTTtAATTtctACaGTCGTTTGTAAACtATTATTCCTGtATTTttAAAAaaTTTTtAAtCGTTTCAAATGGAACTTTTttttAAATTtGTCTGaCTAGGCGTCTGAAAcTtCAGCCCAaTAGAAGAATaTTAAAaGATAATCGCAAATACTACAATTAATATCACTCGCACATAGAGCCATtAAAaCGTTTtGaCTGAATAATAAATTATTATTtATTTGCTAAAaGGTgAatAAAagT

>contig01084

AGAATTCCTAGTTATGAaagCTcGAAGAAAGATTaTGATGATCAAAGAAATAAGTCCCGAGAAAAAGAGAACTCATCTAACTTATATCCTGAAAATTATCGATTTGCTTATAGAGAATTTTTACCAGATCCCAACCcTATGTTTCGAAATTCTTTAAGaGAAAAaCTaGAACGCAAAGATATGATAACAAGAAGATGTtCATTAGATATACCcGAATTTtATGTTGGTTCtATCTTAGCGGTAACGCACTCTGATATTCATGCTCCTGGAAaGTCAAACAGATTTGTAGGGATCTGTATCCAAAGATTAGGgTGTGGATTGAGAGCTTCGTTTATTCTTaGAAACATTGTAGATGGTCAAGGTGTAGAAGTTAATTATGACCTTTATGATCCTTGCATTCAAAAAaTAGAATGTCTAAGGCTtGAGAAGAGaCTCGATGACACCTtGATCTACCTTCGAGaTGCGCCTCCCAAATaCAGCACTGTTCCTTTCGATTTAGAACCTGAACTTGTAACAGACAGAAAaCATGTTCCTGTAAATCCTATCAAAGTTCCACTAAAGCCTCAACCCTGGGATGACAAATACGAGTTATTCAATtACAAAGGAATACaGGAAGaCACTAtAGTTATCAaTAAAAaT

>contig01085

CAGCCAAATGAATCAGGAACTCTGTAATATTCTGGACTCTACTGATAGTATAGTAAAGATGCAGGAAGCCACCTTGAACAATTTAACTGAAATATCAGGAATTACTGTCGAGAAAGCTGCTAAGAAAGTTTATTCAGATGAAGAGAAACGAGAAACaGTTCCAGAAGAGCAGATGGT

>contig01086

AtGACATAAtGTATTTAGACGAAATTCCAGAAGAGAAAGATGTAGTTTTAGAGCTTTTGCGTCACTTTGATTTAGATCAAAATATTCACAAAGATATTGAATTAGTAGAAACGATTGAGGAAGCTTTGGATCTGTTGACAAATGcAGAAAATTTAGAAAAtATCCTAACACCTGATAATTTTtCGGAATCTAATAATTCATCAAGATCAGATAGTGGgATTTTCAaTAAAATTCAAGACCAGTGTTCATGCAAGGAACTAATCGATTaTAAAAATCAAGAGAAT

>contig01087

GGaGCAGTAAGGTGAATGCGCCGGGTCGAAATCGTTGACTTTGACGAAAAACGCTATAAAAaTTAACGCGTGAGAATTCTTGCTTCAAAGCAAAATTTTTtCGGGTATCATGGATCCTCGCAAATTAATAGATTTGcTCCGAGCGACGATAGATCCCGCACAACAAAAGCAGGCGGAAGAACAGCTAAACCAGATtCACAAAATCATTGGATTCGcTCCTACGCTTTTGCAAGTAGTAATGATGTCGGAAGTCGATATGCCAGTCAGACAAGCAGGTGTTATATATTTGAAAAaTTTAATCACGACAAATTGGGCAGATCACGAACCTGATAACGCTTCCTTACAATTTAGTATTCACGAGCAAGATCGTGCTATGATACGTGATGCAATTGTTGATGCAGTA

>contig01088

CGACAGTCATGTCTTATGAAAAAaCACATTATTTTTCATGTAACAAATGTAGAACTCACGAGAAATAATACATTTAATAAAAACTTTTGTAATAGAATTTTTCCTTACTCTCGCTTCCTACCGATTTTGGAAAATGATTCAATTTTCATAAATCATGGCCTTaTTTTTtGGGAAAGATaTTCTCTACACCTCTATCATTTTCAATTTTAAAaGTCATGTTTtCATTGGTTGAAATCATTGAACAAAATTTATAAAAGATACAAATTTTTTTtGAATGACTTTATTTGATGATTATTTAGTTGTTTTCAAAATTGGAATTGCTAAAgCTGTaGAGGATaTTTTC

>contig01089

ACGAAGTAAATCATTACTGtAAATGaTAaTTTCgACATTaCCGTGGATGAACTCTTTtCCATGAtGaGCTTTAGGATTATTGAATAATTCAAAGAAGAATTTTGTTCCAATTAGTTTTGTTTTAAGAATGATTAATTCGATTTACGGAAAATATGAAGTAAATTAATACTGTGGTTTTAATTTTAAGATATTATACTTTCGGAAGATTTGAGTAATAATTAATTATCCTCAACGAGTTTGAATAGGTTACTTTTTGATAATAAATCAATAATTGTTTATCTGAAGGTTATTTATTGTATTCAGCGCCACGTTCTTTAATACT

>contig01090

GtCCATCAACCAAATACTTATCGGAATATGGTATaCATTTCCTTTGTAAGTCACAGGTATTGTCCCTTGTAAATTCAGAAGCTCTTTGCGTGAACCATCGTTGAaCACATAAGGTTCCATTTtGTGCTGCAATCCTTTGTAGATTTGCAAAACGTTAACAACATCTCTGCGGGTGATTTCCGAATTTTtATATTTTGAC

>contig01091

ACTGTTTtATGCTCGTCtCAGTAGCGTGATTCA

>contig01092

AACTTGGATTAAAGTTTtCTACCTTTATTTCACGAAGCACCTTAGGAGCATCATCCTTTTTCAGATCCATCGTGTATATTATTCCAGGAGTCAAGAACGACGTGAATTGATAGAATATTTCTGAATATTTCTTATCACCAGAAAAACCTGCGACAGTTCCAACCTCCATGGGGAACGTTCTGATCAACTCCCCAGATTTTAAACTACGCAGTTGCAAAACATCTTTTACATCTTGGATGTAAGTAACGACAAATTTATCATTATCTACTGCAGTTGCCCAATCTAGAACATCGTGAGAGTGTTCAGGTAATAAATCGGTCCACTTATCCTCCTCGTAATTCAGCAAATCTATGGCtATTAGCTTATAATTAGGCGCATTTTtATTCGTTCGGAAGATCGCCTTCGTG

>contig01093

ACTAAAaTGGCCATATATTAATGAAGATCAAAGACGTCTCACATTTGATTGTCATTTACAAGGAACAgATGAAAaTCGTGTATTATTTATATATGCTCCAGAAGTGACCTTTGAAGGATTGTTGGGTTGCCCcGTATTCCAAAaTAATAAGCTTGTAGGAATTaCTtCcAACTATTATAAGAACTCAGGACGTCTTTACTTTAtACCCGTTATTGACTTAAAaGGTTaTTATCCAAATTTTGAAGCTCCGATAGTTAAGGAAGCCGAGAACAGTTtGGGAGATCcAaTAAATAATGAATCTGGGAGCAGTTCAGGAGTTCAAaGTGAAGAAaCATCGACTACAAATCGTAGGCATAAGGCGAATAGATCAGGCATACCATTCATAAAATG

>contig01094

ACCACGCTATCGTCTCCCCAGTTCTCCCAGGCGCTATCAATGTTCTGGTCTGCTTTGCAGTCCGGCCAGGCGGGCCCAGTCATCCGTCAATTCGGCCTGGGAACGGAGGCTGTCACCGCCGCAGCTAATGGgAaTCTCGAGGAATTCGTCAACGCTCTGGAGTCCTCCAAGGgAGGaCAAGGCCAAaCCGACcAGGGTCAAGAGGACAAGAGCAAGAAaCCATCCCcACcTGGAACTGGATCCAGTGATAAAAAaGACGAGGATGATGAGGGCATGTGTCTTGATTAAGACTGACTTTTCATCATCCCCTTTTTGTAaTGAACGTCAATATGTCATCCGAGTATTGTGCTCTGAAAGTGTCAGTAATTCTGAAATGTTACtATTAaTGCTTTAAATACGATTTGTGTATCCTTTGCAATGAATAATGGTGAGAAGTAGTAGAGTGTATGCTACTCATTATGT

>contig01095

CTCTATCGTGGTTATAAGCTGCAGAGTGCATATTGTCAATAGAAAATGTCTTCTGCTGCAAATTCCCATGTCATTTGGGCAGAAAGATGTTCGTCAACTAGAAGACGGTCAACTTCGGTTAGTTGGGGTTAACCTTAACAATTTTATCACATACACGCCGCATCGCTTCACTCTTGCGCGTTCCcAAACGCCGCCATTTTtCAACACAACACTTGTTCCTTTGCTTTTCGTGGGTGGCTTGGTGGCTATACACTCAGCTATACAAACTAAAGCAGCATTTTTGTAAGGTGCATAGTAAGGTGTAGGTGTAAGCAAGCATAAGCAAGC

>contig01096

ACCCTGAAGTGCTTATTACGCCCTGGTGCTTTAAGAGATACTGAATAATCGCCCATATTGGTTTCGCTATCTCTTATTAAAAAaTCACCATCGTGTCCGTGCTGGTTCAGATGCGAGTCGCATTGCAAACGGGTGATTCTCCCATAATACCAaGGTTTCCCtACAAGATGAGGTCGATCGCCAGGGTCAGGCCTTCTTtCCAGTGAATCTCCGActCCGATTTCACTACTCGTCATTCCTCTTTCACGATATGGCTGGgTAAGATACTCGCTGAGTTCTTGGAGATAATTACGAGGAACAAGGCCGATTTGTCCTTGACCATTTCTGGCTTTATACCATTCAGGGTCAGCAGGTGGACGGTCTAGGATCTCGAGTCTGTCTCCTTTTTCGAATGAGaGTTCCTGATCATTATTTGATGAAAAGGAATACAAAGCGACGACAATATCGAGAACGTTTtCGGCCATAGCGTAGGTgTGTAGCGTGTCATCTGCATCGCCTTCTTCCTGTGTGTAATTTGATGGAAACCACCCGGCTTGATTACCACTTTGTCCTCTCCACCAGCCATCATTACTCTTCTCGAGGATGAGGATTCTGGTTCCTTTCACGAGAGAAAGTTCGTCAGCTTGTTGTGCTTGGTA

>contig01097

ACTAGAGACAGAATCTTTTTATTTtaTAGTTATTGAATATTGTTCAGGTGGAGAACTATTCGATCATaTAGTTGAAAaGAACAGATTGAGTGAAACTGAGTCACGTAAGTTTTTtAGACAGATTGTATCCGCTGTAGCCTACTTACATAGTTTGggaTATGCACATCGAGATTTGAAACCAGAAAATGTGCTGCTAGATAGGGATCAAAaTTTGAAaCTCATAGACTTTGGATTATGTGCAAAaCCAAAAGGtGGCATtCAAAaTCCTTTATACACATCATGTGGATCACCTACTTATGCTGCTCCGGAACTAAtACTGGGCAAGAAGTATTTAGGTTCTCAAATTGACATCTGGAGTATGGgAGTCCTTCTTTACGCTCTCCTTTGtGG

>contig01098

ACCACTTATGCTACATACTAGTCTAGATTGTGAGCAGTGGGCAAAAGGTAAAGGATTAGCAAGTTCATTAAGAGCTTCATTGCAAACGGGACAACTGGAATTCCGTCCCTCTTTATTCCCACTGTAACATTGTGGAGTTTTCAAAGCAGACAAACCAGCCTGCAATGCAACTGTAAACACACTCTGACTAGCCAGTTGGAAAAGTCTGTAGTtCTCATGGCGAAATTGTTCAAtGAGTTTATCCCATCTCTTTtCATCTAAAAGATCTTTGTAGGgACTGAGGTAAGGATTTGCTGGAAAGGCcAACTGACCCATACAGCATTGGATTtCTTGCAATTGATAGTCGTCATAGTTCGAGAAaCATTTTCTGGCGTGTTTAACGGcGTCAAGTCTTCGATCTGCTCTAACTAATTCTATGAATTCTTGAACCcGTAAaTTgAATTCCATCGTGCTTCCCAATTTTCGGAG

>contig01099

AAAGTTTTCTCTCGCCTTTGAaCTGGTAAAAaGTCGATTAAATCTGGAGGTAATAATGGTAATATCCACGATTCACTTAAACTCTCCaCGTATTCAACATTTTtATATGAAAAAaCATTaTTGCTATTTTTCCCAAGGACCCAAGATTCTAGCCACACAGTTAAATAATTtGTAGAAAAATTtGCAcTCaTATTtGTGTTAATGTGTGGCAAAAGTGAACTTTCTAGGACAACACCAATTCTATCATTCGTAAGTGGTTCAGAAATAATGTAATTTTtAATTTTATtAGCTGGGACGGAAACTTGATGCATTATTTCTACTCTTTCCTCTTTTGTTATCAAATTTACAGTAAATATCAACCAAAAACAGACGAGCAAAACTAATTTTGAGTATCGATAGAATGGTTTCTGCTTTTTTtCAAGTTCGTGGACTTCTTcTtCATCAACACTGTATTTGCGTAAAATTCCTCCACCTGGTCGGCCCAAGGACATATTTAAGACCAAaCATTCTCCAGATGGTGCACTTATCGAATACctGCTTCTGTCACCTCCTTTAACTCTCTCATACTCTTTTTGAAAAACTGCCAAACTCGGATCCAACCGTATATCTTCCGGAAGTTGACTCCAAACTCTTAGT

>contig01100

CATAATAGCCAGAGGGAATCAAAGACCTGCTTCTATGTATGAAACCAGAGAAGGTTTGCGAAAGCCATCGACTTGGAATGCAAACGTAAATCAGACAAAGCGCGAAAACGAATCTGTAGCACGACCGTCTACAACTCAAAGTCTTTGGGAATATGGCGCTGCAAATCGTCCCCCTCGTGAAGAAGTTACTAGGCGAACAGGTCAAGTAACAAAaCGTATTCAAGAGCTTTGGATGGCCATGCAGAATCCTAGTCACAGAGAaGCTTTtGTGCCTTGCGCTGAGCGAATCCGtGTTGCGGTAGCTGAACTTACTGCAATTTTCCCTCAGAATCCGATGGAAGAAAATGTAAGATCTGCTTTACGACAACTAAATGGTAATACTGGTCGACTTCAGGCAGAGTGTGCAGGTCTGCAA

>contig01101

ACAACCCTACGGGTCACCGAGTGGTTGGATTAAGTATTATAATTTAATCCAGTTTTTAAAATTTGACAAAAAAaTGAGTTTGTGGGTGGATAAATATCGTCCAGTGAGTCTCAGCAAACTAGATTATCATACTGAACAAGCGGAGCATCTCAAAAATATGGTTCAAAAAGGAGATTTTCCTCATTTACTAGTTTATGGACCACCAGGTGCTGGAAAAAAGaCTCGAATAATGTGTATAATAAGAGAGCTTTATGGAAGTGGAGCAGAAAGGCTTAGAATGGAAAACATGCAATTTGAGACTCCATCTAAAAAGAAGCTGGAGATAATGACAATAAGTAGTAATTATCACATAGAAGTAAATCCGAGTGATGTTGGCATATATGATAGAGTTGTTATAATGGACCTAGTAAAAACTACAGCCCAAACTCATCAAATTGATCCCAGTGGCCAAAGAGAaTTCAAAGTTGTCCTACTGACAAaTGTAGATCAGCTCACGAAAgATGCTCAACATGCTCTACGTCGAACTATGGAAAA

>contig01102

ACAGCTGATCTTAGACTTGTTTACATTTTCTTAATATTATATTTTGGAGCTTTAATTATGTGATTTTCGATACTTAAATAAAAAGCCTTTTATACTTCAGCAAATTGTAT

>contig01103

GGCGACGGAAGTTGAAAAGGCTCAAACTGCAACTCCTGGTGAATCTACCATATTCGGTAGAATATTACGAAAGGAAATACCTTGTAATTTTCTTTATGAAGATGATCAGTGTGTCGCATTCCACGATATTAATGCCCA

>contig01104

ATAAaGCAGCGTCTATGAATGCGTCACTTTGGGCTTTA

>contig01105

GGTGCATGTAGAATTGCAATATTATCActGCTCTTGGCCTGcTtGTAATTGGAATAGGTGTGGATTTTCTTAATATCTATTTGTTGAATTtCATCACTTTTAACCATTACGTTAACTATTTTGAATAGTTTAACATCTTTCATAGTATAAGCATATTTTTtAGATTCCTTATTGAGTATAaCATTAAaGAAAAAGTGAGCTAATGTTAAAACATATTTCGATGATATATAGATTCCATATCCTAAAGTGCTCTCAaTTTGATCAATTTCTTCATGACAATTTTTATTTGTGTG

>contig01106

CAGTTTtGCCAGTTCTTCTCGTAGAGTGTCAAGTTTAGCATTAGTTTCTACTCGATCTGGTTGTTCATTAAtAaCTTTAGCCAAAaCGTCATACTCTATCctGTTTTTGCGAACGCGTTTAGCCTCCTCTAATTCAATTTtCGTctGTTCAATATCATTTTtAGCTTCCTCAATCTCAATTTCAATTTGCTTaGAAaGAG

>contig01107

ACACATTACTCTCATAGCGCCAGGAACTACAACGCGTTTTCTACGATCGTAGGGTGGTGGACAACCTTCGTAGACCTTAAGTCTCCTTAACGCATCTTTTCCTCTTTGAGTTTTGTGTGGGATCATTCCACGGACTGTTTTCCACAGAATTTTGCTTGGCGCGCGGAAGTGGAAGGGCCCACGTGCTGGATTAACGTTACATCTTTTGCGAAGGAAGGACATAAACTTCAGTTTGTTTCTGAAGAAATTTCCTGAGATGTTCAGTTGTTCACTTCGAACAACAATAATTTtGTTACCTTCTAGAGTTTTCTTGGCAATGATAGCTGCCAGCCTTCCAAGAAGATGGCCACGGCCATCTATAAGGaTTGGCTTTtCACTGAAGCCCGTCATTTTGATA

>contig01108

GGAGACCGCACACCACATGAACGGATACGCGAACGGCCACACGCCGCCGGAGCTGCAGCAGAACACCAATTTCCTTTTCACCTCGGAGTCGGTTGGTGAGGGACATCCAGATAAAATGTGCGACCAAATAAGTGACGCTATATTAGACGCACATCTGAAACAAGATCCAGATGCTAAAGTTGCCTGTGAAACAGTGACGAAGACGGGAATGATTCTTTTGTGTGGAGAAATTACATCAAAGGCAGTTGTTGACTACCAAAAAATAGTGCGTGACACTGTCAACCATATAGGCTACGACGATTCGTCGAAAGGTTTTGACTACAAGCTGTGCAGCATTTTGTtGGCACTCGATGCCCaGTCCACAAaTATTGCAGCAGGCGTGCACGAGAATCGCAGTGACGAAGAAGTTGGTGCTGGCGATCAGGGTTtGATGTTTGGTTACGCAACTGATGAAACTGATGAATGtATGCCATTGACTG

>contig01109

GTCGAACGTTCCCTGAGTGTCCATAAGAATTATTGCgaCTTtCTCGCCGTCTgaTAAAGTGCTAAGGAACACTTtGGACCACATCAGGATTCCAGTTGTGTCTCGTTCGGATCCTCCTCGCCAGGAAAaTCCACTCAGTGGATCGTTGTCGTTGCCTAGCCATGAGTCTTCGTTGGTTTTGTGTTCAACATACTTGCTGTTCATGTAACGGAGGAAGAAGTCGAGGAGAAAACTTTTGCCTTTCCTGAAGGCACCAGCAACGGAAACAACGACCACACTTCTGTCCTTGACTTCATCCTGGAGTAAAATATTCGCCAGGGCATCTTCGTCTAATTCAAATGTGTGATCCGAGTGTGAGACTACTATTTGAATCGGTCGACCTGAATCTTGAATTTCTTCCTTATTGTTCATGCAGACTTTTtCTAAGCTCTCTCTCCTTTTtAAGTTGTCCGTGTCTTTTTCTCTCTCTGGTTGTATATAGTTTACATTCTCGTCTTCCGATATCATCGCACGCTTTGTGGCAACACACGTATTATTGCAGTCACCACTCTGTTTATGTTCTTTTGTGTCCGCCATATTGa

>contig01110

CGAGCGAaGGACTTGAGGATTTTATACTCATTTCTTGGAGAGAGGgAATTCCATGCAAGGATAAGTTTCCAAGGTTTGAAGGCATGTGCAACGATTGATTTCCATCACGGTTGTTTATGGAGAGACTGTCCAGACTTTCCATAATAGCTGGACTGCTTGCTTCGACTCTAGGGCCATGCCAGACAGGGtGCGGCTTACCACGATAATCCAGAGCACTCATATTGCCTTTTTTTGGGGGCTACCAGTGCATGGCACCTTGTTGTCGATTTAAGACGATTGTTACTTAATGTGAATCTGCTACTTCACGTCTTAATTATAGGTGTAAATAGGGATAAAATAGAAGGAAACACATAAATTTTGAAACATTTCGGAACATTAACTACGACGCAAAACAGACGCAaCTCCGCCaT

>contig01111

ACTTTATCTTGTAGAAGAAACCTACGAGATTTACAAAaCTATTACTCTTCCTCATATAGAGTCTCAGCAATTTTCTTTGGAGTGGGTTGACAATATtCTTGAGCACAAAGCGGAAAAaGACAGAATTATCTACGAAGATAAGGATAAGGAAACTGGATTTCTGATGATACCTGATCTCAAGtGGGATGGAATTCCTGAATCTTTATACGtCTTGGCGTTACCACTGAAGAGATTAAAaTCGATCAGAGATTTGAATGATTCTCATCTGCCGCTGTTAAAAAATATTCGAGATTCCGGTATTGATGCTATTTATAAGAAaTACAAAGTATCTGGCTCTCGACTCaGGATTTATTtACACTACAGACCTTCTTATTATCATCTTCATGTTCATTTCACGTATCTTAtGTtCGACGGtCCAGGTATATCTGTCGAAAGAGCGCACTTACTTTCTACGGTGATACAAAaTATAGAACtGATGCCAGATTACTACCAAAAaGCCACTTTATTGTTCGCTGTAGCAGAAAaCGATCCACTCTATAAAGAATATCaGGAACATGGGGTGCTGCCTTCAGAAGTTAGTTTAACCAAAAaTAGTTAAGTTAGACATGAATTCATAAACATATTTTGaCTGAAGT

>contig01112

ATTAGTTTTCTTTTAGAAATCAACGAAAATGAAGTTCTCAATAACCTTTTTGGTTCTTTTATGTGTTGCTTTGTCTAAACAAAATGCAGCACTTGTGGGTTGTCGTCTTTCACATCCTGTGCGTAAAGAAACTGAACTTAGAGCGGAATGTATTCATATCCAGTGTAATCCCGATGATGATGAATATATTTCAAAACCGTGTCCATTAGAGACATGTGAGGCGGGGCAGAAAAACATAGCAGAAAATGAACCCAATTTAGATGCTGATACTACTTATCCAAACTGCTGTCCAAAACTATATTGTGTACCAGAATAAGATATACAAAAAAGGCTTGAATTGGATTATATATTTATAGTTATTGGAGAAAATAACACTTTTAAGCTACTGCAATAATTTTTTATTCTTCAATAGATTAT

>contig01113

AGACTGCTAATATAATTtCCTTCATCAGCTCCAaGGCCTTTGCGCAACACCATGCTCCAGAGCACTTCTGAAAGCTGGGCATGGGCTAGTGATAAAGCCCAAAGACGTAAATACGAAGCTGTATGTGATaTtGTCGAAaGCACATATtCTATCGTATGTATTGCTTGATGGATCATCACTTCACTAAAaGCTtCGTCTTCGTGTTCTTGTGATCCTCCTCCcAAATTAGTTGTTGCATTAATTTGACAACCTTCTGGCTGCAATTCAATGTCTTGAGATGCAGAACCATTTGTCATtGTTTtGCCGGGTTTTTCTtGGCGAAGACGGTGTAGAGTGGTTTCCCAAaGAGCATAACTGGAATGCaGAGAAGAGCAGCAAaGACACAAAACTGCTGTAGAACACTTTGACCATCGAACATGAATTCAGAACAACCGTTAGCAACCTTGGAGTGCTTG

>contig01114

ACTTTTtGTAATTTCCACTCATTGTCACAGCCCATGGTTGCTTTTTATCTGCTCTTGTAGCAAATCTTaTAAAAaTTGCCTTTGACTTAGGATTATCGAGCCCTTTTCTCCATATTCCTGGTGGCGGCGGATAACTGATGTCTTTGATATGAATTTCTTTTTCATCAGAGATGCTTTGAATTTTCATACCATTGCTtCCACTACTTCTATCTTTACTGTCTACTTGGATATGAAATTTAGCTTTGCCTTtATTGCATTTtGAGCTCGATTCTATTTGACTTCGGTTACTCAATCCGGTGATTTTCTTGGTTAAACCAATTAATGCTCTTGCAGCAGAAGCAGAATCTATCCACATGACATTACAAGAAAAATCGTTAATCCATTCTAAAGAAACAGGAGCATACTCCTCGAAATATTCAAATATGTCCTC

>contig01115

GGGTCCAGCTGGAATGCTGACCCAAGGATTCTTGTTGTTGATTTCCTCTGCTTGCATAGCCTTGGTAAGATGCATTTATTCCAGGATATGGAGGAGGCATATCTGTCATATACACACTGTTTGCTGGAGGAGCGTCCGGAAATACATTATTTGGTGGAGTCCAGCCGTAAtATCCATTTGGAGCTGCaTAaTAAGCTGGGGgAGGAGCTGCGTAaTAATTAGAAGTCGGTGGCTCATATGGTGGTGGTGCATCAaCTTGAGAACCaTTAAGTTGGACTGCCATTGAAGCTGCTCTCAGCAtAGCTTGACCAAATTCAATGGCTCCCCcACTTTTGAAGTGAAGTTTAAATTTGCATTCACCAACCcAGTTCCCATTAGGTtGAGCTCGACATTTTCCTTTGATATAATTtGCACCAAACATGGGTtGCTCAAGTTCCaCTTCAGAAAGTATAATAAaCGGAAAGCTGAATGATTGCATCTTTtCTTTTtGATCTTTAGCATTAAAAaTCATTCGAtGTGTAGTCAAGTATAGTCGACCTCGTTTACATCCGATGAATTCAATTTGCTCTTGTCCATGAAATTCCATTGTAACATTATCGCAGAACAAAAGTATAGATTCTCCAGAATGGATGAGT

>contig01116

ACGAAATAAACAATGATCCAAACAGAAAGGAGTTCTTGGATGACCTGTTTAGTTTtATGCAGAAAaGAGGAACGtCCGATAAATCGATTACCCATCATGGCAAAGTCAGTCCTAGACCTTtATGAACTCTATAATTTGGTAATTGCCCGAGGCGGATTAGTAGATGTCATTAACAAAAAaCTATGGCAGGAAATCATCAAAGGTCTTCACTTACCGTCAAGCATTACTTCTGCCGCATTTACACTGCGCACTCAATATATGAAGTATCTCTATCCATATGAGTGCGATAGGAGGCATTTGtCAACCCCAGCAGAGTTGCAAGCCGCTATTGAT

>contig01117

ATAaTGAAAATTGTaTTTCCAATAATTCTaCTTGATGGAaGAAACTTTCAGGTAACCATAA

>contig01118

CATGaGTAAATGTAGTGAGATACATTGACTtACAATGAAGTCGTGTCTCTAAAAAaTaCTCATtAAATACCTTTTtGgAGCTTtGTGCAATATTAGGTTCGCGATTCAAGCTGAAATCGTGTTTGGGATTCTTTTAAGTAATTTCGCTTTTtGCGCTGTATATTGGCTTCAACAAGAAAAaGTAAACTGGCTATCAAACTTAATATAGTGCCAGTAACCCCcAAACCAAATGACCATCCCAAATAGTTATTGTCATGACCAGGCATCCAGCcATCTGTGTTTCCCAGGCAAGCAAAAATAATCACTGCGAAagCTCCACAGATTCCACCACATAATAACAAGAATCCAATTGTAAAGATCAGCTCCACATAACGTTTCTGCTCAGGATCGCAACACAATCCAAAGAGCAGT

>contig01119

ATTACAGTTAAATTATGTAGACCtGTTTTTAATTCAGTGGCCAGTCTCAGATTCGAACGAC

>contig01120

ACTTGTAAGCAGACATGGATAGCCcATTTCCGGTTTTGAAAaTTAAAAAAaTGTTTTGCTGAagATTTtGAACAAATCACACTTTTAAAaTTATCCAAACCAAAAaTAGTctATCTGTATCCATTTCtaTCTTTTCTACCATTTCCGCAATTTTTtAAaCATAtAcTtGTAAAAAGctAAAATtAATAACATTTGTTAATTTCCcAAGTaTtACATGTCTATAtCGCCGTATtATAAAaTCCAAAtATAACGTTTTTTtCAAATTGCCTATGAAATTAATAATAATGTGAACGACAATTtcATTTTCTAAATCTTATCGGACTAAACCTGGTTGATCATCAAaTAATAGCTATACAATTTATCGAACTATATAAGAAAAATTCATATCGAAGtAGAGAAGAACAATAATAATATGTTTCATTTACAAACTATTATACCCTAAAAaTATTATTATTTAAAGATTTTCGCTCATTCCGtCaCTTTCtGAGACGAGAGCGCGTTCCAaGATTACACGACCCTCTTtATATCGCTGATTTTCGTCTGTAGCAAATTCATAAATCCATCC

>contig01121

GTATGCATCAGATCGTTACCATCGTCCTCCGGTATTTtGAAAGgCTCtGTTTTTATGCTCTCGTATAAaCTTACGAGAAGCTCTCGTGGAAGGTCTCCGCCATTATTAATCCCCCTGTTCATGGAGATAAACTGCTCCACACTAGGTTTGTCTTtGACACTAGGATTATGTAaggAAGTGTTGAGCATTATGATTGCAAAGCTGAGAACGTAGCAAGTATCCGTATTtGTGAaTATATTGGTATTTAGCTGACAATATCGTTGGGCAAAGCATtCCATCATCCTGTCGATTTTttGGGCCTCTCCTGGTAaTCTAAaCGACCAGAGAAACTGCCTTAAAGCCTGGACCAGGATAAGATCTGTGAAGTCGTGAAGCTCGACAAAAGCTCGAAGAACCCTTTCGTTGAAATCGTGCCTTTCCCCcAGGTAATCACCAATTGCAGTTTTGTTCAAGCCTTCGCCCTTGTAGAGAAACTGAGCAACATCTTCGGGTGTCGCTGCCAAGAGGTTGTGTTCAATCAGGT

>contig01122

AATCTGGGTATTTCCTTCCATCAAAATTCAATTCAGAGGAATTAGTATCCGAAGATTGAGAC

>contig01123

GTGTGCTGTTCTGCACTTCTCTTTATGCAAGAaCAATGTATGTGGATCACGAAaGG

>contig01124

GCATCCTAAGTAACTGGAAAaCTTCGAACTtCCACCGGTtACGGACATAAaCTTTATACTGTTGTTCCAAAAAaTCATTaGATGGCATCTGTTCATAaGGAatGCCAATTTtCATAAaT

>contig01125

GAAGGCtACGAAAtCATAATCGTGGACACGAGTGGAAGACACAAGCAAGAAGAGTCATTGTTCGAAGAAATGTTACAAGTTTCTAATGCAGTAAAACCAGATAATATAATTTTTGTAaTGGACGCAACGATAGGTCAAGCTTGTGAAGCTCAAGCGAAAGCTTTTAAAGaTCGCGTAAACATTGGATCGATTATAATAACAAAaTTAGATGGTCaCGCTAAAGGAGGTGGAGCACTTTCAGCAGTTGCAGCAACACAAAGTCCtGTAATTTTTGTCGGAACCGGaGAGCACATTGACGATTtAGAACCTTtCAAAACGAAACCCTTtATTAGCAAGCTTTTGGGAATGGGAGATATCGAGGGTTTAATCGACCGAGTTAACGAATTAAATCTCGATGATAATGAAGAGTTATTGGAAAAAATTAAACATGGTCATTTCACATTACGAGAT

>contig01126

AGACATTGAAAGGTGCACGTGAAAAGTAATTATATGAGATAA

>contig01127

CTCAATTAGACAAATGACAATTTTTATATAGTTTATAAGAACTGATTCTCTTATTATAAGAGCTCAACGAAACCCGATGAGAAAACCTAACCTGAAAGTGAAAGGAACAACGATATATTGAACAGCTAAAATAACAAAAGTCTACATTTAGTCAaGATGGAATCAATCTTTAAAGTTCCGGAAAATGTTCCAGAAATTTACAAAAACGaTGTtCAAAGAACTCTCTCTTCTATAGCAGAACATATCGAAGGGCGCGGAaGAAATTTtCGTTGGTCAaTGAACGaTTTtCAAGTTGGAGCTCCTCTGGGTCGTGGTAAATTTGGTCGTGtGTTTTtAGCCAGAGAAAAAaCCACACATTACATGGTTGCATTGAAAACTCTTTATAAAATCGAACTCGTAAAAGGAAAAGTCGAAAAACAAGCCTTGCGTGAAATCGAAATTCAATCTCATTTAAGTCATCCTCACATTTTACAACTACTGACTTACTTCCAAGATGATAAAAAAaTATACTTGGT

>contig01128

TATGATGCCTTAAACGTGATTAATGTTGACAACGTTGTGAAATATGTGAAAGAAAGACAACAACCAGATGGCAGTTTCGCAGGAGATATTTGGGGCGAAGTAGACACTAGGTTTTCATTTTGTGCAGTAGCCaGCTTaTCTCTCTTGGGACGATTAGATGAAATAAATGTgAACAAAGCTGTAGAGTTTGtAAAGAACTGTATAAaCTTtGATGGAGGATTTGGTTCAAAACCTGGATCTGAAAGCCATTCTGGACTTATTTACTGCtGTGTTGGtCTGCTATcTATtaCAGgtCACCTACATTTGGTGgATGCGGATAGATTGGGTTGGTGGTTATGtGAAaGGCAATTGGCGTCTGGTGGTTTAAaTGGCAGACCAGAAAAATTACCAGACGTTTGTTATTCGTGGTGGGTTCTCTCCTCT

>contig01129

ACGAAGttCtACAaCTATTCTGTTAATTTCTACTGAAAaTTCAAGAAATAGAaGTCAAAaTCATGTTAATAGCAACGCGATTACTTTCTCGAGGACGCACGGGACTCGGTTGCGACGTCGCTAGAAGGCAGCAGTTCAGCACACTTCTGAAAAaTGGGCCCGCTTtCGTGGCTGCATCTtCGCCTCAAAGGCCTTGCGATTTGCACCAATACAGACTCAAGTCAGAAGGTGTTAAGGGAGCAGTTGTCGGCATAGATTTGGGAACGACATTTTCATGTGTTgCTGTGATGGAGGgTAAACAGGCCAAGGTTATTGAAAATGCAGAGGGTTCaCGAACAaCTCCCTCTTATGTCGCATtCACAAAaGATGGTGAGCGTTTAGTCGGCATGCcAGCAAAACGTCAAGCCGTAACGAATTCAGcAAACaCATtCtATGCCACAAAGCGTCTAATA

>contig01130

ACCAGTTCAATTTGAACAATTTCAAGTCTGTGAAGACAGAAAGGAGGATACGATGTTCCAAGTTTATGAGGACAAACTCCAAGAAGAAACGTCTGTCGCATTGCGTGAAACGAAAGAGGGAAGGGAGTTGAAAATAAAACAAGTAACCGAGCAAGTAACGACTGTTAAAATAGAAAAaGAGAAGCCGATCTTAGAAGTTAATCCAACTCTAATCAACGTTATTCcAACCTCAAGTCACATTCTTCAAGAAATAGACGAGAAGAAGGAAAATGTCGAGTGTCCCATGTCCCTCGAAAAATCCCTCACTTTCACAGACTCTATGAAAAAAGATGTTCGGACCAGAGCCGAAATAAGCAAAGTGATTAGAACGAATTTTTTCGAAGTCAATGAATATAGGGCTGATATTTATAaTTATCTACGAACTGCAGAGACATTACATAGACCTAAACCAGGCTaCATGAAGAAACAACCGGATATCACATACTCAATGAGATCAaTACTAGTCGATTGGTTGGTGGAAGTCGCTGAAGAATATCGATTACAGAGCGAGACtCTTTACCTCGCAaTTTCCTACATAGATCGATTTTTGTCATACATGAGTGTCGTTCGCGCTAAACTACAACTCGTTGGT

>contig01131

ACGACATTTATAGCAATAATCATGATAAATATATCGTGATATCTTTCTGTGACACCCGTCTTATCTTAAATAATGAAAGCTCGCAAAATGCATATTTTACATTCACAGTTTTACTTCATAAAAAAaCATCTCTATATTAGATACATAAAATTACACTGATTTATGTAATGGAAAAAaCTACAAACAATACCAACAAATGCTTTTTACtCTTTCTATAACGAACACGAGAATATTAAATAATTCAAGAGCTGAATAGTAGAGcACCAAAGACTaGAAATATTAAATAAACATATCTCGGCCACCTTTtGTATTGC

>contig01132

ttCTtCTTCTCCAATGAAATCTAACaTATTGAGTGCCACAGCAGCTTCATAACAATTCAATAGAGGcTtCTCAAAGGATATACCCCAATCGATACTCAGTCTTGGgCAAGCAACcTGTATAAAaGCATCTACATCGTTAAAAaGTTTCAGTTTATTTGGAAATATTTCCGACAGTAAAATGATAATATGTTGCTTTCTCAATGTATTTATTTTCTCTTGCAGATGAAAGAGAACATTAGTGTTTCCTtGTCTACC

>contig01133

AATGTCAGGGTCAGGGTGAGCGCATCAATTCGAAGGATCGATGCAAGCAGTGCAACGGCAGAAAAACAATTCGAGACCGTAAGATTTTGGAAGTCCACGTCGACAAGGGTATGGTTGATGGGCAAAAGATTATTTTCAGTGGTGAAGGTGATCAAGAACCAGGATTGGAGCCTGGTGATATTGTCATTCTTCTTGATGAAAAAGAGCACGAAGTTTTTAAGCGTAATGGAAATGACTTGATAATGCGTATGCAAATAGAATTAGTGGAAGCCTTATGtGGATTCCAGAAAGTGATTCGGACTCTGGATGGAAGAGACTTGGTGGTAACTTCCcTGCCAGGAACAGTGACAAAGCATGGTGACTTGAAGTGTA

>contig01134

ACACGAGAATGATCCATCATTAAGTTCGCGATTAAGTGTGAGTTTATTACTCTGAGGgAAGATAATAGTCAGCAAAGTGCCGAGTCTAATGTAAGTGAAAaTTGGAATTCGTGCACTTCCTGCGaGTGCGTGATagATGCAGTGGGgTGTCTGTTTGCCAGTGTGtGAATCACGCTTtCGAGGCGCCCGGCTGTCCCAACGCGAATTTGCTTTTtCCTTtATGACATTGACtCGGTATTACATGACCGCCTCCTCCGTTTGGAaTTTTACCCGAaCCcTCTCGAGAGTGACAAATTGATTTCCACCCTGCTTCATGTCTTCTGTCAATTGCCAAaGACCTCGCTTCCGTGTCTTCTCGCACGATAGAACTAAACCGCACGGCAAATCATTCACC

>contig01135

GGggctgTTGTAAGACGAATGTAAATACGAGCACTACGTTAAAACTAAAGGAAAATATTGCCTCCGAGTTGAGTTTTTGTCTGACACGTTTTCATTTTAAAAAAATATAAATCTAGTAAACATGGACGACGACGGTTTTGATCCTTGTGAATGCATGTGGAGCCAAGACATGGCAATGCAGCGCCTTTTATCCATCTTGCGGCAAACACAGGCTTACTGTACTGACAATGAATGTCTCTCTGTTTCGAGGTTGCCTGGGCCATCAACTGCTTCATCTGAAAACTTTTTCATGATGTTCTTGATGCTTGGAATGGTAATCCTCATGTATGCTCTTCGTCCGAAATCCCTCAGGTGGAAACAGAAAGACGACATGAAAGCTGGACGCAATCAGTCTGATTCTAATGATGAACCTCCGCCGTCACCACCAACAATCAACTAAACATTGAAAaGCCACTCAGCAAATTCCATTGAAGTTTAAACGTTTtATATAATTATTTAACGTTAAAaCTATCAACCAGTAAACTTGTATTCGATTGGGCTATCAACGGACACGATTCAGAAGTATTACTGATCCTAAGTGAGATGGTAATGATGTGATGGTAATCC

>contig01136

ACCAGGGTAGTTTACGAAGATTGGAAATTTCTGTAGAAGAGGAGTATCTGAGTTATTTGAGACAAACGTGCTTTAGGGAAAAGAACTACAGAGAATCAATGATATGGAAAGCGAGAAATTTTGGTGATCAAGATTtATACCAACGGGCCAGGAACATGGAAATGCCTTCTTGTAAAAAGTTGCAAGAGATGCAGGCATCATAGCATTTCTTTGAATACTATTGAGCATTTTtGGTGTATtGTAGATATGTGTATATTTTATTtGCGCGTATTtGAAAaTATTGTTtACACTGTGCGTCAACTATCAAAGTGACATCATATTTATTTAAGCTTGTTAAATTtAAAAATTATACGTGATAtAGTTTTTTCCTATACtGAAAATCGATGATATGATTGTATGTTCATTCTCTCTATATTTATTTGAAATAATTCTTGAATCGACCATGTTTTGAGCTTAtAGAAaGGATATGTGAAAGTTGATTGTAACAAATTTtGATTATATTTGTTTAAAAATGATGATATTCCTATTAGATTTTTTGTATTCAAAGACACGTTTTTAAACATGATTAAATTAGGTCTTTGATCACAATCAATGGTTGTGAGTTATGAAAAGGaGAAaaGGAAAtAgACGT

>contig01137

ACCTCATTCAGGAAATGATTTCCAGTGTTAATCAACTAATCATTTTAGCTTATGCTAGACGTATTGCTCATCTTCCCACCATTGAGACAATAGAAAATTGCATGAAGTTGTGCCCGATGATAGTTCAAGGATtCTGGgAAaaCAAAaaCCcACTTTTGCAGCTTCCCCACATCAAAGAAGAACATCTGAAaCATTTCTTGgCTAAAAaaGCGACAAaTTAAAaGtCTCCAGCAACTAGCTCAATTAAAaGGAGAGGATaGGAGACAATTACTAAGAAATTTGACAGAAAATGAATaTAaTAACGTGATgAAaGTTCTCGGtAGCATGCCTTTtGTCGAATTCAAaGTTCGTTCTGAAGTTATCGATGATGAGAATCCAACAGTATACACAGCGGGTGCAATTGTAACAGTGACCGTGACACTAATTCGAAAGGATaTGAATCATTTGTTCGGTGACGAGACAGtCAAGGAACAGACGATAATAG

>contig01138

ATACATGTGTCtATCAGCCGCGGTGTGAGTCTGACTCTGCACTTTATTAGGACATTCTTTTTtCTCTTTTATAACCTTTATTATTCAATTTTTtATTTtAAATAAGTTCTTAAAGtGTTTAAaCGTAAGCAACTT

>contig01139

CTaTtAAAGTGAAATGGGGTAAGgAGCTATTTCCTAAtGTAGAagTTAATAcGGATGAAGAACcGATGCTTTTCAAAG

>contig01140

ACGGGAGTTCAACCTGAAAGGCAAAAAGTCATGCTAAAGGGGATG

>contig01141

ACGTAGCTTGCCGTCCGATTTACGTCAATATGACACTATTAAATCAGGAGCAAATAAAAGAATTACAACGTGATCCATCTAAATATAAAAGCCCTGTTCGGCAAGTTCCACGtGTGATACCACAACGTTTCAAGAGACAACAATCGGCGCTTCGTtCCAGTCAGGTTGAAaTAACTTTTGGGCACCCAATGGAACAGGGCGAAGATATCATTGTTaTAGACGACGAAGAAATAGTGCCCGAaGAAATGACTATGGCAACTTtAGCACAAATtGGAaGTaTTTCCGGCAGTTTtCATGATCGATCGAAGAATCTTTCCATACGTTCAATAaGTAAAaGACACCGCAGTAAAGCAAAAAaCTCGAGGTCTACTGCTGCAATTATGATTGTTGATCTCTGTTCTtCCGACGAGGAAGATAATgCGAATATGCGCATATCTACTGATGAAAATAAaGACCCTATGGACTGTTTAGAGCCGGAAACAAGTTTTTCGCGAAGTTTTTCGTCGAGTAAGCTTCGTCCAAAACCTTGCATTTATCCCGCGGATTCAACGAACAaTAGATTTGCAGGTAATAATTTGACTGCGGATAAAGACAATCGTGGTAACAAGTGTCTCGGTGaTGTCCTAAAaCA

>contig01142

TTTTTACGGGGTAGTTCTTTGATATTATATCGGAGAAAAAATTATTTTAAACGAGTCATTCTCCATTCCAGAGAAATGTTCACTTTCGATATGCAAAACTCACCGAGTCGATAATCTCATACCGATTTGTGACAAGTCGAAAaTAAaTtCTATACTCTTATAATATAGACCCTCGCTTCCTGGCTTAAGATGGAAGAAGCAGTAACAGCTATCTTCGATCCATAATAAaTTATTATtCAGTtCTTGGgAGATGAAAAaCTCATTTTtCAAGTCTATAAGCTTTAAGATTAGAAGAGTAACGTGCTCATGCCACCCATTTCACTTTGTtCCTTtACGAAAaTCTCGAAGTATTGGTAAATGATTGTCACAGCTAGTAaGATTCCAGTTCCTGATCCGATtGCTCCTAGAAAaTCAGCCAGGACTGAAAGTGCTCCTATGCAGAGACCACCGAAAGCCGCTGCGGTAGGGATGTATCTGTTCAATTCGTGGATtAGAGATTTTTCTCGGTGTCCCTGCATCACCATTTGGGATTCCTTCAACTGCTTAGCTACATCCTTAGCAGAGCTTCCAGAAATTTCGATCCAACtCTTGGAGAAGAAGGCGCAAGATCCTAGCATGAAAaGAatgT

>contig01143

ATCCTTaTGTGTAAGAGTGACATAATATTTGTCTTGGTGTTGTTTCACAAAGAaCATAATTGTAACATTTTCGATATCATCAGATGTATTTAGCACTAACCGGATAtCTAATTTGtCTTTGTAAATGAAACACGCTCTTTTtATTGCATCCCAATCCTGTTTTTtCTCCTTCTTCCGTtCcTCCATTTCGTGTTGCAAATCAaCTATTTCCAGAGATGTTCGCTCCCTTtGTTCCCTGATTTGTTTAAGaTGTTCTTGGAGTTTTtCTtGCTGTTTAATGATATTAGATATTTCCCTGTCTGCAACCTTTtGATTCAACAATACTTCATCCAGTTTCTTTTCAaCATTTTTTATTTGAGTATTTATTTCGAAAATGGATTCAGTATATGAATTACTCTTCaGT

>contig01144
[truncated: 421,572 more chars]
